# Longitudinal Exploratory Analysis of Salivary Microbiota Profiles in Patients with Oral Squamous Cell Carcinoma Before and After Surgery: A Pilot Study

**DOI:** 10.3390/ijms27156873

**Published:** 2026-07-31

**Authors:** Martina Coppini, Rodolfo Mauceri, Davide Vacca, Giorgio Bertolazzi, Vito Carlo Alberto Caponio, Vito Rodolico, Beatrice Belmonte, Giuseppina Campisi

**Affiliations:** 1 Department of Precision Medicine in Medical, Surgical and Critical Care, University of Palermo, 90128 Palermo, Italy; martina.coppini@unipa.it (M.C.); rodolfo.mauceri@unipa.it (R.M.); 2 Tumor Immunology Unit, Department of Sciences for Health Promotion and Mother-Child Care “G. D’Alessandro”, University of Palermo, 90127 Palermo, Italy; davide.vacca@outlook.com (D.V.); beatrice.belmonte@unipa.it (B.B.); 3 Department of Medicine and Surgery, University of Enna Kore, 90100 Enna, Italy; 4 Department of Life Sciences, Health and Health Professions, Link Campus University, 00165 Rome, Italy; vca.caponio@unilink.it; 5 Department of Pathologic Anatomy and Histology, University of Palermo, 90127 Palermo, Italy; vito.rodolico@unipa.it; 6 Department of Biomedicine, Neuroscience and Advanced Diagnostics (Bi.N.D), University of Palermo, 90128 Palermo, Italy; campisi@odonto.unipa.it; 7 Unit of Oral Medicine and Dentistry for Fragile Patients, Department of Rehabilitation, Fragility and Continuity of Care, University Hospital Palermo, 90128 Palermo, Italy

**Keywords:** next-generation sequencing, NGS, Oxford Nanopore Technology, squamous cell carcinoma of head and neck, OSCC, microbiota

## Abstract

Salivary microbiome profiling may represent a promising non-invasive approach for characterizing OSCC-associated microbial patterns and longitudinal microbiome dynamics during patient management. This exploratory pilot study aimed to longitudinally assess salivary microbiota profiles in patients with oral squamous cell carcinoma (OSCC) before and after tumor resection using Oxford Nanopore Technology. Unstimulated saliva samples were collected from 16 patients with OSCC at two time points (before and after tumor resection) and from 10 OSCC-free reference subjects. Microbial DNA was extracted using the QIAamp DNA Blood Kit (QIAGEN GmbH, Hilden, Germany) and subjected to long read metagenomic sequencing using the Oxford Nanopore MinION platform (v. 20.06.4, Oxford Nanopore Technologies, Oxford, UK). Taxonomic profiling was performed to longitudinally characterize salivary microbiota composition within patients and to provide descriptive comparisons with the OSCC-free reference cohort. Longitudinal analysis identified differences in salivary microbiota profiles between pre- and post-resection samples. Before surgery, an increased relative abundance of *Neisseria subflava* and *Leptotrichia buccalis* was observed. Post-surgical samples showed higher levels of *Glaesserella parasuis*, *Streptomyces anulatus*, and *Lactobacillus* species. Distinct microbial patterns were also descriptively observed between OSCC patients and OSCC-free controls, suggesting disease-associated dysbiosis. This exploratory longitudinal pilot study suggests differences in salivary microbiota profiles between samples collected before and after tumor resection in patients with OSCC, including changes in taxonomic composition and reduced alpha diversity. Given the limited sample size and the potential influence of unmeasured perioperative factors, these findings should be considered hypothesis-generating. Larger, well-controlled longitudinal studies incorporating standardized oral health assessment and detailed perioperative metadata are required to clarify the biological and clinical relevance of these observations.

## 1. Introduction

The human oral microbiome represents a highly dynamic and complex ecological ecosystem comprising hundreds of microbial species that play a pivotal role in maintaining local homeostasis and systemic immunity [1]. Oral dysbiosis, defined as a pathological imbalance in the relative abundance of microbial communities, has been proposed as a key contributor to the initiation and progression of oral squamous cell carcinoma (OSCC) [1,2]. The International Agency for Research on Cancer classifies 10 microbial species, including *Helicobacter pylori*, *Hepatitis B virus*, *Hepatitis C virus*, and *Human papillomavirus*, driving 90% of infection-associated cancers [3]. In the oral cavity, periodontal pathogens, including *Fusobacterium nucleatum*, *Prevotella melaninogenica*, *Prevotella intermedia*, and *Prevotella jejuni*, are overrepresented in salivary samples from patients with OSCC [1,4,5]. Several studies have reported alterations in the oral microbiota of patients with OSCC; however, substantial heterogeneity in study design, patient characteristics, sampling procedures, sequencing methodologies, and analytical approaches has limited the identification of a consistent disease-specific microbial signature. Furthermore, the biological interpretation of treatment-associated microbiome changes remains challenging because of the potential influence of multiple clinical and perioperative factors. In this context, saliva has emerged as an encouraging diagnostic biofluid, encompassing a diverse reservoir of molecules indicative of oral pathological conditions.

In the last 15 years, oncogenomics advancements in next-generation sequencing technologies and liquid biopsy is shaping a novel approach for the management of cancer patients [6]. Despite the growing evidence supporting an association between oral dysbiosis and OSCC onset, it is still unclear whether microbial alterations precede the disease or are a consequence of it. For this reason, salivary analysis is emerging as a promising, non-invasive tool for the dynamic monitoring of disease onset and progression, as well as for monitoring microbiota-related changes over time [2,5,6]. Surgical treatment is accompanied by multiple changes in the oral environment, including tissue removal, wound healing processes, inflammation, modifications in salivary flow, alterations in oral hygiene practices, dietary adaptations, and exposure to perioperative medications. These factors may collectively influence oral microbial communities, making the interpretation of longitudinal microbiome dynamics particularly challenging [7]. Characterizing longitudinal microbiome dynamics in patients undergoing surgical treatment may contribute to a better understanding of how oral microbial communities vary during the perioperative period and may help generate hypotheses for future biomarker-oriented investigations.

This exploratory pilot study aimed to longitudinally characterize salivary microbiota profiles in patients with OSCC before and after tumor resection using Oxford Nanopore MinION sequencing platform. 

## 2. Results

### 2.1. Characteristics of Study Participants

The main clinical and demographic characteristics of the study population are summarized in Table 1.

An OSCC-free reference group comprising healthy individuals and patients with OPMDs was additionally recruited to provide contextual comparisons. All OPMD cases were histopathologically confirmed to be free of epithelial dysplasia at the time of enrollment and sampling.

Within the OSCC group, 8 patients were male (8/16, 50%) and 8 were female (8/16, 50%), with a mean age of 63.2 ± 12.9 years (range: 40–83 years). In the OSCC-free reference group, 5 patients were male (5/10, 50%) and 5 were female (5/10, 50%), with a mean age of 63.0 ± 18.6 years (range: 29–83 years).

Regarding tumor localization in the OSCC group, the most frequently affected site was the lateral border of the tongue (C02.1), observed in 9 out of 16 patients (56.3%), followed by the buccal mucosa (C06.0) in 5 patients (31.3%), the upper gingiva (C03.0) in 1 patient (6.2%), and the hard palate (C05.0) in 1 patient (6.2%).

According to the TNM staging system, OSCC cases were distributed as follows: 3 patients were classified as stage I (18.8%), 6 as stage II (37.5%), 4 as stage III (25.0%), and 3 as stage IV (18.8%). Based on this classification, 9 patients (56.3%) were in the early stage, while 7 (43.7%) were in the advanced stage. Concerning lifestyle habits, 4 patients (25%) were current smokers, 2 patients (12.5%) reported both smoking and alcohol consumption, and 3 patients (18.7%) were former smokers. Regarding the potential mechanical trauma, clinical examination revealed the presence of incongruous prostheses in 5 patients (5/16, 31.2%).

The mean follow-up period was 27.75 ± 10.1 months (range 12–36 months). Three patients experienced a recurrence (3/16, 18.7%).

To provide contextual information regarding potential sources of variability, demographic and lifestyle characteristics were descriptively examined. Owing to the limited sample size, a formal assessment of group comparability and confounding was not feasible.

### 2.2. Microbiota Composition

#### 2.2.1. Descriptive Overview of Microbiota Composition

**Figure 1:**
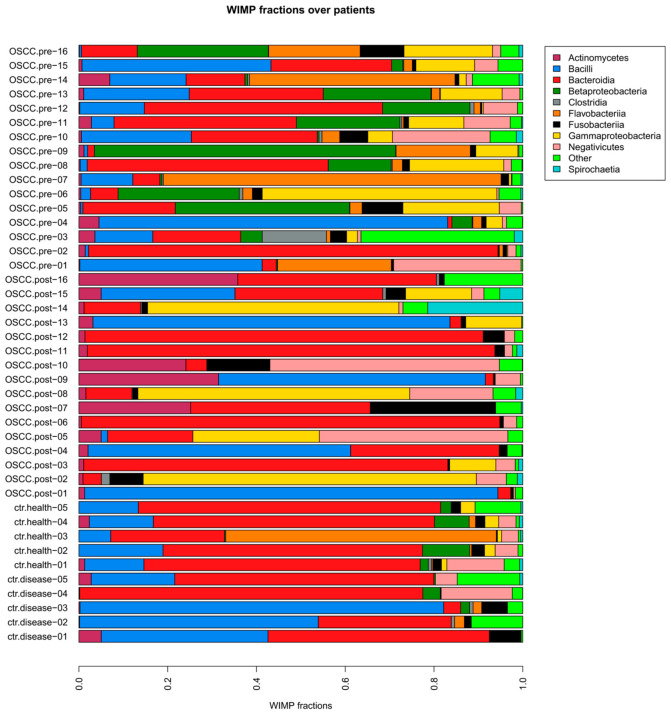
Relative abundance of bacterial classes across all analyzed saliva samples. Relative abundance was calculated for each sample by dividing the number of reads assigned to each bacterial class by the total number of classified bacterial reads in that sample and is reported as a percentage. This within-sample normalization accounts for differences in sequencing depth across samples. Each stacked bar represents an individual saliva sample, and colors indicate the bacterial classes identified.

To summarize microbial composition, we calculated taxa relative abundances across samples. Figure 1 provides a descriptive overview of the most represented taxa. These observations are consistent with previous reports describing the general composition of the oral microbiota [8,9,10]; however, no inferential conclusions regarding group-specific differences can be drawn from this figure alone.

Taxonomic composition was analyzed at multiple levels, including species and class, which are reported separately to avoid ambiguity. The most abundant taxa were represented by *Prevotella melaninogenica* (phylum *Bacteroidetes*, class *Bacteroidia*), *Gemella sanguinis* (phylum *Firmicutes*, class *Bacilli*), and *Haemophilus parainfluenzae* (phylum *Proteobacteria*, class *Gammaproteobacteria*) in OSCC patients, and *Prevotella melaninogenica* and *Prevotella jejuni* (phylum *Bacteroidetes*, class *Bacteroidia*) in OPMD patients and health controls, in line with previous reports describing the core composition of the oral microbiota [8,9,10]. Descriptively, differences in the relative abundance of several bacterial classes were observed across study groups. OSCC samples appeared to show a higher relative abundance of Gammaproteobacteria compared to the OSCC-free reference group; however, these observations should be considered exploratory and purely descriptive, given the limited sample size and the pilot nature of the study. Furthermore, it reported the presence of *Betaproteobacteria* and *Flavobacteria* in the oral microbiota composition of patients affected by OSCC before surgical treatment, compared to patients after surgical treatment and the OSCC-free reference group. Lastly, a prevalence of Bacilli was observed in the OSCC-free reference group and OSCC patients after surgical treatment compared to OSCC patients before surgical treatment.

The observed differences may reflect the influence of preoperative conditions associated with the presence of OSCC, including tumor-related inflammation and local tissue changes, which may contribute to shaping the oral microbiome.

**Figure 2:**
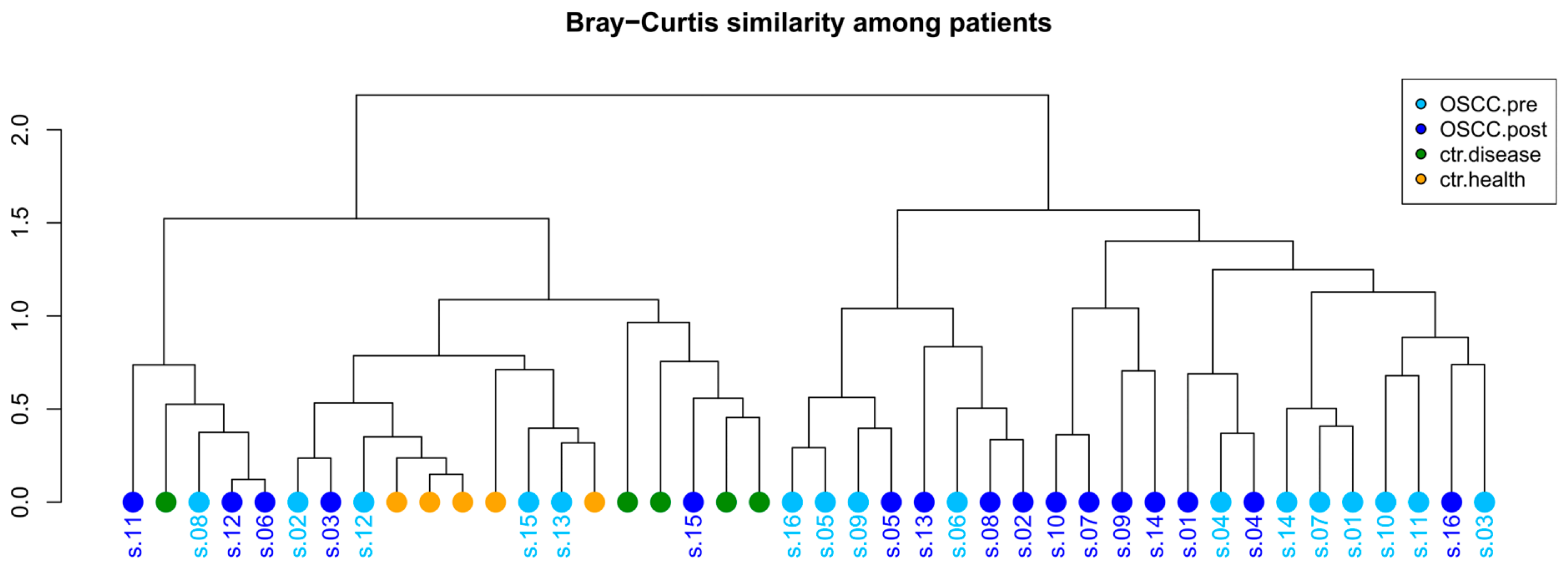
Hierarchical clustering of saliva samples based on microbial community composition. The dendrograms illustrate the relationships among microbial communities across the analyzed samples on the basis of pairwise dissimilarity. Samples clustering more closely exhibit more similar bacterial community profiles, whereas clusters merging at greater dendrogram heights indicate higher microbial dissimilarity. The analysis provides an exploratory overview of similarities and differences in microbiome composition among the study groups.

Hierarchical clustering revealed partial separation between samples obtained from OSCC patients and the OSCC-free reference group (Figure 2, enrichment *p*-value = 0.00012). To evaluate whether clinical factors could influence microbiome profiles, we assessed their distribution across the two main microbiome clusters. Although no clear enrichment of measured clinical variables was observed across clusters, residual confounding cannot be excluded.

To further explore differences between OSCC and controls, we performed an occurrence-based comparison of taxa across groups. Venn diagrams (Supplementary Figure S1) highlight taxa uniquely detected in OSCC samples, as well as those shared with controls. In line with this exploratory approach, taxa occurrence between OSCC and control samples was compared using Fisher’s exact test (Supplementary File S3). *Aggregatibacter segnis* and *Aggregatibacter* sp. *2125159857* were more frequently detected in OSCC samples than in the reference cohort. Given the exploratory nature of the study and the limited sample size, these findings should be considered preliminary.

#### 2.2.2. Pre- vs. Post-Surgical Microbiome Comparison in OSCC Patients

**Figure 3:**
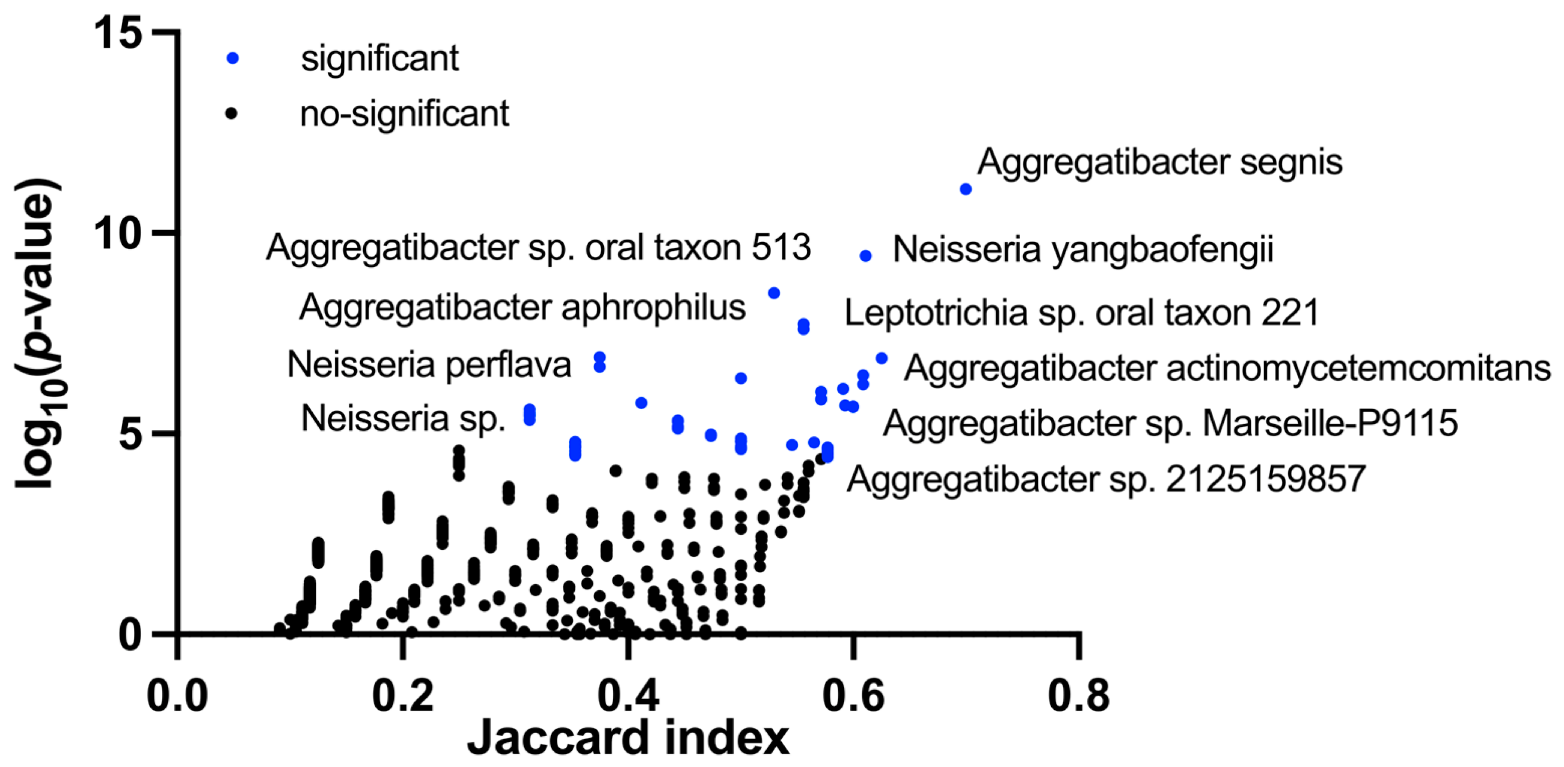
Enrichment analysis of taxa associated with the pre-surgical condition based on taxon presence/absence. Each point represents a taxon, plotted according to its Jaccard index (x-axis), reflecting its preferential occurrence in pre-surgical samples, and the statistical significance of enrichment expressed as −log10(*p*-value) (y-axis). Blue points indicate taxa significantly enriched in pre-surgical samples, while black points represent non-significant taxa. Selected significantly enriched taxa are labeled. This analysis highlights taxa more consistently detected before surgery, supporting a distinct microbiome composition in the pre-surgical condition.

To identify taxa characterizing the pre- and post-surgical microbiome, we performed an enrichment analysis exclusively on the paired longitudinal saliva samples collected from patients with OSCC before and after surgery. The OSCC-free reference cohort (healthy individuals and patients with OPMDs) was not included in this analysis. Taxa occurring significantly more frequently (based on presence/absence) in the pre- or post-surgical OSCC samples were subsequently identified (Figure 3, Supplementary File S4).

This approach allows the identification of taxa that are consistently detected more frequently in one condition compared to another, capturing an additional dimension of the data that may not be fully reflected by abundance-based methods alone. This analysis revealed several taxa enriched in the pre-surgery condition (e.g., *Prevotella*, *Neisseria* and *Aggregatibacter*). In contrast, only a few taxa were enriched in the post-surgery samples (e.g., *Streptococcus* and *Lactobacillus*), indicating a lower number of taxa meeting enrichment criteria in post-resection samples.

These observations indicate differences between pre- and post-resection microbiota profiles; however, they do not allow attribution of such differences to surgery itself, as other perioperative factors may have contributed.

Several taxa exhibited statistically significant differences in relative abundance between pre- and post-resection samples (adjusted *p*-value < 0.05) (Table 2, Supplementary File S5, Supplementary Figure S2).

Taxa that were more abundant in pre-surgery, including *Neisseria subflava* (*p*-value = 0.0180), *Treponema* sp. *OMZ 838* (*p*-value = 0.0290), *Leptotrichia buccalis* (*p*-value = 0.0320), *Veillonella* sp. *S12025-13* (*p*-value = 0.0320), and *Candidatus Minimicrobia vallesae* (*p*-value = 0.0410). On the other hand, although only a few taxa showed enrichment in terms of occurrence in the post-surgery samples, a group of taxa was found to be significantly more abundant in the post-surgery group (Table 2, Supplementary File S5). Among these were *Glaesserella parasuis* (*p*-value = 0.0040), *Mogibacterium neglectum* (*p*-value = 0.0048), *Segatella oris* (*p*-value = 0.0064), *Prevotella herbatica* (*p*-value = 0.0130), *Streptococcus parasuis* (*p*-value = 0.0180), *Streptomyces anulatus* (*p*-value = 0.0190), *Leptotrichia trevisanii* (*p*-value = 0.0350), and *Escherichia coli* (*p*-value = 0.0410).

#### 2.2.3. Alpha Diversity Analysis

**Figure 4:**
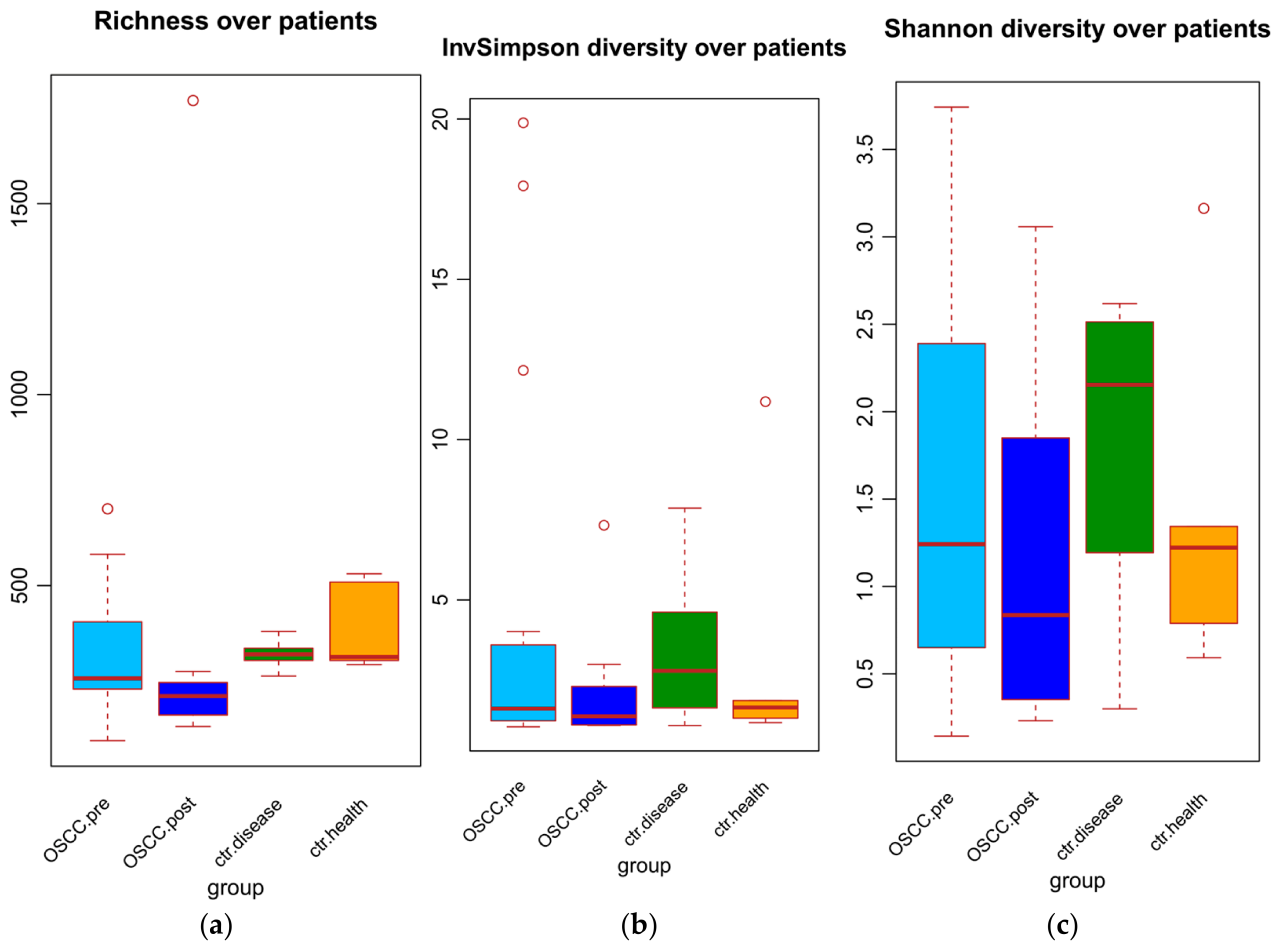
Alpha diversity of the oral microbiome across groups. Boxplots showing the variation of three alpha diversity indices-(**a**) richness, (**b**) inverse Simpson index, and (**c**) Shannon index-across the four study groups. Each plot displays pre- and post-surgical OSCC samples alongside controls. Statistical comparisons indicate a significant decrease in diversity following surgery.

To further characterize microbiome variability, alpha diversity was assessed using three complementary indices (richness, Shannon, and inverse Simpson). All indices consistently showed a significant reduction in alpha diversity in post-resection samples compared with pre-resection samples (Figure 4; Supplementary File S6).

These findings are consistent with previous reports describing differences in oral microbial diversity across OSCC, OPMD, and healthy populations [11].

Although overall alpha diversity decreased in post-resection samples, several taxa remained detectable and some exhibited increased relative abundance (Supplementary File S5), indicating differences in community composition between the two sampling time points.

Given the exploratory nature of the study, the limited sample size, and the absence of detailed perioperative data, including antibiotic exposure, postoperative complications, dietary modifications, and oral hygiene changes, these findings should be interpreted with caution. An additional limitation is that the exact interval between surgery and postoperative saliva collection was not systematically recorded. Although postoperative sampling was performed no earlier than one month after tumor resection during routine follow-up visits, variability in the timing of sample collection may have influenced the observed microbiome profiles because microbial communities can change during the postoperative healing period. Consequently, the relative contribution of surgical treatment and other perioperative factors to the observed microbiome patterns cannot be determined from the present data.

## 3. Discussion

The present single-center longitudinal pilot study explored salivary microbiota profiles in patients with OSCC before and after tumor resection using Oxford Nanopore MinION platform. The primary finding of this study was the detection of longitudinal shifts in microbiota composition and alpha diversity following surgical resection. Given the exploratory design, limited sample size, and the absence of several potentially relevant perioperative variables, these findings should be interpreted as hypothesis-generating observations rather than evidence of microbiome alterations directly attributable to surgical treatment.

To the best of our knowledge, this is the first longitudinal study employing Oxford Nanopore MinION platform sequencing to characterize salivary microbiota profiles in OSCC patients before and after tumor resection. Compared with short-read platforms, ONT provides long-read sequencing capabilities that may facilitate high-resolution taxonomic profiling and represent a promising approach for future microbiome investigations.

To date, only one study has previously analyzed the salivary microbiome profiles of oral cancer patients before and after treatment using Illumina MiSeq System [5].

Given the methodological differences reported in the literature, unstimulated whole saliva has been selected as the most suitable and reproducible matrix for microbiome analysis [12,13].

In the present study, a statistically significant abundance of *Aggregatibacter segnis* and *Aggregatibacter* sp. *2125159857* was observed in OSCC samples compared to OSCC-free reference cohort. The association between periodontal pathogens and oral carcinogenesis has been proposed and investigated for several years. [6,8]. The increased detection frequency of *Aggregatibacter* species in OSCC samples is consistent with previous observations linking periodontal dysbiosis and OSCC [6,9,10]. However, the present data do not allow conclusions regarding causality, biological function, or mechanistic involvement in oral carcinogenesis.

The OSCC-free reference cohort included both healthy individuals and patients with OPMDs without histopathological evidence of epithelial dysplasia. The inclusion of these patients was intended to provide a broader clinical context for descriptive comparisons rather than to represent a purely healthy control population. Nevertheless, because OPMDs may already exhibit microbiome alterations independently of malignant transformation, these comparisons should be interpreted with caution and not as evidence of disease-specific microbial differences [10,11].

Statistically significant differences were observed in OSCC patients before and after surgical treatment. In OSCC patients before surgical therapy, an increase of *Neisseria subflava*, *Treponema* sp. *OMZ 838*, *Leptotrichia buccalis*, *Veillonella* sp. *S12025-13*, and *Candidatus Minimicrobia vallesae* were observed. Among them, *Neisseria* has been previously reported to be involved in oral alcohol-related carcinogenesis due to its ability to produce acetaldehyde via alcohol dehydrogenase, suggesting a possible link with alcohol-related risk factors [8,13]. Moreover, *Leptotrichia buccalis* has been identified in cancer patients following high-dose chemotherapy and hematopoietic stem cell transplantation, particularly in cases with oral mucositis [14,15]. *Leptotrichia buccalis* is considered an opportunistic bacterium and has been associated with infections in immunocompromised patients. In agreement with our findings, in the only other study performed by Mäkinen et al., *Leptotrichia buccalis* was significantly more abundant in the samples collected before surgical therapy compared to after surgical therapy [5].

Moreover, they also reported an abundance of *Actinomyces odontolyticus*, *Streptococcus sanguinis*, *Veillonella rogosae*, *Fusobacterium nucleatum vincentii*, *Capnocytophaga leadbetterii*, *Abiotrophia defectiva, Lautropia mirabilis*, *Solobacterium moorei*, *Porphyromonas endodontalis*, *Mogibacterium diversum/neglectum/pumilum/vescum*, *Leptotrichia buccalis*, and *Streptococcus* sp. in pre-surgical samples compared to post-surgical samples [5].

A significant increase of *Glaesserella parasuis*, *Mogibacterium neglectum*, *Segatella oris*, *Prevotella herbatica*, *Streptococcus parasuis*, *Streptomyces anulatus*, *Leptotrichia trevisanii*, and *Escherichia coli* were observed in OSCC patients after surgical treatment. *Escherichia coli*, a facultative opportunistic pathogen, has been associated with dysbiotic conditions and may reflect alterations in the oral ecological niche.

Interestingly, *Streptomyces* species are known producers of bioactive compounds, although their role in the oral microbiome remains poorly defined [16]. Its increased abundance in post-surgical samples may hypothetically be associated with changes in microbial community composition; however, no functional conclusions can be drawn from the present observational data.

In the study performed by Mäkinen et al., *Lactobacillus casei/paracasei/rhamnosus* and *Actinomyces gerencseriae* were significantly more abundant compared to the pre-surgical samples [5].

Notably, also in our study, a prevalence of *Bacilli* (e.g., *Lactobacillus*) was observed in the OSCC-free reference group and OSCC patients after surgical treatment compared to OSCC patients before surgical treatment.

Notably, an increased abundance of *Bacilli*, including *Lactobacillus* species, was observed in post-surgical samples and in the OSCC-free reference cohort. While *Lactobacillus* is commonly associated with beneficial effects on oral microbial balance, the clinical implications of this finding remain unclear in the context of OSCC and were not directly investigated in this study [17,18,19]. Therefore, the present findings should be interpreted as observational associations rather than evidence of a causal or therapeutic role. Furthermore, the increased abundance of *Lactobacillus* species observed in post-resection samples may reflect changes occurring during the perioperative period rather than effects specifically attributable to surgery itself.

According to our findings, Crispino et al. observed the diversity of microbial profiles across different stages of OSCC, underscoring the intricate nature of the oral microbial ecosystem [20].

Several studies have proposed associations between oral microbial dysbiosis and OSCC development, including inflammatory, immunomodulatory, and metabolic pathways. However, the extent to which specific microorganisms contribute to oral carcinogenesis remains uncertain, and causative relationships have not been conclusively established [2,21,22].

However, the precise mechanisms through which the oral microbiota contributes to OSCC pathogenesis and progression remain to be fully elucidated. Moreover, the development of internationally accepted standard procedures for saliva collection and analysis is essential to enable more conclusive and clinically relevant comparison across studies [23].

Another important aspect to consider is the potential influence of smoking on oral microbiome composition. Previous studies have shown that smoking can affect specific bacterial groups, including *Gammaproteobacteria*, *Betaproteobacteria*, and *Flavobacteria*. In particular, Wu et al. reported that *Betaproteobacteria*, *Gammaproteobacteria*, and *Flavobacteriia* were inversely associated with the number of cigarettes smoked per day and positively associated with years since smoking cessation [24]. Similarly, Liang et al. observed that smoking reduced oral microbial diversity, with decreased abundance of *Bacteroidetes*, *Proteobacteria*, and *Lactobacillus*, and increased abundance of *Staphylococcus* [25].

In our cohort, four OSCC patients were current smokers, and two reported both smoking and alcohol use, whereas only one individual in the OSCC-free reference group was a smoker, suggesting a potential imbalance in lifestyle-related factors. To further explore this aspect, potential confounders, including age, sex, smoking, and alcohol consumption, were evaluated in relation to the identified microbiome clusters. No apparent enrichment of these variables was observed across clusters (Supplementary Files); however, given the limited sample size, these analyses should be considered purely descriptive. Therefore, the absence of detectable enrichment should not be interpreted as evidence that potential confounding effects are absent, and residual confounding cannot be excluded. The paired pre- and post-surgery design may reduce the influence of stable individual factors, although it does not fully account for potential confounders.

Overall, this study provides preliminary evidence that salivary microbiota profiles differ between samples collected before and after tumor resection in patients with OSCC. However, the biological significance, clinical utility, and mechanistic basis of these observations remain uncertain. Future multicenter studies incorporating larger cohorts, standardized perioperative data collection, oral health indices, dietary assessment, and longitudinal follow-up will be necessary to determine whether salivary microbiome profiling can contribute meaningfully to OSCC monitoring and biomarker development.

### Strengths and Limitations

This study has several strengths and limitations that should be considered when interpreting the findings. A key strength is the paired pre-/post-intervention design within the same OSCC patients, which reduces inter-individual variability and allows the assessment of longitudinal microbiome changes. In addition, the application of ONT sequencing represents an innovative and relatively underexplored approach for high-resolution profiling of the salivary microbiome in oral oncology.

The relatively small sample size represents a limitation of this study and restricts the statistical power to detect subtle microbiome differences, as well as the ability to fully control for potential confounding factors. Moreover, no formal a priori power calculation was performed due to the exploratory design and the lack of comparable longitudinal studies using the Oxford Nanopore Technology platform in this clinical context. Therefore, the findings should be interpreted as preliminary and hypothesis-generating.

Importantly, several factors, such as perioperative antibiotic exposure, inflammatory responses, and environmental changes in the oral cavity following surgery, may act as significant confounders. As these variables were not controlled analytically, their independent effects on the microbiome cannot be disentangled from those of the surgical intervention. Relevant variables such as dietary habits and standardized oral health indices (e.g., DMFT, PSR) were not systematically collected, and residual confounding from lifestyle and clinical factors cannot be excluded.

While the cohort’s geographic and ethnic homogeneity (Italian/Sicilian participants) may reduce intra-group variability, it also limits the generalizability of the findings and precludes the evaluation of population-specific microbiome differences. Larger, multicenter studies across diverse populations are warranted to confirm and extend these observations.

## 4. Materials and Methods

### 4.1. Ethics Statement

The study protocol conformed to the ethical guidelines of the 1964 Declaration of Helsinki and its later amendments or comparable ethical standards. The study was approved by the institutional review board of the “Paolo Giaccone” Policlinic University Hospital in Palermo (Italy) (approval #11/2020). Written informed consent was obtained from all participants involved in the study. The study reported was assessed following the STROBE guidelines for observational studies [26].

### 4.2. Population Recruitment

The subjects were recruited from the Unit of Oral Medicine at the “Paolo Giaccone” Policlinic University Hospital in Palermo (Italy), from December 2022 to May 2024. The patients were selected based on the following inclusion criteria:

- Age older than 18 years;
- Patients with a histological diagnosis of OSCC before and after surgical treatment;
- The localization of OSCC is confined exclusively to the oral cavity, identified based on the 2024 NIH/SEER ICD-0-3.2 topographic classification codes, as detailed below:
  - Mobile tongue (including ventral/lateral tongue) (C02.0-C02.1-C02.2-C02.3, C02.8, C02.9);
  - Gingiva (including upper/lower gum and retromolar area) (C03.0, C03.1, C03.9, C06.2);
  - Hard palate (C05.0);
  - Buccal mucosa (C06.0, C06.1);
  - Mouth floor (C04.0, C04.1, C04.8-C04.9).
- No previous history of malignancy or therapeutic intervention in the head and neck areas;
- Capacity to give informed consent.

Patients were excluded if they were pregnant or breastfeeding, had undergone antibiotic treatment or periodontal care within the past three months, were diagnosed with severe or advanced stages of periodontitis, or had any immunocompromising conditions [27,28].

After applying the inclusion and exclusion criteria, 26 participants were included in the study, comprising 16 patients with histologically confirmed OSCC and 10 OSCC-free controls (5 healthy individuals and 5 patients with OPMDs). Salivary samples were obtained from 16 patients diagnosed with OSCC, both before and after surgical treatment, and 10 individuals without OSCC. Among the latter group, 5 patients presented with OPMD, including oral leukoplakia (2/5, 40%) and oral lichen planus (3/5, 60%)—collectively designated as the disease control group—while the remaining 5 were healthy individuals without evidence of dysplasia, comprising the healthy control group.

All enrolled participants completed the study protocol, and paired saliva samples were successfully collected from all OSCC patients before and after surgery. No participants were lost to follow-up after enrollment.

To confirm the diagnosis of OSCC, a histopathological examination was undertaken. After local anesthesia, an incisional biopsy was performed at the Unit of Oral Medicine at the “Paolo Giaccone” Policlinic University Hospital in Palermo. The section from one sample was fixed in formalin solution and sent to the pathology laboratory for histopathological assessment. If the diagnosis of OSCC was confirmed, an unstimulated whole salivary sample was collected. Then, patients were referred to the multidisciplinary unit for treatment. At a routine postoperative follow-up visit, scheduled no earlier than 1 month after tumor resection, a second unstimulated whole saliva sample was collected. Because sample collection was integrated into routine clinical follow-up, the exact postoperative interval was not fully standardized across patients.

Importantly, post-surgical saliva samples were collected before the initiation of any adjuvant therapies (e.g., chemotherapy, radiotherapy), thereby minimizing their potential influence on the observed microbiome changes. Detailed perioperative information regarding antibiotic regimens, postoperative infections, surgical complications, dietary modifications, and oral hygiene changes was not systematically available for all patients and was therefore not included in the analytical models.

An OSCC-free reference group comprising healthy individuals and patients with oral potentially malignant disorders (OPMDs) was additionally recruited to provide contextual comparisons. Given the limited sample size and biological heterogeneity of this cohort, these comparisons were considered descriptive and exploratory.

Given the exploratory nature of this pilot study and the lack of prior longitudinal investigations using Oxford Nanopore Technology in OSCC patients, no formal a priori sample size calculation was performed. The study was primarily designed to generate preliminary data and assess the feasibility of this sequencing approach in a clinical setting.

### 4.3. Outcome Measures

The following information was collected for each patient: demographic characteristics, anatomical site of the OSCC lesion based on the International Classification of Diseases (ICD, 11th edition), TNM stage (9th version) according to the guidelines of American Joint Committee on Cancer (AJCC) and the International Union Against Cancer (UICC) [29], smoking habits, alcohol consumption, mechanical risk factors (such as ill-fitting dentures or sharp tooth cusps), follow-up duration, and recurrence data. Participants were classified based on their smoking behavior as never smokers, current smokers, or former smokers. In terms of alcohol intake, they were grouped into non-drinkers, moderate consumers (less than 16 units per week), or heavy consumers (16 or more units per week).

### 4.4. Sample Collection

Preoperative saliva samples were collected before the diagnostic incisional biopsy. Patients were enrolled in the study only after histopathological confirmation of OSCC. Unstimulated whole saliva samples were collected from each participant in the morning according to standardized saliva collection procedures previously described in the literature [2,4]. Participants were instructed to refrain from eating, drinking, or smoking for at least 2 h before sample collection and to rinse their mouths with sterile saline for 60 s before collection. Saliva was allowed to pool on the floor of the mouth and was subsequently collected into sterile Falcon tubes, which were immediately stored at −80 °C until further analysis.

### 4.5. DNA Extraction and Sample Sequencing

DNA extraction was performed as described by Mauceri et al. in a previous study [4]. No additional mechanical lysis step (e.g., bead-beating) was included in the extraction protocol. Therefore, taxa characterized by more resilient cell wall structures may have been differentially represented, potentially affecting DNA recovery efficiency and relative abundance estimates. Genomic DNA libraries were prepared using Rapid Sequencing DNA–PCR Barcoding Kit 24 V14 (Oxford Nanopore Technologies, Oxford, UK) (SQK-RPB114.24, protocol version RPB_9191_v114_revC_17Sep20-ONT) according to the manufacturer’s instructions. Briefly, a transposase-based reaction was used to simultaneously fragment the DNA and attach sequencing tags to fragment ends. Rapid Sequencing Adapters were subsequently ligated to the tagged ends before loading onto the flow cell [30].

Basecalling was performed using MinKNOW version 20.06.4 (Oxford Nanopore Technologies, Oxford, UK), while quality control and taxonomic classification were conducted via the EPI2ME platform (v.2.11.0, WIMP pipeline).

Briefly, each sample contained 5 ng of DNA in 3 μL of nuclease-free water. DNA tagmentation was performed in a single step by incubating the samples at 30 °C and 80 °C for 1 min each in the fragmentation mix (FRM). For 4 ng of tagmented DNA, 1 μL of Rapid Barcode Primer (10 μM; Oxford Nanopore Technologies, Oxford, UK) and 25 μL of LongAmp^®^ Hot Start Taq 2× Master Mix (New England Biolabs, Ipswich, MA, USA), and nuclease-free water were added to reach a final volume of 50 µL.

The prepared mix was amplified under the following cycling conditions: initial denaturation at 95 °C for 3 min, followed by 14 cycles of 95 °C for 15 s, 56 °C for 15 s, 65 °C for 6 min, and a final extension at 65 °C for 6 min. The amplified DNA was then purified using Agencourt AMPure XP beads (Beckman Coulter Life Sciences, Indianapolis, IN, USA) (1 × concentration) and eluted in 10 μL of freshly prepared T50 buffer. DNA concentration was measured using a Qubit™ 3 Fluorometer (Thermo Fisher Scientific, Waltham, MA, US), and fragment size distribution was assessed with a 2100 Bioanalyzer (Agilent Technologies, Santa Clara, CA, USA) using the DNA 12000 Kit (Agilent Technologies, Santa Clara, CA, USA) according to the manufacturer’s instructions.

Barcoded DNA was pooled to a final concentration of 100 fmol in 10 μL of T50 Buffer (Oxford Nanopore Technologies, Oxford, UK). Adapter ligation was performed by adding 1 µL of Rapid Adapter (Oxford Nanopore Technologies, Oxford, UK).) and incubating for 5 min at room temperature. Library preparation and sequencing were carried out on a MinION Flow Cell R10.4.1 (FLO-MIN114; Oxford Nanopore Technologies, Oxford, UK) connected to a MinION Mk1B device (Oxford Nanopore Technologies, Oxford, UK), following the manufacturer’s instructions. The sequencing run lasted up to 48 h using MinKNOW in live basecalling mode with default parameters, generating barcoded FASTQ files. Sequencing data were deposited in the NCBI SRA database under PRJNA1246956. Metagenomic analysis was performed using the EPI2ME Desktop Bioinformatics Tool version 5.2.3. The study reports, generated as a wf-metagenomics-report.html file, reporting sequencing information such as average reads/bases per sample, read length distribution and others, are available as Supplementary File S1. Patient metadata and analyzed count matrices are available in Supplementary File S2.

Negative extraction controls (blank samples) were included in all sequencing runs except for Run V, due to logistical constraints. These blanks contained very low microbial read counts and were therefore excluded from the downstream analysis. Running the prevalence method of the Decontam R package (v. 1.30.0), we did not identify contaminants.

### 4.6. Bioinformatics and Statistical Analysis

Given the pilot nature of the study and the limited sample size, all inferential analyses should be considered exploratory and hypothesis-generating. ONT is a portable DNA sequencing device that connects directly to a laptop via USB. The device contains an array of protein nanopores immersed in a high-salt buffer. During sequencing, an electrical potential is applied, generating an ionic current through the nanopores. As each DNA strand passes through a nanopore, the corresponding current signal is recorded and saved as an individual file on the connected computer. The MinKNOW software (version 20.06.4, Oxford Nanopore Technologies, Oxford, UK), monitors this folder and performs local basecalling.

Processed reads can then be automatically uploaded to the EPI2ME cloud platform for downstream taxonomic classification through the what’s in my pot (WIMP) workflow available through the EPI2ME cloud platform (Oxford Nanopore Technologies, Oxford, UK; accessed June 2021).

WIMP identifies microbial species in real time using a pre-built data structure shared across all runs. This structure is based on taxonomy and a reference database. It links every 24-mer in the database to a specific node in the NCBI taxonomy tree [31].

Thanks to this pre-processing, new reads can be classified quickly by matching their kmers to the pre-built structure, instead of aligning them to the full reference sequences. During sequencing, the Metrichor agent uploads reads, performs basecalling, and classifies each read using the WIMP model [13]. WIMP relies on the Kraken algorithm to map kmers, compute the Least Common Ancestor (LCA), and determine the most likely taxonomic placement. Each placement receives a classification score, which represents the fraction of kmers assigned to the LCA within the corresponding taxonomic clade. A higher score indicates a more confident classification [31]. The WIMP bacterial, viral, and fungal identification module uses a reference database including all RefSeq genomes from these domains. Organisms can be identified to the subspecies or strain level only if that level is represented in the database. If not, the report assigns them to a higher taxonomic rank, such as species or genus [31]. To prevent the batch effect from influencing the clustering analysis results, the row count matrix was transformed using the ConQuR R package (v. 2.0) [32]. Hierarchical clustering was performed on the batch-adjusted count matrix. The Bray–Curtis metric was used to calculate the distance among patients, and the Ward-D2 aggregation method was used to build the dendrogram through the function hclust from the stats R package (v. 4.50). Specific taxa associated with each study group were first identified using Venn diagrams to visualize shared and unique taxa across conditions. Subsequently, an enrichment analysis based on Fisher’s exact test was performed to assess whether the occurrence of specific taxa in post-surgical compared with pre-surgical OSCC samples was statistically significant. The microbiome differential abundance analysis between pre- and post-treatment groups was performed by applying the ancombc2 function of the ANCOMBC R package (v. 3.22) to the original count matrix [33]. To account for the presence of batch effects and paired samples, the patient group and batch variable were considered as fixed effects, while the patients were considered as random effects. *p*-values from the differential abundance analysis were adjusted for multiple comparisons using the Benjamini–Hochberg method, and adjusted *p*-values < 0.05 were considered statistically significant. The algorithm performs an automatic sensitivity analysis. Taxa that do not pass this analysis are marked in the “passed_ss” column as FALSE and are treated as non-significant. The log-abundance matrix generated by the ANCOM-BC software (v. 3.22) was examined to verify the absence of anomalies in the algorithm’s output. Alpha diversity was assessed by calculating three commonly used indices: richness, Shannon’s index, and inverse Simpson’s index. The indices were computed manually based on the batch-adjusted relative abundance tables and visualized using boxplots. Differences in diversity between groups were evaluated using a bootstrap *t*-test, which accounted for the limited sample size and non-normal data distribution, and *p*-values < 0.05 were considered statistically significant. Analyses were performed at multiple taxonomic levels, including species, genus, family, class, and phylum, to provide a comprehensive assessment of microbial community composition. Results obtained at each level are reported in the Supplementary Files, offering a detailed and interpretable description of differential abundance patterns across taxa. All statistical analyses and graphical representations have been performed using R statistical software v4.5.0.

Comparisons involving the OSCC-free reference cohort were interpreted descriptively because the cohort included both healthy individuals and patients with OPMD, resulting in a biologically heterogeneous reference population. Consequently, the primary analytical focus was placed on the longitudinal within-patient comparison between pre- and post-surgical samples.

Because several clinically relevant variables potentially influencing oral microbiota composition (including perioperative antibiotic exposure, postoperative complications, oral hygiene modifications, dietary changes, salivary flow alterations, and Candida colonization) were not systematically available, these factors were not included as covariates in the statistical models. Consequently, the analyses were designed to characterize longitudinal microbiome dynamics rather than establish causal relationships between surgery and microbiome changes.

## 5. Conclusions

This exploratory longitudinal pilot study characterized salivary microbiota profiles in patients with OSCC before and after tumor resection using Oxford Nanopore sequencing. Differences in microbial composition and alpha diversity were observed between the two sampling time points, indicating longitudinal microbiome dynamics during the perioperative period. However, given the limited sample size and the absence of detailed perioperative, oral health, and lifestyle-related variables, the relative contribution of surgical treatment and other potential confounding factors could not be determined.

Therefore, these findings should be considered hypothesis-generating rather than evidence of causal microbiome changes attributable to surgery. Larger multicenter studies incorporating standardized clinical, oral health, and perioperative data collection are needed to clarify the biological significance and potential clinical relevance of these observations.

## Figures and Tables

**Table 1:** Demographic and clinical characteristics of the study population. The table includes patients with oral squamous cell carcinoma (OSCC) and the OSCC-free reference cohort (healthy individuals and patients with oral potentially malignant disorders). Values are presented as frequencies (n, %) or mean ± standard deviation, as appropriate.

Characteristic	Group		
	**OSCCs**	**Healthy cases**	**OPMDs**
	(*n* = 16)	(*n* = 5)	(*n* = 5)
**Gender**			
Male	8 (50%)	2 (40%)	3 (60%)
Female	8 (50%)	3 (60%)	2 (40%)
**Age distribution**			
Median	61	63	76
Q1–Q3	55–74	35–75	67–80
Mean ± SD	63.2 ± 12.9	55.8 ± 22.5	70.2 ± 15.3
**Risk factors**			
Tobacco smoking	4 (25.0%)	0	1 (20%)
Tobacco + alcohol	2 (12.5%)	0	0
Former smoking	3 (18.7%)	0	0
	**OSCC cases**		
	(n = 16)		
**OSCC anatomical site**			
Border of the tongue	9 (56.3%)		
Buccal mucosa	5 (31.3%)		
Upper gingiva	1 (6.2%)		
Hard palate	1 (6.2%)		
**TNM staging**			
Stage I	3 (18.8%)		
Stage II	6 (37.5%)		
Stage III	4 (25%)		
Stage IV	3 (18.8%)		

**Table 2:** Differentially abundant bacterial taxa identified between OSCC post-surgical and pre-surgical patients. Positive logFC values indicate taxa more abundant in post-surgical patients, while negative logFC values indicate taxa more abundant in pre-surgical patients. Columns “n.total”, “n.group”, and “prop.group” report, respectively: the total number of patients in which the taxon is detected, the number of patients in the group where the taxon is more abundant, and the corresponding proportion. In the upper panel (pre-surgical UP), “n.group” and “prop.group” refer to pre-surgical patients; in the lower panel (post-surgical UP), they refer to post-surgical patients.

	Species	n.total	n.group	prop.group	logFC	adj. *p*-value
**post-surgery UP**	*Glaesserella parasuis*	8	4	0.25	0.87	0.004
	*Mogibacterium neglectum*	11	4	0.25	0.47	0.0048
	*Segatella oris*	25	10	0.62	1.57	0.0064
	*Prevotella herbatica*	11	5	0.31	3.80	0.013
	*Streptococcus parasuis*	16	8	0.5	0.97	0.018
	*Streptomyces anulatus*	16	6	0.38	1.46	0.019
	*Leptotrichia trevisanii*	21	11	0.69	1.56	0.035
	*Escherichia coli*	11	4	0.25	1.37	0.041
**pre-s. UP**	*Neisseria subflava*	28	14	0.88	−1.98	0.018
	*Treponema* sp. *OMZ 838*	11	5	0.31	−0.87	0.029
	*Leptotrichia buccalis*	18	8	0.5	−1.04	0.032
	*Veillonella* sp. *S12025-13*	20	10	0.62	−2.62	0.032
	*Candidatus Minimicrobia vallesae*	11	6	0.38	−2.20	0.041

## Data Availability

The original contributions presented in this study are included in the article/Supplementary Materials. Specifically, the WIMP output and the taxa row count data are available in the Supplementary Materials. Further inquiries can be directed to the corresponding author.

## Supplementary Materials

The following supporting information can be downloaded at https://www.mdpi.com/article/10.3390/ijms27156873/s1.
