# Supplementary material for: Longitudinal Exploratory Analysis of Salivary Microbiota Profiles in Patients with Oral Squamous Cell Carcinoma Before and After Surgery: A Pilot Study
Source: Int J Mol Sci. 2026 Jul 31;27(15):6873. doi: 10.3390/ijms27156873 (PMC13467391; doi:10.3390/ijms27156873)
Supplement: Supplementary file 1 [file ijms-27-06873-s001.zip › ijms-4454609-Supplementary Figures S1 & S2.pdf]

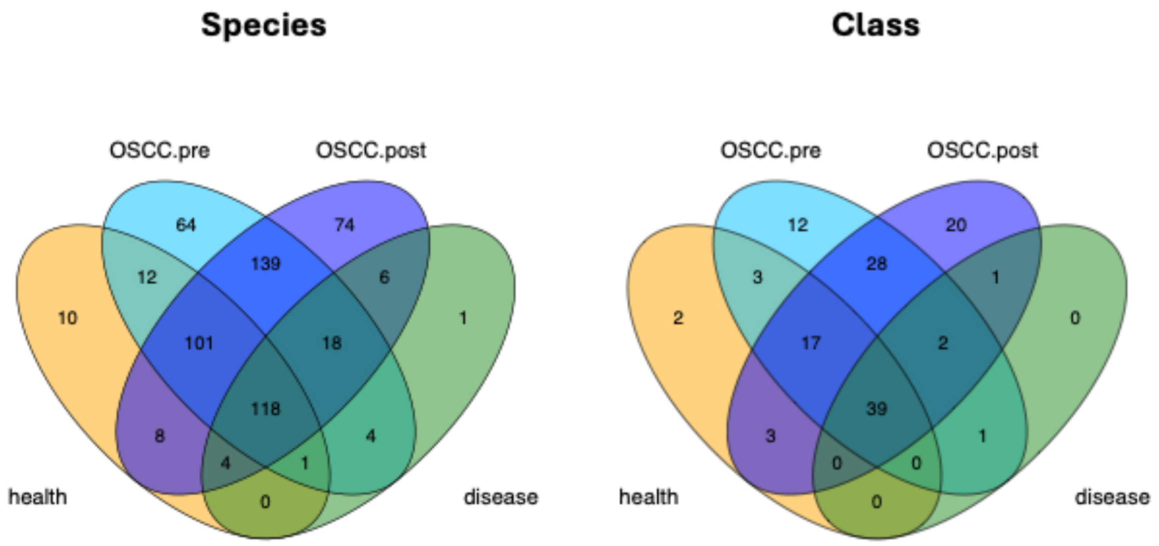

(**a**) (**b**)

Supplementary Figure S1. (**a**) Venn diagram showing the overlap of species among the four study groups. Areas shared by multiple groups indicate the number of species present in all corresponding groups, while non-overlapping areas indicate species unique to a single group. (**b**) Venn diagram showing the overlap of classes among the four study groups, with overlapping areas indicating the number of classes shared across groups and non-overlapping areas indicating unique classes.

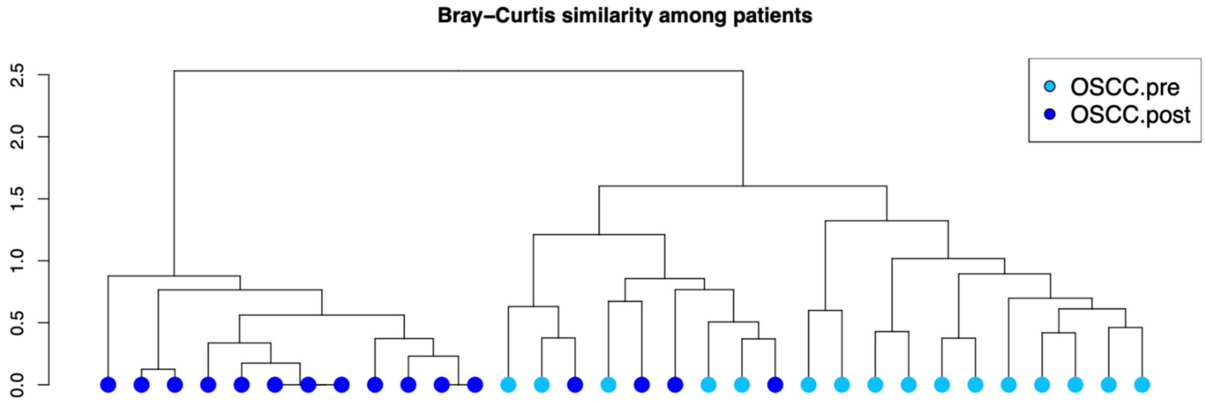

Supplementary Figure S2. Hierarchical clustering of OSCC patients based on the abundance of taxa identified as significantly different between pre- and post-treatment samples (ANCOM-BC). As these taxa were selected based on differential abundance analysis, this clustering represents an exploratory, post hoc visualization aimed at illustrating their ability to separate pre- and post-treatment samples.
