# Supplementary material for: Longitudinal Exploratory Analysis of Salivary Microbiota Profiles in Patients with Oral Squamous Cell Carcinoma Before and After Surgery: A Pilot Study
Source: Int J Mol Sci. 2026 Jul 31;27(15):6873. doi: 10.3390/ijms27156873 (PMC13467391; doi:10.3390/ijms27156873)
Supplement: Supplementary file 1 [file ijms-27-06873-s001.zip › ijms-4454609-Supplementary File S1.html]

wf-metagenomics Sequencing Report


Jump to section... 

Read summaryReadsLineagesSunburstTaxonomyAbundancesDiversity

VersionsParameters

# wf-metagenomics Sequencing Report

Results generated through the wf-metagenomics nextflow workflow provided by Oxford Nanopore Technologies.

Research use only
2025-01-23
v2.11.0


## Read summary

- Dropdown
  - barcode05
  - barcode04
  - barcode10
  - unclassified
  - barcode02
  - barcode01
  - barcode03
  - barcode07
  - barcode06
  - barcode08
  - barcode09
  - barcode12
  - barcode11

## Number of reads

Number of reads after applying read length and quality filters.
Read counts will also reflect host depletion and unclassified
reads (kraken2 approach) and unmapped reads (minimap2 approach).
Percentages are calculated from reads after the filtering.

Note that in the minimap2 approach, there are two filters
based on the mapping identity and coverage that can increase
the number of unclassified sequences. This table shows the
unmapped reads before both filters are applied.

| index | Reads | Unclassified\|Unmapped | Unclassified\|Unmapped (%) |
| --- | --- | --- | --- |
| barcode01 | 6536.0 | 1409.0 | 21.56 |
| barcode02 | 6085.0 | 1058.0 | 17.39 |
| barcode03 | 6475.0 | 1350.0 | 20.85 |
| barcode04 | 10805.0 | 2756.0 | 25.51 |
| barcode05 | 9815.0 | 1867.0 | 19.02 |
| barcode06 | 7871.0 | 2605.0 | 33.1 |
| barcode07 | 17241.0 | 1040.0 | 6.03 |
| barcode08 | 23574.0 | 1418.0 | 6.02 |
| barcode09 | 15731.0 | 519.0 | 3.3 |
| barcode10 | 11078.0 | 1446.0 | 13.05 |
| barcode11 | 13314.0 | 1552.0 | 11.66 |
| barcode12 | 13971.0 | 1325.0 | 9.48 |
| unclassified | 74166.0 | 24901.0 | 33.57 |

## Lineages

- Select sample
- Select rank
- Select cutoff
- [+] Zoom in
- [-] Zoom out
- [ ] Reset zoom

## Sunburst

- Sample
  - barcode01
  - barcode02
  - barcode03
  - barcode04
  - barcode05
  - barcode06
  - barcode07
  - barcode08
  - barcode09
  - barcode10
  - barcode11
  - barcode12
  - unclassified

This visualization can be useful to interactively explore the
taxonomic composition of the sample. It shows hierarchical data:
each layer represents a taxonomic rank. The color indicates the
abundance of that taxon in comparison with the total number of
reads. Zooming into an specific taxon is possible by clicking on it.

This visualization can be useful to interactively explore the
taxonomic composition of the sample. It shows hierarchical data:
each layer represents a taxonomic rank. The color indicates the
abundance of that taxon in comparison with the total number of
reads. Zooming into an specific taxon is possible by clicking on it.

This visualization can be useful to interactively explore the
taxonomic composition of the sample. It shows hierarchical data:
each layer represents a taxonomic rank. The color indicates the
abundance of that taxon in comparison with the total number of
reads. Zooming into an specific taxon is possible by clicking on it.

This visualization can be useful to interactively explore the
taxonomic composition of the sample. It shows hierarchical data:
each layer represents a taxonomic rank. The color indicates the
abundance of that taxon in comparison with the total number of
reads. Zooming into an specific taxon is possible by clicking on it.

This visualization can be useful to interactively explore the
taxonomic composition of the sample. It shows hierarchical data:
each layer represents a taxonomic rank. The color indicates the
abundance of that taxon in comparison with the total number of
reads. Zooming into an specific taxon is possible by clicking on it.

This visualization can be useful to interactively explore the
taxonomic composition of the sample. It shows hierarchical data:
each layer represents a taxonomic rank. The color indicates the
abundance of that taxon in comparison with the total number of
reads. Zooming into an specific taxon is possible by clicking on it.

This visualization can be useful to interactively explore the
taxonomic composition of the sample. It shows hierarchical data:
each layer represents a taxonomic rank. The color indicates the
abundance of that taxon in comparison with the total number of
reads. Zooming into an specific taxon is possible by clicking on it.

This visualization can be useful to interactively explore the
taxonomic composition of the sample. It shows hierarchical data:
each layer represents a taxonomic rank. The color indicates the
abundance of that taxon in comparison with the total number of
reads. Zooming into an specific taxon is possible by clicking on it.

This visualization can be useful to interactively explore the
taxonomic composition of the sample. It shows hierarchical data:
each layer represents a taxonomic rank. The color indicates the
abundance of that taxon in comparison with the total number of
reads. Zooming into an specific taxon is possible by clicking on it.

This visualization can be useful to interactively explore the
taxonomic composition of the sample. It shows hierarchical data:
each layer represents a taxonomic rank. The color indicates the
abundance of that taxon in comparison with the total number of
reads. Zooming into an specific taxon is possible by clicking on it.

This visualization can be useful to interactively explore the
taxonomic composition of the sample. It shows hierarchical data:
each layer represents a taxonomic rank. The color indicates the
abundance of that taxon in comparison with the total number of
reads. Zooming into an specific taxon is possible by clicking on it.

This visualization can be useful to interactively explore the
taxonomic composition of the sample. It shows hierarchical data:
each layer represents a taxonomic rank. The color indicates the
abundance of that taxon in comparison with the total number of
reads. Zooming into an specific taxon is possible by clicking on it.

This visualization can be useful to interactively explore the
taxonomic composition of the sample. It shows hierarchical data:
each layer represents a taxonomic rank. The color indicates the
abundance of that taxon in comparison with the total number of
reads. Zooming into an specific taxon is possible by clicking on it.

## Taxonomy

- Rank
  - phylum
  - class
  - order
  - family
  - genus
  - species

Barplot of the 9 most abundant taxa at the
phylum rank in all the samples.
Any remaining taxa have been collapsed under the 'Other' category
to facilitate the visualization.
The y-axis indicates the relative abundance of each taxon
in percentages for each sample.

Barplot of the 9 most abundant taxa at the
class rank in all the samples.
Any remaining taxa have been collapsed under the 'Other' category
to facilitate the visualization.
The y-axis indicates the relative abundance of each taxon
in percentages for each sample.

Barplot of the 9 most abundant taxa at the
order rank in all the samples.
Any remaining taxa have been collapsed under the 'Other' category
to facilitate the visualization.
The y-axis indicates the relative abundance of each taxon
in percentages for each sample.

Barplot of the 9 most abundant taxa at the
family rank in all the samples.
Any remaining taxa have been collapsed under the 'Other' category
to facilitate the visualization.
The y-axis indicates the relative abundance of each taxon
in percentages for each sample.

Barplot of the 9 most abundant taxa at the
genus rank in all the samples.
Any remaining taxa have been collapsed under the 'Other' category
to facilitate the visualization.
The y-axis indicates the relative abundance of each taxon
in percentages for each sample.

Barplot of the 9 most abundant taxa at the
species rank in all the samples.
Any remaining taxa have been collapsed under the 'Other' category
to facilitate the visualization.
The y-axis indicates the relative abundance of each taxon
in percentages for each sample.

## Abundances

- Abundance tables
  - phylum
  - class
  - order
  - family
  - genus
  - species
- Rarefied Abundance tables
  - phylum
  - class
  - order
  - family
  - genus
  - species

Abundance table for the phylum rank.

Only taxa whose global abundance are above the
`abundance_threshold` parameter will appear in the table.

| phylum | barcode01 | barcode02 | barcode03 | barcode04 | barcode05 | barcode06 | barcode07 | barcode08 | barcode09 | barcode10 | barcode11 | barcode12 | unclassified | total | superkingdom | kingdom | tax |
| --- | --- | --- | --- | --- | --- | --- | --- | --- | --- | --- | --- | --- | --- | --- | --- | --- | --- |
| Euryarchaeota | 1 | 2 | 3 | 1 | 1 | 5 | 2 | 0 | 0 | 1 | 2 | 1 | 5 | 24 | Archaea | Archaea\_none | Archaea;Archaea\_none;Euryarchaeota |
| Nitrososphaerota | 0 | 0 | 0 | 0 | 0 | 1 | 0 | 0 | 0 | 0 | 0 | 0 | 0 | 1 | Archaea | Archaea\_none | Archaea;Archaea\_none;Nitrososphaerota |
| Acidobacteriota | 0 | 0 | 0 | 0 | 0 | 0 | 0 | 0 | 0 | 0 | 0 | 1 | 0 | 1 | Bacteria | Bacteria\_none | Bacteria;Bacteria\_none;Acidobacteriota |
| Actinomycetota | 58 | 146 | 40 | 31 | 144 | 238 | 154 | 81 | 22 | 101 | 212 | 192 | 300 | 1719 | Bacteria | Bacteria\_none | Bacteria;Bacteria\_none;Actinomycetota |
| Bacillota | 3595 | 1795 | 1504 | 888 | 3517 | 3084 | 3090 | 802 | 672 | 1836 | 4389 | 1343 | 7426 | 33941 | Bacteria | Bacteria\_none | Bacteria;Bacteria\_none;Bacillota |
| Bacteroidota | 664 | 1307 | 469 | 1199 | 363 | 431 | 307 | 610 | 341 | 606 | 604 | 477 | 1855 | 9233 | Bacteria | Bacteria\_none | Bacteria;Bacteria\_none;Bacteroidota |
| Balneolota | 0 | 0 | 0 | 1 | 0 | 0 | 0 | 0 | 0 | 0 | 0 | 0 | 0 | 1 | Bacteria | Bacteria\_none | Bacteria;Bacteria\_none;Balneolota |
| Bdellovibrionota | 0 | 0 | 0 | 0 | 0 | 0 | 0 | 0 | 0 | 1 | 0 | 0 | 0 | 1 | Bacteria | Bacteria\_none | Bacteria;Bacteria\_none;Bdellovibrionota |
| Calditrichota | 0 | 0 | 0 | 1 | 0 | 0 | 0 | 0 | 0 | 0 | 0 | 0 | 0 | 1 | Bacteria | Bacteria\_none | Bacteria;Bacteria\_none;Calditrichota |
| Campylobacterota | 1 | 47 | 16 | 11 | 11 | 50 | 2 | 11 | 9 | 12 | 3 | 14 | 61 | 248 | Bacteria | Bacteria\_none | Bacteria;Bacteria\_none;Campylobacterota |
| Candidatus Saccharibacteria | 0 | 8 | 5 | 0 | 13 | 185 | 1 | 2 | 3 | 2 | 94 | 7 | 53 | 373 | Bacteria | Bacteria\_none | Bacteria;Bacteria\_none;Candidatus Saccharibacteria |
| Chlamydiota | 0 | 0 | 0 | 0 | 0 | 0 | 0 | 0 | 0 | 0 | 0 | 0 | 1 | 1 | Bacteria | Bacteria\_none | Bacteria;Bacteria\_none;Chlamydiota |
| Chlorobiota | 0 | 0 | 0 | 0 | 0 | 1 | 0 | 0 | 0 | 0 | 0 | 0 | 0 | 1 | Bacteria | Bacteria\_none | Bacteria;Bacteria\_none;Chlorobiota |
| Chloroflexota | 0 | 0 | 0 | 0 | 0 | 0 | 0 | 0 | 0 | 0 | 1 | 0 | 1 | 2 | Bacteria | Bacteria\_none | Bacteria;Bacteria\_none;Chloroflexota |
| Cyanobacteriota | 1 | 1 | 1 | 1 | 1 | 1 | 1 | 0 | 0 | 2 | 2 | 0 | 3 | 14 | Bacteria | Bacteria\_none | Bacteria;Bacteria\_none;Cyanobacteriota |
| Deferribacterota | 0 | 1 | 0 | 0 | 0 | 0 | 0 | 0 | 0 | 0 | 0 | 0 | 1 | 2 | Bacteria | Bacteria\_none | Bacteria;Bacteria\_none;Deferribacterota |
| Fusobacteriota | 17 | 266 | 237 | 845 | 187 | 258 | 449 | 562 | 24 | 132 | 360 | 130 | 1567 | 5034 | Bacteria | Bacteria\_none | Bacteria;Bacteria\_none;Fusobacteriota |
| Ignavibacteriota | 0 | 0 | 0 | 0 | 0 | 0 | 0 | 0 | 0 | 0 | 0 | 0 | 1 | 1 | Bacteria | Bacteria\_none | Bacteria;Bacteria\_none;Ignavibacteriota |
| Mycoplasmatota | 0 | 11 | 40 | 3 | 5 | 11 | 1 | 3 | 0 | 10 | 4 | 7 | 31 | 126 | Bacteria | Bacteria\_none | Bacteria;Bacteria\_none;Mycoplasmatota |
| Myxococcota | 0 | 0 | 0 | 0 | 2 | 0 | 0 | 0 | 0 | 0 | 1 | 0 | 1 | 4 | Bacteria | Bacteria\_none | Bacteria;Bacteria\_none;Myxococcota |
| Nitrospirota | 0 | 0 | 0 | 0 | 0 | 1 | 0 | 1 | 0 | 0 | 1 | 0 | 0 | 3 | Bacteria | Bacteria\_none | Bacteria;Bacteria\_none;Nitrospirota |
| Planctomycetota | 1 | 0 | 0 | 0 | 0 | 1 | 0 | 0 | 0 | 0 | 2 | 0 | 0 | 4 | Bacteria | Bacteria\_none | Bacteria;Bacteria\_none;Planctomycetota |
| Pseudomonadota | 288 | 356 | 1579 | 4886 | 676 | 606 | 80 | 2159 | 5 | 1787 | 304 | 378 | 3508 | 16612 | Bacteria | Bacteria\_none | Bacteria;Bacteria\_none;Pseudomonadota |
| Spirochaetota | 4 | 74 | 54 | 9 | 40 | 74 | 9 | 112 | 0 | 14 | 56 | 120 | 143 | 709 | Bacteria | Bacteria\_none | Bacteria;Bacteria\_none;Spirochaetota |
| Synergistota | 0 | 0 | 1 | 0 | 0 | 0 | 0 | 0 | 0 | 0 | 1 | 1 | 0 | 3 | Bacteria | Bacteria\_none | Bacteria;Bacteria\_none;Synergistota |
| Thermodesulfobacteriota | 0 | 2 | 2 | 0 | 0 | 2 | 0 | 5 | 0 | 0 | 1 | 1 | 1 | 14 | Bacteria | Bacteria\_none | Bacteria;Bacteria\_none;Thermodesulfobacteriota |
| Thermomicrobiota | 0 | 1 | 0 | 0 | 0 | 0 | 0 | 0 | 0 | 0 | 0 | 0 | 0 | 1 | Bacteria | Bacteria\_none | Bacteria;Bacteria\_none;Thermomicrobiota |
| Thermotogota | 1 | 0 | 0 | 0 | 0 | 0 | 0 | 0 | 0 | 0 | 0 | 0 | 0 | 1 | Bacteria | Bacteria\_none | Bacteria;Bacteria\_none;Thermotogota |
| Verrucomicrobiota | 0 | 1 | 0 | 0 | 0 | 0 | 0 | 0 | 0 | 0 | 0 | 0 | 0 | 1 | Bacteria | Bacteria\_none | Bacteria;Bacteria\_none;Verrucomicrobiota |
| Chordata | 428 | 938 | 1105 | 97 | 2901 | 211 | 12043 | 17730 | 14114 | 5069 | 5655 | 9919 | 34174 | 104384 | Eukaryota | Metazoa | Eukaryota;Metazoa;Chordata |
| Unknown | 1409 | 1058 | 1350 | 2756 | 1867 | 2605 | 1040 | 1418 | 519 | 1446 | 1552 | 1325 | 24901 | 43246 | Unclassified | Unknown | Unclassified;Unknown;Unknown |
| Nucleocytoviricota | 0 | 0 | 0 | 0 | 0 | 0 | 0 | 1 | 0 | 0 | 0 | 1 | 1 | 3 | Viruses | Bamfordvirae | Viruses;Bamfordvirae;Nucleocytoviricota |
| Peploviricota | 8 | 0 | 0 | 0 | 0 | 1 | 0 | 1 | 0 | 0 | 0 | 0 | 2 | 12 | Viruses | Heunggongvirae | Viruses;Heunggongvirae;Peploviricota |
| Uroviricota | 7 | 0 | 0 | 1 | 3 | 1 | 5 | 0 | 0 | 0 | 2 | 1 | 2 | 22 | Viruses | Heunggongvirae | Viruses;Heunggongvirae;Uroviricota |
| Negarnaviricota | 0 | 0 | 0 | 0 | 0 | 0 | 0 | 0 | 0 | 0 | 0 | 0 | 1 | 1 | Viruses | Orthornavirae | Viruses;Orthornavirae;Negarnaviricota |
| Cossaviricota | 0 | 0 | 0 | 0 | 0 | 0 | 0 | 2 | 0 | 0 | 0 | 0 | 0 | 2 | Viruses | Shotokuvirae | Viruses;Shotokuvirae;Cossaviricota |
| Taleaviricota | 0 | 0 | 0 | 0 | 0 | 0 | 0 | 0 | 0 | 0 | 0 | 0 | 1 | 1 | Viruses | Zilligvirae | Viruses;Zilligvirae;Taleaviricota |

Export CSV

Abundance table for the class rank.

Only taxa whose global abundance are above the
`abundance_threshold` parameter will appear in the table.

| class | barcode01 | barcode02 | barcode03 | barcode04 | barcode05 | barcode06 | barcode07 | barcode08 | barcode09 | barcode10 | barcode11 | barcode12 | unclassified | total | superkingdom | kingdom | phylum | tax |
| --- | --- | --- | --- | --- | --- | --- | --- | --- | --- | --- | --- | --- | --- | --- | --- | --- | --- | --- |
| Halobacteria | 1 | 1 | 0 | 0 | 0 | 1 | 1 | 0 | 0 | 1 | 1 | 0 | 1 | 7 | Archaea | Archaea\_none | Euryarchaeota | Archaea;Archaea\_none;Euryarchaeota;Halobacteria |
| Methanobacteria | 0 | 0 | 2 | 0 | 0 | 1 | 1 | 0 | 0 | 0 | 0 | 0 | 1 | 5 | Archaea | Archaea\_none | Euryarchaeota | Archaea;Archaea\_none;Euryarchaeota;Methanobacteria |
| Methanococci | 0 | 0 | 1 | 0 | 0 | 0 | 0 | 0 | 0 | 0 | 0 | 0 | 1 | 2 | Archaea | Archaea\_none | Euryarchaeota | Archaea;Archaea\_none;Euryarchaeota;Methanococci |
| Methanomicrobia | 0 | 1 | 0 | 1 | 1 | 3 | 0 | 0 | 0 | 0 | 1 | 0 | 2 | 9 | Archaea | Archaea\_none | Euryarchaeota | Archaea;Archaea\_none;Euryarchaeota;Methanomicrobia |
| Thermococci | 0 | 0 | 0 | 0 | 0 | 0 | 0 | 0 | 0 | 0 | 0 | 1 | 0 | 1 | Archaea | Archaea\_none | Euryarchaeota | Archaea;Archaea\_none;Euryarchaeota;Thermococci |
| Nitrososphaerota\_Incertae\_sedis | 0 | 0 | 0 | 0 | 0 | 1 | 0 | 0 | 0 | 0 | 0 | 0 | 0 | 1 | Archaea | Archaea\_none | Nitrososphaerota | Archaea;Archaea\_none;Nitrososphaerota;Nitrososphaerota\_Incertae\_sedis |
| Terriglobia | 0 | 0 | 0 | 0 | 0 | 0 | 0 | 0 | 0 | 0 | 0 | 1 | 0 | 1 | Bacteria | Bacteria\_none | Acidobacteriota | Bacteria;Bacteria\_none;Acidobacteriota;Terriglobia |
| Acidimicrobiia | 0 | 0 | 0 | 0 | 0 | 0 | 0 | 1 | 0 | 0 | 0 | 0 | 0 | 1 | Bacteria | Bacteria\_none | Actinomycetota | Bacteria;Bacteria\_none;Actinomycetota;Acidimicrobiia |
| Actinomycetes | 30 | 139 | 30 | 31 | 141 | 103 | 91 | 73 | 21 | 101 | 205 | 188 | 265 | 1418 | Bacteria | Bacteria\_none | Actinomycetota | Bacteria;Bacteria\_none;Actinomycetota;Actinomycetes |
| Coriobacteriia | 28 | 5 | 10 | 0 | 2 | 135 | 63 | 7 | 1 | 0 | 7 | 4 | 35 | 297 | Bacteria | Bacteria\_none | Actinomycetota | Bacteria;Bacteria\_none;Actinomycetota;Coriobacteriia |
| Rubrobacteria | 0 | 0 | 0 | 0 | 1 | 0 | 0 | 0 | 0 | 0 | 0 | 0 | 0 | 1 | Bacteria | Bacteria\_none | Actinomycetota | Bacteria;Bacteria\_none;Actinomycetota;Rubrobacteria |
| Thermoleophilia | 0 | 2 | 0 | 0 | 0 | 0 | 0 | 0 | 0 | 0 | 0 | 0 | 0 | 2 | Bacteria | Bacteria\_none | Actinomycetota | Bacteria;Bacteria\_none;Actinomycetota;Thermoleophilia |
| Bacilli | 2374 | 1579 | 1247 | 722 | 3339 | 2295 | 2962 | 569 | 592 | 1668 | 4255 | 1147 | 6321 | 29070 | Bacteria | Bacteria\_none | Bacillota | Bacteria;Bacteria\_none;Bacillota;Bacilli |
| Bacillota\_Incertae\_sedis | 0 | 0 | 0 | 0 | 1 | 0 | 0 | 0 | 0 | 0 | 0 | 0 | 0 | 1 | Bacteria | Bacteria\_none | Bacillota | Bacteria;Bacteria\_none;Bacillota;Bacillota\_Incertae\_sedis |
| Clostridia | 10 | 80 | 81 | 32 | 32 | 127 | 27 | 64 | 8 | 13 | 21 | 31 | 127 | 653 | Bacteria | Bacteria\_none | Bacillota | Bacteria;Bacteria\_none;Bacillota;Clostridia |
| Erysipelotrichia | 14 | 17 | 92 | 10 | 16 | 523 | 49 | 21 | 1 | 8 | 10 | 2 | 154 | 917 | Bacteria | Bacteria\_none | Bacillota | Bacteria;Bacteria\_none;Bacillota;Erysipelotrichia |
| Negativicutes | 1191 | 85 | 59 | 107 | 78 | 67 | 45 | 83 | 71 | 145 | 18 | 136 | 671 | 2756 | Bacteria | Bacteria\_none | Bacillota | Bacteria;Bacteria\_none;Bacillota;Negativicutes |
| Tissierellia | 6 | 34 | 25 | 17 | 51 | 72 | 7 | 65 | 0 | 2 | 85 | 27 | 153 | 544 | Bacteria | Bacteria\_none | Bacillota | Bacteria;Bacteria\_none;Bacillota;Tissierellia |
| Bacteroidia | 346 | 1152 | 333 | 970 | 169 | 386 | 222 | 226 | 341 | 240 | 377 | 419 | 1262 | 6443 | Bacteria | Bacteria\_none | Bacteroidota | Bacteria;Bacteria\_none;Bacteroidota;Bacteroidia |
| Chitinophagia | 0 | 1 | 0 | 2 | 2 | 1 | 0 | 0 | 0 | 1 | 0 | 1 | 0 | 8 | Bacteria | Bacteria\_none | Bacteroidota | Bacteria;Bacteria\_none;Bacteroidota;Chitinophagia |
| Cytophagia | 1 | 0 | 1 | 3 | 5 | 2 | 1 | 1 | 0 | 1 | 2 | 0 | 8 | 25 | Bacteria | Bacteria\_none | Bacteroidota | Bacteria;Bacteria\_none;Bacteroidota;Cytophagia |
| Flavobacteriia | 317 | 152 | 135 | 220 | 186 | 41 | 84 | 382 | 0 | 363 | 222 | 56 | 582 | 2740 | Bacteria | Bacteria\_none | Bacteroidota | Bacteria;Bacteria\_none;Bacteroidota;Flavobacteriia |
| Saprospiria | 0 | 0 | 0 | 0 | 0 | 0 | 0 | 0 | 0 | 0 | 0 | 0 | 1 | 1 | Bacteria | Bacteria\_none | Bacteroidota | Bacteria;Bacteria\_none;Bacteroidota;Saprospiria |
| Sphingobacteriia | 0 | 2 | 0 | 4 | 1 | 1 | 0 | 1 | 0 | 1 | 3 | 1 | 2 | 16 | Bacteria | Bacteria\_none | Bacteroidota | Bacteria;Bacteria\_none;Bacteroidota;Sphingobacteriia |
| Balneolia | 0 | 0 | 0 | 1 | 0 | 0 | 0 | 0 | 0 | 0 | 0 | 0 | 0 | 1 | Bacteria | Bacteria\_none | Balneolota | Bacteria;Bacteria\_none;Balneolota;Balneolia |
| Bdellovibrionia | 0 | 0 | 0 | 0 | 0 | 0 | 0 | 0 | 0 | 1 | 0 | 0 | 0 | 1 | Bacteria | Bacteria\_none | Bdellovibrionota | Bacteria;Bacteria\_none;Bdellovibrionota;Bdellovibrionia |
| Calditrichia | 0 | 0 | 0 | 1 | 0 | 0 | 0 | 0 | 0 | 0 | 0 | 0 | 0 | 1 | Bacteria | Bacteria\_none | Calditrichota | Bacteria;Bacteria\_none;Calditrichota;Calditrichia |
| Epsilonproteobacteria | 1 | 47 | 16 | 11 | 11 | 50 | 2 | 11 | 9 | 12 | 3 | 14 | 61 | 248 | Bacteria | Bacteria\_none | Campylobacterota | Bacteria;Bacteria\_none;Campylobacterota;Epsilonproteobacteria |
| Candidatus Saccharibacteria\_Incertae\_sedis | 0 | 0 | 0 | 0 | 2 | 20 | 0 | 0 | 0 | 0 | 1 | 1 | 5 | 29 | Bacteria | Bacteria\_none | Candidatus Saccharibacteria | Bacteria;Bacteria\_none;Candidatus Saccharibacteria;Candidatus Saccharibacteria\_Incertae\_sedis |
| Candidatus Saccharimonadia | 0 | 8 | 5 | 0 | 11 | 165 | 1 | 2 | 3 | 2 | 93 | 6 | 48 | 344 | Bacteria | Bacteria\_none | Candidatus Saccharibacteria | Bacteria;Bacteria\_none;Candidatus Saccharibacteria;Candidatus Saccharimonadia |
| Chlamydiia | 0 | 0 | 0 | 0 | 0 | 0 | 0 | 0 | 0 | 0 | 0 | 0 | 1 | 1 | Bacteria | Bacteria\_none | Chlamydiota | Bacteria;Bacteria\_none;Chlamydiota;Chlamydiia |
| Chlorobiia | 0 | 0 | 0 | 0 | 0 | 1 | 0 | 0 | 0 | 0 | 0 | 0 | 0 | 1 | Bacteria | Bacteria\_none | Chlorobiota | Bacteria;Bacteria\_none;Chlorobiota;Chlorobiia |
| Chloroflexia | 0 | 0 | 0 | 0 | 0 | 0 | 0 | 0 | 0 | 0 | 1 | 0 | 0 | 1 | Bacteria | Bacteria\_none | Chloroflexota | Bacteria;Bacteria\_none;Chloroflexota;Chloroflexia |
| Dehalococcoidia | 0 | 0 | 0 | 0 | 0 | 0 | 0 | 0 | 0 | 0 | 0 | 0 | 1 | 1 | Bacteria | Bacteria\_none | Chloroflexota | Bacteria;Bacteria\_none;Chloroflexota;Dehalococcoidia |
| Cyanobacteriota\_Incertae\_sedis | 1 | 0 | 0 | 0 | 0 | 0 | 0 | 0 | 0 | 0 | 0 | 0 | 0 | 1 | Bacteria | Bacteria\_none | Cyanobacteriota | Bacteria;Bacteria\_none;Cyanobacteriota;Cyanobacteriota\_Incertae\_sedis |
| Cyanophyceae | 0 | 1 | 1 | 1 | 1 | 1 | 1 | 0 | 0 | 2 | 2 | 0 | 3 | 13 | Bacteria | Bacteria\_none | Cyanobacteriota | Bacteria;Bacteria\_none;Cyanobacteriota;Cyanophyceae |
| Deferribacteres | 0 | 1 | 0 | 0 | 0 | 0 | 0 | 0 | 0 | 0 | 0 | 0 | 1 | 2 | Bacteria | Bacteria\_none | Deferribacterota | Bacteria;Bacteria\_none;Deferribacterota;Deferribacteres |
| Fusobacteriia | 17 | 266 | 237 | 845 | 187 | 258 | 449 | 562 | 24 | 132 | 360 | 130 | 1567 | 5034 | Bacteria | Bacteria\_none | Fusobacteriota | Bacteria;Bacteria\_none;Fusobacteriota;Fusobacteriia |
| Ignavibacteria | 0 | 0 | 0 | 0 | 0 | 0 | 0 | 0 | 0 | 0 | 0 | 0 | 1 | 1 | Bacteria | Bacteria\_none | Ignavibacteriota | Bacteria;Bacteria\_none;Ignavibacteriota;Ignavibacteria |
| Mollicutes | 0 | 1 | 3 | 1 | 2 | 5 | 0 | 0 | 0 | 1 | 1 | 0 | 4 | 18 | Bacteria | Bacteria\_none | Mycoplasmatota | Bacteria;Bacteria\_none;Mycoplasmatota;Mollicutes |
| Mycoplasmatota\_Incertae\_sedis | 0 | 10 | 37 | 2 | 3 | 6 | 1 | 3 | 0 | 9 | 3 | 7 | 27 | 108 | Bacteria | Bacteria\_none | Mycoplasmatota | Bacteria;Bacteria\_none;Mycoplasmatota;Mycoplasmatota\_Incertae\_sedis |
| Myxococcia | 0 | 0 | 0 | 0 | 0 | 0 | 0 | 0 | 0 | 0 | 0 | 0 | 1 | 1 | Bacteria | Bacteria\_none | Myxococcota | Bacteria;Bacteria\_none;Myxococcota;Myxococcia |
| Myxococcota\_Incertae\_sedis | 0 | 0 | 0 | 0 | 2 | 0 | 0 | 0 | 0 | 0 | 1 | 0 | 0 | 3 | Bacteria | Bacteria\_none | Myxococcota | Bacteria;Bacteria\_none;Myxococcota;Myxococcota\_Incertae\_sedis |
| Nitrospiria | 0 | 0 | 0 | 0 | 0 | 1 | 0 | 1 | 0 | 0 | 0 | 0 | 0 | 2 | Bacteria | Bacteria\_none | Nitrospirota | Bacteria;Bacteria\_none;Nitrospirota;Nitrospiria |
| Thermodesulfovibrionia | 0 | 0 | 0 | 0 | 0 | 0 | 0 | 0 | 0 | 0 | 1 | 0 | 0 | 1 | Bacteria | Bacteria\_none | Nitrospirota | Bacteria;Bacteria\_none;Nitrospirota;Thermodesulfovibrionia |
| Planctomycetia | 1 | 0 | 0 | 0 | 0 | 1 | 0 | 0 | 0 | 0 | 2 | 0 | 0 | 4 | Bacteria | Bacteria\_none | Planctomycetota | Bacteria;Bacteria\_none;Planctomycetota;Planctomycetia |
| Alphaproteobacteria | 3 | 3 | 5 | 3 | 4 | 8 | 4 | 5 | 2 | 1 | 11 | 1 | 13 | 63 | Bacteria | Bacteria\_none | Pseudomonadota | Bacteria;Bacteria\_none;Pseudomonadota;Alphaproteobacteria |
| Betaproteobacteria | 206 | 203 | 1088 | 4097 | 366 | 332 | 66 | 198 | 2 | 1686 | 224 | 124 | 2208 | 10800 | Bacteria | Bacteria\_none | Pseudomonadota | Bacteria;Bacteria\_none;Pseudomonadota;Betaproteobacteria |
| Gammaproteobacteria | 79 | 150 | 486 | 786 | 306 | 266 | 10 | 1956 | 1 | 100 | 69 | 253 | 1287 | 5749 | Bacteria | Bacteria\_none | Pseudomonadota | Bacteria;Bacteria\_none;Pseudomonadota;Gammaproteobacteria |
| Spirochaetia | 4 | 74 | 54 | 9 | 40 | 74 | 9 | 112 | 0 | 14 | 56 | 120 | 143 | 709 | Bacteria | Bacteria\_none | Spirochaetota | Bacteria;Bacteria\_none;Spirochaetota;Spirochaetia |
| Synergistia | 0 | 0 | 1 | 0 | 0 | 0 | 0 | 0 | 0 | 0 | 1 | 1 | 0 | 3 | Bacteria | Bacteria\_none | Synergistota | Bacteria;Bacteria\_none;Synergistota;Synergistia |
| Candidatus Desulfofervidia | 0 | 0 | 0 | 0 | 0 | 1 | 0 | 0 | 0 | 0 | 0 | 0 | 0 | 1 | Bacteria | Bacteria\_none | Thermodesulfobacteriota | Bacteria;Bacteria\_none;Thermodesulfobacteriota;Candidatus Desulfofervidia |
| Desulfarculia | 0 | 0 | 0 | 0 | 0 | 1 | 0 | 0 | 0 | 0 | 0 | 0 | 0 | 1 | Bacteria | Bacteria\_none | Thermodesulfobacteriota | Bacteria;Bacteria\_none;Thermodesulfobacteriota;Desulfarculia |
| Desulfobulbia | 0 | 2 | 1 | 0 | 0 | 0 | 0 | 5 | 0 | 0 | 0 | 0 | 1 | 9 | Bacteria | Bacteria\_none | Thermodesulfobacteriota | Bacteria;Bacteria\_none;Thermodesulfobacteriota;Desulfobulbia |
| Desulfovibrionia | 0 | 0 | 0 | 0 | 0 | 0 | 0 | 0 | 0 | 0 | 0 | 1 | 0 | 1 | Bacteria | Bacteria\_none | Thermodesulfobacteriota | Bacteria;Bacteria\_none;Thermodesulfobacteriota;Desulfovibrionia |
| Desulfuromonadia | 0 | 0 | 1 | 0 | 0 | 0 | 0 | 0 | 0 | 0 | 1 | 0 | 0 | 2 | Bacteria | Bacteria\_none | Thermodesulfobacteriota | Bacteria;Bacteria\_none;Thermodesulfobacteriota;Desulfuromonadia |
| Thermomicrobia | 0 | 1 | 0 | 0 | 0 | 0 | 0 | 0 | 0 | 0 | 0 | 0 | 0 | 1 | Bacteria | Bacteria\_none | Thermomicrobiota | Bacteria;Bacteria\_none;Thermomicrobiota;Thermomicrobia |
| Thermotogae | 1 | 0 | 0 | 0 | 0 | 0 | 0 | 0 | 0 | 0 | 0 | 0 | 0 | 1 | Bacteria | Bacteria\_none | Thermotogota | Bacteria;Bacteria\_none;Thermotogota;Thermotogae |
| Opitutia | 0 | 1 | 0 | 0 | 0 | 0 | 0 | 0 | 0 | 0 | 0 | 0 | 0 | 1 | Bacteria | Bacteria\_none | Verrucomicrobiota | Bacteria;Bacteria\_none;Verrucomicrobiota;Opitutia |
| Mammalia | 428 | 938 | 1105 | 97 | 2901 | 211 | 12043 | 17730 | 14114 | 5069 | 5655 | 9919 | 34174 | 104384 | Eukaryota | Metazoa | Chordata | Eukaryota;Metazoa;Chordata;Mammalia |
| Unknown | 1409 | 1058 | 1350 | 2756 | 1867 | 2605 | 1040 | 1418 | 519 | 1446 | 1552 | 1325 | 24901 | 43246 | Unclassified | Unknown | Unknown | Unclassified;Unknown;Unknown;Unknown |
| Megaviricetes | 0 | 0 | 0 | 0 | 0 | 0 | 0 | 1 | 0 | 0 | 0 | 1 | 1 | 3 | Viruses | Bamfordvirae | Nucleocytoviricota | Viruses;Bamfordvirae;Nucleocytoviricota;Megaviricetes |
| Herviviricetes | 8 | 0 | 0 | 0 | 0 | 1 | 0 | 1 | 0 | 0 | 0 | 0 | 2 | 12 | Viruses | Heunggongvirae | Peploviricota | Viruses;Heunggongvirae;Peploviricota;Herviviricetes |
| Caudoviricetes | 7 | 0 | 0 | 1 | 3 | 1 | 5 | 0 | 0 | 0 | 2 | 1 | 2 | 22 | Viruses | Heunggongvirae | Uroviricota | Viruses;Heunggongvirae;Uroviricota;Caudoviricetes |
| Monjiviricetes | 0 | 0 | 0 | 0 | 0 | 0 | 0 | 0 | 0 | 0 | 0 | 0 | 1 | 1 | Viruses | Orthornavirae | Negarnaviricota | Viruses;Orthornavirae;Negarnaviricota;Monjiviricetes |
| Papovaviricetes | 0 | 0 | 0 | 0 | 0 | 0 | 0 | 2 | 0 | 0 | 0 | 0 | 0 | 2 | Viruses | Shotokuvirae | Cossaviricota | Viruses;Shotokuvirae;Cossaviricota;Papovaviricetes |
| Tokiviricetes | 0 | 0 | 0 | 0 | 0 | 0 | 0 | 0 | 0 | 0 | 0 | 0 | 1 | 1 | Viruses | Zilligvirae | Taleaviricota | Viruses;Zilligvirae;Taleaviricota;Tokiviricetes |

Export CSV

Abundance table for the order rank.

Only taxa whose global abundance are above the
`abundance_threshold` parameter will appear in the table.

| order | barcode01 | barcode02 | barcode03 | barcode04 | barcode05 | barcode06 | barcode07 | barcode08 | barcode09 | barcode10 | barcode11 | barcode12 | unclassified | total | superkingdom | kingdom | phylum | class | tax |
| --- | --- | --- | --- | --- | --- | --- | --- | --- | --- | --- | --- | --- | --- | --- | --- | --- | --- | --- | --- |
| Halobacteriales | 1 | 1 | 0 | 0 | 0 | 1 | 1 | 0 | 0 | 1 | 1 | 0 | 1 | 7 | Archaea | Archaea\_none | Euryarchaeota | Halobacteria | Archaea;Archaea\_none;Euryarchaeota;Halobacteria;Halobacteriales |
| Methanobacteriales | 0 | 0 | 2 | 0 | 0 | 1 | 1 | 0 | 0 | 0 | 0 | 0 | 1 | 5 | Archaea | Archaea\_none | Euryarchaeota | Methanobacteria | Archaea;Archaea\_none;Euryarchaeota;Methanobacteria;Methanobacteriales |
| Methanococcales | 0 | 0 | 1 | 0 | 0 | 0 | 0 | 0 | 0 | 0 | 0 | 0 | 1 | 2 | Archaea | Archaea\_none | Euryarchaeota | Methanococci | Archaea;Archaea\_none;Euryarchaeota;Methanococci;Methanococcales |
| Methanomicrobiales | 0 | 1 | 0 | 0 | 0 | 0 | 0 | 0 | 0 | 0 | 0 | 0 | 1 | 2 | Archaea | Archaea\_none | Euryarchaeota | Methanomicrobia | Archaea;Archaea\_none;Euryarchaeota;Methanomicrobia;Methanomicrobiales |
| Methanosarcinales | 0 | 0 | 0 | 1 | 1 | 3 | 0 | 0 | 0 | 0 | 1 | 0 | 1 | 7 | Archaea | Archaea\_none | Euryarchaeota | Methanomicrobia | Archaea;Archaea\_none;Euryarchaeota;Methanomicrobia;Methanosarcinales |
| Thermococcales | 0 | 0 | 0 | 0 | 0 | 0 | 0 | 0 | 0 | 0 | 0 | 1 | 0 | 1 | Archaea | Archaea\_none | Euryarchaeota | Thermococci | Archaea;Archaea\_none;Euryarchaeota;Thermococci;Thermococcales |
| Nitrososphaerota\_Incertae\_sedis | 0 | 0 | 0 | 0 | 0 | 1 | 0 | 0 | 0 | 0 | 0 | 0 | 0 | 1 | Archaea | Archaea\_none | Nitrososphaerota | Nitrososphaerota\_Incertae\_sedis | Archaea;Archaea\_none;Nitrososphaerota;Nitrososphaerota\_Incertae\_sedis;Nitrososphaerota\_Incertae\_sedis |
| Bryobacterales | 0 | 0 | 0 | 0 | 0 | 0 | 0 | 0 | 0 | 0 | 0 | 1 | 0 | 1 | Bacteria | Bacteria\_none | Acidobacteriota | Terriglobia | Bacteria;Bacteria\_none;Acidobacteriota;Terriglobia;Bryobacterales |
| Acidimicrobiales | 0 | 0 | 0 | 0 | 0 | 0 | 0 | 1 | 0 | 0 | 0 | 0 | 0 | 1 | Bacteria | Bacteria\_none | Actinomycetota | Acidimicrobiia | Bacteria;Bacteria\_none;Actinomycetota;Acidimicrobiia;Acidimicrobiales |
| Actinomycetales | 1 | 17 | 1 | 3 | 3 | 40 | 38 | 4 | 6 | 0 | 1 | 12 | 19 | 145 | Bacteria | Bacteria\_none | Actinomycetota | Actinomycetes | Bacteria;Bacteria\_none;Actinomycetota;Actinomycetes;Actinomycetales |
| Bifidobacteriales | 0 | 1 | 2 | 0 | 1 | 1 | 7 | 4 | 0 | 0 | 0 | 0 | 2 | 18 | Bacteria | Bacteria\_none | Actinomycetota | Actinomycetes | Bacteria;Bacteria\_none;Actinomycetota;Actinomycetes;Bifidobacteriales |
| Geodermatophilales | 0 | 0 | 0 | 0 | 1 | 0 | 0 | 0 | 0 | 0 | 0 | 0 | 0 | 1 | Bacteria | Bacteria\_none | Actinomycetota | Actinomycetes | Bacteria;Bacteria\_none;Actinomycetota;Actinomycetes;Geodermatophilales |
| Kitasatosporales | 0 | 2 | 0 | 2 | 2 | 9 | 4 | 1 | 0 | 2 | 1 | 2 | 5 | 30 | Bacteria | Bacteria\_none | Actinomycetota | Actinomycetes | Bacteria;Bacteria\_none;Actinomycetota;Actinomycetes;Kitasatosporales |
| Micrococcales | 28 | 111 | 23 | 19 | 127 | 42 | 38 | 62 | 15 | 80 | 157 | 167 | 217 | 1086 | Bacteria | Bacteria\_none | Actinomycetota | Actinomycetes | Bacteria;Bacteria\_none;Actinomycetota;Actinomycetes;Micrococcales |
| Micromonosporales | 1 | 0 | 0 | 1 | 0 | 1 | 0 | 1 | 0 | 3 | 0 | 0 | 1 | 8 | Bacteria | Bacteria\_none | Actinomycetota | Actinomycetes | Bacteria;Bacteria\_none;Actinomycetota;Actinomycetes;Micromonosporales |
| Mycobacteriales | 0 | 7 | 2 | 1 | 3 | 5 | 2 | 0 | 0 | 15 | 1 | 7 | 11 | 54 | Bacteria | Bacteria\_none | Actinomycetota | Actinomycetes | Bacteria;Bacteria\_none;Actinomycetota;Actinomycetes;Mycobacteriales |
| Propionibacteriales | 0 | 1 | 1 | 3 | 2 | 3 | 0 | 1 | 0 | 1 | 45 | 0 | 10 | 67 | Bacteria | Bacteria\_none | Actinomycetota | Actinomycetes | Bacteria;Bacteria\_none;Actinomycetota;Actinomycetes;Propionibacteriales |
| Pseudonocardiales | 0 | 0 | 0 | 1 | 1 | 1 | 0 | 0 | 0 | 0 | 0 | 0 | 0 | 3 | Bacteria | Bacteria\_none | Actinomycetota | Actinomycetes | Bacteria;Bacteria\_none;Actinomycetota;Actinomycetes;Pseudonocardiales |
| Streptosporangiales | 0 | 0 | 1 | 1 | 1 | 1 | 2 | 0 | 0 | 0 | 0 | 0 | 0 | 6 | Bacteria | Bacteria\_none | Actinomycetota | Actinomycetes | Bacteria;Bacteria\_none;Actinomycetota;Actinomycetes;Streptosporangiales |
| Coriobacteriales | 28 | 5 | 10 | 0 | 2 | 133 | 62 | 7 | 1 | 0 | 7 | 4 | 35 | 294 | Bacteria | Bacteria\_none | Actinomycetota | Coriobacteriia | Bacteria;Bacteria\_none;Actinomycetota;Coriobacteriia;Coriobacteriales |
| Eggerthellales | 0 | 0 | 0 | 0 | 0 | 2 | 1 | 0 | 0 | 0 | 0 | 0 | 0 | 3 | Bacteria | Bacteria\_none | Actinomycetota | Coriobacteriia | Bacteria;Bacteria\_none;Actinomycetota;Coriobacteriia;Eggerthellales |
| Rubrobacterales | 0 | 0 | 0 | 0 | 1 | 0 | 0 | 0 | 0 | 0 | 0 | 0 | 0 | 1 | Bacteria | Bacteria\_none | Actinomycetota | Rubrobacteria | Bacteria;Bacteria\_none;Actinomycetota;Rubrobacteria;Rubrobacterales |
| Miltoncostaeales | 0 | 1 | 0 | 0 | 0 | 0 | 0 | 0 | 0 | 0 | 0 | 0 | 0 | 1 | Bacteria | Bacteria\_none | Actinomycetota | Thermoleophilia | Bacteria;Bacteria\_none;Actinomycetota;Thermoleophilia;Miltoncostaeales |
| Solirubrobacterales | 0 | 1 | 0 | 0 | 0 | 0 | 0 | 0 | 0 | 0 | 0 | 0 | 0 | 1 | Bacteria | Bacteria\_none | Actinomycetota | Thermoleophilia | Bacteria;Bacteria\_none;Actinomycetota;Thermoleophilia;Solirubrobacterales |
| Bacillales | 82 | 132 | 19 | 146 | 574 | 362 | 141 | 118 | 7 | 391 | 1186 | 102 | 1015 | 4275 | Bacteria | Bacteria\_none | Bacillota | Bacilli | Bacteria;Bacteria\_none;Bacillota;Bacilli;Bacillales |
| Lactobacillales | 2292 | 1447 | 1228 | 576 | 2765 | 1933 | 2821 | 451 | 585 | 1277 | 3069 | 1045 | 5306 | 24795 | Bacteria | Bacteria\_none | Bacillota | Bacilli | Bacteria;Bacteria\_none;Bacillota;Bacilli;Lactobacillales |
| Caldicellulosiruptorales | 0 | 0 | 0 | 0 | 1 | 0 | 0 | 0 | 0 | 0 | 0 | 0 | 0 | 1 | Bacteria | Bacteria\_none | Bacillota | Bacillota\_Incertae\_sedis | Bacteria;Bacteria\_none;Bacillota;Bacillota\_Incertae\_sedis;Caldicellulosiruptorales |
| Eubacteriales | 4 | 5 | 6 | 7 | 6 | 22 | 6 | 2 | 5 | 1 | 6 | 2 | 23 | 95 | Bacteria | Bacteria\_none | Bacillota | Clostridia | Bacteria;Bacteria\_none;Bacillota;Clostridia;Eubacteriales |
| Halanaerobiales | 0 | 0 | 0 | 0 | 0 | 0 | 0 | 0 | 0 | 0 | 0 | 0 | 1 | 1 | Bacteria | Bacteria\_none | Bacillota | Clostridia | Bacteria;Bacteria\_none;Bacillota;Clostridia;Halanaerobiales |
| Lachnospirales | 1 | 35 | 28 | 17 | 15 | 66 | 14 | 1 | 1 | 11 | 5 | 8 | 30 | 232 | Bacteria | Bacteria\_none | Bacillota | Clostridia | Bacteria;Bacteria\_none;Bacillota;Clostridia;Lachnospirales |
| Monoglobales | 0 | 0 | 0 | 0 | 0 | 0 | 0 | 2 | 0 | 0 | 0 | 0 | 0 | 2 | Bacteria | Bacteria\_none | Bacillota | Clostridia | Bacteria;Bacteria\_none;Bacillota;Clostridia;Monoglobales |
| Natranaerobiales | 0 | 0 | 0 | 1 | 0 | 1 | 0 | 0 | 0 | 0 | 0 | 0 | 0 | 2 | Bacteria | Bacteria\_none | Bacillota | Clostridia | Bacteria;Bacteria\_none;Bacillota;Clostridia;Natranaerobiales |
| Peptostreptococcales | 5 | 39 | 47 | 7 | 11 | 38 | 7 | 59 | 2 | 0 | 10 | 21 | 73 | 319 | Bacteria | Bacteria\_none | Bacillota | Clostridia | Bacteria;Bacteria\_none;Bacillota;Clostridia;Peptostreptococcales |
| Thermoanaerobacterales | 0 | 1 | 0 | 0 | 0 | 0 | 0 | 0 | 0 | 1 | 0 | 0 | 0 | 2 | Bacteria | Bacteria\_none | Bacillota | Clostridia | Bacteria;Bacteria\_none;Bacillota;Clostridia;Thermoanaerobacterales |
| Erysipelotrichales | 14 | 17 | 92 | 10 | 16 | 523 | 49 | 21 | 1 | 8 | 10 | 2 | 154 | 917 | Bacteria | Bacteria\_none | Bacillota | Erysipelotrichia | Bacteria;Bacteria\_none;Bacillota;Erysipelotrichia;Erysipelotrichales |
| Selenomonadales | 5 | 7 | 16 | 7 | 9 | 14 | 8 | 10 | 1 | 2 | 4 | 10 | 16 | 109 | Bacteria | Bacteria\_none | Bacillota | Negativicutes | Bacteria;Bacteria\_none;Bacillota;Negativicutes;Selenomonadales |
| Veillonellales | 1186 | 78 | 43 | 100 | 69 | 53 | 37 | 73 | 70 | 143 | 14 | 126 | 655 | 2647 | Bacteria | Bacteria\_none | Bacillota | Negativicutes | Bacteria;Bacteria\_none;Bacillota;Negativicutes;Veillonellales |
| Tissierellales | 6 | 34 | 25 | 16 | 50 | 72 | 7 | 65 | 0 | 2 | 73 | 26 | 148 | 524 | Bacteria | Bacteria\_none | Bacillota | Tissierellia | Bacteria;Bacteria\_none;Bacillota;Tissierellia;Tissierellales |
| Tissierellia\_Incertae\_sedis | 0 | 0 | 0 | 1 | 1 | 0 | 0 | 0 | 0 | 0 | 12 | 1 | 5 | 20 | Bacteria | Bacteria\_none | Bacillota | Tissierellia | Bacteria;Bacteria\_none;Bacillota;Tissierellia;Tissierellia\_Incertae\_sedis |
| Bacteroidales | 345 | 1152 | 331 | 967 | 169 | 385 | 222 | 224 | 341 | 240 | 375 | 419 | 1262 | 6432 | Bacteria | Bacteria\_none | Bacteroidota | Bacteroidia | Bacteria;Bacteria\_none;Bacteroidota;Bacteroidia;Bacteroidales |
| Marinilabiliales | 1 | 0 | 2 | 3 | 0 | 1 | 0 | 2 | 0 | 0 | 2 | 0 | 0 | 11 | Bacteria | Bacteria\_none | Bacteroidota | Bacteroidia | Bacteria;Bacteria\_none;Bacteroidota;Bacteroidia;Marinilabiliales |
| Chitinophagales | 0 | 1 | 0 | 2 | 2 | 1 | 0 | 0 | 0 | 1 | 0 | 1 | 0 | 8 | Bacteria | Bacteria\_none | Bacteroidota | Chitinophagia | Bacteria;Bacteria\_none;Bacteroidota;Chitinophagia;Chitinophagales |
| Cytophagales | 1 | 0 | 1 | 3 | 5 | 2 | 1 | 1 | 0 | 1 | 2 | 0 | 8 | 25 | Bacteria | Bacteria\_none | Bacteroidota | Cytophagia | Bacteria;Bacteria\_none;Bacteroidota;Cytophagia;Cytophagales |
| Flavobacteriales | 317 | 152 | 135 | 220 | 186 | 41 | 84 | 382 | 0 | 363 | 222 | 56 | 582 | 2740 | Bacteria | Bacteria\_none | Bacteroidota | Flavobacteriia | Bacteria;Bacteria\_none;Bacteroidota;Flavobacteriia;Flavobacteriales |
| Saprospirales | 0 | 0 | 0 | 0 | 0 | 0 | 0 | 0 | 0 | 0 | 0 | 0 | 1 | 1 | Bacteria | Bacteria\_none | Bacteroidota | Saprospiria | Bacteria;Bacteria\_none;Bacteroidota;Saprospiria;Saprospirales |
| Sphingobacteriales | 0 | 2 | 0 | 4 | 1 | 1 | 0 | 1 | 0 | 1 | 3 | 1 | 2 | 16 | Bacteria | Bacteria\_none | Bacteroidota | Sphingobacteriia | Bacteria;Bacteria\_none;Bacteroidota;Sphingobacteriia;Sphingobacteriales |
| Balneolales | 0 | 0 | 0 | 1 | 0 | 0 | 0 | 0 | 0 | 0 | 0 | 0 | 0 | 1 | Bacteria | Bacteria\_none | Balneolota | Balneolia | Bacteria;Bacteria\_none;Balneolota;Balneolia;Balneolales |
| Bdellovibrionales | 0 | 0 | 0 | 0 | 0 | 0 | 0 | 0 | 0 | 1 | 0 | 0 | 0 | 1 | Bacteria | Bacteria\_none | Bdellovibrionota | Bdellovibrionia | Bacteria;Bacteria\_none;Bdellovibrionota;Bdellovibrionia;Bdellovibrionales |
| Calditrichales | 0 | 0 | 0 | 1 | 0 | 0 | 0 | 0 | 0 | 0 | 0 | 0 | 0 | 1 | Bacteria | Bacteria\_none | Calditrichota | Calditrichia | Bacteria;Bacteria\_none;Calditrichota;Calditrichia;Calditrichales |
| Campylobacterales | 1 | 47 | 16 | 11 | 11 | 50 | 2 | 11 | 9 | 12 | 3 | 14 | 61 | 248 | Bacteria | Bacteria\_none | Campylobacterota | Epsilonproteobacteria | Bacteria;Bacteria\_none;Campylobacterota;Epsilonproteobacteria;Campylobacterales |
| Candidatus Saccharibacteria\_Incertae\_sedis | 0 | 0 | 0 | 0 | 2 | 20 | 0 | 0 | 0 | 0 | 1 | 1 | 5 | 29 | Bacteria | Bacteria\_none | Candidatus Saccharibacteria | Candidatus Saccharibacteria\_Incertae\_sedis | Bacteria;Bacteria\_none;Candidatus Saccharibacteria;Candidatus Saccharibacteria\_Incertae\_sedis;Candidatus Saccharibacteria\_Incertae\_sedis |
| Candidatus Nanosynbacterales | 0 | 8 | 5 | 0 | 10 | 165 | 1 | 1 | 3 | 2 | 92 | 6 | 48 | 341 | Bacteria | Bacteria\_none | Candidatus Saccharibacteria | Candidatus Saccharimonadia | Bacteria;Bacteria\_none;Candidatus Saccharibacteria;Candidatus Saccharimonadia;Candidatus Nanosynbacterales |
| Candidatus Saccharimonadales | 0 | 0 | 0 | 0 | 1 | 0 | 0 | 1 | 0 | 0 | 1 | 0 | 0 | 3 | Bacteria | Bacteria\_none | Candidatus Saccharibacteria | Candidatus Saccharimonadia | Bacteria;Bacteria\_none;Candidatus Saccharibacteria;Candidatus Saccharimonadia;Candidatus Saccharimonadales |
| Parachlamydiales | 0 | 0 | 0 | 0 | 0 | 0 | 0 | 0 | 0 | 0 | 0 | 0 | 1 | 1 | Bacteria | Bacteria\_none | Chlamydiota | Chlamydiia | Bacteria;Bacteria\_none;Chlamydiota;Chlamydiia;Parachlamydiales |
| Chlorobiales | 0 | 0 | 0 | 0 | 0 | 1 | 0 | 0 | 0 | 0 | 0 | 0 | 0 | 1 | Bacteria | Bacteria\_none | Chlorobiota | Chlorobiia | Bacteria;Bacteria\_none;Chlorobiota;Chlorobiia;Chlorobiales |
| Candidatus Chloroheliales | 0 | 0 | 0 | 0 | 0 | 0 | 0 | 0 | 0 | 0 | 1 | 0 | 0 | 1 | Bacteria | Bacteria\_none | Chloroflexota | Chloroflexia | Bacteria;Bacteria\_none;Chloroflexota;Chloroflexia;Candidatus Chloroheliales |
| Candidatus Lucifugimonadales | 0 | 0 | 0 | 0 | 0 | 0 | 0 | 0 | 0 | 0 | 0 | 0 | 1 | 1 | Bacteria | Bacteria\_none | Chloroflexota | Dehalococcoidia | Bacteria;Bacteria\_none;Chloroflexota;Dehalococcoidia;Candidatus Lucifugimonadales |
| Cyanobacteriota\_Incertae\_sedis | 1 | 0 | 0 | 0 | 0 | 0 | 0 | 0 | 0 | 0 | 0 | 0 | 0 | 1 | Bacteria | Bacteria\_none | Cyanobacteriota | Cyanobacteriota\_Incertae\_sedis | Bacteria;Bacteria\_none;Cyanobacteriota;Cyanobacteriota\_Incertae\_sedis;Cyanobacteriota\_Incertae\_sedis |
| Chroococcales | 0 | 0 | 1 | 0 | 0 | 0 | 0 | 0 | 0 | 0 | 1 | 0 | 0 | 2 | Bacteria | Bacteria\_none | Cyanobacteriota | Cyanophyceae | Bacteria;Bacteria\_none;Cyanobacteriota;Cyanophyceae;Chroococcales |
| Gloeobacterales | 0 | 0 | 0 | 0 | 0 | 0 | 1 | 0 | 0 | 0 | 0 | 0 | 0 | 1 | Bacteria | Bacteria\_none | Cyanobacteriota | Cyanophyceae | Bacteria;Bacteria\_none;Cyanobacteriota;Cyanophyceae;Gloeobacterales |
| Nostocales | 0 | 1 | 0 | 1 | 0 | 0 | 0 | 0 | 0 | 2 | 1 | 0 | 2 | 7 | Bacteria | Bacteria\_none | Cyanobacteriota | Cyanophyceae | Bacteria;Bacteria\_none;Cyanobacteriota;Cyanophyceae;Nostocales |
| Synechococcales | 0 | 0 | 0 | 0 | 1 | 1 | 0 | 0 | 0 | 0 | 0 | 0 | 1 | 3 | Bacteria | Bacteria\_none | Cyanobacteriota | Cyanophyceae | Bacteria;Bacteria\_none;Cyanobacteriota;Cyanophyceae;Synechococcales |
| Deferribacterales | 0 | 1 | 0 | 0 | 0 | 0 | 0 | 0 | 0 | 0 | 0 | 0 | 1 | 2 | Bacteria | Bacteria\_none | Deferribacterota | Deferribacteres | Bacteria;Bacteria\_none;Deferribacterota;Deferribacteres;Deferribacterales |
| Fusobacteriales | 17 | 266 | 237 | 845 | 187 | 258 | 449 | 562 | 24 | 132 | 360 | 130 | 1567 | 5034 | Bacteria | Bacteria\_none | Fusobacteriota | Fusobacteriia | Bacteria;Bacteria\_none;Fusobacteriota;Fusobacteriia;Fusobacteriales |
| Ignavibacteriales | 0 | 0 | 0 | 0 | 0 | 0 | 0 | 0 | 0 | 0 | 0 | 0 | 1 | 1 | Bacteria | Bacteria\_none | Ignavibacteriota | Ignavibacteria | Bacteria;Bacteria\_none;Ignavibacteriota;Ignavibacteria;Ignavibacteriales |
| Acholeplasmatales | 0 | 0 | 0 | 0 | 1 | 1 | 0 | 0 | 0 | 0 | 0 | 0 | 0 | 2 | Bacteria | Bacteria\_none | Mycoplasmatota | Mollicutes | Bacteria;Bacteria\_none;Mycoplasmatota;Mollicutes;Acholeplasmatales |
| Entomoplasmatales | 0 | 0 | 0 | 1 | 1 | 3 | 0 | 0 | 0 | 1 | 0 | 0 | 3 | 9 | Bacteria | Bacteria\_none | Mycoplasmatota | Mollicutes | Bacteria;Bacteria\_none;Mycoplasmatota;Mollicutes;Entomoplasmatales |
| Mycoplasmatales | 0 | 1 | 3 | 0 | 0 | 1 | 0 | 0 | 0 | 0 | 1 | 0 | 1 | 7 | Bacteria | Bacteria\_none | Mycoplasmatota | Mollicutes | Bacteria;Bacteria\_none;Mycoplasmatota;Mollicutes;Mycoplasmatales |
| Mycoplasmoidales | 0 | 10 | 37 | 2 | 3 | 6 | 1 | 3 | 0 | 9 | 3 | 7 | 27 | 108 | Bacteria | Bacteria\_none | Mycoplasmatota | Mycoplasmatota\_Incertae\_sedis | Bacteria;Bacteria\_none;Mycoplasmatota;Mycoplasmatota\_Incertae\_sedis;Mycoplasmoidales |
| Myxococcales | 0 | 0 | 0 | 0 | 0 | 0 | 0 | 0 | 0 | 0 | 0 | 0 | 1 | 1 | Bacteria | Bacteria\_none | Myxococcota | Myxococcia | Bacteria;Bacteria\_none;Myxococcota;Myxococcia;Myxococcales |
| Polyangiales | 0 | 0 | 0 | 0 | 2 | 0 | 0 | 0 | 0 | 0 | 1 | 0 | 0 | 3 | Bacteria | Bacteria\_none | Myxococcota | Myxococcota\_Incertae\_sedis | Bacteria;Bacteria\_none;Myxococcota;Myxococcota\_Incertae\_sedis;Polyangiales |
| Nitrospirales | 0 | 0 | 0 | 0 | 0 | 1 | 0 | 1 | 0 | 0 | 0 | 0 | 0 | 2 | Bacteria | Bacteria\_none | Nitrospirota | Nitrospiria | Bacteria;Bacteria\_none;Nitrospirota;Nitrospiria;Nitrospirales |
| Thermodesulfovibrionales | 0 | 0 | 0 | 0 | 0 | 0 | 0 | 0 | 0 | 0 | 1 | 0 | 0 | 1 | Bacteria | Bacteria\_none | Nitrospirota | Thermodesulfovibrionia | Bacteria;Bacteria\_none;Nitrospirota;Thermodesulfovibrionia;Thermodesulfovibrionales |
| Gemmatales | 0 | 0 | 0 | 0 | 0 | 1 | 0 | 0 | 0 | 0 | 0 | 0 | 0 | 1 | Bacteria | Bacteria\_none | Planctomycetota | Planctomycetia | Bacteria;Bacteria\_none;Planctomycetota;Planctomycetia;Gemmatales |
| Pirellulales | 1 | 0 | 0 | 0 | 0 | 0 | 0 | 0 | 0 | 0 | 0 | 0 | 0 | 1 | Bacteria | Bacteria\_none | Planctomycetota | Planctomycetia | Bacteria;Bacteria\_none;Planctomycetota;Planctomycetia;Pirellulales |
| Planctomycetales | 0 | 0 | 0 | 0 | 0 | 0 | 0 | 0 | 0 | 0 | 2 | 0 | 0 | 2 | Bacteria | Bacteria\_none | Planctomycetota | Planctomycetia | Bacteria;Bacteria\_none;Planctomycetota;Planctomycetia;Planctomycetales |
| Candidatus Pelagibacterales | 0 | 0 | 0 | 0 | 0 | 0 | 0 | 0 | 0 | 0 | 0 | 0 | 1 | 1 | Bacteria | Bacteria\_none | Pseudomonadota | Alphaproteobacteria | Bacteria;Bacteria\_none;Pseudomonadota;Alphaproteobacteria;Candidatus Pelagibacterales |
| Caulobacterales | 0 | 0 | 0 | 0 | 0 | 0 | 0 | 0 | 0 | 0 | 0 | 0 | 1 | 1 | Bacteria | Bacteria\_none | Pseudomonadota | Alphaproteobacteria | Bacteria;Bacteria\_none;Pseudomonadota;Alphaproteobacteria;Caulobacterales |
| Hyphomicrobiales | 1 | 1 | 5 | 0 | 1 | 5 | 2 | 4 | 1 | 1 | 5 | 0 | 3 | 29 | Bacteria | Bacteria\_none | Pseudomonadota | Alphaproteobacteria | Bacteria;Bacteria\_none;Pseudomonadota;Alphaproteobacteria;Hyphomicrobiales |
| Kordiimonadales | 0 | 0 | 0 | 0 | 0 | 0 | 0 | 1 | 0 | 0 | 0 | 0 | 0 | 1 | Bacteria | Bacteria\_none | Pseudomonadota | Alphaproteobacteria | Bacteria;Bacteria\_none;Pseudomonadota;Alphaproteobacteria;Kordiimonadales |
| Rhodobacterales | 2 | 1 | 0 | 1 | 2 | 1 | 0 | 0 | 1 | 0 | 3 | 0 | 2 | 13 | Bacteria | Bacteria\_none | Pseudomonadota | Alphaproteobacteria | Bacteria;Bacteria\_none;Pseudomonadota;Alphaproteobacteria;Rhodobacterales |
| Rhodospirillales | 0 | 0 | 0 | 1 | 1 | 1 | 1 | 0 | 0 | 0 | 1 | 0 | 1 | 6 | Bacteria | Bacteria\_none | Pseudomonadota | Alphaproteobacteria | Bacteria;Bacteria\_none;Pseudomonadota;Alphaproteobacteria;Rhodospirillales |
| Rickettsiales | 0 | 1 | 0 | 0 | 0 | 0 | 1 | 0 | 0 | 0 | 0 | 0 | 3 | 5 | Bacteria | Bacteria\_none | Pseudomonadota | Alphaproteobacteria | Bacteria;Bacteria\_none;Pseudomonadota;Alphaproteobacteria;Rickettsiales |
| Sphingomonadales | 0 | 0 | 0 | 1 | 0 | 1 | 0 | 0 | 0 | 0 | 2 | 1 | 2 | 7 | Bacteria | Bacteria\_none | Pseudomonadota | Alphaproteobacteria | Bacteria;Bacteria\_none;Pseudomonadota;Alphaproteobacteria;Sphingomonadales |
| Betaproteobacteria\_Incertae\_sedis | 0 | 0 | 0 | 0 | 1 | 0 | 0 | 1 | 0 | 0 | 0 | 0 | 0 | 2 | Bacteria | Bacteria\_none | Pseudomonadota | Betaproteobacteria | Bacteria;Bacteria\_none;Pseudomonadota;Betaproteobacteria;Betaproteobacteria\_Incertae\_sedis |
| Burkholderiales | 1 | 1 | 3 | 0 | 8 | 4 | 4 | 3 | 1 | 5 | 15 | 1 | 11 | 57 | Bacteria | Bacteria\_none | Pseudomonadota | Betaproteobacteria | Bacteria;Bacteria\_none;Pseudomonadota;Betaproteobacteria;Burkholderiales |
| Neisseriales | 205 | 202 | 1085 | 4097 | 357 | 328 | 61 | 194 | 1 | 1681 | 209 | 123 | 2195 | 10738 | Bacteria | Bacteria\_none | Pseudomonadota | Betaproteobacteria | Bacteria;Bacteria\_none;Pseudomonadota;Betaproteobacteria;Neisseriales |
| Nitrosomonadales | 0 | 0 | 0 | 0 | 0 | 0 | 0 | 0 | 0 | 0 | 0 | 0 | 2 | 2 | Bacteria | Bacteria\_none | Pseudomonadota | Betaproteobacteria | Bacteria;Bacteria\_none;Pseudomonadota;Betaproteobacteria;Nitrosomonadales |
| Rhodocyclales | 0 | 0 | 0 | 0 | 0 | 0 | 1 | 0 | 0 | 0 | 0 | 0 | 0 | 1 | Bacteria | Bacteria\_none | Pseudomonadota | Betaproteobacteria | Bacteria;Bacteria\_none;Pseudomonadota;Betaproteobacteria;Rhodocyclales |
| Aeromonadales | 0 | 0 | 0 | 0 | 0 | 2 | 0 | 0 | 0 | 0 | 0 | 0 | 1 | 3 | Bacteria | Bacteria\_none | Pseudomonadota | Gammaproteobacteria | Bacteria;Bacteria\_none;Pseudomonadota;Gammaproteobacteria;Aeromonadales |
| Alteromonadales | 3 | 1 | 1 | 0 | 3 | 2 | 0 | 3 | 0 | 0 | 1 | 1 | 2 | 17 | Bacteria | Bacteria\_none | Pseudomonadota | Gammaproteobacteria | Bacteria;Bacteria\_none;Pseudomonadota;Gammaproteobacteria;Alteromonadales |
| Cardiobacteriales | 0 | 3 | 14 | 3 | 2 | 6 | 0 | 2 | 0 | 1 | 14 | 2 | 7 | 54 | Bacteria | Bacteria\_none | Pseudomonadota | Gammaproteobacteria | Bacteria;Bacteria\_none;Pseudomonadota;Gammaproteobacteria;Cardiobacteriales |
| Cellvibrionales | 0 | 0 | 0 | 0 | 0 | 0 | 0 | 1 | 0 | 0 | 0 | 0 | 0 | 1 | Bacteria | Bacteria\_none | Pseudomonadota | Gammaproteobacteria | Bacteria;Bacteria\_none;Pseudomonadota;Gammaproteobacteria;Cellvibrionales |
| Chromatiales | 0 | 0 | 0 | 1 | 0 | 0 | 0 | 0 | 0 | 0 | 0 | 0 | 2 | 3 | Bacteria | Bacteria\_none | Pseudomonadota | Gammaproteobacteria | Bacteria;Bacteria\_none;Pseudomonadota;Gammaproteobacteria;Chromatiales |
| Enterobacterales | 5 | 0 | 1 | 5 | 8 | 3 | 1 | 13 | 1 | 4 | 2 | 3 | 16 | 62 | Bacteria | Bacteria\_none | Pseudomonadota | Gammaproteobacteria | Bacteria;Bacteria\_none;Pseudomonadota;Gammaproteobacteria;Enterobacterales |
| Legionellales | 0 | 0 | 0 | 1 | 0 | 0 | 0 | 0 | 0 | 0 | 0 | 0 | 1 | 2 | Bacteria | Bacteria\_none | Pseudomonadota | Gammaproteobacteria | Bacteria;Bacteria\_none;Pseudomonadota;Gammaproteobacteria;Legionellales |
| Lysobacterales | 0 | 0 | 0 | 1 | 2 | 0 | 0 | 0 | 0 | 1 | 1 | 1 | 2 | 8 | Bacteria | Bacteria\_none | Pseudomonadota | Gammaproteobacteria | Bacteria;Bacteria\_none;Pseudomonadota;Gammaproteobacteria;Lysobacterales |
| Methylococcales | 0 | 0 | 0 | 0 | 0 | 1 | 0 | 0 | 0 | 0 | 0 | 0 | 4 | 5 | Bacteria | Bacteria\_none | Pseudomonadota | Gammaproteobacteria | Bacteria;Bacteria\_none;Pseudomonadota;Gammaproteobacteria;Methylococcales |
| Moraxellales | 0 | 0 | 1 | 2 | 0 | 2 | 0 | 4 | 0 | 2 | 0 | 2 | 5 | 18 | Bacteria | Bacteria\_none | Pseudomonadota | Gammaproteobacteria | Bacteria;Bacteria\_none;Pseudomonadota;Gammaproteobacteria;Moraxellales |
| Oceanospirillales | 0 | 1 | 0 | 0 | 1 | 2 | 0 | 4 | 0 | 0 | 0 | 0 | 1 | 9 | Bacteria | Bacteria\_none | Pseudomonadota | Gammaproteobacteria | Bacteria;Bacteria\_none;Pseudomonadota;Gammaproteobacteria;Oceanospirillales |
| Orbales | 0 | 0 | 0 | 0 | 0 | 0 | 0 | 1 | 0 | 0 | 0 | 0 | 1 | 2 | Bacteria | Bacteria\_none | Pseudomonadota | Gammaproteobacteria | Bacteria;Bacteria\_none;Pseudomonadota;Gammaproteobacteria;Orbales |
| Pasteurellales | 71 | 142 | 466 | 773 | 285 | 244 | 8 | 1922 | 0 | 92 | 49 | 244 | 1237 | 5533 | Bacteria | Bacteria\_none | Pseudomonadota | Gammaproteobacteria | Bacteria;Bacteria\_none;Pseudomonadota;Gammaproteobacteria;Pasteurellales |
| Pseudomonadales | 0 | 3 | 0 | 0 | 2 | 3 | 1 | 1 | 0 | 0 | 2 | 0 | 4 | 16 | Bacteria | Bacteria\_none | Pseudomonadota | Gammaproteobacteria | Bacteria;Bacteria\_none;Pseudomonadota;Gammaproteobacteria;Pseudomonadales |
| Thiotrichales | 0 | 0 | 0 | 0 | 1 | 0 | 0 | 0 | 0 | 0 | 0 | 0 | 0 | 1 | Bacteria | Bacteria\_none | Pseudomonadota | Gammaproteobacteria | Bacteria;Bacteria\_none;Pseudomonadota;Gammaproteobacteria;Thiotrichales |
| Vibrionales | 0 | 0 | 3 | 0 | 2 | 1 | 0 | 5 | 0 | 0 | 0 | 0 | 4 | 15 | Bacteria | Bacteria\_none | Pseudomonadota | Gammaproteobacteria | Bacteria;Bacteria\_none;Pseudomonadota;Gammaproteobacteria;Vibrionales |
| Leptospirales | 1 | 0 | 0 | 0 | 0 | 0 | 0 | 0 | 0 | 0 | 0 | 1 | 0 | 2 | Bacteria | Bacteria\_none | Spirochaetota | Spirochaetia | Bacteria;Bacteria\_none;Spirochaetota;Spirochaetia;Leptospirales |
| Spirochaetales | 3 | 74 | 54 | 9 | 40 | 74 | 9 | 112 | 0 | 14 | 56 | 119 | 143 | 707 | Bacteria | Bacteria\_none | Spirochaetota | Spirochaetia | Bacteria;Bacteria\_none;Spirochaetota;Spirochaetia;Spirochaetales |
| Synergistales | 0 | 0 | 1 | 0 | 0 | 0 | 0 | 0 | 0 | 0 | 1 | 1 | 0 | 3 | Bacteria | Bacteria\_none | Synergistota | Synergistia | Bacteria;Bacteria\_none;Synergistota;Synergistia;Synergistales |
| Candidatus Desulfofervidales | 0 | 0 | 0 | 0 | 0 | 1 | 0 | 0 | 0 | 0 | 0 | 0 | 0 | 1 | Bacteria | Bacteria\_none | Thermodesulfobacteriota | Candidatus Desulfofervidia | Bacteria;Bacteria\_none;Thermodesulfobacteriota;Candidatus Desulfofervidia;Candidatus Desulfofervidales |
| Desulfarculales | 0 | 0 | 0 | 0 | 0 | 1 | 0 | 0 | 0 | 0 | 0 | 0 | 0 | 1 | Bacteria | Bacteria\_none | Thermodesulfobacteriota | Desulfarculia | Bacteria;Bacteria\_none;Thermodesulfobacteriota;Desulfarculia;Desulfarculales |
| Desulfobulbales | 0 | 2 | 1 | 0 | 0 | 0 | 0 | 5 | 0 | 0 | 0 | 0 | 1 | 9 | Bacteria | Bacteria\_none | Thermodesulfobacteriota | Desulfobulbia | Bacteria;Bacteria\_none;Thermodesulfobacteriota;Desulfobulbia;Desulfobulbales |
| Desulfovibrionales | 0 | 0 | 0 | 0 | 0 | 0 | 0 | 0 | 0 | 0 | 0 | 1 | 0 | 1 | Bacteria | Bacteria\_none | Thermodesulfobacteriota | Desulfovibrionia | Bacteria;Bacteria\_none;Thermodesulfobacteriota;Desulfovibrionia;Desulfovibrionales |
| Desulfuromonadales | 0 | 0 | 0 | 0 | 0 | 0 | 0 | 0 | 0 | 0 | 1 | 0 | 0 | 1 | Bacteria | Bacteria\_none | Thermodesulfobacteriota | Desulfuromonadia | Bacteria;Bacteria\_none;Thermodesulfobacteriota;Desulfuromonadia;Desulfuromonadales |
| Geobacterales | 0 | 0 | 1 | 0 | 0 | 0 | 0 | 0 | 0 | 0 | 0 | 0 | 0 | 1 | Bacteria | Bacteria\_none | Thermodesulfobacteriota | Desulfuromonadia | Bacteria;Bacteria\_none;Thermodesulfobacteriota;Desulfuromonadia;Geobacterales |
| Thermomicrobiales | 0 | 1 | 0 | 0 | 0 | 0 | 0 | 0 | 0 | 0 | 0 | 0 | 0 | 1 | Bacteria | Bacteria\_none | Thermomicrobiota | Thermomicrobia | Bacteria;Bacteria\_none;Thermomicrobiota;Thermomicrobia;Thermomicrobiales |
| Thermotogales | 1 | 0 | 0 | 0 | 0 | 0 | 0 | 0 | 0 | 0 | 0 | 0 | 0 | 1 | Bacteria | Bacteria\_none | Thermotogota | Thermotogae | Bacteria;Bacteria\_none;Thermotogota;Thermotogae;Thermotogales |
| Opitutales | 0 | 1 | 0 | 0 | 0 | 0 | 0 | 0 | 0 | 0 | 0 | 0 | 0 | 1 | Bacteria | Bacteria\_none | Verrucomicrobiota | Opitutia | Bacteria;Bacteria\_none;Verrucomicrobiota;Opitutia;Opitutales |
| Primates | 428 | 938 | 1105 | 97 | 2901 | 211 | 12043 | 17730 | 14114 | 5069 | 5655 | 9919 | 34174 | 104384 | Eukaryota | Metazoa | Chordata | Mammalia | Eukaryota;Metazoa;Chordata;Mammalia;Primates |
| Unknown | 1409 | 1058 | 1350 | 2756 | 1867 | 2605 | 1040 | 1418 | 519 | 1446 | 1552 | 1325 | 24901 | 43246 | Unclassified | Unknown | Unknown | Unknown | Unclassified;Unknown;Unknown;Unknown;Unknown |
| Imitervirales | 0 | 0 | 0 | 0 | 0 | 0 | 0 | 1 | 0 | 0 | 0 | 1 | 1 | 3 | Viruses | Bamfordvirae | Nucleocytoviricota | Megaviricetes | Viruses;Bamfordvirae;Nucleocytoviricota;Megaviricetes;Imitervirales |
| Herpesvirales | 8 | 0 | 0 | 0 | 0 | 1 | 0 | 1 | 0 | 0 | 0 | 0 | 2 | 12 | Viruses | Heunggongvirae | Peploviricota | Herviviricetes | Viruses;Heunggongvirae;Peploviricota;Herviviricetes;Herpesvirales |
| Caudoviricetes\_Incertae\_sedis | 5 | 0 | 0 | 1 | 3 | 1 | 5 | 0 | 0 | 0 | 2 | 1 | 2 | 20 | Viruses | Heunggongvirae | Uroviricota | Caudoviricetes | Viruses;Heunggongvirae;Uroviricota;Caudoviricetes;Caudoviricetes\_Incertae\_sedis |
| Crassvirales | 2 | 0 | 0 | 0 | 0 | 0 | 0 | 0 | 0 | 0 | 0 | 0 | 0 | 2 | Viruses | Heunggongvirae | Uroviricota | Caudoviricetes | Viruses;Heunggongvirae;Uroviricota;Caudoviricetes;Crassvirales |
| Mononegavirales | 0 | 0 | 0 | 0 | 0 | 0 | 0 | 0 | 0 | 0 | 0 | 0 | 1 | 1 | Viruses | Orthornavirae | Negarnaviricota | Monjiviricetes | Viruses;Orthornavirae;Negarnaviricota;Monjiviricetes;Mononegavirales |
| Zurhausenvirales | 0 | 0 | 0 | 0 | 0 | 0 | 0 | 2 | 0 | 0 | 0 | 0 | 0 | 2 | Viruses | Shotokuvirae | Cossaviricota | Papovaviricetes | Viruses;Shotokuvirae;Cossaviricota;Papovaviricetes;Zurhausenvirales |
| Ligamenvirales | 0 | 0 | 0 | 0 | 0 | 0 | 0 | 0 | 0 | 0 | 0 | 0 | 1 | 1 | Viruses | Zilligvirae | Taleaviricota | Tokiviricetes | Viruses;Zilligvirae;Taleaviricota;Tokiviricetes;Ligamenvirales |

Export CSV

Abundance table for the family rank.

Only taxa whose global abundance are above the
`abundance_threshold` parameter will appear in the table.

| family | barcode01 | barcode02 | barcode03 | barcode04 | barcode05 | barcode06 | barcode07 | barcode08 | barcode09 | barcode10 | barcode11 | barcode12 | unclassified | total | superkingdom | kingdom | phylum | class | order | tax |
| --- | --- | --- | --- | --- | --- | --- | --- | --- | --- | --- | --- | --- | --- | --- | --- | --- | --- | --- | --- | --- |
| Haladaptataceae | 0 | 1 | 0 | 0 | 0 | 0 | 0 | 0 | 0 | 0 | 0 | 0 | 0 | 1 | Archaea | Archaea\_none | Euryarchaeota | Halobacteria | Halobacteriales | Archaea;Archaea\_none;Euryarchaeota;Halobacteria;Halobacteriales;Haladaptataceae |
| Haloarculaceae | 0 | 0 | 0 | 0 | 0 | 0 | 0 | 0 | 0 | 0 | 1 | 0 | 0 | 1 | Archaea | Archaea\_none | Euryarchaeota | Halobacteria | Halobacteriales | Archaea;Archaea\_none;Euryarchaeota;Halobacteria;Halobacteriales;Haloarculaceae |
| Halobacteriaceae | 1 | 0 | 0 | 0 | 0 | 0 | 0 | 0 | 0 | 0 | 0 | 0 | 0 | 1 | Archaea | Archaea\_none | Euryarchaeota | Halobacteria | Halobacteriales | Archaea;Archaea\_none;Euryarchaeota;Halobacteria;Halobacteriales;Halobacteriaceae |
| Haloferacaceae | 0 | 0 | 0 | 0 | 0 | 1 | 1 | 0 | 0 | 1 | 0 | 0 | 1 | 4 | Archaea | Archaea\_none | Euryarchaeota | Halobacteria | Halobacteriales | Archaea;Archaea\_none;Euryarchaeota;Halobacteria;Halobacteriales;Haloferacaceae |
| Methanobacteriaceae | 0 | 0 | 2 | 0 | 0 | 1 | 1 | 0 | 0 | 0 | 0 | 0 | 1 | 5 | Archaea | Archaea\_none | Euryarchaeota | Methanobacteria | Methanobacteriales | Archaea;Archaea\_none;Euryarchaeota;Methanobacteria;Methanobacteriales;Methanobacteriaceae |
| Methanocaldococcaceae | 0 | 0 | 1 | 0 | 0 | 0 | 0 | 0 | 0 | 0 | 0 | 0 | 1 | 2 | Archaea | Archaea\_none | Euryarchaeota | Methanococci | Methanococcales | Archaea;Archaea\_none;Euryarchaeota;Methanococci;Methanococcales;Methanocaldococcaceae |
| Methanoregulaceae | 0 | 1 | 0 | 0 | 0 | 0 | 0 | 0 | 0 | 0 | 0 | 0 | 1 | 2 | Archaea | Archaea\_none | Euryarchaeota | Methanomicrobia | Methanomicrobiales | Archaea;Archaea\_none;Euryarchaeota;Methanomicrobia;Methanomicrobiales;Methanoregulaceae |
| Methanosarcinaceae | 0 | 0 | 0 | 1 | 1 | 3 | 0 | 0 | 0 | 0 | 1 | 0 | 1 | 7 | Archaea | Archaea\_none | Euryarchaeota | Methanomicrobia | Methanosarcinales | Archaea;Archaea\_none;Euryarchaeota;Methanomicrobia;Methanosarcinales;Methanosarcinaceae |
| Thermococcaceae | 0 | 0 | 0 | 0 | 0 | 0 | 0 | 0 | 0 | 0 | 0 | 1 | 0 | 1 | Archaea | Archaea\_none | Euryarchaeota | Thermococci | Thermococcales | Archaea;Archaea\_none;Euryarchaeota;Thermococci;Thermococcales;Thermococcaceae |
| Nitrososphaerota\_Incertae\_sedis | 0 | 0 | 0 | 0 | 0 | 1 | 0 | 0 | 0 | 0 | 0 | 0 | 0 | 1 | Archaea | Archaea\_none | Nitrososphaerota | Nitrososphaerota\_Incertae\_sedis | Nitrososphaerota\_Incertae\_sedis | Archaea;Archaea\_none;Nitrososphaerota;Nitrososphaerota\_Incertae\_sedis;Nitrososphaerota\_Incertae\_sedis;Nitrososphaerota\_Incertae\_sedis |
| Bryobacteraceae | 0 | 0 | 0 | 0 | 0 | 0 | 0 | 0 | 0 | 0 | 0 | 1 | 0 | 1 | Bacteria | Bacteria\_none | Acidobacteriota | Terriglobia | Bryobacterales | Bacteria;Bacteria\_none;Acidobacteriota;Terriglobia;Bryobacterales;Bryobacteraceae |
| Iamiaceae | 0 | 0 | 0 | 0 | 0 | 0 | 0 | 1 | 0 | 0 | 0 | 0 | 0 | 1 | Bacteria | Bacteria\_none | Actinomycetota | Acidimicrobiia | Acidimicrobiales | Bacteria;Bacteria\_none;Actinomycetota;Acidimicrobiia;Acidimicrobiales;Iamiaceae |
| Actinomycetaceae | 1 | 17 | 1 | 3 | 3 | 40 | 38 | 4 | 6 | 0 | 1 | 12 | 19 | 145 | Bacteria | Bacteria\_none | Actinomycetota | Actinomycetes | Actinomycetales | Bacteria;Bacteria\_none;Actinomycetota;Actinomycetes;Actinomycetales;Actinomycetaceae |
| Bifidobacteriaceae | 0 | 1 | 2 | 0 | 1 | 1 | 7 | 4 | 0 | 0 | 0 | 0 | 2 | 18 | Bacteria | Bacteria\_none | Actinomycetota | Actinomycetes | Bifidobacteriales | Bacteria;Bacteria\_none;Actinomycetota;Actinomycetes;Bifidobacteriales;Bifidobacteriaceae |
| Geodermatophilaceae | 0 | 0 | 0 | 0 | 1 | 0 | 0 | 0 | 0 | 0 | 0 | 0 | 0 | 1 | Bacteria | Bacteria\_none | Actinomycetota | Actinomycetes | Geodermatophilales | Bacteria;Bacteria\_none;Actinomycetota;Actinomycetes;Geodermatophilales;Geodermatophilaceae |
| Streptomycetaceae | 0 | 2 | 0 | 2 | 2 | 9 | 4 | 1 | 0 | 2 | 1 | 2 | 5 | 30 | Bacteria | Bacteria\_none | Actinomycetota | Actinomycetes | Kitasatosporales | Bacteria;Bacteria\_none;Actinomycetota;Actinomycetes;Kitasatosporales;Streptomycetaceae |
| Cellulomonadaceae | 0 | 0 | 0 | 0 | 0 | 0 | 0 | 0 | 0 | 0 | 0 | 0 | 1 | 1 | Bacteria | Bacteria\_none | Actinomycetota | Actinomycetes | Micrococcales | Bacteria;Bacteria\_none;Actinomycetota;Actinomycetes;Micrococcales;Cellulomonadaceae |
| Dermatophilaceae | 0 | 0 | 0 | 0 | 0 | 0 | 0 | 0 | 0 | 0 | 0 | 0 | 1 | 1 | Bacteria | Bacteria\_none | Actinomycetota | Actinomycetes | Micrococcales | Bacteria;Bacteria\_none;Actinomycetota;Actinomycetes;Micrococcales;Dermatophilaceae |
| Microbacteriaceae | 1 | 0 | 0 | 2 | 2 | 0 | 1 | 1 | 1 | 0 | 0 | 0 | 1 | 9 | Bacteria | Bacteria\_none | Actinomycetota | Actinomycetes | Micrococcales | Bacteria;Bacteria\_none;Actinomycetota;Actinomycetes;Micrococcales;Microbacteriaceae |
| Micrococcaceae | 27 | 111 | 23 | 17 | 125 | 42 | 36 | 61 | 14 | 80 | 157 | 167 | 213 | 1073 | Bacteria | Bacteria\_none | Actinomycetota | Actinomycetes | Micrococcales | Bacteria;Bacteria\_none;Actinomycetota;Actinomycetes;Micrococcales;Micrococcaceae |
| Micrococcales\_Incertae\_sedis | 0 | 0 | 0 | 0 | 0 | 0 | 0 | 0 | 0 | 0 | 0 | 0 | 1 | 1 | Bacteria | Bacteria\_none | Actinomycetota | Actinomycetes | Micrococcales | Bacteria;Bacteria\_none;Actinomycetota;Actinomycetes;Micrococcales;Micrococcales\_Incertae\_sedis |
| Ruaniaceae | 0 | 0 | 0 | 0 | 0 | 0 | 1 | 0 | 0 | 0 | 0 | 0 | 0 | 1 | Bacteria | Bacteria\_none | Actinomycetota | Actinomycetes | Micrococcales | Bacteria;Bacteria\_none;Actinomycetota;Actinomycetes;Micrococcales;Ruaniaceae |
| Micromonosporaceae | 1 | 0 | 0 | 1 | 0 | 1 | 0 | 1 | 0 | 3 | 0 | 0 | 1 | 8 | Bacteria | Bacteria\_none | Actinomycetota | Actinomycetes | Micromonosporales | Bacteria;Bacteria\_none;Actinomycetota;Actinomycetes;Micromonosporales;Micromonosporaceae |
| Corynebacteriaceae | 0 | 6 | 0 | 0 | 1 | 3 | 0 | 0 | 0 | 15 | 0 | 7 | 9 | 41 | Bacteria | Bacteria\_none | Actinomycetota | Actinomycetes | Mycobacteriales | Bacteria;Bacteria\_none;Actinomycetota;Actinomycetes;Mycobacteriales;Corynebacteriaceae |
| Gordoniaceae | 0 | 0 | 0 | 0 | 2 | 0 | 0 | 0 | 0 | 0 | 0 | 0 | 0 | 2 | Bacteria | Bacteria\_none | Actinomycetota | Actinomycetes | Mycobacteriales | Bacteria;Bacteria\_none;Actinomycetota;Actinomycetes;Mycobacteriales;Gordoniaceae |
| Mycobacteriaceae | 0 | 1 | 2 | 1 | 0 | 2 | 2 | 0 | 0 | 0 | 0 | 0 | 1 | 9 | Bacteria | Bacteria\_none | Actinomycetota | Actinomycetes | Mycobacteriales | Bacteria;Bacteria\_none;Actinomycetota;Actinomycetes;Mycobacteriales;Mycobacteriaceae |
| Nocardiaceae | 0 | 0 | 0 | 0 | 0 | 0 | 0 | 0 | 0 | 0 | 1 | 0 | 1 | 2 | Bacteria | Bacteria\_none | Actinomycetota | Actinomycetes | Mycobacteriales | Bacteria;Bacteria\_none;Actinomycetota;Actinomycetes;Mycobacteriales;Nocardiaceae |
| Kribbellaceae | 0 | 0 | 0 | 0 | 0 | 0 | 0 | 0 | 0 | 0 | 1 | 0 | 0 | 1 | Bacteria | Bacteria\_none | Actinomycetota | Actinomycetes | Propionibacteriales | Bacteria;Bacteria\_none;Actinomycetota;Actinomycetes;Propionibacteriales;Kribbellaceae |
| Nocardioidaceae | 0 | 0 | 1 | 2 | 1 | 0 | 0 | 0 | 0 | 0 | 0 | 0 | 0 | 4 | Bacteria | Bacteria\_none | Actinomycetota | Actinomycetes | Propionibacteriales | Bacteria;Bacteria\_none;Actinomycetota;Actinomycetes;Propionibacteriales;Nocardioidaceae |
| Propionibacteriaceae | 0 | 1 | 0 | 1 | 1 | 3 | 0 | 1 | 0 | 1 | 44 | 0 | 10 | 62 | Bacteria | Bacteria\_none | Actinomycetota | Actinomycetes | Propionibacteriales | Bacteria;Bacteria\_none;Actinomycetota;Actinomycetes;Propionibacteriales;Propionibacteriaceae |
| Pseudonocardiaceae | 0 | 0 | 0 | 1 | 1 | 1 | 0 | 0 | 0 | 0 | 0 | 0 | 0 | 3 | Bacteria | Bacteria\_none | Actinomycetota | Actinomycetes | Pseudonocardiales | Bacteria;Bacteria\_none;Actinomycetota;Actinomycetes;Pseudonocardiales;Pseudonocardiaceae |
| Streptosporangiaceae | 0 | 0 | 1 | 0 | 1 | 1 | 1 | 0 | 0 | 0 | 0 | 0 | 0 | 4 | Bacteria | Bacteria\_none | Actinomycetota | Actinomycetes | Streptosporangiales | Bacteria;Bacteria\_none;Actinomycetota;Actinomycetes;Streptosporangiales;Streptosporangiaceae |
| Thermomonosporaceae | 0 | 0 | 0 | 1 | 0 | 0 | 1 | 0 | 0 | 0 | 0 | 0 | 0 | 2 | Bacteria | Bacteria\_none | Actinomycetota | Actinomycetes | Streptosporangiales | Bacteria;Bacteria\_none;Actinomycetota;Actinomycetes;Streptosporangiales;Thermomonosporaceae |
| Atopobiaceae | 28 | 5 | 10 | 0 | 2 | 133 | 62 | 7 | 1 | 0 | 7 | 4 | 35 | 294 | Bacteria | Bacteria\_none | Actinomycetota | Coriobacteriia | Coriobacteriales | Bacteria;Bacteria\_none;Actinomycetota;Coriobacteriia;Coriobacteriales;Atopobiaceae |
| Eggerthellaceae | 0 | 0 | 0 | 0 | 0 | 2 | 1 | 0 | 0 | 0 | 0 | 0 | 0 | 3 | Bacteria | Bacteria\_none | Actinomycetota | Coriobacteriia | Eggerthellales | Bacteria;Bacteria\_none;Actinomycetota;Coriobacteriia;Eggerthellales;Eggerthellaceae |
| Rubrobacteraceae | 0 | 0 | 0 | 0 | 1 | 0 | 0 | 0 | 0 | 0 | 0 | 0 | 0 | 1 | Bacteria | Bacteria\_none | Actinomycetota | Rubrobacteria | Rubrobacterales | Bacteria;Bacteria\_none;Actinomycetota;Rubrobacteria;Rubrobacterales;Rubrobacteraceae |
| Miltoncostaeaceae | 0 | 1 | 0 | 0 | 0 | 0 | 0 | 0 | 0 | 0 | 0 | 0 | 0 | 1 | Bacteria | Bacteria\_none | Actinomycetota | Thermoleophilia | Miltoncostaeales | Bacteria;Bacteria\_none;Actinomycetota;Thermoleophilia;Miltoncostaeales;Miltoncostaeaceae |
| Baekduiaceae | 0 | 1 | 0 | 0 | 0 | 0 | 0 | 0 | 0 | 0 | 0 | 0 | 0 | 1 | Bacteria | Bacteria\_none | Actinomycetota | Thermoleophilia | Solirubrobacterales | Bacteria;Bacteria\_none;Actinomycetota;Thermoleophilia;Solirubrobacterales;Baekduiaceae |
| Abyssicoccaceae | 0 | 0 | 0 | 0 | 1 | 0 | 0 | 0 | 0 | 0 | 0 | 0 | 0 | 1 | Bacteria | Bacteria\_none | Bacillota | Bacilli | Bacillales | Bacteria;Bacteria\_none;Bacillota;Bacilli;Bacillales;Abyssicoccaceae |
| Alicyclobacillaceae | 0 | 0 | 0 | 0 | 0 | 0 | 0 | 0 | 0 | 1 | 0 | 0 | 0 | 1 | Bacteria | Bacteria\_none | Bacillota | Bacilli | Bacillales | Bacteria;Bacteria\_none;Bacillota;Bacilli;Bacillales;Alicyclobacillaceae |
| Bacillaceae | 4 | 3 | 7 | 9 | 6 | 13 | 3 | 3 | 0 | 3 | 4 | 0 | 19 | 74 | Bacteria | Bacteria\_none | Bacillota | Bacilli | Bacillales | Bacteria;Bacteria\_none;Bacillota;Bacilli;Bacillales;Bacillaceae |
| Bacillales\_Incertae\_sedis | 0 | 0 | 0 | 1 | 0 | 0 | 0 | 0 | 0 | 0 | 0 | 0 | 0 | 1 | Bacteria | Bacteria\_none | Bacillota | Bacilli | Bacillales | Bacteria;Bacteria\_none;Bacillota;Bacilli;Bacillales;Bacillales\_Incertae\_sedis |
| Gemellaceae | 74 | 125 | 7 | 131 | 563 | 347 | 132 | 115 | 7 | 386 | 1176 | 100 | 981 | 4144 | Bacteria | Bacteria\_none | Bacillota | Bacilli | Bacillales | Bacteria;Bacteria\_none;Bacillota;Bacilli;Bacillales;Gemellaceae |
| Listeriaceae | 0 | 0 | 0 | 0 | 0 | 0 | 0 | 0 | 0 | 0 | 0 | 0 | 2 | 2 | Bacteria | Bacteria\_none | Bacillota | Bacilli | Bacillales | Bacteria;Bacteria\_none;Bacillota;Bacilli;Bacillales;Listeriaceae |
| Paenibacillaceae | 2 | 1 | 2 | 2 | 1 | 2 | 5 | 0 | 0 | 0 | 3 | 0 | 5 | 23 | Bacteria | Bacteria\_none | Bacillota | Bacilli | Bacillales | Bacteria;Bacteria\_none;Bacillota;Bacilli;Bacillales;Paenibacillaceae |
| Planococcaceae | 1 | 0 | 0 | 0 | 0 | 0 | 0 | 0 | 0 | 0 | 0 | 0 | 2 | 3 | Bacteria | Bacteria\_none | Bacillota | Bacilli | Bacillales | Bacteria;Bacteria\_none;Bacillota;Bacilli;Bacillales;Planococcaceae |
| Staphylococcaceae | 1 | 3 | 3 | 3 | 3 | 0 | 1 | 0 | 0 | 1 | 3 | 2 | 6 | 26 | Bacteria | Bacteria\_none | Bacillota | Bacilli | Bacillales | Bacteria;Bacteria\_none;Bacillota;Bacilli;Bacillales;Staphylococcaceae |
| Aerococcaceae | 8 | 1 | 62 | 4 | 78 | 19 | 25 | 0 | 0 | 23 | 81 | 5 | 60 | 366 | Bacteria | Bacteria\_none | Bacillota | Bacilli | Lactobacillales | Bacteria;Bacteria\_none;Bacillota;Bacilli;Lactobacillales;Aerococcaceae |
| Carnobacteriaceae | 432 | 65 | 426 | 147 | 212 | 130 | 519 | 28 | 0 | 216 | 152 | 30 | 479 | 2836 | Bacteria | Bacteria\_none | Bacillota | Bacilli | Lactobacillales | Bacteria;Bacteria\_none;Bacillota;Bacilli;Lactobacillales;Carnobacteriaceae |
| Enterococcaceae | 3 | 10 | 11 | 3 | 3 | 6 | 5 | 3 | 0 | 0 | 5 | 0 | 7 | 56 | Bacteria | Bacteria\_none | Bacillota | Bacilli | Lactobacillales | Bacteria;Bacteria\_none;Bacillota;Bacilli;Lactobacillales;Enterococcaceae |
| Lactobacillaceae | 2 | 0 | 3 | 3 | 3 | 1 | 27 | 5 | 2 | 4 | 4 | 0 | 18 | 72 | Bacteria | Bacteria\_none | Bacillota | Bacilli | Lactobacillales | Bacteria;Bacteria\_none;Bacillota;Bacilli;Lactobacillales;Lactobacillaceae |
| Streptococcaceae | 1847 | 1371 | 726 | 419 | 2469 | 1777 | 2245 | 415 | 583 | 1034 | 2827 | 1010 | 4742 | 21465 | Bacteria | Bacteria\_none | Bacillota | Bacilli | Lactobacillales | Bacteria;Bacteria\_none;Bacillota;Bacilli;Lactobacillales;Streptococcaceae |
| Caldicellulosiruptoraceae | 0 | 0 | 0 | 0 | 1 | 0 | 0 | 0 | 0 | 0 | 0 | 0 | 0 | 1 | Bacteria | Bacteria\_none | Bacillota | Bacillota\_Incertae\_sedis | Caldicellulosiruptorales | Bacteria;Bacteria\_none;Bacillota;Bacillota\_Incertae\_sedis;Caldicellulosiruptorales;Caldicellulosiruptoraceae |
| Aristaeellaceae | 0 | 0 | 0 | 0 | 0 | 0 | 0 | 1 | 0 | 0 | 0 | 0 | 0 | 1 | Bacteria | Bacteria\_none | Bacillota | Clostridia | Eubacteriales | Bacteria;Bacteria\_none;Bacillota;Clostridia;Eubacteriales;Aristaeellaceae |
| Clostridiaceae | 1 | 2 | 3 | 5 | 4 | 12 | 0 | 1 | 4 | 0 | 3 | 1 | 16 | 52 | Bacteria | Bacteria\_none | Bacillota | Clostridia | Eubacteriales | Bacteria;Bacteria\_none;Bacillota;Clostridia;Eubacteriales;Clostridiaceae |
| Clostridiales Family XVI. Incertae Sedis | 0 | 0 | 0 | 0 | 0 | 0 | 0 | 0 | 0 | 0 | 0 | 0 | 1 | 1 | Bacteria | Bacteria\_none | Bacillota | Clostridia | Eubacteriales | Bacteria;Bacteria\_none;Bacillota;Clostridia;Eubacteriales;Clostridiales Family XVI. Incertae Sedis |
| Desulfitobacteriaceae | 0 | 0 | 0 | 0 | 0 | 1 | 0 | 0 | 1 | 0 | 0 | 0 | 1 | 3 | Bacteria | Bacteria\_none | Bacillota | Clostridia | Eubacteriales | Bacteria;Bacteria\_none;Bacillota;Clostridia;Eubacteriales;Desulfitobacteriaceae |
| Eubacteriaceae | 0 | 0 | 1 | 0 | 0 | 1 | 1 | 0 | 0 | 0 | 0 | 0 | 1 | 4 | Bacteria | Bacteria\_none | Bacillota | Clostridia | Eubacteriales | Bacteria;Bacteria\_none;Bacillota;Clostridia;Eubacteriales;Eubacteriaceae |
| Eubacteriales Family XIII. Incertae Sedis | 0 | 0 | 0 | 0 | 0 | 0 | 0 | 0 | 0 | 0 | 0 | 1 | 1 | 2 | Bacteria | Bacteria\_none | Bacillota | Clostridia | Eubacteriales | Bacteria;Bacteria\_none;Bacillota;Clostridia;Eubacteriales;Eubacteriales Family XIII. Incertae Sedis |
| Eubacteriales\_Incertae\_sedis | 0 | 0 | 0 | 0 | 0 | 2 | 0 | 0 | 0 | 0 | 2 | 0 | 0 | 4 | Bacteria | Bacteria\_none | Bacillota | Clostridia | Eubacteriales | Bacteria;Bacteria\_none;Bacillota;Clostridia;Eubacteriales;Eubacteriales\_Incertae\_sedis |
| Oscillospiraceae | 3 | 3 | 2 | 2 | 2 | 6 | 5 | 0 | 0 | 1 | 1 | 0 | 3 | 28 | Bacteria | Bacteria\_none | Bacillota | Clostridia | Eubacteriales | Bacteria;Bacteria\_none;Bacillota;Clostridia;Eubacteriales;Oscillospiraceae |
| Halobacteroidaceae | 0 | 0 | 0 | 0 | 0 | 0 | 0 | 0 | 0 | 0 | 0 | 0 | 1 | 1 | Bacteria | Bacteria\_none | Bacillota | Clostridia | Halanaerobiales | Bacteria;Bacteria\_none;Bacillota;Clostridia;Halanaerobiales;Halobacteroidaceae |
| Anaerotignaceae | 0 | 0 | 0 | 0 | 0 | 0 | 1 | 0 | 0 | 0 | 0 | 0 | 0 | 1 | Bacteria | Bacteria\_none | Bacillota | Clostridia | Lachnospirales | Bacteria;Bacteria\_none;Bacillota;Clostridia;Lachnospirales;Anaerotignaceae |
| Lachnospiraceae | 1 | 35 | 28 | 17 | 15 | 66 | 13 | 1 | 1 | 11 | 5 | 8 | 30 | 231 | Bacteria | Bacteria\_none | Bacillota | Clostridia | Lachnospirales | Bacteria;Bacteria\_none;Bacillota;Clostridia;Lachnospirales;Lachnospiraceae |
| Monoglobaceae | 0 | 0 | 0 | 0 | 0 | 0 | 0 | 2 | 0 | 0 | 0 | 0 | 0 | 2 | Bacteria | Bacteria\_none | Bacillota | Clostridia | Monoglobales | Bacteria;Bacteria\_none;Bacillota;Clostridia;Monoglobales;Monoglobaceae |
| Natranaerobiaceae | 0 | 0 | 0 | 1 | 0 | 0 | 0 | 0 | 0 | 0 | 0 | 0 | 0 | 1 | Bacteria | Bacteria\_none | Bacillota | Clostridia | Natranaerobiales | Bacteria;Bacteria\_none;Bacillota;Clostridia;Natranaerobiales;Natranaerobiaceae |
| Natranaerofabaceae | 0 | 0 | 0 | 0 | 0 | 1 | 0 | 0 | 0 | 0 | 0 | 0 | 0 | 1 | Bacteria | Bacteria\_none | Bacillota | Clostridia | Natranaerobiales | Bacteria;Bacteria\_none;Bacillota;Clostridia;Natranaerobiales;Natranaerofabaceae |
| Anaerovoracaceae | 1 | 0 | 9 | 1 | 2 | 35 | 3 | 5 | 1 | 0 | 4 | 3 | 23 | 87 | Bacteria | Bacteria\_none | Bacillota | Clostridia | Peptostreptococcales | Bacteria;Bacteria\_none;Bacillota;Clostridia;Peptostreptococcales;Anaerovoracaceae |
| Filifactoraceae | 3 | 38 | 35 | 0 | 2 | 2 | 3 | 54 | 0 | 0 | 2 | 18 | 46 | 203 | Bacteria | Bacteria\_none | Bacillota | Clostridia | Peptostreptococcales | Bacteria;Bacteria\_none;Bacillota;Clostridia;Peptostreptococcales;Filifactoraceae |
| Natronincolaceae | 0 | 0 | 0 | 1 | 0 | 0 | 0 | 0 | 0 | 0 | 0 | 0 | 0 | 1 | Bacteria | Bacteria\_none | Bacillota | Clostridia | Peptostreptococcales | Bacteria;Bacteria\_none;Bacillota;Clostridia;Peptostreptococcales;Natronincolaceae |
| Peptostreptococcaceae | 1 | 1 | 3 | 5 | 7 | 1 | 1 | 0 | 1 | 0 | 4 | 0 | 4 | 28 | Bacteria | Bacteria\_none | Bacillota | Clostridia | Peptostreptococcales | Bacteria;Bacteria\_none;Bacillota;Clostridia;Peptostreptococcales;Peptostreptococcaceae |
| Thermoanaerobacteraceae | 0 | 1 | 0 | 0 | 0 | 0 | 0 | 0 | 0 | 1 | 0 | 0 | 0 | 2 | Bacteria | Bacteria\_none | Bacillota | Clostridia | Thermoanaerobacterales | Bacteria;Bacteria\_none;Bacillota;Clostridia;Thermoanaerobacterales;Thermoanaerobacteraceae |
| Coprobacillaceae | 0 | 0 | 0 | 1 | 0 | 1 | 2 | 0 | 0 | 0 | 0 | 0 | 2 | 6 | Bacteria | Bacteria\_none | Bacillota | Erysipelotrichia | Erysipelotrichales | Bacteria;Bacteria\_none;Bacillota;Erysipelotrichia;Erysipelotrichales;Coprobacillaceae |
| Erysipelotrichaceae | 14 | 17 | 92 | 9 | 16 | 522 | 47 | 21 | 1 | 8 | 10 | 2 | 152 | 911 | Bacteria | Bacteria\_none | Bacillota | Erysipelotrichia | Erysipelotrichales | Bacteria;Bacteria\_none;Bacillota;Erysipelotrichia;Erysipelotrichales;Erysipelotrichaceae |
| Selenomonadaceae | 5 | 7 | 16 | 6 | 9 | 14 | 8 | 10 | 0 | 2 | 4 | 10 | 16 | 107 | Bacteria | Bacteria\_none | Bacillota | Negativicutes | Selenomonadales | Bacteria;Bacteria\_none;Bacillota;Negativicutes;Selenomonadales;Selenomonadaceae |
| Sporomusaceae | 0 | 0 | 0 | 1 | 0 | 0 | 0 | 0 | 1 | 0 | 0 | 0 | 0 | 2 | Bacteria | Bacteria\_none | Bacillota | Negativicutes | Selenomonadales | Bacteria;Bacteria\_none;Bacillota;Negativicutes;Selenomonadales;Sporomusaceae |
| Veillonellaceae | 1186 | 78 | 43 | 100 | 69 | 53 | 37 | 73 | 70 | 143 | 14 | 126 | 655 | 2647 | Bacteria | Bacteria\_none | Bacillota | Negativicutes | Veillonellales | Bacteria;Bacteria\_none;Bacillota;Negativicutes;Veillonellales;Veillonellaceae |
| Acidilutibacteraceae | 0 | 0 | 0 | 0 | 1 | 0 | 0 | 0 | 0 | 0 | 0 | 0 | 0 | 1 | Bacteria | Bacteria\_none | Bacillota | Tissierellia | Tissierellales | Bacteria;Bacteria\_none;Bacillota;Tissierellia;Tissierellales;Acidilutibacteraceae |
| Gottschalkiaceae | 0 | 0 | 0 | 0 | 0 | 0 | 0 | 0 | 0 | 0 | 0 | 0 | 1 | 1 | Bacteria | Bacteria\_none | Bacillota | Tissierellia | Tissierellales | Bacteria;Bacteria\_none;Bacillota;Tissierellia;Tissierellales;Gottschalkiaceae |
| Peptoniphilaceae | 6 | 34 | 25 | 16 | 48 | 72 | 7 | 65 | 0 | 2 | 73 | 26 | 147 | 521 | Bacteria | Bacteria\_none | Bacillota | Tissierellia | Tissierellales | Bacteria;Bacteria\_none;Bacillota;Tissierellia;Tissierellales;Peptoniphilaceae |
| Tepidimicrobiaceae | 0 | 0 | 0 | 0 | 1 | 0 | 0 | 0 | 0 | 0 | 0 | 0 | 0 | 1 | Bacteria | Bacteria\_none | Bacillota | Tissierellia | Tissierellales | Bacteria;Bacteria\_none;Bacillota;Tissierellia;Tissierellales;Tepidimicrobiaceae |
| Tissierellia\_Incertae\_sedis | 0 | 0 | 0 | 1 | 1 | 0 | 0 | 0 | 0 | 0 | 12 | 1 | 5 | 20 | Bacteria | Bacteria\_none | Bacillota | Tissierellia | Tissierellia\_Incertae\_sedis | Bacteria;Bacteria\_none;Bacillota;Tissierellia;Tissierellia\_Incertae\_sedis;Tissierellia\_Incertae\_sedis |
| Bacteroidaceae | 2 | 3 | 1 | 10 | 1 | 7 | 0 | 3 | 0 | 1 | 8 | 1 | 10 | 47 | Bacteria | Bacteria\_none | Bacteroidota | Bacteroidia | Bacteroidales | Bacteria;Bacteria\_none;Bacteroidota;Bacteroidia;Bacteroidales;Bacteroidaceae |
| Barnesiellaceae | 0 | 0 | 0 | 0 | 0 | 1 | 0 | 0 | 0 | 0 | 0 | 0 | 0 | 1 | Bacteria | Bacteria\_none | Bacteroidota | Bacteroidia | Bacteroidales | Bacteria;Bacteria\_none;Bacteroidota;Bacteroidia;Bacteroidales;Barnesiellaceae |
| Dysgonomonadaceae | 0 | 0 | 0 | 0 | 0 | 0 | 0 | 0 | 0 | 0 | 0 | 1 | 0 | 1 | Bacteria | Bacteria\_none | Bacteroidota | Bacteroidia | Bacteroidales | Bacteria;Bacteria\_none;Bacteroidota;Bacteroidia;Bacteroidales;Dysgonomonadaceae |
| Muribaculaceae | 0 | 0 | 0 | 0 | 1 | 0 | 0 | 0 | 0 | 0 | 1 | 0 | 1 | 3 | Bacteria | Bacteria\_none | Bacteroidota | Bacteroidia | Bacteroidales | Bacteria;Bacteria\_none;Bacteroidota;Bacteroidia;Bacteroidales;Muribaculaceae |
| Odoribacteraceae | 0 | 0 | 0 | 0 | 0 | 0 | 0 | 0 | 0 | 0 | 0 | 1 | 1 | 2 | Bacteria | Bacteria\_none | Bacteroidota | Bacteroidia | Bacteroidales | Bacteria;Bacteria\_none;Bacteroidota;Bacteroidia;Bacteroidales;Odoribacteraceae |
| Porphyromonadaceae | 5 | 129 | 9 | 52 | 63 | 32 | 13 | 105 | 0 | 52 | 91 | 45 | 103 | 699 | Bacteria | Bacteria\_none | Bacteroidota | Bacteroidia | Bacteroidales | Bacteria;Bacteria\_none;Bacteroidota;Bacteroidia;Bacteroidales;Porphyromonadaceae |
| Prevotellaceae | 336 | 981 | 287 | 891 | 85 | 240 | 204 | 62 | 341 | 142 | 228 | 347 | 1043 | 5187 | Bacteria | Bacteria\_none | Bacteroidota | Bacteroidia | Bacteroidales | Bacteria;Bacteria\_none;Bacteroidota;Bacteroidia;Bacteroidales;Prevotellaceae |
| Rikenellaceae | 0 | 2 | 0 | 0 | 0 | 2 | 0 | 0 | 0 | 1 | 0 | 0 | 1 | 6 | Bacteria | Bacteria\_none | Bacteroidota | Bacteroidia | Bacteroidales | Bacteria;Bacteria\_none;Bacteroidota;Bacteroidia;Bacteroidales;Rikenellaceae |
| Salinivirgaceae | 0 | 0 | 0 | 1 | 0 | 0 | 0 | 0 | 0 | 0 | 0 | 0 | 1 | 2 | Bacteria | Bacteria\_none | Bacteroidota | Bacteroidia | Bacteroidales | Bacteria;Bacteria\_none;Bacteroidota;Bacteroidia;Bacteroidales;Salinivirgaceae |
| Tannerellaceae | 2 | 37 | 34 | 13 | 19 | 103 | 5 | 54 | 0 | 44 | 47 | 24 | 102 | 484 | Bacteria | Bacteria\_none | Bacteroidota | Bacteroidia | Bacteroidales | Bacteria;Bacteria\_none;Bacteroidota;Bacteroidia;Bacteroidales;Tannerellaceae |
| Marinifilaceae | 1 | 0 | 1 | 0 | 0 | 0 | 0 | 0 | 0 | 0 | 0 | 0 | 0 | 2 | Bacteria | Bacteria\_none | Bacteroidota | Bacteroidia | Marinilabiliales | Bacteria;Bacteria\_none;Bacteroidota;Bacteroidia;Marinilabiliales;Marinifilaceae |
| Marinilabiliaceae | 0 | 0 | 1 | 1 | 0 | 0 | 0 | 2 | 0 | 0 | 0 | 0 | 0 | 4 | Bacteria | Bacteria\_none | Bacteroidota | Bacteroidia | Marinilabiliales | Bacteria;Bacteria\_none;Bacteroidota;Bacteroidia;Marinilabiliales;Marinilabiliaceae |
| Prolixibacteraceae | 0 | 0 | 0 | 2 | 0 | 1 | 0 | 0 | 0 | 0 | 2 | 0 | 0 | 5 | Bacteria | Bacteria\_none | Bacteroidota | Bacteroidia | Marinilabiliales | Bacteria;Bacteria\_none;Bacteroidota;Bacteroidia;Marinilabiliales;Prolixibacteraceae |
| Chitinophagaceae | 0 | 1 | 0 | 2 | 2 | 1 | 0 | 0 | 0 | 1 | 0 | 1 | 0 | 8 | Bacteria | Bacteria\_none | Bacteroidota | Chitinophagia | Chitinophagales | Bacteria;Bacteria\_none;Bacteroidota;Chitinophagia;Chitinophagales;Chitinophagaceae |
| Amoebophilaceae | 0 | 0 | 0 | 0 | 0 | 0 | 0 | 0 | 0 | 0 | 1 | 0 | 0 | 1 | Bacteria | Bacteria\_none | Bacteroidota | Cytophagia | Cytophagales | Bacteria;Bacteria\_none;Bacteroidota;Cytophagia;Cytophagales;Amoebophilaceae |
| Cyclobacteriaceae | 0 | 0 | 0 | 1 | 0 | 0 | 0 | 0 | 0 | 0 | 1 | 0 | 1 | 3 | Bacteria | Bacteria\_none | Bacteroidota | Cytophagia | Cytophagales | Bacteria;Bacteria\_none;Bacteroidota;Cytophagia;Cytophagales;Cyclobacteriaceae |
| Cytophagaceae | 0 | 0 | 0 | 0 | 0 | 0 | 0 | 1 | 0 | 0 | 0 | 0 | 0 | 1 | Bacteria | Bacteria\_none | Bacteroidota | Cytophagia | Cytophagales | Bacteria;Bacteria\_none;Bacteroidota;Cytophagia;Cytophagales;Cytophagaceae |
| Flammeovirgaceae | 0 | 0 | 0 | 0 | 2 | 0 | 0 | 0 | 0 | 0 | 0 | 0 | 0 | 2 | Bacteria | Bacteria\_none | Bacteroidota | Cytophagia | Cytophagales | Bacteria;Bacteria\_none;Bacteroidota;Cytophagia;Cytophagales;Flammeovirgaceae |
| Hymenobacteraceae | 0 | 0 | 0 | 1 | 2 | 0 | 1 | 0 | 0 | 1 | 0 | 0 | 3 | 8 | Bacteria | Bacteria\_none | Bacteroidota | Cytophagia | Cytophagales | Bacteria;Bacteria\_none;Bacteroidota;Cytophagia;Cytophagales;Hymenobacteraceae |
| Leadbetterellaceae | 1 | 0 | 1 | 0 | 0 | 0 | 0 | 0 | 0 | 0 | 0 | 0 | 0 | 2 | Bacteria | Bacteria\_none | Bacteroidota | Cytophagia | Cytophagales | Bacteria;Bacteria\_none;Bacteroidota;Cytophagia;Cytophagales;Leadbetterellaceae |
| Marivirgaceae | 0 | 0 | 0 | 0 | 0 | 1 | 0 | 0 | 0 | 0 | 0 | 0 | 1 | 2 | Bacteria | Bacteria\_none | Bacteroidota | Cytophagia | Cytophagales | Bacteria;Bacteria\_none;Bacteroidota;Cytophagia;Cytophagales;Marivirgaceae |
| Persicobacteraceae | 0 | 0 | 0 | 1 | 1 | 0 | 0 | 0 | 0 | 0 | 0 | 0 | 0 | 2 | Bacteria | Bacteria\_none | Bacteroidota | Cytophagia | Cytophagales | Bacteria;Bacteria\_none;Bacteroidota;Cytophagia;Cytophagales;Persicobacteraceae |
| Spirosomataceae | 0 | 0 | 0 | 0 | 0 | 1 | 0 | 0 | 0 | 0 | 0 | 0 | 3 | 4 | Bacteria | Bacteria\_none | Bacteroidota | Cytophagia | Cytophagales | Bacteria;Bacteria\_none;Bacteroidota;Cytophagia;Cytophagales;Spirosomataceae |
| Blattabacteriaceae | 0 | 0 | 0 | 0 | 0 | 0 | 0 | 0 | 0 | 0 | 1 | 0 | 1 | 2 | Bacteria | Bacteria\_none | Bacteroidota | Flavobacteriia | Flavobacteriales | Bacteria;Bacteria\_none;Bacteroidota;Flavobacteriia;Flavobacteriales;Blattabacteriaceae |
| Crocinitomicaceae | 0 | 0 | 0 | 0 | 0 | 0 | 0 | 0 | 0 | 1 | 0 | 0 | 0 | 1 | Bacteria | Bacteria\_none | Bacteroidota | Flavobacteriia | Flavobacteriales | Bacteria;Bacteria\_none;Bacteroidota;Flavobacteriia;Flavobacteriales;Crocinitomicaceae |
| Flavobacteriaceae | 313 | 150 | 130 | 214 | 171 | 36 | 84 | 378 | 0 | 356 | 218 | 55 | 577 | 2682 | Bacteria | Bacteria\_none | Bacteroidota | Flavobacteriia | Flavobacteriales | Bacteria;Bacteria\_none;Bacteroidota;Flavobacteriia;Flavobacteriales;Flavobacteriaceae |
| Owenweeksiaceae | 0 | 1 | 0 | 0 | 1 | 0 | 0 | 0 | 0 | 0 | 0 | 0 | 0 | 2 | Bacteria | Bacteria\_none | Bacteroidota | Flavobacteriia | Flavobacteriales | Bacteria;Bacteria\_none;Bacteroidota;Flavobacteriia;Flavobacteriales;Owenweeksiaceae |
| Sanyastnellaceae | 0 | 0 | 1 | 0 | 0 | 0 | 0 | 0 | 0 | 0 | 0 | 0 | 0 | 1 | Bacteria | Bacteria\_none | Bacteroidota | Flavobacteriia | Flavobacteriales | Bacteria;Bacteria\_none;Bacteroidota;Flavobacteriia;Flavobacteriales;Sanyastnellaceae |
| Weeksellaceae | 4 | 1 | 4 | 6 | 14 | 5 | 0 | 4 | 0 | 6 | 3 | 1 | 4 | 52 | Bacteria | Bacteria\_none | Bacteroidota | Flavobacteriia | Flavobacteriales | Bacteria;Bacteria\_none;Bacteroidota;Flavobacteriia;Flavobacteriales;Weeksellaceae |
| Haliscomenobacteraceae | 0 | 0 | 0 | 0 | 0 | 0 | 0 | 0 | 0 | 0 | 0 | 0 | 1 | 1 | Bacteria | Bacteria\_none | Bacteroidota | Saprospiria | Saprospirales | Bacteria;Bacteria\_none;Bacteroidota;Saprospiria;Saprospirales;Haliscomenobacteraceae |
| Aurantibacillaceae | 0 | 0 | 0 | 1 | 0 | 0 | 0 | 0 | 0 | 0 | 0 | 0 | 0 | 1 | Bacteria | Bacteria\_none | Bacteroidota | Sphingobacteriia | Sphingobacteriales | Bacteria;Bacteria\_none;Bacteroidota;Sphingobacteriia;Sphingobacteriales;Aurantibacillaceae |
| Sphingobacteriaceae | 0 | 2 | 0 | 3 | 1 | 1 | 0 | 1 | 0 | 1 | 3 | 1 | 2 | 15 | Bacteria | Bacteria\_none | Bacteroidota | Sphingobacteriia | Sphingobacteriales | Bacteria;Bacteria\_none;Bacteroidota;Sphingobacteriia;Sphingobacteriales;Sphingobacteriaceae |
| Cyclonatronaceae | 0 | 0 | 0 | 1 | 0 | 0 | 0 | 0 | 0 | 0 | 0 | 0 | 0 | 1 | Bacteria | Bacteria\_none | Balneolota | Balneolia | Balneolales | Bacteria;Bacteria\_none;Balneolota;Balneolia;Balneolales;Cyclonatronaceae |
| Pseudobdellovibrionaceae | 0 | 0 | 0 | 0 | 0 | 0 | 0 | 0 | 0 | 1 | 0 | 0 | 0 | 1 | Bacteria | Bacteria\_none | Bdellovibrionota | Bdellovibrionia | Bdellovibrionales | Bacteria;Bacteria\_none;Bdellovibrionota;Bdellovibrionia;Bdellovibrionales;Pseudobdellovibrionaceae |
| Calditrichaceae | 0 | 0 | 0 | 1 | 0 | 0 | 0 | 0 | 0 | 0 | 0 | 0 | 0 | 1 | Bacteria | Bacteria\_none | Calditrichota | Calditrichia | Calditrichales | Bacteria;Bacteria\_none;Calditrichota;Calditrichia;Calditrichales;Calditrichaceae |
| Arcobacteraceae | 0 | 0 | 2 | 0 | 0 | 0 | 0 | 0 | 0 | 2 | 0 | 0 | 3 | 7 | Bacteria | Bacteria\_none | Campylobacterota | Epsilonproteobacteria | Campylobacterales | Bacteria;Bacteria\_none;Campylobacterota;Epsilonproteobacteria;Campylobacterales;Arcobacteraceae |
| Campylobacteraceae | 0 | 47 | 14 | 11 | 10 | 49 | 2 | 11 | 9 | 9 | 3 | 14 | 57 | 236 | Bacteria | Bacteria\_none | Campylobacterota | Epsilonproteobacteria | Campylobacterales | Bacteria;Bacteria\_none;Campylobacterota;Epsilonproteobacteria;Campylobacterales;Campylobacteraceae |
| Helicobacteraceae | 0 | 0 | 0 | 0 | 1 | 1 | 0 | 0 | 0 | 0 | 0 | 0 | 1 | 3 | Bacteria | Bacteria\_none | Campylobacterota | Epsilonproteobacteria | Campylobacterales | Bacteria;Bacteria\_none;Campylobacterota;Epsilonproteobacteria;Campylobacterales;Helicobacteraceae |
| Sulfurospirillaceae | 0 | 0 | 0 | 0 | 0 | 0 | 0 | 0 | 0 | 1 | 0 | 0 | 0 | 1 | Bacteria | Bacteria\_none | Campylobacterota | Epsilonproteobacteria | Campylobacterales | Bacteria;Bacteria\_none;Campylobacterota;Epsilonproteobacteria;Campylobacterales;Sulfurospirillaceae |
| Sulfurovaceae | 1 | 0 | 0 | 0 | 0 | 0 | 0 | 0 | 0 | 0 | 0 | 0 | 0 | 1 | Bacteria | Bacteria\_none | Campylobacterota | Epsilonproteobacteria | Campylobacterales | Bacteria;Bacteria\_none;Campylobacterota;Epsilonproteobacteria;Campylobacterales;Sulfurovaceae |
| Candidatus Saccharibacteria\_Incertae\_sedis | 0 | 0 | 0 | 0 | 2 | 20 | 0 | 0 | 0 | 0 | 1 | 1 | 5 | 29 | Bacteria | Bacteria\_none | Candidatus Saccharibacteria | Candidatus Saccharibacteria\_Incertae\_sedis | Candidatus Saccharibacteria\_Incertae\_sedis | Bacteria;Bacteria\_none;Candidatus Saccharibacteria;Candidatus Saccharibacteria\_Incertae\_sedis;Candidatus Saccharibacteria\_Incertae\_sedis;Candidatus Saccharibacteria\_Incertae\_sedis |
| Candidatus Nanosynbacteraceae | 0 | 8 | 5 | 0 | 10 | 165 | 1 | 1 | 3 | 2 | 92 | 6 | 48 | 341 | Bacteria | Bacteria\_none | Candidatus Saccharibacteria | Candidatus Saccharimonadia | Candidatus Nanosynbacterales | Bacteria;Bacteria\_none;Candidatus Saccharibacteria;Candidatus Saccharimonadia;Candidatus Nanosynbacterales;Candidatus Nanosynbacteraceae |
| Candidatus Saccharimonadaceae | 0 | 0 | 0 | 0 | 1 | 0 | 0 | 1 | 0 | 0 | 1 | 0 | 0 | 3 | Bacteria | Bacteria\_none | Candidatus Saccharibacteria | Candidatus Saccharimonadia | Candidatus Saccharimonadales | Bacteria;Bacteria\_none;Candidatus Saccharibacteria;Candidatus Saccharimonadia;Candidatus Saccharimonadales;Candidatus Saccharimonadaceae |
| Parachlamydiaceae | 0 | 0 | 0 | 0 | 0 | 0 | 0 | 0 | 0 | 0 | 0 | 0 | 1 | 1 | Bacteria | Bacteria\_none | Chlamydiota | Chlamydiia | Parachlamydiales | Bacteria;Bacteria\_none;Chlamydiota;Chlamydiia;Parachlamydiales;Parachlamydiaceae |
| Chloroherpetonaceae | 0 | 0 | 0 | 0 | 0 | 1 | 0 | 0 | 0 | 0 | 0 | 0 | 0 | 1 | Bacteria | Bacteria\_none | Chlorobiota | Chlorobiia | Chlorobiales | Bacteria;Bacteria\_none;Chlorobiota;Chlorobiia;Chlorobiales;Chloroherpetonaceae |
| Candidatus Chloroheliaceae | 0 | 0 | 0 | 0 | 0 | 0 | 0 | 0 | 0 | 0 | 1 | 0 | 0 | 1 | Bacteria | Bacteria\_none | Chloroflexota | Chloroflexia | Candidatus Chloroheliales | Bacteria;Bacteria\_none;Chloroflexota;Chloroflexia;Candidatus Chloroheliales;Candidatus Chloroheliaceae |
| Candidatus Lucifugimonadaceae | 0 | 0 | 0 | 0 | 0 | 0 | 0 | 0 | 0 | 0 | 0 | 0 | 1 | 1 | Bacteria | Bacteria\_none | Chloroflexota | Dehalococcoidia | Candidatus Lucifugimonadales | Bacteria;Bacteria\_none;Chloroflexota;Dehalococcoidia;Candidatus Lucifugimonadales;Candidatus Lucifugimonadaceae |
| Cyanobacteriota\_Incertae\_sedis | 1 | 0 | 0 | 0 | 0 | 0 | 0 | 0 | 0 | 0 | 0 | 0 | 0 | 1 | Bacteria | Bacteria\_none | Cyanobacteriota | Cyanobacteriota\_Incertae\_sedis | Cyanobacteriota\_Incertae\_sedis | Bacteria;Bacteria\_none;Cyanobacteriota;Cyanobacteriota\_Incertae\_sedis;Cyanobacteriota\_Incertae\_sedis;Cyanobacteriota\_Incertae\_sedis |
| Chroococcaceae | 0 | 0 | 1 | 0 | 0 | 0 | 0 | 0 | 0 | 0 | 0 | 0 | 0 | 1 | Bacteria | Bacteria\_none | Cyanobacteriota | Cyanophyceae | Chroococcales | Bacteria;Bacteria\_none;Cyanobacteriota;Cyanophyceae;Chroococcales;Chroococcaceae |
| Geminocystaceae | 0 | 0 | 0 | 0 | 0 | 0 | 0 | 0 | 0 | 0 | 1 | 0 | 0 | 1 | Bacteria | Bacteria\_none | Cyanobacteriota | Cyanophyceae | Chroococcales | Bacteria;Bacteria\_none;Cyanobacteriota;Cyanophyceae;Chroococcales;Geminocystaceae |
| Gloeobacteraceae | 0 | 0 | 0 | 0 | 0 | 0 | 1 | 0 | 0 | 0 | 0 | 0 | 0 | 1 | Bacteria | Bacteria\_none | Cyanobacteriota | Cyanophyceae | Gloeobacterales | Bacteria;Bacteria\_none;Cyanobacteriota;Cyanophyceae;Gloeobacterales;Gloeobacteraceae |
| Aphanizomenonaceae | 0 | 0 | 0 | 1 | 0 | 0 | 0 | 0 | 0 | 0 | 0 | 0 | 0 | 1 | Bacteria | Bacteria\_none | Cyanobacteriota | Cyanophyceae | Nostocales | Bacteria;Bacteria\_none;Cyanobacteriota;Cyanophyceae;Nostocales;Aphanizomenonaceae |
| Nostocaceae | 0 | 1 | 0 | 0 | 0 | 0 | 0 | 0 | 0 | 2 | 1 | 0 | 2 | 6 | Bacteria | Bacteria\_none | Cyanobacteriota | Cyanophyceae | Nostocales | Bacteria;Bacteria\_none;Cyanobacteriota;Cyanophyceae;Nostocales;Nostocaceae |
| Prochlorococcaceae | 0 | 0 | 0 | 0 | 1 | 0 | 0 | 0 | 0 | 0 | 0 | 0 | 1 | 2 | Bacteria | Bacteria\_none | Cyanobacteriota | Cyanophyceae | Synechococcales | Bacteria;Bacteria\_none;Cyanobacteriota;Cyanophyceae;Synechococcales;Prochlorococcaceae |
| Synechococcaceae | 0 | 0 | 0 | 0 | 0 | 1 | 0 | 0 | 0 | 0 | 0 | 0 | 0 | 1 | Bacteria | Bacteria\_none | Cyanobacteriota | Cyanophyceae | Synechococcales | Bacteria;Bacteria\_none;Cyanobacteriota;Cyanophyceae;Synechococcales;Synechococcaceae |
| Calditerrivibrionaceae | 0 | 1 | 0 | 0 | 0 | 0 | 0 | 0 | 0 | 0 | 0 | 0 | 0 | 1 | Bacteria | Bacteria\_none | Deferribacterota | Deferribacteres | Deferribacterales | Bacteria;Bacteria\_none;Deferribacterota;Deferribacteres;Deferribacterales;Calditerrivibrionaceae |
| Mucispirillaceae | 0 | 0 | 0 | 0 | 0 | 0 | 0 | 0 | 0 | 0 | 0 | 0 | 1 | 1 | Bacteria | Bacteria\_none | Deferribacterota | Deferribacteres | Deferribacterales | Bacteria;Bacteria\_none;Deferribacterota;Deferribacteres;Deferribacterales;Mucispirillaceae |
| Fusobacteriaceae | 1 | 169 | 61 | 742 | 74 | 27 | 17 | 185 | 0 | 38 | 192 | 106 | 868 | 2480 | Bacteria | Bacteria\_none | Fusobacteriota | Fusobacteriia | Fusobacteriales | Bacteria;Bacteria\_none;Fusobacteriota;Fusobacteriia;Fusobacteriales;Fusobacteriaceae |
| Haliovirgaceae | 0 | 0 | 0 | 0 | 0 | 0 | 0 | 0 | 0 | 0 | 0 | 0 | 1 | 1 | Bacteria | Bacteria\_none | Fusobacteriota | Fusobacteriia | Fusobacteriales | Bacteria;Bacteria\_none;Fusobacteriota;Fusobacteriia;Fusobacteriales;Haliovirgaceae |
| Leptotrichiaceae | 16 | 97 | 176 | 103 | 113 | 231 | 432 | 377 | 24 | 94 | 168 | 24 | 698 | 2553 | Bacteria | Bacteria\_none | Fusobacteriota | Fusobacteriia | Fusobacteriales | Bacteria;Bacteria\_none;Fusobacteriota;Fusobacteriia;Fusobacteriales;Leptotrichiaceae |
| Ignavibacteriaceae | 0 | 0 | 0 | 0 | 0 | 0 | 0 | 0 | 0 | 0 | 0 | 0 | 1 | 1 | Bacteria | Bacteria\_none | Ignavibacteriota | Ignavibacteria | Ignavibacteriales | Bacteria;Bacteria\_none;Ignavibacteriota;Ignavibacteria;Ignavibacteriales;Ignavibacteriaceae |
| Acholeplasmataceae | 0 | 0 | 0 | 0 | 1 | 1 | 0 | 0 | 0 | 0 | 0 | 0 | 0 | 2 | Bacteria | Bacteria\_none | Mycoplasmatota | Mollicutes | Acholeplasmatales | Bacteria;Bacteria\_none;Mycoplasmatota;Mollicutes;Acholeplasmatales;Acholeplasmataceae |
| Spiroplasmataceae | 0 | 0 | 0 | 1 | 1 | 3 | 0 | 0 | 0 | 1 | 0 | 0 | 3 | 9 | Bacteria | Bacteria\_none | Mycoplasmatota | Mollicutes | Entomoplasmatales | Bacteria;Bacteria\_none;Mycoplasmatota;Mollicutes;Entomoplasmatales;Spiroplasmataceae |
| Mycoplasmataceae | 0 | 1 | 3 | 0 | 0 | 1 | 0 | 0 | 0 | 0 | 0 | 0 | 1 | 6 | Bacteria | Bacteria\_none | Mycoplasmatota | Mollicutes | Mycoplasmatales | Bacteria;Bacteria\_none;Mycoplasmatota;Mollicutes;Mycoplasmatales;Mycoplasmataceae |
| Mycoplasmatales\_Incertae\_sedis | 0 | 0 | 0 | 0 | 0 | 0 | 0 | 0 | 0 | 0 | 1 | 0 | 0 | 1 | Bacteria | Bacteria\_none | Mycoplasmatota | Mollicutes | Mycoplasmatales | Bacteria;Bacteria\_none;Mycoplasmatota;Mollicutes;Mycoplasmatales;Mycoplasmatales\_Incertae\_sedis |
| Metamycoplasmataceae | 0 | 10 | 37 | 2 | 2 | 6 | 1 | 3 | 0 | 9 | 3 | 7 | 26 | 106 | Bacteria | Bacteria\_none | Mycoplasmatota | Mycoplasmatota\_Incertae\_sedis | Mycoplasmoidales | Bacteria;Bacteria\_none;Mycoplasmatota;Mycoplasmatota\_Incertae\_sedis;Mycoplasmoidales;Metamycoplasmataceae |
| Mycoplasmoidaceae | 0 | 0 | 0 | 0 | 1 | 0 | 0 | 0 | 0 | 0 | 0 | 0 | 1 | 2 | Bacteria | Bacteria\_none | Mycoplasmatota | Mycoplasmatota\_Incertae\_sedis | Mycoplasmoidales | Bacteria;Bacteria\_none;Mycoplasmatota;Mycoplasmatota\_Incertae\_sedis;Mycoplasmoidales;Mycoplasmoidaceae |
| Myxococcaceae | 0 | 0 | 0 | 0 | 0 | 0 | 0 | 0 | 0 | 0 | 0 | 0 | 1 | 1 | Bacteria | Bacteria\_none | Myxococcota | Myxococcia | Myxococcales | Bacteria;Bacteria\_none;Myxococcota;Myxococcia;Myxococcales;Myxococcaceae |
| Polyangiaceae | 0 | 0 | 0 | 0 | 2 | 0 | 0 | 0 | 0 | 0 | 1 | 0 | 0 | 3 | Bacteria | Bacteria\_none | Myxococcota | Myxococcota\_Incertae\_sedis | Polyangiales | Bacteria;Bacteria\_none;Myxococcota;Myxococcota\_Incertae\_sedis;Polyangiales;Polyangiaceae |
| Nitrospiraceae | 0 | 0 | 0 | 0 | 0 | 1 | 0 | 1 | 0 | 0 | 0 | 0 | 0 | 2 | Bacteria | Bacteria\_none | Nitrospirota | Nitrospiria | Nitrospirales | Bacteria;Bacteria\_none;Nitrospirota;Nitrospiria;Nitrospirales;Nitrospiraceae |
| Dissulfurispiraceae | 0 | 0 | 0 | 0 | 0 | 0 | 0 | 0 | 0 | 0 | 1 | 0 | 0 | 1 | Bacteria | Bacteria\_none | Nitrospirota | Thermodesulfovibrionia | Thermodesulfovibrionales | Bacteria;Bacteria\_none;Nitrospirota;Thermodesulfovibrionia;Thermodesulfovibrionales;Dissulfurispiraceae |
| Gemmataceae | 0 | 0 | 0 | 0 | 0 | 1 | 0 | 0 | 0 | 0 | 0 | 0 | 0 | 1 | Bacteria | Bacteria\_none | Planctomycetota | Planctomycetia | Gemmatales | Bacteria;Bacteria\_none;Planctomycetota;Planctomycetia;Gemmatales;Gemmataceae |
| Pirellulaceae | 1 | 0 | 0 | 0 | 0 | 0 | 0 | 0 | 0 | 0 | 0 | 0 | 0 | 1 | Bacteria | Bacteria\_none | Planctomycetota | Planctomycetia | Pirellulales | Bacteria;Bacteria\_none;Planctomycetota;Planctomycetia;Pirellulales;Pirellulaceae |
| Planctomycetaceae | 0 | 0 | 0 | 0 | 0 | 0 | 0 | 0 | 0 | 0 | 2 | 0 | 0 | 2 | Bacteria | Bacteria\_none | Planctomycetota | Planctomycetia | Planctomycetales | Bacteria;Bacteria\_none;Planctomycetota;Planctomycetia;Planctomycetales;Planctomycetaceae |
| Candidatus Pelagibacteraceae | 0 | 0 | 0 | 0 | 0 | 0 | 0 | 0 | 0 | 0 | 0 | 0 | 1 | 1 | Bacteria | Bacteria\_none | Pseudomonadota | Alphaproteobacteria | Candidatus Pelagibacterales | Bacteria;Bacteria\_none;Pseudomonadota;Alphaproteobacteria;Candidatus Pelagibacterales;Candidatus Pelagibacteraceae |
| Caulobacteraceae | 0 | 0 | 0 | 0 | 0 | 0 | 0 | 0 | 0 | 0 | 0 | 0 | 1 | 1 | Bacteria | Bacteria\_none | Pseudomonadota | Alphaproteobacteria | Caulobacterales | Bacteria;Bacteria\_none;Pseudomonadota;Alphaproteobacteria;Caulobacterales;Caulobacteraceae |
| Boseaceae | 0 | 0 | 0 | 0 | 0 | 1 | 0 | 0 | 0 | 0 | 0 | 0 | 0 | 1 | Bacteria | Bacteria\_none | Pseudomonadota | Alphaproteobacteria | Hyphomicrobiales | Bacteria;Bacteria\_none;Pseudomonadota;Alphaproteobacteria;Hyphomicrobiales;Boseaceae |
| Brucellaceae | 0 | 0 | 0 | 0 | 0 | 0 | 0 | 0 | 0 | 0 | 1 | 0 | 0 | 1 | Bacteria | Bacteria\_none | Pseudomonadota | Alphaproteobacteria | Hyphomicrobiales | Bacteria;Bacteria\_none;Pseudomonadota;Alphaproteobacteria;Hyphomicrobiales;Brucellaceae |
| Cohaesibacteraceae | 0 | 0 | 0 | 0 | 0 | 0 | 0 | 0 | 1 | 0 | 0 | 0 | 1 | 2 | Bacteria | Bacteria\_none | Pseudomonadota | Alphaproteobacteria | Hyphomicrobiales | Bacteria;Bacteria\_none;Pseudomonadota;Alphaproteobacteria;Hyphomicrobiales;Cohaesibacteraceae |
| Devosiaceae | 0 | 0 | 1 | 0 | 0 | 0 | 1 | 0 | 0 | 0 | 0 | 0 | 0 | 2 | Bacteria | Bacteria\_none | Pseudomonadota | Alphaproteobacteria | Hyphomicrobiales | Bacteria;Bacteria\_none;Pseudomonadota;Alphaproteobacteria;Hyphomicrobiales;Devosiaceae |
| Methylobacteriaceae | 0 | 0 | 1 | 0 | 0 | 0 | 0 | 0 | 0 | 0 | 1 | 0 | 0 | 2 | Bacteria | Bacteria\_none | Pseudomonadota | Alphaproteobacteria | Hyphomicrobiales | Bacteria;Bacteria\_none;Pseudomonadota;Alphaproteobacteria;Hyphomicrobiales;Methylobacteriaceae |
| Nitrobacteraceae | 0 | 1 | 1 | 0 | 0 | 1 | 0 | 0 | 0 | 0 | 2 | 0 | 0 | 5 | Bacteria | Bacteria\_none | Pseudomonadota | Alphaproteobacteria | Hyphomicrobiales | Bacteria;Bacteria\_none;Pseudomonadota;Alphaproteobacteria;Hyphomicrobiales;Nitrobacteraceae |
| Phyllobacteriaceae | 0 | 0 | 2 | 0 | 0 | 1 | 1 | 3 | 0 | 0 | 0 | 0 | 0 | 7 | Bacteria | Bacteria\_none | Pseudomonadota | Alphaproteobacteria | Hyphomicrobiales | Bacteria;Bacteria\_none;Pseudomonadota;Alphaproteobacteria;Hyphomicrobiales;Phyllobacteriaceae |
| Rhizobiaceae | 1 | 0 | 0 | 0 | 1 | 2 | 0 | 1 | 0 | 1 | 1 | 0 | 2 | 9 | Bacteria | Bacteria\_none | Pseudomonadota | Alphaproteobacteria | Hyphomicrobiales | Bacteria;Bacteria\_none;Pseudomonadota;Alphaproteobacteria;Hyphomicrobiales;Rhizobiaceae |
| Kordiimonadaceae | 0 | 0 | 0 | 0 | 0 | 0 | 0 | 1 | 0 | 0 | 0 | 0 | 0 | 1 | Bacteria | Bacteria\_none | Pseudomonadota | Alphaproteobacteria | Kordiimonadales | Bacteria;Bacteria\_none;Pseudomonadota;Alphaproteobacteria;Kordiimonadales;Kordiimonadaceae |
| Paracoccaceae | 0 | 0 | 0 | 0 | 0 | 1 | 0 | 0 | 1 | 0 | 1 | 0 | 1 | 4 | Bacteria | Bacteria\_none | Pseudomonadota | Alphaproteobacteria | Rhodobacterales | Bacteria;Bacteria\_none;Pseudomonadota;Alphaproteobacteria;Rhodobacterales;Paracoccaceae |
| Roseobacteraceae | 2 | 1 | 0 | 1 | 2 | 0 | 0 | 0 | 0 | 0 | 2 | 0 | 1 | 9 | Bacteria | Bacteria\_none | Pseudomonadota | Alphaproteobacteria | Rhodobacterales | Bacteria;Bacteria\_none;Pseudomonadota;Alphaproteobacteria;Rhodobacterales;Roseobacteraceae |
| Acetobacteraceae | 0 | 0 | 0 | 0 | 0 | 1 | 1 | 0 | 0 | 0 | 0 | 0 | 0 | 2 | Bacteria | Bacteria\_none | Pseudomonadota | Alphaproteobacteria | Rhodospirillales | Bacteria;Bacteria\_none;Pseudomonadota;Alphaproteobacteria;Rhodospirillales;Acetobacteraceae |
| Azospirillaceae | 0 | 0 | 0 | 0 | 1 | 0 | 0 | 0 | 0 | 0 | 1 | 0 | 0 | 2 | Bacteria | Bacteria\_none | Pseudomonadota | Alphaproteobacteria | Rhodospirillales | Bacteria;Bacteria\_none;Pseudomonadota;Alphaproteobacteria;Rhodospirillales;Azospirillaceae |
| Magnetovibrionaceae | 0 | 0 | 0 | 0 | 0 | 0 | 0 | 0 | 0 | 0 | 0 | 0 | 1 | 1 | Bacteria | Bacteria\_none | Pseudomonadota | Alphaproteobacteria | Rhodospirillales | Bacteria;Bacteria\_none;Pseudomonadota;Alphaproteobacteria;Rhodospirillales;Magnetovibrionaceae |
| Thalassospiraceae | 0 | 0 | 0 | 1 | 0 | 0 | 0 | 0 | 0 | 0 | 0 | 0 | 0 | 1 | Bacteria | Bacteria\_none | Pseudomonadota | Alphaproteobacteria | Rhodospirillales | Bacteria;Bacteria\_none;Pseudomonadota;Alphaproteobacteria;Rhodospirillales;Thalassospiraceae |
| Anaplasmataceae | 0 | 1 | 0 | 0 | 0 | 0 | 0 | 0 | 0 | 0 | 0 | 0 | 3 | 4 | Bacteria | Bacteria\_none | Pseudomonadota | Alphaproteobacteria | Rickettsiales | Bacteria;Bacteria\_none;Pseudomonadota;Alphaproteobacteria;Rickettsiales;Anaplasmataceae |
| Rickettsiaceae | 0 | 0 | 0 | 0 | 0 | 0 | 1 | 0 | 0 | 0 | 0 | 0 | 0 | 1 | Bacteria | Bacteria\_none | Pseudomonadota | Alphaproteobacteria | Rickettsiales | Bacteria;Bacteria\_none;Pseudomonadota;Alphaproteobacteria;Rickettsiales;Rickettsiaceae |
| Sphingomonadaceae | 0 | 0 | 0 | 1 | 0 | 1 | 0 | 0 | 0 | 0 | 2 | 1 | 2 | 7 | Bacteria | Bacteria\_none | Pseudomonadota | Alphaproteobacteria | Sphingomonadales | Bacteria;Bacteria\_none;Pseudomonadota;Alphaproteobacteria;Sphingomonadales;Sphingomonadaceae |
| Betaproteobacteria\_Incertae\_sedis | 0 | 0 | 0 | 0 | 1 | 0 | 0 | 0 | 0 | 0 | 0 | 0 | 0 | 1 | Bacteria | Bacteria\_none | Pseudomonadota | Betaproteobacteria | Betaproteobacteria\_Incertae\_sedis | Bacteria;Bacteria\_none;Pseudomonadota;Betaproteobacteria;Betaproteobacteria\_Incertae\_sedis;Betaproteobacteria\_Incertae\_sedis |
| Casimicrobiaceae | 0 | 0 | 0 | 0 | 0 | 0 | 0 | 1 | 0 | 0 | 0 | 0 | 0 | 1 | Bacteria | Bacteria\_none | Pseudomonadota | Betaproteobacteria | Betaproteobacteria\_Incertae\_sedis | Bacteria;Bacteria\_none;Pseudomonadota;Betaproteobacteria;Betaproteobacteria\_Incertae\_sedis;Casimicrobiaceae |
| Alcaligenaceae | 0 | 0 | 0 | 0 | 1 | 1 | 1 | 0 | 0 | 0 | 2 | 0 | 1 | 6 | Bacteria | Bacteria\_none | Pseudomonadota | Betaproteobacteria | Burkholderiales | Bacteria;Bacteria\_none;Pseudomonadota;Betaproteobacteria;Burkholderiales;Alcaligenaceae |
| Burkholderiaceae | 1 | 1 | 2 | 0 | 6 | 1 | 2 | 2 | 0 | 4 | 11 | 0 | 8 | 38 | Bacteria | Bacteria\_none | Pseudomonadota | Betaproteobacteria | Burkholderiales | Bacteria;Bacteria\_none;Pseudomonadota;Betaproteobacteria;Burkholderiales;Burkholderiaceae |
| Comamonadaceae | 0 | 0 | 0 | 0 | 0 | 1 | 1 | 1 | 0 | 0 | 1 | 0 | 1 | 5 | Bacteria | Bacteria\_none | Pseudomonadota | Betaproteobacteria | Burkholderiales | Bacteria;Bacteria\_none;Pseudomonadota;Betaproteobacteria;Burkholderiales;Comamonadaceae |
| Oxalobacteraceae | 0 | 0 | 1 | 0 | 1 | 1 | 0 | 0 | 1 | 1 | 0 | 0 | 1 | 6 | Bacteria | Bacteria\_none | Pseudomonadota | Betaproteobacteria | Burkholderiales | Bacteria;Bacteria\_none;Pseudomonadota;Betaproteobacteria;Burkholderiales;Oxalobacteraceae |
| Sphaerotilaceae | 0 | 0 | 0 | 0 | 0 | 0 | 0 | 0 | 0 | 0 | 1 | 1 | 0 | 2 | Bacteria | Bacteria\_none | Pseudomonadota | Betaproteobacteria | Burkholderiales | Bacteria;Bacteria\_none;Pseudomonadota;Betaproteobacteria;Burkholderiales;Sphaerotilaceae |
| Neisseriaceae | 205 | 202 | 1085 | 4097 | 357 | 328 | 61 | 194 | 1 | 1681 | 209 | 123 | 2195 | 10738 | Bacteria | Bacteria\_none | Pseudomonadota | Betaproteobacteria | Neisseriales | Bacteria;Bacteria\_none;Pseudomonadota;Betaproteobacteria;Neisseriales;Neisseriaceae |
| Gallionellaceae | 0 | 0 | 0 | 0 | 0 | 0 | 0 | 0 | 0 | 0 | 0 | 0 | 1 | 1 | Bacteria | Bacteria\_none | Pseudomonadota | Betaproteobacteria | Nitrosomonadales | Bacteria;Bacteria\_none;Pseudomonadota;Betaproteobacteria;Nitrosomonadales;Gallionellaceae |
| Methylophilaceae | 0 | 0 | 0 | 0 | 0 | 0 | 0 | 0 | 0 | 0 | 0 | 0 | 1 | 1 | Bacteria | Bacteria\_none | Pseudomonadota | Betaproteobacteria | Nitrosomonadales | Bacteria;Bacteria\_none;Pseudomonadota;Betaproteobacteria;Nitrosomonadales;Methylophilaceae |
| Azonexaceae | 0 | 0 | 0 | 0 | 0 | 0 | 1 | 0 | 0 | 0 | 0 | 0 | 0 | 1 | Bacteria | Bacteria\_none | Pseudomonadota | Betaproteobacteria | Rhodocyclales | Bacteria;Bacteria\_none;Pseudomonadota;Betaproteobacteria;Rhodocyclales;Azonexaceae |
| Aeromonadaceae | 0 | 0 | 0 | 0 | 0 | 2 | 0 | 0 | 0 | 0 | 0 | 0 | 1 | 3 | Bacteria | Bacteria\_none | Pseudomonadota | Gammaproteobacteria | Aeromonadales | Bacteria;Bacteria\_none;Pseudomonadota;Gammaproteobacteria;Aeromonadales;Aeromonadaceae |
| Alteromonadaceae | 1 | 0 | 0 | 0 | 0 | 0 | 0 | 0 | 0 | 0 | 0 | 0 | 2 | 3 | Bacteria | Bacteria\_none | Pseudomonadota | Gammaproteobacteria | Alteromonadales | Bacteria;Bacteria\_none;Pseudomonadota;Gammaproteobacteria;Alteromonadales;Alteromonadaceae |
| Colwelliaceae | 0 | 0 | 1 | 0 | 0 | 0 | 0 | 0 | 0 | 0 | 0 | 0 | 0 | 1 | Bacteria | Bacteria\_none | Pseudomonadota | Gammaproteobacteria | Alteromonadales | Bacteria;Bacteria\_none;Pseudomonadota;Gammaproteobacteria;Alteromonadales;Colwelliaceae |
| Ferrimonadaceae | 0 | 0 | 0 | 0 | 1 | 0 | 0 | 0 | 0 | 0 | 0 | 0 | 0 | 1 | Bacteria | Bacteria\_none | Pseudomonadota | Gammaproteobacteria | Alteromonadales | Bacteria;Bacteria\_none;Pseudomonadota;Gammaproteobacteria;Alteromonadales;Ferrimonadaceae |
| Pseudoalteromonadaceae | 1 | 0 | 0 | 0 | 1 | 2 | 0 | 2 | 0 | 0 | 1 | 0 | 0 | 7 | Bacteria | Bacteria\_none | Pseudomonadota | Gammaproteobacteria | Alteromonadales | Bacteria;Bacteria\_none;Pseudomonadota;Gammaproteobacteria;Alteromonadales;Pseudoalteromonadaceae |
| Shewanellaceae | 1 | 1 | 0 | 0 | 1 | 0 | 0 | 1 | 0 | 0 | 0 | 1 | 0 | 5 | Bacteria | Bacteria\_none | Pseudomonadota | Gammaproteobacteria | Alteromonadales | Bacteria;Bacteria\_none;Pseudomonadota;Gammaproteobacteria;Alteromonadales;Shewanellaceae |
| Cardiobacteriaceae | 0 | 2 | 14 | 3 | 2 | 6 | 0 | 2 | 0 | 1 | 14 | 2 | 7 | 53 | Bacteria | Bacteria\_none | Pseudomonadota | Gammaproteobacteria | Cardiobacteriales | Bacteria;Bacteria\_none;Pseudomonadota;Gammaproteobacteria;Cardiobacteriales;Cardiobacteriaceae |
| Ignatzschineriaceae | 0 | 1 | 0 | 0 | 0 | 0 | 0 | 0 | 0 | 0 | 0 | 0 | 0 | 1 | Bacteria | Bacteria\_none | Pseudomonadota | Gammaproteobacteria | Cardiobacteriales | Bacteria;Bacteria\_none;Pseudomonadota;Gammaproteobacteria;Cardiobacteriales;Ignatzschineriaceae |
| Cellvibrionaceae | 0 | 0 | 0 | 0 | 0 | 0 | 0 | 1 | 0 | 0 | 0 | 0 | 0 | 1 | Bacteria | Bacteria\_none | Pseudomonadota | Gammaproteobacteria | Cellvibrionales | Bacteria;Bacteria\_none;Pseudomonadota;Gammaproteobacteria;Cellvibrionales;Cellvibrionaceae |
| Chromatiaceae | 0 | 0 | 0 | 0 | 0 | 0 | 0 | 0 | 0 | 0 | 0 | 0 | 2 | 2 | Bacteria | Bacteria\_none | Pseudomonadota | Gammaproteobacteria | Chromatiales | Bacteria;Bacteria\_none;Pseudomonadota;Gammaproteobacteria;Chromatiales;Chromatiaceae |
| Ectothiorhodospiraceae | 0 | 0 | 0 | 1 | 0 | 0 | 0 | 0 | 0 | 0 | 0 | 0 | 0 | 1 | Bacteria | Bacteria\_none | Pseudomonadota | Gammaproteobacteria | Chromatiales | Bacteria;Bacteria\_none;Pseudomonadota;Gammaproteobacteria;Chromatiales;Ectothiorhodospiraceae |
| Budviciaceae | 0 | 0 | 0 | 0 | 0 | 0 | 0 | 1 | 0 | 0 | 0 | 0 | 0 | 1 | Bacteria | Bacteria\_none | Pseudomonadota | Gammaproteobacteria | Enterobacterales | Bacteria;Bacteria\_none;Pseudomonadota;Gammaproteobacteria;Enterobacterales;Budviciaceae |
| Enterobacteriaceae | 4 | 0 | 0 | 4 | 5 | 1 | 0 | 4 | 0 | 4 | 0 | 1 | 12 | 35 | Bacteria | Bacteria\_none | Pseudomonadota | Gammaproteobacteria | Enterobacterales | Bacteria;Bacteria\_none;Pseudomonadota;Gammaproteobacteria;Enterobacterales;Enterobacteriaceae |
| Erwiniaceae | 0 | 0 | 0 | 0 | 1 | 0 | 0 | 3 | 0 | 0 | 1 | 1 | 2 | 8 | Bacteria | Bacteria\_none | Pseudomonadota | Gammaproteobacteria | Enterobacterales | Bacteria;Bacteria\_none;Pseudomonadota;Gammaproteobacteria;Enterobacterales;Erwiniaceae |
| Morganellaceae | 1 | 0 | 1 | 1 | 1 | 2 | 1 | 5 | 1 | 0 | 0 | 1 | 2 | 16 | Bacteria | Bacteria\_none | Pseudomonadota | Gammaproteobacteria | Enterobacterales | Bacteria;Bacteria\_none;Pseudomonadota;Gammaproteobacteria;Enterobacterales;Morganellaceae |
| Pectobacteriaceae | 0 | 0 | 0 | 0 | 1 | 0 | 0 | 0 | 0 | 0 | 0 | 0 | 0 | 1 | Bacteria | Bacteria\_none | Pseudomonadota | Gammaproteobacteria | Enterobacterales | Bacteria;Bacteria\_none;Pseudomonadota;Gammaproteobacteria;Enterobacterales;Pectobacteriaceae |
| Yersiniaceae | 0 | 0 | 0 | 0 | 0 | 0 | 0 | 0 | 0 | 0 | 1 | 0 | 0 | 1 | Bacteria | Bacteria\_none | Pseudomonadota | Gammaproteobacteria | Enterobacterales | Bacteria;Bacteria\_none;Pseudomonadota;Gammaproteobacteria;Enterobacterales;Yersiniaceae |
| Legionellaceae | 0 | 0 | 0 | 1 | 0 | 0 | 0 | 0 | 0 | 0 | 0 | 0 | 1 | 2 | Bacteria | Bacteria\_none | Pseudomonadota | Gammaproteobacteria | Legionellales | Bacteria;Bacteria\_none;Pseudomonadota;Gammaproteobacteria;Legionellales;Legionellaceae |
| Lysobacteraceae | 0 | 0 | 0 | 1 | 2 | 0 | 0 | 0 | 0 | 0 | 1 | 0 | 2 | 6 | Bacteria | Bacteria\_none | Pseudomonadota | Gammaproteobacteria | Lysobacterales | Bacteria;Bacteria\_none;Pseudomonadota;Gammaproteobacteria;Lysobacterales;Lysobacteraceae |
| Rhodanobacteraceae | 0 | 0 | 0 | 0 | 0 | 0 | 0 | 0 | 0 | 1 | 0 | 1 | 0 | 2 | Bacteria | Bacteria\_none | Pseudomonadota | Gammaproteobacteria | Lysobacterales | Bacteria;Bacteria\_none;Pseudomonadota;Gammaproteobacteria;Lysobacterales;Rhodanobacteraceae |
| Methylococcaceae | 0 | 0 | 0 | 0 | 0 | 1 | 0 | 0 | 0 | 0 | 0 | 0 | 4 | 5 | Bacteria | Bacteria\_none | Pseudomonadota | Gammaproteobacteria | Methylococcales | Bacteria;Bacteria\_none;Pseudomonadota;Gammaproteobacteria;Methylococcales;Methylococcaceae |
| Moraxellaceae | 0 | 0 | 1 | 2 | 0 | 2 | 0 | 4 | 0 | 2 | 0 | 2 | 5 | 18 | Bacteria | Bacteria\_none | Pseudomonadota | Gammaproteobacteria | Moraxellales | Bacteria;Bacteria\_none;Pseudomonadota;Gammaproteobacteria;Moraxellales;Moraxellaceae |
| Alcanivoracaceae | 0 | 0 | 0 | 0 | 0 | 0 | 0 | 1 | 0 | 0 | 0 | 0 | 0 | 1 | Bacteria | Bacteria\_none | Pseudomonadota | Gammaproteobacteria | Oceanospirillales | Bacteria;Bacteria\_none;Pseudomonadota;Gammaproteobacteria;Oceanospirillales;Alcanivoracaceae |
| Halomonadaceae | 0 | 0 | 0 | 0 | 1 | 1 | 0 | 1 | 0 | 0 | 0 | 0 | 0 | 3 | Bacteria | Bacteria\_none | Pseudomonadota | Gammaproteobacteria | Oceanospirillales | Bacteria;Bacteria\_none;Pseudomonadota;Gammaproteobacteria;Oceanospirillales;Halomonadaceae |
| Oceanospirillaceae | 0 | 1 | 0 | 0 | 0 | 1 | 0 | 2 | 0 | 0 | 0 | 0 | 1 | 5 | Bacteria | Bacteria\_none | Pseudomonadota | Gammaproteobacteria | Oceanospirillales | Bacteria;Bacteria\_none;Pseudomonadota;Gammaproteobacteria;Oceanospirillales;Oceanospirillaceae |
| Orbaceae | 0 | 0 | 0 | 0 | 0 | 0 | 0 | 1 | 0 | 0 | 0 | 0 | 1 | 2 | Bacteria | Bacteria\_none | Pseudomonadota | Gammaproteobacteria | Orbales | Bacteria;Bacteria\_none;Pseudomonadota;Gammaproteobacteria;Orbales;Orbaceae |
| Pasteurellaceae | 71 | 142 | 466 | 773 | 285 | 244 | 8 | 1922 | 0 | 92 | 49 | 244 | 1237 | 5533 | Bacteria | Bacteria\_none | Pseudomonadota | Gammaproteobacteria | Pasteurellales | Bacteria;Bacteria\_none;Pseudomonadota;Gammaproteobacteria;Pasteurellales;Pasteurellaceae |
| Pseudomonadaceae | 0 | 3 | 0 | 0 | 2 | 3 | 1 | 1 | 0 | 0 | 2 | 0 | 4 | 16 | Bacteria | Bacteria\_none | Pseudomonadota | Gammaproteobacteria | Pseudomonadales | Bacteria;Bacteria\_none;Pseudomonadota;Gammaproteobacteria;Pseudomonadales;Pseudomonadaceae |
| Piscirickettsiaceae | 0 | 0 | 0 | 0 | 1 | 0 | 0 | 0 | 0 | 0 | 0 | 0 | 0 | 1 | Bacteria | Bacteria\_none | Pseudomonadota | Gammaproteobacteria | Thiotrichales | Bacteria;Bacteria\_none;Pseudomonadota;Gammaproteobacteria;Thiotrichales;Piscirickettsiaceae |
| Vibrionaceae | 0 | 0 | 3 | 0 | 2 | 1 | 0 | 5 | 0 | 0 | 0 | 0 | 4 | 15 | Bacteria | Bacteria\_none | Pseudomonadota | Gammaproteobacteria | Vibrionales | Bacteria;Bacteria\_none;Pseudomonadota;Gammaproteobacteria;Vibrionales;Vibrionaceae |
| Leptospiraceae | 1 | 0 | 0 | 0 | 0 | 0 | 0 | 0 | 0 | 0 | 0 | 1 | 0 | 2 | Bacteria | Bacteria\_none | Spirochaetota | Spirochaetia | Leptospirales | Bacteria;Bacteria\_none;Spirochaetota;Spirochaetia;Leptospirales;Leptospiraceae |
| Borreliaceae | 0 | 0 | 0 | 0 | 0 | 1 | 0 | 0 | 0 | 0 | 0 | 0 | 0 | 1 | Bacteria | Bacteria\_none | Spirochaetota | Spirochaetia | Spirochaetales | Bacteria;Bacteria\_none;Spirochaetota;Spirochaetia;Spirochaetales;Borreliaceae |
| Breznakiellaceae | 0 | 1 | 0 | 0 | 0 | 0 | 0 | 0 | 0 | 0 | 0 | 0 | 0 | 1 | Bacteria | Bacteria\_none | Spirochaetota | Spirochaetia | Spirochaetales | Bacteria;Bacteria\_none;Spirochaetota;Spirochaetia;Spirochaetales;Breznakiellaceae |
| Sphaerochaetaceae | 0 | 1 | 0 | 0 | 0 | 0 | 0 | 0 | 0 | 0 | 0 | 0 | 0 | 1 | Bacteria | Bacteria\_none | Spirochaetota | Spirochaetia | Spirochaetales | Bacteria;Bacteria\_none;Spirochaetota;Spirochaetia;Spirochaetales;Sphaerochaetaceae |
| Treponemataceae | 3 | 72 | 54 | 9 | 40 | 73 | 9 | 112 | 0 | 14 | 56 | 119 | 143 | 704 | Bacteria | Bacteria\_none | Spirochaetota | Spirochaetia | Spirochaetales | Bacteria;Bacteria\_none;Spirochaetota;Spirochaetia;Spirochaetales;Treponemataceae |
| Aminobacteriaceae | 0 | 0 | 1 | 0 | 0 | 0 | 0 | 0 | 0 | 0 | 1 | 1 | 0 | 3 | Bacteria | Bacteria\_none | Synergistota | Synergistia | Synergistales | Bacteria;Bacteria\_none;Synergistota;Synergistia;Synergistales;Aminobacteriaceae |
| Candidatus Desulfofervidaceae | 0 | 0 | 0 | 0 | 0 | 1 | 0 | 0 | 0 | 0 | 0 | 0 | 0 | 1 | Bacteria | Bacteria\_none | Thermodesulfobacteriota | Candidatus Desulfofervidia | Candidatus Desulfofervidales | Bacteria;Bacteria\_none;Thermodesulfobacteriota;Candidatus Desulfofervidia;Candidatus Desulfofervidales;Candidatus Desulfofervidaceae |
| Desulfarculaceae | 0 | 0 | 0 | 0 | 0 | 1 | 0 | 0 | 0 | 0 | 0 | 0 | 0 | 1 | Bacteria | Bacteria\_none | Thermodesulfobacteriota | Desulfarculia | Desulfarculales | Bacteria;Bacteria\_none;Thermodesulfobacteriota;Desulfarculia;Desulfarculales;Desulfarculaceae |
| Desulfobulbaceae | 0 | 2 | 1 | 0 | 0 | 0 | 0 | 5 | 0 | 0 | 0 | 0 | 1 | 9 | Bacteria | Bacteria\_none | Thermodesulfobacteriota | Desulfobulbia | Desulfobulbales | Bacteria;Bacteria\_none;Thermodesulfobacteriota;Desulfobulbia;Desulfobulbales;Desulfobulbaceae |
| Desulfovibrionaceae | 0 | 0 | 0 | 0 | 0 | 0 | 0 | 0 | 0 | 0 | 0 | 1 | 0 | 1 | Bacteria | Bacteria\_none | Thermodesulfobacteriota | Desulfovibrionia | Desulfovibrionales | Bacteria;Bacteria\_none;Thermodesulfobacteriota;Desulfovibrionia;Desulfovibrionales;Desulfovibrionaceae |
| Syntrophotaleaceae | 0 | 0 | 0 | 0 | 0 | 0 | 0 | 0 | 0 | 0 | 1 | 0 | 0 | 1 | Bacteria | Bacteria\_none | Thermodesulfobacteriota | Desulfuromonadia | Desulfuromonadales | Bacteria;Bacteria\_none;Thermodesulfobacteriota;Desulfuromonadia;Desulfuromonadales;Syntrophotaleaceae |
| Geobacteraceae | 0 | 0 | 1 | 0 | 0 | 0 | 0 | 0 | 0 | 0 | 0 | 0 | 0 | 1 | Bacteria | Bacteria\_none | Thermodesulfobacteriota | Desulfuromonadia | Geobacterales | Bacteria;Bacteria\_none;Thermodesulfobacteriota;Desulfuromonadia;Geobacterales;Geobacteraceae |
| Thermomicrobiaceae | 0 | 1 | 0 | 0 | 0 | 0 | 0 | 0 | 0 | 0 | 0 | 0 | 0 | 1 | Bacteria | Bacteria\_none | Thermomicrobiota | Thermomicrobia | Thermomicrobiales | Bacteria;Bacteria\_none;Thermomicrobiota;Thermomicrobia;Thermomicrobiales;Thermomicrobiaceae |
| Fervidobacteriaceae | 1 | 0 | 0 | 0 | 0 | 0 | 0 | 0 | 0 | 0 | 0 | 0 | 0 | 1 | Bacteria | Bacteria\_none | Thermotogota | Thermotogae | Thermotogales | Bacteria;Bacteria\_none;Thermotogota;Thermotogae;Thermotogales;Fervidobacteriaceae |
| Opitutaceae | 0 | 1 | 0 | 0 | 0 | 0 | 0 | 0 | 0 | 0 | 0 | 0 | 0 | 1 | Bacteria | Bacteria\_none | Verrucomicrobiota | Opitutia | Opitutales | Bacteria;Bacteria\_none;Verrucomicrobiota;Opitutia;Opitutales;Opitutaceae |
| Hominidae | 428 | 938 | 1105 | 97 | 2901 | 211 | 12043 | 17730 | 14114 | 5069 | 5655 | 9919 | 34174 | 104384 | Eukaryota | Metazoa | Chordata | Mammalia | Primates | Eukaryota;Metazoa;Chordata;Mammalia;Primates;Hominidae |
| Unknown | 1409 | 1058 | 1350 | 2756 | 1867 | 2605 | 1040 | 1418 | 519 | 1446 | 1552 | 1325 | 24901 | 43246 | Unclassified | Unknown | Unknown | Unknown | Unknown | Unclassified;Unknown;Unknown;Unknown;Unknown;Unknown |
| Mimiviridae | 0 | 0 | 0 | 0 | 0 | 0 | 0 | 1 | 0 | 0 | 0 | 1 | 0 | 2 | Viruses | Bamfordvirae | Nucleocytoviricota | Megaviricetes | Imitervirales | Viruses;Bamfordvirae;Nucleocytoviricota;Megaviricetes;Imitervirales;Mimiviridae |
| Schizomimiviridae | 0 | 0 | 0 | 0 | 0 | 0 | 0 | 0 | 0 | 0 | 0 | 0 | 1 | 1 | Viruses | Bamfordvirae | Nucleocytoviricota | Megaviricetes | Imitervirales | Viruses;Bamfordvirae;Nucleocytoviricota;Megaviricetes;Imitervirales;Schizomimiviridae |
| Orthoherpesviridae | 8 | 0 | 0 | 0 | 0 | 1 | 0 | 1 | 0 | 0 | 0 | 0 | 2 | 12 | Viruses | Heunggongvirae | Peploviricota | Herviviricetes | Herpesvirales | Viruses;Heunggongvirae;Peploviricota;Herviviricetes;Herpesvirales;Orthoherpesviridae |
| Caudoviricetes\_Incertae\_sedis | 5 | 0 | 0 | 1 | 3 | 1 | 5 | 0 | 0 | 0 | 2 | 1 | 2 | 20 | Viruses | Heunggongvirae | Uroviricota | Caudoviricetes | Caudoviricetes\_Incertae\_sedis | Viruses;Heunggongvirae;Uroviricota;Caudoviricetes;Caudoviricetes\_Incertae\_sedis;Caudoviricetes\_Incertae\_sedis |
| Intestiviridae | 2 | 0 | 0 | 0 | 0 | 0 | 0 | 0 | 0 | 0 | 0 | 0 | 0 | 2 | Viruses | Heunggongvirae | Uroviricota | Caudoviricetes | Crassvirales | Viruses;Heunggongvirae;Uroviricota;Caudoviricetes;Crassvirales;Intestiviridae |
| Rhabdoviridae | 0 | 0 | 0 | 0 | 0 | 0 | 0 | 0 | 0 | 0 | 0 | 0 | 1 | 1 | Viruses | Orthornavirae | Negarnaviricota | Monjiviricetes | Mononegavirales | Viruses;Orthornavirae;Negarnaviricota;Monjiviricetes;Mononegavirales;Rhabdoviridae |
| Papillomaviridae | 0 | 0 | 0 | 0 | 0 | 0 | 0 | 2 | 0 | 0 | 0 | 0 | 0 | 2 | Viruses | Shotokuvirae | Cossaviricota | Papovaviricetes | Zurhausenvirales | Viruses;Shotokuvirae;Cossaviricota;Papovaviricetes;Zurhausenvirales;Papillomaviridae |
| Lipothrixviridae | 0 | 0 | 0 | 0 | 0 | 0 | 0 | 0 | 0 | 0 | 0 | 0 | 1 | 1 | Viruses | Zilligvirae | Taleaviricota | Tokiviricetes | Ligamenvirales | Viruses;Zilligvirae;Taleaviricota;Tokiviricetes;Ligamenvirales;Lipothrixviridae |

Export CSV

Abundance table for the genus rank.

Only taxa whose global abundance are above the
`abundance_threshold` parameter will appear in the table.

| genus | barcode01 | barcode02 | barcode03 | barcode04 | barcode05 | barcode06 | barcode07 | barcode08 | barcode09 | barcode10 | barcode11 | barcode12 | unclassified | total | superkingdom | kingdom | phylum | class | order | family | tax |
| --- | --- | --- | --- | --- | --- | --- | --- | --- | --- | --- | --- | --- | --- | --- | --- | --- | --- | --- | --- | --- | --- |
| Haladaptatus | 0 | 1 | 0 | 0 | 0 | 0 | 0 | 0 | 0 | 0 | 0 | 0 | 0 | 1 | Archaea | Archaea\_none | Euryarchaeota | Halobacteria | Halobacteriales | Haladaptataceae | Archaea;Archaea\_none;Euryarchaeota;Halobacteria;Halobacteriales;Haladaptataceae;Haladaptatus |
| Halococcoides | 0 | 0 | 0 | 0 | 0 | 0 | 0 | 0 | 0 | 0 | 1 | 0 | 0 | 1 | Archaea | Archaea\_none | Euryarchaeota | Halobacteria | Halobacteriales | Haloarculaceae | Archaea;Archaea\_none;Euryarchaeota;Halobacteria;Halobacteriales;Haloarculaceae;Halococcoides |
| Halospeciosus | 1 | 0 | 0 | 0 | 0 | 0 | 0 | 0 | 0 | 0 | 0 | 0 | 0 | 1 | Archaea | Archaea\_none | Euryarchaeota | Halobacteria | Halobacteriales | Halobacteriaceae | Archaea;Archaea\_none;Euryarchaeota;Halobacteria;Halobacteriales;Halobacteriaceae;Halospeciosus |
| Halobaculum | 0 | 0 | 0 | 0 | 0 | 0 | 1 | 0 | 0 | 0 | 0 | 0 | 0 | 1 | Archaea | Archaea\_none | Euryarchaeota | Halobacteria | Halobacteriales | Haloferacaceae | Archaea;Archaea\_none;Euryarchaeota;Halobacteria;Halobacteriales;Haloferacaceae;Halobaculum |
| Halogeometricum | 0 | 0 | 0 | 0 | 0 | 1 | 0 | 0 | 0 | 0 | 0 | 0 | 0 | 1 | Archaea | Archaea\_none | Euryarchaeota | Halobacteria | Halobacteriales | Haloferacaceae | Archaea;Archaea\_none;Euryarchaeota;Halobacteria;Halobacteriales;Haloferacaceae;Halogeometricum |
| Halorarum | 0 | 0 | 0 | 0 | 0 | 0 | 0 | 0 | 0 | 1 | 0 | 0 | 0 | 1 | Archaea | Archaea\_none | Euryarchaeota | Halobacteria | Halobacteriales | Haloferacaceae | Archaea;Archaea\_none;Euryarchaeota;Halobacteria;Halobacteriales;Haloferacaceae;Halorarum |
| Halorubrum | 0 | 0 | 0 | 0 | 0 | 0 | 0 | 0 | 0 | 0 | 0 | 0 | 1 | 1 | Archaea | Archaea\_none | Euryarchaeota | Halobacteria | Halobacteriales | Haloferacaceae | Archaea;Archaea\_none;Euryarchaeota;Halobacteria;Halobacteriales;Haloferacaceae;Halorubrum |
| Methanobacterium | 0 | 0 | 0 | 0 | 0 | 0 | 1 | 0 | 0 | 0 | 0 | 0 | 0 | 1 | Archaea | Archaea\_none | Euryarchaeota | Methanobacteria | Methanobacteriales | Methanobacteriaceae | Archaea;Archaea\_none;Euryarchaeota;Methanobacteria;Methanobacteriales;Methanobacteriaceae;Methanobacterium |
| Methanobrevibacter | 0 | 0 | 1 | 0 | 0 | 1 | 0 | 0 | 0 | 0 | 0 | 0 | 1 | 3 | Archaea | Archaea\_none | Euryarchaeota | Methanobacteria | Methanobacteriales | Methanobacteriaceae | Archaea;Archaea\_none;Euryarchaeota;Methanobacteria;Methanobacteriales;Methanobacteriaceae;Methanobrevibacter |
| Methanosphaera | 0 | 0 | 1 | 0 | 0 | 0 | 0 | 0 | 0 | 0 | 0 | 0 | 0 | 1 | Archaea | Archaea\_none | Euryarchaeota | Methanobacteria | Methanobacteriales | Methanobacteriaceae | Archaea;Archaea\_none;Euryarchaeota;Methanobacteria;Methanobacteriales;Methanobacteriaceae;Methanosphaera |
| Methanocaldococcus | 0 | 0 | 1 | 0 | 0 | 0 | 0 | 0 | 0 | 0 | 0 | 0 | 1 | 2 | Archaea | Archaea\_none | Euryarchaeota | Methanococci | Methanococcales | Methanocaldococcaceae | Archaea;Archaea\_none;Euryarchaeota;Methanococci;Methanococcales;Methanocaldococcaceae;Methanocaldococcus |
| Methanoregula | 0 | 1 | 0 | 0 | 0 | 0 | 0 | 0 | 0 | 0 | 0 | 0 | 1 | 2 | Archaea | Archaea\_none | Euryarchaeota | Methanomicrobia | Methanomicrobiales | Methanoregulaceae | Archaea;Archaea\_none;Euryarchaeota;Methanomicrobia;Methanomicrobiales;Methanoregulaceae;Methanoregula |
| Methanimicrococcus | 0 | 0 | 0 | 1 | 0 | 0 | 0 | 0 | 0 | 0 | 0 | 0 | 0 | 1 | Archaea | Archaea\_none | Euryarchaeota | Methanomicrobia | Methanosarcinales | Methanosarcinaceae | Archaea;Archaea\_none;Euryarchaeota;Methanomicrobia;Methanosarcinales;Methanosarcinaceae;Methanimicrococcus |
| Methanolapillus | 0 | 0 | 0 | 0 | 0 | 1 | 0 | 0 | 0 | 0 | 0 | 0 | 0 | 1 | Archaea | Archaea\_none | Euryarchaeota | Methanomicrobia | Methanosarcinales | Methanosarcinaceae | Archaea;Archaea\_none;Euryarchaeota;Methanomicrobia;Methanosarcinales;Methanosarcinaceae;Methanolapillus |
| Methanolobus | 0 | 0 | 0 | 0 | 1 | 1 | 0 | 0 | 0 | 0 | 0 | 0 | 0 | 2 | Archaea | Archaea\_none | Euryarchaeota | Methanomicrobia | Methanosarcinales | Methanosarcinaceae | Archaea;Archaea\_none;Euryarchaeota;Methanomicrobia;Methanosarcinales;Methanosarcinaceae;Methanolobus |
| Methanomethylovorans | 0 | 0 | 0 | 0 | 0 | 0 | 0 | 0 | 0 | 0 | 0 | 0 | 1 | 1 | Archaea | Archaea\_none | Euryarchaeota | Methanomicrobia | Methanosarcinales | Methanosarcinaceae | Archaea;Archaea\_none;Euryarchaeota;Methanomicrobia;Methanosarcinales;Methanosarcinaceae;Methanomethylovorans |
| Methanosarcina | 0 | 0 | 0 | 0 | 0 | 1 | 0 | 0 | 0 | 0 | 1 | 0 | 0 | 2 | Archaea | Archaea\_none | Euryarchaeota | Methanomicrobia | Methanosarcinales | Methanosarcinaceae | Archaea;Archaea\_none;Euryarchaeota;Methanomicrobia;Methanosarcinales;Methanosarcinaceae;Methanosarcina |
| Thermococcus | 0 | 0 | 0 | 0 | 0 | 0 | 0 | 0 | 0 | 0 | 0 | 1 | 0 | 1 | Archaea | Archaea\_none | Euryarchaeota | Thermococci | Thermococcales | Thermococcaceae | Archaea;Archaea\_none;Euryarchaeota;Thermococci;Thermococcales;Thermococcaceae;Thermococcus |
| Candidatus Nitrosotenuis | 0 | 0 | 0 | 0 | 0 | 1 | 0 | 0 | 0 | 0 | 0 | 0 | 0 | 1 | Archaea | Archaea\_none | Nitrososphaerota | Nitrososphaerota\_Incertae\_sedis | Nitrososphaerota\_Incertae\_sedis | Nitrososphaerota\_Incertae\_sedis | Archaea;Archaea\_none;Nitrososphaerota;Nitrososphaerota\_Incertae\_sedis;Nitrososphaerota\_Incertae\_sedis;Nitrososphaerota\_Incertae\_sedis;Candidatus Nitrosotenuis |
| Paludibaculum | 0 | 0 | 0 | 0 | 0 | 0 | 0 | 0 | 0 | 0 | 0 | 1 | 0 | 1 | Bacteria | Bacteria\_none | Acidobacteriota | Terriglobia | Bryobacterales | Bryobacteraceae | Bacteria;Bacteria\_none;Acidobacteriota;Terriglobia;Bryobacterales;Bryobacteraceae;Paludibaculum |
| Aquihabitans | 0 | 0 | 0 | 0 | 0 | 0 | 0 | 1 | 0 | 0 | 0 | 0 | 0 | 1 | Bacteria | Bacteria\_none | Actinomycetota | Acidimicrobiia | Acidimicrobiales | Iamiaceae | Bacteria;Bacteria\_none;Actinomycetota;Acidimicrobiia;Acidimicrobiales;Iamiaceae;Aquihabitans |
| Actinomyces | 1 | 1 | 0 | 0 | 2 | 19 | 12 | 4 | 6 | 0 | 1 | 6 | 8 | 60 | Bacteria | Bacteria\_none | Actinomycetota | Actinomycetes | Actinomycetales | Actinomycetaceae | Bacteria;Bacteria\_none;Actinomycetota;Actinomycetes;Actinomycetales;Actinomycetaceae;Actinomyces |
| Schaalia | 0 | 16 | 1 | 3 | 1 | 21 | 26 | 0 | 0 | 0 | 0 | 6 | 11 | 85 | Bacteria | Bacteria\_none | Actinomycetota | Actinomycetes | Actinomycetales | Actinomycetaceae | Bacteria;Bacteria\_none;Actinomycetota;Actinomycetes;Actinomycetales;Actinomycetaceae;Schaalia |
| Bifidobacterium | 0 | 0 | 1 | 0 | 0 | 1 | 5 | 2 | 0 | 0 | 0 | 0 | 1 | 10 | Bacteria | Bacteria\_none | Actinomycetota | Actinomycetes | Bifidobacteriales | Bifidobacteriaceae | Bacteria;Bacteria\_none;Actinomycetota;Actinomycetes;Bifidobacteriales;Bifidobacteriaceae;Bifidobacterium |
| Gardnerella | 0 | 1 | 1 | 0 | 0 | 0 | 0 | 2 | 0 | 0 | 0 | 0 | 1 | 5 | Bacteria | Bacteria\_none | Actinomycetota | Actinomycetes | Bifidobacteriales | Bifidobacteriaceae | Bacteria;Bacteria\_none;Actinomycetota;Actinomycetes;Bifidobacteriales;Bifidobacteriaceae;Gardnerella |
| Parascardovia | 0 | 0 | 0 | 0 | 1 | 0 | 2 | 0 | 0 | 0 | 0 | 0 | 0 | 3 | Bacteria | Bacteria\_none | Actinomycetota | Actinomycetes | Bifidobacteriales | Bifidobacteriaceae | Bacteria;Bacteria\_none;Actinomycetota;Actinomycetes;Bifidobacteriales;Bifidobacteriaceae;Parascardovia |
| Geodermatophilus | 0 | 0 | 0 | 0 | 1 | 0 | 0 | 0 | 0 | 0 | 0 | 0 | 0 | 1 | Bacteria | Bacteria\_none | Actinomycetota | Actinomycetes | Geodermatophilales | Geodermatophilaceae | Bacteria;Bacteria\_none;Actinomycetota;Actinomycetes;Geodermatophilales;Geodermatophilaceae;Geodermatophilus |
| Streptomyces | 0 | 2 | 0 | 2 | 2 | 9 | 4 | 1 | 0 | 2 | 1 | 2 | 5 | 30 | Bacteria | Bacteria\_none | Actinomycetota | Actinomycetes | Kitasatosporales | Streptomycetaceae | Bacteria;Bacteria\_none;Actinomycetota;Actinomycetes;Kitasatosporales;Streptomycetaceae;Streptomyces |
| Cellulomonas | 0 | 0 | 0 | 0 | 0 | 0 | 0 | 0 | 0 | 0 | 0 | 0 | 1 | 1 | Bacteria | Bacteria\_none | Actinomycetota | Actinomycetes | Micrococcales | Cellulomonadaceae | Bacteria;Bacteria\_none;Actinomycetota;Actinomycetes;Micrococcales;Cellulomonadaceae;Cellulomonas |
| Gephyromycinifex | 0 | 0 | 0 | 0 | 0 | 0 | 0 | 0 | 0 | 0 | 0 | 0 | 1 | 1 | Bacteria | Bacteria\_none | Actinomycetota | Actinomycetes | Micrococcales | Dermatophilaceae | Bacteria;Bacteria\_none;Actinomycetota;Actinomycetes;Micrococcales;Dermatophilaceae;Gephyromycinifex |
| Agromyces | 1 | 0 | 0 | 0 | 0 | 0 | 0 | 0 | 0 | 0 | 0 | 0 | 0 | 1 | Bacteria | Bacteria\_none | Actinomycetota | Actinomycetes | Micrococcales | Microbacteriaceae | Bacteria;Bacteria\_none;Actinomycetota;Actinomycetes;Micrococcales;Microbacteriaceae;Agromyces |
| Curtobacterium | 0 | 0 | 0 | 1 | 1 | 0 | 0 | 0 | 0 | 0 | 0 | 0 | 0 | 2 | Bacteria | Bacteria\_none | Actinomycetota | Actinomycetes | Micrococcales | Microbacteriaceae | Bacteria;Bacteria\_none;Actinomycetota;Actinomycetes;Micrococcales;Microbacteriaceae;Curtobacterium |
| Diaminobutyricibacter | 0 | 0 | 0 | 1 | 0 | 0 | 0 | 0 | 0 | 0 | 0 | 0 | 0 | 1 | Bacteria | Bacteria\_none | Actinomycetota | Actinomycetes | Micrococcales | Microbacteriaceae | Bacteria;Bacteria\_none;Actinomycetota;Actinomycetes;Micrococcales;Microbacteriaceae;Diaminobutyricibacter |
| Leucobacter | 0 | 0 | 0 | 0 | 0 | 0 | 1 | 0 | 0 | 0 | 0 | 0 | 0 | 1 | Bacteria | Bacteria\_none | Actinomycetota | Actinomycetes | Micrococcales | Microbacteriaceae | Bacteria;Bacteria\_none;Actinomycetota;Actinomycetes;Micrococcales;Microbacteriaceae;Leucobacter |
| Microbacterium | 0 | 0 | 0 | 0 | 1 | 0 | 0 | 1 | 0 | 0 | 0 | 0 | 1 | 3 | Bacteria | Bacteria\_none | Actinomycetota | Actinomycetes | Micrococcales | Microbacteriaceae | Bacteria;Bacteria\_none;Actinomycetota;Actinomycetes;Micrococcales;Microbacteriaceae;Microbacterium |
| Microcella | 0 | 0 | 0 | 0 | 0 | 0 | 0 | 0 | 1 | 0 | 0 | 0 | 0 | 1 | Bacteria | Bacteria\_none | Actinomycetota | Actinomycetes | Micrococcales | Microbacteriaceae | Bacteria;Bacteria\_none;Actinomycetota;Actinomycetes;Micrococcales;Microbacteriaceae;Microcella |
| Arthrobacter | 0 | 0 | 0 | 0 | 0 | 1 | 0 | 0 | 0 | 2 | 1 | 0 | 1 | 5 | Bacteria | Bacteria\_none | Actinomycetota | Actinomycetes | Micrococcales | Micrococcaceae | Bacteria;Bacteria\_none;Actinomycetota;Actinomycetes;Micrococcales;Micrococcaceae;Arthrobacter |
| Pseudarthrobacter | 0 | 0 | 0 | 0 | 1 | 0 | 0 | 0 | 0 | 0 | 0 | 0 | 0 | 1 | Bacteria | Bacteria\_none | Actinomycetota | Actinomycetes | Micrococcales | Micrococcaceae | Bacteria;Bacteria\_none;Actinomycetota;Actinomycetes;Micrococcales;Micrococcaceae;Pseudarthrobacter |
| Rothia | 27 | 111 | 23 | 17 | 124 | 41 | 36 | 61 | 14 | 78 | 156 | 167 | 212 | 1067 | Bacteria | Bacteria\_none | Actinomycetota | Actinomycetes | Micrococcales | Micrococcaceae | Bacteria;Bacteria\_none;Actinomycetota;Actinomycetes;Micrococcales;Micrococcaceae;Rothia |
| Luteimicrobium | 0 | 0 | 0 | 0 | 0 | 0 | 0 | 0 | 0 | 0 | 0 | 0 | 1 | 1 | Bacteria | Bacteria\_none | Actinomycetota | Actinomycetes | Micrococcales | Micrococcales\_Incertae\_sedis | Bacteria;Bacteria\_none;Actinomycetota;Actinomycetes;Micrococcales;Micrococcales\_Incertae\_sedis;Luteimicrobium |
| Ruania | 0 | 0 | 0 | 0 | 0 | 0 | 1 | 0 | 0 | 0 | 0 | 0 | 0 | 1 | Bacteria | Bacteria\_none | Actinomycetota | Actinomycetes | Micrococcales | Ruaniaceae | Bacteria;Bacteria\_none;Actinomycetota;Actinomycetes;Micrococcales;Ruaniaceae;Ruania |
| Micromonospora | 1 | 0 | 0 | 1 | 0 | 1 | 0 | 1 | 0 | 2 | 0 | 0 | 1 | 7 | Bacteria | Bacteria\_none | Actinomycetota | Actinomycetes | Micromonosporales | Micromonosporaceae | Bacteria;Bacteria\_none;Actinomycetota;Actinomycetes;Micromonosporales;Micromonosporaceae;Micromonospora |
| Salinispora | 0 | 0 | 0 | 0 | 0 | 0 | 0 | 0 | 0 | 1 | 0 | 0 | 0 | 1 | Bacteria | Bacteria\_none | Actinomycetota | Actinomycetes | Micromonosporales | Micromonosporaceae | Bacteria;Bacteria\_none;Actinomycetota;Actinomycetes;Micromonosporales;Micromonosporaceae;Salinispora |
| Corynebacterium | 0 | 6 | 0 | 0 | 1 | 3 | 0 | 0 | 0 | 15 | 0 | 7 | 9 | 41 | Bacteria | Bacteria\_none | Actinomycetota | Actinomycetes | Mycobacteriales | Corynebacteriaceae | Bacteria;Bacteria\_none;Actinomycetota;Actinomycetes;Mycobacteriales;Corynebacteriaceae;Corynebacterium |
| Gordonia | 0 | 0 | 0 | 0 | 2 | 0 | 0 | 0 | 0 | 0 | 0 | 0 | 0 | 2 | Bacteria | Bacteria\_none | Actinomycetota | Actinomycetes | Mycobacteriales | Gordoniaceae | Bacteria;Bacteria\_none;Actinomycetota;Actinomycetes;Mycobacteriales;Gordoniaceae;Gordonia |
| Mycobacterium | 0 | 0 | 1 | 1 | 0 | 0 | 0 | 0 | 0 | 0 | 0 | 0 | 0 | 2 | Bacteria | Bacteria\_none | Actinomycetota | Actinomycetes | Mycobacteriales | Mycobacteriaceae | Bacteria;Bacteria\_none;Actinomycetota;Actinomycetes;Mycobacteriales;Mycobacteriaceae;Mycobacterium |
| Mycolicibacterium | 0 | 1 | 1 | 0 | 0 | 2 | 2 | 0 | 0 | 0 | 0 | 0 | 1 | 7 | Bacteria | Bacteria\_none | Actinomycetota | Actinomycetes | Mycobacteriales | Mycobacteriaceae | Bacteria;Bacteria\_none;Actinomycetota;Actinomycetes;Mycobacteriales;Mycobacteriaceae;Mycolicibacterium |
| Nocardia | 0 | 0 | 0 | 0 | 0 | 0 | 0 | 0 | 0 | 0 | 0 | 0 | 1 | 1 | Bacteria | Bacteria\_none | Actinomycetota | Actinomycetes | Mycobacteriales | Nocardiaceae | Bacteria;Bacteria\_none;Actinomycetota;Actinomycetes;Mycobacteriales;Nocardiaceae;Nocardia |
| Rhodococcus | 0 | 0 | 0 | 0 | 0 | 0 | 0 | 0 | 0 | 0 | 1 | 0 | 0 | 1 | Bacteria | Bacteria\_none | Actinomycetota | Actinomycetes | Mycobacteriales | Nocardiaceae | Bacteria;Bacteria\_none;Actinomycetota;Actinomycetes;Mycobacteriales;Nocardiaceae;Rhodococcus |
| Kribbella | 0 | 0 | 0 | 0 | 0 | 0 | 0 | 0 | 0 | 0 | 1 | 0 | 0 | 1 | Bacteria | Bacteria\_none | Actinomycetota | Actinomycetes | Propionibacteriales | Kribbellaceae | Bacteria;Bacteria\_none;Actinomycetota;Actinomycetes;Propionibacteriales;Kribbellaceae;Kribbella |
| Aeromicrobium | 0 | 0 | 0 | 1 | 0 | 0 | 0 | 0 | 0 | 0 | 0 | 0 | 0 | 1 | Bacteria | Bacteria\_none | Actinomycetota | Actinomycetes | Propionibacteriales | Nocardioidaceae | Bacteria;Bacteria\_none;Actinomycetota;Actinomycetes;Propionibacteriales;Nocardioidaceae;Aeromicrobium |
| Nocardioides | 0 | 0 | 1 | 1 | 1 | 0 | 0 | 0 | 0 | 0 | 0 | 0 | 0 | 3 | Bacteria | Bacteria\_none | Actinomycetota | Actinomycetes | Propionibacteriales | Nocardioidaceae | Bacteria;Bacteria\_none;Actinomycetota;Actinomycetes;Propionibacteriales;Nocardioidaceae;Nocardioides |
| Acidipropionibacterium | 0 | 0 | 0 | 1 | 0 | 0 | 0 | 0 | 0 | 0 | 0 | 0 | 0 | 1 | Bacteria | Bacteria\_none | Actinomycetota | Actinomycetes | Propionibacteriales | Propionibacteriaceae | Bacteria;Bacteria\_none;Actinomycetota;Actinomycetes;Propionibacteriales;Propionibacteriaceae;Acidipropionibacterium |
| Arachnia | 0 | 1 | 0 | 0 | 0 | 3 | 0 | 1 | 0 | 0 | 44 | 0 | 9 | 58 | Bacteria | Bacteria\_none | Actinomycetota | Actinomycetes | Propionibacteriales | Propionibacteriaceae | Bacteria;Bacteria\_none;Actinomycetota;Actinomycetes;Propionibacteriales;Propionibacteriaceae;Arachnia |
| Cutibacterium | 0 | 0 | 0 | 0 | 0 | 0 | 0 | 0 | 0 | 0 | 0 | 0 | 1 | 1 | Bacteria | Bacteria\_none | Actinomycetota | Actinomycetes | Propionibacteriales | Propionibacteriaceae | Bacteria;Bacteria\_none;Actinomycetota;Actinomycetes;Propionibacteriales;Propionibacteriaceae;Cutibacterium |
| Propionibacterium | 0 | 0 | 0 | 0 | 0 | 0 | 0 | 0 | 0 | 1 | 0 | 0 | 0 | 1 | Bacteria | Bacteria\_none | Actinomycetota | Actinomycetes | Propionibacteriales | Propionibacteriaceae | Bacteria;Bacteria\_none;Actinomycetota;Actinomycetes;Propionibacteriales;Propionibacteriaceae;Propionibacterium |
| Vaginimicrobium | 0 | 0 | 0 | 0 | 1 | 0 | 0 | 0 | 0 | 0 | 0 | 0 | 0 | 1 | Bacteria | Bacteria\_none | Actinomycetota | Actinomycetes | Propionibacteriales | Propionibacteriaceae | Bacteria;Bacteria\_none;Actinomycetota;Actinomycetes;Propionibacteriales;Propionibacteriaceae;Vaginimicrobium |
| Amycolatopsis | 0 | 0 | 0 | 1 | 0 | 0 | 0 | 0 | 0 | 0 | 0 | 0 | 0 | 1 | Bacteria | Bacteria\_none | Actinomycetota | Actinomycetes | Pseudonocardiales | Pseudonocardiaceae | Bacteria;Bacteria\_none;Actinomycetota;Actinomycetes;Pseudonocardiales;Pseudonocardiaceae;Amycolatopsis |
| Crossiella | 0 | 0 | 0 | 0 | 1 | 0 | 0 | 0 | 0 | 0 | 0 | 0 | 0 | 1 | Bacteria | Bacteria\_none | Actinomycetota | Actinomycetes | Pseudonocardiales | Pseudonocardiaceae | Bacteria;Bacteria\_none;Actinomycetota;Actinomycetes;Pseudonocardiales;Pseudonocardiaceae;Crossiella |
| Saccharopolyspora | 0 | 0 | 0 | 0 | 0 | 1 | 0 | 0 | 0 | 0 | 0 | 0 | 0 | 1 | Bacteria | Bacteria\_none | Actinomycetota | Actinomycetes | Pseudonocardiales | Pseudonocardiaceae | Bacteria;Bacteria\_none;Actinomycetota;Actinomycetes;Pseudonocardiales;Pseudonocardiaceae;Saccharopolyspora |
| Nonomuraea | 0 | 0 | 0 | 0 | 0 | 1 | 1 | 0 | 0 | 0 | 0 | 0 | 0 | 2 | Bacteria | Bacteria\_none | Actinomycetota | Actinomycetes | Streptosporangiales | Streptosporangiaceae | Bacteria;Bacteria\_none;Actinomycetota;Actinomycetes;Streptosporangiales;Streptosporangiaceae;Nonomuraea |
| Streptosporangium | 0 | 0 | 1 | 0 | 1 | 0 | 0 | 0 | 0 | 0 | 0 | 0 | 0 | 2 | Bacteria | Bacteria\_none | Actinomycetota | Actinomycetes | Streptosporangiales | Streptosporangiaceae | Bacteria;Bacteria\_none;Actinomycetota;Actinomycetes;Streptosporangiales;Streptosporangiaceae;Streptosporangium |
| Actinomadura | 0 | 0 | 0 | 1 | 0 | 0 | 0 | 0 | 0 | 0 | 0 | 0 | 0 | 1 | Bacteria | Bacteria\_none | Actinomycetota | Actinomycetes | Streptosporangiales | Thermomonosporaceae | Bacteria;Bacteria\_none;Actinomycetota;Actinomycetes;Streptosporangiales;Thermomonosporaceae;Actinomadura |
| Thermomonospora | 0 | 0 | 0 | 0 | 0 | 0 | 1 | 0 | 0 | 0 | 0 | 0 | 0 | 1 | Bacteria | Bacteria\_none | Actinomycetota | Actinomycetes | Streptosporangiales | Thermomonosporaceae | Bacteria;Bacteria\_none;Actinomycetota;Actinomycetes;Streptosporangiales;Thermomonosporaceae;Thermomonospora |
| Atopobium | 0 | 0 | 0 | 0 | 0 | 0 | 0 | 0 | 0 | 0 | 1 | 0 | 0 | 1 | Bacteria | Bacteria\_none | Actinomycetota | Coriobacteriia | Coriobacteriales | Atopobiaceae | Bacteria;Bacteria\_none;Actinomycetota;Coriobacteriia;Coriobacteriales;Atopobiaceae;Atopobium |
| Fannyhessea | 0 | 0 | 0 | 0 | 0 | 0 | 1 | 0 | 0 | 0 | 0 | 0 | 0 | 1 | Bacteria | Bacteria\_none | Actinomycetota | Coriobacteriia | Coriobacteriales | Atopobiaceae | Bacteria;Bacteria\_none;Actinomycetota;Coriobacteriia;Coriobacteriales;Atopobiaceae;Fannyhessea |
| Lancefieldella | 28 | 5 | 10 | 0 | 2 | 132 | 60 | 7 | 1 | 0 | 6 | 4 | 35 | 290 | Bacteria | Bacteria\_none | Actinomycetota | Coriobacteriia | Coriobacteriales | Atopobiaceae | Bacteria;Bacteria\_none;Actinomycetota;Coriobacteriia;Coriobacteriales;Atopobiaceae;Lancefieldella |
| Olsenella | 0 | 0 | 0 | 0 | 0 | 1 | 0 | 0 | 0 | 0 | 0 | 0 | 0 | 1 | Bacteria | Bacteria\_none | Actinomycetota | Coriobacteriia | Coriobacteriales | Atopobiaceae | Bacteria;Bacteria\_none;Actinomycetota;Coriobacteriia;Coriobacteriales;Atopobiaceae;Olsenella |
| Thermophilibacter | 0 | 0 | 0 | 0 | 0 | 0 | 1 | 0 | 0 | 0 | 0 | 0 | 0 | 1 | Bacteria | Bacteria\_none | Actinomycetota | Coriobacteriia | Coriobacteriales | Atopobiaceae | Bacteria;Bacteria\_none;Actinomycetota;Coriobacteriia;Coriobacteriales;Atopobiaceae;Thermophilibacter |
| Cryptobacterium | 0 | 0 | 0 | 0 | 0 | 1 | 0 | 0 | 0 | 0 | 0 | 0 | 0 | 1 | Bacteria | Bacteria\_none | Actinomycetota | Coriobacteriia | Eggerthellales | Eggerthellaceae | Bacteria;Bacteria\_none;Actinomycetota;Coriobacteriia;Eggerthellales;Eggerthellaceae;Cryptobacterium |
| Denitrobacterium | 0 | 0 | 0 | 0 | 0 | 0 | 1 | 0 | 0 | 0 | 0 | 0 | 0 | 1 | Bacteria | Bacteria\_none | Actinomycetota | Coriobacteriia | Eggerthellales | Eggerthellaceae | Bacteria;Bacteria\_none;Actinomycetota;Coriobacteriia;Eggerthellales;Eggerthellaceae;Denitrobacterium |
| Raoultibacter | 0 | 0 | 0 | 0 | 0 | 1 | 0 | 0 | 0 | 0 | 0 | 0 | 0 | 1 | Bacteria | Bacteria\_none | Actinomycetota | Coriobacteriia | Eggerthellales | Eggerthellaceae | Bacteria;Bacteria\_none;Actinomycetota;Coriobacteriia;Eggerthellales;Eggerthellaceae;Raoultibacter |
| Rubrobacter | 0 | 0 | 0 | 0 | 1 | 0 | 0 | 0 | 0 | 0 | 0 | 0 | 0 | 1 | Bacteria | Bacteria\_none | Actinomycetota | Rubrobacteria | Rubrobacterales | Rubrobacteraceae | Bacteria;Bacteria\_none;Actinomycetota;Rubrobacteria;Rubrobacterales;Rubrobacteraceae;Rubrobacter |
| Miltoncostaea | 0 | 1 | 0 | 0 | 0 | 0 | 0 | 0 | 0 | 0 | 0 | 0 | 0 | 1 | Bacteria | Bacteria\_none | Actinomycetota | Thermoleophilia | Miltoncostaeales | Miltoncostaeaceae | Bacteria;Bacteria\_none;Actinomycetota;Thermoleophilia;Miltoncostaeales;Miltoncostaeaceae;Miltoncostaea |
| Baekduia | 0 | 1 | 0 | 0 | 0 | 0 | 0 | 0 | 0 | 0 | 0 | 0 | 0 | 1 | Bacteria | Bacteria\_none | Actinomycetota | Thermoleophilia | Solirubrobacterales | Baekduiaceae | Bacteria;Bacteria\_none;Actinomycetota;Thermoleophilia;Solirubrobacterales;Baekduiaceae;Baekduia |
| Abyssicoccus | 0 | 0 | 0 | 0 | 1 | 0 | 0 | 0 | 0 | 0 | 0 | 0 | 0 | 1 | Bacteria | Bacteria\_none | Bacillota | Bacilli | Bacillales | Abyssicoccaceae | Bacteria;Bacteria\_none;Bacillota;Bacilli;Bacillales;Abyssicoccaceae;Abyssicoccus |
| Alicyclobacillus | 0 | 0 | 0 | 0 | 0 | 0 | 0 | 0 | 0 | 1 | 0 | 0 | 0 | 1 | Bacteria | Bacteria\_none | Bacillota | Bacilli | Bacillales | Alicyclobacillaceae | Bacteria;Bacteria\_none;Bacillota;Bacilli;Bacillales;Alicyclobacillaceae;Alicyclobacillus |
| Alkalihalobacillus | 0 | 0 | 0 | 1 | 0 | 0 | 0 | 0 | 0 | 0 | 0 | 0 | 1 | 2 | Bacteria | Bacteria\_none | Bacillota | Bacilli | Bacillales | Bacillaceae | Bacteria;Bacteria\_none;Bacillota;Bacilli;Bacillales;Bacillaceae;Alkalihalobacillus |
| Alkalihalophilus | 0 | 0 | 0 | 0 | 0 | 0 | 0 | 0 | 0 | 0 | 1 | 0 | 1 | 2 | Bacteria | Bacteria\_none | Bacillota | Bacilli | Bacillales | Bacillaceae | Bacteria;Bacteria\_none;Bacillota;Bacilli;Bacillales;Bacillaceae;Alkalihalophilus |
| Amphibacillus | 0 | 0 | 0 | 0 | 0 | 1 | 0 | 0 | 0 | 0 | 0 | 0 | 0 | 1 | Bacteria | Bacteria\_none | Bacillota | Bacilli | Bacillales | Bacillaceae | Bacteria;Bacteria\_none;Bacillota;Bacilli;Bacillales;Bacillaceae;Amphibacillus |
| Anaerobacillus | 0 | 1 | 0 | 0 | 0 | 0 | 0 | 0 | 0 | 0 | 0 | 0 | 0 | 1 | Bacteria | Bacteria\_none | Bacillota | Bacilli | Bacillales | Bacillaceae | Bacteria;Bacteria\_none;Bacillota;Bacilli;Bacillales;Bacillaceae;Anaerobacillus |
| Anoxybacillus | 1 | 0 | 0 | 0 | 0 | 0 | 0 | 0 | 0 | 0 | 0 | 0 | 0 | 1 | Bacteria | Bacteria\_none | Bacillota | Bacilli | Bacillales | Bacillaceae | Bacteria;Bacteria\_none;Bacillota;Bacilli;Bacillales;Bacillaceae;Anoxybacillus |
| Bacillus | 1 | 0 | 0 | 3 | 1 | 5 | 0 | 1 | 0 | 1 | 1 | 0 | 2 | 15 | Bacteria | Bacteria\_none | Bacillota | Bacilli | Bacillales | Bacillaceae | Bacteria;Bacteria\_none;Bacillota;Bacilli;Bacillales;Bacillaceae;Bacillus |
| Cytobacillus | 0 | 0 | 1 | 1 | 1 | 0 | 0 | 0 | 0 | 0 | 0 | 0 | 1 | 4 | Bacteria | Bacteria\_none | Bacillota | Bacilli | Bacillales | Bacillaceae | Bacteria;Bacteria\_none;Bacillota;Bacilli;Bacillales;Bacillaceae;Cytobacillus |
| Domibacillus | 0 | 0 | 0 | 0 | 0 | 0 | 0 | 0 | 0 | 0 | 0 | 0 | 1 | 1 | Bacteria | Bacteria\_none | Bacillota | Bacilli | Bacillales | Bacillaceae | Bacteria;Bacteria\_none;Bacillota;Bacilli;Bacillales;Bacillaceae;Domibacillus |
| Halalkalibacter | 0 | 0 | 0 | 0 | 0 | 0 | 1 | 0 | 0 | 0 | 0 | 0 | 0 | 1 | Bacteria | Bacteria\_none | Bacillota | Bacilli | Bacillales | Bacillaceae | Bacteria;Bacteria\_none;Bacillota;Bacilli;Bacillales;Bacillaceae;Halalkalibacter |
| Halobacillus | 0 | 0 | 0 | 0 | 0 | 0 | 0 | 0 | 0 | 0 | 0 | 0 | 1 | 1 | Bacteria | Bacteria\_none | Bacillota | Bacilli | Bacillales | Bacillaceae | Bacteria;Bacteria\_none;Bacillota;Bacilli;Bacillales;Bacillaceae;Halobacillus |
| Lysinibacillus | 0 | 1 | 1 | 0 | 0 | 1 | 1 | 0 | 0 | 0 | 1 | 0 | 1 | 6 | Bacteria | Bacteria\_none | Bacillota | Bacilli | Bacillales | Bacillaceae | Bacteria;Bacteria\_none;Bacillota;Bacilli;Bacillales;Bacillaceae;Lysinibacillus |
| Metabacillus | 0 | 1 | 0 | 0 | 0 | 0 | 0 | 0 | 0 | 0 | 0 | 0 | 0 | 1 | Bacteria | Bacteria\_none | Bacillota | Bacilli | Bacillales | Bacillaceae | Bacteria;Bacteria\_none;Bacillota;Bacilli;Bacillales;Bacillaceae;Metabacillus |
| Neobacillus | 0 | 0 | 1 | 0 | 1 | 0 | 0 | 0 | 0 | 0 | 0 | 0 | 0 | 2 | Bacteria | Bacteria\_none | Bacillota | Bacilli | Bacillales | Bacillaceae | Bacteria;Bacteria\_none;Bacillota;Bacilli;Bacillales;Bacillaceae;Neobacillus |
| Niallia | 1 | 0 | 1 | 0 | 0 | 1 | 0 | 0 | 0 | 0 | 0 | 0 | 1 | 4 | Bacteria | Bacteria\_none | Bacillota | Bacilli | Bacillales | Bacillaceae | Bacteria;Bacteria\_none;Bacillota;Bacilli;Bacillales;Bacillaceae;Niallia |
| Oceanobacillus | 0 | 0 | 0 | 0 | 0 | 2 | 0 | 0 | 0 | 0 | 0 | 0 | 1 | 3 | Bacteria | Bacteria\_none | Bacillota | Bacilli | Bacillales | Bacillaceae | Bacteria;Bacteria\_none;Bacillota;Bacilli;Bacillales;Bacillaceae;Oceanobacillus |
| Ornithinibacillus | 0 | 0 | 0 | 0 | 0 | 1 | 0 | 0 | 0 | 0 | 0 | 0 | 0 | 1 | Bacteria | Bacteria\_none | Bacillota | Bacilli | Bacillales | Bacillaceae | Bacteria;Bacteria\_none;Bacillota;Bacilli;Bacillales;Bacillaceae;Ornithinibacillus |
| Parageobacillus | 0 | 0 | 0 | 0 | 0 | 0 | 0 | 0 | 0 | 0 | 0 | 0 | 1 | 1 | Bacteria | Bacteria\_none | Bacillota | Bacilli | Bacillales | Bacillaceae | Bacteria;Bacteria\_none;Bacillota;Bacilli;Bacillales;Bacillaceae;Parageobacillus |
| Peribacillus | 0 | 0 | 0 | 0 | 0 | 0 | 0 | 1 | 0 | 0 | 0 | 0 | 0 | 1 | Bacteria | Bacteria\_none | Bacillota | Bacilli | Bacillales | Bacillaceae | Bacteria;Bacteria\_none;Bacillota;Bacilli;Bacillales;Bacillaceae;Peribacillus |
| Pontibacillus | 0 | 0 | 0 | 1 | 0 | 0 | 0 | 0 | 0 | 0 | 0 | 0 | 0 | 1 | Bacteria | Bacteria\_none | Bacillota | Bacilli | Bacillales | Bacillaceae | Bacteria;Bacteria\_none;Bacillota;Bacilli;Bacillales;Bacillaceae;Pontibacillus |
| Priestia | 0 | 0 | 1 | 0 | 2 | 1 | 1 | 0 | 0 | 2 | 1 | 0 | 2 | 10 | Bacteria | Bacteria\_none | Bacillota | Bacilli | Bacillales | Bacillaceae | Bacteria;Bacteria\_none;Bacillota;Bacilli;Bacillales;Bacillaceae;Priestia |
| Pseudalkalibacillus | 0 | 0 | 0 | 1 | 0 | 0 | 0 | 0 | 0 | 0 | 0 | 0 | 0 | 1 | Bacteria | Bacteria\_none | Bacillota | Bacilli | Bacillales | Bacillaceae | Bacteria;Bacteria\_none;Bacillota;Bacilli;Bacillales;Bacillaceae;Pseudalkalibacillus |
| Psychrobacillus | 1 | 0 | 0 | 0 | 0 | 0 | 0 | 0 | 0 | 0 | 0 | 0 | 1 | 2 | Bacteria | Bacteria\_none | Bacillota | Bacilli | Bacillales | Bacillaceae | Bacteria;Bacteria\_none;Bacillota;Bacilli;Bacillales;Bacillaceae;Psychrobacillus |
| Rossellomorea | 0 | 0 | 0 | 0 | 0 | 1 | 0 | 0 | 0 | 0 | 0 | 0 | 0 | 1 | Bacteria | Bacteria\_none | Bacillota | Bacilli | Bacillales | Bacillaceae | Bacteria;Bacteria\_none;Bacillota;Bacilli;Bacillales;Bacillaceae;Rossellomorea |
| Salipaludibacillus | 0 | 0 | 0 | 1 | 0 | 0 | 0 | 0 | 0 | 0 | 0 | 0 | 0 | 1 | Bacteria | Bacteria\_none | Bacillota | Bacilli | Bacillales | Bacillaceae | Bacteria;Bacteria\_none;Bacillota;Bacilli;Bacillales;Bacillaceae;Salipaludibacillus |
| Shouchella | 0 | 0 | 0 | 0 | 0 | 0 | 0 | 0 | 0 | 0 | 0 | 0 | 2 | 2 | Bacteria | Bacteria\_none | Bacillota | Bacilli | Bacillales | Bacillaceae | Bacteria;Bacteria\_none;Bacillota;Bacilli;Bacillales;Bacillaceae;Shouchella |
| Sutcliffiella | 0 | 0 | 0 | 1 | 1 | 0 | 0 | 0 | 0 | 0 | 0 | 0 | 1 | 3 | Bacteria | Bacteria\_none | Bacillota | Bacilli | Bacillales | Bacillaceae | Bacteria;Bacteria\_none;Bacillota;Bacilli;Bacillales;Bacillaceae;Sutcliffiella |
| Terribacillus | 0 | 0 | 2 | 0 | 0 | 0 | 0 | 0 | 0 | 0 | 0 | 0 | 0 | 2 | Bacteria | Bacteria\_none | Bacillota | Bacilli | Bacillales | Bacillaceae | Bacteria;Bacteria\_none;Bacillota;Bacilli;Bacillales;Bacillaceae;Terribacillus |
| Virgibacillus | 0 | 0 | 0 | 0 | 0 | 0 | 0 | 1 | 0 | 0 | 0 | 0 | 2 | 3 | Bacteria | Bacteria\_none | Bacillota | Bacilli | Bacillales | Bacillaceae | Bacteria;Bacteria\_none;Bacillota;Bacilli;Bacillales;Bacillaceae;Virgibacillus |
| Exiguobacterium | 0 | 0 | 0 | 1 | 0 | 0 | 0 | 0 | 0 | 0 | 0 | 0 | 0 | 1 | Bacteria | Bacteria\_none | Bacillota | Bacilli | Bacillales | Bacillales\_Incertae\_sedis | Bacteria;Bacteria\_none;Bacillota;Bacilli;Bacillales;Bacillales\_Incertae\_sedis;Exiguobacterium |
| Gemella | 74 | 125 | 7 | 131 | 563 | 347 | 132 | 115 | 7 | 386 | 1176 | 100 | 981 | 4144 | Bacteria | Bacteria\_none | Bacillota | Bacilli | Bacillales | Gemellaceae | Bacteria;Bacteria\_none;Bacillota;Bacilli;Bacillales;Gemellaceae;Gemella |
| Brochothrix | 0 | 0 | 0 | 0 | 0 | 0 | 0 | 0 | 0 | 0 | 0 | 0 | 1 | 1 | Bacteria | Bacteria\_none | Bacillota | Bacilli | Bacillales | Listeriaceae | Bacteria;Bacteria\_none;Bacillota;Bacilli;Bacillales;Listeriaceae;Brochothrix |
| Listeria | 0 | 0 | 0 | 0 | 0 | 0 | 0 | 0 | 0 | 0 | 0 | 0 | 1 | 1 | Bacteria | Bacteria\_none | Bacillota | Bacilli | Bacillales | Listeriaceae | Bacteria;Bacteria\_none;Bacillota;Bacilli;Bacillales;Listeriaceae;Listeria |
| Brevibacillus | 0 | 0 | 0 | 1 | 0 | 2 | 0 | 0 | 0 | 0 | 1 | 0 | 0 | 4 | Bacteria | Bacteria\_none | Bacillota | Bacilli | Bacillales | Paenibacillaceae | Bacteria;Bacteria\_none;Bacillota;Bacilli;Bacillales;Paenibacillaceae;Brevibacillus |
| Paenibacillus | 2 | 1 | 2 | 1 | 1 | 0 | 5 | 0 | 0 | 0 | 2 | 0 | 5 | 19 | Bacteria | Bacteria\_none | Bacillota | Bacilli | Bacillales | Paenibacillaceae | Bacteria;Bacteria\_none;Bacillota;Bacilli;Bacillales;Paenibacillaceae;Paenibacillus |
| Paenisporosarcina | 0 | 0 | 0 | 0 | 0 | 0 | 0 | 0 | 0 | 0 | 0 | 0 | 2 | 2 | Bacteria | Bacteria\_none | Bacillota | Bacilli | Bacillales | Planococcaceae | Bacteria;Bacteria\_none;Bacillota;Bacilli;Bacillales;Planococcaceae;Paenisporosarcina |
| Sporosarcina | 1 | 0 | 0 | 0 | 0 | 0 | 0 | 0 | 0 | 0 | 0 | 0 | 0 | 1 | Bacteria | Bacteria\_none | Bacillota | Bacilli | Bacillales | Planococcaceae | Bacteria;Bacteria\_none;Bacillota;Bacilli;Bacillales;Planococcaceae;Sporosarcina |
| Macrococcus | 1 | 0 | 0 | 0 | 0 | 0 | 0 | 0 | 0 | 0 | 0 | 0 | 0 | 1 | Bacteria | Bacteria\_none | Bacillota | Bacilli | Bacillales | Staphylococcaceae | Bacteria;Bacteria\_none;Bacillota;Bacilli;Bacillales;Staphylococcaceae;Macrococcus |
| Staphylococcus | 0 | 3 | 3 | 3 | 3 | 0 | 1 | 0 | 0 | 1 | 3 | 2 | 6 | 25 | Bacteria | Bacteria\_none | Bacillota | Bacilli | Bacillales | Staphylococcaceae | Bacteria;Bacteria\_none;Bacillota;Bacilli;Bacillales;Staphylococcaceae;Staphylococcus |
| Abiotrophia | 7 | 0 | 62 | 4 | 78 | 19 | 25 | 0 | 0 | 23 | 81 | 5 | 57 | 361 | Bacteria | Bacteria\_none | Bacillota | Bacilli | Lactobacillales | Aerococcaceae | Bacteria;Bacteria\_none;Bacillota;Bacilli;Lactobacillales;Aerococcaceae;Abiotrophia |
| Aerococcus | 1 | 0 | 0 | 0 | 0 | 0 | 0 | 0 | 0 | 0 | 0 | 0 | 2 | 3 | Bacteria | Bacteria\_none | Bacillota | Bacilli | Lactobacillales | Aerococcaceae | Bacteria;Bacteria\_none;Bacillota;Bacilli;Lactobacillales;Aerococcaceae;Aerococcus |
| Ruoffia | 0 | 1 | 0 | 0 | 0 | 0 | 0 | 0 | 0 | 0 | 0 | 0 | 1 | 2 | Bacteria | Bacteria\_none | Bacillota | Bacilli | Lactobacillales | Aerococcaceae | Bacteria;Bacteria\_none;Bacillota;Bacilli;Lactobacillales;Aerococcaceae;Ruoffia |
| Dolosigranulum | 1 | 0 | 0 | 0 | 0 | 0 | 0 | 0 | 0 | 3 | 2 | 0 | 0 | 6 | Bacteria | Bacteria\_none | Bacillota | Bacilli | Lactobacillales | Carnobacteriaceae | Bacteria;Bacteria\_none;Bacillota;Bacilli;Lactobacillales;Carnobacteriaceae;Dolosigranulum |
| Granulicatella | 430 | 65 | 426 | 147 | 212 | 130 | 519 | 28 | 0 | 213 | 149 | 30 | 479 | 2828 | Bacteria | Bacteria\_none | Bacillota | Bacilli | Lactobacillales | Carnobacteriaceae | Bacteria;Bacteria\_none;Bacillota;Bacilli;Lactobacillales;Carnobacteriaceae;Granulicatella |
| Jeotgalibaca | 1 | 0 | 0 | 0 | 0 | 0 | 0 | 0 | 0 | 0 | 1 | 0 | 0 | 2 | Bacteria | Bacteria\_none | Bacillota | Bacilli | Lactobacillales | Carnobacteriaceae | Bacteria;Bacteria\_none;Bacillota;Bacilli;Lactobacillales;Carnobacteriaceae;Jeotgalibaca |
| Enterococcus | 2 | 9 | 10 | 3 | 3 | 6 | 2 | 3 | 0 | 0 | 5 | 0 | 6 | 49 | Bacteria | Bacteria\_none | Bacillota | Bacilli | Lactobacillales | Enterococcaceae | Bacteria;Bacteria\_none;Bacillota;Bacilli;Lactobacillales;Enterococcaceae;Enterococcus |
| Tetragenococcus | 0 | 0 | 0 | 0 | 0 | 0 | 2 | 0 | 0 | 0 | 0 | 0 | 0 | 2 | Bacteria | Bacteria\_none | Bacillota | Bacilli | Lactobacillales | Enterococcaceae | Bacteria;Bacteria\_none;Bacillota;Bacilli;Lactobacillales;Enterococcaceae;Tetragenococcus |
| Vagococcus | 1 | 1 | 1 | 0 | 0 | 0 | 1 | 0 | 0 | 0 | 0 | 0 | 1 | 5 | Bacteria | Bacteria\_none | Bacillota | Bacilli | Lactobacillales | Enterococcaceae | Bacteria;Bacteria\_none;Bacillota;Bacilli;Lactobacillales;Enterococcaceae;Vagococcus |
| Lacticaseibacillus | 0 | 0 | 0 | 0 | 0 | 0 | 10 | 0 | 0 | 2 | 0 | 0 | 6 | 18 | Bacteria | Bacteria\_none | Bacillota | Bacilli | Lactobacillales | Lactobacillaceae | Bacteria;Bacteria\_none;Bacillota;Bacilli;Lactobacillales;Lactobacillaceae;Lacticaseibacillus |
| Lactobacillus | 1 | 0 | 0 | 1 | 0 | 0 | 15 | 3 | 0 | 1 | 1 | 0 | 3 | 25 | Bacteria | Bacteria\_none | Bacillota | Bacilli | Lactobacillales | Lactobacillaceae | Bacteria;Bacteria\_none;Bacillota;Bacilli;Lactobacillales;Lactobacillaceae;Lactobacillus |
| Latilactobacillus | 0 | 0 | 1 | 0 | 0 | 0 | 0 | 0 | 0 | 0 | 1 | 0 | 2 | 4 | Bacteria | Bacteria\_none | Bacillota | Bacilli | Lactobacillales | Lactobacillaceae | Bacteria;Bacteria\_none;Bacillota;Bacilli;Lactobacillales;Lactobacillaceae;Latilactobacillus |
| Lentilactobacillus | 0 | 0 | 0 | 0 | 1 | 0 | 0 | 0 | 0 | 1 | 0 | 0 | 0 | 2 | Bacteria | Bacteria\_none | Bacillota | Bacilli | Lactobacillales | Lactobacillaceae | Bacteria;Bacteria\_none;Bacillota;Bacilli;Lactobacillales;Lactobacillaceae;Lentilactobacillus |
| Leuconostoc | 0 | 0 | 0 | 1 | 0 | 1 | 0 | 0 | 0 | 0 | 0 | 0 | 0 | 2 | Bacteria | Bacteria\_none | Bacillota | Bacilli | Lactobacillales | Lactobacillaceae | Bacteria;Bacteria\_none;Bacillota;Bacilli;Lactobacillales;Lactobacillaceae;Leuconostoc |
| Levilactobacillus | 0 | 0 | 1 | 0 | 0 | 0 | 0 | 0 | 0 | 0 | 0 | 0 | 0 | 1 | Bacteria | Bacteria\_none | Bacillota | Bacilli | Lactobacillales | Lactobacillaceae | Bacteria;Bacteria\_none;Bacillota;Bacilli;Lactobacillales;Lactobacillaceae;Levilactobacillus |
| Ligilactobacillus | 1 | 0 | 0 | 0 | 1 | 0 | 1 | 1 | 2 | 0 | 1 | 0 | 1 | 8 | Bacteria | Bacteria\_none | Bacillota | Bacilli | Lactobacillales | Lactobacillaceae | Bacteria;Bacteria\_none;Bacillota;Bacilli;Lactobacillales;Lactobacillaceae;Ligilactobacillus |
| Limosilactobacillus | 0 | 0 | 0 | 0 | 0 | 0 | 0 | 0 | 0 | 0 | 0 | 0 | 5 | 5 | Bacteria | Bacteria\_none | Bacillota | Bacilli | Lactobacillales | Lactobacillaceae | Bacteria;Bacteria\_none;Bacillota;Bacilli;Lactobacillales;Lactobacillaceae;Limosilactobacillus |
| Liquorilactobacillus | 0 | 0 | 0 | 0 | 0 | 0 | 0 | 1 | 0 | 0 | 1 | 0 | 1 | 3 | Bacteria | Bacteria\_none | Bacillota | Bacilli | Lactobacillales | Lactobacillaceae | Bacteria;Bacteria\_none;Bacillota;Bacilli;Lactobacillales;Lactobacillaceae;Liquorilactobacillus |
| Nicoliella | 0 | 0 | 0 | 0 | 1 | 0 | 0 | 0 | 0 | 0 | 0 | 0 | 0 | 1 | Bacteria | Bacteria\_none | Bacillota | Bacilli | Lactobacillales | Lactobacillaceae | Bacteria;Bacteria\_none;Bacillota;Bacilli;Lactobacillales;Lactobacillaceae;Nicoliella |
| Pediococcus | 0 | 0 | 1 | 0 | 0 | 0 | 0 | 0 | 0 | 0 | 0 | 0 | 0 | 1 | Bacteria | Bacteria\_none | Bacillota | Bacilli | Lactobacillales | Lactobacillaceae | Bacteria;Bacteria\_none;Bacillota;Bacilli;Lactobacillales;Lactobacillaceae;Pediococcus |
| Weissella | 0 | 0 | 0 | 1 | 0 | 0 | 1 | 0 | 0 | 0 | 0 | 0 | 0 | 2 | Bacteria | Bacteria\_none | Bacillota | Bacilli | Lactobacillales | Lactobacillaceae | Bacteria;Bacteria\_none;Bacillota;Bacilli;Lactobacillales;Lactobacillaceae;Weissella |
| Lactococcus | 0 | 1 | 0 | 0 | 0 | 5 | 1 | 1 | 0 | 2 | 0 | 0 | 9 | 19 | Bacteria | Bacteria\_none | Bacillota | Bacilli | Lactobacillales | Streptococcaceae | Bacteria;Bacteria\_none;Bacillota;Bacilli;Lactobacillales;Streptococcaceae;Lactococcus |
| Streptococcus | 1847 | 1370 | 726 | 419 | 2469 | 1772 | 2244 | 414 | 583 | 1032 | 2827 | 1010 | 4733 | 21446 | Bacteria | Bacteria\_none | Bacillota | Bacilli | Lactobacillales | Streptococcaceae | Bacteria;Bacteria\_none;Bacillota;Bacilli;Lactobacillales;Streptococcaceae;Streptococcus |
| Caldicellulosiruptor | 0 | 0 | 0 | 0 | 1 | 0 | 0 | 0 | 0 | 0 | 0 | 0 | 0 | 1 | Bacteria | Bacteria\_none | Bacillota | Bacillota\_Incertae\_sedis | Caldicellulosiruptorales | Caldicellulosiruptoraceae | Bacteria;Bacteria\_none;Bacillota;Bacillota\_Incertae\_sedis;Caldicellulosiruptorales;Caldicellulosiruptoraceae;Caldicellulosiruptor |
| Aristaeella | 0 | 0 | 0 | 0 | 0 | 0 | 0 | 1 | 0 | 0 | 0 | 0 | 0 | 1 | Bacteria | Bacteria\_none | Bacillota | Clostridia | Eubacteriales | Aristaeellaceae | Bacteria;Bacteria\_none;Bacillota;Clostridia;Eubacteriales;Aristaeellaceae;Aristaeella |
| Clostridium | 1 | 2 | 3 | 5 | 4 | 7 | 0 | 0 | 3 | 0 | 3 | 1 | 15 | 44 | Bacteria | Bacteria\_none | Bacillota | Clostridia | Eubacteriales | Clostridiaceae | Bacteria;Bacteria\_none;Bacillota;Clostridia;Eubacteriales;Clostridiaceae;Clostridium |
| Crassaminicella | 0 | 0 | 0 | 0 | 0 | 3 | 0 | 0 | 0 | 0 | 0 | 0 | 1 | 4 | Bacteria | Bacteria\_none | Bacillota | Clostridia | Eubacteriales | Clostridiaceae | Bacteria;Bacteria\_none;Bacillota;Clostridia;Eubacteriales;Clostridiaceae;Crassaminicella |
| Oceanirhabdus | 0 | 0 | 0 | 0 | 0 | 1 | 0 | 0 | 0 | 0 | 0 | 0 | 0 | 1 | Bacteria | Bacteria\_none | Bacillota | Clostridia | Eubacteriales | Clostridiaceae | Bacteria;Bacteria\_none;Bacillota;Clostridia;Eubacteriales;Clostridiaceae;Oceanirhabdus |
| Sarcina | 0 | 0 | 0 | 0 | 0 | 1 | 0 | 1 | 1 | 0 | 0 | 0 | 0 | 3 | Bacteria | Bacteria\_none | Bacillota | Clostridia | Eubacteriales | Clostridiaceae | Bacteria;Bacteria\_none;Bacillota;Clostridia;Eubacteriales;Clostridiaceae;Sarcina |
| Carboxydocella | 0 | 0 | 0 | 0 | 0 | 0 | 0 | 0 | 0 | 0 | 0 | 0 | 1 | 1 | Bacteria | Bacteria\_none | Bacillota | Clostridia | Eubacteriales | Clostridiales Family XVI. Incertae Sedis | Bacteria;Bacteria\_none;Bacillota;Clostridia;Eubacteriales;Clostridiales Family XVI. Incertae Sedis;Carboxydocella |
| Dehalobacter | 0 | 0 | 0 | 0 | 0 | 0 | 0 | 0 | 0 | 0 | 0 | 0 | 1 | 1 | Bacteria | Bacteria\_none | Bacillota | Clostridia | Eubacteriales | Desulfitobacteriaceae | Bacteria;Bacteria\_none;Bacillota;Clostridia;Eubacteriales;Desulfitobacteriaceae;Dehalobacter |
| Desulfosporosinus | 0 | 0 | 0 | 0 | 0 | 1 | 0 | 0 | 1 | 0 | 0 | 0 | 0 | 2 | Bacteria | Bacteria\_none | Bacillota | Clostridia | Eubacteriales | Desulfitobacteriaceae | Bacteria;Bacteria\_none;Bacillota;Clostridia;Eubacteriales;Desulfitobacteriaceae;Desulfosporosinus |
| Acetobacterium | 0 | 0 | 1 | 0 | 0 | 0 | 1 | 0 | 0 | 0 | 0 | 0 | 0 | 2 | Bacteria | Bacteria\_none | Bacillota | Clostridia | Eubacteriales | Eubacteriaceae | Bacteria;Bacteria\_none;Bacillota;Clostridia;Eubacteriales;Eubacteriaceae;Acetobacterium |
| Eubacterium | 0 | 0 | 0 | 0 | 0 | 1 | 0 | 0 | 0 | 0 | 0 | 0 | 1 | 2 | Bacteria | Bacteria\_none | Bacillota | Clostridia | Eubacteriales | Eubacteriaceae | Bacteria;Bacteria\_none;Bacillota;Clostridia;Eubacteriales;Eubacteriaceae;Eubacterium |
| Sinanaerobacter | 0 | 0 | 0 | 0 | 0 | 0 | 0 | 0 | 0 | 0 | 0 | 1 | 1 | 2 | Bacteria | Bacteria\_none | Bacillota | Clostridia | Eubacteriales | Eubacteriales Family XIII. Incertae Sedis | Bacteria;Bacteria\_none;Bacillota;Clostridia;Eubacteriales;Eubacteriales Family XIII. Incertae Sedis;Sinanaerobacter |
| Fenollaria | 0 | 0 | 0 | 0 | 0 | 0 | 0 | 0 | 0 | 0 | 2 | 0 | 0 | 2 | Bacteria | Bacteria\_none | Bacillota | Clostridia | Eubacteriales | Eubacteriales\_Incertae\_sedis | Bacteria;Bacteria\_none;Bacillota;Clostridia;Eubacteriales;Eubacteriales\_Incertae\_sedis;Fenollaria |
| Massilistercora | 0 | 0 | 0 | 0 | 0 | 2 | 0 | 0 | 0 | 0 | 0 | 0 | 0 | 2 | Bacteria | Bacteria\_none | Bacillota | Clostridia | Eubacteriales | Eubacteriales\_Incertae\_sedis | Bacteria;Bacteria\_none;Bacillota;Clostridia;Eubacteriales;Eubacteriales\_Incertae\_sedis;Massilistercora |
| Acetivibrio | 0 | 0 | 0 | 0 | 0 | 0 | 0 | 0 | 0 | 1 | 1 | 0 | 0 | 2 | Bacteria | Bacteria\_none | Bacillota | Clostridia | Eubacteriales | Oscillospiraceae | Bacteria;Bacteria\_none;Bacillota;Clostridia;Eubacteriales;Oscillospiraceae;Acetivibrio |
| Amygdalobacter | 0 | 0 | 1 | 0 | 1 | 1 | 0 | 0 | 0 | 0 | 0 | 0 | 0 | 3 | Bacteria | Bacteria\_none | Bacillota | Clostridia | Eubacteriales | Oscillospiraceae | Bacteria;Bacteria\_none;Bacillota;Clostridia;Eubacteriales;Oscillospiraceae;Amygdalobacter |
| Caproicibacterium | 1 | 0 | 0 | 0 | 0 | 2 | 5 | 0 | 0 | 0 | 0 | 0 | 1 | 9 | Bacteria | Bacteria\_none | Bacillota | Clostridia | Eubacteriales | Oscillospiraceae | Bacteria;Bacteria\_none;Bacillota;Clostridia;Eubacteriales;Oscillospiraceae;Caproicibacterium |
| Ethanoligenens | 0 | 0 | 0 | 0 | 0 | 0 | 0 | 0 | 0 | 0 | 0 | 0 | 1 | 1 | Bacteria | Bacteria\_none | Bacillota | Clostridia | Eubacteriales | Oscillospiraceae | Bacteria;Bacteria\_none;Bacillota;Clostridia;Eubacteriales;Oscillospiraceae;Ethanoligenens |
| Faecalibacterium | 0 | 1 | 1 | 0 | 0 | 0 | 0 | 0 | 0 | 0 | 0 | 0 | 0 | 2 | Bacteria | Bacteria\_none | Bacillota | Clostridia | Eubacteriales | Oscillospiraceae | Bacteria;Bacteria\_none;Bacillota;Clostridia;Eubacteriales;Oscillospiraceae;Faecalibacterium |
| Fastidiosipila | 2 | 0 | 0 | 0 | 0 | 1 | 0 | 0 | 0 | 0 | 0 | 0 | 0 | 3 | Bacteria | Bacteria\_none | Bacillota | Clostridia | Eubacteriales | Oscillospiraceae | Bacteria;Bacteria\_none;Bacillota;Clostridia;Eubacteriales;Oscillospiraceae;Fastidiosipila |
| Mageeibacillus | 0 | 0 | 0 | 0 | 0 | 0 | 0 | 0 | 0 | 0 | 0 | 0 | 1 | 1 | Bacteria | Bacteria\_none | Bacillota | Clostridia | Eubacteriales | Oscillospiraceae | Bacteria;Bacteria\_none;Bacillota;Clostridia;Eubacteriales;Oscillospiraceae;Mageeibacillus |
| Paludihabitans | 0 | 0 | 0 | 0 | 0 | 1 | 0 | 0 | 0 | 0 | 0 | 0 | 0 | 1 | Bacteria | Bacteria\_none | Bacillota | Clostridia | Eubacteriales | Oscillospiraceae | Bacteria;Bacteria\_none;Bacillota;Clostridia;Eubacteriales;Oscillospiraceae;Paludihabitans |
| Petroclostridium | 0 | 1 | 0 | 0 | 0 | 0 | 0 | 0 | 0 | 0 | 0 | 0 | 0 | 1 | Bacteria | Bacteria\_none | Bacillota | Clostridia | Eubacteriales | Oscillospiraceae | Bacteria;Bacteria\_none;Bacillota;Clostridia;Eubacteriales;Oscillospiraceae;Petroclostridium |
| Ruminiclostridium | 0 | 0 | 0 | 1 | 0 | 0 | 0 | 0 | 0 | 0 | 0 | 0 | 0 | 1 | Bacteria | Bacteria\_none | Bacillota | Clostridia | Eubacteriales | Oscillospiraceae | Bacteria;Bacteria\_none;Bacillota;Clostridia;Eubacteriales;Oscillospiraceae;Ruminiclostridium |
| Ruminococcus | 0 | 1 | 0 | 0 | 1 | 0 | 0 | 0 | 0 | 0 | 0 | 0 | 0 | 2 | Bacteria | Bacteria\_none | Bacillota | Clostridia | Eubacteriales | Oscillospiraceae | Bacteria;Bacteria\_none;Bacillota;Clostridia;Eubacteriales;Oscillospiraceae;Ruminococcus |
| Thermoclostridium | 0 | 0 | 0 | 1 | 0 | 1 | 0 | 0 | 0 | 0 | 0 | 0 | 0 | 2 | Bacteria | Bacteria\_none | Bacillota | Clostridia | Eubacteriales | Oscillospiraceae | Bacteria;Bacteria\_none;Bacillota;Clostridia;Eubacteriales;Oscillospiraceae;Thermoclostridium |
| Halobacteroides | 0 | 0 | 0 | 0 | 0 | 0 | 0 | 0 | 0 | 0 | 0 | 0 | 1 | 1 | Bacteria | Bacteria\_none | Bacillota | Clostridia | Halanaerobiales | Halobacteroidaceae | Bacteria;Bacteria\_none;Bacillota;Clostridia;Halanaerobiales;Halobacteroidaceae;Halobacteroides |
| Anaerotignum | 0 | 0 | 0 | 0 | 0 | 0 | 1 | 0 | 0 | 0 | 0 | 0 | 0 | 1 | Bacteria | Bacteria\_none | Bacillota | Clostridia | Lachnospirales | Anaerotignaceae | Bacteria;Bacteria\_none;Bacillota;Clostridia;Lachnospirales;Anaerotignaceae;Anaerotignum |
| Agathobacter | 0 | 0 | 1 | 0 | 0 | 0 | 0 | 0 | 0 | 0 | 0 | 0 | 0 | 1 | Bacteria | Bacteria\_none | Bacillota | Clostridia | Lachnospirales | Lachnospiraceae | Bacteria;Bacteria\_none;Bacillota;Clostridia;Lachnospirales;Lachnospiraceae;Agathobacter |
| Anaerobutyricum | 0 | 0 | 0 | 0 | 2 | 0 | 0 | 0 | 0 | 1 | 0 | 0 | 1 | 4 | Bacteria | Bacteria\_none | Bacillota | Clostridia | Lachnospirales | Lachnospiraceae | Bacteria;Bacteria\_none;Bacillota;Clostridia;Lachnospirales;Lachnospiraceae;Anaerobutyricum |
| Anaerocolumna | 0 | 0 | 0 | 0 | 0 | 5 | 0 | 0 | 0 | 0 | 0 | 0 | 0 | 5 | Bacteria | Bacteria\_none | Bacillota | Clostridia | Lachnospirales | Lachnospiraceae | Bacteria;Bacteria\_none;Bacillota;Clostridia;Lachnospirales;Lachnospiraceae;Anaerocolumna |
| Anaeromicropila | 0 | 0 | 1 | 0 | 0 | 0 | 0 | 1 | 0 | 0 | 0 | 0 | 0 | 2 | Bacteria | Bacteria\_none | Bacillota | Clostridia | Lachnospirales | Lachnospiraceae | Bacteria;Bacteria\_none;Bacillota;Clostridia;Lachnospirales;Lachnospiraceae;Anaeromicropila |
| Anaerostipes | 0 | 0 | 0 | 0 | 0 | 0 | 0 | 0 | 0 | 0 | 0 | 0 | 1 | 1 | Bacteria | Bacteria\_none | Bacillota | Clostridia | Lachnospirales | Lachnospiraceae | Bacteria;Bacteria\_none;Bacillota;Clostridia;Lachnospirales;Lachnospiraceae;Anaerostipes |
| Blautia | 0 | 0 | 0 | 0 | 0 | 4 | 2 | 0 | 0 | 0 | 0 | 0 | 1 | 7 | Bacteria | Bacteria\_none | Bacillota | Clostridia | Lachnospirales | Lachnospiraceae | Bacteria;Bacteria\_none;Bacillota;Clostridia;Lachnospirales;Lachnospiraceae;Blautia |
| Butyrivibrio | 0 | 0 | 0 | 0 | 0 | 4 | 0 | 0 | 0 | 0 | 1 | 0 | 0 | 5 | Bacteria | Bacteria\_none | Bacillota | Clostridia | Lachnospirales | Lachnospiraceae | Bacteria;Bacteria\_none;Bacillota;Clostridia;Lachnospirales;Lachnospiraceae;Butyrivibrio |
| Candidatus Epulonipiscium | 0 | 0 | 0 | 0 | 0 | 0 | 0 | 0 | 0 | 0 | 0 | 1 | 0 | 1 | Bacteria | Bacteria\_none | Bacillota | Clostridia | Lachnospirales | Lachnospiraceae | Bacteria;Bacteria\_none;Bacillota;Clostridia;Lachnospirales;Lachnospiraceae;Candidatus Epulonipiscium |
| Enterocloster | 0 | 0 | 0 | 0 | 0 | 0 | 0 | 0 | 0 | 0 | 1 | 0 | 0 | 1 | Bacteria | Bacteria\_none | Bacillota | Clostridia | Lachnospirales | Lachnospiraceae | Bacteria;Bacteria\_none;Bacillota;Clostridia;Lachnospirales;Lachnospiraceae;Enterocloster |
| Hungatella | 0 | 0 | 0 | 0 | 0 | 0 | 1 | 0 | 0 | 0 | 0 | 0 | 0 | 1 | Bacteria | Bacteria\_none | Bacillota | Clostridia | Lachnospirales | Lachnospiraceae | Bacteria;Bacteria\_none;Bacillota;Clostridia;Lachnospirales;Lachnospiraceae;Hungatella |
| Lachnoanaerobaculum | 1 | 26 | 6 | 16 | 10 | 29 | 2 | 0 | 1 | 5 | 1 | 3 | 15 | 115 | Bacteria | Bacteria\_none | Bacillota | Clostridia | Lachnospirales | Lachnospiraceae | Bacteria;Bacteria\_none;Bacillota;Clostridia;Lachnospirales;Lachnospiraceae;Lachnoanaerobaculum |
| Lachnoclostridium | 0 | 0 | 1 | 0 | 0 | 2 | 0 | 0 | 0 | 0 | 0 | 0 | 0 | 3 | Bacteria | Bacteria\_none | Bacillota | Clostridia | Lachnospirales | Lachnospiraceae | Bacteria;Bacteria\_none;Bacillota;Clostridia;Lachnospirales;Lachnospiraceae;Lachnoclostridium |
| Mediterraneibacter | 0 | 0 | 0 | 0 | 0 | 0 | 0 | 0 | 0 | 0 | 0 | 3 | 0 | 3 | Bacteria | Bacteria\_none | Bacillota | Clostridia | Lachnospirales | Lachnospiraceae | Bacteria;Bacteria\_none;Bacillota;Clostridia;Lachnospirales;Lachnospiraceae;Mediterraneibacter |
| Qiania | 0 | 0 | 0 | 0 | 1 | 0 | 0 | 0 | 0 | 0 | 0 | 0 | 0 | 1 | Bacteria | Bacteria\_none | Bacillota | Clostridia | Lachnospirales | Lachnospiraceae | Bacteria;Bacteria\_none;Bacillota;Clostridia;Lachnospirales;Lachnospiraceae;Qiania |
| Roseburia | 0 | 0 | 2 | 0 | 0 | 2 | 0 | 0 | 0 | 0 | 0 | 0 | 1 | 5 | Bacteria | Bacteria\_none | Bacillota | Clostridia | Lachnospirales | Lachnospiraceae | Bacteria;Bacteria\_none;Bacillota;Clostridia;Lachnospirales;Lachnospiraceae;Roseburia |
| Simiaoa | 0 | 0 | 0 | 0 | 0 | 0 | 0 | 0 | 0 | 0 | 0 | 0 | 1 | 1 | Bacteria | Bacteria\_none | Bacillota | Clostridia | Lachnospirales | Lachnospiraceae | Bacteria;Bacteria\_none;Bacillota;Clostridia;Lachnospirales;Lachnospiraceae;Simiaoa |
| Stomatobaculum | 0 | 9 | 17 | 1 | 0 | 20 | 8 | 0 | 0 | 5 | 2 | 1 | 9 | 72 | Bacteria | Bacteria\_none | Bacillota | Clostridia | Lachnospirales | Lachnospiraceae | Bacteria;Bacteria\_none;Bacillota;Clostridia;Lachnospirales;Lachnospiraceae;Stomatobaculum |
| Tyzzerella | 0 | 0 | 0 | 0 | 1 | 0 | 0 | 0 | 0 | 0 | 0 | 0 | 0 | 1 | Bacteria | Bacteria\_none | Bacillota | Clostridia | Lachnospirales | Lachnospiraceae | Bacteria;Bacteria\_none;Bacillota;Clostridia;Lachnospirales;Lachnospiraceae;Tyzzerella |
| Wansuia | 0 | 0 | 0 | 0 | 1 | 0 | 0 | 0 | 0 | 0 | 0 | 0 | 0 | 1 | Bacteria | Bacteria\_none | Bacillota | Clostridia | Lachnospirales | Lachnospiraceae | Bacteria;Bacteria\_none;Bacillota;Clostridia;Lachnospirales;Lachnospiraceae;Wansuia |
| Wujia | 0 | 0 | 0 | 0 | 0 | 0 | 0 | 0 | 0 | 0 | 0 | 0 | 1 | 1 | Bacteria | Bacteria\_none | Bacillota | Clostridia | Lachnospirales | Lachnospiraceae | Bacteria;Bacteria\_none;Bacillota;Clostridia;Lachnospirales;Lachnospiraceae;Wujia |
| Monoglobus | 0 | 0 | 0 | 0 | 0 | 0 | 0 | 2 | 0 | 0 | 0 | 0 | 0 | 2 | Bacteria | Bacteria\_none | Bacillota | Clostridia | Monoglobales | Monoglobaceae | Bacteria;Bacteria\_none;Bacillota;Clostridia;Monoglobales;Monoglobaceae;Monoglobus |
| Natranaerobius | 0 | 0 | 0 | 1 | 0 | 0 | 0 | 0 | 0 | 0 | 0 | 0 | 0 | 1 | Bacteria | Bacteria\_none | Bacillota | Clostridia | Natranaerobiales | Natranaerobiaceae | Bacteria;Bacteria\_none;Bacillota;Clostridia;Natranaerobiales;Natranaerobiaceae;Natranaerobius |
| Natranaerofaba | 0 | 0 | 0 | 0 | 0 | 1 | 0 | 0 | 0 | 0 | 0 | 0 | 0 | 1 | Bacteria | Bacteria\_none | Bacillota | Clostridia | Natranaerobiales | Natranaerofabaceae | Bacteria;Bacteria\_none;Bacillota;Clostridia;Natranaerobiales;Natranaerofabaceae;Natranaerofaba |
| Aminipila | 0 | 0 | 0 | 0 | 0 | 1 | 0 | 0 | 0 | 0 | 0 | 1 | 0 | 2 | Bacteria | Bacteria\_none | Bacillota | Clostridia | Peptostreptococcales | Anaerovoracaceae | Bacteria;Bacteria\_none;Bacillota;Clostridia;Peptostreptococcales;Anaerovoracaceae;Aminipila |
| Emergencia | 0 | 0 | 0 | 0 | 0 | 0 | 0 | 0 | 0 | 0 | 0 | 0 | 1 | 1 | Bacteria | Bacteria\_none | Bacillota | Clostridia | Peptostreptococcales | Anaerovoracaceae | Bacteria;Bacteria\_none;Bacillota;Clostridia;Peptostreptococcales;Anaerovoracaceae;Emergencia |
| Mogibacterium | 1 | 0 | 9 | 1 | 2 | 34 | 3 | 5 | 1 | 0 | 4 | 2 | 22 | 84 | Bacteria | Bacteria\_none | Bacillota | Clostridia | Peptostreptococcales | Anaerovoracaceae | Bacteria;Bacteria\_none;Bacillota;Clostridia;Peptostreptococcales;Anaerovoracaceae;Mogibacterium |
| Filifactor | 3 | 38 | 35 | 0 | 2 | 2 | 3 | 54 | 0 | 0 | 2 | 18 | 46 | 203 | Bacteria | Bacteria\_none | Bacillota | Clostridia | Peptostreptococcales | Filifactoraceae | Bacteria;Bacteria\_none;Bacillota;Clostridia;Peptostreptococcales;Filifactoraceae;Filifactor |
| Serpentinicella | 0 | 0 | 0 | 1 | 0 | 0 | 0 | 0 | 0 | 0 | 0 | 0 | 0 | 1 | Bacteria | Bacteria\_none | Bacillota | Clostridia | Peptostreptococcales | Natronincolaceae | Bacteria;Bacteria\_none;Bacillota;Clostridia;Peptostreptococcales;Natronincolaceae;Serpentinicella |
| Clostridioides | 0 | 0 | 0 | 1 | 4 | 0 | 1 | 0 | 1 | 0 | 1 | 0 | 1 | 9 | Bacteria | Bacteria\_none | Bacillota | Clostridia | Peptostreptococcales | Peptostreptococcaceae | Bacteria;Bacteria\_none;Bacillota;Clostridia;Peptostreptococcales;Peptostreptococcaceae;Clostridioides |
| Peptostreptococcus | 0 | 0 | 3 | 3 | 0 | 1 | 0 | 0 | 0 | 0 | 1 | 0 | 1 | 9 | Bacteria | Bacteria\_none | Bacillota | Clostridia | Peptostreptococcales | Peptostreptococcaceae | Bacteria;Bacteria\_none;Bacillota;Clostridia;Peptostreptococcales;Peptostreptococcaceae;Peptostreptococcus |
| Romboutsia | 0 | 1 | 0 | 1 | 0 | 0 | 0 | 0 | 0 | 0 | 0 | 0 | 0 | 2 | Bacteria | Bacteria\_none | Bacillota | Clostridia | Peptostreptococcales | Peptostreptococcaceae | Bacteria;Bacteria\_none;Bacillota;Clostridia;Peptostreptococcales;Peptostreptococcaceae;Romboutsia |
| Terrisporobacter | 1 | 0 | 0 | 0 | 3 | 0 | 0 | 0 | 0 | 0 | 2 | 0 | 2 | 8 | Bacteria | Bacteria\_none | Bacillota | Clostridia | Peptostreptococcales | Peptostreptococcaceae | Bacteria;Bacteria\_none;Bacillota;Clostridia;Peptostreptococcales;Peptostreptococcaceae;Terrisporobacter |
| Thermoanaerobacterium | 0 | 1 | 0 | 0 | 0 | 0 | 0 | 0 | 0 | 1 | 0 | 0 | 0 | 2 | Bacteria | Bacteria\_none | Bacillota | Clostridia | Thermoanaerobacterales | Thermoanaerobacteraceae | Bacteria;Bacteria\_none;Bacillota;Clostridia;Thermoanaerobacterales;Thermoanaerobacteraceae;Thermoanaerobacterium |
| Catenibacterium | 0 | 0 | 0 | 1 | 0 | 0 | 0 | 0 | 0 | 0 | 0 | 0 | 0 | 1 | Bacteria | Bacteria\_none | Bacillota | Erysipelotrichia | Erysipelotrichales | Coprobacillaceae | Bacteria;Bacteria\_none;Bacillota;Erysipelotrichia;Erysipelotrichales;Coprobacillaceae;Catenibacterium |
| Faecalibacillus | 0 | 0 | 0 | 0 | 0 | 1 | 0 | 0 | 0 | 0 | 0 | 0 | 1 | 2 | Bacteria | Bacteria\_none | Bacillota | Erysipelotrichia | Erysipelotrichales | Coprobacillaceae | Bacteria;Bacteria\_none;Bacillota;Erysipelotrichia;Erysipelotrichales;Coprobacillaceae;Faecalibacillus |
| Thomasclavelia | 0 | 0 | 0 | 0 | 0 | 0 | 2 | 0 | 0 | 0 | 0 | 0 | 1 | 3 | Bacteria | Bacteria\_none | Bacillota | Erysipelotrichia | Erysipelotrichales | Coprobacillaceae | Bacteria;Bacteria\_none;Bacillota;Erysipelotrichia;Erysipelotrichales;Coprobacillaceae;Thomasclavelia |
| Bulleidia | 0 | 0 | 0 | 0 | 0 | 0 | 0 | 0 | 0 | 0 | 2 | 0 | 1 | 3 | Bacteria | Bacteria\_none | Bacillota | Erysipelotrichia | Erysipelotrichales | Erysipelotrichaceae | Bacteria;Bacteria\_none;Bacillota;Erysipelotrichia;Erysipelotrichales;Erysipelotrichaceae;Bulleidia |
| Erysipelothrix | 0 | 1 | 0 | 0 | 1 | 1 | 0 | 0 | 0 | 0 | 0 | 0 | 0 | 3 | Bacteria | Bacteria\_none | Bacillota | Erysipelotrichia | Erysipelotrichales | Erysipelotrichaceae | Bacteria;Bacteria\_none;Bacillota;Erysipelotrichia;Erysipelotrichales;Erysipelotrichaceae;Erysipelothrix |
| Erysipelotrichaceae\_Incertae\_sedis | 0 | 0 | 0 | 0 | 0 | 0 | 0 | 0 | 0 | 2 | 0 | 0 | 0 | 2 | Bacteria | Bacteria\_none | Bacillota | Erysipelotrichia | Erysipelotrichales | Erysipelotrichaceae | Bacteria;Bacteria\_none;Bacillota;Erysipelotrichia;Erysipelotrichales;Erysipelotrichaceae;Erysipelotrichaceae\_Incertae\_sedis |
| Solobacterium | 14 | 16 | 92 | 9 | 15 | 521 | 47 | 21 | 1 | 6 | 8 | 2 | 151 | 903 | Bacteria | Bacteria\_none | Bacillota | Erysipelotrichia | Erysipelotrichales | Erysipelotrichaceae | Bacteria;Bacteria\_none;Bacillota;Erysipelotrichia;Erysipelotrichales;Erysipelotrichaceae;Solobacterium |
| Megamonas | 0 | 0 | 1 | 1 | 0 | 0 | 0 | 0 | 0 | 0 | 1 | 0 | 0 | 3 | Bacteria | Bacteria\_none | Bacillota | Negativicutes | Selenomonadales | Selenomonadaceae | Bacteria;Bacteria\_none;Bacillota;Negativicutes;Selenomonadales;Selenomonadaceae;Megamonas |
| Pectinatus | 0 | 0 | 0 | 0 | 0 | 0 | 1 | 0 | 0 | 0 | 0 | 0 | 0 | 1 | Bacteria | Bacteria\_none | Bacillota | Negativicutes | Selenomonadales | Selenomonadaceae | Bacteria;Bacteria\_none;Bacillota;Negativicutes;Selenomonadales;Selenomonadaceae;Pectinatus |
| Selenomonas | 5 | 7 | 15 | 5 | 9 | 14 | 7 | 10 | 0 | 2 | 3 | 10 | 16 | 103 | Bacteria | Bacteria\_none | Bacillota | Negativicutes | Selenomonadales | Selenomonadaceae | Bacteria;Bacteria\_none;Bacillota;Negativicutes;Selenomonadales;Selenomonadaceae;Selenomonas |
| Sporomusa | 0 | 0 | 0 | 1 | 0 | 0 | 0 | 0 | 1 | 0 | 0 | 0 | 0 | 2 | Bacteria | Bacteria\_none | Bacillota | Negativicutes | Selenomonadales | Sporomusaceae | Bacteria;Bacteria\_none;Bacillota;Negativicutes;Selenomonadales;Sporomusaceae;Sporomusa |
| Dialister | 0 | 0 | 1 | 0 | 0 | 0 | 0 | 1 | 0 | 0 | 2 | 2 | 5 | 11 | Bacteria | Bacteria\_none | Bacillota | Negativicutes | Veillonellales | Veillonellaceae | Bacteria;Bacteria\_none;Bacillota;Negativicutes;Veillonellales;Veillonellaceae;Dialister |
| Megasphaera | 0 | 0 | 0 | 0 | 0 | 0 | 0 | 0 | 0 | 0 | 1 | 0 | 0 | 1 | Bacteria | Bacteria\_none | Bacillota | Negativicutes | Veillonellales | Veillonellaceae | Bacteria;Bacteria\_none;Bacillota;Negativicutes;Veillonellales;Veillonellaceae;Megasphaera |
| Veillonella | 1186 | 78 | 42 | 100 | 69 | 53 | 37 | 72 | 70 | 143 | 11 | 124 | 650 | 2635 | Bacteria | Bacteria\_none | Bacillota | Negativicutes | Veillonellales | Veillonellaceae | Bacteria;Bacteria\_none;Bacillota;Negativicutes;Veillonellales;Veillonellaceae;Veillonella |
| Acidilutibacter | 0 | 0 | 0 | 0 | 1 | 0 | 0 | 0 | 0 | 0 | 0 | 0 | 0 | 1 | Bacteria | Bacteria\_none | Bacillota | Tissierellia | Tissierellales | Acidilutibacteraceae | Bacteria;Bacteria\_none;Bacillota;Tissierellia;Tissierellales;Acidilutibacteraceae;Acidilutibacter |
| Gottschalkia | 0 | 0 | 0 | 0 | 0 | 0 | 0 | 0 | 0 | 0 | 0 | 0 | 1 | 1 | Bacteria | Bacteria\_none | Bacillota | Tissierellia | Tissierellales | Gottschalkiaceae | Bacteria;Bacteria\_none;Bacillota;Tissierellia;Tissierellales;Gottschalkiaceae;Gottschalkia |
| Anaerococcus | 1 | 0 | 0 | 0 | 0 | 0 | 0 | 0 | 0 | 0 | 1 | 0 | 0 | 2 | Bacteria | Bacteria\_none | Bacillota | Tissierellia | Tissierellales | Peptoniphilaceae | Bacteria;Bacteria\_none;Bacillota;Tissierellia;Tissierellales;Peptoniphilaceae;Anaerococcus |
| Finegoldia | 0 | 0 | 0 | 0 | 0 | 1 | 0 | 0 | 0 | 0 | 1 | 0 | 0 | 2 | Bacteria | Bacteria\_none | Bacillota | Tissierellia | Tissierellales | Peptoniphilaceae | Bacteria;Bacteria\_none;Bacillota;Tissierellia;Tissierellales;Peptoniphilaceae;Finegoldia |
| Helcococcus | 0 | 0 | 0 | 1 | 0 | 0 | 0 | 2 | 0 | 0 | 0 | 1 | 1 | 5 | Bacteria | Bacteria\_none | Bacillota | Tissierellia | Tissierellales | Peptoniphilaceae | Bacteria;Bacteria\_none;Bacillota;Tissierellia;Tissierellales;Peptoniphilaceae;Helcococcus |
| Miniphocaeibacter | 0 | 0 | 0 | 0 | 0 | 0 | 0 | 0 | 0 | 0 | 0 | 0 | 1 | 1 | Bacteria | Bacteria\_none | Bacillota | Tissierellia | Tissierellales | Peptoniphilaceae | Bacteria;Bacteria\_none;Bacillota;Tissierellia;Tissierellales;Peptoniphilaceae;Miniphocaeibacter |
| Parvimonas | 5 | 34 | 25 | 15 | 48 | 70 | 7 | 63 | 0 | 2 | 71 | 25 | 145 | 510 | Bacteria | Bacteria\_none | Bacillota | Tissierellia | Tissierellales | Peptoniphilaceae | Bacteria;Bacteria\_none;Bacillota;Tissierellia;Tissierellales;Peptoniphilaceae;Parvimonas |
| Peptoniphilus | 0 | 0 | 0 | 0 | 0 | 1 | 0 | 0 | 0 | 0 | 0 | 0 | 0 | 1 | Bacteria | Bacteria\_none | Bacillota | Tissierellia | Tissierellales | Peptoniphilaceae | Bacteria;Bacteria\_none;Bacillota;Tissierellia;Tissierellales;Peptoniphilaceae;Peptoniphilus |
| Keratinibaculum | 0 | 0 | 0 | 0 | 1 | 0 | 0 | 0 | 0 | 0 | 0 | 0 | 0 | 1 | Bacteria | Bacteria\_none | Bacillota | Tissierellia | Tissierellales | Tepidimicrobiaceae | Bacteria;Bacteria\_none;Bacillota;Tissierellia;Tissierellales;Tepidimicrobiaceae;Keratinibaculum |
| Ezakiella | 0 | 0 | 0 | 1 | 1 | 0 | 0 | 0 | 0 | 0 | 12 | 1 | 5 | 20 | Bacteria | Bacteria\_none | Bacillota | Tissierellia | Tissierellia\_Incertae\_sedis | Tissierellia\_Incertae\_sedis | Bacteria;Bacteria\_none;Bacillota;Tissierellia;Tissierellia\_Incertae\_sedis;Tissierellia\_Incertae\_sedis;Ezakiella |
| Bacteroides | 2 | 3 | 1 | 9 | 0 | 6 | 0 | 3 | 0 | 1 | 7 | 1 | 8 | 41 | Bacteria | Bacteria\_none | Bacteroidota | Bacteroidia | Bacteroidales | Bacteroidaceae | Bacteria;Bacteria\_none;Bacteroidota;Bacteroidia;Bacteroidales;Bacteroidaceae;Bacteroides |
| Phocaeicola | 0 | 0 | 0 | 1 | 1 | 1 | 0 | 0 | 0 | 0 | 1 | 0 | 2 | 6 | Bacteria | Bacteria\_none | Bacteroidota | Bacteroidia | Bacteroidales | Bacteroidaceae | Bacteria;Bacteria\_none;Bacteroidota;Bacteroidia;Bacteroidales;Bacteroidaceae;Phocaeicola |
| Barnesiella | 0 | 0 | 0 | 0 | 0 | 1 | 0 | 0 | 0 | 0 | 0 | 0 | 0 | 1 | Bacteria | Bacteria\_none | Bacteroidota | Bacteroidia | Bacteroidales | Barnesiellaceae | Bacteria;Bacteria\_none;Bacteroidota;Bacteroidia;Bacteroidales;Barnesiellaceae;Barnesiella |
| Proteiniphilum | 0 | 0 | 0 | 0 | 0 | 0 | 0 | 0 | 0 | 0 | 0 | 1 | 0 | 1 | Bacteria | Bacteria\_none | Bacteroidota | Bacteroidia | Bacteroidales | Dysgonomonadaceae | Bacteria;Bacteria\_none;Bacteroidota;Bacteroidia;Bacteroidales;Dysgonomonadaceae;Proteiniphilum |
| Duncaniella | 0 | 0 | 0 | 0 | 0 | 0 | 0 | 0 | 0 | 0 | 1 | 0 | 0 | 1 | Bacteria | Bacteria\_none | Bacteroidota | Bacteroidia | Bacteroidales | Muribaculaceae | Bacteria;Bacteria\_none;Bacteroidota;Bacteroidia;Bacteroidales;Muribaculaceae;Duncaniella |
| Sodaliphilus | 0 | 0 | 0 | 0 | 1 | 0 | 0 | 0 | 0 | 0 | 0 | 0 | 1 | 2 | Bacteria | Bacteria\_none | Bacteroidota | Bacteroidia | Bacteroidales | Muribaculaceae | Bacteria;Bacteria\_none;Bacteroidota;Bacteroidia;Bacteroidales;Muribaculaceae;Sodaliphilus |
| Butyricimonas | 0 | 0 | 0 | 0 | 0 | 0 | 0 | 0 | 0 | 0 | 0 | 1 | 0 | 1 | Bacteria | Bacteria\_none | Bacteroidota | Bacteroidia | Bacteroidales | Odoribacteraceae | Bacteria;Bacteria\_none;Bacteroidota;Bacteroidia;Bacteroidales;Odoribacteraceae;Butyricimonas |
| Odoribacter | 0 | 0 | 0 | 0 | 0 | 0 | 0 | 0 | 0 | 0 | 0 | 0 | 1 | 1 | Bacteria | Bacteria\_none | Bacteroidota | Bacteroidia | Bacteroidales | Odoribacteraceae | Bacteria;Bacteria\_none;Bacteroidota;Bacteroidia;Bacteroidales;Odoribacteraceae;Odoribacter |
| Porphyromonas | 5 | 129 | 9 | 52 | 63 | 32 | 13 | 105 | 0 | 52 | 91 | 45 | 103 | 699 | Bacteria | Bacteria\_none | Bacteroidota | Bacteroidia | Bacteroidales | Porphyromonadaceae | Bacteria;Bacteria\_none;Bacteroidota;Bacteroidia;Bacteroidales;Porphyromonadaceae;Porphyromonas |
| Hoylesella | 1 | 4 | 3 | 5 | 1 | 5 | 1 | 10 | 1 | 3 | 11 | 6 | 3 | 54 | Bacteria | Bacteria\_none | Bacteroidota | Bacteroidia | Bacteroidales | Prevotellaceae | Bacteria;Bacteria\_none;Bacteroidota;Bacteroidia;Bacteroidales;Prevotellaceae;Hoylesella |
| Prevotella | 334 | 971 | 271 | 874 | 78 | 231 | 190 | 36 | 340 | 125 | 171 | 303 | 1010 | 4934 | Bacteria | Bacteria\_none | Bacteroidota | Bacteroidia | Bacteroidales | Prevotellaceae | Bacteria;Bacteria\_none;Bacteroidota;Bacteroidia;Bacteroidales;Prevotellaceae;Prevotella |
| Segatella | 1 | 6 | 13 | 12 | 6 | 4 | 12 | 16 | 0 | 14 | 46 | 38 | 29 | 197 | Bacteria | Bacteria\_none | Bacteroidota | Bacteroidia | Bacteroidales | Prevotellaceae | Bacteria;Bacteria\_none;Bacteroidota;Bacteroidia;Bacteroidales;Prevotellaceae;Segatella |
| Xylanibacter | 0 | 0 | 0 | 0 | 0 | 0 | 1 | 0 | 0 | 0 | 0 | 0 | 1 | 2 | Bacteria | Bacteria\_none | Bacteroidota | Bacteroidia | Bacteroidales | Prevotellaceae | Bacteria;Bacteria\_none;Bacteroidota;Bacteroidia;Bacteroidales;Prevotellaceae;Xylanibacter |
| Alistipes | 0 | 2 | 0 | 0 | 0 | 2 | 0 | 0 | 0 | 1 | 0 | 0 | 1 | 6 | Bacteria | Bacteria\_none | Bacteroidota | Bacteroidia | Bacteroidales | Rikenellaceae | Bacteria;Bacteria\_none;Bacteroidota;Bacteroidia;Bacteroidales;Rikenellaceae;Alistipes |
| Salinivirga | 0 | 0 | 0 | 1 | 0 | 0 | 0 | 0 | 0 | 0 | 0 | 0 | 1 | 2 | Bacteria | Bacteria\_none | Bacteroidota | Bacteroidia | Bacteroidales | Salinivirgaceae | Bacteria;Bacteria\_none;Bacteroidota;Bacteroidia;Bacteroidales;Salinivirgaceae;Salinivirga |
| Parabacteroides | 0 | 0 | 0 | 0 | 0 | 0 | 0 | 1 | 0 | 0 | 1 | 0 | 2 | 4 | Bacteria | Bacteria\_none | Bacteroidota | Bacteroidia | Bacteroidales | Tannerellaceae | Bacteria;Bacteria\_none;Bacteroidota;Bacteroidia;Bacteroidales;Tannerellaceae;Parabacteroides |
| Tannerella | 2 | 37 | 34 | 13 | 19 | 103 | 5 | 53 | 0 | 44 | 46 | 24 | 100 | 480 | Bacteria | Bacteria\_none | Bacteroidota | Bacteroidia | Bacteroidales | Tannerellaceae | Bacteria;Bacteria\_none;Bacteroidota;Bacteroidia;Bacteroidales;Tannerellaceae;Tannerella |
| Marinifilum | 1 | 0 | 1 | 0 | 0 | 0 | 0 | 0 | 0 | 0 | 0 | 0 | 0 | 2 | Bacteria | Bacteria\_none | Bacteroidota | Bacteroidia | Marinilabiliales | Marinifilaceae | Bacteria;Bacteria\_none;Bacteroidota;Bacteroidia;Marinilabiliales;Marinifilaceae;Marinifilum |
| Alkalitalea | 0 | 0 | 0 | 0 | 0 | 0 | 0 | 1 | 0 | 0 | 0 | 0 | 0 | 1 | Bacteria | Bacteria\_none | Bacteroidota | Bacteroidia | Marinilabiliales | Marinilabiliaceae | Bacteria;Bacteria\_none;Bacteroidota;Bacteroidia;Marinilabiliales;Marinilabiliaceae;Alkalitalea |
| Carboxylicivirga | 0 | 0 | 1 | 1 | 0 | 0 | 0 | 1 | 0 | 0 | 0 | 0 | 0 | 3 | Bacteria | Bacteria\_none | Bacteroidota | Bacteroidia | Marinilabiliales | Marinilabiliaceae | Bacteria;Bacteria\_none;Bacteroidota;Bacteroidia;Marinilabiliales;Marinilabiliaceae;Carboxylicivirga |
| Draconibacterium | 0 | 0 | 0 | 1 | 0 | 0 | 0 | 0 | 0 | 0 | 2 | 0 | 0 | 3 | Bacteria | Bacteria\_none | Bacteroidota | Bacteroidia | Marinilabiliales | Prolixibacteraceae | Bacteria;Bacteria\_none;Bacteroidota;Bacteroidia;Marinilabiliales;Prolixibacteraceae;Draconibacterium |
| Mangrovibacterium | 0 | 0 | 0 | 0 | 0 | 1 | 0 | 0 | 0 | 0 | 0 | 0 | 0 | 1 | Bacteria | Bacteria\_none | Bacteroidota | Bacteroidia | Marinilabiliales | Prolixibacteraceae | Bacteria;Bacteria\_none;Bacteroidota;Bacteroidia;Marinilabiliales;Prolixibacteraceae;Mangrovibacterium |
| Maribellus | 0 | 0 | 0 | 1 | 0 | 0 | 0 | 0 | 0 | 0 | 0 | 0 | 0 | 1 | Bacteria | Bacteria\_none | Bacteroidota | Bacteroidia | Marinilabiliales | Prolixibacteraceae | Bacteria;Bacteria\_none;Bacteroidota;Bacteroidia;Marinilabiliales;Prolixibacteraceae;Maribellus |
| Chitinophaga | 0 | 1 | 0 | 1 | 2 | 0 | 0 | 0 | 0 | 1 | 0 | 0 | 0 | 5 | Bacteria | Bacteria\_none | Bacteroidota | Chitinophagia | Chitinophagales | Chitinophagaceae | Bacteria;Bacteria\_none;Bacteroidota;Chitinophagia;Chitinophagales;Chitinophagaceae;Chitinophaga |
| Ferruginibacter | 0 | 0 | 0 | 0 | 0 | 1 | 0 | 0 | 0 | 0 | 0 | 0 | 0 | 1 | Bacteria | Bacteria\_none | Bacteroidota | Chitinophagia | Chitinophagales | Chitinophagaceae | Bacteria;Bacteria\_none;Bacteroidota;Chitinophagia;Chitinophagales;Chitinophagaceae;Ferruginibacter |
| Flavihumibacter | 0 | 0 | 0 | 1 | 0 | 0 | 0 | 0 | 0 | 0 | 0 | 0 | 0 | 1 | Bacteria | Bacteria\_none | Bacteroidota | Chitinophagia | Chitinophagales | Chitinophagaceae | Bacteria;Bacteria\_none;Bacteroidota;Chitinophagia;Chitinophagales;Chitinophagaceae;Flavihumibacter |
| Pseudobacter | 0 | 0 | 0 | 0 | 0 | 0 | 0 | 0 | 0 | 0 | 0 | 1 | 0 | 1 | Bacteria | Bacteria\_none | Bacteroidota | Chitinophagia | Chitinophagales | Chitinophagaceae | Bacteria;Bacteria\_none;Bacteroidota;Chitinophagia;Chitinophagales;Chitinophagaceae;Pseudobacter |
| Candidatus Cardinium | 0 | 0 | 0 | 0 | 0 | 0 | 0 | 0 | 0 | 0 | 1 | 0 | 0 | 1 | Bacteria | Bacteria\_none | Bacteroidota | Cytophagia | Cytophagales | Amoebophilaceae | Bacteria;Bacteria\_none;Bacteroidota;Cytophagia;Cytophagales;Amoebophilaceae;Candidatus Cardinium |
| Algoriphagus | 0 | 0 | 0 | 0 | 0 | 0 | 0 | 0 | 0 | 0 | 1 | 0 | 0 | 1 | Bacteria | Bacteria\_none | Bacteroidota | Cytophagia | Cytophagales | Cyclobacteriaceae | Bacteria;Bacteria\_none;Bacteroidota;Cytophagia;Cytophagales;Cyclobacteriaceae;Algoriphagus |
| Cyclobacterium | 0 | 0 | 0 | 1 | 0 | 0 | 0 | 0 | 0 | 0 | 0 | 0 | 1 | 2 | Bacteria | Bacteria\_none | Bacteroidota | Cytophagia | Cytophagales | Cyclobacteriaceae | Bacteria;Bacteria\_none;Bacteroidota;Cytophagia;Cytophagales;Cyclobacteriaceae;Cyclobacterium |
| Spirosoma | 0 | 0 | 0 | 0 | 0 | 0 | 0 | 1 | 0 | 0 | 0 | 0 | 0 | 1 | Bacteria | Bacteria\_none | Bacteroidota | Cytophagia | Cytophagales | Cytophagaceae | Bacteria;Bacteria\_none;Bacteroidota;Cytophagia;Cytophagales;Cytophagaceae;Spirosoma |
| Flammeovirga | 0 | 0 | 0 | 0 | 2 | 0 | 0 | 0 | 0 | 0 | 0 | 0 | 0 | 2 | Bacteria | Bacteria\_none | Bacteroidota | Cytophagia | Cytophagales | Flammeovirgaceae | Bacteria;Bacteria\_none;Bacteroidota;Cytophagia;Cytophagales;Flammeovirgaceae;Flammeovirga |
| Hymenobacter | 0 | 0 | 0 | 1 | 2 | 0 | 1 | 0 | 0 | 0 | 0 | 0 | 3 | 7 | Bacteria | Bacteria\_none | Bacteroidota | Cytophagia | Cytophagales | Hymenobacteraceae | Bacteria;Bacteria\_none;Bacteroidota;Cytophagia;Cytophagales;Hymenobacteraceae;Hymenobacter |
| Rufibacter | 0 | 0 | 0 | 0 | 0 | 0 | 0 | 0 | 0 | 1 | 0 | 0 | 0 | 1 | Bacteria | Bacteria\_none | Bacteroidota | Cytophagia | Cytophagales | Hymenobacteraceae | Bacteria;Bacteria\_none;Bacteroidota;Cytophagia;Cytophagales;Hymenobacteraceae;Rufibacter |
| Arcticibacterium | 0 | 0 | 1 | 0 | 0 | 0 | 0 | 0 | 0 | 0 | 0 | 0 | 0 | 1 | Bacteria | Bacteria\_none | Bacteroidota | Cytophagia | Cytophagales | Leadbetterellaceae | Bacteria;Bacteria\_none;Bacteroidota;Cytophagia;Cytophagales;Leadbetterellaceae;Arcticibacterium |
| Emticicia | 1 | 0 | 0 | 0 | 0 | 0 | 0 | 0 | 0 | 0 | 0 | 0 | 0 | 1 | Bacteria | Bacteria\_none | Bacteroidota | Cytophagia | Cytophagales | Leadbetterellaceae | Bacteria;Bacteria\_none;Bacteroidota;Cytophagia;Cytophagales;Leadbetterellaceae;Emticicia |
| Marivirga | 0 | 0 | 0 | 0 | 0 | 1 | 0 | 0 | 0 | 0 | 0 | 0 | 1 | 2 | Bacteria | Bacteria\_none | Bacteroidota | Cytophagia | Cytophagales | Marivirgaceae | Bacteria;Bacteria\_none;Bacteroidota;Cytophagia;Cytophagales;Marivirgaceae;Marivirga |
| Fulvitalea | 0 | 0 | 0 | 1 | 1 | 0 | 0 | 0 | 0 | 0 | 0 | 0 | 0 | 2 | Bacteria | Bacteria\_none | Bacteroidota | Cytophagia | Cytophagales | Persicobacteraceae | Bacteria;Bacteria\_none;Bacteroidota;Cytophagia;Cytophagales;Persicobacteraceae;Fulvitalea |
| Dyadobacter | 0 | 0 | 0 | 0 | 0 | 1 | 0 | 0 | 0 | 0 | 0 | 0 | 2 | 3 | Bacteria | Bacteria\_none | Bacteroidota | Cytophagia | Cytophagales | Spirosomataceae | Bacteria;Bacteria\_none;Bacteroidota;Cytophagia;Cytophagales;Spirosomataceae;Dyadobacter |
| Fibrella | 0 | 0 | 0 | 0 | 0 | 0 | 0 | 0 | 0 | 0 | 0 | 0 | 1 | 1 | Bacteria | Bacteria\_none | Bacteroidota | Cytophagia | Cytophagales | Spirosomataceae | Bacteria;Bacteria\_none;Bacteroidota;Cytophagia;Cytophagales;Spirosomataceae;Fibrella |
| Blattabacterium | 0 | 0 | 0 | 0 | 0 | 0 | 0 | 0 | 0 | 0 | 1 | 0 | 1 | 2 | Bacteria | Bacteria\_none | Bacteroidota | Flavobacteriia | Flavobacteriales | Blattabacteriaceae | Bacteria;Bacteria\_none;Bacteroidota;Flavobacteriia;Flavobacteriales;Blattabacteriaceae;Blattabacterium |
| Paracrocinitomix | 0 | 0 | 0 | 0 | 0 | 0 | 0 | 0 | 0 | 1 | 0 | 0 | 0 | 1 | Bacteria | Bacteria\_none | Bacteroidota | Flavobacteriia | Flavobacteriales | Crocinitomicaceae | Bacteria;Bacteria\_none;Bacteroidota;Flavobacteriia;Flavobacteriales;Crocinitomicaceae;Paracrocinitomix |
| Aequorivita | 0 | 0 | 0 | 2 | 0 | 0 | 0 | 0 | 0 | 0 | 0 | 0 | 0 | 2 | Bacteria | Bacteria\_none | Bacteroidota | Flavobacteriia | Flavobacteriales | Flavobacteriaceae | Bacteria;Bacteria\_none;Bacteroidota;Flavobacteriia;Flavobacteriales;Flavobacteriaceae;Aequorivita |
| Algibacter | 0 | 0 | 0 | 0 | 0 | 1 | 0 | 0 | 0 | 0 | 0 | 1 | 0 | 2 | Bacteria | Bacteria\_none | Bacteroidota | Flavobacteriia | Flavobacteriales | Flavobacteriaceae | Bacteria;Bacteria\_none;Bacteroidota;Flavobacteriia;Flavobacteriales;Flavobacteriaceae;Algibacter |
| Aquimarina | 0 | 0 | 0 | 0 | 1 | 0 | 0 | 1 | 0 | 0 | 0 | 0 | 0 | 2 | Bacteria | Bacteria\_none | Bacteroidota | Flavobacteriia | Flavobacteriales | Flavobacteriaceae | Bacteria;Bacteria\_none;Bacteroidota;Flavobacteriia;Flavobacteriales;Flavobacteriaceae;Aquimarina |
| Capnocytophaga | 311 | 146 | 128 | 204 | 157 | 30 | 82 | 376 | 0 | 352 | 209 | 52 | 561 | 2608 | Bacteria | Bacteria\_none | Bacteroidota | Flavobacteriia | Flavobacteriales | Flavobacteriaceae | Bacteria;Bacteria\_none;Bacteroidota;Flavobacteriia;Flavobacteriales;Flavobacteriaceae;Capnocytophaga |
| Christiangramia | 0 | 0 | 0 | 0 | 0 | 0 | 0 | 0 | 0 | 0 | 1 | 0 | 1 | 2 | Bacteria | Bacteria\_none | Bacteroidota | Flavobacteriia | Flavobacteriales | Flavobacteriaceae | Bacteria;Bacteria\_none;Bacteroidota;Flavobacteriia;Flavobacteriales;Flavobacteriaceae;Christiangramia |
| Costertonia | 0 | 0 | 0 | 0 | 0 | 0 | 0 | 0 | 0 | 0 | 1 | 0 | 0 | 1 | Bacteria | Bacteria\_none | Bacteroidota | Flavobacteriia | Flavobacteriales | Flavobacteriaceae | Bacteria;Bacteria\_none;Bacteroidota;Flavobacteriia;Flavobacteriales;Flavobacteriaceae;Costertonia |
| Dokdonia | 0 | 1 | 0 | 0 | 0 | 1 | 0 | 0 | 0 | 0 | 0 | 0 | 0 | 2 | Bacteria | Bacteria\_none | Bacteroidota | Flavobacteriia | Flavobacteriales | Flavobacteriaceae | Bacteria;Bacteria\_none;Bacteroidota;Flavobacteriia;Flavobacteriales;Flavobacteriaceae;Dokdonia |
| Euzebyella | 0 | 0 | 0 | 0 | 0 | 0 | 0 | 0 | 0 | 0 | 0 | 0 | 1 | 1 | Bacteria | Bacteria\_none | Bacteroidota | Flavobacteriia | Flavobacteriales | Flavobacteriaceae | Bacteria;Bacteria\_none;Bacteroidota;Flavobacteriia;Flavobacteriales;Flavobacteriaceae;Euzebyella |
| Flagellimonas | 0 | 0 | 0 | 0 | 0 | 0 | 0 | 1 | 0 | 0 | 1 | 0 | 0 | 2 | Bacteria | Bacteria\_none | Bacteroidota | Flavobacteriia | Flavobacteriales | Flavobacteriaceae | Bacteria;Bacteria\_none;Bacteroidota;Flavobacteriia;Flavobacteriales;Flavobacteriaceae;Flagellimonas |
| Flavobacterium | 2 | 2 | 0 | 4 | 5 | 2 | 1 | 0 | 0 | 2 | 2 | 2 | 3 | 25 | Bacteria | Bacteria\_none | Bacteroidota | Flavobacteriia | Flavobacteriales | Flavobacteriaceae | Bacteria;Bacteria\_none;Bacteroidota;Flavobacteriia;Flavobacteriales;Flavobacteriaceae;Flavobacterium |
| Formosa | 0 | 0 | 0 | 0 | 1 | 1 | 0 | 0 | 0 | 0 | 0 | 0 | 2 | 4 | Bacteria | Bacteria\_none | Bacteroidota | Flavobacteriia | Flavobacteriales | Flavobacteriaceae | Bacteria;Bacteria\_none;Bacteroidota;Flavobacteriia;Flavobacteriales;Flavobacteriaceae;Formosa |
| Kordia | 0 | 0 | 0 | 1 | 0 | 0 | 0 | 0 | 0 | 0 | 0 | 0 | 2 | 3 | Bacteria | Bacteria\_none | Bacteroidota | Flavobacteriia | Flavobacteriales | Flavobacteriaceae | Bacteria;Bacteria\_none;Bacteroidota;Flavobacteriia;Flavobacteriales;Flavobacteriaceae;Kordia |
| Lacinutrix | 0 | 0 | 0 | 1 | 0 | 0 | 0 | 0 | 0 | 0 | 0 | 0 | 3 | 4 | Bacteria | Bacteria\_none | Bacteroidota | Flavobacteriia | Flavobacteriales | Flavobacteriaceae | Bacteria;Bacteria\_none;Bacteroidota;Flavobacteriia;Flavobacteriales;Flavobacteriaceae;Lacinutrix |
| Leeuwenhoekiella | 0 | 0 | 0 | 0 | 1 | 0 | 0 | 0 | 0 | 0 | 0 | 0 | 0 | 1 | Bacteria | Bacteria\_none | Bacteroidota | Flavobacteriia | Flavobacteriales | Flavobacteriaceae | Bacteria;Bacteria\_none;Bacteroidota;Flavobacteriia;Flavobacteriales;Flavobacteriaceae;Leeuwenhoekiella |
| Lutibacter | 0 | 0 | 0 | 0 | 0 | 0 | 0 | 0 | 0 | 0 | 0 | 0 | 1 | 1 | Bacteria | Bacteria\_none | Bacteroidota | Flavobacteriia | Flavobacteriales | Flavobacteriaceae | Bacteria;Bacteria\_none;Bacteroidota;Flavobacteriia;Flavobacteriales;Flavobacteriaceae;Lutibacter |
| Mangrovimonas | 0 | 0 | 0 | 0 | 0 | 0 | 0 | 0 | 0 | 1 | 0 | 0 | 0 | 1 | Bacteria | Bacteria\_none | Bacteroidota | Flavobacteriia | Flavobacteriales | Flavobacteriaceae | Bacteria;Bacteria\_none;Bacteroidota;Flavobacteriia;Flavobacteriales;Flavobacteriaceae;Mangrovimonas |
| Maribacter | 0 | 0 | 0 | 0 | 1 | 0 | 0 | 0 | 0 | 0 | 0 | 0 | 1 | 2 | Bacteria | Bacteria\_none | Bacteroidota | Flavobacteriia | Flavobacteriales | Flavobacteriaceae | Bacteria;Bacteria\_none;Bacteroidota;Flavobacteriia;Flavobacteriales;Flavobacteriaceae;Maribacter |
| Myroides | 0 | 0 | 0 | 0 | 1 | 0 | 0 | 0 | 0 | 1 | 0 | 0 | 0 | 2 | Bacteria | Bacteria\_none | Bacteroidota | Flavobacteriia | Flavobacteriales | Flavobacteriaceae | Bacteria;Bacteria\_none;Bacteroidota;Flavobacteriia;Flavobacteriales;Flavobacteriaceae;Myroides |
| Nonlabens | 0 | 0 | 0 | 0 | 0 | 0 | 0 | 0 | 0 | 0 | 0 | 0 | 1 | 1 | Bacteria | Bacteria\_none | Bacteroidota | Flavobacteriia | Flavobacteriales | Flavobacteriaceae | Bacteria;Bacteria\_none;Bacteroidota;Flavobacteriia;Flavobacteriales;Flavobacteriaceae;Nonlabens |
| Polaribacter | 0 | 0 | 1 | 2 | 1 | 1 | 1 | 0 | 0 | 0 | 1 | 0 | 1 | 8 | Bacteria | Bacteria\_none | Bacteroidota | Flavobacteriia | Flavobacteriales | Flavobacteriaceae | Bacteria;Bacteria\_none;Bacteroidota;Flavobacteriia;Flavobacteriales;Flavobacteriaceae;Polaribacter |
| Psychroflexus | 0 | 0 | 0 | 0 | 0 | 0 | 0 | 0 | 0 | 0 | 1 | 0 | 0 | 1 | Bacteria | Bacteria\_none | Bacteroidota | Flavobacteriia | Flavobacteriales | Flavobacteriaceae | Bacteria;Bacteria\_none;Bacteroidota;Flavobacteriia;Flavobacteriales;Flavobacteriaceae;Psychroflexus |
| Rasiella | 0 | 0 | 0 | 0 | 0 | 0 | 0 | 0 | 0 | 0 | 1 | 0 | 0 | 1 | Bacteria | Bacteria\_none | Bacteroidota | Flavobacteriia | Flavobacteriales | Flavobacteriaceae | Bacteria;Bacteria\_none;Bacteroidota;Flavobacteriia;Flavobacteriales;Flavobacteriaceae;Rasiella |
| Salegentibacter | 0 | 1 | 0 | 0 | 0 | 0 | 0 | 0 | 0 | 0 | 0 | 0 | 0 | 1 | Bacteria | Bacteria\_none | Bacteroidota | Flavobacteriia | Flavobacteriales | Flavobacteriaceae | Bacteria;Bacteria\_none;Bacteroidota;Flavobacteriia;Flavobacteriales;Flavobacteriaceae;Salegentibacter |
| Tenacibaculum | 0 | 0 | 1 | 0 | 2 | 0 | 0 | 0 | 0 | 0 | 1 | 0 | 0 | 4 | Bacteria | Bacteria\_none | Bacteroidota | Flavobacteriia | Flavobacteriales | Flavobacteriaceae | Bacteria;Bacteria\_none;Bacteroidota;Flavobacteriia;Flavobacteriales;Flavobacteriaceae;Tenacibaculum |
| Zobellia | 0 | 0 | 0 | 0 | 1 | 0 | 0 | 0 | 0 | 0 | 0 | 0 | 0 | 1 | Bacteria | Bacteria\_none | Bacteroidota | Flavobacteriia | Flavobacteriales | Flavobacteriaceae | Bacteria;Bacteria\_none;Bacteroidota;Flavobacteriia;Flavobacteriales;Flavobacteriaceae;Zobellia |
| Croceimicrobium | 0 | 1 | 0 | 0 | 1 | 0 | 0 | 0 | 0 | 0 | 0 | 0 | 0 | 2 | Bacteria | Bacteria\_none | Bacteroidota | Flavobacteriia | Flavobacteriales | Owenweeksiaceae | Bacteria;Bacteria\_none;Bacteroidota;Flavobacteriia;Flavobacteriales;Owenweeksiaceae;Croceimicrobium |
| Sanyastnella | 0 | 0 | 1 | 0 | 0 | 0 | 0 | 0 | 0 | 0 | 0 | 0 | 0 | 1 | Bacteria | Bacteria\_none | Bacteroidota | Flavobacteriia | Flavobacteriales | Sanyastnellaceae | Bacteria;Bacteria\_none;Bacteroidota;Flavobacteriia;Flavobacteriales;Sanyastnellaceae;Sanyastnella |
| Apibacter | 0 | 0 | 0 | 0 | 1 | 0 | 0 | 0 | 0 | 0 | 0 | 0 | 0 | 1 | Bacteria | Bacteria\_none | Bacteroidota | Flavobacteriia | Flavobacteriales | Weeksellaceae | Bacteria;Bacteria\_none;Bacteroidota;Flavobacteriia;Flavobacteriales;Weeksellaceae;Apibacter |
| Bergeyella | 0 | 0 | 1 | 0 | 2 | 0 | 0 | 0 | 0 | 0 | 1 | 0 | 0 | 4 | Bacteria | Bacteria\_none | Bacteroidota | Flavobacteriia | Flavobacteriales | Weeksellaceae | Bacteria;Bacteria\_none;Bacteroidota;Flavobacteriia;Flavobacteriales;Weeksellaceae;Bergeyella |
| Chryseobacterium | 2 | 0 | 2 | 2 | 6 | 2 | 0 | 2 | 0 | 5 | 1 | 1 | 2 | 25 | Bacteria | Bacteria\_none | Bacteroidota | Flavobacteriia | Flavobacteriales | Weeksellaceae | Bacteria;Bacteria\_none;Bacteroidota;Flavobacteriia;Flavobacteriales;Weeksellaceae;Chryseobacterium |
| Cloacibacterium | 0 | 0 | 0 | 0 | 1 | 0 | 0 | 0 | 0 | 0 | 1 | 0 | 0 | 2 | Bacteria | Bacteria\_none | Bacteroidota | Flavobacteriia | Flavobacteriales | Weeksellaceae | Bacteria;Bacteria\_none;Bacteroidota;Flavobacteriia;Flavobacteriales;Weeksellaceae;Cloacibacterium |
| Elizabethkingia | 0 | 1 | 1 | 0 | 2 | 1 | 0 | 1 | 0 | 0 | 0 | 0 | 0 | 6 | Bacteria | Bacteria\_none | Bacteroidota | Flavobacteriia | Flavobacteriales | Weeksellaceae | Bacteria;Bacteria\_none;Bacteroidota;Flavobacteriia;Flavobacteriales;Weeksellaceae;Elizabethkingia |
| Empedobacter | 1 | 0 | 0 | 1 | 0 | 0 | 0 | 0 | 0 | 0 | 0 | 0 | 2 | 4 | Bacteria | Bacteria\_none | Bacteroidota | Flavobacteriia | Flavobacteriales | Weeksellaceae | Bacteria;Bacteria\_none;Bacteroidota;Flavobacteriia;Flavobacteriales;Weeksellaceae;Empedobacter |
| Epilithonimonas | 0 | 0 | 0 | 0 | 1 | 1 | 0 | 0 | 0 | 0 | 0 | 0 | 0 | 2 | Bacteria | Bacteria\_none | Bacteroidota | Flavobacteriia | Flavobacteriales | Weeksellaceae | Bacteria;Bacteria\_none;Bacteroidota;Flavobacteriia;Flavobacteriales;Weeksellaceae;Epilithonimonas |
| Ornithobacterium | 1 | 0 | 0 | 0 | 0 | 0 | 0 | 1 | 0 | 0 | 0 | 0 | 0 | 2 | Bacteria | Bacteria\_none | Bacteroidota | Flavobacteriia | Flavobacteriales | Weeksellaceae | Bacteria;Bacteria\_none;Bacteroidota;Flavobacteriia;Flavobacteriales;Weeksellaceae;Ornithobacterium |
| Riemerella | 0 | 0 | 0 | 3 | 1 | 1 | 0 | 0 | 0 | 1 | 0 | 0 | 0 | 6 | Bacteria | Bacteria\_none | Bacteroidota | Flavobacteriia | Flavobacteriales | Weeksellaceae | Bacteria;Bacteria\_none;Bacteroidota;Flavobacteriia;Flavobacteriales;Weeksellaceae;Riemerella |
| Haliscomenobacter | 0 | 0 | 0 | 0 | 0 | 0 | 0 | 0 | 0 | 0 | 0 | 0 | 1 | 1 | Bacteria | Bacteria\_none | Bacteroidota | Saprospiria | Saprospirales | Haliscomenobacteraceae | Bacteria;Bacteria\_none;Bacteroidota;Saprospiria;Saprospirales;Haliscomenobacteraceae;Haliscomenobacter |
| Aurantibacillus | 0 | 0 | 0 | 1 | 0 | 0 | 0 | 0 | 0 | 0 | 0 | 0 | 0 | 1 | Bacteria | Bacteria\_none | Bacteroidota | Sphingobacteriia | Sphingobacteriales | Aurantibacillaceae | Bacteria;Bacteria\_none;Bacteroidota;Sphingobacteriia;Sphingobacteriales;Aurantibacillaceae;Aurantibacillus |
| Mucilaginibacter | 0 | 2 | 0 | 2 | 0 | 0 | 0 | 0 | 0 | 0 | 0 | 1 | 0 | 5 | Bacteria | Bacteria\_none | Bacteroidota | Sphingobacteriia | Sphingobacteriales | Sphingobacteriaceae | Bacteria;Bacteria\_none;Bacteroidota;Sphingobacteriia;Sphingobacteriales;Sphingobacteriaceae;Mucilaginibacter |
| Olivibacter | 0 | 0 | 0 | 1 | 0 | 0 | 0 | 0 | 0 | 0 | 0 | 0 | 0 | 1 | Bacteria | Bacteria\_none | Bacteroidota | Sphingobacteriia | Sphingobacteriales | Sphingobacteriaceae | Bacteria;Bacteria\_none;Bacteroidota;Sphingobacteriia;Sphingobacteriales;Sphingobacteriaceae;Olivibacter |
| Pedobacter | 0 | 0 | 0 | 0 | 0 | 1 | 0 | 1 | 0 | 0 | 2 | 0 | 0 | 4 | Bacteria | Bacteria\_none | Bacteroidota | Sphingobacteriia | Sphingobacteriales | Sphingobacteriaceae | Bacteria;Bacteria\_none;Bacteroidota;Sphingobacteriia;Sphingobacteriales;Sphingobacteriaceae;Pedobacter |
| Solitalea | 0 | 0 | 0 | 0 | 0 | 0 | 0 | 0 | 0 | 0 | 0 | 0 | 1 | 1 | Bacteria | Bacteria\_none | Bacteroidota | Sphingobacteriia | Sphingobacteriales | Sphingobacteriaceae | Bacteria;Bacteria\_none;Bacteroidota;Sphingobacteriia;Sphingobacteriales;Sphingobacteriaceae;Solitalea |
| Sphingobacterium | 0 | 0 | 0 | 0 | 1 | 0 | 0 | 0 | 0 | 1 | 1 | 0 | 1 | 4 | Bacteria | Bacteria\_none | Bacteroidota | Sphingobacteriia | Sphingobacteriales | Sphingobacteriaceae | Bacteria;Bacteria\_none;Bacteroidota;Sphingobacteriia;Sphingobacteriales;Sphingobacteriaceae;Sphingobacterium |
| Cyclonatronum | 0 | 0 | 0 | 1 | 0 | 0 | 0 | 0 | 0 | 0 | 0 | 0 | 0 | 1 | Bacteria | Bacteria\_none | Balneolota | Balneolia | Balneolales | Cyclonatronaceae | Bacteria;Bacteria\_none;Balneolota;Balneolia;Balneolales;Cyclonatronaceae;Cyclonatronum |
| Bdellovibrio | 0 | 0 | 0 | 0 | 0 | 0 | 0 | 0 | 0 | 1 | 0 | 0 | 0 | 1 | Bacteria | Bacteria\_none | Bdellovibrionota | Bdellovibrionia | Bdellovibrionales | Pseudobdellovibrionaceae | Bacteria;Bacteria\_none;Bdellovibrionota;Bdellovibrionia;Bdellovibrionales;Pseudobdellovibrionaceae;Bdellovibrio |
| Caldithrix | 0 | 0 | 0 | 1 | 0 | 0 | 0 | 0 | 0 | 0 | 0 | 0 | 0 | 1 | Bacteria | Bacteria\_none | Calditrichota | Calditrichia | Calditrichales | Calditrichaceae | Bacteria;Bacteria\_none;Calditrichota;Calditrichia;Calditrichales;Calditrichaceae;Caldithrix |
| Aliarcobacter | 0 | 0 | 0 | 0 | 0 | 0 | 0 | 0 | 0 | 0 | 0 | 0 | 2 | 2 | Bacteria | Bacteria\_none | Campylobacterota | Epsilonproteobacteria | Campylobacterales | Arcobacteraceae | Bacteria;Bacteria\_none;Campylobacterota;Epsilonproteobacteria;Campylobacterales;Arcobacteraceae;Aliarcobacter |
| Arcobacter | 0 | 0 | 2 | 0 | 0 | 0 | 0 | 0 | 0 | 1 | 0 | 0 | 0 | 3 | Bacteria | Bacteria\_none | Campylobacterota | Epsilonproteobacteria | Campylobacterales | Arcobacteraceae | Bacteria;Bacteria\_none;Campylobacterota;Epsilonproteobacteria;Campylobacterales;Arcobacteraceae;Arcobacter |
| Halarcobacter | 0 | 0 | 0 | 0 | 0 | 0 | 0 | 0 | 0 | 1 | 0 | 0 | 0 | 1 | Bacteria | Bacteria\_none | Campylobacterota | Epsilonproteobacteria | Campylobacterales | Arcobacteraceae | Bacteria;Bacteria\_none;Campylobacterota;Epsilonproteobacteria;Campylobacterales;Arcobacteraceae;Halarcobacter |
| Malaciobacter | 0 | 0 | 0 | 0 | 0 | 0 | 0 | 0 | 0 | 0 | 0 | 0 | 1 | 1 | Bacteria | Bacteria\_none | Campylobacterota | Epsilonproteobacteria | Campylobacterales | Arcobacteraceae | Bacteria;Bacteria\_none;Campylobacterota;Epsilonproteobacteria;Campylobacterales;Arcobacteraceae;Malaciobacter |
| Campylobacter | 0 | 47 | 14 | 11 | 10 | 49 | 2 | 11 | 9 | 9 | 3 | 14 | 57 | 236 | Bacteria | Bacteria\_none | Campylobacterota | Epsilonproteobacteria | Campylobacterales | Campylobacteraceae | Bacteria;Bacteria\_none;Campylobacterota;Epsilonproteobacteria;Campylobacterales;Campylobacteraceae;Campylobacter |
| Helicobacter | 0 | 0 | 0 | 0 | 1 | 1 | 0 | 0 | 0 | 0 | 0 | 0 | 1 | 3 | Bacteria | Bacteria\_none | Campylobacterota | Epsilonproteobacteria | Campylobacterales | Helicobacteraceae | Bacteria;Bacteria\_none;Campylobacterota;Epsilonproteobacteria;Campylobacterales;Helicobacteraceae;Helicobacter |
| Sulfurospirillum | 0 | 0 | 0 | 0 | 0 | 0 | 0 | 0 | 0 | 1 | 0 | 0 | 0 | 1 | Bacteria | Bacteria\_none | Campylobacterota | Epsilonproteobacteria | Campylobacterales | Sulfurospirillaceae | Bacteria;Bacteria\_none;Campylobacterota;Epsilonproteobacteria;Campylobacterales;Sulfurospirillaceae;Sulfurospirillum |
| Sulfurovum | 1 | 0 | 0 | 0 | 0 | 0 | 0 | 0 | 0 | 0 | 0 | 0 | 0 | 1 | Bacteria | Bacteria\_none | Campylobacterota | Epsilonproteobacteria | Campylobacterales | Sulfurovaceae | Bacteria;Bacteria\_none;Campylobacterota;Epsilonproteobacteria;Campylobacterales;Sulfurovaceae;Sulfurovum |
| Candidatus Minimicrobia | 0 | 0 | 0 | 0 | 2 | 19 | 0 | 0 | 0 | 0 | 1 | 1 | 5 | 28 | Bacteria | Bacteria\_none | Candidatus Saccharibacteria | Candidatus Saccharibacteria\_Incertae\_sedis | Candidatus Saccharibacteria\_Incertae\_sedis | Candidatus Saccharibacteria\_Incertae\_sedis | Bacteria;Bacteria\_none;Candidatus Saccharibacteria;Candidatus Saccharibacteria\_Incertae\_sedis;Candidatus Saccharibacteria\_Incertae\_sedis;Candidatus Saccharibacteria\_Incertae\_sedis;Candidatus Minimicrobia |
| Candidatus Mycosynbacter | 0 | 0 | 0 | 0 | 0 | 1 | 0 | 0 | 0 | 0 | 0 | 0 | 0 | 1 | Bacteria | Bacteria\_none | Candidatus Saccharibacteria | Candidatus Saccharibacteria\_Incertae\_sedis | Candidatus Saccharibacteria\_Incertae\_sedis | Candidatus Saccharibacteria\_Incertae\_sedis | Bacteria;Bacteria\_none;Candidatus Saccharibacteria;Candidatus Saccharibacteria\_Incertae\_sedis;Candidatus Saccharibacteria\_Incertae\_sedis;Candidatus Saccharibacteria\_Incertae\_sedis;Candidatus Mycosynbacter |
| Candidatus Nanosynbacter | 0 | 8 | 5 | 0 | 10 | 165 | 1 | 1 | 3 | 2 | 92 | 6 | 48 | 341 | Bacteria | Bacteria\_none | Candidatus Saccharibacteria | Candidatus Saccharimonadia | Candidatus Nanosynbacterales | Candidatus Nanosynbacteraceae | Bacteria;Bacteria\_none;Candidatus Saccharibacteria;Candidatus Saccharimonadia;Candidatus Nanosynbacterales;Candidatus Nanosynbacteraceae;Candidatus Nanosynbacter |
| Candidatus Saccharimonas | 0 | 0 | 0 | 0 | 1 | 0 | 0 | 1 | 0 | 0 | 1 | 0 | 0 | 3 | Bacteria | Bacteria\_none | Candidatus Saccharibacteria | Candidatus Saccharimonadia | Candidatus Saccharimonadales | Candidatus Saccharimonadaceae | Bacteria;Bacteria\_none;Candidatus Saccharibacteria;Candidatus Saccharimonadia;Candidatus Saccharimonadales;Candidatus Saccharimonadaceae;Candidatus Saccharimonas |
| Candidatus Protochlamydia | 0 | 0 | 0 | 0 | 0 | 0 | 0 | 0 | 0 | 0 | 0 | 0 | 1 | 1 | Bacteria | Bacteria\_none | Chlamydiota | Chlamydiia | Parachlamydiales | Parachlamydiaceae | Bacteria;Bacteria\_none;Chlamydiota;Chlamydiia;Parachlamydiales;Parachlamydiaceae;Candidatus Protochlamydia |
| Chloroherpeton | 0 | 0 | 0 | 0 | 0 | 1 | 0 | 0 | 0 | 0 | 0 | 0 | 0 | 1 | Bacteria | Bacteria\_none | Chlorobiota | Chlorobiia | Chlorobiales | Chloroherpetonaceae | Bacteria;Bacteria\_none;Chlorobiota;Chlorobiia;Chlorobiales;Chloroherpetonaceae;Chloroherpeton |
| Candidatus Chlorohelix | 0 | 0 | 0 | 0 | 0 | 0 | 0 | 0 | 0 | 0 | 1 | 0 | 0 | 1 | Bacteria | Bacteria\_none | Chloroflexota | Chloroflexia | Candidatus Chloroheliales | Candidatus Chloroheliaceae | Bacteria;Bacteria\_none;Chloroflexota;Chloroflexia;Candidatus Chloroheliales;Candidatus Chloroheliaceae;Candidatus Chlorohelix |
| Candidatus Lucifugimonas | 0 | 0 | 0 | 0 | 0 | 0 | 0 | 0 | 0 | 0 | 0 | 0 | 1 | 1 | Bacteria | Bacteria\_none | Chloroflexota | Dehalococcoidia | Candidatus Lucifugimonadales | Candidatus Lucifugimonadaceae | Bacteria;Bacteria\_none;Chloroflexota;Dehalococcoidia;Candidatus Lucifugimonadales;Candidatus Lucifugimonadaceae;Candidatus Lucifugimonas |
| Cyanobacteriota\_Incertae\_sedis | 1 | 0 | 0 | 0 | 0 | 0 | 0 | 0 | 0 | 0 | 0 | 0 | 0 | 1 | Bacteria | Bacteria\_none | Cyanobacteriota | Cyanobacteriota\_Incertae\_sedis | Cyanobacteriota\_Incertae\_sedis | Cyanobacteriota\_Incertae\_sedis | Bacteria;Bacteria\_none;Cyanobacteriota;Cyanobacteriota\_Incertae\_sedis;Cyanobacteriota\_Incertae\_sedis;Cyanobacteriota\_Incertae\_sedis;Cyanobacteriota\_Incertae\_sedis |
| Gloeocapsa | 0 | 0 | 1 | 0 | 0 | 0 | 0 | 0 | 0 | 0 | 0 | 0 | 0 | 1 | Bacteria | Bacteria\_none | Cyanobacteriota | Cyanophyceae | Chroococcales | Chroococcaceae | Bacteria;Bacteria\_none;Cyanobacteriota;Cyanophyceae;Chroococcales;Chroococcaceae;Gloeocapsa |
| Geminocystis | 0 | 0 | 0 | 0 | 0 | 0 | 0 | 0 | 0 | 0 | 1 | 0 | 0 | 1 | Bacteria | Bacteria\_none | Cyanobacteriota | Cyanophyceae | Chroococcales | Geminocystaceae | Bacteria;Bacteria\_none;Cyanobacteriota;Cyanophyceae;Chroococcales;Geminocystaceae;Geminocystis |
| Gloeobacter | 0 | 0 | 0 | 0 | 0 | 0 | 1 | 0 | 0 | 0 | 0 | 0 | 0 | 1 | Bacteria | Bacteria\_none | Cyanobacteriota | Cyanophyceae | Gloeobacterales | Gloeobacteraceae | Bacteria;Bacteria\_none;Cyanobacteriota;Cyanophyceae;Gloeobacterales;Gloeobacteraceae;Gloeobacter |
| Cylindrospermopsis | 0 | 0 | 0 | 1 | 0 | 0 | 0 | 0 | 0 | 0 | 0 | 0 | 0 | 1 | Bacteria | Bacteria\_none | Cyanobacteriota | Cyanophyceae | Nostocales | Aphanizomenonaceae | Bacteria;Bacteria\_none;Cyanobacteriota;Cyanophyceae;Nostocales;Aphanizomenonaceae;Cylindrospermopsis |
| Nostoc | 0 | 1 | 0 | 0 | 0 | 0 | 0 | 0 | 0 | 2 | 1 | 0 | 2 | 6 | Bacteria | Bacteria\_none | Cyanobacteriota | Cyanophyceae | Nostocales | Nostocaceae | Bacteria;Bacteria\_none;Cyanobacteriota;Cyanophyceae;Nostocales;Nostocaceae;Nostoc |
| Prochlorococcus | 0 | 0 | 0 | 0 | 1 | 0 | 0 | 0 | 0 | 0 | 0 | 0 | 1 | 2 | Bacteria | Bacteria\_none | Cyanobacteriota | Cyanophyceae | Synechococcales | Prochlorococcaceae | Bacteria;Bacteria\_none;Cyanobacteriota;Cyanophyceae;Synechococcales;Prochlorococcaceae;Prochlorococcus |
| Synechococcus | 0 | 0 | 0 | 0 | 0 | 1 | 0 | 0 | 0 | 0 | 0 | 0 | 0 | 1 | Bacteria | Bacteria\_none | Cyanobacteriota | Cyanophyceae | Synechococcales | Synechococcaceae | Bacteria;Bacteria\_none;Cyanobacteriota;Cyanophyceae;Synechococcales;Synechococcaceae;Synechococcus |
| Calditerrivibrio | 0 | 1 | 0 | 0 | 0 | 0 | 0 | 0 | 0 | 0 | 0 | 0 | 0 | 1 | Bacteria | Bacteria\_none | Deferribacterota | Deferribacteres | Deferribacterales | Calditerrivibrionaceae | Bacteria;Bacteria\_none;Deferribacterota;Deferribacteres;Deferribacterales;Calditerrivibrionaceae;Calditerrivibrio |
| Mucispirillum | 0 | 0 | 0 | 0 | 0 | 0 | 0 | 0 | 0 | 0 | 0 | 0 | 1 | 1 | Bacteria | Bacteria\_none | Deferribacterota | Deferribacteres | Deferribacterales | Mucispirillaceae | Bacteria;Bacteria\_none;Deferribacterota;Deferribacteres;Deferribacterales;Mucispirillaceae;Mucispirillum |
| Cetobacterium | 0 | 0 | 0 | 0 | 1 | 0 | 0 | 0 | 0 | 0 | 0 | 0 | 1 | 2 | Bacteria | Bacteria\_none | Fusobacteriota | Fusobacteriia | Fusobacteriales | Fusobacteriaceae | Bacteria;Bacteria\_none;Fusobacteriota;Fusobacteriia;Fusobacteriales;Fusobacteriaceae;Cetobacterium |
| Fusobacterium | 1 | 169 | 61 | 742 | 72 | 27 | 17 | 185 | 0 | 38 | 192 | 106 | 867 | 2477 | Bacteria | Bacteria\_none | Fusobacteriota | Fusobacteriia | Fusobacteriales | Fusobacteriaceae | Bacteria;Bacteria\_none;Fusobacteriota;Fusobacteriia;Fusobacteriales;Fusobacteriaceae;Fusobacterium |
| Ilyobacter | 0 | 0 | 0 | 0 | 1 | 0 | 0 | 0 | 0 | 0 | 0 | 0 | 0 | 1 | Bacteria | Bacteria\_none | Fusobacteriota | Fusobacteriia | Fusobacteriales | Fusobacteriaceae | Bacteria;Bacteria\_none;Fusobacteriota;Fusobacteriia;Fusobacteriales;Fusobacteriaceae;Ilyobacter |
| Haliovirga | 0 | 0 | 0 | 0 | 0 | 0 | 0 | 0 | 0 | 0 | 0 | 0 | 1 | 1 | Bacteria | Bacteria\_none | Fusobacteriota | Fusobacteriia | Fusobacteriales | Haliovirgaceae | Bacteria;Bacteria\_none;Fusobacteriota;Fusobacteriia;Fusobacteriales;Haliovirgaceae;Haliovirga |
| Leptotrichia | 16 | 96 | 175 | 84 | 78 | 230 | 432 | 376 | 24 | 92 | 107 | 24 | 666 | 2400 | Bacteria | Bacteria\_none | Fusobacteriota | Fusobacteriia | Fusobacteriales | Leptotrichiaceae | Bacteria;Bacteria\_none;Fusobacteriota;Fusobacteriia;Fusobacteriales;Leptotrichiaceae;Leptotrichia |
| Pseudoleptotrichia | 0 | 0 | 0 | 14 | 35 | 0 | 0 | 0 | 0 | 2 | 61 | 0 | 32 | 144 | Bacteria | Bacteria\_none | Fusobacteriota | Fusobacteriia | Fusobacteriales | Leptotrichiaceae | Bacteria;Bacteria\_none;Fusobacteriota;Fusobacteriia;Fusobacteriales;Leptotrichiaceae;Pseudoleptotrichia |
| Sebaldella | 0 | 0 | 0 | 0 | 0 | 0 | 0 | 1 | 0 | 0 | 0 | 0 | 0 | 1 | Bacteria | Bacteria\_none | Fusobacteriota | Fusobacteriia | Fusobacteriales | Leptotrichiaceae | Bacteria;Bacteria\_none;Fusobacteriota;Fusobacteriia;Fusobacteriales;Leptotrichiaceae;Sebaldella |
| Sneathia | 0 | 1 | 0 | 0 | 0 | 1 | 0 | 0 | 0 | 0 | 0 | 0 | 0 | 2 | Bacteria | Bacteria\_none | Fusobacteriota | Fusobacteriia | Fusobacteriales | Leptotrichiaceae | Bacteria;Bacteria\_none;Fusobacteriota;Fusobacteriia;Fusobacteriales;Leptotrichiaceae;Sneathia |
| Streptobacillus | 0 | 0 | 1 | 5 | 0 | 0 | 0 | 0 | 0 | 0 | 0 | 0 | 0 | 6 | Bacteria | Bacteria\_none | Fusobacteriota | Fusobacteriia | Fusobacteriales | Leptotrichiaceae | Bacteria;Bacteria\_none;Fusobacteriota;Fusobacteriia;Fusobacteriales;Leptotrichiaceae;Streptobacillus |
| Ignavibacterium | 0 | 0 | 0 | 0 | 0 | 0 | 0 | 0 | 0 | 0 | 0 | 0 | 1 | 1 | Bacteria | Bacteria\_none | Ignavibacteriota | Ignavibacteria | Ignavibacteriales | Ignavibacteriaceae | Bacteria;Bacteria\_none;Ignavibacteriota;Ignavibacteria;Ignavibacteriales;Ignavibacteriaceae;Ignavibacterium |
| Acholeplasma | 0 | 0 | 0 | 0 | 1 | 0 | 0 | 0 | 0 | 0 | 0 | 0 | 0 | 1 | Bacteria | Bacteria\_none | Mycoplasmatota | Mollicutes | Acholeplasmatales | Acholeplasmataceae | Bacteria;Bacteria\_none;Mycoplasmatota;Mollicutes;Acholeplasmatales;Acholeplasmataceae;Acholeplasma |
| Mariniplasma | 0 | 0 | 0 | 0 | 0 | 1 | 0 | 0 | 0 | 0 | 0 | 0 | 0 | 1 | Bacteria | Bacteria\_none | Mycoplasmatota | Mollicutes | Acholeplasmatales | Acholeplasmataceae | Bacteria;Bacteria\_none;Mycoplasmatota;Mollicutes;Acholeplasmatales;Acholeplasmataceae;Mariniplasma |
| Spiroplasma | 0 | 0 | 0 | 1 | 1 | 3 | 0 | 0 | 0 | 1 | 0 | 0 | 3 | 9 | Bacteria | Bacteria\_none | Mycoplasmatota | Mollicutes | Entomoplasmatales | Spiroplasmataceae | Bacteria;Bacteria\_none;Mycoplasmatota;Mollicutes;Entomoplasmatales;Spiroplasmataceae;Spiroplasma |
| Mycoplasma | 0 | 1 | 3 | 0 | 0 | 1 | 0 | 0 | 0 | 0 | 0 | 0 | 1 | 6 | Bacteria | Bacteria\_none | Mycoplasmatota | Mollicutes | Mycoplasmatales | Mycoplasmataceae | Bacteria;Bacteria\_none;Mycoplasmatota;Mollicutes;Mycoplasmatales;Mycoplasmataceae;Mycoplasma |
| Mycoplasmatales\_Incertae\_sedis | 0 | 0 | 0 | 0 | 0 | 0 | 0 | 0 | 0 | 0 | 1 | 0 | 0 | 1 | Bacteria | Bacteria\_none | Mycoplasmatota | Mollicutes | Mycoplasmatales | Mycoplasmatales\_Incertae\_sedis | Bacteria;Bacteria\_none;Mycoplasmatota;Mollicutes;Mycoplasmatales;Mycoplasmatales\_Incertae\_sedis;Mycoplasmatales\_Incertae\_sedis |
| Metamycoplasma | 0 | 8 | 35 | 2 | 2 | 6 | 0 | 3 | 0 | 9 | 3 | 6 | 26 | 100 | Bacteria | Bacteria\_none | Mycoplasmatota | Mycoplasmatota\_Incertae\_sedis | Mycoplasmoidales | Metamycoplasmataceae | Bacteria;Bacteria\_none;Mycoplasmatota;Mycoplasmatota\_Incertae\_sedis;Mycoplasmoidales;Metamycoplasmataceae;Metamycoplasma |
| Mycoplasmopsis | 0 | 2 | 2 | 0 | 0 | 0 | 1 | 0 | 0 | 0 | 0 | 1 | 0 | 6 | Bacteria | Bacteria\_none | Mycoplasmatota | Mycoplasmatota\_Incertae\_sedis | Mycoplasmoidales | Metamycoplasmataceae | Bacteria;Bacteria\_none;Mycoplasmatota;Mycoplasmatota\_Incertae\_sedis;Mycoplasmoidales;Metamycoplasmataceae;Mycoplasmopsis |
| Malacoplasma | 0 | 0 | 0 | 0 | 1 | 0 | 0 | 0 | 0 | 0 | 0 | 0 | 0 | 1 | Bacteria | Bacteria\_none | Mycoplasmatota | Mycoplasmatota\_Incertae\_sedis | Mycoplasmoidales | Mycoplasmoidaceae | Bacteria;Bacteria\_none;Mycoplasmatota;Mycoplasmatota\_Incertae\_sedis;Mycoplasmoidales;Mycoplasmoidaceae;Malacoplasma |
| Mycoplasmoides | 0 | 0 | 0 | 0 | 0 | 0 | 0 | 0 | 0 | 0 | 0 | 0 | 1 | 1 | Bacteria | Bacteria\_none | Mycoplasmatota | Mycoplasmatota\_Incertae\_sedis | Mycoplasmoidales | Mycoplasmoidaceae | Bacteria;Bacteria\_none;Mycoplasmatota;Mycoplasmatota\_Incertae\_sedis;Mycoplasmoidales;Mycoplasmoidaceae;Mycoplasmoides |
| Corallococcus | 0 | 0 | 0 | 0 | 0 | 0 | 0 | 0 | 0 | 0 | 0 | 0 | 1 | 1 | Bacteria | Bacteria\_none | Myxococcota | Myxococcia | Myxococcales | Myxococcaceae | Bacteria;Bacteria\_none;Myxococcota;Myxococcia;Myxococcales;Myxococcaceae;Corallococcus |
| Sorangium | 0 | 0 | 0 | 0 | 2 | 0 | 0 | 0 | 0 | 0 | 1 | 0 | 0 | 3 | Bacteria | Bacteria\_none | Myxococcota | Myxococcota\_Incertae\_sedis | Polyangiales | Polyangiaceae | Bacteria;Bacteria\_none;Myxococcota;Myxococcota\_Incertae\_sedis;Polyangiales;Polyangiaceae;Sorangium |
| Nitrospira | 0 | 0 | 0 | 0 | 0 | 1 | 0 | 1 | 0 | 0 | 0 | 0 | 0 | 2 | Bacteria | Bacteria\_none | Nitrospirota | Nitrospiria | Nitrospirales | Nitrospiraceae | Bacteria;Bacteria\_none;Nitrospirota;Nitrospiria;Nitrospirales;Nitrospiraceae;Nitrospira |
| Dissulfurispira | 0 | 0 | 0 | 0 | 0 | 0 | 0 | 0 | 0 | 0 | 1 | 0 | 0 | 1 | Bacteria | Bacteria\_none | Nitrospirota | Thermodesulfovibrionia | Thermodesulfovibrionales | Dissulfurispiraceae | Bacteria;Bacteria\_none;Nitrospirota;Thermodesulfovibrionia;Thermodesulfovibrionales;Dissulfurispiraceae;Dissulfurispira |
| Tuwongella | 0 | 0 | 0 | 0 | 0 | 1 | 0 | 0 | 0 | 0 | 0 | 0 | 0 | 1 | Bacteria | Bacteria\_none | Planctomycetota | Planctomycetia | Gemmatales | Gemmataceae | Bacteria;Bacteria\_none;Planctomycetota;Planctomycetia;Gemmatales;Gemmataceae;Tuwongella |
| Lignipirellula | 1 | 0 | 0 | 0 | 0 | 0 | 0 | 0 | 0 | 0 | 0 | 0 | 0 | 1 | Bacteria | Bacteria\_none | Planctomycetota | Planctomycetia | Pirellulales | Pirellulaceae | Bacteria;Bacteria\_none;Planctomycetota;Planctomycetia;Pirellulales;Pirellulaceae;Lignipirellula |
| Fuerstiella | 0 | 0 | 0 | 0 | 0 | 0 | 0 | 0 | 0 | 0 | 1 | 0 | 0 | 1 | Bacteria | Bacteria\_none | Planctomycetota | Planctomycetia | Planctomycetales | Planctomycetaceae | Bacteria;Bacteria\_none;Planctomycetota;Planctomycetia;Planctomycetales;Planctomycetaceae;Fuerstiella |
| Gimesia | 0 | 0 | 0 | 0 | 0 | 0 | 0 | 0 | 0 | 0 | 1 | 0 | 0 | 1 | Bacteria | Bacteria\_none | Planctomycetota | Planctomycetia | Planctomycetales | Planctomycetaceae | Bacteria;Bacteria\_none;Planctomycetota;Planctomycetia;Planctomycetales;Planctomycetaceae;Gimesia |
| Candidatus Pelagibacter | 0 | 0 | 0 | 0 | 0 | 0 | 0 | 0 | 0 | 0 | 0 | 0 | 1 | 1 | Bacteria | Bacteria\_none | Pseudomonadota | Alphaproteobacteria | Candidatus Pelagibacterales | Candidatus Pelagibacteraceae | Bacteria;Bacteria\_none;Pseudomonadota;Alphaproteobacteria;Candidatus Pelagibacterales;Candidatus Pelagibacteraceae;Candidatus Pelagibacter |
| Asticcacaulis | 0 | 0 | 0 | 0 | 0 | 0 | 0 | 0 | 0 | 0 | 0 | 0 | 1 | 1 | Bacteria | Bacteria\_none | Pseudomonadota | Alphaproteobacteria | Caulobacterales | Caulobacteraceae | Bacteria;Bacteria\_none;Pseudomonadota;Alphaproteobacteria;Caulobacterales;Caulobacteraceae;Asticcacaulis |
| Bosea | 0 | 0 | 0 | 0 | 0 | 1 | 0 | 0 | 0 | 0 | 0 | 0 | 0 | 1 | Bacteria | Bacteria\_none | Pseudomonadota | Alphaproteobacteria | Hyphomicrobiales | Boseaceae | Bacteria;Bacteria\_none;Pseudomonadota;Alphaproteobacteria;Hyphomicrobiales;Boseaceae;Bosea |
| Brucella | 0 | 0 | 0 | 0 | 0 | 0 | 0 | 0 | 0 | 0 | 1 | 0 | 0 | 1 | Bacteria | Bacteria\_none | Pseudomonadota | Alphaproteobacteria | Hyphomicrobiales | Brucellaceae | Bacteria;Bacteria\_none;Pseudomonadota;Alphaproteobacteria;Hyphomicrobiales;Brucellaceae;Brucella |
| Cohaesibacter | 0 | 0 | 0 | 0 | 0 | 0 | 0 | 0 | 1 | 0 | 0 | 0 | 1 | 2 | Bacteria | Bacteria\_none | Pseudomonadota | Alphaproteobacteria | Hyphomicrobiales | Cohaesibacteraceae | Bacteria;Bacteria\_none;Pseudomonadota;Alphaproteobacteria;Hyphomicrobiales;Cohaesibacteraceae;Cohaesibacter |
| Devosia | 0 | 0 | 0 | 0 | 0 | 0 | 1 | 0 | 0 | 0 | 0 | 0 | 0 | 1 | Bacteria | Bacteria\_none | Pseudomonadota | Alphaproteobacteria | Hyphomicrobiales | Devosiaceae | Bacteria;Bacteria\_none;Pseudomonadota;Alphaproteobacteria;Hyphomicrobiales;Devosiaceae;Devosia |
| Pelagibacterium | 0 | 0 | 1 | 0 | 0 | 0 | 0 | 0 | 0 | 0 | 0 | 0 | 0 | 1 | Bacteria | Bacteria\_none | Pseudomonadota | Alphaproteobacteria | Hyphomicrobiales | Devosiaceae | Bacteria;Bacteria\_none;Pseudomonadota;Alphaproteobacteria;Hyphomicrobiales;Devosiaceae;Pelagibacterium |
| Methylobacterium | 0 | 0 | 1 | 0 | 0 | 0 | 0 | 0 | 0 | 0 | 0 | 0 | 0 | 1 | Bacteria | Bacteria\_none | Pseudomonadota | Alphaproteobacteria | Hyphomicrobiales | Methylobacteriaceae | Bacteria;Bacteria\_none;Pseudomonadota;Alphaproteobacteria;Hyphomicrobiales;Methylobacteriaceae;Methylobacterium |
| Microvirga | 0 | 0 | 0 | 0 | 0 | 0 | 0 | 0 | 0 | 0 | 1 | 0 | 0 | 1 | Bacteria | Bacteria\_none | Pseudomonadota | Alphaproteobacteria | Hyphomicrobiales | Methylobacteriaceae | Bacteria;Bacteria\_none;Pseudomonadota;Alphaproteobacteria;Hyphomicrobiales;Methylobacteriaceae;Microvirga |
| Afipia | 0 | 1 | 0 | 0 | 0 | 0 | 0 | 0 | 0 | 0 | 0 | 0 | 0 | 1 | Bacteria | Bacteria\_none | Pseudomonadota | Alphaproteobacteria | Hyphomicrobiales | Nitrobacteraceae | Bacteria;Bacteria\_none;Pseudomonadota;Alphaproteobacteria;Hyphomicrobiales;Nitrobacteraceae;Afipia |
| Bradyrhizobium | 0 | 0 | 1 | 0 | 0 | 1 | 0 | 0 | 0 | 0 | 2 | 0 | 0 | 4 | Bacteria | Bacteria\_none | Pseudomonadota | Alphaproteobacteria | Hyphomicrobiales | Nitrobacteraceae | Bacteria;Bacteria\_none;Pseudomonadota;Alphaproteobacteria;Hyphomicrobiales;Nitrobacteraceae;Bradyrhizobium |
| Aquibium | 0 | 0 | 0 | 0 | 0 | 0 | 0 | 2 | 0 | 0 | 0 | 0 | 0 | 2 | Bacteria | Bacteria\_none | Pseudomonadota | Alphaproteobacteria | Hyphomicrobiales | Phyllobacteriaceae | Bacteria;Bacteria\_none;Pseudomonadota;Alphaproteobacteria;Hyphomicrobiales;Phyllobacteriaceae;Aquibium |
| Mesorhizobium | 0 | 0 | 2 | 0 | 0 | 1 | 0 | 1 | 0 | 0 | 0 | 0 | 0 | 4 | Bacteria | Bacteria\_none | Pseudomonadota | Alphaproteobacteria | Hyphomicrobiales | Phyllobacteriaceae | Bacteria;Bacteria\_none;Pseudomonadota;Alphaproteobacteria;Hyphomicrobiales;Phyllobacteriaceae;Mesorhizobium |
| Nitratireductor | 0 | 0 | 0 | 0 | 0 | 0 | 1 | 0 | 0 | 0 | 0 | 0 | 0 | 1 | Bacteria | Bacteria\_none | Pseudomonadota | Alphaproteobacteria | Hyphomicrobiales | Phyllobacteriaceae | Bacteria;Bacteria\_none;Pseudomonadota;Alphaproteobacteria;Hyphomicrobiales;Phyllobacteriaceae;Nitratireductor |
| Agrobacterium | 0 | 0 | 0 | 0 | 0 | 0 | 0 | 1 | 0 | 0 | 0 | 0 | 0 | 1 | Bacteria | Bacteria\_none | Pseudomonadota | Alphaproteobacteria | Hyphomicrobiales | Rhizobiaceae | Bacteria;Bacteria\_none;Pseudomonadota;Alphaproteobacteria;Hyphomicrobiales;Rhizobiaceae;Agrobacterium |
| Liberibacter | 0 | 0 | 0 | 0 | 0 | 0 | 0 | 0 | 0 | 0 | 1 | 0 | 0 | 1 | Bacteria | Bacteria\_none | Pseudomonadota | Alphaproteobacteria | Hyphomicrobiales | Rhizobiaceae | Bacteria;Bacteria\_none;Pseudomonadota;Alphaproteobacteria;Hyphomicrobiales;Rhizobiaceae;Liberibacter |
| Pararhizobium | 0 | 0 | 0 | 0 | 0 | 1 | 0 | 0 | 0 | 0 | 0 | 0 | 0 | 1 | Bacteria | Bacteria\_none | Pseudomonadota | Alphaproteobacteria | Hyphomicrobiales | Rhizobiaceae | Bacteria;Bacteria\_none;Pseudomonadota;Alphaproteobacteria;Hyphomicrobiales;Rhizobiaceae;Pararhizobium |
| Rhizobium | 0 | 0 | 0 | 0 | 0 | 0 | 0 | 0 | 0 | 1 | 0 | 0 | 2 | 3 | Bacteria | Bacteria\_none | Pseudomonadota | Alphaproteobacteria | Hyphomicrobiales | Rhizobiaceae | Bacteria;Bacteria\_none;Pseudomonadota;Alphaproteobacteria;Hyphomicrobiales;Rhizobiaceae;Rhizobium |
| Shinella | 0 | 0 | 0 | 0 | 0 | 1 | 0 | 0 | 0 | 0 | 0 | 0 | 0 | 1 | Bacteria | Bacteria\_none | Pseudomonadota | Alphaproteobacteria | Hyphomicrobiales | Rhizobiaceae | Bacteria;Bacteria\_none;Pseudomonadota;Alphaproteobacteria;Hyphomicrobiales;Rhizobiaceae;Shinella |
| Sinorhizobium | 1 | 0 | 0 | 0 | 1 | 0 | 0 | 0 | 0 | 0 | 0 | 0 | 0 | 2 | Bacteria | Bacteria\_none | Pseudomonadota | Alphaproteobacteria | Hyphomicrobiales | Rhizobiaceae | Bacteria;Bacteria\_none;Pseudomonadota;Alphaproteobacteria;Hyphomicrobiales;Rhizobiaceae;Sinorhizobium |
| Kordiimonas | 0 | 0 | 0 | 0 | 0 | 0 | 0 | 1 | 0 | 0 | 0 | 0 | 0 | 1 | Bacteria | Bacteria\_none | Pseudomonadota | Alphaproteobacteria | Kordiimonadales | Kordiimonadaceae | Bacteria;Bacteria\_none;Pseudomonadota;Alphaproteobacteria;Kordiimonadales;Kordiimonadaceae;Kordiimonas |
| Cereibacter | 0 | 0 | 0 | 0 | 0 | 0 | 0 | 0 | 0 | 0 | 1 | 0 | 0 | 1 | Bacteria | Bacteria\_none | Pseudomonadota | Alphaproteobacteria | Rhodobacterales | Paracoccaceae | Bacteria;Bacteria\_none;Pseudomonadota;Alphaproteobacteria;Rhodobacterales;Paracoccaceae;Cereibacter |
| Paracoccus | 0 | 0 | 0 | 0 | 0 | 1 | 0 | 0 | 1 | 0 | 0 | 0 | 0 | 2 | Bacteria | Bacteria\_none | Pseudomonadota | Alphaproteobacteria | Rhodobacterales | Paracoccaceae | Bacteria;Bacteria\_none;Pseudomonadota;Alphaproteobacteria;Rhodobacterales;Paracoccaceae;Paracoccus |
| Tabrizicola | 0 | 0 | 0 | 0 | 0 | 0 | 0 | 0 | 0 | 0 | 0 | 0 | 1 | 1 | Bacteria | Bacteria\_none | Pseudomonadota | Alphaproteobacteria | Rhodobacterales | Paracoccaceae | Bacteria;Bacteria\_none;Pseudomonadota;Alphaproteobacteria;Rhodobacterales;Paracoccaceae;Tabrizicola |
| Phaeobacter | 0 | 0 | 0 | 0 | 1 | 0 | 0 | 0 | 0 | 0 | 0 | 0 | 0 | 1 | Bacteria | Bacteria\_none | Pseudomonadota | Alphaproteobacteria | Rhodobacterales | Roseobacteraceae | Bacteria;Bacteria\_none;Pseudomonadota;Alphaproteobacteria;Rhodobacterales;Roseobacteraceae;Phaeobacter |
| Ponticoccus | 2 | 0 | 0 | 0 | 0 | 0 | 0 | 0 | 0 | 0 | 0 | 0 | 0 | 2 | Bacteria | Bacteria\_none | Pseudomonadota | Alphaproteobacteria | Rhodobacterales | Roseobacteraceae | Bacteria;Bacteria\_none;Pseudomonadota;Alphaproteobacteria;Rhodobacterales;Roseobacteraceae;Ponticoccus |
| Pseudosulfitobacter | 0 | 1 | 0 | 0 | 0 | 0 | 0 | 0 | 0 | 0 | 0 | 0 | 0 | 1 | Bacteria | Bacteria\_none | Pseudomonadota | Alphaproteobacteria | Rhodobacterales | Roseobacteraceae | Bacteria;Bacteria\_none;Pseudomonadota;Alphaproteobacteria;Rhodobacterales;Roseobacteraceae;Pseudosulfitobacter |
| Roseobacter | 0 | 0 | 0 | 1 | 0 | 0 | 0 | 0 | 0 | 0 | 0 | 0 | 0 | 1 | Bacteria | Bacteria\_none | Pseudomonadota | Alphaproteobacteria | Rhodobacterales | Roseobacteraceae | Bacteria;Bacteria\_none;Pseudomonadota;Alphaproteobacteria;Rhodobacterales;Roseobacteraceae;Roseobacter |
| Ruegeria | 0 | 0 | 0 | 0 | 1 | 0 | 0 | 0 | 0 | 0 | 0 | 0 | 1 | 2 | Bacteria | Bacteria\_none | Pseudomonadota | Alphaproteobacteria | Rhodobacterales | Roseobacteraceae | Bacteria;Bacteria\_none;Pseudomonadota;Alphaproteobacteria;Rhodobacterales;Roseobacteraceae;Ruegeria |
| Sulfitobacter | 0 | 0 | 0 | 0 | 0 | 0 | 0 | 0 | 0 | 0 | 2 | 0 | 0 | 2 | Bacteria | Bacteria\_none | Pseudomonadota | Alphaproteobacteria | Rhodobacterales | Roseobacteraceae | Bacteria;Bacteria\_none;Pseudomonadota;Alphaproteobacteria;Rhodobacterales;Roseobacteraceae;Sulfitobacter |
| Komagataeibacter | 0 | 0 | 0 | 0 | 0 | 1 | 0 | 0 | 0 | 0 | 0 | 0 | 0 | 1 | Bacteria | Bacteria\_none | Pseudomonadota | Alphaproteobacteria | Rhodospirillales | Acetobacteraceae | Bacteria;Bacteria\_none;Pseudomonadota;Alphaproteobacteria;Rhodospirillales;Acetobacteraceae;Komagataeibacter |
| Pseudoroseomonas | 0 | 0 | 0 | 0 | 0 | 0 | 1 | 0 | 0 | 0 | 0 | 0 | 0 | 1 | Bacteria | Bacteria\_none | Pseudomonadota | Alphaproteobacteria | Rhodospirillales | Acetobacteraceae | Bacteria;Bacteria\_none;Pseudomonadota;Alphaproteobacteria;Rhodospirillales;Acetobacteraceae;Pseudoroseomonas |
| Azospirillum | 0 | 0 | 0 | 0 | 1 | 0 | 0 | 0 | 0 | 0 | 1 | 0 | 0 | 2 | Bacteria | Bacteria\_none | Pseudomonadota | Alphaproteobacteria | Rhodospirillales | Azospirillaceae | Bacteria;Bacteria\_none;Pseudomonadota;Alphaproteobacteria;Rhodospirillales;Azospirillaceae;Azospirillum |
| Varunaivibrio | 0 | 0 | 0 | 0 | 0 | 0 | 0 | 0 | 0 | 0 | 0 | 0 | 1 | 1 | Bacteria | Bacteria\_none | Pseudomonadota | Alphaproteobacteria | Rhodospirillales | Magnetovibrionaceae | Bacteria;Bacteria\_none;Pseudomonadota;Alphaproteobacteria;Rhodospirillales;Magnetovibrionaceae;Varunaivibrio |
| Thalassospira | 0 | 0 | 0 | 1 | 0 | 0 | 0 | 0 | 0 | 0 | 0 | 0 | 0 | 1 | Bacteria | Bacteria\_none | Pseudomonadota | Alphaproteobacteria | Rhodospirillales | Thalassospiraceae | Bacteria;Bacteria\_none;Pseudomonadota;Alphaproteobacteria;Rhodospirillales;Thalassospiraceae;Thalassospira |
| Neorickettsia | 0 | 1 | 0 | 0 | 0 | 0 | 0 | 0 | 0 | 0 | 0 | 0 | 0 | 1 | Bacteria | Bacteria\_none | Pseudomonadota | Alphaproteobacteria | Rickettsiales | Anaplasmataceae | Bacteria;Bacteria\_none;Pseudomonadota;Alphaproteobacteria;Rickettsiales;Anaplasmataceae;Neorickettsia |
| Wolbachia | 0 | 0 | 0 | 0 | 0 | 0 | 0 | 0 | 0 | 0 | 0 | 0 | 3 | 3 | Bacteria | Bacteria\_none | Pseudomonadota | Alphaproteobacteria | Rickettsiales | Anaplasmataceae | Bacteria;Bacteria\_none;Pseudomonadota;Alphaproteobacteria;Rickettsiales;Anaplasmataceae;Wolbachia |
| Candidatus Phycorickettsia | 0 | 0 | 0 | 0 | 0 | 0 | 1 | 0 | 0 | 0 | 0 | 0 | 0 | 1 | Bacteria | Bacteria\_none | Pseudomonadota | Alphaproteobacteria | Rickettsiales | Rickettsiaceae | Bacteria;Bacteria\_none;Pseudomonadota;Alphaproteobacteria;Rickettsiales;Rickettsiaceae;Candidatus Phycorickettsia |
| Novosphingobium | 0 | 0 | 0 | 0 | 0 | 0 | 0 | 0 | 0 | 0 | 1 | 0 | 0 | 1 | Bacteria | Bacteria\_none | Pseudomonadota | Alphaproteobacteria | Sphingomonadales | Sphingomonadaceae | Bacteria;Bacteria\_none;Pseudomonadota;Alphaproteobacteria;Sphingomonadales;Sphingomonadaceae;Novosphingobium |
| Sphingomonas | 0 | 0 | 0 | 0 | 0 | 0 | 0 | 0 | 0 | 0 | 1 | 1 | 2 | 4 | Bacteria | Bacteria\_none | Pseudomonadota | Alphaproteobacteria | Sphingomonadales | Sphingomonadaceae | Bacteria;Bacteria\_none;Pseudomonadota;Alphaproteobacteria;Sphingomonadales;Sphingomonadaceae;Sphingomonas |
| Sphingopyxis | 0 | 0 | 0 | 1 | 0 | 1 | 0 | 0 | 0 | 0 | 0 | 0 | 0 | 2 | Bacteria | Bacteria\_none | Pseudomonadota | Alphaproteobacteria | Sphingomonadales | Sphingomonadaceae | Bacteria;Bacteria\_none;Pseudomonadota;Alphaproteobacteria;Sphingomonadales;Sphingomonadaceae;Sphingopyxis |
| Candidatus Profftella | 0 | 0 | 0 | 0 | 1 | 0 | 0 | 0 | 0 | 0 | 0 | 0 | 0 | 1 | Bacteria | Bacteria\_none | Pseudomonadota | Betaproteobacteria | Betaproteobacteria\_Incertae\_sedis | Betaproteobacteria\_Incertae\_sedis | Bacteria;Bacteria\_none;Pseudomonadota;Betaproteobacteria;Betaproteobacteria\_Incertae\_sedis;Betaproteobacteria\_Incertae\_sedis;Candidatus Profftella |
| Casimicrobium | 0 | 0 | 0 | 0 | 0 | 0 | 0 | 1 | 0 | 0 | 0 | 0 | 0 | 1 | Bacteria | Bacteria\_none | Pseudomonadota | Betaproteobacteria | Betaproteobacteria\_Incertae\_sedis | Casimicrobiaceae | Bacteria;Bacteria\_none;Pseudomonadota;Betaproteobacteria;Betaproteobacteria\_Incertae\_sedis;Casimicrobiaceae;Casimicrobium |
| Advenella | 0 | 0 | 0 | 0 | 1 | 0 | 0 | 0 | 0 | 0 | 0 | 0 | 0 | 1 | Bacteria | Bacteria\_none | Pseudomonadota | Betaproteobacteria | Burkholderiales | Alcaligenaceae | Bacteria;Bacteria\_none;Pseudomonadota;Betaproteobacteria;Burkholderiales;Alcaligenaceae;Advenella |
| Bordetella | 0 | 0 | 0 | 0 | 0 | 0 | 0 | 0 | 0 | 0 | 1 | 0 | 0 | 1 | Bacteria | Bacteria\_none | Pseudomonadota | Betaproteobacteria | Burkholderiales | Alcaligenaceae | Bacteria;Bacteria\_none;Pseudomonadota;Betaproteobacteria;Burkholderiales;Alcaligenaceae;Bordetella |
| Neopusillimonas | 0 | 0 | 0 | 0 | 0 | 0 | 1 | 0 | 0 | 0 | 0 | 0 | 0 | 1 | Bacteria | Bacteria\_none | Pseudomonadota | Betaproteobacteria | Burkholderiales | Alcaligenaceae | Bacteria;Bacteria\_none;Pseudomonadota;Betaproteobacteria;Burkholderiales;Alcaligenaceae;Neopusillimonas |
| Orrella | 0 | 0 | 0 | 0 | 0 | 1 | 0 | 0 | 0 | 0 | 0 | 0 | 0 | 1 | Bacteria | Bacteria\_none | Pseudomonadota | Betaproteobacteria | Burkholderiales | Alcaligenaceae | Bacteria;Bacteria\_none;Pseudomonadota;Betaproteobacteria;Burkholderiales;Alcaligenaceae;Orrella |
| Pollutimonas | 0 | 0 | 0 | 0 | 0 | 0 | 0 | 0 | 0 | 0 | 1 | 0 | 0 | 1 | Bacteria | Bacteria\_none | Pseudomonadota | Betaproteobacteria | Burkholderiales | Alcaligenaceae | Bacteria;Bacteria\_none;Pseudomonadota;Betaproteobacteria;Burkholderiales;Alcaligenaceae;Pollutimonas |
| Pusillimonas | 0 | 0 | 0 | 0 | 0 | 0 | 0 | 0 | 0 | 0 | 0 | 0 | 1 | 1 | Bacteria | Bacteria\_none | Pseudomonadota | Betaproteobacteria | Burkholderiales | Alcaligenaceae | Bacteria;Bacteria\_none;Pseudomonadota;Betaproteobacteria;Burkholderiales;Alcaligenaceae;Pusillimonas |
| Burkholderia | 0 | 0 | 2 | 0 | 0 | 1 | 0 | 0 | 0 | 1 | 0 | 0 | 4 | 8 | Bacteria | Bacteria\_none | Pseudomonadota | Betaproteobacteria | Burkholderiales | Burkholderiaceae | Bacteria;Bacteria\_none;Pseudomonadota;Betaproteobacteria;Burkholderiales;Burkholderiaceae;Burkholderia |
| Cupriavidus | 0 | 0 | 0 | 0 | 1 | 0 | 0 | 0 | 0 | 0 | 0 | 0 | 0 | 1 | Bacteria | Bacteria\_none | Pseudomonadota | Betaproteobacteria | Burkholderiales | Burkholderiaceae | Bacteria;Bacteria\_none;Pseudomonadota;Betaproteobacteria;Burkholderiales;Burkholderiaceae;Cupriavidus |
| Lautropia | 0 | 1 | 0 | 0 | 4 | 0 | 0 | 2 | 0 | 2 | 11 | 0 | 3 | 23 | Bacteria | Bacteria\_none | Pseudomonadota | Betaproteobacteria | Burkholderiales | Burkholderiaceae | Bacteria;Bacteria\_none;Pseudomonadota;Betaproteobacteria;Burkholderiales;Burkholderiaceae;Lautropia |
| Mycoavidus | 0 | 0 | 0 | 0 | 0 | 0 | 0 | 0 | 0 | 1 | 0 | 0 | 0 | 1 | Bacteria | Bacteria\_none | Pseudomonadota | Betaproteobacteria | Burkholderiales | Burkholderiaceae | Bacteria;Bacteria\_none;Pseudomonadota;Betaproteobacteria;Burkholderiales;Burkholderiaceae;Mycoavidus |
| Pandoraea | 1 | 0 | 0 | 0 | 0 | 0 | 0 | 0 | 0 | 0 | 0 | 0 | 0 | 1 | Bacteria | Bacteria\_none | Pseudomonadota | Betaproteobacteria | Burkholderiales | Burkholderiaceae | Bacteria;Bacteria\_none;Pseudomonadota;Betaproteobacteria;Burkholderiales;Burkholderiaceae;Pandoraea |
| Paraburkholderia | 0 | 0 | 0 | 0 | 0 | 0 | 2 | 0 | 0 | 0 | 0 | 0 | 0 | 2 | Bacteria | Bacteria\_none | Pseudomonadota | Betaproteobacteria | Burkholderiales | Burkholderiaceae | Bacteria;Bacteria\_none;Pseudomonadota;Betaproteobacteria;Burkholderiales;Burkholderiaceae;Paraburkholderia |
| Polynucleobacter | 0 | 0 | 0 | 0 | 0 | 0 | 0 | 0 | 0 | 0 | 0 | 0 | 1 | 1 | Bacteria | Bacteria\_none | Pseudomonadota | Betaproteobacteria | Burkholderiales | Burkholderiaceae | Bacteria;Bacteria\_none;Pseudomonadota;Betaproteobacteria;Burkholderiales;Burkholderiaceae;Polynucleobacter |
| Ralstonia | 0 | 0 | 0 | 0 | 1 | 0 | 0 | 0 | 0 | 0 | 0 | 0 | 0 | 1 | Bacteria | Bacteria\_none | Pseudomonadota | Betaproteobacteria | Burkholderiales | Burkholderiaceae | Bacteria;Bacteria\_none;Pseudomonadota;Betaproteobacteria;Burkholderiales;Burkholderiaceae;Ralstonia |
| Acidovorax | 0 | 0 | 0 | 0 | 0 | 0 | 0 | 0 | 0 | 0 | 1 | 0 | 0 | 1 | Bacteria | Bacteria\_none | Pseudomonadota | Betaproteobacteria | Burkholderiales | Comamonadaceae | Bacteria;Bacteria\_none;Pseudomonadota;Betaproteobacteria;Burkholderiales;Comamonadaceae;Acidovorax |
| Hydrogenophaga | 0 | 0 | 0 | 0 | 0 | 1 | 0 | 0 | 0 | 0 | 0 | 0 | 0 | 1 | Bacteria | Bacteria\_none | Pseudomonadota | Betaproteobacteria | Burkholderiales | Comamonadaceae | Bacteria;Bacteria\_none;Pseudomonadota;Betaproteobacteria;Burkholderiales;Comamonadaceae;Hydrogenophaga |
| Ottowia | 0 | 0 | 0 | 0 | 0 | 0 | 0 | 1 | 0 | 0 | 0 | 0 | 0 | 1 | Bacteria | Bacteria\_none | Pseudomonadota | Betaproteobacteria | Burkholderiales | Comamonadaceae | Bacteria;Bacteria\_none;Pseudomonadota;Betaproteobacteria;Burkholderiales;Comamonadaceae;Ottowia |
| Variovorax | 0 | 0 | 0 | 0 | 0 | 0 | 1 | 0 | 0 | 0 | 0 | 0 | 1 | 2 | Bacteria | Bacteria\_none | Pseudomonadota | Betaproteobacteria | Burkholderiales | Comamonadaceae | Bacteria;Bacteria\_none;Pseudomonadota;Betaproteobacteria;Burkholderiales;Comamonadaceae;Variovorax |
| Candidatus Zinderia | 0 | 0 | 0 | 0 | 1 | 0 | 0 | 0 | 0 | 0 | 0 | 0 | 0 | 1 | Bacteria | Bacteria\_none | Pseudomonadota | Betaproteobacteria | Burkholderiales | Oxalobacteraceae | Bacteria;Bacteria\_none;Pseudomonadota;Betaproteobacteria;Burkholderiales;Oxalobacteraceae;Candidatus Zinderia |
| Janthinobacterium | 0 | 0 | 0 | 0 | 0 | 1 | 0 | 0 | 0 | 0 | 0 | 0 | 0 | 1 | Bacteria | Bacteria\_none | Pseudomonadota | Betaproteobacteria | Burkholderiales | Oxalobacteraceae | Bacteria;Bacteria\_none;Pseudomonadota;Betaproteobacteria;Burkholderiales;Oxalobacteraceae;Janthinobacterium |
| Massilia | 0 | 0 | 0 | 0 | 0 | 0 | 0 | 0 | 0 | 1 | 0 | 0 | 0 | 1 | Bacteria | Bacteria\_none | Pseudomonadota | Betaproteobacteria | Burkholderiales | Oxalobacteraceae | Bacteria;Bacteria\_none;Pseudomonadota;Betaproteobacteria;Burkholderiales;Oxalobacteraceae;Massilia |
| Noviherbaspirillum | 0 | 0 | 1 | 0 | 0 | 0 | 0 | 0 | 0 | 0 | 0 | 0 | 0 | 1 | Bacteria | Bacteria\_none | Pseudomonadota | Betaproteobacteria | Burkholderiales | Oxalobacteraceae | Bacteria;Bacteria\_none;Pseudomonadota;Betaproteobacteria;Burkholderiales;Oxalobacteraceae;Noviherbaspirillum |
| Oxalobacter | 0 | 0 | 0 | 0 | 0 | 0 | 0 | 0 | 0 | 0 | 0 | 0 | 1 | 1 | Bacteria | Bacteria\_none | Pseudomonadota | Betaproteobacteria | Burkholderiales | Oxalobacteraceae | Bacteria;Bacteria\_none;Pseudomonadota;Betaproteobacteria;Burkholderiales;Oxalobacteraceae;Oxalobacter |
| Pseudoduganella | 0 | 0 | 0 | 0 | 0 | 0 | 0 | 0 | 1 | 0 | 0 | 0 | 0 | 1 | Bacteria | Bacteria\_none | Pseudomonadota | Betaproteobacteria | Burkholderiales | Oxalobacteraceae | Bacteria;Bacteria\_none;Pseudomonadota;Betaproteobacteria;Burkholderiales;Oxalobacteraceae;Pseudoduganella |
| Piscinibacter | 0 | 0 | 0 | 0 | 0 | 0 | 0 | 0 | 0 | 0 | 0 | 1 | 0 | 1 | Bacteria | Bacteria\_none | Pseudomonadota | Betaproteobacteria | Burkholderiales | Sphaerotilaceae | Bacteria;Bacteria\_none;Pseudomonadota;Betaproteobacteria;Burkholderiales;Sphaerotilaceae;Piscinibacter |
| Rubrivivax | 0 | 0 | 0 | 0 | 0 | 0 | 0 | 0 | 0 | 0 | 1 | 0 | 0 | 1 | Bacteria | Bacteria\_none | Pseudomonadota | Betaproteobacteria | Burkholderiales | Sphaerotilaceae | Bacteria;Bacteria\_none;Pseudomonadota;Betaproteobacteria;Burkholderiales;Sphaerotilaceae;Rubrivivax |
| Alysiella | 0 | 0 | 0 | 0 | 1 | 0 | 0 | 0 | 0 | 0 | 0 | 1 | 0 | 2 | Bacteria | Bacteria\_none | Pseudomonadota | Betaproteobacteria | Neisseriales | Neisseriaceae | Bacteria;Bacteria\_none;Pseudomonadota;Betaproteobacteria;Neisseriales;Neisseriaceae;Alysiella |
| Aquella | 0 | 0 | 0 | 0 | 0 | 1 | 0 | 0 | 0 | 0 | 0 | 0 | 0 | 1 | Bacteria | Bacteria\_none | Pseudomonadota | Betaproteobacteria | Neisseriales | Neisseriaceae | Bacteria;Bacteria\_none;Pseudomonadota;Betaproteobacteria;Neisseriales;Neisseriaceae;Aquella |
| Conchiformibius | 0 | 0 | 1 | 0 | 0 | 0 | 0 | 0 | 0 | 0 | 0 | 0 | 0 | 1 | Bacteria | Bacteria\_none | Pseudomonadota | Betaproteobacteria | Neisseriales | Neisseriaceae | Bacteria;Bacteria\_none;Pseudomonadota;Betaproteobacteria;Neisseriales;Neisseriaceae;Conchiformibius |
| Eikenella | 0 | 2 | 30 | 9 | 15 | 0 | 0 | 3 | 0 | 3 | 5 | 1 | 15 | 83 | Bacteria | Bacteria\_none | Pseudomonadota | Betaproteobacteria | Neisseriales | Neisseriaceae | Bacteria;Bacteria\_none;Pseudomonadota;Betaproteobacteria;Neisseriales;Neisseriaceae;Eikenella |
| Kingella | 1 | 25 | 3 | 43 | 0 | 1 | 0 | 4 | 0 | 17 | 4 | 2 | 7 | 107 | Bacteria | Bacteria\_none | Pseudomonadota | Betaproteobacteria | Neisseriales | Neisseriaceae | Bacteria;Bacteria\_none;Pseudomonadota;Betaproteobacteria;Neisseriales;Neisseriaceae;Kingella |
| Morococcus | 0 | 3 | 9 | 26 | 0 | 0 | 0 | 2 | 0 | 3 | 2 | 4 | 25 | 74 | Bacteria | Bacteria\_none | Pseudomonadota | Betaproteobacteria | Neisseriales | Neisseriaceae | Bacteria;Bacteria\_none;Pseudomonadota;Betaproteobacteria;Neisseriales;Neisseriaceae;Morococcus |
| Neisseria | 204 | 172 | 1042 | 4018 | 341 | 325 | 61 | 185 | 1 | 1658 | 198 | 115 | 2147 | 10467 | Bacteria | Bacteria\_none | Pseudomonadota | Betaproteobacteria | Neisseriales | Neisseriaceae | Bacteria;Bacteria\_none;Pseudomonadota;Betaproteobacteria;Neisseriales;Neisseriaceae;Neisseria |
| Simonsiella | 0 | 0 | 0 | 1 | 0 | 0 | 0 | 0 | 0 | 0 | 0 | 0 | 0 | 1 | Bacteria | Bacteria\_none | Pseudomonadota | Betaproteobacteria | Neisseriales | Neisseriaceae | Bacteria;Bacteria\_none;Pseudomonadota;Betaproteobacteria;Neisseriales;Neisseriaceae;Simonsiella |
| Snodgrassella | 0 | 0 | 0 | 0 | 0 | 1 | 0 | 0 | 0 | 0 | 0 | 0 | 0 | 1 | Bacteria | Bacteria\_none | Pseudomonadota | Betaproteobacteria | Neisseriales | Neisseriaceae | Bacteria;Bacteria\_none;Pseudomonadota;Betaproteobacteria;Neisseriales;Neisseriaceae;Snodgrassella |
| Uruburuella | 0 | 0 | 0 | 0 | 0 | 0 | 0 | 0 | 0 | 0 | 0 | 0 | 1 | 1 | Bacteria | Bacteria\_none | Pseudomonadota | Betaproteobacteria | Neisseriales | Neisseriaceae | Bacteria;Bacteria\_none;Pseudomonadota;Betaproteobacteria;Neisseriales;Neisseriaceae;Uruburuella |
| Ferriphaselus | 0 | 0 | 0 | 0 | 0 | 0 | 0 | 0 | 0 | 0 | 0 | 0 | 1 | 1 | Bacteria | Bacteria\_none | Pseudomonadota | Betaproteobacteria | Nitrosomonadales | Gallionellaceae | Bacteria;Bacteria\_none;Pseudomonadota;Betaproteobacteria;Nitrosomonadales;Gallionellaceae;Ferriphaselus |
| Methylophilus | 0 | 0 | 0 | 0 | 0 | 0 | 0 | 0 | 0 | 0 | 0 | 0 | 1 | 1 | Bacteria | Bacteria\_none | Pseudomonadota | Betaproteobacteria | Nitrosomonadales | Methylophilaceae | Bacteria;Bacteria\_none;Pseudomonadota;Betaproteobacteria;Nitrosomonadales;Methylophilaceae;Methylophilus |
| Dechloromonas | 0 | 0 | 0 | 0 | 0 | 0 | 1 | 0 | 0 | 0 | 0 | 0 | 0 | 1 | Bacteria | Bacteria\_none | Pseudomonadota | Betaproteobacteria | Rhodocyclales | Azonexaceae | Bacteria;Bacteria\_none;Pseudomonadota;Betaproteobacteria;Rhodocyclales;Azonexaceae;Dechloromonas |
| Aeromonas | 0 | 0 | 0 | 0 | 0 | 1 | 0 | 0 | 0 | 0 | 0 | 0 | 0 | 1 | Bacteria | Bacteria\_none | Pseudomonadota | Gammaproteobacteria | Aeromonadales | Aeromonadaceae | Bacteria;Bacteria\_none;Pseudomonadota;Gammaproteobacteria;Aeromonadales;Aeromonadaceae;Aeromonas |
| Oceanisphaera | 0 | 0 | 0 | 0 | 0 | 0 | 0 | 0 | 0 | 0 | 0 | 0 | 1 | 1 | Bacteria | Bacteria\_none | Pseudomonadota | Gammaproteobacteria | Aeromonadales | Aeromonadaceae | Bacteria;Bacteria\_none;Pseudomonadota;Gammaproteobacteria;Aeromonadales;Aeromonadaceae;Oceanisphaera |
| Tolumonas | 0 | 0 | 0 | 0 | 0 | 1 | 0 | 0 | 0 | 0 | 0 | 0 | 0 | 1 | Bacteria | Bacteria\_none | Pseudomonadota | Gammaproteobacteria | Aeromonadales | Aeromonadaceae | Bacteria;Bacteria\_none;Pseudomonadota;Gammaproteobacteria;Aeromonadales;Aeromonadaceae;Tolumonas |
| Alteromonas | 1 | 0 | 0 | 0 | 0 | 0 | 0 | 0 | 0 | 0 | 0 | 0 | 2 | 3 | Bacteria | Bacteria\_none | Pseudomonadota | Gammaproteobacteria | Alteromonadales | Alteromonadaceae | Bacteria;Bacteria\_none;Pseudomonadota;Gammaproteobacteria;Alteromonadales;Alteromonadaceae;Alteromonas |
| Thalassotalea | 0 | 0 | 1 | 0 | 0 | 0 | 0 | 0 | 0 | 0 | 0 | 0 | 0 | 1 | Bacteria | Bacteria\_none | Pseudomonadota | Gammaproteobacteria | Alteromonadales | Colwelliaceae | Bacteria;Bacteria\_none;Pseudomonadota;Gammaproteobacteria;Alteromonadales;Colwelliaceae;Thalassotalea |
| Ferrimonas | 0 | 0 | 0 | 0 | 1 | 0 | 0 | 0 | 0 | 0 | 0 | 0 | 0 | 1 | Bacteria | Bacteria\_none | Pseudomonadota | Gammaproteobacteria | Alteromonadales | Ferrimonadaceae | Bacteria;Bacteria\_none;Pseudomonadota;Gammaproteobacteria;Alteromonadales;Ferrimonadaceae;Ferrimonas |
| Flocculibacter | 1 | 0 | 0 | 0 | 0 | 0 | 0 | 0 | 0 | 0 | 0 | 0 | 0 | 1 | Bacteria | Bacteria\_none | Pseudomonadota | Gammaproteobacteria | Alteromonadales | Pseudoalteromonadaceae | Bacteria;Bacteria\_none;Pseudomonadota;Gammaproteobacteria;Alteromonadales;Pseudoalteromonadaceae;Flocculibacter |
| Pseudoalteromonas | 0 | 0 | 0 | 0 | 1 | 2 | 0 | 2 | 0 | 0 | 1 | 0 | 0 | 6 | Bacteria | Bacteria\_none | Pseudomonadota | Gammaproteobacteria | Alteromonadales | Pseudoalteromonadaceae | Bacteria;Bacteria\_none;Pseudomonadota;Gammaproteobacteria;Alteromonadales;Pseudoalteromonadaceae;Pseudoalteromonas |
| Shewanella | 1 | 1 | 0 | 0 | 1 | 0 | 0 | 1 | 0 | 0 | 0 | 1 | 0 | 5 | Bacteria | Bacteria\_none | Pseudomonadota | Gammaproteobacteria | Alteromonadales | Shewanellaceae | Bacteria;Bacteria\_none;Pseudomonadota;Gammaproteobacteria;Alteromonadales;Shewanellaceae;Shewanella |
| Cardiobacterium | 0 | 2 | 14 | 3 | 2 | 6 | 0 | 2 | 0 | 1 | 14 | 2 | 7 | 53 | Bacteria | Bacteria\_none | Pseudomonadota | Gammaproteobacteria | Cardiobacteriales | Cardiobacteriaceae | Bacteria;Bacteria\_none;Pseudomonadota;Gammaproteobacteria;Cardiobacteriales;Cardiobacteriaceae;Cardiobacterium |
| Ignatzschineria | 0 | 1 | 0 | 0 | 0 | 0 | 0 | 0 | 0 | 0 | 0 | 0 | 0 | 1 | Bacteria | Bacteria\_none | Pseudomonadota | Gammaproteobacteria | Cardiobacteriales | Ignatzschineriaceae | Bacteria;Bacteria\_none;Pseudomonadota;Gammaproteobacteria;Cardiobacteriales;Ignatzschineriaceae;Ignatzschineria |
| Agarilytica | 0 | 0 | 0 | 0 | 0 | 0 | 0 | 1 | 0 | 0 | 0 | 0 | 0 | 1 | Bacteria | Bacteria\_none | Pseudomonadota | Gammaproteobacteria | Cellvibrionales | Cellvibrionaceae | Bacteria;Bacteria\_none;Pseudomonadota;Gammaproteobacteria;Cellvibrionales;Cellvibrionaceae;Agarilytica |
| Allochromatium | 0 | 0 | 0 | 0 | 0 | 0 | 0 | 0 | 0 | 0 | 0 | 0 | 1 | 1 | Bacteria | Bacteria\_none | Pseudomonadota | Gammaproteobacteria | Chromatiales | Chromatiaceae | Bacteria;Bacteria\_none;Pseudomonadota;Gammaproteobacteria;Chromatiales;Chromatiaceae;Allochromatium |
| Candidatus Nitrosoglobus | 0 | 0 | 0 | 0 | 0 | 0 | 0 | 0 | 0 | 0 | 0 | 0 | 1 | 1 | Bacteria | Bacteria\_none | Pseudomonadota | Gammaproteobacteria | Chromatiales | Chromatiaceae | Bacteria;Bacteria\_none;Pseudomonadota;Gammaproteobacteria;Chromatiales;Chromatiaceae;Candidatus Nitrosoglobus |
| Thioalkalivibrio | 0 | 0 | 0 | 1 | 0 | 0 | 0 | 0 | 0 | 0 | 0 | 0 | 0 | 1 | Bacteria | Bacteria\_none | Pseudomonadota | Gammaproteobacteria | Chromatiales | Ectothiorhodospiraceae | Bacteria;Bacteria\_none;Pseudomonadota;Gammaproteobacteria;Chromatiales;Ectothiorhodospiraceae;Thioalkalivibrio |
| Limnobaculum | 0 | 0 | 0 | 0 | 0 | 0 | 0 | 1 | 0 | 0 | 0 | 0 | 0 | 1 | Bacteria | Bacteria\_none | Pseudomonadota | Gammaproteobacteria | Enterobacterales | Budviciaceae | Bacteria;Bacteria\_none;Pseudomonadota;Gammaproteobacteria;Enterobacterales;Budviciaceae;Limnobaculum |
| Candidatus Blochmanniella | 0 | 0 | 0 | 0 | 0 | 0 | 0 | 0 | 0 | 1 | 0 | 0 | 0 | 1 | Bacteria | Bacteria\_none | Pseudomonadota | Gammaproteobacteria | Enterobacterales | Enterobacteriaceae | Bacteria;Bacteria\_none;Pseudomonadota;Gammaproteobacteria;Enterobacterales;Enterobacteriaceae;Candidatus Blochmanniella |
| Candidatus Profftia | 0 | 0 | 0 | 0 | 0 | 0 | 0 | 0 | 0 | 1 | 0 | 0 | 0 | 1 | Bacteria | Bacteria\_none | Pseudomonadota | Gammaproteobacteria | Enterobacterales | Enterobacteriaceae | Bacteria;Bacteria\_none;Pseudomonadota;Gammaproteobacteria;Enterobacterales;Enterobacteriaceae;Candidatus Profftia |
| Candidatus Purcelliella | 0 | 0 | 0 | 0 | 0 | 1 | 0 | 0 | 0 | 0 | 0 | 0 | 0 | 1 | Bacteria | Bacteria\_none | Pseudomonadota | Gammaproteobacteria | Enterobacterales | Enterobacteriaceae | Bacteria;Bacteria\_none;Pseudomonadota;Gammaproteobacteria;Enterobacterales;Enterobacteriaceae;Candidatus Purcelliella |
| Enterobacter | 0 | 0 | 0 | 0 | 0 | 0 | 0 | 0 | 0 | 2 | 0 | 0 | 1 | 3 | Bacteria | Bacteria\_none | Pseudomonadota | Gammaproteobacteria | Enterobacterales | Enterobacteriaceae | Bacteria;Bacteria\_none;Pseudomonadota;Gammaproteobacteria;Enterobacterales;Enterobacteriaceae;Enterobacter |
| Enterobacteriaceae\_Incertae\_sedis | 1 | 0 | 0 | 0 | 0 | 0 | 0 | 0 | 0 | 0 | 0 | 0 | 0 | 1 | Bacteria | Bacteria\_none | Pseudomonadota | Gammaproteobacteria | Enterobacterales | Enterobacteriaceae | Bacteria;Bacteria\_none;Pseudomonadota;Gammaproteobacteria;Enterobacterales;Enterobacteriaceae;Enterobacteriaceae\_Incertae\_sedis |
| Escherichia | 1 | 0 | 0 | 0 | 1 | 0 | 0 | 0 | 0 | 0 | 0 | 1 | 2 | 5 | Bacteria | Bacteria\_none | Pseudomonadota | Gammaproteobacteria | Enterobacterales | Enterobacteriaceae | Bacteria;Bacteria\_none;Pseudomonadota;Gammaproteobacteria;Enterobacterales;Enterobacteriaceae;Escherichia |
| Klebsiella | 0 | 0 | 0 | 4 | 4 | 0 | 0 | 0 | 0 | 0 | 0 | 0 | 2 | 10 | Bacteria | Bacteria\_none | Pseudomonadota | Gammaproteobacteria | Enterobacterales | Enterobacteriaceae | Bacteria;Bacteria\_none;Pseudomonadota;Gammaproteobacteria;Enterobacterales;Enterobacteriaceae;Klebsiella |
| Salmonella | 2 | 0 | 0 | 0 | 0 | 0 | 0 | 4 | 0 | 0 | 0 | 0 | 6 | 12 | Bacteria | Bacteria\_none | Pseudomonadota | Gammaproteobacteria | Enterobacterales | Enterobacteriaceae | Bacteria;Bacteria\_none;Pseudomonadota;Gammaproteobacteria;Enterobacterales;Enterobacteriaceae;Salmonella |
| Symbiopectobacterium | 0 | 0 | 0 | 0 | 0 | 0 | 0 | 0 | 0 | 0 | 0 | 0 | 1 | 1 | Bacteria | Bacteria\_none | Pseudomonadota | Gammaproteobacteria | Enterobacterales | Enterobacteriaceae | Bacteria;Bacteria\_none;Pseudomonadota;Gammaproteobacteria;Enterobacterales;Enterobacteriaceae;Symbiopectobacterium |
| Buchnera | 0 | 0 | 0 | 0 | 0 | 0 | 0 | 1 | 0 | 0 | 0 | 0 | 2 | 3 | Bacteria | Bacteria\_none | Pseudomonadota | Gammaproteobacteria | Enterobacterales | Erwiniaceae | Bacteria;Bacteria\_none;Pseudomonadota;Gammaproteobacteria;Enterobacterales;Erwiniaceae;Buchnera |
| Erwinia | 0 | 0 | 0 | 0 | 1 | 0 | 0 | 1 | 0 | 0 | 0 | 1 | 0 | 3 | Bacteria | Bacteria\_none | Pseudomonadota | Gammaproteobacteria | Enterobacterales | Erwiniaceae | Bacteria;Bacteria\_none;Pseudomonadota;Gammaproteobacteria;Enterobacterales;Erwiniaceae;Erwinia |
| Pantoea | 0 | 0 | 0 | 0 | 0 | 0 | 0 | 0 | 0 | 0 | 1 | 0 | 0 | 1 | Bacteria | Bacteria\_none | Pseudomonadota | Gammaproteobacteria | Enterobacterales | Erwiniaceae | Bacteria;Bacteria\_none;Pseudomonadota;Gammaproteobacteria;Enterobacterales;Erwiniaceae;Pantoea |
| Tatumella | 0 | 0 | 0 | 0 | 0 | 0 | 0 | 1 | 0 | 0 | 0 | 0 | 0 | 1 | Bacteria | Bacteria\_none | Pseudomonadota | Gammaproteobacteria | Enterobacterales | Erwiniaceae | Bacteria;Bacteria\_none;Pseudomonadota;Gammaproteobacteria;Enterobacterales;Erwiniaceae;Tatumella |
| Arsenophonus | 0 | 0 | 0 | 0 | 0 | 0 | 0 | 0 | 1 | 0 | 0 | 0 | 0 | 1 | Bacteria | Bacteria\_none | Pseudomonadota | Gammaproteobacteria | Enterobacterales | Morganellaceae | Bacteria;Bacteria\_none;Pseudomonadota;Gammaproteobacteria;Enterobacterales;Morganellaceae;Arsenophonus |
| Morganella | 0 | 0 | 0 | 1 | 0 | 0 | 0 | 0 | 0 | 0 | 0 | 0 | 1 | 2 | Bacteria | Bacteria\_none | Pseudomonadota | Gammaproteobacteria | Enterobacterales | Morganellaceae | Bacteria;Bacteria\_none;Pseudomonadota;Gammaproteobacteria;Enterobacterales;Morganellaceae;Morganella |
| Proteus | 1 | 0 | 1 | 0 | 0 | 1 | 0 | 4 | 0 | 0 | 0 | 1 | 0 | 8 | Bacteria | Bacteria\_none | Pseudomonadota | Gammaproteobacteria | Enterobacterales | Morganellaceae | Bacteria;Bacteria\_none;Pseudomonadota;Gammaproteobacteria;Enterobacterales;Morganellaceae;Proteus |
| Providencia | 0 | 0 | 0 | 0 | 1 | 1 | 0 | 1 | 0 | 0 | 0 | 0 | 0 | 3 | Bacteria | Bacteria\_none | Pseudomonadota | Gammaproteobacteria | Enterobacterales | Morganellaceae | Bacteria;Bacteria\_none;Pseudomonadota;Gammaproteobacteria;Enterobacterales;Morganellaceae;Providencia |
| Xenorhabdus | 0 | 0 | 0 | 0 | 0 | 0 | 1 | 0 | 0 | 0 | 0 | 0 | 1 | 2 | Bacteria | Bacteria\_none | Pseudomonadota | Gammaproteobacteria | Enterobacterales | Morganellaceae | Bacteria;Bacteria\_none;Pseudomonadota;Gammaproteobacteria;Enterobacterales;Morganellaceae;Xenorhabdus |
| Dickeya | 0 | 0 | 0 | 0 | 1 | 0 | 0 | 0 | 0 | 0 | 0 | 0 | 0 | 1 | Bacteria | Bacteria\_none | Pseudomonadota | Gammaproteobacteria | Enterobacterales | Pectobacteriaceae | Bacteria;Bacteria\_none;Pseudomonadota;Gammaproteobacteria;Enterobacterales;Pectobacteriaceae;Dickeya |
| Serratia | 0 | 0 | 0 | 0 | 0 | 0 | 0 | 0 | 0 | 0 | 1 | 0 | 0 | 1 | Bacteria | Bacteria\_none | Pseudomonadota | Gammaproteobacteria | Enterobacterales | Yersiniaceae | Bacteria;Bacteria\_none;Pseudomonadota;Gammaproteobacteria;Enterobacterales;Yersiniaceae;Serratia |
| Legionella | 0 | 0 | 0 | 1 | 0 | 0 | 0 | 0 | 0 | 0 | 0 | 0 | 1 | 2 | Bacteria | Bacteria\_none | Pseudomonadota | Gammaproteobacteria | Legionellales | Legionellaceae | Bacteria;Bacteria\_none;Pseudomonadota;Gammaproteobacteria;Legionellales;Legionellaceae;Legionella |
| Lysobacter | 0 | 0 | 0 | 1 | 0 | 0 | 0 | 0 | 0 | 0 | 0 | 0 | 0 | 1 | Bacteria | Bacteria\_none | Pseudomonadota | Gammaproteobacteria | Lysobacterales | Lysobacteraceae | Bacteria;Bacteria\_none;Pseudomonadota;Gammaproteobacteria;Lysobacterales;Lysobacteraceae;Lysobacter |
| Pseudoxanthomonas | 0 | 0 | 0 | 0 | 0 | 0 | 0 | 0 | 0 | 0 | 1 | 0 | 0 | 1 | Bacteria | Bacteria\_none | Pseudomonadota | Gammaproteobacteria | Lysobacterales | Lysobacteraceae | Bacteria;Bacteria\_none;Pseudomonadota;Gammaproteobacteria;Lysobacterales;Lysobacteraceae;Pseudoxanthomonas |
| Stenotrophomonas | 0 | 0 | 0 | 0 | 2 | 0 | 0 | 0 | 0 | 0 | 0 | 0 | 0 | 2 | Bacteria | Bacteria\_none | Pseudomonadota | Gammaproteobacteria | Lysobacterales | Lysobacteraceae | Bacteria;Bacteria\_none;Pseudomonadota;Gammaproteobacteria;Lysobacterales;Lysobacteraceae;Stenotrophomonas |
| Xanthomonas | 0 | 0 | 0 | 0 | 0 | 0 | 0 | 0 | 0 | 0 | 0 | 0 | 1 | 1 | Bacteria | Bacteria\_none | Pseudomonadota | Gammaproteobacteria | Lysobacterales | Lysobacteraceae | Bacteria;Bacteria\_none;Pseudomonadota;Gammaproteobacteria;Lysobacterales;Lysobacteraceae;Xanthomonas |
| Xylella | 0 | 0 | 0 | 0 | 0 | 0 | 0 | 0 | 0 | 0 | 0 | 0 | 1 | 1 | Bacteria | Bacteria\_none | Pseudomonadota | Gammaproteobacteria | Lysobacterales | Lysobacteraceae | Bacteria;Bacteria\_none;Pseudomonadota;Gammaproteobacteria;Lysobacterales;Lysobacteraceae;Xylella |
| Dyella | 0 | 0 | 0 | 0 | 0 | 0 | 0 | 0 | 0 | 0 | 0 | 1 | 0 | 1 | Bacteria | Bacteria\_none | Pseudomonadota | Gammaproteobacteria | Lysobacterales | Rhodanobacteraceae | Bacteria;Bacteria\_none;Pseudomonadota;Gammaproteobacteria;Lysobacterales;Rhodanobacteraceae;Dyella |
| Tahibacter | 0 | 0 | 0 | 0 | 0 | 0 | 0 | 0 | 0 | 1 | 0 | 0 | 0 | 1 | Bacteria | Bacteria\_none | Pseudomonadota | Gammaproteobacteria | Lysobacterales | Rhodanobacteraceae | Bacteria;Bacteria\_none;Pseudomonadota;Gammaproteobacteria;Lysobacterales;Rhodanobacteraceae;Tahibacter |
| Candidatus Methylospira | 0 | 0 | 0 | 0 | 0 | 0 | 0 | 0 | 0 | 0 | 0 | 0 | 1 | 1 | Bacteria | Bacteria\_none | Pseudomonadota | Gammaproteobacteria | Methylococcales | Methylococcaceae | Bacteria;Bacteria\_none;Pseudomonadota;Gammaproteobacteria;Methylococcales;Methylococcaceae;Candidatus Methylospira |
| Methylobacter | 0 | 0 | 0 | 0 | 0 | 0 | 0 | 0 | 0 | 0 | 0 | 0 | 2 | 2 | Bacteria | Bacteria\_none | Pseudomonadota | Gammaproteobacteria | Methylococcales | Methylococcaceae | Bacteria;Bacteria\_none;Pseudomonadota;Gammaproteobacteria;Methylococcales;Methylococcaceae;Methylobacter |
| Methylomonas | 0 | 0 | 0 | 0 | 0 | 1 | 0 | 0 | 0 | 0 | 0 | 0 | 0 | 1 | Bacteria | Bacteria\_none | Pseudomonadota | Gammaproteobacteria | Methylococcales | Methylococcaceae | Bacteria;Bacteria\_none;Pseudomonadota;Gammaproteobacteria;Methylococcales;Methylococcaceae;Methylomonas |
| Methylotuvimicrobium | 0 | 0 | 0 | 0 | 0 | 0 | 0 | 0 | 0 | 0 | 0 | 0 | 1 | 1 | Bacteria | Bacteria\_none | Pseudomonadota | Gammaproteobacteria | Methylococcales | Methylococcaceae | Bacteria;Bacteria\_none;Pseudomonadota;Gammaproteobacteria;Methylococcales;Methylococcaceae;Methylotuvimicrobium |
| Acinetobacter | 0 | 0 | 0 | 1 | 0 | 2 | 0 | 2 | 0 | 2 | 0 | 0 | 2 | 9 | Bacteria | Bacteria\_none | Pseudomonadota | Gammaproteobacteria | Moraxellales | Moraxellaceae | Bacteria;Bacteria\_none;Pseudomonadota;Gammaproteobacteria;Moraxellales;Moraxellaceae;Acinetobacter |
| Moraxella | 0 | 0 | 0 | 1 | 0 | 0 | 0 | 0 | 0 | 0 | 0 | 0 | 3 | 4 | Bacteria | Bacteria\_none | Pseudomonadota | Gammaproteobacteria | Moraxellales | Moraxellaceae | Bacteria;Bacteria\_none;Pseudomonadota;Gammaproteobacteria;Moraxellales;Moraxellaceae;Moraxella |
| Psychrobacter | 0 | 0 | 1 | 0 | 0 | 0 | 0 | 2 | 0 | 0 | 0 | 2 | 0 | 5 | Bacteria | Bacteria\_none | Pseudomonadota | Gammaproteobacteria | Moraxellales | Moraxellaceae | Bacteria;Bacteria\_none;Pseudomonadota;Gammaproteobacteria;Moraxellales;Moraxellaceae;Psychrobacter |
| Alcanivorax | 0 | 0 | 0 | 0 | 0 | 0 | 0 | 1 | 0 | 0 | 0 | 0 | 0 | 1 | Bacteria | Bacteria\_none | Pseudomonadota | Gammaproteobacteria | Oceanospirillales | Alcanivoracaceae | Bacteria;Bacteria\_none;Pseudomonadota;Gammaproteobacteria;Oceanospirillales;Alcanivoracaceae;Alcanivorax |
| Halomonas | 0 | 0 | 0 | 0 | 1 | 1 | 0 | 1 | 0 | 0 | 0 | 0 | 0 | 3 | Bacteria | Bacteria\_none | Pseudomonadota | Gammaproteobacteria | Oceanospirillales | Halomonadaceae | Bacteria;Bacteria\_none;Pseudomonadota;Gammaproteobacteria;Oceanospirillales;Halomonadaceae;Halomonas |
| Amphritea | 0 | 0 | 0 | 0 | 0 | 0 | 0 | 1 | 0 | 0 | 0 | 0 | 0 | 1 | Bacteria | Bacteria\_none | Pseudomonadota | Gammaproteobacteria | Oceanospirillales | Oceanospirillaceae | Bacteria;Bacteria\_none;Pseudomonadota;Gammaproteobacteria;Oceanospirillales;Oceanospirillaceae;Amphritea |
| Marinomonas | 0 | 0 | 0 | 0 | 0 | 1 | 0 | 1 | 0 | 0 | 0 | 0 | 1 | 3 | Bacteria | Bacteria\_none | Pseudomonadota | Gammaproteobacteria | Oceanospirillales | Oceanospirillaceae | Bacteria;Bacteria\_none;Pseudomonadota;Gammaproteobacteria;Oceanospirillales;Oceanospirillaceae;Marinomonas |
| Thalassolituus | 0 | 1 | 0 | 0 | 0 | 0 | 0 | 0 | 0 | 0 | 0 | 0 | 0 | 1 | Bacteria | Bacteria\_none | Pseudomonadota | Gammaproteobacteria | Oceanospirillales | Oceanospirillaceae | Bacteria;Bacteria\_none;Pseudomonadota;Gammaproteobacteria;Oceanospirillales;Oceanospirillaceae;Thalassolituus |
| Gilliamella | 0 | 0 | 0 | 0 | 0 | 0 | 0 | 1 | 0 | 0 | 0 | 0 | 1 | 2 | Bacteria | Bacteria\_none | Pseudomonadota | Gammaproteobacteria | Orbales | Orbaceae | Bacteria;Bacteria\_none;Pseudomonadota;Gammaproteobacteria;Orbales;Orbaceae;Gilliamella |
| Actinobacillus | 0 | 0 | 1 | 0 | 2 | 0 | 0 | 36 | 0 | 0 | 0 | 0 | 4 | 43 | Bacteria | Bacteria\_none | Pseudomonadota | Gammaproteobacteria | Pasteurellales | Pasteurellaceae | Bacteria;Bacteria\_none;Pseudomonadota;Gammaproteobacteria;Pasteurellales;Pasteurellaceae;Actinobacillus |
| Aggregatibacter | 0 | 36 | 12 | 193 | 88 | 49 | 0 | 320 | 0 | 11 | 0 | 20 | 167 | 896 | Bacteria | Bacteria\_none | Pseudomonadota | Gammaproteobacteria | Pasteurellales | Pasteurellaceae | Bacteria;Bacteria\_none;Pseudomonadota;Gammaproteobacteria;Pasteurellales;Pasteurellaceae;Aggregatibacter |
| Avibacterium | 0 | 0 | 0 | 0 | 1 | 1 | 0 | 2 | 0 | 0 | 0 | 0 | 0 | 4 | Bacteria | Bacteria\_none | Pseudomonadota | Gammaproteobacteria | Pasteurellales | Pasteurellaceae | Bacteria;Bacteria\_none;Pseudomonadota;Gammaproteobacteria;Pasteurellales;Pasteurellaceae;Avibacterium |
| Bibersteinia | 0 | 0 | 0 | 0 | 0 | 0 | 0 | 1 | 0 | 0 | 0 | 0 | 0 | 1 | Bacteria | Bacteria\_none | Pseudomonadota | Gammaproteobacteria | Pasteurellales | Pasteurellaceae | Bacteria;Bacteria\_none;Pseudomonadota;Gammaproteobacteria;Pasteurellales;Pasteurellaceae;Bibersteinia |
| Bisgaardia | 0 | 0 | 0 | 0 | 0 | 0 | 0 | 1 | 0 | 0 | 0 | 0 | 0 | 1 | Bacteria | Bacteria\_none | Pseudomonadota | Gammaproteobacteria | Pasteurellales | Pasteurellaceae | Bacteria;Bacteria\_none;Pseudomonadota;Gammaproteobacteria;Pasteurellales;Pasteurellaceae;Bisgaardia |
| Frederiksenia | 0 | 0 | 0 | 0 | 0 | 0 | 0 | 0 | 0 | 0 | 0 | 0 | 1 | 1 | Bacteria | Bacteria\_none | Pseudomonadota | Gammaproteobacteria | Pasteurellales | Pasteurellaceae | Bacteria;Bacteria\_none;Pseudomonadota;Gammaproteobacteria;Pasteurellales;Pasteurellaceae;Frederiksenia |
| Gallibacterium | 0 | 0 | 0 | 0 | 0 | 2 | 0 | 1 | 0 | 0 | 0 | 0 | 0 | 3 | Bacteria | Bacteria\_none | Pseudomonadota | Gammaproteobacteria | Pasteurellales | Pasteurellaceae | Bacteria;Bacteria\_none;Pseudomonadota;Gammaproteobacteria;Pasteurellales;Pasteurellaceae;Gallibacterium |
| Glaesserella | 0 | 1 | 0 | 0 | 0 | 0 | 1 | 3 | 0 | 0 | 0 | 0 | 0 | 5 | Bacteria | Bacteria\_none | Pseudomonadota | Gammaproteobacteria | Pasteurellales | Pasteurellaceae | Bacteria;Bacteria\_none;Pseudomonadota;Gammaproteobacteria;Pasteurellales;Pasteurellaceae;Glaesserella |
| Haemophilus | 71 | 105 | 452 | 579 | 193 | 191 | 6 | 1521 | 0 | 80 | 49 | 224 | 1055 | 4526 | Bacteria | Bacteria\_none | Pseudomonadota | Gammaproteobacteria | Pasteurellales | Pasteurellaceae | Bacteria;Bacteria\_none;Pseudomonadota;Gammaproteobacteria;Pasteurellales;Pasteurellaceae;Haemophilus |
| Histophilus | 0 | 0 | 0 | 0 | 0 | 0 | 0 | 1 | 0 | 0 | 0 | 0 | 0 | 1 | Bacteria | Bacteria\_none | Pseudomonadota | Gammaproteobacteria | Pasteurellales | Pasteurellaceae | Bacteria;Bacteria\_none;Pseudomonadota;Gammaproteobacteria;Pasteurellales;Pasteurellaceae;Histophilus |
| Mannheimia | 0 | 0 | 0 | 0 | 1 | 0 | 0 | 20 | 0 | 0 | 0 | 0 | 4 | 25 | Bacteria | Bacteria\_none | Pseudomonadota | Gammaproteobacteria | Pasteurellales | Pasteurellaceae | Bacteria;Bacteria\_none;Pseudomonadota;Gammaproteobacteria;Pasteurellales;Pasteurellaceae;Mannheimia |
| Mergibacter | 0 | 0 | 0 | 0 | 0 | 0 | 0 | 1 | 0 | 0 | 0 | 0 | 1 | 2 | Bacteria | Bacteria\_none | Pseudomonadota | Gammaproteobacteria | Pasteurellales | Pasteurellaceae | Bacteria;Bacteria\_none;Pseudomonadota;Gammaproteobacteria;Pasteurellales;Pasteurellaceae;Mergibacter |
| Otariodibacter | 0 | 0 | 0 | 0 | 0 | 0 | 0 | 1 | 0 | 0 | 0 | 0 | 2 | 3 | Bacteria | Bacteria\_none | Pseudomonadota | Gammaproteobacteria | Pasteurellales | Pasteurellaceae | Bacteria;Bacteria\_none;Pseudomonadota;Gammaproteobacteria;Pasteurellales;Pasteurellaceae;Otariodibacter |
| Pasteurella | 0 | 0 | 1 | 1 | 0 | 1 | 1 | 10 | 0 | 1 | 0 | 0 | 3 | 18 | Bacteria | Bacteria\_none | Pseudomonadota | Gammaproteobacteria | Pasteurellales | Pasteurellaceae | Bacteria;Bacteria\_none;Pseudomonadota;Gammaproteobacteria;Pasteurellales;Pasteurellaceae;Pasteurella |
| Rodentibacter | 0 | 0 | 0 | 0 | 0 | 0 | 0 | 3 | 0 | 0 | 0 | 0 | 0 | 3 | Bacteria | Bacteria\_none | Pseudomonadota | Gammaproteobacteria | Pasteurellales | Pasteurellaceae | Bacteria;Bacteria\_none;Pseudomonadota;Gammaproteobacteria;Pasteurellales;Pasteurellaceae;Rodentibacter |
| Vespertiliibacter | 0 | 0 | 0 | 0 | 0 | 0 | 0 | 1 | 0 | 0 | 0 | 0 | 0 | 1 | Bacteria | Bacteria\_none | Pseudomonadota | Gammaproteobacteria | Pasteurellales | Pasteurellaceae | Bacteria;Bacteria\_none;Pseudomonadota;Gammaproteobacteria;Pasteurellales;Pasteurellaceae;Vespertiliibacter |
| Pseudomonas | 0 | 3 | 0 | 0 | 2 | 3 | 1 | 1 | 0 | 0 | 2 | 0 | 4 | 16 | Bacteria | Bacteria\_none | Pseudomonadota | Gammaproteobacteria | Pseudomonadales | Pseudomonadaceae | Bacteria;Bacteria\_none;Pseudomonadota;Gammaproteobacteria;Pseudomonadales;Pseudomonadaceae;Pseudomonas |
| Thiomicrorhabdus | 0 | 0 | 0 | 0 | 1 | 0 | 0 | 0 | 0 | 0 | 0 | 0 | 0 | 1 | Bacteria | Bacteria\_none | Pseudomonadota | Gammaproteobacteria | Thiotrichales | Piscirickettsiaceae | Bacteria;Bacteria\_none;Pseudomonadota;Gammaproteobacteria;Thiotrichales;Piscirickettsiaceae;Thiomicrorhabdus |
| Aliivibrio | 0 | 0 | 0 | 0 | 0 | 0 | 0 | 0 | 0 | 0 | 0 | 0 | 1 | 1 | Bacteria | Bacteria\_none | Pseudomonadota | Gammaproteobacteria | Vibrionales | Vibrionaceae | Bacteria;Bacteria\_none;Pseudomonadota;Gammaproteobacteria;Vibrionales;Vibrionaceae;Aliivibrio |
| Enterovibrio | 0 | 0 | 0 | 0 | 0 | 0 | 0 | 1 | 0 | 0 | 0 | 0 | 0 | 1 | Bacteria | Bacteria\_none | Pseudomonadota | Gammaproteobacteria | Vibrionales | Vibrionaceae | Bacteria;Bacteria\_none;Pseudomonadota;Gammaproteobacteria;Vibrionales;Vibrionaceae;Enterovibrio |
| Thaumasiovibrio | 0 | 0 | 0 | 0 | 0 | 0 | 0 | 1 | 0 | 0 | 0 | 0 | 0 | 1 | Bacteria | Bacteria\_none | Pseudomonadota | Gammaproteobacteria | Vibrionales | Vibrionaceae | Bacteria;Bacteria\_none;Pseudomonadota;Gammaproteobacteria;Vibrionales;Vibrionaceae;Thaumasiovibrio |
| Vibrio | 0 | 0 | 3 | 0 | 2 | 1 | 0 | 3 | 0 | 0 | 0 | 0 | 3 | 12 | Bacteria | Bacteria\_none | Pseudomonadota | Gammaproteobacteria | Vibrionales | Vibrionaceae | Bacteria;Bacteria\_none;Pseudomonadota;Gammaproteobacteria;Vibrionales;Vibrionaceae;Vibrio |
| Leptospira | 1 | 0 | 0 | 0 | 0 | 0 | 0 | 0 | 0 | 0 | 0 | 1 | 0 | 2 | Bacteria | Bacteria\_none | Spirochaetota | Spirochaetia | Leptospirales | Leptospiraceae | Bacteria;Bacteria\_none;Spirochaetota;Spirochaetia;Leptospirales;Leptospiraceae;Leptospira |
| Borreliella | 0 | 0 | 0 | 0 | 0 | 1 | 0 | 0 | 0 | 0 | 0 | 0 | 0 | 1 | Bacteria | Bacteria\_none | Spirochaetota | Spirochaetia | Spirochaetales | Borreliaceae | Bacteria;Bacteria\_none;Spirochaetota;Spirochaetia;Spirochaetales;Borreliaceae;Borreliella |
| Breznakiella | 0 | 1 | 0 | 0 | 0 | 0 | 0 | 0 | 0 | 0 | 0 | 0 | 0 | 1 | Bacteria | Bacteria\_none | Spirochaetota | Spirochaetia | Spirochaetales | Breznakiellaceae | Bacteria;Bacteria\_none;Spirochaetota;Spirochaetia;Spirochaetales;Breznakiellaceae;Breznakiella |
| Sphaerochaeta | 0 | 1 | 0 | 0 | 0 | 0 | 0 | 0 | 0 | 0 | 0 | 0 | 0 | 1 | Bacteria | Bacteria\_none | Spirochaetota | Spirochaetia | Spirochaetales | Sphaerochaetaceae | Bacteria;Bacteria\_none;Spirochaetota;Spirochaetia;Spirochaetales;Sphaerochaetaceae;Sphaerochaeta |
| Treponema | 3 | 72 | 54 | 9 | 40 | 73 | 9 | 112 | 0 | 14 | 56 | 119 | 143 | 704 | Bacteria | Bacteria\_none | Spirochaetota | Spirochaetia | Spirochaetales | Treponemataceae | Bacteria;Bacteria\_none;Spirochaetota;Spirochaetia;Spirochaetales;Treponemataceae;Treponema |
| Fretibacterium | 0 | 0 | 1 | 0 | 0 | 0 | 0 | 0 | 0 | 0 | 1 | 1 | 0 | 3 | Bacteria | Bacteria\_none | Synergistota | Synergistia | Synergistales | Aminobacteriaceae | Bacteria;Bacteria\_none;Synergistota;Synergistia;Synergistales;Aminobacteriaceae;Fretibacterium |
| Candidatus Desulfofervidus | 0 | 0 | 0 | 0 | 0 | 1 | 0 | 0 | 0 | 0 | 0 | 0 | 0 | 1 | Bacteria | Bacteria\_none | Thermodesulfobacteriota | Candidatus Desulfofervidia | Candidatus Desulfofervidales | Candidatus Desulfofervidaceae | Bacteria;Bacteria\_none;Thermodesulfobacteriota;Candidatus Desulfofervidia;Candidatus Desulfofervidales;Candidatus Desulfofervidaceae;Candidatus Desulfofervidus |
| Desulfoferula | 0 | 0 | 0 | 0 | 0 | 1 | 0 | 0 | 0 | 0 | 0 | 0 | 0 | 1 | Bacteria | Bacteria\_none | Thermodesulfobacteriota | Desulfarculia | Desulfarculales | Desulfarculaceae | Bacteria;Bacteria\_none;Thermodesulfobacteriota;Desulfarculia;Desulfarculales;Desulfarculaceae;Desulfoferula |
| Desulfobulbus | 0 | 2 | 1 | 0 | 0 | 0 | 0 | 5 | 0 | 0 | 0 | 0 | 1 | 9 | Bacteria | Bacteria\_none | Thermodesulfobacteriota | Desulfobulbia | Desulfobulbales | Desulfobulbaceae | Bacteria;Bacteria\_none;Thermodesulfobacteriota;Desulfobulbia;Desulfobulbales;Desulfobulbaceae;Desulfobulbus |
| Lawsonia | 0 | 0 | 0 | 0 | 0 | 0 | 0 | 0 | 0 | 0 | 0 | 1 | 0 | 1 | Bacteria | Bacteria\_none | Thermodesulfobacteriota | Desulfovibrionia | Desulfovibrionales | Desulfovibrionaceae | Bacteria;Bacteria\_none;Thermodesulfobacteriota;Desulfovibrionia;Desulfovibrionales;Desulfovibrionaceae;Lawsonia |
| Syntrophotalea | 0 | 0 | 0 | 0 | 0 | 0 | 0 | 0 | 0 | 0 | 1 | 0 | 0 | 1 | Bacteria | Bacteria\_none | Thermodesulfobacteriota | Desulfuromonadia | Desulfuromonadales | Syntrophotaleaceae | Bacteria;Bacteria\_none;Thermodesulfobacteriota;Desulfuromonadia;Desulfuromonadales;Syntrophotaleaceae;Syntrophotalea |
| Geobacter | 0 | 0 | 1 | 0 | 0 | 0 | 0 | 0 | 0 | 0 | 0 | 0 | 0 | 1 | Bacteria | Bacteria\_none | Thermodesulfobacteriota | Desulfuromonadia | Geobacterales | Geobacteraceae | Bacteria;Bacteria\_none;Thermodesulfobacteriota;Desulfuromonadia;Geobacterales;Geobacteraceae;Geobacter |
| Thermomicrobium | 0 | 1 | 0 | 0 | 0 | 0 | 0 | 0 | 0 | 0 | 0 | 0 | 0 | 1 | Bacteria | Bacteria\_none | Thermomicrobiota | Thermomicrobia | Thermomicrobiales | Thermomicrobiaceae | Bacteria;Bacteria\_none;Thermomicrobiota;Thermomicrobia;Thermomicrobiales;Thermomicrobiaceae;Thermomicrobium |
| Thermosipho | 1 | 0 | 0 | 0 | 0 | 0 | 0 | 0 | 0 | 0 | 0 | 0 | 0 | 1 | Bacteria | Bacteria\_none | Thermotogota | Thermotogae | Thermotogales | Fervidobacteriaceae | Bacteria;Bacteria\_none;Thermotogota;Thermotogae;Thermotogales;Fervidobacteriaceae;Thermosipho |
| Lacunisphaera | 0 | 1 | 0 | 0 | 0 | 0 | 0 | 0 | 0 | 0 | 0 | 0 | 0 | 1 | Bacteria | Bacteria\_none | Verrucomicrobiota | Opitutia | Opitutales | Opitutaceae | Bacteria;Bacteria\_none;Verrucomicrobiota;Opitutia;Opitutales;Opitutaceae;Lacunisphaera |
| Homo | 428 | 938 | 1105 | 97 | 2901 | 211 | 12043 | 17730 | 14114 | 5069 | 5655 | 9919 | 34174 | 104384 | Eukaryota | Metazoa | Chordata | Mammalia | Primates | Hominidae | Eukaryota;Metazoa;Chordata;Mammalia;Primates;Hominidae;Homo |
| Unknown | 1409 | 1058 | 1350 | 2756 | 1867 | 2605 | 1040 | 1418 | 519 | 1446 | 1552 | 1325 | 24901 | 43246 | Unclassified | Unknown | Unknown | Unknown | Unknown | Unknown | Unclassified;Unknown;Unknown;Unknown;Unknown;Unknown;Unknown |
| Mimivirus | 0 | 0 | 0 | 0 | 0 | 0 | 0 | 0 | 0 | 0 | 0 | 1 | 0 | 1 | Viruses | Bamfordvirae | Nucleocytoviricota | Megaviricetes | Imitervirales | Mimiviridae | Viruses;Bamfordvirae;Nucleocytoviricota;Megaviricetes;Imitervirales;Mimiviridae;Mimivirus |
| Theiavirus | 0 | 0 | 0 | 0 | 0 | 0 | 0 | 1 | 0 | 0 | 0 | 0 | 0 | 1 | Viruses | Bamfordvirae | Nucleocytoviricota | Megaviricetes | Imitervirales | Mimiviridae | Viruses;Bamfordvirae;Nucleocytoviricota;Megaviricetes;Imitervirales;Mimiviridae;Theiavirus |
| Biavirus | 0 | 0 | 0 | 0 | 0 | 0 | 0 | 0 | 0 | 0 | 0 | 0 | 1 | 1 | Viruses | Bamfordvirae | Nucleocytoviricota | Megaviricetes | Imitervirales | Schizomimiviridae | Viruses;Bamfordvirae;Nucleocytoviricota;Megaviricetes;Imitervirales;Schizomimiviridae;Biavirus |
| Lymphocryptovirus | 8 | 0 | 0 | 0 | 0 | 1 | 0 | 1 | 0 | 0 | 0 | 0 | 2 | 12 | Viruses | Heunggongvirae | Peploviricota | Herviviricetes | Herpesvirales | Orthoherpesviridae | Viruses;Heunggongvirae;Peploviricota;Herviviricetes;Herpesvirales;Orthoherpesviridae;Lymphocryptovirus |
| Caudoviricetes\_Incertae\_sedis | 5 | 0 | 0 | 1 | 3 | 1 | 5 | 0 | 0 | 0 | 2 | 1 | 2 | 20 | Viruses | Heunggongvirae | Uroviricota | Caudoviricetes | Caudoviricetes\_Incertae\_sedis | Caudoviricetes\_Incertae\_sedis | Viruses;Heunggongvirae;Uroviricota;Caudoviricetes;Caudoviricetes\_Incertae\_sedis;Caudoviricetes\_Incertae\_sedis;Caudoviricetes\_Incertae\_sedis |
| Whopevirus | 2 | 0 | 0 | 0 | 0 | 0 | 0 | 0 | 0 | 0 | 0 | 0 | 0 | 2 | Viruses | Heunggongvirae | Uroviricota | Caudoviricetes | Crassvirales | Intestiviridae | Viruses;Heunggongvirae;Uroviricota;Caudoviricetes;Crassvirales;Intestiviridae;Whopevirus |
| Cytorhabdovirus | 0 | 0 | 0 | 0 | 0 | 0 | 0 | 0 | 0 | 0 | 0 | 0 | 1 | 1 | Viruses | Orthornavirae | Negarnaviricota | Monjiviricetes | Mononegavirales | Rhabdoviridae | Viruses;Orthornavirae;Negarnaviricota;Monjiviricetes;Mononegavirales;Rhabdoviridae;Cytorhabdovirus |
| Alphapapillomavirus | 0 | 0 | 0 | 0 | 0 | 0 | 0 | 2 | 0 | 0 | 0 | 0 | 0 | 2 | Viruses | Shotokuvirae | Cossaviricota | Papovaviricetes | Zurhausenvirales | Papillomaviridae | Viruses;Shotokuvirae;Cossaviricota;Papovaviricetes;Zurhausenvirales;Papillomaviridae;Alphapapillomavirus |
| Alphalipothrixvirus | 0 | 0 | 0 | 0 | 0 | 0 | 0 | 0 | 0 | 0 | 0 | 0 | 1 | 1 | Viruses | Zilligvirae | Taleaviricota | Tokiviricetes | Ligamenvirales | Lipothrixviridae | Viruses;Zilligvirae;Taleaviricota;Tokiviricetes;Ligamenvirales;Lipothrixviridae;Alphalipothrixvirus |

Export CSV

Abundance table for the species rank.

Only taxa whose global abundance are above the
`abundance_threshold` parameter will appear in the table.

| species | barcode01 | barcode02 | barcode03 | barcode04 | barcode05 | barcode06 | barcode07 | barcode08 | barcode09 | barcode10 | barcode11 | barcode12 | unclassified | total | superkingdom | kingdom | phylum | class | order | family | genus | tax |
| --- | --- | --- | --- | --- | --- | --- | --- | --- | --- | --- | --- | --- | --- | --- | --- | --- | --- | --- | --- | --- | --- | --- |
| Haladaptatus salinisoli | 0 | 1 | 0 | 0 | 0 | 0 | 0 | 0 | 0 | 0 | 0 | 0 | 0 | 1 | Archaea | Archaea\_none | Euryarchaeota | Halobacteria | Halobacteriales | Haladaptataceae | Haladaptatus | Archaea;Archaea\_none;Euryarchaeota;Halobacteria;Halobacteriales;Haladaptataceae;Haladaptatus;Haladaptatus salinisoli |
| Halococcoides cellulosivorans | 0 | 0 | 0 | 0 | 0 | 0 | 0 | 0 | 0 | 0 | 1 | 0 | 0 | 1 | Archaea | Archaea\_none | Euryarchaeota | Halobacteria | Halobacteriales | Haloarculaceae | Halococcoides | Archaea;Archaea\_none;Euryarchaeota;Halobacteria;Halobacteriales;Haloarculaceae;Halococcoides;Halococcoides cellulosivorans |
| Halospeciosus flavus | 1 | 0 | 0 | 0 | 0 | 0 | 0 | 0 | 0 | 0 | 0 | 0 | 0 | 1 | Archaea | Archaea\_none | Euryarchaeota | Halobacteria | Halobacteriales | Halobacteriaceae | Halospeciosus | Archaea;Archaea\_none;Euryarchaeota;Halobacteria;Halobacteriales;Halobacteriaceae;Halospeciosus;Halospeciosus flavus |
| Halobaculum halobium | 0 | 0 | 0 | 0 | 0 | 0 | 1 | 0 | 0 | 0 | 0 | 0 | 0 | 1 | Archaea | Archaea\_none | Euryarchaeota | Halobacteria | Halobacteriales | Haloferacaceae | Halobaculum | Archaea;Archaea\_none;Euryarchaeota;Halobacteria;Halobacteriales;Haloferacaceae;Halobaculum;Halobaculum halobium |
| Halogeometricum borinquense | 0 | 0 | 0 | 0 | 0 | 1 | 0 | 0 | 0 | 0 | 0 | 0 | 0 | 1 | Archaea | Archaea\_none | Euryarchaeota | Halobacteria | Halobacteriales | Haloferacaceae | Halogeometricum | Archaea;Archaea\_none;Euryarchaeota;Halobacteria;Halobacteriales;Haloferacaceae;Halogeometricum;Halogeometricum borinquense |
| Halorarum salinum | 0 | 0 | 0 | 0 | 0 | 0 | 0 | 0 | 0 | 1 | 0 | 0 | 0 | 1 | Archaea | Archaea\_none | Euryarchaeota | Halobacteria | Halobacteriales | Haloferacaceae | Halorarum | Archaea;Archaea\_none;Euryarchaeota;Halobacteria;Halobacteriales;Haloferacaceae;Halorarum;Halorarum salinum |
| Halorubrum ruber | 0 | 0 | 0 | 0 | 0 | 0 | 0 | 0 | 0 | 0 | 0 | 0 | 1 | 1 | Archaea | Archaea\_none | Euryarchaeota | Halobacteria | Halobacteriales | Haloferacaceae | Halorubrum | Archaea;Archaea\_none;Euryarchaeota;Halobacteria;Halobacteriales;Haloferacaceae;Halorubrum;Halorubrum ruber |
| Methanobacterium\_uncultured | 0 | 0 | 0 | 0 | 0 | 0 | 1 | 0 | 0 | 0 | 0 | 0 | 0 | 1 | Archaea | Archaea\_none | Euryarchaeota | Methanobacteria | Methanobacteriales | Methanobacteriaceae | Methanobacterium | Archaea;Archaea\_none;Euryarchaeota;Methanobacteria;Methanobacteriales;Methanobacteriaceae;Methanobacterium;Methanobacterium\_uncultured |
| Methanobrevibacter arboriphilus | 0 | 0 | 1 | 0 | 0 | 0 | 0 | 0 | 0 | 0 | 0 | 0 | 0 | 1 | Archaea | Archaea\_none | Euryarchaeota | Methanobacteria | Methanobacteriales | Methanobacteriaceae | Methanobrevibacter | Archaea;Archaea\_none;Euryarchaeota;Methanobacteria;Methanobacteriales;Methanobacteriaceae;Methanobrevibacter;Methanobrevibacter arboriphilus |
| Methanobrevibacter smithii | 0 | 0 | 0 | 0 | 0 | 1 | 0 | 0 | 0 | 0 | 0 | 0 | 0 | 1 | Archaea | Archaea\_none | Euryarchaeota | Methanobacteria | Methanobacteriales | Methanobacteriaceae | Methanobrevibacter | Archaea;Archaea\_none;Euryarchaeota;Methanobacteria;Methanobacteriales;Methanobacteriaceae;Methanobrevibacter;Methanobrevibacter smithii |
| Methanobrevibacter sp. YE315 | 0 | 0 | 0 | 0 | 0 | 0 | 0 | 0 | 0 | 0 | 0 | 0 | 1 | 1 | Archaea | Archaea\_none | Euryarchaeota | Methanobacteria | Methanobacteriales | Methanobacteriaceae | Methanobrevibacter | Archaea;Archaea\_none;Euryarchaeota;Methanobacteria;Methanobacteriales;Methanobacteriaceae;Methanobrevibacter;Methanobrevibacter sp. YE315 |
| Methanosphaera stadtmanae | 0 | 0 | 1 | 0 | 0 | 0 | 0 | 0 | 0 | 0 | 0 | 0 | 0 | 1 | Archaea | Archaea\_none | Euryarchaeota | Methanobacteria | Methanobacteriales | Methanobacteriaceae | Methanosphaera | Archaea;Archaea\_none;Euryarchaeota;Methanobacteria;Methanobacteriales;Methanobacteriaceae;Methanosphaera;Methanosphaera stadtmanae |
| Methanocaldococcus sp. FS406-22 | 0 | 0 | 0 | 0 | 0 | 0 | 0 | 0 | 0 | 0 | 0 | 0 | 1 | 1 | Archaea | Archaea\_none | Euryarchaeota | Methanococci | Methanococcales | Methanocaldococcaceae | Methanocaldococcus | Archaea;Archaea\_none;Euryarchaeota;Methanococci;Methanococcales;Methanocaldococcaceae;Methanocaldococcus;Methanocaldococcus sp. FS406-22 |
| Methanocaldococcus vulcanius | 0 | 0 | 1 | 0 | 0 | 0 | 0 | 0 | 0 | 0 | 0 | 0 | 0 | 1 | Archaea | Archaea\_none | Euryarchaeota | Methanococci | Methanococcales | Methanocaldococcaceae | Methanocaldococcus | Archaea;Archaea\_none;Euryarchaeota;Methanococci;Methanococcales;Methanocaldococcaceae;Methanocaldococcus;Methanocaldococcus vulcanius |
| Methanoregula\_uncultured | 0 | 1 | 0 | 0 | 0 | 0 | 0 | 0 | 0 | 0 | 0 | 0 | 1 | 2 | Archaea | Archaea\_none | Euryarchaeota | Methanomicrobia | Methanomicrobiales | Methanoregulaceae | Methanoregula | Archaea;Archaea\_none;Euryarchaeota;Methanomicrobia;Methanomicrobiales;Methanoregulaceae;Methanoregula;Methanoregula\_uncultured |
| Methanimicrococcus hongohii | 0 | 0 | 0 | 1 | 0 | 0 | 0 | 0 | 0 | 0 | 0 | 0 | 0 | 1 | Archaea | Archaea\_none | Euryarchaeota | Methanomicrobia | Methanosarcinales | Methanosarcinaceae | Methanimicrococcus | Archaea;Archaea\_none;Euryarchaeota;Methanomicrobia;Methanosarcinales;Methanosarcinaceae;Methanimicrococcus;Methanimicrococcus hongohii |
| Methanolapillus millepedarum | 0 | 0 | 0 | 0 | 0 | 1 | 0 | 0 | 0 | 0 | 0 | 0 | 0 | 1 | Archaea | Archaea\_none | Euryarchaeota | Methanomicrobia | Methanosarcinales | Methanosarcinaceae | Methanolapillus | Archaea;Archaea\_none;Euryarchaeota;Methanomicrobia;Methanosarcinales;Methanosarcinaceae;Methanolapillus;Methanolapillus millepedarum |
| Methanolobus\_uncultured | 0 | 0 | 0 | 0 | 1 | 1 | 0 | 0 | 0 | 0 | 0 | 0 | 0 | 2 | Archaea | Archaea\_none | Euryarchaeota | Methanomicrobia | Methanosarcinales | Methanosarcinaceae | Methanolobus | Archaea;Archaea\_none;Euryarchaeota;Methanomicrobia;Methanosarcinales;Methanosarcinaceae;Methanolobus;Methanolobus\_uncultured |
| Methanomethylovorans\_uncultured | 0 | 0 | 0 | 0 | 0 | 0 | 0 | 0 | 0 | 0 | 0 | 0 | 1 | 1 | Archaea | Archaea\_none | Euryarchaeota | Methanomicrobia | Methanosarcinales | Methanosarcinaceae | Methanomethylovorans | Archaea;Archaea\_none;Euryarchaeota;Methanomicrobia;Methanosarcinales;Methanosarcinaceae;Methanomethylovorans;Methanomethylovorans\_uncultured |
| Methanosarcina horonobensis | 0 | 0 | 0 | 0 | 0 | 0 | 0 | 0 | 0 | 0 | 1 | 0 | 0 | 1 | Archaea | Archaea\_none | Euryarchaeota | Methanomicrobia | Methanosarcinales | Methanosarcinaceae | Methanosarcina | Archaea;Archaea\_none;Euryarchaeota;Methanomicrobia;Methanosarcinales;Methanosarcinaceae;Methanosarcina;Methanosarcina horonobensis |
| Methanosarcina siciliae | 0 | 0 | 0 | 0 | 0 | 1 | 0 | 0 | 0 | 0 | 0 | 0 | 0 | 1 | Archaea | Archaea\_none | Euryarchaeota | Methanomicrobia | Methanosarcinales | Methanosarcinaceae | Methanosarcina | Archaea;Archaea\_none;Euryarchaeota;Methanomicrobia;Methanosarcinales;Methanosarcinaceae;Methanosarcina;Methanosarcina siciliae |
| Thermococcus litoralis | 0 | 0 | 0 | 0 | 0 | 0 | 0 | 0 | 0 | 0 | 0 | 1 | 0 | 1 | Archaea | Archaea\_none | Euryarchaeota | Thermococci | Thermococcales | Thermococcaceae | Thermococcus | Archaea;Archaea\_none;Euryarchaeota;Thermococci;Thermococcales;Thermococcaceae;Thermococcus;Thermococcus litoralis |
| Candidatus Nitrosotenuis chungbukensis | 0 | 0 | 0 | 0 | 0 | 1 | 0 | 0 | 0 | 0 | 0 | 0 | 0 | 1 | Archaea | Archaea\_none | Nitrososphaerota | Nitrososphaerota\_Incertae\_sedis | Nitrososphaerota\_Incertae\_sedis | Nitrososphaerota\_Incertae\_sedis | Candidatus Nitrosotenuis | Archaea;Archaea\_none;Nitrososphaerota;Nitrososphaerota\_Incertae\_sedis;Nitrososphaerota\_Incertae\_sedis;Nitrososphaerota\_Incertae\_sedis;Candidatus Nitrosotenuis;Candidatus Nitrosotenuis chungbukensis |
| Paludibaculum fermentans | 0 | 0 | 0 | 0 | 0 | 0 | 0 | 0 | 0 | 0 | 0 | 1 | 0 | 1 | Bacteria | Bacteria\_none | Acidobacteriota | Terriglobia | Bryobacterales | Bryobacteraceae | Paludibaculum | Bacteria;Bacteria\_none;Acidobacteriota;Terriglobia;Bryobacterales;Bryobacteraceae;Paludibaculum;Paludibaculum fermentans |
| Aquihabitans sp. G128 | 0 | 0 | 0 | 0 | 0 | 0 | 0 | 1 | 0 | 0 | 0 | 0 | 0 | 1 | Bacteria | Bacteria\_none | Actinomycetota | Acidimicrobiia | Acidimicrobiales | Iamiaceae | Aquihabitans | Bacteria;Bacteria\_none;Actinomycetota;Acidimicrobiia;Acidimicrobiales;Iamiaceae;Aquihabitans;Aquihabitans sp. G128 |
| Actinomyces naeslundii | 0 | 0 | 0 | 0 | 0 | 0 | 0 | 0 | 0 | 0 | 0 | 1 | 0 | 1 | Bacteria | Bacteria\_none | Actinomycetota | Actinomycetes | Actinomycetales | Actinomycetaceae | Actinomyces | Bacteria;Bacteria\_none;Actinomycetota;Actinomycetes;Actinomycetales;Actinomycetaceae;Actinomyces;Actinomyces naeslundii |
| Actinomyces oris | 0 | 0 | 0 | 0 | 0 | 0 | 0 | 0 | 0 | 0 | 0 | 1 | 0 | 1 | Bacteria | Bacteria\_none | Actinomycetota | Actinomycetes | Actinomycetales | Actinomycetaceae | Actinomyces | Bacteria;Bacteria\_none;Actinomycetota;Actinomycetes;Actinomycetales;Actinomycetaceae;Actinomyces;Actinomyces oris |
| Actinomyces pacaensis | 0 | 1 | 0 | 0 | 2 | 19 | 12 | 4 | 6 | 0 | 0 | 4 | 7 | 55 | Bacteria | Bacteria\_none | Actinomycetota | Actinomycetes | Actinomycetales | Actinomycetaceae | Actinomyces | Bacteria;Bacteria\_none;Actinomycetota;Actinomycetes;Actinomycetales;Actinomycetaceae;Actinomyces;Actinomyces pacaensis |
| Actinomyces qiguomingii | 1 | 0 | 0 | 0 | 0 | 0 | 0 | 0 | 0 | 0 | 0 | 0 | 0 | 1 | Bacteria | Bacteria\_none | Actinomycetota | Actinomycetes | Actinomycetales | Actinomycetaceae | Actinomyces | Bacteria;Bacteria\_none;Actinomycetota;Actinomycetes;Actinomycetales;Actinomycetaceae;Actinomyces;Actinomyces qiguomingii |
| Actinomyces sp. oral taxon 414 | 0 | 0 | 0 | 0 | 0 | 0 | 0 | 0 | 0 | 0 | 1 | 0 | 0 | 1 | Bacteria | Bacteria\_none | Actinomycetota | Actinomycetes | Actinomycetales | Actinomycetaceae | Actinomyces | Bacteria;Bacteria\_none;Actinomycetota;Actinomycetes;Actinomycetales;Actinomycetaceae;Actinomyces;Actinomyces sp. oral taxon 414 |
| Actinomyces trachealis | 0 | 0 | 0 | 0 | 0 | 0 | 0 | 0 | 0 | 0 | 0 | 0 | 1 | 1 | Bacteria | Bacteria\_none | Actinomycetota | Actinomycetes | Actinomycetales | Actinomycetaceae | Actinomyces | Bacteria;Bacteria\_none;Actinomycetota;Actinomycetes;Actinomycetales;Actinomycetaceae;Actinomyces;Actinomyces trachealis |
| Schaalia meyeri | 0 | 0 | 0 | 0 | 0 | 1 | 0 | 0 | 0 | 0 | 0 | 0 | 1 | 2 | Bacteria | Bacteria\_none | Actinomycetota | Actinomycetes | Actinomycetales | Actinomycetaceae | Schaalia | Bacteria;Bacteria\_none;Actinomycetota;Actinomycetes;Actinomycetales;Actinomycetaceae;Schaalia;Schaalia meyeri |
| Schaalia odontolytica | 0 | 16 | 0 | 3 | 1 | 11 | 16 | 0 | 0 | 0 | 0 | 5 | 6 | 58 | Bacteria | Bacteria\_none | Actinomycetota | Actinomycetes | Actinomycetales | Actinomycetaceae | Schaalia | Bacteria;Bacteria\_none;Actinomycetota;Actinomycetes;Actinomycetales;Actinomycetaceae;Schaalia;Schaalia odontolytica |
| Schaalia sp. HMT-172 | 0 | 0 | 0 | 0 | 0 | 9 | 10 | 0 | 0 | 0 | 0 | 1 | 4 | 24 | Bacteria | Bacteria\_none | Actinomycetota | Actinomycetes | Actinomycetales | Actinomycetaceae | Schaalia | Bacteria;Bacteria\_none;Actinomycetota;Actinomycetes;Actinomycetales;Actinomycetaceae;Schaalia;Schaalia sp. HMT-172 |
| Schaalia sp. ZJ405 | 0 | 0 | 1 | 0 | 0 | 0 | 0 | 0 | 0 | 0 | 0 | 0 | 0 | 1 | Bacteria | Bacteria\_none | Actinomycetota | Actinomycetes | Actinomycetales | Actinomycetaceae | Schaalia | Bacteria;Bacteria\_none;Actinomycetota;Actinomycetes;Actinomycetales;Actinomycetaceae;Schaalia;Schaalia sp. ZJ405 |
| Bifidobacterium asteroides | 0 | 0 | 1 | 0 | 0 | 0 | 0 | 0 | 0 | 0 | 0 | 0 | 0 | 1 | Bacteria | Bacteria\_none | Actinomycetota | Actinomycetes | Bifidobacteriales | Bifidobacteriaceae | Bifidobacterium | Bacteria;Bacteria\_none;Actinomycetota;Actinomycetes;Bifidobacteriales;Bifidobacteriaceae;Bifidobacterium;Bifidobacterium asteroides |
| Bifidobacterium dentium | 0 | 0 | 0 | 0 | 0 | 0 | 2 | 2 | 0 | 0 | 0 | 0 | 1 | 5 | Bacteria | Bacteria\_none | Actinomycetota | Actinomycetes | Bifidobacteriales | Bifidobacteriaceae | Bifidobacterium | Bacteria;Bacteria\_none;Actinomycetota;Actinomycetes;Bifidobacteriales;Bifidobacteriaceae;Bifidobacterium;Bifidobacterium dentium |
| Bifidobacterium longum | 0 | 0 | 0 | 0 | 0 | 1 | 2 | 0 | 0 | 0 | 0 | 0 | 0 | 3 | Bacteria | Bacteria\_none | Actinomycetota | Actinomycetes | Bifidobacteriales | Bifidobacteriaceae | Bifidobacterium | Bacteria;Bacteria\_none;Actinomycetota;Actinomycetes;Bifidobacteriales;Bifidobacteriaceae;Bifidobacterium;Bifidobacterium longum |
| Bifidobacterium pullorum | 0 | 0 | 0 | 0 | 0 | 0 | 1 | 0 | 0 | 0 | 0 | 0 | 0 | 1 | Bacteria | Bacteria\_none | Actinomycetota | Actinomycetes | Bifidobacteriales | Bifidobacteriaceae | Bifidobacterium | Bacteria;Bacteria\_none;Actinomycetota;Actinomycetes;Bifidobacteriales;Bifidobacteriaceae;Bifidobacterium;Bifidobacterium pullorum |
| Gardnerella vaginalis | 0 | 1 | 1 | 0 | 0 | 0 | 0 | 2 | 0 | 0 | 0 | 0 | 1 | 5 | Bacteria | Bacteria\_none | Actinomycetota | Actinomycetes | Bifidobacteriales | Bifidobacteriaceae | Gardnerella | Bacteria;Bacteria\_none;Actinomycetota;Actinomycetes;Bifidobacteriales;Bifidobacteriaceae;Gardnerella;Gardnerella vaginalis |
| Parascardovia denticolens | 0 | 0 | 0 | 0 | 1 | 0 | 2 | 0 | 0 | 0 | 0 | 0 | 0 | 3 | Bacteria | Bacteria\_none | Actinomycetota | Actinomycetes | Bifidobacteriales | Bifidobacteriaceae | Parascardovia | Bacteria;Bacteria\_none;Actinomycetota;Actinomycetes;Bifidobacteriales;Bifidobacteriaceae;Parascardovia;Parascardovia denticolens |
| Geodermatophilus obscurus | 0 | 0 | 0 | 0 | 1 | 0 | 0 | 0 | 0 | 0 | 0 | 0 | 0 | 1 | Bacteria | Bacteria\_none | Actinomycetota | Actinomycetes | Geodermatophilales | Geodermatophilaceae | Geodermatophilus | Bacteria;Bacteria\_none;Actinomycetota;Actinomycetes;Geodermatophilales;Geodermatophilaceae;Geodermatophilus;Geodermatophilus obscurus |
| Streptomyces cadmiisoli | 0 | 0 | 0 | 0 | 0 | 0 | 0 | 1 | 0 | 0 | 0 | 0 | 0 | 1 | Bacteria | Bacteria\_none | Actinomycetota | Actinomycetes | Kitasatosporales | Streptomycetaceae | Streptomyces | Bacteria;Bacteria\_none;Actinomycetota;Actinomycetes;Kitasatosporales;Streptomycetaceae;Streptomyces;Streptomyces cadmiisoli |
| Streptomyces caniferus | 0 | 0 | 0 | 0 | 0 | 0 | 0 | 0 | 0 | 0 | 0 | 0 | 1 | 1 | Bacteria | Bacteria\_none | Actinomycetota | Actinomycetes | Kitasatosporales | Streptomycetaceae | Streptomyces | Bacteria;Bacteria\_none;Actinomycetota;Actinomycetes;Kitasatosporales;Streptomycetaceae;Streptomyces;Streptomyces caniferus |
| Streptomyces gilvosporeus | 0 | 0 | 0 | 0 | 0 | 0 | 0 | 0 | 0 | 1 | 0 | 0 | 0 | 1 | Bacteria | Bacteria\_none | Actinomycetota | Actinomycetes | Kitasatosporales | Streptomycetaceae | Streptomyces | Bacteria;Bacteria\_none;Actinomycetota;Actinomycetes;Kitasatosporales;Streptomycetaceae;Streptomyces;Streptomyces gilvosporeus |
| Streptomyces noursei | 0 | 0 | 0 | 0 | 0 | 0 | 0 | 0 | 0 | 0 | 0 | 0 | 1 | 1 | Bacteria | Bacteria\_none | Actinomycetota | Actinomycetes | Kitasatosporales | Streptomycetaceae | Streptomyces | Bacteria;Bacteria\_none;Actinomycetota;Actinomycetes;Kitasatosporales;Streptomycetaceae;Streptomyces;Streptomyces noursei |
| Streptomyces qaidamensis | 0 | 0 | 0 | 0 | 0 | 0 | 0 | 0 | 0 | 0 | 0 | 1 | 0 | 1 | Bacteria | Bacteria\_none | Actinomycetota | Actinomycetes | Kitasatosporales | Streptomycetaceae | Streptomyces | Bacteria;Bacteria\_none;Actinomycetota;Actinomycetes;Kitasatosporales;Streptomycetaceae;Streptomyces;Streptomyces qaidamensis |
| Streptomyces rimosus | 0 | 0 | 0 | 0 | 0 | 0 | 1 | 0 | 0 | 0 | 0 | 0 | 0 | 1 | Bacteria | Bacteria\_none | Actinomycetota | Actinomycetes | Kitasatosporales | Streptomycetaceae | Streptomyces | Bacteria;Bacteria\_none;Actinomycetota;Actinomycetes;Kitasatosporales;Streptomycetaceae;Streptomyces;Streptomyces rimosus |
| Streptomyces sp. 11x1 | 0 | 0 | 0 | 0 | 0 | 1 | 0 | 0 | 0 | 0 | 0 | 0 | 0 | 1 | Bacteria | Bacteria\_none | Actinomycetota | Actinomycetes | Kitasatosporales | Streptomycetaceae | Streptomyces | Bacteria;Bacteria\_none;Actinomycetota;Actinomycetes;Kitasatosporales;Streptomycetaceae;Streptomyces;Streptomyces sp. 11x1 |
| Streptomyces sp. 2114.2 | 0 | 0 | 0 | 0 | 0 | 3 | 0 | 0 | 0 | 0 | 0 | 0 | 0 | 3 | Bacteria | Bacteria\_none | Actinomycetota | Actinomycetes | Kitasatosporales | Streptomycetaceae | Streptomyces | Bacteria;Bacteria\_none;Actinomycetota;Actinomycetes;Kitasatosporales;Streptomycetaceae;Streptomyces;Streptomyces sp. 2114.2 |
| Streptomyces sp. CB00271 | 0 | 0 | 0 | 0 | 0 | 0 | 0 | 0 | 0 | 0 | 0 | 1 | 0 | 1 | Bacteria | Bacteria\_none | Actinomycetota | Actinomycetes | Kitasatosporales | Streptomycetaceae | Streptomyces | Bacteria;Bacteria\_none;Actinomycetota;Actinomycetes;Kitasatosporales;Streptomycetaceae;Streptomyces;Streptomyces sp. CB00271 |
| Streptomyces sp. CNQ-509 | 0 | 0 | 0 | 0 | 2 | 0 | 0 | 0 | 0 | 0 | 0 | 0 | 0 | 2 | Bacteria | Bacteria\_none | Actinomycetota | Actinomycetes | Kitasatosporales | Streptomycetaceae | Streptomyces | Bacteria;Bacteria\_none;Actinomycetota;Actinomycetes;Kitasatosporales;Streptomycetaceae;Streptomyces;Streptomyces sp. CNQ-509 |
| Streptomyces sp. FXJ1.172 | 0 | 0 | 0 | 0 | 0 | 0 | 0 | 0 | 0 | 0 | 0 | 0 | 1 | 1 | Bacteria | Bacteria\_none | Actinomycetota | Actinomycetes | Kitasatosporales | Streptomycetaceae | Streptomyces | Bacteria;Bacteria\_none;Actinomycetota;Actinomycetes;Kitasatosporales;Streptomycetaceae;Streptomyces;Streptomyces sp. FXJ1.172 |
| Streptomyces sp. HUAS 15-9 | 0 | 0 | 0 | 0 | 0 | 1 | 0 | 0 | 0 | 0 | 0 | 0 | 0 | 1 | Bacteria | Bacteria\_none | Actinomycetota | Actinomycetes | Kitasatosporales | Streptomycetaceae | Streptomyces | Bacteria;Bacteria\_none;Actinomycetota;Actinomycetes;Kitasatosporales;Streptomycetaceae;Streptomyces;Streptomyces sp. HUAS 15-9 |
| Streptomyces sp. Li-HN-5-11 | 0 | 0 | 0 | 2 | 0 | 0 | 0 | 0 | 0 | 0 | 0 | 0 | 0 | 2 | Bacteria | Bacteria\_none | Actinomycetota | Actinomycetes | Kitasatosporales | Streptomycetaceae | Streptomyces | Bacteria;Bacteria\_none;Actinomycetota;Actinomycetes;Kitasatosporales;Streptomycetaceae;Streptomyces;Streptomyces sp. Li-HN-5-11 |
| Streptomyces sp. NBC\_00247 | 0 | 0 | 0 | 0 | 0 | 0 | 0 | 0 | 0 | 0 | 0 | 0 | 1 | 1 | Bacteria | Bacteria\_none | Actinomycetota | Actinomycetes | Kitasatosporales | Streptomycetaceae | Streptomyces | Bacteria;Bacteria\_none;Actinomycetota;Actinomycetes;Kitasatosporales;Streptomycetaceae;Streptomyces;Streptomyces sp. NBC\_00247 |
| Streptomyces sp. NBC\_00250 | 0 | 0 | 0 | 0 | 0 | 1 | 0 | 0 | 0 | 0 | 0 | 0 | 0 | 1 | Bacteria | Bacteria\_none | Actinomycetota | Actinomycetes | Kitasatosporales | Streptomycetaceae | Streptomyces | Bacteria;Bacteria\_none;Actinomycetota;Actinomycetes;Kitasatosporales;Streptomycetaceae;Streptomyces;Streptomyces sp. NBC\_00250 |
| Streptomyces sp. NBC\_00370 | 0 | 1 | 0 | 0 | 0 | 0 | 0 | 0 | 0 | 0 | 0 | 0 | 0 | 1 | Bacteria | Bacteria\_none | Actinomycetota | Actinomycetes | Kitasatosporales | Streptomycetaceae | Streptomyces | Bacteria;Bacteria\_none;Actinomycetota;Actinomycetes;Kitasatosporales;Streptomycetaceae;Streptomyces;Streptomyces sp. NBC\_00370 |
| Streptomyces sp. NBC\_00425 | 0 | 0 | 0 | 0 | 0 | 1 | 0 | 0 | 0 | 0 | 0 | 0 | 0 | 1 | Bacteria | Bacteria\_none | Actinomycetota | Actinomycetes | Kitasatosporales | Streptomycetaceae | Streptomyces | Bacteria;Bacteria\_none;Actinomycetota;Actinomycetes;Kitasatosporales;Streptomycetaceae;Streptomyces;Streptomyces sp. NBC\_00425 |
| Streptomyces sp. NBC\_00490 | 0 | 0 | 0 | 0 | 0 | 0 | 2 | 0 | 0 | 0 | 0 | 0 | 0 | 2 | Bacteria | Bacteria\_none | Actinomycetota | Actinomycetes | Kitasatosporales | Streptomycetaceae | Streptomyces | Bacteria;Bacteria\_none;Actinomycetota;Actinomycetes;Kitasatosporales;Streptomycetaceae;Streptomyces;Streptomyces sp. NBC\_00490 |
| Streptomyces sp. NBC\_01341 | 0 | 0 | 0 | 0 | 0 | 0 | 0 | 0 | 0 | 0 | 0 | 0 | 1 | 1 | Bacteria | Bacteria\_none | Actinomycetota | Actinomycetes | Kitasatosporales | Streptomycetaceae | Streptomyces | Bacteria;Bacteria\_none;Actinomycetota;Actinomycetes;Kitasatosporales;Streptomycetaceae;Streptomyces;Streptomyces sp. NBC\_01341 |
| Streptomyces sp. NBC\_01435 | 0 | 0 | 0 | 0 | 0 | 0 | 0 | 0 | 0 | 1 | 0 | 0 | 0 | 1 | Bacteria | Bacteria\_none | Actinomycetota | Actinomycetes | Kitasatosporales | Streptomycetaceae | Streptomyces | Bacteria;Bacteria\_none;Actinomycetota;Actinomycetes;Kitasatosporales;Streptomycetaceae;Streptomyces;Streptomyces sp. NBC\_01435 |
| Streptomyces sp. NBC\_01788 | 0 | 0 | 0 | 0 | 0 | 1 | 0 | 0 | 0 | 0 | 0 | 0 | 0 | 1 | Bacteria | Bacteria\_none | Actinomycetota | Actinomycetes | Kitasatosporales | Streptomycetaceae | Streptomyces | Bacteria;Bacteria\_none;Actinomycetota;Actinomycetes;Kitasatosporales;Streptomycetaceae;Streptomyces;Streptomyces sp. NBC\_01788 |
| Streptomyces sp. PCS3-D2 | 0 | 0 | 0 | 0 | 0 | 1 | 0 | 0 | 0 | 0 | 0 | 0 | 0 | 1 | Bacteria | Bacteria\_none | Actinomycetota | Actinomycetes | Kitasatosporales | Streptomycetaceae | Streptomyces | Bacteria;Bacteria\_none;Actinomycetota;Actinomycetes;Kitasatosporales;Streptomycetaceae;Streptomyces;Streptomyces sp. PCS3-D2 |
| Streptomyces sp. Tu 2975 | 0 | 1 | 0 | 0 | 0 | 0 | 0 | 0 | 0 | 0 | 0 | 0 | 0 | 1 | Bacteria | Bacteria\_none | Actinomycetota | Actinomycetes | Kitasatosporales | Streptomycetaceae | Streptomyces | Bacteria;Bacteria\_none;Actinomycetota;Actinomycetes;Kitasatosporales;Streptomycetaceae;Streptomyces;Streptomyces sp. Tu 2975 |
| Streptomyces tubbatahanensis | 0 | 0 | 0 | 0 | 0 | 0 | 1 | 0 | 0 | 0 | 0 | 0 | 0 | 1 | Bacteria | Bacteria\_none | Actinomycetota | Actinomycetes | Kitasatosporales | Streptomycetaceae | Streptomyces | Bacteria;Bacteria\_none;Actinomycetota;Actinomycetes;Kitasatosporales;Streptomycetaceae;Streptomyces;Streptomyces tubbatahanensis |
| Streptomyces tubercidicus | 0 | 0 | 0 | 0 | 0 | 0 | 0 | 0 | 0 | 0 | 1 | 0 | 0 | 1 | Bacteria | Bacteria\_none | Actinomycetota | Actinomycetes | Kitasatosporales | Streptomycetaceae | Streptomyces | Bacteria;Bacteria\_none;Actinomycetota;Actinomycetes;Kitasatosporales;Streptomycetaceae;Streptomyces;Streptomyces tubercidicus |
| Cellulomonas wangleii | 0 | 0 | 0 | 0 | 0 | 0 | 0 | 0 | 0 | 0 | 0 | 0 | 1 | 1 | Bacteria | Bacteria\_none | Actinomycetota | Actinomycetes | Micrococcales | Cellulomonadaceae | Cellulomonas | Bacteria;Bacteria\_none;Actinomycetota;Actinomycetes;Micrococcales;Cellulomonadaceae;Cellulomonas;Cellulomonas wangleii |
| Gephyromycinifex aptenodytis | 0 | 0 | 0 | 0 | 0 | 0 | 0 | 0 | 0 | 0 | 0 | 0 | 1 | 1 | Bacteria | Bacteria\_none | Actinomycetota | Actinomycetes | Micrococcales | Dermatophilaceae | Gephyromycinifex | Bacteria;Bacteria\_none;Actinomycetota;Actinomycetes;Micrococcales;Dermatophilaceae;Gephyromycinifex;Gephyromycinifex aptenodytis |
| Agromyces soli | 1 | 0 | 0 | 0 | 0 | 0 | 0 | 0 | 0 | 0 | 0 | 0 | 0 | 1 | Bacteria | Bacteria\_none | Actinomycetota | Actinomycetes | Micrococcales | Microbacteriaceae | Agromyces | Bacteria;Bacteria\_none;Actinomycetota;Actinomycetes;Micrococcales;Microbacteriaceae;Agromyces;Agromyces soli |
| Curtobacterium sp. BH-2-1-1 | 0 | 0 | 0 | 0 | 1 | 0 | 0 | 0 | 0 | 0 | 0 | 0 | 0 | 1 | Bacteria | Bacteria\_none | Actinomycetota | Actinomycetes | Micrococcales | Microbacteriaceae | Curtobacterium | Bacteria;Bacteria\_none;Actinomycetota;Actinomycetes;Micrococcales;Microbacteriaceae;Curtobacterium;Curtobacterium sp. BH-2-1-1 |
| Curtobacterium sp. MCPF17\_021 | 0 | 0 | 0 | 1 | 0 | 0 | 0 | 0 | 0 | 0 | 0 | 0 | 0 | 1 | Bacteria | Bacteria\_none | Actinomycetota | Actinomycetes | Micrococcales | Microbacteriaceae | Curtobacterium | Bacteria;Bacteria\_none;Actinomycetota;Actinomycetes;Micrococcales;Microbacteriaceae;Curtobacterium;Curtobacterium sp. MCPF17\_021 |
| Diaminobutyricibacter sp. McL0608 | 0 | 0 | 0 | 1 | 0 | 0 | 0 | 0 | 0 | 0 | 0 | 0 | 0 | 1 | Bacteria | Bacteria\_none | Actinomycetota | Actinomycetes | Micrococcales | Microbacteriaceae | Diaminobutyricibacter | Bacteria;Bacteria\_none;Actinomycetota;Actinomycetes;Micrococcales;Microbacteriaceae;Diaminobutyricibacter;Diaminobutyricibacter sp. McL0608 |
| Leucobacter viscericola | 0 | 0 | 0 | 0 | 0 | 0 | 1 | 0 | 0 | 0 | 0 | 0 | 0 | 1 | Bacteria | Bacteria\_none | Actinomycetota | Actinomycetes | Micrococcales | Microbacteriaceae | Leucobacter | Bacteria;Bacteria\_none;Actinomycetota;Actinomycetes;Micrococcales;Microbacteriaceae;Leucobacter;Leucobacter viscericola |
| Microbacterium binotii | 0 | 0 | 0 | 0 | 0 | 0 | 0 | 1 | 0 | 0 | 0 | 0 | 0 | 1 | Bacteria | Bacteria\_none | Actinomycetota | Actinomycetes | Micrococcales | Microbacteriaceae | Microbacterium | Bacteria;Bacteria\_none;Actinomycetota;Actinomycetes;Micrococcales;Microbacteriaceae;Microbacterium;Microbacterium binotii |
| Microbacterium sp. Clip185 | 0 | 0 | 0 | 0 | 1 | 0 | 0 | 0 | 0 | 0 | 0 | 0 | 1 | 2 | Bacteria | Bacteria\_none | Actinomycetota | Actinomycetes | Micrococcales | Microbacteriaceae | Microbacterium | Bacteria;Bacteria\_none;Actinomycetota;Actinomycetes;Micrococcales;Microbacteriaceae;Microbacterium;Microbacterium sp. Clip185 |
| Microcella alkaliphila | 0 | 0 | 0 | 0 | 0 | 0 | 0 | 0 | 1 | 0 | 0 | 0 | 0 | 1 | Bacteria | Bacteria\_none | Actinomycetota | Actinomycetes | Micrococcales | Microbacteriaceae | Microcella | Bacteria;Bacteria\_none;Actinomycetota;Actinomycetes;Micrococcales;Microbacteriaceae;Microcella;Microcella alkaliphila |
| Arthrobacter citreus | 0 | 0 | 0 | 0 | 0 | 0 | 0 | 0 | 0 | 0 | 1 | 0 | 0 | 1 | Bacteria | Bacteria\_none | Actinomycetota | Actinomycetes | Micrococcales | Micrococcaceae | Arthrobacter | Bacteria;Bacteria\_none;Actinomycetota;Actinomycetes;Micrococcales;Micrococcaceae;Arthrobacter;Arthrobacter citreus |
| Arthrobacter oryzae | 0 | 0 | 0 | 0 | 0 | 1 | 0 | 0 | 0 | 0 | 0 | 0 | 0 | 1 | Bacteria | Bacteria\_none | Actinomycetota | Actinomycetes | Micrococcales | Micrococcaceae | Arthrobacter | Bacteria;Bacteria\_none;Actinomycetota;Actinomycetes;Micrococcales;Micrococcaceae;Arthrobacter;Arthrobacter oryzae |
| Arthrobacter sp. ATA002 | 0 | 0 | 0 | 0 | 0 | 0 | 0 | 0 | 0 | 0 | 0 | 0 | 1 | 1 | Bacteria | Bacteria\_none | Actinomycetota | Actinomycetes | Micrococcales | Micrococcaceae | Arthrobacter | Bacteria;Bacteria\_none;Actinomycetota;Actinomycetes;Micrococcales;Micrococcaceae;Arthrobacter;Arthrobacter sp. ATA002 |
| Arthrobacter sp. FW306-2-2C-D06B | 0 | 0 | 0 | 0 | 0 | 0 | 0 | 0 | 0 | 2 | 0 | 0 | 0 | 2 | Bacteria | Bacteria\_none | Actinomycetota | Actinomycetes | Micrococcales | Micrococcaceae | Arthrobacter | Bacteria;Bacteria\_none;Actinomycetota;Actinomycetes;Micrococcales;Micrococcaceae;Arthrobacter;Arthrobacter sp. FW306-2-2C-D06B |
| Pseudarthrobacter sp. NS4 | 0 | 0 | 0 | 0 | 1 | 0 | 0 | 0 | 0 | 0 | 0 | 0 | 0 | 1 | Bacteria | Bacteria\_none | Actinomycetota | Actinomycetes | Micrococcales | Micrococcaceae | Pseudarthrobacter | Bacteria;Bacteria\_none;Actinomycetota;Actinomycetes;Micrococcales;Micrococcaceae;Pseudarthrobacter;Pseudarthrobacter sp. NS4 |
| Rothia aeria | 13 | 1 | 0 | 3 | 48 | 0 | 0 | 60 | 0 | 2 | 92 | 13 | 54 | 286 | Bacteria | Bacteria\_none | Actinomycetota | Actinomycetes | Micrococcales | Micrococcaceae | Rothia | Bacteria;Bacteria\_none;Actinomycetota;Actinomycetes;Micrococcales;Micrococcaceae;Rothia;Rothia aeria |
| Rothia dentocariosa | 5 | 23 | 1 | 3 | 7 | 3 | 5 | 1 | 0 | 0 | 3 | 21 | 24 | 96 | Bacteria | Bacteria\_none | Actinomycetota | Actinomycetes | Micrococcales | Micrococcaceae | Rothia | Bacteria;Bacteria\_none;Actinomycetota;Actinomycetes;Micrococcales;Micrococcaceae;Rothia;Rothia dentocariosa |
| Rothia mucilaginosa | 9 | 87 | 22 | 11 | 69 | 38 | 31 | 0 | 14 | 76 | 61 | 133 | 134 | 685 | Bacteria | Bacteria\_none | Actinomycetota | Actinomycetes | Micrococcales | Micrococcaceae | Rothia | Bacteria;Bacteria\_none;Actinomycetota;Actinomycetes;Micrococcales;Micrococcaceae;Rothia;Rothia mucilaginosa |
| Luteimicrobium xylanilyticum | 0 | 0 | 0 | 0 | 0 | 0 | 0 | 0 | 0 | 0 | 0 | 0 | 1 | 1 | Bacteria | Bacteria\_none | Actinomycetota | Actinomycetes | Micrococcales | Micrococcales\_Incertae\_sedis | Luteimicrobium | Bacteria;Bacteria\_none;Actinomycetota;Actinomycetes;Micrococcales;Micrococcales\_Incertae\_sedis;Luteimicrobium;Luteimicrobium xylanilyticum |
| Ruania halotolerans | 0 | 0 | 0 | 0 | 0 | 0 | 1 | 0 | 0 | 0 | 0 | 0 | 0 | 1 | Bacteria | Bacteria\_none | Actinomycetota | Actinomycetes | Micrococcales | Ruaniaceae | Ruania | Bacteria;Bacteria\_none;Actinomycetota;Actinomycetes;Micrococcales;Ruaniaceae;Ruania;Ruania halotolerans |
| Micromonospora aurantiaca | 0 | 0 | 0 | 0 | 0 | 0 | 0 | 0 | 0 | 0 | 0 | 0 | 1 | 1 | Bacteria | Bacteria\_none | Actinomycetota | Actinomycetes | Micromonosporales | Micromonosporaceae | Micromonospora | Bacteria;Bacteria\_none;Actinomycetota;Actinomycetes;Micromonosporales;Micromonosporaceae;Micromonospora;Micromonospora aurantiaca |
| Micromonospora coriariae | 0 | 0 | 0 | 0 | 0 | 0 | 0 | 0 | 0 | 2 | 0 | 0 | 0 | 2 | Bacteria | Bacteria\_none | Actinomycetota | Actinomycetes | Micromonosporales | Micromonosporaceae | Micromonospora | Bacteria;Bacteria\_none;Actinomycetota;Actinomycetes;Micromonosporales;Micromonosporaceae;Micromonospora;Micromonospora coriariae |
| Micromonospora halotolerans | 1 | 0 | 0 | 0 | 0 | 0 | 0 | 0 | 0 | 0 | 0 | 0 | 0 | 1 | Bacteria | Bacteria\_none | Actinomycetota | Actinomycetes | Micromonosporales | Micromonosporaceae | Micromonospora | Bacteria;Bacteria\_none;Actinomycetota;Actinomycetes;Micromonosporales;Micromonosporaceae;Micromonospora;Micromonospora halotolerans |
| Micromonospora sp. DSM 102119 | 0 | 0 | 0 | 1 | 0 | 0 | 0 | 0 | 0 | 0 | 0 | 0 | 0 | 1 | Bacteria | Bacteria\_none | Actinomycetota | Actinomycetes | Micromonosporales | Micromonosporaceae | Micromonospora | Bacteria;Bacteria\_none;Actinomycetota;Actinomycetes;Micromonosporales;Micromonosporaceae;Micromonospora;Micromonospora sp. DSM 102119 |
| Micromonospora sp. WMMD1076 | 0 | 0 | 0 | 0 | 0 | 1 | 0 | 0 | 0 | 0 | 0 | 0 | 0 | 1 | Bacteria | Bacteria\_none | Actinomycetota | Actinomycetes | Micromonosporales | Micromonosporaceae | Micromonospora | Bacteria;Bacteria\_none;Actinomycetota;Actinomycetes;Micromonosporales;Micromonosporaceae;Micromonospora;Micromonospora sp. WMMD1076 |
| Micromonospora sp. WMMD882 | 0 | 0 | 0 | 0 | 0 | 0 | 0 | 1 | 0 | 0 | 0 | 0 | 0 | 1 | Bacteria | Bacteria\_none | Actinomycetota | Actinomycetes | Micromonosporales | Micromonosporaceae | Micromonospora | Bacteria;Bacteria\_none;Actinomycetota;Actinomycetes;Micromonosporales;Micromonosporaceae;Micromonospora;Micromonospora sp. WMMD882 |
| Salinispora tropica | 0 | 0 | 0 | 0 | 0 | 0 | 0 | 0 | 0 | 1 | 0 | 0 | 0 | 1 | Bacteria | Bacteria\_none | Actinomycetota | Actinomycetes | Micromonosporales | Micromonosporaceae | Salinispora | Bacteria;Bacteria\_none;Actinomycetota;Actinomycetes;Micromonosporales;Micromonosporaceae;Salinispora;Salinispora tropica |
| Corynebacterium amycolatum | 0 | 0 | 0 | 0 | 0 | 1 | 0 | 0 | 0 | 0 | 0 | 0 | 0 | 1 | Bacteria | Bacteria\_none | Actinomycetota | Actinomycetes | Mycobacteriales | Corynebacteriaceae | Corynebacterium | Bacteria;Bacteria\_none;Actinomycetota;Actinomycetes;Mycobacteriales;Corynebacteriaceae;Corynebacterium;Corynebacterium amycolatum |
| Corynebacterium choanae | 0 | 0 | 0 | 0 | 0 | 0 | 0 | 0 | 0 | 0 | 0 | 1 | 0 | 1 | Bacteria | Bacteria\_none | Actinomycetota | Actinomycetes | Mycobacteriales | Corynebacteriaceae | Corynebacterium | Bacteria;Bacteria\_none;Actinomycetota;Actinomycetes;Mycobacteriales;Corynebacteriaceae;Corynebacterium;Corynebacterium choanae |
| Corynebacterium durum | 0 | 0 | 0 | 0 | 1 | 1 | 0 | 0 | 0 | 7 | 0 | 1 | 4 | 14 | Bacteria | Bacteria\_none | Actinomycetota | Actinomycetes | Mycobacteriales | Corynebacteriaceae | Corynebacterium | Bacteria;Bacteria\_none;Actinomycetota;Actinomycetes;Mycobacteriales;Corynebacteriaceae;Corynebacterium;Corynebacterium durum |
| Corynebacterium matruchotii | 0 | 6 | 0 | 0 | 0 | 1 | 0 | 0 | 0 | 8 | 0 | 5 | 5 | 25 | Bacteria | Bacteria\_none | Actinomycetota | Actinomycetes | Mycobacteriales | Corynebacteriaceae | Corynebacterium | Bacteria;Bacteria\_none;Actinomycetota;Actinomycetes;Mycobacteriales;Corynebacteriaceae;Corynebacterium;Corynebacterium matruchotii |
| Gordonia sp. L191 | 0 | 0 | 0 | 0 | 1 | 0 | 0 | 0 | 0 | 0 | 0 | 0 | 0 | 1 | Bacteria | Bacteria\_none | Actinomycetota | Actinomycetes | Mycobacteriales | Gordoniaceae | Gordonia | Bacteria;Bacteria\_none;Actinomycetota;Actinomycetes;Mycobacteriales;Gordoniaceae;Gordonia;Gordonia sp. L191 |
| Gordonia sp. PP30 | 0 | 0 | 0 | 0 | 1 | 0 | 0 | 0 | 0 | 0 | 0 | 0 | 0 | 1 | Bacteria | Bacteria\_none | Actinomycetota | Actinomycetes | Mycobacteriales | Gordoniaceae | Gordonia | Bacteria;Bacteria\_none;Actinomycetota;Actinomycetes;Mycobacteriales;Gordoniaceae;Gordonia;Gordonia sp. PP30 |
| Mycobacterium gallinarum | 0 | 0 | 1 | 0 | 0 | 0 | 0 | 0 | 0 | 0 | 0 | 0 | 0 | 1 | Bacteria | Bacteria\_none | Actinomycetota | Actinomycetes | Mycobacteriales | Mycobacteriaceae | Mycobacterium | Bacteria;Bacteria\_none;Actinomycetota;Actinomycetes;Mycobacteriales;Mycobacteriaceae;Mycobacterium;Mycobacterium gallinarum |
| Mycobacterium leprae | 0 | 0 | 0 | 1 | 0 | 0 | 0 | 0 | 0 | 0 | 0 | 0 | 0 | 1 | Bacteria | Bacteria\_none | Actinomycetota | Actinomycetes | Mycobacteriales | Mycobacteriaceae | Mycobacterium | Bacteria;Bacteria\_none;Actinomycetota;Actinomycetes;Mycobacteriales;Mycobacteriaceae;Mycobacterium;Mycobacterium leprae |
| Mycolicibacterium alvei | 0 | 0 | 0 | 0 | 0 | 0 | 1 | 0 | 0 | 0 | 0 | 0 | 0 | 1 | Bacteria | Bacteria\_none | Actinomycetota | Actinomycetes | Mycobacteriales | Mycobacteriaceae | Mycolicibacterium | Bacteria;Bacteria\_none;Actinomycetota;Actinomycetes;Mycobacteriales;Mycobacteriaceae;Mycolicibacterium;Mycolicibacterium alvei |
| Mycolicibacterium aubagnense | 0 | 0 | 1 | 0 | 0 | 1 | 0 | 0 | 0 | 0 | 0 | 0 | 0 | 2 | Bacteria | Bacteria\_none | Actinomycetota | Actinomycetes | Mycobacteriales | Mycobacteriaceae | Mycolicibacterium | Bacteria;Bacteria\_none;Actinomycetota;Actinomycetes;Mycobacteriales;Mycobacteriaceae;Mycolicibacterium;Mycolicibacterium aubagnense |
| Mycolicibacterium diernhoferi | 0 | 1 | 0 | 0 | 0 | 0 | 0 | 0 | 0 | 0 | 0 | 0 | 0 | 1 | Bacteria | Bacteria\_none | Actinomycetota | Actinomycetes | Mycobacteriales | Mycobacteriaceae | Mycolicibacterium | Bacteria;Bacteria\_none;Actinomycetota;Actinomycetes;Mycobacteriales;Mycobacteriaceae;Mycolicibacterium;Mycolicibacterium diernhoferi |
| Mycolicibacterium duvalii | 0 | 0 | 0 | 0 | 0 | 1 | 0 | 0 | 0 | 0 | 0 | 0 | 0 | 1 | Bacteria | Bacteria\_none | Actinomycetota | Actinomycetes | Mycobacteriales | Mycobacteriaceae | Mycolicibacterium | Bacteria;Bacteria\_none;Actinomycetota;Actinomycetes;Mycobacteriales;Mycobacteriaceae;Mycolicibacterium;Mycolicibacterium duvalii |
| Mycolicibacterium gadium | 0 | 0 | 0 | 0 | 0 | 0 | 0 | 0 | 0 | 0 | 0 | 0 | 1 | 1 | Bacteria | Bacteria\_none | Actinomycetota | Actinomycetes | Mycobacteriales | Mycobacteriaceae | Mycolicibacterium | Bacteria;Bacteria\_none;Actinomycetota;Actinomycetes;Mycobacteriales;Mycobacteriaceae;Mycolicibacterium;Mycolicibacterium gadium |
| Mycolicibacterium rhodesiae | 0 | 0 | 0 | 0 | 0 | 0 | 1 | 0 | 0 | 0 | 0 | 0 | 0 | 1 | Bacteria | Bacteria\_none | Actinomycetota | Actinomycetes | Mycobacteriales | Mycobacteriaceae | Mycolicibacterium | Bacteria;Bacteria\_none;Actinomycetota;Actinomycetes;Mycobacteriales;Mycobacteriaceae;Mycolicibacterium;Mycolicibacterium rhodesiae |
| Nocardia sp. NBC\_01503 | 0 | 0 | 0 | 0 | 0 | 0 | 0 | 0 | 0 | 0 | 0 | 0 | 1 | 1 | Bacteria | Bacteria\_none | Actinomycetota | Actinomycetes | Mycobacteriales | Nocardiaceae | Nocardia | Bacteria;Bacteria\_none;Actinomycetota;Actinomycetes;Mycobacteriales;Nocardiaceae;Nocardia;Nocardia sp. NBC\_01503 |
| Rhodococcus sp. USK10 | 0 | 0 | 0 | 0 | 0 | 0 | 0 | 0 | 0 | 0 | 1 | 0 | 0 | 1 | Bacteria | Bacteria\_none | Actinomycetota | Actinomycetes | Mycobacteriales | Nocardiaceae | Rhodococcus | Bacteria;Bacteria\_none;Actinomycetota;Actinomycetes;Mycobacteriales;Nocardiaceae;Rhodococcus;Rhodococcus sp. USK10 |
| Kribbella sp. NBC\_01245 | 0 | 0 | 0 | 0 | 0 | 0 | 0 | 0 | 0 | 0 | 1 | 0 | 0 | 1 | Bacteria | Bacteria\_none | Actinomycetota | Actinomycetes | Propionibacteriales | Kribbellaceae | Kribbella | Bacteria;Bacteria\_none;Actinomycetota;Actinomycetes;Propionibacteriales;Kribbellaceae;Kribbella;Kribbella sp. NBC\_01245 |
| Aeromicrobium wangtongii | 0 | 0 | 0 | 1 | 0 | 0 | 0 | 0 | 0 | 0 | 0 | 0 | 0 | 1 | Bacteria | Bacteria\_none | Actinomycetota | Actinomycetes | Propionibacteriales | Nocardioidaceae | Aeromicrobium | Bacteria;Bacteria\_none;Actinomycetota;Actinomycetes;Propionibacteriales;Nocardioidaceae;Aeromicrobium;Aeromicrobium wangtongii |
| Nocardioides baekrokdamisoli | 0 | 0 | 1 | 0 | 0 | 0 | 0 | 0 | 0 | 0 | 0 | 0 | 0 | 1 | Bacteria | Bacteria\_none | Actinomycetota | Actinomycetes | Propionibacteriales | Nocardioidaceae | Nocardioides | Bacteria;Bacteria\_none;Actinomycetota;Actinomycetes;Propionibacteriales;Nocardioidaceae;Nocardioides;Nocardioides baekrokdamisoli |
| Nocardioides euryhalodurans | 0 | 0 | 0 | 1 | 0 | 0 | 0 | 0 | 0 | 0 | 0 | 0 | 0 | 1 | Bacteria | Bacteria\_none | Actinomycetota | Actinomycetes | Propionibacteriales | Nocardioidaceae | Nocardioides | Bacteria;Bacteria\_none;Actinomycetota;Actinomycetes;Propionibacteriales;Nocardioidaceae;Nocardioides;Nocardioides euryhalodurans |
| Nocardioides scoriae | 0 | 0 | 0 | 0 | 1 | 0 | 0 | 0 | 0 | 0 | 0 | 0 | 0 | 1 | Bacteria | Bacteria\_none | Actinomycetota | Actinomycetes | Propionibacteriales | Nocardioidaceae | Nocardioides | Bacteria;Bacteria\_none;Actinomycetota;Actinomycetes;Propionibacteriales;Nocardioidaceae;Nocardioides;Nocardioides scoriae |
| Acidipropionibacterium acidipropionici | 0 | 0 | 0 | 1 | 0 | 0 | 0 | 0 | 0 | 0 | 0 | 0 | 0 | 1 | Bacteria | Bacteria\_none | Actinomycetota | Actinomycetes | Propionibacteriales | Propionibacteriaceae | Acidipropionibacterium | Bacteria;Bacteria\_none;Actinomycetota;Actinomycetes;Propionibacteriales;Propionibacteriaceae;Acidipropionibacterium;Acidipropionibacterium acidipropionici |
| Arachnia propionica | 0 | 1 | 0 | 0 | 0 | 3 | 0 | 1 | 0 | 0 | 15 | 0 | 6 | 26 | Bacteria | Bacteria\_none | Actinomycetota | Actinomycetes | Propionibacteriales | Propionibacteriaceae | Arachnia | Bacteria;Bacteria\_none;Actinomycetota;Actinomycetes;Propionibacteriales;Propionibacteriaceae;Arachnia;Arachnia propionica |
| Arachnia rubra | 0 | 0 | 0 | 0 | 0 | 0 | 0 | 0 | 0 | 0 | 29 | 0 | 3 | 32 | Bacteria | Bacteria\_none | Actinomycetota | Actinomycetes | Propionibacteriales | Propionibacteriaceae | Arachnia | Bacteria;Bacteria\_none;Actinomycetota;Actinomycetes;Propionibacteriales;Propionibacteriaceae;Arachnia;Arachnia rubra |
| Cutibacterium acnes | 0 | 0 | 0 | 0 | 0 | 0 | 0 | 0 | 0 | 0 | 0 | 0 | 1 | 1 | Bacteria | Bacteria\_none | Actinomycetota | Actinomycetes | Propionibacteriales | Propionibacteriaceae | Cutibacterium | Bacteria;Bacteria\_none;Actinomycetota;Actinomycetes;Propionibacteriales;Propionibacteriaceae;Cutibacterium;Cutibacterium acnes |
| Propionibacterium freudenreichii | 0 | 0 | 0 | 0 | 0 | 0 | 0 | 0 | 0 | 1 | 0 | 0 | 0 | 1 | Bacteria | Bacteria\_none | Actinomycetota | Actinomycetes | Propionibacteriales | Propionibacteriaceae | Propionibacterium | Bacteria;Bacteria\_none;Actinomycetota;Actinomycetes;Propionibacteriales;Propionibacteriaceae;Propionibacterium;Propionibacterium freudenreichii |
| Vaginimicrobium propionicum | 0 | 0 | 0 | 0 | 1 | 0 | 0 | 0 | 0 | 0 | 0 | 0 | 0 | 1 | Bacteria | Bacteria\_none | Actinomycetota | Actinomycetes | Propionibacteriales | Propionibacteriaceae | Vaginimicrobium | Bacteria;Bacteria\_none;Actinomycetota;Actinomycetes;Propionibacteriales;Propionibacteriaceae;Vaginimicrobium;Vaginimicrobium propionicum |
| Amycolatopsis sp. YIM 10 | 0 | 0 | 0 | 1 | 0 | 0 | 0 | 0 | 0 | 0 | 0 | 0 | 0 | 1 | Bacteria | Bacteria\_none | Actinomycetota | Actinomycetes | Pseudonocardiales | Pseudonocardiaceae | Amycolatopsis | Bacteria;Bacteria\_none;Actinomycetota;Actinomycetes;Pseudonocardiales;Pseudonocardiaceae;Amycolatopsis;Amycolatopsis sp. YIM 10 |
| Crossiella sp. CA-258035 | 0 | 0 | 0 | 0 | 1 | 0 | 0 | 0 | 0 | 0 | 0 | 0 | 0 | 1 | Bacteria | Bacteria\_none | Actinomycetota | Actinomycetes | Pseudonocardiales | Pseudonocardiaceae | Crossiella | Bacteria;Bacteria\_none;Actinomycetota;Actinomycetes;Pseudonocardiales;Pseudonocardiaceae;Crossiella;Crossiella sp. CA-258035 |
| Saccharopolyspora spinosa | 0 | 0 | 0 | 0 | 0 | 1 | 0 | 0 | 0 | 0 | 0 | 0 | 0 | 1 | Bacteria | Bacteria\_none | Actinomycetota | Actinomycetes | Pseudonocardiales | Pseudonocardiaceae | Saccharopolyspora | Bacteria;Bacteria\_none;Actinomycetota;Actinomycetes;Pseudonocardiales;Pseudonocardiaceae;Saccharopolyspora;Saccharopolyspora spinosa |
| Nonomuraea fuscirosea | 0 | 0 | 0 | 0 | 0 | 0 | 1 | 0 | 0 | 0 | 0 | 0 | 0 | 1 | Bacteria | Bacteria\_none | Actinomycetota | Actinomycetes | Streptosporangiales | Streptosporangiaceae | Nonomuraea | Bacteria;Bacteria\_none;Actinomycetota;Actinomycetes;Streptosporangiales;Streptosporangiaceae;Nonomuraea;Nonomuraea fuscirosea |
| Nonomuraea phyllanthi | 0 | 0 | 0 | 0 | 0 | 1 | 0 | 0 | 0 | 0 | 0 | 0 | 0 | 1 | Bacteria | Bacteria\_none | Actinomycetota | Actinomycetes | Streptosporangiales | Streptosporangiaceae | Nonomuraea | Bacteria;Bacteria\_none;Actinomycetota;Actinomycetes;Streptosporangiales;Streptosporangiaceae;Nonomuraea;Nonomuraea phyllanthi |
| Streptosporangium roseum | 0 | 0 | 0 | 0 | 1 | 0 | 0 | 0 | 0 | 0 | 0 | 0 | 0 | 1 | Bacteria | Bacteria\_none | Actinomycetota | Actinomycetes | Streptosporangiales | Streptosporangiaceae | Streptosporangium | Bacteria;Bacteria\_none;Actinomycetota;Actinomycetes;Streptosporangiales;Streptosporangiaceae;Streptosporangium;Streptosporangium roseum |
| Streptosporangium sp. NBC\_01495 | 0 | 0 | 1 | 0 | 0 | 0 | 0 | 0 | 0 | 0 | 0 | 0 | 0 | 1 | Bacteria | Bacteria\_none | Actinomycetota | Actinomycetes | Streptosporangiales | Streptosporangiaceae | Streptosporangium | Bacteria;Bacteria\_none;Actinomycetota;Actinomycetes;Streptosporangiales;Streptosporangiaceae;Streptosporangium;Streptosporangium sp. NBC\_01495 |
| Actinomadura madurae | 0 | 0 | 0 | 1 | 0 | 0 | 0 | 0 | 0 | 0 | 0 | 0 | 0 | 1 | Bacteria | Bacteria\_none | Actinomycetota | Actinomycetes | Streptosporangiales | Thermomonosporaceae | Actinomadura | Bacteria;Bacteria\_none;Actinomycetota;Actinomycetes;Streptosporangiales;Thermomonosporaceae;Actinomadura;Actinomadura madurae |
| Thermomonospora amylolytica | 0 | 0 | 0 | 0 | 0 | 0 | 1 | 0 | 0 | 0 | 0 | 0 | 0 | 1 | Bacteria | Bacteria\_none | Actinomycetota | Actinomycetes | Streptosporangiales | Thermomonosporaceae | Thermomonospora | Bacteria;Bacteria\_none;Actinomycetota;Actinomycetes;Streptosporangiales;Thermomonosporaceae;Thermomonospora;Thermomonospora amylolytica |
| Atopobium sp. oral taxon 416 | 0 | 0 | 0 | 0 | 0 | 0 | 0 | 0 | 0 | 0 | 1 | 0 | 0 | 1 | Bacteria | Bacteria\_none | Actinomycetota | Coriobacteriia | Coriobacteriales | Atopobiaceae | Atopobium | Bacteria;Bacteria\_none;Actinomycetota;Coriobacteriia;Coriobacteriales;Atopobiaceae;Atopobium;Atopobium sp. oral taxon 416 |
| Fannyhessea vaginae | 0 | 0 | 0 | 0 | 0 | 0 | 1 | 0 | 0 | 0 | 0 | 0 | 0 | 1 | Bacteria | Bacteria\_none | Actinomycetota | Coriobacteriia | Coriobacteriales | Atopobiaceae | Fannyhessea | Bacteria;Bacteria\_none;Actinomycetota;Coriobacteriia;Coriobacteriales;Atopobiaceae;Fannyhessea;Fannyhessea vaginae |
| Lancefieldella parvula | 28 | 5 | 8 | 0 | 2 | 131 | 60 | 7 | 1 | 0 | 1 | 4 | 34 | 281 | Bacteria | Bacteria\_none | Actinomycetota | Coriobacteriia | Coriobacteriales | Atopobiaceae | Lancefieldella | Bacteria;Bacteria\_none;Actinomycetota;Coriobacteriia;Coriobacteriales;Atopobiaceae;Lancefieldella;Lancefieldella parvula |
| Lancefieldella sp. Marseille-Q7238 | 0 | 0 | 2 | 0 | 0 | 1 | 0 | 0 | 0 | 0 | 5 | 0 | 1 | 9 | Bacteria | Bacteria\_none | Actinomycetota | Coriobacteriia | Coriobacteriales | Atopobiaceae | Lancefieldella | Bacteria;Bacteria\_none;Actinomycetota;Coriobacteriia;Coriobacteriales;Atopobiaceae;Lancefieldella;Lancefieldella sp. Marseille-Q7238 |
| Olsenella uli | 0 | 0 | 0 | 0 | 0 | 1 | 0 | 0 | 0 | 0 | 0 | 0 | 0 | 1 | Bacteria | Bacteria\_none | Actinomycetota | Coriobacteriia | Coriobacteriales | Atopobiaceae | Olsenella | Bacteria;Bacteria\_none;Actinomycetota;Coriobacteriia;Coriobacteriales;Atopobiaceae;Olsenella;Olsenella uli |
| Thermophilibacter mediterraneus | 0 | 0 | 0 | 0 | 0 | 0 | 1 | 0 | 0 | 0 | 0 | 0 | 0 | 1 | Bacteria | Bacteria\_none | Actinomycetota | Coriobacteriia | Coriobacteriales | Atopobiaceae | Thermophilibacter | Bacteria;Bacteria\_none;Actinomycetota;Coriobacteriia;Coriobacteriales;Atopobiaceae;Thermophilibacter;Thermophilibacter mediterraneus |
| Cryptobacterium curtum | 0 | 0 | 0 | 0 | 0 | 1 | 0 | 0 | 0 | 0 | 0 | 0 | 0 | 1 | Bacteria | Bacteria\_none | Actinomycetota | Coriobacteriia | Eggerthellales | Eggerthellaceae | Cryptobacterium | Bacteria;Bacteria\_none;Actinomycetota;Coriobacteriia;Eggerthellales;Eggerthellaceae;Cryptobacterium;Cryptobacterium curtum |
| Denitrobacterium detoxificans | 0 | 0 | 0 | 0 | 0 | 0 | 1 | 0 | 0 | 0 | 0 | 0 | 0 | 1 | Bacteria | Bacteria\_none | Actinomycetota | Coriobacteriia | Eggerthellales | Eggerthellaceae | Denitrobacterium | Bacteria;Bacteria\_none;Actinomycetota;Coriobacteriia;Eggerthellales;Eggerthellaceae;Denitrobacterium;Denitrobacterium detoxificans |
| Raoultibacter timonensis | 0 | 0 | 0 | 0 | 0 | 1 | 0 | 0 | 0 | 0 | 0 | 0 | 0 | 1 | Bacteria | Bacteria\_none | Actinomycetota | Coriobacteriia | Eggerthellales | Eggerthellaceae | Raoultibacter | Bacteria;Bacteria\_none;Actinomycetota;Coriobacteriia;Eggerthellales;Eggerthellaceae;Raoultibacter;Raoultibacter timonensis |
| Rubrobacter radiotolerans | 0 | 0 | 0 | 0 | 1 | 0 | 0 | 0 | 0 | 0 | 0 | 0 | 0 | 1 | Bacteria | Bacteria\_none | Actinomycetota | Rubrobacteria | Rubrobacterales | Rubrobacteraceae | Rubrobacter | Bacteria;Bacteria\_none;Actinomycetota;Rubrobacteria;Rubrobacterales;Rubrobacteraceae;Rubrobacter;Rubrobacter radiotolerans |
| Miltoncostaea marina | 0 | 1 | 0 | 0 | 0 | 0 | 0 | 0 | 0 | 0 | 0 | 0 | 0 | 1 | Bacteria | Bacteria\_none | Actinomycetota | Thermoleophilia | Miltoncostaeales | Miltoncostaeaceae | Miltoncostaea | Bacteria;Bacteria\_none;Actinomycetota;Thermoleophilia;Miltoncostaeales;Miltoncostaeaceae;Miltoncostaea;Miltoncostaea marina |
| Baekduia alba | 0 | 1 | 0 | 0 | 0 | 0 | 0 | 0 | 0 | 0 | 0 | 0 | 0 | 1 | Bacteria | Bacteria\_none | Actinomycetota | Thermoleophilia | Solirubrobacterales | Baekduiaceae | Baekduia | Bacteria;Bacteria\_none;Actinomycetota;Thermoleophilia;Solirubrobacterales;Baekduiaceae;Baekduia;Baekduia alba |
| Abyssicoccus albus | 0 | 0 | 0 | 0 | 1 | 0 | 0 | 0 | 0 | 0 | 0 | 0 | 0 | 1 | Bacteria | Bacteria\_none | Bacillota | Bacilli | Bacillales | Abyssicoccaceae | Abyssicoccus | Bacteria;Bacteria\_none;Bacillota;Bacilli;Bacillales;Abyssicoccaceae;Abyssicoccus;Abyssicoccus albus |
| Alicyclobacillus fastidiosus | 0 | 0 | 0 | 0 | 0 | 0 | 0 | 0 | 0 | 1 | 0 | 0 | 0 | 1 | Bacteria | Bacteria\_none | Bacillota | Bacilli | Bacillales | Alicyclobacillaceae | Alicyclobacillus | Bacteria;Bacteria\_none;Bacillota;Bacilli;Bacillales;Alicyclobacillaceae;Alicyclobacillus;Alicyclobacillus fastidiosus |
| Alkalihalobacillus sp. LMS39 | 0 | 0 | 0 | 1 | 0 | 0 | 0 | 0 | 0 | 0 | 0 | 0 | 1 | 2 | Bacteria | Bacteria\_none | Bacillota | Bacilli | Bacillales | Bacillaceae | Alkalihalobacillus | Bacteria;Bacteria\_none;Bacillota;Bacilli;Bacillales;Bacillaceae;Alkalihalobacillus;Alkalihalobacillus sp. LMS39 |
| Alkalihalophilus pseudofirmus | 0 | 0 | 0 | 0 | 0 | 0 | 0 | 0 | 0 | 0 | 1 | 0 | 1 | 2 | Bacteria | Bacteria\_none | Bacillota | Bacilli | Bacillales | Bacillaceae | Alkalihalophilus | Bacteria;Bacteria\_none;Bacillota;Bacilli;Bacillales;Bacillaceae;Alkalihalophilus;Alkalihalophilus pseudofirmus |
| Amphibacillus xylanus | 0 | 0 | 0 | 0 | 0 | 1 | 0 | 0 | 0 | 0 | 0 | 0 | 0 | 1 | Bacteria | Bacteria\_none | Bacillota | Bacilli | Bacillales | Bacillaceae | Amphibacillus | Bacteria;Bacteria\_none;Bacillota;Bacilli;Bacillales;Bacillaceae;Amphibacillus;Amphibacillus xylanus |
| Anaerobacillus isosaccharinicus | 0 | 1 | 0 | 0 | 0 | 0 | 0 | 0 | 0 | 0 | 0 | 0 | 0 | 1 | Bacteria | Bacteria\_none | Bacillota | Bacilli | Bacillales | Bacillaceae | Anaerobacillus | Bacteria;Bacteria\_none;Bacillota;Bacilli;Bacillales;Bacillaceae;Anaerobacillus;Anaerobacillus isosaccharinicus |
| Anoxybacillus sp. FSL W8-1294 | 1 | 0 | 0 | 0 | 0 | 0 | 0 | 0 | 0 | 0 | 0 | 0 | 0 | 1 | Bacteria | Bacteria\_none | Bacillota | Bacilli | Bacillales | Bacillaceae | Anoxybacillus | Bacteria;Bacteria\_none;Bacillota;Bacilli;Bacillales;Bacillaceae;Anoxybacillus;Anoxybacillus sp. FSL W8-1294 |
| Bacillus amyloliquefaciens | 0 | 0 | 0 | 0 | 0 | 0 | 0 | 0 | 0 | 0 | 1 | 0 | 0 | 1 | Bacteria | Bacteria\_none | Bacillota | Bacilli | Bacillales | Bacillaceae | Bacillus | Bacteria;Bacteria\_none;Bacillota;Bacilli;Bacillales;Bacillaceae;Bacillus;Bacillus amyloliquefaciens |
| Bacillus cereus | 0 | 0 | 0 | 0 | 0 | 2 | 0 | 0 | 0 | 0 | 0 | 0 | 0 | 2 | Bacteria | Bacteria\_none | Bacillota | Bacilli | Bacillales | Bacillaceae | Bacillus | Bacteria;Bacteria\_none;Bacillota;Bacilli;Bacillales;Bacillaceae;Bacillus;Bacillus cereus |
| Bacillus methanolicus | 0 | 0 | 0 | 0 | 1 | 0 | 0 | 0 | 0 | 0 | 0 | 0 | 0 | 1 | Bacteria | Bacteria\_none | Bacillota | Bacilli | Bacillales | Bacillaceae | Bacillus | Bacteria;Bacteria\_none;Bacillota;Bacilli;Bacillales;Bacillaceae;Bacillus;Bacillus methanolicus |
| Bacillus mycoides | 0 | 0 | 0 | 0 | 0 | 1 | 0 | 0 | 0 | 0 | 0 | 0 | 0 | 1 | Bacteria | Bacteria\_none | Bacillota | Bacilli | Bacillales | Bacillaceae | Bacillus | Bacteria;Bacteria\_none;Bacillota;Bacilli;Bacillales;Bacillaceae;Bacillus;Bacillus mycoides |
| Bacillus nitratireducens | 0 | 0 | 0 | 1 | 0 | 0 | 0 | 1 | 0 | 0 | 0 | 0 | 0 | 2 | Bacteria | Bacteria\_none | Bacillota | Bacilli | Bacillales | Bacillaceae | Bacillus | Bacteria;Bacteria\_none;Bacillota;Bacilli;Bacillales;Bacillaceae;Bacillus;Bacillus nitratireducens |
| Bacillus pseudomycoides | 0 | 0 | 0 | 0 | 0 | 1 | 0 | 0 | 0 | 0 | 0 | 0 | 0 | 1 | Bacteria | Bacteria\_none | Bacillota | Bacilli | Bacillales | Bacillaceae | Bacillus | Bacteria;Bacteria\_none;Bacillota;Bacilli;Bacillales;Bacillaceae;Bacillus;Bacillus pseudomycoides |
| Bacillus safensis | 0 | 0 | 0 | 0 | 0 | 0 | 0 | 0 | 0 | 1 | 0 | 0 | 0 | 1 | Bacteria | Bacteria\_none | Bacillota | Bacilli | Bacillales | Bacillaceae | Bacillus | Bacteria;Bacteria\_none;Bacillota;Bacilli;Bacillales;Bacillaceae;Bacillus;Bacillus safensis |
| Bacillus sp. 1NLA3E | 0 | 0 | 0 | 1 | 0 | 0 | 0 | 0 | 0 | 0 | 0 | 0 | 0 | 1 | Bacteria | Bacteria\_none | Bacillota | Bacilli | Bacillales | Bacillaceae | Bacillus | Bacteria;Bacteria\_none;Bacillota;Bacilli;Bacillales;Bacillaceae;Bacillus;Bacillus sp. 1NLA3E |
| Bacillus sp. FJAT-22090 | 0 | 0 | 0 | 0 | 0 | 1 | 0 | 0 | 0 | 0 | 0 | 0 | 0 | 1 | Bacteria | Bacteria\_none | Bacillota | Bacilli | Bacillales | Bacillaceae | Bacillus | Bacteria;Bacteria\_none;Bacillota;Bacilli;Bacillales;Bacillaceae;Bacillus;Bacillus sp. FJAT-22090 |
| Bacillus sp. N5-665 | 0 | 0 | 0 | 0 | 0 | 0 | 0 | 0 | 0 | 0 | 0 | 0 | 1 | 1 | Bacteria | Bacteria\_none | Bacillota | Bacilli | Bacillales | Bacillaceae | Bacillus | Bacteria;Bacteria\_none;Bacillota;Bacilli;Bacillales;Bacillaceae;Bacillus;Bacillus sp. N5-665 |
| Bacillus sp. NEB1478 | 1 | 0 | 0 | 0 | 0 | 0 | 0 | 0 | 0 | 0 | 0 | 0 | 0 | 1 | Bacteria | Bacteria\_none | Bacillota | Bacilli | Bacillales | Bacillaceae | Bacillus | Bacteria;Bacteria\_none;Bacillota;Bacilli;Bacillales;Bacillaceae;Bacillus;Bacillus sp. NEB1478 |
| Bacillus thuringiensis | 0 | 0 | 0 | 1 | 0 | 0 | 0 | 0 | 0 | 0 | 0 | 0 | 0 | 1 | Bacteria | Bacteria\_none | Bacillota | Bacilli | Bacillales | Bacillaceae | Bacillus | Bacteria;Bacteria\_none;Bacillota;Bacilli;Bacillales;Bacillaceae;Bacillus;Bacillus thuringiensis |
| Bacillus wiedmannii | 0 | 0 | 0 | 0 | 0 | 0 | 0 | 0 | 0 | 0 | 0 | 0 | 1 | 1 | Bacteria | Bacteria\_none | Bacillota | Bacilli | Bacillales | Bacillaceae | Bacillus | Bacteria;Bacteria\_none;Bacillota;Bacilli;Bacillales;Bacillaceae;Bacillus;Bacillus wiedmannii |
| Cytobacillus firmus | 0 | 0 | 0 | 0 | 0 | 0 | 0 | 0 | 0 | 0 | 0 | 0 | 1 | 1 | Bacteria | Bacteria\_none | Bacillota | Bacilli | Bacillales | Bacillaceae | Cytobacillus | Bacteria;Bacteria\_none;Bacillota;Bacilli;Bacillales;Bacillaceae;Cytobacillus;Cytobacillus firmus |
| Cytobacillus gottheilii | 0 | 0 | 1 | 0 | 1 | 0 | 0 | 0 | 0 | 0 | 0 | 0 | 0 | 2 | Bacteria | Bacteria\_none | Bacillota | Bacilli | Bacillales | Bacillaceae | Cytobacillus | Bacteria;Bacteria\_none;Bacillota;Bacilli;Bacillales;Bacillaceae;Cytobacillus;Cytobacillus gottheilii |
| Cytobacillus oceanisediminis | 0 | 0 | 0 | 1 | 0 | 0 | 0 | 0 | 0 | 0 | 0 | 0 | 0 | 1 | Bacteria | Bacteria\_none | Bacillota | Bacilli | Bacillales | Bacillaceae | Cytobacillus | Bacteria;Bacteria\_none;Bacillota;Bacilli;Bacillales;Bacillaceae;Cytobacillus;Cytobacillus oceanisediminis |
| Domibacillus sp. DTU\_2020\_1001157\_1\_SI\_ALB\_TIR\_016 | 0 | 0 | 0 | 0 | 0 | 0 | 0 | 0 | 0 | 0 | 0 | 0 | 1 | 1 | Bacteria | Bacteria\_none | Bacillota | Bacilli | Bacillales | Bacillaceae | Domibacillus | Bacteria;Bacteria\_none;Bacillota;Bacilli;Bacillales;Bacillaceae;Domibacillus;Domibacillus sp. DTU\_2020\_1001157\_1\_SI\_ALB\_TIR\_016 |
| Halalkalibacter krulwichiae | 0 | 0 | 0 | 0 | 0 | 0 | 1 | 0 | 0 | 0 | 0 | 0 | 0 | 1 | Bacteria | Bacteria\_none | Bacillota | Bacilli | Bacillales | Bacillaceae | Halalkalibacter | Bacteria;Bacteria\_none;Bacillota;Bacilli;Bacillales;Bacillaceae;Halalkalibacter;Halalkalibacter krulwichiae |
| Halobacillus litoralis | 0 | 0 | 0 | 0 | 0 | 0 | 0 | 0 | 0 | 0 | 0 | 0 | 1 | 1 | Bacteria | Bacteria\_none | Bacillota | Bacilli | Bacillales | Bacillaceae | Halobacillus | Bacteria;Bacteria\_none;Bacillota;Bacilli;Bacillales;Bacillaceae;Halobacillus;Halobacillus litoralis |
| Lysinibacillus capsici | 0 | 1 | 0 | 0 | 0 | 0 | 0 | 0 | 0 | 0 | 0 | 0 | 0 | 1 | Bacteria | Bacteria\_none | Bacillota | Bacilli | Bacillales | Bacillaceae | Lysinibacillus | Bacteria;Bacteria\_none;Bacillota;Bacilli;Bacillales;Bacillaceae;Lysinibacillus;Lysinibacillus capsici |
| Lysinibacillus fusiformis | 0 | 0 | 0 | 0 | 0 | 0 | 1 | 0 | 0 | 0 | 0 | 0 | 0 | 1 | Bacteria | Bacteria\_none | Bacillota | Bacilli | Bacillales | Bacillaceae | Lysinibacillus | Bacteria;Bacteria\_none;Bacillota;Bacilli;Bacillales;Bacillaceae;Lysinibacillus;Lysinibacillus fusiformis |
| Lysinibacillus irui | 0 | 0 | 1 | 0 | 0 | 1 | 0 | 0 | 0 | 0 | 0 | 0 | 0 | 2 | Bacteria | Bacteria\_none | Bacillota | Bacilli | Bacillales | Bacillaceae | Lysinibacillus | Bacteria;Bacteria\_none;Bacillota;Bacilli;Bacillales;Bacillaceae;Lysinibacillus;Lysinibacillus irui |
| Lysinibacillus macroides | 0 | 0 | 0 | 0 | 0 | 0 | 0 | 0 | 0 | 0 | 1 | 0 | 0 | 1 | Bacteria | Bacteria\_none | Bacillota | Bacilli | Bacillales | Bacillaceae | Lysinibacillus | Bacteria;Bacteria\_none;Bacillota;Bacilli;Bacillales;Bacillaceae;Lysinibacillus;Lysinibacillus macroides |
| Lysinibacillus sphaericus | 0 | 0 | 0 | 0 | 0 | 0 | 0 | 0 | 0 | 0 | 0 | 0 | 1 | 1 | Bacteria | Bacteria\_none | Bacillota | Bacilli | Bacillales | Bacillaceae | Lysinibacillus | Bacteria;Bacteria\_none;Bacillota;Bacilli;Bacillales;Bacillaceae;Lysinibacillus;Lysinibacillus sphaericus |
| Metabacillus sp. FJAT-53654 | 0 | 1 | 0 | 0 | 0 | 0 | 0 | 0 | 0 | 0 | 0 | 0 | 0 | 1 | Bacteria | Bacteria\_none | Bacillota | Bacilli | Bacillales | Bacillaceae | Metabacillus | Bacteria;Bacteria\_none;Bacillota;Bacilli;Bacillales;Bacillaceae;Metabacillus;Metabacillus sp. FJAT-53654 |
| Neobacillus sp. FSL H8-0543 | 0 | 0 | 1 | 0 | 0 | 0 | 0 | 0 | 0 | 0 | 0 | 0 | 0 | 1 | Bacteria | Bacteria\_none | Bacillota | Bacilli | Bacillales | Bacillaceae | Neobacillus | Bacteria;Bacteria\_none;Bacillota;Bacilli;Bacillales;Bacillaceae;Neobacillus;Neobacillus sp. FSL H8-0543 |
| Neobacillus sp. OS1-2 | 0 | 0 | 0 | 0 | 1 | 0 | 0 | 0 | 0 | 0 | 0 | 0 | 0 | 1 | Bacteria | Bacteria\_none | Bacillota | Bacilli | Bacillales | Bacillaceae | Neobacillus | Bacteria;Bacteria\_none;Bacillota;Bacilli;Bacillales;Bacillaceae;Neobacillus;Neobacillus sp. OS1-2 |
| Niallia oryzisoli | 0 | 0 | 1 | 0 | 0 | 1 | 0 | 0 | 0 | 0 | 0 | 0 | 0 | 2 | Bacteria | Bacteria\_none | Bacillota | Bacilli | Bacillales | Bacillaceae | Niallia | Bacteria;Bacteria\_none;Bacillota;Bacilli;Bacillales;Bacillaceae;Niallia;Niallia oryzisoli |
| Niallia sp. XMNu-256 | 0 | 0 | 0 | 0 | 0 | 0 | 0 | 0 | 0 | 0 | 0 | 0 | 1 | 1 | Bacteria | Bacteria\_none | Bacillota | Bacilli | Bacillales | Bacillaceae | Niallia | Bacteria;Bacteria\_none;Bacillota;Bacilli;Bacillales;Bacillaceae;Niallia;Niallia sp. XMNu-256 |
| Niallia taxi | 1 | 0 | 0 | 0 | 0 | 0 | 0 | 0 | 0 | 0 | 0 | 0 | 0 | 1 | Bacteria | Bacteria\_none | Bacillota | Bacilli | Bacillales | Bacillaceae | Niallia | Bacteria;Bacteria\_none;Bacillota;Bacilli;Bacillales;Bacillaceae;Niallia;Niallia taxi |
| Oceanobacillus kimchii | 0 | 0 | 0 | 0 | 0 | 1 | 0 | 0 | 0 | 0 | 0 | 0 | 1 | 2 | Bacteria | Bacteria\_none | Bacillota | Bacilli | Bacillales | Bacillaceae | Oceanobacillus | Bacteria;Bacteria\_none;Bacillota;Bacilli;Bacillales;Bacillaceae;Oceanobacillus;Oceanobacillus kimchii |
| Oceanobacillus sp. FSL W7-1293 | 0 | 0 | 0 | 0 | 0 | 1 | 0 | 0 | 0 | 0 | 0 | 0 | 0 | 1 | Bacteria | Bacteria\_none | Bacillota | Bacilli | Bacillales | Bacillaceae | Oceanobacillus | Bacteria;Bacteria\_none;Bacillota;Bacilli;Bacillales;Bacillaceae;Oceanobacillus;Oceanobacillus sp. FSL W7-1293 |
| Ornithinibacillus sp. FSL M8-0202 | 0 | 0 | 0 | 0 | 0 | 1 | 0 | 0 | 0 | 0 | 0 | 0 | 0 | 1 | Bacteria | Bacteria\_none | Bacillota | Bacilli | Bacillales | Bacillaceae | Ornithinibacillus | Bacteria;Bacteria\_none;Bacillota;Bacilli;Bacillales;Bacillaceae;Ornithinibacillus;Ornithinibacillus sp. FSL M8-0202 |
| Parageobacillus toebii | 0 | 0 | 0 | 0 | 0 | 0 | 0 | 0 | 0 | 0 | 0 | 0 | 1 | 1 | Bacteria | Bacteria\_none | Bacillota | Bacilli | Bacillales | Bacillaceae | Parageobacillus | Bacteria;Bacteria\_none;Bacillota;Bacilli;Bacillales;Bacillaceae;Parageobacillus;Parageobacillus toebii |
| Peribacillus butanolivorans | 0 | 0 | 0 | 0 | 0 | 0 | 0 | 1 | 0 | 0 | 0 | 0 | 0 | 1 | Bacteria | Bacteria\_none | Bacillota | Bacilli | Bacillales | Bacillaceae | Peribacillus | Bacteria;Bacteria\_none;Bacillota;Bacilli;Bacillales;Bacillaceae;Peribacillus;Peribacillus butanolivorans |
| Pontibacillus sp. HMF3514 | 0 | 0 | 0 | 1 | 0 | 0 | 0 | 0 | 0 | 0 | 0 | 0 | 0 | 1 | Bacteria | Bacteria\_none | Bacillota | Bacilli | Bacillales | Bacillaceae | Pontibacillus | Bacteria;Bacteria\_none;Bacillota;Bacilli;Bacillales;Bacillaceae;Pontibacillus;Pontibacillus sp. HMF3514 |
| Priestia aryabhattai | 0 | 0 | 0 | 0 | 2 | 0 | 0 | 0 | 0 | 0 | 0 | 0 | 0 | 2 | Bacteria | Bacteria\_none | Bacillota | Bacilli | Bacillales | Bacillaceae | Priestia | Bacteria;Bacteria\_none;Bacillota;Bacilli;Bacillales;Bacillaceae;Priestia;Priestia aryabhattai |
| Priestia megaterium | 0 | 0 | 1 | 0 | 0 | 1 | 1 | 0 | 0 | 2 | 1 | 0 | 2 | 8 | Bacteria | Bacteria\_none | Bacillota | Bacilli | Bacillales | Bacillaceae | Priestia | Bacteria;Bacteria\_none;Bacillota;Bacilli;Bacillales;Bacillaceae;Priestia;Priestia megaterium |
| Pseudalkalibacillus hwajinpoensis | 0 | 0 | 0 | 1 | 0 | 0 | 0 | 0 | 0 | 0 | 0 | 0 | 0 | 1 | Bacteria | Bacteria\_none | Bacillota | Bacilli | Bacillales | Bacillaceae | Pseudalkalibacillus | Bacteria;Bacteria\_none;Bacillota;Bacilli;Bacillales;Bacillaceae;Pseudalkalibacillus;Pseudalkalibacillus hwajinpoensis |
| Psychrobacillus glaciei | 1 | 0 | 0 | 0 | 0 | 0 | 0 | 0 | 0 | 0 | 0 | 0 | 0 | 1 | Bacteria | Bacteria\_none | Bacillota | Bacilli | Bacillales | Bacillaceae | Psychrobacillus | Bacteria;Bacteria\_none;Bacillota;Bacilli;Bacillales;Bacillaceae;Psychrobacillus;Psychrobacillus glaciei |
| Psychrobacillus sp. INOP01 | 0 | 0 | 0 | 0 | 0 | 0 | 0 | 0 | 0 | 0 | 0 | 0 | 1 | 1 | Bacteria | Bacteria\_none | Bacillota | Bacilli | Bacillales | Bacillaceae | Psychrobacillus | Bacteria;Bacteria\_none;Bacillota;Bacilli;Bacillales;Bacillaceae;Psychrobacillus;Psychrobacillus sp. INOP01 |
| Rossellomorea aquimaris | 0 | 0 | 0 | 0 | 0 | 1 | 0 | 0 | 0 | 0 | 0 | 0 | 0 | 1 | Bacteria | Bacteria\_none | Bacillota | Bacilli | Bacillales | Bacillaceae | Rossellomorea | Bacteria;Bacteria\_none;Bacillota;Bacilli;Bacillales;Bacillaceae;Rossellomorea;Rossellomorea aquimaris |
| Salipaludibacillus sp. LMS25 | 0 | 0 | 0 | 1 | 0 | 0 | 0 | 0 | 0 | 0 | 0 | 0 | 0 | 1 | Bacteria | Bacteria\_none | Bacillota | Bacilli | Bacillales | Bacillaceae | Salipaludibacillus | Bacteria;Bacteria\_none;Bacillota;Bacilli;Bacillales;Bacillaceae;Salipaludibacillus;Salipaludibacillus sp. LMS25 |
| Shouchella lehensis | 0 | 0 | 0 | 0 | 0 | 0 | 0 | 0 | 0 | 0 | 0 | 0 | 2 | 2 | Bacteria | Bacteria\_none | Bacillota | Bacilli | Bacillales | Bacillaceae | Shouchella | Bacteria;Bacteria\_none;Bacillota;Bacilli;Bacillales;Bacillaceae;Shouchella;Shouchella lehensis |
| Sutcliffiella horikoshii | 0 | 0 | 0 | 1 | 1 | 0 | 0 | 0 | 0 | 0 | 0 | 0 | 1 | 3 | Bacteria | Bacteria\_none | Bacillota | Bacilli | Bacillales | Bacillaceae | Sutcliffiella | Bacteria;Bacteria\_none;Bacillota;Bacilli;Bacillales;Bacillaceae;Sutcliffiella;Sutcliffiella horikoshii |
| Terribacillus saccharophilus | 0 | 0 | 2 | 0 | 0 | 0 | 0 | 0 | 0 | 0 | 0 | 0 | 0 | 2 | Bacteria | Bacteria\_none | Bacillota | Bacilli | Bacillales | Bacillaceae | Terribacillus | Bacteria;Bacteria\_none;Bacillota;Bacilli;Bacillales;Bacillaceae;Terribacillus;Terribacillus saccharophilus |
| Virgibacillus sp. NKC19-16 | 0 | 0 | 0 | 0 | 0 | 0 | 0 | 1 | 0 | 0 | 0 | 0 | 2 | 3 | Bacteria | Bacteria\_none | Bacillota | Bacilli | Bacillales | Bacillaceae | Virgibacillus | Bacteria;Bacteria\_none;Bacillota;Bacilli;Bacillales;Bacillaceae;Virgibacillus;Virgibacillus sp. NKC19-16 |
| Exiguobacterium sp. 9-2 | 0 | 0 | 0 | 1 | 0 | 0 | 0 | 0 | 0 | 0 | 0 | 0 | 0 | 1 | Bacteria | Bacteria\_none | Bacillota | Bacilli | Bacillales | Bacillales\_Incertae\_sedis | Exiguobacterium | Bacteria;Bacteria\_none;Bacillota;Bacilli;Bacillales;Bacillales\_Incertae\_sedis;Exiguobacterium;Exiguobacterium sp. 9-2 |
| Gemella haemolysans | 28 | 33 | 2 | 111 | 303 | 158 | 7 | 17 | 1 | 301 | 1001 | 16 | 565 | 2543 | Bacteria | Bacteria\_none | Bacillota | Bacilli | Bacillales | Gemellaceae | Gemella | Bacteria;Bacteria\_none;Bacillota;Bacilli;Bacillales;Gemellaceae;Gemella;Gemella haemolysans |
| Gemella morbillorum | 0 | 21 | 4 | 2 | 183 | 111 | 1 | 44 | 1 | 32 | 113 | 29 | 185 | 726 | Bacteria | Bacteria\_none | Bacillota | Bacilli | Bacillales | Gemellaceae | Gemella | Bacteria;Bacteria\_none;Bacillota;Bacilli;Bacillales;Gemellaceae;Gemella;Gemella morbillorum |
| Gemella sanguinis | 46 | 71 | 1 | 18 | 77 | 77 | 124 | 51 | 5 | 53 | 60 | 55 | 229 | 867 | Bacteria | Bacteria\_none | Bacillota | Bacilli | Bacillales | Gemellaceae | Gemella | Bacteria;Bacteria\_none;Bacillota;Bacilli;Bacillales;Gemellaceae;Gemella;Gemella sanguinis |
| Gemella sp. oral taxon 928 | 0 | 0 | 0 | 0 | 0 | 1 | 0 | 3 | 0 | 0 | 0 | 0 | 2 | 6 | Bacteria | Bacteria\_none | Bacillota | Bacilli | Bacillales | Gemellaceae | Gemella | Bacteria;Bacteria\_none;Bacillota;Bacilli;Bacillales;Gemellaceae;Gemella;Gemella sp. oral taxon 928 |
| Gemella sp. zg-570 | 0 | 0 | 0 | 0 | 0 | 0 | 0 | 0 | 0 | 0 | 2 | 0 | 0 | 2 | Bacteria | Bacteria\_none | Bacillota | Bacilli | Bacillales | Gemellaceae | Gemella | Bacteria;Bacteria\_none;Bacillota;Bacilli;Bacillales;Gemellaceae;Gemella;Gemella sp. zg-570 |
| Brochothrix thermosphacta | 0 | 0 | 0 | 0 | 0 | 0 | 0 | 0 | 0 | 0 | 0 | 0 | 1 | 1 | Bacteria | Bacteria\_none | Bacillota | Bacilli | Bacillales | Listeriaceae | Brochothrix | Bacteria;Bacteria\_none;Bacillota;Bacilli;Bacillales;Listeriaceae;Brochothrix;Brochothrix thermosphacta |
| Listeria monocytogenes | 0 | 0 | 0 | 0 | 0 | 0 | 0 | 0 | 0 | 0 | 0 | 0 | 1 | 1 | Bacteria | Bacteria\_none | Bacillota | Bacilli | Bacillales | Listeriaceae | Listeria | Bacteria;Bacteria\_none;Bacillota;Bacilli;Bacillales;Listeriaceae;Listeria;Listeria monocytogenes |
| Brevibacillus borstelensis | 0 | 0 | 0 | 1 | 0 | 0 | 0 | 0 | 0 | 0 | 0 | 0 | 0 | 1 | Bacteria | Bacteria\_none | Bacillota | Bacilli | Bacillales | Paenibacillaceae | Brevibacillus | Bacteria;Bacteria\_none;Bacillota;Bacilli;Bacillales;Paenibacillaceae;Brevibacillus;Brevibacillus borstelensis |
| Brevibacillus brevis | 0 | 0 | 0 | 0 | 0 | 1 | 0 | 0 | 0 | 0 | 0 | 0 | 0 | 1 | Bacteria | Bacteria\_none | Bacillota | Bacilli | Bacillales | Paenibacillaceae | Brevibacillus | Bacteria;Bacteria\_none;Bacillota;Bacilli;Bacillales;Paenibacillaceae;Brevibacillus;Brevibacillus brevis |
| Brevibacillus formosus | 0 | 0 | 0 | 0 | 0 | 1 | 0 | 0 | 0 | 0 | 0 | 0 | 0 | 1 | Bacteria | Bacteria\_none | Bacillota | Bacilli | Bacillales | Paenibacillaceae | Brevibacillus | Bacteria;Bacteria\_none;Bacillota;Bacilli;Bacillales;Paenibacillaceae;Brevibacillus;Brevibacillus formosus |
| Brevibacillus laterosporus | 0 | 0 | 0 | 0 | 0 | 0 | 0 | 0 | 0 | 0 | 1 | 0 | 0 | 1 | Bacteria | Bacteria\_none | Bacillota | Bacilli | Bacillales | Paenibacillaceae | Brevibacillus | Bacteria;Bacteria\_none;Bacillota;Bacilli;Bacillales;Paenibacillaceae;Brevibacillus;Brevibacillus laterosporus |
| Paenibacillus andongensis | 0 | 0 | 0 | 0 | 1 | 0 | 0 | 0 | 0 | 0 | 0 | 0 | 0 | 1 | Bacteria | Bacteria\_none | Bacillota | Bacilli | Bacillales | Paenibacillaceae | Paenibacillus | Bacteria;Bacteria\_none;Bacillota;Bacilli;Bacillales;Paenibacillaceae;Paenibacillus;Paenibacillus andongensis |
| Paenibacillus azoreducens | 0 | 0 | 0 | 0 | 0 | 0 | 0 | 0 | 0 | 0 | 0 | 0 | 1 | 1 | Bacteria | Bacteria\_none | Bacillota | Bacilli | Bacillales | Paenibacillaceae | Paenibacillus | Bacteria;Bacteria\_none;Bacillota;Bacilli;Bacillales;Paenibacillaceae;Paenibacillus;Paenibacillus azoreducens |
| Paenibacillus barcinonensis | 0 | 0 | 1 | 0 | 0 | 0 | 0 | 0 | 0 | 0 | 0 | 0 | 0 | 1 | Bacteria | Bacteria\_none | Bacillota | Bacilli | Bacillales | Paenibacillaceae | Paenibacillus | Bacteria;Bacteria\_none;Bacillota;Bacilli;Bacillales;Paenibacillaceae;Paenibacillus;Paenibacillus barcinonensis |
| Paenibacillus chitinolyticus | 0 | 0 | 1 | 0 | 0 | 0 | 0 | 0 | 0 | 0 | 0 | 0 | 0 | 1 | Bacteria | Bacteria\_none | Bacillota | Bacilli | Bacillales | Paenibacillaceae | Paenibacillus | Bacteria;Bacteria\_none;Bacillota;Bacilli;Bacillales;Paenibacillaceae;Paenibacillus;Paenibacillus chitinolyticus |
| Paenibacillus marchantiae | 0 | 1 | 0 | 0 | 0 | 0 | 0 | 0 | 0 | 0 | 0 | 0 | 0 | 1 | Bacteria | Bacteria\_none | Bacillota | Bacilli | Bacillales | Paenibacillaceae | Paenibacillus | Bacteria;Bacteria\_none;Bacillota;Bacilli;Bacillales;Paenibacillaceae;Paenibacillus;Paenibacillus marchantiae |
| Paenibacillus sophorae | 0 | 0 | 0 | 0 | 0 | 0 | 3 | 0 | 0 | 0 | 0 | 0 | 0 | 3 | Bacteria | Bacteria\_none | Bacillota | Bacilli | Bacillales | Paenibacillaceae | Paenibacillus | Bacteria;Bacteria\_none;Bacillota;Bacilli;Bacillales;Paenibacillaceae;Paenibacillus;Paenibacillus sophorae |
| Paenibacillus sp. FSL H8-0332 | 1 | 0 | 0 | 0 | 0 | 0 | 0 | 0 | 0 | 0 | 0 | 0 | 0 | 1 | Bacteria | Bacteria\_none | Bacillota | Bacilli | Bacillales | Paenibacillaceae | Paenibacillus | Bacteria;Bacteria\_none;Bacillota;Bacilli;Bacillales;Paenibacillaceae;Paenibacillus;Paenibacillus sp. FSL H8-0332 |
| Paenibacillus sp. FSL H8-0537 | 0 | 0 | 0 | 0 | 0 | 0 | 0 | 0 | 0 | 0 | 1 | 0 | 0 | 1 | Bacteria | Bacteria\_none | Bacillota | Bacilli | Bacillales | Paenibacillaceae | Paenibacillus | Bacteria;Bacteria\_none;Bacillota;Bacilli;Bacillales;Paenibacillaceae;Paenibacillus;Paenibacillus sp. FSL H8-0537 |
| Paenibacillus sp. FSL L8-0435 | 0 | 0 | 0 | 0 | 0 | 0 | 0 | 0 | 0 | 0 | 0 | 0 | 2 | 2 | Bacteria | Bacteria\_none | Bacillota | Bacilli | Bacillales | Paenibacillaceae | Paenibacillus | Bacteria;Bacteria\_none;Bacillota;Bacilli;Bacillales;Paenibacillaceae;Paenibacillus;Paenibacillus sp. FSL L8-0435 |
| Paenibacillus sp. FSL R10-2734 | 0 | 0 | 0 | 0 | 0 | 0 | 0 | 0 | 0 | 0 | 0 | 0 | 2 | 2 | Bacteria | Bacteria\_none | Bacillota | Bacilli | Bacillales | Paenibacillaceae | Paenibacillus | Bacteria;Bacteria\_none;Bacillota;Bacilli;Bacillales;Paenibacillaceae;Paenibacillus;Paenibacillus sp. FSL R10-2734 |
| Paenibacillus sp. FSL R10-2796 | 0 | 0 | 0 | 1 | 0 | 0 | 0 | 0 | 0 | 0 | 0 | 0 | 0 | 1 | Bacteria | Bacteria\_none | Bacillota | Bacilli | Bacillales | Paenibacillaceae | Paenibacillus | Bacteria;Bacteria\_none;Bacillota;Bacilli;Bacillales;Paenibacillaceae;Paenibacillus;Paenibacillus sp. FSL R10-2796 |
| Paenibacillus sp. FSL R5-0490 | 0 | 0 | 0 | 0 | 0 | 0 | 0 | 0 | 0 | 0 | 1 | 0 | 0 | 1 | Bacteria | Bacteria\_none | Bacillota | Bacilli | Bacillales | Paenibacillaceae | Paenibacillus | Bacteria;Bacteria\_none;Bacillota;Bacilli;Bacillales;Paenibacillaceae;Paenibacillus;Paenibacillus sp. FSL R5-0490 |
| Paenibacillus sp. FSL R5-0517 | 1 | 0 | 0 | 0 | 0 | 0 | 0 | 0 | 0 | 0 | 0 | 0 | 0 | 1 | Bacteria | Bacteria\_none | Bacillota | Bacilli | Bacillales | Paenibacillaceae | Paenibacillus | Bacteria;Bacteria\_none;Bacillota;Bacilli;Bacillales;Paenibacillaceae;Paenibacillus;Paenibacillus sp. FSL R5-0517 |
| Paenibacillus sp. JDR-2 | 0 | 0 | 0 | 0 | 0 | 0 | 2 | 0 | 0 | 0 | 0 | 0 | 0 | 2 | Bacteria | Bacteria\_none | Bacillota | Bacilli | Bacillales | Paenibacillaceae | Paenibacillus | Bacteria;Bacteria\_none;Bacillota;Bacilli;Bacillales;Paenibacillaceae;Paenibacillus;Paenibacillus sp. JDR-2 |
| Paenisporosarcina sp. FSL H8-0542 | 0 | 0 | 0 | 0 | 0 | 0 | 0 | 0 | 0 | 0 | 0 | 0 | 2 | 2 | Bacteria | Bacteria\_none | Bacillota | Bacilli | Bacillales | Planococcaceae | Paenisporosarcina | Bacteria;Bacteria\_none;Bacillota;Bacilli;Bacillales;Planococcaceae;Paenisporosarcina;Paenisporosarcina sp. FSL H8-0542 |
| Sporosarcina ureilytica | 1 | 0 | 0 | 0 | 0 | 0 | 0 | 0 | 0 | 0 | 0 | 0 | 0 | 1 | Bacteria | Bacteria\_none | Bacillota | Bacilli | Bacillales | Planococcaceae | Sporosarcina | Bacteria;Bacteria\_none;Bacillota;Bacilli;Bacillales;Planococcaceae;Sporosarcina;Sporosarcina ureilytica |
| Macrococcus armenti | 1 | 0 | 0 | 0 | 0 | 0 | 0 | 0 | 0 | 0 | 0 | 0 | 0 | 1 | Bacteria | Bacteria\_none | Bacillota | Bacilli | Bacillales | Staphylococcaceae | Macrococcus | Bacteria;Bacteria\_none;Bacillota;Bacilli;Bacillales;Staphylococcaceae;Macrococcus;Macrococcus armenti |
| Staphylococcus aureus | 0 | 0 | 0 | 0 | 0 | 0 | 0 | 0 | 0 | 0 | 2 | 1 | 5 | 8 | Bacteria | Bacteria\_none | Bacillota | Bacilli | Bacillales | Staphylococcaceae | Staphylococcus | Bacteria;Bacteria\_none;Bacillota;Bacilli;Bacillales;Staphylococcaceae;Staphylococcus;Staphylococcus aureus |
| Staphylococcus carnosus | 0 | 0 | 0 | 1 | 0 | 0 | 0 | 0 | 0 | 0 | 0 | 0 | 0 | 1 | Bacteria | Bacteria\_none | Bacillota | Bacilli | Bacillales | Staphylococcaceae | Staphylococcus | Bacteria;Bacteria\_none;Bacillota;Bacilli;Bacillales;Staphylococcaceae;Staphylococcus;Staphylococcus carnosus |
| Staphylococcus cohnii | 0 | 0 | 0 | 0 | 1 | 0 | 0 | 0 | 0 | 0 | 0 | 0 | 0 | 1 | Bacteria | Bacteria\_none | Bacillota | Bacilli | Bacillales | Staphylococcaceae | Staphylococcus | Bacteria;Bacteria\_none;Bacillota;Bacilli;Bacillales;Staphylococcaceae;Staphylococcus;Staphylococcus cohnii |
| Staphylococcus delphini | 0 | 0 | 0 | 0 | 0 | 0 | 0 | 0 | 0 | 0 | 0 | 1 | 0 | 1 | Bacteria | Bacteria\_none | Bacillota | Bacilli | Bacillales | Staphylococcaceae | Staphylococcus | Bacteria;Bacteria\_none;Bacillota;Bacilli;Bacillales;Staphylococcaceae;Staphylococcus;Staphylococcus delphini |
| Staphylococcus devriesei | 0 | 0 | 0 | 0 | 0 | 0 | 0 | 0 | 0 | 0 | 0 | 0 | 1 | 1 | Bacteria | Bacteria\_none | Bacillota | Bacilli | Bacillales | Staphylococcaceae | Staphylococcus | Bacteria;Bacteria\_none;Bacillota;Bacilli;Bacillales;Staphylococcaceae;Staphylococcus;Staphylococcus devriesei |
| Staphylococcus epidermidis | 0 | 3 | 0 | 0 | 0 | 0 | 0 | 0 | 0 | 0 | 0 | 0 | 0 | 3 | Bacteria | Bacteria\_none | Bacillota | Bacilli | Bacillales | Staphylococcaceae | Staphylococcus | Bacteria;Bacteria\_none;Bacillota;Bacilli;Bacillales;Staphylococcaceae;Staphylococcus;Staphylococcus epidermidis |
| Staphylococcus felis | 0 | 0 | 0 | 0 | 1 | 0 | 0 | 0 | 0 | 0 | 0 | 0 | 0 | 1 | Bacteria | Bacteria\_none | Bacillota | Bacilli | Bacillales | Staphylococcaceae | Staphylococcus | Bacteria;Bacteria\_none;Bacillota;Bacilli;Bacillales;Staphylococcaceae;Staphylococcus;Staphylococcus felis |
| Staphylococcus hyicus | 0 | 0 | 0 | 1 | 0 | 0 | 0 | 0 | 0 | 0 | 0 | 0 | 0 | 1 | Bacteria | Bacteria\_none | Bacillota | Bacilli | Bacillales | Staphylococcaceae | Staphylococcus | Bacteria;Bacteria\_none;Bacillota;Bacilli;Bacillales;Staphylococcaceae;Staphylococcus;Staphylococcus hyicus |
| Staphylococcus lloydii | 0 | 0 | 0 | 0 | 0 | 0 | 0 | 0 | 0 | 0 | 1 | 0 | 0 | 1 | Bacteria | Bacteria\_none | Bacillota | Bacilli | Bacillales | Staphylococcaceae | Staphylococcus | Bacteria;Bacteria\_none;Bacillota;Bacilli;Bacillales;Staphylococcaceae;Staphylococcus;Staphylococcus lloydii |
| Staphylococcus sp. 17KM0847 | 0 | 0 | 3 | 0 | 0 | 0 | 0 | 0 | 0 | 0 | 0 | 0 | 0 | 3 | Bacteria | Bacteria\_none | Bacillota | Bacilli | Bacillales | Staphylococcaceae | Staphylococcus | Bacteria;Bacteria\_none;Bacillota;Bacilli;Bacillales;Staphylococcaceae;Staphylococcus;Staphylococcus sp. 17KM0847 |
| Staphylococcus sp. MI 10-1553 | 0 | 0 | 0 | 0 | 0 | 0 | 1 | 0 | 0 | 0 | 0 | 0 | 0 | 1 | Bacteria | Bacteria\_none | Bacillota | Bacilli | Bacillales | Staphylococcaceae | Staphylococcus | Bacteria;Bacteria\_none;Bacillota;Bacilli;Bacillales;Staphylococcaceae;Staphylococcus;Staphylococcus sp. MI 10-1553 |
| Staphylococcus succinus | 0 | 0 | 0 | 0 | 0 | 0 | 0 | 0 | 0 | 1 | 0 | 0 | 0 | 1 | Bacteria | Bacteria\_none | Bacillota | Bacilli | Bacillales | Staphylococcaceae | Staphylococcus | Bacteria;Bacteria\_none;Bacillota;Bacilli;Bacillales;Staphylococcaceae;Staphylococcus;Staphylococcus succinus |
| Staphylococcus xylosus | 0 | 0 | 0 | 1 | 1 | 0 | 0 | 0 | 0 | 0 | 0 | 0 | 0 | 2 | Bacteria | Bacteria\_none | Bacillota | Bacilli | Bacillales | Staphylococcaceae | Staphylococcus | Bacteria;Bacteria\_none;Bacillota;Bacilli;Bacillales;Staphylococcaceae;Staphylococcus;Staphylococcus xylosus |
| Abiotrophia defectiva | 7 | 0 | 62 | 4 | 78 | 19 | 25 | 0 | 0 | 23 | 81 | 5 | 57 | 361 | Bacteria | Bacteria\_none | Bacillota | Bacilli | Lactobacillales | Aerococcaceae | Abiotrophia | Bacteria;Bacteria\_none;Bacillota;Bacilli;Lactobacillales;Aerococcaceae;Abiotrophia;Abiotrophia defectiva |
| Aerococcus urinaeequi | 1 | 0 | 0 | 0 | 0 | 0 | 0 | 0 | 0 | 0 | 0 | 0 | 2 | 3 | Bacteria | Bacteria\_none | Bacillota | Bacilli | Lactobacillales | Aerococcaceae | Aerococcus | Bacteria;Bacteria\_none;Bacillota;Bacilli;Lactobacillales;Aerococcaceae;Aerococcus;Aerococcus urinaeequi |
| Ruoffia tabacinasalis | 0 | 1 | 0 | 0 | 0 | 0 | 0 | 0 | 0 | 0 | 0 | 0 | 1 | 2 | Bacteria | Bacteria\_none | Bacillota | Bacilli | Lactobacillales | Aerococcaceae | Ruoffia | Bacteria;Bacteria\_none;Bacillota;Bacilli;Lactobacillales;Aerococcaceae;Ruoffia;Ruoffia tabacinasalis |
| Dolosigranulum pigrum | 1 | 0 | 0 | 0 | 0 | 0 | 0 | 0 | 0 | 3 | 2 | 0 | 0 | 6 | Bacteria | Bacteria\_none | Bacillota | Bacilli | Lactobacillales | Carnobacteriaceae | Dolosigranulum | Bacteria;Bacteria\_none;Bacillota;Bacilli;Lactobacillales;Carnobacteriaceae;Dolosigranulum;Dolosigranulum pigrum |
| Granulicatella adiacens | 430 | 54 | 423 | 19 | 39 | 124 | 518 | 28 | 0 | 137 | 116 | 27 | 398 | 2313 | Bacteria | Bacteria\_none | Bacillota | Bacilli | Lactobacillales | Carnobacteriaceae | Granulicatella | Bacteria;Bacteria\_none;Bacillota;Bacilli;Lactobacillales;Carnobacteriaceae;Granulicatella;Granulicatella adiacens |
| Granulicatella elegans | 0 | 11 | 3 | 128 | 173 | 6 | 1 | 0 | 0 | 76 | 33 | 3 | 81 | 515 | Bacteria | Bacteria\_none | Bacillota | Bacilli | Lactobacillales | Carnobacteriaceae | Granulicatella | Bacteria;Bacteria\_none;Bacillota;Bacilli;Lactobacillales;Carnobacteriaceae;Granulicatella;Granulicatella elegans |
| Jeotgalibaca dankookensis | 1 | 0 | 0 | 0 | 0 | 0 | 0 | 0 | 0 | 0 | 0 | 0 | 0 | 1 | Bacteria | Bacteria\_none | Bacillota | Bacilli | Lactobacillales | Carnobacteriaceae | Jeotgalibaca | Bacteria;Bacteria\_none;Bacillota;Bacilli;Lactobacillales;Carnobacteriaceae;Jeotgalibaca;Jeotgalibaca dankookensis |
| Jeotgalibaca porci | 0 | 0 | 0 | 0 | 0 | 0 | 0 | 0 | 0 | 0 | 1 | 0 | 0 | 1 | Bacteria | Bacteria\_none | Bacillota | Bacilli | Lactobacillales | Carnobacteriaceae | Jeotgalibaca | Bacteria;Bacteria\_none;Bacillota;Bacilli;Lactobacillales;Carnobacteriaceae;Jeotgalibaca;Jeotgalibaca porci |
| Enterococcus avium | 0 | 0 | 0 | 0 | 0 | 1 | 0 | 0 | 0 | 0 | 1 | 0 | 0 | 2 | Bacteria | Bacteria\_none | Bacillota | Bacilli | Lactobacillales | Enterococcaceae | Enterococcus | Bacteria;Bacteria\_none;Bacillota;Bacilli;Lactobacillales;Enterococcaceae;Enterococcus;Enterococcus avium |
| Enterococcus cecorum | 0 | 0 | 0 | 0 | 0 | 0 | 1 | 0 | 0 | 0 | 1 | 0 | 0 | 2 | Bacteria | Bacteria\_none | Bacillota | Bacilli | Lactobacillales | Enterococcaceae | Enterococcus | Bacteria;Bacteria\_none;Bacillota;Bacilli;Lactobacillales;Enterococcaceae;Enterococcus;Enterococcus cecorum |
| Enterococcus faecalis | 0 | 2 | 0 | 2 | 3 | 1 | 0 | 0 | 0 | 0 | 0 | 0 | 2 | 10 | Bacteria | Bacteria\_none | Bacillota | Bacilli | Lactobacillales | Enterococcaceae | Enterococcus | Bacteria;Bacteria\_none;Bacillota;Bacilli;Lactobacillales;Enterococcaceae;Enterococcus;Enterococcus faecalis |
| Enterococcus faecium | 1 | 7 | 9 | 0 | 0 | 3 | 1 | 3 | 0 | 0 | 2 | 0 | 4 | 30 | Bacteria | Bacteria\_none | Bacillota | Bacilli | Lactobacillales | Enterococcaceae | Enterococcus | Bacteria;Bacteria\_none;Bacillota;Bacilli;Lactobacillales;Enterococcaceae;Enterococcus;Enterococcus faecium |
| Enterococcus gilvus | 1 | 0 | 0 | 0 | 0 | 0 | 0 | 0 | 0 | 0 | 0 | 0 | 0 | 1 | Bacteria | Bacteria\_none | Bacillota | Bacilli | Lactobacillales | Enterococcaceae | Enterococcus | Bacteria;Bacteria\_none;Bacillota;Bacilli;Lactobacillales;Enterococcaceae;Enterococcus;Enterococcus gilvus |
| Enterococcus mundtii | 0 | 0 | 0 | 1 | 0 | 1 | 0 | 0 | 0 | 0 | 0 | 0 | 0 | 2 | Bacteria | Bacteria\_none | Bacillota | Bacilli | Lactobacillales | Enterococcaceae | Enterococcus | Bacteria;Bacteria\_none;Bacillota;Bacilli;Lactobacillales;Enterococcaceae;Enterococcus;Enterococcus mundtii |
| Enterococcus rotai | 0 | 0 | 1 | 0 | 0 | 0 | 0 | 0 | 0 | 0 | 0 | 0 | 0 | 1 | Bacteria | Bacteria\_none | Bacillota | Bacilli | Lactobacillales | Enterococcaceae | Enterococcus | Bacteria;Bacteria\_none;Bacillota;Bacilli;Lactobacillales;Enterococcaceae;Enterococcus;Enterococcus rotai |
| Enterococcus thailandicus | 0 | 0 | 0 | 0 | 0 | 0 | 0 | 0 | 0 | 0 | 1 | 0 | 0 | 1 | Bacteria | Bacteria\_none | Bacillota | Bacilli | Lactobacillales | Enterococcaceae | Enterococcus | Bacteria;Bacteria\_none;Bacillota;Bacilli;Lactobacillales;Enterococcaceae;Enterococcus;Enterococcus thailandicus |
| Tetragenococcus osmophilus | 0 | 0 | 0 | 0 | 0 | 0 | 2 | 0 | 0 | 0 | 0 | 0 | 0 | 2 | Bacteria | Bacteria\_none | Bacillota | Bacilli | Lactobacillales | Enterococcaceae | Tetragenococcus | Bacteria;Bacteria\_none;Bacillota;Bacilli;Lactobacillales;Enterococcaceae;Tetragenococcus;Tetragenococcus osmophilus |
| Vagococcus carniphilus | 1 | 0 | 1 | 0 | 0 | 0 | 1 | 0 | 0 | 0 | 0 | 0 | 1 | 4 | Bacteria | Bacteria\_none | Bacillota | Bacilli | Lactobacillales | Enterococcaceae | Vagococcus | Bacteria;Bacteria\_none;Bacillota;Bacilli;Lactobacillales;Enterococcaceae;Vagococcus;Vagococcus carniphilus |
| Vagococcus zengguangii | 0 | 1 | 0 | 0 | 0 | 0 | 0 | 0 | 0 | 0 | 0 | 0 | 0 | 1 | Bacteria | Bacteria\_none | Bacillota | Bacilli | Lactobacillales | Enterococcaceae | Vagococcus | Bacteria;Bacteria\_none;Bacillota;Bacilli;Lactobacillales;Enterococcaceae;Vagococcus;Vagococcus zengguangii |
| Lacticaseibacillus paracasei | 0 | 0 | 0 | 0 | 0 | 0 | 0 | 0 | 0 | 2 | 0 | 0 | 1 | 3 | Bacteria | Bacteria\_none | Bacillota | Bacilli | Lactobacillales | Lactobacillaceae | Lacticaseibacillus | Bacteria;Bacteria\_none;Bacillota;Bacilli;Lactobacillales;Lactobacillaceae;Lacticaseibacillus;Lacticaseibacillus paracasei |
| Lacticaseibacillus rhamnosus | 0 | 0 | 0 | 0 | 0 | 0 | 10 | 0 | 0 | 0 | 0 | 0 | 5 | 15 | Bacteria | Bacteria\_none | Bacillota | Bacilli | Lactobacillales | Lactobacillaceae | Lacticaseibacillus | Bacteria;Bacteria\_none;Bacillota;Bacilli;Lactobacillales;Lactobacillaceae;Lacticaseibacillus;Lacticaseibacillus rhamnosus |
| Lactobacillus amylolyticus | 1 | 0 | 0 | 0 | 0 | 0 | 0 | 0 | 0 | 0 | 0 | 0 | 0 | 1 | Bacteria | Bacteria\_none | Bacillota | Bacilli | Lactobacillales | Lactobacillaceae | Lactobacillus | Bacteria;Bacteria\_none;Bacillota;Bacilli;Lactobacillales;Lactobacillaceae;Lactobacillus;Lactobacillus amylolyticus |
| Lactobacillus crispatus | 0 | 0 | 0 | 0 | 0 | 0 | 1 | 0 | 0 | 0 | 0 | 0 | 0 | 1 | Bacteria | Bacteria\_none | Bacillota | Bacilli | Lactobacillales | Lactobacillaceae | Lactobacillus | Bacteria;Bacteria\_none;Bacillota;Bacilli;Lactobacillales;Lactobacillaceae;Lactobacillus;Lactobacillus crispatus |
| Lactobacillus gasseri | 0 | 0 | 0 | 0 | 0 | 0 | 9 | 3 | 0 | 1 | 0 | 0 | 0 | 13 | Bacteria | Bacteria\_none | Bacillota | Bacilli | Lactobacillales | Lactobacillaceae | Lactobacillus | Bacteria;Bacteria\_none;Bacillota;Bacilli;Lactobacillales;Lactobacillaceae;Lactobacillus;Lactobacillus gasseri |
| Lactobacillus iners | 0 | 0 | 0 | 1 | 0 | 0 | 0 | 0 | 0 | 0 | 1 | 0 | 0 | 2 | Bacteria | Bacteria\_none | Bacillota | Bacilli | Lactobacillales | Lactobacillaceae | Lactobacillus | Bacteria;Bacteria\_none;Bacillota;Bacilli;Lactobacillales;Lactobacillaceae;Lactobacillus;Lactobacillus iners |
| Lactobacillus johnsonii | 0 | 0 | 0 | 0 | 0 | 0 | 1 | 0 | 0 | 0 | 0 | 0 | 0 | 1 | Bacteria | Bacteria\_none | Bacillota | Bacilli | Lactobacillales | Lactobacillaceae | Lactobacillus | Bacteria;Bacteria\_none;Bacillota;Bacilli;Lactobacillales;Lactobacillaceae;Lactobacillus;Lactobacillus johnsonii |
| Lactobacillus kefiranofaciens | 0 | 0 | 0 | 0 | 0 | 0 | 1 | 0 | 0 | 0 | 0 | 0 | 0 | 1 | Bacteria | Bacteria\_none | Bacillota | Bacilli | Lactobacillales | Lactobacillaceae | Lactobacillus | Bacteria;Bacteria\_none;Bacillota;Bacilli;Lactobacillales;Lactobacillaceae;Lactobacillus;Lactobacillus kefiranofaciens |
| Lactobacillus paragasseri | 0 | 0 | 0 | 0 | 0 | 0 | 1 | 0 | 0 | 0 | 0 | 0 | 0 | 1 | Bacteria | Bacteria\_none | Bacillota | Bacilli | Lactobacillales | Lactobacillaceae | Lactobacillus | Bacteria;Bacteria\_none;Bacillota;Bacilli;Lactobacillales;Lactobacillaceae;Lactobacillus;Lactobacillus paragasseri |
| Lactobacillus ultunensis | 0 | 0 | 0 | 0 | 0 | 0 | 2 | 0 | 0 | 0 | 0 | 0 | 3 | 5 | Bacteria | Bacteria\_none | Bacillota | Bacilli | Lactobacillales | Lactobacillaceae | Lactobacillus | Bacteria;Bacteria\_none;Bacillota;Bacilli;Lactobacillales;Lactobacillaceae;Lactobacillus;Lactobacillus ultunensis |
| Latilactobacillus graminis | 0 | 0 | 0 | 0 | 0 | 0 | 0 | 0 | 0 | 0 | 1 | 0 | 2 | 3 | Bacteria | Bacteria\_none | Bacillota | Bacilli | Lactobacillales | Lactobacillaceae | Latilactobacillus | Bacteria;Bacteria\_none;Bacillota;Bacilli;Lactobacillales;Lactobacillaceae;Latilactobacillus;Latilactobacillus graminis |
| Latilactobacillus sakei | 0 | 0 | 1 | 0 | 0 | 0 | 0 | 0 | 0 | 0 | 0 | 0 | 0 | 1 | Bacteria | Bacteria\_none | Bacillota | Bacilli | Lactobacillales | Lactobacillaceae | Latilactobacillus | Bacteria;Bacteria\_none;Bacillota;Bacilli;Lactobacillales;Lactobacillaceae;Latilactobacillus;Latilactobacillus sakei |
| Lentilactobacillus buchneri | 0 | 0 | 0 | 0 | 0 | 0 | 0 | 0 | 0 | 1 | 0 | 0 | 0 | 1 | Bacteria | Bacteria\_none | Bacillota | Bacilli | Lactobacillales | Lactobacillaceae | Lentilactobacillus | Bacteria;Bacteria\_none;Bacillota;Bacilli;Lactobacillales;Lactobacillaceae;Lentilactobacillus;Lentilactobacillus buchneri |
| Lentilactobacillus kefiri | 0 | 0 | 0 | 0 | 1 | 0 | 0 | 0 | 0 | 0 | 0 | 0 | 0 | 1 | Bacteria | Bacteria\_none | Bacillota | Bacilli | Lactobacillales | Lactobacillaceae | Lentilactobacillus | Bacteria;Bacteria\_none;Bacillota;Bacilli;Lactobacillales;Lactobacillaceae;Lentilactobacillus;Lentilactobacillus kefiri |
| Leuconostoc falkenbergense | 0 | 0 | 0 | 0 | 0 | 1 | 0 | 0 | 0 | 0 | 0 | 0 | 0 | 1 | Bacteria | Bacteria\_none | Bacillota | Bacilli | Lactobacillales | Lactobacillaceae | Leuconostoc | Bacteria;Bacteria\_none;Bacillota;Bacilli;Lactobacillales;Lactobacillaceae;Leuconostoc;Leuconostoc falkenbergense |
| Leuconostoc kimchii | 0 | 0 | 0 | 1 | 0 | 0 | 0 | 0 | 0 | 0 | 0 | 0 | 0 | 1 | Bacteria | Bacteria\_none | Bacillota | Bacilli | Lactobacillales | Lactobacillaceae | Leuconostoc | Bacteria;Bacteria\_none;Bacillota;Bacilli;Lactobacillales;Lactobacillaceae;Leuconostoc;Leuconostoc kimchii |
| Levilactobacillus brevis | 0 | 0 | 1 | 0 | 0 | 0 | 0 | 0 | 0 | 0 | 0 | 0 | 0 | 1 | Bacteria | Bacteria\_none | Bacillota | Bacilli | Lactobacillales | Lactobacillaceae | Levilactobacillus | Bacteria;Bacteria\_none;Bacillota;Bacilli;Lactobacillales;Lactobacillaceae;Levilactobacillus;Levilactobacillus brevis |
| Ligilactobacillus animalis | 0 | 0 | 0 | 0 | 1 | 0 | 0 | 0 | 0 | 0 | 0 | 0 | 0 | 1 | Bacteria | Bacteria\_none | Bacillota | Bacilli | Lactobacillales | Lactobacillaceae | Ligilactobacillus | Bacteria;Bacteria\_none;Bacillota;Bacilli;Lactobacillales;Lactobacillaceae;Ligilactobacillus;Ligilactobacillus animalis |
| Ligilactobacillus aviarius | 0 | 0 | 0 | 0 | 0 | 0 | 0 | 0 | 0 | 0 | 1 | 0 | 0 | 1 | Bacteria | Bacteria\_none | Bacillota | Bacilli | Lactobacillales | Lactobacillaceae | Ligilactobacillus | Bacteria;Bacteria\_none;Bacillota;Bacilli;Lactobacillales;Lactobacillaceae;Ligilactobacillus;Ligilactobacillus aviarius |
| Ligilactobacillus murinus | 0 | 0 | 0 | 0 | 0 | 0 | 1 | 0 | 0 | 0 | 0 | 0 | 0 | 1 | Bacteria | Bacteria\_none | Bacillota | Bacilli | Lactobacillales | Lactobacillaceae | Ligilactobacillus | Bacteria;Bacteria\_none;Bacillota;Bacilli;Lactobacillales;Lactobacillaceae;Ligilactobacillus;Ligilactobacillus murinus |
| Ligilactobacillus ruminis | 0 | 0 | 0 | 0 | 0 | 0 | 0 | 0 | 2 | 0 | 0 | 0 | 0 | 2 | Bacteria | Bacteria\_none | Bacillota | Bacilli | Lactobacillales | Lactobacillaceae | Ligilactobacillus | Bacteria;Bacteria\_none;Bacillota;Bacilli;Lactobacillales;Lactobacillaceae;Ligilactobacillus;Ligilactobacillus ruminis |
| Ligilactobacillus saerimneri | 0 | 0 | 0 | 0 | 0 | 0 | 0 | 1 | 0 | 0 | 0 | 0 | 0 | 1 | Bacteria | Bacteria\_none | Bacillota | Bacilli | Lactobacillales | Lactobacillaceae | Ligilactobacillus | Bacteria;Bacteria\_none;Bacillota;Bacilli;Lactobacillales;Lactobacillaceae;Ligilactobacillus;Ligilactobacillus saerimneri |
| Ligilactobacillus salivarius | 1 | 0 | 0 | 0 | 0 | 0 | 0 | 0 | 0 | 0 | 0 | 0 | 1 | 2 | Bacteria | Bacteria\_none | Bacillota | Bacilli | Lactobacillales | Lactobacillaceae | Ligilactobacillus | Bacteria;Bacteria\_none;Bacillota;Bacilli;Lactobacillales;Lactobacillaceae;Ligilactobacillus;Ligilactobacillus salivarius |
| Limosilactobacillus portuensis | 0 | 0 | 0 | 0 | 0 | 0 | 0 | 0 | 0 | 0 | 0 | 0 | 1 | 1 | Bacteria | Bacteria\_none | Bacillota | Bacilli | Lactobacillales | Lactobacillaceae | Limosilactobacillus | Bacteria;Bacteria\_none;Bacillota;Bacilli;Lactobacillales;Lactobacillaceae;Limosilactobacillus;Limosilactobacillus portuensis |
| Limosilactobacillus reuteri | 0 | 0 | 0 | 0 | 0 | 0 | 0 | 0 | 0 | 0 | 0 | 0 | 2 | 2 | Bacteria | Bacteria\_none | Bacillota | Bacilli | Lactobacillales | Lactobacillaceae | Limosilactobacillus | Bacteria;Bacteria\_none;Bacillota;Bacilli;Lactobacillales;Lactobacillaceae;Limosilactobacillus;Limosilactobacillus reuteri |
| Limosilactobacillus vaginalis | 0 | 0 | 0 | 0 | 0 | 0 | 0 | 0 | 0 | 0 | 0 | 0 | 2 | 2 | Bacteria | Bacteria\_none | Bacillota | Bacilli | Lactobacillales | Lactobacillaceae | Limosilactobacillus | Bacteria;Bacteria\_none;Bacillota;Bacilli;Lactobacillales;Lactobacillaceae;Limosilactobacillus;Limosilactobacillus vaginalis |
| Liquorilactobacillus hordei | 0 | 0 | 0 | 0 | 0 | 0 | 0 | 0 | 0 | 0 | 1 | 0 | 0 | 1 | Bacteria | Bacteria\_none | Bacillota | Bacilli | Lactobacillales | Lactobacillaceae | Liquorilactobacillus | Bacteria;Bacteria\_none;Bacillota;Bacilli;Lactobacillales;Lactobacillaceae;Liquorilactobacillus;Liquorilactobacillus hordei |
| Liquorilactobacillus nagelii | 0 | 0 | 0 | 0 | 0 | 0 | 0 | 1 | 0 | 0 | 0 | 0 | 1 | 2 | Bacteria | Bacteria\_none | Bacillota | Bacilli | Lactobacillales | Lactobacillaceae | Liquorilactobacillus | Bacteria;Bacteria\_none;Bacillota;Bacilli;Lactobacillales;Lactobacillaceae;Liquorilactobacillus;Liquorilactobacillus nagelii |
| Nicoliella spurrieriana | 0 | 0 | 0 | 0 | 1 | 0 | 0 | 0 | 0 | 0 | 0 | 0 | 0 | 1 | Bacteria | Bacteria\_none | Bacillota | Bacilli | Lactobacillales | Lactobacillaceae | Nicoliella | Bacteria;Bacteria\_none;Bacillota;Bacilli;Lactobacillales;Lactobacillaceae;Nicoliella;Nicoliella spurrieriana |
| Pediococcus acidilactici | 0 | 0 | 1 | 0 | 0 | 0 | 0 | 0 | 0 | 0 | 0 | 0 | 0 | 1 | Bacteria | Bacteria\_none | Bacillota | Bacilli | Lactobacillales | Lactobacillaceae | Pediococcus | Bacteria;Bacteria\_none;Bacillota;Bacilli;Lactobacillales;Lactobacillaceae;Pediococcus;Pediococcus acidilactici |
| Weissella confusa | 0 | 0 | 0 | 1 | 0 | 0 | 0 | 0 | 0 | 0 | 0 | 0 | 0 | 1 | Bacteria | Bacteria\_none | Bacillota | Bacilli | Lactobacillales | Lactobacillaceae | Weissella | Bacteria;Bacteria\_none;Bacillota;Bacilli;Lactobacillales;Lactobacillaceae;Weissella;Weissella confusa |
| Weissella jogaejeotgali | 0 | 0 | 0 | 0 | 0 | 0 | 1 | 0 | 0 | 0 | 0 | 0 | 0 | 1 | Bacteria | Bacteria\_none | Bacillota | Bacilli | Lactobacillales | Lactobacillaceae | Weissella | Bacteria;Bacteria\_none;Bacillota;Bacilli;Lactobacillales;Lactobacillaceae;Weissella;Weissella jogaejeotgali |
| Lactococcus cremoris | 0 | 0 | 0 | 0 | 0 | 3 | 0 | 1 | 0 | 0 | 0 | 0 | 4 | 8 | Bacteria | Bacteria\_none | Bacillota | Bacilli | Lactobacillales | Streptococcaceae | Lactococcus | Bacteria;Bacteria\_none;Bacillota;Bacilli;Lactobacillales;Streptococcaceae;Lactococcus;Lactococcus cremoris |
| Lactococcus garvieae | 0 | 0 | 0 | 0 | 0 | 0 | 0 | 0 | 0 | 1 | 0 | 0 | 0 | 1 | Bacteria | Bacteria\_none | Bacillota | Bacilli | Lactobacillales | Streptococcaceae | Lactococcus | Bacteria;Bacteria\_none;Bacillota;Bacilli;Lactobacillales;Streptococcaceae;Lactococcus;Lactococcus garvieae |
| Lactococcus lactis | 0 | 1 | 0 | 0 | 0 | 2 | 0 | 0 | 0 | 0 | 0 | 0 | 5 | 8 | Bacteria | Bacteria\_none | Bacillota | Bacilli | Lactobacillales | Streptococcaceae | Lactococcus | Bacteria;Bacteria\_none;Bacillota;Bacilli;Lactobacillales;Streptococcaceae;Lactococcus;Lactococcus lactis |
| Lactococcus paracarnosus | 0 | 0 | 0 | 0 | 0 | 0 | 0 | 0 | 0 | 1 | 0 | 0 | 0 | 1 | Bacteria | Bacteria\_none | Bacillota | Bacilli | Lactobacillales | Streptococcaceae | Lactococcus | Bacteria;Bacteria\_none;Bacillota;Bacilli;Lactobacillales;Streptococcaceae;Lactococcus;Lactococcus paracarnosus |
| Lactococcus protaetiae | 0 | 0 | 0 | 0 | 0 | 0 | 1 | 0 | 0 | 0 | 0 | 0 | 0 | 1 | Bacteria | Bacteria\_none | Bacillota | Bacilli | Lactobacillales | Streptococcaceae | Lactococcus | Bacteria;Bacteria\_none;Bacillota;Bacilli;Lactobacillales;Streptococcaceae;Lactococcus;Lactococcus protaetiae |
| Streptococcus acidominimus | 0 | 1 | 0 | 0 | 0 | 1 | 0 | 0 | 0 | 0 | 0 | 0 | 1 | 3 | Bacteria | Bacteria\_none | Bacillota | Bacilli | Lactobacillales | Streptococcaceae | Streptococcus | Bacteria;Bacteria\_none;Bacillota;Bacilli;Lactobacillales;Streptococcaceae;Streptococcus;Streptococcus acidominimus |
| Streptococcus agalactiae | 4 | 9 | 7 | 0 | 19 | 3 | 7 | 2 | 3 | 3 | 0 | 8 | 35 | 100 | Bacteria | Bacteria\_none | Bacillota | Bacilli | Lactobacillales | Streptococcaceae | Streptococcus | Bacteria;Bacteria\_none;Bacillota;Bacilli;Lactobacillales;Streptococcaceae;Streptococcus;Streptococcus agalactiae |
| Streptococcus alactolyticus | 0 | 0 | 0 | 1 | 0 | 0 | 1 | 0 | 0 | 0 | 0 | 0 | 0 | 2 | Bacteria | Bacteria\_none | Bacillota | Bacilli | Lactobacillales | Streptococcaceae | Streptococcus | Bacteria;Bacteria\_none;Bacillota;Bacilli;Lactobacillales;Streptococcaceae;Streptococcus;Streptococcus alactolyticus |
| Streptococcus anginosus | 5 | 15 | 18 | 1 | 19 | 19 | 3 | 59 | 0 | 7 | 36 | 23 | 50 | 255 | Bacteria | Bacteria\_none | Bacillota | Bacilli | Lactobacillales | Streptococcaceae | Streptococcus | Bacteria;Bacteria\_none;Bacillota;Bacilli;Lactobacillales;Streptococcaceae;Streptococcus;Streptococcus anginosus |
| Streptococcus australis | 21 | 21 | 2 | 7 | 23 | 63 | 18 | 6 | 4 | 108 | 25 | 15 | 100 | 413 | Bacteria | Bacteria\_none | Bacillota | Bacilli | Lactobacillales | Streptococcaceae | Streptococcus | Bacteria;Bacteria\_none;Bacillota;Bacilli;Lactobacillales;Streptococcaceae;Streptococcus;Streptococcus australis |
| Streptococcus canis | 0 | 1 | 0 | 0 | 0 | 0 | 1 | 0 | 0 | 0 | 0 | 0 | 1 | 3 | Bacteria | Bacteria\_none | Bacillota | Bacilli | Lactobacillales | Streptococcaceae | Streptococcus | Bacteria;Bacteria\_none;Bacillota;Bacilli;Lactobacillales;Streptococcaceae;Streptococcus;Streptococcus canis |
| Streptococcus chenjunshii | 0 | 0 | 0 | 0 | 0 | 0 | 0 | 0 | 0 | 0 | 0 | 0 | 1 | 1 | Bacteria | Bacteria\_none | Bacillota | Bacilli | Lactobacillales | Streptococcaceae | Streptococcus | Bacteria;Bacteria\_none;Bacillota;Bacilli;Lactobacillales;Streptococcaceae;Streptococcus;Streptococcus chenjunshii |
| Streptococcus constellatus | 0 | 5 | 9 | 0 | 2 | 9 | 1 | 35 | 0 | 1 | 93 | 3 | 74 | 232 | Bacteria | Bacteria\_none | Bacillota | Bacilli | Lactobacillales | Streptococcaceae | Streptococcus | Bacteria;Bacteria\_none;Bacillota;Bacilli;Lactobacillales;Streptococcaceae;Streptococcus;Streptococcus constellatus |
| Streptococcus cristatus | 2 | 27 | 24 | 11 | 28 | 54 | 9 | 0 | 0 | 89 | 10 | 57 | 53 | 364 | Bacteria | Bacteria\_none | Bacillota | Bacilli | Lactobacillales | Streptococcaceae | Streptococcus | Bacteria;Bacteria\_none;Bacillota;Bacilli;Lactobacillales;Streptococcaceae;Streptococcus;Streptococcus cristatus |
| Streptococcus didelphis | 0 | 0 | 1 | 0 | 0 | 0 | 0 | 0 | 0 | 1 | 0 | 2 | 2 | 6 | Bacteria | Bacteria\_none | Bacillota | Bacilli | Lactobacillales | Streptococcaceae | Streptococcus | Bacteria;Bacteria\_none;Bacillota;Bacilli;Lactobacillales;Streptococcaceae;Streptococcus;Streptococcus didelphis |
| Streptococcus dysgalactiae | 1 | 2 | 1 | 0 | 0 | 4 | 5 | 1 | 12 | 0 | 4 | 0 | 0 | 30 | Bacteria | Bacteria\_none | Bacillota | Bacilli | Lactobacillales | Streptococcaceae | Streptococcus | Bacteria;Bacteria\_none;Bacillota;Bacilli;Lactobacillales;Streptococcaceae;Streptococcus;Streptococcus dysgalactiae |
| Streptococcus equi | 0 | 2 | 1 | 0 | 3 | 3 | 2 | 3 | 0 | 0 | 4 | 1 | 1 | 20 | Bacteria | Bacteria\_none | Bacillota | Bacilli | Lactobacillales | Streptococcaceae | Streptococcus | Bacteria;Bacteria\_none;Bacillota;Bacilli;Lactobacillales;Streptococcaceae;Streptococcus;Streptococcus equi |
| Streptococcus equinus | 0 | 0 | 0 | 0 | 1 | 0 | 0 | 1 | 0 | 0 | 1 | 0 | 3 | 6 | Bacteria | Bacteria\_none | Bacillota | Bacilli | Lactobacillales | Streptococcaceae | Streptococcus | Bacteria;Bacteria\_none;Bacillota;Bacilli;Lactobacillales;Streptococcaceae;Streptococcus;Streptococcus equinus |
| Streptococcus ferus | 0 | 0 | 1 | 0 | 1 | 0 | 0 | 0 | 0 | 0 | 0 | 0 | 0 | 2 | Bacteria | Bacteria\_none | Bacillota | Bacilli | Lactobacillales | Streptococcaceae | Streptococcus | Bacteria;Bacteria\_none;Bacillota;Bacilli;Lactobacillales;Streptococcaceae;Streptococcus;Streptococcus ferus |
| Streptococcus gallolyticus | 0 | 0 | 0 | 0 | 0 | 0 | 0 | 0 | 0 | 0 | 0 | 0 | 3 | 3 | Bacteria | Bacteria\_none | Bacillota | Bacilli | Lactobacillales | Streptococcaceae | Streptococcus | Bacteria;Bacteria\_none;Bacillota;Bacilli;Lactobacillales;Streptococcaceae;Streptococcus;Streptococcus gallolyticus |
| Streptococcus gordonii | 25 | 12 | 5 | 2 | 12 | 19 | 172 | 3 | 6 | 32 | 100 | 58 | 142 | 588 | Bacteria | Bacteria\_none | Bacillota | Bacilli | Lactobacillales | Streptococcaceae | Streptococcus | Bacteria;Bacteria\_none;Bacillota;Bacilli;Lactobacillales;Streptococcaceae;Streptococcus;Streptococcus gordonii |
| Streptococcus gwangjuense | 2 | 12 | 1 | 12 | 47 | 10 | 9 | 0 | 0 | 25 | 25 | 8 | 39 | 190 | Bacteria | Bacteria\_none | Bacillota | Bacilli | Lactobacillales | Streptococcaceae | Streptococcus | Bacteria;Bacteria\_none;Bacillota;Bacilli;Lactobacillales;Streptococcaceae;Streptococcus;Streptococcus gwangjuense |
| Streptococcus halichoeri | 0 | 0 | 0 | 0 | 0 | 1 | 0 | 0 | 0 | 1 | 0 | 0 | 1 | 3 | Bacteria | Bacteria\_none | Bacillota | Bacilli | Lactobacillales | Streptococcaceae | Streptococcus | Bacteria;Bacteria\_none;Bacillota;Bacilli;Lactobacillales;Streptococcaceae;Streptococcus;Streptococcus halichoeri |
| Streptococcus halotolerans | 0 | 0 | 0 | 0 | 0 | 1 | 0 | 0 | 0 | 0 | 0 | 0 | 1 | 2 | Bacteria | Bacteria\_none | Bacillota | Bacilli | Lactobacillales | Streptococcaceae | Streptococcus | Bacteria;Bacteria\_none;Bacillota;Bacilli;Lactobacillales;Streptococcaceae;Streptococcus;Streptococcus halotolerans |
| Streptococcus himalayensis | 1 | 0 | 0 | 0 | 0 | 0 | 0 | 1 | 0 | 0 | 1 | 0 | 1 | 4 | Bacteria | Bacteria\_none | Bacillota | Bacilli | Lactobacillales | Streptococcaceae | Streptococcus | Bacteria;Bacteria\_none;Bacillota;Bacilli;Lactobacillales;Streptococcaceae;Streptococcus;Streptococcus himalayensis |
| Streptococcus ilei | 7 | 15 | 1 | 0 | 2 | 34 | 4 | 0 | 5 | 14 | 10 | 1 | 57 | 150 | Bacteria | Bacteria\_none | Bacillota | Bacilli | Lactobacillales | Streptococcaceae | Streptococcus | Bacteria;Bacteria\_none;Bacillota;Bacilli;Lactobacillales;Streptococcaceae;Streptococcus;Streptococcus ilei |
| Streptococcus infantarius | 0 | 1 | 0 | 0 | 0 | 0 | 0 | 0 | 0 | 0 | 0 | 0 | 0 | 1 | Bacteria | Bacteria\_none | Bacillota | Bacilli | Lactobacillales | Streptococcaceae | Streptococcus | Bacteria;Bacteria\_none;Bacillota;Bacilli;Lactobacillales;Streptococcaceae;Streptococcus;Streptococcus infantarius |
| Streptococcus infantis | 40 | 11 | 34 | 28 | 10 | 26 | 7 | 2 | 4 | 2 | 59 | 5 | 61 | 289 | Bacteria | Bacteria\_none | Bacillota | Bacilli | Lactobacillales | Streptococcaceae | Streptococcus | Bacteria;Bacteria\_none;Bacillota;Bacilli;Lactobacillales;Streptococcaceae;Streptococcus;Streptococcus infantis |
| Streptococcus iniae | 0 | 1 | 0 | 0 | 1 | 0 | 1 | 0 | 0 | 0 | 1 | 0 | 0 | 4 | Bacteria | Bacteria\_none | Bacillota | Bacilli | Lactobacillales | Streptococcaceae | Streptococcus | Bacteria;Bacteria\_none;Bacillota;Bacilli;Lactobacillales;Streptococcaceae;Streptococcus;Streptococcus iniae |
| Streptococcus intermedius | 1 | 5 | 4 | 0 | 5 | 3 | 1 | 7 | 0 | 3 | 223 | 3 | 79 | 334 | Bacteria | Bacteria\_none | Bacillota | Bacilli | Lactobacillales | Streptococcaceae | Streptococcus | Bacteria;Bacteria\_none;Bacillota;Bacilli;Lactobacillales;Streptococcaceae;Streptococcus;Streptococcus intermedius |
| Streptococcus koreensis | 7 | 4 | 3 | 0 | 1 | 16 | 6 | 0 | 6 | 30 | 1 | 0 | 20 | 94 | Bacteria | Bacteria\_none | Bacillota | Bacilli | Lactobacillales | Streptococcaceae | Streptococcus | Bacteria;Bacteria\_none;Bacillota;Bacilli;Lactobacillales;Streptococcaceae;Streptococcus;Streptococcus koreensis |
| Streptococcus lactarius | 10 | 5 | 1 | 0 | 3 | 7 | 11 | 0 | 13 | 5 | 0 | 9 | 19 | 83 | Bacteria | Bacteria\_none | Bacillota | Bacilli | Lactobacillales | Streptococcaceae | Streptococcus | Bacteria;Bacteria\_none;Bacillota;Bacilli;Lactobacillales;Streptococcaceae;Streptococcus;Streptococcus lactarius |
| Streptococcus lutetiensis | 0 | 0 | 0 | 1 | 0 | 0 | 0 | 0 | 0 | 0 | 0 | 0 | 1 | 2 | Bacteria | Bacteria\_none | Bacillota | Bacilli | Lactobacillales | Streptococcaceae | Streptococcus | Bacteria;Bacteria\_none;Bacillota;Bacilli;Lactobacillales;Streptococcaceae;Streptococcus;Streptococcus lutetiensis |
| Streptococcus merionis | 0 | 1 | 1 | 0 | 0 | 0 | 1 | 0 | 1 | 0 | 2 | 0 | 0 | 6 | Bacteria | Bacteria\_none | Bacillota | Bacilli | Lactobacillales | Streptococcaceae | Streptococcus | Bacteria;Bacteria\_none;Bacillota;Bacilli;Lactobacillales;Streptococcaceae;Streptococcus;Streptococcus merionis |
| Streptococcus milleri | 0 | 0 | 0 | 0 | 3 | 0 | 2 | 4 | 0 | 0 | 24 | 3 | 21 | 57 | Bacteria | Bacteria\_none | Bacillota | Bacilli | Lactobacillales | Streptococcaceae | Streptococcus | Bacteria;Bacteria\_none;Bacillota;Bacilli;Lactobacillales;Streptococcaceae;Streptococcus;Streptococcus milleri |
| Streptococcus mitis | 61 | 146 | 26 | 111 | 920 | 162 | 85 | 19 | 12 | 215 | 604 | 88 | 506 | 2955 | Bacteria | Bacteria\_none | Bacillota | Bacilli | Lactobacillales | Streptococcaceae | Streptococcus | Bacteria;Bacteria\_none;Bacillota;Bacilli;Lactobacillales;Streptococcaceae;Streptococcus;Streptococcus mitis |
| Streptococcus mutans | 0 | 1 | 1 | 0 | 2 | 1 | 0 | 0 | 0 | 1 | 2 | 0 | 3 | 11 | Bacteria | Bacteria\_none | Bacillota | Bacilli | Lactobacillales | Streptococcaceae | Streptococcus | Bacteria;Bacteria\_none;Bacillota;Bacilli;Lactobacillales;Streptococcaceae;Streptococcus;Streptococcus mutans |
| Streptococcus oralis | 610 | 193 | 155 | 32 | 240 | 185 | 862 | 103 | 2 | 66 | 418 | 262 | 902 | 4030 | Bacteria | Bacteria\_none | Bacillota | Bacilli | Lactobacillales | Streptococcaceae | Streptococcus | Bacteria;Bacteria\_none;Bacillota;Bacilli;Lactobacillales;Streptococcaceae;Streptococcus;Streptococcus oralis |
| Streptococcus oriscaviae | 1 | 0 | 0 | 0 | 0 | 0 | 1 | 0 | 0 | 0 | 3 | 0 | 0 | 5 | Bacteria | Bacteria\_none | Bacillota | Bacilli | Lactobacillales | Streptococcaceae | Streptococcus | Bacteria;Bacteria\_none;Bacillota;Bacilli;Lactobacillales;Streptococcaceae;Streptococcus;Streptococcus oriscaviae |
| Streptococcus pantholopis | 0 | 0 | 1 | 0 | 0 | 0 | 0 | 0 | 0 | 1 | 2 | 1 | 0 | 5 | Bacteria | Bacteria\_none | Bacillota | Bacilli | Lactobacillales | Streptococcaceae | Streptococcus | Bacteria;Bacteria\_none;Bacillota;Bacilli;Lactobacillales;Streptococcaceae;Streptococcus;Streptococcus pantholopis |
| Streptococcus parapneumoniae | 7 | 14 | 0 | 14 | 98 | 9 | 9 | 1 | 0 | 35 | 16 | 13 | 43 | 259 | Bacteria | Bacteria\_none | Bacillota | Bacilli | Lactobacillales | Streptococcaceae | Streptococcus | Bacteria;Bacteria\_none;Bacillota;Bacilli;Lactobacillales;Streptococcaceae;Streptococcus;Streptococcus parapneumoniae |
| Streptococcus parasanguinis | 221 | 233 | 144 | 0 | 5 | 173 | 314 | 16 | 262 | 36 | 9 | 169 | 464 | 2046 | Bacteria | Bacteria\_none | Bacillota | Bacilli | Lactobacillales | Streptococcaceae | Streptococcus | Bacteria;Bacteria\_none;Bacillota;Bacilli;Lactobacillales;Streptococcaceae;Streptococcus;Streptococcus parasanguinis |
| Streptococcus parasuis | 1 | 2 | 0 | 1 | 0 | 1 | 0 | 0 | 0 | 2 | 0 | 2 | 3 | 12 | Bacteria | Bacteria\_none | Bacillota | Bacilli | Lactobacillales | Streptococcaceae | Streptococcus | Bacteria;Bacteria\_none;Bacillota;Bacilli;Lactobacillales;Streptococcaceae;Streptococcus;Streptococcus parasuis |
| Streptococcus parauberis | 0 | 0 | 0 | 0 | 2 | 1 | 0 | 0 | 0 | 1 | 0 | 0 | 0 | 4 | Bacteria | Bacteria\_none | Bacillota | Bacilli | Lactobacillales | Streptococcaceae | Streptococcus | Bacteria;Bacteria\_none;Bacillota;Bacilli;Lactobacillales;Streptococcaceae;Streptococcus;Streptococcus parauberis |
| Streptococcus pasteurianus | 2 | 0 | 0 | 0 | 0 | 0 | 0 | 0 | 2 | 2 | 0 | 0 | 10 | 16 | Bacteria | Bacteria\_none | Bacillota | Bacilli | Lactobacillales | Streptococcaceae | Streptococcus | Bacteria;Bacteria\_none;Bacillota;Bacilli;Lactobacillales;Streptococcaceae;Streptococcus;Streptococcus pasteurianus |
| Streptococcus periodonticum | 1 | 0 | 0 | 1 | 1 | 0 | 1 | 1 | 0 | 0 | 3 | 1 | 5 | 14 | Bacteria | Bacteria\_none | Bacillota | Bacilli | Lactobacillales | Streptococcaceae | Streptococcus | Bacteria;Bacteria\_none;Bacillota;Bacilli;Lactobacillales;Streptococcaceae;Streptococcus;Streptococcus periodonticum |
| Streptococcus pluranimalium | 0 | 1 | 0 | 1 | 0 | 0 | 0 | 0 | 0 | 0 | 0 | 1 | 3 | 6 | Bacteria | Bacteria\_none | Bacillota | Bacilli | Lactobacillales | Streptococcaceae | Streptococcus | Bacteria;Bacteria\_none;Bacillota;Bacilli;Lactobacillales;Streptococcaceae;Streptococcus;Streptococcus pluranimalium |
| Streptococcus pneumoniae | 69 | 69 | 11 | 108 | 371 | 54 | 34 | 4 | 3 | 137 | 158 | 55 | 272 | 1345 | Bacteria | Bacteria\_none | Bacillota | Bacilli | Lactobacillales | Streptococcaceae | Streptococcus | Bacteria;Bacteria\_none;Bacillota;Bacilli;Lactobacillales;Streptococcaceae;Streptococcus;Streptococcus pneumoniae |
| Streptococcus porcinus | 0 | 0 | 0 | 2 | 1 | 2 | 0 | 0 | 0 | 0 | 1 | 0 | 3 | 9 | Bacteria | Bacteria\_none | Bacillota | Bacilli | Lactobacillales | Streptococcaceae | Streptococcus | Bacteria;Bacteria\_none;Bacillota;Bacilli;Lactobacillales;Streptococcaceae;Streptococcus;Streptococcus porcinus |
| Streptococcus pseudopneumoniae | 4 | 6 | 1 | 11 | 68 | 10 | 4 | 1 | 1 | 21 | 19 | 7 | 29 | 182 | Bacteria | Bacteria\_none | Bacillota | Bacilli | Lactobacillales | Streptococcaceae | Streptococcus | Bacteria;Bacteria\_none;Bacillota;Bacilli;Lactobacillales;Streptococcaceae;Streptococcus;Streptococcus pseudopneumoniae |
| Streptococcus pseudoporcinus | 0 | 1 | 0 | 1 | 0 | 1 | 0 | 1 | 0 | 0 | 1 | 0 | 4 | 9 | Bacteria | Bacteria\_none | Bacillota | Bacilli | Lactobacillales | Streptococcaceae | Streptococcus | Bacteria;Bacteria\_none;Bacillota;Bacilli;Lactobacillales;Streptococcaceae;Streptococcus;Streptococcus pseudoporcinus |
| Streptococcus pyogenes | 0 | 2 | 1 | 2 | 1 | 4 | 1 | 0 | 1 | 1 | 8 | 1 | 79 | 101 | Bacteria | Bacteria\_none | Bacillota | Bacilli | Lactobacillales | Streptococcaceae | Streptococcus | Bacteria;Bacteria\_none;Bacillota;Bacilli;Lactobacillales;Streptococcaceae;Streptococcus;Streptococcus pyogenes |
| Streptococcus ratti | 0 | 0 | 1 | 0 | 0 | 0 | 1 | 0 | 0 | 0 | 0 | 0 | 0 | 2 | Bacteria | Bacteria\_none | Bacillota | Bacilli | Lactobacillales | Streptococcaceae | Streptococcus | Bacteria;Bacteria\_none;Bacillota;Bacilli;Lactobacillales;Streptococcaceae;Streptococcus;Streptococcus ratti |
| Streptococcus respiraculi | 0 | 1 | 0 | 0 | 0 | 0 | 0 | 0 | 0 | 0 | 3 | 0 | 0 | 4 | Bacteria | Bacteria\_none | Bacillota | Bacilli | Lactobacillales | Streptococcaceae | Streptococcus | Bacteria;Bacteria\_none;Bacillota;Bacilli;Lactobacillales;Streptococcaceae;Streptococcus;Streptococcus respiraculi |
| Streptococcus rubneri | 6 | 2 | 1 | 0 | 3 | 23 | 4 | 0 | 3 | 5 | 2 | 2 | 11 | 62 | Bacteria | Bacteria\_none | Bacillota | Bacilli | Lactobacillales | Streptococcaceae | Streptococcus | Bacteria;Bacteria\_none;Bacillota;Bacilli;Lactobacillales;Streptococcaceae;Streptococcus;Streptococcus rubneri |
| Streptococcus ruminantium | 0 | 0 | 0 | 0 | 0 | 0 | 0 | 0 | 0 | 0 | 1 | 0 | 0 | 1 | Bacteria | Bacteria\_none | Bacillota | Bacilli | Lactobacillales | Streptococcaceae | Streptococcus | Bacteria;Bacteria\_none;Bacillota;Bacilli;Lactobacillales;Streptococcaceae;Streptococcus;Streptococcus ruminantium |
| Streptococcus salivarius | 7 | 17 | 2 | 0 | 8 | 43 | 48 | 2 | 2 | 9 | 5 | 21 | 42 | 206 | Bacteria | Bacteria\_none | Bacillota | Bacilli | Lactobacillales | Streptococcaceae | Streptococcus | Bacteria;Bacteria\_none;Bacillota;Bacilli;Lactobacillales;Streptococcaceae;Streptococcus;Streptococcus salivarius |
| Streptococcus sanguinis | 80 | 20 | 67 | 4 | 121 | 86 | 139 | 38 | 2 | 20 | 408 | 8 | 299 | 1292 | Bacteria | Bacteria\_none | Bacillota | Bacilli | Lactobacillales | Streptococcaceae | Streptococcus | Bacteria;Bacteria\_none;Bacillota;Bacilli;Lactobacillales;Streptococcaceae;Streptococcus;Streptococcus sanguinis |
| Streptococcus sp. 116-D4 | 3 | 18 | 1 | 5 | 61 | 15 | 4 | 0 | 0 | 14 | 52 | 28 | 47 | 248 | Bacteria | Bacteria\_none | Bacillota | Bacilli | Lactobacillales | Streptococcaceae | Streptococcus | Bacteria;Bacteria\_none;Bacillota;Bacilli;Lactobacillales;Streptococcaceae;Streptococcus;Streptococcus sp. 116-D4 |
| Streptococcus sp. 1643 | 37 | 2 | 6 | 5 | 7 | 5 | 42 | 0 | 1 | 4 | 14 | 4 | 61 | 188 | Bacteria | Bacteria\_none | Bacillota | Bacilli | Lactobacillales | Streptococcaceae | Streptococcus | Bacteria;Bacteria\_none;Bacillota;Bacilli;Lactobacillales;Streptococcaceae;Streptococcus;Streptococcus sp. 1643 |
| Streptococcus sp. 29887 | 0 | 0 | 0 | 0 | 0 | 0 | 0 | 0 | 0 | 0 | 1 | 0 | 0 | 1 | Bacteria | Bacteria\_none | Bacillota | Bacilli | Lactobacillales | Streptococcaceae | Streptococcus | Bacteria;Bacteria\_none;Bacillota;Bacilli;Lactobacillales;Streptococcaceae;Streptococcus;Streptococcus sp. 29887 |
| Streptococcus sp. 29892 | 0 | 0 | 0 | 0 | 0 | 0 | 0 | 0 | 0 | 0 | 1 | 1 | 0 | 2 | Bacteria | Bacteria\_none | Bacillota | Bacilli | Lactobacillales | Streptococcaceae | Streptococcus | Bacteria;Bacteria\_none;Bacillota;Bacilli;Lactobacillales;Streptococcaceae;Streptococcus;Streptococcus sp. 29892 |
| Streptococcus sp. 29896 | 0 | 0 | 0 | 0 | 0 | 0 | 0 | 0 | 0 | 0 | 1 | 0 | 0 | 1 | Bacteria | Bacteria\_none | Bacillota | Bacilli | Lactobacillales | Streptococcaceae | Streptococcus | Bacteria;Bacteria\_none;Bacillota;Bacilli;Lactobacillales;Streptococcaceae;Streptococcus;Streptococcus sp. 29896 |
| Streptococcus sp. A12 | 23 | 7 | 1 | 0 | 14 | 76 | 6 | 0 | 6 | 53 | 20 | 1 | 57 | 264 | Bacteria | Bacteria\_none | Bacillota | Bacilli | Lactobacillales | Streptococcaceae | Streptococcus | Bacteria;Bacteria\_none;Bacillota;Bacilli;Lactobacillales;Streptococcaceae;Streptococcus;Streptococcus sp. A12 |
| Streptococcus sp. D7B5 | 9 | 3 | 36 | 0 | 9 | 15 | 21 | 1 | 0 | 0 | 8 | 6 | 47 | 155 | Bacteria | Bacteria\_none | Bacillota | Bacilli | Lactobacillales | Streptococcaceae | Streptococcus | Bacteria;Bacteria\_none;Bacillota;Bacilli;Lactobacillales;Streptococcaceae;Streptococcus;Streptococcus sp. D7B5 |
| Streptococcus sp. DTU\_2020\_1000888\_1\_SI\_GRL\_NUU\_041A | 8 | 1 | 5 | 0 | 4 | 5 | 5 | 1 | 0 | 0 | 15 | 0 | 21 | 65 | Bacteria | Bacteria\_none | Bacillota | Bacilli | Lactobacillales | Streptococcaceae | Streptococcus | Bacteria;Bacteria\_none;Bacillota;Bacilli;Lactobacillales;Streptococcaceae;Streptococcus;Streptococcus sp. DTU\_2020\_1000888\_1\_SI\_GRL\_NUU\_041A |
| Streptococcus sp. DTU\_2020\_1001019\_1\_SI\_AUS\_MUR\_006 | 85 | 51 | 12 | 4 | 42 | 96 | 47 | 12 | 3 | 14 | 46 | 5 | 130 | 547 | Bacteria | Bacteria\_none | Bacillota | Bacilli | Lactobacillales | Streptococcaceae | Streptococcus | Bacteria;Bacteria\_none;Bacillota;Bacilli;Lactobacillales;Streptococcaceae;Streptococcus;Streptococcus sp. DTU\_2020\_1001019\_1\_SI\_AUS\_MUR\_006 |
| Streptococcus sp. FDAARGOS\_192 | 0 | 0 | 0 | 0 | 1 | 0 | 0 | 0 | 0 | 0 | 0 | 0 | 5 | 6 | Bacteria | Bacteria\_none | Bacillota | Bacilli | Lactobacillales | Streptococcaceae | Streptococcus | Bacteria;Bacteria\_none;Bacillota;Bacilli;Lactobacillales;Streptococcaceae;Streptococcus;Streptococcus sp. FDAARGOS\_192 |
| Streptococcus sp. FDAARGOS\_522 | 0 | 0 | 0 | 0 | 0 | 0 | 11 | 0 | 25 | 0 | 0 | 0 | 0 | 36 | Bacteria | Bacteria\_none | Bacillota | Bacilli | Lactobacillales | Streptococcaceae | Streptococcus | Bacteria;Bacteria\_none;Bacillota;Bacilli;Lactobacillales;Streptococcaceae;Streptococcus;Streptococcus sp. FDAARGOS\_522 |
| Streptococcus sp. FSL K6-1323 | 0 | 2 | 0 | 0 | 0 | 2 | 0 | 0 | 0 | 0 | 0 | 0 | 0 | 4 | Bacteria | Bacteria\_none | Bacillota | Bacilli | Lactobacillales | Streptococcaceae | Streptococcus | Bacteria;Bacteria\_none;Bacillota;Bacilli;Lactobacillales;Streptococcaceae;Streptococcus;Streptococcus sp. FSL K6-1323 |
| Streptococcus sp. FSL R7-0248 | 0 | 0 | 0 | 0 | 0 | 0 | 9 | 0 | 0 | 0 | 0 | 0 | 2 | 11 | Bacteria | Bacteria\_none | Bacillota | Bacilli | Lactobacillales | Streptococcaceae | Streptococcus | Bacteria;Bacteria\_none;Bacillota;Bacilli;Lactobacillales;Streptococcaceae;Streptococcus;Streptococcus sp. FSL R7-0248 |
| Streptococcus sp. FSL W7-1342 | 0 | 0 | 0 | 0 | 0 | 10 | 0 | 0 | 0 | 0 | 0 | 0 | 6 | 16 | Bacteria | Bacteria\_none | Bacillota | Bacilli | Lactobacillales | Streptococcaceae | Streptococcus | Bacteria;Bacteria\_none;Bacillota;Bacilli;Lactobacillales;Streptococcaceae;Streptococcus;Streptococcus sp. FSL W7-1342 |
| Streptococcus sp. FSL W8-0197 | 25 | 36 | 9 | 0 | 3 | 9 | 18 | 36 | 0 | 0 | 16 | 1 | 75 | 228 | Bacteria | Bacteria\_none | Bacillota | Bacilli | Lactobacillales | Streptococcaceae | Streptococcus | Bacteria;Bacteria\_none;Bacillota;Bacilli;Lactobacillales;Streptococcaceae;Streptococcus;Streptococcus sp. FSL W8-0197 |
| Streptococcus sp. KS 6 | 0 | 0 | 0 | 0 | 0 | 0 | 1 | 0 | 0 | 1 | 0 | 1 | 0 | 3 | Bacteria | Bacteria\_none | Bacillota | Bacilli | Lactobacillales | Streptococcaceae | Streptococcus | Bacteria;Bacteria\_none;Bacillota;Bacilli;Lactobacillales;Streptococcaceae;Streptococcus;Streptococcus sp. KS 6 |
| Streptococcus sp. LPB0220 | 46 | 48 | 31 | 0 | 4 | 26 | 45 | 0 | 72 | 10 | 2 | 21 | 73 | 378 | Bacteria | Bacteria\_none | Bacillota | Bacilli | Lactobacillales | Streptococcaceae | Streptococcus | Bacteria;Bacteria\_none;Bacillota;Bacilli;Lactobacillales;Streptococcaceae;Streptococcus;Streptococcus sp. LPB0220 |
| Streptococcus sp. Marseille-Q3533 | 65 | 50 | 8 | 5 | 59 | 101 | 21 | 4 | 0 | 2 | 50 | 11 | 85 | 461 | Bacteria | Bacteria\_none | Bacillota | Bacilli | Lactobacillales | Streptococcaceae | Streptococcus | Bacteria;Bacteria\_none;Bacillota;Bacilli;Lactobacillales;Streptococcaceae;Streptococcus;Streptococcus sp. Marseille-Q3533 |
| Streptococcus sp. Marseille-Q6470 | 77 | 80 | 12 | 14 | 23 | 56 | 13 | 1 | 4 | 1 | 74 | 6 | 76 | 437 | Bacteria | Bacteria\_none | Bacillota | Bacilli | Lactobacillales | Streptococcaceae | Streptococcus | Bacteria;Bacteria\_none;Bacillota;Bacilli;Lactobacillales;Streptococcaceae;Streptococcus;Streptococcus sp. Marseille-Q6470 |
| Streptococcus sp. NCTC 11567 | 0 | 0 | 0 | 0 | 6 | 0 | 0 | 0 | 0 | 0 | 0 | 0 | 0 | 6 | Bacteria | Bacteria\_none | Bacillota | Bacilli | Lactobacillales | Streptococcaceae | Streptococcus | Bacteria;Bacteria\_none;Bacillota;Bacilli;Lactobacillales;Streptococcaceae;Streptococcus;Streptococcus sp. NCTC 11567 |
| Streptococcus sp. NPS 308 | 11 | 2 | 6 | 2 | 47 | 1 | 15 | 12 | 1 | 2 | 29 | 12 | 53 | 193 | Bacteria | Bacteria\_none | Bacillota | Bacilli | Lactobacillales | Streptococcaceae | Streptococcus | Bacteria;Bacteria\_none;Bacillota;Bacilli;Lactobacillales;Streptococcaceae;Streptococcus;Streptococcus sp. NPS 308 |
| Streptococcus sp. NSJ-72 | 2 | 4 | 0 | 0 | 4 | 0 | 1 | 18 | 0 | 0 | 17 | 6 | 29 | 81 | Bacteria | Bacteria\_none | Bacillota | Bacilli | Lactobacillales | Streptococcaceae | Streptococcus | Bacteria;Bacteria\_none;Bacillota;Bacilli;Lactobacillales;Streptococcaceae;Streptococcus;Streptococcus sp. NSJ-72 |
| Streptococcus sp. S1 | 21 | 19 | 13 | 0 | 2 | 23 | 34 | 2 | 44 | 6 | 4 | 20 | 42 | 230 | Bacteria | Bacteria\_none | Bacillota | Bacilli | Lactobacillales | Streptococcaceae | Streptococcus | Bacteria;Bacteria\_none;Bacillota;Bacilli;Lactobacillales;Streptococcaceae;Streptococcus;Streptococcus sp. S1 |
| Streptococcus sp. S5 | 45 | 52 | 35 | 2 | 0 | 20 | 39 | 0 | 77 | 8 | 1 | 30 | 93 | 402 | Bacteria | Bacteria\_none | Bacillota | Bacilli | Lactobacillales | Streptococcaceae | Streptococcus | Bacteria;Bacteria\_none;Bacillota;Bacilli;Lactobacillales;Streptococcaceae;Streptococcus;Streptococcus sp. S5 |
| Streptococcus sp. oral taxon 061 | 56 | 44 | 16 | 11 | 10 | 74 | 15 | 1 | 3 | 0 | 57 | 5 | 86 | 378 | Bacteria | Bacteria\_none | Bacillota | Bacilli | Lactobacillales | Streptococcaceae | Streptococcus | Bacteria;Bacteria\_none;Bacillota;Bacilli;Lactobacillales;Streptococcaceae;Streptococcus;Streptococcus sp. oral taxon 061 |
| Streptococcus sp. oral taxon 064 | 35 | 9 | 0 | 3 | 0 | 6 | 79 | 1 | 0 | 0 | 6 | 0 | 93 | 232 | Bacteria | Bacteria\_none | Bacillota | Bacilli | Lactobacillales | Streptococcaceae | Streptococcus | Bacteria;Bacteria\_none;Bacillota;Bacilli;Lactobacillales;Streptococcaceae;Streptococcus;Streptococcus sp. oral taxon 064 |
| Streptococcus sp. oral taxon 431 | 95 | 69 | 4 | 2 | 57 | 171 | 34 | 10 | 0 | 9 | 60 | 4 | 125 | 640 | Bacteria | Bacteria\_none | Bacillota | Bacilli | Lactobacillales | Streptococcaceae | Streptococcus | Bacteria;Bacteria\_none;Bacillota;Bacilli;Lactobacillales;Streptococcaceae;Streptococcus;Streptococcus sp. oral taxon 431 |
| Streptococcus sp. zg-86 | 0 | 0 | 0 | 0 | 0 | 0 | 0 | 0 | 0 | 0 | 1 | 1 | 0 | 2 | Bacteria | Bacteria\_none | Bacillota | Bacilli | Lactobacillales | Streptococcaceae | Streptococcus | Bacteria;Bacteria\_none;Bacillota;Bacilli;Lactobacillales;Streptococcaceae;Streptococcus;Streptococcus sp. zg-86 |
| Streptococcus suis | 4 | 1 | 3 | 0 | 6 | 4 | 7 | 1 | 0 | 17 | 5 | 1 | 9 | 58 | Bacteria | Bacteria\_none | Bacillota | Bacilli | Lactobacillales | Streptococcaceae | Streptococcus | Bacteria;Bacteria\_none;Bacillota;Bacilli;Lactobacillales;Streptococcaceae;Streptococcus;Streptococcus suis |
| Streptococcus thermophilus | 3 | 2 | 3 | 2 | 3 | 17 | 3 | 1 | 0 | 4 | 6 | 1 | 6 | 51 | Bacteria | Bacteria\_none | Bacillota | Bacilli | Lactobacillales | Streptococcaceae | Streptococcus | Bacteria;Bacteria\_none;Bacillota;Bacilli;Lactobacillales;Streptococcaceae;Streptococcus;Streptococcus thermophilus |
| Streptococcus toyakuensis | 1 | 12 | 0 | 12 | 82 | 10 | 9 | 3 | 2 | 14 | 59 | 15 | 65 | 284 | Bacteria | Bacteria\_none | Bacillota | Bacilli | Lactobacillales | Streptococcaceae | Streptococcus | Bacteria;Bacteria\_none;Bacillota;Bacilli;Lactobacillales;Streptococcaceae;Streptococcus;Streptococcus toyakuensis |
| Streptococcus troglodytae | 0 | 0 | 0 | 0 | 1 | 0 | 0 | 0 | 0 | 0 | 0 | 1 | 0 | 2 | Bacteria | Bacteria\_none | Bacillota | Bacilli | Lactobacillales | Streptococcaceae | Streptococcus | Bacteria;Bacteria\_none;Bacillota;Bacilli;Lactobacillales;Streptococcaceae;Streptococcus;Streptococcus troglodytae |
| Streptococcus uberis | 0 | 0 | 0 | 0 | 0 | 0 | 1 | 0 | 0 | 0 | 0 | 1 | 0 | 2 | Bacteria | Bacteria\_none | Bacillota | Bacilli | Lactobacillales | Streptococcaceae | Streptococcus | Bacteria;Bacteria\_none;Bacillota;Bacilli;Lactobacillales;Streptococcaceae;Streptococcus;Streptococcus uberis |
| Streptococcus urinalis | 0 | 0 | 0 | 0 | 0 | 0 | 0 | 0 | 0 | 0 | 0 | 1 | 0 | 1 | Bacteria | Bacteria\_none | Bacillota | Bacilli | Lactobacillales | Streptococcaceae | Streptococcus | Bacteria;Bacteria\_none;Bacillota;Bacilli;Lactobacillales;Streptococcaceae;Streptococcus;Streptococcus urinalis |
| Streptococcus vestibularis | 0 | 0 | 0 | 1 | 3 | 2 | 0 | 0 | 1 | 0 | 0 | 0 | 3 | 10 | Bacteria | Bacteria\_none | Bacillota | Bacilli | Lactobacillales | Streptococcaceae | Streptococcus | Bacteria;Bacteria\_none;Bacillota;Bacilli;Lactobacillales;Streptococcaceae;Streptococcus;Streptococcus vestibularis |
| Caldicellulosiruptor morganii | 0 | 0 | 0 | 0 | 1 | 0 | 0 | 0 | 0 | 0 | 0 | 0 | 0 | 1 | Bacteria | Bacteria\_none | Bacillota | Bacillota\_Incertae\_sedis | Caldicellulosiruptorales | Caldicellulosiruptoraceae | Caldicellulosiruptor | Bacteria;Bacteria\_none;Bacillota;Bacillota\_Incertae\_sedis;Caldicellulosiruptorales;Caldicellulosiruptoraceae;Caldicellulosiruptor;Caldicellulosiruptor morganii |
| Aristaeella lactis | 0 | 0 | 0 | 0 | 0 | 0 | 0 | 1 | 0 | 0 | 0 | 0 | 0 | 1 | Bacteria | Bacteria\_none | Bacillota | Clostridia | Eubacteriales | Aristaeellaceae | Aristaeella | Bacteria;Bacteria\_none;Bacillota;Clostridia;Eubacteriales;Aristaeellaceae;Aristaeella;Aristaeella lactis |
| Clostridium aceticum | 0 | 0 | 0 | 1 | 0 | 0 | 0 | 0 | 0 | 0 | 0 | 0 | 0 | 1 | Bacteria | Bacteria\_none | Bacillota | Clostridia | Eubacteriales | Clostridiaceae | Clostridium | Bacteria;Bacteria\_none;Bacillota;Clostridia;Eubacteriales;Clostridiaceae;Clostridium;Clostridium aceticum |
| Clostridium acetobutylicum | 1 | 0 | 0 | 0 | 0 | 0 | 0 | 0 | 0 | 0 | 0 | 0 | 0 | 1 | Bacteria | Bacteria\_none | Bacillota | Clostridia | Eubacteriales | Clostridiaceae | Clostridium | Bacteria;Bacteria\_none;Bacillota;Clostridia;Eubacteriales;Clostridiaceae;Clostridium;Clostridium acetobutylicum |
| Clostridium argentinense | 0 | 0 | 0 | 0 | 0 | 1 | 0 | 0 | 0 | 0 | 0 | 0 | 0 | 1 | Bacteria | Bacteria\_none | Bacillota | Clostridia | Eubacteriales | Clostridiaceae | Clostridium | Bacteria;Bacteria\_none;Bacillota;Clostridia;Eubacteriales;Clostridiaceae;Clostridium;Clostridium argentinense |
| Clostridium baratii | 0 | 0 | 0 | 0 | 1 | 0 | 0 | 0 | 0 | 0 | 0 | 0 | 1 | 2 | Bacteria | Bacteria\_none | Bacillota | Clostridia | Eubacteriales | Clostridiaceae | Clostridium | Bacteria;Bacteria\_none;Bacillota;Clostridia;Eubacteriales;Clostridiaceae;Clostridium;Clostridium baratii |
| Clostridium beijerinckii | 0 | 0 | 0 | 0 | 0 | 0 | 0 | 0 | 0 | 0 | 0 | 1 | 4 | 5 | Bacteria | Bacteria\_none | Bacillota | Clostridia | Eubacteriales | Clostridiaceae | Clostridium | Bacteria;Bacteria\_none;Bacillota;Clostridia;Eubacteriales;Clostridiaceae;Clostridium;Clostridium beijerinckii |
| Clostridium botulinum | 0 | 0 | 1 | 1 | 1 | 1 | 0 | 0 | 0 | 0 | 1 | 0 | 0 | 5 | Bacteria | Bacteria\_none | Bacillota | Clostridia | Eubacteriales | Clostridiaceae | Clostridium | Bacteria;Bacteria\_none;Bacillota;Clostridia;Eubacteriales;Clostridiaceae;Clostridium;Clostridium botulinum |
| Clostridium butyricum | 0 | 0 | 0 | 0 | 0 | 4 | 0 | 0 | 0 | 0 | 0 | 0 | 0 | 4 | Bacteria | Bacteria\_none | Bacillota | Clostridia | Eubacteriales | Clostridiaceae | Clostridium | Bacteria;Bacteria\_none;Bacillota;Clostridia;Eubacteriales;Clostridiaceae;Clostridium;Clostridium butyricum |
| Clostridium cadaveris | 0 | 0 | 0 | 0 | 0 | 0 | 0 | 0 | 0 | 0 | 1 | 0 | 0 | 1 | Bacteria | Bacteria\_none | Bacillota | Clostridia | Eubacteriales | Clostridiaceae | Clostridium | Bacteria;Bacteria\_none;Bacillota;Clostridia;Eubacteriales;Clostridiaceae;Clostridium;Clostridium cadaveris |
| Clostridium chauvoei | 0 | 0 | 0 | 0 | 0 | 0 | 0 | 0 | 0 | 0 | 0 | 0 | 1 | 1 | Bacteria | Bacteria\_none | Bacillota | Clostridia | Eubacteriales | Clostridiaceae | Clostridium | Bacteria;Bacteria\_none;Bacillota;Clostridia;Eubacteriales;Clostridiaceae;Clostridium;Clostridium chauvoei |
| Clostridium estertheticum | 0 | 0 | 0 | 0 | 0 | 0 | 0 | 0 | 1 | 0 | 0 | 0 | 1 | 2 | Bacteria | Bacteria\_none | Bacillota | Clostridia | Eubacteriales | Clostridiaceae | Clostridium | Bacteria;Bacteria\_none;Bacillota;Clostridia;Eubacteriales;Clostridiaceae;Clostridium;Clostridium estertheticum |
| Clostridium formicaceticum | 0 | 2 | 0 | 0 | 0 | 0 | 0 | 0 | 0 | 0 | 0 | 0 | 0 | 2 | Bacteria | Bacteria\_none | Bacillota | Clostridia | Eubacteriales | Clostridiaceae | Clostridium | Bacteria;Bacteria\_none;Bacillota;Clostridia;Eubacteriales;Clostridiaceae;Clostridium;Clostridium formicaceticum |
| Clostridium gasigenes | 0 | 0 | 0 | 1 | 0 | 0 | 0 | 0 | 0 | 0 | 0 | 0 | 0 | 1 | Bacteria | Bacteria\_none | Bacillota | Clostridia | Eubacteriales | Clostridiaceae | Clostridium | Bacteria;Bacteria\_none;Bacillota;Clostridia;Eubacteriales;Clostridiaceae;Clostridium;Clostridium gasigenes |
| Clostridium intestinale | 0 | 0 | 0 | 0 | 0 | 0 | 0 | 0 | 0 | 0 | 0 | 0 | 1 | 1 | Bacteria | Bacteria\_none | Bacillota | Clostridia | Eubacteriales | Clostridiaceae | Clostridium | Bacteria;Bacteria\_none;Bacillota;Clostridia;Eubacteriales;Clostridiaceae;Clostridium;Clostridium intestinale |
| Clostridium isatidis | 0 | 0 | 0 | 0 | 0 | 0 | 0 | 0 | 0 | 0 | 0 | 0 | 1 | 1 | Bacteria | Bacteria\_none | Bacillota | Clostridia | Eubacteriales | Clostridiaceae | Clostridium | Bacteria;Bacteria\_none;Bacillota;Clostridia;Eubacteriales;Clostridiaceae;Clostridium;Clostridium isatidis |
| Clostridium kluyveri | 0 | 0 | 1 | 0 | 0 | 0 | 0 | 0 | 0 | 0 | 0 | 0 | 0 | 1 | Bacteria | Bacteria\_none | Bacillota | Clostridia | Eubacteriales | Clostridiaceae | Clostridium | Bacteria;Bacteria\_none;Bacillota;Clostridia;Eubacteriales;Clostridiaceae;Clostridium;Clostridium kluyveri |
| Clostridium pasteurianum | 0 | 0 | 0 | 0 | 0 | 0 | 0 | 0 | 0 | 0 | 0 | 0 | 1 | 1 | Bacteria | Bacteria\_none | Bacillota | Clostridia | Eubacteriales | Clostridiaceae | Clostridium | Bacteria;Bacteria\_none;Bacillota;Clostridia;Eubacteriales;Clostridiaceae;Clostridium;Clostridium pasteurianum |
| Clostridium perfringens | 0 | 0 | 0 | 1 | 0 | 0 | 0 | 0 | 1 | 0 | 0 | 0 | 0 | 2 | Bacteria | Bacteria\_none | Bacillota | Clostridia | Eubacteriales | Clostridiaceae | Clostridium | Bacteria;Bacteria\_none;Bacillota;Clostridia;Eubacteriales;Clostridiaceae;Clostridium;Clostridium perfringens |
| Clostridium saccharobutylicum | 0 | 0 | 0 | 1 | 0 | 0 | 0 | 0 | 0 | 0 | 0 | 0 | 0 | 1 | Bacteria | Bacteria\_none | Bacillota | Clostridia | Eubacteriales | Clostridiaceae | Clostridium | Bacteria;Bacteria\_none;Bacillota;Clostridia;Eubacteriales;Clostridiaceae;Clostridium;Clostridium saccharobutylicum |
| Clostridium septicum | 0 | 0 | 0 | 0 | 0 | 0 | 0 | 0 | 0 | 0 | 1 | 0 | 1 | 2 | Bacteria | Bacteria\_none | Bacillota | Clostridia | Eubacteriales | Clostridiaceae | Clostridium | Bacteria;Bacteria\_none;Bacillota;Clostridia;Eubacteriales;Clostridiaceae;Clostridium;Clostridium septicum |
| Clostridium sp. C1 | 0 | 0 | 0 | 0 | 0 | 0 | 0 | 0 | 0 | 0 | 0 | 0 | 2 | 2 | Bacteria | Bacteria\_none | Bacillota | Clostridia | Eubacteriales | Clostridiaceae | Clostridium | Bacteria;Bacteria\_none;Bacillota;Clostridia;Eubacteriales;Clostridiaceae;Clostridium;Clostridium sp. C1 |
| Clostridium sp. DL-VIII | 0 | 0 | 0 | 0 | 0 | 0 | 0 | 0 | 0 | 0 | 0 | 0 | 1 | 1 | Bacteria | Bacteria\_none | Bacillota | Clostridia | Eubacteriales | Clostridiaceae | Clostridium | Bacteria;Bacteria\_none;Bacillota;Clostridia;Eubacteriales;Clostridiaceae;Clostridium;Clostridium sp. DL-VIII |
| Clostridium sp. JN-9 | 0 | 0 | 0 | 0 | 0 | 0 | 0 | 0 | 0 | 0 | 0 | 0 | 1 | 1 | Bacteria | Bacteria\_none | Bacillota | Clostridia | Eubacteriales | Clostridiaceae | Clostridium | Bacteria;Bacteria\_none;Bacillota;Clostridia;Eubacteriales;Clostridiaceae;Clostridium;Clostridium sp. JN-9 |
| Clostridium sp. MB40-C1 | 0 | 0 | 1 | 0 | 1 | 0 | 0 | 0 | 1 | 0 | 0 | 0 | 0 | 3 | Bacteria | Bacteria\_none | Bacillota | Clostridia | Eubacteriales | Clostridiaceae | Clostridium | Bacteria;Bacteria\_none;Bacillota;Clostridia;Eubacteriales;Clostridiaceae;Clostridium;Clostridium sp. MB40-C1 |
| Clostridium sp. MD294 | 0 | 0 | 0 | 0 | 0 | 1 | 0 | 0 | 0 | 0 | 0 | 0 | 0 | 1 | Bacteria | Bacteria\_none | Bacillota | Clostridia | Eubacteriales | Clostridiaceae | Clostridium | Bacteria;Bacteria\_none;Bacillota;Clostridia;Eubacteriales;Clostridiaceae;Clostridium;Clostridium sp. MD294 |
| Clostridium tyrobutyricum | 0 | 0 | 0 | 0 | 1 | 0 | 0 | 0 | 0 | 0 | 0 | 0 | 0 | 1 | Bacteria | Bacteria\_none | Bacillota | Clostridia | Eubacteriales | Clostridiaceae | Clostridium | Bacteria;Bacteria\_none;Bacillota;Clostridia;Eubacteriales;Clostridiaceae;Clostridium;Clostridium tyrobutyricum |
| Crassaminicella profunda | 0 | 0 | 0 | 0 | 0 | 3 | 0 | 0 | 0 | 0 | 0 | 0 | 0 | 3 | Bacteria | Bacteria\_none | Bacillota | Clostridia | Eubacteriales | Clostridiaceae | Crassaminicella | Bacteria;Bacteria\_none;Bacillota;Clostridia;Eubacteriales;Clostridiaceae;Crassaminicella;Crassaminicella profunda |
| Crassaminicella thermophila | 0 | 0 | 0 | 0 | 0 | 0 | 0 | 0 | 0 | 0 | 0 | 0 | 1 | 1 | Bacteria | Bacteria\_none | Bacillota | Clostridia | Eubacteriales | Clostridiaceae | Crassaminicella | Bacteria;Bacteria\_none;Bacillota;Clostridia;Eubacteriales;Clostridiaceae;Crassaminicella;Crassaminicella thermophila |
| Oceanirhabdus sp. W0125-5 | 0 | 0 | 0 | 0 | 0 | 1 | 0 | 0 | 0 | 0 | 0 | 0 | 0 | 1 | Bacteria | Bacteria\_none | Bacillota | Clostridia | Eubacteriales | Clostridiaceae | Oceanirhabdus | Bacteria;Bacteria\_none;Bacillota;Clostridia;Eubacteriales;Clostridiaceae;Oceanirhabdus;Oceanirhabdus sp. W0125-5 |
| Sarcina sp. JB2 | 0 | 0 | 0 | 0 | 0 | 1 | 0 | 1 | 1 | 0 | 0 | 0 | 0 | 3 | Bacteria | Bacteria\_none | Bacillota | Clostridia | Eubacteriales | Clostridiaceae | Sarcina | Bacteria;Bacteria\_none;Bacillota;Clostridia;Eubacteriales;Clostridiaceae;Sarcina;Sarcina sp. JB2 |
| Carboxydocella thermautotrophica | 0 | 0 | 0 | 0 | 0 | 0 | 0 | 0 | 0 | 0 | 0 | 0 | 1 | 1 | Bacteria | Bacteria\_none | Bacillota | Clostridia | Eubacteriales | Clostridiales Family XVI. Incertae Sedis | Carboxydocella | Bacteria;Bacteria\_none;Bacillota;Clostridia;Eubacteriales;Clostridiales Family XVI. Incertae Sedis;Carboxydocella;Carboxydocella thermautotrophica |
| Dehalobacter restrictus | 0 | 0 | 0 | 0 | 0 | 0 | 0 | 0 | 0 | 0 | 0 | 0 | 1 | 1 | Bacteria | Bacteria\_none | Bacillota | Clostridia | Eubacteriales | Desulfitobacteriaceae | Dehalobacter | Bacteria;Bacteria\_none;Bacillota;Clostridia;Eubacteriales;Desulfitobacteriaceae;Dehalobacter;Dehalobacter restrictus |
| Desulfosporosinus meridiei | 0 | 0 | 0 | 0 | 0 | 0 | 0 | 0 | 1 | 0 | 0 | 0 | 0 | 1 | Bacteria | Bacteria\_none | Bacillota | Clostridia | Eubacteriales | Desulfitobacteriaceae | Desulfosporosinus | Bacteria;Bacteria\_none;Bacillota;Clostridia;Eubacteriales;Desulfitobacteriaceae;Desulfosporosinus;Desulfosporosinus meridiei |
| Desulfosporosinus orientis | 0 | 0 | 0 | 0 | 0 | 1 | 0 | 0 | 0 | 0 | 0 | 0 | 0 | 1 | Bacteria | Bacteria\_none | Bacillota | Clostridia | Eubacteriales | Desulfitobacteriaceae | Desulfosporosinus | Bacteria;Bacteria\_none;Bacillota;Clostridia;Eubacteriales;Desulfitobacteriaceae;Desulfosporosinus;Desulfosporosinus orientis |
| Acetobacterium woodii | 0 | 0 | 1 | 0 | 0 | 0 | 0 | 0 | 0 | 0 | 0 | 0 | 0 | 1 | Bacteria | Bacteria\_none | Bacillota | Clostridia | Eubacteriales | Eubacteriaceae | Acetobacterium | Bacteria;Bacteria\_none;Bacillota;Clostridia;Eubacteriales;Eubacteriaceae;Acetobacterium;Acetobacterium woodii |
| Acetobacterium\_uncultured | 0 | 0 | 0 | 0 | 0 | 0 | 1 | 0 | 0 | 0 | 0 | 0 | 0 | 1 | Bacteria | Bacteria\_none | Bacillota | Clostridia | Eubacteriales | Eubacteriaceae | Acetobacterium | Bacteria;Bacteria\_none;Bacillota;Clostridia;Eubacteriales;Eubacteriaceae;Acetobacterium;Acetobacterium\_uncultured |
| Eubacterium sp. MSJ-33 | 0 | 0 | 0 | 0 | 0 | 1 | 0 | 0 | 0 | 0 | 0 | 0 | 0 | 1 | Bacteria | Bacteria\_none | Bacillota | Clostridia | Eubacteriales | Eubacteriaceae | Eubacterium | Bacteria;Bacteria\_none;Bacillota;Clostridia;Eubacteriales;Eubacteriaceae;Eubacterium;Eubacterium sp. MSJ-33 |
| Eubacterium ventriosum | 0 | 0 | 0 | 0 | 0 | 0 | 0 | 0 | 0 | 0 | 0 | 0 | 1 | 1 | Bacteria | Bacteria\_none | Bacillota | Clostridia | Eubacteriales | Eubacteriaceae | Eubacterium | Bacteria;Bacteria\_none;Bacillota;Clostridia;Eubacteriales;Eubacteriaceae;Eubacterium;Eubacterium ventriosum |
| Sinanaerobacter sp. ZZT-01 | 0 | 0 | 0 | 0 | 0 | 0 | 0 | 0 | 0 | 0 | 0 | 1 | 1 | 2 | Bacteria | Bacteria\_none | Bacillota | Clostridia | Eubacteriales | Eubacteriales Family XIII. Incertae Sedis | Sinanaerobacter | Bacteria;Bacteria\_none;Bacillota;Clostridia;Eubacteriales;Eubacteriales Family XIII. Incertae Sedis;Sinanaerobacter;Sinanaerobacter sp. ZZT-01 |
| Fenollaria massiliensis | 0 | 0 | 0 | 0 | 0 | 0 | 0 | 0 | 0 | 0 | 2 | 0 | 0 | 2 | Bacteria | Bacteria\_none | Bacillota | Clostridia | Eubacteriales | Eubacteriales\_Incertae\_sedis | Fenollaria | Bacteria;Bacteria\_none;Bacillota;Clostridia;Eubacteriales;Eubacteriales\_Incertae\_sedis;Fenollaria;Fenollaria massiliensis |
| Massilistercora timonensis | 0 | 0 | 0 | 0 | 0 | 2 | 0 | 0 | 0 | 0 | 0 | 0 | 0 | 2 | Bacteria | Bacteria\_none | Bacillota | Clostridia | Eubacteriales | Eubacteriales\_Incertae\_sedis | Massilistercora | Bacteria;Bacteria\_none;Bacillota;Clostridia;Eubacteriales;Eubacteriales\_Incertae\_sedis;Massilistercora;Massilistercora timonensis |
| Acetivibrio clariflavus | 0 | 0 | 0 | 0 | 0 | 0 | 0 | 0 | 0 | 1 | 1 | 0 | 0 | 2 | Bacteria | Bacteria\_none | Bacillota | Clostridia | Eubacteriales | Oscillospiraceae | Acetivibrio | Bacteria;Bacteria\_none;Bacillota;Clostridia;Eubacteriales;Oscillospiraceae;Acetivibrio;Acetivibrio clariflavus |
| Amygdalobacter indicium | 0 | 0 | 1 | 0 | 1 | 1 | 0 | 0 | 0 | 0 | 0 | 0 | 0 | 3 | Bacteria | Bacteria\_none | Bacillota | Clostridia | Eubacteriales | Oscillospiraceae | Amygdalobacter | Bacteria;Bacteria\_none;Bacillota;Clostridia;Eubacteriales;Oscillospiraceae;Amygdalobacter;Amygdalobacter indicium |
| Caproicibacterium amylolyticum | 0 | 0 | 0 | 0 | 0 | 1 | 0 | 0 | 0 | 0 | 0 | 0 | 0 | 1 | Bacteria | Bacteria\_none | Bacillota | Clostridia | Eubacteriales | Oscillospiraceae | Caproicibacterium | Bacteria;Bacteria\_none;Bacillota;Clostridia;Eubacteriales;Oscillospiraceae;Caproicibacterium;Caproicibacterium amylolyticum |
| Caproicibacterium sp. BJN0003 | 1 | 0 | 0 | 0 | 0 | 1 | 5 | 0 | 0 | 0 | 0 | 0 | 1 | 8 | Bacteria | Bacteria\_none | Bacillota | Clostridia | Eubacteriales | Oscillospiraceae | Caproicibacterium | Bacteria;Bacteria\_none;Bacillota;Clostridia;Eubacteriales;Oscillospiraceae;Caproicibacterium;Caproicibacterium sp. BJN0003 |
| Ethanoligenens harbinense | 0 | 0 | 0 | 0 | 0 | 0 | 0 | 0 | 0 | 0 | 0 | 0 | 1 | 1 | Bacteria | Bacteria\_none | Bacillota | Clostridia | Eubacteriales | Oscillospiraceae | Ethanoligenens | Bacteria;Bacteria\_none;Bacillota;Clostridia;Eubacteriales;Oscillospiraceae;Ethanoligenens;Ethanoligenens harbinense |
| Faecalibacterium prausnitzii | 0 | 1 | 0 | 0 | 0 | 0 | 0 | 0 | 0 | 0 | 0 | 0 | 0 | 1 | Bacteria | Bacteria\_none | Bacillota | Clostridia | Eubacteriales | Oscillospiraceae | Faecalibacterium | Bacteria;Bacteria\_none;Bacillota;Clostridia;Eubacteriales;Oscillospiraceae;Faecalibacterium;Faecalibacterium prausnitzii |
| Faecalibacterium sp. I4-1-79 | 0 | 0 | 1 | 0 | 0 | 0 | 0 | 0 | 0 | 0 | 0 | 0 | 0 | 1 | Bacteria | Bacteria\_none | Bacillota | Clostridia | Eubacteriales | Oscillospiraceae | Faecalibacterium | Bacteria;Bacteria\_none;Bacillota;Clostridia;Eubacteriales;Oscillospiraceae;Faecalibacterium;Faecalibacterium sp. I4-1-79 |
| Fastidiosipila sanguinis | 2 | 0 | 0 | 0 | 0 | 1 | 0 | 0 | 0 | 0 | 0 | 0 | 0 | 3 | Bacteria | Bacteria\_none | Bacillota | Clostridia | Eubacteriales | Oscillospiraceae | Fastidiosipila | Bacteria;Bacteria\_none;Bacillota;Clostridia;Eubacteriales;Oscillospiraceae;Fastidiosipila;Fastidiosipila sanguinis |
| Mageeibacillus indolicus | 0 | 0 | 0 | 0 | 0 | 0 | 0 | 0 | 0 | 0 | 0 | 0 | 1 | 1 | Bacteria | Bacteria\_none | Bacillota | Clostridia | Eubacteriales | Oscillospiraceae | Mageeibacillus | Bacteria;Bacteria\_none;Bacillota;Clostridia;Eubacteriales;Oscillospiraceae;Mageeibacillus;Mageeibacillus indolicus |
| Paludicola sp. MB14-C6 | 0 | 0 | 0 | 0 | 0 | 1 | 0 | 0 | 0 | 0 | 0 | 0 | 0 | 1 | Bacteria | Bacteria\_none | Bacillota | Clostridia | Eubacteriales | Oscillospiraceae | Paludihabitans | Bacteria;Bacteria\_none;Bacillota;Clostridia;Eubacteriales;Oscillospiraceae;Paludihabitans;Paludicola sp. MB14-C6 |
| Petroclostridium sp. X23 | 0 | 1 | 0 | 0 | 0 | 0 | 0 | 0 | 0 | 0 | 0 | 0 | 0 | 1 | Bacteria | Bacteria\_none | Bacillota | Clostridia | Eubacteriales | Oscillospiraceae | Petroclostridium | Bacteria;Bacteria\_none;Bacillota;Clostridia;Eubacteriales;Oscillospiraceae;Petroclostridium;Petroclostridium sp. X23 |
| Ruminiclostridium cellulolyticum | 0 | 0 | 0 | 1 | 0 | 0 | 0 | 0 | 0 | 0 | 0 | 0 | 0 | 1 | Bacteria | Bacteria\_none | Bacillota | Clostridia | Eubacteriales | Oscillospiraceae | Ruminiclostridium | Bacteria;Bacteria\_none;Bacillota;Clostridia;Eubacteriales;Oscillospiraceae;Ruminiclostridium;Ruminiclostridium cellulolyticum |
| Ruminococcus bicirculans (ex Wegman et al. 2014) | 0 | 1 | 0 | 0 | 1 | 0 | 0 | 0 | 0 | 0 | 0 | 0 | 0 | 2 | Bacteria | Bacteria\_none | Bacillota | Clostridia | Eubacteriales | Oscillospiraceae | Ruminococcus | Bacteria;Bacteria\_none;Bacillota;Clostridia;Eubacteriales;Oscillospiraceae;Ruminococcus;Ruminococcus bicirculans (ex Wegman et al. 2014) |
| Thermoclostridium stercorarium | 0 | 0 | 0 | 1 | 0 | 1 | 0 | 0 | 0 | 0 | 0 | 0 | 0 | 2 | Bacteria | Bacteria\_none | Bacillota | Clostridia | Eubacteriales | Oscillospiraceae | Thermoclostridium | Bacteria;Bacteria\_none;Bacillota;Clostridia;Eubacteriales;Oscillospiraceae;Thermoclostridium;Thermoclostridium stercorarium |
| Halobacteroides halobius | 0 | 0 | 0 | 0 | 0 | 0 | 0 | 0 | 0 | 0 | 0 | 0 | 1 | 1 | Bacteria | Bacteria\_none | Bacillota | Clostridia | Halanaerobiales | Halobacteroidaceae | Halobacteroides | Bacteria;Bacteria\_none;Bacillota;Clostridia;Halanaerobiales;Halobacteroidaceae;Halobacteroides;Halobacteroides halobius |
| Anaerotignum sp. MB30-C6 | 0 | 0 | 0 | 0 | 0 | 0 | 1 | 0 | 0 | 0 | 0 | 0 | 0 | 1 | Bacteria | Bacteria\_none | Bacillota | Clostridia | Lachnospirales | Anaerotignaceae | Anaerotignum | Bacteria;Bacteria\_none;Bacillota;Clostridia;Lachnospirales;Anaerotignaceae;Anaerotignum;Anaerotignum sp. MB30-C6 |
| Agathobacter rectalis | 0 | 0 | 1 | 0 | 0 | 0 | 0 | 0 | 0 | 0 | 0 | 0 | 0 | 1 | Bacteria | Bacteria\_none | Bacillota | Clostridia | Lachnospirales | Lachnospiraceae | Agathobacter | Bacteria;Bacteria\_none;Bacillota;Clostridia;Lachnospirales;Lachnospiraceae;Agathobacter;Agathobacter rectalis |
| Anaerobutyricum hallii | 0 | 0 | 0 | 0 | 2 | 0 | 0 | 0 | 0 | 1 | 0 | 0 | 1 | 4 | Bacteria | Bacteria\_none | Bacillota | Clostridia | Lachnospirales | Lachnospiraceae | Anaerobutyricum | Bacteria;Bacteria\_none;Bacillota;Clostridia;Lachnospirales;Lachnospiraceae;Anaerobutyricum;Anaerobutyricum hallii |
| Anaerocolumna sedimenticola | 0 | 0 | 0 | 0 | 0 | 1 | 0 | 0 | 0 | 0 | 0 | 0 | 0 | 1 | Bacteria | Bacteria\_none | Bacillota | Clostridia | Lachnospirales | Lachnospiraceae | Anaerocolumna | Bacteria;Bacteria\_none;Bacillota;Clostridia;Lachnospirales;Lachnospiraceae;Anaerocolumna;Anaerocolumna sedimenticola |
| Anaerocolumna sp. AGMB13020 | 0 | 0 | 0 | 0 | 0 | 1 | 0 | 0 | 0 | 0 | 0 | 0 | 0 | 1 | Bacteria | Bacteria\_none | Bacillota | Clostridia | Lachnospirales | Lachnospiraceae | Anaerocolumna | Bacteria;Bacteria\_none;Bacillota;Clostridia;Lachnospirales;Lachnospiraceae;Anaerocolumna;Anaerocolumna sp. AGMB13020 |
| Anaerocolumna sp. MB42-C2 | 0 | 0 | 0 | 0 | 0 | 3 | 0 | 0 | 0 | 0 | 0 | 0 | 0 | 3 | Bacteria | Bacteria\_none | Bacillota | Clostridia | Lachnospirales | Lachnospiraceae | Anaerocolumna | Bacteria;Bacteria\_none;Bacillota;Clostridia;Lachnospirales;Lachnospiraceae;Anaerocolumna;Anaerocolumna sp. MB42-C2 |
| Anaeromicropila herbilytica | 0 | 0 | 1 | 0 | 0 | 0 | 0 | 1 | 0 | 0 | 0 | 0 | 0 | 2 | Bacteria | Bacteria\_none | Bacillota | Clostridia | Lachnospirales | Lachnospiraceae | Anaeromicropila | Bacteria;Bacteria\_none;Bacillota;Clostridia;Lachnospirales;Lachnospiraceae;Anaeromicropila;Anaeromicropila herbilytica |
| Anaerostipes hadrus | 0 | 0 | 0 | 0 | 0 | 0 | 0 | 0 | 0 | 0 | 0 | 0 | 1 | 1 | Bacteria | Bacteria\_none | Bacillota | Clostridia | Lachnospirales | Lachnospiraceae | Anaerostipes | Bacteria;Bacteria\_none;Bacillota;Clostridia;Lachnospirales;Lachnospiraceae;Anaerostipes;Anaerostipes hadrus |
| Blautia hydrogenotrophica | 0 | 0 | 0 | 0 | 0 | 0 | 0 | 0 | 0 | 0 | 0 | 0 | 1 | 1 | Bacteria | Bacteria\_none | Bacillota | Clostridia | Lachnospirales | Lachnospiraceae | Blautia | Bacteria;Bacteria\_none;Bacillota;Clostridia;Lachnospirales;Lachnospiraceae;Blautia;Blautia hydrogenotrophica |
| Blautia obeum | 0 | 0 | 0 | 0 | 0 | 1 | 2 | 0 | 0 | 0 | 0 | 0 | 0 | 3 | Bacteria | Bacteria\_none | Bacillota | Clostridia | Lachnospirales | Lachnospiraceae | Blautia | Bacteria;Bacteria\_none;Bacillota;Clostridia;Lachnospirales;Lachnospiraceae;Blautia;Blautia obeum |
| Blautia parvula | 0 | 0 | 0 | 0 | 0 | 2 | 0 | 0 | 0 | 0 | 0 | 0 | 0 | 2 | Bacteria | Bacteria\_none | Bacillota | Clostridia | Lachnospirales | Lachnospiraceae | Blautia | Bacteria;Bacteria\_none;Bacillota;Clostridia;Lachnospirales;Lachnospiraceae;Blautia;Blautia parvula |
| Blautia pseudococcoides | 0 | 0 | 0 | 0 | 0 | 1 | 0 | 0 | 0 | 0 | 0 | 0 | 0 | 1 | Bacteria | Bacteria\_none | Bacillota | Clostridia | Lachnospirales | Lachnospiraceae | Blautia | Bacteria;Bacteria\_none;Bacillota;Clostridia;Lachnospirales;Lachnospiraceae;Blautia;Blautia pseudococcoides |
| Butyrivibrio crossotus | 0 | 0 | 0 | 0 | 0 | 2 | 0 | 0 | 0 | 0 | 1 | 0 | 0 | 3 | Bacteria | Bacteria\_none | Bacillota | Clostridia | Lachnospirales | Lachnospiraceae | Butyrivibrio | Bacteria;Bacteria\_none;Bacillota;Clostridia;Lachnospirales;Lachnospiraceae;Butyrivibrio;Butyrivibrio crossotus |
| Butyrivibrio fibrisolvens | 0 | 0 | 0 | 0 | 0 | 1 | 0 | 0 | 0 | 0 | 0 | 0 | 0 | 1 | Bacteria | Bacteria\_none | Bacillota | Clostridia | Lachnospirales | Lachnospiraceae | Butyrivibrio | Bacteria;Bacteria\_none;Bacillota;Clostridia;Lachnospirales;Lachnospiraceae;Butyrivibrio;Butyrivibrio fibrisolvens |
| Butyrivibrio proteoclasticus | 0 | 0 | 0 | 0 | 0 | 1 | 0 | 0 | 0 | 0 | 0 | 0 | 0 | 1 | Bacteria | Bacteria\_none | Bacillota | Clostridia | Lachnospirales | Lachnospiraceae | Butyrivibrio | Bacteria;Bacteria\_none;Bacillota;Clostridia;Lachnospirales;Lachnospiraceae;Butyrivibrio;Butyrivibrio proteoclasticus |
| Candidatus Epulonipiscium viviparus | 0 | 0 | 0 | 0 | 0 | 0 | 0 | 0 | 0 | 0 | 0 | 1 | 0 | 1 | Bacteria | Bacteria\_none | Bacillota | Clostridia | Lachnospirales | Lachnospiraceae | Candidatus Epulonipiscium | Bacteria;Bacteria\_none;Bacillota;Clostridia;Lachnospirales;Lachnospiraceae;Candidatus Epulonipiscium;Candidatus Epulonipiscium viviparus |
| Enterocloster bolteae | 0 | 0 | 0 | 0 | 0 | 0 | 0 | 0 | 0 | 0 | 1 | 0 | 0 | 1 | Bacteria | Bacteria\_none | Bacillota | Clostridia | Lachnospirales | Lachnospiraceae | Enterocloster | Bacteria;Bacteria\_none;Bacillota;Clostridia;Lachnospirales;Lachnospiraceae;Enterocloster;Enterocloster bolteae |
| Hungatella hathewayi | 0 | 0 | 0 | 0 | 0 | 0 | 1 | 0 | 0 | 0 | 0 | 0 | 0 | 1 | Bacteria | Bacteria\_none | Bacillota | Clostridia | Lachnospirales | Lachnospiraceae | Hungatella | Bacteria;Bacteria\_none;Bacillota;Clostridia;Lachnospirales;Lachnospiraceae;Hungatella;Hungatella hathewayi |
| Lachnoanaerobaculum gingivalis | 1 | 22 | 6 | 13 | 5 | 20 | 1 | 0 | 1 | 4 | 1 | 3 | 11 | 88 | Bacteria | Bacteria\_none | Bacillota | Clostridia | Lachnospirales | Lachnospiraceae | Lachnoanaerobaculum | Bacteria;Bacteria\_none;Bacillota;Clostridia;Lachnospirales;Lachnospiraceae;Lachnoanaerobaculum;Lachnoanaerobaculum gingivalis |
| Lachnoanaerobaculum umeaense | 0 | 4 | 0 | 3 | 5 | 9 | 1 | 0 | 0 | 1 | 0 | 0 | 4 | 27 | Bacteria | Bacteria\_none | Bacillota | Clostridia | Lachnospirales | Lachnospiraceae | Lachnoanaerobaculum | Bacteria;Bacteria\_none;Bacillota;Clostridia;Lachnospirales;Lachnospiraceae;Lachnoanaerobaculum;Lachnoanaerobaculum umeaense |
| [Clostridium] scindens | 0 | 0 | 1 | 0 | 0 | 2 | 0 | 0 | 0 | 0 | 0 | 0 | 0 | 3 | Bacteria | Bacteria\_none | Bacillota | Clostridia | Lachnospirales | Lachnospiraceae | Lachnoclostridium | Bacteria;Bacteria\_none;Bacillota;Clostridia;Lachnospirales;Lachnospiraceae;Lachnoclostridium;[Clostridium] scindens |
| [Ruminococcus] torques | 0 | 0 | 0 | 0 | 0 | 0 | 0 | 0 | 0 | 0 | 0 | 3 | 0 | 3 | Bacteria | Bacteria\_none | Bacillota | Clostridia | Lachnospirales | Lachnospiraceae | Mediterraneibacter | Bacteria;Bacteria\_none;Bacillota;Clostridia;Lachnospirales;Lachnospiraceae;Mediterraneibacter;[Ruminococcus] torques |
| Qiania dongpingensis | 0 | 0 | 0 | 0 | 1 | 0 | 0 | 0 | 0 | 0 | 0 | 0 | 0 | 1 | Bacteria | Bacteria\_none | Bacillota | Clostridia | Lachnospirales | Lachnospiraceae | Qiania | Bacteria;Bacteria\_none;Bacillota;Clostridia;Lachnospirales;Lachnospiraceae;Qiania;Qiania dongpingensis |
| Roseburia hominis | 0 | 0 | 2 | 0 | 0 | 0 | 0 | 0 | 0 | 0 | 0 | 0 | 1 | 3 | Bacteria | Bacteria\_none | Bacillota | Clostridia | Lachnospirales | Lachnospiraceae | Roseburia | Bacteria;Bacteria\_none;Bacillota;Clostridia;Lachnospirales;Lachnospiraceae;Roseburia;Roseburia hominis |
| Roseburia sp. 499 | 0 | 0 | 0 | 0 | 0 | 1 | 0 | 0 | 0 | 0 | 0 | 0 | 0 | 1 | Bacteria | Bacteria\_none | Bacillota | Clostridia | Lachnospirales | Lachnospiraceae | Roseburia | Bacteria;Bacteria\_none;Bacillota;Clostridia;Lachnospirales;Lachnospiraceae;Roseburia;Roseburia sp. 499 |
| Roseburia sp. 831b | 0 | 0 | 0 | 0 | 0 | 1 | 0 | 0 | 0 | 0 | 0 | 0 | 0 | 1 | Bacteria | Bacteria\_none | Bacillota | Clostridia | Lachnospirales | Lachnospiraceae | Roseburia | Bacteria;Bacteria\_none;Bacillota;Clostridia;Lachnospirales;Lachnospiraceae;Roseburia;Roseburia sp. 831b |
| Simiaoa sunii | 0 | 0 | 0 | 0 | 0 | 0 | 0 | 0 | 0 | 0 | 0 | 0 | 1 | 1 | Bacteria | Bacteria\_none | Bacillota | Clostridia | Lachnospirales | Lachnospiraceae | Simiaoa | Bacteria;Bacteria\_none;Bacillota;Clostridia;Lachnospirales;Lachnospiraceae;Simiaoa;Simiaoa sunii |
| Stomatobaculum sp. F0698 | 0 | 9 | 17 | 1 | 0 | 20 | 8 | 0 | 0 | 5 | 2 | 1 | 9 | 72 | Bacteria | Bacteria\_none | Bacillota | Clostridia | Lachnospirales | Lachnospiraceae | Stomatobaculum | Bacteria;Bacteria\_none;Bacillota;Clostridia;Lachnospirales;Lachnospiraceae;Stomatobaculum;Stomatobaculum sp. F0698 |
| [Clostridium] colinum | 0 | 0 | 0 | 0 | 1 | 0 | 0 | 0 | 0 | 0 | 0 | 0 | 0 | 1 | Bacteria | Bacteria\_none | Bacillota | Clostridia | Lachnospirales | Lachnospiraceae | Tyzzerella | Bacteria;Bacteria\_none;Bacillota;Clostridia;Lachnospirales;Lachnospiraceae;Tyzzerella;[Clostridium] colinum |
| Wansuia hejianensis | 0 | 0 | 0 | 0 | 1 | 0 | 0 | 0 | 0 | 0 | 0 | 0 | 0 | 1 | Bacteria | Bacteria\_none | Bacillota | Clostridia | Lachnospirales | Lachnospiraceae | Wansuia | Bacteria;Bacteria\_none;Bacillota;Clostridia;Lachnospirales;Lachnospiraceae;Wansuia;Wansuia hejianensis |
| Wujia chipingensis | 0 | 0 | 0 | 0 | 0 | 0 | 0 | 0 | 0 | 0 | 0 | 0 | 1 | 1 | Bacteria | Bacteria\_none | Bacillota | Clostridia | Lachnospirales | Lachnospiraceae | Wujia | Bacteria;Bacteria\_none;Bacillota;Clostridia;Lachnospirales;Lachnospiraceae;Wujia;Wujia chipingensis |
| Monoglobus pectinilyticus | 0 | 0 | 0 | 0 | 0 | 0 | 0 | 2 | 0 | 0 | 0 | 0 | 0 | 2 | Bacteria | Bacteria\_none | Bacillota | Clostridia | Monoglobales | Monoglobaceae | Monoglobus | Bacteria;Bacteria\_none;Bacillota;Clostridia;Monoglobales;Monoglobaceae;Monoglobus;Monoglobus pectinilyticus |
| Natranaerobius thermophilus | 0 | 0 | 0 | 1 | 0 | 0 | 0 | 0 | 0 | 0 | 0 | 0 | 0 | 1 | Bacteria | Bacteria\_none | Bacillota | Clostridia | Natranaerobiales | Natranaerobiaceae | Natranaerobius | Bacteria;Bacteria\_none;Bacillota;Clostridia;Natranaerobiales;Natranaerobiaceae;Natranaerobius;Natranaerobius thermophilus |
| Natranaerofaba carboxydovora | 0 | 0 | 0 | 0 | 0 | 1 | 0 | 0 | 0 | 0 | 0 | 0 | 0 | 1 | Bacteria | Bacteria\_none | Bacillota | Clostridia | Natranaerobiales | Natranaerofabaceae | Natranaerofaba | Bacteria;Bacteria\_none;Bacillota;Clostridia;Natranaerobiales;Natranaerofabaceae;Natranaerofaba;Natranaerofaba carboxydovora |
| Aminipila butyrica | 0 | 0 | 0 | 0 | 0 | 1 | 0 | 0 | 0 | 0 | 0 | 0 | 0 | 1 | Bacteria | Bacteria\_none | Bacillota | Clostridia | Peptostreptococcales | Anaerovoracaceae | Aminipila | Bacteria;Bacteria\_none;Bacillota;Clostridia;Peptostreptococcales;Anaerovoracaceae;Aminipila;Aminipila butyrica |
| Aminipila luticellarii | 0 | 0 | 0 | 0 | 0 | 0 | 0 | 0 | 0 | 0 | 0 | 1 | 0 | 1 | Bacteria | Bacteria\_none | Bacillota | Clostridia | Peptostreptococcales | Anaerovoracaceae | Aminipila | Bacteria;Bacteria\_none;Bacillota;Clostridia;Peptostreptococcales;Anaerovoracaceae;Aminipila;Aminipila luticellarii |
| Emergencia timonensis | 0 | 0 | 0 | 0 | 0 | 0 | 0 | 0 | 0 | 0 | 0 | 0 | 1 | 1 | Bacteria | Bacteria\_none | Bacillota | Clostridia | Peptostreptococcales | Anaerovoracaceae | Emergencia | Bacteria;Bacteria\_none;Bacillota;Clostridia;Peptostreptococcales;Anaerovoracaceae;Emergencia;Emergencia timonensis |
| Mogibacterium diversum | 1 | 0 | 4 | 1 | 0 | 31 | 2 | 2 | 1 | 0 | 0 | 2 | 21 | 65 | Bacteria | Bacteria\_none | Bacillota | Clostridia | Peptostreptococcales | Anaerovoracaceae | Mogibacterium | Bacteria;Bacteria\_none;Bacillota;Clostridia;Peptostreptococcales;Anaerovoracaceae;Mogibacterium;Mogibacterium diversum |
| Mogibacterium neglectum | 0 | 0 | 3 | 0 | 2 | 3 | 0 | 0 | 0 | 0 | 4 | 0 | 1 | 13 | Bacteria | Bacteria\_none | Bacillota | Clostridia | Peptostreptococcales | Anaerovoracaceae | Mogibacterium | Bacteria;Bacteria\_none;Bacillota;Clostridia;Peptostreptococcales;Anaerovoracaceae;Mogibacterium;Mogibacterium neglectum |
| Mogibacterium pumilum | 0 | 0 | 2 | 0 | 0 | 0 | 1 | 3 | 0 | 0 | 0 | 0 | 0 | 6 | Bacteria | Bacteria\_none | Bacillota | Clostridia | Peptostreptococcales | Anaerovoracaceae | Mogibacterium | Bacteria;Bacteria\_none;Bacillota;Clostridia;Peptostreptococcales;Anaerovoracaceae;Mogibacterium;Mogibacterium pumilum |
| Filifactor alocis | 3 | 38 | 35 | 0 | 2 | 2 | 3 | 54 | 0 | 0 | 2 | 18 | 46 | 203 | Bacteria | Bacteria\_none | Bacillota | Clostridia | Peptostreptococcales | Filifactoraceae | Filifactor | Bacteria;Bacteria\_none;Bacillota;Clostridia;Peptostreptococcales;Filifactoraceae;Filifactor;Filifactor alocis |
| Serpentinicella alkaliphila | 0 | 0 | 0 | 1 | 0 | 0 | 0 | 0 | 0 | 0 | 0 | 0 | 0 | 1 | Bacteria | Bacteria\_none | Bacillota | Clostridia | Peptostreptococcales | Natronincolaceae | Serpentinicella | Bacteria;Bacteria\_none;Bacillota;Clostridia;Peptostreptococcales;Natronincolaceae;Serpentinicella;Serpentinicella alkaliphila |
| Clostridioides difficile | 0 | 0 | 0 | 1 | 4 | 0 | 1 | 0 | 1 | 0 | 1 | 0 | 1 | 9 | Bacteria | Bacteria\_none | Bacillota | Clostridia | Peptostreptococcales | Peptostreptococcaceae | Clostridioides | Bacteria;Bacteria\_none;Bacillota;Clostridia;Peptostreptococcales;Peptostreptococcaceae;Clostridioides;Clostridioides difficile |
| Peptostreptococcus equinus | 0 | 0 | 3 | 3 | 0 | 1 | 0 | 0 | 0 | 0 | 1 | 0 | 1 | 9 | Bacteria | Bacteria\_none | Bacillota | Clostridia | Peptostreptococcales | Peptostreptococcaceae | Peptostreptococcus | Bacteria;Bacteria\_none;Bacillota;Clostridia;Peptostreptococcales;Peptostreptococcaceae;Peptostreptococcus;Peptostreptococcus equinus |
| Romboutsia ilealis | 0 | 0 | 0 | 1 | 0 | 0 | 0 | 0 | 0 | 0 | 0 | 0 | 0 | 1 | Bacteria | Bacteria\_none | Bacillota | Clostridia | Peptostreptococcales | Peptostreptococcaceae | Romboutsia | Bacteria;Bacteria\_none;Bacillota;Clostridia;Peptostreptococcales;Peptostreptococcaceae;Romboutsia;Romboutsia ilealis |
| Romboutsia sp. 13368 | 0 | 1 | 0 | 0 | 0 | 0 | 0 | 0 | 0 | 0 | 0 | 0 | 0 | 1 | Bacteria | Bacteria\_none | Bacillota | Clostridia | Peptostreptococcales | Peptostreptococcaceae | Romboutsia | Bacteria;Bacteria\_none;Bacillota;Clostridia;Peptostreptococcales;Peptostreptococcaceae;Romboutsia;Romboutsia sp. 13368 |
| Terrisporobacter glycolicus | 0 | 0 | 0 | 0 | 2 | 0 | 0 | 0 | 0 | 0 | 0 | 0 | 1 | 3 | Bacteria | Bacteria\_none | Bacillota | Clostridia | Peptostreptococcales | Peptostreptococcaceae | Terrisporobacter | Bacteria;Bacteria\_none;Bacillota;Clostridia;Peptostreptococcales;Peptostreptococcaceae;Terrisporobacter;Terrisporobacter glycolicus |
| Terrisporobacter petrolearius | 1 | 0 | 0 | 0 | 1 | 0 | 0 | 0 | 0 | 0 | 2 | 0 | 1 | 5 | Bacteria | Bacteria\_none | Bacillota | Clostridia | Peptostreptococcales | Peptostreptococcaceae | Terrisporobacter | Bacteria;Bacteria\_none;Bacillota;Clostridia;Peptostreptococcales;Peptostreptococcaceae;Terrisporobacter;Terrisporobacter petrolearius |
| Thermoanaerobacterium sp. RBIITD | 0 | 1 | 0 | 0 | 0 | 0 | 0 | 0 | 0 | 1 | 0 | 0 | 0 | 2 | Bacteria | Bacteria\_none | Bacillota | Clostridia | Thermoanaerobacterales | Thermoanaerobacteraceae | Thermoanaerobacterium | Bacteria;Bacteria\_none;Bacillota;Clostridia;Thermoanaerobacterales;Thermoanaerobacteraceae;Thermoanaerobacterium;Thermoanaerobacterium sp. RBIITD |
| Catenibacterium sp. co\_0103 | 0 | 0 | 0 | 1 | 0 | 0 | 0 | 0 | 0 | 0 | 0 | 0 | 0 | 1 | Bacteria | Bacteria\_none | Bacillota | Erysipelotrichia | Erysipelotrichales | Coprobacillaceae | Catenibacterium | Bacteria;Bacteria\_none;Bacillota;Erysipelotrichia;Erysipelotrichales;Coprobacillaceae;Catenibacterium;Catenibacterium sp. co\_0103 |
| Faecalibacillus intestinalis | 0 | 0 | 0 | 0 | 0 | 1 | 0 | 0 | 0 | 0 | 0 | 0 | 1 | 2 | Bacteria | Bacteria\_none | Bacillota | Erysipelotrichia | Erysipelotrichales | Coprobacillaceae | Faecalibacillus | Bacteria;Bacteria\_none;Bacillota;Erysipelotrichia;Erysipelotrichales;Coprobacillaceae;Faecalibacillus;Faecalibacillus intestinalis |
| Erysipelatoclostridium sp. An173 | 0 | 0 | 0 | 0 | 0 | 0 | 0 | 0 | 0 | 0 | 0 | 0 | 1 | 1 | Bacteria | Bacteria\_none | Bacillota | Erysipelotrichia | Erysipelotrichales | Coprobacillaceae | Thomasclavelia | Bacteria;Bacteria\_none;Bacillota;Erysipelotrichia;Erysipelotrichales;Coprobacillaceae;Thomasclavelia;Erysipelatoclostridium sp. An173 |
| Thomasclavelia ramosa | 0 | 0 | 0 | 0 | 0 | 0 | 1 | 0 | 0 | 0 | 0 | 0 | 0 | 1 | Bacteria | Bacteria\_none | Bacillota | Erysipelotrichia | Erysipelotrichales | Coprobacillaceae | Thomasclavelia | Bacteria;Bacteria\_none;Bacillota;Erysipelotrichia;Erysipelotrichales;Coprobacillaceae;Thomasclavelia;Thomasclavelia ramosa |
| Thomasclavelia spiroformis | 0 | 0 | 0 | 0 | 0 | 0 | 1 | 0 | 0 | 0 | 0 | 0 | 0 | 1 | Bacteria | Bacteria\_none | Bacillota | Erysipelotrichia | Erysipelotrichales | Coprobacillaceae | Thomasclavelia | Bacteria;Bacteria\_none;Bacillota;Erysipelotrichia;Erysipelotrichales;Coprobacillaceae;Thomasclavelia;Thomasclavelia spiroformis |
| Bulleidia sp. zg-1006 | 0 | 0 | 0 | 0 | 0 | 0 | 0 | 0 | 0 | 0 | 2 | 0 | 1 | 3 | Bacteria | Bacteria\_none | Bacillota | Erysipelotrichia | Erysipelotrichales | Erysipelotrichaceae | Bulleidia | Bacteria;Bacteria\_none;Bacillota;Erysipelotrichia;Erysipelotrichales;Erysipelotrichaceae;Bulleidia;Bulleidia sp. zg-1006 |
| Erysipelothrix inopinata | 0 | 1 | 0 | 0 | 0 | 0 | 0 | 0 | 0 | 0 | 0 | 0 | 0 | 1 | Bacteria | Bacteria\_none | Bacillota | Erysipelotrichia | Erysipelotrichales | Erysipelotrichaceae | Erysipelothrix | Bacteria;Bacteria\_none;Bacillota;Erysipelotrichia;Erysipelotrichales;Erysipelotrichaceae;Erysipelothrix;Erysipelothrix inopinata |
| Erysipelothrix sp. HDW6A | 0 | 0 | 0 | 0 | 1 | 1 | 0 | 0 | 0 | 0 | 0 | 0 | 0 | 2 | Bacteria | Bacteria\_none | Bacillota | Erysipelotrichia | Erysipelotrichales | Erysipelotrichaceae | Erysipelothrix | Bacteria;Bacteria\_none;Bacillota;Erysipelotrichia;Erysipelotrichales;Erysipelotrichaceae;Erysipelothrix;Erysipelothrix sp. HDW6A |
| [Clostridium] innocuum | 0 | 0 | 0 | 0 | 0 | 0 | 0 | 0 | 0 | 2 | 0 | 0 | 0 | 2 | Bacteria | Bacteria\_none | Bacillota | Erysipelotrichia | Erysipelotrichales | Erysipelotrichaceae | Erysipelotrichaceae\_Incertae\_sedis | Bacteria;Bacteria\_none;Bacillota;Erysipelotrichia;Erysipelotrichales;Erysipelotrichaceae;Erysipelotrichaceae\_Incertae\_sedis;[Clostridium] innocuum |
| Solobacterium moorei | 14 | 16 | 92 | 9 | 15 | 521 | 47 | 21 | 1 | 6 | 8 | 2 | 151 | 903 | Bacteria | Bacteria\_none | Bacillota | Erysipelotrichia | Erysipelotrichales | Erysipelotrichaceae | Solobacterium | Bacteria;Bacteria\_none;Bacillota;Erysipelotrichia;Erysipelotrichales;Erysipelotrichaceae;Solobacterium;Solobacterium moorei |
| Megamonas funiformis | 0 | 0 | 0 | 1 | 0 | 0 | 0 | 0 | 0 | 0 | 1 | 0 | 0 | 2 | Bacteria | Bacteria\_none | Bacillota | Negativicutes | Selenomonadales | Selenomonadaceae | Megamonas | Bacteria;Bacteria\_none;Bacillota;Negativicutes;Selenomonadales;Selenomonadaceae;Megamonas;Megamonas funiformis |
| Megamonas hypermegale | 0 | 0 | 1 | 0 | 0 | 0 | 0 | 0 | 0 | 0 | 0 | 0 | 0 | 1 | Bacteria | Bacteria\_none | Bacillota | Negativicutes | Selenomonadales | Selenomonadaceae | Megamonas | Bacteria;Bacteria\_none;Bacillota;Negativicutes;Selenomonadales;Selenomonadaceae;Megamonas;Megamonas hypermegale |
| Pectinatus frisingensis | 0 | 0 | 0 | 0 | 0 | 0 | 1 | 0 | 0 | 0 | 0 | 0 | 0 | 1 | Bacteria | Bacteria\_none | Bacillota | Negativicutes | Selenomonadales | Selenomonadaceae | Pectinatus | Bacteria;Bacteria\_none;Bacillota;Negativicutes;Selenomonadales;Selenomonadaceae;Pectinatus;Pectinatus frisingensis |
| Selenomonas dianae | 0 | 0 | 0 | 2 | 3 | 0 | 0 | 0 | 0 | 0 | 0 | 2 | 1 | 8 | Bacteria | Bacteria\_none | Bacillota | Negativicutes | Selenomonadales | Selenomonadaceae | Selenomonas | Bacteria;Bacteria\_none;Bacillota;Negativicutes;Selenomonadales;Selenomonadaceae;Selenomonas;Selenomonas dianae |
| Selenomonas sp. oral taxon 126 | 1 | 0 | 1 | 0 | 1 | 0 | 0 | 3 | 0 | 0 | 0 | 1 | 3 | 10 | Bacteria | Bacteria\_none | Bacillota | Negativicutes | Selenomonadales | Selenomonadaceae | Selenomonas | Bacteria;Bacteria\_none;Bacillota;Negativicutes;Selenomonadales;Selenomonadaceae;Selenomonas;Selenomonas sp. oral taxon 126 |
| Selenomonas sp. oral taxon 136 | 1 | 2 | 0 | 1 | 1 | 1 | 0 | 1 | 0 | 0 | 1 | 0 | 0 | 8 | Bacteria | Bacteria\_none | Bacillota | Negativicutes | Selenomonadales | Selenomonadaceae | Selenomonas | Bacteria;Bacteria\_none;Bacillota;Negativicutes;Selenomonadales;Selenomonadaceae;Selenomonas;Selenomonas sp. oral taxon 136 |
| Selenomonas sp. oral taxon 478 | 0 | 5 | 0 | 0 | 1 | 8 | 5 | 1 | 0 | 1 | 0 | 2 | 2 | 25 | Bacteria | Bacteria\_none | Bacillota | Negativicutes | Selenomonadales | Selenomonadaceae | Selenomonas | Bacteria;Bacteria\_none;Bacillota;Negativicutes;Selenomonadales;Selenomonadaceae;Selenomonas;Selenomonas sp. oral taxon 478 |
| Selenomonas sp. oral taxon 920 | 0 | 0 | 5 | 2 | 1 | 0 | 0 | 0 | 0 | 0 | 0 | 0 | 0 | 8 | Bacteria | Bacteria\_none | Bacillota | Negativicutes | Selenomonadales | Selenomonadaceae | Selenomonas | Bacteria;Bacteria\_none;Bacillota;Negativicutes;Selenomonadales;Selenomonadaceae;Selenomonas;Selenomonas sp. oral taxon 920 |
| Selenomonas sputigena | 3 | 0 | 4 | 0 | 2 | 5 | 2 | 3 | 0 | 1 | 1 | 4 | 8 | 33 | Bacteria | Bacteria\_none | Bacillota | Negativicutes | Selenomonadales | Selenomonadaceae | Selenomonas | Bacteria;Bacteria\_none;Bacillota;Negativicutes;Selenomonadales;Selenomonadaceae;Selenomonas;Selenomonas sputigena |
| Selenomonas timonae | 0 | 0 | 5 | 0 | 0 | 0 | 0 | 2 | 0 | 0 | 1 | 1 | 2 | 11 | Bacteria | Bacteria\_none | Bacillota | Negativicutes | Selenomonadales | Selenomonadaceae | Selenomonas | Bacteria;Bacteria\_none;Bacillota;Negativicutes;Selenomonadales;Selenomonadaceae;Selenomonas;Selenomonas timonae |
| Sporomusa ovata | 0 | 0 | 0 | 1 | 0 | 0 | 0 | 0 | 0 | 0 | 0 | 0 | 0 | 1 | Bacteria | Bacteria\_none | Bacillota | Negativicutes | Selenomonadales | Sporomusaceae | Sporomusa | Bacteria;Bacteria\_none;Bacillota;Negativicutes;Selenomonadales;Sporomusaceae;Sporomusa;Sporomusa ovata |
| Sporomusa termitida | 0 | 0 | 0 | 0 | 0 | 0 | 0 | 0 | 1 | 0 | 0 | 0 | 0 | 1 | Bacteria | Bacteria\_none | Bacillota | Negativicutes | Selenomonadales | Sporomusaceae | Sporomusa | Bacteria;Bacteria\_none;Bacillota;Negativicutes;Selenomonadales;Sporomusaceae;Sporomusa;Sporomusa termitida |
| Dialister pneumosintes | 0 | 0 | 1 | 0 | 0 | 0 | 0 | 1 | 0 | 0 | 2 | 2 | 5 | 11 | Bacteria | Bacteria\_none | Bacillota | Negativicutes | Veillonellales | Veillonellaceae | Dialister | Bacteria;Bacteria\_none;Bacillota;Negativicutes;Veillonellales;Veillonellaceae;Dialister;Dialister pneumosintes |
| Megasphaera elsdenii | 0 | 0 | 0 | 0 | 0 | 0 | 0 | 0 | 0 | 0 | 1 | 0 | 0 | 1 | Bacteria | Bacteria\_none | Bacillota | Negativicutes | Veillonellales | Veillonellaceae | Megasphaera | Bacteria;Bacteria\_none;Bacillota;Negativicutes;Veillonellales;Veillonellaceae;Megasphaera;Megasphaera elsdenii |
| Veillonella atypica | 83 | 15 | 5 | 2 | 8 | 36 | 8 | 1 | 55 | 6 | 4 | 39 | 72 | 334 | Bacteria | Bacteria\_none | Bacillota | Negativicutes | Veillonellales | Veillonellaceae | Veillonella | Bacteria;Bacteria\_none;Bacillota;Negativicutes;Veillonellales;Veillonellaceae;Veillonella;Veillonella atypica |
| Veillonella dispar | 141 | 6 | 2 | 0 | 1 | 4 | 5 | 0 | 0 | 2 | 0 | 3 | 62 | 226 | Bacteria | Bacteria\_none | Bacillota | Negativicutes | Veillonellales | Veillonellaceae | Veillonella | Bacteria;Bacteria\_none;Bacillota;Negativicutes;Veillonellales;Veillonellaceae;Veillonella;Veillonella dispar |
| Veillonella nakazawae | 316 | 15 | 3 | 2 | 2 | 4 | 5 | 2 | 1 | 5 | 0 | 1 | 131 | 487 | Bacteria | Bacteria\_none | Bacillota | Negativicutes | Veillonellales | Veillonellaceae | Veillonella | Bacteria;Bacteria\_none;Bacillota;Negativicutes;Veillonellales;Veillonellaceae;Veillonella;Veillonella nakazawae |
| Veillonella parvula | 186 | 14 | 29 | 14 | 13 | 2 | 16 | 37 | 13 | 42 | 7 | 46 | 136 | 555 | Bacteria | Bacteria\_none | Bacillota | Negativicutes | Veillonellales | Veillonellaceae | Veillonella | Bacteria;Bacteria\_none;Bacillota;Negativicutes;Veillonellales;Veillonellaceae;Veillonella;Veillonella parvula |
| Veillonella rodentium | 2 | 0 | 0 | 3 | 0 | 1 | 0 | 1 | 0 | 0 | 0 | 0 | 2 | 9 | Bacteria | Bacteria\_none | Bacillota | Negativicutes | Veillonellales | Veillonellaceae | Veillonella | Bacteria;Bacteria\_none;Bacillota;Negativicutes;Veillonellales;Veillonellaceae;Veillonella;Veillonella rodentium |
| Veillonella rogosae | 250 | 16 | 3 | 79 | 45 | 2 | 3 | 30 | 0 | 87 | 0 | 35 | 142 | 692 | Bacteria | Bacteria\_none | Bacillota | Negativicutes | Veillonellales | Veillonellaceae | Veillonella | Bacteria;Bacteria\_none;Bacillota;Negativicutes;Veillonellales;Veillonellaceae;Veillonella;Veillonella rogosae |
| Veillonella sp. OK1 | 13 | 0 | 0 | 0 | 0 | 0 | 0 | 0 | 0 | 0 | 0 | 0 | 5 | 18 | Bacteria | Bacteria\_none | Bacillota | Negativicutes | Veillonellales | Veillonellaceae | Veillonella | Bacteria;Bacteria\_none;Bacillota;Negativicutes;Veillonellales;Veillonellaceae;Veillonella;Veillonella sp. OK1 |
| Veillonella sp. S12025-13 | 195 | 12 | 0 | 0 | 0 | 4 | 0 | 1 | 1 | 1 | 0 | 0 | 100 | 314 | Bacteria | Bacteria\_none | Bacillota | Negativicutes | Veillonellales | Veillonellaceae | Veillonella | Bacteria;Bacteria\_none;Bacillota;Negativicutes;Veillonellales;Veillonellaceae;Veillonella;Veillonella sp. S12025-13 |
| Acidilutibacter cellobiosedens | 0 | 0 | 0 | 0 | 1 | 0 | 0 | 0 | 0 | 0 | 0 | 0 | 0 | 1 | Bacteria | Bacteria\_none | Bacillota | Tissierellia | Tissierellales | Acidilutibacteraceae | Acidilutibacter | Bacteria;Bacteria\_none;Bacillota;Tissierellia;Tissierellales;Acidilutibacteraceae;Acidilutibacter;Acidilutibacter cellobiosedens |
| Gottschalkia acidurici | 0 | 0 | 0 | 0 | 0 | 0 | 0 | 0 | 0 | 0 | 0 | 0 | 1 | 1 | Bacteria | Bacteria\_none | Bacillota | Tissierellia | Tissierellales | Gottschalkiaceae | Gottschalkia | Bacteria;Bacteria\_none;Bacillota;Tissierellia;Tissierellales;Gottschalkiaceae;Gottschalkia;Gottschalkia acidurici |
| Anaerococcus mediterraneensis | 1 | 0 | 0 | 0 | 0 | 0 | 0 | 0 | 0 | 0 | 0 | 0 | 0 | 1 | Bacteria | Bacteria\_none | Bacillota | Tissierellia | Tissierellales | Peptoniphilaceae | Anaerococcus | Bacteria;Bacteria\_none;Bacillota;Tissierellia;Tissierellales;Peptoniphilaceae;Anaerococcus;Anaerococcus mediterraneensis |
| Anaerococcus sp. Marseille-Q7828 | 0 | 0 | 0 | 0 | 0 | 0 | 0 | 0 | 0 | 0 | 1 | 0 | 0 | 1 | Bacteria | Bacteria\_none | Bacillota | Tissierellia | Tissierellales | Peptoniphilaceae | Anaerococcus | Bacteria;Bacteria\_none;Bacillota;Tissierellia;Tissierellales;Peptoniphilaceae;Anaerococcus;Anaerococcus sp. Marseille-Q7828 |
| Finegoldia magna | 0 | 0 | 0 | 0 | 0 | 1 | 0 | 0 | 0 | 0 | 1 | 0 | 0 | 2 | Bacteria | Bacteria\_none | Bacillota | Tissierellia | Tissierellales | Peptoniphilaceae | Finegoldia | Bacteria;Bacteria\_none;Bacillota;Tissierellia;Tissierellales;Peptoniphilaceae;Finegoldia;Finegoldia magna |
| Helcococcus ovis | 0 | 0 | 0 | 1 | 0 | 0 | 0 | 2 | 0 | 0 | 0 | 1 | 1 | 5 | Bacteria | Bacteria\_none | Bacillota | Tissierellia | Tissierellales | Peptoniphilaceae | Helcococcus | Bacteria;Bacteria\_none;Bacillota;Tissierellia;Tissierellales;Peptoniphilaceae;Helcococcus;Helcococcus ovis |
| Miniphocaeibacter halophilus | 0 | 0 | 0 | 0 | 0 | 0 | 0 | 0 | 0 | 0 | 0 | 0 | 1 | 1 | Bacteria | Bacteria\_none | Bacillota | Tissierellia | Tissierellales | Peptoniphilaceae | Miniphocaeibacter | Bacteria;Bacteria\_none;Bacillota;Tissierellia;Tissierellales;Peptoniphilaceae;Miniphocaeibacter;Miniphocaeibacter halophilus |
| Parvimonas micra | 5 | 34 | 25 | 15 | 48 | 70 | 7 | 63 | 0 | 2 | 71 | 25 | 145 | 510 | Bacteria | Bacteria\_none | Bacillota | Tissierellia | Tissierellales | Peptoniphilaceae | Parvimonas | Bacteria;Bacteria\_none;Bacillota;Tissierellia;Tissierellales;Peptoniphilaceae;Parvimonas;Parvimonas micra |
| Peptoniphilus genitalis | 0 | 0 | 0 | 0 | 0 | 1 | 0 | 0 | 0 | 0 | 0 | 0 | 0 | 1 | Bacteria | Bacteria\_none | Bacillota | Tissierellia | Tissierellales | Peptoniphilaceae | Peptoniphilus | Bacteria;Bacteria\_none;Bacillota;Tissierellia;Tissierellales;Peptoniphilaceae;Peptoniphilus;Peptoniphilus genitalis |
| Keratinibaculum paraultunense | 0 | 0 | 0 | 0 | 1 | 0 | 0 | 0 | 0 | 0 | 0 | 0 | 0 | 1 | Bacteria | Bacteria\_none | Bacillota | Tissierellia | Tissierellales | Tepidimicrobiaceae | Keratinibaculum | Bacteria;Bacteria\_none;Bacillota;Tissierellia;Tissierellales;Tepidimicrobiaceae;Keratinibaculum;Keratinibaculum paraultunense |
| Ezakiella coagulans | 0 | 0 | 0 | 1 | 0 | 0 | 0 | 0 | 0 | 0 | 12 | 1 | 5 | 19 | Bacteria | Bacteria\_none | Bacillota | Tissierellia | Tissierellia\_Incertae\_sedis | Tissierellia\_Incertae\_sedis | Ezakiella | Bacteria;Bacteria\_none;Bacillota;Tissierellia;Tissierellia\_Incertae\_sedis;Tissierellia\_Incertae\_sedis;Ezakiella;Ezakiella coagulans |
| Ezakiella massiliensis | 0 | 0 | 0 | 0 | 1 | 0 | 0 | 0 | 0 | 0 | 0 | 0 | 0 | 1 | Bacteria | Bacteria\_none | Bacillota | Tissierellia | Tissierellia\_Incertae\_sedis | Tissierellia\_Incertae\_sedis | Ezakiella | Bacteria;Bacteria\_none;Bacillota;Tissierellia;Tissierellia\_Incertae\_sedis;Tissierellia\_Incertae\_sedis;Ezakiella;Ezakiella massiliensis |
| Bacteroides caccae | 0 | 0 | 0 | 0 | 0 | 0 | 0 | 0 | 0 | 0 | 0 | 0 | 1 | 1 | Bacteria | Bacteria\_none | Bacteroidota | Bacteroidia | Bacteroidales | Bacteroidaceae | Bacteroides | Bacteria;Bacteria\_none;Bacteroidota;Bacteroidia;Bacteroidales;Bacteroidaceae;Bacteroides;Bacteroides caccae |
| Bacteroides caecimuris | 0 | 0 | 0 | 0 | 0 | 0 | 0 | 0 | 0 | 0 | 0 | 0 | 2 | 2 | Bacteria | Bacteria\_none | Bacteroidota | Bacteroidia | Bacteroidales | Bacteroidaceae | Bacteroides | Bacteria;Bacteria\_none;Bacteroidota;Bacteroidia;Bacteroidales;Bacteroidaceae;Bacteroides;Bacteroides caecimuris |
| Bacteroides cellulosilyticus | 2 | 0 | 0 | 1 | 0 | 0 | 0 | 0 | 0 | 0 | 0 | 0 | 0 | 3 | Bacteria | Bacteria\_none | Bacteroidota | Bacteroidia | Bacteroidales | Bacteroidaceae | Bacteroides | Bacteria;Bacteria\_none;Bacteroidota;Bacteroidia;Bacteroidales;Bacteroidaceae;Bacteroides;Bacteroides cellulosilyticus |
| Bacteroides eggerthii | 0 | 0 | 0 | 0 | 0 | 0 | 0 | 0 | 0 | 0 | 0 | 0 | 1 | 1 | Bacteria | Bacteria\_none | Bacteroidota | Bacteroidia | Bacteroidales | Bacteroidaceae | Bacteroides | Bacteria;Bacteria\_none;Bacteroidota;Bacteroidia;Bacteroidales;Bacteroidaceae;Bacteroides;Bacteroides eggerthii |
| Bacteroides faecium | 0 | 0 | 0 | 0 | 0 | 1 | 0 | 0 | 0 | 0 | 0 | 0 | 0 | 1 | Bacteria | Bacteria\_none | Bacteroidota | Bacteroidia | Bacteroidales | Bacteroidaceae | Bacteroides | Bacteria;Bacteria\_none;Bacteroidota;Bacteroidia;Bacteroidales;Bacteroidaceae;Bacteroides;Bacteroides faecium |
| Bacteroides fragilis | 0 | 0 | 0 | 0 | 0 | 0 | 0 | 0 | 0 | 0 | 1 | 0 | 0 | 1 | Bacteria | Bacteria\_none | Bacteroidota | Bacteroidia | Bacteroidales | Bacteroidaceae | Bacteroides | Bacteria;Bacteria\_none;Bacteroidota;Bacteroidia;Bacteroidales;Bacteroidaceae;Bacteroides;Bacteroides fragilis |
| Bacteroides graminisolvens | 0 | 0 | 0 | 0 | 0 | 0 | 0 | 0 | 0 | 0 | 0 | 0 | 1 | 1 | Bacteria | Bacteria\_none | Bacteroidota | Bacteroidia | Bacteroidales | Bacteroidaceae | Bacteroides | Bacteria;Bacteria\_none;Bacteroidota;Bacteroidia;Bacteroidales;Bacteroidaceae;Bacteroides;Bacteroides graminisolvens |
| Bacteroides helcogenes | 0 | 0 | 0 | 0 | 0 | 1 | 0 | 0 | 0 | 0 | 0 | 0 | 0 | 1 | Bacteria | Bacteria\_none | Bacteroidota | Bacteroidia | Bacteroidales | Bacteroidaceae | Bacteroides | Bacteria;Bacteria\_none;Bacteroidota;Bacteroidia;Bacteroidales;Bacteroidaceae;Bacteroides;Bacteroides helcogenes |
| Bacteroides heparinolyticus | 0 | 1 | 1 | 0 | 0 | 2 | 0 | 1 | 0 | 0 | 0 | 1 | 0 | 6 | Bacteria | Bacteria\_none | Bacteroidota | Bacteroidia | Bacteroidales | Bacteroidaceae | Bacteroides | Bacteria;Bacteria\_none;Bacteroidota;Bacteroidia;Bacteroidales;Bacteroidaceae;Bacteroides;Bacteroides heparinolyticus |
| Bacteroides humanifaecis | 0 | 0 | 0 | 6 | 0 | 0 | 0 | 0 | 0 | 0 | 0 | 0 | 0 | 6 | Bacteria | Bacteria\_none | Bacteroidota | Bacteroidia | Bacteroidales | Bacteroidaceae | Bacteroides | Bacteria;Bacteria\_none;Bacteroidota;Bacteroidia;Bacteroidales;Bacteroidaceae;Bacteroides;Bacteroides humanifaecis |
| Bacteroides intestinalis | 0 | 0 | 0 | 0 | 0 | 0 | 0 | 0 | 0 | 0 | 1 | 0 | 0 | 1 | Bacteria | Bacteria\_none | Bacteroidota | Bacteroidia | Bacteroidales | Bacteroidaceae | Bacteroides | Bacteria;Bacteria\_none;Bacteroidota;Bacteroidia;Bacteroidales;Bacteroidaceae;Bacteroides;Bacteroides intestinalis |
| Bacteroides ovatus | 0 | 0 | 0 | 0 | 0 | 1 | 0 | 0 | 0 | 0 | 0 | 0 | 0 | 1 | Bacteria | Bacteria\_none | Bacteroidota | Bacteroidia | Bacteroidales | Bacteroidaceae | Bacteroides | Bacteria;Bacteria\_none;Bacteroidota;Bacteroidia;Bacteroidales;Bacteroidaceae;Bacteroides;Bacteroides ovatus |
| Bacteroides sp. MSB163 | 0 | 0 | 0 | 1 | 0 | 0 | 0 | 0 | 0 | 0 | 0 | 0 | 0 | 1 | Bacteria | Bacteria\_none | Bacteroidota | Bacteroidia | Bacteroidales | Bacteroidaceae | Bacteroides | Bacteria;Bacteria\_none;Bacteroidota;Bacteroidia;Bacteroidales;Bacteroidaceae;Bacteroides;Bacteroides sp. MSB163 |
| Bacteroides thetaiotaomicron | 0 | 2 | 0 | 0 | 0 | 0 | 0 | 0 | 0 | 0 | 2 | 0 | 0 | 4 | Bacteria | Bacteria\_none | Bacteroidota | Bacteroidia | Bacteroidales | Bacteroidaceae | Bacteroides | Bacteria;Bacteria\_none;Bacteroidota;Bacteroidia;Bacteroidales;Bacteroidaceae;Bacteroides;Bacteroides thetaiotaomicron |
| Bacteroides uniformis | 0 | 0 | 0 | 0 | 0 | 1 | 0 | 0 | 0 | 0 | 0 | 0 | 0 | 1 | Bacteria | Bacteria\_none | Bacteroidota | Bacteroidia | Bacteroidales | Bacteroidaceae | Bacteroides | Bacteria;Bacteria\_none;Bacteroidota;Bacteroidia;Bacteroidales;Bacteroidaceae;Bacteroides;Bacteroides uniformis |
| Bacteroides xylanisolvens | 0 | 0 | 0 | 0 | 0 | 0 | 0 | 0 | 0 | 0 | 0 | 0 | 2 | 2 | Bacteria | Bacteria\_none | Bacteroidota | Bacteroidia | Bacteroidales | Bacteroidaceae | Bacteroides | Bacteria;Bacteria\_none;Bacteroidota;Bacteroidia;Bacteroidales;Bacteroidaceae;Bacteroides;Bacteroides xylanisolvens |
| Bacteroides zhangwenhongii | 0 | 0 | 0 | 0 | 0 | 0 | 0 | 1 | 0 | 0 | 0 | 0 | 0 | 1 | Bacteria | Bacteria\_none | Bacteroidota | Bacteroidia | Bacteroidales | Bacteroidaceae | Bacteroides | Bacteria;Bacteria\_none;Bacteroidota;Bacteroidia;Bacteroidales;Bacteroidaceae;Bacteroides;Bacteroides zhangwenhongii |
| Bacteroides zoogleoformans | 0 | 0 | 0 | 1 | 0 | 0 | 0 | 1 | 0 | 0 | 0 | 0 | 0 | 2 | Bacteria | Bacteria\_none | Bacteroidota | Bacteroidia | Bacteroidales | Bacteroidaceae | Bacteroides | Bacteria;Bacteria\_none;Bacteroidota;Bacteroidia;Bacteroidales;Bacteroidaceae;Bacteroides;Bacteroides zoogleoformans |
| Bacteroides\_uncultured | 0 | 0 | 0 | 0 | 0 | 0 | 0 | 0 | 0 | 1 | 3 | 0 | 1 | 5 | Bacteria | Bacteria\_none | Bacteroidota | Bacteroidia | Bacteroidales | Bacteroidaceae | Bacteroides | Bacteria;Bacteria\_none;Bacteroidota;Bacteroidia;Bacteroidales;Bacteroidaceae;Bacteroides;Bacteroides\_uncultured |
| Phocaeicola coprophilus | 0 | 0 | 0 | 0 | 0 | 1 | 0 | 0 | 0 | 0 | 0 | 0 | 0 | 1 | Bacteria | Bacteria\_none | Bacteroidota | Bacteroidia | Bacteroidales | Bacteroidaceae | Phocaeicola | Bacteria;Bacteria\_none;Bacteroidota;Bacteroidia;Bacteroidales;Bacteroidaceae;Phocaeicola;Phocaeicola coprophilus |
| Phocaeicola dorei | 0 | 0 | 0 | 0 | 0 | 0 | 0 | 0 | 0 | 0 | 0 | 0 | 2 | 2 | Bacteria | Bacteria\_none | Bacteroidota | Bacteroidia | Bacteroidales | Bacteroidaceae | Phocaeicola | Bacteria;Bacteria\_none;Bacteroidota;Bacteroidia;Bacteroidales;Bacteroidaceae;Phocaeicola;Phocaeicola dorei |
| Phocaeicola salanitronis | 0 | 0 | 0 | 1 | 0 | 0 | 0 | 0 | 0 | 0 | 0 | 0 | 0 | 1 | Bacteria | Bacteria\_none | Bacteroidota | Bacteroidia | Bacteroidales | Bacteroidaceae | Phocaeicola | Bacteria;Bacteria\_none;Bacteroidota;Bacteroidia;Bacteroidales;Bacteroidaceae;Phocaeicola;Phocaeicola salanitronis |
| Phocaeicola vulgatus | 0 | 0 | 0 | 0 | 1 | 0 | 0 | 0 | 0 | 0 | 1 | 0 | 0 | 2 | Bacteria | Bacteria\_none | Bacteroidota | Bacteroidia | Bacteroidales | Bacteroidaceae | Phocaeicola | Bacteria;Bacteria\_none;Bacteroidota;Bacteroidia;Bacteroidales;Bacteroidaceae;Phocaeicola;Phocaeicola vulgatus |
| Barnesiella sp. An22 | 0 | 0 | 0 | 0 | 0 | 1 | 0 | 0 | 0 | 0 | 0 | 0 | 0 | 1 | Bacteria | Bacteria\_none | Bacteroidota | Bacteroidia | Bacteroidales | Barnesiellaceae | Barnesiella | Bacteria;Bacteria\_none;Bacteroidota;Bacteroidia;Bacteroidales;Barnesiellaceae;Barnesiella;Barnesiella sp. An22 |
| Proteiniphilum saccharofermentans | 0 | 0 | 0 | 0 | 0 | 0 | 0 | 0 | 0 | 0 | 0 | 1 | 0 | 1 | Bacteria | Bacteria\_none | Bacteroidota | Bacteroidia | Bacteroidales | Dysgonomonadaceae | Proteiniphilum | Bacteria;Bacteria\_none;Bacteroidota;Bacteroidia;Bacteroidales;Dysgonomonadaceae;Proteiniphilum;Proteiniphilum saccharofermentans |
| Duncaniella dubosii | 0 | 0 | 0 | 0 | 0 | 0 | 0 | 0 | 0 | 0 | 1 | 0 | 0 | 1 | Bacteria | Bacteria\_none | Bacteroidota | Bacteroidia | Bacteroidales | Muribaculaceae | Duncaniella | Bacteria;Bacteria\_none;Bacteroidota;Bacteroidia;Bacteroidales;Muribaculaceae;Duncaniella;Duncaniella dubosii |
| Sodaliphilus pleomorphus | 0 | 0 | 0 | 0 | 1 | 0 | 0 | 0 | 0 | 0 | 0 | 0 | 1 | 2 | Bacteria | Bacteria\_none | Bacteroidota | Bacteroidia | Bacteroidales | Muribaculaceae | Sodaliphilus | Bacteria;Bacteria\_none;Bacteroidota;Bacteroidia;Bacteroidales;Muribaculaceae;Sodaliphilus;Sodaliphilus pleomorphus |
| Butyricimonas faecihominis | 0 | 0 | 0 | 0 | 0 | 0 | 0 | 0 | 0 | 0 | 0 | 1 | 0 | 1 | Bacteria | Bacteria\_none | Bacteroidota | Bacteroidia | Bacteroidales | Odoribacteraceae | Butyricimonas | Bacteria;Bacteria\_none;Bacteroidota;Bacteroidia;Bacteroidales;Odoribacteraceae;Butyricimonas;Butyricimonas faecihominis |
| Odoribacter splanchnicus | 0 | 0 | 0 | 0 | 0 | 0 | 0 | 0 | 0 | 0 | 0 | 0 | 1 | 1 | Bacteria | Bacteria\_none | Bacteroidota | Bacteroidia | Bacteroidales | Odoribacteraceae | Odoribacter | Bacteria;Bacteria\_none;Bacteroidota;Bacteroidia;Bacteroidales;Odoribacteraceae;Odoribacter;Odoribacter splanchnicus |
| Porphyromonas asaccharolytica | 0 | 0 | 0 | 0 | 0 | 0 | 0 | 0 | 0 | 1 | 0 | 0 | 0 | 1 | Bacteria | Bacteria\_none | Bacteroidota | Bacteroidia | Bacteroidales | Porphyromonadaceae | Porphyromonas | Bacteria;Bacteria\_none;Bacteroidota;Bacteroidia;Bacteroidales;Porphyromonadaceae;Porphyromonas;Porphyromonas asaccharolytica |
| Porphyromonas cangingivalis | 0 | 0 | 0 | 1 | 2 | 0 | 0 | 1 | 0 | 0 | 0 | 0 | 0 | 4 | Bacteria | Bacteria\_none | Bacteroidota | Bacteroidia | Bacteroidales | Porphyromonadaceae | Porphyromonas | Bacteria;Bacteria\_none;Bacteroidota;Bacteroidia;Bacteroidales;Porphyromonadaceae;Porphyromonas;Porphyromonas cangingivalis |
| Porphyromonas crevioricanis | 0 | 0 | 0 | 0 | 1 | 1 | 0 | 0 | 0 | 1 | 1 | 0 | 0 | 4 | Bacteria | Bacteria\_none | Bacteroidota | Bacteroidia | Bacteroidales | Porphyromonadaceae | Porphyromonas | Bacteria;Bacteria\_none;Bacteroidota;Bacteroidia;Bacteroidales;Porphyromonadaceae;Porphyromonas;Porphyromonas crevioricanis |
| Porphyromonas endodontalis | 2 | 109 | 3 | 19 | 23 | 3 | 12 | 93 | 0 | 14 | 80 | 10 | 65 | 433 | Bacteria | Bacteria\_none | Bacteroidota | Bacteroidia | Bacteroidales | Porphyromonadaceae | Porphyromonas | Bacteria;Bacteria\_none;Bacteroidota;Bacteroidia;Bacteroidales;Porphyromonadaceae;Porphyromonas;Porphyromonas endodontalis |
| Porphyromonas gingivalis | 0 | 7 | 4 | 5 | 1 | 4 | 0 | 11 | 0 | 2 | 4 | 32 | 32 | 102 | Bacteria | Bacteria\_none | Bacteroidota | Bacteroidia | Bacteroidales | Porphyromonadaceae | Porphyromonas | Bacteria;Bacteria\_none;Bacteroidota;Bacteroidia;Bacteroidales;Porphyromonadaceae;Porphyromonas;Porphyromonas gingivalis |
| Porphyromonas somerae | 0 | 0 | 0 | 0 | 0 | 0 | 0 | 0 | 0 | 1 | 0 | 0 | 0 | 1 | Bacteria | Bacteria\_none | Bacteroidota | Bacteroidia | Bacteroidales | Porphyromonadaceae | Porphyromonas | Bacteria;Bacteria\_none;Bacteroidota;Bacteroidia;Bacteroidales;Porphyromonadaceae;Porphyromonas;Porphyromonas somerae |
| Porphyromonas sp. oral taxon 275 | 3 | 13 | 2 | 27 | 36 | 24 | 1 | 0 | 0 | 33 | 6 | 3 | 6 | 154 | Bacteria | Bacteria\_none | Bacteroidota | Bacteroidia | Bacteroidales | Porphyromonadaceae | Porphyromonas | Bacteria;Bacteria\_none;Bacteroidota;Bacteroidia;Bacteroidales;Porphyromonadaceae;Porphyromonas;Porphyromonas sp. oral taxon 275 |
| Hoylesella buccalis | 1 | 0 | 0 | 3 | 1 | 0 | 0 | 0 | 0 | 0 | 1 | 0 | 0 | 6 | Bacteria | Bacteria\_none | Bacteroidota | Bacteroidia | Bacteroidales | Prevotellaceae | Hoylesella | Bacteria;Bacteria\_none;Bacteroidota;Bacteroidia;Bacteroidales;Prevotellaceae;Hoylesella;Hoylesella buccalis |
| Hoylesella enoeca | 0 | 4 | 3 | 2 | 0 | 5 | 1 | 10 | 1 | 3 | 10 | 6 | 3 | 48 | Bacteria | Bacteria\_none | Bacteroidota | Bacteroidia | Bacteroidales | Prevotellaceae | Hoylesella | Bacteria;Bacteria\_none;Bacteroidota;Bacteroidia;Bacteroidales;Prevotellaceae;Hoylesella;Hoylesella enoeca |
| Prevotella bivia | 4 | 3 | 1 | 5 | 0 | 0 | 1 | 1 | 0 | 0 | 1 | 0 | 2 | 18 | Bacteria | Bacteria\_none | Bacteroidota | Bacteroidia | Bacteroidales | Prevotellaceae | Prevotella | Bacteria;Bacteria\_none;Bacteroidota;Bacteroidia;Bacteroidales;Prevotellaceae;Prevotella;Prevotella bivia |
| Prevotella communis | 0 | 0 | 0 | 1 | 0 | 1 | 0 | 0 | 0 | 0 | 0 | 0 | 0 | 2 | Bacteria | Bacteria\_none | Bacteroidota | Bacteroidia | Bacteroidales | Prevotellaceae | Prevotella | Bacteria;Bacteria\_none;Bacteroidota;Bacteroidia;Bacteroidales;Prevotellaceae;Prevotella;Prevotella communis |
| Prevotella corporis | 3 | 2 | 2 | 7 | 0 | 0 | 3 | 0 | 0 | 0 | 1 | 0 | 8 | 26 | Bacteria | Bacteria\_none | Bacteroidota | Bacteroidia | Bacteroidales | Prevotellaceae | Prevotella | Bacteria;Bacteria\_none;Bacteroidota;Bacteroidia;Bacteroidales;Prevotellaceae;Prevotella;Prevotella corporis |
| Prevotella dentalis | 0 | 1 | 0 | 2 | 0 | 0 | 1 | 0 | 0 | 0 | 0 | 0 | 0 | 4 | Bacteria | Bacteria\_none | Bacteroidota | Bacteroidia | Bacteroidales | Prevotellaceae | Prevotella | Bacteria;Bacteria\_none;Bacteroidota;Bacteroidia;Bacteroidales;Prevotellaceae;Prevotella;Prevotella dentalis |
| Prevotella denticola | 3 | 13 | 7 | 16 | 3 | 2 | 2 | 5 | 1 | 1 | 12 | 12 | 19 | 96 | Bacteria | Bacteria\_none | Bacteroidota | Bacteroidia | Bacteroidales | Prevotellaceae | Prevotella | Bacteria;Bacteria\_none;Bacteroidota;Bacteroidia;Bacteroidales;Prevotellaceae;Prevotella;Prevotella denticola |
| Prevotella fusca | 4 | 2 | 0 | 5 | 0 | 0 | 1 | 9 | 0 | 0 | 0 | 2 | 5 | 28 | Bacteria | Bacteria\_none | Bacteroidota | Bacteroidia | Bacteroidales | Prevotellaceae | Prevotella | Bacteria;Bacteria\_none;Bacteroidota;Bacteroidia;Bacteroidales;Prevotellaceae;Prevotella;Prevotella fusca |
| Prevotella herbatica | 0 | 1 | 0 | 0 | 0 | 0 | 0 | 0 | 0 | 0 | 0 | 0 | 0 | 1 | Bacteria | Bacteria\_none | Bacteroidota | Bacteroidia | Bacteroidales | Prevotellaceae | Prevotella | Bacteria;Bacteria\_none;Bacteroidota;Bacteroidia;Bacteroidales;Prevotellaceae;Prevotella;Prevotella herbatica |
| Prevotella histicola | 0 | 1 | 2 | 7 | 0 | 3 | 1 | 0 | 0 | 0 | 0 | 10 | 4 | 28 | Bacteria | Bacteria\_none | Bacteroidota | Bacteroidia | Bacteroidales | Prevotellaceae | Prevotella | Bacteria;Bacteria\_none;Bacteroidota;Bacteroidia;Bacteroidales;Prevotellaceae;Prevotella;Prevotella histicola |
| Prevotella intermedia | 11 | 33 | 14 | 65 | 2 | 5 | 7 | 6 | 2 | 5 | 14 | 35 | 36 | 235 | Bacteria | Bacteria\_none | Bacteroidota | Bacteroidia | Bacteroidales | Prevotellaceae | Prevotella | Bacteria;Bacteria\_none;Bacteroidota;Bacteroidia;Bacteroidales;Prevotellaceae;Prevotella;Prevotella intermedia |
| Prevotella jejuni | 48 | 124 | 7 | 27 | 2 | 52 | 77 | 1 | 267 | 4 | 17 | 4 | 184 | 814 | Bacteria | Bacteria\_none | Bacteroidota | Bacteroidia | Bacteroidales | Prevotellaceae | Prevotella | Bacteria;Bacteria\_none;Bacteroidota;Bacteroidia;Bacteroidales;Prevotellaceae;Prevotella;Prevotella jejuni |
| Prevotella melaninogenica | 19 | 677 | 37 | 164 | 44 | 124 | 28 | 10 | 54 | 83 | 105 | 169 | 408 | 1922 | Bacteria | Bacteria\_none | Bacteroidota | Bacteroidia | Bacteroidales | Prevotellaceae | Prevotella | Bacteria;Bacteria\_none;Bacteroidota;Bacteroidia;Bacteroidales;Prevotellaceae;Prevotella;Prevotella melaninogenica |
| Prevotella multiformis | 0 | 3 | 1 | 2 | 0 | 1 | 1 | 2 | 0 | 1 | 2 | 0 | 1 | 14 | Bacteria | Bacteria\_none | Bacteroidota | Bacteroidia | Bacteroidales | Prevotellaceae | Prevotella | Bacteria;Bacteria\_none;Bacteroidota;Bacteroidia;Bacteroidales;Prevotellaceae;Prevotella;Prevotella multiformis |
| Prevotella nigrescens | 7 | 36 | 50 | 143 | 7 | 3 | 10 | 1 | 1 | 4 | 13 | 48 | 82 | 405 | Bacteria | Bacteria\_none | Bacteroidota | Bacteroidia | Bacteroidales | Prevotellaceae | Prevotella | Bacteria;Bacteria\_none;Bacteroidota;Bacteroidia;Bacteroidales;Prevotellaceae;Prevotella;Prevotella nigrescens |
| Prevotella scopos | 10 | 7 | 0 | 1 | 0 | 2 | 17 | 0 | 6 | 0 | 0 | 2 | 23 | 68 | Bacteria | Bacteria\_none | Bacteroidota | Bacteroidia | Bacteroidales | Prevotellaceae | Prevotella | Bacteria;Bacteria\_none;Bacteroidota;Bacteroidia;Bacteroidales;Prevotellaceae;Prevotella;Prevotella scopos |
| Prevotella sp. E15-22 | 0 | 0 | 0 | 0 | 0 | 1 | 0 | 0 | 0 | 0 | 0 | 0 | 0 | 1 | Bacteria | Bacteria\_none | Bacteroidota | Bacteroidia | Bacteroidales | Prevotellaceae | Prevotella | Bacteria;Bacteria\_none;Bacteroidota;Bacteroidia;Bacteroidales;Prevotellaceae;Prevotella;Prevotella sp. E15-22 |
| Prevotella sp. E2-28 | 0 | 0 | 0 | 2 | 0 | 0 | 0 | 0 | 0 | 0 | 0 | 0 | 0 | 2 | Bacteria | Bacteria\_none | Bacteroidota | Bacteroidia | Bacteroidales | Prevotellaceae | Prevotella | Bacteria;Bacteria\_none;Bacteroidota;Bacteroidia;Bacteroidales;Prevotellaceae;Prevotella;Prevotella sp. E2-28 |
| Prevotella sp. Rep29 | 0 | 0 | 0 | 0 | 0 | 0 | 0 | 0 | 0 | 0 | 0 | 0 | 1 | 1 | Bacteria | Bacteria\_none | Bacteroidota | Bacteroidia | Bacteroidales | Prevotellaceae | Prevotella | Bacteria;Bacteria\_none;Bacteroidota;Bacteroidia;Bacteroidales;Prevotellaceae;Prevotella;Prevotella sp. Rep29 |
| Prevotella sp. oral taxon 299 | 225 | 15 | 146 | 393 | 16 | 2 | 40 | 0 | 3 | 25 | 0 | 1 | 212 | 1078 | Bacteria | Bacteria\_none | Bacteroidota | Bacteroidia | Bacteroidales | Prevotellaceae | Prevotella | Bacteria;Bacteria\_none;Bacteroidota;Bacteroidia;Bacteroidales;Prevotellaceae;Prevotella;Prevotella sp. oral taxon 299 |
| Prevotella sp. oral taxon 313 | 0 | 0 | 1 | 0 | 0 | 0 | 0 | 0 | 0 | 0 | 0 | 0 | 0 | 1 | Bacteria | Bacteria\_none | Bacteroidota | Bacteroidia | Bacteroidales | Prevotellaceae | Prevotella | Bacteria;Bacteria\_none;Bacteroidota;Bacteroidia;Bacteroidales;Prevotellaceae;Prevotella;Prevotella sp. oral taxon 313 |
| Prevotella sp. oral taxon 475 | 0 | 1 | 1 | 0 | 3 | 1 | 0 | 0 | 0 | 2 | 6 | 2 | 6 | 22 | Bacteria | Bacteria\_none | Bacteroidota | Bacteroidia | Bacteroidales | Prevotellaceae | Prevotella | Bacteria;Bacteria\_none;Bacteroidota;Bacteroidia;Bacteroidales;Prevotellaceae;Prevotella;Prevotella sp. oral taxon 475 |
| Prevotella veroralis | 0 | 52 | 2 | 34 | 1 | 34 | 1 | 1 | 6 | 0 | 0 | 18 | 19 | 168 | Bacteria | Bacteria\_none | Bacteroidota | Bacteroidia | Bacteroidales | Prevotellaceae | Prevotella | Bacteria;Bacteria\_none;Bacteroidota;Bacteroidia;Bacteroidales;Prevotellaceae;Prevotella;Prevotella veroralis |
| Segatella copri | 0 | 1 | 0 | 0 | 0 | 1 | 1 | 0 | 0 | 0 | 0 | 0 | 1 | 4 | Bacteria | Bacteria\_none | Bacteroidota | Bacteroidia | Bacteroidales | Prevotellaceae | Segatella | Bacteria;Bacteria\_none;Bacteroidota;Bacteroidia;Bacteroidales;Prevotellaceae;Segatella;Segatella copri |
| Segatella oris | 1 | 5 | 13 | 12 | 6 | 3 | 11 | 16 | 0 | 14 | 46 | 38 | 28 | 193 | Bacteria | Bacteria\_none | Bacteroidota | Bacteroidia | Bacteroidales | Prevotellaceae | Segatella | Bacteria;Bacteria\_none;Bacteroidota;Bacteroidia;Bacteroidales;Prevotellaceae;Segatella;Segatella oris |
| Xylanibacter ruminicola | 0 | 0 | 0 | 0 | 0 | 0 | 1 | 0 | 0 | 0 | 0 | 0 | 1 | 2 | Bacteria | Bacteria\_none | Bacteroidota | Bacteroidia | Bacteroidales | Prevotellaceae | Xylanibacter | Bacteria;Bacteria\_none;Bacteroidota;Bacteroidia;Bacteroidales;Prevotellaceae;Xylanibacter;Xylanibacter ruminicola |
| Alistipes communis | 0 | 0 | 0 | 0 | 0 | 1 | 0 | 0 | 0 | 0 | 0 | 0 | 0 | 1 | Bacteria | Bacteria\_none | Bacteroidota | Bacteroidia | Bacteroidales | Rikenellaceae | Alistipes | Bacteria;Bacteria\_none;Bacteroidota;Bacteroidia;Bacteroidales;Rikenellaceae;Alistipes;Alistipes communis |
| Alistipes finegoldii | 0 | 1 | 0 | 0 | 0 | 1 | 0 | 0 | 0 | 0 | 0 | 0 | 0 | 2 | Bacteria | Bacteria\_none | Bacteroidota | Bacteroidia | Bacteroidales | Rikenellaceae | Alistipes | Bacteria;Bacteria\_none;Bacteroidota;Bacteroidia;Bacteroidales;Rikenellaceae;Alistipes;Alistipes finegoldii |
| Alistipes onderdonkii | 0 | 1 | 0 | 0 | 0 | 0 | 0 | 0 | 0 | 0 | 0 | 0 | 0 | 1 | Bacteria | Bacteria\_none | Bacteroidota | Bacteroidia | Bacteroidales | Rikenellaceae | Alistipes | Bacteria;Bacteria\_none;Bacteroidota;Bacteroidia;Bacteroidales;Rikenellaceae;Alistipes;Alistipes onderdonkii |
| Alistipes shahii | 0 | 0 | 0 | 0 | 0 | 0 | 0 | 0 | 0 | 0 | 0 | 0 | 1 | 1 | Bacteria | Bacteria\_none | Bacteroidota | Bacteroidia | Bacteroidales | Rikenellaceae | Alistipes | Bacteria;Bacteria\_none;Bacteroidota;Bacteroidia;Bacteroidales;Rikenellaceae;Alistipes;Alistipes shahii |
| Alistipes sp. dk3624 | 0 | 0 | 0 | 0 | 0 | 0 | 0 | 0 | 0 | 1 | 0 | 0 | 0 | 1 | Bacteria | Bacteria\_none | Bacteroidota | Bacteroidia | Bacteroidales | Rikenellaceae | Alistipes | Bacteria;Bacteria\_none;Bacteroidota;Bacteroidia;Bacteroidales;Rikenellaceae;Alistipes;Alistipes sp. dk3624 |
| Salinivirga cyanobacteriivorans | 0 | 0 | 0 | 1 | 0 | 0 | 0 | 0 | 0 | 0 | 0 | 0 | 1 | 2 | Bacteria | Bacteria\_none | Bacteroidota | Bacteroidia | Bacteroidales | Salinivirgaceae | Salinivirga | Bacteria;Bacteria\_none;Bacteroidota;Bacteroidia;Bacteroidales;Salinivirgaceae;Salinivirga;Salinivirga cyanobacteriivorans |
| Parabacteroides chongii | 0 | 0 | 0 | 0 | 0 | 0 | 0 | 0 | 0 | 0 | 0 | 0 | 1 | 1 | Bacteria | Bacteria\_none | Bacteroidota | Bacteroidia | Bacteroidales | Tannerellaceae | Parabacteroides | Bacteria;Bacteria\_none;Bacteroidota;Bacteroidia;Bacteroidales;Tannerellaceae;Parabacteroides;Parabacteroides chongii |
| Parabacteroides distasonis | 0 | 0 | 0 | 0 | 0 | 0 | 0 | 1 | 0 | 0 | 0 | 0 | 0 | 1 | Bacteria | Bacteria\_none | Bacteroidota | Bacteroidia | Bacteroidales | Tannerellaceae | Parabacteroides | Bacteria;Bacteria\_none;Bacteroidota;Bacteroidia;Bacteroidales;Tannerellaceae;Parabacteroides;Parabacteroides distasonis |
| Parabacteroides faecis | 0 | 0 | 0 | 0 | 0 | 0 | 0 | 0 | 0 | 0 | 0 | 0 | 1 | 1 | Bacteria | Bacteria\_none | Bacteroidota | Bacteroidia | Bacteroidales | Tannerellaceae | Parabacteroides | Bacteria;Bacteria\_none;Bacteroidota;Bacteroidia;Bacteroidales;Tannerellaceae;Parabacteroides;Parabacteroides faecis |
| Parabacteroides goldsteinii | 0 | 0 | 0 | 0 | 0 | 0 | 0 | 0 | 0 | 0 | 1 | 0 | 0 | 1 | Bacteria | Bacteria\_none | Bacteroidota | Bacteroidia | Bacteroidales | Tannerellaceae | Parabacteroides | Bacteria;Bacteria\_none;Bacteroidota;Bacteroidia;Bacteroidales;Tannerellaceae;Parabacteroides;Parabacteroides goldsteinii |
| Tannerella forsythia | 2 | 19 | 28 | 8 | 9 | 15 | 5 | 53 | 0 | 22 | 24 | 20 | 69 | 274 | Bacteria | Bacteria\_none | Bacteroidota | Bacteroidia | Bacteroidales | Tannerellaceae | Tannerella | Bacteria;Bacteria\_none;Bacteroidota;Bacteroidia;Bacteroidales;Tannerellaceae;Tannerella;Tannerella forsythia |
| Tannerella serpentiformis | 0 | 18 | 6 | 5 | 10 | 88 | 0 | 0 | 0 | 22 | 22 | 4 | 31 | 206 | Bacteria | Bacteria\_none | Bacteroidota | Bacteroidia | Bacteroidales | Tannerellaceae | Tannerella | Bacteria;Bacteria\_none;Bacteroidota;Bacteroidia;Bacteroidales;Tannerellaceae;Tannerella;Tannerella serpentiformis |
| Marinifilum fragile | 0 | 0 | 1 | 0 | 0 | 0 | 0 | 0 | 0 | 0 | 0 | 0 | 0 | 1 | Bacteria | Bacteria\_none | Bacteroidota | Bacteroidia | Marinilabiliales | Marinifilaceae | Marinifilum | Bacteria;Bacteria\_none;Bacteroidota;Bacteroidia;Marinilabiliales;Marinifilaceae;Marinifilum;Marinifilum fragile |
| Marinifilum\_uncultured | 1 | 0 | 0 | 0 | 0 | 0 | 0 | 0 | 0 | 0 | 0 | 0 | 0 | 1 | Bacteria | Bacteria\_none | Bacteroidota | Bacteroidia | Marinilabiliales | Marinifilaceae | Marinifilum | Bacteria;Bacteria\_none;Bacteroidota;Bacteroidia;Marinilabiliales;Marinifilaceae;Marinifilum;Marinifilum\_uncultured |
| Alkalitalea saponilacus | 0 | 0 | 0 | 0 | 0 | 0 | 0 | 1 | 0 | 0 | 0 | 0 | 0 | 1 | Bacteria | Bacteria\_none | Bacteroidota | Bacteroidia | Marinilabiliales | Marinilabiliaceae | Alkalitalea | Bacteria;Bacteria\_none;Bacteroidota;Bacteroidia;Marinilabiliales;Marinilabiliaceae;Alkalitalea;Alkalitalea saponilacus |
| Carboxylicivirga\_uncultured | 0 | 0 | 1 | 1 | 0 | 0 | 0 | 1 | 0 | 0 | 0 | 0 | 0 | 3 | Bacteria | Bacteria\_none | Bacteroidota | Bacteroidia | Marinilabiliales | Marinilabiliaceae | Carboxylicivirga | Bacteria;Bacteria\_none;Bacteroidota;Bacteroidia;Marinilabiliales;Marinilabiliaceae;Carboxylicivirga;Carboxylicivirga\_uncultured |
| Draconibacterium\_uncultured | 0 | 0 | 0 | 1 | 0 | 0 | 0 | 0 | 0 | 0 | 2 | 0 | 0 | 3 | Bacteria | Bacteria\_none | Bacteroidota | Bacteroidia | Marinilabiliales | Prolixibacteraceae | Draconibacterium | Bacteria;Bacteria\_none;Bacteroidota;Bacteroidia;Marinilabiliales;Prolixibacteraceae;Draconibacterium;Draconibacterium\_uncultured |
| Mangrovibacterium marinum | 0 | 0 | 0 | 0 | 0 | 1 | 0 | 0 | 0 | 0 | 0 | 0 | 0 | 1 | Bacteria | Bacteria\_none | Bacteroidota | Bacteroidia | Marinilabiliales | Prolixibacteraceae | Mangrovibacterium | Bacteria;Bacteria\_none;Bacteroidota;Bacteroidia;Marinilabiliales;Prolixibacteraceae;Mangrovibacterium;Mangrovibacterium marinum |
| Maribellus comscasis | 0 | 0 | 0 | 1 | 0 | 0 | 0 | 0 | 0 | 0 | 0 | 0 | 0 | 1 | Bacteria | Bacteria\_none | Bacteroidota | Bacteroidia | Marinilabiliales | Prolixibacteraceae | Maribellus | Bacteria;Bacteria\_none;Bacteroidota;Bacteroidia;Marinilabiliales;Prolixibacteraceae;Maribellus;Maribellus comscasis |
| Chitinophaga agri | 0 | 0 | 0 | 0 | 0 | 0 | 0 | 0 | 0 | 1 | 0 | 0 | 0 | 1 | Bacteria | Bacteria\_none | Bacteroidota | Chitinophagia | Chitinophagales | Chitinophagaceae | Chitinophaga | Bacteria;Bacteria\_none;Bacteroidota;Chitinophagia;Chitinophagales;Chitinophagaceae;Chitinophaga;Chitinophaga agri |
| Chitinophaga pollutisoli | 0 | 0 | 0 | 0 | 1 | 0 | 0 | 0 | 0 | 0 | 0 | 0 | 0 | 1 | Bacteria | Bacteria\_none | Bacteroidota | Chitinophagia | Chitinophagales | Chitinophagaceae | Chitinophaga | Bacteria;Bacteria\_none;Bacteroidota;Chitinophagia;Chitinophagales;Chitinophagaceae;Chitinophaga;Chitinophaga pollutisoli |
| Chitinophaga sancti | 0 | 0 | 0 | 0 | 1 | 0 | 0 | 0 | 0 | 0 | 0 | 0 | 0 | 1 | Bacteria | Bacteria\_none | Bacteroidota | Chitinophagia | Chitinophagales | Chitinophagaceae | Chitinophaga | Bacteria;Bacteria\_none;Bacteroidota;Chitinophagia;Chitinophagales;Chitinophagaceae;Chitinophaga;Chitinophaga sancti |
| Chitinophaga sp. HK235 | 0 | 1 | 0 | 0 | 0 | 0 | 0 | 0 | 0 | 0 | 0 | 0 | 0 | 1 | Bacteria | Bacteria\_none | Bacteroidota | Chitinophagia | Chitinophagales | Chitinophagaceae | Chitinophaga | Bacteria;Bacteria\_none;Bacteroidota;Chitinophagia;Chitinophagales;Chitinophagaceae;Chitinophaga;Chitinophaga sp. HK235 |
| Chitinophaga sp. XS-30 | 0 | 0 | 0 | 1 | 0 | 0 | 0 | 0 | 0 | 0 | 0 | 0 | 0 | 1 | Bacteria | Bacteria\_none | Bacteroidota | Chitinophagia | Chitinophagales | Chitinophagaceae | Chitinophaga | Bacteria;Bacteria\_none;Bacteroidota;Chitinophagia;Chitinophagales;Chitinophagaceae;Chitinophaga;Chitinophaga sp. XS-30 |
| Ferruginibacter lapsinanis | 0 | 0 | 0 | 0 | 0 | 1 | 0 | 0 | 0 | 0 | 0 | 0 | 0 | 1 | Bacteria | Bacteria\_none | Bacteroidota | Chitinophagia | Chitinophagales | Chitinophagaceae | Ferruginibacter | Bacteria;Bacteria\_none;Bacteroidota;Chitinophagia;Chitinophagales;Chitinophagaceae;Ferruginibacter;Ferruginibacter lapsinanis |
| Flavihumibacter fluvii | 0 | 0 | 0 | 1 | 0 | 0 | 0 | 0 | 0 | 0 | 0 | 0 | 0 | 1 | Bacteria | Bacteria\_none | Bacteroidota | Chitinophagia | Chitinophagales | Chitinophagaceae | Flavihumibacter | Bacteria;Bacteria\_none;Bacteroidota;Chitinophagia;Chitinophagales;Chitinophagaceae;Flavihumibacter;Flavihumibacter fluvii |
| Pseudobacter ginsenosidimutans | 0 | 0 | 0 | 0 | 0 | 0 | 0 | 0 | 0 | 0 | 0 | 1 | 0 | 1 | Bacteria | Bacteria\_none | Bacteroidota | Chitinophagia | Chitinophagales | Chitinophagaceae | Pseudobacter | Bacteria;Bacteria\_none;Bacteroidota;Chitinophagia;Chitinophagales;Chitinophagaceae;Pseudobacter;Pseudobacter ginsenosidimutans |
| Cardinium endosymbiont of Tipula unca | 0 | 0 | 0 | 0 | 0 | 0 | 0 | 0 | 0 | 0 | 1 | 0 | 0 | 1 | Bacteria | Bacteria\_none | Bacteroidota | Cytophagia | Cytophagales | Amoebophilaceae | Candidatus Cardinium | Bacteria;Bacteria\_none;Bacteroidota;Cytophagia;Cytophagales;Amoebophilaceae;Candidatus Cardinium;Cardinium endosymbiont of Tipula unca |
| Algoriphagus sanaruensis | 0 | 0 | 0 | 0 | 0 | 0 | 0 | 0 | 0 | 0 | 1 | 0 | 0 | 1 | Bacteria | Bacteria\_none | Bacteroidota | Cytophagia | Cytophagales | Cyclobacteriaceae | Algoriphagus | Bacteria;Bacteria\_none;Bacteroidota;Cytophagia;Cytophagales;Cyclobacteriaceae;Algoriphagus;Algoriphagus sanaruensis |
| Cyclobacterium amurskyense | 0 | 0 | 0 | 1 | 0 | 0 | 0 | 0 | 0 | 0 | 0 | 0 | 0 | 1 | Bacteria | Bacteria\_none | Bacteroidota | Cytophagia | Cytophagales | Cyclobacteriaceae | Cyclobacterium | Bacteria;Bacteria\_none;Bacteroidota;Cytophagia;Cytophagales;Cyclobacteriaceae;Cyclobacterium;Cyclobacterium amurskyense |
| Cyclobacterium marinum | 0 | 0 | 0 | 0 | 0 | 0 | 0 | 0 | 0 | 0 | 0 | 0 | 1 | 1 | Bacteria | Bacteria\_none | Bacteroidota | Cytophagia | Cytophagales | Cyclobacteriaceae | Cyclobacterium | Bacteria;Bacteria\_none;Bacteroidota;Cytophagia;Cytophagales;Cyclobacteriaceae;Cyclobacterium;Cyclobacterium marinum |
| Spirosoma sp. KCTC 42546 | 0 | 0 | 0 | 0 | 0 | 0 | 0 | 1 | 0 | 0 | 0 | 0 | 0 | 1 | Bacteria | Bacteria\_none | Bacteroidota | Cytophagia | Cytophagales | Cytophagaceae | Spirosoma | Bacteria;Bacteria\_none;Bacteroidota;Cytophagia;Cytophagales;Cytophagaceae;Spirosoma;Spirosoma sp. KCTC 42546 |
| Flammeovirga pectinis | 0 | 0 | 0 | 0 | 2 | 0 | 0 | 0 | 0 | 0 | 0 | 0 | 0 | 2 | Bacteria | Bacteria\_none | Bacteroidota | Cytophagia | Cytophagales | Flammeovirgaceae | Flammeovirga | Bacteria;Bacteria\_none;Bacteroidota;Cytophagia;Cytophagales;Flammeovirgaceae;Flammeovirga;Flammeovirga pectinis |
| Hymenobacter jejuensis | 0 | 0 | 0 | 0 | 1 | 0 | 0 | 0 | 0 | 0 | 0 | 0 | 0 | 1 | Bacteria | Bacteria\_none | Bacteroidota | Cytophagia | Cytophagales | Hymenobacteraceae | Hymenobacter | Bacteria;Bacteria\_none;Bacteroidota;Cytophagia;Cytophagales;Hymenobacteraceae;Hymenobacter;Hymenobacter jejuensis |
| Hymenobacter sp. BRD128 | 0 | 0 | 0 | 0 | 1 | 0 | 0 | 0 | 0 | 0 | 0 | 0 | 0 | 1 | Bacteria | Bacteria\_none | Bacteroidota | Cytophagia | Cytophagales | Hymenobacteraceae | Hymenobacter | Bacteria;Bacteria\_none;Bacteroidota;Cytophagia;Cytophagales;Hymenobacteraceae;Hymenobacter;Hymenobacter sp. BRD128 |
| Hymenobacter sp. BT18 | 0 | 0 | 0 | 0 | 0 | 0 | 0 | 0 | 0 | 0 | 0 | 0 | 1 | 1 | Bacteria | Bacteria\_none | Bacteroidota | Cytophagia | Cytophagales | Hymenobacteraceae | Hymenobacter | Bacteria;Bacteria\_none;Bacteroidota;Cytophagia;Cytophagales;Hymenobacteraceae;Hymenobacter;Hymenobacter sp. BT18 |
| Hymenobacter sp. YIM 151500-1 | 0 | 0 | 0 | 0 | 0 | 0 | 0 | 0 | 0 | 0 | 0 | 0 | 1 | 1 | Bacteria | Bacteria\_none | Bacteroidota | Cytophagia | Cytophagales | Hymenobacteraceae | Hymenobacter | Bacteria;Bacteria\_none;Bacteroidota;Cytophagia;Cytophagales;Hymenobacteraceae;Hymenobacter;Hymenobacter sp. YIM 151500-1 |
| Hymenobacter swuensis | 0 | 0 | 0 | 0 | 0 | 0 | 1 | 0 | 0 | 0 | 0 | 0 | 0 | 1 | Bacteria | Bacteria\_none | Bacteroidota | Cytophagia | Cytophagales | Hymenobacteraceae | Hymenobacter | Bacteria;Bacteria\_none;Bacteroidota;Cytophagia;Cytophagales;Hymenobacteraceae;Hymenobacter;Hymenobacter swuensis |
| Hymenobacter volaticus | 0 | 0 | 0 | 0 | 0 | 0 | 0 | 0 | 0 | 0 | 0 | 0 | 1 | 1 | Bacteria | Bacteria\_none | Bacteroidota | Cytophagia | Cytophagales | Hymenobacteraceae | Hymenobacter | Bacteria;Bacteria\_none;Bacteroidota;Cytophagia;Cytophagales;Hymenobacteraceae;Hymenobacter;Hymenobacter volaticus |
| Hymenobacter yonginensis | 0 | 0 | 0 | 1 | 0 | 0 | 0 | 0 | 0 | 0 | 0 | 0 | 0 | 1 | Bacteria | Bacteria\_none | Bacteroidota | Cytophagia | Cytophagales | Hymenobacteraceae | Hymenobacter | Bacteria;Bacteria\_none;Bacteroidota;Cytophagia;Cytophagales;Hymenobacteraceae;Hymenobacter;Hymenobacter yonginensis |
| Rufibacter radiotolerans | 0 | 0 | 0 | 0 | 0 | 0 | 0 | 0 | 0 | 1 | 0 | 0 | 0 | 1 | Bacteria | Bacteria\_none | Bacteroidota | Cytophagia | Cytophagales | Hymenobacteraceae | Rufibacter | Bacteria;Bacteria\_none;Bacteroidota;Cytophagia;Cytophagales;Hymenobacteraceae;Rufibacter;Rufibacter radiotolerans |
| Arcticibacterium luteifluviistationis | 0 | 0 | 1 | 0 | 0 | 0 | 0 | 0 | 0 | 0 | 0 | 0 | 0 | 1 | Bacteria | Bacteria\_none | Bacteroidota | Cytophagia | Cytophagales | Leadbetterellaceae | Arcticibacterium | Bacteria;Bacteria\_none;Bacteroidota;Cytophagia;Cytophagales;Leadbetterellaceae;Arcticibacterium;Arcticibacterium luteifluviistationis |
| Emticicia oligotrophica | 1 | 0 | 0 | 0 | 0 | 0 | 0 | 0 | 0 | 0 | 0 | 0 | 0 | 1 | Bacteria | Bacteria\_none | Bacteroidota | Cytophagia | Cytophagales | Leadbetterellaceae | Emticicia | Bacteria;Bacteria\_none;Bacteroidota;Cytophagia;Cytophagales;Leadbetterellaceae;Emticicia;Emticicia oligotrophica |
| Marivirga salinae | 0 | 0 | 0 | 0 | 0 | 1 | 0 | 0 | 0 | 0 | 0 | 0 | 1 | 2 | Bacteria | Bacteria\_none | Bacteroidota | Cytophagia | Cytophagales | Marivirgaceae | Marivirga | Bacteria;Bacteria\_none;Bacteroidota;Cytophagia;Cytophagales;Marivirgaceae;Marivirga;Marivirga salinae |
| Fulvitalea axinellae | 0 | 0 | 0 | 1 | 1 | 0 | 0 | 0 | 0 | 0 | 0 | 0 | 0 | 2 | Bacteria | Bacteria\_none | Bacteroidota | Cytophagia | Cytophagales | Persicobacteraceae | Fulvitalea | Bacteria;Bacteria\_none;Bacteroidota;Cytophagia;Cytophagales;Persicobacteraceae;Fulvitalea;Fulvitalea axinellae |
| Dyadobacter chenwenxiniae | 0 | 0 | 0 | 0 | 0 | 0 | 0 | 0 | 0 | 0 | 0 | 0 | 1 | 1 | Bacteria | Bacteria\_none | Bacteroidota | Cytophagia | Cytophagales | Spirosomataceae | Dyadobacter | Bacteria;Bacteria\_none;Bacteroidota;Cytophagia;Cytophagales;Spirosomataceae;Dyadobacter;Dyadobacter chenwenxiniae |
| Dyadobacter pollutisoli | 0 | 0 | 0 | 0 | 0 | 1 | 0 | 0 | 0 | 0 | 0 | 0 | 0 | 1 | Bacteria | Bacteria\_none | Bacteroidota | Cytophagia | Cytophagales | Spirosomataceae | Dyadobacter | Bacteria;Bacteria\_none;Bacteroidota;Cytophagia;Cytophagales;Spirosomataceae;Dyadobacter;Dyadobacter pollutisoli |
| Dyadobacter sp. NIV53 | 0 | 0 | 0 | 0 | 0 | 0 | 0 | 0 | 0 | 0 | 0 | 0 | 1 | 1 | Bacteria | Bacteria\_none | Bacteroidota | Cytophagia | Cytophagales | Spirosomataceae | Dyadobacter | Bacteria;Bacteria\_none;Bacteroidota;Cytophagia;Cytophagales;Spirosomataceae;Dyadobacter;Dyadobacter sp. NIV53 |
| Fibrella aestuarina | 0 | 0 | 0 | 0 | 0 | 0 | 0 | 0 | 0 | 0 | 0 | 0 | 1 | 1 | Bacteria | Bacteria\_none | Bacteroidota | Cytophagia | Cytophagales | Spirosomataceae | Fibrella | Bacteria;Bacteria\_none;Bacteroidota;Cytophagia;Cytophagales;Spirosomataceae;Fibrella;Fibrella aestuarina |
| Blattabacterium cuenoti | 0 | 0 | 0 | 0 | 0 | 0 | 0 | 0 | 0 | 0 | 1 | 0 | 1 | 2 | Bacteria | Bacteria\_none | Bacteroidota | Flavobacteriia | Flavobacteriales | Blattabacteriaceae | Blattabacterium | Bacteria;Bacteria\_none;Bacteroidota;Flavobacteriia;Flavobacteriales;Blattabacteriaceae;Blattabacterium;Blattabacterium cuenoti |
| Paracrocinitomix mangrovi | 0 | 0 | 0 | 0 | 0 | 0 | 0 | 0 | 0 | 1 | 0 | 0 | 0 | 1 | Bacteria | Bacteria\_none | Bacteroidota | Flavobacteriia | Flavobacteriales | Crocinitomicaceae | Paracrocinitomix | Bacteria;Bacteria\_none;Bacteroidota;Flavobacteriia;Flavobacteriales;Crocinitomicaceae;Paracrocinitomix;Paracrocinitomix mangrovi |
| Aequorivita marisscotiae | 0 | 0 | 0 | 2 | 0 | 0 | 0 | 0 | 0 | 0 | 0 | 0 | 0 | 2 | Bacteria | Bacteria\_none | Bacteroidota | Flavobacteriia | Flavobacteriales | Flavobacteriaceae | Aequorivita | Bacteria;Bacteria\_none;Bacteroidota;Flavobacteriia;Flavobacteriales;Flavobacteriaceae;Aequorivita;Aequorivita marisscotiae |
| Algibacter luteus | 0 | 0 | 0 | 0 | 0 | 0 | 0 | 0 | 0 | 0 | 0 | 1 | 0 | 1 | Bacteria | Bacteria\_none | Bacteroidota | Flavobacteriia | Flavobacteriales | Flavobacteriaceae | Algibacter | Bacteria;Bacteria\_none;Bacteroidota;Flavobacteriia;Flavobacteriales;Flavobacteriaceae;Algibacter;Algibacter luteus |
| Algibacter sp. L1A34 | 0 | 0 | 0 | 0 | 0 | 1 | 0 | 0 | 0 | 0 | 0 | 0 | 0 | 1 | Bacteria | Bacteria\_none | Bacteroidota | Flavobacteriia | Flavobacteriales | Flavobacteriaceae | Algibacter | Bacteria;Bacteria\_none;Bacteroidota;Flavobacteriia;Flavobacteriales;Flavobacteriaceae;Algibacter;Algibacter sp. L1A34 |
| Aquimarina sp. AD10 | 0 | 0 | 0 | 0 | 1 | 0 | 0 | 1 | 0 | 0 | 0 | 0 | 0 | 2 | Bacteria | Bacteria\_none | Bacteroidota | Flavobacteriia | Flavobacteriales | Flavobacteriaceae | Aquimarina | Bacteria;Bacteria\_none;Bacteroidota;Flavobacteriia;Flavobacteriales;Flavobacteriaceae;Aquimarina;Aquimarina sp. AD10 |
| Capnocytophaga canimorsus | 0 | 0 | 0 | 0 | 0 | 0 | 0 | 0 | 0 | 1 | 0 | 0 | 0 | 1 | Bacteria | Bacteria\_none | Bacteroidota | Flavobacteriia | Flavobacteriales | Flavobacteriaceae | Capnocytophaga | Bacteria;Bacteria\_none;Bacteroidota;Flavobacteriia;Flavobacteriales;Flavobacteriaceae;Capnocytophaga;Capnocytophaga canimorsus |
| Capnocytophaga endodontalis | 6 | 4 | 1 | 6 | 0 | 7 | 3 | 26 | 0 | 15 | 19 | 1 | 40 | 128 | Bacteria | Bacteria\_none | Bacteroidota | Flavobacteriia | Flavobacteriales | Flavobacteriaceae | Capnocytophaga | Bacteria;Bacteria\_none;Bacteroidota;Flavobacteriia;Flavobacteriales;Flavobacteriaceae;Capnocytophaga;Capnocytophaga endodontalis |
| Capnocytophaga gingivalis | 57 | 47 | 39 | 29 | 40 | 4 | 17 | 59 | 0 | 49 | 44 | 9 | 83 | 477 | Bacteria | Bacteria\_none | Bacteroidota | Flavobacteriia | Flavobacteriales | Flavobacteriaceae | Capnocytophaga | Bacteria;Bacteria\_none;Bacteroidota;Flavobacteriia;Flavobacteriales;Flavobacteriaceae;Capnocytophaga;Capnocytophaga gingivalis |
| Capnocytophaga haemolytica | 1 | 0 | 0 | 0 | 2 | 0 | 0 | 0 | 0 | 1 | 6 | 0 | 3 | 13 | Bacteria | Bacteria\_none | Bacteroidota | Flavobacteriia | Flavobacteriales | Flavobacteriaceae | Capnocytophaga | Bacteria;Bacteria\_none;Bacteroidota;Flavobacteriia;Flavobacteriales;Flavobacteriaceae;Capnocytophaga;Capnocytophaga haemolytica |
| Capnocytophaga leadbetteri | 61 | 29 | 13 | 74 | 14 | 4 | 3 | 29 | 0 | 80 | 42 | 13 | 72 | 434 | Bacteria | Bacteria\_none | Bacteroidota | Flavobacteriia | Flavobacteriales | Flavobacteriaceae | Capnocytophaga | Bacteria;Bacteria\_none;Bacteroidota;Flavobacteriia;Flavobacteriales;Flavobacteriaceae;Capnocytophaga;Capnocytophaga leadbetteri |
| Capnocytophaga ochracea | 1 | 8 | 12 | 6 | 10 | 8 | 3 | 14 | 0 | 15 | 8 | 8 | 32 | 125 | Bacteria | Bacteria\_none | Bacteroidota | Flavobacteriia | Flavobacteriales | Flavobacteriaceae | Capnocytophaga | Bacteria;Bacteria\_none;Bacteroidota;Flavobacteriia;Flavobacteriales;Flavobacteriaceae;Capnocytophaga;Capnocytophaga ochracea |
| Capnocytophaga sp. FDAARGOS\_737 | 53 | 38 | 8 | 52 | 30 | 4 | 2 | 25 | 0 | 129 | 49 | 14 | 89 | 493 | Bacteria | Bacteria\_none | Bacteroidota | Flavobacteriia | Flavobacteriales | Flavobacteriaceae | Capnocytophaga | Bacteria;Bacteria\_none;Bacteroidota;Flavobacteriia;Flavobacteriales;Flavobacteriaceae;Capnocytophaga;Capnocytophaga sp. FDAARGOS\_737 |
| Capnocytophaga sp. H4358 | 0 | 0 | 0 | 0 | 0 | 0 | 0 | 0 | 0 | 1 | 0 | 0 | 0 | 1 | Bacteria | Bacteria\_none | Bacteroidota | Flavobacteriia | Flavobacteriales | Flavobacteriaceae | Capnocytophaga | Bacteria;Bacteria\_none;Bacteroidota;Flavobacteriia;Flavobacteriales;Flavobacteriaceae;Capnocytophaga;Capnocytophaga sp. H4358 |
| Capnocytophaga sp. oral taxon 323 | 2 | 5 | 2 | 0 | 0 | 0 | 1 | 0 | 0 | 0 | 1 | 0 | 9 | 20 | Bacteria | Bacteria\_none | Bacteroidota | Flavobacteriia | Flavobacteriales | Flavobacteriaceae | Capnocytophaga | Bacteria;Bacteria\_none;Bacteroidota;Flavobacteriia;Flavobacteriales;Flavobacteriaceae;Capnocytophaga;Capnocytophaga sp. oral taxon 323 |
| Capnocytophaga sp. oral taxon 864 | 0 | 2 | 2 | 1 | 0 | 0 | 0 | 2 | 0 | 0 | 0 | 0 | 12 | 19 | Bacteria | Bacteria\_none | Bacteroidota | Flavobacteriia | Flavobacteriales | Flavobacteriaceae | Capnocytophaga | Bacteria;Bacteria\_none;Bacteroidota;Flavobacteriia;Flavobacteriales;Flavobacteriaceae;Capnocytophaga;Capnocytophaga sp. oral taxon 864 |
| Capnocytophaga sp. oral taxon 878 | 13 | 3 | 0 | 6 | 22 | 1 | 4 | 11 | 0 | 20 | 8 | 2 | 20 | 110 | Bacteria | Bacteria\_none | Bacteroidota | Flavobacteriia | Flavobacteriales | Flavobacteriaceae | Capnocytophaga | Bacteria;Bacteria\_none;Bacteroidota;Flavobacteriia;Flavobacteriales;Flavobacteriaceae;Capnocytophaga;Capnocytophaga sp. oral taxon 878 |
| Capnocytophaga sp. oral taxon 902 | 2 | 1 | 4 | 0 | 3 | 0 | 0 | 0 | 0 | 0 | 0 | 0 | 3 | 13 | Bacteria | Bacteria\_none | Bacteroidota | Flavobacteriia | Flavobacteriales | Flavobacteriaceae | Capnocytophaga | Bacteria;Bacteria\_none;Bacteroidota;Flavobacteriia;Flavobacteriales;Flavobacteriaceae;Capnocytophaga;Capnocytophaga sp. oral taxon 902 |
| Capnocytophaga sputigena | 115 | 9 | 47 | 30 | 35 | 2 | 49 | 210 | 0 | 40 | 32 | 5 | 198 | 772 | Bacteria | Bacteria\_none | Bacteroidota | Flavobacteriia | Flavobacteriales | Flavobacteriaceae | Capnocytophaga | Bacteria;Bacteria\_none;Bacteroidota;Flavobacteriia;Flavobacteriales;Flavobacteriaceae;Capnocytophaga;Capnocytophaga sputigena |
| Capnocytophaga stomatis | 0 | 0 | 0 | 0 | 1 | 0 | 0 | 0 | 0 | 1 | 0 | 0 | 0 | 2 | Bacteria | Bacteria\_none | Bacteroidota | Flavobacteriia | Flavobacteriales | Flavobacteriaceae | Capnocytophaga | Bacteria;Bacteria\_none;Bacteroidota;Flavobacteriia;Flavobacteriales;Flavobacteriaceae;Capnocytophaga;Capnocytophaga stomatis |
| Christiangramia flava | 0 | 0 | 0 | 0 | 0 | 0 | 0 | 0 | 0 | 0 | 1 | 0 | 0 | 1 | Bacteria | Bacteria\_none | Bacteroidota | Flavobacteriia | Flavobacteriales | Flavobacteriaceae | Christiangramia | Bacteria;Bacteria\_none;Bacteroidota;Flavobacteriia;Flavobacteriales;Flavobacteriaceae;Christiangramia;Christiangramia flava |
| Gramella sp. MT6 | 0 | 0 | 0 | 0 | 0 | 0 | 0 | 0 | 0 | 0 | 0 | 0 | 1 | 1 | Bacteria | Bacteria\_none | Bacteroidota | Flavobacteriia | Flavobacteriales | Flavobacteriaceae | Christiangramia | Bacteria;Bacteria\_none;Bacteroidota;Flavobacteriia;Flavobacteriales;Flavobacteriaceae;Christiangramia;Gramella sp. MT6 |
| Costertonia aggregata | 0 | 0 | 0 | 0 | 0 | 0 | 0 | 0 | 0 | 0 | 1 | 0 | 0 | 1 | Bacteria | Bacteria\_none | Bacteroidota | Flavobacteriia | Flavobacteriales | Flavobacteriaceae | Costertonia | Bacteria;Bacteria\_none;Bacteroidota;Flavobacteriia;Flavobacteriales;Flavobacteriaceae;Costertonia;Costertonia aggregata |
| Dokdonia donghaensis | 0 | 1 | 0 | 0 | 0 | 0 | 0 | 0 | 0 | 0 | 0 | 0 | 0 | 1 | Bacteria | Bacteria\_none | Bacteroidota | Flavobacteriia | Flavobacteriales | Flavobacteriaceae | Dokdonia | Bacteria;Bacteria\_none;Bacteroidota;Flavobacteriia;Flavobacteriales;Flavobacteriaceae;Dokdonia;Dokdonia donghaensis |
| Dokdonia sp. Dokd-P16 | 0 | 0 | 0 | 0 | 0 | 1 | 0 | 0 | 0 | 0 | 0 | 0 | 0 | 1 | Bacteria | Bacteria\_none | Bacteroidota | Flavobacteriia | Flavobacteriales | Flavobacteriaceae | Dokdonia | Bacteria;Bacteria\_none;Bacteroidota;Flavobacteriia;Flavobacteriales;Flavobacteriaceae;Dokdonia;Dokdonia sp. Dokd-P16 |
| Euzebyella marina | 0 | 0 | 0 | 0 | 0 | 0 | 0 | 0 | 0 | 0 | 0 | 0 | 1 | 1 | Bacteria | Bacteria\_none | Bacteroidota | Flavobacteriia | Flavobacteriales | Flavobacteriaceae | Euzebyella | Bacteria;Bacteria\_none;Bacteroidota;Flavobacteriia;Flavobacteriales;Flavobacteriaceae;Euzebyella;Euzebyella marina |
| Flagellimonas oceani | 0 | 0 | 0 | 0 | 0 | 0 | 0 | 1 | 0 | 0 | 0 | 0 | 0 | 1 | Bacteria | Bacteria\_none | Bacteroidota | Flavobacteriia | Flavobacteriales | Flavobacteriaceae | Flagellimonas | Bacteria;Bacteria\_none;Bacteroidota;Flavobacteriia;Flavobacteriales;Flavobacteriaceae;Flagellimonas;Flagellimonas oceani |
| Flagellimonas sp. CMM7 | 0 | 0 | 0 | 0 | 0 | 0 | 0 | 0 | 0 | 0 | 1 | 0 | 0 | 1 | Bacteria | Bacteria\_none | Bacteroidota | Flavobacteriia | Flavobacteriales | Flavobacteriaceae | Flagellimonas | Bacteria;Bacteria\_none;Bacteroidota;Flavobacteriia;Flavobacteriales;Flavobacteriaceae;Flagellimonas;Flagellimonas sp. CMM7 |
| Flavobacterium aestivum | 0 | 0 | 0 | 0 | 0 | 0 | 0 | 0 | 0 | 0 | 1 | 0 | 0 | 1 | Bacteria | Bacteria\_none | Bacteroidota | Flavobacteriia | Flavobacteriales | Flavobacteriaceae | Flavobacterium | Bacteria;Bacteria\_none;Bacteroidota;Flavobacteriia;Flavobacteriales;Flavobacteriaceae;Flavobacterium;Flavobacterium aestivum |
| Flavobacterium agricola | 0 | 0 | 0 | 0 | 1 | 0 | 0 | 0 | 0 | 0 | 0 | 0 | 0 | 1 | Bacteria | Bacteria\_none | Bacteroidota | Flavobacteriia | Flavobacteriales | Flavobacteriaceae | Flavobacterium | Bacteria;Bacteria\_none;Bacteroidota;Flavobacteriia;Flavobacteriales;Flavobacteriaceae;Flavobacterium;Flavobacterium agricola |
| Flavobacterium alkalisoli | 0 | 0 | 0 | 0 | 0 | 0 | 1 | 0 | 0 | 0 | 0 | 0 | 0 | 1 | Bacteria | Bacteria\_none | Bacteroidota | Flavobacteriia | Flavobacteriales | Flavobacteriaceae | Flavobacterium | Bacteria;Bacteria\_none;Bacteroidota;Flavobacteriia;Flavobacteriales;Flavobacteriaceae;Flavobacterium;Flavobacterium alkalisoli |
| Flavobacterium aquiphilum | 0 | 0 | 0 | 1 | 0 | 0 | 0 | 0 | 0 | 0 | 0 | 0 | 0 | 1 | Bacteria | Bacteria\_none | Bacteroidota | Flavobacteriia | Flavobacteriales | Flavobacteriaceae | Flavobacterium | Bacteria;Bacteria\_none;Bacteroidota;Flavobacteriia;Flavobacteriales;Flavobacteriaceae;Flavobacterium;Flavobacterium aquiphilum |
| Flavobacterium arcticum | 0 | 1 | 0 | 0 | 0 | 0 | 0 | 0 | 0 | 0 | 0 | 0 | 0 | 1 | Bacteria | Bacteria\_none | Bacteroidota | Flavobacteriia | Flavobacteriales | Flavobacteriaceae | Flavobacterium | Bacteria;Bacteria\_none;Bacteroidota;Flavobacteriia;Flavobacteriales;Flavobacteriaceae;Flavobacterium;Flavobacterium arcticum |
| Flavobacterium azooxidireducens | 0 | 0 | 0 | 0 | 0 | 0 | 0 | 0 | 0 | 0 | 0 | 1 | 0 | 1 | Bacteria | Bacteria\_none | Bacteroidota | Flavobacteriia | Flavobacteriales | Flavobacteriaceae | Flavobacterium | Bacteria;Bacteria\_none;Bacteroidota;Flavobacteriia;Flavobacteriales;Flavobacteriaceae;Flavobacterium;Flavobacterium azooxidireducens |
| Flavobacterium bizetiae | 0 | 0 | 0 | 0 | 1 | 0 | 0 | 0 | 0 | 0 | 0 | 0 | 0 | 1 | Bacteria | Bacteria\_none | Bacteroidota | Flavobacteriia | Flavobacteriales | Flavobacteriaceae | Flavobacterium | Bacteria;Bacteria\_none;Bacteroidota;Flavobacteriia;Flavobacteriales;Flavobacteriaceae;Flavobacterium;Flavobacterium bizetiae |
| Flavobacterium crassostreae | 0 | 0 | 0 | 0 | 0 | 0 | 0 | 0 | 0 | 0 | 1 | 0 | 0 | 1 | Bacteria | Bacteria\_none | Bacteroidota | Flavobacteriia | Flavobacteriales | Flavobacteriaceae | Flavobacterium | Bacteria;Bacteria\_none;Bacteroidota;Flavobacteriia;Flavobacteriales;Flavobacteriaceae;Flavobacterium;Flavobacterium crassostreae |
| Flavobacterium dauae | 0 | 0 | 0 | 1 | 0 | 0 | 0 | 0 | 0 | 0 | 0 | 0 | 0 | 1 | Bacteria | Bacteria\_none | Bacteroidota | Flavobacteriia | Flavobacteriales | Flavobacteriaceae | Flavobacterium | Bacteria;Bacteria\_none;Bacteroidota;Flavobacteriia;Flavobacteriales;Flavobacteriaceae;Flavobacterium;Flavobacterium dauae |
| Flavobacterium faecale | 1 | 0 | 0 | 0 | 0 | 0 | 0 | 0 | 0 | 0 | 0 | 0 | 0 | 1 | Bacteria | Bacteria\_none | Bacteroidota | Flavobacteriia | Flavobacteriales | Flavobacteriaceae | Flavobacterium | Bacteria;Bacteria\_none;Bacteroidota;Flavobacteriia;Flavobacteriales;Flavobacteriaceae;Flavobacterium;Flavobacterium faecale |
| Flavobacterium flavipigmentatum | 0 | 0 | 0 | 0 | 1 | 0 | 0 | 0 | 0 | 0 | 0 | 0 | 0 | 1 | Bacteria | Bacteria\_none | Bacteroidota | Flavobacteriia | Flavobacteriales | Flavobacteriaceae | Flavobacterium | Bacteria;Bacteria\_none;Bacteroidota;Flavobacteriia;Flavobacteriales;Flavobacteriaceae;Flavobacterium;Flavobacterium flavipigmentatum |
| Flavobacterium ginsengisoli | 0 | 0 | 0 | 0 | 0 | 0 | 0 | 0 | 0 | 0 | 0 | 0 | 1 | 1 | Bacteria | Bacteria\_none | Bacteroidota | Flavobacteriia | Flavobacteriales | Flavobacteriaceae | Flavobacterium | Bacteria;Bacteria\_none;Bacteroidota;Flavobacteriia;Flavobacteriales;Flavobacteriaceae;Flavobacterium;Flavobacterium ginsengisoli |
| Flavobacterium indicum | 0 | 0 | 0 | 1 | 0 | 0 | 0 | 0 | 0 | 0 | 0 | 0 | 0 | 1 | Bacteria | Bacteria\_none | Bacteroidota | Flavobacteriia | Flavobacteriales | Flavobacteriaceae | Flavobacterium | Bacteria;Bacteria\_none;Bacteroidota;Flavobacteriia;Flavobacteriales;Flavobacteriaceae;Flavobacterium;Flavobacterium indicum |
| Flavobacterium limnophilum | 0 | 0 | 0 | 0 | 0 | 2 | 0 | 0 | 0 | 0 | 0 | 0 | 0 | 2 | Bacteria | Bacteria\_none | Bacteroidota | Flavobacteriia | Flavobacteriales | Flavobacteriaceae | Flavobacterium | Bacteria;Bacteria\_none;Bacteroidota;Flavobacteriia;Flavobacteriales;Flavobacteriaceae;Flavobacterium;Flavobacterium limnophilum |
| Flavobacterium psychrophilum | 0 | 0 | 0 | 1 | 0 | 0 | 0 | 0 | 0 | 0 | 0 | 0 | 0 | 1 | Bacteria | Bacteria\_none | Bacteroidota | Flavobacteriia | Flavobacteriales | Flavobacteriaceae | Flavobacterium | Bacteria;Bacteria\_none;Bacteroidota;Flavobacteriia;Flavobacteriales;Flavobacteriaceae;Flavobacterium;Flavobacterium psychrophilum |
| Flavobacterium sp. 140616W15 | 0 | 0 | 0 | 0 | 0 | 0 | 0 | 0 | 0 | 0 | 0 | 1 | 0 | 1 | Bacteria | Bacteria\_none | Bacteroidota | Flavobacteriia | Flavobacteriales | Flavobacteriaceae | Flavobacterium | Bacteria;Bacteria\_none;Bacteroidota;Flavobacteriia;Flavobacteriales;Flavobacteriaceae;Flavobacterium;Flavobacterium sp. 140616W15 |
| Flavobacterium sp. CLA17 | 1 | 0 | 0 | 0 | 0 | 0 | 0 | 0 | 0 | 1 | 0 | 0 | 0 | 2 | Bacteria | Bacteria\_none | Bacteroidota | Flavobacteriia | Flavobacteriales | Flavobacteriaceae | Flavobacterium | Bacteria;Bacteria\_none;Bacteroidota;Flavobacteriia;Flavobacteriales;Flavobacteriaceae;Flavobacterium;Flavobacterium sp. CLA17 |
| Flavobacterium sp. I3-2 | 0 | 1 | 0 | 0 | 2 | 0 | 0 | 0 | 0 | 0 | 0 | 0 | 0 | 3 | Bacteria | Bacteria\_none | Bacteroidota | Flavobacteriia | Flavobacteriales | Flavobacteriaceae | Flavobacterium | Bacteria;Bacteria\_none;Bacteroidota;Flavobacteriia;Flavobacteriales;Flavobacteriaceae;Flavobacterium;Flavobacterium sp. I3-2 |
| Flavobacterium sp. IMCC34518 | 0 | 0 | 0 | 0 | 0 | 0 | 0 | 0 | 0 | 0 | 0 | 0 | 1 | 1 | Bacteria | Bacteria\_none | Bacteroidota | Flavobacteriia | Flavobacteriales | Flavobacteriaceae | Flavobacterium | Bacteria;Bacteria\_none;Bacteroidota;Flavobacteriia;Flavobacteriales;Flavobacteriaceae;Flavobacterium;Flavobacterium sp. IMCC34518 |
| Flavobacterium sp. IMCC34852 | 0 | 0 | 0 | 0 | 0 | 0 | 0 | 0 | 0 | 1 | 0 | 0 | 0 | 1 | Bacteria | Bacteria\_none | Bacteroidota | Flavobacteriia | Flavobacteriales | Flavobacteriaceae | Flavobacterium | Bacteria;Bacteria\_none;Bacteroidota;Flavobacteriia;Flavobacteriales;Flavobacteriaceae;Flavobacterium;Flavobacterium sp. IMCC34852 |
| Flavobacterium sp. KBS0721 | 0 | 0 | 0 | 0 | 0 | 0 | 0 | 0 | 0 | 0 | 0 | 0 | 1 | 1 | Bacteria | Bacteria\_none | Bacteroidota | Flavobacteriia | Flavobacteriales | Flavobacteriaceae | Flavobacterium | Bacteria;Bacteria\_none;Bacteroidota;Flavobacteriia;Flavobacteriales;Flavobacteriaceae;Flavobacterium;Flavobacterium sp. KBS0721 |
| Formosa sediminum | 0 | 0 | 0 | 0 | 0 | 1 | 0 | 0 | 0 | 0 | 0 | 0 | 1 | 2 | Bacteria | Bacteria\_none | Bacteroidota | Flavobacteriia | Flavobacteriales | Flavobacteriaceae | Formosa | Bacteria;Bacteria\_none;Bacteroidota;Flavobacteriia;Flavobacteriales;Flavobacteriaceae;Formosa;Formosa sediminum |
| Formosa sp. Hel1\_31\_208 | 0 | 0 | 0 | 0 | 0 | 0 | 0 | 0 | 0 | 0 | 0 | 0 | 1 | 1 | Bacteria | Bacteria\_none | Bacteroidota | Flavobacteriia | Flavobacteriales | Flavobacteriaceae | Formosa | Bacteria;Bacteria\_none;Bacteroidota;Flavobacteriia;Flavobacteriales;Flavobacteriaceae;Formosa;Formosa sp. Hel1\_31\_208 |
| Formosa sp. L2A11 | 0 | 0 | 0 | 0 | 1 | 0 | 0 | 0 | 0 | 0 | 0 | 0 | 0 | 1 | Bacteria | Bacteria\_none | Bacteroidota | Flavobacteriia | Flavobacteriales | Flavobacteriaceae | Formosa | Bacteria;Bacteria\_none;Bacteroidota;Flavobacteriia;Flavobacteriales;Flavobacteriaceae;Formosa;Formosa sp. L2A11 |
| Kordia antarctica | 0 | 0 | 0 | 1 | 0 | 0 | 0 | 0 | 0 | 0 | 0 | 0 | 1 | 2 | Bacteria | Bacteria\_none | Bacteroidota | Flavobacteriia | Flavobacteriales | Flavobacteriaceae | Kordia | Bacteria;Bacteria\_none;Bacteroidota;Flavobacteriia;Flavobacteriales;Flavobacteriaceae;Kordia;Kordia antarctica |
| Kordia sp. SMS9 | 0 | 0 | 0 | 0 | 0 | 0 | 0 | 0 | 0 | 0 | 0 | 0 | 1 | 1 | Bacteria | Bacteria\_none | Bacteroidota | Flavobacteriia | Flavobacteriales | Flavobacteriaceae | Kordia | Bacteria;Bacteria\_none;Bacteroidota;Flavobacteriia;Flavobacteriales;Flavobacteriaceae;Kordia;Kordia sp. SMS9 |
| Lacinutrix neustonica | 0 | 0 | 0 | 0 | 0 | 0 | 0 | 0 | 0 | 0 | 0 | 0 | 1 | 1 | Bacteria | Bacteria\_none | Bacteroidota | Flavobacteriia | Flavobacteriales | Flavobacteriaceae | Lacinutrix | Bacteria;Bacteria\_none;Bacteroidota;Flavobacteriia;Flavobacteriales;Flavobacteriaceae;Lacinutrix;Lacinutrix neustonica |
| Lacinutrix sp. 5H-3-7-4 | 0 | 0 | 0 | 1 | 0 | 0 | 0 | 0 | 0 | 0 | 0 | 0 | 0 | 1 | Bacteria | Bacteria\_none | Bacteroidota | Flavobacteriia | Flavobacteriales | Flavobacteriaceae | Lacinutrix | Bacteria;Bacteria\_none;Bacteroidota;Flavobacteriia;Flavobacteriales;Flavobacteriaceae;Lacinutrix;Lacinutrix sp. 5H-3-7-4 |
| Lacinutrix sp. WUR7 | 0 | 0 | 0 | 0 | 0 | 0 | 0 | 0 | 0 | 0 | 0 | 0 | 1 | 1 | Bacteria | Bacteria\_none | Bacteroidota | Flavobacteriia | Flavobacteriales | Flavobacteriaceae | Lacinutrix | Bacteria;Bacteria\_none;Bacteroidota;Flavobacteriia;Flavobacteriales;Flavobacteriaceae;Lacinutrix;Lacinutrix sp. WUR7 |
| Lacinutrix venerupis | 0 | 0 | 0 | 0 | 0 | 0 | 0 | 0 | 0 | 0 | 0 | 0 | 1 | 1 | Bacteria | Bacteria\_none | Bacteroidota | Flavobacteriia | Flavobacteriales | Flavobacteriaceae | Lacinutrix | Bacteria;Bacteria\_none;Bacteroidota;Flavobacteriia;Flavobacteriales;Flavobacteriaceae;Lacinutrix;Lacinutrix venerupis |
| Leeuwenhoekiella sp. ZYFB001 | 0 | 0 | 0 | 0 | 1 | 0 | 0 | 0 | 0 | 0 | 0 | 0 | 0 | 1 | Bacteria | Bacteria\_none | Bacteroidota | Flavobacteriia | Flavobacteriales | Flavobacteriaceae | Leeuwenhoekiella | Bacteria;Bacteria\_none;Bacteroidota;Flavobacteriia;Flavobacteriales;Flavobacteriaceae;Leeuwenhoekiella;Leeuwenhoekiella sp. ZYFB001 |
| Lutibacter sp. A80 | 0 | 0 | 0 | 0 | 0 | 0 | 0 | 0 | 0 | 0 | 0 | 0 | 1 | 1 | Bacteria | Bacteria\_none | Bacteroidota | Flavobacteriia | Flavobacteriales | Flavobacteriaceae | Lutibacter | Bacteria;Bacteria\_none;Bacteroidota;Flavobacteriia;Flavobacteriales;Flavobacteriaceae;Lutibacter;Lutibacter sp. A80 |
| Mangrovimonas sp. YM274 | 0 | 0 | 0 | 0 | 0 | 0 | 0 | 0 | 0 | 1 | 0 | 0 | 0 | 1 | Bacteria | Bacteria\_none | Bacteroidota | Flavobacteriia | Flavobacteriales | Flavobacteriaceae | Mangrovimonas | Bacteria;Bacteria\_none;Bacteroidota;Flavobacteriia;Flavobacteriales;Flavobacteriaceae;Mangrovimonas;Mangrovimonas sp. YM274 |
| Maribacter hydrothermalis | 0 | 0 | 0 | 0 | 0 | 0 | 0 | 0 | 0 | 0 | 0 | 0 | 1 | 1 | Bacteria | Bacteria\_none | Bacteroidota | Flavobacteriia | Flavobacteriales | Flavobacteriaceae | Maribacter | Bacteria;Bacteria\_none;Bacteroidota;Flavobacteriia;Flavobacteriales;Flavobacteriaceae;Maribacter;Maribacter hydrothermalis |
| Maribacter sp. BPC-D8 | 0 | 0 | 0 | 0 | 1 | 0 | 0 | 0 | 0 | 0 | 0 | 0 | 0 | 1 | Bacteria | Bacteria\_none | Bacteroidota | Flavobacteriia | Flavobacteriales | Flavobacteriaceae | Maribacter | Bacteria;Bacteria\_none;Bacteroidota;Flavobacteriia;Flavobacteriales;Flavobacteriaceae;Maribacter;Maribacter sp. BPC-D8 |
| Myroides albus | 0 | 0 | 0 | 0 | 1 | 0 | 0 | 0 | 0 | 0 | 0 | 0 | 0 | 1 | Bacteria | Bacteria\_none | Bacteroidota | Flavobacteriia | Flavobacteriales | Flavobacteriaceae | Myroides | Bacteria;Bacteria\_none;Bacteroidota;Flavobacteriia;Flavobacteriales;Flavobacteriaceae;Myroides;Myroides albus |
| Myroides fluvii | 0 | 0 | 0 | 0 | 0 | 0 | 0 | 0 | 0 | 1 | 0 | 0 | 0 | 1 | Bacteria | Bacteria\_none | Bacteroidota | Flavobacteriia | Flavobacteriales | Flavobacteriaceae | Myroides | Bacteria;Bacteria\_none;Bacteroidota;Flavobacteriia;Flavobacteriales;Flavobacteriaceae;Myroides;Myroides fluvii |
| Nonlabens sp. MIC269 | 0 | 0 | 0 | 0 | 0 | 0 | 0 | 0 | 0 | 0 | 0 | 0 | 1 | 1 | Bacteria | Bacteria\_none | Bacteroidota | Flavobacteriia | Flavobacteriales | Flavobacteriaceae | Nonlabens | Bacteria;Bacteria\_none;Bacteroidota;Flavobacteriia;Flavobacteriales;Flavobacteriaceae;Nonlabens;Nonlabens sp. MIC269 |
| Polaribacter gangjinensis | 0 | 0 | 0 | 0 | 0 | 0 | 0 | 0 | 0 | 0 | 1 | 0 | 0 | 1 | Bacteria | Bacteria\_none | Bacteroidota | Flavobacteriia | Flavobacteriales | Flavobacteriaceae | Polaribacter | Bacteria;Bacteria\_none;Bacteroidota;Flavobacteriia;Flavobacteriales;Flavobacteriaceae;Polaribacter;Polaribacter gangjinensis |
| Polaribacter haliotis | 0 | 0 | 0 | 0 | 1 | 0 | 0 | 0 | 0 | 0 | 0 | 0 | 0 | 1 | Bacteria | Bacteria\_none | Bacteroidota | Flavobacteriia | Flavobacteriales | Flavobacteriaceae | Polaribacter | Bacteria;Bacteria\_none;Bacteroidota;Flavobacteriia;Flavobacteriales;Flavobacteriaceae;Polaribacter;Polaribacter haliotis |
| Polaribacter litorisediminis | 0 | 0 | 0 | 0 | 0 | 0 | 1 | 0 | 0 | 0 | 0 | 0 | 0 | 1 | Bacteria | Bacteria\_none | Bacteroidota | Flavobacteriia | Flavobacteriales | Flavobacteriaceae | Polaribacter | Bacteria;Bacteria\_none;Bacteroidota;Flavobacteriia;Flavobacteriales;Flavobacteriaceae;Polaribacter;Polaribacter litorisediminis |
| Polaribacter pectinis | 0 | 0 | 0 | 0 | 0 | 0 | 0 | 0 | 0 | 0 | 0 | 0 | 1 | 1 | Bacteria | Bacteria\_none | Bacteroidota | Flavobacteriia | Flavobacteriales | Flavobacteriaceae | Polaribacter | Bacteria;Bacteria\_none;Bacteroidota;Flavobacteriia;Flavobacteriales;Flavobacteriaceae;Polaribacter;Polaribacter pectinis |
| Polaribacter sejongensis | 0 | 0 | 1 | 0 | 0 | 1 | 0 | 0 | 0 | 0 | 0 | 0 | 0 | 2 | Bacteria | Bacteria\_none | Bacteroidota | Flavobacteriia | Flavobacteriales | Flavobacteriaceae | Polaribacter | Bacteria;Bacteria\_none;Bacteroidota;Flavobacteriia;Flavobacteriales;Flavobacteriaceae;Polaribacter;Polaribacter sejongensis |
| Polaribacter sp. SA4-12 | 0 | 0 | 0 | 2 | 0 | 0 | 0 | 0 | 0 | 0 | 0 | 0 | 0 | 2 | Bacteria | Bacteria\_none | Bacteroidota | Flavobacteriia | Flavobacteriales | Flavobacteriaceae | Polaribacter | Bacteria;Bacteria\_none;Bacteroidota;Flavobacteriia;Flavobacteriales;Flavobacteriaceae;Polaribacter;Polaribacter sp. SA4-12 |
| Psychroflexus torquis | 0 | 0 | 0 | 0 | 0 | 0 | 0 | 0 | 0 | 0 | 1 | 0 | 0 | 1 | Bacteria | Bacteria\_none | Bacteroidota | Flavobacteriia | Flavobacteriales | Flavobacteriaceae | Psychroflexus | Bacteria;Bacteria\_none;Bacteroidota;Flavobacteriia;Flavobacteriales;Flavobacteriaceae;Psychroflexus;Psychroflexus torquis |
| Rasiella rasia | 0 | 0 | 0 | 0 | 0 | 0 | 0 | 0 | 0 | 0 | 1 | 0 | 0 | 1 | Bacteria | Bacteria\_none | Bacteroidota | Flavobacteriia | Flavobacteriales | Flavobacteriaceae | Rasiella | Bacteria;Bacteria\_none;Bacteroidota;Flavobacteriia;Flavobacteriales;Flavobacteriaceae;Rasiella;Rasiella rasia |
| Salegentibacter salegens | 0 | 1 | 0 | 0 | 0 | 0 | 0 | 0 | 0 | 0 | 0 | 0 | 0 | 1 | Bacteria | Bacteria\_none | Bacteroidota | Flavobacteriia | Flavobacteriales | Flavobacteriaceae | Salegentibacter | Bacteria;Bacteria\_none;Bacteroidota;Flavobacteriia;Flavobacteriales;Flavobacteriaceae;Salegentibacter;Salegentibacter salegens |
| Tenacibaculum jejuense | 0 | 0 | 0 | 0 | 1 | 0 | 0 | 0 | 0 | 0 | 0 | 0 | 0 | 1 | Bacteria | Bacteria\_none | Bacteroidota | Flavobacteriia | Flavobacteriales | Flavobacteriaceae | Tenacibaculum | Bacteria;Bacteria\_none;Bacteroidota;Flavobacteriia;Flavobacteriales;Flavobacteriaceae;Tenacibaculum;Tenacibaculum jejuense |
| Tenacibaculum maritimum | 0 | 0 | 0 | 0 | 1 | 0 | 0 | 0 | 0 | 0 | 0 | 0 | 0 | 1 | Bacteria | Bacteria\_none | Bacteroidota | Flavobacteriia | Flavobacteriales | Flavobacteriaceae | Tenacibaculum | Bacteria;Bacteria\_none;Bacteroidota;Flavobacteriia;Flavobacteriales;Flavobacteriaceae;Tenacibaculum;Tenacibaculum maritimum |
| Tenacibaculum pacificus | 0 | 0 | 0 | 0 | 0 | 0 | 0 | 0 | 0 | 0 | 1 | 0 | 0 | 1 | Bacteria | Bacteria\_none | Bacteroidota | Flavobacteriia | Flavobacteriales | Flavobacteriaceae | Tenacibaculum | Bacteria;Bacteria\_none;Bacteroidota;Flavobacteriia;Flavobacteriales;Flavobacteriaceae;Tenacibaculum;Tenacibaculum pacificus |
| Tenacibaculum retecalamus | 0 | 0 | 1 | 0 | 0 | 0 | 0 | 0 | 0 | 0 | 0 | 0 | 0 | 1 | Bacteria | Bacteria\_none | Bacteroidota | Flavobacteriia | Flavobacteriales | Flavobacteriaceae | Tenacibaculum | Bacteria;Bacteria\_none;Bacteroidota;Flavobacteriia;Flavobacteriales;Flavobacteriaceae;Tenacibaculum;Tenacibaculum retecalamus |
| Zobellia alginiliquefaciens | 0 | 0 | 0 | 0 | 1 | 0 | 0 | 0 | 0 | 0 | 0 | 0 | 0 | 1 | Bacteria | Bacteria\_none | Bacteroidota | Flavobacteriia | Flavobacteriales | Flavobacteriaceae | Zobellia | Bacteria;Bacteria\_none;Bacteroidota;Flavobacteriia;Flavobacteriales;Flavobacteriaceae;Zobellia;Zobellia alginiliquefaciens |
| Croceimicrobium hydrocarbonivorans | 0 | 1 | 0 | 0 | 1 | 0 | 0 | 0 | 0 | 0 | 0 | 0 | 0 | 2 | Bacteria | Bacteria\_none | Bacteroidota | Flavobacteriia | Flavobacteriales | Owenweeksiaceae | Croceimicrobium | Bacteria;Bacteria\_none;Bacteroidota;Flavobacteriia;Flavobacteriales;Owenweeksiaceae;Croceimicrobium;Croceimicrobium hydrocarbonivorans |
| Sanyastnella coralliicola | 0 | 0 | 1 | 0 | 0 | 0 | 0 | 0 | 0 | 0 | 0 | 0 | 0 | 1 | Bacteria | Bacteria\_none | Bacteroidota | Flavobacteriia | Flavobacteriales | Sanyastnellaceae | Sanyastnella | Bacteria;Bacteria\_none;Bacteroidota;Flavobacteriia;Flavobacteriales;Sanyastnellaceae;Sanyastnella;Sanyastnella coralliicola |
| Apibacter sp. ESL0432 | 0 | 0 | 0 | 0 | 1 | 0 | 0 | 0 | 0 | 0 | 0 | 0 | 0 | 1 | Bacteria | Bacteria\_none | Bacteroidota | Flavobacteriia | Flavobacteriales | Weeksellaceae | Apibacter | Bacteria;Bacteria\_none;Bacteroidota;Flavobacteriia;Flavobacteriales;Weeksellaceae;Apibacter;Apibacter sp. ESL0432 |
| Bergeyella cardium | 0 | 0 | 0 | 0 | 2 | 0 | 0 | 0 | 0 | 0 | 1 | 0 | 0 | 3 | Bacteria | Bacteria\_none | Bacteroidota | Flavobacteriia | Flavobacteriales | Weeksellaceae | Bergeyella | Bacteria;Bacteria\_none;Bacteroidota;Flavobacteriia;Flavobacteriales;Weeksellaceae;Bergeyella;Bergeyella cardium |
| Bergeyella porcorum | 0 | 0 | 1 | 0 | 0 | 0 | 0 | 0 | 0 | 0 | 0 | 0 | 0 | 1 | Bacteria | Bacteria\_none | Bacteroidota | Flavobacteriia | Flavobacteriales | Weeksellaceae | Bergeyella | Bacteria;Bacteria\_none;Bacteroidota;Flavobacteriia;Flavobacteriales;Weeksellaceae;Bergeyella;Bergeyella porcorum |
| Chryseobacterium arthrosphaerae | 2 | 0 | 0 | 0 | 0 | 0 | 0 | 0 | 0 | 0 | 0 | 0 | 0 | 2 | Bacteria | Bacteria\_none | Bacteroidota | Flavobacteriia | Flavobacteriales | Weeksellaceae | Chryseobacterium | Bacteria;Bacteria\_none;Bacteroidota;Flavobacteriia;Flavobacteriales;Weeksellaceae;Chryseobacterium;Chryseobacterium arthrosphaerae |
| Chryseobacterium aureum | 0 | 0 | 0 | 0 | 0 | 0 | 0 | 0 | 0 | 1 | 0 | 0 | 0 | 1 | Bacteria | Bacteria\_none | Bacteroidota | Flavobacteriia | Flavobacteriales | Weeksellaceae | Chryseobacterium | Bacteria;Bacteria\_none;Bacteroidota;Flavobacteriia;Flavobacteriales;Weeksellaceae;Chryseobacterium;Chryseobacterium aureum |
| Chryseobacterium balustinum | 0 | 0 | 0 | 0 | 2 | 0 | 0 | 0 | 0 | 0 | 0 | 0 | 0 | 2 | Bacteria | Bacteria\_none | Bacteroidota | Flavobacteriia | Flavobacteriales | Weeksellaceae | Chryseobacterium | Bacteria;Bacteria\_none;Bacteroidota;Flavobacteriia;Flavobacteriales;Weeksellaceae;Chryseobacterium;Chryseobacterium balustinum |
| Chryseobacterium carnipullorum | 0 | 0 | 0 | 1 | 0 | 0 | 0 | 0 | 0 | 0 | 0 | 0 | 0 | 1 | Bacteria | Bacteria\_none | Bacteroidota | Flavobacteriia | Flavobacteriales | Weeksellaceae | Chryseobacterium | Bacteria;Bacteria\_none;Bacteroidota;Flavobacteriia;Flavobacteriales;Weeksellaceae;Chryseobacterium;Chryseobacterium carnipullorum |
| Chryseobacterium daecheongense | 0 | 0 | 0 | 0 | 0 | 0 | 0 | 2 | 0 | 0 | 0 | 0 | 0 | 2 | Bacteria | Bacteria\_none | Bacteroidota | Flavobacteriia | Flavobacteriales | Weeksellaceae | Chryseobacterium | Bacteria;Bacteria\_none;Bacteroidota;Flavobacteriia;Flavobacteriales;Weeksellaceae;Chryseobacterium;Chryseobacterium daecheongense |
| Chryseobacterium gallinarum | 0 | 0 | 0 | 0 | 0 | 2 | 0 | 0 | 0 | 0 | 0 | 0 | 0 | 2 | Bacteria | Bacteria\_none | Bacteroidota | Flavobacteriia | Flavobacteriales | Weeksellaceae | Chryseobacterium | Bacteria;Bacteria\_none;Bacteroidota;Flavobacteriia;Flavobacteriales;Weeksellaceae;Chryseobacterium;Chryseobacterium gallinarum |
| Chryseobacterium gambrini | 0 | 0 | 1 | 0 | 0 | 0 | 0 | 0 | 0 | 0 | 0 | 0 | 0 | 1 | Bacteria | Bacteria\_none | Bacteroidota | Flavobacteriia | Flavobacteriales | Weeksellaceae | Chryseobacterium | Bacteria;Bacteria\_none;Bacteroidota;Flavobacteriia;Flavobacteriales;Weeksellaceae;Chryseobacterium;Chryseobacterium gambrini |
| Chryseobacterium indoltheticum | 0 | 0 | 0 | 0 | 0 | 0 | 0 | 0 | 0 | 1 | 0 | 0 | 0 | 1 | Bacteria | Bacteria\_none | Bacteroidota | Flavobacteriia | Flavobacteriales | Weeksellaceae | Chryseobacterium | Bacteria;Bacteria\_none;Bacteroidota;Flavobacteriia;Flavobacteriales;Weeksellaceae;Chryseobacterium;Chryseobacterium indoltheticum |
| Chryseobacterium joostei | 0 | 0 | 0 | 0 | 0 | 0 | 0 | 0 | 0 | 1 | 0 | 0 | 0 | 1 | Bacteria | Bacteria\_none | Bacteroidota | Flavobacteriia | Flavobacteriales | Weeksellaceae | Chryseobacterium | Bacteria;Bacteria\_none;Bacteroidota;Flavobacteriia;Flavobacteriales;Weeksellaceae;Chryseobacterium;Chryseobacterium joostei |
| Chryseobacterium manosquense | 0 | 0 | 0 | 0 | 1 | 0 | 0 | 0 | 0 | 0 | 0 | 0 | 0 | 1 | Bacteria | Bacteria\_none | Bacteroidota | Flavobacteriia | Flavobacteriales | Weeksellaceae | Chryseobacterium | Bacteria;Bacteria\_none;Bacteroidota;Flavobacteriia;Flavobacteriales;Weeksellaceae;Chryseobacterium;Chryseobacterium manosquense |
| Chryseobacterium paludis | 0 | 0 | 0 | 0 | 0 | 0 | 0 | 0 | 0 | 1 | 0 | 0 | 0 | 1 | Bacteria | Bacteria\_none | Bacteroidota | Flavobacteriia | Flavobacteriales | Weeksellaceae | Chryseobacterium | Bacteria;Bacteria\_none;Bacteroidota;Flavobacteriia;Flavobacteriales;Weeksellaceae;Chryseobacterium;Chryseobacterium paludis |
| Chryseobacterium sp. | 0 | 0 | 1 | 0 | 0 | 0 | 0 | 0 | 0 | 0 | 0 | 0 | 0 | 1 | Bacteria | Bacteria\_none | Bacteroidota | Flavobacteriia | Flavobacteriales | Weeksellaceae | Chryseobacterium | Bacteria;Bacteria\_none;Bacteroidota;Flavobacteriia;Flavobacteriales;Weeksellaceae;Chryseobacterium;Chryseobacterium sp. |
| Chryseobacterium sp. G0162 | 0 | 0 | 0 | 1 | 0 | 0 | 0 | 0 | 0 | 0 | 0 | 0 | 0 | 1 | Bacteria | Bacteria\_none | Bacteroidota | Flavobacteriia | Flavobacteriales | Weeksellaceae | Chryseobacterium | Bacteria;Bacteria\_none;Bacteroidota;Flavobacteriia;Flavobacteriales;Weeksellaceae;Chryseobacterium;Chryseobacterium sp. G0162 |
| Chryseobacterium sp. G0186 | 0 | 0 | 0 | 0 | 2 | 0 | 0 | 0 | 0 | 0 | 1 | 0 | 0 | 3 | Bacteria | Bacteria\_none | Bacteroidota | Flavobacteriia | Flavobacteriales | Weeksellaceae | Chryseobacterium | Bacteria;Bacteria\_none;Bacteroidota;Flavobacteriia;Flavobacteriales;Weeksellaceae;Chryseobacterium;Chryseobacterium sp. G0186 |
| Chryseobacterium sp. PET-29 | 0 | 0 | 0 | 0 | 0 | 0 | 0 | 0 | 0 | 0 | 0 | 0 | 1 | 1 | Bacteria | Bacteria\_none | Bacteroidota | Flavobacteriia | Flavobacteriales | Weeksellaceae | Chryseobacterium | Bacteria;Bacteria\_none;Bacteroidota;Flavobacteriia;Flavobacteriales;Weeksellaceae;Chryseobacterium;Chryseobacterium sp. PET-29 |
| Chryseobacterium sp. StRB126 | 0 | 0 | 0 | 0 | 0 | 0 | 0 | 0 | 0 | 1 | 0 | 0 | 0 | 1 | Bacteria | Bacteria\_none | Bacteroidota | Flavobacteriia | Flavobacteriales | Weeksellaceae | Chryseobacterium | Bacteria;Bacteria\_none;Bacteroidota;Flavobacteriia;Flavobacteriales;Weeksellaceae;Chryseobacterium;Chryseobacterium sp. StRB126 |
| Chryseobacterium suipulveris | 0 | 0 | 0 | 0 | 0 | 0 | 0 | 0 | 0 | 0 | 0 | 1 | 1 | 2 | Bacteria | Bacteria\_none | Bacteroidota | Flavobacteriia | Flavobacteriales | Weeksellaceae | Chryseobacterium | Bacteria;Bacteria\_none;Bacteroidota;Flavobacteriia;Flavobacteriales;Weeksellaceae;Chryseobacterium;Chryseobacterium suipulveris |
| Chryseobacterium taklimakanense | 0 | 0 | 0 | 0 | 1 | 0 | 0 | 0 | 0 | 0 | 0 | 0 | 0 | 1 | Bacteria | Bacteria\_none | Bacteroidota | Flavobacteriia | Flavobacteriales | Weeksellaceae | Chryseobacterium | Bacteria;Bacteria\_none;Bacteroidota;Flavobacteriia;Flavobacteriales;Weeksellaceae;Chryseobacterium;Chryseobacterium taklimakanense |
| Cloacibacterium sp. TD35 | 0 | 0 | 0 | 0 | 1 | 0 | 0 | 0 | 0 | 0 | 1 | 0 | 0 | 2 | Bacteria | Bacteria\_none | Bacteroidota | Flavobacteriia | Flavobacteriales | Weeksellaceae | Cloacibacterium | Bacteria;Bacteria\_none;Bacteroidota;Flavobacteriia;Flavobacteriales;Weeksellaceae;Cloacibacterium;Cloacibacterium sp. TD35 |
| Elizabethkingia anophelis | 0 | 0 | 0 | 0 | 0 | 0 | 0 | 1 | 0 | 0 | 0 | 0 | 0 | 1 | Bacteria | Bacteria\_none | Bacteroidota | Flavobacteriia | Flavobacteriales | Weeksellaceae | Elizabethkingia | Bacteria;Bacteria\_none;Bacteroidota;Flavobacteriia;Flavobacteriales;Weeksellaceae;Elizabethkingia;Elizabethkingia anophelis |
| Elizabethkingia meningoseptica | 0 | 0 | 0 | 0 | 1 | 1 | 0 | 0 | 0 | 0 | 0 | 0 | 0 | 2 | Bacteria | Bacteria\_none | Bacteroidota | Flavobacteriia | Flavobacteriales | Weeksellaceae | Elizabethkingia | Bacteria;Bacteria\_none;Bacteroidota;Flavobacteriia;Flavobacteriales;Weeksellaceae;Elizabethkingia;Elizabethkingia meningoseptica |
| Elizabethkingia sp. JS20170427COW | 0 | 1 | 1 | 0 | 0 | 0 | 0 | 0 | 0 | 0 | 0 | 0 | 0 | 2 | Bacteria | Bacteria\_none | Bacteroidota | Flavobacteriia | Flavobacteriales | Weeksellaceae | Elizabethkingia | Bacteria;Bacteria\_none;Bacteroidota;Flavobacteriia;Flavobacteriales;Weeksellaceae;Elizabethkingia;Elizabethkingia sp. JS20170427COW |
| Elizabethkingia ursingii | 0 | 0 | 0 | 0 | 1 | 0 | 0 | 0 | 0 | 0 | 0 | 0 | 0 | 1 | Bacteria | Bacteria\_none | Bacteroidota | Flavobacteriia | Flavobacteriales | Weeksellaceae | Elizabethkingia | Bacteria;Bacteria\_none;Bacteroidota;Flavobacteriia;Flavobacteriales;Weeksellaceae;Elizabethkingia;Elizabethkingia ursingii |
| Empedobacter brevis | 0 | 0 | 0 | 0 | 0 | 0 | 0 | 0 | 0 | 0 | 0 | 0 | 1 | 1 | Bacteria | Bacteria\_none | Bacteroidota | Flavobacteriia | Flavobacteriales | Weeksellaceae | Empedobacter | Bacteria;Bacteria\_none;Bacteroidota;Flavobacteriia;Flavobacteriales;Weeksellaceae;Empedobacter;Empedobacter brevis |
| Empedobacter falsenii | 1 | 0 | 0 | 0 | 0 | 0 | 0 | 0 | 0 | 0 | 0 | 0 | 0 | 1 | Bacteria | Bacteria\_none | Bacteroidota | Flavobacteriia | Flavobacteriales | Weeksellaceae | Empedobacter | Bacteria;Bacteria\_none;Bacteroidota;Flavobacteriia;Flavobacteriales;Weeksellaceae;Empedobacter;Empedobacter falsenii |
| Empedobacter stercoris | 0 | 0 | 0 | 1 | 0 | 0 | 0 | 0 | 0 | 0 | 0 | 0 | 1 | 2 | Bacteria | Bacteria\_none | Bacteroidota | Flavobacteriia | Flavobacteriales | Weeksellaceae | Empedobacter | Bacteria;Bacteria\_none;Bacteroidota;Flavobacteriia;Flavobacteriales;Weeksellaceae;Empedobacter;Empedobacter stercoris |
| Epilithonimonas zeae | 0 | 0 | 0 | 0 | 1 | 1 | 0 | 0 | 0 | 0 | 0 | 0 | 0 | 2 | Bacteria | Bacteria\_none | Bacteroidota | Flavobacteriia | Flavobacteriales | Weeksellaceae | Epilithonimonas | Bacteria;Bacteria\_none;Bacteroidota;Flavobacteriia;Flavobacteriales;Weeksellaceae;Epilithonimonas;Epilithonimonas zeae |
| Candidatus Ornithobacterium hominis | 1 | 0 | 0 | 0 | 0 | 0 | 0 | 1 | 0 | 0 | 0 | 0 | 0 | 2 | Bacteria | Bacteria\_none | Bacteroidota | Flavobacteriia | Flavobacteriales | Weeksellaceae | Ornithobacterium | Bacteria;Bacteria\_none;Bacteroidota;Flavobacteriia;Flavobacteriales;Weeksellaceae;Ornithobacterium;Candidatus Ornithobacterium hominis |
| Riemerella anatipestifer | 0 | 0 | 0 | 3 | 1 | 0 | 0 | 0 | 0 | 1 | 0 | 0 | 0 | 5 | Bacteria | Bacteria\_none | Bacteroidota | Flavobacteriia | Flavobacteriales | Weeksellaceae | Riemerella | Bacteria;Bacteria\_none;Bacteroidota;Flavobacteriia;Flavobacteriales;Weeksellaceae;Riemerella;Riemerella anatipestifer |
| Riemerella columbina | 0 | 0 | 0 | 0 | 0 | 1 | 0 | 0 | 0 | 0 | 0 | 0 | 0 | 1 | Bacteria | Bacteria\_none | Bacteroidota | Flavobacteriia | Flavobacteriales | Weeksellaceae | Riemerella | Bacteria;Bacteria\_none;Bacteroidota;Flavobacteriia;Flavobacteriales;Weeksellaceae;Riemerella;Riemerella columbina |
| Haliscomenobacter hydrossis | 0 | 0 | 0 | 0 | 0 | 0 | 0 | 0 | 0 | 0 | 0 | 0 | 1 | 1 | Bacteria | Bacteria\_none | Bacteroidota | Saprospiria | Saprospirales | Haliscomenobacteraceae | Haliscomenobacter | Bacteria;Bacteria\_none;Bacteroidota;Saprospiria;Saprospirales;Haliscomenobacteraceae;Haliscomenobacter;Haliscomenobacter hydrossis |
| Aurantibacillus circumpalustris | 0 | 0 | 0 | 1 | 0 | 0 | 0 | 0 | 0 | 0 | 0 | 0 | 0 | 1 | Bacteria | Bacteria\_none | Bacteroidota | Sphingobacteriia | Sphingobacteriales | Aurantibacillaceae | Aurantibacillus | Bacteria;Bacteria\_none;Bacteroidota;Sphingobacteriia;Sphingobacteriales;Aurantibacillaceae;Aurantibacillus;Aurantibacillus circumpalustris |
| Mucilaginibacter gotjawali | 0 | 0 | 0 | 1 | 0 | 0 | 0 | 0 | 0 | 0 | 0 | 0 | 0 | 1 | Bacteria | Bacteria\_none | Bacteroidota | Sphingobacteriia | Sphingobacteriales | Sphingobacteriaceae | Mucilaginibacter | Bacteria;Bacteria\_none;Bacteroidota;Sphingobacteriia;Sphingobacteriales;Sphingobacteriaceae;Mucilaginibacter;Mucilaginibacter gotjawali |
| Mucilaginibacter mallensis | 0 | 0 | 0 | 1 | 0 | 0 | 0 | 0 | 0 | 0 | 0 | 0 | 0 | 1 | Bacteria | Bacteria\_none | Bacteroidota | Sphingobacteriia | Sphingobacteriales | Sphingobacteriaceae | Mucilaginibacter | Bacteria;Bacteria\_none;Bacteroidota;Sphingobacteriia;Sphingobacteriales;Sphingobacteriaceae;Mucilaginibacter;Mucilaginibacter mallensis |
| Mucilaginibacter robiniae | 0 | 1 | 0 | 0 | 0 | 0 | 0 | 0 | 0 | 0 | 0 | 0 | 0 | 1 | Bacteria | Bacteria\_none | Bacteroidota | Sphingobacteriia | Sphingobacteriales | Sphingobacteriaceae | Mucilaginibacter | Bacteria;Bacteria\_none;Bacteroidota;Sphingobacteriia;Sphingobacteriales;Sphingobacteriaceae;Mucilaginibacter;Mucilaginibacter robiniae |
| Mucilaginibacter sp. 14171R-50 | 0 | 1 | 0 | 0 | 0 | 0 | 0 | 0 | 0 | 0 | 0 | 0 | 0 | 1 | Bacteria | Bacteria\_none | Bacteroidota | Sphingobacteriia | Sphingobacteriales | Sphingobacteriaceae | Mucilaginibacter | Bacteria;Bacteria\_none;Bacteroidota;Sphingobacteriia;Sphingobacteriales;Sphingobacteriaceae;Mucilaginibacter;Mucilaginibacter sp. 14171R-50 |
| Mucilaginibacter sp. SMC90 | 0 | 0 | 0 | 0 | 0 | 0 | 0 | 0 | 0 | 0 | 0 | 1 | 0 | 1 | Bacteria | Bacteria\_none | Bacteroidota | Sphingobacteriia | Sphingobacteriales | Sphingobacteriaceae | Mucilaginibacter | Bacteria;Bacteria\_none;Bacteroidota;Sphingobacteriia;Sphingobacteriales;Sphingobacteriaceae;Mucilaginibacter;Mucilaginibacter sp. SMC90 |
| Olivibacter sp. SDN3 | 0 | 0 | 0 | 1 | 0 | 0 | 0 | 0 | 0 | 0 | 0 | 0 | 0 | 1 | Bacteria | Bacteria\_none | Bacteroidota | Sphingobacteriia | Sphingobacteriales | Sphingobacteriaceae | Olivibacter | Bacteria;Bacteria\_none;Bacteroidota;Sphingobacteriia;Sphingobacteriales;Sphingobacteriaceae;Olivibacter;Olivibacter sp. SDN3 |
| Pedobacter gandavensis | 0 | 0 | 0 | 0 | 0 | 0 | 0 | 0 | 0 | 0 | 1 | 0 | 0 | 1 | Bacteria | Bacteria\_none | Bacteroidota | Sphingobacteriia | Sphingobacteriales | Sphingobacteriaceae | Pedobacter | Bacteria;Bacteria\_none;Bacteroidota;Sphingobacteriia;Sphingobacteriales;Sphingobacteriaceae;Pedobacter;Pedobacter gandavensis |
| Pedobacter ginsengisoli | 0 | 0 | 0 | 0 | 0 | 0 | 0 | 0 | 0 | 0 | 1 | 0 | 0 | 1 | Bacteria | Bacteria\_none | Bacteroidota | Sphingobacteriia | Sphingobacteriales | Sphingobacteriaceae | Pedobacter | Bacteria;Bacteria\_none;Bacteroidota;Sphingobacteriia;Sphingobacteriales;Sphingobacteriaceae;Pedobacter;Pedobacter ginsengisoli |
| Pedobacter roseus | 0 | 0 | 0 | 0 | 0 | 0 | 0 | 1 | 0 | 0 | 0 | 0 | 0 | 1 | Bacteria | Bacteria\_none | Bacteroidota | Sphingobacteriia | Sphingobacteriales | Sphingobacteriaceae | Pedobacter | Bacteria;Bacteria\_none;Bacteroidota;Sphingobacteriia;Sphingobacteriales;Sphingobacteriaceae;Pedobacter;Pedobacter roseus |
| Pedobacter sp. SL55 | 0 | 0 | 0 | 0 | 0 | 1 | 0 | 0 | 0 | 0 | 0 | 0 | 0 | 1 | Bacteria | Bacteria\_none | Bacteroidota | Sphingobacteriia | Sphingobacteriales | Sphingobacteriaceae | Pedobacter | Bacteria;Bacteria\_none;Bacteroidota;Sphingobacteriia;Sphingobacteriales;Sphingobacteriaceae;Pedobacter;Pedobacter sp. SL55 |
| Solitalea lacus | 0 | 0 | 0 | 0 | 0 | 0 | 0 | 0 | 0 | 0 | 0 | 0 | 1 | 1 | Bacteria | Bacteria\_none | Bacteroidota | Sphingobacteriia | Sphingobacteriales | Sphingobacteriaceae | Solitalea | Bacteria;Bacteria\_none;Bacteroidota;Sphingobacteriia;Sphingobacteriales;Sphingobacteriaceae;Solitalea;Solitalea lacus |
| Sphingobacterium sp. LZ7M1 | 0 | 0 | 0 | 0 | 0 | 0 | 0 | 0 | 0 | 0 | 0 | 0 | 1 | 1 | Bacteria | Bacteria\_none | Bacteroidota | Sphingobacteriia | Sphingobacteriales | Sphingobacteriaceae | Sphingobacterium | Bacteria;Bacteria\_none;Bacteroidota;Sphingobacteriia;Sphingobacteriales;Sphingobacteriaceae;Sphingobacterium;Sphingobacterium sp. LZ7M1 |
| Sphingobacterium sp. SYP-B4668 | 0 | 0 | 0 | 0 | 1 | 0 | 0 | 0 | 0 | 0 | 0 | 0 | 0 | 1 | Bacteria | Bacteria\_none | Bacteroidota | Sphingobacteriia | Sphingobacteriales | Sphingobacteriaceae | Sphingobacterium | Bacteria;Bacteria\_none;Bacteroidota;Sphingobacteriia;Sphingobacteriales;Sphingobacteriaceae;Sphingobacterium;Sphingobacterium sp. SYP-B4668 |
| Sphingobacterium spiritivorum | 0 | 0 | 0 | 0 | 0 | 0 | 0 | 0 | 0 | 1 | 1 | 0 | 0 | 2 | Bacteria | Bacteria\_none | Bacteroidota | Sphingobacteriia | Sphingobacteriales | Sphingobacteriaceae | Sphingobacterium | Bacteria;Bacteria\_none;Bacteroidota;Sphingobacteriia;Sphingobacteriales;Sphingobacteriaceae;Sphingobacterium;Sphingobacterium spiritivorum |
| Cyclonatronum proteinivorum | 0 | 0 | 0 | 1 | 0 | 0 | 0 | 0 | 0 | 0 | 0 | 0 | 0 | 1 | Bacteria | Bacteria\_none | Balneolota | Balneolia | Balneolales | Cyclonatronaceae | Cyclonatronum | Bacteria;Bacteria\_none;Balneolota;Balneolia;Balneolales;Cyclonatronaceae;Cyclonatronum;Cyclonatronum proteinivorum |
| Bdellovibrio bacteriovorus | 0 | 0 | 0 | 0 | 0 | 0 | 0 | 0 | 0 | 1 | 0 | 0 | 0 | 1 | Bacteria | Bacteria\_none | Bdellovibrionota | Bdellovibrionia | Bdellovibrionales | Pseudobdellovibrionaceae | Bdellovibrio | Bacteria;Bacteria\_none;Bdellovibrionota;Bdellovibrionia;Bdellovibrionales;Pseudobdellovibrionaceae;Bdellovibrio;Bdellovibrio bacteriovorus |
| Caldithrix abyssi | 0 | 0 | 0 | 1 | 0 | 0 | 0 | 0 | 0 | 0 | 0 | 0 | 0 | 1 | Bacteria | Bacteria\_none | Calditrichota | Calditrichia | Calditrichales | Calditrichaceae | Caldithrix | Bacteria;Bacteria\_none;Calditrichota;Calditrichia;Calditrichales;Calditrichaceae;Caldithrix;Caldithrix abyssi |
| Aliarcobacter butzleri | 0 | 0 | 0 | 0 | 0 | 0 | 0 | 0 | 0 | 0 | 0 | 0 | 1 | 1 | Bacteria | Bacteria\_none | Campylobacterota | Epsilonproteobacteria | Campylobacterales | Arcobacteraceae | Aliarcobacter | Bacteria;Bacteria\_none;Campylobacterota;Epsilonproteobacteria;Campylobacterales;Arcobacteraceae;Aliarcobacter;Aliarcobacter butzleri |
| Aliarcobacter cryaerophilus | 0 | 0 | 0 | 0 | 0 | 0 | 0 | 0 | 0 | 0 | 0 | 0 | 1 | 1 | Bacteria | Bacteria\_none | Campylobacterota | Epsilonproteobacteria | Campylobacterales | Arcobacteraceae | Aliarcobacter | Bacteria;Bacteria\_none;Campylobacterota;Epsilonproteobacteria;Campylobacterales;Arcobacteraceae;Aliarcobacter;Aliarcobacter cryaerophilus |
| Arcobacter ellisii | 0 | 0 | 1 | 0 | 0 | 0 | 0 | 0 | 0 | 0 | 0 | 0 | 0 | 1 | Bacteria | Bacteria\_none | Campylobacterota | Epsilonproteobacteria | Campylobacterales | Arcobacteraceae | Arcobacter | Bacteria;Bacteria\_none;Campylobacterota;Epsilonproteobacteria;Campylobacterales;Arcobacteraceae;Arcobacter;Arcobacter ellisii |
| Arcobacter suis | 0 | 0 | 1 | 0 | 0 | 0 | 0 | 0 | 0 | 0 | 0 | 0 | 0 | 1 | Bacteria | Bacteria\_none | Campylobacterota | Epsilonproteobacteria | Campylobacterales | Arcobacteraceae | Arcobacter | Bacteria;Bacteria\_none;Campylobacterota;Epsilonproteobacteria;Campylobacterales;Arcobacteraceae;Arcobacter;Arcobacter suis |
| Arcobacter venerupis | 0 | 0 | 0 | 0 | 0 | 0 | 0 | 0 | 0 | 1 | 0 | 0 | 0 | 1 | Bacteria | Bacteria\_none | Campylobacterota | Epsilonproteobacteria | Campylobacterales | Arcobacteraceae | Arcobacter | Bacteria;Bacteria\_none;Campylobacterota;Epsilonproteobacteria;Campylobacterales;Arcobacteraceae;Arcobacter;Arcobacter venerupis |
| Halarcobacter sp. | 0 | 0 | 0 | 0 | 0 | 0 | 0 | 0 | 0 | 1 | 0 | 0 | 0 | 1 | Bacteria | Bacteria\_none | Campylobacterota | Epsilonproteobacteria | Campylobacterales | Arcobacteraceae | Halarcobacter | Bacteria;Bacteria\_none;Campylobacterota;Epsilonproteobacteria;Campylobacterales;Arcobacteraceae;Halarcobacter;Halarcobacter sp. |
| Malaciobacter pacificus | 0 | 0 | 0 | 0 | 0 | 0 | 0 | 0 | 0 | 0 | 0 | 0 | 1 | 1 | Bacteria | Bacteria\_none | Campylobacterota | Epsilonproteobacteria | Campylobacterales | Arcobacteraceae | Malaciobacter | Bacteria;Bacteria\_none;Campylobacterota;Epsilonproteobacteria;Campylobacterales;Arcobacteraceae;Malaciobacter;Malaciobacter pacificus |
| Campylobacter coli | 0 | 1 | 0 | 0 | 0 | 0 | 0 | 0 | 0 | 0 | 0 | 0 | 0 | 1 | Bacteria | Bacteria\_none | Campylobacterota | Epsilonproteobacteria | Campylobacterales | Campylobacteraceae | Campylobacter | Bacteria;Bacteria\_none;Campylobacterota;Epsilonproteobacteria;Campylobacterales;Campylobacteraceae;Campylobacter;Campylobacter coli |
| Campylobacter concisus | 0 | 26 | 1 | 1 | 4 | 41 | 0 | 2 | 7 | 0 | 2 | 2 | 44 | 130 | Bacteria | Bacteria\_none | Campylobacterota | Epsilonproteobacteria | Campylobacterales | Campylobacteraceae | Campylobacter | Bacteria;Bacteria\_none;Campylobacterota;Epsilonproteobacteria;Campylobacterales;Campylobacteraceae;Campylobacter;Campylobacter concisus |
| Campylobacter cuniculorum | 0 | 0 | 0 | 0 | 0 | 0 | 0 | 1 | 0 | 0 | 0 | 0 | 0 | 1 | Bacteria | Bacteria\_none | Campylobacterota | Epsilonproteobacteria | Campylobacterales | Campylobacteraceae | Campylobacter | Bacteria;Bacteria\_none;Campylobacterota;Epsilonproteobacteria;Campylobacterales;Campylobacteraceae;Campylobacter;Campylobacter cuniculorum |
| Campylobacter curvus | 0 | 0 | 1 | 0 | 0 | 0 | 1 | 0 | 0 | 0 | 0 | 0 | 0 | 2 | Bacteria | Bacteria\_none | Campylobacterota | Epsilonproteobacteria | Campylobacterales | Campylobacteraceae | Campylobacter | Bacteria;Bacteria\_none;Campylobacterota;Epsilonproteobacteria;Campylobacterales;Campylobacteraceae;Campylobacter;Campylobacter curvus |
| Campylobacter gracilis | 0 | 2 | 1 | 1 | 0 | 0 | 0 | 0 | 0 | 2 | 0 | 2 | 0 | 8 | Bacteria | Bacteria\_none | Campylobacterota | Epsilonproteobacteria | Campylobacterales | Campylobacteraceae | Campylobacter | Bacteria;Bacteria\_none;Campylobacterota;Epsilonproteobacteria;Campylobacterales;Campylobacteraceae;Campylobacter;Campylobacter gracilis |
| Campylobacter hyointestinalis | 0 | 0 | 0 | 0 | 0 | 0 | 0 | 0 | 0 | 0 | 0 | 1 | 0 | 1 | Bacteria | Bacteria\_none | Campylobacterota | Epsilonproteobacteria | Campylobacterales | Campylobacteraceae | Campylobacter | Bacteria;Bacteria\_none;Campylobacterota;Epsilonproteobacteria;Campylobacterales;Campylobacteraceae;Campylobacter;Campylobacter hyointestinalis |
| Campylobacter insulaenigrae | 0 | 0 | 0 | 0 | 0 | 2 | 0 | 0 | 0 | 0 | 0 | 0 | 0 | 2 | Bacteria | Bacteria\_none | Campylobacterota | Epsilonproteobacteria | Campylobacterales | Campylobacteraceae | Campylobacter | Bacteria;Bacteria\_none;Campylobacterota;Epsilonproteobacteria;Campylobacterales;Campylobacteraceae;Campylobacter;Campylobacter insulaenigrae |
| Campylobacter lanienae | 0 | 0 | 0 | 0 | 0 | 0 | 0 | 0 | 0 | 0 | 0 | 0 | 1 | 1 | Bacteria | Bacteria\_none | Campylobacterota | Epsilonproteobacteria | Campylobacterales | Campylobacteraceae | Campylobacter | Bacteria;Bacteria\_none;Campylobacterota;Epsilonproteobacteria;Campylobacterales;Campylobacteraceae;Campylobacter;Campylobacter lanienae |
| Campylobacter peloridis | 0 | 0 | 0 | 1 | 0 | 0 | 0 | 0 | 0 | 0 | 0 | 0 | 0 | 1 | Bacteria | Bacteria\_none | Campylobacterota | Epsilonproteobacteria | Campylobacterales | Campylobacteraceae | Campylobacter | Bacteria;Bacteria\_none;Campylobacterota;Epsilonproteobacteria;Campylobacterales;Campylobacteraceae;Campylobacter;Campylobacter peloridis |
| Campylobacter pinnipediorum | 0 | 0 | 0 | 0 | 0 | 0 | 0 | 0 | 0 | 0 | 0 | 1 | 0 | 1 | Bacteria | Bacteria\_none | Campylobacterota | Epsilonproteobacteria | Campylobacterales | Campylobacteraceae | Campylobacter | Bacteria;Bacteria\_none;Campylobacterota;Epsilonproteobacteria;Campylobacterales;Campylobacteraceae;Campylobacter;Campylobacter pinnipediorum |
| Campylobacter rectus | 0 | 10 | 1 | 2 | 0 | 4 | 0 | 6 | 0 | 1 | 1 | 7 | 7 | 39 | Bacteria | Bacteria\_none | Campylobacterota | Epsilonproteobacteria | Campylobacterales | Campylobacteraceae | Campylobacter | Bacteria;Bacteria\_none;Campylobacterota;Epsilonproteobacteria;Campylobacterales;Campylobacteraceae;Campylobacter;Campylobacter rectus |
| Campylobacter showae | 0 | 7 | 10 | 6 | 5 | 2 | 0 | 1 | 2 | 6 | 0 | 1 | 4 | 44 | Bacteria | Bacteria\_none | Campylobacterota | Epsilonproteobacteria | Campylobacterales | Campylobacteraceae | Campylobacter | Bacteria;Bacteria\_none;Campylobacterota;Epsilonproteobacteria;Campylobacterales;Campylobacteraceae;Campylobacter;Campylobacter showae |
| Campylobacter sp. RM16187 | 0 | 0 | 0 | 0 | 0 | 0 | 0 | 1 | 0 | 0 | 0 | 0 | 0 | 1 | Bacteria | Bacteria\_none | Campylobacterota | Epsilonproteobacteria | Campylobacterales | Campylobacteraceae | Campylobacter | Bacteria;Bacteria\_none;Campylobacterota;Epsilonproteobacteria;Campylobacterales;Campylobacteraceae;Campylobacter;Campylobacter sp. RM16187 |
| Campylobacter sp. RM5004 | 0 | 0 | 0 | 0 | 0 | 0 | 1 | 0 | 0 | 0 | 0 | 0 | 0 | 1 | Bacteria | Bacteria\_none | Campylobacterota | Epsilonproteobacteria | Campylobacterales | Campylobacteraceae | Campylobacter | Bacteria;Bacteria\_none;Campylobacterota;Epsilonproteobacteria;Campylobacterales;Campylobacteraceae;Campylobacter;Campylobacter sp. RM5004 |
| Campylobacter ureolyticus | 0 | 1 | 0 | 0 | 0 | 0 | 0 | 0 | 0 | 0 | 0 | 0 | 1 | 2 | Bacteria | Bacteria\_none | Campylobacterota | Epsilonproteobacteria | Campylobacterales | Campylobacteraceae | Campylobacter | Bacteria;Bacteria\_none;Campylobacterota;Epsilonproteobacteria;Campylobacterales;Campylobacteraceae;Campylobacter;Campylobacter ureolyticus |
| Candidatus Campylobacter infans | 0 | 0 | 0 | 0 | 1 | 0 | 0 | 0 | 0 | 0 | 0 | 0 | 0 | 1 | Bacteria | Bacteria\_none | Campylobacterota | Epsilonproteobacteria | Campylobacterales | Campylobacteraceae | Campylobacter | Bacteria;Bacteria\_none;Campylobacterota;Epsilonproteobacteria;Campylobacterales;Campylobacteraceae;Campylobacter;Candidatus Campylobacter infans |
| Helicobacter canadensis | 0 | 0 | 0 | 0 | 0 | 0 | 0 | 0 | 0 | 0 | 0 | 0 | 1 | 1 | Bacteria | Bacteria\_none | Campylobacterota | Epsilonproteobacteria | Campylobacterales | Helicobacteraceae | Helicobacter | Bacteria;Bacteria\_none;Campylobacterota;Epsilonproteobacteria;Campylobacterales;Helicobacteraceae;Helicobacter;Helicobacter canadensis |
| Helicobacter heilmannii | 0 | 0 | 0 | 0 | 0 | 1 | 0 | 0 | 0 | 0 | 0 | 0 | 0 | 1 | Bacteria | Bacteria\_none | Campylobacterota | Epsilonproteobacteria | Campylobacterales | Helicobacteraceae | Helicobacter | Bacteria;Bacteria\_none;Campylobacterota;Epsilonproteobacteria;Campylobacterales;Helicobacteraceae;Helicobacter;Helicobacter heilmannii |
| Helicobacter pylori | 0 | 0 | 0 | 0 | 1 | 0 | 0 | 0 | 0 | 0 | 0 | 0 | 0 | 1 | Bacteria | Bacteria\_none | Campylobacterota | Epsilonproteobacteria | Campylobacterales | Helicobacteraceae | Helicobacter | Bacteria;Bacteria\_none;Campylobacterota;Epsilonproteobacteria;Campylobacterales;Helicobacteraceae;Helicobacter;Helicobacter pylori |
| Sulfurospirillum barnesii | 0 | 0 | 0 | 0 | 0 | 0 | 0 | 0 | 0 | 1 | 0 | 0 | 0 | 1 | Bacteria | Bacteria\_none | Campylobacterota | Epsilonproteobacteria | Campylobacterales | Sulfurospirillaceae | Sulfurospirillum | Bacteria;Bacteria\_none;Campylobacterota;Epsilonproteobacteria;Campylobacterales;Sulfurospirillaceae;Sulfurospirillum;Sulfurospirillum barnesii |
| Sulfurovum indicum | 1 | 0 | 0 | 0 | 0 | 0 | 0 | 0 | 0 | 0 | 0 | 0 | 0 | 1 | Bacteria | Bacteria\_none | Campylobacterota | Epsilonproteobacteria | Campylobacterales | Sulfurovaceae | Sulfurovum | Bacteria;Bacteria\_none;Campylobacterota;Epsilonproteobacteria;Campylobacterales;Sulfurovaceae;Sulfurovum;Sulfurovum indicum |
| Candidatus Minimicrobia sp. QA0096 | 0 | 0 | 0 | 0 | 2 | 6 | 0 | 0 | 0 | 0 | 0 | 0 | 2 | 10 | Bacteria | Bacteria\_none | Candidatus Saccharibacteria | Candidatus Saccharibacteria\_Incertae\_sedis | Candidatus Saccharibacteria\_Incertae\_sedis | Candidatus Saccharibacteria\_Incertae\_sedis | Candidatus Minimicrobia | Bacteria;Bacteria\_none;Candidatus Saccharibacteria;Candidatus Saccharibacteria\_Incertae\_sedis;Candidatus Saccharibacteria\_Incertae\_sedis;Candidatus Saccharibacteria\_Incertae\_sedis;Candidatus Minimicrobia;Candidatus Minimicrobia sp. QA0096 |
| Candidatus Minimicrobia vallesae | 0 | 0 | 0 | 0 | 0 | 13 | 0 | 0 | 0 | 0 | 1 | 1 | 3 | 18 | Bacteria | Bacteria\_none | Candidatus Saccharibacteria | Candidatus Saccharibacteria\_Incertae\_sedis | Candidatus Saccharibacteria\_Incertae\_sedis | Candidatus Saccharibacteria\_Incertae\_sedis | Candidatus Minimicrobia | Bacteria;Bacteria\_none;Candidatus Saccharibacteria;Candidatus Saccharibacteria\_Incertae\_sedis;Candidatus Saccharibacteria\_Incertae\_sedis;Candidatus Saccharibacteria\_Incertae\_sedis;Candidatus Minimicrobia;Candidatus Minimicrobia vallesae |
| Candidatus Mycosynbacter amalyticus | 0 | 0 | 0 | 0 | 0 | 1 | 0 | 0 | 0 | 0 | 0 | 0 | 0 | 1 | Bacteria | Bacteria\_none | Candidatus Saccharibacteria | Candidatus Saccharibacteria\_Incertae\_sedis | Candidatus Saccharibacteria\_Incertae\_sedis | Candidatus Saccharibacteria\_Incertae\_sedis | Candidatus Mycosynbacter | Bacteria;Bacteria\_none;Candidatus Saccharibacteria;Candidatus Saccharibacteria\_Incertae\_sedis;Candidatus Saccharibacteria\_Incertae\_sedis;Candidatus Saccharibacteria\_Incertae\_sedis;Candidatus Mycosynbacter;Candidatus Mycosynbacter amalyticus |
| Candidatus Nanosynbacter featherlites | 0 | 1 | 4 | 0 | 2 | 1 | 1 | 0 | 0 | 2 | 25 | 2 | 3 | 41 | Bacteria | Bacteria\_none | Candidatus Saccharibacteria | Candidatus Saccharimonadia | Candidatus Nanosynbacterales | Candidatus Nanosynbacteraceae | Candidatus Nanosynbacter | Bacteria;Bacteria\_none;Candidatus Saccharibacteria;Candidatus Saccharimonadia;Candidatus Nanosynbacterales;Candidatus Nanosynbacteraceae;Candidatus Nanosynbacter;Candidatus Nanosynbacter featherlites |
| Candidatus Nanosynbacter lyticus | 0 | 2 | 1 | 0 | 3 | 5 | 0 | 1 | 0 | 0 | 65 | 2 | 7 | 86 | Bacteria | Bacteria\_none | Candidatus Saccharibacteria | Candidatus Saccharimonadia | Candidatus Nanosynbacterales | Candidatus Nanosynbacteraceae | Candidatus Nanosynbacter | Bacteria;Bacteria\_none;Candidatus Saccharibacteria;Candidatus Saccharimonadia;Candidatus Nanosynbacterales;Candidatus Nanosynbacteraceae;Candidatus Nanosynbacter;Candidatus Nanosynbacter lyticus |
| Candidatus Nanosynbacter sp. HMT-352 | 0 | 5 | 0 | 0 | 5 | 159 | 0 | 0 | 3 | 0 | 2 | 2 | 38 | 214 | Bacteria | Bacteria\_none | Candidatus Saccharibacteria | Candidatus Saccharimonadia | Candidatus Nanosynbacterales | Candidatus Nanosynbacteraceae | Candidatus Nanosynbacter | Bacteria;Bacteria\_none;Candidatus Saccharibacteria;Candidatus Saccharimonadia;Candidatus Nanosynbacterales;Candidatus Nanosynbacteraceae;Candidatus Nanosynbacter;Candidatus Nanosynbacter sp. HMT-352 |
| Candidatus Saccharimonas aalborgensis | 0 | 0 | 0 | 0 | 1 | 0 | 0 | 1 | 0 | 0 | 1 | 0 | 0 | 3 | Bacteria | Bacteria\_none | Candidatus Saccharibacteria | Candidatus Saccharimonadia | Candidatus Saccharimonadales | Candidatus Saccharimonadaceae | Candidatus Saccharimonas | Bacteria;Bacteria\_none;Candidatus Saccharibacteria;Candidatus Saccharimonadia;Candidatus Saccharimonadales;Candidatus Saccharimonadaceae;Candidatus Saccharimonas;Candidatus Saccharimonas aalborgensis |
| Candidatus Protochlamydia naegleriophila | 0 | 0 | 0 | 0 | 0 | 0 | 0 | 0 | 0 | 0 | 0 | 0 | 1 | 1 | Bacteria | Bacteria\_none | Chlamydiota | Chlamydiia | Parachlamydiales | Parachlamydiaceae | Candidatus Protochlamydia | Bacteria;Bacteria\_none;Chlamydiota;Chlamydiia;Parachlamydiales;Parachlamydiaceae;Candidatus Protochlamydia;Candidatus Protochlamydia naegleriophila |
| Chloroherpeton thalassium | 0 | 0 | 0 | 0 | 0 | 1 | 0 | 0 | 0 | 0 | 0 | 0 | 0 | 1 | Bacteria | Bacteria\_none | Chlorobiota | Chlorobiia | Chlorobiales | Chloroherpetonaceae | Chloroherpeton | Bacteria;Bacteria\_none;Chlorobiota;Chlorobiia;Chlorobiales;Chloroherpetonaceae;Chloroherpeton;Chloroherpeton thalassium |
| Candidatus Chlorohelix allophototropha | 0 | 0 | 0 | 0 | 0 | 0 | 0 | 0 | 0 | 0 | 1 | 0 | 0 | 1 | Bacteria | Bacteria\_none | Chloroflexota | Chloroflexia | Candidatus Chloroheliales | Candidatus Chloroheliaceae | Candidatus Chlorohelix | Bacteria;Bacteria\_none;Chloroflexota;Chloroflexia;Candidatus Chloroheliales;Candidatus Chloroheliaceae;Candidatus Chlorohelix;Candidatus Chlorohelix allophototropha |
| Candidatus Lucifugimonas marina | 0 | 0 | 0 | 0 | 0 | 0 | 0 | 0 | 0 | 0 | 0 | 0 | 1 | 1 | Bacteria | Bacteria\_none | Chloroflexota | Dehalococcoidia | Candidatus Lucifugimonadales | Candidatus Lucifugimonadaceae | Candidatus Lucifugimonas | Bacteria;Bacteria\_none;Chloroflexota;Dehalococcoidia;Candidatus Lucifugimonadales;Candidatus Lucifugimonadaceae;Candidatus Lucifugimonas;Candidatus Lucifugimonas marina |
| cyanobacterium endosymbiont of Epithemia clementina EcSB | 1 | 0 | 0 | 0 | 0 | 0 | 0 | 0 | 0 | 0 | 0 | 0 | 0 | 1 | Bacteria | Bacteria\_none | Cyanobacteriota | Cyanobacteriota\_Incertae\_sedis | Cyanobacteriota\_Incertae\_sedis | Cyanobacteriota\_Incertae\_sedis | Cyanobacteriota\_Incertae\_sedis | Bacteria;Bacteria\_none;Cyanobacteriota;Cyanobacteriota\_Incertae\_sedis;Cyanobacteriota\_Incertae\_sedis;Cyanobacteriota\_Incertae\_sedis;Cyanobacteriota\_Incertae\_sedis;cyanobacterium endosymbiont of Epithemia clementina EcSB |
| Gloeocapsa sp. PCC 7428 | 0 | 0 | 1 | 0 | 0 | 0 | 0 | 0 | 0 | 0 | 0 | 0 | 0 | 1 | Bacteria | Bacteria\_none | Cyanobacteriota | Cyanophyceae | Chroococcales | Chroococcaceae | Gloeocapsa | Bacteria;Bacteria\_none;Cyanobacteriota;Cyanophyceae;Chroococcales;Chroococcaceae;Gloeocapsa;Gloeocapsa sp. PCC 7428 |
| Geminocystis herdmanii | 0 | 0 | 0 | 0 | 0 | 0 | 0 | 0 | 0 | 0 | 1 | 0 | 0 | 1 | Bacteria | Bacteria\_none | Cyanobacteriota | Cyanophyceae | Chroococcales | Geminocystaceae | Geminocystis | Bacteria;Bacteria\_none;Cyanobacteriota;Cyanophyceae;Chroococcales;Geminocystaceae;Geminocystis;Geminocystis herdmanii |
| Gloeobacter morelensis | 0 | 0 | 0 | 0 | 0 | 0 | 1 | 0 | 0 | 0 | 0 | 0 | 0 | 1 | Bacteria | Bacteria\_none | Cyanobacteriota | Cyanophyceae | Gloeobacterales | Gloeobacteraceae | Gloeobacter | Bacteria;Bacteria\_none;Cyanobacteriota;Cyanophyceae;Gloeobacterales;Gloeobacteraceae;Gloeobacter;Gloeobacter morelensis |
| Cylindrospermopsis raciborskii | 0 | 0 | 0 | 1 | 0 | 0 | 0 | 0 | 0 | 0 | 0 | 0 | 0 | 1 | Bacteria | Bacteria\_none | Cyanobacteriota | Cyanophyceae | Nostocales | Aphanizomenonaceae | Cylindrospermopsis | Bacteria;Bacteria\_none;Cyanobacteriota;Cyanophyceae;Nostocales;Aphanizomenonaceae;Cylindrospermopsis;Cylindrospermopsis raciborskii |
| Nostoc sp. 'Lobaria pulmonaria (5183) cyanobiont' | 0 | 0 | 0 | 0 | 0 | 0 | 0 | 0 | 0 | 0 | 1 | 0 | 0 | 1 | Bacteria | Bacteria\_none | Cyanobacteriota | Cyanophyceae | Nostocales | Nostocaceae | Nostoc | Bacteria;Bacteria\_none;Cyanobacteriota;Cyanophyceae;Nostocales;Nostocaceae;Nostoc;Nostoc sp. 'Lobaria pulmonaria (5183) cyanobiont' |
| Nostoc sp. 'Peltigera membranacea cyanobiont' N6 | 0 | 1 | 0 | 0 | 0 | 0 | 0 | 0 | 0 | 0 | 0 | 0 | 0 | 1 | Bacteria | Bacteria\_none | Cyanobacteriota | Cyanophyceae | Nostocales | Nostocaceae | Nostoc | Bacteria;Bacteria\_none;Cyanobacteriota;Cyanophyceae;Nostocales;Nostocaceae;Nostoc;Nostoc sp. 'Peltigera membranacea cyanobiont' N6 |
| Nostoc sp. NIES-3756 | 0 | 0 | 0 | 0 | 0 | 0 | 0 | 0 | 0 | 1 | 0 | 0 | 1 | 2 | Bacteria | Bacteria\_none | Cyanobacteriota | Cyanophyceae | Nostocales | Nostocaceae | Nostoc | Bacteria;Bacteria\_none;Cyanobacteriota;Cyanophyceae;Nostocales;Nostocaceae;Nostoc;Nostoc sp. NIES-3756 |
| Nostoc sp. TCL26-01 | 0 | 0 | 0 | 0 | 0 | 0 | 0 | 0 | 0 | 1 | 0 | 0 | 0 | 1 | Bacteria | Bacteria\_none | Cyanobacteriota | Cyanophyceae | Nostocales | Nostocaceae | Nostoc | Bacteria;Bacteria\_none;Cyanobacteriota;Cyanophyceae;Nostocales;Nostocaceae;Nostoc;Nostoc sp. TCL26-01 |
| Nostoc sphaeroides | 0 | 0 | 0 | 0 | 0 | 0 | 0 | 0 | 0 | 0 | 0 | 0 | 1 | 1 | Bacteria | Bacteria\_none | Cyanobacteriota | Cyanophyceae | Nostocales | Nostocaceae | Nostoc | Bacteria;Bacteria\_none;Cyanobacteriota;Cyanophyceae;Nostocales;Nostocaceae;Nostoc;Nostoc sphaeroides |
| Prochlorococcus marinus | 0 | 0 | 0 | 0 | 1 | 0 | 0 | 0 | 0 | 0 | 0 | 0 | 1 | 2 | Bacteria | Bacteria\_none | Cyanobacteriota | Cyanophyceae | Synechococcales | Prochlorococcaceae | Prochlorococcus | Bacteria;Bacteria\_none;Cyanobacteriota;Cyanophyceae;Synechococcales;Prochlorococcaceae;Prochlorococcus;Prochlorococcus marinus |
| Synechococcus sp. JA-3-3Ab | 0 | 0 | 0 | 0 | 0 | 1 | 0 | 0 | 0 | 0 | 0 | 0 | 0 | 1 | Bacteria | Bacteria\_none | Cyanobacteriota | Cyanophyceae | Synechococcales | Synechococcaceae | Synechococcus | Bacteria;Bacteria\_none;Cyanobacteriota;Cyanophyceae;Synechococcales;Synechococcaceae;Synechococcus;Synechococcus sp. JA-3-3Ab |
| Calditerrivibrio nitroreducens | 0 | 1 | 0 | 0 | 0 | 0 | 0 | 0 | 0 | 0 | 0 | 0 | 0 | 1 | Bacteria | Bacteria\_none | Deferribacterota | Deferribacteres | Deferribacterales | Calditerrivibrionaceae | Calditerrivibrio | Bacteria;Bacteria\_none;Deferribacterota;Deferribacteres;Deferribacterales;Calditerrivibrionaceae;Calditerrivibrio;Calditerrivibrio nitroreducens |
| Mucispirillum schaedleri | 0 | 0 | 0 | 0 | 0 | 0 | 0 | 0 | 0 | 0 | 0 | 0 | 1 | 1 | Bacteria | Bacteria\_none | Deferribacterota | Deferribacteres | Deferribacterales | Mucispirillaceae | Mucispirillum | Bacteria;Bacteria\_none;Deferribacterota;Deferribacteres;Deferribacterales;Mucispirillaceae;Mucispirillum;Mucispirillum schaedleri |
| Cetobacterium somerae | 0 | 0 | 0 | 0 | 1 | 0 | 0 | 0 | 0 | 0 | 0 | 0 | 1 | 2 | Bacteria | Bacteria\_none | Fusobacteriota | Fusobacteriia | Fusobacteriales | Fusobacteriaceae | Cetobacterium | Bacteria;Bacteria\_none;Fusobacteriota;Fusobacteriia;Fusobacteriales;Fusobacteriaceae;Cetobacterium;Cetobacterium somerae |
| Fusobacterium animalis | 0 | 5 | 6 | 22 | 6 | 1 | 2 | 17 | 0 | 1 | 67 | 25 | 75 | 227 | Bacteria | Bacteria\_none | Fusobacteriota | Fusobacteriia | Fusobacteriales | Fusobacteriaceae | Fusobacterium | Bacteria;Bacteria\_none;Fusobacteriota;Fusobacteriia;Fusobacteriales;Fusobacteriaceae;Fusobacterium;Fusobacterium animalis |
| Fusobacterium canifelinum | 0 | 1 | 0 | 15 | 0 | 0 | 0 | 2 | 0 | 0 | 5 | 0 | 17 | 40 | Bacteria | Bacteria\_none | Fusobacteriota | Fusobacteriia | Fusobacteriales | Fusobacteriaceae | Fusobacterium | Bacteria;Bacteria\_none;Fusobacteriota;Fusobacteriia;Fusobacteriales;Fusobacteriaceae;Fusobacterium;Fusobacterium canifelinum |
| Fusobacterium gonidiaformans | 0 | 0 | 0 | 0 | 0 | 0 | 0 | 0 | 0 | 0 | 0 | 0 | 1 | 1 | Bacteria | Bacteria\_none | Fusobacteriota | Fusobacteriia | Fusobacteriales | Fusobacteriaceae | Fusobacterium | Bacteria;Bacteria\_none;Fusobacteriota;Fusobacteriia;Fusobacteriales;Fusobacteriaceae;Fusobacterium;Fusobacterium gonidiaformans |
| Fusobacterium hwasookii | 0 | 5 | 1 | 40 | 8 | 1 | 0 | 2 | 0 | 0 | 1 | 2 | 41 | 101 | Bacteria | Bacteria\_none | Fusobacteriota | Fusobacteriia | Fusobacteriales | Fusobacteriaceae | Fusobacterium | Bacteria;Bacteria\_none;Fusobacteriota;Fusobacteriia;Fusobacteriales;Fusobacteriaceae;Fusobacterium;Fusobacterium hwasookii |
| Fusobacterium mortiferum | 0 | 0 | 0 | 0 | 0 | 0 | 0 | 0 | 0 | 0 | 0 | 0 | 2 | 2 | Bacteria | Bacteria\_none | Fusobacteriota | Fusobacteriia | Fusobacteriales | Fusobacteriaceae | Fusobacterium | Bacteria;Bacteria\_none;Fusobacteriota;Fusobacteriia;Fusobacteriales;Fusobacteriaceae;Fusobacterium;Fusobacterium mortiferum |
| Fusobacterium necrophorum | 0 | 1 | 0 | 0 | 0 | 0 | 1 | 2 | 0 | 0 | 0 | 0 | 0 | 4 | Bacteria | Bacteria\_none | Fusobacteriota | Fusobacteriia | Fusobacteriales | Fusobacteriaceae | Fusobacterium | Bacteria;Bacteria\_none;Fusobacteriota;Fusobacteriia;Fusobacteriales;Fusobacteriaceae;Fusobacterium;Fusobacterium necrophorum |
| Fusobacterium nucleatum | 0 | 18 | 8 | 27 | 6 | 6 | 1 | 42 | 0 | 5 | 21 | 12 | 91 | 237 | Bacteria | Bacteria\_none | Fusobacteriota | Fusobacteriia | Fusobacteriales | Fusobacteriaceae | Fusobacterium | Bacteria;Bacteria\_none;Fusobacteriota;Fusobacteriia;Fusobacteriales;Fusobacteriaceae;Fusobacterium;Fusobacterium nucleatum |
| Fusobacterium periodonticum | 0 | 5 | 0 | 12 | 1 | 0 | 0 | 0 | 0 | 1 | 0 | 0 | 10 | 29 | Bacteria | Bacteria\_none | Fusobacteriota | Fusobacteriia | Fusobacteriales | Fusobacteriaceae | Fusobacterium | Bacteria;Bacteria\_none;Fusobacteriota;Fusobacteriia;Fusobacteriales;Fusobacteriaceae;Fusobacterium;Fusobacterium periodonticum |
| Fusobacterium polymorphum | 1 | 61 | 19 | 143 | 11 | 3 | 2 | 109 | 0 | 15 | 41 | 34 | 239 | 678 | Bacteria | Bacteria\_none | Fusobacteriota | Fusobacteriia | Fusobacteriales | Fusobacteriaceae | Fusobacterium | Bacteria;Bacteria\_none;Fusobacteriota;Fusobacteriia;Fusobacteriales;Fusobacteriaceae;Fusobacterium;Fusobacterium polymorphum |
| Fusobacterium pseudoperiodonticum | 0 | 60 | 27 | 466 | 36 | 13 | 1 | 10 | 0 | 16 | 3 | 14 | 281 | 927 | Bacteria | Bacteria\_none | Fusobacteriota | Fusobacteriia | Fusobacteriales | Fusobacteriaceae | Fusobacterium | Bacteria;Bacteria\_none;Fusobacteriota;Fusobacteriia;Fusobacteriales;Fusobacteriaceae;Fusobacterium;Fusobacterium pseudoperiodonticum |
| Fusobacterium sp. oral taxon 203 | 0 | 0 | 0 | 0 | 0 | 0 | 4 | 0 | 0 | 0 | 1 | 0 | 13 | 18 | Bacteria | Bacteria\_none | Fusobacteriota | Fusobacteriia | Fusobacteriales | Fusobacteriaceae | Fusobacterium | Bacteria;Bacteria\_none;Fusobacteriota;Fusobacteriia;Fusobacteriales;Fusobacteriaceae;Fusobacterium;Fusobacterium sp. oral taxon 203 |
| Fusobacterium varium | 0 | 0 | 0 | 0 | 0 | 1 | 1 | 0 | 0 | 0 | 0 | 0 | 0 | 2 | Bacteria | Bacteria\_none | Fusobacteriota | Fusobacteriia | Fusobacteriales | Fusobacteriaceae | Fusobacterium | Bacteria;Bacteria\_none;Fusobacteriota;Fusobacteriia;Fusobacteriales;Fusobacteriaceae;Fusobacterium;Fusobacterium varium |
| Fusobacterium vincentii | 0 | 13 | 0 | 17 | 4 | 2 | 5 | 1 | 0 | 0 | 53 | 19 | 97 | 211 | Bacteria | Bacteria\_none | Fusobacteriota | Fusobacteriia | Fusobacteriales | Fusobacteriaceae | Fusobacterium | Bacteria;Bacteria\_none;Fusobacteriota;Fusobacteriia;Fusobacteriales;Fusobacteriaceae;Fusobacterium;Fusobacterium vincentii |
| Ilyobacter\_uncultured | 0 | 0 | 0 | 0 | 1 | 0 | 0 | 0 | 0 | 0 | 0 | 0 | 0 | 1 | Bacteria | Bacteria\_none | Fusobacteriota | Fusobacteriia | Fusobacteriales | Fusobacteriaceae | Ilyobacter | Bacteria;Bacteria\_none;Fusobacteriota;Fusobacteriia;Fusobacteriales;Fusobacteriaceae;Ilyobacter;Ilyobacter\_uncultured |
| Haliovirga abyssi | 0 | 0 | 0 | 0 | 0 | 0 | 0 | 0 | 0 | 0 | 0 | 0 | 1 | 1 | Bacteria | Bacteria\_none | Fusobacteriota | Fusobacteriia | Fusobacteriales | Haliovirgaceae | Haliovirga | Bacteria;Bacteria\_none;Fusobacteriota;Fusobacteriia;Fusobacteriales;Haliovirgaceae;Haliovirga;Haliovirga abyssi |
| Leptotrichia buccalis | 0 | 12 | 3 | 3 | 4 | 0 | 3 | 5 | 0 | 1 | 1 | 1 | 15 | 48 | Bacteria | Bacteria\_none | Fusobacteriota | Fusobacteriia | Fusobacteriales | Leptotrichiaceae | Leptotrichia | Bacteria;Bacteria\_none;Fusobacteriota;Fusobacteriia;Fusobacteriales;Leptotrichiaceae;Leptotrichia;Leptotrichia buccalis |
| Leptotrichia hofstadii | 0 | 2 | 5 | 4 | 4 | 3 | 2 | 4 | 1 | 0 | 0 | 0 | 11 | 36 | Bacteria | Bacteria\_none | Fusobacteriota | Fusobacteriia | Fusobacteriales | Leptotrichiaceae | Leptotrichia | Bacteria;Bacteria\_none;Fusobacteriota;Fusobacteriia;Fusobacteriales;Leptotrichiaceae;Leptotrichia;Leptotrichia hofstadii |
| Leptotrichia hongkongensis | 0 | 5 | 6 | 0 | 2 | 0 | 3 | 5 | 1 | 26 | 8 | 1 | 15 | 72 | Bacteria | Bacteria\_none | Fusobacteriota | Fusobacteriia | Fusobacteriales | Leptotrichiaceae | Leptotrichia | Bacteria;Bacteria\_none;Fusobacteriota;Fusobacteriia;Fusobacteriales;Leptotrichiaceae;Leptotrichia;Leptotrichia hongkongensis |
| Leptotrichia shahii | 0 | 3 | 3 | 1 | 2 | 0 | 7 | 0 | 0 | 1 | 0 | 0 | 8 | 25 | Bacteria | Bacteria\_none | Fusobacteriota | Fusobacteriia | Fusobacteriales | Leptotrichiaceae | Leptotrichia | Bacteria;Bacteria\_none;Fusobacteriota;Fusobacteriia;Fusobacteriales;Leptotrichiaceae;Leptotrichia;Leptotrichia shahii |
| Leptotrichia sp. HMT-225 | 0 | 1 | 5 | 19 | 3 | 4 | 1 | 58 | 1 | 1 | 0 | 1 | 37 | 131 | Bacteria | Bacteria\_none | Fusobacteriota | Fusobacteriia | Fusobacteriales | Leptotrichiaceae | Leptotrichia | Bacteria;Bacteria\_none;Fusobacteriota;Fusobacteriia;Fusobacteriales;Leptotrichiaceae;Leptotrichia;Leptotrichia sp. HMT-225 |
| Leptotrichia sp. oral taxon 212 | 4 | 44 | 24 | 42 | 54 | 34 | 1 | 284 | 1 | 22 | 26 | 7 | 191 | 734 | Bacteria | Bacteria\_none | Fusobacteriota | Fusobacteriia | Fusobacteriales | Leptotrichiaceae | Leptotrichia | Bacteria;Bacteria\_none;Fusobacteriota;Fusobacteriia;Fusobacteriales;Leptotrichiaceae;Leptotrichia;Leptotrichia sp. oral taxon 212 |
| Leptotrichia sp. oral taxon 218 | 0 | 0 | 7 | 1 | 0 | 9 | 28 | 1 | 0 | 0 | 0 | 1 | 11 | 58 | Bacteria | Bacteria\_none | Fusobacteriota | Fusobacteriia | Fusobacteriales | Leptotrichiaceae | Leptotrichia | Bacteria;Bacteria\_none;Fusobacteriota;Fusobacteriia;Fusobacteriales;Leptotrichiaceae;Leptotrichia;Leptotrichia sp. oral taxon 218 |
| Leptotrichia sp. oral taxon 221 | 0 | 3 | 5 | 1 | 1 | 10 | 25 | 0 | 0 | 0 | 4 | 0 | 14 | 63 | Bacteria | Bacteria\_none | Fusobacteriota | Fusobacteriia | Fusobacteriales | Leptotrichiaceae | Leptotrichia | Bacteria;Bacteria\_none;Fusobacteriota;Fusobacteriia;Fusobacteriales;Leptotrichiaceae;Leptotrichia;Leptotrichia sp. oral taxon 221 |
| Leptotrichia sp. oral taxon 498 | 0 | 1 | 2 | 1 | 0 | 2 | 12 | 0 | 0 | 2 | 0 | 4 | 3 | 27 | Bacteria | Bacteria\_none | Fusobacteriota | Fusobacteriia | Fusobacteriales | Leptotrichiaceae | Leptotrichia | Bacteria;Bacteria\_none;Fusobacteriota;Fusobacteriia;Fusobacteriales;Leptotrichiaceae;Leptotrichia;Leptotrichia sp. oral taxon 498 |
| Leptotrichia sp. oral taxon 847 | 1 | 0 | 5 | 0 | 1 | 4 | 2 | 0 | 0 | 0 | 0 | 0 | 3 | 16 | Bacteria | Bacteria\_none | Fusobacteriota | Fusobacteriia | Fusobacteriales | Leptotrichiaceae | Leptotrichia | Bacteria;Bacteria\_none;Fusobacteriota;Fusobacteriia;Fusobacteriales;Leptotrichiaceae;Leptotrichia;Leptotrichia sp. oral taxon 847 |
| Leptotrichia trevisanii | 0 | 5 | 3 | 6 | 2 | 4 | 10 | 14 | 17 | 3 | 65 | 0 | 50 | 179 | Bacteria | Bacteria\_none | Fusobacteriota | Fusobacteriia | Fusobacteriales | Leptotrichiaceae | Leptotrichia | Bacteria;Bacteria\_none;Fusobacteriota;Fusobacteriia;Fusobacteriales;Leptotrichiaceae;Leptotrichia;Leptotrichia trevisanii |
| Leptotrichia wadei | 11 | 20 | 107 | 6 | 5 | 160 | 338 | 5 | 3 | 36 | 3 | 9 | 308 | 1011 | Bacteria | Bacteria\_none | Fusobacteriota | Fusobacteriia | Fusobacteriales | Leptotrichiaceae | Leptotrichia | Bacteria;Bacteria\_none;Fusobacteriota;Fusobacteriia;Fusobacteriales;Leptotrichiaceae;Leptotrichia;Leptotrichia wadei |
| Pseudoleptotrichia goodfellowii | 0 | 0 | 0 | 14 | 35 | 0 | 0 | 0 | 0 | 2 | 61 | 0 | 32 | 144 | Bacteria | Bacteria\_none | Fusobacteriota | Fusobacteriia | Fusobacteriales | Leptotrichiaceae | Pseudoleptotrichia | Bacteria;Bacteria\_none;Fusobacteriota;Fusobacteriia;Fusobacteriales;Leptotrichiaceae;Pseudoleptotrichia;Pseudoleptotrichia goodfellowii |
| Sebaldella termitidis | 0 | 0 | 0 | 0 | 0 | 0 | 0 | 1 | 0 | 0 | 0 | 0 | 0 | 1 | Bacteria | Bacteria\_none | Fusobacteriota | Fusobacteriia | Fusobacteriales | Leptotrichiaceae | Sebaldella | Bacteria;Bacteria\_none;Fusobacteriota;Fusobacteriia;Fusobacteriales;Leptotrichiaceae;Sebaldella;Sebaldella termitidis |
| Sneathia vaginalis | 0 | 1 | 0 | 0 | 0 | 1 | 0 | 0 | 0 | 0 | 0 | 0 | 0 | 2 | Bacteria | Bacteria\_none | Fusobacteriota | Fusobacteriia | Fusobacteriales | Leptotrichiaceae | Sneathia | Bacteria;Bacteria\_none;Fusobacteriota;Fusobacteriia;Fusobacteriales;Leptotrichiaceae;Sneathia;Sneathia vaginalis |
| Streptobacillus moniliformis | 0 | 0 | 1 | 5 | 0 | 0 | 0 | 0 | 0 | 0 | 0 | 0 | 0 | 6 | Bacteria | Bacteria\_none | Fusobacteriota | Fusobacteriia | Fusobacteriales | Leptotrichiaceae | Streptobacillus | Bacteria;Bacteria\_none;Fusobacteriota;Fusobacteriia;Fusobacteriales;Leptotrichiaceae;Streptobacillus;Streptobacillus moniliformis |
| Ignavibacterium sp. | 0 | 0 | 0 | 0 | 0 | 0 | 0 | 0 | 0 | 0 | 0 | 0 | 1 | 1 | Bacteria | Bacteria\_none | Ignavibacteriota | Ignavibacteria | Ignavibacteriales | Ignavibacteriaceae | Ignavibacterium | Bacteria;Bacteria\_none;Ignavibacteriota;Ignavibacteria;Ignavibacteriales;Ignavibacteriaceae;Ignavibacterium;Ignavibacterium sp. |
| Acholeplasma hippikon | 0 | 0 | 0 | 0 | 1 | 0 | 0 | 0 | 0 | 0 | 0 | 0 | 0 | 1 | Bacteria | Bacteria\_none | Mycoplasmatota | Mollicutes | Acholeplasmatales | Acholeplasmataceae | Acholeplasma | Bacteria;Bacteria\_none;Mycoplasmatota;Mollicutes;Acholeplasmatales;Acholeplasmataceae;Acholeplasma;Acholeplasma hippikon |
| Mariniplasma anaerobium | 0 | 0 | 0 | 0 | 0 | 1 | 0 | 0 | 0 | 0 | 0 | 0 | 0 | 1 | Bacteria | Bacteria\_none | Mycoplasmatota | Mollicutes | Acholeplasmatales | Acholeplasmataceae | Mariniplasma | Bacteria;Bacteria\_none;Mycoplasmatota;Mollicutes;Acholeplasmatales;Acholeplasmataceae;Mariniplasma;Mariniplasma anaerobium |
| Spiroplasma culicicola | 0 | 0 | 0 | 0 | 1 | 0 | 0 | 0 | 0 | 0 | 0 | 0 | 0 | 1 | Bacteria | Bacteria\_none | Mycoplasmatota | Mollicutes | Entomoplasmatales | Spiroplasmataceae | Spiroplasma | Bacteria;Bacteria\_none;Mycoplasmatota;Mollicutes;Entomoplasmatales;Spiroplasmataceae;Spiroplasma;Spiroplasma culicicola |
| Spiroplasma endosymbiont of 'Nebria riversi' | 0 | 0 | 0 | 0 | 0 | 1 | 0 | 0 | 0 | 0 | 0 | 0 | 0 | 1 | Bacteria | Bacteria\_none | Mycoplasmatota | Mollicutes | Entomoplasmatales | Spiroplasmataceae | Spiroplasma | Bacteria;Bacteria\_none;Mycoplasmatota;Mollicutes;Entomoplasmatales;Spiroplasmataceae;Spiroplasma;Spiroplasma endosymbiont of 'Nebria riversi' |
| Spiroplasma endosymbiont of Agriotes lineatus | 0 | 0 | 0 | 0 | 0 | 0 | 0 | 0 | 0 | 0 | 0 | 0 | 1 | 1 | Bacteria | Bacteria\_none | Mycoplasmatota | Mollicutes | Entomoplasmatales | Spiroplasmataceae | Spiroplasma | Bacteria;Bacteria\_none;Mycoplasmatota;Mollicutes;Entomoplasmatales;Spiroplasmataceae;Spiroplasma;Spiroplasma endosymbiont of Agriotes lineatus |
| Spiroplasma endosymbiont of Cantharis nigra | 0 | 0 | 0 | 0 | 0 | 1 | 0 | 0 | 0 | 0 | 0 | 0 | 0 | 1 | Bacteria | Bacteria\_none | Mycoplasmatota | Mollicutes | Entomoplasmatales | Spiroplasmataceae | Spiroplasma | Bacteria;Bacteria\_none;Mycoplasmatota;Mollicutes;Entomoplasmatales;Spiroplasmataceae;Spiroplasma;Spiroplasma endosymbiont of Cantharis nigra |
| Spiroplasma endosymbiont of Dromius quadrimaculatus | 0 | 0 | 0 | 0 | 0 | 0 | 0 | 0 | 0 | 0 | 0 | 0 | 1 | 1 | Bacteria | Bacteria\_none | Mycoplasmatota | Mollicutes | Entomoplasmatales | Spiroplasmataceae | Spiroplasma | Bacteria;Bacteria\_none;Mycoplasmatota;Mollicutes;Entomoplasmatales;Spiroplasmataceae;Spiroplasma;Spiroplasma endosymbiont of Dromius quadrimaculatus |
| Spiroplasma gladiatoris | 0 | 0 | 0 | 0 | 0 | 0 | 0 | 0 | 0 | 1 | 0 | 0 | 0 | 1 | Bacteria | Bacteria\_none | Mycoplasmatota | Mollicutes | Entomoplasmatales | Spiroplasmataceae | Spiroplasma | Bacteria;Bacteria\_none;Mycoplasmatota;Mollicutes;Entomoplasmatales;Spiroplasmataceae;Spiroplasma;Spiroplasma gladiatoris |
| Spiroplasma monobiae | 0 | 0 | 0 | 1 | 0 | 0 | 0 | 0 | 0 | 0 | 0 | 0 | 0 | 1 | Bacteria | Bacteria\_none | Mycoplasmatota | Mollicutes | Entomoplasmatales | Spiroplasmataceae | Spiroplasma | Bacteria;Bacteria\_none;Mycoplasmatota;Mollicutes;Entomoplasmatales;Spiroplasmataceae;Spiroplasma;Spiroplasma monobiae |
| Spiroplasma syrphidicola | 0 | 0 | 0 | 0 | 0 | 0 | 0 | 0 | 0 | 0 | 0 | 0 | 1 | 1 | Bacteria | Bacteria\_none | Mycoplasmatota | Mollicutes | Entomoplasmatales | Spiroplasmataceae | Spiroplasma | Bacteria;Bacteria\_none;Mycoplasmatota;Mollicutes;Entomoplasmatales;Spiroplasmataceae;Spiroplasma;Spiroplasma syrphidicola |
| Spiroplasma tabanidicola | 0 | 0 | 0 | 0 | 0 | 1 | 0 | 0 | 0 | 0 | 0 | 0 | 0 | 1 | Bacteria | Bacteria\_none | Mycoplasmatota | Mollicutes | Entomoplasmatales | Spiroplasmataceae | Spiroplasma | Bacteria;Bacteria\_none;Mycoplasmatota;Mollicutes;Entomoplasmatales;Spiroplasmataceae;Spiroplasma;Spiroplasma tabanidicola |
| Mycoplasma anserisalpingitidis | 0 | 0 | 0 | 0 | 0 | 0 | 0 | 0 | 0 | 0 | 0 | 0 | 1 | 1 | Bacteria | Bacteria\_none | Mycoplasmatota | Mollicutes | Mycoplasmatales | Mycoplasmataceae | Mycoplasma | Bacteria;Bacteria\_none;Mycoplasmatota;Mollicutes;Mycoplasmatales;Mycoplasmataceae;Mycoplasma;Mycoplasma anserisalpingitidis |
| Mycoplasma miroungirhinis | 0 | 0 | 2 | 0 | 0 | 1 | 0 | 0 | 0 | 0 | 0 | 0 | 0 | 3 | Bacteria | Bacteria\_none | Mycoplasmatota | Mollicutes | Mycoplasmatales | Mycoplasmataceae | Mycoplasma | Bacteria;Bacteria\_none;Mycoplasmatota;Mollicutes;Mycoplasmatales;Mycoplasmataceae;Mycoplasma;Mycoplasma miroungirhinis |
| Mycoplasma seminis | 0 | 1 | 0 | 0 | 0 | 0 | 0 | 0 | 0 | 0 | 0 | 0 | 0 | 1 | Bacteria | Bacteria\_none | Mycoplasmatota | Mollicutes | Mycoplasmatales | Mycoplasmataceae | Mycoplasma | Bacteria;Bacteria\_none;Mycoplasmatota;Mollicutes;Mycoplasmatales;Mycoplasmataceae;Mycoplasma;Mycoplasma seminis |
| Mycoplasma struthionis | 0 | 0 | 1 | 0 | 0 | 0 | 0 | 0 | 0 | 0 | 0 | 0 | 0 | 1 | Bacteria | Bacteria\_none | Mycoplasmatota | Mollicutes | Mycoplasmatales | Mycoplasmataceae | Mycoplasma | Bacteria;Bacteria\_none;Mycoplasmatota;Mollicutes;Mycoplasmatales;Mycoplasmataceae;Mycoplasma;Mycoplasma struthionis |
| endosymbiont DhMRE of Dentiscutata heterogama | 0 | 0 | 0 | 0 | 0 | 0 | 0 | 0 | 0 | 0 | 1 | 0 | 0 | 1 | Bacteria | Bacteria\_none | Mycoplasmatota | Mollicutes | Mycoplasmatales | Mycoplasmatales\_Incertae\_sedis | Mycoplasmatales\_Incertae\_sedis | Bacteria;Bacteria\_none;Mycoplasmatota;Mollicutes;Mycoplasmatales;Mycoplasmatales\_Incertae\_sedis;Mycoplasmatales\_Incertae\_sedis;endosymbiont DhMRE of Dentiscutata heterogama |
| Metamycoplasma alkalescens | 0 | 0 | 0 | 0 | 0 | 0 | 0 | 0 | 0 | 0 | 0 | 0 | 1 | 1 | Bacteria | Bacteria\_none | Mycoplasmatota | Mycoplasmatota\_Incertae\_sedis | Mycoplasmoidales | Metamycoplasmataceae | Metamycoplasma | Bacteria;Bacteria\_none;Mycoplasmatota;Mycoplasmatota\_Incertae\_sedis;Mycoplasmoidales;Metamycoplasmataceae;Metamycoplasma;Metamycoplasma alkalescens |
| Metamycoplasma arthritidis | 0 | 0 | 1 | 0 | 0 | 0 | 0 | 0 | 0 | 0 | 0 | 1 | 0 | 2 | Bacteria | Bacteria\_none | Mycoplasmatota | Mycoplasmatota\_Incertae\_sedis | Mycoplasmoidales | Metamycoplasmataceae | Metamycoplasma | Bacteria;Bacteria\_none;Mycoplasmatota;Mycoplasmatota\_Incertae\_sedis;Mycoplasmoidales;Metamycoplasmataceae;Metamycoplasma;Metamycoplasma arthritidis |
| Metamycoplasma cloacale | 0 | 0 | 0 | 0 | 0 | 0 | 0 | 0 | 0 | 0 | 0 | 0 | 1 | 1 | Bacteria | Bacteria\_none | Mycoplasmatota | Mycoplasmatota\_Incertae\_sedis | Mycoplasmoidales | Metamycoplasmataceae | Metamycoplasma | Bacteria;Bacteria\_none;Mycoplasmatota;Mycoplasmatota\_Incertae\_sedis;Mycoplasmoidales;Metamycoplasmataceae;Metamycoplasma;Metamycoplasma cloacale |
| Metamycoplasma equirhinis | 0 | 0 | 1 | 0 | 0 | 0 | 0 | 0 | 0 | 0 | 0 | 1 | 0 | 2 | Bacteria | Bacteria\_none | Mycoplasmatota | Mycoplasmatota\_Incertae\_sedis | Mycoplasmoidales | Metamycoplasmataceae | Metamycoplasma | Bacteria;Bacteria\_none;Mycoplasmatota;Mycoplasmatota\_Incertae\_sedis;Mycoplasmoidales;Metamycoplasmataceae;Metamycoplasma;Metamycoplasma equirhinis |
| Metamycoplasma gateae | 0 | 3 | 0 | 0 | 0 | 0 | 0 | 0 | 0 | 0 | 0 | 1 | 0 | 4 | Bacteria | Bacteria\_none | Mycoplasmatota | Mycoplasmatota\_Incertae\_sedis | Mycoplasmoidales | Metamycoplasmataceae | Metamycoplasma | Bacteria;Bacteria\_none;Mycoplasmatota;Mycoplasmatota\_Incertae\_sedis;Mycoplasmoidales;Metamycoplasmataceae;Metamycoplasma;Metamycoplasma gateae |
| Metamycoplasma hominis | 0 | 1 | 1 | 0 | 0 | 0 | 0 | 0 | 0 | 0 | 0 | 0 | 1 | 3 | Bacteria | Bacteria\_none | Mycoplasmatota | Mycoplasmatota\_Incertae\_sedis | Mycoplasmoidales | Metamycoplasmataceae | Metamycoplasma | Bacteria;Bacteria\_none;Mycoplasmatota;Mycoplasmatota\_Incertae\_sedis;Mycoplasmoidales;Metamycoplasmataceae;Metamycoplasma;Metamycoplasma hominis |
| Metamycoplasma hyosynoviae | 0 | 0 | 2 | 0 | 0 | 0 | 0 | 0 | 0 | 1 | 0 | 0 | 1 | 4 | Bacteria | Bacteria\_none | Mycoplasmatota | Mycoplasmatota\_Incertae\_sedis | Mycoplasmoidales | Metamycoplasmataceae | Metamycoplasma | Bacteria;Bacteria\_none;Mycoplasmatota;Mycoplasmatota\_Incertae\_sedis;Mycoplasmoidales;Metamycoplasmataceae;Metamycoplasma;Metamycoplasma hyosynoviae |
| Metamycoplasma orale | 0 | 1 | 1 | 0 | 1 | 1 | 0 | 0 | 0 | 0 | 0 | 1 | 1 | 6 | Bacteria | Bacteria\_none | Mycoplasmatota | Mycoplasmatota\_Incertae\_sedis | Mycoplasmoidales | Metamycoplasmataceae | Metamycoplasma | Bacteria;Bacteria\_none;Mycoplasmatota;Mycoplasmatota\_Incertae\_sedis;Mycoplasmoidales;Metamycoplasmataceae;Metamycoplasma;Metamycoplasma orale |
| Metamycoplasma phocicerebrale | 0 | 0 | 0 | 0 | 0 | 1 | 0 | 0 | 0 | 0 | 0 | 0 | 0 | 1 | Bacteria | Bacteria\_none | Mycoplasmatota | Mycoplasmatota\_Incertae\_sedis | Mycoplasmoidales | Metamycoplasmataceae | Metamycoplasma | Bacteria;Bacteria\_none;Mycoplasmatota;Mycoplasmatota\_Incertae\_sedis;Mycoplasmoidales;Metamycoplasmataceae;Metamycoplasma;Metamycoplasma phocicerebrale |
| Metamycoplasma salivarium | 0 | 3 | 28 | 2 | 1 | 4 | 0 | 3 | 0 | 8 | 3 | 1 | 20 | 73 | Bacteria | Bacteria\_none | Mycoplasmatota | Mycoplasmatota\_Incertae\_sedis | Mycoplasmoidales | Metamycoplasmataceae | Metamycoplasma | Bacteria;Bacteria\_none;Mycoplasmatota;Mycoplasmatota\_Incertae\_sedis;Mycoplasmoidales;Metamycoplasmataceae;Metamycoplasma;Metamycoplasma salivarium |
| Metamycoplasma subdolum | 0 | 0 | 0 | 0 | 0 | 0 | 0 | 0 | 0 | 0 | 0 | 1 | 1 | 2 | Bacteria | Bacteria\_none | Mycoplasmatota | Mycoplasmatota\_Incertae\_sedis | Mycoplasmoidales | Metamycoplasmataceae | Metamycoplasma | Bacteria;Bacteria\_none;Mycoplasmatota;Mycoplasmatota\_Incertae\_sedis;Mycoplasmoidales;Metamycoplasmataceae;Metamycoplasma;Metamycoplasma subdolum |
| [Mycoplasma] phocae | 0 | 0 | 1 | 0 | 0 | 0 | 0 | 0 | 0 | 0 | 0 | 0 | 0 | 1 | Bacteria | Bacteria\_none | Mycoplasmatota | Mycoplasmatota\_Incertae\_sedis | Mycoplasmoidales | Metamycoplasmataceae | Metamycoplasma | Bacteria;Bacteria\_none;Mycoplasmatota;Mycoplasmatota\_Incertae\_sedis;Mycoplasmoidales;Metamycoplasmataceae;Metamycoplasma;[Mycoplasma] phocae |
| Mycoplasmopsis anatis | 0 | 0 | 0 | 0 | 0 | 0 | 1 | 0 | 0 | 0 | 0 | 0 | 0 | 1 | Bacteria | Bacteria\_none | Mycoplasmatota | Mycoplasmatota\_Incertae\_sedis | Mycoplasmoidales | Metamycoplasmataceae | Mycoplasmopsis | Bacteria;Bacteria\_none;Mycoplasmatota;Mycoplasmatota\_Incertae\_sedis;Mycoplasmoidales;Metamycoplasmataceae;Mycoplasmopsis;Mycoplasmopsis anatis |
| Mycoplasmopsis citelli | 0 | 0 | 1 | 0 | 0 | 0 | 0 | 0 | 0 | 0 | 0 | 0 | 0 | 1 | Bacteria | Bacteria\_none | Mycoplasmatota | Mycoplasmatota\_Incertae\_sedis | Mycoplasmoidales | Metamycoplasmataceae | Mycoplasmopsis | Bacteria;Bacteria\_none;Mycoplasmatota;Mycoplasmatota\_Incertae\_sedis;Mycoplasmoidales;Metamycoplasmataceae;Mycoplasmopsis;Mycoplasmopsis citelli |
| Mycoplasmopsis columbina | 0 | 0 | 1 | 0 | 0 | 0 | 0 | 0 | 0 | 0 | 0 | 0 | 0 | 1 | Bacteria | Bacteria\_none | Mycoplasmatota | Mycoplasmatota\_Incertae\_sedis | Mycoplasmoidales | Metamycoplasmataceae | Mycoplasmopsis | Bacteria;Bacteria\_none;Mycoplasmatota;Mycoplasmatota\_Incertae\_sedis;Mycoplasmoidales;Metamycoplasmataceae;Mycoplasmopsis;Mycoplasmopsis columbina |
| Mycoplasmopsis cynos | 0 | 1 | 0 | 0 | 0 | 0 | 0 | 0 | 0 | 0 | 0 | 0 | 0 | 1 | Bacteria | Bacteria\_none | Mycoplasmatota | Mycoplasmatota\_Incertae\_sedis | Mycoplasmoidales | Metamycoplasmataceae | Mycoplasmopsis | Bacteria;Bacteria\_none;Mycoplasmatota;Mycoplasmatota\_Incertae\_sedis;Mycoplasmoidales;Metamycoplasmataceae;Mycoplasmopsis;Mycoplasmopsis cynos |
| Mycoplasmopsis fermentans | 0 | 1 | 0 | 0 | 0 | 0 | 0 | 0 | 0 | 0 | 0 | 0 | 0 | 1 | Bacteria | Bacteria\_none | Mycoplasmatota | Mycoplasmatota\_Incertae\_sedis | Mycoplasmoidales | Metamycoplasmataceae | Mycoplasmopsis | Bacteria;Bacteria\_none;Mycoplasmatota;Mycoplasmatota\_Incertae\_sedis;Mycoplasmoidales;Metamycoplasmataceae;Mycoplasmopsis;Mycoplasmopsis fermentans |
| Mycoplasmopsis pulmonis | 0 | 0 | 0 | 0 | 0 | 0 | 0 | 0 | 0 | 0 | 0 | 1 | 0 | 1 | Bacteria | Bacteria\_none | Mycoplasmatota | Mycoplasmatota\_Incertae\_sedis | Mycoplasmoidales | Metamycoplasmataceae | Mycoplasmopsis | Bacteria;Bacteria\_none;Mycoplasmatota;Mycoplasmatota\_Incertae\_sedis;Mycoplasmoidales;Metamycoplasmataceae;Mycoplasmopsis;Mycoplasmopsis pulmonis |
| Malacoplasma iowae | 0 | 0 | 0 | 0 | 1 | 0 | 0 | 0 | 0 | 0 | 0 | 0 | 0 | 1 | Bacteria | Bacteria\_none | Mycoplasmatota | Mycoplasmatota\_Incertae\_sedis | Mycoplasmoidales | Mycoplasmoidaceae | Malacoplasma | Bacteria;Bacteria\_none;Mycoplasmatota;Mycoplasmatota\_Incertae\_sedis;Mycoplasmoidales;Mycoplasmoidaceae;Malacoplasma;Malacoplasma iowae |
| Mycoplasmoides gallisepticum | 0 | 0 | 0 | 0 | 0 | 0 | 0 | 0 | 0 | 0 | 0 | 0 | 1 | 1 | Bacteria | Bacteria\_none | Mycoplasmatota | Mycoplasmatota\_Incertae\_sedis | Mycoplasmoidales | Mycoplasmoidaceae | Mycoplasmoides | Bacteria;Bacteria\_none;Mycoplasmatota;Mycoplasmatota\_Incertae\_sedis;Mycoplasmoidales;Mycoplasmoidaceae;Mycoplasmoides;Mycoplasmoides gallisepticum |
| Corallococcus coralloides | 0 | 0 | 0 | 0 | 0 | 0 | 0 | 0 | 0 | 0 | 0 | 0 | 1 | 1 | Bacteria | Bacteria\_none | Myxococcota | Myxococcia | Myxococcales | Myxococcaceae | Corallococcus | Bacteria;Bacteria\_none;Myxococcota;Myxococcia;Myxococcales;Myxococcaceae;Corallococcus;Corallococcus coralloides |
| Sorangium cellulosum | 0 | 0 | 0 | 0 | 2 | 0 | 0 | 0 | 0 | 0 | 1 | 0 | 0 | 3 | Bacteria | Bacteria\_none | Myxococcota | Myxococcota\_Incertae\_sedis | Polyangiales | Polyangiaceae | Sorangium | Bacteria;Bacteria\_none;Myxococcota;Myxococcota\_Incertae\_sedis;Polyangiales;Polyangiaceae;Sorangium;Sorangium cellulosum |
| Candidatus Nitrospira neomarina | 0 | 0 | 0 | 0 | 0 | 1 | 0 | 1 | 0 | 0 | 0 | 0 | 0 | 2 | Bacteria | Bacteria\_none | Nitrospirota | Nitrospiria | Nitrospirales | Nitrospiraceae | Nitrospira | Bacteria;Bacteria\_none;Nitrospirota;Nitrospiria;Nitrospirales;Nitrospiraceae;Nitrospira;Candidatus Nitrospira neomarina |
| Dissulfurispira thermophila | 0 | 0 | 0 | 0 | 0 | 0 | 0 | 0 | 0 | 0 | 1 | 0 | 0 | 1 | Bacteria | Bacteria\_none | Nitrospirota | Thermodesulfovibrionia | Thermodesulfovibrionales | Dissulfurispiraceae | Dissulfurispira | Bacteria;Bacteria\_none;Nitrospirota;Thermodesulfovibrionia;Thermodesulfovibrionales;Dissulfurispiraceae;Dissulfurispira;Dissulfurispira thermophila |
| Tuwongella immobilis | 0 | 0 | 0 | 0 | 0 | 1 | 0 | 0 | 0 | 0 | 0 | 0 | 0 | 1 | Bacteria | Bacteria\_none | Planctomycetota | Planctomycetia | Gemmatales | Gemmataceae | Tuwongella | Bacteria;Bacteria\_none;Planctomycetota;Planctomycetia;Gemmatales;Gemmataceae;Tuwongella;Tuwongella immobilis |
| Lignipirellula cremea | 1 | 0 | 0 | 0 | 0 | 0 | 0 | 0 | 0 | 0 | 0 | 0 | 0 | 1 | Bacteria | Bacteria\_none | Planctomycetota | Planctomycetia | Pirellulales | Pirellulaceae | Lignipirellula | Bacteria;Bacteria\_none;Planctomycetota;Planctomycetia;Pirellulales;Pirellulaceae;Lignipirellula;Lignipirellula cremea |
| Fuerstiella marisgermanici | 0 | 0 | 0 | 0 | 0 | 0 | 0 | 0 | 0 | 0 | 1 | 0 | 0 | 1 | Bacteria | Bacteria\_none | Planctomycetota | Planctomycetia | Planctomycetales | Planctomycetaceae | Fuerstiella | Bacteria;Bacteria\_none;Planctomycetota;Planctomycetia;Planctomycetales;Planctomycetaceae;Fuerstiella;Fuerstiella marisgermanici |
| Gimesia panareensis | 0 | 0 | 0 | 0 | 0 | 0 | 0 | 0 | 0 | 0 | 1 | 0 | 0 | 1 | Bacteria | Bacteria\_none | Planctomycetota | Planctomycetia | Planctomycetales | Planctomycetaceae | Gimesia | Bacteria;Bacteria\_none;Planctomycetota;Planctomycetia;Planctomycetales;Planctomycetaceae;Gimesia;Gimesia panareensis |
| Candidatus Pelagibacter sp. RS40 | 0 | 0 | 0 | 0 | 0 | 0 | 0 | 0 | 0 | 0 | 0 | 0 | 1 | 1 | Bacteria | Bacteria\_none | Pseudomonadota | Alphaproteobacteria | Candidatus Pelagibacterales | Candidatus Pelagibacteraceae | Candidatus Pelagibacter | Bacteria;Bacteria\_none;Pseudomonadota;Alphaproteobacteria;Candidatus Pelagibacterales;Candidatus Pelagibacteraceae;Candidatus Pelagibacter;Candidatus Pelagibacter sp. RS40 |
| Asticcacaulis sp. DW145 | 0 | 0 | 0 | 0 | 0 | 0 | 0 | 0 | 0 | 0 | 0 | 0 | 1 | 1 | Bacteria | Bacteria\_none | Pseudomonadota | Alphaproteobacteria | Caulobacterales | Caulobacteraceae | Asticcacaulis | Bacteria;Bacteria\_none;Pseudomonadota;Alphaproteobacteria;Caulobacterales;Caulobacteraceae;Asticcacaulis;Asticcacaulis sp. DW145 |
| Bosea sp. AS-1 | 0 | 0 | 0 | 0 | 0 | 1 | 0 | 0 | 0 | 0 | 0 | 0 | 0 | 1 | Bacteria | Bacteria\_none | Pseudomonadota | Alphaproteobacteria | Hyphomicrobiales | Boseaceae | Bosea | Bacteria;Bacteria\_none;Pseudomonadota;Alphaproteobacteria;Hyphomicrobiales;Boseaceae;Bosea;Bosea sp. AS-1 |
| Brucella sp. NM4 | 0 | 0 | 0 | 0 | 0 | 0 | 0 | 0 | 0 | 0 | 1 | 0 | 0 | 1 | Bacteria | Bacteria\_none | Pseudomonadota | Alphaproteobacteria | Hyphomicrobiales | Brucellaceae | Brucella | Bacteria;Bacteria\_none;Pseudomonadota;Alphaproteobacteria;Hyphomicrobiales;Brucellaceae;Brucella;Brucella sp. NM4 |
| Cohaesibacter\_uncultured | 0 | 0 | 0 | 0 | 0 | 0 | 0 | 0 | 1 | 0 | 0 | 0 | 1 | 2 | Bacteria | Bacteria\_none | Pseudomonadota | Alphaproteobacteria | Hyphomicrobiales | Cohaesibacteraceae | Cohaesibacter | Bacteria;Bacteria\_none;Pseudomonadota;Alphaproteobacteria;Hyphomicrobiales;Cohaesibacteraceae;Cohaesibacter;Cohaesibacter\_uncultured |
| Devosia oryziradicis | 0 | 0 | 0 | 0 | 0 | 0 | 1 | 0 | 0 | 0 | 0 | 0 | 0 | 1 | Bacteria | Bacteria\_none | Pseudomonadota | Alphaproteobacteria | Hyphomicrobiales | Devosiaceae | Devosia | Bacteria;Bacteria\_none;Pseudomonadota;Alphaproteobacteria;Hyphomicrobiales;Devosiaceae;Devosia;Devosia oryziradicis |
| Pelagibacterium flavum | 0 | 0 | 1 | 0 | 0 | 0 | 0 | 0 | 0 | 0 | 0 | 0 | 0 | 1 | Bacteria | Bacteria\_none | Pseudomonadota | Alphaproteobacteria | Hyphomicrobiales | Devosiaceae | Pelagibacterium | Bacteria;Bacteria\_none;Pseudomonadota;Alphaproteobacteria;Hyphomicrobiales;Devosiaceae;Pelagibacterium;Pelagibacterium flavum |
| Methylobacterium durans | 0 | 0 | 1 | 0 | 0 | 0 | 0 | 0 | 0 | 0 | 0 | 0 | 0 | 1 | Bacteria | Bacteria\_none | Pseudomonadota | Alphaproteobacteria | Hyphomicrobiales | Methylobacteriaceae | Methylobacterium | Bacteria;Bacteria\_none;Pseudomonadota;Alphaproteobacteria;Hyphomicrobiales;Methylobacteriaceae;Methylobacterium;Methylobacterium durans |
| Microvirga sp. VF16 | 0 | 0 | 0 | 0 | 0 | 0 | 0 | 0 | 0 | 0 | 1 | 0 | 0 | 1 | Bacteria | Bacteria\_none | Pseudomonadota | Alphaproteobacteria | Hyphomicrobiales | Methylobacteriaceae | Microvirga | Bacteria;Bacteria\_none;Pseudomonadota;Alphaproteobacteria;Hyphomicrobiales;Methylobacteriaceae;Microvirga;Microvirga sp. VF16 |
| [Pseudomonas] carboxydohydrogena | 0 | 1 | 0 | 0 | 0 | 0 | 0 | 0 | 0 | 0 | 0 | 0 | 0 | 1 | Bacteria | Bacteria\_none | Pseudomonadota | Alphaproteobacteria | Hyphomicrobiales | Nitrobacteraceae | Afipia | Bacteria;Bacteria\_none;Pseudomonadota;Alphaproteobacteria;Hyphomicrobiales;Nitrobacteraceae;Afipia;[Pseudomonas] carboxydohydrogena |
| Bradyrhizobium sp. | 0 | 0 | 0 | 0 | 0 | 0 | 0 | 0 | 0 | 0 | 1 | 0 | 0 | 1 | Bacteria | Bacteria\_none | Pseudomonadota | Alphaproteobacteria | Hyphomicrobiales | Nitrobacteraceae | Bradyrhizobium | Bacteria;Bacteria\_none;Pseudomonadota;Alphaproteobacteria;Hyphomicrobiales;Nitrobacteraceae;Bradyrhizobium;Bradyrhizobium sp. |
| Bradyrhizobium sp. 170 | 0 | 0 | 0 | 0 | 0 | 1 | 0 | 0 | 0 | 0 | 0 | 0 | 0 | 1 | Bacteria | Bacteria\_none | Pseudomonadota | Alphaproteobacteria | Hyphomicrobiales | Nitrobacteraceae | Bradyrhizobium | Bacteria;Bacteria\_none;Pseudomonadota;Alphaproteobacteria;Hyphomicrobiales;Nitrobacteraceae;Bradyrhizobium;Bradyrhizobium sp. 170 |
| Bradyrhizobium sp. BWA-3-5 | 0 | 0 | 0 | 0 | 0 | 0 | 0 | 0 | 0 | 0 | 1 | 0 | 0 | 1 | Bacteria | Bacteria\_none | Pseudomonadota | Alphaproteobacteria | Hyphomicrobiales | Nitrobacteraceae | Bradyrhizobium | Bacteria;Bacteria\_none;Pseudomonadota;Alphaproteobacteria;Hyphomicrobiales;Nitrobacteraceae;Bradyrhizobium;Bradyrhizobium sp. BWA-3-5 |
| Bradyrhizobium sp. NDS-1 | 0 | 0 | 1 | 0 | 0 | 0 | 0 | 0 | 0 | 0 | 0 | 0 | 0 | 1 | Bacteria | Bacteria\_none | Pseudomonadota | Alphaproteobacteria | Hyphomicrobiales | Nitrobacteraceae | Bradyrhizobium | Bacteria;Bacteria\_none;Pseudomonadota;Alphaproteobacteria;Hyphomicrobiales;Nitrobacteraceae;Bradyrhizobium;Bradyrhizobium sp. NDS-1 |
| Aquibium microcysteis | 0 | 0 | 0 | 0 | 0 | 0 | 0 | 1 | 0 | 0 | 0 | 0 | 0 | 1 | Bacteria | Bacteria\_none | Pseudomonadota | Alphaproteobacteria | Hyphomicrobiales | Phyllobacteriaceae | Aquibium | Bacteria;Bacteria\_none;Pseudomonadota;Alphaproteobacteria;Hyphomicrobiales;Phyllobacteriaceae;Aquibium;Aquibium microcysteis |
| Aquibium oceanicum | 0 | 0 | 0 | 0 | 0 | 0 | 0 | 1 | 0 | 0 | 0 | 0 | 0 | 1 | Bacteria | Bacteria\_none | Pseudomonadota | Alphaproteobacteria | Hyphomicrobiales | Phyllobacteriaceae | Aquibium | Bacteria;Bacteria\_none;Pseudomonadota;Alphaproteobacteria;Hyphomicrobiales;Phyllobacteriaceae;Aquibium;Aquibium oceanicum |
| Mesorhizobium huakuii | 0 | 0 | 0 | 0 | 0 | 0 | 0 | 1 | 0 | 0 | 0 | 0 | 0 | 1 | Bacteria | Bacteria\_none | Pseudomonadota | Alphaproteobacteria | Hyphomicrobiales | Phyllobacteriaceae | Mesorhizobium | Bacteria;Bacteria\_none;Pseudomonadota;Alphaproteobacteria;Hyphomicrobiales;Phyllobacteriaceae;Mesorhizobium;Mesorhizobium huakuii |
| Mesorhizobium koreense | 0 | 0 | 1 | 0 | 0 | 0 | 0 | 0 | 0 | 0 | 0 | 0 | 0 | 1 | Bacteria | Bacteria\_none | Pseudomonadota | Alphaproteobacteria | Hyphomicrobiales | Phyllobacteriaceae | Mesorhizobium | Bacteria;Bacteria\_none;Pseudomonadota;Alphaproteobacteria;Hyphomicrobiales;Phyllobacteriaceae;Mesorhizobium;Mesorhizobium koreense |
| Mesorhizobium sp. M6A.T.Cr.TU.016.01.1.1 | 0 | 0 | 1 | 0 | 0 | 0 | 0 | 0 | 0 | 0 | 0 | 0 | 0 | 1 | Bacteria | Bacteria\_none | Pseudomonadota | Alphaproteobacteria | Hyphomicrobiales | Phyllobacteriaceae | Mesorhizobium | Bacteria;Bacteria\_none;Pseudomonadota;Alphaproteobacteria;Hyphomicrobiales;Phyllobacteriaceae;Mesorhizobium;Mesorhizobium sp. M6A.T.Cr.TU.016.01.1.1 |
| Mesorhizobium sp. NZP2077 | 0 | 0 | 0 | 0 | 0 | 1 | 0 | 0 | 0 | 0 | 0 | 0 | 0 | 1 | Bacteria | Bacteria\_none | Pseudomonadota | Alphaproteobacteria | Hyphomicrobiales | Phyllobacteriaceae | Mesorhizobium | Bacteria;Bacteria\_none;Pseudomonadota;Alphaproteobacteria;Hyphomicrobiales;Phyllobacteriaceae;Mesorhizobium;Mesorhizobium sp. NZP2077 |
| Nitratireductor rhodophyticola | 0 | 0 | 0 | 0 | 0 | 0 | 1 | 0 | 0 | 0 | 0 | 0 | 0 | 1 | Bacteria | Bacteria\_none | Pseudomonadota | Alphaproteobacteria | Hyphomicrobiales | Phyllobacteriaceae | Nitratireductor | Bacteria;Bacteria\_none;Pseudomonadota;Alphaproteobacteria;Hyphomicrobiales;Phyllobacteriaceae;Nitratireductor;Nitratireductor rhodophyticola |
| Agrobacterium fabrum | 0 | 0 | 0 | 0 | 0 | 0 | 0 | 1 | 0 | 0 | 0 | 0 | 0 | 1 | Bacteria | Bacteria\_none | Pseudomonadota | Alphaproteobacteria | Hyphomicrobiales | Rhizobiaceae | Agrobacterium | Bacteria;Bacteria\_none;Pseudomonadota;Alphaproteobacteria;Hyphomicrobiales;Rhizobiaceae;Agrobacterium;Agrobacterium fabrum |
| Candidatus Liberibacter asiaticus | 0 | 0 | 0 | 0 | 0 | 0 | 0 | 0 | 0 | 0 | 1 | 0 | 0 | 1 | Bacteria | Bacteria\_none | Pseudomonadota | Alphaproteobacteria | Hyphomicrobiales | Rhizobiaceae | Liberibacter | Bacteria;Bacteria\_none;Pseudomonadota;Alphaproteobacteria;Hyphomicrobiales;Rhizobiaceae;Liberibacter;Candidatus Liberibacter asiaticus |
| Pararhizobium qamdonense | 0 | 0 | 0 | 0 | 0 | 1 | 0 | 0 | 0 | 0 | 0 | 0 | 0 | 1 | Bacteria | Bacteria\_none | Pseudomonadota | Alphaproteobacteria | Hyphomicrobiales | Rhizobiaceae | Pararhizobium | Bacteria;Bacteria\_none;Pseudomonadota;Alphaproteobacteria;Hyphomicrobiales;Rhizobiaceae;Pararhizobium;Pararhizobium qamdonense |
| Rhizobium hidalgonense | 0 | 0 | 0 | 0 | 0 | 0 | 0 | 0 | 0 | 0 | 0 | 0 | 1 | 1 | Bacteria | Bacteria\_none | Pseudomonadota | Alphaproteobacteria | Hyphomicrobiales | Rhizobiaceae | Rhizobium | Bacteria;Bacteria\_none;Pseudomonadota;Alphaproteobacteria;Hyphomicrobiales;Rhizobiaceae;Rhizobium;Rhizobium hidalgonense |
| Rhizobium rhizogenes | 0 | 0 | 0 | 0 | 0 | 0 | 0 | 0 | 0 | 0 | 0 | 0 | 1 | 1 | Bacteria | Bacteria\_none | Pseudomonadota | Alphaproteobacteria | Hyphomicrobiales | Rhizobiaceae | Rhizobium | Bacteria;Bacteria\_none;Pseudomonadota;Alphaproteobacteria;Hyphomicrobiales;Rhizobiaceae;Rhizobium;Rhizobium rhizogenes |
| Rhizobium tumorigenes | 0 | 0 | 0 | 0 | 0 | 0 | 0 | 0 | 0 | 1 | 0 | 0 | 0 | 1 | Bacteria | Bacteria\_none | Pseudomonadota | Alphaproteobacteria | Hyphomicrobiales | Rhizobiaceae | Rhizobium | Bacteria;Bacteria\_none;Pseudomonadota;Alphaproteobacteria;Hyphomicrobiales;Rhizobiaceae;Rhizobium;Rhizobium tumorigenes |
| Shinella zoogloeoides | 0 | 0 | 0 | 0 | 0 | 1 | 0 | 0 | 0 | 0 | 0 | 0 | 0 | 1 | Bacteria | Bacteria\_none | Pseudomonadota | Alphaproteobacteria | Hyphomicrobiales | Rhizobiaceae | Shinella | Bacteria;Bacteria\_none;Pseudomonadota;Alphaproteobacteria;Hyphomicrobiales;Rhizobiaceae;Shinella;Shinella zoogloeoides |
| Sinorhizobium medicae | 1 | 0 | 0 | 0 | 0 | 0 | 0 | 0 | 0 | 0 | 0 | 0 | 0 | 1 | Bacteria | Bacteria\_none | Pseudomonadota | Alphaproteobacteria | Hyphomicrobiales | Rhizobiaceae | Sinorhizobium | Bacteria;Bacteria\_none;Pseudomonadota;Alphaproteobacteria;Hyphomicrobiales;Rhizobiaceae;Sinorhizobium;Sinorhizobium medicae |
| Sinorhizobium numidicum | 0 | 0 | 0 | 0 | 1 | 0 | 0 | 0 | 0 | 0 | 0 | 0 | 0 | 1 | Bacteria | Bacteria\_none | Pseudomonadota | Alphaproteobacteria | Hyphomicrobiales | Rhizobiaceae | Sinorhizobium | Bacteria;Bacteria\_none;Pseudomonadota;Alphaproteobacteria;Hyphomicrobiales;Rhizobiaceae;Sinorhizobium;Sinorhizobium numidicum |
| Kordiimonas pumila | 0 | 0 | 0 | 0 | 0 | 0 | 0 | 1 | 0 | 0 | 0 | 0 | 0 | 1 | Bacteria | Bacteria\_none | Pseudomonadota | Alphaproteobacteria | Kordiimonadales | Kordiimonadaceae | Kordiimonas | Bacteria;Bacteria\_none;Pseudomonadota;Alphaproteobacteria;Kordiimonadales;Kordiimonadaceae;Kordiimonas;Kordiimonas pumila |
| Cereibacter sphaeroides | 0 | 0 | 0 | 0 | 0 | 0 | 0 | 0 | 0 | 0 | 1 | 0 | 0 | 1 | Bacteria | Bacteria\_none | Pseudomonadota | Alphaproteobacteria | Rhodobacterales | Paracoccaceae | Cereibacter | Bacteria;Bacteria\_none;Pseudomonadota;Alphaproteobacteria;Rhodobacterales;Paracoccaceae;Cereibacter;Cereibacter sphaeroides |
| Paracoccus aminovorans | 0 | 0 | 0 | 0 | 0 | 0 | 0 | 0 | 1 | 0 | 0 | 0 | 0 | 1 | Bacteria | Bacteria\_none | Pseudomonadota | Alphaproteobacteria | Rhodobacterales | Paracoccaceae | Paracoccus | Bacteria;Bacteria\_none;Pseudomonadota;Alphaproteobacteria;Rhodobacterales;Paracoccaceae;Paracoccus;Paracoccus aminovorans |
| Paracoccus mutanolyticus | 0 | 0 | 0 | 0 | 0 | 1 | 0 | 0 | 0 | 0 | 0 | 0 | 0 | 1 | Bacteria | Bacteria\_none | Pseudomonadota | Alphaproteobacteria | Rhodobacterales | Paracoccaceae | Paracoccus | Bacteria;Bacteria\_none;Pseudomonadota;Alphaproteobacteria;Rhodobacterales;Paracoccaceae;Paracoccus;Paracoccus mutanolyticus |
| Tabrizicola piscis | 0 | 0 | 0 | 0 | 0 | 0 | 0 | 0 | 0 | 0 | 0 | 0 | 1 | 1 | Bacteria | Bacteria\_none | Pseudomonadota | Alphaproteobacteria | Rhodobacterales | Paracoccaceae | Tabrizicola | Bacteria;Bacteria\_none;Pseudomonadota;Alphaproteobacteria;Rhodobacterales;Paracoccaceae;Tabrizicola;Tabrizicola piscis |
| Phaeobacter inhibens | 0 | 0 | 0 | 0 | 1 | 0 | 0 | 0 | 0 | 0 | 0 | 0 | 0 | 1 | Bacteria | Bacteria\_none | Pseudomonadota | Alphaproteobacteria | Rhodobacterales | Roseobacteraceae | Phaeobacter | Bacteria;Bacteria\_none;Pseudomonadota;Alphaproteobacteria;Rhodobacterales;Roseobacteraceae;Phaeobacter;Phaeobacter inhibens |
| Ponticoccus alexandrii | 2 | 0 | 0 | 0 | 0 | 0 | 0 | 0 | 0 | 0 | 0 | 0 | 0 | 2 | Bacteria | Bacteria\_none | Pseudomonadota | Alphaproteobacteria | Rhodobacterales | Roseobacteraceae | Ponticoccus | Bacteria;Bacteria\_none;Pseudomonadota;Alphaproteobacteria;Rhodobacterales;Roseobacteraceae;Ponticoccus;Ponticoccus alexandrii |
| Pseudosulfitobacter pseudonitzschiae | 0 | 1 | 0 | 0 | 0 | 0 | 0 | 0 | 0 | 0 | 0 | 0 | 0 | 1 | Bacteria | Bacteria\_none | Pseudomonadota | Alphaproteobacteria | Rhodobacterales | Roseobacteraceae | Pseudosulfitobacter | Bacteria;Bacteria\_none;Pseudomonadota;Alphaproteobacteria;Rhodobacterales;Roseobacteraceae;Pseudosulfitobacter;Pseudosulfitobacter pseudonitzschiae |
| Roseobacter litoralis | 0 | 0 | 0 | 1 | 0 | 0 | 0 | 0 | 0 | 0 | 0 | 0 | 0 | 1 | Bacteria | Bacteria\_none | Pseudomonadota | Alphaproteobacteria | Rhodobacterales | Roseobacteraceae | Roseobacter | Bacteria;Bacteria\_none;Pseudomonadota;Alphaproteobacteria;Rhodobacterales;Roseobacteraceae;Roseobacter;Roseobacter litoralis |
| Ruegeria\_uncultured | 0 | 0 | 0 | 0 | 1 | 0 | 0 | 0 | 0 | 0 | 0 | 0 | 1 | 2 | Bacteria | Bacteria\_none | Pseudomonadota | Alphaproteobacteria | Rhodobacterales | Roseobacteraceae | Ruegeria | Bacteria;Bacteria\_none;Pseudomonadota;Alphaproteobacteria;Rhodobacterales;Roseobacteraceae;Ruegeria;Ruegeria\_uncultured |
| Sulfitobacter alexandrii | 0 | 0 | 0 | 0 | 0 | 0 | 0 | 0 | 0 | 0 | 1 | 0 | 0 | 1 | Bacteria | Bacteria\_none | Pseudomonadota | Alphaproteobacteria | Rhodobacterales | Roseobacteraceae | Sulfitobacter | Bacteria;Bacteria\_none;Pseudomonadota;Alphaproteobacteria;Rhodobacterales;Roseobacteraceae;Sulfitobacter;Sulfitobacter alexandrii |
| Sulfitobacter faviae | 0 | 0 | 0 | 0 | 0 | 0 | 0 | 0 | 0 | 0 | 1 | 0 | 0 | 1 | Bacteria | Bacteria\_none | Pseudomonadota | Alphaproteobacteria | Rhodobacterales | Roseobacteraceae | Sulfitobacter | Bacteria;Bacteria\_none;Pseudomonadota;Alphaproteobacteria;Rhodobacterales;Roseobacteraceae;Sulfitobacter;Sulfitobacter faviae |
| Komagataeibacter rhaeticus | 0 | 0 | 0 | 0 | 0 | 1 | 0 | 0 | 0 | 0 | 0 | 0 | 0 | 1 | Bacteria | Bacteria\_none | Pseudomonadota | Alphaproteobacteria | Rhodospirillales | Acetobacteraceae | Komagataeibacter | Bacteria;Bacteria\_none;Pseudomonadota;Alphaproteobacteria;Rhodospirillales;Acetobacteraceae;Komagataeibacter;Komagataeibacter rhaeticus |
| Pseudoroseomonas cervicalis | 0 | 0 | 0 | 0 | 0 | 0 | 1 | 0 | 0 | 0 | 0 | 0 | 0 | 1 | Bacteria | Bacteria\_none | Pseudomonadota | Alphaproteobacteria | Rhodospirillales | Acetobacteraceae | Pseudoroseomonas | Bacteria;Bacteria\_none;Pseudomonadota;Alphaproteobacteria;Rhodospirillales;Acetobacteraceae;Pseudoroseomonas;Pseudoroseomonas cervicalis |
| Azospirillum ramasamyi | 0 | 0 | 0 | 0 | 0 | 0 | 0 | 0 | 0 | 0 | 1 | 0 | 0 | 1 | Bacteria | Bacteria\_none | Pseudomonadota | Alphaproteobacteria | Rhodospirillales | Azospirillaceae | Azospirillum | Bacteria;Bacteria\_none;Pseudomonadota;Alphaproteobacteria;Rhodospirillales;Azospirillaceae;Azospirillum;Azospirillum ramasamyi |
| Azospirillum sp. TSH100 | 0 | 0 | 0 | 0 | 1 | 0 | 0 | 0 | 0 | 0 | 0 | 0 | 0 | 1 | Bacteria | Bacteria\_none | Pseudomonadota | Alphaproteobacteria | Rhodospirillales | Azospirillaceae | Azospirillum | Bacteria;Bacteria\_none;Pseudomonadota;Alphaproteobacteria;Rhodospirillales;Azospirillaceae;Azospirillum;Azospirillum sp. TSH100 |
| Varunaivibrio sulfuroxidans | 0 | 0 | 0 | 0 | 0 | 0 | 0 | 0 | 0 | 0 | 0 | 0 | 1 | 1 | Bacteria | Bacteria\_none | Pseudomonadota | Alphaproteobacteria | Rhodospirillales | Magnetovibrionaceae | Varunaivibrio | Bacteria;Bacteria\_none;Pseudomonadota;Alphaproteobacteria;Rhodospirillales;Magnetovibrionaceae;Varunaivibrio;Varunaivibrio sulfuroxidans |
| Thalassospira sp. SN3W | 0 | 0 | 0 | 1 | 0 | 0 | 0 | 0 | 0 | 0 | 0 | 0 | 0 | 1 | Bacteria | Bacteria\_none | Pseudomonadota | Alphaproteobacteria | Rhodospirillales | Thalassospiraceae | Thalassospira | Bacteria;Bacteria\_none;Pseudomonadota;Alphaproteobacteria;Rhodospirillales;Thalassospiraceae;Thalassospira;Thalassospira sp. SN3W |
| Neorickettsia sennetsu | 0 | 1 | 0 | 0 | 0 | 0 | 0 | 0 | 0 | 0 | 0 | 0 | 0 | 1 | Bacteria | Bacteria\_none | Pseudomonadota | Alphaproteobacteria | Rickettsiales | Anaplasmataceae | Neorickettsia | Bacteria;Bacteria\_none;Pseudomonadota;Alphaproteobacteria;Rickettsiales;Anaplasmataceae;Neorickettsia;Neorickettsia sennetsu |
| Wolbachia endosymbiont (group B) of Phasia obesa | 0 | 0 | 0 | 0 | 0 | 0 | 0 | 0 | 0 | 0 | 0 | 0 | 1 | 1 | Bacteria | Bacteria\_none | Pseudomonadota | Alphaproteobacteria | Rickettsiales | Anaplasmataceae | Wolbachia | Bacteria;Bacteria\_none;Pseudomonadota;Alphaproteobacteria;Rickettsiales;Anaplasmataceae;Wolbachia;Wolbachia endosymbiont (group B) of Phasia obesa |
| Wolbachia endosymbiont of Ctenocephalides felis wCfeT | 0 | 0 | 0 | 0 | 0 | 0 | 0 | 0 | 0 | 0 | 0 | 0 | 1 | 1 | Bacteria | Bacteria\_none | Pseudomonadota | Alphaproteobacteria | Rickettsiales | Anaplasmataceae | Wolbachia | Bacteria;Bacteria\_none;Pseudomonadota;Alphaproteobacteria;Rickettsiales;Anaplasmataceae;Wolbachia;Wolbachia endosymbiont of Ctenocephalides felis wCfeT |
| Wolbachia endosymbiont of Dirofilaria (Dirofilaria) immitis | 0 | 0 | 0 | 0 | 0 | 0 | 0 | 0 | 0 | 0 | 0 | 0 | 1 | 1 | Bacteria | Bacteria\_none | Pseudomonadota | Alphaproteobacteria | Rickettsiales | Anaplasmataceae | Wolbachia | Bacteria;Bacteria\_none;Pseudomonadota;Alphaproteobacteria;Rickettsiales;Anaplasmataceae;Wolbachia;Wolbachia endosymbiont of Dirofilaria (Dirofilaria) immitis |
| Candidatus Phycorickettsia trachydisci | 0 | 0 | 0 | 0 | 0 | 0 | 1 | 0 | 0 | 0 | 0 | 0 | 0 | 1 | Bacteria | Bacteria\_none | Pseudomonadota | Alphaproteobacteria | Rickettsiales | Rickettsiaceae | Candidatus Phycorickettsia | Bacteria;Bacteria\_none;Pseudomonadota;Alphaproteobacteria;Rickettsiales;Rickettsiaceae;Candidatus Phycorickettsia;Candidatus Phycorickettsia trachydisci |
| Novosphingobium sp. KACC 22771 | 0 | 0 | 0 | 0 | 0 | 0 | 0 | 0 | 0 | 0 | 1 | 0 | 0 | 1 | Bacteria | Bacteria\_none | Pseudomonadota | Alphaproteobacteria | Sphingomonadales | Sphingomonadaceae | Novosphingobium | Bacteria;Bacteria\_none;Pseudomonadota;Alphaproteobacteria;Sphingomonadales;Sphingomonadaceae;Novosphingobium;Novosphingobium sp. KACC 22771 |
| Sphingomonas flavescens | 0 | 0 | 0 | 0 | 0 | 0 | 0 | 0 | 0 | 0 | 1 | 0 | 1 | 2 | Bacteria | Bacteria\_none | Pseudomonadota | Alphaproteobacteria | Sphingomonadales | Sphingomonadaceae | Sphingomonas | Bacteria;Bacteria\_none;Pseudomonadota;Alphaproteobacteria;Sphingomonadales;Sphingomonadaceae;Sphingomonas;Sphingomonas flavescens |
| Sphingomonas ginsengisoli An et al. 2013 | 0 | 0 | 0 | 0 | 0 | 0 | 0 | 0 | 0 | 0 | 0 | 0 | 1 | 1 | Bacteria | Bacteria\_none | Pseudomonadota | Alphaproteobacteria | Sphingomonadales | Sphingomonadaceae | Sphingomonas | Bacteria;Bacteria\_none;Pseudomonadota;Alphaproteobacteria;Sphingomonadales;Sphingomonadaceae;Sphingomonas;Sphingomonas ginsengisoli An et al. 2013 |
| Sphingomonas xanthus | 0 | 0 | 0 | 0 | 0 | 0 | 0 | 0 | 0 | 0 | 0 | 1 | 0 | 1 | Bacteria | Bacteria\_none | Pseudomonadota | Alphaproteobacteria | Sphingomonadales | Sphingomonadaceae | Sphingomonas | Bacteria;Bacteria\_none;Pseudomonadota;Alphaproteobacteria;Sphingomonadales;Sphingomonadaceae;Sphingomonas;Sphingomonas xanthus |
| Sphingopyxis fribergensis | 0 | 0 | 0 | 0 | 0 | 1 | 0 | 0 | 0 | 0 | 0 | 0 | 0 | 1 | Bacteria | Bacteria\_none | Pseudomonadota | Alphaproteobacteria | Sphingomonadales | Sphingomonadaceae | Sphingopyxis | Bacteria;Bacteria\_none;Pseudomonadota;Alphaproteobacteria;Sphingomonadales;Sphingomonadaceae;Sphingopyxis;Sphingopyxis fribergensis |
| Sphingopyxis sp. TUF1 | 0 | 0 | 0 | 1 | 0 | 0 | 0 | 0 | 0 | 0 | 0 | 0 | 0 | 1 | Bacteria | Bacteria\_none | Pseudomonadota | Alphaproteobacteria | Sphingomonadales | Sphingomonadaceae | Sphingopyxis | Bacteria;Bacteria\_none;Pseudomonadota;Alphaproteobacteria;Sphingomonadales;Sphingomonadaceae;Sphingopyxis;Sphingopyxis sp. TUF1 |
| Candidatus Profftella armatura | 0 | 0 | 0 | 0 | 1 | 0 | 0 | 0 | 0 | 0 | 0 | 0 | 0 | 1 | Bacteria | Bacteria\_none | Pseudomonadota | Betaproteobacteria | Betaproteobacteria\_Incertae\_sedis | Betaproteobacteria\_Incertae\_sedis | Candidatus Profftella | Bacteria;Bacteria\_none;Pseudomonadota;Betaproteobacteria;Betaproteobacteria\_Incertae\_sedis;Betaproteobacteria\_Incertae\_sedis;Candidatus Profftella;Candidatus Profftella armatura |
| Casimicrobium huifangae | 0 | 0 | 0 | 0 | 0 | 0 | 0 | 1 | 0 | 0 | 0 | 0 | 0 | 1 | Bacteria | Bacteria\_none | Pseudomonadota | Betaproteobacteria | Betaproteobacteria\_Incertae\_sedis | Casimicrobiaceae | Casimicrobium | Bacteria;Bacteria\_none;Pseudomonadota;Betaproteobacteria;Betaproteobacteria\_Incertae\_sedis;Casimicrobiaceae;Casimicrobium;Casimicrobium huifangae |
| Advenella alkanexedens | 0 | 0 | 0 | 0 | 1 | 0 | 0 | 0 | 0 | 0 | 0 | 0 | 0 | 1 | Bacteria | Bacteria\_none | Pseudomonadota | Betaproteobacteria | Burkholderiales | Alcaligenaceae | Advenella | Bacteria;Bacteria\_none;Pseudomonadota;Betaproteobacteria;Burkholderiales;Alcaligenaceae;Advenella;Advenella alkanexedens |
| Bordetella avium | 0 | 0 | 0 | 0 | 0 | 0 | 0 | 0 | 0 | 0 | 1 | 0 | 0 | 1 | Bacteria | Bacteria\_none | Pseudomonadota | Betaproteobacteria | Burkholderiales | Alcaligenaceae | Bordetella | Bacteria;Bacteria\_none;Pseudomonadota;Betaproteobacteria;Burkholderiales;Alcaligenaceae;Bordetella;Bordetella avium |
| Neopusillimonas aromaticivorans | 0 | 0 | 0 | 0 | 0 | 0 | 1 | 0 | 0 | 0 | 0 | 0 | 0 | 1 | Bacteria | Bacteria\_none | Pseudomonadota | Betaproteobacteria | Burkholderiales | Alcaligenaceae | Neopusillimonas | Bacteria;Bacteria\_none;Pseudomonadota;Betaproteobacteria;Burkholderiales;Alcaligenaceae;Neopusillimonas;Neopusillimonas aromaticivorans |
| Orrella dioscoreae | 0 | 0 | 0 | 0 | 0 | 1 | 0 | 0 | 0 | 0 | 0 | 0 | 0 | 1 | Bacteria | Bacteria\_none | Pseudomonadota | Betaproteobacteria | Burkholderiales | Alcaligenaceae | Orrella | Bacteria;Bacteria\_none;Pseudomonadota;Betaproteobacteria;Burkholderiales;Alcaligenaceae;Orrella;Orrella dioscoreae |
| Pollutimonas thiosulfatoxidans | 0 | 0 | 0 | 0 | 0 | 0 | 0 | 0 | 0 | 0 | 1 | 0 | 0 | 1 | Bacteria | Bacteria\_none | Pseudomonadota | Betaproteobacteria | Burkholderiales | Alcaligenaceae | Pollutimonas | Bacteria;Bacteria\_none;Pseudomonadota;Betaproteobacteria;Burkholderiales;Alcaligenaceae;Pollutimonas;Pollutimonas thiosulfatoxidans |
| Pusillimonas sp. T7-7 | 0 | 0 | 0 | 0 | 0 | 0 | 0 | 0 | 0 | 0 | 0 | 0 | 1 | 1 | Bacteria | Bacteria\_none | Pseudomonadota | Betaproteobacteria | Burkholderiales | Alcaligenaceae | Pusillimonas | Bacteria;Bacteria\_none;Pseudomonadota;Betaproteobacteria;Burkholderiales;Alcaligenaceae;Pusillimonas;Pusillimonas sp. T7-7 |
| Burkholderia cenocepacia | 0 | 0 | 0 | 0 | 0 | 0 | 0 | 0 | 0 | 0 | 0 | 0 | 1 | 1 | Bacteria | Bacteria\_none | Pseudomonadota | Betaproteobacteria | Burkholderiales | Burkholderiaceae | Burkholderia | Bacteria;Bacteria\_none;Pseudomonadota;Betaproteobacteria;Burkholderiales;Burkholderiaceae;Burkholderia;Burkholderia cenocepacia |
| Burkholderia cepacia | 0 | 0 | 0 | 0 | 0 | 0 | 0 | 0 | 0 | 1 | 0 | 0 | 0 | 1 | Bacteria | Bacteria\_none | Pseudomonadota | Betaproteobacteria | Burkholderiales | Burkholderiaceae | Burkholderia | Bacteria;Bacteria\_none;Pseudomonadota;Betaproteobacteria;Burkholderiales;Burkholderiaceae;Burkholderia;Burkholderia cepacia |
| Burkholderia dolosa | 0 | 0 | 0 | 0 | 0 | 1 | 0 | 0 | 0 | 0 | 0 | 0 | 0 | 1 | Bacteria | Bacteria\_none | Pseudomonadota | Betaproteobacteria | Burkholderiales | Burkholderiaceae | Burkholderia | Bacteria;Bacteria\_none;Pseudomonadota;Betaproteobacteria;Burkholderiales;Burkholderiaceae;Burkholderia;Burkholderia dolosa |
| Burkholderia multivorans | 0 | 0 | 2 | 0 | 0 | 0 | 0 | 0 | 0 | 0 | 0 | 0 | 3 | 5 | Bacteria | Bacteria\_none | Pseudomonadota | Betaproteobacteria | Burkholderiales | Burkholderiaceae | Burkholderia | Bacteria;Bacteria\_none;Pseudomonadota;Betaproteobacteria;Burkholderiales;Burkholderiaceae;Burkholderia;Burkholderia multivorans |
| Cupriavidus oxalaticus | 0 | 0 | 0 | 0 | 1 | 0 | 0 | 0 | 0 | 0 | 0 | 0 | 0 | 1 | Bacteria | Bacteria\_none | Pseudomonadota | Betaproteobacteria | Burkholderiales | Burkholderiaceae | Cupriavidus | Bacteria;Bacteria\_none;Pseudomonadota;Betaproteobacteria;Burkholderiales;Burkholderiaceae;Cupriavidus;Cupriavidus oxalaticus |
| Lautropia mirabilis | 0 | 1 | 0 | 0 | 4 | 0 | 0 | 2 | 0 | 2 | 11 | 0 | 3 | 23 | Bacteria | Bacteria\_none | Pseudomonadota | Betaproteobacteria | Burkholderiales | Burkholderiaceae | Lautropia | Bacteria;Bacteria\_none;Pseudomonadota;Betaproteobacteria;Burkholderiales;Burkholderiaceae;Lautropia;Lautropia mirabilis |
| Mycoavidus sp. B2-EB | 0 | 0 | 0 | 0 | 0 | 0 | 0 | 0 | 0 | 1 | 0 | 0 | 0 | 1 | Bacteria | Bacteria\_none | Pseudomonadota | Betaproteobacteria | Burkholderiales | Burkholderiaceae | Mycoavidus | Bacteria;Bacteria\_none;Pseudomonadota;Betaproteobacteria;Burkholderiales;Burkholderiaceae;Mycoavidus;Mycoavidus sp. B2-EB |
| Pandoraea pnomenusa | 1 | 0 | 0 | 0 | 0 | 0 | 0 | 0 | 0 | 0 | 0 | 0 | 0 | 1 | Bacteria | Bacteria\_none | Pseudomonadota | Betaproteobacteria | Burkholderiales | Burkholderiaceae | Pandoraea | Bacteria;Bacteria\_none;Pseudomonadota;Betaproteobacteria;Burkholderiales;Burkholderiaceae;Pandoraea;Pandoraea pnomenusa |
| Paraburkholderia acidisoli | 0 | 0 | 0 | 0 | 0 | 0 | 1 | 0 | 0 | 0 | 0 | 0 | 0 | 1 | Bacteria | Bacteria\_none | Pseudomonadota | Betaproteobacteria | Burkholderiales | Burkholderiaceae | Paraburkholderia | Bacteria;Bacteria\_none;Pseudomonadota;Betaproteobacteria;Burkholderiales;Burkholderiaceae;Paraburkholderia;Paraburkholderia acidisoli |
| Paraburkholderia sp. PGU19 | 0 | 0 | 0 | 0 | 0 | 0 | 1 | 0 | 0 | 0 | 0 | 0 | 0 | 1 | Bacteria | Bacteria\_none | Pseudomonadota | Betaproteobacteria | Burkholderiales | Burkholderiaceae | Paraburkholderia | Bacteria;Bacteria\_none;Pseudomonadota;Betaproteobacteria;Burkholderiales;Burkholderiaceae;Paraburkholderia;Paraburkholderia sp. PGU19 |
| Polynucleobacter necessarius | 0 | 0 | 0 | 0 | 0 | 0 | 0 | 0 | 0 | 0 | 0 | 0 | 1 | 1 | Bacteria | Bacteria\_none | Pseudomonadota | Betaproteobacteria | Burkholderiales | Burkholderiaceae | Polynucleobacter | Bacteria;Bacteria\_none;Pseudomonadota;Betaproteobacteria;Burkholderiales;Burkholderiaceae;Polynucleobacter;Polynucleobacter necessarius |
| Ralstonia solanacearum | 0 | 0 | 0 | 0 | 1 | 0 | 0 | 0 | 0 | 0 | 0 | 0 | 0 | 1 | Bacteria | Bacteria\_none | Pseudomonadota | Betaproteobacteria | Burkholderiales | Burkholderiaceae | Ralstonia | Bacteria;Bacteria\_none;Pseudomonadota;Betaproteobacteria;Burkholderiales;Burkholderiaceae;Ralstonia;Ralstonia solanacearum |
| Acidovorax sp. FHTAMBA | 0 | 0 | 0 | 0 | 0 | 0 | 0 | 0 | 0 | 0 | 1 | 0 | 0 | 1 | Bacteria | Bacteria\_none | Pseudomonadota | Betaproteobacteria | Burkholderiales | Comamonadaceae | Acidovorax | Bacteria;Bacteria\_none;Pseudomonadota;Betaproteobacteria;Burkholderiales;Comamonadaceae;Acidovorax;Acidovorax sp. FHTAMBA |
| Hydrogenophaga sp. SL48 | 0 | 0 | 0 | 0 | 0 | 1 | 0 | 0 | 0 | 0 | 0 | 0 | 0 | 1 | Bacteria | Bacteria\_none | Pseudomonadota | Betaproteobacteria | Burkholderiales | Comamonadaceae | Hydrogenophaga | Bacteria;Bacteria\_none;Pseudomonadota;Betaproteobacteria;Burkholderiales;Comamonadaceae;Hydrogenophaga;Hydrogenophaga sp. SL48 |
| Ottowia sp. oral taxon 894 | 0 | 0 | 0 | 0 | 0 | 0 | 0 | 1 | 0 | 0 | 0 | 0 | 0 | 1 | Bacteria | Bacteria\_none | Pseudomonadota | Betaproteobacteria | Burkholderiales | Comamonadaceae | Ottowia | Bacteria;Bacteria\_none;Pseudomonadota;Betaproteobacteria;Burkholderiales;Comamonadaceae;Ottowia;Ottowia sp. oral taxon 894 |
| Variovorax paradoxus | 0 | 0 | 0 | 0 | 0 | 0 | 1 | 0 | 0 | 0 | 0 | 0 | 0 | 1 | Bacteria | Bacteria\_none | Pseudomonadota | Betaproteobacteria | Burkholderiales | Comamonadaceae | Variovorax | Bacteria;Bacteria\_none;Pseudomonadota;Betaproteobacteria;Burkholderiales;Comamonadaceae;Variovorax;Variovorax paradoxus |
| Variovorax sp. HW608 | 0 | 0 | 0 | 0 | 0 | 0 | 0 | 0 | 0 | 0 | 0 | 0 | 1 | 1 | Bacteria | Bacteria\_none | Pseudomonadota | Betaproteobacteria | Burkholderiales | Comamonadaceae | Variovorax | Bacteria;Bacteria\_none;Pseudomonadota;Betaproteobacteria;Burkholderiales;Comamonadaceae;Variovorax;Variovorax sp. HW608 |
| Candidatus Zinderia endosymbiont of Aphrophora alni | 0 | 0 | 0 | 0 | 1 | 0 | 0 | 0 | 0 | 0 | 0 | 0 | 0 | 1 | Bacteria | Bacteria\_none | Pseudomonadota | Betaproteobacteria | Burkholderiales | Oxalobacteraceae | Candidatus Zinderia | Bacteria;Bacteria\_none;Pseudomonadota;Betaproteobacteria;Burkholderiales;Oxalobacteraceae;Candidatus Zinderia;Candidatus Zinderia endosymbiont of Aphrophora alni |
| Janthinobacterium sp. TB1-E2 | 0 | 0 | 0 | 0 | 0 | 1 | 0 | 0 | 0 | 0 | 0 | 0 | 0 | 1 | Bacteria | Bacteria\_none | Pseudomonadota | Betaproteobacteria | Burkholderiales | Oxalobacteraceae | Janthinobacterium | Bacteria;Bacteria\_none;Pseudomonadota;Betaproteobacteria;Burkholderiales;Oxalobacteraceae;Janthinobacterium;Janthinobacterium sp. TB1-E2 |
| Massilia litorea | 0 | 0 | 0 | 0 | 0 | 0 | 0 | 0 | 0 | 1 | 0 | 0 | 0 | 1 | Bacteria | Bacteria\_none | Pseudomonadota | Betaproteobacteria | Burkholderiales | Oxalobacteraceae | Massilia | Bacteria;Bacteria\_none;Pseudomonadota;Betaproteobacteria;Burkholderiales;Oxalobacteraceae;Massilia;Massilia litorea |
| Noviherbaspirillum sp. UKPF54 | 0 | 0 | 1 | 0 | 0 | 0 | 0 | 0 | 0 | 0 | 0 | 0 | 0 | 1 | Bacteria | Bacteria\_none | Pseudomonadota | Betaproteobacteria | Burkholderiales | Oxalobacteraceae | Noviherbaspirillum | Bacteria;Bacteria\_none;Pseudomonadota;Betaproteobacteria;Burkholderiales;Oxalobacteraceae;Noviherbaspirillum;Noviherbaspirillum sp. UKPF54 |
| Oxalobacter formigenes | 0 | 0 | 0 | 0 | 0 | 0 | 0 | 0 | 0 | 0 | 0 | 0 | 1 | 1 | Bacteria | Bacteria\_none | Pseudomonadota | Betaproteobacteria | Burkholderiales | Oxalobacteraceae | Oxalobacter | Bacteria;Bacteria\_none;Pseudomonadota;Betaproteobacteria;Burkholderiales;Oxalobacteraceae;Oxalobacter;Oxalobacter formigenes |
| Pseudoduganella plicata | 0 | 0 | 0 | 0 | 0 | 0 | 0 | 0 | 1 | 0 | 0 | 0 | 0 | 1 | Bacteria | Bacteria\_none | Pseudomonadota | Betaproteobacteria | Burkholderiales | Oxalobacteraceae | Pseudoduganella | Bacteria;Bacteria\_none;Pseudomonadota;Betaproteobacteria;Burkholderiales;Oxalobacteraceae;Pseudoduganella;Pseudoduganella plicata |
| Piscinibacter gummiphilus | 0 | 0 | 0 | 0 | 0 | 0 | 0 | 0 | 0 | 0 | 0 | 1 | 0 | 1 | Bacteria | Bacteria\_none | Pseudomonadota | Betaproteobacteria | Burkholderiales | Sphaerotilaceae | Piscinibacter | Bacteria;Bacteria\_none;Pseudomonadota;Betaproteobacteria;Burkholderiales;Sphaerotilaceae;Piscinibacter;Piscinibacter gummiphilus |
| Rubrivivax gelatinosus | 0 | 0 | 0 | 0 | 0 | 0 | 0 | 0 | 0 | 0 | 1 | 0 | 0 | 1 | Bacteria | Bacteria\_none | Pseudomonadota | Betaproteobacteria | Burkholderiales | Sphaerotilaceae | Rubrivivax | Bacteria;Bacteria\_none;Pseudomonadota;Betaproteobacteria;Burkholderiales;Sphaerotilaceae;Rubrivivax;Rubrivivax gelatinosus |
| Alysiella crassa | 0 | 0 | 0 | 0 | 0 | 0 | 0 | 0 | 0 | 0 | 0 | 1 | 0 | 1 | Bacteria | Bacteria\_none | Pseudomonadota | Betaproteobacteria | Neisseriales | Neisseriaceae | Alysiella | Bacteria;Bacteria\_none;Pseudomonadota;Betaproteobacteria;Neisseriales;Neisseriaceae;Alysiella;Alysiella crassa |
| Alysiella filiformis | 0 | 0 | 0 | 0 | 1 | 0 | 0 | 0 | 0 | 0 | 0 | 0 | 0 | 1 | Bacteria | Bacteria\_none | Pseudomonadota | Betaproteobacteria | Neisseriales | Neisseriaceae | Alysiella | Bacteria;Bacteria\_none;Pseudomonadota;Betaproteobacteria;Neisseriales;Neisseriaceae;Alysiella;Alysiella filiformis |
| Aquella oligotrophica | 0 | 0 | 0 | 0 | 0 | 1 | 0 | 0 | 0 | 0 | 0 | 0 | 0 | 1 | Bacteria | Bacteria\_none | Pseudomonadota | Betaproteobacteria | Neisseriales | Neisseriaceae | Aquella | Bacteria;Bacteria\_none;Pseudomonadota;Betaproteobacteria;Neisseriales;Neisseriaceae;Aquella;Aquella oligotrophica |
| Conchiformibius steedae | 0 | 0 | 1 | 0 | 0 | 0 | 0 | 0 | 0 | 0 | 0 | 0 | 0 | 1 | Bacteria | Bacteria\_none | Pseudomonadota | Betaproteobacteria | Neisseriales | Neisseriaceae | Conchiformibius | Bacteria;Bacteria\_none;Pseudomonadota;Betaproteobacteria;Neisseriales;Neisseriaceae;Conchiformibius;Conchiformibius steedae |
| Eikenella corrodens | 0 | 2 | 29 | 7 | 15 | 0 | 0 | 3 | 0 | 2 | 4 | 0 | 14 | 76 | Bacteria | Bacteria\_none | Pseudomonadota | Betaproteobacteria | Neisseriales | Neisseriaceae | Eikenella | Bacteria;Bacteria\_none;Pseudomonadota;Betaproteobacteria;Neisseriales;Neisseriaceae;Eikenella;Eikenella corrodens |
| Eikenella exigua | 0 | 0 | 1 | 2 | 0 | 0 | 0 | 0 | 0 | 1 | 1 | 1 | 1 | 7 | Bacteria | Bacteria\_none | Pseudomonadota | Betaproteobacteria | Neisseriales | Neisseriaceae | Eikenella | Bacteria;Bacteria\_none;Pseudomonadota;Betaproteobacteria;Neisseriales;Neisseriaceae;Eikenella;Eikenella exigua |
| Kingella denitrificans | 0 | 16 | 1 | 41 | 0 | 0 | 0 | 0 | 0 | 0 | 3 | 0 | 5 | 66 | Bacteria | Bacteria\_none | Pseudomonadota | Betaproteobacteria | Neisseriales | Neisseriaceae | Kingella | Bacteria;Bacteria\_none;Pseudomonadota;Betaproteobacteria;Neisseriales;Neisseriaceae;Kingella;Kingella denitrificans |
| Kingella kingae | 0 | 0 | 0 | 0 | 0 | 0 | 0 | 0 | 0 | 1 | 0 | 0 | 1 | 2 | Bacteria | Bacteria\_none | Pseudomonadota | Betaproteobacteria | Neisseriales | Neisseriaceae | Kingella | Bacteria;Bacteria\_none;Pseudomonadota;Betaproteobacteria;Neisseriales;Neisseriaceae;Kingella;Kingella kingae |
| Kingella negevensis | 0 | 0 | 0 | 1 | 0 | 1 | 0 | 0 | 0 | 4 | 0 | 0 | 0 | 6 | Bacteria | Bacteria\_none | Pseudomonadota | Betaproteobacteria | Neisseriales | Neisseriaceae | Kingella | Bacteria;Bacteria\_none;Pseudomonadota;Betaproteobacteria;Neisseriales;Neisseriaceae;Kingella;Kingella negevensis |
| Kingella oralis | 1 | 9 | 2 | 1 | 0 | 0 | 0 | 4 | 0 | 12 | 1 | 2 | 1 | 33 | Bacteria | Bacteria\_none | Pseudomonadota | Betaproteobacteria | Neisseriales | Neisseriaceae | Kingella | Bacteria;Bacteria\_none;Pseudomonadota;Betaproteobacteria;Neisseriales;Neisseriaceae;Kingella;Kingella oralis |
| Morococcus cerebrosus | 0 | 3 | 9 | 26 | 0 | 0 | 0 | 2 | 0 | 3 | 2 | 4 | 25 | 74 | Bacteria | Bacteria\_none | Pseudomonadota | Betaproteobacteria | Neisseriales | Neisseriaceae | Morococcus | Bacteria;Bacteria\_none;Pseudomonadota;Betaproteobacteria;Neisseriales;Neisseriaceae;Morococcus;Morococcus cerebrosus |
| Neisseria animalis | 0 | 1 | 1 | 5 | 0 | 0 | 0 | 0 | 0 | 1 | 0 | 0 | 1 | 9 | Bacteria | Bacteria\_none | Pseudomonadota | Betaproteobacteria | Neisseriales | Neisseriaceae | Neisseria | Bacteria;Bacteria\_none;Pseudomonadota;Betaproteobacteria;Neisseriales;Neisseriaceae;Neisseria;Neisseria animalis |
| Neisseria animaloris | 1 | 0 | 0 | 2 | 1 | 0 | 0 | 0 | 0 | 2 | 0 | 0 | 2 | 8 | Bacteria | Bacteria\_none | Pseudomonadota | Betaproteobacteria | Neisseriales | Neisseriaceae | Neisseria | Bacteria;Bacteria\_none;Pseudomonadota;Betaproteobacteria;Neisseriales;Neisseriaceae;Neisseria;Neisseria animaloris |
| Neisseria arctica | 0 | 0 | 0 | 8 | 0 | 0 | 0 | 0 | 0 | 3 | 0 | 0 | 1 | 12 | Bacteria | Bacteria\_none | Pseudomonadota | Betaproteobacteria | Neisseriales | Neisseriaceae | Neisseria | Bacteria;Bacteria\_none;Pseudomonadota;Betaproteobacteria;Neisseriales;Neisseriaceae;Neisseria;Neisseria arctica |
| Neisseria bacilliformis | 0 | 2 | 0 | 4 | 0 | 0 | 0 | 3 | 0 | 0 | 0 | 0 | 1 | 10 | Bacteria | Bacteria\_none | Pseudomonadota | Betaproteobacteria | Neisseriales | Neisseriaceae | Neisseria | Bacteria;Bacteria\_none;Pseudomonadota;Betaproteobacteria;Neisseriales;Neisseriaceae;Neisseria;Neisseria bacilliformis |
| Neisseria brasiliensis | 0 | 0 | 0 | 7 | 0 | 0 | 0 | 1 | 0 | 2 | 0 | 0 | 2 | 12 | Bacteria | Bacteria\_none | Pseudomonadota | Betaproteobacteria | Neisseriales | Neisseriaceae | Neisseria | Bacteria;Bacteria\_none;Pseudomonadota;Betaproteobacteria;Neisseriales;Neisseriaceae;Neisseria;Neisseria brasiliensis |
| Neisseria canis | 0 | 0 | 1 | 3 | 0 | 0 | 0 | 0 | 0 | 0 | 0 | 0 | 0 | 4 | Bacteria | Bacteria\_none | Pseudomonadota | Betaproteobacteria | Neisseriales | Neisseriaceae | Neisseria | Bacteria;Bacteria\_none;Pseudomonadota;Betaproteobacteria;Neisseriales;Neisseriaceae;Neisseria;Neisseria canis |
| Neisseria chenwenguii | 0 | 2 | 0 | 5 | 0 | 0 | 0 | 0 | 0 | 0 | 0 | 0 | 3 | 10 | Bacteria | Bacteria\_none | Pseudomonadota | Betaproteobacteria | Neisseriales | Neisseriaceae | Neisseria | Bacteria;Bacteria\_none;Pseudomonadota;Betaproteobacteria;Neisseriales;Neisseriaceae;Neisseria;Neisseria chenwenguii |
| Neisseria cinerea | 0 | 1 | 7 | 60 | 9 | 1 | 0 | 1 | 0 | 381 | 1 | 1 | 59 | 521 | Bacteria | Bacteria\_none | Pseudomonadota | Betaproteobacteria | Neisseriales | Neisseriaceae | Neisseria | Bacteria;Bacteria\_none;Pseudomonadota;Betaproteobacteria;Neisseriales;Neisseriaceae;Neisseria;Neisseria cinerea |
| Neisseria dentiae | 0 | 0 | 0 | 2 | 0 | 2 | 0 | 0 | 0 | 0 | 0 | 0 | 0 | 4 | Bacteria | Bacteria\_none | Pseudomonadota | Betaproteobacteria | Neisseriales | Neisseriaceae | Neisseria | Bacteria;Bacteria\_none;Pseudomonadota;Betaproteobacteria;Neisseriales;Neisseriaceae;Neisseria;Neisseria dentiae |
| Neisseria dumasiana | 0 | 0 | 1 | 2 | 0 | 0 | 0 | 0 | 0 | 0 | 0 | 0 | 0 | 3 | Bacteria | Bacteria\_none | Pseudomonadota | Betaproteobacteria | Neisseriales | Neisseriaceae | Neisseria | Bacteria;Bacteria\_none;Pseudomonadota;Betaproteobacteria;Neisseriales;Neisseriaceae;Neisseria;Neisseria dumasiana |
| Neisseria elongata | 2 | 21 | 11 | 76 | 6 | 4 | 1 | 3 | 0 | 73 | 1 | 4 | 34 | 236 | Bacteria | Bacteria\_none | Pseudomonadota | Betaproteobacteria | Neisseriales | Neisseriaceae | Neisseria | Bacteria;Bacteria\_none;Pseudomonadota;Betaproteobacteria;Neisseriales;Neisseriaceae;Neisseria;Neisseria elongata |
| Neisseria flavescens | 4 | 8 | 26 | 228 | 18 | 6 | 0 | 1 | 0 | 12 | 0 | 2 | 85 | 390 | Bacteria | Bacteria\_none | Pseudomonadota | Betaproteobacteria | Neisseriales | Neisseriaceae | Neisseria | Bacteria;Bacteria\_none;Pseudomonadota;Betaproteobacteria;Neisseriales;Neisseriaceae;Neisseria;Neisseria flavescens |
| Neisseria gonorrhoeae | 2 | 1 | 29 | 40 | 2 | 1 | 0 | 1 | 0 | 38 | 2 | 1 | 24 | 141 | Bacteria | Bacteria\_none | Pseudomonadota | Betaproteobacteria | Neisseriales | Neisseriaceae | Neisseria | Bacteria;Bacteria\_none;Pseudomonadota;Betaproteobacteria;Neisseriales;Neisseriaceae;Neisseria;Neisseria gonorrhoeae |
| Neisseria lactamica | 1 | 0 | 4 | 32 | 1 | 2 | 1 | 1 | 0 | 25 | 0 | 0 | 16 | 83 | Bacteria | Bacteria\_none | Pseudomonadota | Betaproteobacteria | Neisseriales | Neisseriaceae | Neisseria | Bacteria;Bacteria\_none;Pseudomonadota;Betaproteobacteria;Neisseriales;Neisseriaceae;Neisseria;Neisseria lactamica |
| Neisseria leonii | 0 | 0 | 0 | 0 | 0 | 1 | 0 | 0 | 0 | 0 | 0 | 0 | 0 | 1 | Bacteria | Bacteria\_none | Pseudomonadota | Betaproteobacteria | Neisseriales | Neisseriaceae | Neisseria | Bacteria;Bacteria\_none;Pseudomonadota;Betaproteobacteria;Neisseriales;Neisseriaceae;Neisseria;Neisseria leonii |
| Neisseria lisongii | 0 | 0 | 1 | 5 | 1 | 0 | 0 | 1 | 0 | 2 | 0 | 0 | 1 | 11 | Bacteria | Bacteria\_none | Pseudomonadota | Betaproteobacteria | Neisseriales | Neisseriaceae | Neisseria | Bacteria;Bacteria\_none;Pseudomonadota;Betaproteobacteria;Neisseriales;Neisseriaceae;Neisseria;Neisseria lisongii |
| Neisseria macacae | 1 | 3 | 7 | 46 | 5 | 0 | 0 | 16 | 0 | 14 | 8 | 8 | 26 | 134 | Bacteria | Bacteria\_none | Pseudomonadota | Betaproteobacteria | Neisseriales | Neisseriaceae | Neisseria | Bacteria;Bacteria\_none;Pseudomonadota;Betaproteobacteria;Neisseriales;Neisseriaceae;Neisseria;Neisseria macacae |
| Neisseria meningitidis | 5 | 5 | 35 | 202 | 7 | 5 | 2 | 5 | 0 | 127 | 0 | 1 | 60 | 454 | Bacteria | Bacteria\_none | Pseudomonadota | Betaproteobacteria | Neisseriales | Neisseriaceae | Neisseria | Bacteria;Bacteria\_none;Pseudomonadota;Betaproteobacteria;Neisseriales;Neisseriaceae;Neisseria;Neisseria meningitidis |
| Neisseria mucosa | 7 | 6 | 123 | 173 | 49 | 29 | 1 | 9 | 0 | 35 | 5 | 25 | 138 | 600 | Bacteria | Bacteria\_none | Pseudomonadota | Betaproteobacteria | Neisseriales | Neisseriaceae | Neisseria | Bacteria;Bacteria\_none;Pseudomonadota;Betaproteobacteria;Neisseriales;Neisseriaceae;Neisseria;Neisseria mucosa |
| Neisseria musculi | 0 | 0 | 1 | 1 | 0 | 0 | 0 | 0 | 0 | 0 | 0 | 0 | 3 | 5 | Bacteria | Bacteria\_none | Pseudomonadota | Betaproteobacteria | Neisseriales | Neisseriaceae | Neisseria | Bacteria;Bacteria\_none;Pseudomonadota;Betaproteobacteria;Neisseriales;Neisseriaceae;Neisseria;Neisseria musculi |
| Neisseria perflava | 8 | 7 | 50 | 141 | 26 | 18 | 0 | 4 | 0 | 369 | 0 | 0 | 317 | 940 | Bacteria | Bacteria\_none | Pseudomonadota | Betaproteobacteria | Neisseriales | Neisseriaceae | Neisseria | Bacteria;Bacteria\_none;Pseudomonadota;Betaproteobacteria;Neisseriales;Neisseriaceae;Neisseria;Neisseria perflava |
| Neisseria shayeganii | 1 | 0 | 1 | 0 | 1 | 0 | 0 | 0 | 0 | 0 | 0 | 0 | 1 | 4 | Bacteria | Bacteria\_none | Pseudomonadota | Betaproteobacteria | Neisseriales | Neisseriaceae | Neisseria | Bacteria;Bacteria\_none;Pseudomonadota;Betaproteobacteria;Neisseriales;Neisseriaceae;Neisseria;Neisseria shayeganii |
| Neisseria sicca | 28 | 19 | 152 | 607 | 14 | 6 | 1 | 28 | 0 | 32 | 5 | 11 | 175 | 1078 | Bacteria | Bacteria\_none | Pseudomonadota | Betaproteobacteria | Neisseriales | Neisseriaceae | Neisseria | Bacteria;Bacteria\_none;Pseudomonadota;Betaproteobacteria;Neisseriales;Neisseriaceae;Neisseria;Neisseria sicca |
| Neisseria sp. DTU\_2021\_1001991\_1\_SI\_NGA\_ILE\_055 | 4 | 2 | 74 | 137 | 13 | 49 | 1 | 5 | 0 | 29 | 1 | 0 | 79 | 394 | Bacteria | Bacteria\_none | Pseudomonadota | Betaproteobacteria | Neisseriales | Neisseriaceae | Neisseria | Bacteria;Bacteria\_none;Pseudomonadota;Betaproteobacteria;Neisseriales;Neisseriaceae;Neisseria;Neisseria sp. DTU\_2021\_1001991\_1\_SI\_NGA\_ILE\_055 |
| Neisseria sp. Dent CA1/247 | 0 | 0 | 0 | 0 | 0 | 0 | 0 | 0 | 0 | 1 | 0 | 0 | 0 | 1 | Bacteria | Bacteria\_none | Pseudomonadota | Betaproteobacteria | Neisseriales | Neisseriaceae | Neisseria | Bacteria;Bacteria\_none;Pseudomonadota;Betaproteobacteria;Neisseriales;Neisseriaceae;Neisseria;Neisseria sp. Dent CA1/247 |
| Neisseria sp. KEM232 | 1 | 0 | 0 | 2 | 2 | 3 | 0 | 1 | 0 | 2 | 0 | 0 | 0 | 11 | Bacteria | Bacteria\_none | Pseudomonadota | Betaproteobacteria | Neisseriales | Neisseriaceae | Neisseria | Bacteria;Bacteria\_none;Pseudomonadota;Betaproteobacteria;Neisseriales;Neisseriaceae;Neisseria;Neisseria sp. KEM232 |
| Neisseria sp. Marseille-Q5346 | 26 | 4 | 47 | 199 | 17 | 6 | 10 | 11 | 0 | 24 | 2 | 5 | 107 | 458 | Bacteria | Bacteria\_none | Pseudomonadota | Betaproteobacteria | Neisseriales | Neisseriaceae | Neisseria | Bacteria;Bacteria\_none;Pseudomonadota;Betaproteobacteria;Neisseriales;Neisseriaceae;Neisseria;Neisseria sp. Marseille-Q5346 |
| Neisseria sp. Marseille-Q6792 | 1 | 0 | 2 | 21 | 3 | 0 | 0 | 0 | 0 | 41 | 0 | 0 | 22 | 90 | Bacteria | Bacteria\_none | Pseudomonadota | Betaproteobacteria | Neisseriales | Neisseriaceae | Neisseria | Bacteria;Bacteria\_none;Pseudomonadota;Betaproteobacteria;Neisseriales;Neisseriaceae;Neisseria;Neisseria sp. Marseille-Q6792 |
| Neisseria sp. RH3002v2g | 10 | 2 | 78 | 110 | 6 | 49 | 4 | 1 | 0 | 16 | 0 | 1 | 97 | 374 | Bacteria | Bacteria\_none | Pseudomonadota | Betaproteobacteria | Neisseriales | Neisseriaceae | Neisseria | Bacteria;Bacteria\_none;Pseudomonadota;Betaproteobacteria;Neisseriales;Neisseriaceae;Neisseria;Neisseria sp. RH3002v2g |
| Neisseria sp. oral taxon 014 | 1 | 0 | 16 | 28 | 3 | 0 | 7 | 20 | 0 | 8 | 169 | 2 | 61 | 315 | Bacteria | Bacteria\_none | Pseudomonadota | Betaproteobacteria | Neisseriales | Neisseriaceae | Neisseria | Bacteria;Bacteria\_none;Pseudomonadota;Betaproteobacteria;Neisseriales;Neisseriaceae;Neisseria;Neisseria sp. oral taxon 014 |
| Neisseria subflava | 101 | 87 | 374 | 1856 | 157 | 142 | 32 | 73 | 1 | 416 | 4 | 54 | 825 | 4122 | Bacteria | Bacteria\_none | Pseudomonadota | Betaproteobacteria | Neisseriales | Neisseriaceae | Neisseria | Bacteria;Bacteria\_none;Pseudomonadota;Betaproteobacteria;Neisseriales;Neisseriaceae;Neisseria;Neisseria subflava |
| Neisseria wadsworthii | 0 | 0 | 0 | 0 | 0 | 0 | 1 | 0 | 0 | 0 | 0 | 0 | 1 | 2 | Bacteria | Bacteria\_none | Pseudomonadota | Betaproteobacteria | Neisseriales | Neisseriaceae | Neisseria | Bacteria;Bacteria\_none;Pseudomonadota;Betaproteobacteria;Neisseriales;Neisseriaceae;Neisseria;Neisseria wadsworthii |
| Neisseria weaveri | 0 | 0 | 0 | 3 | 0 | 0 | 0 | 0 | 0 | 3 | 0 | 0 | 0 | 6 | Bacteria | Bacteria\_none | Pseudomonadota | Betaproteobacteria | Neisseriales | Neisseriaceae | Neisseria | Bacteria;Bacteria\_none;Pseudomonadota;Betaproteobacteria;Neisseriales;Neisseriaceae;Neisseria;Neisseria weaveri |
| Neisseria weixii | 0 | 0 | 0 | 2 | 0 | 0 | 0 | 0 | 0 | 1 | 0 | 0 | 1 | 4 | Bacteria | Bacteria\_none | Pseudomonadota | Betaproteobacteria | Neisseriales | Neisseriaceae | Neisseria | Bacteria;Bacteria\_none;Pseudomonadota;Betaproteobacteria;Neisseriales;Neisseriaceae;Neisseria;Neisseria weixii |
| Neisseria yangbaofengii | 0 | 1 | 1 | 6 | 0 | 1 | 0 | 0 | 0 | 0 | 0 | 0 | 3 | 12 | Bacteria | Bacteria\_none | Pseudomonadota | Betaproteobacteria | Neisseriales | Neisseriaceae | Neisseria | Bacteria;Bacteria\_none;Pseudomonadota;Betaproteobacteria;Neisseriales;Neisseriaceae;Neisseria;Neisseria yangbaofengii |
| Neisseria zalophi | 0 | 0 | 0 | 1 | 0 | 0 | 0 | 0 | 0 | 1 | 0 | 0 | 1 | 3 | Bacteria | Bacteria\_none | Pseudomonadota | Betaproteobacteria | Neisseriales | Neisseriaceae | Neisseria | Bacteria;Bacteria\_none;Pseudomonadota;Betaproteobacteria;Neisseriales;Neisseriaceae;Neisseria;Neisseria zalophi |
| Neisseria zoodegmatis | 0 | 0 | 0 | 4 | 0 | 0 | 0 | 0 | 0 | 0 | 0 | 0 | 1 | 5 | Bacteria | Bacteria\_none | Pseudomonadota | Betaproteobacteria | Neisseriales | Neisseriaceae | Neisseria | Bacteria;Bacteria\_none;Pseudomonadota;Betaproteobacteria;Neisseriales;Neisseriaceae;Neisseria;Neisseria zoodegmatis |
| Simonsiella muelleri | 0 | 0 | 0 | 1 | 0 | 0 | 0 | 0 | 0 | 0 | 0 | 0 | 0 | 1 | Bacteria | Bacteria\_none | Pseudomonadota | Betaproteobacteria | Neisseriales | Neisseriaceae | Simonsiella | Bacteria;Bacteria\_none;Pseudomonadota;Betaproteobacteria;Neisseriales;Neisseriaceae;Simonsiella;Simonsiella muelleri |
| Snodgrassella alvi | 0 | 0 | 0 | 0 | 0 | 1 | 0 | 0 | 0 | 0 | 0 | 0 | 0 | 1 | Bacteria | Bacteria\_none | Pseudomonadota | Betaproteobacteria | Neisseriales | Neisseriaceae | Snodgrassella | Bacteria;Bacteria\_none;Pseudomonadota;Betaproteobacteria;Neisseriales;Neisseriaceae;Snodgrassella;Snodgrassella alvi |
| Uruburuella testudinis | 0 | 0 | 0 | 0 | 0 | 0 | 0 | 0 | 0 | 0 | 0 | 0 | 1 | 1 | Bacteria | Bacteria\_none | Pseudomonadota | Betaproteobacteria | Neisseriales | Neisseriaceae | Uruburuella | Bacteria;Bacteria\_none;Pseudomonadota;Betaproteobacteria;Neisseriales;Neisseriaceae;Uruburuella;Uruburuella testudinis |
| Ferriphaselus amnicola | 0 | 0 | 0 | 0 | 0 | 0 | 0 | 0 | 0 | 0 | 0 | 0 | 1 | 1 | Bacteria | Bacteria\_none | Pseudomonadota | Betaproteobacteria | Nitrosomonadales | Gallionellaceae | Ferriphaselus | Bacteria;Bacteria\_none;Pseudomonadota;Betaproteobacteria;Nitrosomonadales;Gallionellaceae;Ferriphaselus;Ferriphaselus amnicola |
| Methylophilus sp. DW102 | 0 | 0 | 0 | 0 | 0 | 0 | 0 | 0 | 0 | 0 | 0 | 0 | 1 | 1 | Bacteria | Bacteria\_none | Pseudomonadota | Betaproteobacteria | Nitrosomonadales | Methylophilaceae | Methylophilus | Bacteria;Bacteria\_none;Pseudomonadota;Betaproteobacteria;Nitrosomonadales;Methylophilaceae;Methylophilus;Methylophilus sp. DW102 |
| Dechloromonas sp. A34 | 0 | 0 | 0 | 0 | 0 | 0 | 1 | 0 | 0 | 0 | 0 | 0 | 0 | 1 | Bacteria | Bacteria\_none | Pseudomonadota | Betaproteobacteria | Rhodocyclales | Azonexaceae | Dechloromonas | Bacteria;Bacteria\_none;Pseudomonadota;Betaproteobacteria;Rhodocyclales;Azonexaceae;Dechloromonas;Dechloromonas sp. A34 |
| Aeromonas veronii | 0 | 0 | 0 | 0 | 0 | 1 | 0 | 0 | 0 | 0 | 0 | 0 | 0 | 1 | Bacteria | Bacteria\_none | Pseudomonadota | Gammaproteobacteria | Aeromonadales | Aeromonadaceae | Aeromonas | Bacteria;Bacteria\_none;Pseudomonadota;Gammaproteobacteria;Aeromonadales;Aeromonadaceae;Aeromonas;Aeromonas veronii |
| Oceanisphaera avium | 0 | 0 | 0 | 0 | 0 | 0 | 0 | 0 | 0 | 0 | 0 | 0 | 1 | 1 | Bacteria | Bacteria\_none | Pseudomonadota | Gammaproteobacteria | Aeromonadales | Aeromonadaceae | Oceanisphaera | Bacteria;Bacteria\_none;Pseudomonadota;Gammaproteobacteria;Aeromonadales;Aeromonadaceae;Oceanisphaera;Oceanisphaera avium |
| Tolumonas auensis | 0 | 0 | 0 | 0 | 0 | 1 | 0 | 0 | 0 | 0 | 0 | 0 | 0 | 1 | Bacteria | Bacteria\_none | Pseudomonadota | Gammaproteobacteria | Aeromonadales | Aeromonadaceae | Tolumonas | Bacteria;Bacteria\_none;Pseudomonadota;Gammaproteobacteria;Aeromonadales;Aeromonadaceae;Tolumonas;Tolumonas auensis |
| Alteromonas macleodii | 0 | 0 | 0 | 0 | 0 | 0 | 0 | 0 | 0 | 0 | 0 | 0 | 2 | 2 | Bacteria | Bacteria\_none | Pseudomonadota | Gammaproteobacteria | Alteromonadales | Alteromonadaceae | Alteromonas | Bacteria;Bacteria\_none;Pseudomonadota;Gammaproteobacteria;Alteromonadales;Alteromonadaceae;Alteromonas;Alteromonas macleodii |
| Alteromonas stellipolaris | 1 | 0 | 0 | 0 | 0 | 0 | 0 | 0 | 0 | 0 | 0 | 0 | 0 | 1 | Bacteria | Bacteria\_none | Pseudomonadota | Gammaproteobacteria | Alteromonadales | Alteromonadaceae | Alteromonas | Bacteria;Bacteria\_none;Pseudomonadota;Gammaproteobacteria;Alteromonadales;Alteromonadaceae;Alteromonas;Alteromonas stellipolaris |
| Thalassotalea piscium | 0 | 0 | 1 | 0 | 0 | 0 | 0 | 0 | 0 | 0 | 0 | 0 | 0 | 1 | Bacteria | Bacteria\_none | Pseudomonadota | Gammaproteobacteria | Alteromonadales | Colwelliaceae | Thalassotalea | Bacteria;Bacteria\_none;Pseudomonadota;Gammaproteobacteria;Alteromonadales;Colwelliaceae;Thalassotalea;Thalassotalea piscium |
| Ferrimonas sp. SCSIO 43195 | 0 | 0 | 0 | 0 | 1 | 0 | 0 | 0 | 0 | 0 | 0 | 0 | 0 | 1 | Bacteria | Bacteria\_none | Pseudomonadota | Gammaproteobacteria | Alteromonadales | Ferrimonadaceae | Ferrimonas | Bacteria;Bacteria\_none;Pseudomonadota;Gammaproteobacteria;Alteromonadales;Ferrimonadaceae;Ferrimonas;Ferrimonas sp. SCSIO 43195 |
| Flocculibacter collagenilyticus | 1 | 0 | 0 | 0 | 0 | 0 | 0 | 0 | 0 | 0 | 0 | 0 | 0 | 1 | Bacteria | Bacteria\_none | Pseudomonadota | Gammaproteobacteria | Alteromonadales | Pseudoalteromonadaceae | Flocculibacter | Bacteria;Bacteria\_none;Pseudomonadota;Gammaproteobacteria;Alteromonadales;Pseudoalteromonadaceae;Flocculibacter;Flocculibacter collagenilyticus |
| Pseudoalteromonas luteoviolacea | 0 | 0 | 0 | 0 | 1 | 0 | 0 | 0 | 0 | 0 | 0 | 0 | 0 | 1 | Bacteria | Bacteria\_none | Pseudomonadota | Gammaproteobacteria | Alteromonadales | Pseudoalteromonadaceae | Pseudoalteromonas | Bacteria;Bacteria\_none;Pseudomonadota;Gammaproteobacteria;Alteromonadales;Pseudoalteromonadaceae;Pseudoalteromonas;Pseudoalteromonas luteoviolacea |
| Pseudoalteromonas piscicida | 0 | 0 | 0 | 0 | 0 | 0 | 0 | 0 | 0 | 0 | 1 | 0 | 0 | 1 | Bacteria | Bacteria\_none | Pseudomonadota | Gammaproteobacteria | Alteromonadales | Pseudoalteromonadaceae | Pseudoalteromonas | Bacteria;Bacteria\_none;Pseudomonadota;Gammaproteobacteria;Alteromonadales;Pseudoalteromonadaceae;Pseudoalteromonas;Pseudoalteromonas piscicida |
| Pseudoalteromonas sp. CuT4-3 | 0 | 0 | 0 | 0 | 0 | 1 | 0 | 0 | 0 | 0 | 0 | 0 | 0 | 1 | Bacteria | Bacteria\_none | Pseudomonadota | Gammaproteobacteria | Alteromonadales | Pseudoalteromonadaceae | Pseudoalteromonas | Bacteria;Bacteria\_none;Pseudomonadota;Gammaproteobacteria;Alteromonadales;Pseudoalteromonadaceae;Pseudoalteromonas;Pseudoalteromonas sp. CuT4-3 |
| Pseudoalteromonas sp. N1230-9 | 0 | 0 | 0 | 0 | 0 | 1 | 0 | 0 | 0 | 0 | 0 | 0 | 0 | 1 | Bacteria | Bacteria\_none | Pseudomonadota | Gammaproteobacteria | Alteromonadales | Pseudoalteromonadaceae | Pseudoalteromonas | Bacteria;Bacteria\_none;Pseudomonadota;Gammaproteobacteria;Alteromonadales;Pseudoalteromonadaceae;Pseudoalteromonas;Pseudoalteromonas sp. N1230-9 |
| Pseudoalteromonas sp. SCSIO 43088 | 0 | 0 | 0 | 0 | 0 | 0 | 0 | 1 | 0 | 0 | 0 | 0 | 0 | 1 | Bacteria | Bacteria\_none | Pseudomonadota | Gammaproteobacteria | Alteromonadales | Pseudoalteromonadaceae | Pseudoalteromonas | Bacteria;Bacteria\_none;Pseudomonadota;Gammaproteobacteria;Alteromonadales;Pseudoalteromonadaceae;Pseudoalteromonas;Pseudoalteromonas sp. SCSIO 43088 |
| Pseudoalteromonas xiamenensis | 0 | 0 | 0 | 0 | 0 | 0 | 0 | 1 | 0 | 0 | 0 | 0 | 0 | 1 | Bacteria | Bacteria\_none | Pseudomonadota | Gammaproteobacteria | Alteromonadales | Pseudoalteromonadaceae | Pseudoalteromonas | Bacteria;Bacteria\_none;Pseudomonadota;Gammaproteobacteria;Alteromonadales;Pseudoalteromonadaceae;Pseudoalteromonas;Pseudoalteromonas xiamenensis |
| Shewanella algae | 1 | 0 | 0 | 0 | 1 | 0 | 0 | 0 | 0 | 0 | 0 | 0 | 0 | 2 | Bacteria | Bacteria\_none | Pseudomonadota | Gammaproteobacteria | Alteromonadales | Shewanellaceae | Shewanella | Bacteria;Bacteria\_none;Pseudomonadota;Gammaproteobacteria;Alteromonadales;Shewanellaceae;Shewanella;Shewanella algae |
| Shewanella dokdonensis | 0 | 0 | 0 | 0 | 0 | 0 | 0 | 1 | 0 | 0 | 0 | 0 | 0 | 1 | Bacteria | Bacteria\_none | Pseudomonadota | Gammaproteobacteria | Alteromonadales | Shewanellaceae | Shewanella | Bacteria;Bacteria\_none;Pseudomonadota;Gammaproteobacteria;Alteromonadales;Shewanellaceae;Shewanella;Shewanella dokdonensis |
| Shewanella psychromarinicola | 0 | 1 | 0 | 0 | 0 | 0 | 0 | 0 | 0 | 0 | 0 | 0 | 0 | 1 | Bacteria | Bacteria\_none | Pseudomonadota | Gammaproteobacteria | Alteromonadales | Shewanellaceae | Shewanella | Bacteria;Bacteria\_none;Pseudomonadota;Gammaproteobacteria;Alteromonadales;Shewanellaceae;Shewanella;Shewanella psychromarinicola |
| Shewanella sp. MEBiC00475 | 0 | 0 | 0 | 0 | 0 | 0 | 0 | 0 | 0 | 0 | 0 | 1 | 0 | 1 | Bacteria | Bacteria\_none | Pseudomonadota | Gammaproteobacteria | Alteromonadales | Shewanellaceae | Shewanella | Bacteria;Bacteria\_none;Pseudomonadota;Gammaproteobacteria;Alteromonadales;Shewanellaceae;Shewanella;Shewanella sp. MEBiC00475 |
| Cardiobacterium hominis | 0 | 2 | 2 | 3 | 1 | 6 | 0 | 0 | 0 | 1 | 9 | 0 | 5 | 29 | Bacteria | Bacteria\_none | Pseudomonadota | Gammaproteobacteria | Cardiobacteriales | Cardiobacteriaceae | Cardiobacterium | Bacteria;Bacteria\_none;Pseudomonadota;Gammaproteobacteria;Cardiobacteriales;Cardiobacteriaceae;Cardiobacterium;Cardiobacterium hominis |
| Cardiobacterium sp. Marseille-Q4385 | 0 | 0 | 12 | 0 | 1 | 0 | 0 | 2 | 0 | 0 | 5 | 2 | 2 | 24 | Bacteria | Bacteria\_none | Pseudomonadota | Gammaproteobacteria | Cardiobacteriales | Cardiobacteriaceae | Cardiobacterium | Bacteria;Bacteria\_none;Pseudomonadota;Gammaproteobacteria;Cardiobacteriales;Cardiobacteriaceae;Cardiobacterium;Cardiobacterium sp. Marseille-Q4385 |
| Ignatzschineria rhizosphaerae | 0 | 1 | 0 | 0 | 0 | 0 | 0 | 0 | 0 | 0 | 0 | 0 | 0 | 1 | Bacteria | Bacteria\_none | Pseudomonadota | Gammaproteobacteria | Cardiobacteriales | Ignatzschineriaceae | Ignatzschineria | Bacteria;Bacteria\_none;Pseudomonadota;Gammaproteobacteria;Cardiobacteriales;Ignatzschineriaceae;Ignatzschineria;Ignatzschineria rhizosphaerae |
| Agarilytica rhodophyticola | 0 | 0 | 0 | 0 | 0 | 0 | 0 | 1 | 0 | 0 | 0 | 0 | 0 | 1 | Bacteria | Bacteria\_none | Pseudomonadota | Gammaproteobacteria | Cellvibrionales | Cellvibrionaceae | Agarilytica | Bacteria;Bacteria\_none;Pseudomonadota;Gammaproteobacteria;Cellvibrionales;Cellvibrionaceae;Agarilytica;Agarilytica rhodophyticola |
| Allochromatium vinosum | 0 | 0 | 0 | 0 | 0 | 0 | 0 | 0 | 0 | 0 | 0 | 0 | 1 | 1 | Bacteria | Bacteria\_none | Pseudomonadota | Gammaproteobacteria | Chromatiales | Chromatiaceae | Allochromatium | Bacteria;Bacteria\_none;Pseudomonadota;Gammaproteobacteria;Chromatiales;Chromatiaceae;Allochromatium;Allochromatium vinosum |
| Candidatus Nitrosoglobus terrae | 0 | 0 | 0 | 0 | 0 | 0 | 0 | 0 | 0 | 0 | 0 | 0 | 1 | 1 | Bacteria | Bacteria\_none | Pseudomonadota | Gammaproteobacteria | Chromatiales | Chromatiaceae | Candidatus Nitrosoglobus | Bacteria;Bacteria\_none;Pseudomonadota;Gammaproteobacteria;Chromatiales;Chromatiaceae;Candidatus Nitrosoglobus;Candidatus Nitrosoglobus terrae |
| Thioalkalivibrio paradoxus | 0 | 0 | 0 | 1 | 0 | 0 | 0 | 0 | 0 | 0 | 0 | 0 | 0 | 1 | Bacteria | Bacteria\_none | Pseudomonadota | Gammaproteobacteria | Chromatiales | Ectothiorhodospiraceae | Thioalkalivibrio | Bacteria;Bacteria\_none;Pseudomonadota;Gammaproteobacteria;Chromatiales;Ectothiorhodospiraceae;Thioalkalivibrio;Thioalkalivibrio paradoxus |
| Limnobaculum zhutongyuii | 0 | 0 | 0 | 0 | 0 | 0 | 0 | 1 | 0 | 0 | 0 | 0 | 0 | 1 | Bacteria | Bacteria\_none | Pseudomonadota | Gammaproteobacteria | Enterobacterales | Budviciaceae | Limnobaculum | Bacteria;Bacteria\_none;Pseudomonadota;Gammaproteobacteria;Enterobacterales;Budviciaceae;Limnobaculum;Limnobaculum zhutongyuii |
| Candidatus Blochmanniella vafra | 0 | 0 | 0 | 0 | 0 | 0 | 0 | 0 | 0 | 1 | 0 | 0 | 0 | 1 | Bacteria | Bacteria\_none | Pseudomonadota | Gammaproteobacteria | Enterobacterales | Enterobacteriaceae | Candidatus Blochmanniella | Bacteria;Bacteria\_none;Pseudomonadota;Gammaproteobacteria;Enterobacterales;Enterobacteriaceae;Candidatus Blochmanniella;Candidatus Blochmanniella vafra |
| Candidatus Profftia sp. (ex Adelges kitamiensis) | 0 | 0 | 0 | 0 | 0 | 0 | 0 | 0 | 0 | 1 | 0 | 0 | 0 | 1 | Bacteria | Bacteria\_none | Pseudomonadota | Gammaproteobacteria | Enterobacterales | Enterobacteriaceae | Candidatus Profftia | Bacteria;Bacteria\_none;Pseudomonadota;Gammaproteobacteria;Enterobacterales;Enterobacteriaceae;Candidatus Profftia;Candidatus Profftia sp. (ex Adelges kitamiensis) |
| Candidatus Purcelliella pentastirinorum | 0 | 0 | 0 | 0 | 0 | 1 | 0 | 0 | 0 | 0 | 0 | 0 | 0 | 1 | Bacteria | Bacteria\_none | Pseudomonadota | Gammaproteobacteria | Enterobacterales | Enterobacteriaceae | Candidatus Purcelliella | Bacteria;Bacteria\_none;Pseudomonadota;Gammaproteobacteria;Enterobacterales;Enterobacteriaceae;Candidatus Purcelliella;Candidatus Purcelliella pentastirinorum |
| Enterobacter cloacae | 0 | 0 | 0 | 0 | 0 | 0 | 0 | 0 | 0 | 2 | 0 | 0 | 1 | 3 | Bacteria | Bacteria\_none | Pseudomonadota | Gammaproteobacteria | Enterobacterales | Enterobacteriaceae | Enterobacter | Bacteria;Bacteria\_none;Pseudomonadota;Gammaproteobacteria;Enterobacterales;Enterobacteriaceae;Enterobacter;Enterobacter cloacae |
| Enterobacteriaceae endosymbiont of Donacia thalassina | 1 | 0 | 0 | 0 | 0 | 0 | 0 | 0 | 0 | 0 | 0 | 0 | 0 | 1 | Bacteria | Bacteria\_none | Pseudomonadota | Gammaproteobacteria | Enterobacterales | Enterobacteriaceae | Enterobacteriaceae\_Incertae\_sedis | Bacteria;Bacteria\_none;Pseudomonadota;Gammaproteobacteria;Enterobacterales;Enterobacteriaceae;Enterobacteriaceae\_Incertae\_sedis;Enterobacteriaceae endosymbiont of Donacia thalassina |
| Escherichia coli | 1 | 0 | 0 | 0 | 0 | 0 | 0 | 0 | 0 | 0 | 0 | 0 | 2 | 3 | Bacteria | Bacteria\_none | Pseudomonadota | Gammaproteobacteria | Enterobacterales | Enterobacteriaceae | Escherichia | Bacteria;Bacteria\_none;Pseudomonadota;Gammaproteobacteria;Enterobacterales;Enterobacteriaceae;Escherichia;Escherichia coli |
| Escherichia marmotae | 0 | 0 | 0 | 0 | 1 | 0 | 0 | 0 | 0 | 0 | 0 | 1 | 0 | 2 | Bacteria | Bacteria\_none | Pseudomonadota | Gammaproteobacteria | Enterobacterales | Enterobacteriaceae | Escherichia | Bacteria;Bacteria\_none;Pseudomonadota;Gammaproteobacteria;Enterobacterales;Enterobacteriaceae;Escherichia;Escherichia marmotae |
| Klebsiella aerogenes | 0 | 0 | 0 | 0 | 0 | 0 | 0 | 0 | 0 | 0 | 0 | 0 | 2 | 2 | Bacteria | Bacteria\_none | Pseudomonadota | Gammaproteobacteria | Enterobacterales | Enterobacteriaceae | Klebsiella | Bacteria;Bacteria\_none;Pseudomonadota;Gammaproteobacteria;Enterobacterales;Enterobacteriaceae;Klebsiella;Klebsiella aerogenes |
| Klebsiella pneumoniae | 0 | 0 | 0 | 4 | 4 | 0 | 0 | 0 | 0 | 0 | 0 | 0 | 0 | 8 | Bacteria | Bacteria\_none | Pseudomonadota | Gammaproteobacteria | Enterobacterales | Enterobacteriaceae | Klebsiella | Bacteria;Bacteria\_none;Pseudomonadota;Gammaproteobacteria;Enterobacterales;Enterobacteriaceae;Klebsiella;Klebsiella pneumoniae |
| Salmonella enterica | 2 | 0 | 0 | 0 | 0 | 0 | 0 | 4 | 0 | 0 | 0 | 0 | 6 | 12 | Bacteria | Bacteria\_none | Pseudomonadota | Gammaproteobacteria | Enterobacterales | Enterobacteriaceae | Salmonella | Bacteria;Bacteria\_none;Pseudomonadota;Gammaproteobacteria;Enterobacterales;Enterobacteriaceae;Salmonella;Salmonella enterica |
| Symbiopectobacterium purcellii | 0 | 0 | 0 | 0 | 0 | 0 | 0 | 0 | 0 | 0 | 0 | 0 | 1 | 1 | Bacteria | Bacteria\_none | Pseudomonadota | Gammaproteobacteria | Enterobacterales | Enterobacteriaceae | Symbiopectobacterium | Bacteria;Bacteria\_none;Pseudomonadota;Gammaproteobacteria;Enterobacterales;Enterobacteriaceae;Symbiopectobacterium;Symbiopectobacterium purcellii |
| Buchnera aphidicola | 0 | 0 | 0 | 0 | 0 | 0 | 0 | 1 | 0 | 0 | 0 | 0 | 2 | 3 | Bacteria | Bacteria\_none | Pseudomonadota | Gammaproteobacteria | Enterobacterales | Erwiniaceae | Buchnera | Bacteria;Bacteria\_none;Pseudomonadota;Gammaproteobacteria;Enterobacterales;Erwiniaceae;Buchnera;Buchnera aphidicola |
| Candidatus Erwinia haradaeae | 0 | 0 | 0 | 0 | 1 | 0 | 0 | 1 | 0 | 0 | 0 | 0 | 0 | 2 | Bacteria | Bacteria\_none | Pseudomonadota | Gammaproteobacteria | Enterobacterales | Erwiniaceae | Erwinia | Bacteria;Bacteria\_none;Pseudomonadota;Gammaproteobacteria;Enterobacterales;Erwiniaceae;Erwinia;Candidatus Erwinia haradaeae |
| Erwinia aphidicola | 0 | 0 | 0 | 0 | 0 | 0 | 0 | 0 | 0 | 0 | 0 | 1 | 0 | 1 | Bacteria | Bacteria\_none | Pseudomonadota | Gammaproteobacteria | Enterobacterales | Erwiniaceae | Erwinia | Bacteria;Bacteria\_none;Pseudomonadota;Gammaproteobacteria;Enterobacterales;Erwiniaceae;Erwinia;Erwinia aphidicola |
| Pantoea vagans | 0 | 0 | 0 | 0 | 0 | 0 | 0 | 0 | 0 | 0 | 1 | 0 | 0 | 1 | Bacteria | Bacteria\_none | Pseudomonadota | Gammaproteobacteria | Enterobacterales | Erwiniaceae | Pantoea | Bacteria;Bacteria\_none;Pseudomonadota;Gammaproteobacteria;Enterobacterales;Erwiniaceae;Pantoea;Pantoea vagans |
| Tatumella ptyseos | 0 | 0 | 0 | 0 | 0 | 0 | 0 | 1 | 0 | 0 | 0 | 0 | 0 | 1 | Bacteria | Bacteria\_none | Pseudomonadota | Gammaproteobacteria | Enterobacterales | Erwiniaceae | Tatumella | Bacteria;Bacteria\_none;Pseudomonadota;Gammaproteobacteria;Enterobacterales;Erwiniaceae;Tatumella;Tatumella ptyseos |
| Arsenophonus nasoniae | 0 | 0 | 0 | 0 | 0 | 0 | 0 | 0 | 1 | 0 | 0 | 0 | 0 | 1 | Bacteria | Bacteria\_none | Pseudomonadota | Gammaproteobacteria | Enterobacterales | Morganellaceae | Arsenophonus | Bacteria;Bacteria\_none;Pseudomonadota;Gammaproteobacteria;Enterobacterales;Morganellaceae;Arsenophonus;Arsenophonus nasoniae |
| Morganella morganii | 0 | 0 | 0 | 1 | 0 | 0 | 0 | 0 | 0 | 0 | 0 | 0 | 1 | 2 | Bacteria | Bacteria\_none | Pseudomonadota | Gammaproteobacteria | Enterobacterales | Morganellaceae | Morganella | Bacteria;Bacteria\_none;Pseudomonadota;Gammaproteobacteria;Enterobacterales;Morganellaceae;Morganella;Morganella morganii |
| Proteus mirabilis | 1 | 0 | 1 | 0 | 0 | 0 | 0 | 4 | 0 | 0 | 0 | 1 | 0 | 7 | Bacteria | Bacteria\_none | Pseudomonadota | Gammaproteobacteria | Enterobacterales | Morganellaceae | Proteus | Bacteria;Bacteria\_none;Pseudomonadota;Gammaproteobacteria;Enterobacterales;Morganellaceae;Proteus;Proteus mirabilis |
| Proteus sp. NMG38-2 | 0 | 0 | 0 | 0 | 0 | 1 | 0 | 0 | 0 | 0 | 0 | 0 | 0 | 1 | Bacteria | Bacteria\_none | Pseudomonadota | Gammaproteobacteria | Enterobacterales | Morganellaceae | Proteus | Bacteria;Bacteria\_none;Pseudomonadota;Gammaproteobacteria;Enterobacterales;Morganellaceae;Proteus;Proteus sp. NMG38-2 |
| Providencia alcalifaciens | 0 | 0 | 0 | 0 | 1 | 1 | 0 | 0 | 0 | 0 | 0 | 0 | 0 | 2 | Bacteria | Bacteria\_none | Pseudomonadota | Gammaproteobacteria | Enterobacterales | Morganellaceae | Providencia | Bacteria;Bacteria\_none;Pseudomonadota;Gammaproteobacteria;Enterobacterales;Morganellaceae;Providencia;Providencia alcalifaciens |
| Providencia manganoxydans | 0 | 0 | 0 | 0 | 0 | 0 | 0 | 1 | 0 | 0 | 0 | 0 | 0 | 1 | Bacteria | Bacteria\_none | Pseudomonadota | Gammaproteobacteria | Enterobacterales | Morganellaceae | Providencia | Bacteria;Bacteria\_none;Pseudomonadota;Gammaproteobacteria;Enterobacterales;Morganellaceae;Providencia;Providencia manganoxydans |
| Xenorhabdus bovienii | 0 | 0 | 0 | 0 | 0 | 0 | 1 | 0 | 0 | 0 | 0 | 0 | 0 | 1 | Bacteria | Bacteria\_none | Pseudomonadota | Gammaproteobacteria | Enterobacterales | Morganellaceae | Xenorhabdus | Bacteria;Bacteria\_none;Pseudomonadota;Gammaproteobacteria;Enterobacterales;Morganellaceae;Xenorhabdus;Xenorhabdus bovienii |
| Xenorhabdus sp. SF857 | 0 | 0 | 0 | 0 | 0 | 0 | 0 | 0 | 0 | 0 | 0 | 0 | 1 | 1 | Bacteria | Bacteria\_none | Pseudomonadota | Gammaproteobacteria | Enterobacterales | Morganellaceae | Xenorhabdus | Bacteria;Bacteria\_none;Pseudomonadota;Gammaproteobacteria;Enterobacterales;Morganellaceae;Xenorhabdus;Xenorhabdus sp. SF857 |
| Dickeya dadantii | 0 | 0 | 0 | 0 | 1 | 0 | 0 | 0 | 0 | 0 | 0 | 0 | 0 | 1 | Bacteria | Bacteria\_none | Pseudomonadota | Gammaproteobacteria | Enterobacterales | Pectobacteriaceae | Dickeya | Bacteria;Bacteria\_none;Pseudomonadota;Gammaproteobacteria;Enterobacterales;Pectobacteriaceae;Dickeya;Dickeya dadantii |
| Serratia marcescens | 0 | 0 | 0 | 0 | 0 | 0 | 0 | 0 | 0 | 0 | 1 | 0 | 0 | 1 | Bacteria | Bacteria\_none | Pseudomonadota | Gammaproteobacteria | Enterobacterales | Yersiniaceae | Serratia | Bacteria;Bacteria\_none;Pseudomonadota;Gammaproteobacteria;Enterobacterales;Yersiniaceae;Serratia;Serratia marcescens |
| Legionella sp. PC1000 | 0 | 0 | 0 | 1 | 0 | 0 | 0 | 0 | 0 | 0 | 0 | 0 | 0 | 1 | Bacteria | Bacteria\_none | Pseudomonadota | Gammaproteobacteria | Legionellales | Legionellaceae | Legionella | Bacteria;Bacteria\_none;Pseudomonadota;Gammaproteobacteria;Legionellales;Legionellaceae;Legionella;Legionella sp. PC1000 |
| Legionella sp. PC997 | 0 | 0 | 0 | 0 | 0 | 0 | 0 | 0 | 0 | 0 | 0 | 0 | 1 | 1 | Bacteria | Bacteria\_none | Pseudomonadota | Gammaproteobacteria | Legionellales | Legionellaceae | Legionella | Bacteria;Bacteria\_none;Pseudomonadota;Gammaproteobacteria;Legionellales;Legionellaceae;Legionella;Legionella sp. PC997 |
| Lysobacter capsici | 0 | 0 | 0 | 1 | 0 | 0 | 0 | 0 | 0 | 0 | 0 | 0 | 0 | 1 | Bacteria | Bacteria\_none | Pseudomonadota | Gammaproteobacteria | Lysobacterales | Lysobacteraceae | Lysobacter | Bacteria;Bacteria\_none;Pseudomonadota;Gammaproteobacteria;Lysobacterales;Lysobacteraceae;Lysobacter;Lysobacter capsici |
| Pseudoxanthomonas daejeonensis | 0 | 0 | 0 | 0 | 0 | 0 | 0 | 0 | 0 | 0 | 1 | 0 | 0 | 1 | Bacteria | Bacteria\_none | Pseudomonadota | Gammaproteobacteria | Lysobacterales | Lysobacteraceae | Pseudoxanthomonas | Bacteria;Bacteria\_none;Pseudomonadota;Gammaproteobacteria;Lysobacterales;Lysobacteraceae;Pseudoxanthomonas;Pseudoxanthomonas daejeonensis |
| Stenotrophomonas maltophilia | 0 | 0 | 0 | 0 | 1 | 0 | 0 | 0 | 0 | 0 | 0 | 0 | 0 | 1 | Bacteria | Bacteria\_none | Pseudomonadota | Gammaproteobacteria | Lysobacterales | Lysobacteraceae | Stenotrophomonas | Bacteria;Bacteria\_none;Pseudomonadota;Gammaproteobacteria;Lysobacterales;Lysobacteraceae;Stenotrophomonas;Stenotrophomonas maltophilia |
| Stenotrophomonas sp. 57 | 0 | 0 | 0 | 0 | 1 | 0 | 0 | 0 | 0 | 0 | 0 | 0 | 0 | 1 | Bacteria | Bacteria\_none | Pseudomonadota | Gammaproteobacteria | Lysobacterales | Lysobacteraceae | Stenotrophomonas | Bacteria;Bacteria\_none;Pseudomonadota;Gammaproteobacteria;Lysobacterales;Lysobacteraceae;Stenotrophomonas;Stenotrophomonas sp. 57 |
| Xanthomonas sp. CFBP 8443 | 0 | 0 | 0 | 0 | 0 | 0 | 0 | 0 | 0 | 0 | 0 | 0 | 1 | 1 | Bacteria | Bacteria\_none | Pseudomonadota | Gammaproteobacteria | Lysobacterales | Lysobacteraceae | Xanthomonas | Bacteria;Bacteria\_none;Pseudomonadota;Gammaproteobacteria;Lysobacterales;Lysobacteraceae;Xanthomonas;Xanthomonas sp. CFBP 8443 |
| Xylella fastidiosa | 0 | 0 | 0 | 0 | 0 | 0 | 0 | 0 | 0 | 0 | 0 | 0 | 1 | 1 | Bacteria | Bacteria\_none | Pseudomonadota | Gammaproteobacteria | Lysobacterales | Lysobacteraceae | Xylella | Bacteria;Bacteria\_none;Pseudomonadota;Gammaproteobacteria;Lysobacterales;Lysobacteraceae;Xylella;Xylella fastidiosa |
| Dyella terrae | 0 | 0 | 0 | 0 | 0 | 0 | 0 | 0 | 0 | 0 | 0 | 1 | 0 | 1 | Bacteria | Bacteria\_none | Pseudomonadota | Gammaproteobacteria | Lysobacterales | Rhodanobacteraceae | Dyella | Bacteria;Bacteria\_none;Pseudomonadota;Gammaproteobacteria;Lysobacterales;Rhodanobacteraceae;Dyella;Dyella terrae |
| Tahibacter amnicola | 0 | 0 | 0 | 0 | 0 | 0 | 0 | 0 | 0 | 1 | 0 | 0 | 0 | 1 | Bacteria | Bacteria\_none | Pseudomonadota | Gammaproteobacteria | Lysobacterales | Rhodanobacteraceae | Tahibacter | Bacteria;Bacteria\_none;Pseudomonadota;Gammaproteobacteria;Lysobacterales;Rhodanobacteraceae;Tahibacter;Tahibacter amnicola |
| Candidatus Methylospira mobilis | 0 | 0 | 0 | 0 | 0 | 0 | 0 | 0 | 0 | 0 | 0 | 0 | 1 | 1 | Bacteria | Bacteria\_none | Pseudomonadota | Gammaproteobacteria | Methylococcales | Methylococcaceae | Candidatus Methylospira | Bacteria;Bacteria\_none;Pseudomonadota;Gammaproteobacteria;Methylococcales;Methylococcaceae;Candidatus Methylospira;Candidatus Methylospira mobilis |
| Methylobacter sp. S3L5C | 0 | 0 | 0 | 0 | 0 | 0 | 0 | 0 | 0 | 0 | 0 | 0 | 1 | 1 | Bacteria | Bacteria\_none | Pseudomonadota | Gammaproteobacteria | Methylococcales | Methylococcaceae | Methylobacter | Bacteria;Bacteria\_none;Pseudomonadota;Gammaproteobacteria;Methylococcales;Methylococcaceae;Methylobacter;Methylobacter sp. S3L5C |
| Methylobacter sp. YRD-M1 | 0 | 0 | 0 | 0 | 0 | 0 | 0 | 0 | 0 | 0 | 0 | 0 | 1 | 1 | Bacteria | Bacteria\_none | Pseudomonadota | Gammaproteobacteria | Methylococcales | Methylococcaceae | Methylobacter | Bacteria;Bacteria\_none;Pseudomonadota;Gammaproteobacteria;Methylococcales;Methylococcaceae;Methylobacter;Methylobacter sp. YRD-M1 |
| Methylomonas denitrificans | 0 | 0 | 0 | 0 | 0 | 1 | 0 | 0 | 0 | 0 | 0 | 0 | 0 | 1 | Bacteria | Bacteria\_none | Pseudomonadota | Gammaproteobacteria | Methylococcales | Methylococcaceae | Methylomonas | Bacteria;Bacteria\_none;Pseudomonadota;Gammaproteobacteria;Methylococcales;Methylococcaceae;Methylomonas;Methylomonas denitrificans |
| Methylotuvimicrobium alcaliphilum | 0 | 0 | 0 | 0 | 0 | 0 | 0 | 0 | 0 | 0 | 0 | 0 | 1 | 1 | Bacteria | Bacteria\_none | Pseudomonadota | Gammaproteobacteria | Methylococcales | Methylococcaceae | Methylotuvimicrobium | Bacteria;Bacteria\_none;Pseudomonadota;Gammaproteobacteria;Methylococcales;Methylococcaceae;Methylotuvimicrobium;Methylotuvimicrobium alcaliphilum |
| Acinetobacter baumannii | 0 | 0 | 0 | 0 | 0 | 0 | 0 | 0 | 0 | 1 | 0 | 0 | 1 | 2 | Bacteria | Bacteria\_none | Pseudomonadota | Gammaproteobacteria | Moraxellales | Moraxellaceae | Acinetobacter | Bacteria;Bacteria\_none;Pseudomonadota;Gammaproteobacteria;Moraxellales;Moraxellaceae;Acinetobacter;Acinetobacter baumannii |
| Acinetobacter chinensis | 0 | 0 | 0 | 0 | 0 | 0 | 0 | 0 | 0 | 0 | 0 | 0 | 1 | 1 | Bacteria | Bacteria\_none | Pseudomonadota | Gammaproteobacteria | Moraxellales | Moraxellaceae | Acinetobacter | Bacteria;Bacteria\_none;Pseudomonadota;Gammaproteobacteria;Moraxellales;Moraxellaceae;Acinetobacter;Acinetobacter chinensis |
| Acinetobacter colistiniresistens | 0 | 0 | 0 | 0 | 0 | 1 | 0 | 2 | 0 | 0 | 0 | 0 | 0 | 3 | Bacteria | Bacteria\_none | Pseudomonadota | Gammaproteobacteria | Moraxellales | Moraxellaceae | Acinetobacter | Bacteria;Bacteria\_none;Pseudomonadota;Gammaproteobacteria;Moraxellales;Moraxellaceae;Acinetobacter;Acinetobacter colistiniresistens |
| Acinetobacter guillouiae | 0 | 0 | 0 | 0 | 0 | 1 | 0 | 0 | 0 | 0 | 0 | 0 | 0 | 1 | Bacteria | Bacteria\_none | Pseudomonadota | Gammaproteobacteria | Moraxellales | Moraxellaceae | Acinetobacter | Bacteria;Bacteria\_none;Pseudomonadota;Gammaproteobacteria;Moraxellales;Moraxellaceae;Acinetobacter;Acinetobacter guillouiae |
| Acinetobacter pittii | 0 | 0 | 0 | 1 | 0 | 0 | 0 | 0 | 0 | 0 | 0 | 0 | 0 | 1 | Bacteria | Bacteria\_none | Pseudomonadota | Gammaproteobacteria | Moraxellales | Moraxellaceae | Acinetobacter | Bacteria;Bacteria\_none;Pseudomonadota;Gammaproteobacteria;Moraxellales;Moraxellaceae;Acinetobacter;Acinetobacter pittii |
| Acinetobacter sp. Z1 | 0 | 0 | 0 | 0 | 0 | 0 | 0 | 0 | 0 | 1 | 0 | 0 | 0 | 1 | Bacteria | Bacteria\_none | Pseudomonadota | Gammaproteobacteria | Moraxellales | Moraxellaceae | Acinetobacter | Bacteria;Bacteria\_none;Pseudomonadota;Gammaproteobacteria;Moraxellales;Moraxellaceae;Acinetobacter;Acinetobacter sp. Z1 |
| Moraxella catarrhalis | 0 | 0 | 0 | 1 | 0 | 0 | 0 | 0 | 0 | 0 | 0 | 0 | 2 | 3 | Bacteria | Bacteria\_none | Pseudomonadota | Gammaproteobacteria | Moraxellales | Moraxellaceae | Moraxella | Bacteria;Bacteria\_none;Pseudomonadota;Gammaproteobacteria;Moraxellales;Moraxellaceae;Moraxella;Moraxella catarrhalis |
| Moraxella ovis | 0 | 0 | 0 | 0 | 0 | 0 | 0 | 0 | 0 | 0 | 0 | 0 | 1 | 1 | Bacteria | Bacteria\_none | Pseudomonadota | Gammaproteobacteria | Moraxellales | Moraxellaceae | Moraxella | Bacteria;Bacteria\_none;Pseudomonadota;Gammaproteobacteria;Moraxellales;Moraxellaceae;Moraxella;Moraxella ovis |
| Psychrobacter raelei | 0 | 0 | 0 | 0 | 0 | 0 | 0 | 2 | 0 | 0 | 0 | 0 | 0 | 2 | Bacteria | Bacteria\_none | Pseudomonadota | Gammaproteobacteria | Moraxellales | Moraxellaceae | Psychrobacter | Bacteria;Bacteria\_none;Pseudomonadota;Gammaproteobacteria;Moraxellales;Moraxellaceae;Psychrobacter;Psychrobacter raelei |
| Psychrobacter sp. 28M-43 | 0 | 0 | 0 | 0 | 0 | 0 | 0 | 0 | 0 | 0 | 0 | 1 | 0 | 1 | Bacteria | Bacteria\_none | Pseudomonadota | Gammaproteobacteria | Moraxellales | Moraxellaceae | Psychrobacter | Bacteria;Bacteria\_none;Pseudomonadota;Gammaproteobacteria;Moraxellales;Moraxellaceae;Psychrobacter;Psychrobacter sp. 28M-43 |
| Psychrobacter sp. van23A | 0 | 0 | 1 | 0 | 0 | 0 | 0 | 0 | 0 | 0 | 0 | 1 | 0 | 2 | Bacteria | Bacteria\_none | Pseudomonadota | Gammaproteobacteria | Moraxellales | Moraxellaceae | Psychrobacter | Bacteria;Bacteria\_none;Pseudomonadota;Gammaproteobacteria;Moraxellales;Moraxellaceae;Psychrobacter;Psychrobacter sp. van23A |
| Alcanivorax sp. | 0 | 0 | 0 | 0 | 0 | 0 | 0 | 1 | 0 | 0 | 0 | 0 | 0 | 1 | Bacteria | Bacteria\_none | Pseudomonadota | Gammaproteobacteria | Oceanospirillales | Alcanivoracaceae | Alcanivorax | Bacteria;Bacteria\_none;Pseudomonadota;Gammaproteobacteria;Oceanospirillales;Alcanivoracaceae;Alcanivorax;Alcanivorax sp. |
| Halomonas meridiana | 0 | 0 | 0 | 0 | 0 | 0 | 0 | 1 | 0 | 0 | 0 | 0 | 0 | 1 | Bacteria | Bacteria\_none | Pseudomonadota | Gammaproteobacteria | Oceanospirillales | Halomonadaceae | Halomonas | Bacteria;Bacteria\_none;Pseudomonadota;Gammaproteobacteria;Oceanospirillales;Halomonadaceae;Halomonas;Halomonas meridiana |
| Halomonas sp. KO116 | 0 | 0 | 0 | 0 | 0 | 1 | 0 | 0 | 0 | 0 | 0 | 0 | 0 | 1 | Bacteria | Bacteria\_none | Pseudomonadota | Gammaproteobacteria | Oceanospirillales | Halomonadaceae | Halomonas | Bacteria;Bacteria\_none;Pseudomonadota;Gammaproteobacteria;Oceanospirillales;Halomonadaceae;Halomonas;Halomonas sp. KO116 |
| Halomonas sp. LR3S48 | 0 | 0 | 0 | 0 | 1 | 0 | 0 | 0 | 0 | 0 | 0 | 0 | 0 | 1 | Bacteria | Bacteria\_none | Pseudomonadota | Gammaproteobacteria | Oceanospirillales | Halomonadaceae | Halomonas | Bacteria;Bacteria\_none;Pseudomonadota;Gammaproteobacteria;Oceanospirillales;Halomonadaceae;Halomonas;Halomonas sp. LR3S48 |
| Amphritea\_uncultured | 0 | 0 | 0 | 0 | 0 | 0 | 0 | 1 | 0 | 0 | 0 | 0 | 0 | 1 | Bacteria | Bacteria\_none | Pseudomonadota | Gammaproteobacteria | Oceanospirillales | Oceanospirillaceae | Amphritea | Bacteria;Bacteria\_none;Pseudomonadota;Gammaproteobacteria;Oceanospirillales;Oceanospirillaceae;Amphritea;Amphritea\_uncultured |
| Marinomonas posidonica | 0 | 0 | 0 | 0 | 0 | 0 | 0 | 0 | 0 | 0 | 0 | 0 | 1 | 1 | Bacteria | Bacteria\_none | Pseudomonadota | Gammaproteobacteria | Oceanospirillales | Oceanospirillaceae | Marinomonas | Bacteria;Bacteria\_none;Pseudomonadota;Gammaproteobacteria;Oceanospirillales;Oceanospirillaceae;Marinomonas;Marinomonas posidonica |
| Marinomonas primoryensis | 0 | 0 | 0 | 0 | 0 | 1 | 0 | 0 | 0 | 0 | 0 | 0 | 0 | 1 | Bacteria | Bacteria\_none | Pseudomonadota | Gammaproteobacteria | Oceanospirillales | Oceanospirillaceae | Marinomonas | Bacteria;Bacteria\_none;Pseudomonadota;Gammaproteobacteria;Oceanospirillales;Oceanospirillaceae;Marinomonas;Marinomonas primoryensis |
| Marinomonas sp. A3A | 0 | 0 | 0 | 0 | 0 | 0 | 0 | 1 | 0 | 0 | 0 | 0 | 0 | 1 | Bacteria | Bacteria\_none | Pseudomonadota | Gammaproteobacteria | Oceanospirillales | Oceanospirillaceae | Marinomonas | Bacteria;Bacteria\_none;Pseudomonadota;Gammaproteobacteria;Oceanospirillales;Oceanospirillaceae;Marinomonas;Marinomonas sp. A3A |
| Thalassolituus hydrocarboniclasticus | 0 | 1 | 0 | 0 | 0 | 0 | 0 | 0 | 0 | 0 | 0 | 0 | 0 | 1 | Bacteria | Bacteria\_none | Pseudomonadota | Gammaproteobacteria | Oceanospirillales | Oceanospirillaceae | Thalassolituus | Bacteria;Bacteria\_none;Pseudomonadota;Gammaproteobacteria;Oceanospirillales;Oceanospirillaceae;Thalassolituus;Thalassolituus hydrocarboniclasticus |
| Gilliamella sp. ESL0443 | 0 | 0 | 0 | 0 | 0 | 0 | 0 | 1 | 0 | 0 | 0 | 0 | 0 | 1 | Bacteria | Bacteria\_none | Pseudomonadota | Gammaproteobacteria | Orbales | Orbaceae | Gilliamella | Bacteria;Bacteria\_none;Pseudomonadota;Gammaproteobacteria;Orbales;Orbaceae;Gilliamella;Gilliamella sp. ESL0443 |
| Gilliamella sp. wkB7 | 0 | 0 | 0 | 0 | 0 | 0 | 0 | 0 | 0 | 0 | 0 | 0 | 1 | 1 | Bacteria | Bacteria\_none | Pseudomonadota | Gammaproteobacteria | Orbales | Orbaceae | Gilliamella | Bacteria;Bacteria\_none;Pseudomonadota;Gammaproteobacteria;Orbales;Orbaceae;Gilliamella;Gilliamella sp. wkB7 |
| Actinobacillus arthritidis | 0 | 0 | 0 | 0 | 0 | 0 | 0 | 0 | 0 | 0 | 0 | 0 | 1 | 1 | Bacteria | Bacteria\_none | Pseudomonadota | Gammaproteobacteria | Pasteurellales | Pasteurellaceae | Actinobacillus | Bacteria;Bacteria\_none;Pseudomonadota;Gammaproteobacteria;Pasteurellales;Pasteurellaceae;Actinobacillus;Actinobacillus arthritidis |
| Actinobacillus equuli | 0 | 0 | 0 | 0 | 0 | 0 | 0 | 11 | 0 | 0 | 0 | 0 | 0 | 11 | Bacteria | Bacteria\_none | Pseudomonadota | Gammaproteobacteria | Pasteurellales | Pasteurellaceae | Actinobacillus | Bacteria;Bacteria\_none;Pseudomonadota;Gammaproteobacteria;Pasteurellales;Pasteurellaceae;Actinobacillus;Actinobacillus equuli |
| Actinobacillus genomosp. 1 | 0 | 0 | 0 | 0 | 0 | 0 | 0 | 3 | 0 | 0 | 0 | 0 | 0 | 3 | Bacteria | Bacteria\_none | Pseudomonadota | Gammaproteobacteria | Pasteurellales | Pasteurellaceae | Actinobacillus | Bacteria;Bacteria\_none;Pseudomonadota;Gammaproteobacteria;Pasteurellales;Pasteurellaceae;Actinobacillus;Actinobacillus genomosp. 1 |
| Actinobacillus genomosp. 2 | 0 | 0 | 0 | 0 | 1 | 0 | 0 | 1 | 0 | 0 | 0 | 0 | 2 | 4 | Bacteria | Bacteria\_none | Pseudomonadota | Gammaproteobacteria | Pasteurellales | Pasteurellaceae | Actinobacillus | Bacteria;Bacteria\_none;Pseudomonadota;Gammaproteobacteria;Pasteurellales;Pasteurellaceae;Actinobacillus;Actinobacillus genomosp. 2 |
| Actinobacillus pleuropneumoniae | 0 | 0 | 0 | 0 | 1 | 0 | 0 | 10 | 0 | 0 | 0 | 0 | 0 | 11 | Bacteria | Bacteria\_none | Pseudomonadota | Gammaproteobacteria | Pasteurellales | Pasteurellaceae | Actinobacillus | Bacteria;Bacteria\_none;Pseudomonadota;Gammaproteobacteria;Pasteurellales;Pasteurellaceae;Actinobacillus;Actinobacillus pleuropneumoniae |
| Actinobacillus porcitonsillarum | 0 | 0 | 0 | 0 | 0 | 0 | 0 | 11 | 0 | 0 | 0 | 0 | 0 | 11 | Bacteria | Bacteria\_none | Pseudomonadota | Gammaproteobacteria | Pasteurellales | Pasteurellaceae | Actinobacillus | Bacteria;Bacteria\_none;Pseudomonadota;Gammaproteobacteria;Pasteurellales;Pasteurellaceae;Actinobacillus;Actinobacillus porcitonsillarum |
| Actinobacillus suis | 0 | 0 | 1 | 0 | 0 | 0 | 0 | 0 | 0 | 0 | 0 | 0 | 1 | 2 | Bacteria | Bacteria\_none | Pseudomonadota | Gammaproteobacteria | Pasteurellales | Pasteurellaceae | Actinobacillus | Bacteria;Bacteria\_none;Pseudomonadota;Gammaproteobacteria;Pasteurellales;Pasteurellaceae;Actinobacillus;Actinobacillus suis |
| Aggregatibacter actinomycetemcomitans | 0 | 3 | 0 | 4 | 0 | 0 | 0 | 8 | 0 | 0 | 0 | 0 | 1 | 16 | Bacteria | Bacteria\_none | Pseudomonadota | Gammaproteobacteria | Pasteurellales | Pasteurellaceae | Aggregatibacter | Bacteria;Bacteria\_none;Pseudomonadota;Gammaproteobacteria;Pasteurellales;Pasteurellaceae;Aggregatibacter;Aggregatibacter actinomycetemcomitans |
| Aggregatibacter aphrophilus | 0 | 14 | 1 | 23 | 74 | 4 | 0 | 295 | 0 | 2 | 0 | 1 | 84 | 498 | Bacteria | Bacteria\_none | Pseudomonadota | Gammaproteobacteria | Pasteurellales | Pasteurellaceae | Aggregatibacter | Bacteria;Bacteria\_none;Pseudomonadota;Gammaproteobacteria;Pasteurellales;Pasteurellaceae;Aggregatibacter;Aggregatibacter aphrophilus |
| Aggregatibacter segnis | 0 | 6 | 1 | 53 | 1 | 5 | 0 | 5 | 0 | 4 | 0 | 0 | 22 | 97 | Bacteria | Bacteria\_none | Pseudomonadota | Gammaproteobacteria | Pasteurellales | Pasteurellaceae | Aggregatibacter | Bacteria;Bacteria\_none;Pseudomonadota;Gammaproteobacteria;Pasteurellales;Pasteurellaceae;Aggregatibacter;Aggregatibacter segnis |
| Aggregatibacter sp. 2125159857 | 0 | 3 | 2 | 27 | 7 | 13 | 0 | 2 | 0 | 5 | 0 | 7 | 12 | 78 | Bacteria | Bacteria\_none | Pseudomonadota | Gammaproteobacteria | Pasteurellales | Pasteurellaceae | Aggregatibacter | Bacteria;Bacteria\_none;Pseudomonadota;Gammaproteobacteria;Pasteurellales;Pasteurellaceae;Aggregatibacter;Aggregatibacter sp. 2125159857 |
| Aggregatibacter sp. Marseille-P9115 | 0 | 6 | 4 | 43 | 3 | 22 | 0 | 2 | 0 | 0 | 0 | 12 | 21 | 113 | Bacteria | Bacteria\_none | Pseudomonadota | Gammaproteobacteria | Pasteurellales | Pasteurellaceae | Aggregatibacter | Bacteria;Bacteria\_none;Pseudomonadota;Gammaproteobacteria;Pasteurellales;Pasteurellaceae;Aggregatibacter;Aggregatibacter sp. Marseille-P9115 |
| Aggregatibacter sp. oral taxon 513 | 0 | 4 | 4 | 43 | 3 | 5 | 0 | 8 | 0 | 0 | 0 | 0 | 27 | 94 | Bacteria | Bacteria\_none | Pseudomonadota | Gammaproteobacteria | Pasteurellales | Pasteurellaceae | Aggregatibacter | Bacteria;Bacteria\_none;Pseudomonadota;Gammaproteobacteria;Pasteurellales;Pasteurellaceae;Aggregatibacter;Aggregatibacter sp. oral taxon 513 |
| Avibacterium paragallinarum | 0 | 0 | 0 | 0 | 1 | 1 | 0 | 0 | 0 | 0 | 0 | 0 | 0 | 2 | Bacteria | Bacteria\_none | Pseudomonadota | Gammaproteobacteria | Pasteurellales | Pasteurellaceae | Avibacterium | Bacteria;Bacteria\_none;Pseudomonadota;Gammaproteobacteria;Pasteurellales;Pasteurellaceae;Avibacterium;Avibacterium paragallinarum |
| Avibacterium volantium | 0 | 0 | 0 | 0 | 0 | 0 | 0 | 2 | 0 | 0 | 0 | 0 | 0 | 2 | Bacteria | Bacteria\_none | Pseudomonadota | Gammaproteobacteria | Pasteurellales | Pasteurellaceae | Avibacterium | Bacteria;Bacteria\_none;Pseudomonadota;Gammaproteobacteria;Pasteurellales;Pasteurellaceae;Avibacterium;Avibacterium volantium |
| Bibersteinia trehalosi | 0 | 0 | 0 | 0 | 0 | 0 | 0 | 1 | 0 | 0 | 0 | 0 | 0 | 1 | Bacteria | Bacteria\_none | Pseudomonadota | Gammaproteobacteria | Pasteurellales | Pasteurellaceae | Bibersteinia | Bacteria;Bacteria\_none;Pseudomonadota;Gammaproteobacteria;Pasteurellales;Pasteurellaceae;Bibersteinia;Bibersteinia trehalosi |
| Bisgaardia hudsonensis | 0 | 0 | 0 | 0 | 0 | 0 | 0 | 1 | 0 | 0 | 0 | 0 | 0 | 1 | Bacteria | Bacteria\_none | Pseudomonadota | Gammaproteobacteria | Pasteurellales | Pasteurellaceae | Bisgaardia | Bacteria;Bacteria\_none;Pseudomonadota;Gammaproteobacteria;Pasteurellales;Pasteurellaceae;Bisgaardia;Bisgaardia hudsonensis |
| Frederiksenia canicola | 0 | 0 | 0 | 0 | 0 | 0 | 0 | 0 | 0 | 0 | 0 | 0 | 1 | 1 | Bacteria | Bacteria\_none | Pseudomonadota | Gammaproteobacteria | Pasteurellales | Pasteurellaceae | Frederiksenia | Bacteria;Bacteria\_none;Pseudomonadota;Gammaproteobacteria;Pasteurellales;Pasteurellaceae;Frederiksenia;Frederiksenia canicola |
| Gallibacterium anatis | 0 | 0 | 0 | 0 | 0 | 2 | 0 | 0 | 0 | 0 | 0 | 0 | 0 | 2 | Bacteria | Bacteria\_none | Pseudomonadota | Gammaproteobacteria | Pasteurellales | Pasteurellaceae | Gallibacterium | Bacteria;Bacteria\_none;Pseudomonadota;Gammaproteobacteria;Pasteurellales;Pasteurellaceae;Gallibacterium;Gallibacterium anatis |
| Gallibacterium salpingitidis | 0 | 0 | 0 | 0 | 0 | 0 | 0 | 1 | 0 | 0 | 0 | 0 | 0 | 1 | Bacteria | Bacteria\_none | Pseudomonadota | Gammaproteobacteria | Pasteurellales | Pasteurellaceae | Gallibacterium | Bacteria;Bacteria\_none;Pseudomonadota;Gammaproteobacteria;Pasteurellales;Pasteurellaceae;Gallibacterium;Gallibacterium salpingitidis |
| Glaesserella parasuis | 0 | 1 | 0 | 0 | 0 | 0 | 0 | 2 | 0 | 0 | 0 | 0 | 0 | 3 | Bacteria | Bacteria\_none | Pseudomonadota | Gammaproteobacteria | Pasteurellales | Pasteurellaceae | Glaesserella | Bacteria;Bacteria\_none;Pseudomonadota;Gammaproteobacteria;Pasteurellales;Pasteurellaceae;Glaesserella;Glaesserella parasuis |
| Glaesserella sp. 15-184 | 0 | 0 | 0 | 0 | 0 | 0 | 1 | 1 | 0 | 0 | 0 | 0 | 0 | 2 | Bacteria | Bacteria\_none | Pseudomonadota | Gammaproteobacteria | Pasteurellales | Pasteurellaceae | Glaesserella | Bacteria;Bacteria\_none;Pseudomonadota;Gammaproteobacteria;Pasteurellales;Pasteurellaceae;Glaesserella;Glaesserella sp. 15-184 |
| Haemophilus haemolyticus | 0 | 10 | 0 | 197 | 31 | 11 | 0 | 22 | 0 | 3 | 15 | 2 | 66 | 357 | Bacteria | Bacteria\_none | Pseudomonadota | Gammaproteobacteria | Pasteurellales | Pasteurellaceae | Haemophilus | Bacteria;Bacteria\_none;Pseudomonadota;Gammaproteobacteria;Pasteurellales;Pasteurellaceae;Haemophilus;Haemophilus haemolyticus |
| Haemophilus influenzae | 2 | 8 | 5 | 42 | 5 | 5 | 0 | 26 | 0 | 0 | 2 | 7 | 24 | 126 | Bacteria | Bacteria\_none | Pseudomonadota | Gammaproteobacteria | Pasteurellales | Pasteurellaceae | Haemophilus | Bacteria;Bacteria\_none;Pseudomonadota;Gammaproteobacteria;Pasteurellales;Pasteurellaceae;Haemophilus;Haemophilus influenzae |
| Haemophilus parahaemolyticus | 1 | 0 | 0 | 0 | 0 | 1 | 0 | 18 | 0 | 0 | 0 | 1 | 2 | 23 | Bacteria | Bacteria\_none | Pseudomonadota | Gammaproteobacteria | Pasteurellales | Pasteurellaceae | Haemophilus | Bacteria;Bacteria\_none;Pseudomonadota;Gammaproteobacteria;Pasteurellales;Pasteurellaceae;Haemophilus;Haemophilus parahaemolyticus |
| Haemophilus parainfluenzae | 68 | 85 | 447 | 326 | 146 | 170 | 6 | 1179 | 0 | 77 | 31 | 214 | 882 | 3631 | Bacteria | Bacteria\_none | Pseudomonadota | Gammaproteobacteria | Pasteurellales | Pasteurellaceae | Haemophilus | Bacteria;Bacteria\_none;Pseudomonadota;Gammaproteobacteria;Pasteurellales;Pasteurellaceae;Haemophilus;Haemophilus parainfluenzae |
| Haemophilus pittmaniae | 0 | 2 | 0 | 1 | 6 | 3 | 0 | 268 | 0 | 0 | 1 | 0 | 70 | 351 | Bacteria | Bacteria\_none | Pseudomonadota | Gammaproteobacteria | Pasteurellales | Pasteurellaceae | Haemophilus | Bacteria;Bacteria\_none;Pseudomonadota;Gammaproteobacteria;Pasteurellales;Pasteurellaceae;Haemophilus;Haemophilus pittmaniae |
| Haemophilus sp. oral taxon 036 | 0 | 0 | 0 | 13 | 4 | 0 | 0 | 5 | 0 | 0 | 0 | 0 | 11 | 33 | Bacteria | Bacteria\_none | Pseudomonadota | Gammaproteobacteria | Pasteurellales | Pasteurellaceae | Haemophilus | Bacteria;Bacteria\_none;Pseudomonadota;Gammaproteobacteria;Pasteurellales;Pasteurellaceae;Haemophilus;Haemophilus sp. oral taxon 036 |
| [Haemophilus] ducreyi | 0 | 0 | 0 | 0 | 1 | 1 | 0 | 3 | 0 | 0 | 0 | 0 | 0 | 5 | Bacteria | Bacteria\_none | Pseudomonadota | Gammaproteobacteria | Pasteurellales | Pasteurellaceae | Haemophilus | Bacteria;Bacteria\_none;Pseudomonadota;Gammaproteobacteria;Pasteurellales;Pasteurellaceae;Haemophilus;[Haemophilus] ducreyi |
| Histophilus somni | 0 | 0 | 0 | 0 | 0 | 0 | 0 | 1 | 0 | 0 | 0 | 0 | 0 | 1 | Bacteria | Bacteria\_none | Pseudomonadota | Gammaproteobacteria | Pasteurellales | Pasteurellaceae | Histophilus | Bacteria;Bacteria\_none;Pseudomonadota;Gammaproteobacteria;Pasteurellales;Pasteurellaceae;Histophilus;Histophilus somni |
| Mannheimia bovis | 0 | 0 | 0 | 0 | 0 | 0 | 0 | 2 | 0 | 0 | 0 | 0 | 0 | 2 | Bacteria | Bacteria\_none | Pseudomonadota | Gammaproteobacteria | Pasteurellales | Pasteurellaceae | Mannheimia | Bacteria;Bacteria\_none;Pseudomonadota;Gammaproteobacteria;Pasteurellales;Pasteurellaceae;Mannheimia;Mannheimia bovis |
| Mannheimia granulomatis | 0 | 0 | 0 | 0 | 1 | 0 | 0 | 6 | 0 | 0 | 0 | 0 | 1 | 8 | Bacteria | Bacteria\_none | Pseudomonadota | Gammaproteobacteria | Pasteurellales | Pasteurellaceae | Mannheimia | Bacteria;Bacteria\_none;Pseudomonadota;Gammaproteobacteria;Pasteurellales;Pasteurellaceae;Mannheimia;Mannheimia granulomatis |
| Mannheimia haemolytica | 0 | 0 | 0 | 0 | 0 | 0 | 0 | 3 | 0 | 0 | 0 | 0 | 3 | 6 | Bacteria | Bacteria\_none | Pseudomonadota | Gammaproteobacteria | Pasteurellales | Pasteurellaceae | Mannheimia | Bacteria;Bacteria\_none;Pseudomonadota;Gammaproteobacteria;Pasteurellales;Pasteurellaceae;Mannheimia;Mannheimia haemolytica |
| Mannheimia pernigra | 0 | 0 | 0 | 0 | 0 | 0 | 0 | 5 | 0 | 0 | 0 | 0 | 0 | 5 | Bacteria | Bacteria\_none | Pseudomonadota | Gammaproteobacteria | Pasteurellales | Pasteurellaceae | Mannheimia | Bacteria;Bacteria\_none;Pseudomonadota;Gammaproteobacteria;Pasteurellales;Pasteurellaceae;Mannheimia;Mannheimia pernigra |
| Mannheimia sp. USDA-ARS-USMARC-1261 | 0 | 0 | 0 | 0 | 0 | 0 | 0 | 3 | 0 | 0 | 0 | 0 | 0 | 3 | Bacteria | Bacteria\_none | Pseudomonadota | Gammaproteobacteria | Pasteurellales | Pasteurellaceae | Mannheimia | Bacteria;Bacteria\_none;Pseudomonadota;Gammaproteobacteria;Pasteurellales;Pasteurellaceae;Mannheimia;Mannheimia sp. USDA-ARS-USMARC-1261 |
| Mannheimia varigena | 0 | 0 | 0 | 0 | 0 | 0 | 0 | 1 | 0 | 0 | 0 | 0 | 0 | 1 | Bacteria | Bacteria\_none | Pseudomonadota | Gammaproteobacteria | Pasteurellales | Pasteurellaceae | Mannheimia | Bacteria;Bacteria\_none;Pseudomonadota;Gammaproteobacteria;Pasteurellales;Pasteurellaceae;Mannheimia;Mannheimia varigena |
| Mergibacter septicus | 0 | 0 | 0 | 0 | 0 | 0 | 0 | 1 | 0 | 0 | 0 | 0 | 1 | 2 | Bacteria | Bacteria\_none | Pseudomonadota | Gammaproteobacteria | Pasteurellales | Pasteurellaceae | Mergibacter | Bacteria;Bacteria\_none;Pseudomonadota;Gammaproteobacteria;Pasteurellales;Pasteurellaceae;Mergibacter;Mergibacter septicus |
| Otariodibacter oris | 0 | 0 | 0 | 0 | 0 | 0 | 0 | 1 | 0 | 0 | 0 | 0 | 2 | 3 | Bacteria | Bacteria\_none | Pseudomonadota | Gammaproteobacteria | Pasteurellales | Pasteurellaceae | Otariodibacter | Bacteria;Bacteria\_none;Pseudomonadota;Gammaproteobacteria;Pasteurellales;Pasteurellaceae;Otariodibacter;Otariodibacter oris |
| Pasteurella atlantica | 0 | 0 | 0 | 0 | 0 | 0 | 0 | 1 | 0 | 0 | 0 | 0 | 1 | 2 | Bacteria | Bacteria\_none | Pseudomonadota | Gammaproteobacteria | Pasteurellales | Pasteurellaceae | Pasteurella | Bacteria;Bacteria\_none;Pseudomonadota;Gammaproteobacteria;Pasteurellales;Pasteurellaceae;Pasteurella;Pasteurella atlantica |
| Pasteurella canis | 0 | 0 | 0 | 1 | 0 | 0 | 0 | 3 | 0 | 0 | 0 | 0 | 0 | 4 | Bacteria | Bacteria\_none | Pseudomonadota | Gammaproteobacteria | Pasteurellales | Pasteurellaceae | Pasteurella | Bacteria;Bacteria\_none;Pseudomonadota;Gammaproteobacteria;Pasteurellales;Pasteurellaceae;Pasteurella;Pasteurella canis |
| Pasteurella dagmatis | 0 | 0 | 0 | 0 | 0 | 0 | 0 | 1 | 0 | 0 | 0 | 0 | 0 | 1 | Bacteria | Bacteria\_none | Pseudomonadota | Gammaproteobacteria | Pasteurellales | Pasteurellaceae | Pasteurella | Bacteria;Bacteria\_none;Pseudomonadota;Gammaproteobacteria;Pasteurellales;Pasteurellaceae;Pasteurella;Pasteurella dagmatis |
| Pasteurella multocida | 0 | 0 | 1 | 0 | 0 | 1 | 1 | 5 | 0 | 1 | 0 | 0 | 1 | 10 | Bacteria | Bacteria\_none | Pseudomonadota | Gammaproteobacteria | Pasteurellales | Pasteurellaceae | Pasteurella | Bacteria;Bacteria\_none;Pseudomonadota;Gammaproteobacteria;Pasteurellales;Pasteurellaceae;Pasteurella;Pasteurella multocida |
| Pasteurella skyensis | 0 | 0 | 0 | 0 | 0 | 0 | 0 | 0 | 0 | 0 | 0 | 0 | 1 | 1 | Bacteria | Bacteria\_none | Pseudomonadota | Gammaproteobacteria | Pasteurellales | Pasteurellaceae | Pasteurella | Bacteria;Bacteria\_none;Pseudomonadota;Gammaproteobacteria;Pasteurellales;Pasteurellaceae;Pasteurella;Pasteurella skyensis |
| Rodentibacter caecimuris | 0 | 0 | 0 | 0 | 0 | 0 | 0 | 3 | 0 | 0 | 0 | 0 | 0 | 3 | Bacteria | Bacteria\_none | Pseudomonadota | Gammaproteobacteria | Pasteurellales | Pasteurellaceae | Rodentibacter | Bacteria;Bacteria\_none;Pseudomonadota;Gammaproteobacteria;Pasteurellales;Pasteurellaceae;Rodentibacter;Rodentibacter caecimuris |
| Vespertiliibacter pulmonis | 0 | 0 | 0 | 0 | 0 | 0 | 0 | 1 | 0 | 0 | 0 | 0 | 0 | 1 | Bacteria | Bacteria\_none | Pseudomonadota | Gammaproteobacteria | Pasteurellales | Pasteurellaceae | Vespertiliibacter | Bacteria;Bacteria\_none;Pseudomonadota;Gammaproteobacteria;Pasteurellales;Pasteurellaceae;Vespertiliibacter;Vespertiliibacter pulmonis |
| Pseudomonas agarici | 0 | 0 | 0 | 0 | 0 | 1 | 0 | 0 | 0 | 0 | 0 | 0 | 0 | 1 | Bacteria | Bacteria\_none | Pseudomonadota | Gammaproteobacteria | Pseudomonadales | Pseudomonadaceae | Pseudomonas | Bacteria;Bacteria\_none;Pseudomonadota;Gammaproteobacteria;Pseudomonadales;Pseudomonadaceae;Pseudomonas;Pseudomonas agarici |
| Pseudomonas asturiensis | 0 | 0 | 0 | 0 | 0 | 1 | 0 | 0 | 0 | 0 | 0 | 0 | 0 | 1 | Bacteria | Bacteria\_none | Pseudomonadota | Gammaproteobacteria | Pseudomonadales | Pseudomonadaceae | Pseudomonas | Bacteria;Bacteria\_none;Pseudomonadota;Gammaproteobacteria;Pseudomonadales;Pseudomonadaceae;Pseudomonas;Pseudomonas asturiensis |
| Pseudomonas canadensis | 0 | 0 | 0 | 0 | 0 | 1 | 0 | 0 | 0 | 0 | 0 | 0 | 0 | 1 | Bacteria | Bacteria\_none | Pseudomonadota | Gammaproteobacteria | Pseudomonadales | Pseudomonadaceae | Pseudomonas | Bacteria;Bacteria\_none;Pseudomonadota;Gammaproteobacteria;Pseudomonadales;Pseudomonadaceae;Pseudomonas;Pseudomonas canadensis |
| Pseudomonas citronellolis | 0 | 3 | 0 | 0 | 0 | 0 | 0 | 0 | 0 | 0 | 0 | 0 | 0 | 3 | Bacteria | Bacteria\_none | Pseudomonadota | Gammaproteobacteria | Pseudomonadales | Pseudomonadaceae | Pseudomonas | Bacteria;Bacteria\_none;Pseudomonadota;Gammaproteobacteria;Pseudomonadales;Pseudomonadaceae;Pseudomonas;Pseudomonas citronellolis |
| Pseudomonas frederiksbergensis | 0 | 0 | 0 | 0 | 0 | 0 | 0 | 1 | 0 | 0 | 0 | 0 | 0 | 1 | Bacteria | Bacteria\_none | Pseudomonadota | Gammaproteobacteria | Pseudomonadales | Pseudomonadaceae | Pseudomonas | Bacteria;Bacteria\_none;Pseudomonadota;Gammaproteobacteria;Pseudomonadales;Pseudomonadaceae;Pseudomonas;Pseudomonas frederiksbergensis |
| Pseudomonas guguanensis | 0 | 0 | 0 | 0 | 0 | 0 | 0 | 0 | 0 | 0 | 0 | 0 | 4 | 4 | Bacteria | Bacteria\_none | Pseudomonadota | Gammaproteobacteria | Pseudomonadales | Pseudomonadaceae | Pseudomonas | Bacteria;Bacteria\_none;Pseudomonadota;Gammaproteobacteria;Pseudomonadales;Pseudomonadaceae;Pseudomonas;Pseudomonas guguanensis |
| Pseudomonas hydrolytica | 0 | 0 | 0 | 0 | 0 | 0 | 0 | 0 | 0 | 0 | 1 | 0 | 0 | 1 | Bacteria | Bacteria\_none | Pseudomonadota | Gammaproteobacteria | Pseudomonadales | Pseudomonadaceae | Pseudomonas | Bacteria;Bacteria\_none;Pseudomonadota;Gammaproteobacteria;Pseudomonadales;Pseudomonadaceae;Pseudomonas;Pseudomonas hydrolytica |
| Pseudomonas psychrophila | 0 | 0 | 0 | 0 | 0 | 0 | 0 | 0 | 0 | 0 | 1 | 0 | 0 | 1 | Bacteria | Bacteria\_none | Pseudomonadota | Gammaproteobacteria | Pseudomonadales | Pseudomonadaceae | Pseudomonas | Bacteria;Bacteria\_none;Pseudomonadota;Gammaproteobacteria;Pseudomonadales;Pseudomonadaceae;Pseudomonas;Pseudomonas psychrophila |
| Pseudomonas putida | 0 | 0 | 0 | 0 | 0 | 0 | 1 | 0 | 0 | 0 | 0 | 0 | 0 | 1 | Bacteria | Bacteria\_none | Pseudomonadota | Gammaproteobacteria | Pseudomonadales | Pseudomonadaceae | Pseudomonas | Bacteria;Bacteria\_none;Pseudomonadota;Gammaproteobacteria;Pseudomonadales;Pseudomonadaceae;Pseudomonas;Pseudomonas putida |
| Pseudomonas triclosanedens | 0 | 0 | 0 | 0 | 2 | 0 | 0 | 0 | 0 | 0 | 0 | 0 | 0 | 2 | Bacteria | Bacteria\_none | Pseudomonadota | Gammaproteobacteria | Pseudomonadales | Pseudomonadaceae | Pseudomonas | Bacteria;Bacteria\_none;Pseudomonadota;Gammaproteobacteria;Pseudomonadales;Pseudomonadaceae;Pseudomonas;Pseudomonas triclosanedens |
| Thiomicrorhabdus indica | 0 | 0 | 0 | 0 | 1 | 0 | 0 | 0 | 0 | 0 | 0 | 0 | 0 | 1 | Bacteria | Bacteria\_none | Pseudomonadota | Gammaproteobacteria | Thiotrichales | Piscirickettsiaceae | Thiomicrorhabdus | Bacteria;Bacteria\_none;Pseudomonadota;Gammaproteobacteria;Thiotrichales;Piscirickettsiaceae;Thiomicrorhabdus;Thiomicrorhabdus indica |
| Aliivibrio salmonicida | 0 | 0 | 0 | 0 | 0 | 0 | 0 | 0 | 0 | 0 | 0 | 0 | 1 | 1 | Bacteria | Bacteria\_none | Pseudomonadota | Gammaproteobacteria | Vibrionales | Vibrionaceae | Aliivibrio | Bacteria;Bacteria\_none;Pseudomonadota;Gammaproteobacteria;Vibrionales;Vibrionaceae;Aliivibrio;Aliivibrio salmonicida |
| Candidatus Enterovibrio luxaltus | 0 | 0 | 0 | 0 | 0 | 0 | 0 | 1 | 0 | 0 | 0 | 0 | 0 | 1 | Bacteria | Bacteria\_none | Pseudomonadota | Gammaproteobacteria | Vibrionales | Vibrionaceae | Enterovibrio | Bacteria;Bacteria\_none;Pseudomonadota;Gammaproteobacteria;Vibrionales;Vibrionaceae;Enterovibrio;Candidatus Enterovibrio luxaltus |
| Thaumasiovibrio subtropicus | 0 | 0 | 0 | 0 | 0 | 0 | 0 | 1 | 0 | 0 | 0 | 0 | 0 | 1 | Bacteria | Bacteria\_none | Pseudomonadota | Gammaproteobacteria | Vibrionales | Vibrionaceae | Thaumasiovibrio | Bacteria;Bacteria\_none;Pseudomonadota;Gammaproteobacteria;Vibrionales;Vibrionaceae;Thaumasiovibrio;Thaumasiovibrio subtropicus |
| Vibrio artabrorum | 0 | 0 | 1 | 0 | 0 | 0 | 0 | 0 | 0 | 0 | 0 | 0 | 0 | 1 | Bacteria | Bacteria\_none | Pseudomonadota | Gammaproteobacteria | Vibrionales | Vibrionaceae | Vibrio | Bacteria;Bacteria\_none;Pseudomonadota;Gammaproteobacteria;Vibrionales;Vibrionaceae;Vibrio;Vibrio artabrorum |
| Vibrio campbellii | 0 | 0 | 0 | 0 | 0 | 0 | 0 | 1 | 0 | 0 | 0 | 0 | 0 | 1 | Bacteria | Bacteria\_none | Pseudomonadota | Gammaproteobacteria | Vibrionales | Vibrionaceae | Vibrio | Bacteria;Bacteria\_none;Pseudomonadota;Gammaproteobacteria;Vibrionales;Vibrionaceae;Vibrio;Vibrio campbellii |
| Vibrio cholerae | 0 | 0 | 2 | 0 | 0 | 0 | 0 | 0 | 0 | 0 | 0 | 0 | 0 | 2 | Bacteria | Bacteria\_none | Pseudomonadota | Gammaproteobacteria | Vibrionales | Vibrionaceae | Vibrio | Bacteria;Bacteria\_none;Pseudomonadota;Gammaproteobacteria;Vibrionales;Vibrionaceae;Vibrio;Vibrio cholerae |
| Vibrio crassostreae | 0 | 0 | 0 | 0 | 0 | 0 | 0 | 0 | 0 | 0 | 0 | 0 | 2 | 2 | Bacteria | Bacteria\_none | Pseudomonadota | Gammaproteobacteria | Vibrionales | Vibrionaceae | Vibrio | Bacteria;Bacteria\_none;Pseudomonadota;Gammaproteobacteria;Vibrionales;Vibrionaceae;Vibrio;Vibrio crassostreae |
| Vibrio gangliei | 0 | 0 | 0 | 0 | 0 | 1 | 0 | 0 | 0 | 0 | 0 | 0 | 0 | 1 | Bacteria | Bacteria\_none | Pseudomonadota | Gammaproteobacteria | Vibrionales | Vibrionaceae | Vibrio | Bacteria;Bacteria\_none;Pseudomonadota;Gammaproteobacteria;Vibrionales;Vibrionaceae;Vibrio;Vibrio gangliei |
| Vibrio gazogenes | 0 | 0 | 0 | 0 | 0 | 0 | 0 | 0 | 0 | 0 | 0 | 0 | 1 | 1 | Bacteria | Bacteria\_none | Pseudomonadota | Gammaproteobacteria | Vibrionales | Vibrionaceae | Vibrio | Bacteria;Bacteria\_none;Pseudomonadota;Gammaproteobacteria;Vibrionales;Vibrionaceae;Vibrio;Vibrio gazogenes |
| Vibrio rumoiensis | 0 | 0 | 0 | 0 | 0 | 0 | 0 | 1 | 0 | 0 | 0 | 0 | 0 | 1 | Bacteria | Bacteria\_none | Pseudomonadota | Gammaproteobacteria | Vibrionales | Vibrionaceae | Vibrio | Bacteria;Bacteria\_none;Pseudomonadota;Gammaproteobacteria;Vibrionales;Vibrionaceae;Vibrio;Vibrio rumoiensis |
| Vibrio sp. VB16 | 0 | 0 | 0 | 0 | 0 | 0 | 0 | 1 | 0 | 0 | 0 | 0 | 0 | 1 | Bacteria | Bacteria\_none | Pseudomonadota | Gammaproteobacteria | Vibrionales | Vibrionaceae | Vibrio | Bacteria;Bacteria\_none;Pseudomonadota;Gammaproteobacteria;Vibrionales;Vibrionaceae;Vibrio;Vibrio sp. VB16 |
| Vibrio zhugei | 0 | 0 | 0 | 0 | 2 | 0 | 0 | 0 | 0 | 0 | 0 | 0 | 0 | 2 | Bacteria | Bacteria\_none | Pseudomonadota | Gammaproteobacteria | Vibrionales | Vibrionaceae | Vibrio | Bacteria;Bacteria\_none;Pseudomonadota;Gammaproteobacteria;Vibrionales;Vibrionaceae;Vibrio;Vibrio zhugei |
| Leptospira santarosai | 1 | 0 | 0 | 0 | 0 | 0 | 0 | 0 | 0 | 0 | 0 | 1 | 0 | 2 | Bacteria | Bacteria\_none | Spirochaetota | Spirochaetia | Leptospirales | Leptospiraceae | Leptospira | Bacteria;Bacteria\_none;Spirochaetota;Spirochaetia;Leptospirales;Leptospiraceae;Leptospira;Leptospira santarosai |
| Borreliella garinii | 0 | 0 | 0 | 0 | 0 | 1 | 0 | 0 | 0 | 0 | 0 | 0 | 0 | 1 | Bacteria | Bacteria\_none | Spirochaetota | Spirochaetia | Spirochaetales | Borreliaceae | Borreliella | Bacteria;Bacteria\_none;Spirochaetota;Spirochaetia;Spirochaetales;Borreliaceae;Borreliella;Borreliella garinii |
| Breznakiella homolactica | 0 | 1 | 0 | 0 | 0 | 0 | 0 | 0 | 0 | 0 | 0 | 0 | 0 | 1 | Bacteria | Bacteria\_none | Spirochaetota | Spirochaetia | Spirochaetales | Breznakiellaceae | Breznakiella | Bacteria;Bacteria\_none;Spirochaetota;Spirochaetia;Spirochaetales;Breznakiellaceae;Breznakiella;Breznakiella homolactica |
| Sphaerochaeta\_uncultured | 0 | 1 | 0 | 0 | 0 | 0 | 0 | 0 | 0 | 0 | 0 | 0 | 0 | 1 | Bacteria | Bacteria\_none | Spirochaetota | Spirochaetia | Spirochaetales | Sphaerochaetaceae | Sphaerochaeta | Bacteria;Bacteria\_none;Spirochaetota;Spirochaetia;Spirochaetales;Sphaerochaetaceae;Sphaerochaeta;Sphaerochaeta\_uncultured |
| Treponema bryantii | 0 | 1 | 0 | 0 | 0 | 0 | 0 | 0 | 0 | 0 | 0 | 0 | 1 | 2 | Bacteria | Bacteria\_none | Spirochaetota | Spirochaetia | Spirochaetales | Treponemataceae | Treponema | Bacteria;Bacteria\_none;Spirochaetota;Spirochaetia;Spirochaetales;Treponemataceae;Treponema;Treponema bryantii |
| Treponema denticola | 0 | 16 | 41 | 1 | 13 | 30 | 3 | 62 | 0 | 6 | 28 | 37 | 78 | 315 | Bacteria | Bacteria\_none | Spirochaetota | Spirochaetia | Spirochaetales | Treponemataceae | Treponema | Bacteria;Bacteria\_none;Spirochaetota;Spirochaetia;Spirochaetales;Treponemataceae;Treponema;Treponema denticola |
| Treponema medium | 0 | 3 | 1 | 2 | 3 | 5 | 0 | 6 | 0 | 0 | 2 | 11 | 3 | 36 | Bacteria | Bacteria\_none | Spirochaetota | Spirochaetia | Spirochaetales | Treponemataceae | Treponema | Bacteria;Bacteria\_none;Spirochaetota;Spirochaetia;Spirochaetales;Treponemataceae;Treponema;Treponema medium |
| Treponema parvum | 0 | 1 | 1 | 0 | 0 | 1 | 0 | 1 | 0 | 0 | 0 | 3 | 2 | 9 | Bacteria | Bacteria\_none | Spirochaetota | Spirochaetia | Spirochaetales | Treponemataceae | Treponema | Bacteria;Bacteria\_none;Spirochaetota;Spirochaetia;Spirochaetales;Treponemataceae;Treponema;Treponema parvum |
| Treponema pedis | 0 | 1 | 0 | 2 | 1 | 1 | 1 | 0 | 0 | 1 | 3 | 0 | 3 | 13 | Bacteria | Bacteria\_none | Spirochaetota | Spirochaetia | Spirochaetales | Treponemataceae | Treponema | Bacteria;Bacteria\_none;Spirochaetota;Spirochaetia;Spirochaetales;Treponemataceae;Treponema;Treponema pedis |
| Treponema phagedenis | 0 | 0 | 0 | 0 | 0 | 1 | 0 | 0 | 0 | 0 | 1 | 0 | 0 | 2 | Bacteria | Bacteria\_none | Spirochaetota | Spirochaetia | Spirochaetales | Treponemataceae | Treponema | Bacteria;Bacteria\_none;Spirochaetota;Spirochaetia;Spirochaetales;Treponemataceae;Treponema;Treponema phagedenis |
| Treponema putidum | 0 | 2 | 1 | 0 | 0 | 1 | 0 | 1 | 0 | 0 | 0 | 1 | 5 | 11 | Bacteria | Bacteria\_none | Spirochaetota | Spirochaetia | Spirochaetales | Treponemataceae | Treponema | Bacteria;Bacteria\_none;Spirochaetota;Spirochaetia;Spirochaetales;Treponemataceae;Treponema;Treponema putidum |
| Treponema ruminis | 0 | 1 | 0 | 0 | 0 | 0 | 0 | 0 | 0 | 0 | 0 | 0 | 1 | 2 | Bacteria | Bacteria\_none | Spirochaetota | Spirochaetia | Spirochaetales | Treponemataceae | Treponema | Bacteria;Bacteria\_none;Spirochaetota;Spirochaetia;Spirochaetales;Treponemataceae;Treponema;Treponema ruminis |
| Treponema saccharophilum | 0 | 0 | 0 | 0 | 1 | 0 | 0 | 0 | 0 | 0 | 0 | 0 | 0 | 1 | Bacteria | Bacteria\_none | Spirochaetota | Spirochaetia | Spirochaetales | Treponemataceae | Treponema | Bacteria;Bacteria\_none;Spirochaetota;Spirochaetia;Spirochaetales;Treponemataceae;Treponema;Treponema saccharophilum |
| Treponema socranskii | 1 | 4 | 3 | 0 | 1 | 0 | 0 | 5 | 0 | 1 | 7 | 5 | 3 | 30 | Bacteria | Bacteria\_none | Spirochaetota | Spirochaetia | Spirochaetales | Treponemataceae | Treponema | Bacteria;Bacteria\_none;Spirochaetota;Spirochaetia;Spirochaetales;Treponemataceae;Treponema;Treponema socranskii |
| Treponema sp. B152 | 0 | 0 | 0 | 0 | 0 | 0 | 0 | 0 | 0 | 0 | 1 | 2 | 0 | 3 | Bacteria | Bacteria\_none | Spirochaetota | Spirochaetia | Spirochaetales | Treponemataceae | Treponema | Bacteria;Bacteria\_none;Spirochaetota;Spirochaetia;Spirochaetales;Treponemataceae;Treponema;Treponema sp. B152 |
| Treponema sp. Marseille-Q4132 | 0 | 1 | 0 | 0 | 2 | 1 | 0 | 1 | 0 | 0 | 1 | 4 | 0 | 10 | Bacteria | Bacteria\_none | Spirochaetota | Spirochaetia | Spirochaetales | Treponemataceae | Treponema | Bacteria;Bacteria\_none;Spirochaetota;Spirochaetia;Spirochaetales;Treponemataceae;Treponema;Treponema sp. Marseille-Q4132 |
| Treponema sp. OMZ 305 | 0 | 3 | 0 | 0 | 0 | 5 | 0 | 4 | 0 | 0 | 0 | 3 | 5 | 20 | Bacteria | Bacteria\_none | Spirochaetota | Spirochaetia | Spirochaetales | Treponemataceae | Treponema | Bacteria;Bacteria\_none;Spirochaetota;Spirochaetia;Spirochaetales;Treponemataceae;Treponema;Treponema sp. OMZ 305 |
| Treponema sp. OMZ 787 | 0 | 0 | 0 | 1 | 0 | 0 | 0 | 0 | 0 | 0 | 0 | 0 | 1 | 2 | Bacteria | Bacteria\_none | Spirochaetota | Spirochaetia | Spirochaetales | Treponemataceae | Treponema | Bacteria;Bacteria\_none;Spirochaetota;Spirochaetia;Spirochaetales;Treponemataceae;Treponema;Treponema sp. OMZ 787 |
| Treponema sp. OMZ 788 | 0 | 1 | 0 | 0 | 0 | 1 | 0 | 0 | 0 | 0 | 0 | 3 | 1 | 6 | Bacteria | Bacteria\_none | Spirochaetota | Spirochaetia | Spirochaetales | Treponemataceae | Treponema | Bacteria;Bacteria\_none;Spirochaetota;Spirochaetia;Spirochaetales;Treponemataceae;Treponema;Treponema sp. OMZ 788 |
| Treponema sp. OMZ 799 | 0 | 0 | 0 | 0 | 0 | 2 | 0 | 0 | 0 | 0 | 0 | 0 | 0 | 2 | Bacteria | Bacteria\_none | Spirochaetota | Spirochaetia | Spirochaetales | Treponemataceae | Treponema | Bacteria;Bacteria\_none;Spirochaetota;Spirochaetia;Spirochaetales;Treponemataceae;Treponema;Treponema sp. OMZ 799 |
| Treponema sp. OMZ 803 | 0 | 1 | 0 | 2 | 1 | 0 | 0 | 0 | 0 | 0 | 4 | 12 | 10 | 30 | Bacteria | Bacteria\_none | Spirochaetota | Spirochaetia | Spirochaetales | Treponemataceae | Treponema | Bacteria;Bacteria\_none;Spirochaetota;Spirochaetia;Spirochaetales;Treponemataceae;Treponema;Treponema sp. OMZ 803 |
| Treponema sp. OMZ 838 | 0 | 0 | 0 | 0 | 2 | 0 | 0 | 3 | 0 | 0 | 1 | 6 | 2 | 14 | Bacteria | Bacteria\_none | Spirochaetota | Spirochaetia | Spirochaetales | Treponemataceae | Treponema | Bacteria;Bacteria\_none;Spirochaetota;Spirochaetia;Spirochaetales;Treponemataceae;Treponema;Treponema sp. OMZ 838 |
| Treponema sp. OMZ 855 | 0 | 2 | 0 | 0 | 0 | 2 | 0 | 3 | 0 | 0 | 1 | 2 | 0 | 10 | Bacteria | Bacteria\_none | Spirochaetota | Spirochaetia | Spirochaetales | Treponemataceae | Treponema | Bacteria;Bacteria\_none;Spirochaetota;Spirochaetia;Spirochaetales;Treponemataceae;Treponema;Treponema sp. OMZ 855 |
| Treponema sp. OMZ 857 | 0 | 11 | 3 | 0 | 4 | 6 | 0 | 7 | 0 | 2 | 1 | 5 | 4 | 43 | Bacteria | Bacteria\_none | Spirochaetota | Spirochaetia | Spirochaetales | Treponemataceae | Treponema | Bacteria;Bacteria\_none;Spirochaetota;Spirochaetia;Spirochaetales;Treponemataceae;Treponema;Treponema sp. OMZ 857 |
| Treponema sp. OMZ 906 | 1 | 7 | 3 | 0 | 7 | 1 | 4 | 3 | 0 | 3 | 1 | 1 | 10 | 41 | Bacteria | Bacteria\_none | Spirochaetota | Spirochaetia | Spirochaetales | Treponemataceae | Treponema | Bacteria;Bacteria\_none;Spirochaetota;Spirochaetia;Spirochaetales;Treponemataceae;Treponema;Treponema sp. OMZ 906 |
| Treponema succinifaciens | 0 | 0 | 0 | 0 | 0 | 0 | 0 | 0 | 0 | 0 | 0 | 1 | 0 | 1 | Bacteria | Bacteria\_none | Spirochaetota | Spirochaetia | Spirochaetales | Treponemataceae | Treponema | Bacteria;Bacteria\_none;Spirochaetota;Spirochaetia;Spirochaetales;Treponemataceae;Treponema;Treponema succinifaciens |
| Treponema vincentii | 1 | 17 | 1 | 1 | 5 | 16 | 1 | 16 | 0 | 1 | 5 | 23 | 14 | 101 | Bacteria | Bacteria\_none | Spirochaetota | Spirochaetia | Spirochaetales | Treponemataceae | Treponema | Bacteria;Bacteria\_none;Spirochaetota;Spirochaetia;Spirochaetales;Treponemataceae;Treponema;Treponema vincentii |
| Fretibacterium fastidiosum | 0 | 0 | 1 | 0 | 0 | 0 | 0 | 0 | 0 | 0 | 1 | 1 | 0 | 3 | Bacteria | Bacteria\_none | Synergistota | Synergistia | Synergistales | Aminobacteriaceae | Fretibacterium | Bacteria;Bacteria\_none;Synergistota;Synergistia;Synergistales;Aminobacteriaceae;Fretibacterium;Fretibacterium fastidiosum |
| Candidatus Desulfofervidus auxilii | 0 | 0 | 0 | 0 | 0 | 1 | 0 | 0 | 0 | 0 | 0 | 0 | 0 | 1 | Bacteria | Bacteria\_none | Thermodesulfobacteriota | Candidatus Desulfofervidia | Candidatus Desulfofervidales | Candidatus Desulfofervidaceae | Candidatus Desulfofervidus | Bacteria;Bacteria\_none;Thermodesulfobacteriota;Candidatus Desulfofervidia;Candidatus Desulfofervidales;Candidatus Desulfofervidaceae;Candidatus Desulfofervidus;Candidatus Desulfofervidus auxilii |
| Desulfoferula mesophila | 0 | 0 | 0 | 0 | 0 | 1 | 0 | 0 | 0 | 0 | 0 | 0 | 0 | 1 | Bacteria | Bacteria\_none | Thermodesulfobacteriota | Desulfarculia | Desulfarculales | Desulfarculaceae | Desulfoferula | Bacteria;Bacteria\_none;Thermodesulfobacteriota;Desulfarculia;Desulfarculales;Desulfarculaceae;Desulfoferula;Desulfoferula mesophila |
| Desulfobulbus oralis | 0 | 2 | 1 | 0 | 0 | 0 | 0 | 5 | 0 | 0 | 0 | 0 | 1 | 9 | Bacteria | Bacteria\_none | Thermodesulfobacteriota | Desulfobulbia | Desulfobulbales | Desulfobulbaceae | Desulfobulbus | Bacteria;Bacteria\_none;Thermodesulfobacteriota;Desulfobulbia;Desulfobulbales;Desulfobulbaceae;Desulfobulbus;Desulfobulbus oralis |
| Lawsonia intracellularis | 0 | 0 | 0 | 0 | 0 | 0 | 0 | 0 | 0 | 0 | 0 | 1 | 0 | 1 | Bacteria | Bacteria\_none | Thermodesulfobacteriota | Desulfovibrionia | Desulfovibrionales | Desulfovibrionaceae | Lawsonia | Bacteria;Bacteria\_none;Thermodesulfobacteriota;Desulfovibrionia;Desulfovibrionales;Desulfovibrionaceae;Lawsonia;Lawsonia intracellularis |
| Syntrophotalea acetylenivorans | 0 | 0 | 0 | 0 | 0 | 0 | 0 | 0 | 0 | 0 | 1 | 0 | 0 | 1 | Bacteria | Bacteria\_none | Thermodesulfobacteriota | Desulfuromonadia | Desulfuromonadales | Syntrophotaleaceae | Syntrophotalea | Bacteria;Bacteria\_none;Thermodesulfobacteriota;Desulfuromonadia;Desulfuromonadales;Syntrophotaleaceae;Syntrophotalea;Syntrophotalea acetylenivorans |
| Geobacter sulfurreducens | 0 | 0 | 1 | 0 | 0 | 0 | 0 | 0 | 0 | 0 | 0 | 0 | 0 | 1 | Bacteria | Bacteria\_none | Thermodesulfobacteriota | Desulfuromonadia | Geobacterales | Geobacteraceae | Geobacter | Bacteria;Bacteria\_none;Thermodesulfobacteriota;Desulfuromonadia;Geobacterales;Geobacteraceae;Geobacter;Geobacter sulfurreducens |
| Thermomicrobium roseum | 0 | 1 | 0 | 0 | 0 | 0 | 0 | 0 | 0 | 0 | 0 | 0 | 0 | 1 | Bacteria | Bacteria\_none | Thermomicrobiota | Thermomicrobia | Thermomicrobiales | Thermomicrobiaceae | Thermomicrobium | Bacteria;Bacteria\_none;Thermomicrobiota;Thermomicrobia;Thermomicrobiales;Thermomicrobiaceae;Thermomicrobium;Thermomicrobium roseum |
| Thermosipho ferrireducens | 1 | 0 | 0 | 0 | 0 | 0 | 0 | 0 | 0 | 0 | 0 | 0 | 0 | 1 | Bacteria | Bacteria\_none | Thermotogota | Thermotogae | Thermotogales | Fervidobacteriaceae | Thermosipho | Bacteria;Bacteria\_none;Thermotogota;Thermotogae;Thermotogales;Fervidobacteriaceae;Thermosipho;Thermosipho ferrireducens |
| Lacunisphaera limnophila | 0 | 1 | 0 | 0 | 0 | 0 | 0 | 0 | 0 | 0 | 0 | 0 | 0 | 1 | Bacteria | Bacteria\_none | Verrucomicrobiota | Opitutia | Opitutales | Opitutaceae | Lacunisphaera | Bacteria;Bacteria\_none;Verrucomicrobiota;Opitutia;Opitutales;Opitutaceae;Lacunisphaera;Lacunisphaera limnophila |
| Homo sapiens | 428 | 938 | 1105 | 97 | 2901 | 211 | 12043 | 17730 | 14114 | 5069 | 5655 | 9919 | 34174 | 104384 | Eukaryota | Metazoa | Chordata | Mammalia | Primates | Hominidae | Homo | Eukaryota;Metazoa;Chordata;Mammalia;Primates;Hominidae;Homo;Homo sapiens |
| Unknown | 1409 | 1058 | 1350 | 2756 | 1867 | 2605 | 1040 | 1418 | 519 | 1446 | 1552 | 1325 | 24901 | 43246 | Unclassified | Unknown | Unknown | Unknown | Unknown | Unknown | Unknown | Unclassified;Unknown;Unknown;Unknown;Unknown;Unknown;Unknown;Unknown |
| Mimivirus bradfordmassiliense | 0 | 0 | 0 | 0 | 0 | 0 | 0 | 0 | 0 | 0 | 0 | 1 | 0 | 1 | Viruses | Bamfordvirae | Nucleocytoviricota | Megaviricetes | Imitervirales | Mimiviridae | Mimivirus | Viruses;Bamfordvirae;Nucleocytoviricota;Megaviricetes;Imitervirales;Mimiviridae;Mimivirus;Mimivirus bradfordmassiliense |
| Theiavirus salishense | 0 | 0 | 0 | 0 | 0 | 0 | 0 | 1 | 0 | 0 | 0 | 0 | 0 | 1 | Viruses | Bamfordvirae | Nucleocytoviricota | Megaviricetes | Imitervirales | Mimiviridae | Theiavirus | Viruses;Bamfordvirae;Nucleocytoviricota;Megaviricetes;Imitervirales;Mimiviridae;Theiavirus;Theiavirus salishense |
| Biavirus raunefjordenense | 0 | 0 | 0 | 0 | 0 | 0 | 0 | 0 | 0 | 0 | 0 | 0 | 1 | 1 | Viruses | Bamfordvirae | Nucleocytoviricota | Megaviricetes | Imitervirales | Schizomimiviridae | Biavirus | Viruses;Bamfordvirae;Nucleocytoviricota;Megaviricetes;Imitervirales;Schizomimiviridae;Biavirus;Biavirus raunefjordenense |
| Lymphocryptovirus humangamma4 | 8 | 0 | 0 | 0 | 0 | 1 | 0 | 1 | 0 | 0 | 0 | 0 | 2 | 12 | Viruses | Heunggongvirae | Peploviricota | Herviviricetes | Herpesvirales | Orthoherpesviridae | Lymphocryptovirus | Viruses;Heunggongvirae;Peploviricota;Herviviricetes;Herpesvirales;Orthoherpesviridae;Lymphocryptovirus;Lymphocryptovirus humangamma4 |
| Streptococcus phage EJ-1 | 0 | 0 | 0 | 0 | 2 | 0 | 0 | 0 | 0 | 0 | 0 | 0 | 0 | 2 | Viruses | Heunggongvirae | Uroviricota | Caudoviricetes | Caudoviricetes\_Incertae\_sedis | Caudoviricetes\_Incertae\_sedis | Caudoviricetes\_Incertae\_sedis | Viruses;Heunggongvirae;Uroviricota;Caudoviricetes;Caudoviricetes\_Incertae\_sedis;Caudoviricetes\_Incertae\_sedis;Caudoviricetes\_Incertae\_sedis;Streptococcus phage EJ-1 |
| Streptococcus phage PH10 | 5 | 0 | 0 | 1 | 0 | 0 | 4 | 0 | 0 | 0 | 2 | 0 | 2 | 14 | Viruses | Heunggongvirae | Uroviricota | Caudoviricetes | Caudoviricetes\_Incertae\_sedis | Caudoviricetes\_Incertae\_sedis | Caudoviricetes\_Incertae\_sedis | Viruses;Heunggongvirae;Uroviricota;Caudoviricetes;Caudoviricetes\_Incertae\_sedis;Caudoviricetes\_Incertae\_sedis;Caudoviricetes\_Incertae\_sedis;Streptococcus phage PH10 |
| Streptococcus phage SpSL1 | 0 | 0 | 0 | 0 | 1 | 0 | 1 | 0 | 0 | 0 | 0 | 1 | 0 | 3 | Viruses | Heunggongvirae | Uroviricota | Caudoviricetes | Caudoviricetes\_Incertae\_sedis | Caudoviricetes\_Incertae\_sedis | Caudoviricetes\_Incertae\_sedis | Viruses;Heunggongvirae;Uroviricota;Caudoviricetes;Caudoviricetes\_Incertae\_sedis;Caudoviricetes\_Incertae\_sedis;Caudoviricetes\_Incertae\_sedis;Streptococcus phage SpSL1 |
| Vibrio phage qdvp001 | 0 | 0 | 0 | 0 | 0 | 1 | 0 | 0 | 0 | 0 | 0 | 0 | 0 | 1 | Viruses | Heunggongvirae | Uroviricota | Caudoviricetes | Caudoviricetes\_Incertae\_sedis | Caudoviricetes\_Incertae\_sedis | Caudoviricetes\_Incertae\_sedis | Viruses;Heunggongvirae;Uroviricota;Caudoviricetes;Caudoviricetes\_Incertae\_sedis;Caudoviricetes\_Incertae\_sedis;Caudoviricetes\_Incertae\_sedis;Vibrio phage qdvp001 |
| Whopevirus animalis | 2 | 0 | 0 | 0 | 0 | 0 | 0 | 0 | 0 | 0 | 0 | 0 | 0 | 2 | Viruses | Heunggongvirae | Uroviricota | Caudoviricetes | Crassvirales | Intestiviridae | Whopevirus | Viruses;Heunggongvirae;Uroviricota;Caudoviricetes;Crassvirales;Intestiviridae;Whopevirus;Whopevirus animalis |
| Cytorhabdovirus caricae | 0 | 0 | 0 | 0 | 0 | 0 | 0 | 0 | 0 | 0 | 0 | 0 | 1 | 1 | Viruses | Orthornavirae | Negarnaviricota | Monjiviricetes | Mononegavirales | Rhabdoviridae | Cytorhabdovirus | Viruses;Orthornavirae;Negarnaviricota;Monjiviricetes;Mononegavirales;Rhabdoviridae;Cytorhabdovirus;Cytorhabdovirus caricae |
| Alphapapillomavirus 9 | 0 | 0 | 0 | 0 | 0 | 0 | 0 | 2 | 0 | 0 | 0 | 0 | 0 | 2 | Viruses | Shotokuvirae | Cossaviricota | Papovaviricetes | Zurhausenvirales | Papillomaviridae | Alphapapillomavirus | Viruses;Shotokuvirae;Cossaviricota;Papovaviricetes;Zurhausenvirales;Papillomaviridae;Alphapapillomavirus;Alphapapillomavirus 9 |
| Alphalipothrixvirus beppuense | 0 | 0 | 0 | 0 | 0 | 0 | 0 | 0 | 0 | 0 | 0 | 0 | 1 | 1 | Viruses | Zilligvirae | Taleaviricota | Tokiviricetes | Ligamenvirales | Lipothrixviridae | Alphalipothrixvirus | Viruses;Zilligvirae;Taleaviricota;Tokiviricetes;Ligamenvirales;Lipothrixviridae;Alphalipothrixvirus;Alphalipothrixvirus beppuense |

Export CSV

Rarefied abundance table for the phylum rank.

All samples have been randomly subsetted to have the same number
of reads.

| phylum | barcode01 | barcode02 | barcode03 | barcode04 | barcode05 | barcode06 | barcode07 | barcode08 | barcode09 | barcode10 | barcode11 | barcode12 | total | unclassified | superkingdom | kingdom | tax |
| --- | --- | --- | --- | --- | --- | --- | --- | --- | --- | --- | --- | --- | --- | --- | --- | --- | --- |
| Euryarchaeota | 1 | 2 | 2 | 1 | 1 | 4 | 0 | 0 | 0 | 1 | 1 | 1 | 1 | 1 | Archaea | Archaea\_none | Archaea;Archaea\_none;Euryarchaeota |
| Nitrososphaerota | 0 | 0 | 0 | 0 | 0 | 1 | 0 | 0 | 0 | 0 | 0 | 0 | 0 | 0 | Archaea | Archaea\_none | Archaea;Archaea\_none;Nitrososphaerota |
| Acidobacteriota | 0 | 0 | 0 | 0 | 0 | 0 | 0 | 0 | 0 | 0 | 0 | 1 | 0 | 0 | Bacteria | Bacteria\_none | Bacteria;Bacteria\_none;Acidobacteriota |
| Actinomycetota | 51 | 138 | 35 | 20 | 86 | 161 | 56 | 26 | 9 | 52 | 96 | 79 | 53 | 24 | Bacteria | Bacteria\_none | Bacteria;Bacteria\_none;Actinomycetota |
| Bacillota | 3153 | 1701 | 1337 | 485 | 2030 | 2300 | 1018 | 196 | 238 | 979 | 1866 | 536 | 923 | 555 | Bacteria | Bacteria\_none | Bacteria;Bacteria\_none;Bacillota |
| Bacteroidota | 598 | 1250 | 420 | 631 | 201 | 314 | 104 | 148 | 144 | 324 | 253 | 204 | 240 | 126 | Bacteria | Bacteria\_none | Bacteria;Bacteria\_none;Bacteroidota |
| Balneolota | 0 | 0 | 0 | 1 | 0 | 0 | 0 | 0 | 0 | 0 | 0 | 0 | 0 | 0 | Bacteria | Bacteria\_none | Bacteria;Bacteria\_none;Balneolota |
| Bdellovibrionota | 0 | 0 | 0 | 0 | 0 | 0 | 0 | 0 | 0 | 1 | 0 | 0 | 0 | 0 | Bacteria | Bacteria\_none | Bacteria;Bacteria\_none;Bdellovibrionota |
| Calditrichota | 0 | 0 | 0 | 0 | 0 | 0 | 0 | 0 | 0 | 0 | 0 | 0 | 0 | 0 | Bacteria | Bacteria\_none | Bacteria;Bacteria\_none;Calditrichota |
| Campylobacterota | 1 | 43 | 14 | 6 | 5 | 31 | 0 | 3 | 6 | 8 | 2 | 4 | 5 | 6 | Bacteria | Bacteria\_none | Bacteria;Bacteria\_none;Campylobacterota |
| Candidatus Saccharibacteria | 0 | 7 | 5 | 0 | 9 | 138 | 0 | 1 | 1 | 1 | 48 | 4 | 10 | 5 | Bacteria | Bacteria\_none | Bacteria;Bacteria\_none;Candidatus Saccharibacteria |
| Chlamydiota | 0 | 0 | 0 | 0 | 0 | 0 | 0 | 0 | 0 | 0 | 0 | 0 | 0 | 0 | Bacteria | Bacteria\_none | Bacteria;Bacteria\_none;Chlamydiota |
| Chlorobiota | 0 | 0 | 0 | 0 | 0 | 1 | 0 | 0 | 0 | 0 | 0 | 0 | 0 | 0 | Bacteria | Bacteria\_none | Bacteria;Bacteria\_none;Chlorobiota |
| Chloroflexota | 0 | 0 | 0 | 0 | 0 | 0 | 0 | 0 | 0 | 0 | 1 | 0 | 0 | 0 | Bacteria | Bacteria\_none | Bacteria;Bacteria\_none;Chloroflexota |
| Cyanobacteriota | 1 | 1 | 1 | 1 | 0 | 1 | 0 | 0 | 0 | 2 | 1 | 0 | 0 | 0 | Bacteria | Bacteria\_none | Bacteria;Bacteria\_none;Cyanobacteriota |
| Deferribacterota | 0 | 1 | 0 | 0 | 0 | 0 | 0 | 0 | 0 | 0 | 0 | 0 | 0 | 0 | Bacteria | Bacteria\_none | Bacteria;Bacteria\_none;Deferribacterota |
| Fusobacteriota | 15 | 251 | 209 | 438 | 121 | 195 | 164 | 142 | 6 | 75 | 149 | 51 | 136 | 125 | Bacteria | Bacteria\_none | Bacteria;Bacteria\_none;Fusobacteriota |
| Ignavibacteriota | 0 | 0 | 0 | 0 | 0 | 0 | 0 | 0 | 0 | 0 | 0 | 0 | 0 | 0 | Bacteria | Bacteria\_none | Bacteria;Bacteria\_none;Ignavibacteriota |
| Mycoplasmatota | 0 | 11 | 37 | 1 | 1 | 10 | 1 | 0 | 0 | 5 | 0 | 4 | 4 | 1 | Bacteria | Bacteria\_none | Bacteria;Bacteria\_none;Mycoplasmatota |
| Myxococcota | 0 | 0 | 0 | 0 | 0 | 0 | 0 | 0 | 0 | 0 | 0 | 0 | 0 | 0 | Bacteria | Bacteria\_none | Bacteria;Bacteria\_none;Myxococcota |
| Nitrospirota | 0 | 0 | 0 | 0 | 0 | 1 | 0 | 0 | 0 | 0 | 0 | 0 | 0 | 0 | Bacteria | Bacteria\_none | Bacteria;Bacteria\_none;Nitrospirota |
| Planctomycetota | 1 | 0 | 0 | 0 | 0 | 0 | 0 | 0 | 0 | 0 | 0 | 0 | 0 | 0 | Bacteria | Bacteria\_none | Bacteria;Bacteria\_none;Planctomycetota |
| Pseudomonadota | 251 | 346 | 1414 | 2608 | 417 | 433 | 35 | 520 | 2 | 919 | 147 | 150 | 436 | 265 | Bacteria | Bacteria\_none | Bacteria;Bacteria\_none;Pseudomonadota |
| Spirochaetota | 4 | 69 | 47 | 4 | 22 | 57 | 3 | 25 | 0 | 7 | 21 | 50 | 21 | 10 | Bacteria | Bacteria\_none | Bacteria;Bacteria\_none;Spirochaetota |
| Synergistota | 0 | 0 | 1 | 0 | 0 | 0 | 0 | 0 | 0 | 0 | 0 | 1 | 0 | 0 | Bacteria | Bacteria\_none | Bacteria;Bacteria\_none;Synergistota |
| Thermodesulfobacteriota | 0 | 2 | 2 | 0 | 0 | 1 | 0 | 0 | 0 | 0 | 1 | 0 | 0 | 0 | Bacteria | Bacteria\_none | Bacteria;Bacteria\_none;Thermodesulfobacteriota |
| Thermomicrobiota | 0 | 1 | 0 | 0 | 0 | 0 | 0 | 0 | 0 | 0 | 0 | 0 | 0 | 0 | Bacteria | Bacteria\_none | Bacteria;Bacteria\_none;Thermomicrobiota |
| Thermotogota | 0 | 0 | 0 | 0 | 0 | 0 | 0 | 0 | 0 | 0 | 0 | 0 | 0 | 0 | Bacteria | Bacteria\_none | Bacteria;Bacteria\_none;Thermotogota |
| Verrucomicrobiota | 0 | 1 | 0 | 0 | 0 | 0 | 0 | 0 | 0 | 0 | 0 | 0 | 0 | 0 | Bacteria | Bacteria\_none | Bacteria;Bacteria\_none;Verrucomicrobiota |
| Chordata | 369 | 887 | 988 | 49 | 1701 | 155 | 3987 | 4315 | 5117 | 2626 | 2486 | 4099 | 2696 | 2664 | Eukaryota | Metazoa | Eukaryota;Metazoa;Chordata |
| Unknown | 1254 | 1002 | 1201 | 1467 | 1118 | 1908 | 344 | 337 | 190 | 713 | 641 | 528 | 1188 | 1931 | Unclassified | Unknown | Unclassified;Unknown;Unknown |
| Nucleocytoviricota | 0 | 0 | 0 | 0 | 0 | 0 | 0 | 0 | 0 | 0 | 0 | 0 | 0 | 0 | Viruses | Bamfordvirae | Viruses;Bamfordvirae;Nucleocytoviricota |
| Peploviricota | 7 | 0 | 0 | 0 | 0 | 1 | 0 | 0 | 0 | 0 | 0 | 0 | 0 | 0 | Viruses | Heunggongvirae | Viruses;Heunggongvirae;Peploviricota |
| Uroviricota | 7 | 0 | 0 | 1 | 1 | 1 | 1 | 0 | 0 | 0 | 0 | 1 | 0 | 0 | Viruses | Heunggongvirae | Viruses;Heunggongvirae;Uroviricota |
| Negarnaviricota | 0 | 0 | 0 | 0 | 0 | 0 | 0 | 0 | 0 | 0 | 0 | 0 | 0 | 0 | Viruses | Orthornavirae | Viruses;Orthornavirae;Negarnaviricota |
| Cossaviricota | 0 | 0 | 0 | 0 | 0 | 0 | 0 | 0 | 0 | 0 | 0 | 0 | 0 | 0 | Viruses | Shotokuvirae | Viruses;Shotokuvirae;Cossaviricota |
| Taleaviricota | 0 | 0 | 0 | 0 | 0 | 0 | 0 | 0 | 0 | 0 | 0 | 0 | 0 | 0 | Viruses | Zilligvirae | Viruses;Zilligvirae;Taleaviricota |

Export CSV

Rarefied abundance table for the class rank.

All samples have been randomly subsetted to have the same number
of reads.

| class | barcode01 | barcode02 | barcode03 | barcode04 | barcode05 | barcode06 | barcode07 | barcode08 | barcode09 | barcode10 | barcode11 | barcode12 | total | unclassified | superkingdom | kingdom | phylum | tax |
| --- | --- | --- | --- | --- | --- | --- | --- | --- | --- | --- | --- | --- | --- | --- | --- | --- | --- | --- |
| Halobacteria | 1 | 0 | 0 | 0 | 0 | 1 | 1 | 0 | 0 | 0 | 0 | 0 | 1 | 0 | Archaea | Archaea\_none | Euryarchaeota | Archaea;Archaea\_none;Euryarchaeota;Halobacteria |
| Methanobacteria | 0 | 0 | 2 | 0 | 0 | 1 | 1 | 0 | 0 | 0 | 0 | 0 | 1 | 0 | Archaea | Archaea\_none | Euryarchaeota | Archaea;Archaea\_none;Euryarchaeota;Methanobacteria |
| Methanococci | 0 | 0 | 1 | 0 | 0 | 0 | 0 | 0 | 0 | 0 | 0 | 0 | 0 | 0 | Archaea | Archaea\_none | Euryarchaeota | Archaea;Archaea\_none;Euryarchaeota;Methanococci |
| Methanomicrobia | 0 | 1 | 0 | 0 | 1 | 3 | 0 | 0 | 0 | 0 | 1 | 0 | 0 | 0 | Archaea | Archaea\_none | Euryarchaeota | Archaea;Archaea\_none;Euryarchaeota;Methanomicrobia |
| Thermococci | 0 | 0 | 0 | 0 | 0 | 0 | 0 | 0 | 0 | 0 | 0 | 1 | 0 | 0 | Archaea | Archaea\_none | Euryarchaeota | Archaea;Archaea\_none;Euryarchaeota;Thermococci |
| Nitrososphaerota\_Incertae\_sedis | 0 | 0 | 0 | 0 | 0 | 1 | 0 | 0 | 0 | 0 | 0 | 0 | 0 | 0 | Archaea | Archaea\_none | Nitrososphaerota | Archaea;Archaea\_none;Nitrososphaerota;Nitrososphaerota\_Incertae\_sedis |
| Terriglobia | 0 | 0 | 0 | 0 | 0 | 0 | 0 | 0 | 0 | 0 | 0 | 0 | 0 | 0 | Bacteria | Bacteria\_none | Acidobacteriota | Bacteria;Bacteria\_none;Acidobacteriota;Terriglobia |
| Acidimicrobiia | 0 | 0 | 0 | 0 | 0 | 0 | 0 | 1 | 0 | 0 | 0 | 0 | 0 | 0 | Bacteria | Bacteria\_none | Actinomycetota | Bacteria;Bacteria\_none;Actinomycetota;Acidimicrobiia |
| Actinomycetes | 29 | 135 | 27 | 20 | 82 | 71 | 27 | 23 | 8 | 60 | 84 | 72 | 47 | 16 | Bacteria | Bacteria\_none | Actinomycetota | Bacteria;Bacteria\_none;Actinomycetota;Actinomycetes |
| Coriobacteriia | 26 | 5 | 9 | 0 | 1 | 98 | 17 | 0 | 1 | 0 | 2 | 2 | 9 | 0 | Bacteria | Bacteria\_none | Actinomycetota | Bacteria;Bacteria\_none;Actinomycetota;Coriobacteriia |
| Rubrobacteria | 0 | 0 | 0 | 0 | 0 | 0 | 0 | 0 | 0 | 0 | 0 | 0 | 0 | 0 | Bacteria | Bacteria\_none | Actinomycetota | Bacteria;Bacteria\_none;Actinomycetota;Rubrobacteria |
| Thermoleophilia | 0 | 2 | 0 | 0 | 0 | 0 | 0 | 0 | 0 | 0 | 0 | 0 | 0 | 0 | Bacteria | Bacteria\_none | Actinomycetota | Bacteria;Bacteria\_none;Actinomycetota;Thermoleophilia |
| Bacilli | 2087 | 1504 | 1113 | 395 | 1953 | 1676 | 974 | 140 | 208 | 854 | 1837 | 456 | 755 | 508 | Bacteria | Bacteria\_none | Bacillota | Bacteria;Bacteria\_none;Bacillota;Bacilli |
| Bacillota\_Incertae\_sedis | 0 | 0 | 0 | 0 | 0 | 0 | 0 | 0 | 0 | 0 | 0 | 0 | 0 | 0 | Bacteria | Bacteria\_none | Bacillota | Bacteria;Bacteria\_none;Bacillota;Bacillota\_Incertae\_sedis |
| Clostridia | 7 | 78 | 70 | 21 | 16 | 95 | 8 | 18 | 2 | 8 | 9 | 9 | 19 | 9 | Bacteria | Bacteria\_none | Bacillota | Bacteria;Bacteria\_none;Bacillota;Clostridia |
| Erysipelotrichia | 14 | 15 | 84 | 5 | 5 | 385 | 18 | 11 | 1 | 2 | 3 | 2 | 24 | 15 | Bacteria | Bacteria\_none | Bacillota | Bacteria;Bacteria\_none;Bacillota;Erysipelotrichia |
| Negativicutes | 1061 | 80 | 51 | 56 | 50 | 45 | 9 | 20 | 33 | 74 | 9 | 52 | 89 | 55 | Bacteria | Bacteria\_none | Bacillota | Bacteria;Bacteria\_none;Bacillota;Negativicutes |
| Tissierellia | 6 | 33 | 24 | 5 | 29 | 49 | 2 | 16 | 0 | 1 | 38 | 11 | 10 | 10 | Bacteria | Bacteria\_none | Bacillota | Bacteria;Bacteria\_none;Bacillota;Tissierellia |
| Bacteroidia | 305 | 1089 | 290 | 518 | 101 | 292 | 85 | 51 | 116 | 126 | 150 | 184 | 176 | 84 | Bacteria | Bacteria\_none | Bacteroidota | Bacteria;Bacteria\_none;Bacteroidota;Bacteroidia |
| Chitinophagia | 0 | 1 | 0 | 0 | 0 | 0 | 0 | 0 | 0 | 0 | 0 | 1 | 1 | 0 | Bacteria | Bacteria\_none | Bacteroidota | Bacteria;Bacteria\_none;Bacteroidota;Chitinophagia |
| Cytophagia | 0 | 0 | 1 | 2 | 2 | 2 | 0 | 0 | 0 | 1 | 1 | 0 | 0 | 0 | Bacteria | Bacteria\_none | Bacteroidota | Bacteria;Bacteria\_none;Bacteroidota;Cytophagia |
| Flavobacteriia | 293 | 145 | 126 | 126 | 108 | 29 | 28 | 91 | 0 | 178 | 87 | 24 | 88 | 56 | Bacteria | Bacteria\_none | Bacteroidota | Bacteria;Bacteria\_none;Bacteroidota;Flavobacteriia |
| Saprospiria | 0 | 0 | 0 | 0 | 0 | 0 | 0 | 0 | 0 | 0 | 0 | 0 | 0 | 0 | Bacteria | Bacteria\_none | Bacteroidota | Bacteria;Bacteria\_none;Bacteroidota;Saprospiria |
| Sphingobacteriia | 0 | 2 | 0 | 2 | 1 | 0 | 0 | 0 | 0 | 1 | 2 | 0 | 0 | 0 | Bacteria | Bacteria\_none | Bacteroidota | Bacteria;Bacteria\_none;Bacteroidota;Sphingobacteriia |
| Balneolia | 0 | 0 | 0 | 0 | 0 | 0 | 0 | 0 | 0 | 0 | 0 | 0 | 0 | 0 | Bacteria | Bacteria\_none | Balneolota | Bacteria;Bacteria\_none;Balneolota;Balneolia |
| Bdellovibrionia | 0 | 0 | 0 | 0 | 0 | 0 | 0 | 0 | 0 | 1 | 0 | 0 | 0 | 0 | Bacteria | Bacteria\_none | Bdellovibrionota | Bacteria;Bacteria\_none;Bdellovibrionota;Bdellovibrionia |
| Calditrichia | 0 | 0 | 0 | 0 | 0 | 0 | 0 | 0 | 0 | 0 | 0 | 0 | 0 | 0 | Bacteria | Bacteria\_none | Calditrichota | Bacteria;Bacteria\_none;Calditrichota;Calditrichia |
| Epsilonproteobacteria | 1 | 46 | 15 | 6 | 8 | 35 | 1 | 2 | 4 | 8 | 1 | 2 | 7 | 8 | Bacteria | Bacteria\_none | Campylobacterota | Bacteria;Bacteria\_none;Campylobacterota;Epsilonproteobacteria |
| Candidatus Saccharibacteria\_Incertae\_sedis | 0 | 0 | 0 | 0 | 2 | 12 | 0 | 0 | 0 | 0 | 0 | 1 | 0 | 1 | Bacteria | Bacteria\_none | Candidatus Saccharibacteria | Bacteria;Bacteria\_none;Candidatus Saccharibacteria;Candidatus Saccharibacteria\_Incertae\_sedis |
| Candidatus Saccharimonadia | 0 | 8 | 5 | 0 | 9 | 119 | 0 | 0 | 2 | 1 | 43 | 2 | 9 | 4 | Bacteria | Bacteria\_none | Candidatus Saccharibacteria | Bacteria;Bacteria\_none;Candidatus Saccharibacteria;Candidatus Saccharimonadia |
| Chlamydiia | 0 | 0 | 0 | 0 | 0 | 0 | 0 | 0 | 0 | 0 | 0 | 0 | 0 | 0 | Bacteria | Bacteria\_none | Chlamydiota | Bacteria;Bacteria\_none;Chlamydiota;Chlamydiia |
| Chlorobiia | 0 | 0 | 0 | 0 | 0 | 1 | 0 | 0 | 0 | 0 | 0 | 0 | 0 | 0 | Bacteria | Bacteria\_none | Chlorobiota | Bacteria;Bacteria\_none;Chlorobiota;Chlorobiia |
| Chloroflexia | 0 | 0 | 0 | 0 | 0 | 0 | 0 | 0 | 0 | 0 | 1 | 0 | 0 | 0 | Bacteria | Bacteria\_none | Chloroflexota | Bacteria;Bacteria\_none;Chloroflexota;Chloroflexia |
| Dehalococcoidia | 0 | 0 | 0 | 0 | 0 | 0 | 0 | 0 | 0 | 0 | 0 | 0 | 0 | 0 | Bacteria | Bacteria\_none | Chloroflexota | Bacteria;Bacteria\_none;Chloroflexota;Dehalococcoidia |
| Cyanobacteriota\_Incertae\_sedis | 0 | 0 | 0 | 0 | 0 | 0 | 0 | 0 | 0 | 0 | 0 | 0 | 0 | 0 | Bacteria | Bacteria\_none | Cyanobacteriota | Bacteria;Bacteria\_none;Cyanobacteriota;Cyanobacteriota\_Incertae\_sedis |
| Cyanophyceae | 0 | 1 | 1 | 1 | 1 | 0 | 1 | 0 | 0 | 1 | 2 | 0 | 0 | 0 | Bacteria | Bacteria\_none | Cyanobacteriota | Bacteria;Bacteria\_none;Cyanobacteriota;Cyanophyceae |
| Deferribacteres | 0 | 1 | 0 | 0 | 0 | 0 | 0 | 0 | 0 | 0 | 0 | 0 | 0 | 0 | Bacteria | Bacteria\_none | Deferribacterota | Bacteria;Bacteria\_none;Deferribacterota;Deferribacteres |
| Fusobacteriia | 16 | 251 | 205 | 432 | 114 | 190 | 155 | 146 | 9 | 76 | 172 | 49 | 153 | 134 | Bacteria | Bacteria\_none | Fusobacteriota | Bacteria;Bacteria\_none;Fusobacteriota;Fusobacteriia |
| Ignavibacteria | 0 | 0 | 0 | 0 | 0 | 0 | 0 | 0 | 0 | 0 | 0 | 0 | 0 | 0 | Bacteria | Bacteria\_none | Ignavibacteriota | Bacteria;Bacteria\_none;Ignavibacteriota;Ignavibacteria |
| Mollicutes | 0 | 1 | 3 | 1 | 1 | 4 | 0 | 0 | 0 | 0 | 1 | 0 | 0 | 0 | Bacteria | Bacteria\_none | Mycoplasmatota | Bacteria;Bacteria\_none;Mycoplasmatota;Mollicutes |
| Mycoplasmatota\_Incertae\_sedis | 0 | 10 | 34 | 2 | 3 | 4 | 0 | 1 | 0 | 3 | 3 | 0 | 1 | 2 | Bacteria | Bacteria\_none | Mycoplasmatota | Bacteria;Bacteria\_none;Mycoplasmatota;Mycoplasmatota\_Incertae\_sedis |
| Myxococcia | 0 | 0 | 0 | 0 | 0 | 0 | 0 | 0 | 0 | 0 | 0 | 0 | 0 | 0 | Bacteria | Bacteria\_none | Myxococcota | Bacteria;Bacteria\_none;Myxococcota;Myxococcia |
| Myxococcota\_Incertae\_sedis | 0 | 0 | 0 | 0 | 1 | 0 | 0 | 0 | 0 | 0 | 1 | 0 | 0 | 0 | Bacteria | Bacteria\_none | Myxococcota | Bacteria;Bacteria\_none;Myxococcota;Myxococcota\_Incertae\_sedis |
| Nitrospiria | 0 | 0 | 0 | 0 | 0 | 0 | 0 | 0 | 0 | 0 | 0 | 0 | 0 | 0 | Bacteria | Bacteria\_none | Nitrospirota | Bacteria;Bacteria\_none;Nitrospirota;Nitrospiria |
| Thermodesulfovibrionia | 0 | 0 | 0 | 0 | 0 | 0 | 0 | 0 | 0 | 0 | 0 | 0 | 0 | 0 | Bacteria | Bacteria\_none | Nitrospirota | Bacteria;Bacteria\_none;Nitrospirota;Thermodesulfovibrionia |
| Planctomycetia | 1 | 0 | 0 | 0 | 0 | 1 | 0 | 0 | 0 | 0 | 1 | 0 | 0 | 0 | Bacteria | Bacteria\_none | Planctomycetota | Bacteria;Bacteria\_none;Planctomycetota;Planctomycetia |
| Alphaproteobacteria | 3 | 3 | 5 | 1 | 3 | 6 | 0 | 2 | 0 | 0 | 4 | 0 | 1 | 0 | Bacteria | Bacteria\_none | Pseudomonadota | Bacteria;Bacteria\_none;Pseudomonadota;Alphaproteobacteria |
| Betaproteobacteria | 179 | 194 | 981 | 2162 | 227 | 251 | 22 | 43 | 2 | 880 | 89 | 53 | 275 | 162 | Bacteria | Bacteria\_none | Pseudomonadota | Bacteria;Bacteria\_none;Pseudomonadota;Betaproteobacteria |
| Gammaproteobacteria | 68 | 142 | 423 | 430 | 193 | 195 | 3 | 493 | 0 | 49 | 26 | 93 | 123 | 104 | Bacteria | Bacteria\_none | Pseudomonadota | Bacteria;Bacteria\_none;Pseudomonadota;Gammaproteobacteria |
| Spirochaetia | 4 | 70 | 48 | 6 | 29 | 56 | 3 | 23 | 0 | 5 | 33 | 50 | 18 | 12 | Bacteria | Bacteria\_none | Spirochaetota | Bacteria;Bacteria\_none;Spirochaetota;Spirochaetia |
| Synergistia | 0 | 0 | 0 | 0 | 0 | 0 | 0 | 0 | 0 | 0 | 1 | 0 | 0 | 0 | Bacteria | Bacteria\_none | Synergistota | Bacteria;Bacteria\_none;Synergistota;Synergistia |
| Candidatus Desulfofervidia | 0 | 0 | 0 | 0 | 0 | 0 | 0 | 0 | 0 | 0 | 0 | 0 | 0 | 0 | Bacteria | Bacteria\_none | Thermodesulfobacteriota | Bacteria;Bacteria\_none;Thermodesulfobacteriota;Candidatus Desulfofervidia |
| Desulfarculia | 0 | 0 | 0 | 0 | 0 | 1 | 0 | 0 | 0 | 0 | 0 | 0 | 0 | 0 | Bacteria | Bacteria\_none | Thermodesulfobacteriota | Bacteria;Bacteria\_none;Thermodesulfobacteriota;Desulfarculia |
| Desulfobulbia | 0 | 2 | 1 | 0 | 0 | 0 | 0 | 1 | 0 | 0 | 0 | 0 | 0 | 0 | Bacteria | Bacteria\_none | Thermodesulfobacteriota | Bacteria;Bacteria\_none;Thermodesulfobacteriota;Desulfobulbia |
| Desulfovibrionia | 0 | 0 | 0 | 0 | 0 | 0 | 0 | 0 | 0 | 0 | 0 | 0 | 0 | 0 | Bacteria | Bacteria\_none | Thermodesulfobacteriota | Bacteria;Bacteria\_none;Thermodesulfobacteriota;Desulfovibrionia |
| Desulfuromonadia | 0 | 0 | 1 | 0 | 0 | 0 | 0 | 0 | 0 | 0 | 0 | 0 | 0 | 0 | Bacteria | Bacteria\_none | Thermodesulfobacteriota | Bacteria;Bacteria\_none;Thermodesulfobacteriota;Desulfuromonadia |
| Thermomicrobia | 0 | 1 | 0 | 0 | 0 | 0 | 0 | 0 | 0 | 0 | 0 | 0 | 0 | 0 | Bacteria | Bacteria\_none | Thermomicrobiota | Bacteria;Bacteria\_none;Thermomicrobiota;Thermomicrobia |
| Thermotogae | 0 | 0 | 0 | 0 | 0 | 0 | 0 | 0 | 0 | 0 | 0 | 0 | 0 | 0 | Bacteria | Bacteria\_none | Thermotogota | Bacteria;Bacteria\_none;Thermotogota;Thermotogae |
| Opitutia | 0 | 1 | 0 | 0 | 0 | 0 | 0 | 0 | 0 | 0 | 0 | 0 | 0 | 0 | Bacteria | Bacteria\_none | Verrucomicrobiota | Bacteria;Bacteria\_none;Verrucomicrobiota;Opitutia |
| Mammalia | 376 | 887 | 973 | 47 | 1684 | 149 | 4003 | 4266 | 5139 | 2635 | 2441 | 4093 | 2798 | 2637 | Eukaryota | Metazoa | Chordata | Eukaryota;Metazoa;Chordata;Mammalia |
| Unknown | 1221 | 1005 | 1220 | 1474 | 1088 | 1940 | 353 | 365 | 188 | 749 | 670 | 555 | 1107 | 1895 | Unclassified | Unknown | Unknown | Unclassified;Unknown;Unknown;Unknown |
| Megaviricetes | 0 | 0 | 0 | 0 | 0 | 0 | 0 | 0 | 0 | 0 | 0 | 1 | 0 | 1 | Viruses | Bamfordvirae | Nucleocytoviricota | Viruses;Bamfordvirae;Nucleocytoviricota;Megaviricetes |
| Herviviricetes | 8 | 0 | 0 | 0 | 0 | 1 | 0 | 0 | 0 | 0 | 0 | 0 | 0 | 0 | Viruses | Heunggongvirae | Peploviricota | Viruses;Heunggongvirae;Peploviricota;Herviviricetes |
| Caudoviricetes | 7 | 0 | 0 | 1 | 1 | 0 | 2 | 0 | 0 | 0 | 1 | 0 | 1 | 0 | Viruses | Heunggongvirae | Uroviricota | Viruses;Heunggongvirae;Uroviricota;Caudoviricetes |
| Monjiviricetes | 0 | 0 | 0 | 0 | 0 | 0 | 0 | 0 | 0 | 0 | 0 | 0 | 0 | 0 | Viruses | Orthornavirae | Negarnaviricota | Viruses;Orthornavirae;Negarnaviricota;Monjiviricetes |
| Papovaviricetes | 0 | 0 | 0 | 0 | 0 | 0 | 0 | 0 | 0 | 0 | 0 | 0 | 0 | 0 | Viruses | Shotokuvirae | Cossaviricota | Viruses;Shotokuvirae;Cossaviricota;Papovaviricetes |
| Tokiviricetes | 0 | 0 | 0 | 0 | 0 | 0 | 0 | 0 | 0 | 0 | 0 | 0 | 0 | 0 | Viruses | Zilligvirae | Taleaviricota | Viruses;Zilligvirae;Taleaviricota;Tokiviricetes |

Export CSV

Rarefied abundance table for the order rank.

All samples have been randomly subsetted to have the same number
of reads.

| order | barcode01 | barcode02 | barcode03 | barcode04 | barcode05 | barcode06 | barcode07 | barcode08 | barcode09 | barcode10 | barcode11 | barcode12 | total | unclassified | superkingdom | kingdom | phylum | class | tax |
| --- | --- | --- | --- | --- | --- | --- | --- | --- | --- | --- | --- | --- | --- | --- | --- | --- | --- | --- | --- |
| Halobacteriales | 1 | 1 | 0 | 0 | 0 | 1 | 1 | 0 | 0 | 0 | 1 | 0 | 0 | 0 | Archaea | Archaea\_none | Euryarchaeota | Halobacteria | Archaea;Archaea\_none;Euryarchaeota;Halobacteria;Halobacteriales |
| Methanobacteriales | 0 | 0 | 1 | 0 | 0 | 1 | 0 | 0 | 0 | 0 | 0 | 0 | 0 | 0 | Archaea | Archaea\_none | Euryarchaeota | Methanobacteria | Archaea;Archaea\_none;Euryarchaeota;Methanobacteria;Methanobacteriales |
| Methanococcales | 0 | 0 | 1 | 0 | 0 | 0 | 0 | 0 | 0 | 0 | 0 | 0 | 0 | 0 | Archaea | Archaea\_none | Euryarchaeota | Methanococci | Archaea;Archaea\_none;Euryarchaeota;Methanococci;Methanococcales |
| Methanomicrobiales | 0 | 0 | 0 | 0 | 0 | 0 | 0 | 0 | 0 | 0 | 0 | 0 | 0 | 0 | Archaea | Archaea\_none | Euryarchaeota | Methanomicrobia | Archaea;Archaea\_none;Euryarchaeota;Methanomicrobia;Methanomicrobiales |
| Methanosarcinales | 0 | 0 | 0 | 1 | 0 | 2 | 0 | 0 | 0 | 0 | 1 | 0 | 0 | 0 | Archaea | Archaea\_none | Euryarchaeota | Methanomicrobia | Archaea;Archaea\_none;Euryarchaeota;Methanomicrobia;Methanosarcinales |
| Thermococcales | 0 | 0 | 0 | 0 | 0 | 0 | 0 | 0 | 0 | 0 | 0 | 0 | 0 | 0 | Archaea | Archaea\_none | Euryarchaeota | Thermococci | Archaea;Archaea\_none;Euryarchaeota;Thermococci;Thermococcales |
| Nitrososphaerota\_Incertae\_sedis | 0 | 0 | 0 | 0 | 0 | 1 | 0 | 0 | 0 | 0 | 0 | 0 | 0 | 0 | Archaea | Archaea\_none | Nitrososphaerota | Nitrososphaerota\_Incertae\_sedis | Archaea;Archaea\_none;Nitrososphaerota;Nitrososphaerota\_Incertae\_sedis;Nitrososphaerota\_Incertae\_sedis |
| Bryobacterales | 0 | 0 | 0 | 0 | 0 | 0 | 0 | 0 | 0 | 0 | 0 | 0 | 0 | 0 | Bacteria | Bacteria\_none | Acidobacteriota | Terriglobia | Bacteria;Bacteria\_none;Acidobacteriota;Terriglobia;Bryobacterales |
| Acidimicrobiales | 0 | 0 | 0 | 0 | 0 | 0 | 0 | 0 | 0 | 0 | 0 | 0 | 0 | 0 | Bacteria | Bacteria\_none | Actinomycetota | Acidimicrobiia | Bacteria;Bacteria\_none;Actinomycetota;Acidimicrobiia;Acidimicrobiales |
| Actinomycetales | 0 | 17 | 1 | 2 | 2 | 27 | 14 | 1 | 2 | 0 | 1 | 5 | 7 | 2 | Bacteria | Bacteria\_none | Actinomycetota | Actinomycetes | Bacteria;Bacteria\_none;Actinomycetota;Actinomycetes;Actinomycetales |
| Bifidobacteriales | 0 | 1 | 2 | 0 | 0 | 1 | 1 | 1 | 0 | 0 | 0 | 0 | 1 | 0 | Bacteria | Bacteria\_none | Actinomycetota | Actinomycetes | Bacteria;Bacteria\_none;Actinomycetota;Actinomycetes;Bifidobacteriales |
| Geodermatophilales | 0 | 0 | 0 | 0 | 1 | 0 | 0 | 0 | 0 | 0 | 0 | 0 | 0 | 0 | Bacteria | Bacteria\_none | Actinomycetota | Actinomycetes | Bacteria;Bacteria\_none;Actinomycetota;Actinomycetes;Geodermatophilales |
| Kitasatosporales | 0 | 2 | 0 | 1 | 1 | 9 | 0 | 0 | 0 | 2 | 1 | 1 | 1 | 1 | Bacteria | Bacteria\_none | Actinomycetota | Actinomycetes | Bacteria;Bacteria\_none;Actinomycetota;Actinomycetes;Kitasatosporales |
| Micrococcales | 26 | 105 | 22 | 10 | 72 | 30 | 13 | 20 | 8 | 41 | 62 | 70 | 21 | 22 | Bacteria | Bacteria\_none | Actinomycetota | Actinomycetes | Bacteria;Bacteria\_none;Actinomycetota;Actinomycetes;Micrococcales |
| Micromonosporales | 1 | 0 | 0 | 1 | 0 | 1 | 0 | 0 | 0 | 1 | 0 | 0 | 0 | 0 | Bacteria | Bacteria\_none | Actinomycetota | Actinomycetes | Bacteria;Bacteria\_none;Actinomycetota;Actinomycetes;Micromonosporales |
| Mycobacteriales | 0 | 7 | 2 | 1 | 1 | 5 | 1 | 0 | 0 | 9 | 0 | 2 | 4 | 1 | Bacteria | Bacteria\_none | Actinomycetota | Actinomycetes | Bacteria;Bacteria\_none;Actinomycetota;Actinomycetes;Mycobacteriales |
| Propionibacteriales | 0 | 1 | 1 | 1 | 0 | 2 | 0 | 0 | 0 | 0 | 22 | 0 | 2 | 0 | Bacteria | Bacteria\_none | Actinomycetota | Actinomycetes | Bacteria;Bacteria\_none;Actinomycetota;Actinomycetes;Propionibacteriales |
| Pseudonocardiales | 0 | 0 | 0 | 0 | 1 | 1 | 0 | 0 | 0 | 0 | 0 | 0 | 0 | 0 | Bacteria | Bacteria\_none | Actinomycetota | Actinomycetes | Bacteria;Bacteria\_none;Actinomycetota;Actinomycetes;Pseudonocardiales |
| Streptosporangiales | 0 | 0 | 1 | 0 | 0 | 1 | 1 | 0 | 0 | 0 | 0 | 0 | 0 | 0 | Bacteria | Bacteria\_none | Actinomycetota | Actinomycetes | Bacteria;Bacteria\_none;Actinomycetota;Actinomycetes;Streptosporangiales |
| Coriobacteriales | 26 | 5 | 10 | 0 | 0 | 96 | 21 | 0 | 0 | 0 | 5 | 1 | 8 | 2 | Bacteria | Bacteria\_none | Actinomycetota | Coriobacteriia | Bacteria;Bacteria\_none;Actinomycetota;Coriobacteriia;Coriobacteriales |
| Eggerthellales | 0 | 0 | 0 | 0 | 0 | 2 | 1 | 0 | 0 | 0 | 0 | 0 | 0 | 0 | Bacteria | Bacteria\_none | Actinomycetota | Coriobacteriia | Bacteria;Bacteria\_none;Actinomycetota;Coriobacteriia;Eggerthellales |
| Rubrobacterales | 0 | 0 | 0 | 0 | 1 | 0 | 0 | 0 | 0 | 0 | 0 | 0 | 0 | 0 | Bacteria | Bacteria\_none | Actinomycetota | Rubrobacteria | Bacteria;Bacteria\_none;Actinomycetota;Rubrobacteria;Rubrobacterales |
| Miltoncostaeales | 0 | 1 | 0 | 0 | 0 | 0 | 0 | 0 | 0 | 0 | 0 | 0 | 0 | 0 | Bacteria | Bacteria\_none | Actinomycetota | Thermoleophilia | Bacteria;Bacteria\_none;Actinomycetota;Thermoleophilia;Miltoncostaeales |
| Solirubrobacterales | 0 | 1 | 0 | 0 | 0 | 0 | 0 | 0 | 0 | 0 | 0 | 0 | 0 | 0 | Bacteria | Bacteria\_none | Actinomycetota | Thermoleophilia | Bacteria;Bacteria\_none;Actinomycetota;Thermoleophilia;Solirubrobacterales |
| Bacillales | 74 | 129 | 17 | 74 | 358 | 264 | 64 | 24 | 2 | 190 | 518 | 37 | 126 | 78 | Bacteria | Bacteria\_none | Bacillota | Bacilli | Bacteria;Bacteria\_none;Bacillota;Bacilli;Bacillales |
| Lactobacillales | 2022 | 1377 | 1079 | 304 | 1634 | 1436 | 958 | 116 | 200 | 665 | 1339 | 428 | 661 | 395 | Bacteria | Bacteria\_none | Bacillota | Bacilli | Bacteria;Bacteria\_none;Bacillota;Bacilli;Lactobacillales |
| Caldicellulosiruptorales | 0 | 0 | 0 | 0 | 1 | 0 | 0 | 0 | 0 | 0 | 0 | 0 | 0 | 0 | Bacteria | Bacteria\_none | Bacillota | Bacillota\_Incertae\_sedis | Bacteria;Bacteria\_none;Bacillota;Bacillota\_Incertae\_sedis;Caldicellulosiruptorales |
| Eubacteriales | 4 | 4 | 5 | 4 | 3 | 16 | 2 | 2 | 3 | 0 | 2 | 0 | 0 | 2 | Bacteria | Bacteria\_none | Bacillota | Clostridia | Bacteria;Bacteria\_none;Bacillota;Clostridia;Eubacteriales |
| Halanaerobiales | 0 | 0 | 0 | 0 | 0 | 0 | 0 | 0 | 0 | 0 | 0 | 0 | 0 | 0 | Bacteria | Bacteria\_none | Bacillota | Clostridia | Bacteria;Bacteria\_none;Bacillota;Clostridia;Halanaerobiales |
| Lachnospirales | 0 | 33 | 23 | 7 | 7 | 49 | 2 | 0 | 0 | 6 | 3 | 1 | 8 | 1 | Bacteria | Bacteria\_none | Bacillota | Clostridia | Bacteria;Bacteria\_none;Bacillota;Clostridia;Lachnospirales |
| Monoglobales | 0 | 0 | 0 | 0 | 0 | 0 | 0 | 0 | 0 | 0 | 0 | 0 | 0 | 0 | Bacteria | Bacteria\_none | Bacillota | Clostridia | Bacteria;Bacteria\_none;Bacillota;Clostridia;Monoglobales |
| Natranaerobiales | 0 | 0 | 0 | 1 | 0 | 1 | 0 | 0 | 0 | 0 | 0 | 0 | 0 | 0 | Bacteria | Bacteria\_none | Bacillota | Clostridia | Bacteria;Bacteria\_none;Bacillota;Clostridia;Natranaerobiales |
| Peptostreptococcales | 4 | 39 | 40 | 4 | 7 | 35 | 1 | 10 | 1 | 0 | 7 | 11 | 11 | 5 | Bacteria | Bacteria\_none | Bacillota | Clostridia | Bacteria;Bacteria\_none;Bacillota;Clostridia;Peptostreptococcales |
| Thermoanaerobacterales | 0 | 1 | 0 | 0 | 0 | 0 | 0 | 0 | 0 | 0 | 0 | 0 | 0 | 0 | Bacteria | Bacteria\_none | Bacillota | Clostridia | Bacteria;Bacteria\_none;Bacillota;Clostridia;Thermoanaerobacterales |
| Erysipelotrichales | 13 | 16 | 86 | 5 | 10 | 372 | 14 | 7 | 0 | 4 | 3 | 0 | 24 | 8 | Bacteria | Bacteria\_none | Bacillota | Erysipelotrichia | Bacteria;Bacteria\_none;Bacillota;Erysipelotrichia;Erysipelotrichales |
| Selenomonadales | 4 | 7 | 13 | 1 | 5 | 11 | 3 | 4 | 1 | 1 | 2 | 6 | 3 | 3 | Bacteria | Bacteria\_none | Bacillota | Negativicutes | Bacteria;Bacteria\_none;Bacillota;Negativicutes;Selenomonadales |
| Veillonellales | 1035 | 75 | 37 | 53 | 36 | 42 | 11 | 16 | 22 | 73 | 7 | 57 | 75 | 49 | Bacteria | Bacteria\_none | Bacillota | Negativicutes | Bacteria;Bacteria\_none;Bacillota;Negativicutes;Veillonellales |
| Tissierellales | 4 | 31 | 18 | 7 | 27 | 55 | 2 | 22 | 0 | 0 | 28 | 11 | 13 | 9 | Bacteria | Bacteria\_none | Bacillota | Tissierellia | Bacteria;Bacteria\_none;Bacillota;Tissierellia;Tissierellales |
| Tissierellia\_Incertae\_sedis | 0 | 0 | 0 | 1 | 0 | 0 | 0 | 0 | 0 | 0 | 6 | 0 | 0 | 0 | Bacteria | Bacteria\_none | Bacillota | Tissierellia | Bacteria;Bacteria\_none;Bacillota;Tissierellia;Tissierellia\_Incertae\_sedis |
| Bacteroidales | 305 | 1092 | 298 | 514 | 95 | 303 | 76 | 59 | 120 | 128 | 169 | 171 | 183 | 101 | Bacteria | Bacteria\_none | Bacteroidota | Bacteroidia | Bacteria;Bacteria\_none;Bacteroidota;Bacteroidia;Bacteroidales |
| Marinilabiliales | 1 | 0 | 2 | 2 | 0 | 1 | 0 | 0 | 0 | 0 | 1 | 0 | 0 | 0 | Bacteria | Bacteria\_none | Bacteroidota | Bacteroidia | Bacteria;Bacteria\_none;Bacteroidota;Bacteroidia;Marinilabiliales |
| Chitinophagales | 0 | 1 | 0 | 0 | 2 | 1 | 0 | 0 | 0 | 0 | 0 | 0 | 0 | 0 | Bacteria | Bacteria\_none | Bacteroidota | Chitinophagia | Bacteria;Bacteria\_none;Bacteroidota;Chitinophagia;Chitinophagales |
| Cytophagales | 0 | 0 | 1 | 1 | 2 | 2 | 1 | 1 | 0 | 1 | 0 | 0 | 1 | 1 | Bacteria | Bacteria\_none | Bacteroidota | Cytophagia | Bacteria;Bacteria\_none;Bacteroidota;Cytophagia;Cytophagales |
| Flavobacteriales | 281 | 140 | 118 | 117 | 108 | 31 | 28 | 101 | 0 | 187 | 96 | 22 | 80 | 45 | Bacteria | Bacteria\_none | Bacteroidota | Flavobacteriia | Bacteria;Bacteria\_none;Bacteroidota;Flavobacteriia;Flavobacteriales |
| Saprospirales | 0 | 0 | 0 | 0 | 0 | 0 | 0 | 0 | 0 | 0 | 0 | 0 | 0 | 0 | Bacteria | Bacteria\_none | Bacteroidota | Saprospiria | Bacteria;Bacteria\_none;Bacteroidota;Saprospiria;Saprospirales |
| Sphingobacteriales | 0 | 2 | 0 | 2 | 1 | 1 | 0 | 0 | 0 | 0 | 1 | 0 | 1 | 1 | Bacteria | Bacteria\_none | Bacteroidota | Sphingobacteriia | Bacteria;Bacteria\_none;Bacteroidota;Sphingobacteriia;Sphingobacteriales |
| Balneolales | 0 | 0 | 0 | 1 | 0 | 0 | 0 | 0 | 0 | 0 | 0 | 0 | 1 | 0 | Bacteria | Bacteria\_none | Balneolota | Balneolia | Bacteria;Bacteria\_none;Balneolota;Balneolia;Balneolales |
| Bdellovibrionales | 0 | 0 | 0 | 0 | 0 | 0 | 0 | 0 | 0 | 1 | 0 | 0 | 0 | 0 | Bacteria | Bacteria\_none | Bdellovibrionota | Bdellovibrionia | Bacteria;Bacteria\_none;Bdellovibrionota;Bdellovibrionia;Bdellovibrionales |
| Calditrichales | 0 | 0 | 0 | 1 | 0 | 0 | 0 | 0 | 0 | 0 | 0 | 0 | 0 | 0 | Bacteria | Bacteria\_none | Calditrichota | Calditrichia | Bacteria;Bacteria\_none;Calditrichota;Calditrichia;Calditrichales |
| Campylobacterales | 1 | 45 | 13 | 5 | 7 | 38 | 0 | 3 | 2 | 5 | 1 | 5 | 6 | 4 | Bacteria | Bacteria\_none | Campylobacterota | Epsilonproteobacteria | Bacteria;Bacteria\_none;Campylobacterota;Epsilonproteobacteria;Campylobacterales |
| Candidatus Saccharibacteria\_Incertae\_sedis | 0 | 0 | 0 | 0 | 1 | 13 | 0 | 0 | 0 | 0 | 1 | 0 | 0 | 0 | Bacteria | Bacteria\_none | Candidatus Saccharibacteria | Candidatus Saccharibacteria\_Incertae\_sedis | Bacteria;Bacteria\_none;Candidatus Saccharibacteria;Candidatus Saccharibacteria\_Incertae\_sedis;Candidatus Saccharibacteria\_Incertae\_sedis |
| Candidatus Nanosynbacterales | 0 | 7 | 5 | 0 | 5 | 120 | 0 | 0 | 1 | 0 | 49 | 3 | 11 | 6 | Bacteria | Bacteria\_none | Candidatus Saccharibacteria | Candidatus Saccharimonadia | Bacteria;Bacteria\_none;Candidatus Saccharibacteria;Candidatus Saccharimonadia;Candidatus Nanosynbacterales |
| Candidatus Saccharimonadales | 0 | 0 | 0 | 0 | 1 | 0 | 0 | 0 | 0 | 0 | 1 | 0 | 0 | 0 | Bacteria | Bacteria\_none | Candidatus Saccharibacteria | Candidatus Saccharimonadia | Bacteria;Bacteria\_none;Candidatus Saccharibacteria;Candidatus Saccharimonadia;Candidatus Saccharimonadales |
| Parachlamydiales | 0 | 0 | 0 | 0 | 0 | 0 | 0 | 0 | 0 | 0 | 0 | 0 | 0 | 0 | Bacteria | Bacteria\_none | Chlamydiota | Chlamydiia | Bacteria;Bacteria\_none;Chlamydiota;Chlamydiia;Parachlamydiales |
| Chlorobiales | 0 | 0 | 0 | 0 | 0 | 0 | 0 | 0 | 0 | 0 | 0 | 0 | 0 | 0 | Bacteria | Bacteria\_none | Chlorobiota | Chlorobiia | Bacteria;Bacteria\_none;Chlorobiota;Chlorobiia;Chlorobiales |
| Candidatus Chloroheliales | 0 | 0 | 0 | 0 | 0 | 0 | 0 | 0 | 0 | 0 | 1 | 0 | 0 | 0 | Bacteria | Bacteria\_none | Chloroflexota | Chloroflexia | Bacteria;Bacteria\_none;Chloroflexota;Chloroflexia;Candidatus Chloroheliales |
| Candidatus Lucifugimonadales | 0 | 0 | 0 | 0 | 0 | 0 | 0 | 0 | 0 | 0 | 0 | 0 | 0 | 1 | Bacteria | Bacteria\_none | Chloroflexota | Dehalococcoidia | Bacteria;Bacteria\_none;Chloroflexota;Dehalococcoidia;Candidatus Lucifugimonadales |
| Cyanobacteriota\_Incertae\_sedis | 1 | 0 | 0 | 0 | 0 | 0 | 0 | 0 | 0 | 0 | 0 | 0 | 0 | 0 | Bacteria | Bacteria\_none | Cyanobacteriota | Cyanobacteriota\_Incertae\_sedis | Bacteria;Bacteria\_none;Cyanobacteriota;Cyanobacteriota\_Incertae\_sedis;Cyanobacteriota\_Incertae\_sedis |
| Chroococcales | 0 | 0 | 1 | 0 | 0 | 0 | 0 | 0 | 0 | 0 | 0 | 0 | 0 | 0 | Bacteria | Bacteria\_none | Cyanobacteriota | Cyanophyceae | Bacteria;Bacteria\_none;Cyanobacteriota;Cyanophyceae;Chroococcales |
| Gloeobacterales | 0 | 0 | 0 | 0 | 0 | 0 | 1 | 0 | 0 | 0 | 0 | 0 | 0 | 0 | Bacteria | Bacteria\_none | Cyanobacteriota | Cyanophyceae | Bacteria;Bacteria\_none;Cyanobacteriota;Cyanophyceae;Gloeobacterales |
| Nostocales | 0 | 1 | 0 | 0 | 0 | 0 | 0 | 0 | 0 | 1 | 0 | 0 | 1 | 0 | Bacteria | Bacteria\_none | Cyanobacteriota | Cyanophyceae | Bacteria;Bacteria\_none;Cyanobacteriota;Cyanophyceae;Nostocales |
| Synechococcales | 0 | 0 | 0 | 0 | 1 | 1 | 0 | 0 | 0 | 0 | 0 | 0 | 0 | 0 | Bacteria | Bacteria\_none | Cyanobacteriota | Cyanophyceae | Bacteria;Bacteria\_none;Cyanobacteriota;Cyanophyceae;Synechococcales |
| Deferribacterales | 0 | 1 | 0 | 0 | 0 | 0 | 0 | 0 | 0 | 0 | 0 | 0 | 0 | 0 | Bacteria | Bacteria\_none | Deferribacterota | Deferribacteres | Bacteria;Bacteria\_none;Deferribacterota;Deferribacteres;Deferribacterales |
| Fusobacteriales | 15 | 252 | 208 | 462 | 114 | 198 | 148 | 133 | 10 | 79 | 141 | 51 | 114 | 129 | Bacteria | Bacteria\_none | Fusobacteriota | Fusobacteriia | Bacteria;Bacteria\_none;Fusobacteriota;Fusobacteriia;Fusobacteriales |
| Ignavibacteriales | 0 | 0 | 0 | 0 | 0 | 0 | 0 | 0 | 0 | 0 | 0 | 0 | 0 | 0 | Bacteria | Bacteria\_none | Ignavibacteriota | Ignavibacteria | Bacteria;Bacteria\_none;Ignavibacteriota;Ignavibacteria;Ignavibacteriales |
| Acholeplasmatales | 0 | 0 | 0 | 0 | 1 | 0 | 0 | 0 | 0 | 0 | 0 | 0 | 0 | 0 | Bacteria | Bacteria\_none | Mycoplasmatota | Mollicutes | Bacteria;Bacteria\_none;Mycoplasmatota;Mollicutes;Acholeplasmatales |
| Entomoplasmatales | 0 | 0 | 0 | 0 | 0 | 2 | 0 | 0 | 0 | 1 | 0 | 0 | 0 | 0 | Bacteria | Bacteria\_none | Mycoplasmatota | Mollicutes | Bacteria;Bacteria\_none;Mycoplasmatota;Mollicutes;Entomoplasmatales |
| Mycoplasmatales | 0 | 1 | 3 | 0 | 0 | 1 | 0 | 0 | 0 | 0 | 0 | 0 | 1 | 0 | Bacteria | Bacteria\_none | Mycoplasmatota | Mollicutes | Bacteria;Bacteria\_none;Mycoplasmatota;Mollicutes;Mycoplasmatales |
| Mycoplasmoidales | 0 | 10 | 33 | 1 | 2 | 5 | 0 | 0 | 0 | 5 | 1 | 2 | 2 | 2 | Bacteria | Bacteria\_none | Mycoplasmatota | Mycoplasmatota\_Incertae\_sedis | Bacteria;Bacteria\_none;Mycoplasmatota;Mycoplasmatota\_Incertae\_sedis;Mycoplasmoidales |
| Myxococcales | 0 | 0 | 0 | 0 | 0 | 0 | 0 | 0 | 0 | 0 | 0 | 0 | 0 | 1 | Bacteria | Bacteria\_none | Myxococcota | Myxococcia | Bacteria;Bacteria\_none;Myxococcota;Myxococcia;Myxococcales |
| Polyangiales | 0 | 0 | 0 | 0 | 2 | 0 | 0 | 0 | 0 | 0 | 1 | 0 | 0 | 0 | Bacteria | Bacteria\_none | Myxococcota | Myxococcota\_Incertae\_sedis | Bacteria;Bacteria\_none;Myxococcota;Myxococcota\_Incertae\_sedis;Polyangiales |
| Nitrospirales | 0 | 0 | 0 | 0 | 0 | 1 | 0 | 0 | 0 | 0 | 0 | 0 | 0 | 0 | Bacteria | Bacteria\_none | Nitrospirota | Nitrospiria | Bacteria;Bacteria\_none;Nitrospirota;Nitrospiria;Nitrospirales |
| Thermodesulfovibrionales | 0 | 0 | 0 | 0 | 0 | 0 | 0 | 0 | 0 | 0 | 0 | 0 | 0 | 0 | Bacteria | Bacteria\_none | Nitrospirota | Thermodesulfovibrionia | Bacteria;Bacteria\_none;Nitrospirota;Thermodesulfovibrionia;Thermodesulfovibrionales |
| Gemmatales | 0 | 0 | 0 | 0 | 0 | 0 | 0 | 0 | 0 | 0 | 0 | 0 | 0 | 0 | Bacteria | Bacteria\_none | Planctomycetota | Planctomycetia | Bacteria;Bacteria\_none;Planctomycetota;Planctomycetia;Gemmatales |
| Pirellulales | 1 | 0 | 0 | 0 | 0 | 0 | 0 | 0 | 0 | 0 | 0 | 0 | 0 | 0 | Bacteria | Bacteria\_none | Planctomycetota | Planctomycetia | Bacteria;Bacteria\_none;Planctomycetota;Planctomycetia;Pirellulales |
| Planctomycetales | 0 | 0 | 0 | 0 | 0 | 0 | 0 | 0 | 0 | 0 | 0 | 0 | 0 | 0 | Bacteria | Bacteria\_none | Planctomycetota | Planctomycetia | Bacteria;Bacteria\_none;Planctomycetota;Planctomycetia;Planctomycetales |
| Candidatus Pelagibacterales | 0 | 0 | 0 | 0 | 0 | 0 | 0 | 0 | 0 | 0 | 0 | 0 | 0 | 0 | Bacteria | Bacteria\_none | Pseudomonadota | Alphaproteobacteria | Bacteria;Bacteria\_none;Pseudomonadota;Alphaproteobacteria;Candidatus Pelagibacterales |
| Caulobacterales | 0 | 0 | 0 | 0 | 0 | 0 | 0 | 0 | 0 | 0 | 0 | 0 | 0 | 0 | Bacteria | Bacteria\_none | Pseudomonadota | Alphaproteobacteria | Bacteria;Bacteria\_none;Pseudomonadota;Alphaproteobacteria;Caulobacterales |
| Hyphomicrobiales | 1 | 1 | 5 | 0 | 0 | 3 | 0 | 1 | 0 | 1 | 5 | 0 | 0 | 0 | Bacteria | Bacteria\_none | Pseudomonadota | Alphaproteobacteria | Bacteria;Bacteria\_none;Pseudomonadota;Alphaproteobacteria;Hyphomicrobiales |
| Kordiimonadales | 0 | 0 | 0 | 0 | 0 | 0 | 0 | 0 | 0 | 0 | 0 | 0 | 0 | 0 | Bacteria | Bacteria\_none | Pseudomonadota | Alphaproteobacteria | Bacteria;Bacteria\_none;Pseudomonadota;Alphaproteobacteria;Kordiimonadales |
| Rhodobacterales | 2 | 1 | 0 | 1 | 1 | 0 | 0 | 0 | 1 | 0 | 3 | 0 | 1 | 0 | Bacteria | Bacteria\_none | Pseudomonadota | Alphaproteobacteria | Bacteria;Bacteria\_none;Pseudomonadota;Alphaproteobacteria;Rhodobacterales |
| Rhodospirillales | 0 | 0 | 0 | 1 | 1 | 0 | 0 | 0 | 0 | 0 | 0 | 0 | 1 | 0 | Bacteria | Bacteria\_none | Pseudomonadota | Alphaproteobacteria | Bacteria;Bacteria\_none;Pseudomonadota;Alphaproteobacteria;Rhodospirillales |
| Rickettsiales | 0 | 1 | 0 | 0 | 0 | 0 | 1 | 0 | 0 | 0 | 0 | 0 | 0 | 1 | Bacteria | Bacteria\_none | Pseudomonadota | Alphaproteobacteria | Bacteria;Bacteria\_none;Pseudomonadota;Alphaproteobacteria;Rickettsiales |
| Sphingomonadales | 0 | 0 | 0 | 0 | 0 | 1 | 0 | 0 | 0 | 0 | 0 | 0 | 0 | 0 | Bacteria | Bacteria\_none | Pseudomonadota | Alphaproteobacteria | Bacteria;Bacteria\_none;Pseudomonadota;Alphaproteobacteria;Sphingomonadales |
| Betaproteobacteria\_Incertae\_sedis | 0 | 0 | 0 | 0 | 1 | 0 | 0 | 0 | 0 | 0 | 0 | 0 | 0 | 0 | Bacteria | Bacteria\_none | Pseudomonadota | Betaproteobacteria | Bacteria;Bacteria\_none;Pseudomonadota;Betaproteobacteria;Betaproteobacteria\_Incertae\_sedis |
| Burkholderiales | 1 | 1 | 3 | 0 | 4 | 4 | 1 | 0 | 0 | 3 | 4 | 1 | 2 | 1 | Bacteria | Bacteria\_none | Pseudomonadota | Betaproteobacteria | Bacteria;Bacteria\_none;Pseudomonadota;Betaproteobacteria;Burkholderiales |
| Neisseriales | 176 | 197 | 974 | 2209 | 211 | 227 | 20 | 44 | 0 | 868 | 89 | 46 | 288 | 166 | Bacteria | Bacteria\_none | Pseudomonadota | Betaproteobacteria | Bacteria;Bacteria\_none;Pseudomonadota;Betaproteobacteria;Neisseriales |
| Nitrosomonadales | 0 | 0 | 0 | 0 | 0 | 0 | 0 | 0 | 0 | 0 | 0 | 0 | 0 | 0 | Bacteria | Bacteria\_none | Pseudomonadota | Betaproteobacteria | Bacteria;Bacteria\_none;Pseudomonadota;Betaproteobacteria;Nitrosomonadales |
| Rhodocyclales | 0 | 0 | 0 | 0 | 0 | 0 | 0 | 0 | 0 | 0 | 0 | 0 | 0 | 0 | Bacteria | Bacteria\_none | Pseudomonadota | Betaproteobacteria | Bacteria;Bacteria\_none;Pseudomonadota;Betaproteobacteria;Rhodocyclales |
| Aeromonadales | 0 | 0 | 0 | 0 | 0 | 2 | 0 | 0 | 0 | 0 | 0 | 0 | 0 | 1 | Bacteria | Bacteria\_none | Pseudomonadota | Gammaproteobacteria | Bacteria;Bacteria\_none;Pseudomonadota;Gammaproteobacteria;Aeromonadales |
| Alteromonadales | 2 | 1 | 1 | 0 | 1 | 1 | 0 | 1 | 0 | 0 | 0 | 1 | 0 | 0 | Bacteria | Bacteria\_none | Pseudomonadota | Gammaproteobacteria | Bacteria;Bacteria\_none;Pseudomonadota;Gammaproteobacteria;Alteromonadales |
| Cardiobacteriales | 0 | 3 | 13 | 2 | 2 | 4 | 0 | 0 | 0 | 0 | 2 | 0 | 1 | 3 | Bacteria | Bacteria\_none | Pseudomonadota | Gammaproteobacteria | Bacteria;Bacteria\_none;Pseudomonadota;Gammaproteobacteria;Cardiobacteriales |
| Cellvibrionales | 0 | 0 | 0 | 0 | 0 | 0 | 0 | 0 | 0 | 0 | 0 | 0 | 0 | 0 | Bacteria | Bacteria\_none | Pseudomonadota | Gammaproteobacteria | Bacteria;Bacteria\_none;Pseudomonadota;Gammaproteobacteria;Cellvibrionales |
| Chromatiales | 0 | 0 | 0 | 0 | 0 | 0 | 0 | 0 | 0 | 0 | 0 | 0 | 0 | 0 | Bacteria | Bacteria\_none | Pseudomonadota | Gammaproteobacteria | Bacteria;Bacteria\_none;Pseudomonadota;Gammaproteobacteria;Chromatiales |
| Enterobacterales | 5 | 0 | 1 | 1 | 3 | 2 | 0 | 3 | 0 | 2 | 1 | 0 | 1 | 2 | Bacteria | Bacteria\_none | Pseudomonadota | Gammaproteobacteria | Bacteria;Bacteria\_none;Pseudomonadota;Gammaproteobacteria;Enterobacterales |
| Legionellales | 0 | 0 | 0 | 1 | 0 | 0 | 0 | 0 | 0 | 0 | 0 | 0 | 0 | 0 | Bacteria | Bacteria\_none | Pseudomonadota | Gammaproteobacteria | Bacteria;Bacteria\_none;Pseudomonadota;Gammaproteobacteria;Legionellales |
| Lysobacterales | 0 | 0 | 0 | 0 | 2 | 0 | 0 | 0 | 0 | 0 | 0 | 1 | 0 | 0 | Bacteria | Bacteria\_none | Pseudomonadota | Gammaproteobacteria | Bacteria;Bacteria\_none;Pseudomonadota;Gammaproteobacteria;Lysobacterales |
| Methylococcales | 0 | 0 | 0 | 0 | 0 | 1 | 0 | 0 | 0 | 0 | 0 | 0 | 0 | 0 | Bacteria | Bacteria\_none | Pseudomonadota | Gammaproteobacteria | Bacteria;Bacteria\_none;Pseudomonadota;Gammaproteobacteria;Methylococcales |
| Moraxellales | 0 | 0 | 1 | 2 | 0 | 1 | 0 | 1 | 0 | 2 | 0 | 1 | 1 | 0 | Bacteria | Bacteria\_none | Pseudomonadota | Gammaproteobacteria | Bacteria;Bacteria\_none;Pseudomonadota;Gammaproteobacteria;Moraxellales |
| Oceanospirillales | 0 | 1 | 0 | 0 | 0 | 1 | 0 | 1 | 0 | 0 | 0 | 0 | 1 | 1 | Bacteria | Bacteria\_none | Pseudomonadota | Gammaproteobacteria | Bacteria;Bacteria\_none;Pseudomonadota;Gammaproteobacteria;Oceanospirillales |
| Orbales | 0 | 0 | 0 | 0 | 0 | 0 | 0 | 1 | 0 | 0 | 0 | 0 | 0 | 0 | Bacteria | Bacteria\_none | Pseudomonadota | Gammaproteobacteria | Bacteria;Bacteria\_none;Pseudomonadota;Gammaproteobacteria;Orbales |
| Pasteurellales | 65 | 137 | 416 | 402 | 167 | 167 | 1 | 462 | 0 | 46 | 15 | 108 | 144 | 110 | Bacteria | Bacteria\_none | Pseudomonadota | Gammaproteobacteria | Bacteria;Bacteria\_none;Pseudomonadota;Gammaproteobacteria;Pasteurellales |
| Pseudomonadales | 0 | 2 | 0 | 0 | 2 | 0 | 0 | 0 | 0 | 0 | 2 | 0 | 0 | 0 | Bacteria | Bacteria\_none | Pseudomonadota | Gammaproteobacteria | Bacteria;Bacteria\_none;Pseudomonadota;Gammaproteobacteria;Pseudomonadales |
| Thiotrichales | 0 | 0 | 0 | 0 | 0 | 0 | 0 | 0 | 0 | 0 | 0 | 0 | 0 | 0 | Bacteria | Bacteria\_none | Pseudomonadota | Gammaproteobacteria | Bacteria;Bacteria\_none;Pseudomonadota;Gammaproteobacteria;Thiotrichales |
| Vibrionales | 0 | 0 | 3 | 0 | 1 | 1 | 0 | 0 | 0 | 0 | 0 | 0 | 0 | 1 | Bacteria | Bacteria\_none | Pseudomonadota | Gammaproteobacteria | Bacteria;Bacteria\_none;Pseudomonadota;Gammaproteobacteria;Vibrionales |
| Leptospirales | 1 | 0 | 0 | 0 | 0 | 0 | 0 | 0 | 0 | 0 | 0 | 0 | 0 | 0 | Bacteria | Bacteria\_none | Spirochaetota | Spirochaetia | Bacteria;Bacteria\_none;Spirochaetota;Spirochaetia;Leptospirales |
| Spirochaetales | 2 | 74 | 47 | 7 | 16 | 53 | 3 | 26 | 0 | 9 | 23 | 55 | 26 | 14 | Bacteria | Bacteria\_none | Spirochaetota | Spirochaetia | Bacteria;Bacteria\_none;Spirochaetota;Spirochaetia;Spirochaetales |
| Synergistales | 0 | 0 | 1 | 0 | 0 | 0 | 0 | 0 | 0 | 0 | 0 | 0 | 0 | 0 | Bacteria | Bacteria\_none | Synergistota | Synergistia | Bacteria;Bacteria\_none;Synergistota;Synergistia;Synergistales |
| Candidatus Desulfofervidales | 0 | 0 | 0 | 0 | 0 | 1 | 0 | 0 | 0 | 0 | 0 | 0 | 0 | 0 | Bacteria | Bacteria\_none | Thermodesulfobacteriota | Candidatus Desulfofervidia | Bacteria;Bacteria\_none;Thermodesulfobacteriota;Candidatus Desulfofervidia;Candidatus Desulfofervidales |
| Desulfarculales | 0 | 0 | 0 | 0 | 0 | 0 | 0 | 0 | 0 | 0 | 0 | 0 | 0 | 0 | Bacteria | Bacteria\_none | Thermodesulfobacteriota | Desulfarculia | Bacteria;Bacteria\_none;Thermodesulfobacteriota;Desulfarculia;Desulfarculales |
| Desulfobulbales | 0 | 2 | 1 | 0 | 0 | 0 | 0 | 1 | 0 | 0 | 0 | 0 | 0 | 0 | Bacteria | Bacteria\_none | Thermodesulfobacteriota | Desulfobulbia | Bacteria;Bacteria\_none;Thermodesulfobacteriota;Desulfobulbia;Desulfobulbales |
| Desulfovibrionales | 0 | 0 | 0 | 0 | 0 | 0 | 0 | 0 | 0 | 0 | 0 | 1 | 0 | 0 | Bacteria | Bacteria\_none | Thermodesulfobacteriota | Desulfovibrionia | Bacteria;Bacteria\_none;Thermodesulfobacteriota;Desulfovibrionia;Desulfovibrionales |
| Desulfuromonadales | 0 | 0 | 0 | 0 | 0 | 0 | 0 | 0 | 0 | 0 | 0 | 0 | 0 | 0 | Bacteria | Bacteria\_none | Thermodesulfobacteriota | Desulfuromonadia | Bacteria;Bacteria\_none;Thermodesulfobacteriota;Desulfuromonadia;Desulfuromonadales |
| Geobacterales | 0 | 0 | 1 | 0 | 0 | 0 | 0 | 0 | 0 | 0 | 0 | 0 | 0 | 0 | Bacteria | Bacteria\_none | Thermodesulfobacteriota | Desulfuromonadia | Bacteria;Bacteria\_none;Thermodesulfobacteriota;Desulfuromonadia;Geobacterales |
| Thermomicrobiales | 0 | 1 | 0 | 0 | 0 | 0 | 0 | 0 | 0 | 0 | 0 | 0 | 0 | 0 | Bacteria | Bacteria\_none | Thermomicrobiota | Thermomicrobia | Bacteria;Bacteria\_none;Thermomicrobiota;Thermomicrobia;Thermomicrobiales |
| Thermotogales | 1 | 0 | 0 | 0 | 0 | 0 | 0 | 0 | 0 | 0 | 0 | 0 | 0 | 0 | Bacteria | Bacteria\_none | Thermotogota | Thermotogae | Bacteria;Bacteria\_none;Thermotogota;Thermotogae;Thermotogales |
| Opitutales | 0 | 1 | 0 | 0 | 0 | 0 | 0 | 0 | 0 | 0 | 0 | 0 | 0 | 0 | Bacteria | Bacteria\_none | Verrucomicrobiota | Opitutia | Bacteria;Bacteria\_none;Verrucomicrobiota;Opitutia;Opitutales |
| Primates | 386 | 892 | 984 | 51 | 1685 | 159 | 3976 | 4314 | 5153 | 2628 | 2454 | 4068 | 2718 | 2666 | Eukaryota | Metazoa | Chordata | Mammalia | Eukaryota;Metazoa;Chordata;Mammalia;Primates |
| Unknown | 1241 | 993 | 1221 | 1452 | 1107 | 1905 | 346 | 336 | 187 | 754 | 643 | 545 | 1163 | 1877 | Unclassified | Unknown | Unknown | Unknown | Unclassified;Unknown;Unknown;Unknown;Unknown |
| Imitervirales | 0 | 0 | 0 | 0 | 0 | 0 | 0 | 0 | 0 | 0 | 0 | 1 | 0 | 0 | Viruses | Bamfordvirae | Nucleocytoviricota | Megaviricetes | Viruses;Bamfordvirae;Nucleocytoviricota;Megaviricetes;Imitervirales |
| Herpesvirales | 6 | 0 | 0 | 0 | 0 | 0 | 0 | 1 | 0 | 0 | 0 | 0 | 0 | 0 | Viruses | Heunggongvirae | Peploviricota | Herviviricetes | Viruses;Heunggongvirae;Peploviricota;Herviviricetes;Herpesvirales |
| Caudoviricetes\_Incertae\_sedis | 3 | 0 | 0 | 0 | 0 | 1 | 1 | 0 | 0 | 0 | 1 | 1 | 0 | 1 | Viruses | Heunggongvirae | Uroviricota | Caudoviricetes | Viruses;Heunggongvirae;Uroviricota;Caudoviricetes;Caudoviricetes\_Incertae\_sedis |
| Crassvirales | 2 | 0 | 0 | 0 | 0 | 0 | 0 | 0 | 0 | 0 | 0 | 0 | 0 | 0 | Viruses | Heunggongvirae | Uroviricota | Caudoviricetes | Viruses;Heunggongvirae;Uroviricota;Caudoviricetes;Crassvirales |
| Mononegavirales | 0 | 0 | 0 | 0 | 0 | 0 | 0 | 0 | 0 | 0 | 0 | 0 | 0 | 0 | Viruses | Orthornavirae | Negarnaviricota | Monjiviricetes | Viruses;Orthornavirae;Negarnaviricota;Monjiviricetes;Mononegavirales |
| Zurhausenvirales | 0 | 0 | 0 | 0 | 0 | 0 | 0 | 1 | 0 | 0 | 0 | 0 | 0 | 0 | Viruses | Shotokuvirae | Cossaviricota | Papovaviricetes | Viruses;Shotokuvirae;Cossaviricota;Papovaviricetes;Zurhausenvirales |
| Ligamenvirales | 0 | 0 | 0 | 0 | 0 | 0 | 0 | 0 | 0 | 0 | 0 | 0 | 0 | 0 | Viruses | Zilligvirae | Taleaviricota | Tokiviricetes | Viruses;Zilligvirae;Taleaviricota;Tokiviricetes;Ligamenvirales |

Export CSV

Rarefied abundance table for the family rank.

All samples have been randomly subsetted to have the same number
of reads.

| family | barcode01 | barcode02 | barcode03 | barcode04 | barcode05 | barcode06 | barcode07 | barcode08 | barcode09 | barcode10 | barcode11 | barcode12 | total | unclassified | superkingdom | kingdom | phylum | class | order | tax |
| --- | --- | --- | --- | --- | --- | --- | --- | --- | --- | --- | --- | --- | --- | --- | --- | --- | --- | --- | --- | --- |
| Haladaptataceae | 0 | 1 | 0 | 0 | 0 | 0 | 0 | 0 | 0 | 0 | 0 | 0 | 0 | 0 | Archaea | Archaea\_none | Euryarchaeota | Halobacteria | Halobacteriales | Archaea;Archaea\_none;Euryarchaeota;Halobacteria;Halobacteriales;Haladaptataceae |
| Haloarculaceae | 0 | 0 | 0 | 0 | 0 | 0 | 0 | 0 | 0 | 0 | 0 | 0 | 0 | 0 | Archaea | Archaea\_none | Euryarchaeota | Halobacteria | Halobacteriales | Archaea;Archaea\_none;Euryarchaeota;Halobacteria;Halobacteriales;Haloarculaceae |
| Halobacteriaceae | 1 | 0 | 0 | 0 | 0 | 0 | 0 | 0 | 0 | 0 | 0 | 0 | 0 | 0 | Archaea | Archaea\_none | Euryarchaeota | Halobacteria | Halobacteriales | Archaea;Archaea\_none;Euryarchaeota;Halobacteria;Halobacteriales;Halobacteriaceae |
| Haloferacaceae | 0 | 0 | 0 | 0 | 0 | 1 | 1 | 0 | 0 | 0 | 0 | 0 | 0 | 0 | Archaea | Archaea\_none | Euryarchaeota | Halobacteria | Halobacteriales | Archaea;Archaea\_none;Euryarchaeota;Halobacteria;Halobacteriales;Haloferacaceae |
| Methanobacteriaceae | 0 | 0 | 2 | 0 | 0 | 0 | 1 | 0 | 0 | 0 | 0 | 0 | 0 | 0 | Archaea | Archaea\_none | Euryarchaeota | Methanobacteria | Methanobacteriales | Archaea;Archaea\_none;Euryarchaeota;Methanobacteria;Methanobacteriales;Methanobacteriaceae |
| Methanocaldococcaceae | 0 | 0 | 1 | 0 | 0 | 0 | 0 | 0 | 0 | 0 | 0 | 0 | 0 | 0 | Archaea | Archaea\_none | Euryarchaeota | Methanococci | Methanococcales | Archaea;Archaea\_none;Euryarchaeota;Methanococci;Methanococcales;Methanocaldococcaceae |
| Methanoregulaceae | 0 | 1 | 0 | 0 | 0 | 0 | 0 | 0 | 0 | 0 | 0 | 0 | 0 | 0 | Archaea | Archaea\_none | Euryarchaeota | Methanomicrobia | Methanomicrobiales | Archaea;Archaea\_none;Euryarchaeota;Methanomicrobia;Methanomicrobiales;Methanoregulaceae |
| Methanosarcinaceae | 0 | 0 | 0 | 0 | 0 | 0 | 0 | 0 | 0 | 0 | 1 | 0 | 0 | 0 | Archaea | Archaea\_none | Euryarchaeota | Methanomicrobia | Methanosarcinales | Archaea;Archaea\_none;Euryarchaeota;Methanomicrobia;Methanosarcinales;Methanosarcinaceae |
| Thermococcaceae | 0 | 0 | 0 | 0 | 0 | 0 | 0 | 0 | 0 | 0 | 0 | 0 | 0 | 0 | Archaea | Archaea\_none | Euryarchaeota | Thermococci | Thermococcales | Archaea;Archaea\_none;Euryarchaeota;Thermococci;Thermococcales;Thermococcaceae |
| Nitrososphaerota\_Incertae\_sedis | 0 | 0 | 0 | 0 | 0 | 1 | 0 | 0 | 0 | 0 | 0 | 0 | 0 | 0 | Archaea | Archaea\_none | Nitrososphaerota | Nitrososphaerota\_Incertae\_sedis | Nitrososphaerota\_Incertae\_sedis | Archaea;Archaea\_none;Nitrososphaerota;Nitrososphaerota\_Incertae\_sedis;Nitrososphaerota\_Incertae\_sedis;Nitrososphaerota\_Incertae\_sedis |
| Bryobacteraceae | 0 | 0 | 0 | 0 | 0 | 0 | 0 | 0 | 0 | 0 | 0 | 0 | 0 | 0 | Bacteria | Bacteria\_none | Acidobacteriota | Terriglobia | Bryobacterales | Bacteria;Bacteria\_none;Acidobacteriota;Terriglobia;Bryobacterales;Bryobacteraceae |
| Iamiaceae | 0 | 0 | 0 | 0 | 0 | 0 | 0 | 0 | 0 | 0 | 0 | 0 | 0 | 0 | Bacteria | Bacteria\_none | Actinomycetota | Acidimicrobiia | Acidimicrobiales | Bacteria;Bacteria\_none;Actinomycetota;Acidimicrobiia;Acidimicrobiales;Iamiaceae |
| Actinomycetaceae | 1 | 17 | 1 | 2 | 3 | 30 | 11 | 2 | 3 | 0 | 0 | 5 | 7 | 2 | Bacteria | Bacteria\_none | Actinomycetota | Actinomycetes | Actinomycetales | Bacteria;Bacteria\_none;Actinomycetota;Actinomycetes;Actinomycetales;Actinomycetaceae |
| Bifidobacteriaceae | 0 | 1 | 2 | 0 | 1 | 0 | 1 | 0 | 0 | 0 | 0 | 0 | 0 | 1 | Bacteria | Bacteria\_none | Actinomycetota | Actinomycetes | Bifidobacteriales | Bacteria;Bacteria\_none;Actinomycetota;Actinomycetes;Bifidobacteriales;Bifidobacteriaceae |
| Geodermatophilaceae | 0 | 0 | 0 | 0 | 1 | 0 | 0 | 0 | 0 | 0 | 0 | 0 | 0 | 0 | Bacteria | Bacteria\_none | Actinomycetota | Actinomycetes | Geodermatophilales | Bacteria;Bacteria\_none;Actinomycetota;Actinomycetes;Geodermatophilales;Geodermatophilaceae |
| Streptomycetaceae | 0 | 2 | 0 | 1 | 2 | 6 | 1 | 0 | 0 | 0 | 1 | 1 | 1 | 0 | Bacteria | Bacteria\_none | Actinomycetota | Actinomycetes | Kitasatosporales | Bacteria;Bacteria\_none;Actinomycetota;Actinomycetes;Kitasatosporales;Streptomycetaceae |
| Cellulomonadaceae | 0 | 0 | 0 | 0 | 0 | 0 | 0 | 0 | 0 | 0 | 0 | 0 | 0 | 0 | Bacteria | Bacteria\_none | Actinomycetota | Actinomycetes | Micrococcales | Bacteria;Bacteria\_none;Actinomycetota;Actinomycetes;Micrococcales;Cellulomonadaceae |
| Dermatophilaceae | 0 | 0 | 0 | 0 | 0 | 0 | 0 | 0 | 0 | 0 | 0 | 0 | 0 | 0 | Bacteria | Bacteria\_none | Actinomycetota | Actinomycetes | Micrococcales | Bacteria;Bacteria\_none;Actinomycetota;Actinomycetes;Micrococcales;Dermatophilaceae |
| Microbacteriaceae | 1 | 0 | 0 | 0 | 2 | 0 | 0 | 0 | 0 | 0 | 0 | 0 | 1 | 0 | Bacteria | Bacteria\_none | Actinomycetota | Actinomycetes | Micrococcales | Bacteria;Bacteria\_none;Actinomycetota;Actinomycetes;Micrococcales;Microbacteriaceae |
| Micrococcaceae | 23 | 105 | 18 | 4 | 71 | 30 | 8 | 12 | 3 | 37 | 62 | 76 | 35 | 17 | Bacteria | Bacteria\_none | Actinomycetota | Actinomycetes | Micrococcales | Bacteria;Bacteria\_none;Actinomycetota;Actinomycetes;Micrococcales;Micrococcaceae |
| Micrococcales\_Incertae\_sedis | 0 | 0 | 0 | 0 | 0 | 0 | 0 | 0 | 0 | 0 | 0 | 0 | 0 | 0 | Bacteria | Bacteria\_none | Actinomycetota | Actinomycetes | Micrococcales | Bacteria;Bacteria\_none;Actinomycetota;Actinomycetes;Micrococcales;Micrococcales\_Incertae\_sedis |
| Ruaniaceae | 0 | 0 | 0 | 0 | 0 | 0 | 0 | 0 | 0 | 0 | 0 | 0 | 0 | 0 | Bacteria | Bacteria\_none | Actinomycetota | Actinomycetes | Micrococcales | Bacteria;Bacteria\_none;Actinomycetota;Actinomycetes;Micrococcales;Ruaniaceae |
| Micromonosporaceae | 1 | 0 | 0 | 0 | 0 | 1 | 0 | 0 | 0 | 2 | 0 | 0 | 0 | 0 | Bacteria | Bacteria\_none | Actinomycetota | Actinomycetes | Micromonosporales | Bacteria;Bacteria\_none;Actinomycetota;Actinomycetes;Micromonosporales;Micromonosporaceae |
| Corynebacteriaceae | 0 | 6 | 0 | 0 | 1 | 1 | 0 | 0 | 0 | 10 | 0 | 1 | 1 | 2 | Bacteria | Bacteria\_none | Actinomycetota | Actinomycetes | Mycobacteriales | Bacteria;Bacteria\_none;Actinomycetota;Actinomycetes;Mycobacteriales;Corynebacteriaceae |
| Gordoniaceae | 0 | 0 | 0 | 0 | 1 | 0 | 0 | 0 | 0 | 0 | 0 | 0 | 0 | 0 | Bacteria | Bacteria\_none | Actinomycetota | Actinomycetes | Mycobacteriales | Bacteria;Bacteria\_none;Actinomycetota;Actinomycetes;Mycobacteriales;Gordoniaceae |
| Mycobacteriaceae | 0 | 1 | 2 | 0 | 0 | 2 | 1 | 0 | 0 | 0 | 0 | 0 | 1 | 0 | Bacteria | Bacteria\_none | Actinomycetota | Actinomycetes | Mycobacteriales | Bacteria;Bacteria\_none;Actinomycetota;Actinomycetes;Mycobacteriales;Mycobacteriaceae |
| Nocardiaceae | 0 | 0 | 0 | 0 | 0 | 0 | 0 | 0 | 0 | 0 | 0 | 0 | 0 | 0 | Bacteria | Bacteria\_none | Actinomycetota | Actinomycetes | Mycobacteriales | Bacteria;Bacteria\_none;Actinomycetota;Actinomycetes;Mycobacteriales;Nocardiaceae |
| Kribbellaceae | 0 | 0 | 0 | 0 | 0 | 0 | 0 | 0 | 0 | 0 | 1 | 0 | 0 | 0 | Bacteria | Bacteria\_none | Actinomycetota | Actinomycetes | Propionibacteriales | Bacteria;Bacteria\_none;Actinomycetota;Actinomycetes;Propionibacteriales;Kribbellaceae |
| Nocardioidaceae | 0 | 0 | 1 | 0 | 1 | 0 | 0 | 0 | 0 | 0 | 0 | 0 | 0 | 0 | Bacteria | Bacteria\_none | Actinomycetota | Actinomycetes | Propionibacteriales | Bacteria;Bacteria\_none;Actinomycetota;Actinomycetes;Propionibacteriales;Nocardioidaceae |
| Propionibacteriaceae | 0 | 1 | 0 | 1 | 1 | 2 | 0 | 0 | 0 | 1 | 20 | 0 | 2 | 4 | Bacteria | Bacteria\_none | Actinomycetota | Actinomycetes | Propionibacteriales | Bacteria;Bacteria\_none;Actinomycetota;Actinomycetes;Propionibacteriales;Propionibacteriaceae |
| Pseudonocardiaceae | 0 | 0 | 0 | 0 | 1 | 1 | 0 | 0 | 0 | 0 | 0 | 0 | 0 | 0 | Bacteria | Bacteria\_none | Actinomycetota | Actinomycetes | Pseudonocardiales | Bacteria;Bacteria\_none;Actinomycetota;Actinomycetes;Pseudonocardiales;Pseudonocardiaceae |
| Streptosporangiaceae | 0 | 0 | 1 | 0 | 0 | 1 | 1 | 0 | 0 | 0 | 0 | 0 | 1 | 0 | Bacteria | Bacteria\_none | Actinomycetota | Actinomycetes | Streptosporangiales | Bacteria;Bacteria\_none;Actinomycetota;Actinomycetes;Streptosporangiales;Streptosporangiaceae |
| Thermomonosporaceae | 0 | 0 | 0 | 1 | 0 | 0 | 0 | 0 | 0 | 0 | 0 | 0 | 0 | 0 | Bacteria | Bacteria\_none | Actinomycetota | Actinomycetes | Streptosporangiales | Bacteria;Bacteria\_none;Actinomycetota;Actinomycetes;Streptosporangiales;Thermomonosporaceae |
| Atopobiaceae | 27 | 4 | 10 | 0 | 1 | 101 | 18 | 1 | 0 | 0 | 5 | 1 | 15 | 4 | Bacteria | Bacteria\_none | Actinomycetota | Coriobacteriia | Coriobacteriales | Bacteria;Bacteria\_none;Actinomycetota;Coriobacteriia;Coriobacteriales;Atopobiaceae |
| Eggerthellaceae | 0 | 0 | 0 | 0 | 0 | 1 | 1 | 0 | 0 | 0 | 0 | 0 | 0 | 0 | Bacteria | Bacteria\_none | Actinomycetota | Coriobacteriia | Eggerthellales | Bacteria;Bacteria\_none;Actinomycetota;Coriobacteriia;Eggerthellales;Eggerthellaceae |
| Rubrobacteraceae | 0 | 0 | 0 | 0 | 1 | 0 | 0 | 0 | 0 | 0 | 0 | 0 | 0 | 0 | Bacteria | Bacteria\_none | Actinomycetota | Rubrobacteria | Rubrobacterales | Bacteria;Bacteria\_none;Actinomycetota;Rubrobacteria;Rubrobacterales;Rubrobacteraceae |
| Miltoncostaeaceae | 0 | 1 | 0 | 0 | 0 | 0 | 0 | 0 | 0 | 0 | 0 | 0 | 0 | 0 | Bacteria | Bacteria\_none | Actinomycetota | Thermoleophilia | Miltoncostaeales | Bacteria;Bacteria\_none;Actinomycetota;Thermoleophilia;Miltoncostaeales;Miltoncostaeaceae |
| Baekduiaceae | 0 | 1 | 0 | 0 | 0 | 0 | 0 | 0 | 0 | 0 | 0 | 0 | 0 | 0 | Bacteria | Bacteria\_none | Actinomycetota | Thermoleophilia | Solirubrobacterales | Bacteria;Bacteria\_none;Actinomycetota;Thermoleophilia;Solirubrobacterales;Baekduiaceae |
| Abyssicoccaceae | 0 | 0 | 0 | 0 | 1 | 0 | 0 | 0 | 0 | 0 | 0 | 0 | 0 | 0 | Bacteria | Bacteria\_none | Bacillota | Bacilli | Bacillales | Bacteria;Bacteria\_none;Bacillota;Bacilli;Bacillales;Abyssicoccaceae |
| Alicyclobacillaceae | 0 | 0 | 0 | 0 | 0 | 0 | 0 | 0 | 0 | 0 | 0 | 0 | 0 | 0 | Bacteria | Bacteria\_none | Bacillota | Bacilli | Bacillales | Bacteria;Bacteria\_none;Bacillota;Bacilli;Bacillales;Alicyclobacillaceae |
| Bacillaceae | 3 | 2 | 6 | 5 | 4 | 9 | 0 | 1 | 0 | 2 | 0 | 0 | 2 | 2 | Bacteria | Bacteria\_none | Bacillota | Bacilli | Bacillales | Bacteria;Bacteria\_none;Bacillota;Bacilli;Bacillales;Bacillaceae |
| Bacillales\_Incertae\_sedis | 0 | 0 | 0 | 0 | 0 | 0 | 0 | 0 | 0 | 0 | 0 | 0 | 0 | 0 | Bacteria | Bacteria\_none | Bacillota | Bacilli | Bacillales | Bacteria;Bacteria\_none;Bacillota;Bacilli;Bacillales;Bacillales\_Incertae\_sedis |
| Gemellaceae | 66 | 117 | 6 | 68 | 318 | 254 | 44 | 33 | 3 | 211 | 516 | 41 | 113 | 72 | Bacteria | Bacteria\_none | Bacillota | Bacilli | Bacillales | Bacteria;Bacteria\_none;Bacillota;Bacilli;Bacillales;Gemellaceae |
| Listeriaceae | 0 | 0 | 0 | 0 | 0 | 0 | 0 | 0 | 0 | 0 | 0 | 0 | 0 | 0 | Bacteria | Bacteria\_none | Bacillota | Bacilli | Bacillales | Bacteria;Bacteria\_none;Bacillota;Bacilli;Bacillales;Listeriaceae |
| Paenibacillaceae | 1 | 1 | 2 | 2 | 0 | 2 | 2 | 0 | 0 | 0 | 1 | 0 | 1 | 0 | Bacteria | Bacteria\_none | Bacillota | Bacilli | Bacillales | Bacteria;Bacteria\_none;Bacillota;Bacilli;Bacillales;Paenibacillaceae |
| Planococcaceae | 1 | 0 | 0 | 0 | 0 | 0 | 0 | 0 | 0 | 0 | 0 | 0 | 0 | 0 | Bacteria | Bacteria\_none | Bacillota | Bacilli | Bacillales | Bacteria;Bacteria\_none;Bacillota;Bacilli;Bacillales;Planococcaceae |
| Staphylococcaceae | 1 | 3 | 3 | 2 | 1 | 0 | 0 | 0 | 0 | 1 | 0 | 1 | 0 | 0 | Bacteria | Bacteria\_none | Bacillota | Bacilli | Bacillales | Bacteria;Bacteria\_none;Bacillota;Bacilli;Bacillales;Staphylococcaceae |
| Aerococcaceae | 6 | 1 | 57 | 3 | 49 | 14 | 7 | 0 | 0 | 14 | 40 | 1 | 4 | 6 | Bacteria | Bacteria\_none | Bacillota | Bacilli | Lactobacillales | Bacteria;Bacteria\_none;Bacillota;Bacilli;Lactobacillales;Aerococcaceae |
| Carnobacteriaceae | 381 | 59 | 383 | 86 | 131 | 98 | 178 | 5 | 0 | 106 | 66 | 10 | 66 | 34 | Bacteria | Bacteria\_none | Bacillota | Bacilli | Lactobacillales | Bacteria;Bacteria\_none;Bacillota;Bacilli;Lactobacillales;Carnobacteriaceae |
| Enterococcaceae | 2 | 9 | 10 | 1 | 2 | 4 | 2 | 0 | 0 | 0 | 1 | 0 | 1 | 0 | Bacteria | Bacteria\_none | Bacillota | Bacilli | Lactobacillales | Bacteria;Bacteria\_none;Bacillota;Bacilli;Lactobacillales;Enterococcaceae |
| Lactobacillaceae | 2 | 0 | 2 | 1 | 2 | 1 | 10 | 1 | 1 | 3 | 3 | 0 | 1 | 3 | Bacteria | Bacteria\_none | Bacillota | Bacilli | Lactobacillales | Bacteria;Bacteria\_none;Bacillota;Bacilli;Lactobacillales;Lactobacillaceae |
| Streptococcaceae | 1614 | 1304 | 649 | 225 | 1433 | 1313 | 735 | 109 | 204 | 532 | 1231 | 420 | 581 | 370 | Bacteria | Bacteria\_none | Bacillota | Bacilli | Lactobacillales | Bacteria;Bacteria\_none;Bacillota;Bacilli;Lactobacillales;Streptococcaceae |
| Caldicellulosiruptoraceae | 0 | 0 | 0 | 0 | 0 | 0 | 0 | 0 | 0 | 0 | 0 | 0 | 0 | 0 | Bacteria | Bacteria\_none | Bacillota | Bacillota\_Incertae\_sedis | Caldicellulosiruptorales | Bacteria;Bacteria\_none;Bacillota;Bacillota\_Incertae\_sedis;Caldicellulosiruptorales;Caldicellulosiruptoraceae |
| Aristaeellaceae | 0 | 0 | 0 | 0 | 0 | 0 | 0 | 0 | 0 | 0 | 0 | 0 | 0 | 0 | Bacteria | Bacteria\_none | Bacillota | Clostridia | Eubacteriales | Bacteria;Bacteria\_none;Bacillota;Clostridia;Eubacteriales;Aristaeellaceae |
| Clostridiaceae | 1 | 2 | 2 | 2 | 4 | 8 | 0 | 1 | 0 | 0 | 1 | 0 | 1 | 0 | Bacteria | Bacteria\_none | Bacillota | Clostridia | Eubacteriales | Bacteria;Bacteria\_none;Bacillota;Clostridia;Eubacteriales;Clostridiaceae |
| Clostridiales Family XVI. Incertae Sedis | 0 | 0 | 0 | 0 | 0 | 0 | 0 | 0 | 0 | 0 | 0 | 0 | 0 | 0 | Bacteria | Bacteria\_none | Bacillota | Clostridia | Eubacteriales | Bacteria;Bacteria\_none;Bacillota;Clostridia;Eubacteriales;Clostridiales Family XVI. Incertae Sedis |
| Desulfitobacteriaceae | 0 | 0 | 0 | 0 | 0 | 1 | 0 | 0 | 0 | 0 | 0 | 0 | 0 | 0 | Bacteria | Bacteria\_none | Bacillota | Clostridia | Eubacteriales | Bacteria;Bacteria\_none;Bacillota;Clostridia;Eubacteriales;Desulfitobacteriaceae |
| Eubacteriaceae | 0 | 0 | 1 | 0 | 0 | 1 | 0 | 0 | 0 | 0 | 0 | 0 | 0 | 0 | Bacteria | Bacteria\_none | Bacillota | Clostridia | Eubacteriales | Bacteria;Bacteria\_none;Bacillota;Clostridia;Eubacteriales;Eubacteriaceae |
| Eubacteriales Family XIII. Incertae Sedis | 0 | 0 | 0 | 0 | 0 | 0 | 0 | 0 | 0 | 0 | 0 | 0 | 0 | 0 | Bacteria | Bacteria\_none | Bacillota | Clostridia | Eubacteriales | Bacteria;Bacteria\_none;Bacillota;Clostridia;Eubacteriales;Eubacteriales Family XIII. Incertae Sedis |
| Eubacteriales\_Incertae\_sedis | 0 | 0 | 0 | 0 | 0 | 2 | 0 | 0 | 0 | 0 | 2 | 0 | 0 | 0 | Bacteria | Bacteria\_none | Bacillota | Clostridia | Eubacteriales | Bacteria;Bacteria\_none;Bacillota;Clostridia;Eubacteriales;Eubacteriales\_Incertae\_sedis |
| Oscillospiraceae | 3 | 3 | 2 | 1 | 1 | 4 | 1 | 0 | 0 | 1 | 0 | 0 | 1 | 0 | Bacteria | Bacteria\_none | Bacillota | Clostridia | Eubacteriales | Bacteria;Bacteria\_none;Bacillota;Clostridia;Eubacteriales;Oscillospiraceae |
| Halobacteroidaceae | 0 | 0 | 0 | 0 | 0 | 0 | 0 | 0 | 0 | 0 | 0 | 0 | 0 | 0 | Bacteria | Bacteria\_none | Bacillota | Clostridia | Halanaerobiales | Bacteria;Bacteria\_none;Bacillota;Clostridia;Halanaerobiales;Halobacteroidaceae |
| Anaerotignaceae | 0 | 0 | 0 | 0 | 0 | 0 | 0 | 0 | 0 | 0 | 0 | 0 | 0 | 0 | Bacteria | Bacteria\_none | Bacillota | Clostridia | Lachnospirales | Bacteria;Bacteria\_none;Bacillota;Clostridia;Lachnospirales;Anaerotignaceae |
| Lachnospiraceae | 0 | 34 | 27 | 10 | 8 | 45 | 3 | 0 | 1 | 8 | 3 | 2 | 1 | 2 | Bacteria | Bacteria\_none | Bacillota | Clostridia | Lachnospirales | Bacteria;Bacteria\_none;Bacillota;Clostridia;Lachnospirales;Lachnospiraceae |
| Monoglobaceae | 0 | 0 | 0 | 0 | 0 | 0 | 0 | 0 | 0 | 0 | 0 | 0 | 0 | 0 | Bacteria | Bacteria\_none | Bacillota | Clostridia | Monoglobales | Bacteria;Bacteria\_none;Bacillota;Clostridia;Monoglobales;Monoglobaceae |
| Natranaerobiaceae | 0 | 0 | 0 | 1 | 0 | 0 | 0 | 0 | 0 | 0 | 0 | 0 | 0 | 0 | Bacteria | Bacteria\_none | Bacillota | Clostridia | Natranaerobiales | Bacteria;Bacteria\_none;Bacillota;Clostridia;Natranaerobiales;Natranaerobiaceae |
| Natranaerofabaceae | 0 | 0 | 0 | 0 | 0 | 1 | 0 | 0 | 0 | 0 | 0 | 0 | 0 | 0 | Bacteria | Bacteria\_none | Bacillota | Clostridia | Natranaerobiales | Bacteria;Bacteria\_none;Bacillota;Clostridia;Natranaerobiales;Natranaerofabaceae |
| Anaerovoracaceae | 1 | 0 | 7 | 1 | 1 | 29 | 0 | 1 | 0 | 0 | 1 | 2 | 5 | 1 | Bacteria | Bacteria\_none | Bacillota | Clostridia | Peptostreptococcales | Bacteria;Bacteria\_none;Bacillota;Clostridia;Peptostreptococcales;Anaerovoracaceae |
| Filifactoraceae | 2 | 36 | 34 | 0 | 2 | 1 | 1 | 14 | 0 | 0 | 0 | 11 | 8 | 4 | Bacteria | Bacteria\_none | Bacillota | Clostridia | Peptostreptococcales | Bacteria;Bacteria\_none;Bacillota;Clostridia;Peptostreptococcales;Filifactoraceae |
| Natronincolaceae | 0 | 0 | 0 | 1 | 0 | 0 | 0 | 0 | 0 | 0 | 0 | 0 | 0 | 0 | Bacteria | Bacteria\_none | Bacillota | Clostridia | Peptostreptococcales | Bacteria;Bacteria\_none;Bacillota;Clostridia;Peptostreptococcales;Natronincolaceae |
| Peptostreptococcaceae | 1 | 1 | 3 | 0 | 3 | 0 | 1 | 0 | 1 | 0 | 4 | 0 | 0 | 0 | Bacteria | Bacteria\_none | Bacillota | Clostridia | Peptostreptococcales | Bacteria;Bacteria\_none;Bacillota;Clostridia;Peptostreptococcales;Peptostreptococcaceae |
| Thermoanaerobacteraceae | 0 | 1 | 0 | 0 | 0 | 0 | 0 | 0 | 0 | 0 | 0 | 0 | 0 | 0 | Bacteria | Bacteria\_none | Bacillota | Clostridia | Thermoanaerobacterales | Bacteria;Bacteria\_none;Bacillota;Clostridia;Thermoanaerobacterales;Thermoanaerobacteraceae |
| Coprobacillaceae | 0 | 0 | 0 | 1 | 0 | 1 | 2 | 0 | 0 | 0 | 0 | 0 | 0 | 0 | Bacteria | Bacteria\_none | Bacillota | Erysipelotrichia | Erysipelotrichales | Bacteria;Bacteria\_none;Bacillota;Erysipelotrichia;Erysipelotrichales;Coprobacillaceae |
| Erysipelotrichaceae | 14 | 16 | 75 | 5 | 11 | 396 | 19 | 3 | 0 | 3 | 3 | 0 | 16 | 8 | Bacteria | Bacteria\_none | Bacillota | Erysipelotrichia | Erysipelotrichales | Bacteria;Bacteria\_none;Bacillota;Erysipelotrichia;Erysipelotrichales;Erysipelotrichaceae |
| Selenomonadaceae | 4 | 7 | 12 | 3 | 3 | 14 | 2 | 2 | 0 | 2 | 2 | 6 | 2 | 1 | Bacteria | Bacteria\_none | Bacillota | Negativicutes | Selenomonadales | Bacteria;Bacteria\_none;Bacillota;Negativicutes;Selenomonadales;Selenomonadaceae |
| Sporomusaceae | 0 | 0 | 0 | 0 | 0 | 0 | 0 | 0 | 0 | 0 | 0 | 0 | 0 | 0 | Bacteria | Bacteria\_none | Bacillota | Negativicutes | Selenomonadales | Bacteria;Bacteria\_none;Bacillota;Negativicutes;Selenomonadales;Sporomusaceae |
| Veillonellaceae | 1047 | 70 | 37 | 57 | 45 | 42 | 8 | 18 | 27 | 76 | 4 | 41 | 64 | 39 | Bacteria | Bacteria\_none | Bacillota | Negativicutes | Veillonellales | Bacteria;Bacteria\_none;Bacillota;Negativicutes;Veillonellales;Veillonellaceae |
| Acidilutibacteraceae | 0 | 0 | 0 | 0 | 1 | 0 | 0 | 0 | 0 | 0 | 0 | 0 | 0 | 0 | Bacteria | Bacteria\_none | Bacillota | Tissierellia | Tissierellales | Bacteria;Bacteria\_none;Bacillota;Tissierellia;Tissierellales;Acidilutibacteraceae |
| Gottschalkiaceae | 0 | 0 | 0 | 0 | 0 | 0 | 0 | 0 | 0 | 0 | 0 | 0 | 0 | 0 | Bacteria | Bacteria\_none | Bacillota | Tissierellia | Tissierellales | Bacteria;Bacteria\_none;Bacillota;Tissierellia;Tissierellales;Gottschalkiaceae |
| Peptoniphilaceae | 5 | 34 | 22 | 7 | 29 | 49 | 2 | 10 | 0 | 1 | 29 | 10 | 17 | 12 | Bacteria | Bacteria\_none | Bacillota | Tissierellia | Tissierellales | Bacteria;Bacteria\_none;Bacillota;Tissierellia;Tissierellales;Peptoniphilaceae |
| Tepidimicrobiaceae | 0 | 0 | 0 | 0 | 1 | 0 | 0 | 0 | 0 | 0 | 0 | 0 | 0 | 0 | Bacteria | Bacteria\_none | Bacillota | Tissierellia | Tissierellales | Bacteria;Bacteria\_none;Bacillota;Tissierellia;Tissierellales;Tepidimicrobiaceae |
| Tissierellia\_Incertae\_sedis | 0 | 0 | 0 | 0 | 1 | 0 | 0 | 0 | 0 | 0 | 4 | 0 | 0 | 0 | Bacteria | Bacteria\_none | Bacillota | Tissierellia | Tissierellia\_Incertae\_sedis | Bacteria;Bacteria\_none;Bacillota;Tissierellia;Tissierellia\_Incertae\_sedis;Tissierellia\_Incertae\_sedis |
| Bacteroidaceae | 1 | 2 | 1 | 7 | 1 | 5 | 0 | 1 | 0 | 1 | 2 | 0 | 3 | 0 | Bacteria | Bacteria\_none | Bacteroidota | Bacteroidia | Bacteroidales | Bacteria;Bacteria\_none;Bacteroidota;Bacteroidia;Bacteroidales;Bacteroidaceae |
| Barnesiellaceae | 0 | 0 | 0 | 0 | 0 | 1 | 0 | 0 | 0 | 0 | 0 | 0 | 0 | 0 | Bacteria | Bacteria\_none | Bacteroidota | Bacteroidia | Bacteroidales | Bacteria;Bacteria\_none;Bacteroidota;Bacteroidia;Bacteroidales;Barnesiellaceae |
| Dysgonomonadaceae | 0 | 0 | 0 | 0 | 0 | 0 | 0 | 0 | 0 | 0 | 0 | 1 | 0 | 0 | Bacteria | Bacteria\_none | Bacteroidota | Bacteroidia | Bacteroidales | Bacteria;Bacteria\_none;Bacteroidota;Bacteroidia;Bacteroidales;Dysgonomonadaceae |
| Muribaculaceae | 0 | 0 | 0 | 0 | 1 | 0 | 0 | 0 | 0 | 0 | 0 | 0 | 0 | 0 | Bacteria | Bacteria\_none | Bacteroidota | Bacteroidia | Bacteroidales | Bacteria;Bacteria\_none;Bacteroidota;Bacteroidia;Bacteroidales;Muribaculaceae |
| Odoribacteraceae | 0 | 0 | 0 | 0 | 0 | 0 | 0 | 0 | 0 | 0 | 0 | 0 | 0 | 0 | Bacteria | Bacteria\_none | Bacteroidota | Bacteroidia | Bacteroidales | Bacteria;Bacteria\_none;Bacteroidota;Bacteroidia;Bacteroidales;Odoribacteraceae |
| Porphyromonadaceae | 5 | 123 | 7 | 26 | 35 | 23 | 6 | 24 | 0 | 25 | 46 | 22 | 16 | 6 | Bacteria | Bacteria\_none | Bacteroidota | Bacteroidia | Bacteroidales | Bacteria;Bacteria\_none;Bacteroidota;Bacteroidia;Bacteroidales;Porphyromonadaceae |
| Prevotellaceae | 298 | 927 | 256 | 494 | 41 | 169 | 66 | 20 | 137 | 75 | 108 | 132 | 156 | 95 | Bacteria | Bacteria\_none | Bacteroidota | Bacteroidia | Bacteroidales | Bacteria;Bacteria\_none;Bacteroidota;Bacteroidia;Bacteroidales;Prevotellaceae |
| Rikenellaceae | 0 | 2 | 0 | 0 | 0 | 2 | 0 | 0 | 0 | 0 | 0 | 0 | 0 | 0 | Bacteria | Bacteria\_none | Bacteroidota | Bacteroidia | Bacteroidales | Bacteria;Bacteria\_none;Bacteroidota;Bacteroidia;Bacteroidales;Rikenellaceae |
| Salinivirgaceae | 0 | 0 | 0 | 1 | 0 | 0 | 0 | 0 | 0 | 0 | 0 | 0 | 0 | 0 | Bacteria | Bacteria\_none | Bacteroidota | Bacteroidia | Bacteroidales | Bacteria;Bacteria\_none;Bacteroidota;Bacteroidia;Bacteroidales;Salinivirgaceae |
| Tannerellaceae | 1 | 34 | 31 | 5 | 8 | 74 | 2 | 8 | 0 | 21 | 22 | 13 | 11 | 10 | Bacteria | Bacteria\_none | Bacteroidota | Bacteroidia | Bacteroidales | Bacteria;Bacteria\_none;Bacteroidota;Bacteroidia;Bacteroidales;Tannerellaceae |
| Marinifilaceae | 1 | 0 | 1 | 0 | 0 | 0 | 0 | 0 | 0 | 0 | 0 | 0 | 0 | 0 | Bacteria | Bacteria\_none | Bacteroidota | Bacteroidia | Marinilabiliales | Bacteria;Bacteria\_none;Bacteroidota;Bacteroidia;Marinilabiliales;Marinifilaceae |
| Marinilabiliaceae | 0 | 0 | 1 | 1 | 0 | 0 | 0 | 2 | 0 | 0 | 0 | 0 | 0 | 0 | Bacteria | Bacteria\_none | Bacteroidota | Bacteroidia | Marinilabiliales | Bacteria;Bacteria\_none;Bacteroidota;Bacteroidia;Marinilabiliales;Marinilabiliaceae |
| Prolixibacteraceae | 0 | 0 | 0 | 1 | 0 | 0 | 0 | 0 | 0 | 0 | 1 | 0 | 0 | 0 | Bacteria | Bacteria\_none | Bacteroidota | Bacteroidia | Marinilabiliales | Bacteria;Bacteria\_none;Bacteroidota;Bacteroidia;Marinilabiliales;Prolixibacteraceae |
| Chitinophagaceae | 0 | 1 | 0 | 1 | 0 | 0 | 0 | 0 | 0 | 0 | 0 | 0 | 0 | 0 | Bacteria | Bacteria\_none | Bacteroidota | Chitinophagia | Chitinophagales | Bacteria;Bacteria\_none;Bacteroidota;Chitinophagia;Chitinophagales;Chitinophagaceae |
| Amoebophilaceae | 0 | 0 | 0 | 0 | 0 | 0 | 0 | 0 | 0 | 0 | 0 | 0 | 0 | 0 | Bacteria | Bacteria\_none | Bacteroidota | Cytophagia | Cytophagales | Bacteria;Bacteria\_none;Bacteroidota;Cytophagia;Cytophagales;Amoebophilaceae |
| Cyclobacteriaceae | 0 | 0 | 0 | 0 | 0 | 0 | 0 | 0 | 0 | 0 | 1 | 0 | 1 | 0 | Bacteria | Bacteria\_none | Bacteroidota | Cytophagia | Cytophagales | Bacteria;Bacteria\_none;Bacteroidota;Cytophagia;Cytophagales;Cyclobacteriaceae |
| Cytophagaceae | 0 | 0 | 0 | 0 | 0 | 0 | 0 | 0 | 0 | 0 | 0 | 0 | 0 | 0 | Bacteria | Bacteria\_none | Bacteroidota | Cytophagia | Cytophagales | Bacteria;Bacteria\_none;Bacteroidota;Cytophagia;Cytophagales;Cytophagaceae |
| Flammeovirgaceae | 0 | 0 | 0 | 0 | 2 | 0 | 0 | 0 | 0 | 0 | 0 | 0 | 0 | 0 | Bacteria | Bacteria\_none | Bacteroidota | Cytophagia | Cytophagales | Bacteria;Bacteria\_none;Bacteroidota;Cytophagia;Cytophagales;Flammeovirgaceae |
| Hymenobacteraceae | 0 | 0 | 0 | 0 | 2 | 0 | 1 | 0 | 0 | 0 | 0 | 0 | 1 | 0 | Bacteria | Bacteria\_none | Bacteroidota | Cytophagia | Cytophagales | Bacteria;Bacteria\_none;Bacteroidota;Cytophagia;Cytophagales;Hymenobacteraceae |
| Leadbetterellaceae | 1 | 0 | 1 | 0 | 0 | 0 | 0 | 0 | 0 | 0 | 0 | 0 | 0 | 0 | Bacteria | Bacteria\_none | Bacteroidota | Cytophagia | Cytophagales | Bacteria;Bacteria\_none;Bacteroidota;Cytophagia;Cytophagales;Leadbetterellaceae |
| Marivirgaceae | 0 | 0 | 0 | 0 | 0 | 1 | 0 | 0 | 0 | 0 | 0 | 0 | 0 | 0 | Bacteria | Bacteria\_none | Bacteroidota | Cytophagia | Cytophagales | Bacteria;Bacteria\_none;Bacteroidota;Cytophagia;Cytophagales;Marivirgaceae |
| Persicobacteraceae | 0 | 0 | 0 | 0 | 0 | 0 | 0 | 0 | 0 | 0 | 0 | 0 | 0 | 0 | Bacteria | Bacteria\_none | Bacteroidota | Cytophagia | Cytophagales | Bacteria;Bacteria\_none;Bacteroidota;Cytophagia;Cytophagales;Persicobacteraceae |
| Spirosomataceae | 0 | 0 | 0 | 0 | 0 | 0 | 0 | 0 | 0 | 0 | 0 | 0 | 0 | 0 | Bacteria | Bacteria\_none | Bacteroidota | Cytophagia | Cytophagales | Bacteria;Bacteria\_none;Bacteroidota;Cytophagia;Cytophagales;Spirosomataceae |
| Blattabacteriaceae | 0 | 0 | 0 | 0 | 0 | 0 | 0 | 0 | 0 | 0 | 0 | 0 | 0 | 0 | Bacteria | Bacteria\_none | Bacteroidota | Flavobacteriia | Flavobacteriales | Bacteria;Bacteria\_none;Bacteroidota;Flavobacteriia;Flavobacteriales;Blattabacteriaceae |
| Crocinitomicaceae | 0 | 0 | 0 | 0 | 0 | 0 | 0 | 0 | 0 | 0 | 0 | 0 | 0 | 0 | Bacteria | Bacteria\_none | Bacteroidota | Flavobacteriia | Flavobacteriales | Bacteria;Bacteria\_none;Bacteroidota;Flavobacteriia;Flavobacteriales;Crocinitomicaceae |
| Flavobacteriaceae | 266 | 145 | 115 | 105 | 97 | 28 | 23 | 93 | 0 | 204 | 76 | 22 | 71 | 45 | Bacteria | Bacteria\_none | Bacteroidota | Flavobacteriia | Flavobacteriales | Bacteria;Bacteria\_none;Bacteroidota;Flavobacteriia;Flavobacteriales;Flavobacteriaceae |
| Owenweeksiaceae | 0 | 1 | 0 | 0 | 1 | 0 | 0 | 0 | 0 | 0 | 0 | 0 | 0 | 0 | Bacteria | Bacteria\_none | Bacteroidota | Flavobacteriia | Flavobacteriales | Bacteria;Bacteria\_none;Bacteroidota;Flavobacteriia;Flavobacteriales;Owenweeksiaceae |
| Sanyastnellaceae | 0 | 0 | 1 | 0 | 0 | 0 | 0 | 0 | 0 | 0 | 0 | 0 | 0 | 0 | Bacteria | Bacteria\_none | Bacteroidota | Flavobacteriia | Flavobacteriales | Bacteria;Bacteria\_none;Bacteroidota;Flavobacteriia;Flavobacteriales;Sanyastnellaceae |
| Weeksellaceae | 4 | 1 | 4 | 3 | 11 | 5 | 0 | 1 | 0 | 4 | 2 | 1 | 1 | 0 | Bacteria | Bacteria\_none | Bacteroidota | Flavobacteriia | Flavobacteriales | Bacteria;Bacteria\_none;Bacteroidota;Flavobacteriia;Flavobacteriales;Weeksellaceae |
| Haliscomenobacteraceae | 0 | 0 | 0 | 0 | 0 | 0 | 0 | 0 | 0 | 0 | 0 | 0 | 0 | 0 | Bacteria | Bacteria\_none | Bacteroidota | Saprospiria | Saprospirales | Bacteria;Bacteria\_none;Bacteroidota;Saprospiria;Saprospirales;Haliscomenobacteraceae |
| Aurantibacillaceae | 0 | 0 | 0 | 0 | 0 | 0 | 0 | 0 | 0 | 0 | 0 | 0 | 0 | 0 | Bacteria | Bacteria\_none | Bacteroidota | Sphingobacteriia | Sphingobacteriales | Bacteria;Bacteria\_none;Bacteroidota;Sphingobacteriia;Sphingobacteriales;Aurantibacillaceae |
| Sphingobacteriaceae | 0 | 2 | 0 | 1 | 0 | 1 | 0 | 0 | 0 | 1 | 2 | 1 | 1 | 0 | Bacteria | Bacteria\_none | Bacteroidota | Sphingobacteriia | Sphingobacteriales | Bacteria;Bacteria\_none;Bacteroidota;Sphingobacteriia;Sphingobacteriales;Sphingobacteriaceae |
| Cyclonatronaceae | 0 | 0 | 0 | 0 | 0 | 0 | 0 | 0 | 0 | 0 | 0 | 0 | 0 | 0 | Bacteria | Bacteria\_none | Balneolota | Balneolia | Balneolales | Bacteria;Bacteria\_none;Balneolota;Balneolia;Balneolales;Cyclonatronaceae |
| Pseudobdellovibrionaceae | 0 | 0 | 0 | 0 | 0 | 0 | 0 | 0 | 0 | 1 | 0 | 0 | 0 | 0 | Bacteria | Bacteria\_none | Bdellovibrionota | Bdellovibrionia | Bdellovibrionales | Bacteria;Bacteria\_none;Bdellovibrionota;Bdellovibrionia;Bdellovibrionales;Pseudobdellovibrionaceae |
| Calditrichaceae | 0 | 0 | 0 | 0 | 0 | 0 | 0 | 0 | 0 | 0 | 0 | 0 | 0 | 0 | Bacteria | Bacteria\_none | Calditrichota | Calditrichia | Calditrichales | Bacteria;Bacteria\_none;Calditrichota;Calditrichia;Calditrichales;Calditrichaceae |
| Arcobacteraceae | 0 | 0 | 2 | 0 | 0 | 0 | 0 | 0 | 0 | 1 | 0 | 0 | 1 | 0 | Bacteria | Bacteria\_none | Campylobacterota | Epsilonproteobacteria | Campylobacterales | Bacteria;Bacteria\_none;Campylobacterota;Epsilonproteobacteria;Campylobacterales;Arcobacteraceae |
| Campylobacteraceae | 0 | 44 | 12 | 6 | 9 | 35 | 0 | 2 | 5 | 3 | 1 | 3 | 6 | 7 | Bacteria | Bacteria\_none | Campylobacterota | Epsilonproteobacteria | Campylobacterales | Bacteria;Bacteria\_none;Campylobacterota;Epsilonproteobacteria;Campylobacterales;Campylobacteraceae |
| Helicobacteraceae | 0 | 0 | 0 | 0 | 0 | 1 | 0 | 0 | 0 | 0 | 0 | 0 | 0 | 0 | Bacteria | Bacteria\_none | Campylobacterota | Epsilonproteobacteria | Campylobacterales | Bacteria;Bacteria\_none;Campylobacterota;Epsilonproteobacteria;Campylobacterales;Helicobacteraceae |
| Sulfurospirillaceae | 0 | 0 | 0 | 0 | 0 | 0 | 0 | 0 | 0 | 0 | 0 | 0 | 0 | 0 | Bacteria | Bacteria\_none | Campylobacterota | Epsilonproteobacteria | Campylobacterales | Bacteria;Bacteria\_none;Campylobacterota;Epsilonproteobacteria;Campylobacterales;Sulfurospirillaceae |
| Sulfurovaceae | 1 | 0 | 0 | 0 | 0 | 0 | 0 | 0 | 0 | 0 | 0 | 0 | 0 | 0 | Bacteria | Bacteria\_none | Campylobacterota | Epsilonproteobacteria | Campylobacterales | Bacteria;Bacteria\_none;Campylobacterota;Epsilonproteobacteria;Campylobacterales;Sulfurovaceae |
| Candidatus Saccharibacteria\_Incertae\_sedis | 0 | 0 | 0 | 0 | 1 | 17 | 0 | 0 | 0 | 0 | 1 | 1 | 0 | 0 | Bacteria | Bacteria\_none | Candidatus Saccharibacteria | Candidatus Saccharibacteria\_Incertae\_sedis | Candidatus Saccharibacteria\_Incertae\_sedis | Bacteria;Bacteria\_none;Candidatus Saccharibacteria;Candidatus Saccharibacteria\_Incertae\_sedis;Candidatus Saccharibacteria\_Incertae\_sedis;Candidatus Saccharibacteria\_Incertae\_sedis |
| Candidatus Nanosynbacteraceae | 0 | 8 | 5 | 0 | 5 | 126 | 1 | 0 | 1 | 1 | 28 | 1 | 6 | 1 | Bacteria | Bacteria\_none | Candidatus Saccharibacteria | Candidatus Saccharimonadia | Candidatus Nanosynbacterales | Bacteria;Bacteria\_none;Candidatus Saccharibacteria;Candidatus Saccharimonadia;Candidatus Nanosynbacterales;Candidatus Nanosynbacteraceae |
| Candidatus Saccharimonadaceae | 0 | 0 | 0 | 0 | 0 | 0 | 0 | 1 | 0 | 0 | 0 | 0 | 0 | 0 | Bacteria | Bacteria\_none | Candidatus Saccharibacteria | Candidatus Saccharimonadia | Candidatus Saccharimonadales | Bacteria;Bacteria\_none;Candidatus Saccharibacteria;Candidatus Saccharimonadia;Candidatus Saccharimonadales;Candidatus Saccharimonadaceae |
| Parachlamydiaceae | 0 | 0 | 0 | 0 | 0 | 0 | 0 | 0 | 0 | 0 | 0 | 0 | 0 | 0 | Bacteria | Bacteria\_none | Chlamydiota | Chlamydiia | Parachlamydiales | Bacteria;Bacteria\_none;Chlamydiota;Chlamydiia;Parachlamydiales;Parachlamydiaceae |
| Chloroherpetonaceae | 0 | 0 | 0 | 0 | 0 | 1 | 0 | 0 | 0 | 0 | 0 | 0 | 0 | 0 | Bacteria | Bacteria\_none | Chlorobiota | Chlorobiia | Chlorobiales | Bacteria;Bacteria\_none;Chlorobiota;Chlorobiia;Chlorobiales;Chloroherpetonaceae |
| Candidatus Chloroheliaceae | 0 | 0 | 0 | 0 | 0 | 0 | 0 | 0 | 0 | 0 | 0 | 0 | 0 | 0 | Bacteria | Bacteria\_none | Chloroflexota | Chloroflexia | Candidatus Chloroheliales | Bacteria;Bacteria\_none;Chloroflexota;Chloroflexia;Candidatus Chloroheliales;Candidatus Chloroheliaceae |
| Candidatus Lucifugimonadaceae | 0 | 0 | 0 | 0 | 0 | 0 | 0 | 0 | 0 | 0 | 0 | 0 | 0 | 0 | Bacteria | Bacteria\_none | Chloroflexota | Dehalococcoidia | Candidatus Lucifugimonadales | Bacteria;Bacteria\_none;Chloroflexota;Dehalococcoidia;Candidatus Lucifugimonadales;Candidatus Lucifugimonadaceae |
| Cyanobacteriota\_Incertae\_sedis | 1 | 0 | 0 | 0 | 0 | 0 | 0 | 0 | 0 | 0 | 0 | 0 | 0 | 0 | Bacteria | Bacteria\_none | Cyanobacteriota | Cyanobacteriota\_Incertae\_sedis | Cyanobacteriota\_Incertae\_sedis | Bacteria;Bacteria\_none;Cyanobacteriota;Cyanobacteriota\_Incertae\_sedis;Cyanobacteriota\_Incertae\_sedis;Cyanobacteriota\_Incertae\_sedis |
| Chroococcaceae | 0 | 0 | 1 | 0 | 0 | 0 | 0 | 0 | 0 | 0 | 0 | 0 | 0 | 0 | Bacteria | Bacteria\_none | Cyanobacteriota | Cyanophyceae | Chroococcales | Bacteria;Bacteria\_none;Cyanobacteriota;Cyanophyceae;Chroococcales;Chroococcaceae |
| Geminocystaceae | 0 | 0 | 0 | 0 | 0 | 0 | 0 | 0 | 0 | 0 | 0 | 0 | 0 | 0 | Bacteria | Bacteria\_none | Cyanobacteriota | Cyanophyceae | Chroococcales | Bacteria;Bacteria\_none;Cyanobacteriota;Cyanophyceae;Chroococcales;Geminocystaceae |
| Gloeobacteraceae | 0 | 0 | 0 | 0 | 0 | 0 | 0 | 0 | 0 | 0 | 0 | 0 | 0 | 0 | Bacteria | Bacteria\_none | Cyanobacteriota | Cyanophyceae | Gloeobacterales | Bacteria;Bacteria\_none;Cyanobacteriota;Cyanophyceae;Gloeobacterales;Gloeobacteraceae |
| Aphanizomenonaceae | 0 | 0 | 0 | 1 | 0 | 0 | 0 | 0 | 0 | 0 | 0 | 0 | 0 | 0 | Bacteria | Bacteria\_none | Cyanobacteriota | Cyanophyceae | Nostocales | Bacteria;Bacteria\_none;Cyanobacteriota;Cyanophyceae;Nostocales;Aphanizomenonaceae |
| Nostocaceae | 0 | 1 | 0 | 0 | 0 | 0 | 0 | 0 | 0 | 0 | 0 | 0 | 0 | 0 | Bacteria | Bacteria\_none | Cyanobacteriota | Cyanophyceae | Nostocales | Bacteria;Bacteria\_none;Cyanobacteriota;Cyanophyceae;Nostocales;Nostocaceae |
| Prochlorococcaceae | 0 | 0 | 0 | 0 | 1 | 0 | 0 | 0 | 0 | 0 | 0 | 0 | 0 | 0 | Bacteria | Bacteria\_none | Cyanobacteriota | Cyanophyceae | Synechococcales | Bacteria;Bacteria\_none;Cyanobacteriota;Cyanophyceae;Synechococcales;Prochlorococcaceae |
| Synechococcaceae | 0 | 0 | 0 | 0 | 0 | 1 | 0 | 0 | 0 | 0 | 0 | 0 | 0 | 0 | Bacteria | Bacteria\_none | Cyanobacteriota | Cyanophyceae | Synechococcales | Bacteria;Bacteria\_none;Cyanobacteriota;Cyanophyceae;Synechococcales;Synechococcaceae |
| Calditerrivibrionaceae | 0 | 1 | 0 | 0 | 0 | 0 | 0 | 0 | 0 | 0 | 0 | 0 | 0 | 0 | Bacteria | Bacteria\_none | Deferribacterota | Deferribacteres | Deferribacterales | Bacteria;Bacteria\_none;Deferribacterota;Deferribacteres;Deferribacterales;Calditerrivibrionaceae |
| Mucispirillaceae | 0 | 0 | 0 | 0 | 0 | 0 | 0 | 0 | 0 | 0 | 0 | 0 | 0 | 0 | Bacteria | Bacteria\_none | Deferribacterota | Deferribacteres | Deferribacterales | Bacteria;Bacteria\_none;Deferribacterota;Deferribacteres;Deferribacterales;Mucispirillaceae |
| Fusobacteriaceae | 1 | 164 | 53 | 397 | 45 | 21 | 11 | 51 | 0 | 20 | 92 | 38 | 66 | 71 | Bacteria | Bacteria\_none | Fusobacteriota | Fusobacteriia | Fusobacteriales | Bacteria;Bacteria\_none;Fusobacteriota;Fusobacteriia;Fusobacteriales;Fusobacteriaceae |
| Haliovirgaceae | 0 | 0 | 0 | 0 | 0 | 0 | 0 | 0 | 0 | 0 | 0 | 0 | 0 | 0 | Bacteria | Bacteria\_none | Fusobacteriota | Fusobacteriia | Fusobacteriales | Bacteria;Bacteria\_none;Fusobacteriota;Fusobacteriia;Fusobacteriales;Haliovirgaceae |
| Leptotrichiaceae | 16 | 93 | 150 | 61 | 70 | 172 | 139 | 106 | 6 | 51 | 72 | 9 | 68 | 62 | Bacteria | Bacteria\_none | Fusobacteriota | Fusobacteriia | Fusobacteriales | Bacteria;Bacteria\_none;Fusobacteriota;Fusobacteriia;Fusobacteriales;Leptotrichiaceae |
| Ignavibacteriaceae | 0 | 0 | 0 | 0 | 0 | 0 | 0 | 0 | 0 | 0 | 0 | 0 | 0 | 0 | Bacteria | Bacteria\_none | Ignavibacteriota | Ignavibacteria | Ignavibacteriales | Bacteria;Bacteria\_none;Ignavibacteriota;Ignavibacteria;Ignavibacteriales;Ignavibacteriaceae |
| Acholeplasmataceae | 0 | 0 | 0 | 0 | 1 | 0 | 0 | 0 | 0 | 0 | 0 | 0 | 0 | 0 | Bacteria | Bacteria\_none | Mycoplasmatota | Mollicutes | Acholeplasmatales | Bacteria;Bacteria\_none;Mycoplasmatota;Mollicutes;Acholeplasmatales;Acholeplasmataceae |
| Spiroplasmataceae | 0 | 0 | 0 | 0 | 0 | 3 | 0 | 0 | 0 | 0 | 0 | 0 | 1 | 2 | Bacteria | Bacteria\_none | Mycoplasmatota | Mollicutes | Entomoplasmatales | Bacteria;Bacteria\_none;Mycoplasmatota;Mollicutes;Entomoplasmatales;Spiroplasmataceae |
| Mycoplasmataceae | 0 | 1 | 3 | 0 | 0 | 0 | 0 | 0 | 0 | 0 | 0 | 0 | 0 | 0 | Bacteria | Bacteria\_none | Mycoplasmatota | Mollicutes | Mycoplasmatales | Bacteria;Bacteria\_none;Mycoplasmatota;Mollicutes;Mycoplasmatales;Mycoplasmataceae |
| Mycoplasmatales\_Incertae\_sedis | 0 | 0 | 0 | 0 | 0 | 0 | 0 | 0 | 0 | 0 | 0 | 0 | 0 | 0 | Bacteria | Bacteria\_none | Mycoplasmatota | Mollicutes | Mycoplasmatales | Bacteria;Bacteria\_none;Mycoplasmatota;Mollicutes;Mycoplasmatales;Mycoplasmatales\_Incertae\_sedis |
| Metamycoplasmataceae | 0 | 10 | 35 | 1 | 1 | 5 | 0 | 1 | 0 | 3 | 1 | 6 | 3 | 4 | Bacteria | Bacteria\_none | Mycoplasmatota | Mycoplasmatota\_Incertae\_sedis | Mycoplasmoidales | Bacteria;Bacteria\_none;Mycoplasmatota;Mycoplasmatota\_Incertae\_sedis;Mycoplasmoidales;Metamycoplasmataceae |
| Mycoplasmoidaceae | 0 | 0 | 0 | 0 | 1 | 0 | 0 | 0 | 0 | 0 | 0 | 0 | 0 | 0 | Bacteria | Bacteria\_none | Mycoplasmatota | Mycoplasmatota\_Incertae\_sedis | Mycoplasmoidales | Bacteria;Bacteria\_none;Mycoplasmatota;Mycoplasmatota\_Incertae\_sedis;Mycoplasmoidales;Mycoplasmoidaceae |
| Myxococcaceae | 0 | 0 | 0 | 0 | 0 | 0 | 0 | 0 | 0 | 0 | 0 | 0 | 0 | 0 | Bacteria | Bacteria\_none | Myxococcota | Myxococcia | Myxococcales | Bacteria;Bacteria\_none;Myxococcota;Myxococcia;Myxococcales;Myxococcaceae |
| Polyangiaceae | 0 | 0 | 0 | 0 | 2 | 0 | 0 | 0 | 0 | 0 | 1 | 0 | 0 | 0 | Bacteria | Bacteria\_none | Myxococcota | Myxococcota\_Incertae\_sedis | Polyangiales | Bacteria;Bacteria\_none;Myxococcota;Myxococcota\_Incertae\_sedis;Polyangiales;Polyangiaceae |
| Nitrospiraceae | 0 | 0 | 0 | 0 | 0 | 1 | 0 | 0 | 0 | 0 | 0 | 0 | 0 | 0 | Bacteria | Bacteria\_none | Nitrospirota | Nitrospiria | Nitrospirales | Bacteria;Bacteria\_none;Nitrospirota;Nitrospiria;Nitrospirales;Nitrospiraceae |
| Dissulfurispiraceae | 0 | 0 | 0 | 0 | 0 | 0 | 0 | 0 | 0 | 0 | 0 | 0 | 0 | 0 | Bacteria | Bacteria\_none | Nitrospirota | Thermodesulfovibrionia | Thermodesulfovibrionales | Bacteria;Bacteria\_none;Nitrospirota;Thermodesulfovibrionia;Thermodesulfovibrionales;Dissulfurispiraceae |
| Gemmataceae | 0 | 0 | 0 | 0 | 0 | 1 | 0 | 0 | 0 | 0 | 0 | 0 | 0 | 0 | Bacteria | Bacteria\_none | Planctomycetota | Planctomycetia | Gemmatales | Bacteria;Bacteria\_none;Planctomycetota;Planctomycetia;Gemmatales;Gemmataceae |
| Pirellulaceae | 1 | 0 | 0 | 0 | 0 | 0 | 0 | 0 | 0 | 0 | 0 | 0 | 0 | 0 | Bacteria | Bacteria\_none | Planctomycetota | Planctomycetia | Pirellulales | Bacteria;Bacteria\_none;Planctomycetota;Planctomycetia;Pirellulales;Pirellulaceae |
| Planctomycetaceae | 0 | 0 | 0 | 0 | 0 | 0 | 0 | 0 | 0 | 0 | 1 | 0 | 0 | 0 | Bacteria | Bacteria\_none | Planctomycetota | Planctomycetia | Planctomycetales | Bacteria;Bacteria\_none;Planctomycetota;Planctomycetia;Planctomycetales;Planctomycetaceae |
| Candidatus Pelagibacteraceae | 0 | 0 | 0 | 0 | 0 | 0 | 0 | 0 | 0 | 0 | 0 | 0 | 0 | 0 | Bacteria | Bacteria\_none | Pseudomonadota | Alphaproteobacteria | Candidatus Pelagibacterales | Bacteria;Bacteria\_none;Pseudomonadota;Alphaproteobacteria;Candidatus Pelagibacterales;Candidatus Pelagibacteraceae |
| Caulobacteraceae | 0 | 0 | 0 | 0 | 0 | 0 | 0 | 0 | 0 | 0 | 0 | 0 | 0 | 0 | Bacteria | Bacteria\_none | Pseudomonadota | Alphaproteobacteria | Caulobacterales | Bacteria;Bacteria\_none;Pseudomonadota;Alphaproteobacteria;Caulobacterales;Caulobacteraceae |
| Boseaceae | 0 | 0 | 0 | 0 | 0 | 1 | 0 | 0 | 0 | 0 | 0 | 0 | 0 | 0 | Bacteria | Bacteria\_none | Pseudomonadota | Alphaproteobacteria | Hyphomicrobiales | Bacteria;Bacteria\_none;Pseudomonadota;Alphaproteobacteria;Hyphomicrobiales;Boseaceae |
| Brucellaceae | 0 | 0 | 0 | 0 | 0 | 0 | 0 | 0 | 0 | 0 | 0 | 0 | 0 | 0 | Bacteria | Bacteria\_none | Pseudomonadota | Alphaproteobacteria | Hyphomicrobiales | Bacteria;Bacteria\_none;Pseudomonadota;Alphaproteobacteria;Hyphomicrobiales;Brucellaceae |
| Cohaesibacteraceae | 0 | 0 | 0 | 0 | 0 | 0 | 0 | 0 | 1 | 0 | 0 | 0 | 0 | 0 | Bacteria | Bacteria\_none | Pseudomonadota | Alphaproteobacteria | Hyphomicrobiales | Bacteria;Bacteria\_none;Pseudomonadota;Alphaproteobacteria;Hyphomicrobiales;Cohaesibacteraceae |
| Devosiaceae | 0 | 0 | 1 | 0 | 0 | 0 | 0 | 0 | 0 | 0 | 0 | 0 | 0 | 0 | Bacteria | Bacteria\_none | Pseudomonadota | Alphaproteobacteria | Hyphomicrobiales | Bacteria;Bacteria\_none;Pseudomonadota;Alphaproteobacteria;Hyphomicrobiales;Devosiaceae |
| Methylobacteriaceae | 0 | 0 | 0 | 0 | 0 | 0 | 0 | 0 | 0 | 0 | 0 | 0 | 0 | 0 | Bacteria | Bacteria\_none | Pseudomonadota | Alphaproteobacteria | Hyphomicrobiales | Bacteria;Bacteria\_none;Pseudomonadota;Alphaproteobacteria;Hyphomicrobiales;Methylobacteriaceae |
| Nitrobacteraceae | 0 | 1 | 0 | 0 | 0 | 1 | 0 | 0 | 0 | 0 | 1 | 0 | 0 | 0 | Bacteria | Bacteria\_none | Pseudomonadota | Alphaproteobacteria | Hyphomicrobiales | Bacteria;Bacteria\_none;Pseudomonadota;Alphaproteobacteria;Hyphomicrobiales;Nitrobacteraceae |
| Phyllobacteriaceae | 0 | 0 | 2 | 0 | 0 | 1 | 1 | 2 | 0 | 0 | 0 | 0 | 0 | 0 | Bacteria | Bacteria\_none | Pseudomonadota | Alphaproteobacteria | Hyphomicrobiales | Bacteria;Bacteria\_none;Pseudomonadota;Alphaproteobacteria;Hyphomicrobiales;Phyllobacteriaceae |
| Rhizobiaceae | 1 | 0 | 0 | 0 | 1 | 1 | 0 | 0 | 0 | 1 | 0 | 0 | 0 | 0 | Bacteria | Bacteria\_none | Pseudomonadota | Alphaproteobacteria | Hyphomicrobiales | Bacteria;Bacteria\_none;Pseudomonadota;Alphaproteobacteria;Hyphomicrobiales;Rhizobiaceae |
| Kordiimonadaceae | 0 | 0 | 0 | 0 | 0 | 0 | 0 | 0 | 0 | 0 | 0 | 0 | 0 | 0 | Bacteria | Bacteria\_none | Pseudomonadota | Alphaproteobacteria | Kordiimonadales | Bacteria;Bacteria\_none;Pseudomonadota;Alphaproteobacteria;Kordiimonadales;Kordiimonadaceae |
| Paracoccaceae | 0 | 0 | 0 | 0 | 0 | 1 | 0 | 0 | 1 | 0 | 1 | 0 | 0 | 0 | Bacteria | Bacteria\_none | Pseudomonadota | Alphaproteobacteria | Rhodobacterales | Bacteria;Bacteria\_none;Pseudomonadota;Alphaproteobacteria;Rhodobacterales;Paracoccaceae |
| Roseobacteraceae | 2 | 1 | 0 | 0 | 0 | 0 | 0 | 0 | 0 | 0 | 0 | 0 | 0 | 0 | Bacteria | Bacteria\_none | Pseudomonadota | Alphaproteobacteria | Rhodobacterales | Bacteria;Bacteria\_none;Pseudomonadota;Alphaproteobacteria;Rhodobacterales;Roseobacteraceae |
| Acetobacteraceae | 0 | 0 | 0 | 0 | 0 | 1 | 0 | 0 | 0 | 0 | 0 | 0 | 0 | 0 | Bacteria | Bacteria\_none | Pseudomonadota | Alphaproteobacteria | Rhodospirillales | Bacteria;Bacteria\_none;Pseudomonadota;Alphaproteobacteria;Rhodospirillales;Acetobacteraceae |
| Azospirillaceae | 0 | 0 | 0 | 0 | 0 | 0 | 0 | 0 | 0 | 0 | 1 | 0 | 0 | 0 | Bacteria | Bacteria\_none | Pseudomonadota | Alphaproteobacteria | Rhodospirillales | Bacteria;Bacteria\_none;Pseudomonadota;Alphaproteobacteria;Rhodospirillales;Azospirillaceae |
| Magnetovibrionaceae | 0 | 0 | 0 | 0 | 0 | 0 | 0 | 0 | 0 | 0 | 0 | 0 | 0 | 0 | Bacteria | Bacteria\_none | Pseudomonadota | Alphaproteobacteria | Rhodospirillales | Bacteria;Bacteria\_none;Pseudomonadota;Alphaproteobacteria;Rhodospirillales;Magnetovibrionaceae |
| Thalassospiraceae | 0 | 0 | 0 | 1 | 0 | 0 | 0 | 0 | 0 | 0 | 0 | 0 | 0 | 0 | Bacteria | Bacteria\_none | Pseudomonadota | Alphaproteobacteria | Rhodospirillales | Bacteria;Bacteria\_none;Pseudomonadota;Alphaproteobacteria;Rhodospirillales;Thalassospiraceae |
| Anaplasmataceae | 0 | 1 | 0 | 0 | 0 | 0 | 0 | 0 | 0 | 0 | 0 | 0 | 1 | 0 | Bacteria | Bacteria\_none | Pseudomonadota | Alphaproteobacteria | Rickettsiales | Bacteria;Bacteria\_none;Pseudomonadota;Alphaproteobacteria;Rickettsiales;Anaplasmataceae |
| Rickettsiaceae | 0 | 0 | 0 | 0 | 0 | 0 | 0 | 0 | 0 | 0 | 0 | 0 | 0 | 0 | Bacteria | Bacteria\_none | Pseudomonadota | Alphaproteobacteria | Rickettsiales | Bacteria;Bacteria\_none;Pseudomonadota;Alphaproteobacteria;Rickettsiales;Rickettsiaceae |
| Sphingomonadaceae | 0 | 0 | 0 | 1 | 0 | 1 | 0 | 0 | 0 | 0 | 1 | 1 | 0 | 0 | Bacteria | Bacteria\_none | Pseudomonadota | Alphaproteobacteria | Sphingomonadales | Bacteria;Bacteria\_none;Pseudomonadota;Alphaproteobacteria;Sphingomonadales;Sphingomonadaceae |
| Betaproteobacteria\_Incertae\_sedis | 0 | 0 | 0 | 0 | 1 | 0 | 0 | 0 | 0 | 0 | 0 | 0 | 0 | 0 | Bacteria | Bacteria\_none | Pseudomonadota | Betaproteobacteria | Betaproteobacteria\_Incertae\_sedis | Bacteria;Bacteria\_none;Pseudomonadota;Betaproteobacteria;Betaproteobacteria\_Incertae\_sedis;Betaproteobacteria\_Incertae\_sedis |
| Casimicrobiaceae | 0 | 0 | 0 | 0 | 0 | 0 | 0 | 0 | 0 | 0 | 0 | 0 | 0 | 0 | Bacteria | Bacteria\_none | Pseudomonadota | Betaproteobacteria | Betaproteobacteria\_Incertae\_sedis | Bacteria;Bacteria\_none;Pseudomonadota;Betaproteobacteria;Betaproteobacteria\_Incertae\_sedis;Casimicrobiaceae |
| Alcaligenaceae | 0 | 0 | 0 | 0 | 1 | 1 | 1 | 0 | 0 | 0 | 0 | 0 | 0 | 0 | Bacteria | Bacteria\_none | Pseudomonadota | Betaproteobacteria | Burkholderiales | Bacteria;Bacteria\_none;Pseudomonadota;Betaproteobacteria;Burkholderiales;Alcaligenaceae |
| Burkholderiaceae | 1 | 1 | 1 | 0 | 2 | 1 | 0 | 1 | 0 | 1 | 6 | 0 | 3 | 1 | Bacteria | Bacteria\_none | Pseudomonadota | Betaproteobacteria | Burkholderiales | Bacteria;Bacteria\_none;Pseudomonadota;Betaproteobacteria;Burkholderiales;Burkholderiaceae |
| Comamonadaceae | 0 | 0 | 0 | 0 | 0 | 1 | 1 | 0 | 0 | 0 | 1 | 0 | 0 | 0 | Bacteria | Bacteria\_none | Pseudomonadota | Betaproteobacteria | Burkholderiales | Bacteria;Bacteria\_none;Pseudomonadota;Betaproteobacteria;Burkholderiales;Comamonadaceae |
| Oxalobacteraceae | 0 | 0 | 1 | 0 | 0 | 1 | 0 | 0 | 0 | 1 | 0 | 0 | 0 | 0 | Bacteria | Bacteria\_none | Pseudomonadota | Betaproteobacteria | Burkholderiales | Bacteria;Bacteria\_none;Pseudomonadota;Betaproteobacteria;Burkholderiales;Oxalobacteraceae |
| Sphaerotilaceae | 0 | 0 | 0 | 0 | 0 | 0 | 0 | 0 | 0 | 0 | 1 | 1 | 0 | 0 | Bacteria | Bacteria\_none | Pseudomonadota | Betaproteobacteria | Burkholderiales | Bacteria;Bacteria\_none;Pseudomonadota;Betaproteobacteria;Burkholderiales;Sphaerotilaceae |
| Neisseriaceae | 184 | 195 | 978 | 2167 | 217 | 235 | 20 | 46 | 0 | 868 | 79 | 40 | 279 | 162 | Bacteria | Bacteria\_none | Pseudomonadota | Betaproteobacteria | Neisseriales | Bacteria;Bacteria\_none;Pseudomonadota;Betaproteobacteria;Neisseriales;Neisseriaceae |
| Gallionellaceae | 0 | 0 | 0 | 0 | 0 | 0 | 0 | 0 | 0 | 0 | 0 | 0 | 0 | 0 | Bacteria | Bacteria\_none | Pseudomonadota | Betaproteobacteria | Nitrosomonadales | Bacteria;Bacteria\_none;Pseudomonadota;Betaproteobacteria;Nitrosomonadales;Gallionellaceae |
| Methylophilaceae | 0 | 0 | 0 | 0 | 0 | 0 | 0 | 0 | 0 | 0 | 0 | 0 | 0 | 0 | Bacteria | Bacteria\_none | Pseudomonadota | Betaproteobacteria | Nitrosomonadales | Bacteria;Bacteria\_none;Pseudomonadota;Betaproteobacteria;Nitrosomonadales;Methylophilaceae |
| Azonexaceae | 0 | 0 | 0 | 0 | 0 | 0 | 0 | 0 | 0 | 0 | 0 | 0 | 0 | 0 | Bacteria | Bacteria\_none | Pseudomonadota | Betaproteobacteria | Rhodocyclales | Bacteria;Bacteria\_none;Pseudomonadota;Betaproteobacteria;Rhodocyclales;Azonexaceae |
| Aeromonadaceae | 0 | 0 | 0 | 0 | 0 | 2 | 0 | 0 | 0 | 0 | 0 | 0 | 1 | 0 | Bacteria | Bacteria\_none | Pseudomonadota | Gammaproteobacteria | Aeromonadales | Bacteria;Bacteria\_none;Pseudomonadota;Gammaproteobacteria;Aeromonadales;Aeromonadaceae |
| Alteromonadaceae | 1 | 0 | 0 | 0 | 0 | 0 | 0 | 0 | 0 | 0 | 0 | 0 | 0 | 0 | Bacteria | Bacteria\_none | Pseudomonadota | Gammaproteobacteria | Alteromonadales | Bacteria;Bacteria\_none;Pseudomonadota;Gammaproteobacteria;Alteromonadales;Alteromonadaceae |
| Colwelliaceae | 0 | 0 | 1 | 0 | 0 | 0 | 0 | 0 | 0 | 0 | 0 | 0 | 0 | 0 | Bacteria | Bacteria\_none | Pseudomonadota | Gammaproteobacteria | Alteromonadales | Bacteria;Bacteria\_none;Pseudomonadota;Gammaproteobacteria;Alteromonadales;Colwelliaceae |
| Ferrimonadaceae | 0 | 0 | 0 | 0 | 0 | 0 | 0 | 0 | 0 | 0 | 0 | 0 | 0 | 0 | Bacteria | Bacteria\_none | Pseudomonadota | Gammaproteobacteria | Alteromonadales | Bacteria;Bacteria\_none;Pseudomonadota;Gammaproteobacteria;Alteromonadales;Ferrimonadaceae |
| Pseudoalteromonadaceae | 1 | 0 | 0 | 0 | 1 | 2 | 0 | 2 | 0 | 0 | 0 | 0 | 0 | 0 | Bacteria | Bacteria\_none | Pseudomonadota | Gammaproteobacteria | Alteromonadales | Bacteria;Bacteria\_none;Pseudomonadota;Gammaproteobacteria;Alteromonadales;Pseudoalteromonadaceae |
| Shewanellaceae | 1 | 1 | 0 | 0 | 0 | 0 | 0 | 1 | 0 | 0 | 0 | 1 | 0 | 0 | Bacteria | Bacteria\_none | Pseudomonadota | Gammaproteobacteria | Alteromonadales | Bacteria;Bacteria\_none;Pseudomonadota;Gammaproteobacteria;Alteromonadales;Shewanellaceae |
| Cardiobacteriaceae | 0 | 2 | 13 | 1 | 0 | 6 | 0 | 0 | 0 | 0 | 4 | 0 | 3 | 1 | Bacteria | Bacteria\_none | Pseudomonadota | Gammaproteobacteria | Cardiobacteriales | Bacteria;Bacteria\_none;Pseudomonadota;Gammaproteobacteria;Cardiobacteriales;Cardiobacteriaceae |
| Ignatzschineriaceae | 0 | 1 | 0 | 0 | 0 | 0 | 0 | 0 | 0 | 0 | 0 | 0 | 0 | 0 | Bacteria | Bacteria\_none | Pseudomonadota | Gammaproteobacteria | Cardiobacteriales | Bacteria;Bacteria\_none;Pseudomonadota;Gammaproteobacteria;Cardiobacteriales;Ignatzschineriaceae |
| Cellvibrionaceae | 0 | 0 | 0 | 0 | 0 | 0 | 0 | 0 | 0 | 0 | 0 | 0 | 0 | 0 | Bacteria | Bacteria\_none | Pseudomonadota | Gammaproteobacteria | Cellvibrionales | Bacteria;Bacteria\_none;Pseudomonadota;Gammaproteobacteria;Cellvibrionales;Cellvibrionaceae |
| Chromatiaceae | 0 | 0 | 0 | 0 | 0 | 0 | 0 | 0 | 0 | 0 | 0 | 0 | 0 | 0 | Bacteria | Bacteria\_none | Pseudomonadota | Gammaproteobacteria | Chromatiales | Bacteria;Bacteria\_none;Pseudomonadota;Gammaproteobacteria;Chromatiales;Chromatiaceae |
| Ectothiorhodospiraceae | 0 | 0 | 0 | 1 | 0 | 0 | 0 | 0 | 0 | 0 | 0 | 0 | 0 | 0 | Bacteria | Bacteria\_none | Pseudomonadota | Gammaproteobacteria | Chromatiales | Bacteria;Bacteria\_none;Pseudomonadota;Gammaproteobacteria;Chromatiales;Ectothiorhodospiraceae |
| Budviciaceae | 0 | 0 | 0 | 0 | 0 | 0 | 0 | 1 | 0 | 0 | 0 | 0 | 0 | 0 | Bacteria | Bacteria\_none | Pseudomonadota | Gammaproteobacteria | Enterobacterales | Bacteria;Bacteria\_none;Pseudomonadota;Gammaproteobacteria;Enterobacterales;Budviciaceae |
| Enterobacteriaceae | 4 | 0 | 0 | 3 | 3 | 1 | 0 | 3 | 0 | 3 | 0 | 0 | 0 | 1 | Bacteria | Bacteria\_none | Pseudomonadota | Gammaproteobacteria | Enterobacterales | Bacteria;Bacteria\_none;Pseudomonadota;Gammaproteobacteria;Enterobacterales;Enterobacteriaceae |
| Erwiniaceae | 0 | 0 | 0 | 0 | 1 | 0 | 0 | 1 | 0 | 0 | 0 | 1 | 1 | 0 | Bacteria | Bacteria\_none | Pseudomonadota | Gammaproteobacteria | Enterobacterales | Bacteria;Bacteria\_none;Pseudomonadota;Gammaproteobacteria;Enterobacterales;Erwiniaceae |
| Morganellaceae | 1 | 0 | 1 | 0 | 1 | 2 | 1 | 3 | 0 | 0 | 0 | 0 | 1 | 0 | Bacteria | Bacteria\_none | Pseudomonadota | Gammaproteobacteria | Enterobacterales | Bacteria;Bacteria\_none;Pseudomonadota;Gammaproteobacteria;Enterobacterales;Morganellaceae |
| Pectobacteriaceae | 0 | 0 | 0 | 0 | 1 | 0 | 0 | 0 | 0 | 0 | 0 | 0 | 0 | 0 | Bacteria | Bacteria\_none | Pseudomonadota | Gammaproteobacteria | Enterobacterales | Bacteria;Bacteria\_none;Pseudomonadota;Gammaproteobacteria;Enterobacterales;Pectobacteriaceae |
| Yersiniaceae | 0 | 0 | 0 | 0 | 0 | 0 | 0 | 0 | 0 | 0 | 0 | 0 | 0 | 0 | Bacteria | Bacteria\_none | Pseudomonadota | Gammaproteobacteria | Enterobacterales | Bacteria;Bacteria\_none;Pseudomonadota;Gammaproteobacteria;Enterobacterales;Yersiniaceae |
| Legionellaceae | 0 | 0 | 0 | 1 | 0 | 0 | 0 | 0 | 0 | 0 | 0 | 0 | 0 | 0 | Bacteria | Bacteria\_none | Pseudomonadota | Gammaproteobacteria | Legionellales | Bacteria;Bacteria\_none;Pseudomonadota;Gammaproteobacteria;Legionellales;Legionellaceae |
| Lysobacteraceae | 0 | 0 | 0 | 0 | 0 | 0 | 0 | 0 | 0 | 0 | 0 | 0 | 0 | 0 | Bacteria | Bacteria\_none | Pseudomonadota | Gammaproteobacteria | Lysobacterales | Bacteria;Bacteria\_none;Pseudomonadota;Gammaproteobacteria;Lysobacterales;Lysobacteraceae |
| Rhodanobacteraceae | 0 | 0 | 0 | 0 | 0 | 0 | 0 | 0 | 0 | 1 | 0 | 0 | 0 | 0 | Bacteria | Bacteria\_none | Pseudomonadota | Gammaproteobacteria | Lysobacterales | Bacteria;Bacteria\_none;Pseudomonadota;Gammaproteobacteria;Lysobacterales;Rhodanobacteraceae |
| Methylococcaceae | 0 | 0 | 0 | 0 | 0 | 1 | 0 | 0 | 0 | 0 | 0 | 0 | 0 | 1 | Bacteria | Bacteria\_none | Pseudomonadota | Gammaproteobacteria | Methylococcales | Bacteria;Bacteria\_none;Pseudomonadota;Gammaproteobacteria;Methylococcales;Methylococcaceae |
| Moraxellaceae | 0 | 0 | 1 | 1 | 0 | 2 | 0 | 1 | 0 | 2 | 0 | 1 | 0 | 0 | Bacteria | Bacteria\_none | Pseudomonadota | Gammaproteobacteria | Moraxellales | Bacteria;Bacteria\_none;Pseudomonadota;Gammaproteobacteria;Moraxellales;Moraxellaceae |
| Alcanivoracaceae | 0 | 0 | 0 | 0 | 0 | 0 | 0 | 1 | 0 | 0 | 0 | 0 | 0 | 0 | Bacteria | Bacteria\_none | Pseudomonadota | Gammaproteobacteria | Oceanospirillales | Bacteria;Bacteria\_none;Pseudomonadota;Gammaproteobacteria;Oceanospirillales;Alcanivoracaceae |
| Halomonadaceae | 0 | 0 | 0 | 0 | 0 | 1 | 0 | 0 | 0 | 0 | 0 | 0 | 0 | 0 | Bacteria | Bacteria\_none | Pseudomonadota | Gammaproteobacteria | Oceanospirillales | Bacteria;Bacteria\_none;Pseudomonadota;Gammaproteobacteria;Oceanospirillales;Halomonadaceae |
| Oceanospirillaceae | 0 | 1 | 0 | 0 | 0 | 1 | 0 | 0 | 0 | 0 | 0 | 0 | 0 | 0 | Bacteria | Bacteria\_none | Pseudomonadota | Gammaproteobacteria | Oceanospirillales | Bacteria;Bacteria\_none;Pseudomonadota;Gammaproteobacteria;Oceanospirillales;Oceanospirillaceae |
| Orbaceae | 0 | 0 | 0 | 0 | 0 | 0 | 0 | 1 | 0 | 0 | 0 | 0 | 0 | 1 | Bacteria | Bacteria\_none | Pseudomonadota | Gammaproteobacteria | Orbales | Bacteria;Bacteria\_none;Pseudomonadota;Gammaproteobacteria;Orbales;Orbaceae |
| Pasteurellaceae | 61 | 137 | 416 | 408 | 169 | 176 | 2 | 457 | 0 | 48 | 19 | 97 | 130 | 72 | Bacteria | Bacteria\_none | Pseudomonadota | Gammaproteobacteria | Pasteurellales | Bacteria;Bacteria\_none;Pseudomonadota;Gammaproteobacteria;Pasteurellales;Pasteurellaceae |
| Pseudomonadaceae | 0 | 3 | 0 | 0 | 2 | 2 | 0 | 0 | 0 | 0 | 1 | 0 | 0 | 0 | Bacteria | Bacteria\_none | Pseudomonadota | Gammaproteobacteria | Pseudomonadales | Bacteria;Bacteria\_none;Pseudomonadota;Gammaproteobacteria;Pseudomonadales;Pseudomonadaceae |
| Piscirickettsiaceae | 0 | 0 | 0 | 0 | 1 | 0 | 0 | 0 | 0 | 0 | 0 | 0 | 0 | 0 | Bacteria | Bacteria\_none | Pseudomonadota | Gammaproteobacteria | Thiotrichales | Bacteria;Bacteria\_none;Pseudomonadota;Gammaproteobacteria;Thiotrichales;Piscirickettsiaceae |
| Vibrionaceae | 0 | 0 | 2 | 0 | 1 | 1 | 0 | 2 | 0 | 0 | 0 | 0 | 1 | 0 | Bacteria | Bacteria\_none | Pseudomonadota | Gammaproteobacteria | Vibrionales | Bacteria;Bacteria\_none;Pseudomonadota;Gammaproteobacteria;Vibrionales;Vibrionaceae |
| Leptospiraceae | 1 | 0 | 0 | 0 | 0 | 0 | 0 | 0 | 0 | 0 | 0 | 0 | 0 | 0 | Bacteria | Bacteria\_none | Spirochaetota | Spirochaetia | Leptospirales | Bacteria;Bacteria\_none;Spirochaetota;Spirochaetia;Leptospirales;Leptospiraceae |
| Borreliaceae | 0 | 0 | 0 | 0 | 0 | 1 | 0 | 0 | 0 | 0 | 0 | 0 | 0 | 0 | Bacteria | Bacteria\_none | Spirochaetota | Spirochaetia | Spirochaetales | Bacteria;Bacteria\_none;Spirochaetota;Spirochaetia;Spirochaetales;Borreliaceae |
| Breznakiellaceae | 0 | 1 | 0 | 0 | 0 | 0 | 0 | 0 | 0 | 0 | 0 | 0 | 0 | 0 | Bacteria | Bacteria\_none | Spirochaetota | Spirochaetia | Spirochaetales | Bacteria;Bacteria\_none;Spirochaetota;Spirochaetia;Spirochaetales;Breznakiellaceae |
| Sphaerochaetaceae | 0 | 1 | 0 | 0 | 0 | 0 | 0 | 0 | 0 | 0 | 0 | 0 | 0 | 0 | Bacteria | Bacteria\_none | Spirochaetota | Spirochaetia | Spirochaetales | Bacteria;Bacteria\_none;Spirochaetota;Spirochaetia;Spirochaetales;Sphaerochaetaceae |
| Treponemataceae | 3 | 67 | 47 | 5 | 27 | 58 | 2 | 33 | 0 | 7 | 29 | 42 | 23 | 10 | Bacteria | Bacteria\_none | Spirochaetota | Spirochaetia | Spirochaetales | Bacteria;Bacteria\_none;Spirochaetota;Spirochaetia;Spirochaetales;Treponemataceae |
| Aminobacteriaceae | 0 | 0 | 1 | 0 | 0 | 0 | 0 | 0 | 0 | 0 | 1 | 1 | 0 | 0 | Bacteria | Bacteria\_none | Synergistota | Synergistia | Synergistales | Bacteria;Bacteria\_none;Synergistota;Synergistia;Synergistales;Aminobacteriaceae |
| Candidatus Desulfofervidaceae | 0 | 0 | 0 | 0 | 0 | 1 | 0 | 0 | 0 | 0 | 0 | 0 | 0 | 0 | Bacteria | Bacteria\_none | Thermodesulfobacteriota | Candidatus Desulfofervidia | Candidatus Desulfofervidales | Bacteria;Bacteria\_none;Thermodesulfobacteriota;Candidatus Desulfofervidia;Candidatus Desulfofervidales;Candidatus Desulfofervidaceae |
| Desulfarculaceae | 0 | 0 | 0 | 0 | 0 | 0 | 0 | 0 | 0 | 0 | 0 | 0 | 0 | 0 | Bacteria | Bacteria\_none | Thermodesulfobacteriota | Desulfarculia | Desulfarculales | Bacteria;Bacteria\_none;Thermodesulfobacteriota;Desulfarculia;Desulfarculales;Desulfarculaceae |
| Desulfobulbaceae | 0 | 1 | 1 | 0 | 0 | 0 | 0 | 1 | 0 | 0 | 0 | 0 | 0 | 0 | Bacteria | Bacteria\_none | Thermodesulfobacteriota | Desulfobulbia | Desulfobulbales | Bacteria;Bacteria\_none;Thermodesulfobacteriota;Desulfobulbia;Desulfobulbales;Desulfobulbaceae |
| Desulfovibrionaceae | 0 | 0 | 0 | 0 | 0 | 0 | 0 | 0 | 0 | 0 | 0 | 0 | 0 | 0 | Bacteria | Bacteria\_none | Thermodesulfobacteriota | Desulfovibrionia | Desulfovibrionales | Bacteria;Bacteria\_none;Thermodesulfobacteriota;Desulfovibrionia;Desulfovibrionales;Desulfovibrionaceae |
| Syntrophotaleaceae | 0 | 0 | 0 | 0 | 0 | 0 | 0 | 0 | 0 | 0 | 1 | 0 | 0 | 0 | Bacteria | Bacteria\_none | Thermodesulfobacteriota | Desulfuromonadia | Desulfuromonadales | Bacteria;Bacteria\_none;Thermodesulfobacteriota;Desulfuromonadia;Desulfuromonadales;Syntrophotaleaceae |
| Geobacteraceae | 0 | 0 | 1 | 0 | 0 | 0 | 0 | 0 | 0 | 0 | 0 | 0 | 0 | 0 | Bacteria | Bacteria\_none | Thermodesulfobacteriota | Desulfuromonadia | Geobacterales | Bacteria;Bacteria\_none;Thermodesulfobacteriota;Desulfuromonadia;Geobacterales;Geobacteraceae |
| Thermomicrobiaceae | 0 | 1 | 0 | 0 | 0 | 0 | 0 | 0 | 0 | 0 | 0 | 0 | 0 | 0 | Bacteria | Bacteria\_none | Thermomicrobiota | Thermomicrobia | Thermomicrobiales | Bacteria;Bacteria\_none;Thermomicrobiota;Thermomicrobia;Thermomicrobiales;Thermomicrobiaceae |
| Fervidobacteriaceae | 1 | 0 | 0 | 0 | 0 | 0 | 0 | 0 | 0 | 0 | 0 | 0 | 0 | 0 | Bacteria | Bacteria\_none | Thermotogota | Thermotogae | Thermotogales | Bacteria;Bacteria\_none;Thermotogota;Thermotogae;Thermotogales;Fervidobacteriaceae |
| Opitutaceae | 0 | 1 | 0 | 0 | 0 | 0 | 0 | 0 | 0 | 0 | 0 | 0 | 0 | 0 | Bacteria | Bacteria\_none | Verrucomicrobiota | Opitutia | Opitutales | Bacteria;Bacteria\_none;Verrucomicrobiota;Opitutia;Opitutales;Opitutaceae |
| Hominidae | 376 | 885 | 987 | 56 | 1722 | 157 | 4047 | 4297 | 5129 | 2611 | 2430 | 4145 | 2771 | 2608 | Eukaryota | Metazoa | Chordata | Mammalia | Primates | Eukaryota;Metazoa;Chordata;Mammalia;Primates;Hominidae |
| Unknown | 1256 | 1011 | 1202 | 1466 | 1086 | 1893 | 327 | 336 | 190 | 749 | 678 | 504 | 1135 | 1968 | Unclassified | Unknown | Unknown | Unknown | Unknown | Unclassified;Unknown;Unknown;Unknown;Unknown;Unknown |
| Mimiviridae | 0 | 0 | 0 | 0 | 0 | 0 | 0 | 0 | 0 | 0 | 0 | 0 | 1 | 0 | Viruses | Bamfordvirae | Nucleocytoviricota | Megaviricetes | Imitervirales | Viruses;Bamfordvirae;Nucleocytoviricota;Megaviricetes;Imitervirales;Mimiviridae |
| Schizomimiviridae | 0 | 0 | 0 | 0 | 0 | 0 | 0 | 0 | 0 | 0 | 0 | 0 | 0 | 0 | Viruses | Bamfordvirae | Nucleocytoviricota | Megaviricetes | Imitervirales | Viruses;Bamfordvirae;Nucleocytoviricota;Megaviricetes;Imitervirales;Schizomimiviridae |
| Orthoherpesviridae | 7 | 0 | 0 | 0 | 0 | 0 | 0 | 0 | 0 | 0 | 0 | 0 | 2 | 0 | Viruses | Heunggongvirae | Peploviricota | Herviviricetes | Herpesvirales | Viruses;Heunggongvirae;Peploviricota;Herviviricetes;Herpesvirales;Orthoherpesviridae |
| Caudoviricetes\_Incertae\_sedis | 4 | 0 | 0 | 0 | 3 | 0 | 1 | 0 | 0 | 0 | 1 | 0 | 1 | 0 | Viruses | Heunggongvirae | Uroviricota | Caudoviricetes | Caudoviricetes\_Incertae\_sedis | Viruses;Heunggongvirae;Uroviricota;Caudoviricetes;Caudoviricetes\_Incertae\_sedis;Caudoviricetes\_Incertae\_sedis |
| Intestiviridae | 2 | 0 | 0 | 0 | 0 | 0 | 0 | 0 | 0 | 0 | 0 | 0 | 0 | 0 | Viruses | Heunggongvirae | Uroviricota | Caudoviricetes | Crassvirales | Viruses;Heunggongvirae;Uroviricota;Caudoviricetes;Crassvirales;Intestiviridae |
| Rhabdoviridae | 0 | 0 | 0 | 0 | 0 | 0 | 0 | 0 | 0 | 0 | 0 | 0 | 0 | 1 | Viruses | Orthornavirae | Negarnaviricota | Monjiviricetes | Mononegavirales | Viruses;Orthornavirae;Negarnaviricota;Monjiviricetes;Mononegavirales;Rhabdoviridae |
| Papillomaviridae | 0 | 0 | 0 | 0 | 0 | 0 | 0 | 1 | 0 | 0 | 0 | 0 | 0 | 0 | Viruses | Shotokuvirae | Cossaviricota | Papovaviricetes | Zurhausenvirales | Viruses;Shotokuvirae;Cossaviricota;Papovaviricetes;Zurhausenvirales;Papillomaviridae |
| Lipothrixviridae | 0 | 0 | 0 | 0 | 0 | 0 | 0 | 0 | 0 | 0 | 0 | 0 | 0 | 0 | Viruses | Zilligvirae | Taleaviricota | Tokiviricetes | Ligamenvirales | Viruses;Zilligvirae;Taleaviricota;Tokiviricetes;Ligamenvirales;Lipothrixviridae |

Export CSV

Rarefied abundance table for the genus rank.

All samples have been randomly subsetted to have the same number
of reads.

| genus | barcode01 | barcode02 | barcode03 | barcode04 | barcode05 | barcode06 | barcode07 | barcode08 | barcode09 | barcode10 | barcode11 | barcode12 | total | unclassified | superkingdom | kingdom | phylum | class | order | family | tax |
| --- | --- | --- | --- | --- | --- | --- | --- | --- | --- | --- | --- | --- | --- | --- | --- | --- | --- | --- | --- | --- | --- |
| Haladaptatus | 0 | 1 | 0 | 0 | 0 | 0 | 0 | 0 | 0 | 0 | 0 | 0 | 0 | 0 | Archaea | Archaea\_none | Euryarchaeota | Halobacteria | Halobacteriales | Haladaptataceae | Archaea;Archaea\_none;Euryarchaeota;Halobacteria;Halobacteriales;Haladaptataceae;Haladaptatus |
| Halococcoides | 0 | 0 | 0 | 0 | 0 | 0 | 0 | 0 | 0 | 0 | 0 | 0 | 0 | 0 | Archaea | Archaea\_none | Euryarchaeota | Halobacteria | Halobacteriales | Haloarculaceae | Archaea;Archaea\_none;Euryarchaeota;Halobacteria;Halobacteriales;Haloarculaceae;Halococcoides |
| Halospeciosus | 1 | 0 | 0 | 0 | 0 | 0 | 0 | 0 | 0 | 0 | 0 | 0 | 0 | 0 | Archaea | Archaea\_none | Euryarchaeota | Halobacteria | Halobacteriales | Halobacteriaceae | Archaea;Archaea\_none;Euryarchaeota;Halobacteria;Halobacteriales;Halobacteriaceae;Halospeciosus |
| Halobaculum | 0 | 0 | 0 | 0 | 0 | 0 | 0 | 0 | 0 | 0 | 0 | 0 | 1 | 0 | Archaea | Archaea\_none | Euryarchaeota | Halobacteria | Halobacteriales | Haloferacaceae | Archaea;Archaea\_none;Euryarchaeota;Halobacteria;Halobacteriales;Haloferacaceae;Halobaculum |
| Halogeometricum | 0 | 0 | 0 | 0 | 0 | 1 | 0 | 0 | 0 | 0 | 0 | 0 | 0 | 0 | Archaea | Archaea\_none | Euryarchaeota | Halobacteria | Halobacteriales | Haloferacaceae | Archaea;Archaea\_none;Euryarchaeota;Halobacteria;Halobacteriales;Haloferacaceae;Halogeometricum |
| Halorarum | 0 | 0 | 0 | 0 | 0 | 0 | 0 | 0 | 0 | 0 | 0 | 0 | 0 | 0 | Archaea | Archaea\_none | Euryarchaeota | Halobacteria | Halobacteriales | Haloferacaceae | Archaea;Archaea\_none;Euryarchaeota;Halobacteria;Halobacteriales;Haloferacaceae;Halorarum |
| Halorubrum | 0 | 0 | 0 | 0 | 0 | 0 | 0 | 0 | 0 | 0 | 0 | 0 | 0 | 0 | Archaea | Archaea\_none | Euryarchaeota | Halobacteria | Halobacteriales | Haloferacaceae | Archaea;Archaea\_none;Euryarchaeota;Halobacteria;Halobacteriales;Haloferacaceae;Halorubrum |
| Methanobacterium | 0 | 0 | 0 | 0 | 0 | 0 | 0 | 0 | 0 | 0 | 0 | 0 | 0 | 0 | Archaea | Archaea\_none | Euryarchaeota | Methanobacteria | Methanobacteriales | Methanobacteriaceae | Archaea;Archaea\_none;Euryarchaeota;Methanobacteria;Methanobacteriales;Methanobacteriaceae;Methanobacterium |
| Methanobrevibacter | 0 | 0 | 1 | 0 | 0 | 1 | 0 | 0 | 0 | 0 | 0 | 0 | 0 | 0 | Archaea | Archaea\_none | Euryarchaeota | Methanobacteria | Methanobacteriales | Methanobacteriaceae | Archaea;Archaea\_none;Euryarchaeota;Methanobacteria;Methanobacteriales;Methanobacteriaceae;Methanobrevibacter |
| Methanosphaera | 0 | 0 | 1 | 0 | 0 | 0 | 0 | 0 | 0 | 0 | 0 | 0 | 0 | 0 | Archaea | Archaea\_none | Euryarchaeota | Methanobacteria | Methanobacteriales | Methanobacteriaceae | Archaea;Archaea\_none;Euryarchaeota;Methanobacteria;Methanobacteriales;Methanobacteriaceae;Methanosphaera |
| Methanocaldococcus | 0 | 0 | 1 | 0 | 0 | 0 | 0 | 0 | 0 | 0 | 0 | 0 | 0 | 0 | Archaea | Archaea\_none | Euryarchaeota | Methanococci | Methanococcales | Methanocaldococcaceae | Archaea;Archaea\_none;Euryarchaeota;Methanococci;Methanococcales;Methanocaldococcaceae;Methanocaldococcus |
| Methanoregula | 0 | 1 | 0 | 0 | 0 | 0 | 0 | 0 | 0 | 0 | 0 | 0 | 0 | 0 | Archaea | Archaea\_none | Euryarchaeota | Methanomicrobia | Methanomicrobiales | Methanoregulaceae | Archaea;Archaea\_none;Euryarchaeota;Methanomicrobia;Methanomicrobiales;Methanoregulaceae;Methanoregula |
| Methanimicrococcus | 0 | 0 | 0 | 1 | 0 | 0 | 0 | 0 | 0 | 0 | 0 | 0 | 0 | 0 | Archaea | Archaea\_none | Euryarchaeota | Methanomicrobia | Methanosarcinales | Methanosarcinaceae | Archaea;Archaea\_none;Euryarchaeota;Methanomicrobia;Methanosarcinales;Methanosarcinaceae;Methanimicrococcus |
| Methanolapillus | 0 | 0 | 0 | 0 | 0 | 0 | 0 | 0 | 0 | 0 | 0 | 0 | 0 | 0 | Archaea | Archaea\_none | Euryarchaeota | Methanomicrobia | Methanosarcinales | Methanosarcinaceae | Archaea;Archaea\_none;Euryarchaeota;Methanomicrobia;Methanosarcinales;Methanosarcinaceae;Methanolapillus |
| Methanolobus | 0 | 0 | 0 | 0 | 1 | 1 | 0 | 0 | 0 | 0 | 0 | 0 | 0 | 0 | Archaea | Archaea\_none | Euryarchaeota | Methanomicrobia | Methanosarcinales | Methanosarcinaceae | Archaea;Archaea\_none;Euryarchaeota;Methanomicrobia;Methanosarcinales;Methanosarcinaceae;Methanolobus |
| Methanomethylovorans | 0 | 0 | 0 | 0 | 0 | 0 | 0 | 0 | 0 | 0 | 0 | 0 | 0 | 0 | Archaea | Archaea\_none | Euryarchaeota | Methanomicrobia | Methanosarcinales | Methanosarcinaceae | Archaea;Archaea\_none;Euryarchaeota;Methanomicrobia;Methanosarcinales;Methanosarcinaceae;Methanomethylovorans |
| Methanosarcina | 0 | 0 | 0 | 0 | 0 | 1 | 0 | 0 | 0 | 0 | 1 | 0 | 0 | 0 | Archaea | Archaea\_none | Euryarchaeota | Methanomicrobia | Methanosarcinales | Methanosarcinaceae | Archaea;Archaea\_none;Euryarchaeota;Methanomicrobia;Methanosarcinales;Methanosarcinaceae;Methanosarcina |
| Thermococcus | 0 | 0 | 0 | 0 | 0 | 0 | 0 | 0 | 0 | 0 | 0 | 1 | 0 | 0 | Archaea | Archaea\_none | Euryarchaeota | Thermococci | Thermococcales | Thermococcaceae | Archaea;Archaea\_none;Euryarchaeota;Thermococci;Thermococcales;Thermococcaceae;Thermococcus |
| Candidatus Nitrosotenuis | 0 | 0 | 0 | 0 | 0 | 1 | 0 | 0 | 0 | 0 | 0 | 0 | 0 | 0 | Archaea | Archaea\_none | Nitrososphaerota | Nitrososphaerota\_Incertae\_sedis | Nitrososphaerota\_Incertae\_sedis | Nitrososphaerota\_Incertae\_sedis | Archaea;Archaea\_none;Nitrososphaerota;Nitrososphaerota\_Incertae\_sedis;Nitrososphaerota\_Incertae\_sedis;Nitrososphaerota\_Incertae\_sedis;Candidatus Nitrosotenuis |
| Paludibaculum | 0 | 0 | 0 | 0 | 0 | 0 | 0 | 0 | 0 | 0 | 0 | 0 | 0 | 0 | Bacteria | Bacteria\_none | Acidobacteriota | Terriglobia | Bryobacterales | Bryobacteraceae | Bacteria;Bacteria\_none;Acidobacteriota;Terriglobia;Bryobacterales;Bryobacteraceae;Paludibaculum |
| Aquihabitans | 0 | 0 | 0 | 0 | 0 | 0 | 0 | 0 | 0 | 0 | 0 | 0 | 0 | 0 | Bacteria | Bacteria\_none | Actinomycetota | Acidimicrobiia | Acidimicrobiales | Iamiaceae | Bacteria;Bacteria\_none;Actinomycetota;Acidimicrobiia;Acidimicrobiales;Iamiaceae;Aquihabitans |
| Actinomyces | 1 | 1 | 0 | 0 | 2 | 12 | 1 | 1 | 2 | 0 | 0 | 1 | 1 | 0 | Bacteria | Bacteria\_none | Actinomycetota | Actinomycetes | Actinomycetales | Actinomycetaceae | Bacteria;Bacteria\_none;Actinomycetota;Actinomycetes;Actinomycetales;Actinomycetaceae;Actinomyces |
| Schaalia | 0 | 15 | 1 | 3 | 1 | 12 | 8 | 0 | 0 | 0 | 0 | 4 | 3 | 0 | Bacteria | Bacteria\_none | Actinomycetota | Actinomycetes | Actinomycetales | Actinomycetaceae | Bacteria;Bacteria\_none;Actinomycetota;Actinomycetes;Actinomycetales;Actinomycetaceae;Schaalia |
| Bifidobacterium | 0 | 0 | 1 | 0 | 0 | 0 | 1 | 1 | 0 | 0 | 0 | 0 | 0 | 0 | Bacteria | Bacteria\_none | Actinomycetota | Actinomycetes | Bifidobacteriales | Bifidobacteriaceae | Bacteria;Bacteria\_none;Actinomycetota;Actinomycetes;Bifidobacteriales;Bifidobacteriaceae;Bifidobacterium |
| Gardnerella | 0 | 1 | 1 | 0 | 0 | 0 | 0 | 0 | 0 | 0 | 0 | 0 | 0 | 0 | Bacteria | Bacteria\_none | Actinomycetota | Actinomycetes | Bifidobacteriales | Bifidobacteriaceae | Bacteria;Bacteria\_none;Actinomycetota;Actinomycetes;Bifidobacteriales;Bifidobacteriaceae;Gardnerella |
| Parascardovia | 0 | 0 | 0 | 0 | 1 | 0 | 0 | 0 | 0 | 0 | 0 | 0 | 1 | 0 | Bacteria | Bacteria\_none | Actinomycetota | Actinomycetes | Bifidobacteriales | Bifidobacteriaceae | Bacteria;Bacteria\_none;Actinomycetota;Actinomycetes;Bifidobacteriales;Bifidobacteriaceae;Parascardovia |
| Geodermatophilus | 0 | 0 | 0 | 0 | 1 | 0 | 0 | 0 | 0 | 0 | 0 | 0 | 0 | 0 | Bacteria | Bacteria\_none | Actinomycetota | Actinomycetes | Geodermatophilales | Geodermatophilaceae | Bacteria;Bacteria\_none;Actinomycetota;Actinomycetes;Geodermatophilales;Geodermatophilaceae;Geodermatophilus |
| Streptomyces | 0 | 2 | 0 | 0 | 2 | 6 | 1 | 1 | 0 | 2 | 0 | 1 | 1 | 1 | Bacteria | Bacteria\_none | Actinomycetota | Actinomycetes | Kitasatosporales | Streptomycetaceae | Bacteria;Bacteria\_none;Actinomycetota;Actinomycetes;Kitasatosporales;Streptomycetaceae;Streptomyces |
| Cellulomonas | 0 | 0 | 0 | 0 | 0 | 0 | 0 | 0 | 0 | 0 | 0 | 0 | 0 | 0 | Bacteria | Bacteria\_none | Actinomycetota | Actinomycetes | Micrococcales | Cellulomonadaceae | Bacteria;Bacteria\_none;Actinomycetota;Actinomycetes;Micrococcales;Cellulomonadaceae;Cellulomonas |
| Gephyromycinifex | 0 | 0 | 0 | 0 | 0 | 0 | 0 | 0 | 0 | 0 | 0 | 0 | 0 | 1 | Bacteria | Bacteria\_none | Actinomycetota | Actinomycetes | Micrococcales | Dermatophilaceae | Bacteria;Bacteria\_none;Actinomycetota;Actinomycetes;Micrococcales;Dermatophilaceae;Gephyromycinifex |
| Agromyces | 1 | 0 | 0 | 0 | 0 | 0 | 0 | 0 | 0 | 0 | 0 | 0 | 0 | 0 | Bacteria | Bacteria\_none | Actinomycetota | Actinomycetes | Micrococcales | Microbacteriaceae | Bacteria;Bacteria\_none;Actinomycetota;Actinomycetes;Micrococcales;Microbacteriaceae;Agromyces |
| Curtobacterium | 0 | 0 | 0 | 1 | 0 | 0 | 0 | 0 | 0 | 0 | 0 | 0 | 0 | 0 | Bacteria | Bacteria\_none | Actinomycetota | Actinomycetes | Micrococcales | Microbacteriaceae | Bacteria;Bacteria\_none;Actinomycetota;Actinomycetes;Micrococcales;Microbacteriaceae;Curtobacterium |
| Diaminobutyricibacter | 0 | 0 | 0 | 0 | 0 | 0 | 0 | 0 | 0 | 0 | 0 | 0 | 0 | 0 | Bacteria | Bacteria\_none | Actinomycetota | Actinomycetes | Micrococcales | Microbacteriaceae | Bacteria;Bacteria\_none;Actinomycetota;Actinomycetes;Micrococcales;Microbacteriaceae;Diaminobutyricibacter |
| Leucobacter | 0 | 0 | 0 | 0 | 0 | 0 | 0 | 0 | 0 | 0 | 0 | 0 | 0 | 0 | Bacteria | Bacteria\_none | Actinomycetota | Actinomycetes | Micrococcales | Microbacteriaceae | Bacteria;Bacteria\_none;Actinomycetota;Actinomycetes;Micrococcales;Microbacteriaceae;Leucobacter |
| Microbacterium | 0 | 0 | 0 | 0 | 1 | 0 | 0 | 0 | 0 | 0 | 0 | 0 | 0 | 0 | Bacteria | Bacteria\_none | Actinomycetota | Actinomycetes | Micrococcales | Microbacteriaceae | Bacteria;Bacteria\_none;Actinomycetota;Actinomycetes;Micrococcales;Microbacteriaceae;Microbacterium |
| Microcella | 0 | 0 | 0 | 0 | 0 | 0 | 0 | 0 | 0 | 0 | 0 | 0 | 0 | 0 | Bacteria | Bacteria\_none | Actinomycetota | Actinomycetes | Micrococcales | Microbacteriaceae | Bacteria;Bacteria\_none;Actinomycetota;Actinomycetes;Micrococcales;Microbacteriaceae;Microcella |
| Arthrobacter | 0 | 0 | 0 | 0 | 0 | 1 | 0 | 0 | 0 | 1 | 1 | 0 | 0 | 0 | Bacteria | Bacteria\_none | Actinomycetota | Actinomycetes | Micrococcales | Micrococcaceae | Bacteria;Bacteria\_none;Actinomycetota;Actinomycetes;Micrococcales;Micrococcaceae;Arthrobacter |
| Pseudarthrobacter | 0 | 0 | 0 | 0 | 0 | 0 | 0 | 0 | 0 | 0 | 0 | 0 | 0 | 0 | Bacteria | Bacteria\_none | Actinomycetota | Actinomycetes | Micrococcales | Micrococcaceae | Bacteria;Bacteria\_none;Actinomycetota;Actinomycetes;Micrococcales;Micrococcaceae;Pseudarthrobacter |
| Rothia | 23 | 100 | 21 | 8 | 60 | 35 | 11 | 14 | 7 | 39 | 66 | 81 | 31 | 16 | Bacteria | Bacteria\_none | Actinomycetota | Actinomycetes | Micrococcales | Micrococcaceae | Bacteria;Bacteria\_none;Actinomycetota;Actinomycetes;Micrococcales;Micrococcaceae;Rothia |
| Luteimicrobium | 0 | 0 | 0 | 0 | 0 | 0 | 0 | 0 | 0 | 0 | 0 | 0 | 0 | 0 | Bacteria | Bacteria\_none | Actinomycetota | Actinomycetes | Micrococcales | Micrococcales\_Incertae\_sedis | Bacteria;Bacteria\_none;Actinomycetota;Actinomycetes;Micrococcales;Micrococcales\_Incertae\_sedis;Luteimicrobium |
| Ruania | 0 | 0 | 0 | 0 | 0 | 0 | 0 | 0 | 0 | 0 | 0 | 0 | 0 | 0 | Bacteria | Bacteria\_none | Actinomycetota | Actinomycetes | Micrococcales | Ruaniaceae | Bacteria;Bacteria\_none;Actinomycetota;Actinomycetes;Micrococcales;Ruaniaceae;Ruania |
| Micromonospora | 1 | 0 | 0 | 1 | 0 | 0 | 0 | 0 | 0 | 1 | 0 | 0 | 0 | 0 | Bacteria | Bacteria\_none | Actinomycetota | Actinomycetes | Micromonosporales | Micromonosporaceae | Bacteria;Bacteria\_none;Actinomycetota;Actinomycetes;Micromonosporales;Micromonosporaceae;Micromonospora |
| Salinispora | 0 | 0 | 0 | 0 | 0 | 0 | 0 | 0 | 0 | 0 | 0 | 0 | 0 | 0 | Bacteria | Bacteria\_none | Actinomycetota | Actinomycetes | Micromonosporales | Micromonosporaceae | Bacteria;Bacteria\_none;Actinomycetota;Actinomycetes;Micromonosporales;Micromonosporaceae;Salinispora |
| Corynebacterium | 0 | 6 | 0 | 0 | 1 | 3 | 0 | 0 | 0 | 8 | 0 | 4 | 1 | 2 | Bacteria | Bacteria\_none | Actinomycetota | Actinomycetes | Mycobacteriales | Corynebacteriaceae | Bacteria;Bacteria\_none;Actinomycetota;Actinomycetes;Mycobacteriales;Corynebacteriaceae;Corynebacterium |
| Gordonia | 0 | 0 | 0 | 0 | 1 | 0 | 0 | 0 | 0 | 0 | 0 | 0 | 0 | 0 | Bacteria | Bacteria\_none | Actinomycetota | Actinomycetes | Mycobacteriales | Gordoniaceae | Bacteria;Bacteria\_none;Actinomycetota;Actinomycetes;Mycobacteriales;Gordoniaceae;Gordonia |
| Mycobacterium | 0 | 0 | 1 | 1 | 0 | 0 | 0 | 0 | 0 | 0 | 0 | 0 | 0 | 0 | Bacteria | Bacteria\_none | Actinomycetota | Actinomycetes | Mycobacteriales | Mycobacteriaceae | Bacteria;Bacteria\_none;Actinomycetota;Actinomycetes;Mycobacteriales;Mycobacteriaceae;Mycobacterium |
| Mycolicibacterium | 0 | 0 | 1 | 0 | 0 | 1 | 0 | 0 | 0 | 0 | 0 | 0 | 0 | 0 | Bacteria | Bacteria\_none | Actinomycetota | Actinomycetes | Mycobacteriales | Mycobacteriaceae | Bacteria;Bacteria\_none;Actinomycetota;Actinomycetes;Mycobacteriales;Mycobacteriaceae;Mycolicibacterium |
| Nocardia | 0 | 0 | 0 | 0 | 0 | 0 | 0 | 0 | 0 | 0 | 0 | 0 | 0 | 0 | Bacteria | Bacteria\_none | Actinomycetota | Actinomycetes | Mycobacteriales | Nocardiaceae | Bacteria;Bacteria\_none;Actinomycetota;Actinomycetes;Mycobacteriales;Nocardiaceae;Nocardia |
| Rhodococcus | 0 | 0 | 0 | 0 | 0 | 0 | 0 | 0 | 0 | 0 | 0 | 0 | 0 | 0 | Bacteria | Bacteria\_none | Actinomycetota | Actinomycetes | Mycobacteriales | Nocardiaceae | Bacteria;Bacteria\_none;Actinomycetota;Actinomycetes;Mycobacteriales;Nocardiaceae;Rhodococcus |
| Kribbella | 0 | 0 | 0 | 0 | 0 | 0 | 0 | 0 | 0 | 0 | 0 | 0 | 0 | 0 | Bacteria | Bacteria\_none | Actinomycetota | Actinomycetes | Propionibacteriales | Kribbellaceae | Bacteria;Bacteria\_none;Actinomycetota;Actinomycetes;Propionibacteriales;Kribbellaceae;Kribbella |
| Aeromicrobium | 0 | 0 | 0 | 1 | 0 | 0 | 0 | 0 | 0 | 0 | 0 | 0 | 0 | 0 | Bacteria | Bacteria\_none | Actinomycetota | Actinomycetes | Propionibacteriales | Nocardioidaceae | Bacteria;Bacteria\_none;Actinomycetota;Actinomycetes;Propionibacteriales;Nocardioidaceae;Aeromicrobium |
| Nocardioides | 0 | 0 | 1 | 1 | 1 | 0 | 0 | 0 | 0 | 0 | 0 | 0 | 0 | 0 | Bacteria | Bacteria\_none | Actinomycetota | Actinomycetes | Propionibacteriales | Nocardioidaceae | Bacteria;Bacteria\_none;Actinomycetota;Actinomycetes;Propionibacteriales;Nocardioidaceae;Nocardioides |
| Acidipropionibacterium | 0 | 0 | 0 | 0 | 0 | 0 | 0 | 0 | 0 | 0 | 0 | 0 | 0 | 0 | Bacteria | Bacteria\_none | Actinomycetota | Actinomycetes | Propionibacteriales | Propionibacteriaceae | Bacteria;Bacteria\_none;Actinomycetota;Actinomycetes;Propionibacteriales;Propionibacteriaceae;Acidipropionibacterium |
| Arachnia | 0 | 1 | 0 | 0 | 0 | 0 | 0 | 0 | 0 | 0 | 23 | 0 | 0 | 0 | Bacteria | Bacteria\_none | Actinomycetota | Actinomycetes | Propionibacteriales | Propionibacteriaceae | Bacteria;Bacteria\_none;Actinomycetota;Actinomycetes;Propionibacteriales;Propionibacteriaceae;Arachnia |
| Cutibacterium | 0 | 0 | 0 | 0 | 0 | 0 | 0 | 0 | 0 | 0 | 0 | 0 | 0 | 0 | Bacteria | Bacteria\_none | Actinomycetota | Actinomycetes | Propionibacteriales | Propionibacteriaceae | Bacteria;Bacteria\_none;Actinomycetota;Actinomycetes;Propionibacteriales;Propionibacteriaceae;Cutibacterium |
| Propionibacterium | 0 | 0 | 0 | 0 | 0 | 0 | 0 | 0 | 0 | 1 | 0 | 0 | 0 | 0 | Bacteria | Bacteria\_none | Actinomycetota | Actinomycetes | Propionibacteriales | Propionibacteriaceae | Bacteria;Bacteria\_none;Actinomycetota;Actinomycetes;Propionibacteriales;Propionibacteriaceae;Propionibacterium |
| Vaginimicrobium | 0 | 0 | 0 | 0 | 1 | 0 | 0 | 0 | 0 | 0 | 0 | 0 | 0 | 0 | Bacteria | Bacteria\_none | Actinomycetota | Actinomycetes | Propionibacteriales | Propionibacteriaceae | Bacteria;Bacteria\_none;Actinomycetota;Actinomycetes;Propionibacteriales;Propionibacteriaceae;Vaginimicrobium |
| Amycolatopsis | 0 | 0 | 0 | 1 | 0 | 0 | 0 | 0 | 0 | 0 | 0 | 0 | 0 | 0 | Bacteria | Bacteria\_none | Actinomycetota | Actinomycetes | Pseudonocardiales | Pseudonocardiaceae | Bacteria;Bacteria\_none;Actinomycetota;Actinomycetes;Pseudonocardiales;Pseudonocardiaceae;Amycolatopsis |
| Crossiella | 0 | 0 | 0 | 0 | 0 | 0 | 0 | 0 | 0 | 0 | 0 | 0 | 0 | 0 | Bacteria | Bacteria\_none | Actinomycetota | Actinomycetes | Pseudonocardiales | Pseudonocardiaceae | Bacteria;Bacteria\_none;Actinomycetota;Actinomycetes;Pseudonocardiales;Pseudonocardiaceae;Crossiella |
| Saccharopolyspora | 0 | 0 | 0 | 0 | 0 | 1 | 0 | 0 | 0 | 0 | 0 | 0 | 0 | 0 | Bacteria | Bacteria\_none | Actinomycetota | Actinomycetes | Pseudonocardiales | Pseudonocardiaceae | Bacteria;Bacteria\_none;Actinomycetota;Actinomycetes;Pseudonocardiales;Pseudonocardiaceae;Saccharopolyspora |
| Nonomuraea | 0 | 0 | 0 | 0 | 0 | 1 | 0 | 0 | 0 | 0 | 0 | 0 | 0 | 0 | Bacteria | Bacteria\_none | Actinomycetota | Actinomycetes | Streptosporangiales | Streptosporangiaceae | Bacteria;Bacteria\_none;Actinomycetota;Actinomycetes;Streptosporangiales;Streptosporangiaceae;Nonomuraea |
| Streptosporangium | 0 | 0 | 0 | 0 | 1 | 0 | 0 | 0 | 0 | 0 | 0 | 0 | 0 | 0 | Bacteria | Bacteria\_none | Actinomycetota | Actinomycetes | Streptosporangiales | Streptosporangiaceae | Bacteria;Bacteria\_none;Actinomycetota;Actinomycetes;Streptosporangiales;Streptosporangiaceae;Streptosporangium |
| Actinomadura | 0 | 0 | 0 | 1 | 0 | 0 | 0 | 0 | 0 | 0 | 0 | 0 | 0 | 0 | Bacteria | Bacteria\_none | Actinomycetota | Actinomycetes | Streptosporangiales | Thermomonosporaceae | Bacteria;Bacteria\_none;Actinomycetota;Actinomycetes;Streptosporangiales;Thermomonosporaceae;Actinomadura |
| Thermomonospora | 0 | 0 | 0 | 0 | 0 | 0 | 0 | 0 | 0 | 0 | 0 | 0 | 0 | 0 | Bacteria | Bacteria\_none | Actinomycetota | Actinomycetes | Streptosporangiales | Thermomonosporaceae | Bacteria;Bacteria\_none;Actinomycetota;Actinomycetes;Streptosporangiales;Thermomonosporaceae;Thermomonospora |
| Atopobium | 0 | 0 | 0 | 0 | 0 | 0 | 0 | 0 | 0 | 0 | 1 | 0 | 0 | 0 | Bacteria | Bacteria\_none | Actinomycetota | Coriobacteriia | Coriobacteriales | Atopobiaceae | Bacteria;Bacteria\_none;Actinomycetota;Coriobacteriia;Coriobacteriales;Atopobiaceae;Atopobium |
| Fannyhessea | 0 | 0 | 0 | 0 | 0 | 0 | 1 | 0 | 0 | 0 | 0 | 0 | 0 | 0 | Bacteria | Bacteria\_none | Actinomycetota | Coriobacteriia | Coriobacteriales | Atopobiaceae | Bacteria;Bacteria\_none;Actinomycetota;Coriobacteriia;Coriobacteriales;Atopobiaceae;Fannyhessea |
| Lancefieldella | 26 | 5 | 8 | 0 | 1 | 104 | 24 | 0 | 0 | 0 | 2 | 2 | 8 | 2 | Bacteria | Bacteria\_none | Actinomycetota | Coriobacteriia | Coriobacteriales | Atopobiaceae | Bacteria;Bacteria\_none;Actinomycetota;Coriobacteriia;Coriobacteriales;Atopobiaceae;Lancefieldella |
| Olsenella | 0 | 0 | 0 | 0 | 0 | 1 | 0 | 0 | 0 | 0 | 0 | 0 | 0 | 0 | Bacteria | Bacteria\_none | Actinomycetota | Coriobacteriia | Coriobacteriales | Atopobiaceae | Bacteria;Bacteria\_none;Actinomycetota;Coriobacteriia;Coriobacteriales;Atopobiaceae;Olsenella |
| Thermophilibacter | 0 | 0 | 0 | 0 | 0 | 0 | 1 | 0 | 0 | 0 | 0 | 0 | 0 | 0 | Bacteria | Bacteria\_none | Actinomycetota | Coriobacteriia | Coriobacteriales | Atopobiaceae | Bacteria;Bacteria\_none;Actinomycetota;Coriobacteriia;Coriobacteriales;Atopobiaceae;Thermophilibacter |
| Cryptobacterium | 0 | 0 | 0 | 0 | 0 | 1 | 0 | 0 | 0 | 0 | 0 | 0 | 0 | 0 | Bacteria | Bacteria\_none | Actinomycetota | Coriobacteriia | Eggerthellales | Eggerthellaceae | Bacteria;Bacteria\_none;Actinomycetota;Coriobacteriia;Eggerthellales;Eggerthellaceae;Cryptobacterium |
| Denitrobacterium | 0 | 0 | 0 | 0 | 0 | 0 | 0 | 0 | 0 | 0 | 0 | 0 | 0 | 0 | Bacteria | Bacteria\_none | Actinomycetota | Coriobacteriia | Eggerthellales | Eggerthellaceae | Bacteria;Bacteria\_none;Actinomycetota;Coriobacteriia;Eggerthellales;Eggerthellaceae;Denitrobacterium |
| Raoultibacter | 0 | 0 | 0 | 0 | 0 | 1 | 0 | 0 | 0 | 0 | 0 | 0 | 0 | 0 | Bacteria | Bacteria\_none | Actinomycetota | Coriobacteriia | Eggerthellales | Eggerthellaceae | Bacteria;Bacteria\_none;Actinomycetota;Coriobacteriia;Eggerthellales;Eggerthellaceae;Raoultibacter |
| Rubrobacter | 0 | 0 | 0 | 0 | 1 | 0 | 0 | 0 | 0 | 0 | 0 | 0 | 0 | 0 | Bacteria | Bacteria\_none | Actinomycetota | Rubrobacteria | Rubrobacterales | Rubrobacteraceae | Bacteria;Bacteria\_none;Actinomycetota;Rubrobacteria;Rubrobacterales;Rubrobacteraceae;Rubrobacter |
| Miltoncostaea | 0 | 1 | 0 | 0 | 0 | 0 | 0 | 0 | 0 | 0 | 0 | 0 | 0 | 0 | Bacteria | Bacteria\_none | Actinomycetota | Thermoleophilia | Miltoncostaeales | Miltoncostaeaceae | Bacteria;Bacteria\_none;Actinomycetota;Thermoleophilia;Miltoncostaeales;Miltoncostaeaceae;Miltoncostaea |
| Baekduia | 0 | 1 | 0 | 0 | 0 | 0 | 0 | 0 | 0 | 0 | 0 | 0 | 0 | 0 | Bacteria | Bacteria\_none | Actinomycetota | Thermoleophilia | Solirubrobacterales | Baekduiaceae | Bacteria;Bacteria\_none;Actinomycetota;Thermoleophilia;Solirubrobacterales;Baekduiaceae;Baekduia |
| Abyssicoccus | 0 | 0 | 0 | 0 | 1 | 0 | 0 | 0 | 0 | 0 | 0 | 0 | 0 | 0 | Bacteria | Bacteria\_none | Bacillota | Bacilli | Bacillales | Abyssicoccaceae | Bacteria;Bacteria\_none;Bacillota;Bacilli;Bacillales;Abyssicoccaceae;Abyssicoccus |
| Alicyclobacillus | 0 | 0 | 0 | 0 | 0 | 0 | 0 | 0 | 0 | 1 | 0 | 0 | 0 | 0 | Bacteria | Bacteria\_none | Bacillota | Bacilli | Bacillales | Alicyclobacillaceae | Bacteria;Bacteria\_none;Bacillota;Bacilli;Bacillales;Alicyclobacillaceae;Alicyclobacillus |
| Alkalihalobacillus | 0 | 0 | 0 | 1 | 0 | 0 | 0 | 0 | 0 | 0 | 0 | 0 | 0 | 0 | Bacteria | Bacteria\_none | Bacillota | Bacilli | Bacillales | Bacillaceae | Bacteria;Bacteria\_none;Bacillota;Bacilli;Bacillales;Bacillaceae;Alkalihalobacillus |
| Alkalihalophilus | 0 | 0 | 0 | 0 | 0 | 0 | 0 | 0 | 0 | 0 | 1 | 0 | 0 | 0 | Bacteria | Bacteria\_none | Bacillota | Bacilli | Bacillales | Bacillaceae | Bacteria;Bacteria\_none;Bacillota;Bacilli;Bacillales;Bacillaceae;Alkalihalophilus |
| Amphibacillus | 0 | 0 | 0 | 0 | 0 | 0 | 0 | 0 | 0 | 0 | 0 | 0 | 0 | 0 | Bacteria | Bacteria\_none | Bacillota | Bacilli | Bacillales | Bacillaceae | Bacteria;Bacteria\_none;Bacillota;Bacilli;Bacillales;Bacillaceae;Amphibacillus |
| Anaerobacillus | 0 | 1 | 0 | 0 | 0 | 0 | 0 | 0 | 0 | 0 | 0 | 0 | 0 | 0 | Bacteria | Bacteria\_none | Bacillota | Bacilli | Bacillales | Bacillaceae | Bacteria;Bacteria\_none;Bacillota;Bacilli;Bacillales;Bacillaceae;Anaerobacillus |
| Anoxybacillus | 1 | 0 | 0 | 0 | 0 | 0 | 0 | 0 | 0 | 0 | 0 | 0 | 0 | 0 | Bacteria | Bacteria\_none | Bacillota | Bacilli | Bacillales | Bacillaceae | Bacteria;Bacteria\_none;Bacillota;Bacilli;Bacillales;Bacillaceae;Anoxybacillus |
| Bacillus | 1 | 0 | 0 | 2 | 0 | 4 | 0 | 0 | 0 | 1 | 0 | 0 | 1 | 0 | Bacteria | Bacteria\_none | Bacillota | Bacilli | Bacillales | Bacillaceae | Bacteria;Bacteria\_none;Bacillota;Bacilli;Bacillales;Bacillaceae;Bacillus |
| Cytobacillus | 0 | 0 | 1 | 1 | 1 | 0 | 0 | 0 | 0 | 0 | 0 | 0 | 0 | 0 | Bacteria | Bacteria\_none | Bacillota | Bacilli | Bacillales | Bacillaceae | Bacteria;Bacteria\_none;Bacillota;Bacilli;Bacillales;Bacillaceae;Cytobacillus |
| Domibacillus | 0 | 0 | 0 | 0 | 0 | 0 | 0 | 0 | 0 | 0 | 0 | 0 | 0 | 0 | Bacteria | Bacteria\_none | Bacillota | Bacilli | Bacillales | Bacillaceae | Bacteria;Bacteria\_none;Bacillota;Bacilli;Bacillales;Bacillaceae;Domibacillus |
| Halalkalibacter | 0 | 0 | 0 | 0 | 0 | 0 | 0 | 0 | 0 | 0 | 0 | 0 | 0 | 0 | Bacteria | Bacteria\_none | Bacillota | Bacilli | Bacillales | Bacillaceae | Bacteria;Bacteria\_none;Bacillota;Bacilli;Bacillales;Bacillaceae;Halalkalibacter |
| Halobacillus | 0 | 0 | 0 | 0 | 0 | 0 | 0 | 0 | 0 | 0 | 0 | 0 | 0 | 0 | Bacteria | Bacteria\_none | Bacillota | Bacilli | Bacillales | Bacillaceae | Bacteria;Bacteria\_none;Bacillota;Bacilli;Bacillales;Bacillaceae;Halobacillus |
| Lysinibacillus | 0 | 0 | 1 | 0 | 0 | 1 | 1 | 0 | 0 | 0 | 0 | 0 | 1 | 0 | Bacteria | Bacteria\_none | Bacillota | Bacilli | Bacillales | Bacillaceae | Bacteria;Bacteria\_none;Bacillota;Bacilli;Bacillales;Bacillaceae;Lysinibacillus |
| Metabacillus | 0 | 1 | 0 | 0 | 0 | 0 | 0 | 0 | 0 | 0 | 0 | 0 | 0 | 0 | Bacteria | Bacteria\_none | Bacillota | Bacilli | Bacillales | Bacillaceae | Bacteria;Bacteria\_none;Bacillota;Bacilli;Bacillales;Bacillaceae;Metabacillus |
| Neobacillus | 0 | 0 | 1 | 0 | 0 | 0 | 0 | 0 | 0 | 0 | 0 | 0 | 0 | 0 | Bacteria | Bacteria\_none | Bacillota | Bacilli | Bacillales | Bacillaceae | Bacteria;Bacteria\_none;Bacillota;Bacilli;Bacillales;Bacillaceae;Neobacillus |
| Niallia | 1 | 0 | 1 | 0 | 0 | 0 | 0 | 0 | 0 | 0 | 0 | 0 | 0 | 1 | Bacteria | Bacteria\_none | Bacillota | Bacilli | Bacillales | Bacillaceae | Bacteria;Bacteria\_none;Bacillota;Bacilli;Bacillales;Bacillaceae;Niallia |
| Oceanobacillus | 0 | 0 | 0 | 0 | 0 | 1 | 0 | 0 | 0 | 0 | 0 | 0 | 1 | 0 | Bacteria | Bacteria\_none | Bacillota | Bacilli | Bacillales | Bacillaceae | Bacteria;Bacteria\_none;Bacillota;Bacilli;Bacillales;Bacillaceae;Oceanobacillus |
| Ornithinibacillus | 0 | 0 | 0 | 0 | 0 | 1 | 0 | 0 | 0 | 0 | 0 | 0 | 0 | 0 | Bacteria | Bacteria\_none | Bacillota | Bacilli | Bacillales | Bacillaceae | Bacteria;Bacteria\_none;Bacillota;Bacilli;Bacillales;Bacillaceae;Ornithinibacillus |
| Parageobacillus | 0 | 0 | 0 | 0 | 0 | 0 | 0 | 0 | 0 | 0 | 0 | 0 | 0 | 0 | Bacteria | Bacteria\_none | Bacillota | Bacilli | Bacillales | Bacillaceae | Bacteria;Bacteria\_none;Bacillota;Bacilli;Bacillales;Bacillaceae;Parageobacillus |
| Peribacillus | 0 | 0 | 0 | 0 | 0 | 0 | 0 | 1 | 0 | 0 | 0 | 0 | 0 | 0 | Bacteria | Bacteria\_none | Bacillota | Bacilli | Bacillales | Bacillaceae | Bacteria;Bacteria\_none;Bacillota;Bacilli;Bacillales;Bacillaceae;Peribacillus |
| Pontibacillus | 0 | 0 | 0 | 0 | 0 | 0 | 0 | 0 | 0 | 0 | 0 | 0 | 0 | 0 | Bacteria | Bacteria\_none | Bacillota | Bacilli | Bacillales | Bacillaceae | Bacteria;Bacteria\_none;Bacillota;Bacilli;Bacillales;Bacillaceae;Pontibacillus |
| Priestia | 0 | 0 | 1 | 0 | 2 | 1 | 1 | 0 | 0 | 2 | 0 | 0 | 0 | 0 | Bacteria | Bacteria\_none | Bacillota | Bacilli | Bacillales | Bacillaceae | Bacteria;Bacteria\_none;Bacillota;Bacilli;Bacillales;Bacillaceae;Priestia |
| Pseudalkalibacillus | 0 | 0 | 0 | 1 | 0 | 0 | 0 | 0 | 0 | 0 | 0 | 0 | 0 | 0 | Bacteria | Bacteria\_none | Bacillota | Bacilli | Bacillales | Bacillaceae | Bacteria;Bacteria\_none;Bacillota;Bacilli;Bacillales;Bacillaceae;Pseudalkalibacillus |
| Psychrobacillus | 1 | 0 | 0 | 0 | 0 | 0 | 0 | 0 | 0 | 0 | 0 | 0 | 0 | 0 | Bacteria | Bacteria\_none | Bacillota | Bacilli | Bacillales | Bacillaceae | Bacteria;Bacteria\_none;Bacillota;Bacilli;Bacillales;Bacillaceae;Psychrobacillus |
| Rossellomorea | 0 | 0 | 0 | 0 | 0 | 1 | 0 | 0 | 0 | 0 | 0 | 0 | 0 | 0 | Bacteria | Bacteria\_none | Bacillota | Bacilli | Bacillales | Bacillaceae | Bacteria;Bacteria\_none;Bacillota;Bacilli;Bacillales;Bacillaceae;Rossellomorea |
| Salipaludibacillus | 0 | 0 | 0 | 0 | 0 | 0 | 0 | 0 | 0 | 0 | 0 | 0 | 0 | 0 | Bacteria | Bacteria\_none | Bacillota | Bacilli | Bacillales | Bacillaceae | Bacteria;Bacteria\_none;Bacillota;Bacilli;Bacillales;Bacillaceae;Salipaludibacillus |
| Shouchella | 0 | 0 | 0 | 0 | 0 | 0 | 0 | 0 | 0 | 0 | 0 | 0 | 0 | 0 | Bacteria | Bacteria\_none | Bacillota | Bacilli | Bacillales | Bacillaceae | Bacteria;Bacteria\_none;Bacillota;Bacilli;Bacillales;Bacillaceae;Shouchella |
| Sutcliffiella | 0 | 0 | 0 | 1 | 0 | 0 | 0 | 0 | 0 | 0 | 0 | 0 | 0 | 0 | Bacteria | Bacteria\_none | Bacillota | Bacilli | Bacillales | Bacillaceae | Bacteria;Bacteria\_none;Bacillota;Bacilli;Bacillales;Bacillaceae;Sutcliffiella |
| Terribacillus | 0 | 0 | 2 | 0 | 0 | 0 | 0 | 0 | 0 | 0 | 0 | 0 | 0 | 0 | Bacteria | Bacteria\_none | Bacillota | Bacilli | Bacillales | Bacillaceae | Bacteria;Bacteria\_none;Bacillota;Bacilli;Bacillales;Bacillaceae;Terribacillus |
| Virgibacillus | 0 | 0 | 0 | 0 | 0 | 0 | 0 | 1 | 0 | 0 | 0 | 0 | 0 | 1 | Bacteria | Bacteria\_none | Bacillota | Bacilli | Bacillales | Bacillaceae | Bacteria;Bacteria\_none;Bacillota;Bacilli;Bacillales;Bacillaceae;Virgibacillus |
| Exiguobacterium | 0 | 0 | 0 | 1 | 0 | 0 | 0 | 0 | 0 | 0 | 0 | 0 | 0 | 0 | Bacteria | Bacteria\_none | Bacillota | Bacilli | Bacillales | Bacillales\_Incertae\_sedis | Bacteria;Bacteria\_none;Bacillota;Bacilli;Bacillales;Bacillales\_Incertae\_sedis;Exiguobacterium |
| Gemella | 62 | 118 | 7 | 72 | 331 | 253 | 43 | 28 | 2 | 210 | 524 | 41 | 107 | 65 | Bacteria | Bacteria\_none | Bacillota | Bacilli | Bacillales | Gemellaceae | Bacteria;Bacteria\_none;Bacillota;Bacilli;Bacillales;Gemellaceae;Gemella |
| Brochothrix | 0 | 0 | 0 | 0 | 0 | 0 | 0 | 0 | 0 | 0 | 0 | 0 | 0 | 0 | Bacteria | Bacteria\_none | Bacillota | Bacilli | Bacillales | Listeriaceae | Bacteria;Bacteria\_none;Bacillota;Bacilli;Bacillales;Listeriaceae;Brochothrix |
| Listeria | 0 | 0 | 0 | 0 | 0 | 0 | 0 | 0 | 0 | 0 | 0 | 0 | 0 | 1 | Bacteria | Bacteria\_none | Bacillota | Bacilli | Bacillales | Listeriaceae | Bacteria;Bacteria\_none;Bacillota;Bacilli;Bacillales;Listeriaceae;Listeria |
| Brevibacillus | 0 | 0 | 0 | 1 | 0 | 1 | 0 | 0 | 0 | 0 | 0 | 0 | 0 | 0 | Bacteria | Bacteria\_none | Bacillota | Bacilli | Bacillales | Paenibacillaceae | Bacteria;Bacteria\_none;Bacillota;Bacilli;Bacillales;Paenibacillaceae;Brevibacillus |
| Paenibacillus | 2 | 1 | 2 | 0 | 0 | 0 | 3 | 0 | 0 | 0 | 2 | 0 | 0 | 0 | Bacteria | Bacteria\_none | Bacillota | Bacilli | Bacillales | Paenibacillaceae | Bacteria;Bacteria\_none;Bacillota;Bacilli;Bacillales;Paenibacillaceae;Paenibacillus |
| Paenisporosarcina | 0 | 0 | 0 | 0 | 0 | 0 | 0 | 0 | 0 | 0 | 0 | 0 | 0 | 0 | Bacteria | Bacteria\_none | Bacillota | Bacilli | Bacillales | Planococcaceae | Bacteria;Bacteria\_none;Bacillota;Bacilli;Bacillales;Planococcaceae;Paenisporosarcina |
| Sporosarcina | 1 | 0 | 0 | 0 | 0 | 0 | 0 | 0 | 0 | 0 | 0 | 0 | 0 | 0 | Bacteria | Bacteria\_none | Bacillota | Bacilli | Bacillales | Planococcaceae | Bacteria;Bacteria\_none;Bacillota;Bacilli;Bacillales;Planococcaceae;Sporosarcina |
| Macrococcus | 1 | 0 | 0 | 0 | 0 | 0 | 0 | 0 | 0 | 0 | 0 | 0 | 0 | 0 | Bacteria | Bacteria\_none | Bacillota | Bacilli | Bacillales | Staphylococcaceae | Bacteria;Bacteria\_none;Bacillota;Bacilli;Bacillales;Staphylococcaceae;Macrococcus |
| Staphylococcus | 0 | 3 | 2 | 2 | 2 | 0 | 0 | 0 | 0 | 1 | 0 | 2 | 1 | 0 | Bacteria | Bacteria\_none | Bacillota | Bacilli | Bacillales | Staphylococcaceae | Bacteria;Bacteria\_none;Bacillota;Bacilli;Bacillales;Staphylococcaceae;Staphylococcus |
| Abiotrophia | 7 | 0 | 56 | 3 | 43 | 15 | 8 | 0 | 0 | 12 | 34 | 2 | 8 | 6 | Bacteria | Bacteria\_none | Bacillota | Bacilli | Lactobacillales | Aerococcaceae | Bacteria;Bacteria\_none;Bacillota;Bacilli;Lactobacillales;Aerococcaceae;Abiotrophia |
| Aerococcus | 1 | 0 | 0 | 0 | 0 | 0 | 0 | 0 | 0 | 0 | 0 | 0 | 0 | 0 | Bacteria | Bacteria\_none | Bacillota | Bacilli | Lactobacillales | Aerococcaceae | Bacteria;Bacteria\_none;Bacillota;Bacilli;Lactobacillales;Aerococcaceae;Aerococcus |
| Ruoffia | 0 | 1 | 0 | 0 | 0 | 0 | 0 | 0 | 0 | 0 | 0 | 0 | 0 | 0 | Bacteria | Bacteria\_none | Bacillota | Bacilli | Lactobacillales | Aerococcaceae | Bacteria;Bacteria\_none;Bacillota;Bacilli;Lactobacillales;Aerococcaceae;Ruoffia |
| Dolosigranulum | 1 | 0 | 0 | 0 | 0 | 0 | 0 | 0 | 0 | 1 | 0 | 0 | 1 | 0 | Bacteria | Bacteria\_none | Bacillota | Bacilli | Lactobacillales | Carnobacteriaceae | Bacteria;Bacteria\_none;Bacillota;Bacilli;Lactobacillales;Carnobacteriaceae;Dolosigranulum |
| Granulicatella | 384 | 60 | 387 | 81 | 118 | 99 | 169 | 8 | 0 | 101 | 69 | 15 | 82 | 49 | Bacteria | Bacteria\_none | Bacillota | Bacilli | Lactobacillales | Carnobacteriaceae | Bacteria;Bacteria\_none;Bacillota;Bacilli;Lactobacillales;Carnobacteriaceae;Granulicatella |
| Jeotgalibaca | 1 | 0 | 0 | 0 | 0 | 0 | 0 | 0 | 0 | 0 | 0 | 0 | 0 | 0 | Bacteria | Bacteria\_none | Bacillota | Bacilli | Lactobacillales | Carnobacteriaceae | Bacteria;Bacteria\_none;Bacillota;Bacilli;Lactobacillales;Carnobacteriaceae;Jeotgalibaca |
| Enterococcus | 2 | 8 | 10 | 1 | 2 | 5 | 0 | 0 | 0 | 0 | 4 | 0 | 0 | 0 | Bacteria | Bacteria\_none | Bacillota | Bacilli | Lactobacillales | Enterococcaceae | Bacteria;Bacteria\_none;Bacillota;Bacilli;Lactobacillales;Enterococcaceae;Enterococcus |
| Tetragenococcus | 0 | 0 | 0 | 0 | 0 | 0 | 2 | 0 | 0 | 0 | 0 | 0 | 0 | 0 | Bacteria | Bacteria\_none | Bacillota | Bacilli | Lactobacillales | Enterococcaceae | Bacteria;Bacteria\_none;Bacillota;Bacilli;Lactobacillales;Enterococcaceae;Tetragenococcus |
| Vagococcus | 1 | 1 | 1 | 0 | 0 | 0 | 0 | 0 | 0 | 0 | 0 | 0 | 0 | 0 | Bacteria | Bacteria\_none | Bacillota | Bacilli | Lactobacillales | Enterococcaceae | Bacteria;Bacteria\_none;Bacillota;Bacilli;Lactobacillales;Enterococcaceae;Vagococcus |
| Lacticaseibacillus | 0 | 0 | 0 | 0 | 0 | 0 | 7 | 0 | 0 | 2 | 0 | 0 | 0 | 0 | Bacteria | Bacteria\_none | Bacillota | Bacilli | Lactobacillales | Lactobacillaceae | Bacteria;Bacteria\_none;Bacillota;Bacilli;Lactobacillales;Lactobacillaceae;Lacticaseibacillus |
| Lactobacillus | 1 | 0 | 0 | 0 | 0 | 0 | 6 | 0 | 0 | 1 | 1 | 0 | 0 | 0 | Bacteria | Bacteria\_none | Bacillota | Bacilli | Lactobacillales | Lactobacillaceae | Bacteria;Bacteria\_none;Bacillota;Bacilli;Lactobacillales;Lactobacillaceae;Lactobacillus |
| Latilactobacillus | 0 | 0 | 1 | 0 | 0 | 0 | 0 | 0 | 0 | 0 | 1 | 0 | 0 | 1 | Bacteria | Bacteria\_none | Bacillota | Bacilli | Lactobacillales | Lactobacillaceae | Bacteria;Bacteria\_none;Bacillota;Bacilli;Lactobacillales;Lactobacillaceae;Latilactobacillus |
| Lentilactobacillus | 0 | 0 | 0 | 0 | 1 | 0 | 0 | 0 | 0 | 0 | 0 | 0 | 0 | 0 | Bacteria | Bacteria\_none | Bacillota | Bacilli | Lactobacillales | Lactobacillaceae | Bacteria;Bacteria\_none;Bacillota;Bacilli;Lactobacillales;Lactobacillaceae;Lentilactobacillus |
| Leuconostoc | 0 | 0 | 0 | 0 | 0 | 1 | 0 | 0 | 0 | 0 | 0 | 0 | 0 | 0 | Bacteria | Bacteria\_none | Bacillota | Bacilli | Lactobacillales | Lactobacillaceae | Bacteria;Bacteria\_none;Bacillota;Bacilli;Lactobacillales;Lactobacillaceae;Leuconostoc |
| Levilactobacillus | 0 | 0 | 1 | 0 | 0 | 0 | 0 | 0 | 0 | 0 | 0 | 0 | 0 | 0 | Bacteria | Bacteria\_none | Bacillota | Bacilli | Lactobacillales | Lactobacillaceae | Bacteria;Bacteria\_none;Bacillota;Bacilli;Lactobacillales;Lactobacillaceae;Levilactobacillus |
| Ligilactobacillus | 1 | 0 | 0 | 0 | 1 | 0 | 0 | 1 | 1 | 0 | 0 | 0 | 0 | 0 | Bacteria | Bacteria\_none | Bacillota | Bacilli | Lactobacillales | Lactobacillaceae | Bacteria;Bacteria\_none;Bacillota;Bacilli;Lactobacillales;Lactobacillaceae;Ligilactobacillus |
| Limosilactobacillus | 0 | 0 | 0 | 0 | 0 | 0 | 0 | 0 | 0 | 0 | 0 | 0 | 0 | 0 | Bacteria | Bacteria\_none | Bacillota | Bacilli | Lactobacillales | Lactobacillaceae | Bacteria;Bacteria\_none;Bacillota;Bacilli;Lactobacillales;Lactobacillaceae;Limosilactobacillus |
| Liquorilactobacillus | 0 | 0 | 0 | 0 | 0 | 0 | 0 | 1 | 0 | 0 | 0 | 0 | 0 | 0 | Bacteria | Bacteria\_none | Bacillota | Bacilli | Lactobacillales | Lactobacillaceae | Bacteria;Bacteria\_none;Bacillota;Bacilli;Lactobacillales;Lactobacillaceae;Liquorilactobacillus |
| Nicoliella | 0 | 0 | 0 | 0 | 0 | 0 | 0 | 0 | 0 | 0 | 0 | 0 | 1 | 0 | Bacteria | Bacteria\_none | Bacillota | Bacilli | Lactobacillales | Lactobacillaceae | Bacteria;Bacteria\_none;Bacillota;Bacilli;Lactobacillales;Lactobacillaceae;Nicoliella |
| Pediococcus | 0 | 0 | 1 | 0 | 0 | 0 | 0 | 0 | 0 | 0 | 0 | 0 | 0 | 0 | Bacteria | Bacteria\_none | Bacillota | Bacilli | Lactobacillales | Lactobacillaceae | Bacteria;Bacteria\_none;Bacillota;Bacilli;Lactobacillales;Lactobacillaceae;Pediococcus |
| Weissella | 0 | 0 | 0 | 0 | 0 | 0 | 0 | 0 | 0 | 0 | 0 | 0 | 0 | 0 | Bacteria | Bacteria\_none | Bacillota | Bacilli | Lactobacillales | Lactobacillaceae | Bacteria;Bacteria\_none;Bacillota;Bacilli;Lactobacillales;Lactobacillaceae;Weissella |
| Lactococcus | 0 | 1 | 0 | 0 | 0 | 5 | 0 | 0 | 0 | 1 | 0 | 0 | 0 | 1 | Bacteria | Bacteria\_none | Bacillota | Bacilli | Lactobacillales | Streptococcaceae | Bacteria;Bacteria\_none;Bacillota;Bacilli;Lactobacillales;Streptococcaceae;Lactococcus |
| Streptococcus | 1619 | 1298 | 650 | 229 | 1434 | 1302 | 743 | 98 | 221 | 514 | 1177 | 404 | 515 | 350 | Bacteria | Bacteria\_none | Bacillota | Bacilli | Lactobacillales | Streptococcaceae | Bacteria;Bacteria\_none;Bacillota;Bacilli;Lactobacillales;Streptococcaceae;Streptococcus |
| Caldicellulosiruptor | 0 | 0 | 0 | 0 | 1 | 0 | 0 | 0 | 0 | 0 | 0 | 0 | 0 | 0 | Bacteria | Bacteria\_none | Bacillota | Bacillota\_Incertae\_sedis | Caldicellulosiruptorales | Caldicellulosiruptoraceae | Bacteria;Bacteria\_none;Bacillota;Bacillota\_Incertae\_sedis;Caldicellulosiruptorales;Caldicellulosiruptoraceae;Caldicellulosiruptor |
| Aristaeella | 0 | 0 | 0 | 0 | 0 | 0 | 0 | 0 | 0 | 0 | 0 | 0 | 0 | 0 | Bacteria | Bacteria\_none | Bacillota | Clostridia | Eubacteriales | Aristaeellaceae | Bacteria;Bacteria\_none;Bacillota;Clostridia;Eubacteriales;Aristaeellaceae;Aristaeella |
| Clostridium | 1 | 2 | 3 | 3 | 2 | 6 | 0 | 0 | 2 | 0 | 0 | 0 | 4 | 0 | Bacteria | Bacteria\_none | Bacillota | Clostridia | Eubacteriales | Clostridiaceae | Bacteria;Bacteria\_none;Bacillota;Clostridia;Eubacteriales;Clostridiaceae;Clostridium |
| Crassaminicella | 0 | 0 | 0 | 0 | 0 | 3 | 0 | 0 | 0 | 0 | 0 | 0 | 0 | 0 | Bacteria | Bacteria\_none | Bacillota | Clostridia | Eubacteriales | Clostridiaceae | Bacteria;Bacteria\_none;Bacillota;Clostridia;Eubacteriales;Clostridiaceae;Crassaminicella |
| Oceanirhabdus | 0 | 0 | 0 | 0 | 0 | 1 | 0 | 0 | 0 | 0 | 0 | 0 | 0 | 0 | Bacteria | Bacteria\_none | Bacillota | Clostridia | Eubacteriales | Clostridiaceae | Bacteria;Bacteria\_none;Bacillota;Clostridia;Eubacteriales;Clostridiaceae;Oceanirhabdus |
| Sarcina | 0 | 0 | 0 | 0 | 0 | 1 | 0 | 0 | 1 | 0 | 0 | 0 | 0 | 0 | Bacteria | Bacteria\_none | Bacillota | Clostridia | Eubacteriales | Clostridiaceae | Bacteria;Bacteria\_none;Bacillota;Clostridia;Eubacteriales;Clostridiaceae;Sarcina |
| Carboxydocella | 0 | 0 | 0 | 0 | 0 | 0 | 0 | 0 | 0 | 0 | 0 | 0 | 0 | 0 | Bacteria | Bacteria\_none | Bacillota | Clostridia | Eubacteriales | Clostridiales Family XVI. Incertae Sedis | Bacteria;Bacteria\_none;Bacillota;Clostridia;Eubacteriales;Clostridiales Family XVI. Incertae Sedis;Carboxydocella |
| Dehalobacter | 0 | 0 | 0 | 0 | 0 | 0 | 0 | 0 | 0 | 0 | 0 | 0 | 0 | 0 | Bacteria | Bacteria\_none | Bacillota | Clostridia | Eubacteriales | Desulfitobacteriaceae | Bacteria;Bacteria\_none;Bacillota;Clostridia;Eubacteriales;Desulfitobacteriaceae;Dehalobacter |
| Desulfosporosinus | 0 | 0 | 0 | 0 | 0 | 1 | 0 | 0 | 1 | 0 | 0 | 0 | 0 | 0 | Bacteria | Bacteria\_none | Bacillota | Clostridia | Eubacteriales | Desulfitobacteriaceae | Bacteria;Bacteria\_none;Bacillota;Clostridia;Eubacteriales;Desulfitobacteriaceae;Desulfosporosinus |
| Acetobacterium | 0 | 0 | 1 | 0 | 0 | 0 | 0 | 0 | 0 | 0 | 0 | 0 | 0 | 0 | Bacteria | Bacteria\_none | Bacillota | Clostridia | Eubacteriales | Eubacteriaceae | Bacteria;Bacteria\_none;Bacillota;Clostridia;Eubacteriales;Eubacteriaceae;Acetobacterium |
| Eubacterium | 0 | 0 | 0 | 0 | 0 | 1 | 0 | 0 | 0 | 0 | 0 | 0 | 0 | 0 | Bacteria | Bacteria\_none | Bacillota | Clostridia | Eubacteriales | Eubacteriaceae | Bacteria;Bacteria\_none;Bacillota;Clostridia;Eubacteriales;Eubacteriaceae;Eubacterium |
| Sinanaerobacter | 0 | 0 | 0 | 0 | 0 | 0 | 0 | 0 | 0 | 0 | 0 | 0 | 0 | 0 | Bacteria | Bacteria\_none | Bacillota | Clostridia | Eubacteriales | Eubacteriales Family XIII. Incertae Sedis | Bacteria;Bacteria\_none;Bacillota;Clostridia;Eubacteriales;Eubacteriales Family XIII. Incertae Sedis;Sinanaerobacter |
| Fenollaria | 0 | 0 | 0 | 0 | 0 | 0 | 0 | 0 | 0 | 0 | 1 | 0 | 0 | 0 | Bacteria | Bacteria\_none | Bacillota | Clostridia | Eubacteriales | Eubacteriales\_Incertae\_sedis | Bacteria;Bacteria\_none;Bacillota;Clostridia;Eubacteriales;Eubacteriales\_Incertae\_sedis;Fenollaria |
| Massilistercora | 0 | 0 | 0 | 0 | 0 | 2 | 0 | 0 | 0 | 0 | 0 | 0 | 0 | 0 | Bacteria | Bacteria\_none | Bacillota | Clostridia | Eubacteriales | Eubacteriales\_Incertae\_sedis | Bacteria;Bacteria\_none;Bacillota;Clostridia;Eubacteriales;Eubacteriales\_Incertae\_sedis;Massilistercora |
| Acetivibrio | 0 | 0 | 0 | 0 | 0 | 0 | 0 | 0 | 0 | 0 | 1 | 0 | 0 | 0 | Bacteria | Bacteria\_none | Bacillota | Clostridia | Eubacteriales | Oscillospiraceae | Bacteria;Bacteria\_none;Bacillota;Clostridia;Eubacteriales;Oscillospiraceae;Acetivibrio |
| Amygdalobacter | 0 | 0 | 1 | 0 | 1 | 1 | 0 | 0 | 0 | 0 | 0 | 0 | 0 | 0 | Bacteria | Bacteria\_none | Bacillota | Clostridia | Eubacteriales | Oscillospiraceae | Bacteria;Bacteria\_none;Bacillota;Clostridia;Eubacteriales;Oscillospiraceae;Amygdalobacter |
| Caproicibacterium | 1 | 0 | 0 | 0 | 0 | 2 | 1 | 0 | 0 | 0 | 0 | 0 | 0 | 0 | Bacteria | Bacteria\_none | Bacillota | Clostridia | Eubacteriales | Oscillospiraceae | Bacteria;Bacteria\_none;Bacillota;Clostridia;Eubacteriales;Oscillospiraceae;Caproicibacterium |
| Ethanoligenens | 0 | 0 | 0 | 0 | 0 | 0 | 0 | 0 | 0 | 0 | 0 | 0 | 0 | 0 | Bacteria | Bacteria\_none | Bacillota | Clostridia | Eubacteriales | Oscillospiraceae | Bacteria;Bacteria\_none;Bacillota;Clostridia;Eubacteriales;Oscillospiraceae;Ethanoligenens |
| Faecalibacterium | 0 | 1 | 1 | 0 | 0 | 0 | 0 | 0 | 0 | 0 | 0 | 0 | 0 | 0 | Bacteria | Bacteria\_none | Bacillota | Clostridia | Eubacteriales | Oscillospiraceae | Bacteria;Bacteria\_none;Bacillota;Clostridia;Eubacteriales;Oscillospiraceae;Faecalibacterium |
| Fastidiosipila | 2 | 0 | 0 | 0 | 0 | 1 | 0 | 0 | 0 | 0 | 0 | 0 | 0 | 0 | Bacteria | Bacteria\_none | Bacillota | Clostridia | Eubacteriales | Oscillospiraceae | Bacteria;Bacteria\_none;Bacillota;Clostridia;Eubacteriales;Oscillospiraceae;Fastidiosipila |
| Mageeibacillus | 0 | 0 | 0 | 0 | 0 | 0 | 0 | 0 | 0 | 0 | 0 | 0 | 0 | 0 | Bacteria | Bacteria\_none | Bacillota | Clostridia | Eubacteriales | Oscillospiraceae | Bacteria;Bacteria\_none;Bacillota;Clostridia;Eubacteriales;Oscillospiraceae;Mageeibacillus |
| Paludihabitans | 0 | 0 | 0 | 0 | 0 | 1 | 0 | 0 | 0 | 0 | 0 | 0 | 0 | 0 | Bacteria | Bacteria\_none | Bacillota | Clostridia | Eubacteriales | Oscillospiraceae | Bacteria;Bacteria\_none;Bacillota;Clostridia;Eubacteriales;Oscillospiraceae;Paludihabitans |
| Petroclostridium | 0 | 1 | 0 | 0 | 0 | 0 | 0 | 0 | 0 | 0 | 0 | 0 | 0 | 0 | Bacteria | Bacteria\_none | Bacillota | Clostridia | Eubacteriales | Oscillospiraceae | Bacteria;Bacteria\_none;Bacillota;Clostridia;Eubacteriales;Oscillospiraceae;Petroclostridium |
| Ruminiclostridium | 0 | 0 | 0 | 1 | 0 | 0 | 0 | 0 | 0 | 0 | 0 | 0 | 0 | 0 | Bacteria | Bacteria\_none | Bacillota | Clostridia | Eubacteriales | Oscillospiraceae | Bacteria;Bacteria\_none;Bacillota;Clostridia;Eubacteriales;Oscillospiraceae;Ruminiclostridium |
| Ruminococcus | 0 | 1 | 0 | 0 | 0 | 0 | 0 | 0 | 0 | 0 | 0 | 0 | 0 | 0 | Bacteria | Bacteria\_none | Bacillota | Clostridia | Eubacteriales | Oscillospiraceae | Bacteria;Bacteria\_none;Bacillota;Clostridia;Eubacteriales;Oscillospiraceae;Ruminococcus |
| Thermoclostridium | 0 | 0 | 0 | 0 | 0 | 1 | 0 | 0 | 0 | 0 | 0 | 0 | 0 | 0 | Bacteria | Bacteria\_none | Bacillota | Clostridia | Eubacteriales | Oscillospiraceae | Bacteria;Bacteria\_none;Bacillota;Clostridia;Eubacteriales;Oscillospiraceae;Thermoclostridium |
| Halobacteroides | 0 | 0 | 0 | 0 | 0 | 0 | 0 | 0 | 0 | 0 | 0 | 0 | 0 | 0 | Bacteria | Bacteria\_none | Bacillota | Clostridia | Halanaerobiales | Halobacteroidaceae | Bacteria;Bacteria\_none;Bacillota;Clostridia;Halanaerobiales;Halobacteroidaceae;Halobacteroides |
| Anaerotignum | 0 | 0 | 0 | 0 | 0 | 0 | 0 | 0 | 0 | 0 | 0 | 0 | 0 | 0 | Bacteria | Bacteria\_none | Bacillota | Clostridia | Lachnospirales | Anaerotignaceae | Bacteria;Bacteria\_none;Bacillota;Clostridia;Lachnospirales;Anaerotignaceae;Anaerotignum |
| Agathobacter | 0 | 0 | 1 | 0 | 0 | 0 | 0 | 0 | 0 | 0 | 0 | 0 | 0 | 0 | Bacteria | Bacteria\_none | Bacillota | Clostridia | Lachnospirales | Lachnospiraceae | Bacteria;Bacteria\_none;Bacillota;Clostridia;Lachnospirales;Lachnospiraceae;Agathobacter |
| Anaerobutyricum | 0 | 0 | 0 | 0 | 1 | 0 | 0 | 0 | 0 | 0 | 0 | 0 | 0 | 0 | Bacteria | Bacteria\_none | Bacillota | Clostridia | Lachnospirales | Lachnospiraceae | Bacteria;Bacteria\_none;Bacillota;Clostridia;Lachnospirales;Lachnospiraceae;Anaerobutyricum |
| Anaerocolumna | 0 | 0 | 0 | 0 | 0 | 3 | 0 | 0 | 0 | 0 | 0 | 0 | 0 | 0 | Bacteria | Bacteria\_none | Bacillota | Clostridia | Lachnospirales | Lachnospiraceae | Bacteria;Bacteria\_none;Bacillota;Clostridia;Lachnospirales;Lachnospiraceae;Anaerocolumna |
| Anaeromicropila | 0 | 0 | 1 | 0 | 0 | 0 | 0 | 0 | 0 | 0 | 0 | 0 | 0 | 0 | Bacteria | Bacteria\_none | Bacillota | Clostridia | Lachnospirales | Lachnospiraceae | Bacteria;Bacteria\_none;Bacillota;Clostridia;Lachnospirales;Lachnospiraceae;Anaeromicropila |
| Anaerostipes | 0 | 0 | 0 | 0 | 0 | 0 | 0 | 0 | 0 | 0 | 0 | 0 | 0 | 0 | Bacteria | Bacteria\_none | Bacillota | Clostridia | Lachnospirales | Lachnospiraceae | Bacteria;Bacteria\_none;Bacillota;Clostridia;Lachnospirales;Lachnospiraceae;Anaerostipes |
| Blautia | 0 | 0 | 0 | 0 | 0 | 4 | 0 | 0 | 0 | 0 | 0 | 0 | 0 | 1 | Bacteria | Bacteria\_none | Bacillota | Clostridia | Lachnospirales | Lachnospiraceae | Bacteria;Bacteria\_none;Bacillota;Clostridia;Lachnospirales;Lachnospiraceae;Blautia |
| Butyrivibrio | 0 | 0 | 0 | 0 | 0 | 1 | 0 | 0 | 0 | 0 | 0 | 0 | 0 | 0 | Bacteria | Bacteria\_none | Bacillota | Clostridia | Lachnospirales | Lachnospiraceae | Bacteria;Bacteria\_none;Bacillota;Clostridia;Lachnospirales;Lachnospiraceae;Butyrivibrio |
| Candidatus Epulonipiscium | 0 | 0 | 0 | 0 | 0 | 0 | 0 | 0 | 0 | 0 | 0 | 0 | 0 | 0 | Bacteria | Bacteria\_none | Bacillota | Clostridia | Lachnospirales | Lachnospiraceae | Bacteria;Bacteria\_none;Bacillota;Clostridia;Lachnospirales;Lachnospiraceae;Candidatus Epulonipiscium |
| Enterocloster | 0 | 0 | 0 | 0 | 0 | 0 | 0 | 0 | 0 | 0 | 0 | 0 | 0 | 0 | Bacteria | Bacteria\_none | Bacillota | Clostridia | Lachnospirales | Lachnospiraceae | Bacteria;Bacteria\_none;Bacillota;Clostridia;Lachnospirales;Lachnospiraceae;Enterocloster |
| Hungatella | 0 | 0 | 0 | 0 | 0 | 0 | 0 | 0 | 0 | 0 | 0 | 0 | 0 | 0 | Bacteria | Bacteria\_none | Bacillota | Clostridia | Lachnospirales | Lachnospiraceae | Bacteria;Bacteria\_none;Bacillota;Clostridia;Lachnospirales;Lachnospiraceae;Hungatella |
| Lachnoanaerobaculum | 1 | 23 | 6 | 10 | 5 | 21 | 0 | 0 | 0 | 2 | 0 | 1 | 5 | 1 | Bacteria | Bacteria\_none | Bacillota | Clostridia | Lachnospirales | Lachnospiraceae | Bacteria;Bacteria\_none;Bacillota;Clostridia;Lachnospirales;Lachnospiraceae;Lachnoanaerobaculum |
| Lachnoclostridium | 0 | 0 | 1 | 0 | 0 | 2 | 0 | 0 | 0 | 0 | 0 | 0 | 0 | 0 | Bacteria | Bacteria\_none | Bacillota | Clostridia | Lachnospirales | Lachnospiraceae | Bacteria;Bacteria\_none;Bacillota;Clostridia;Lachnospirales;Lachnospiraceae;Lachnoclostridium |
| Mediterraneibacter | 0 | 0 | 0 | 0 | 0 | 0 | 0 | 0 | 0 | 0 | 0 | 2 | 0 | 0 | Bacteria | Bacteria\_none | Bacillota | Clostridia | Lachnospirales | Lachnospiraceae | Bacteria;Bacteria\_none;Bacillota;Clostridia;Lachnospirales;Lachnospiraceae;Mediterraneibacter |
| Qiania | 0 | 0 | 0 | 0 | 1 | 0 | 0 | 0 | 0 | 0 | 0 | 0 | 0 | 0 | Bacteria | Bacteria\_none | Bacillota | Clostridia | Lachnospirales | Lachnospiraceae | Bacteria;Bacteria\_none;Bacillota;Clostridia;Lachnospirales;Lachnospiraceae;Qiania |
| Roseburia | 0 | 0 | 2 | 0 | 0 | 2 | 0 | 0 | 0 | 0 | 0 | 0 | 0 | 1 | Bacteria | Bacteria\_none | Bacillota | Clostridia | Lachnospirales | Lachnospiraceae | Bacteria;Bacteria\_none;Bacillota;Clostridia;Lachnospirales;Lachnospiraceae;Roseburia |
| Simiaoa | 0 | 0 | 0 | 0 | 0 | 0 | 0 | 0 | 0 | 0 | 0 | 0 | 0 | 0 | Bacteria | Bacteria\_none | Bacillota | Clostridia | Lachnospirales | Lachnospiraceae | Bacteria;Bacteria\_none;Bacillota;Clostridia;Lachnospirales;Lachnospiraceae;Simiaoa |
| Stomatobaculum | 0 | 8 | 16 | 0 | 0 | 9 | 3 | 0 | 0 | 3 | 0 | 1 | 3 | 0 | Bacteria | Bacteria\_none | Bacillota | Clostridia | Lachnospirales | Lachnospiraceae | Bacteria;Bacteria\_none;Bacillota;Clostridia;Lachnospirales;Lachnospiraceae;Stomatobaculum |
| Tyzzerella | 0 | 0 | 0 | 0 | 1 | 0 | 0 | 0 | 0 | 0 | 0 | 0 | 0 | 0 | Bacteria | Bacteria\_none | Bacillota | Clostridia | Lachnospirales | Lachnospiraceae | Bacteria;Bacteria\_none;Bacillota;Clostridia;Lachnospirales;Lachnospiraceae;Tyzzerella |
| Wansuia | 0 | 0 | 0 | 0 | 1 | 0 | 0 | 0 | 0 | 0 | 0 | 0 | 0 | 0 | Bacteria | Bacteria\_none | Bacillota | Clostridia | Lachnospirales | Lachnospiraceae | Bacteria;Bacteria\_none;Bacillota;Clostridia;Lachnospirales;Lachnospiraceae;Wansuia |
| Wujia | 0 | 0 | 0 | 0 | 0 | 0 | 0 | 0 | 0 | 0 | 0 | 0 | 0 | 0 | Bacteria | Bacteria\_none | Bacillota | Clostridia | Lachnospirales | Lachnospiraceae | Bacteria;Bacteria\_none;Bacillota;Clostridia;Lachnospirales;Lachnospiraceae;Wujia |
| Monoglobus | 0 | 0 | 0 | 0 | 0 | 0 | 0 | 0 | 0 | 0 | 0 | 0 | 0 | 0 | Bacteria | Bacteria\_none | Bacillota | Clostridia | Monoglobales | Monoglobaceae | Bacteria;Bacteria\_none;Bacillota;Clostridia;Monoglobales;Monoglobaceae;Monoglobus |
| Natranaerobius | 0 | 0 | 0 | 0 | 0 | 0 | 0 | 0 | 0 | 0 | 0 | 0 | 0 | 0 | Bacteria | Bacteria\_none | Bacillota | Clostridia | Natranaerobiales | Natranaerobiaceae | Bacteria;Bacteria\_none;Bacillota;Clostridia;Natranaerobiales;Natranaerobiaceae;Natranaerobius |
| Natranaerofaba | 0 | 0 | 0 | 0 | 0 | 1 | 0 | 0 | 0 | 0 | 0 | 0 | 0 | 0 | Bacteria | Bacteria\_none | Bacillota | Clostridia | Natranaerobiales | Natranaerofabaceae | Bacteria;Bacteria\_none;Bacillota;Clostridia;Natranaerobiales;Natranaerofabaceae;Natranaerofaba |
| Aminipila | 0 | 0 | 0 | 0 | 0 | 1 | 0 | 0 | 0 | 0 | 0 | 0 | 0 | 0 | Bacteria | Bacteria\_none | Bacillota | Clostridia | Peptostreptococcales | Anaerovoracaceae | Bacteria;Bacteria\_none;Bacillota;Clostridia;Peptostreptococcales;Anaerovoracaceae;Aminipila |
| Emergencia | 0 | 0 | 0 | 0 | 0 | 0 | 0 | 0 | 0 | 0 | 0 | 0 | 0 | 0 | Bacteria | Bacteria\_none | Bacillota | Clostridia | Peptostreptococcales | Anaerovoracaceae | Bacteria;Bacteria\_none;Bacillota;Clostridia;Peptostreptococcales;Anaerovoracaceae;Emergencia |
| Mogibacterium | 1 | 0 | 8 | 1 | 1 | 24 | 0 | 2 | 1 | 0 | 2 | 0 | 4 | 1 | Bacteria | Bacteria\_none | Bacillota | Clostridia | Peptostreptococcales | Anaerovoracaceae | Bacteria;Bacteria\_none;Bacillota;Clostridia;Peptostreptococcales;Anaerovoracaceae;Mogibacterium |
| Filifactor | 3 | 37 | 34 | 0 | 1 | 2 | 2 | 12 | 0 | 0 | 1 | 7 | 5 | 2 | Bacteria | Bacteria\_none | Bacillota | Clostridia | Peptostreptococcales | Filifactoraceae | Bacteria;Bacteria\_none;Bacillota;Clostridia;Peptostreptococcales;Filifactoraceae;Filifactor |
| Serpentinicella | 0 | 0 | 0 | 1 | 0 | 0 | 0 | 0 | 0 | 0 | 0 | 0 | 0 | 0 | Bacteria | Bacteria\_none | Bacillota | Clostridia | Peptostreptococcales | Natronincolaceae | Bacteria;Bacteria\_none;Bacillota;Clostridia;Peptostreptococcales;Natronincolaceae;Serpentinicella |
| Clostridioides | 0 | 0 | 0 | 0 | 2 | 0 | 0 | 0 | 0 | 0 | 0 | 0 | 0 | 0 | Bacteria | Bacteria\_none | Bacillota | Clostridia | Peptostreptococcales | Peptostreptococcaceae | Bacteria;Bacteria\_none;Bacillota;Clostridia;Peptostreptococcales;Peptostreptococcaceae;Clostridioides |
| Peptostreptococcus | 0 | 0 | 3 | 2 | 0 | 1 | 0 | 0 | 0 | 0 | 1 | 0 | 0 | 0 | Bacteria | Bacteria\_none | Bacillota | Clostridia | Peptostreptococcales | Peptostreptococcaceae | Bacteria;Bacteria\_none;Bacillota;Clostridia;Peptostreptococcales;Peptostreptococcaceae;Peptostreptococcus |
| Romboutsia | 0 | 1 | 0 | 1 | 0 | 0 | 0 | 0 | 0 | 0 | 0 | 0 | 0 | 0 | Bacteria | Bacteria\_none | Bacillota | Clostridia | Peptostreptococcales | Peptostreptococcaceae | Bacteria;Bacteria\_none;Bacillota;Clostridia;Peptostreptococcales;Peptostreptococcaceae;Romboutsia |
| Terrisporobacter | 1 | 0 | 0 | 0 | 2 | 0 | 0 | 0 | 0 | 0 | 2 | 0 | 0 | 1 | Bacteria | Bacteria\_none | Bacillota | Clostridia | Peptostreptococcales | Peptostreptococcaceae | Bacteria;Bacteria\_none;Bacillota;Clostridia;Peptostreptococcales;Peptostreptococcaceae;Terrisporobacter |
| Thermoanaerobacterium | 0 | 1 | 0 | 0 | 0 | 0 | 0 | 0 | 0 | 0 | 0 | 0 | 0 | 0 | Bacteria | Bacteria\_none | Bacillota | Clostridia | Thermoanaerobacterales | Thermoanaerobacteraceae | Bacteria;Bacteria\_none;Bacillota;Clostridia;Thermoanaerobacterales;Thermoanaerobacteraceae;Thermoanaerobacterium |
| Catenibacterium | 0 | 0 | 0 | 1 | 0 | 0 | 0 | 0 | 0 | 0 | 0 | 0 | 0 | 0 | Bacteria | Bacteria\_none | Bacillota | Erysipelotrichia | Erysipelotrichales | Coprobacillaceae | Bacteria;Bacteria\_none;Bacillota;Erysipelotrichia;Erysipelotrichales;Coprobacillaceae;Catenibacterium |
| Faecalibacillus | 0 | 0 | 0 | 0 | 0 | 1 | 0 | 0 | 0 | 0 | 0 | 0 | 0 | 1 | Bacteria | Bacteria\_none | Bacillota | Erysipelotrichia | Erysipelotrichales | Coprobacillaceae | Bacteria;Bacteria\_none;Bacillota;Erysipelotrichia;Erysipelotrichales;Coprobacillaceae;Faecalibacillus |
| Thomasclavelia | 0 | 0 | 0 | 0 | 0 | 0 | 0 | 0 | 0 | 0 | 0 | 0 | 0 | 0 | Bacteria | Bacteria\_none | Bacillota | Erysipelotrichia | Erysipelotrichales | Coprobacillaceae | Bacteria;Bacteria\_none;Bacillota;Erysipelotrichia;Erysipelotrichales;Coprobacillaceae;Thomasclavelia |
| Bulleidia | 0 | 0 | 0 | 0 | 0 | 0 | 0 | 0 | 0 | 0 | 2 | 0 | 0 | 0 | Bacteria | Bacteria\_none | Bacillota | Erysipelotrichia | Erysipelotrichales | Erysipelotrichaceae | Bacteria;Bacteria\_none;Bacillota;Erysipelotrichia;Erysipelotrichales;Erysipelotrichaceae;Bulleidia |
| Erysipelothrix | 0 | 1 | 0 | 0 | 0 | 1 | 0 | 0 | 0 | 0 | 0 | 0 | 0 | 0 | Bacteria | Bacteria\_none | Bacillota | Erysipelotrichia | Erysipelotrichales | Erysipelotrichaceae | Bacteria;Bacteria\_none;Bacillota;Erysipelotrichia;Erysipelotrichales;Erysipelotrichaceae;Erysipelothrix |
| Erysipelotrichaceae\_Incertae\_sedis | 0 | 0 | 0 | 0 | 0 | 0 | 0 | 0 | 0 | 1 | 0 | 0 | 1 | 0 | Bacteria | Bacteria\_none | Bacillota | Erysipelotrichia | Erysipelotrichales | Erysipelotrichaceae | Bacteria;Bacteria\_none;Bacillota;Erysipelotrichia;Erysipelotrichales;Erysipelotrichaceae;Erysipelotrichaceae\_Incertae\_sedis |
| Solobacterium | 14 | 16 | 85 | 6 | 10 | 372 | 12 | 4 | 0 | 2 | 5 | 0 | 29 | 11 | Bacteria | Bacteria\_none | Bacillota | Erysipelotrichia | Erysipelotrichales | Erysipelotrichaceae | Bacteria;Bacteria\_none;Bacillota;Erysipelotrichia;Erysipelotrichales;Erysipelotrichaceae;Solobacterium |
| Megamonas | 0 | 0 | 1 | 0 | 0 | 0 | 0 | 0 | 0 | 0 | 1 | 0 | 0 | 0 | Bacteria | Bacteria\_none | Bacillota | Negativicutes | Selenomonadales | Selenomonadaceae | Bacteria;Bacteria\_none;Bacillota;Negativicutes;Selenomonadales;Selenomonadaceae;Megamonas |
| Pectinatus | 0 | 0 | 0 | 0 | 0 | 0 | 0 | 0 | 0 | 0 | 0 | 0 | 0 | 0 | Bacteria | Bacteria\_none | Bacillota | Negativicutes | Selenomonadales | Selenomonadaceae | Bacteria;Bacteria\_none;Bacillota;Negativicutes;Selenomonadales;Selenomonadaceae;Pectinatus |
| Selenomonas | 5 | 7 | 12 | 0 | 6 | 10 | 1 | 2 | 0 | 1 | 1 | 4 | 2 | 2 | Bacteria | Bacteria\_none | Bacillota | Negativicutes | Selenomonadales | Selenomonadaceae | Bacteria;Bacteria\_none;Bacillota;Negativicutes;Selenomonadales;Selenomonadaceae;Selenomonas |
| Sporomusa | 0 | 0 | 0 | 0 | 0 | 0 | 0 | 0 | 0 | 0 | 0 | 0 | 0 | 0 | Bacteria | Bacteria\_none | Bacillota | Negativicutes | Selenomonadales | Sporomusaceae | Bacteria;Bacteria\_none;Bacillota;Negativicutes;Selenomonadales;Sporomusaceae;Sporomusa |
| Dialister | 0 | 0 | 1 | 0 | 0 | 0 | 0 | 0 | 0 | 0 | 0 | 1 | 1 | 0 | Bacteria | Bacteria\_none | Bacillota | Negativicutes | Veillonellales | Veillonellaceae | Bacteria;Bacteria\_none;Bacillota;Negativicutes;Veillonellales;Veillonellaceae;Dialister |
| Megasphaera | 0 | 0 | 0 | 0 | 0 | 0 | 0 | 0 | 0 | 0 | 1 | 0 | 0 | 0 | Bacteria | Bacteria\_none | Bacillota | Negativicutes | Veillonellales | Veillonellaceae | Bacteria;Bacteria\_none;Bacillota;Negativicutes;Veillonellales;Veillonellaceae;Megasphaera |
| Veillonella | 1044 | 75 | 32 | 59 | 41 | 43 | 8 | 22 | 23 | 80 | 3 | 57 | 86 | 47 | Bacteria | Bacteria\_none | Bacillota | Negativicutes | Veillonellales | Veillonellaceae | Bacteria;Bacteria\_none;Bacillota;Negativicutes;Veillonellales;Veillonellaceae;Veillonella |
| Acidilutibacter | 0 | 0 | 0 | 0 | 1 | 0 | 0 | 0 | 0 | 0 | 0 | 0 | 0 | 0 | Bacteria | Bacteria\_none | Bacillota | Tissierellia | Tissierellales | Acidilutibacteraceae | Bacteria;Bacteria\_none;Bacillota;Tissierellia;Tissierellales;Acidilutibacteraceae;Acidilutibacter |
| Gottschalkia | 0 | 0 | 0 | 0 | 0 | 0 | 0 | 0 | 0 | 0 | 0 | 0 | 0 | 0 | Bacteria | Bacteria\_none | Bacillota | Tissierellia | Tissierellales | Gottschalkiaceae | Bacteria;Bacteria\_none;Bacillota;Tissierellia;Tissierellales;Gottschalkiaceae;Gottschalkia |
| Anaerococcus | 1 | 0 | 0 | 0 | 0 | 0 | 0 | 0 | 0 | 0 | 1 | 0 | 0 | 0 | Bacteria | Bacteria\_none | Bacillota | Tissierellia | Tissierellales | Peptoniphilaceae | Bacteria;Bacteria\_none;Bacillota;Tissierellia;Tissierellales;Peptoniphilaceae;Anaerococcus |
| Finegoldia | 0 | 0 | 0 | 0 | 0 | 1 | 0 | 0 | 0 | 0 | 1 | 0 | 0 | 0 | Bacteria | Bacteria\_none | Bacillota | Tissierellia | Tissierellales | Peptoniphilaceae | Bacteria;Bacteria\_none;Bacillota;Tissierellia;Tissierellales;Peptoniphilaceae;Finegoldia |
| Helcococcus | 0 | 0 | 0 | 1 | 0 | 0 | 0 | 0 | 0 | 0 | 0 | 1 | 0 | 0 | Bacteria | Bacteria\_none | Bacillota | Tissierellia | Tissierellales | Peptoniphilaceae | Bacteria;Bacteria\_none;Bacillota;Tissierellia;Tissierellales;Peptoniphilaceae;Helcococcus |
| Miniphocaeibacter | 0 | 0 | 0 | 0 | 0 | 0 | 0 | 0 | 0 | 0 | 0 | 0 | 0 | 0 | Bacteria | Bacteria\_none | Bacillota | Tissierellia | Tissierellales | Peptoniphilaceae | Bacteria;Bacteria\_none;Bacillota;Tissierellia;Tissierellales;Peptoniphilaceae;Miniphocaeibacter |
| Parvimonas | 4 | 33 | 22 | 7 | 33 | 49 | 0 | 20 | 0 | 0 | 37 | 12 | 14 | 11 | Bacteria | Bacteria\_none | Bacillota | Tissierellia | Tissierellales | Peptoniphilaceae | Bacteria;Bacteria\_none;Bacillota;Tissierellia;Tissierellales;Peptoniphilaceae;Parvimonas |
| Peptoniphilus | 0 | 0 | 0 | 0 | 0 | 1 | 0 | 0 | 0 | 0 | 0 | 0 | 0 | 0 | Bacteria | Bacteria\_none | Bacillota | Tissierellia | Tissierellales | Peptoniphilaceae | Bacteria;Bacteria\_none;Bacillota;Tissierellia;Tissierellales;Peptoniphilaceae;Peptoniphilus |
| Keratinibaculum | 0 | 0 | 0 | 0 | 1 | 0 | 0 | 0 | 0 | 0 | 0 | 0 | 0 | 0 | Bacteria | Bacteria\_none | Bacillota | Tissierellia | Tissierellales | Tepidimicrobiaceae | Bacteria;Bacteria\_none;Bacillota;Tissierellia;Tissierellales;Tepidimicrobiaceae;Keratinibaculum |
| Ezakiella | 0 | 0 | 0 | 0 | 0 | 0 | 0 | 0 | 0 | 0 | 5 | 1 | 0 | 0 | Bacteria | Bacteria\_none | Bacillota | Tissierellia | Tissierellia\_Incertae\_sedis | Tissierellia\_Incertae\_sedis | Bacteria;Bacteria\_none;Bacillota;Tissierellia;Tissierellia\_Incertae\_sedis;Tissierellia\_Incertae\_sedis;Ezakiella |
| Bacteroides | 2 | 2 | 1 | 5 | 0 | 2 | 0 | 0 | 0 | 0 | 4 | 1 | 2 | 1 | Bacteria | Bacteria\_none | Bacteroidota | Bacteroidia | Bacteroidales | Bacteroidaceae | Bacteria;Bacteria\_none;Bacteroidota;Bacteroidia;Bacteroidales;Bacteroidaceae;Bacteroides |
| Phocaeicola | 0 | 0 | 0 | 1 | 1 | 1 | 0 | 0 | 0 | 0 | 1 | 0 | 1 | 0 | Bacteria | Bacteria\_none | Bacteroidota | Bacteroidia | Bacteroidales | Bacteroidaceae | Bacteria;Bacteria\_none;Bacteroidota;Bacteroidia;Bacteroidales;Bacteroidaceae;Phocaeicola |
| Barnesiella | 0 | 0 | 0 | 0 | 0 | 0 | 0 | 0 | 0 | 0 | 0 | 0 | 0 | 0 | Bacteria | Bacteria\_none | Bacteroidota | Bacteroidia | Bacteroidales | Barnesiellaceae | Bacteria;Bacteria\_none;Bacteroidota;Bacteroidia;Bacteroidales;Barnesiellaceae;Barnesiella |
| Proteiniphilum | 0 | 0 | 0 | 0 | 0 | 0 | 0 | 0 | 0 | 0 | 0 | 1 | 0 | 0 | Bacteria | Bacteria\_none | Bacteroidota | Bacteroidia | Bacteroidales | Dysgonomonadaceae | Bacteria;Bacteria\_none;Bacteroidota;Bacteroidia;Bacteroidales;Dysgonomonadaceae;Proteiniphilum |
| Duncaniella | 0 | 0 | 0 | 0 | 0 | 0 | 0 | 0 | 0 | 0 | 1 | 0 | 0 | 0 | Bacteria | Bacteria\_none | Bacteroidota | Bacteroidia | Bacteroidales | Muribaculaceae | Bacteria;Bacteria\_none;Bacteroidota;Bacteroidia;Bacteroidales;Muribaculaceae;Duncaniella |
| Sodaliphilus | 0 | 0 | 0 | 0 | 1 | 0 | 0 | 0 | 0 | 0 | 0 | 0 | 0 | 0 | Bacteria | Bacteria\_none | Bacteroidota | Bacteroidia | Bacteroidales | Muribaculaceae | Bacteria;Bacteria\_none;Bacteroidota;Bacteroidia;Bacteroidales;Muribaculaceae;Sodaliphilus |
| Butyricimonas | 0 | 0 | 0 | 0 | 0 | 0 | 0 | 0 | 0 | 0 | 0 | 0 | 0 | 0 | Bacteria | Bacteria\_none | Bacteroidota | Bacteroidia | Bacteroidales | Odoribacteraceae | Bacteria;Bacteria\_none;Bacteroidota;Bacteroidia;Bacteroidales;Odoribacteraceae;Butyricimonas |
| Odoribacter | 0 | 0 | 0 | 0 | 0 | 0 | 0 | 0 | 0 | 0 | 0 | 0 | 0 | 0 | Bacteria | Bacteria\_none | Bacteroidota | Bacteroidia | Bacteroidales | Odoribacteraceae | Bacteria;Bacteria\_none;Bacteroidota;Bacteroidia;Bacteroidales;Odoribacteraceae;Odoribacter |
| Porphyromonas | 5 | 122 | 7 | 28 | 34 | 24 | 4 | 23 | 0 | 27 | 41 | 13 | 17 | 10 | Bacteria | Bacteria\_none | Bacteroidota | Bacteroidia | Bacteroidales | Porphyromonadaceae | Bacteria;Bacteria\_none;Bacteroidota;Bacteroidia;Bacteroidales;Porphyromonadaceae;Porphyromonas |
| Hoylesella | 1 | 3 | 3 | 2 | 1 | 5 | 0 | 2 | 0 | 1 | 5 | 2 | 2 | 1 | Bacteria | Bacteria\_none | Bacteroidota | Bacteroidia | Bacteroidales | Prevotellaceae | Bacteria;Bacteria\_none;Bacteroidota;Bacteroidia;Bacteroidales;Prevotellaceae;Hoylesella |
| Prevotella | 299 | 918 | 243 | 464 | 43 | 180 | 70 | 9 | 140 | 65 | 79 | 136 | 138 | 88 | Bacteria | Bacteria\_none | Bacteroidota | Bacteroidia | Bacteroidales | Prevotellaceae | Bacteria;Bacteria\_none;Bacteroidota;Bacteroidia;Bacteroidales;Prevotellaceae;Prevotella |
| Segatella | 1 | 4 | 10 | 6 | 5 | 4 | 5 | 4 | 0 | 10 | 16 | 18 | 3 | 6 | Bacteria | Bacteria\_none | Bacteroidota | Bacteroidia | Bacteroidales | Prevotellaceae | Bacteria;Bacteria\_none;Bacteroidota;Bacteroidia;Bacteroidales;Prevotellaceae;Segatella |
| Xylanibacter | 0 | 0 | 0 | 0 | 0 | 0 | 0 | 0 | 0 | 0 | 0 | 0 | 0 | 0 | Bacteria | Bacteria\_none | Bacteroidota | Bacteroidia | Bacteroidales | Prevotellaceae | Bacteria;Bacteria\_none;Bacteroidota;Bacteroidia;Bacteroidales;Prevotellaceae;Xylanibacter |
| Alistipes | 0 | 2 | 0 | 0 | 0 | 0 | 0 | 0 | 0 | 0 | 0 | 0 | 0 | 0 | Bacteria | Bacteria\_none | Bacteroidota | Bacteroidia | Bacteroidales | Rikenellaceae | Bacteria;Bacteria\_none;Bacteroidota;Bacteroidia;Bacteroidales;Rikenellaceae;Alistipes |
| Salinivirga | 0 | 0 | 0 | 0 | 0 | 0 | 0 | 0 | 0 | 0 | 0 | 0 | 0 | 1 | Bacteria | Bacteria\_none | Bacteroidota | Bacteroidia | Bacteroidales | Salinivirgaceae | Bacteria;Bacteria\_none;Bacteroidota;Bacteroidia;Bacteroidales;Salinivirgaceae;Salinivirga |
| Parabacteroides | 0 | 0 | 0 | 0 | 0 | 0 | 0 | 0 | 0 | 0 | 0 | 0 | 0 | 0 | Bacteria | Bacteria\_none | Bacteroidota | Bacteroidia | Bacteroidales | Tannerellaceae | Bacteria;Bacteria\_none;Bacteroidota;Bacteroidia;Bacteroidales;Tannerellaceae;Parabacteroides |
| Tannerella | 2 | 37 | 29 | 8 | 11 | 82 | 5 | 14 | 0 | 23 | 25 | 10 | 16 | 11 | Bacteria | Bacteria\_none | Bacteroidota | Bacteroidia | Bacteroidales | Tannerellaceae | Bacteria;Bacteria\_none;Bacteroidota;Bacteroidia;Bacteroidales;Tannerellaceae;Tannerella |
| Marinifilum | 1 | 0 | 1 | 0 | 0 | 0 | 0 | 0 | 0 | 0 | 0 | 0 | 0 | 0 | Bacteria | Bacteria\_none | Bacteroidota | Bacteroidia | Marinilabiliales | Marinifilaceae | Bacteria;Bacteria\_none;Bacteroidota;Bacteroidia;Marinilabiliales;Marinifilaceae;Marinifilum |
| Alkalitalea | 0 | 0 | 0 | 0 | 0 | 0 | 0 | 1 | 0 | 0 | 0 | 0 | 0 | 0 | Bacteria | Bacteria\_none | Bacteroidota | Bacteroidia | Marinilabiliales | Marinilabiliaceae | Bacteria;Bacteria\_none;Bacteroidota;Bacteroidia;Marinilabiliales;Marinilabiliaceae;Alkalitalea |
| Carboxylicivirga | 0 | 0 | 1 | 1 | 0 | 0 | 0 | 0 | 0 | 0 | 0 | 0 | 0 | 0 | Bacteria | Bacteria\_none | Bacteroidota | Bacteroidia | Marinilabiliales | Marinilabiliaceae | Bacteria;Bacteria\_none;Bacteroidota;Bacteroidia;Marinilabiliales;Marinilabiliaceae;Carboxylicivirga |
| Draconibacterium | 0 | 0 | 0 | 1 | 0 | 0 | 0 | 0 | 0 | 0 | 1 | 0 | 0 | 0 | Bacteria | Bacteria\_none | Bacteroidota | Bacteroidia | Marinilabiliales | Prolixibacteraceae | Bacteria;Bacteria\_none;Bacteroidota;Bacteroidia;Marinilabiliales;Prolixibacteraceae;Draconibacterium |
| Mangrovibacterium | 0 | 0 | 0 | 0 | 0 | 1 | 0 | 0 | 0 | 0 | 0 | 0 | 0 | 0 | Bacteria | Bacteria\_none | Bacteroidota | Bacteroidia | Marinilabiliales | Prolixibacteraceae | Bacteria;Bacteria\_none;Bacteroidota;Bacteroidia;Marinilabiliales;Prolixibacteraceae;Mangrovibacterium |
| Maribellus | 0 | 0 | 0 | 1 | 0 | 0 | 0 | 0 | 0 | 0 | 0 | 0 | 0 | 0 | Bacteria | Bacteria\_none | Bacteroidota | Bacteroidia | Marinilabiliales | Prolixibacteraceae | Bacteria;Bacteria\_none;Bacteroidota;Bacteroidia;Marinilabiliales;Prolixibacteraceae;Maribellus |
| Chitinophaga | 0 | 1 | 0 | 1 | 2 | 0 | 0 | 0 | 0 | 0 | 0 | 0 | 0 | 0 | Bacteria | Bacteria\_none | Bacteroidota | Chitinophagia | Chitinophagales | Chitinophagaceae | Bacteria;Bacteria\_none;Bacteroidota;Chitinophagia;Chitinophagales;Chitinophagaceae;Chitinophaga |
| Ferruginibacter | 0 | 0 | 0 | 0 | 0 | 1 | 0 | 0 | 0 | 0 | 0 | 0 | 0 | 0 | Bacteria | Bacteria\_none | Bacteroidota | Chitinophagia | Chitinophagales | Chitinophagaceae | Bacteria;Bacteria\_none;Bacteroidota;Chitinophagia;Chitinophagales;Chitinophagaceae;Ferruginibacter |
| Flavihumibacter | 0 | 0 | 0 | 1 | 0 | 0 | 0 | 0 | 0 | 0 | 0 | 0 | 0 | 0 | Bacteria | Bacteria\_none | Bacteroidota | Chitinophagia | Chitinophagales | Chitinophagaceae | Bacteria;Bacteria\_none;Bacteroidota;Chitinophagia;Chitinophagales;Chitinophagaceae;Flavihumibacter |
| Pseudobacter | 0 | 0 | 0 | 0 | 0 | 0 | 0 | 0 | 0 | 0 | 0 | 0 | 0 | 0 | Bacteria | Bacteria\_none | Bacteroidota | Chitinophagia | Chitinophagales | Chitinophagaceae | Bacteria;Bacteria\_none;Bacteroidota;Chitinophagia;Chitinophagales;Chitinophagaceae;Pseudobacter |
| Candidatus Cardinium | 0 | 0 | 0 | 0 | 0 | 0 | 0 | 0 | 0 | 0 | 0 | 0 | 0 | 0 | Bacteria | Bacteria\_none | Bacteroidota | Cytophagia | Cytophagales | Amoebophilaceae | Bacteria;Bacteria\_none;Bacteroidota;Cytophagia;Cytophagales;Amoebophilaceae;Candidatus Cardinium |
| Algoriphagus | 0 | 0 | 0 | 0 | 0 | 0 | 0 | 0 | 0 | 0 | 1 | 0 | 0 | 0 | Bacteria | Bacteria\_none | Bacteroidota | Cytophagia | Cytophagales | Cyclobacteriaceae | Bacteria;Bacteria\_none;Bacteroidota;Cytophagia;Cytophagales;Cyclobacteriaceae;Algoriphagus |
| Cyclobacterium | 0 | 0 | 0 | 1 | 0 | 0 | 0 | 0 | 0 | 0 | 0 | 0 | 0 | 0 | Bacteria | Bacteria\_none | Bacteroidota | Cytophagia | Cytophagales | Cyclobacteriaceae | Bacteria;Bacteria\_none;Bacteroidota;Cytophagia;Cytophagales;Cyclobacteriaceae;Cyclobacterium |
| Spirosoma | 0 | 0 | 0 | 0 | 0 | 0 | 0 | 1 | 0 | 0 | 0 | 0 | 0 | 0 | Bacteria | Bacteria\_none | Bacteroidota | Cytophagia | Cytophagales | Cytophagaceae | Bacteria;Bacteria\_none;Bacteroidota;Cytophagia;Cytophagales;Cytophagaceae;Spirosoma |
| Flammeovirga | 0 | 0 | 0 | 0 | 1 | 0 | 0 | 0 | 0 | 0 | 0 | 0 | 0 | 0 | Bacteria | Bacteria\_none | Bacteroidota | Cytophagia | Cytophagales | Flammeovirgaceae | Bacteria;Bacteria\_none;Bacteroidota;Cytophagia;Cytophagales;Flammeovirgaceae;Flammeovirga |
| Hymenobacter | 0 | 0 | 0 | 0 | 0 | 0 | 1 | 0 | 0 | 0 | 0 | 0 | 0 | 0 | Bacteria | Bacteria\_none | Bacteroidota | Cytophagia | Cytophagales | Hymenobacteraceae | Bacteria;Bacteria\_none;Bacteroidota;Cytophagia;Cytophagales;Hymenobacteraceae;Hymenobacter |
| Rufibacter | 0 | 0 | 0 | 0 | 0 | 0 | 0 | 0 | 0 | 1 | 0 | 0 | 0 | 0 | Bacteria | Bacteria\_none | Bacteroidota | Cytophagia | Cytophagales | Hymenobacteraceae | Bacteria;Bacteria\_none;Bacteroidota;Cytophagia;Cytophagales;Hymenobacteraceae;Rufibacter |
| Arcticibacterium | 0 | 0 | 1 | 0 | 0 | 0 | 0 | 0 | 0 | 0 | 0 | 0 | 0 | 0 | Bacteria | Bacteria\_none | Bacteroidota | Cytophagia | Cytophagales | Leadbetterellaceae | Bacteria;Bacteria\_none;Bacteroidota;Cytophagia;Cytophagales;Leadbetterellaceae;Arcticibacterium |
| Emticicia | 1 | 0 | 0 | 0 | 0 | 0 | 0 | 0 | 0 | 0 | 0 | 0 | 0 | 0 | Bacteria | Bacteria\_none | Bacteroidota | Cytophagia | Cytophagales | Leadbetterellaceae | Bacteria;Bacteria\_none;Bacteroidota;Cytophagia;Cytophagales;Leadbetterellaceae;Emticicia |
| Marivirga | 0 | 0 | 0 | 0 | 0 | 1 | 0 | 0 | 0 | 0 | 0 | 0 | 0 | 0 | Bacteria | Bacteria\_none | Bacteroidota | Cytophagia | Cytophagales | Marivirgaceae | Bacteria;Bacteria\_none;Bacteroidota;Cytophagia;Cytophagales;Marivirgaceae;Marivirga |
| Fulvitalea | 0 | 0 | 0 | 1 | 1 | 0 | 0 | 0 | 0 | 0 | 0 | 0 | 0 | 0 | Bacteria | Bacteria\_none | Bacteroidota | Cytophagia | Cytophagales | Persicobacteraceae | Bacteria;Bacteria\_none;Bacteroidota;Cytophagia;Cytophagales;Persicobacteraceae;Fulvitalea |
| Dyadobacter | 0 | 0 | 0 | 0 | 0 | 1 | 0 | 0 | 0 | 0 | 0 | 0 | 0 | 0 | Bacteria | Bacteria\_none | Bacteroidota | Cytophagia | Cytophagales | Spirosomataceae | Bacteria;Bacteria\_none;Bacteroidota;Cytophagia;Cytophagales;Spirosomataceae;Dyadobacter |
| Fibrella | 0 | 0 | 0 | 0 | 0 | 0 | 0 | 0 | 0 | 0 | 0 | 0 | 0 | 0 | Bacteria | Bacteria\_none | Bacteroidota | Cytophagia | Cytophagales | Spirosomataceae | Bacteria;Bacteria\_none;Bacteroidota;Cytophagia;Cytophagales;Spirosomataceae;Fibrella |
| Blattabacterium | 0 | 0 | 0 | 0 | 0 | 0 | 0 | 0 | 0 | 0 | 1 | 0 | 0 | 0 | Bacteria | Bacteria\_none | Bacteroidota | Flavobacteriia | Flavobacteriales | Blattabacteriaceae | Bacteria;Bacteria\_none;Bacteroidota;Flavobacteriia;Flavobacteriales;Blattabacteriaceae;Blattabacterium |
| Paracrocinitomix | 0 | 0 | 0 | 0 | 0 | 0 | 0 | 0 | 0 | 1 | 0 | 0 | 0 | 0 | Bacteria | Bacteria\_none | Bacteroidota | Flavobacteriia | Flavobacteriales | Crocinitomicaceae | Bacteria;Bacteria\_none;Bacteroidota;Flavobacteriia;Flavobacteriales;Crocinitomicaceae;Paracrocinitomix |
| Aequorivita | 0 | 0 | 0 | 1 | 0 | 0 | 0 | 0 | 0 | 0 | 0 | 0 | 0 | 0 | Bacteria | Bacteria\_none | Bacteroidota | Flavobacteriia | Flavobacteriales | Flavobacteriaceae | Bacteria;Bacteria\_none;Bacteroidota;Flavobacteriia;Flavobacteriales;Flavobacteriaceae;Aequorivita |
| Algibacter | 0 | 0 | 0 | 0 | 0 | 1 | 0 | 0 | 0 | 0 | 0 | 0 | 0 | 0 | Bacteria | Bacteria\_none | Bacteroidota | Flavobacteriia | Flavobacteriales | Flavobacteriaceae | Bacteria;Bacteria\_none;Bacteroidota;Flavobacteriia;Flavobacteriales;Flavobacteriaceae;Algibacter |
| Aquimarina | 0 | 0 | 0 | 0 | 1 | 0 | 0 | 1 | 0 | 0 | 0 | 0 | 0 | 0 | Bacteria | Bacteria\_none | Bacteroidota | Flavobacteriia | Flavobacteriales | Flavobacteriaceae | Bacteria;Bacteria\_none;Bacteroidota;Flavobacteriia;Flavobacteriales;Flavobacteriaceae;Aquimarina |
| Capnocytophaga | 270 | 139 | 117 | 118 | 83 | 22 | 25 | 88 | 0 | 168 | 95 | 19 | 71 | 51 | Bacteria | Bacteria\_none | Bacteroidota | Flavobacteriia | Flavobacteriales | Flavobacteriaceae | Bacteria;Bacteria\_none;Bacteroidota;Flavobacteriia;Flavobacteriales;Flavobacteriaceae;Capnocytophaga |
| Christiangramia | 0 | 0 | 0 | 0 | 0 | 0 | 0 | 0 | 0 | 0 | 0 | 0 | 0 | 0 | Bacteria | Bacteria\_none | Bacteroidota | Flavobacteriia | Flavobacteriales | Flavobacteriaceae | Bacteria;Bacteria\_none;Bacteroidota;Flavobacteriia;Flavobacteriales;Flavobacteriaceae;Christiangramia |
| Costertonia | 0 | 0 | 0 | 0 | 0 | 0 | 0 | 0 | 0 | 0 | 1 | 0 | 0 | 0 | Bacteria | Bacteria\_none | Bacteroidota | Flavobacteriia | Flavobacteriales | Flavobacteriaceae | Bacteria;Bacteria\_none;Bacteroidota;Flavobacteriia;Flavobacteriales;Flavobacteriaceae;Costertonia |
| Dokdonia | 0 | 1 | 0 | 0 | 0 | 0 | 0 | 0 | 0 | 0 | 0 | 0 | 0 | 0 | Bacteria | Bacteria\_none | Bacteroidota | Flavobacteriia | Flavobacteriales | Flavobacteriaceae | Bacteria;Bacteria\_none;Bacteroidota;Flavobacteriia;Flavobacteriales;Flavobacteriaceae;Dokdonia |
| Euzebyella | 0 | 0 | 0 | 0 | 0 | 0 | 0 | 0 | 0 | 0 | 0 | 0 | 0 | 0 | Bacteria | Bacteria\_none | Bacteroidota | Flavobacteriia | Flavobacteriales | Flavobacteriaceae | Bacteria;Bacteria\_none;Bacteroidota;Flavobacteriia;Flavobacteriales;Flavobacteriaceae;Euzebyella |
| Flagellimonas | 0 | 0 | 0 | 0 | 0 | 0 | 0 | 0 | 0 | 0 | 1 | 0 | 0 | 0 | Bacteria | Bacteria\_none | Bacteroidota | Flavobacteriia | Flavobacteriales | Flavobacteriaceae | Bacteria;Bacteria\_none;Bacteroidota;Flavobacteriia;Flavobacteriales;Flavobacteriaceae;Flagellimonas |
| Flavobacterium | 2 | 2 | 0 | 3 | 2 | 2 | 0 | 0 | 0 | 0 | 1 | 1 | 2 | 0 | Bacteria | Bacteria\_none | Bacteroidota | Flavobacteriia | Flavobacteriales | Flavobacteriaceae | Bacteria;Bacteria\_none;Bacteroidota;Flavobacteriia;Flavobacteriales;Flavobacteriaceae;Flavobacterium |
| Formosa | 0 | 0 | 0 | 0 | 0 | 1 | 0 | 0 | 0 | 0 | 0 | 0 | 0 | 1 | Bacteria | Bacteria\_none | Bacteroidota | Flavobacteriia | Flavobacteriales | Flavobacteriaceae | Bacteria;Bacteria\_none;Bacteroidota;Flavobacteriia;Flavobacteriales;Flavobacteriaceae;Formosa |
| Kordia | 0 | 0 | 0 | 0 | 0 | 0 | 0 | 0 | 0 | 0 | 0 | 0 | 0 | 0 | Bacteria | Bacteria\_none | Bacteroidota | Flavobacteriia | Flavobacteriales | Flavobacteriaceae | Bacteria;Bacteria\_none;Bacteroidota;Flavobacteriia;Flavobacteriales;Flavobacteriaceae;Kordia |
| Lacinutrix | 0 | 0 | 0 | 0 | 0 | 0 | 0 | 0 | 0 | 0 | 0 | 0 | 0 | 0 | Bacteria | Bacteria\_none | Bacteroidota | Flavobacteriia | Flavobacteriales | Flavobacteriaceae | Bacteria;Bacteria\_none;Bacteroidota;Flavobacteriia;Flavobacteriales;Flavobacteriaceae;Lacinutrix |
| Leeuwenhoekiella | 0 | 0 | 0 | 0 | 1 | 0 | 0 | 0 | 0 | 0 | 0 | 0 | 0 | 0 | Bacteria | Bacteria\_none | Bacteroidota | Flavobacteriia | Flavobacteriales | Flavobacteriaceae | Bacteria;Bacteria\_none;Bacteroidota;Flavobacteriia;Flavobacteriales;Flavobacteriaceae;Leeuwenhoekiella |
| Lutibacter | 0 | 0 | 0 | 0 | 0 | 0 | 0 | 0 | 0 | 0 | 0 | 0 | 0 | 0 | Bacteria | Bacteria\_none | Bacteroidota | Flavobacteriia | Flavobacteriales | Flavobacteriaceae | Bacteria;Bacteria\_none;Bacteroidota;Flavobacteriia;Flavobacteriales;Flavobacteriaceae;Lutibacter |
| Mangrovimonas | 0 | 0 | 0 | 0 | 0 | 0 | 0 | 0 | 0 | 1 | 0 | 0 | 0 | 0 | Bacteria | Bacteria\_none | Bacteroidota | Flavobacteriia | Flavobacteriales | Flavobacteriaceae | Bacteria;Bacteria\_none;Bacteroidota;Flavobacteriia;Flavobacteriales;Flavobacteriaceae;Mangrovimonas |
| Maribacter | 0 | 0 | 0 | 0 | 0 | 0 | 0 | 0 | 0 | 0 | 0 | 0 | 0 | 0 | Bacteria | Bacteria\_none | Bacteroidota | Flavobacteriia | Flavobacteriales | Flavobacteriaceae | Bacteria;Bacteria\_none;Bacteroidota;Flavobacteriia;Flavobacteriales;Flavobacteriaceae;Maribacter |
| Myroides | 0 | 0 | 0 | 0 | 1 | 0 | 0 | 0 | 0 | 0 | 0 | 0 | 0 | 0 | Bacteria | Bacteria\_none | Bacteroidota | Flavobacteriia | Flavobacteriales | Flavobacteriaceae | Bacteria;Bacteria\_none;Bacteroidota;Flavobacteriia;Flavobacteriales;Flavobacteriaceae;Myroides |
| Nonlabens | 0 | 0 | 0 | 0 | 0 | 0 | 0 | 0 | 0 | 0 | 0 | 0 | 0 | 1 | Bacteria | Bacteria\_none | Bacteroidota | Flavobacteriia | Flavobacteriales | Flavobacteriaceae | Bacteria;Bacteria\_none;Bacteroidota;Flavobacteriia;Flavobacteriales;Flavobacteriaceae;Nonlabens |
| Polaribacter | 0 | 0 | 1 | 1 | 1 | 1 | 1 | 0 | 0 | 0 | 1 | 0 | 0 | 0 | Bacteria | Bacteria\_none | Bacteroidota | Flavobacteriia | Flavobacteriales | Flavobacteriaceae | Bacteria;Bacteria\_none;Bacteroidota;Flavobacteriia;Flavobacteriales;Flavobacteriaceae;Polaribacter |
| Psychroflexus | 0 | 0 | 0 | 0 | 0 | 0 | 0 | 0 | 0 | 0 | 1 | 0 | 0 | 0 | Bacteria | Bacteria\_none | Bacteroidota | Flavobacteriia | Flavobacteriales | Flavobacteriaceae | Bacteria;Bacteria\_none;Bacteroidota;Flavobacteriia;Flavobacteriales;Flavobacteriaceae;Psychroflexus |
| Rasiella | 0 | 0 | 0 | 0 | 0 | 0 | 0 | 0 | 0 | 0 | 0 | 0 | 0 | 0 | Bacteria | Bacteria\_none | Bacteroidota | Flavobacteriia | Flavobacteriales | Flavobacteriaceae | Bacteria;Bacteria\_none;Bacteroidota;Flavobacteriia;Flavobacteriales;Flavobacteriaceae;Rasiella |
| Salegentibacter | 0 | 1 | 0 | 0 | 0 | 0 | 0 | 0 | 0 | 0 | 0 | 0 | 0 | 0 | Bacteria | Bacteria\_none | Bacteroidota | Flavobacteriia | Flavobacteriales | Flavobacteriaceae | Bacteria;Bacteria\_none;Bacteroidota;Flavobacteriia;Flavobacteriales;Flavobacteriaceae;Salegentibacter |
| Tenacibaculum | 0 | 0 | 1 | 0 | 2 | 0 | 0 | 0 | 0 | 0 | 0 | 0 | 0 | 0 | Bacteria | Bacteria\_none | Bacteroidota | Flavobacteriia | Flavobacteriales | Flavobacteriaceae | Bacteria;Bacteria\_none;Bacteroidota;Flavobacteriia;Flavobacteriales;Flavobacteriaceae;Tenacibaculum |
| Zobellia | 0 | 0 | 0 | 0 | 1 | 0 | 0 | 0 | 0 | 0 | 0 | 0 | 0 | 0 | Bacteria | Bacteria\_none | Bacteroidota | Flavobacteriia | Flavobacteriales | Flavobacteriaceae | Bacteria;Bacteria\_none;Bacteroidota;Flavobacteriia;Flavobacteriales;Flavobacteriaceae;Zobellia |
| Croceimicrobium | 0 | 1 | 0 | 0 | 1 | 0 | 0 | 0 | 0 | 0 | 0 | 0 | 0 | 0 | Bacteria | Bacteria\_none | Bacteroidota | Flavobacteriia | Flavobacteriales | Owenweeksiaceae | Bacteria;Bacteria\_none;Bacteroidota;Flavobacteriia;Flavobacteriales;Owenweeksiaceae;Croceimicrobium |
| Sanyastnella | 0 | 0 | 0 | 0 | 0 | 0 | 0 | 0 | 0 | 0 | 0 | 0 | 0 | 0 | Bacteria | Bacteria\_none | Bacteroidota | Flavobacteriia | Flavobacteriales | Sanyastnellaceae | Bacteria;Bacteria\_none;Bacteroidota;Flavobacteriia;Flavobacteriales;Sanyastnellaceae;Sanyastnella |
| Apibacter | 0 | 0 | 0 | 0 | 0 | 0 | 0 | 0 | 0 | 0 | 0 | 0 | 0 | 0 | Bacteria | Bacteria\_none | Bacteroidota | Flavobacteriia | Flavobacteriales | Weeksellaceae | Bacteria;Bacteria\_none;Bacteroidota;Flavobacteriia;Flavobacteriales;Weeksellaceae;Apibacter |
| Bergeyella | 0 | 0 | 1 | 0 | 1 | 0 | 0 | 0 | 0 | 0 | 0 | 0 | 0 | 0 | Bacteria | Bacteria\_none | Bacteroidota | Flavobacteriia | Flavobacteriales | Weeksellaceae | Bacteria;Bacteria\_none;Bacteroidota;Flavobacteriia;Flavobacteriales;Weeksellaceae;Bergeyella |
| Chryseobacterium | 2 | 0 | 2 | 1 | 3 | 2 | 0 | 0 | 0 | 1 | 0 | 1 | 1 | 1 | Bacteria | Bacteria\_none | Bacteroidota | Flavobacteriia | Flavobacteriales | Weeksellaceae | Bacteria;Bacteria\_none;Bacteroidota;Flavobacteriia;Flavobacteriales;Weeksellaceae;Chryseobacterium |
| Cloacibacterium | 0 | 0 | 0 | 0 | 0 | 0 | 0 | 0 | 0 | 0 | 0 | 0 | 0 | 0 | Bacteria | Bacteria\_none | Bacteroidota | Flavobacteriia | Flavobacteriales | Weeksellaceae | Bacteria;Bacteria\_none;Bacteroidota;Flavobacteriia;Flavobacteriales;Weeksellaceae;Cloacibacterium |
| Elizabethkingia | 0 | 1 | 1 | 0 | 2 | 1 | 0 | 0 | 0 | 0 | 0 | 0 | 0 | 0 | Bacteria | Bacteria\_none | Bacteroidota | Flavobacteriia | Flavobacteriales | Weeksellaceae | Bacteria;Bacteria\_none;Bacteroidota;Flavobacteriia;Flavobacteriales;Weeksellaceae;Elizabethkingia |
| Empedobacter | 1 | 0 | 0 | 0 | 0 | 0 | 0 | 0 | 0 | 0 | 0 | 0 | 0 | 0 | Bacteria | Bacteria\_none | Bacteroidota | Flavobacteriia | Flavobacteriales | Weeksellaceae | Bacteria;Bacteria\_none;Bacteroidota;Flavobacteriia;Flavobacteriales;Weeksellaceae;Empedobacter |
| Epilithonimonas | 0 | 0 | 0 | 0 | 1 | 0 | 0 | 0 | 0 | 0 | 0 | 0 | 0 | 0 | Bacteria | Bacteria\_none | Bacteroidota | Flavobacteriia | Flavobacteriales | Weeksellaceae | Bacteria;Bacteria\_none;Bacteroidota;Flavobacteriia;Flavobacteriales;Weeksellaceae;Epilithonimonas |
| Ornithobacterium | 1 | 0 | 0 | 0 | 0 | 0 | 0 | 0 | 0 | 0 | 0 | 0 | 0 | 0 | Bacteria | Bacteria\_none | Bacteroidota | Flavobacteriia | Flavobacteriales | Weeksellaceae | Bacteria;Bacteria\_none;Bacteroidota;Flavobacteriia;Flavobacteriales;Weeksellaceae;Ornithobacterium |
| Riemerella | 0 | 0 | 0 | 1 | 0 | 1 | 0 | 0 | 0 | 0 | 0 | 0 | 0 | 0 | Bacteria | Bacteria\_none | Bacteroidota | Flavobacteriia | Flavobacteriales | Weeksellaceae | Bacteria;Bacteria\_none;Bacteroidota;Flavobacteriia;Flavobacteriales;Weeksellaceae;Riemerella |
| Haliscomenobacter | 0 | 0 | 0 | 0 | 0 | 0 | 0 | 0 | 0 | 0 | 0 | 0 | 0 | 1 | Bacteria | Bacteria\_none | Bacteroidota | Saprospiria | Saprospirales | Haliscomenobacteraceae | Bacteria;Bacteria\_none;Bacteroidota;Saprospiria;Saprospirales;Haliscomenobacteraceae;Haliscomenobacter |
| Aurantibacillus | 0 | 0 | 0 | 0 | 0 | 0 | 0 | 0 | 0 | 0 | 0 | 0 | 0 | 0 | Bacteria | Bacteria\_none | Bacteroidota | Sphingobacteriia | Sphingobacteriales | Aurantibacillaceae | Bacteria;Bacteria\_none;Bacteroidota;Sphingobacteriia;Sphingobacteriales;Aurantibacillaceae;Aurantibacillus |
| Mucilaginibacter | 0 | 2 | 0 | 0 | 0 | 0 | 0 | 0 | 0 | 0 | 0 | 1 | 0 | 0 | Bacteria | Bacteria\_none | Bacteroidota | Sphingobacteriia | Sphingobacteriales | Sphingobacteriaceae | Bacteria;Bacteria\_none;Bacteroidota;Sphingobacteriia;Sphingobacteriales;Sphingobacteriaceae;Mucilaginibacter |
| Olivibacter | 0 | 0 | 0 | 0 | 0 | 0 | 0 | 0 | 0 | 0 | 0 | 0 | 0 | 0 | Bacteria | Bacteria\_none | Bacteroidota | Sphingobacteriia | Sphingobacteriales | Sphingobacteriaceae | Bacteria;Bacteria\_none;Bacteroidota;Sphingobacteriia;Sphingobacteriales;Sphingobacteriaceae;Olivibacter |
| Pedobacter | 0 | 0 | 0 | 0 | 0 | 0 | 0 | 1 | 0 | 0 | 1 | 0 | 0 | 0 | Bacteria | Bacteria\_none | Bacteroidota | Sphingobacteriia | Sphingobacteriales | Sphingobacteriaceae | Bacteria;Bacteria\_none;Bacteroidota;Sphingobacteriia;Sphingobacteriales;Sphingobacteriaceae;Pedobacter |
| Solitalea | 0 | 0 | 0 | 0 | 0 | 0 | 0 | 0 | 0 | 0 | 0 | 0 | 0 | 0 | Bacteria | Bacteria\_none | Bacteroidota | Sphingobacteriia | Sphingobacteriales | Sphingobacteriaceae | Bacteria;Bacteria\_none;Bacteroidota;Sphingobacteriia;Sphingobacteriales;Sphingobacteriaceae;Solitalea |
| Sphingobacterium | 0 | 0 | 0 | 0 | 1 | 0 | 0 | 0 | 0 | 1 | 0 | 0 | 0 | 0 | Bacteria | Bacteria\_none | Bacteroidota | Sphingobacteriia | Sphingobacteriales | Sphingobacteriaceae | Bacteria;Bacteria\_none;Bacteroidota;Sphingobacteriia;Sphingobacteriales;Sphingobacteriaceae;Sphingobacterium |
| Cyclonatronum | 0 | 0 | 0 | 0 | 0 | 0 | 0 | 0 | 0 | 0 | 0 | 0 | 0 | 0 | Bacteria | Bacteria\_none | Balneolota | Balneolia | Balneolales | Cyclonatronaceae | Bacteria;Bacteria\_none;Balneolota;Balneolia;Balneolales;Cyclonatronaceae;Cyclonatronum |
| Bdellovibrio | 0 | 0 | 0 | 0 | 0 | 0 | 0 | 0 | 0 | 0 | 0 | 0 | 0 | 0 | Bacteria | Bacteria\_none | Bdellovibrionota | Bdellovibrionia | Bdellovibrionales | Pseudobdellovibrionaceae | Bacteria;Bacteria\_none;Bdellovibrionota;Bdellovibrionia;Bdellovibrionales;Pseudobdellovibrionaceae;Bdellovibrio |
| Caldithrix | 0 | 0 | 0 | 0 | 0 | 0 | 0 | 0 | 0 | 0 | 0 | 0 | 0 | 0 | Bacteria | Bacteria\_none | Calditrichota | Calditrichia | Calditrichales | Calditrichaceae | Bacteria;Bacteria\_none;Calditrichota;Calditrichia;Calditrichales;Calditrichaceae;Caldithrix |
| Aliarcobacter | 0 | 0 | 0 | 0 | 0 | 0 | 0 | 0 | 0 | 0 | 0 | 0 | 0 | 0 | Bacteria | Bacteria\_none | Campylobacterota | Epsilonproteobacteria | Campylobacterales | Arcobacteraceae | Bacteria;Bacteria\_none;Campylobacterota;Epsilonproteobacteria;Campylobacterales;Arcobacteraceae;Aliarcobacter |
| Arcobacter | 0 | 0 | 1 | 0 | 0 | 0 | 0 | 0 | 0 | 0 | 0 | 0 | 0 | 0 | Bacteria | Bacteria\_none | Campylobacterota | Epsilonproteobacteria | Campylobacterales | Arcobacteraceae | Bacteria;Bacteria\_none;Campylobacterota;Epsilonproteobacteria;Campylobacterales;Arcobacteraceae;Arcobacter |
| Halarcobacter | 0 | 0 | 0 | 0 | 0 | 0 | 0 | 0 | 0 | 1 | 0 | 0 | 0 | 0 | Bacteria | Bacteria\_none | Campylobacterota | Epsilonproteobacteria | Campylobacterales | Arcobacteraceae | Bacteria;Bacteria\_none;Campylobacterota;Epsilonproteobacteria;Campylobacterales;Arcobacteraceae;Halarcobacter |
| Malaciobacter | 0 | 0 | 0 | 0 | 0 | 0 | 0 | 0 | 0 | 0 | 0 | 0 | 0 | 0 | Bacteria | Bacteria\_none | Campylobacterota | Epsilonproteobacteria | Campylobacterales | Arcobacteraceae | Bacteria;Bacteria\_none;Campylobacterota;Epsilonproteobacteria;Campylobacterales;Arcobacteraceae;Malaciobacter |
| Campylobacter | 0 | 46 | 14 | 3 | 2 | 38 | 0 | 4 | 3 | 6 | 1 | 4 | 5 | 4 | Bacteria | Bacteria\_none | Campylobacterota | Epsilonproteobacteria | Campylobacterales | Campylobacteraceae | Bacteria;Bacteria\_none;Campylobacterota;Epsilonproteobacteria;Campylobacterales;Campylobacteraceae;Campylobacter |
| Helicobacter | 0 | 0 | 0 | 0 | 1 | 1 | 0 | 0 | 0 | 0 | 0 | 0 | 0 | 0 | Bacteria | Bacteria\_none | Campylobacterota | Epsilonproteobacteria | Campylobacterales | Helicobacteraceae | Bacteria;Bacteria\_none;Campylobacterota;Epsilonproteobacteria;Campylobacterales;Helicobacteraceae;Helicobacter |
| Sulfurospirillum | 0 | 0 | 0 | 0 | 0 | 0 | 0 | 0 | 0 | 1 | 0 | 0 | 0 | 0 | Bacteria | Bacteria\_none | Campylobacterota | Epsilonproteobacteria | Campylobacterales | Sulfurospirillaceae | Bacteria;Bacteria\_none;Campylobacterota;Epsilonproteobacteria;Campylobacterales;Sulfurospirillaceae;Sulfurospirillum |
| Sulfurovum | 1 | 0 | 0 | 0 | 0 | 0 | 0 | 0 | 0 | 0 | 0 | 0 | 0 | 0 | Bacteria | Bacteria\_none | Campylobacterota | Epsilonproteobacteria | Campylobacterales | Sulfurovaceae | Bacteria;Bacteria\_none;Campylobacterota;Epsilonproteobacteria;Campylobacterales;Sulfurovaceae;Sulfurovum |
| Candidatus Minimicrobia | 0 | 0 | 0 | 0 | 0 | 17 | 0 | 0 | 0 | 0 | 1 | 0 | 1 | 0 | Bacteria | Bacteria\_none | Candidatus Saccharibacteria | Candidatus Saccharibacteria\_Incertae\_sedis | Candidatus Saccharibacteria\_Incertae\_sedis | Candidatus Saccharibacteria\_Incertae\_sedis | Bacteria;Bacteria\_none;Candidatus Saccharibacteria;Candidatus Saccharibacteria\_Incertae\_sedis;Candidatus Saccharibacteria\_Incertae\_sedis;Candidatus Saccharibacteria\_Incertae\_sedis;Candidatus Minimicrobia |
| Candidatus Mycosynbacter | 0 | 0 | 0 | 0 | 0 | 0 | 0 | 0 | 0 | 0 | 0 | 0 | 0 | 0 | Bacteria | Bacteria\_none | Candidatus Saccharibacteria | Candidatus Saccharibacteria\_Incertae\_sedis | Candidatus Saccharibacteria\_Incertae\_sedis | Candidatus Saccharibacteria\_Incertae\_sedis | Bacteria;Bacteria\_none;Candidatus Saccharibacteria;Candidatus Saccharibacteria\_Incertae\_sedis;Candidatus Saccharibacteria\_Incertae\_sedis;Candidatus Saccharibacteria\_Incertae\_sedis;Candidatus Mycosynbacter |
| Candidatus Nanosynbacter | 0 | 7 | 5 | 0 | 6 | 118 | 0 | 0 | 1 | 2 | 39 | 3 | 6 | 2 | Bacteria | Bacteria\_none | Candidatus Saccharibacteria | Candidatus Saccharimonadia | Candidatus Nanosynbacterales | Candidatus Nanosynbacteraceae | Bacteria;Bacteria\_none;Candidatus Saccharibacteria;Candidatus Saccharimonadia;Candidatus Nanosynbacterales;Candidatus Nanosynbacteraceae;Candidatus Nanosynbacter |
| Candidatus Saccharimonas | 0 | 0 | 0 | 0 | 1 | 0 | 0 | 1 | 0 | 0 | 0 | 0 | 0 | 0 | Bacteria | Bacteria\_none | Candidatus Saccharibacteria | Candidatus Saccharimonadia | Candidatus Saccharimonadales | Candidatus Saccharimonadaceae | Bacteria;Bacteria\_none;Candidatus Saccharibacteria;Candidatus Saccharimonadia;Candidatus Saccharimonadales;Candidatus Saccharimonadaceae;Candidatus Saccharimonas |
| Candidatus Protochlamydia | 0 | 0 | 0 | 0 | 0 | 0 | 0 | 0 | 0 | 0 | 0 | 0 | 0 | 1 | Bacteria | Bacteria\_none | Chlamydiota | Chlamydiia | Parachlamydiales | Parachlamydiaceae | Bacteria;Bacteria\_none;Chlamydiota;Chlamydiia;Parachlamydiales;Parachlamydiaceae;Candidatus Protochlamydia |
| Chloroherpeton | 0 | 0 | 0 | 0 | 0 | 0 | 0 | 0 | 0 | 0 | 0 | 0 | 0 | 0 | Bacteria | Bacteria\_none | Chlorobiota | Chlorobiia | Chlorobiales | Chloroherpetonaceae | Bacteria;Bacteria\_none;Chlorobiota;Chlorobiia;Chlorobiales;Chloroherpetonaceae;Chloroherpeton |
| Candidatus Chlorohelix | 0 | 0 | 0 | 0 | 0 | 0 | 0 | 0 | 0 | 0 | 0 | 0 | 0 | 0 | Bacteria | Bacteria\_none | Chloroflexota | Chloroflexia | Candidatus Chloroheliales | Candidatus Chloroheliaceae | Bacteria;Bacteria\_none;Chloroflexota;Chloroflexia;Candidatus Chloroheliales;Candidatus Chloroheliaceae;Candidatus Chlorohelix |
| Candidatus Lucifugimonas | 0 | 0 | 0 | 0 | 0 | 0 | 0 | 0 | 0 | 0 | 0 | 0 | 0 | 0 | Bacteria | Bacteria\_none | Chloroflexota | Dehalococcoidia | Candidatus Lucifugimonadales | Candidatus Lucifugimonadaceae | Bacteria;Bacteria\_none;Chloroflexota;Dehalococcoidia;Candidatus Lucifugimonadales;Candidatus Lucifugimonadaceae;Candidatus Lucifugimonas |
| Cyanobacteriota\_Incertae\_sedis | 1 | 0 | 0 | 0 | 0 | 0 | 0 | 0 | 0 | 0 | 0 | 0 | 0 | 0 | Bacteria | Bacteria\_none | Cyanobacteriota | Cyanobacteriota\_Incertae\_sedis | Cyanobacteriota\_Incertae\_sedis | Cyanobacteriota\_Incertae\_sedis | Bacteria;Bacteria\_none;Cyanobacteriota;Cyanobacteriota\_Incertae\_sedis;Cyanobacteriota\_Incertae\_sedis;Cyanobacteriota\_Incertae\_sedis;Cyanobacteriota\_Incertae\_sedis |
| Gloeocapsa | 0 | 0 | 1 | 0 | 0 | 0 | 0 | 0 | 0 | 0 | 0 | 0 | 0 | 0 | Bacteria | Bacteria\_none | Cyanobacteriota | Cyanophyceae | Chroococcales | Chroococcaceae | Bacteria;Bacteria\_none;Cyanobacteriota;Cyanophyceae;Chroococcales;Chroococcaceae;Gloeocapsa |
| Geminocystis | 0 | 0 | 0 | 0 | 0 | 0 | 0 | 0 | 0 | 0 | 1 | 0 | 0 | 0 | Bacteria | Bacteria\_none | Cyanobacteriota | Cyanophyceae | Chroococcales | Geminocystaceae | Bacteria;Bacteria\_none;Cyanobacteriota;Cyanophyceae;Chroococcales;Geminocystaceae;Geminocystis |
| Gloeobacter | 0 | 0 | 0 | 0 | 0 | 0 | 1 | 0 | 0 | 0 | 0 | 0 | 0 | 0 | Bacteria | Bacteria\_none | Cyanobacteriota | Cyanophyceae | Gloeobacterales | Gloeobacteraceae | Bacteria;Bacteria\_none;Cyanobacteriota;Cyanophyceae;Gloeobacterales;Gloeobacteraceae;Gloeobacter |
| Cylindrospermopsis | 0 | 0 | 0 | 1 | 0 | 0 | 0 | 0 | 0 | 0 | 0 | 0 | 0 | 0 | Bacteria | Bacteria\_none | Cyanobacteriota | Cyanophyceae | Nostocales | Aphanizomenonaceae | Bacteria;Bacteria\_none;Cyanobacteriota;Cyanophyceae;Nostocales;Aphanizomenonaceae;Cylindrospermopsis |
| Nostoc | 0 | 1 | 0 | 0 | 0 | 0 | 0 | 0 | 0 | 2 | 1 | 0 | 0 | 0 | Bacteria | Bacteria\_none | Cyanobacteriota | Cyanophyceae | Nostocales | Nostocaceae | Bacteria;Bacteria\_none;Cyanobacteriota;Cyanophyceae;Nostocales;Nostocaceae;Nostoc |
| Prochlorococcus | 0 | 0 | 0 | 0 | 0 | 0 | 0 | 0 | 0 | 0 | 0 | 0 | 0 | 0 | Bacteria | Bacteria\_none | Cyanobacteriota | Cyanophyceae | Synechococcales | Prochlorococcaceae | Bacteria;Bacteria\_none;Cyanobacteriota;Cyanophyceae;Synechococcales;Prochlorococcaceae;Prochlorococcus |
| Synechococcus | 0 | 0 | 0 | 0 | 0 | 1 | 0 | 0 | 0 | 0 | 0 | 0 | 0 | 0 | Bacteria | Bacteria\_none | Cyanobacteriota | Cyanophyceae | Synechococcales | Synechococcaceae | Bacteria;Bacteria\_none;Cyanobacteriota;Cyanophyceae;Synechococcales;Synechococcaceae;Synechococcus |
| Calditerrivibrio | 0 | 1 | 0 | 0 | 0 | 0 | 0 | 0 | 0 | 0 | 0 | 0 | 0 | 0 | Bacteria | Bacteria\_none | Deferribacterota | Deferribacteres | Deferribacterales | Calditerrivibrionaceae | Bacteria;Bacteria\_none;Deferribacterota;Deferribacteres;Deferribacterales;Calditerrivibrionaceae;Calditerrivibrio |
| Mucispirillum | 0 | 0 | 0 | 0 | 0 | 0 | 0 | 0 | 0 | 0 | 0 | 0 | 0 | 0 | Bacteria | Bacteria\_none | Deferribacterota | Deferribacteres | Deferribacterales | Mucispirillaceae | Bacteria;Bacteria\_none;Deferribacterota;Deferribacteres;Deferribacterales;Mucispirillaceae;Mucispirillum |
| Cetobacterium | 0 | 0 | 0 | 0 | 0 | 0 | 0 | 0 | 0 | 0 | 0 | 0 | 0 | 0 | Bacteria | Bacteria\_none | Fusobacteriota | Fusobacteriia | Fusobacteriales | Fusobacteriaceae | Bacteria;Bacteria\_none;Fusobacteriota;Fusobacteriia;Fusobacteriales;Fusobacteriaceae;Cetobacterium |
| Fusobacterium | 0 | 162 | 49 | 371 | 32 | 20 | 5 | 46 | 0 | 24 | 94 | 53 | 66 | 67 | Bacteria | Bacteria\_none | Fusobacteriota | Fusobacteriia | Fusobacteriales | Fusobacteriaceae | Bacteria;Bacteria\_none;Fusobacteriota;Fusobacteriia;Fusobacteriales;Fusobacteriaceae;Fusobacterium |
| Ilyobacter | 0 | 0 | 0 | 0 | 0 | 0 | 0 | 0 | 0 | 0 | 0 | 0 | 0 | 0 | Bacteria | Bacteria\_none | Fusobacteriota | Fusobacteriia | Fusobacteriales | Fusobacteriaceae | Bacteria;Bacteria\_none;Fusobacteriota;Fusobacteriia;Fusobacteriales;Fusobacteriaceae;Ilyobacter |
| Haliovirga | 0 | 0 | 0 | 0 | 0 | 0 | 0 | 0 | 0 | 0 | 0 | 0 | 0 | 1 | Bacteria | Bacteria\_none | Fusobacteriota | Fusobacteriia | Fusobacteriales | Haliovirgaceae | Bacteria;Bacteria\_none;Fusobacteriota;Fusobacteriia;Fusobacteriales;Haliovirgaceae;Haliovirga |
| Leptotrichia | 13 | 91 | 150 | 46 | 50 | 169 | 150 | 111 | 9 | 54 | 49 | 10 | 60 | 45 | Bacteria | Bacteria\_none | Fusobacteriota | Fusobacteriia | Fusobacteriales | Leptotrichiaceae | Bacteria;Bacteria\_none;Fusobacteriota;Fusobacteriia;Fusobacteriales;Leptotrichiaceae;Leptotrichia |
| Pseudoleptotrichia | 0 | 0 | 0 | 10 | 23 | 0 | 0 | 0 | 0 | 0 | 28 | 0 | 2 | 1 | Bacteria | Bacteria\_none | Fusobacteriota | Fusobacteriia | Fusobacteriales | Leptotrichiaceae | Bacteria;Bacteria\_none;Fusobacteriota;Fusobacteriia;Fusobacteriales;Leptotrichiaceae;Pseudoleptotrichia |
| Sebaldella | 0 | 0 | 0 | 0 | 0 | 0 | 0 | 0 | 0 | 0 | 0 | 0 | 0 | 0 | Bacteria | Bacteria\_none | Fusobacteriota | Fusobacteriia | Fusobacteriales | Leptotrichiaceae | Bacteria;Bacteria\_none;Fusobacteriota;Fusobacteriia;Fusobacteriales;Leptotrichiaceae;Sebaldella |
| Sneathia | 0 | 1 | 0 | 0 | 0 | 0 | 0 | 0 | 0 | 0 | 0 | 0 | 0 | 0 | Bacteria | Bacteria\_none | Fusobacteriota | Fusobacteriia | Fusobacteriales | Leptotrichiaceae | Bacteria;Bacteria\_none;Fusobacteriota;Fusobacteriia;Fusobacteriales;Leptotrichiaceae;Sneathia |
| Streptobacillus | 0 | 0 | 1 | 0 | 0 | 0 | 0 | 0 | 0 | 0 | 0 | 0 | 0 | 0 | Bacteria | Bacteria\_none | Fusobacteriota | Fusobacteriia | Fusobacteriales | Leptotrichiaceae | Bacteria;Bacteria\_none;Fusobacteriota;Fusobacteriia;Fusobacteriales;Leptotrichiaceae;Streptobacillus |
| Ignavibacterium | 0 | 0 | 0 | 0 | 0 | 0 | 0 | 0 | 0 | 0 | 0 | 0 | 0 | 0 | Bacteria | Bacteria\_none | Ignavibacteriota | Ignavibacteria | Ignavibacteriales | Ignavibacteriaceae | Bacteria;Bacteria\_none;Ignavibacteriota;Ignavibacteria;Ignavibacteriales;Ignavibacteriaceae;Ignavibacterium |
| Acholeplasma | 0 | 0 | 0 | 0 | 1 | 0 | 0 | 0 | 0 | 0 | 0 | 0 | 0 | 0 | Bacteria | Bacteria\_none | Mycoplasmatota | Mollicutes | Acholeplasmatales | Acholeplasmataceae | Bacteria;Bacteria\_none;Mycoplasmatota;Mollicutes;Acholeplasmatales;Acholeplasmataceae;Acholeplasma |
| Mariniplasma | 0 | 0 | 0 | 0 | 0 | 1 | 0 | 0 | 0 | 0 | 0 | 0 | 0 | 0 | Bacteria | Bacteria\_none | Mycoplasmatota | Mollicutes | Acholeplasmatales | Acholeplasmataceae | Bacteria;Bacteria\_none;Mycoplasmatota;Mollicutes;Acholeplasmatales;Acholeplasmataceae;Mariniplasma |
| Spiroplasma | 0 | 0 | 0 | 1 | 1 | 2 | 0 | 0 | 0 | 0 | 0 | 0 | 0 | 0 | Bacteria | Bacteria\_none | Mycoplasmatota | Mollicutes | Entomoplasmatales | Spiroplasmataceae | Bacteria;Bacteria\_none;Mycoplasmatota;Mollicutes;Entomoplasmatales;Spiroplasmataceae;Spiroplasma |
| Mycoplasma | 0 | 1 | 3 | 0 | 0 | 1 | 0 | 0 | 0 | 0 | 0 | 0 | 0 | 0 | Bacteria | Bacteria\_none | Mycoplasmatota | Mollicutes | Mycoplasmatales | Mycoplasmataceae | Bacteria;Bacteria\_none;Mycoplasmatota;Mollicutes;Mycoplasmatales;Mycoplasmataceae;Mycoplasma |
| Mycoplasmatales\_Incertae\_sedis | 0 | 0 | 0 | 0 | 0 | 0 | 0 | 0 | 0 | 0 | 0 | 0 | 0 | 0 | Bacteria | Bacteria\_none | Mycoplasmatota | Mollicutes | Mycoplasmatales | Mycoplasmatales\_Incertae\_sedis | Bacteria;Bacteria\_none;Mycoplasmatota;Mollicutes;Mycoplasmatales;Mycoplasmatales\_Incertae\_sedis;Mycoplasmatales\_Incertae\_sedis |
| Metamycoplasma | 0 | 8 | 32 | 1 | 2 | 4 | 0 | 1 | 0 | 6 | 0 | 4 | 3 | 3 | Bacteria | Bacteria\_none | Mycoplasmatota | Mycoplasmatota\_Incertae\_sedis | Mycoplasmoidales | Metamycoplasmataceae | Bacteria;Bacteria\_none;Mycoplasmatota;Mycoplasmatota\_Incertae\_sedis;Mycoplasmoidales;Metamycoplasmataceae;Metamycoplasma |
| Mycoplasmopsis | 0 | 2 | 2 | 0 | 0 | 0 | 0 | 0 | 0 | 0 | 0 | 0 | 0 | 0 | Bacteria | Bacteria\_none | Mycoplasmatota | Mycoplasmatota\_Incertae\_sedis | Mycoplasmoidales | Metamycoplasmataceae | Bacteria;Bacteria\_none;Mycoplasmatota;Mycoplasmatota\_Incertae\_sedis;Mycoplasmoidales;Metamycoplasmataceae;Mycoplasmopsis |
| Malacoplasma | 0 | 0 | 0 | 0 | 1 | 0 | 0 | 0 | 0 | 0 | 0 | 0 | 0 | 0 | Bacteria | Bacteria\_none | Mycoplasmatota | Mycoplasmatota\_Incertae\_sedis | Mycoplasmoidales | Mycoplasmoidaceae | Bacteria;Bacteria\_none;Mycoplasmatota;Mycoplasmatota\_Incertae\_sedis;Mycoplasmoidales;Mycoplasmoidaceae;Malacoplasma |
| Mycoplasmoides | 0 | 0 | 0 | 0 | 0 | 0 | 0 | 0 | 0 | 0 | 0 | 0 | 0 | 0 | Bacteria | Bacteria\_none | Mycoplasmatota | Mycoplasmatota\_Incertae\_sedis | Mycoplasmoidales | Mycoplasmoidaceae | Bacteria;Bacteria\_none;Mycoplasmatota;Mycoplasmatota\_Incertae\_sedis;Mycoplasmoidales;Mycoplasmoidaceae;Mycoplasmoides |
| Corallococcus | 0 | 0 | 0 | 0 | 0 | 0 | 0 | 0 | 0 | 0 | 0 | 0 | 0 | 0 | Bacteria | Bacteria\_none | Myxococcota | Myxococcia | Myxococcales | Myxococcaceae | Bacteria;Bacteria\_none;Myxococcota;Myxococcia;Myxococcales;Myxococcaceae;Corallococcus |
| Sorangium | 0 | 0 | 0 | 0 | 2 | 0 | 0 | 0 | 0 | 0 | 1 | 0 | 0 | 0 | Bacteria | Bacteria\_none | Myxococcota | Myxococcota\_Incertae\_sedis | Polyangiales | Polyangiaceae | Bacteria;Bacteria\_none;Myxococcota;Myxococcota\_Incertae\_sedis;Polyangiales;Polyangiaceae;Sorangium |
| Nitrospira | 0 | 0 | 0 | 0 | 0 | 0 | 0 | 0 | 0 | 0 | 0 | 0 | 0 | 0 | Bacteria | Bacteria\_none | Nitrospirota | Nitrospiria | Nitrospirales | Nitrospiraceae | Bacteria;Bacteria\_none;Nitrospirota;Nitrospiria;Nitrospirales;Nitrospiraceae;Nitrospira |
| Dissulfurispira | 0 | 0 | 0 | 0 | 0 | 0 | 0 | 0 | 0 | 0 | 0 | 0 | 0 | 0 | Bacteria | Bacteria\_none | Nitrospirota | Thermodesulfovibrionia | Thermodesulfovibrionales | Dissulfurispiraceae | Bacteria;Bacteria\_none;Nitrospirota;Thermodesulfovibrionia;Thermodesulfovibrionales;Dissulfurispiraceae;Dissulfurispira |
| Tuwongella | 0 | 0 | 0 | 0 | 0 | 0 | 0 | 0 | 0 | 0 | 0 | 0 | 0 | 0 | Bacteria | Bacteria\_none | Planctomycetota | Planctomycetia | Gemmatales | Gemmataceae | Bacteria;Bacteria\_none;Planctomycetota;Planctomycetia;Gemmatales;Gemmataceae;Tuwongella |
| Lignipirellula | 1 | 0 | 0 | 0 | 0 | 0 | 0 | 0 | 0 | 0 | 0 | 0 | 0 | 0 | Bacteria | Bacteria\_none | Planctomycetota | Planctomycetia | Pirellulales | Pirellulaceae | Bacteria;Bacteria\_none;Planctomycetota;Planctomycetia;Pirellulales;Pirellulaceae;Lignipirellula |
| Fuerstiella | 0 | 0 | 0 | 0 | 0 | 0 | 0 | 0 | 0 | 0 | 0 | 0 | 0 | 0 | Bacteria | Bacteria\_none | Planctomycetota | Planctomycetia | Planctomycetales | Planctomycetaceae | Bacteria;Bacteria\_none;Planctomycetota;Planctomycetia;Planctomycetales;Planctomycetaceae;Fuerstiella |
| Gimesia | 0 | 0 | 0 | 0 | 0 | 0 | 0 | 0 | 0 | 0 | 1 | 0 | 0 | 0 | Bacteria | Bacteria\_none | Planctomycetota | Planctomycetia | Planctomycetales | Planctomycetaceae | Bacteria;Bacteria\_none;Planctomycetota;Planctomycetia;Planctomycetales;Planctomycetaceae;Gimesia |
| Candidatus Pelagibacter | 0 | 0 | 0 | 0 | 0 | 0 | 0 | 0 | 0 | 0 | 0 | 0 | 0 | 0 | Bacteria | Bacteria\_none | Pseudomonadota | Alphaproteobacteria | Candidatus Pelagibacterales | Candidatus Pelagibacteraceae | Bacteria;Bacteria\_none;Pseudomonadota;Alphaproteobacteria;Candidatus Pelagibacterales;Candidatus Pelagibacteraceae;Candidatus Pelagibacter |
| Asticcacaulis | 0 | 0 | 0 | 0 | 0 | 0 | 0 | 0 | 0 | 0 | 0 | 0 | 0 | 0 | Bacteria | Bacteria\_none | Pseudomonadota | Alphaproteobacteria | Caulobacterales | Caulobacteraceae | Bacteria;Bacteria\_none;Pseudomonadota;Alphaproteobacteria;Caulobacterales;Caulobacteraceae;Asticcacaulis |
| Bosea | 0 | 0 | 0 | 0 | 0 | 1 | 0 | 0 | 0 | 0 | 0 | 0 | 0 | 0 | Bacteria | Bacteria\_none | Pseudomonadota | Alphaproteobacteria | Hyphomicrobiales | Boseaceae | Bacteria;Bacteria\_none;Pseudomonadota;Alphaproteobacteria;Hyphomicrobiales;Boseaceae;Bosea |
| Brucella | 0 | 0 | 0 | 0 | 0 | 0 | 0 | 0 | 0 | 0 | 0 | 0 | 0 | 0 | Bacteria | Bacteria\_none | Pseudomonadota | Alphaproteobacteria | Hyphomicrobiales | Brucellaceae | Bacteria;Bacteria\_none;Pseudomonadota;Alphaproteobacteria;Hyphomicrobiales;Brucellaceae;Brucella |
| Cohaesibacter | 0 | 0 | 0 | 0 | 0 | 0 | 0 | 0 | 0 | 0 | 0 | 0 | 0 | 0 | Bacteria | Bacteria\_none | Pseudomonadota | Alphaproteobacteria | Hyphomicrobiales | Cohaesibacteraceae | Bacteria;Bacteria\_none;Pseudomonadota;Alphaproteobacteria;Hyphomicrobiales;Cohaesibacteraceae;Cohaesibacter |
| Devosia | 0 | 0 | 0 | 0 | 0 | 0 | 0 | 0 | 0 | 0 | 0 | 0 | 0 | 0 | Bacteria | Bacteria\_none | Pseudomonadota | Alphaproteobacteria | Hyphomicrobiales | Devosiaceae | Bacteria;Bacteria\_none;Pseudomonadota;Alphaproteobacteria;Hyphomicrobiales;Devosiaceae;Devosia |
| Pelagibacterium | 0 | 0 | 1 | 0 | 0 | 0 | 0 | 0 | 0 | 0 | 0 | 0 | 0 | 0 | Bacteria | Bacteria\_none | Pseudomonadota | Alphaproteobacteria | Hyphomicrobiales | Devosiaceae | Bacteria;Bacteria\_none;Pseudomonadota;Alphaproteobacteria;Hyphomicrobiales;Devosiaceae;Pelagibacterium |
| Methylobacterium | 0 | 0 | 1 | 0 | 0 | 0 | 0 | 0 | 0 | 0 | 0 | 0 | 0 | 0 | Bacteria | Bacteria\_none | Pseudomonadota | Alphaproteobacteria | Hyphomicrobiales | Methylobacteriaceae | Bacteria;Bacteria\_none;Pseudomonadota;Alphaproteobacteria;Hyphomicrobiales;Methylobacteriaceae;Methylobacterium |
| Microvirga | 0 | 0 | 0 | 0 | 0 | 0 | 0 | 0 | 0 | 0 | 0 | 0 | 0 | 0 | Bacteria | Bacteria\_none | Pseudomonadota | Alphaproteobacteria | Hyphomicrobiales | Methylobacteriaceae | Bacteria;Bacteria\_none;Pseudomonadota;Alphaproteobacteria;Hyphomicrobiales;Methylobacteriaceae;Microvirga |
| Afipia | 0 | 1 | 0 | 0 | 0 | 0 | 0 | 0 | 0 | 0 | 0 | 0 | 0 | 0 | Bacteria | Bacteria\_none | Pseudomonadota | Alphaproteobacteria | Hyphomicrobiales | Nitrobacteraceae | Bacteria;Bacteria\_none;Pseudomonadota;Alphaproteobacteria;Hyphomicrobiales;Nitrobacteraceae;Afipia |
| Bradyrhizobium | 0 | 0 | 1 | 0 | 0 | 1 | 0 | 0 | 0 | 0 | 0 | 0 | 0 | 0 | Bacteria | Bacteria\_none | Pseudomonadota | Alphaproteobacteria | Hyphomicrobiales | Nitrobacteraceae | Bacteria;Bacteria\_none;Pseudomonadota;Alphaproteobacteria;Hyphomicrobiales;Nitrobacteraceae;Bradyrhizobium |
| Aquibium | 0 | 0 | 0 | 0 | 0 | 0 | 0 | 1 | 0 | 0 | 0 | 0 | 0 | 0 | Bacteria | Bacteria\_none | Pseudomonadota | Alphaproteobacteria | Hyphomicrobiales | Phyllobacteriaceae | Bacteria;Bacteria\_none;Pseudomonadota;Alphaproteobacteria;Hyphomicrobiales;Phyllobacteriaceae;Aquibium |
| Mesorhizobium | 0 | 0 | 1 | 0 | 0 | 1 | 0 | 1 | 0 | 0 | 0 | 0 | 0 | 0 | Bacteria | Bacteria\_none | Pseudomonadota | Alphaproteobacteria | Hyphomicrobiales | Phyllobacteriaceae | Bacteria;Bacteria\_none;Pseudomonadota;Alphaproteobacteria;Hyphomicrobiales;Phyllobacteriaceae;Mesorhizobium |
| Nitratireductor | 0 | 0 | 0 | 0 | 0 | 0 | 1 | 0 | 0 | 0 | 0 | 0 | 0 | 0 | Bacteria | Bacteria\_none | Pseudomonadota | Alphaproteobacteria | Hyphomicrobiales | Phyllobacteriaceae | Bacteria;Bacteria\_none;Pseudomonadota;Alphaproteobacteria;Hyphomicrobiales;Phyllobacteriaceae;Nitratireductor |
| Agrobacterium | 0 | 0 | 0 | 0 | 0 | 0 | 0 | 1 | 0 | 0 | 0 | 0 | 0 | 0 | Bacteria | Bacteria\_none | Pseudomonadota | Alphaproteobacteria | Hyphomicrobiales | Rhizobiaceae | Bacteria;Bacteria\_none;Pseudomonadota;Alphaproteobacteria;Hyphomicrobiales;Rhizobiaceae;Agrobacterium |
| Liberibacter | 0 | 0 | 0 | 0 | 0 | 0 | 0 | 0 | 0 | 0 | 0 | 0 | 0 | 0 | Bacteria | Bacteria\_none | Pseudomonadota | Alphaproteobacteria | Hyphomicrobiales | Rhizobiaceae | Bacteria;Bacteria\_none;Pseudomonadota;Alphaproteobacteria;Hyphomicrobiales;Rhizobiaceae;Liberibacter |
| Pararhizobium | 0 | 0 | 0 | 0 | 0 | 1 | 0 | 0 | 0 | 0 | 0 | 0 | 1 | 0 | Bacteria | Bacteria\_none | Pseudomonadota | Alphaproteobacteria | Hyphomicrobiales | Rhizobiaceae | Bacteria;Bacteria\_none;Pseudomonadota;Alphaproteobacteria;Hyphomicrobiales;Rhizobiaceae;Pararhizobium |
| Rhizobium | 0 | 0 | 0 | 0 | 0 | 0 | 0 | 0 | 0 | 0 | 0 | 0 | 1 | 1 | Bacteria | Bacteria\_none | Pseudomonadota | Alphaproteobacteria | Hyphomicrobiales | Rhizobiaceae | Bacteria;Bacteria\_none;Pseudomonadota;Alphaproteobacteria;Hyphomicrobiales;Rhizobiaceae;Rhizobium |
| Shinella | 0 | 0 | 0 | 0 | 0 | 1 | 0 | 0 | 0 | 0 | 0 | 0 | 0 | 0 | Bacteria | Bacteria\_none | Pseudomonadota | Alphaproteobacteria | Hyphomicrobiales | Rhizobiaceae | Bacteria;Bacteria\_none;Pseudomonadota;Alphaproteobacteria;Hyphomicrobiales;Rhizobiaceae;Shinella |
| Sinorhizobium | 1 | 0 | 0 | 0 | 0 | 0 | 0 | 0 | 0 | 0 | 0 | 0 | 0 | 0 | Bacteria | Bacteria\_none | Pseudomonadota | Alphaproteobacteria | Hyphomicrobiales | Rhizobiaceae | Bacteria;Bacteria\_none;Pseudomonadota;Alphaproteobacteria;Hyphomicrobiales;Rhizobiaceae;Sinorhizobium |
| Kordiimonas | 0 | 0 | 0 | 0 | 0 | 0 | 0 | 0 | 0 | 0 | 0 | 0 | 0 | 0 | Bacteria | Bacteria\_none | Pseudomonadota | Alphaproteobacteria | Kordiimonadales | Kordiimonadaceae | Bacteria;Bacteria\_none;Pseudomonadota;Alphaproteobacteria;Kordiimonadales;Kordiimonadaceae;Kordiimonas |
| Cereibacter | 0 | 0 | 0 | 0 | 0 | 0 | 0 | 0 | 0 | 0 | 0 | 0 | 0 | 0 | Bacteria | Bacteria\_none | Pseudomonadota | Alphaproteobacteria | Rhodobacterales | Paracoccaceae | Bacteria;Bacteria\_none;Pseudomonadota;Alphaproteobacteria;Rhodobacterales;Paracoccaceae;Cereibacter |
| Paracoccus | 0 | 0 | 0 | 0 | 0 | 1 | 0 | 0 | 0 | 0 | 0 | 0 | 0 | 0 | Bacteria | Bacteria\_none | Pseudomonadota | Alphaproteobacteria | Rhodobacterales | Paracoccaceae | Bacteria;Bacteria\_none;Pseudomonadota;Alphaproteobacteria;Rhodobacterales;Paracoccaceae;Paracoccus |
| Tabrizicola | 0 | 0 | 0 | 0 | 0 | 0 | 0 | 0 | 0 | 0 | 0 | 0 | 0 | 1 | Bacteria | Bacteria\_none | Pseudomonadota | Alphaproteobacteria | Rhodobacterales | Paracoccaceae | Bacteria;Bacteria\_none;Pseudomonadota;Alphaproteobacteria;Rhodobacterales;Paracoccaceae;Tabrizicola |
| Phaeobacter | 0 | 0 | 0 | 0 | 1 | 0 | 0 | 0 | 0 | 0 | 0 | 0 | 1 | 0 | Bacteria | Bacteria\_none | Pseudomonadota | Alphaproteobacteria | Rhodobacterales | Roseobacteraceae | Bacteria;Bacteria\_none;Pseudomonadota;Alphaproteobacteria;Rhodobacterales;Roseobacteraceae;Phaeobacter |
| Ponticoccus | 1 | 0 | 0 | 0 | 0 | 0 | 0 | 0 | 0 | 0 | 0 | 0 | 0 | 0 | Bacteria | Bacteria\_none | Pseudomonadota | Alphaproteobacteria | Rhodobacterales | Roseobacteraceae | Bacteria;Bacteria\_none;Pseudomonadota;Alphaproteobacteria;Rhodobacterales;Roseobacteraceae;Ponticoccus |
| Pseudosulfitobacter | 0 | 1 | 0 | 0 | 0 | 0 | 0 | 0 | 0 | 0 | 0 | 0 | 0 | 0 | Bacteria | Bacteria\_none | Pseudomonadota | Alphaproteobacteria | Rhodobacterales | Roseobacteraceae | Bacteria;Bacteria\_none;Pseudomonadota;Alphaproteobacteria;Rhodobacterales;Roseobacteraceae;Pseudosulfitobacter |
| Roseobacter | 0 | 0 | 0 | 1 | 0 | 0 | 0 | 0 | 0 | 0 | 0 | 0 | 0 | 0 | Bacteria | Bacteria\_none | Pseudomonadota | Alphaproteobacteria | Rhodobacterales | Roseobacteraceae | Bacteria;Bacteria\_none;Pseudomonadota;Alphaproteobacteria;Rhodobacterales;Roseobacteraceae;Roseobacter |
| Ruegeria | 0 | 0 | 0 | 0 | 0 | 0 | 0 | 0 | 0 | 0 | 0 | 0 | 0 | 0 | Bacteria | Bacteria\_none | Pseudomonadota | Alphaproteobacteria | Rhodobacterales | Roseobacteraceae | Bacteria;Bacteria\_none;Pseudomonadota;Alphaproteobacteria;Rhodobacterales;Roseobacteraceae;Ruegeria |
| Sulfitobacter | 0 | 0 | 0 | 0 | 0 | 0 | 0 | 0 | 0 | 0 | 0 | 0 | 0 | 0 | Bacteria | Bacteria\_none | Pseudomonadota | Alphaproteobacteria | Rhodobacterales | Roseobacteraceae | Bacteria;Bacteria\_none;Pseudomonadota;Alphaproteobacteria;Rhodobacterales;Roseobacteraceae;Sulfitobacter |
| Komagataeibacter | 0 | 0 | 0 | 0 | 0 | 1 | 0 | 0 | 0 | 0 | 0 | 0 | 0 | 0 | Bacteria | Bacteria\_none | Pseudomonadota | Alphaproteobacteria | Rhodospirillales | Acetobacteraceae | Bacteria;Bacteria\_none;Pseudomonadota;Alphaproteobacteria;Rhodospirillales;Acetobacteraceae;Komagataeibacter |
| Pseudoroseomonas | 0 | 0 | 0 | 0 | 0 | 0 | 1 | 0 | 0 | 0 | 0 | 0 | 0 | 0 | Bacteria | Bacteria\_none | Pseudomonadota | Alphaproteobacteria | Rhodospirillales | Acetobacteraceae | Bacteria;Bacteria\_none;Pseudomonadota;Alphaproteobacteria;Rhodospirillales;Acetobacteraceae;Pseudoroseomonas |
| Azospirillum | 0 | 0 | 0 | 0 | 0 | 0 | 0 | 0 | 0 | 0 | 1 | 0 | 0 | 0 | Bacteria | Bacteria\_none | Pseudomonadota | Alphaproteobacteria | Rhodospirillales | Azospirillaceae | Bacteria;Bacteria\_none;Pseudomonadota;Alphaproteobacteria;Rhodospirillales;Azospirillaceae;Azospirillum |
| Varunaivibrio | 0 | 0 | 0 | 0 | 0 | 0 | 0 | 0 | 0 | 0 | 0 | 0 | 0 | 0 | Bacteria | Bacteria\_none | Pseudomonadota | Alphaproteobacteria | Rhodospirillales | Magnetovibrionaceae | Bacteria;Bacteria\_none;Pseudomonadota;Alphaproteobacteria;Rhodospirillales;Magnetovibrionaceae;Varunaivibrio |
| Thalassospira | 0 | 0 | 0 | 0 | 0 | 0 | 0 | 0 | 0 | 0 | 0 | 0 | 0 | 0 | Bacteria | Bacteria\_none | Pseudomonadota | Alphaproteobacteria | Rhodospirillales | Thalassospiraceae | Bacteria;Bacteria\_none;Pseudomonadota;Alphaproteobacteria;Rhodospirillales;Thalassospiraceae;Thalassospira |
| Neorickettsia | 0 | 1 | 0 | 0 | 0 | 0 | 0 | 0 | 0 | 0 | 0 | 0 | 0 | 0 | Bacteria | Bacteria\_none | Pseudomonadota | Alphaproteobacteria | Rickettsiales | Anaplasmataceae | Bacteria;Bacteria\_none;Pseudomonadota;Alphaproteobacteria;Rickettsiales;Anaplasmataceae;Neorickettsia |
| Wolbachia | 0 | 0 | 0 | 0 | 0 | 0 | 0 | 0 | 0 | 0 | 0 | 0 | 0 | 0 | Bacteria | Bacteria\_none | Pseudomonadota | Alphaproteobacteria | Rickettsiales | Anaplasmataceae | Bacteria;Bacteria\_none;Pseudomonadota;Alphaproteobacteria;Rickettsiales;Anaplasmataceae;Wolbachia |
| Candidatus Phycorickettsia | 0 | 0 | 0 | 0 | 0 | 0 | 1 | 0 | 0 | 0 | 0 | 0 | 0 | 0 | Bacteria | Bacteria\_none | Pseudomonadota | Alphaproteobacteria | Rickettsiales | Rickettsiaceae | Bacteria;Bacteria\_none;Pseudomonadota;Alphaproteobacteria;Rickettsiales;Rickettsiaceae;Candidatus Phycorickettsia |
| Novosphingobium | 0 | 0 | 0 | 0 | 0 | 0 | 0 | 0 | 0 | 0 | 0 | 0 | 0 | 0 | Bacteria | Bacteria\_none | Pseudomonadota | Alphaproteobacteria | Sphingomonadales | Sphingomonadaceae | Bacteria;Bacteria\_none;Pseudomonadota;Alphaproteobacteria;Sphingomonadales;Sphingomonadaceae;Novosphingobium |
| Sphingomonas | 0 | 0 | 0 | 0 | 0 | 0 | 0 | 0 | 0 | 0 | 0 | 1 | 0 | 0 | Bacteria | Bacteria\_none | Pseudomonadota | Alphaproteobacteria | Sphingomonadales | Sphingomonadaceae | Bacteria;Bacteria\_none;Pseudomonadota;Alphaproteobacteria;Sphingomonadales;Sphingomonadaceae;Sphingomonas |
| Sphingopyxis | 0 | 0 | 0 | 1 | 0 | 1 | 0 | 0 | 0 | 0 | 0 | 0 | 0 | 0 | Bacteria | Bacteria\_none | Pseudomonadota | Alphaproteobacteria | Sphingomonadales | Sphingomonadaceae | Bacteria;Bacteria\_none;Pseudomonadota;Alphaproteobacteria;Sphingomonadales;Sphingomonadaceae;Sphingopyxis |
| Candidatus Profftella | 0 | 0 | 0 | 0 | 0 | 0 | 0 | 0 | 0 | 0 | 0 | 0 | 0 | 0 | Bacteria | Bacteria\_none | Pseudomonadota | Betaproteobacteria | Betaproteobacteria\_Incertae\_sedis | Betaproteobacteria\_Incertae\_sedis | Bacteria;Bacteria\_none;Pseudomonadota;Betaproteobacteria;Betaproteobacteria\_Incertae\_sedis;Betaproteobacteria\_Incertae\_sedis;Candidatus Profftella |
| Casimicrobium | 0 | 0 | 0 | 0 | 0 | 0 | 0 | 0 | 0 | 0 | 0 | 0 | 0 | 0 | Bacteria | Bacteria\_none | Pseudomonadota | Betaproteobacteria | Betaproteobacteria\_Incertae\_sedis | Casimicrobiaceae | Bacteria;Bacteria\_none;Pseudomonadota;Betaproteobacteria;Betaproteobacteria\_Incertae\_sedis;Casimicrobiaceae;Casimicrobium |
| Advenella | 0 | 0 | 0 | 0 | 1 | 0 | 0 | 0 | 0 | 0 | 0 | 0 | 0 | 0 | Bacteria | Bacteria\_none | Pseudomonadota | Betaproteobacteria | Burkholderiales | Alcaligenaceae | Bacteria;Bacteria\_none;Pseudomonadota;Betaproteobacteria;Burkholderiales;Alcaligenaceae;Advenella |
| Bordetella | 0 | 0 | 0 | 0 | 0 | 0 | 0 | 0 | 0 | 0 | 0 | 0 | 0 | 0 | Bacteria | Bacteria\_none | Pseudomonadota | Betaproteobacteria | Burkholderiales | Alcaligenaceae | Bacteria;Bacteria\_none;Pseudomonadota;Betaproteobacteria;Burkholderiales;Alcaligenaceae;Bordetella |
| Neopusillimonas | 0 | 0 | 0 | 0 | 0 | 0 | 0 | 0 | 0 | 0 | 0 | 0 | 0 | 0 | Bacteria | Bacteria\_none | Pseudomonadota | Betaproteobacteria | Burkholderiales | Alcaligenaceae | Bacteria;Bacteria\_none;Pseudomonadota;Betaproteobacteria;Burkholderiales;Alcaligenaceae;Neopusillimonas |
| Orrella | 0 | 0 | 0 | 0 | 0 | 1 | 0 | 0 | 0 | 0 | 0 | 0 | 0 | 0 | Bacteria | Bacteria\_none | Pseudomonadota | Betaproteobacteria | Burkholderiales | Alcaligenaceae | Bacteria;Bacteria\_none;Pseudomonadota;Betaproteobacteria;Burkholderiales;Alcaligenaceae;Orrella |
| Pollutimonas | 0 | 0 | 0 | 0 | 0 | 0 | 0 | 0 | 0 | 0 | 0 | 0 | 0 | 0 | Bacteria | Bacteria\_none | Pseudomonadota | Betaproteobacteria | Burkholderiales | Alcaligenaceae | Bacteria;Bacteria\_none;Pseudomonadota;Betaproteobacteria;Burkholderiales;Alcaligenaceae;Pollutimonas |
| Pusillimonas | 0 | 0 | 0 | 0 | 0 | 0 | 0 | 0 | 0 | 0 | 0 | 0 | 0 | 0 | Bacteria | Bacteria\_none | Pseudomonadota | Betaproteobacteria | Burkholderiales | Alcaligenaceae | Bacteria;Bacteria\_none;Pseudomonadota;Betaproteobacteria;Burkholderiales;Alcaligenaceae;Pusillimonas |
| Burkholderia | 0 | 0 | 1 | 0 | 0 | 1 | 0 | 0 | 0 | 0 | 0 | 0 | 0 | 0 | Bacteria | Bacteria\_none | Pseudomonadota | Betaproteobacteria | Burkholderiales | Burkholderiaceae | Bacteria;Bacteria\_none;Pseudomonadota;Betaproteobacteria;Burkholderiales;Burkholderiaceae;Burkholderia |
| Cupriavidus | 0 | 0 | 0 | 0 | 0 | 0 | 0 | 0 | 0 | 0 | 0 | 0 | 0 | 0 | Bacteria | Bacteria\_none | Pseudomonadota | Betaproteobacteria | Burkholderiales | Burkholderiaceae | Bacteria;Bacteria\_none;Pseudomonadota;Betaproteobacteria;Burkholderiales;Burkholderiaceae;Cupriavidus |
| Lautropia | 0 | 1 | 0 | 0 | 3 | 0 | 0 | 0 | 0 | 0 | 7 | 0 | 0 | 0 | Bacteria | Bacteria\_none | Pseudomonadota | Betaproteobacteria | Burkholderiales | Burkholderiaceae | Bacteria;Bacteria\_none;Pseudomonadota;Betaproteobacteria;Burkholderiales;Burkholderiaceae;Lautropia |
| Mycoavidus | 0 | 0 | 0 | 0 | 0 | 0 | 0 | 0 | 0 | 1 | 0 | 0 | 0 | 0 | Bacteria | Bacteria\_none | Pseudomonadota | Betaproteobacteria | Burkholderiales | Burkholderiaceae | Bacteria;Bacteria\_none;Pseudomonadota;Betaproteobacteria;Burkholderiales;Burkholderiaceae;Mycoavidus |
| Pandoraea | 1 | 0 | 0 | 0 | 0 | 0 | 0 | 0 | 0 | 0 | 0 | 0 | 0 | 0 | Bacteria | Bacteria\_none | Pseudomonadota | Betaproteobacteria | Burkholderiales | Burkholderiaceae | Bacteria;Bacteria\_none;Pseudomonadota;Betaproteobacteria;Burkholderiales;Burkholderiaceae;Pandoraea |
| Paraburkholderia | 0 | 0 | 0 | 0 | 0 | 0 | 0 | 0 | 0 | 0 | 0 | 0 | 0 | 0 | Bacteria | Bacteria\_none | Pseudomonadota | Betaproteobacteria | Burkholderiales | Burkholderiaceae | Bacteria;Bacteria\_none;Pseudomonadota;Betaproteobacteria;Burkholderiales;Burkholderiaceae;Paraburkholderia |
| Polynucleobacter | 0 | 0 | 0 | 0 | 0 | 0 | 0 | 0 | 0 | 0 | 0 | 0 | 0 | 0 | Bacteria | Bacteria\_none | Pseudomonadota | Betaproteobacteria | Burkholderiales | Burkholderiaceae | Bacteria;Bacteria\_none;Pseudomonadota;Betaproteobacteria;Burkholderiales;Burkholderiaceae;Polynucleobacter |
| Ralstonia | 0 | 0 | 0 | 0 | 1 | 0 | 0 | 0 | 0 | 0 | 0 | 0 | 0 | 0 | Bacteria | Bacteria\_none | Pseudomonadota | Betaproteobacteria | Burkholderiales | Burkholderiaceae | Bacteria;Bacteria\_none;Pseudomonadota;Betaproteobacteria;Burkholderiales;Burkholderiaceae;Ralstonia |
| Acidovorax | 0 | 0 | 0 | 0 | 0 | 0 | 0 | 0 | 0 | 0 | 0 | 0 | 0 | 0 | Bacteria | Bacteria\_none | Pseudomonadota | Betaproteobacteria | Burkholderiales | Comamonadaceae | Bacteria;Bacteria\_none;Pseudomonadota;Betaproteobacteria;Burkholderiales;Comamonadaceae;Acidovorax |
| Hydrogenophaga | 0 | 0 | 0 | 0 | 0 | 1 | 0 | 0 | 0 | 0 | 0 | 0 | 0 | 0 | Bacteria | Bacteria\_none | Pseudomonadota | Betaproteobacteria | Burkholderiales | Comamonadaceae | Bacteria;Bacteria\_none;Pseudomonadota;Betaproteobacteria;Burkholderiales;Comamonadaceae;Hydrogenophaga |
| Ottowia | 0 | 0 | 0 | 0 | 0 | 0 | 0 | 0 | 0 | 0 | 0 | 0 | 0 | 0 | Bacteria | Bacteria\_none | Pseudomonadota | Betaproteobacteria | Burkholderiales | Comamonadaceae | Bacteria;Bacteria\_none;Pseudomonadota;Betaproteobacteria;Burkholderiales;Comamonadaceae;Ottowia |
| Variovorax | 0 | 0 | 0 | 0 | 0 | 0 | 0 | 0 | 0 | 0 | 0 | 0 | 0 | 0 | Bacteria | Bacteria\_none | Pseudomonadota | Betaproteobacteria | Burkholderiales | Comamonadaceae | Bacteria;Bacteria\_none;Pseudomonadota;Betaproteobacteria;Burkholderiales;Comamonadaceae;Variovorax |
| Candidatus Zinderia | 0 | 0 | 0 | 0 | 1 | 0 | 0 | 0 | 0 | 0 | 0 | 0 | 0 | 0 | Bacteria | Bacteria\_none | Pseudomonadota | Betaproteobacteria | Burkholderiales | Oxalobacteraceae | Bacteria;Bacteria\_none;Pseudomonadota;Betaproteobacteria;Burkholderiales;Oxalobacteraceae;Candidatus Zinderia |
| Janthinobacterium | 0 | 0 | 0 | 0 | 0 | 0 | 0 | 0 | 0 | 0 | 0 | 0 | 0 | 0 | Bacteria | Bacteria\_none | Pseudomonadota | Betaproteobacteria | Burkholderiales | Oxalobacteraceae | Bacteria;Bacteria\_none;Pseudomonadota;Betaproteobacteria;Burkholderiales;Oxalobacteraceae;Janthinobacterium |
| Massilia | 0 | 0 | 0 | 0 | 0 | 0 | 0 | 0 | 0 | 1 | 0 | 0 | 0 | 0 | Bacteria | Bacteria\_none | Pseudomonadota | Betaproteobacteria | Burkholderiales | Oxalobacteraceae | Bacteria;Bacteria\_none;Pseudomonadota;Betaproteobacteria;Burkholderiales;Oxalobacteraceae;Massilia |
| Noviherbaspirillum | 0 | 0 | 1 | 0 | 0 | 0 | 0 | 0 | 0 | 0 | 0 | 0 | 0 | 0 | Bacteria | Bacteria\_none | Pseudomonadota | Betaproteobacteria | Burkholderiales | Oxalobacteraceae | Bacteria;Bacteria\_none;Pseudomonadota;Betaproteobacteria;Burkholderiales;Oxalobacteraceae;Noviherbaspirillum |
| Oxalobacter | 0 | 0 | 0 | 0 | 0 | 0 | 0 | 0 | 0 | 0 | 0 | 0 | 0 | 0 | Bacteria | Bacteria\_none | Pseudomonadota | Betaproteobacteria | Burkholderiales | Oxalobacteraceae | Bacteria;Bacteria\_none;Pseudomonadota;Betaproteobacteria;Burkholderiales;Oxalobacteraceae;Oxalobacter |
| Pseudoduganella | 0 | 0 | 0 | 0 | 0 | 0 | 0 | 0 | 1 | 0 | 0 | 0 | 0 | 0 | Bacteria | Bacteria\_none | Pseudomonadota | Betaproteobacteria | Burkholderiales | Oxalobacteraceae | Bacteria;Bacteria\_none;Pseudomonadota;Betaproteobacteria;Burkholderiales;Oxalobacteraceae;Pseudoduganella |
| Piscinibacter | 0 | 0 | 0 | 0 | 0 | 0 | 0 | 0 | 0 | 0 | 0 | 1 | 0 | 0 | Bacteria | Bacteria\_none | Pseudomonadota | Betaproteobacteria | Burkholderiales | Sphaerotilaceae | Bacteria;Bacteria\_none;Pseudomonadota;Betaproteobacteria;Burkholderiales;Sphaerotilaceae;Piscinibacter |
| Rubrivivax | 0 | 0 | 0 | 0 | 0 | 0 | 0 | 0 | 0 | 0 | 1 | 0 | 0 | 0 | Bacteria | Bacteria\_none | Pseudomonadota | Betaproteobacteria | Burkholderiales | Sphaerotilaceae | Bacteria;Bacteria\_none;Pseudomonadota;Betaproteobacteria;Burkholderiales;Sphaerotilaceae;Rubrivivax |
| Alysiella | 0 | 0 | 0 | 0 | 1 | 0 | 0 | 0 | 0 | 0 | 0 | 0 | 0 | 0 | Bacteria | Bacteria\_none | Pseudomonadota | Betaproteobacteria | Neisseriales | Neisseriaceae | Bacteria;Bacteria\_none;Pseudomonadota;Betaproteobacteria;Neisseriales;Neisseriaceae;Alysiella |
| Aquella | 0 | 0 | 0 | 0 | 0 | 1 | 0 | 0 | 0 | 0 | 0 | 0 | 0 | 0 | Bacteria | Bacteria\_none | Pseudomonadota | Betaproteobacteria | Neisseriales | Neisseriaceae | Bacteria;Bacteria\_none;Pseudomonadota;Betaproteobacteria;Neisseriales;Neisseriaceae;Aquella |
| Conchiformibius | 0 | 0 | 1 | 0 | 0 | 0 | 0 | 0 | 0 | 0 | 0 | 0 | 0 | 0 | Bacteria | Bacteria\_none | Pseudomonadota | Betaproteobacteria | Neisseriales | Neisseriaceae | Bacteria;Bacteria\_none;Pseudomonadota;Betaproteobacteria;Neisseriales;Neisseriaceae;Conchiformibius |
| Eikenella | 0 | 2 | 30 | 7 | 9 | 0 | 0 | 0 | 0 | 2 | 2 | 1 | 2 | 0 | Bacteria | Bacteria\_none | Pseudomonadota | Betaproteobacteria | Neisseriales | Neisseriaceae | Bacteria;Bacteria\_none;Pseudomonadota;Betaproteobacteria;Neisseriales;Neisseriaceae;Eikenella |
| Kingella | 1 | 24 | 3 | 26 | 0 | 0 | 0 | 1 | 0 | 9 | 3 | 0 | 3 | 0 | Bacteria | Bacteria\_none | Pseudomonadota | Betaproteobacteria | Neisseriales | Neisseriaceae | Bacteria;Bacteria\_none;Pseudomonadota;Betaproteobacteria;Neisseriales;Neisseriaceae;Kingella |
| Morococcus | 0 | 3 | 9 | 14 | 0 | 0 | 0 | 0 | 0 | 2 | 1 | 1 | 1 | 0 | Bacteria | Bacteria\_none | Pseudomonadota | Betaproteobacteria | Neisseriales | Neisseriaceae | Bacteria;Bacteria\_none;Pseudomonadota;Betaproteobacteria;Neisseriales;Neisseriaceae;Morococcus |
| Neisseria | 174 | 164 | 939 | 2171 | 196 | 234 | 19 | 43 | 0 | 860 | 68 | 57 | 278 | 150 | Bacteria | Bacteria\_none | Pseudomonadota | Betaproteobacteria | Neisseriales | Neisseriaceae | Bacteria;Bacteria\_none;Pseudomonadota;Betaproteobacteria;Neisseriales;Neisseriaceae;Neisseria |
| Simonsiella | 0 | 0 | 0 | 1 | 0 | 0 | 0 | 0 | 0 | 0 | 0 | 0 | 0 | 0 | Bacteria | Bacteria\_none | Pseudomonadota | Betaproteobacteria | Neisseriales | Neisseriaceae | Bacteria;Bacteria\_none;Pseudomonadota;Betaproteobacteria;Neisseriales;Neisseriaceae;Simonsiella |
| Snodgrassella | 0 | 0 | 0 | 0 | 0 | 1 | 0 | 0 | 0 | 0 | 0 | 0 | 0 | 0 | Bacteria | Bacteria\_none | Pseudomonadota | Betaproteobacteria | Neisseriales | Neisseriaceae | Bacteria;Bacteria\_none;Pseudomonadota;Betaproteobacteria;Neisseriales;Neisseriaceae;Snodgrassella |
| Uruburuella | 0 | 0 | 0 | 0 | 0 | 0 | 0 | 0 | 0 | 0 | 0 | 0 | 0 | 0 | Bacteria | Bacteria\_none | Pseudomonadota | Betaproteobacteria | Neisseriales | Neisseriaceae | Bacteria;Bacteria\_none;Pseudomonadota;Betaproteobacteria;Neisseriales;Neisseriaceae;Uruburuella |
| Ferriphaselus | 0 | 0 | 0 | 0 | 0 | 0 | 0 | 0 | 0 | 0 | 0 | 0 | 0 | 0 | Bacteria | Bacteria\_none | Pseudomonadota | Betaproteobacteria | Nitrosomonadales | Gallionellaceae | Bacteria;Bacteria\_none;Pseudomonadota;Betaproteobacteria;Nitrosomonadales;Gallionellaceae;Ferriphaselus |
| Methylophilus | 0 | 0 | 0 | 0 | 0 | 0 | 0 | 0 | 0 | 0 | 0 | 0 | 0 | 0 | Bacteria | Bacteria\_none | Pseudomonadota | Betaproteobacteria | Nitrosomonadales | Methylophilaceae | Bacteria;Bacteria\_none;Pseudomonadota;Betaproteobacteria;Nitrosomonadales;Methylophilaceae;Methylophilus |
| Dechloromonas | 0 | 0 | 0 | 0 | 0 | 0 | 0 | 0 | 0 | 0 | 0 | 0 | 0 | 0 | Bacteria | Bacteria\_none | Pseudomonadota | Betaproteobacteria | Rhodocyclales | Azonexaceae | Bacteria;Bacteria\_none;Pseudomonadota;Betaproteobacteria;Rhodocyclales;Azonexaceae;Dechloromonas |
| Aeromonas | 0 | 0 | 0 | 0 | 0 | 1 | 0 | 0 | 0 | 0 | 0 | 0 | 0 | 0 | Bacteria | Bacteria\_none | Pseudomonadota | Gammaproteobacteria | Aeromonadales | Aeromonadaceae | Bacteria;Bacteria\_none;Pseudomonadota;Gammaproteobacteria;Aeromonadales;Aeromonadaceae;Aeromonas |
| Oceanisphaera | 0 | 0 | 0 | 0 | 0 | 0 | 0 | 0 | 0 | 0 | 0 | 0 | 0 | 1 | Bacteria | Bacteria\_none | Pseudomonadota | Gammaproteobacteria | Aeromonadales | Aeromonadaceae | Bacteria;Bacteria\_none;Pseudomonadota;Gammaproteobacteria;Aeromonadales;Aeromonadaceae;Oceanisphaera |
| Tolumonas | 0 | 0 | 0 | 0 | 0 | 1 | 0 | 0 | 0 | 0 | 0 | 0 | 0 | 0 | Bacteria | Bacteria\_none | Pseudomonadota | Gammaproteobacteria | Aeromonadales | Aeromonadaceae | Bacteria;Bacteria\_none;Pseudomonadota;Gammaproteobacteria;Aeromonadales;Aeromonadaceae;Tolumonas |
| Alteromonas | 1 | 0 | 0 | 0 | 0 | 0 | 0 | 0 | 0 | 0 | 0 | 0 | 0 | 0 | Bacteria | Bacteria\_none | Pseudomonadota | Gammaproteobacteria | Alteromonadales | Alteromonadaceae | Bacteria;Bacteria\_none;Pseudomonadota;Gammaproteobacteria;Alteromonadales;Alteromonadaceae;Alteromonas |
| Thalassotalea | 0 | 0 | 0 | 0 | 0 | 0 | 0 | 0 | 0 | 0 | 0 | 0 | 0 | 0 | Bacteria | Bacteria\_none | Pseudomonadota | Gammaproteobacteria | Alteromonadales | Colwelliaceae | Bacteria;Bacteria\_none;Pseudomonadota;Gammaproteobacteria;Alteromonadales;Colwelliaceae;Thalassotalea |
| Ferrimonas | 0 | 0 | 0 | 0 | 0 | 0 | 0 | 0 | 0 | 0 | 0 | 0 | 0 | 0 | Bacteria | Bacteria\_none | Pseudomonadota | Gammaproteobacteria | Alteromonadales | Ferrimonadaceae | Bacteria;Bacteria\_none;Pseudomonadota;Gammaproteobacteria;Alteromonadales;Ferrimonadaceae;Ferrimonas |
| Flocculibacter | 1 | 0 | 0 | 0 | 0 | 0 | 0 | 0 | 0 | 0 | 0 | 0 | 0 | 0 | Bacteria | Bacteria\_none | Pseudomonadota | Gammaproteobacteria | Alteromonadales | Pseudoalteromonadaceae | Bacteria;Bacteria\_none;Pseudomonadota;Gammaproteobacteria;Alteromonadales;Pseudoalteromonadaceae;Flocculibacter |
| Pseudoalteromonas | 0 | 0 | 0 | 0 | 0 | 2 | 0 | 2 | 0 | 0 | 1 | 0 | 1 | 0 | Bacteria | Bacteria\_none | Pseudomonadota | Gammaproteobacteria | Alteromonadales | Pseudoalteromonadaceae | Bacteria;Bacteria\_none;Pseudomonadota;Gammaproteobacteria;Alteromonadales;Pseudoalteromonadaceae;Pseudoalteromonas |
| Shewanella | 1 | 1 | 0 | 0 | 1 | 0 | 0 | 0 | 0 | 0 | 0 | 1 | 0 | 0 | Bacteria | Bacteria\_none | Pseudomonadota | Gammaproteobacteria | Alteromonadales | Shewanellaceae | Bacteria;Bacteria\_none;Pseudomonadota;Gammaproteobacteria;Alteromonadales;Shewanellaceae;Shewanella |
| Cardiobacterium | 0 | 2 | 14 | 0 | 1 | 6 | 0 | 0 | 0 | 1 | 6 | 1 | 1 | 1 | Bacteria | Bacteria\_none | Pseudomonadota | Gammaproteobacteria | Cardiobacteriales | Cardiobacteriaceae | Bacteria;Bacteria\_none;Pseudomonadota;Gammaproteobacteria;Cardiobacteriales;Cardiobacteriaceae;Cardiobacterium |
| Ignatzschineria | 0 | 1 | 0 | 0 | 0 | 0 | 0 | 0 | 0 | 0 | 0 | 0 | 0 | 0 | Bacteria | Bacteria\_none | Pseudomonadota | Gammaproteobacteria | Cardiobacteriales | Ignatzschineriaceae | Bacteria;Bacteria\_none;Pseudomonadota;Gammaproteobacteria;Cardiobacteriales;Ignatzschineriaceae;Ignatzschineria |
| Agarilytica | 0 | 0 | 0 | 0 | 0 | 0 | 0 | 0 | 0 | 0 | 0 | 0 | 0 | 0 | Bacteria | Bacteria\_none | Pseudomonadota | Gammaproteobacteria | Cellvibrionales | Cellvibrionaceae | Bacteria;Bacteria\_none;Pseudomonadota;Gammaproteobacteria;Cellvibrionales;Cellvibrionaceae;Agarilytica |
| Allochromatium | 0 | 0 | 0 | 0 | 0 | 0 | 0 | 0 | 0 | 0 | 0 | 0 | 0 | 0 | Bacteria | Bacteria\_none | Pseudomonadota | Gammaproteobacteria | Chromatiales | Chromatiaceae | Bacteria;Bacteria\_none;Pseudomonadota;Gammaproteobacteria;Chromatiales;Chromatiaceae;Allochromatium |
| Candidatus Nitrosoglobus | 0 | 0 | 0 | 0 | 0 | 0 | 0 | 0 | 0 | 0 | 0 | 0 | 0 | 0 | Bacteria | Bacteria\_none | Pseudomonadota | Gammaproteobacteria | Chromatiales | Chromatiaceae | Bacteria;Bacteria\_none;Pseudomonadota;Gammaproteobacteria;Chromatiales;Chromatiaceae;Candidatus Nitrosoglobus |
| Thioalkalivibrio | 0 | 0 | 0 | 1 | 0 | 0 | 0 | 0 | 0 | 0 | 0 | 0 | 0 | 0 | Bacteria | Bacteria\_none | Pseudomonadota | Gammaproteobacteria | Chromatiales | Ectothiorhodospiraceae | Bacteria;Bacteria\_none;Pseudomonadota;Gammaproteobacteria;Chromatiales;Ectothiorhodospiraceae;Thioalkalivibrio |
| Limnobaculum | 0 | 0 | 0 | 0 | 0 | 0 | 0 | 0 | 0 | 0 | 0 | 0 | 0 | 0 | Bacteria | Bacteria\_none | Pseudomonadota | Gammaproteobacteria | Enterobacterales | Budviciaceae | Bacteria;Bacteria\_none;Pseudomonadota;Gammaproteobacteria;Enterobacterales;Budviciaceae;Limnobaculum |
| Candidatus Blochmanniella | 0 | 0 | 0 | 0 | 0 | 0 | 0 | 0 | 0 | 0 | 0 | 0 | 0 | 0 | Bacteria | Bacteria\_none | Pseudomonadota | Gammaproteobacteria | Enterobacterales | Enterobacteriaceae | Bacteria;Bacteria\_none;Pseudomonadota;Gammaproteobacteria;Enterobacterales;Enterobacteriaceae;Candidatus Blochmanniella |
| Candidatus Profftia | 0 | 0 | 0 | 0 | 0 | 0 | 0 | 0 | 0 | 1 | 0 | 0 | 0 | 0 | Bacteria | Bacteria\_none | Pseudomonadota | Gammaproteobacteria | Enterobacterales | Enterobacteriaceae | Bacteria;Bacteria\_none;Pseudomonadota;Gammaproteobacteria;Enterobacterales;Enterobacteriaceae;Candidatus Profftia |
| Candidatus Purcelliella | 0 | 0 | 0 | 0 | 0 | 0 | 0 | 0 | 0 | 0 | 0 | 0 | 0 | 0 | Bacteria | Bacteria\_none | Pseudomonadota | Gammaproteobacteria | Enterobacterales | Enterobacteriaceae | Bacteria;Bacteria\_none;Pseudomonadota;Gammaproteobacteria;Enterobacterales;Enterobacteriaceae;Candidatus Purcelliella |
| Enterobacter | 0 | 0 | 0 | 0 | 0 | 0 | 0 | 0 | 0 | 1 | 0 | 0 | 0 | 0 | Bacteria | Bacteria\_none | Pseudomonadota | Gammaproteobacteria | Enterobacterales | Enterobacteriaceae | Bacteria;Bacteria\_none;Pseudomonadota;Gammaproteobacteria;Enterobacterales;Enterobacteriaceae;Enterobacter |
| Enterobacteriaceae\_Incertae\_sedis | 1 | 0 | 0 | 0 | 0 | 0 | 0 | 0 | 0 | 0 | 0 | 0 | 0 | 0 | Bacteria | Bacteria\_none | Pseudomonadota | Gammaproteobacteria | Enterobacterales | Enterobacteriaceae | Bacteria;Bacteria\_none;Pseudomonadota;Gammaproteobacteria;Enterobacterales;Enterobacteriaceae;Enterobacteriaceae\_Incertae\_sedis |
| Escherichia | 1 | 0 | 0 | 0 | 1 | 0 | 0 | 0 | 0 | 0 | 0 | 0 | 0 | 0 | Bacteria | Bacteria\_none | Pseudomonadota | Gammaproteobacteria | Enterobacterales | Enterobacteriaceae | Bacteria;Bacteria\_none;Pseudomonadota;Gammaproteobacteria;Enterobacterales;Enterobacteriaceae;Escherichia |
| Klebsiella | 0 | 0 | 0 | 2 | 2 | 0 | 0 | 0 | 0 | 0 | 0 | 0 | 0 | 0 | Bacteria | Bacteria\_none | Pseudomonadota | Gammaproteobacteria | Enterobacterales | Enterobacteriaceae | Bacteria;Bacteria\_none;Pseudomonadota;Gammaproteobacteria;Enterobacterales;Enterobacteriaceae;Klebsiella |
| Salmonella | 2 | 0 | 0 | 0 | 0 | 0 | 0 | 2 | 0 | 0 | 0 | 0 | 0 | 1 | Bacteria | Bacteria\_none | Pseudomonadota | Gammaproteobacteria | Enterobacterales | Enterobacteriaceae | Bacteria;Bacteria\_none;Pseudomonadota;Gammaproteobacteria;Enterobacterales;Enterobacteriaceae;Salmonella |
| Symbiopectobacterium | 0 | 0 | 0 | 0 | 0 | 0 | 0 | 0 | 0 | 0 | 0 | 0 | 0 | 0 | Bacteria | Bacteria\_none | Pseudomonadota | Gammaproteobacteria | Enterobacterales | Enterobacteriaceae | Bacteria;Bacteria\_none;Pseudomonadota;Gammaproteobacteria;Enterobacterales;Enterobacteriaceae;Symbiopectobacterium |
| Buchnera | 0 | 0 | 0 | 0 | 0 | 0 | 0 | 1 | 0 | 0 | 0 | 0 | 0 | 0 | Bacteria | Bacteria\_none | Pseudomonadota | Gammaproteobacteria | Enterobacterales | Erwiniaceae | Bacteria;Bacteria\_none;Pseudomonadota;Gammaproteobacteria;Enterobacterales;Erwiniaceae;Buchnera |
| Erwinia | 0 | 0 | 0 | 0 | 1 | 0 | 0 | 0 | 0 | 0 | 0 | 1 | 0 | 0 | Bacteria | Bacteria\_none | Pseudomonadota | Gammaproteobacteria | Enterobacterales | Erwiniaceae | Bacteria;Bacteria\_none;Pseudomonadota;Gammaproteobacteria;Enterobacterales;Erwiniaceae;Erwinia |
| Pantoea | 0 | 0 | 0 | 0 | 0 | 0 | 0 | 0 | 0 | 0 | 0 | 0 | 0 | 0 | Bacteria | Bacteria\_none | Pseudomonadota | Gammaproteobacteria | Enterobacterales | Erwiniaceae | Bacteria;Bacteria\_none;Pseudomonadota;Gammaproteobacteria;Enterobacterales;Erwiniaceae;Pantoea |
| Tatumella | 0 | 0 | 0 | 0 | 0 | 0 | 0 | 1 | 0 | 0 | 0 | 0 | 0 | 0 | Bacteria | Bacteria\_none | Pseudomonadota | Gammaproteobacteria | Enterobacterales | Erwiniaceae | Bacteria;Bacteria\_none;Pseudomonadota;Gammaproteobacteria;Enterobacterales;Erwiniaceae;Tatumella |
| Arsenophonus | 0 | 0 | 0 | 0 | 0 | 0 | 0 | 0 | 0 | 0 | 0 | 0 | 0 | 0 | Bacteria | Bacteria\_none | Pseudomonadota | Gammaproteobacteria | Enterobacterales | Morganellaceae | Bacteria;Bacteria\_none;Pseudomonadota;Gammaproteobacteria;Enterobacterales;Morganellaceae;Arsenophonus |
| Morganella | 0 | 0 | 0 | 0 | 0 | 0 | 0 | 0 | 0 | 0 | 0 | 0 | 0 | 0 | Bacteria | Bacteria\_none | Pseudomonadota | Gammaproteobacteria | Enterobacterales | Morganellaceae | Bacteria;Bacteria\_none;Pseudomonadota;Gammaproteobacteria;Enterobacterales;Morganellaceae;Morganella |
| Proteus | 1 | 0 | 1 | 0 | 0 | 1 | 0 | 1 | 0 | 0 | 0 | 1 | 0 | 0 | Bacteria | Bacteria\_none | Pseudomonadota | Gammaproteobacteria | Enterobacterales | Morganellaceae | Bacteria;Bacteria\_none;Pseudomonadota;Gammaproteobacteria;Enterobacterales;Morganellaceae;Proteus |
| Providencia | 0 | 0 | 0 | 0 | 1 | 1 | 0 | 0 | 0 | 0 | 0 | 0 | 0 | 0 | Bacteria | Bacteria\_none | Pseudomonadota | Gammaproteobacteria | Enterobacterales | Morganellaceae | Bacteria;Bacteria\_none;Pseudomonadota;Gammaproteobacteria;Enterobacterales;Morganellaceae;Providencia |
| Xenorhabdus | 0 | 0 | 0 | 0 | 0 | 0 | 1 | 0 | 0 | 0 | 0 | 0 | 0 | 0 | Bacteria | Bacteria\_none | Pseudomonadota | Gammaproteobacteria | Enterobacterales | Morganellaceae | Bacteria;Bacteria\_none;Pseudomonadota;Gammaproteobacteria;Enterobacterales;Morganellaceae;Xenorhabdus |
| Dickeya | 0 | 0 | 0 | 0 | 0 | 0 | 0 | 0 | 0 | 0 | 0 | 0 | 0 | 0 | Bacteria | Bacteria\_none | Pseudomonadota | Gammaproteobacteria | Enterobacterales | Pectobacteriaceae | Bacteria;Bacteria\_none;Pseudomonadota;Gammaproteobacteria;Enterobacterales;Pectobacteriaceae;Dickeya |
| Serratia | 0 | 0 | 0 | 0 | 0 | 0 | 0 | 0 | 0 | 0 | 0 | 0 | 0 | 0 | Bacteria | Bacteria\_none | Pseudomonadota | Gammaproteobacteria | Enterobacterales | Yersiniaceae | Bacteria;Bacteria\_none;Pseudomonadota;Gammaproteobacteria;Enterobacterales;Yersiniaceae;Serratia |
| Legionella | 0 | 0 | 0 | 1 | 0 | 0 | 0 | 0 | 0 | 0 | 0 | 0 | 1 | 1 | Bacteria | Bacteria\_none | Pseudomonadota | Gammaproteobacteria | Legionellales | Legionellaceae | Bacteria;Bacteria\_none;Pseudomonadota;Gammaproteobacteria;Legionellales;Legionellaceae;Legionella |
| Lysobacter | 0 | 0 | 0 | 1 | 0 | 0 | 0 | 0 | 0 | 0 | 0 | 0 | 0 | 0 | Bacteria | Bacteria\_none | Pseudomonadota | Gammaproteobacteria | Lysobacterales | Lysobacteraceae | Bacteria;Bacteria\_none;Pseudomonadota;Gammaproteobacteria;Lysobacterales;Lysobacteraceae;Lysobacter |
| Pseudoxanthomonas | 0 | 0 | 0 | 0 | 0 | 0 | 0 | 0 | 0 | 0 | 1 | 0 | 0 | 0 | Bacteria | Bacteria\_none | Pseudomonadota | Gammaproteobacteria | Lysobacterales | Lysobacteraceae | Bacteria;Bacteria\_none;Pseudomonadota;Gammaproteobacteria;Lysobacterales;Lysobacteraceae;Pseudoxanthomonas |
| Stenotrophomonas | 0 | 0 | 0 | 0 | 2 | 0 | 0 | 0 | 0 | 0 | 0 | 0 | 0 | 0 | Bacteria | Bacteria\_none | Pseudomonadota | Gammaproteobacteria | Lysobacterales | Lysobacteraceae | Bacteria;Bacteria\_none;Pseudomonadota;Gammaproteobacteria;Lysobacterales;Lysobacteraceae;Stenotrophomonas |
| Xanthomonas | 0 | 0 | 0 | 0 | 0 | 0 | 0 | 0 | 0 | 0 | 0 | 0 | 0 | 0 | Bacteria | Bacteria\_none | Pseudomonadota | Gammaproteobacteria | Lysobacterales | Lysobacteraceae | Bacteria;Bacteria\_none;Pseudomonadota;Gammaproteobacteria;Lysobacterales;Lysobacteraceae;Xanthomonas |
| Xylella | 0 | 0 | 0 | 0 | 0 | 0 | 0 | 0 | 0 | 0 | 0 | 0 | 0 | 0 | Bacteria | Bacteria\_none | Pseudomonadota | Gammaproteobacteria | Lysobacterales | Lysobacteraceae | Bacteria;Bacteria\_none;Pseudomonadota;Gammaproteobacteria;Lysobacterales;Lysobacteraceae;Xylella |
| Dyella | 0 | 0 | 0 | 0 | 0 | 0 | 0 | 0 | 0 | 0 | 0 | 1 | 0 | 0 | Bacteria | Bacteria\_none | Pseudomonadota | Gammaproteobacteria | Lysobacterales | Rhodanobacteraceae | Bacteria;Bacteria\_none;Pseudomonadota;Gammaproteobacteria;Lysobacterales;Rhodanobacteraceae;Dyella |
| Tahibacter | 0 | 0 | 0 | 0 | 0 | 0 | 0 | 0 | 0 | 0 | 0 | 0 | 0 | 0 | Bacteria | Bacteria\_none | Pseudomonadota | Gammaproteobacteria | Lysobacterales | Rhodanobacteraceae | Bacteria;Bacteria\_none;Pseudomonadota;Gammaproteobacteria;Lysobacterales;Rhodanobacteraceae;Tahibacter |
| Candidatus Methylospira | 0 | 0 | 0 | 0 | 0 | 0 | 0 | 0 | 0 | 0 | 0 | 0 | 0 | 0 | Bacteria | Bacteria\_none | Pseudomonadota | Gammaproteobacteria | Methylococcales | Methylococcaceae | Bacteria;Bacteria\_none;Pseudomonadota;Gammaproteobacteria;Methylococcales;Methylococcaceae;Candidatus Methylospira |
| Methylobacter | 0 | 0 | 0 | 0 | 0 | 0 | 0 | 0 | 0 | 0 | 0 | 0 | 0 | 0 | Bacteria | Bacteria\_none | Pseudomonadota | Gammaproteobacteria | Methylococcales | Methylococcaceae | Bacteria;Bacteria\_none;Pseudomonadota;Gammaproteobacteria;Methylococcales;Methylococcaceae;Methylobacter |
| Methylomonas | 0 | 0 | 0 | 0 | 0 | 0 | 0 | 0 | 0 | 0 | 0 | 0 | 0 | 0 | Bacteria | Bacteria\_none | Pseudomonadota | Gammaproteobacteria | Methylococcales | Methylococcaceae | Bacteria;Bacteria\_none;Pseudomonadota;Gammaproteobacteria;Methylococcales;Methylococcaceae;Methylomonas |
| Methylotuvimicrobium | 0 | 0 | 0 | 0 | 0 | 0 | 0 | 0 | 0 | 0 | 0 | 0 | 0 | 0 | Bacteria | Bacteria\_none | Pseudomonadota | Gammaproteobacteria | Methylococcales | Methylococcaceae | Bacteria;Bacteria\_none;Pseudomonadota;Gammaproteobacteria;Methylococcales;Methylococcaceae;Methylotuvimicrobium |
| Acinetobacter | 0 | 0 | 0 | 0 | 0 | 2 | 0 | 1 | 0 | 1 | 0 | 0 | 0 | 1 | Bacteria | Bacteria\_none | Pseudomonadota | Gammaproteobacteria | Moraxellales | Moraxellaceae | Bacteria;Bacteria\_none;Pseudomonadota;Gammaproteobacteria;Moraxellales;Moraxellaceae;Acinetobacter |
| Moraxella | 0 | 0 | 0 | 0 | 0 | 0 | 0 | 0 | 0 | 0 | 0 | 0 | 0 | 0 | Bacteria | Bacteria\_none | Pseudomonadota | Gammaproteobacteria | Moraxellales | Moraxellaceae | Bacteria;Bacteria\_none;Pseudomonadota;Gammaproteobacteria;Moraxellales;Moraxellaceae;Moraxella |
| Psychrobacter | 0 | 0 | 1 | 0 | 0 | 0 | 0 | 1 | 0 | 0 | 0 | 0 | 0 | 0 | Bacteria | Bacteria\_none | Pseudomonadota | Gammaproteobacteria | Moraxellales | Moraxellaceae | Bacteria;Bacteria\_none;Pseudomonadota;Gammaproteobacteria;Moraxellales;Moraxellaceae;Psychrobacter |
| Alcanivorax | 0 | 0 | 0 | 0 | 0 | 0 | 0 | 0 | 0 | 0 | 0 | 0 | 0 | 0 | Bacteria | Bacteria\_none | Pseudomonadota | Gammaproteobacteria | Oceanospirillales | Alcanivoracaceae | Bacteria;Bacteria\_none;Pseudomonadota;Gammaproteobacteria;Oceanospirillales;Alcanivoracaceae;Alcanivorax |
| Halomonas | 0 | 0 | 0 | 0 | 1 | 0 | 0 | 0 | 0 | 0 | 0 | 0 | 1 | 0 | Bacteria | Bacteria\_none | Pseudomonadota | Gammaproteobacteria | Oceanospirillales | Halomonadaceae | Bacteria;Bacteria\_none;Pseudomonadota;Gammaproteobacteria;Oceanospirillales;Halomonadaceae;Halomonas |
| Amphritea | 0 | 0 | 0 | 0 | 0 | 0 | 0 | 0 | 0 | 0 | 0 | 0 | 0 | 0 | Bacteria | Bacteria\_none | Pseudomonadota | Gammaproteobacteria | Oceanospirillales | Oceanospirillaceae | Bacteria;Bacteria\_none;Pseudomonadota;Gammaproteobacteria;Oceanospirillales;Oceanospirillaceae;Amphritea |
| Marinomonas | 0 | 0 | 0 | 0 | 0 | 0 | 0 | 0 | 0 | 0 | 0 | 0 | 0 | 0 | Bacteria | Bacteria\_none | Pseudomonadota | Gammaproteobacteria | Oceanospirillales | Oceanospirillaceae | Bacteria;Bacteria\_none;Pseudomonadota;Gammaproteobacteria;Oceanospirillales;Oceanospirillaceae;Marinomonas |
| Thalassolituus | 0 | 1 | 0 | 0 | 0 | 0 | 0 | 0 | 0 | 0 | 0 | 0 | 0 | 0 | Bacteria | Bacteria\_none | Pseudomonadota | Gammaproteobacteria | Oceanospirillales | Oceanospirillaceae | Bacteria;Bacteria\_none;Pseudomonadota;Gammaproteobacteria;Oceanospirillales;Oceanospirillaceae;Thalassolituus |
| Gilliamella | 0 | 0 | 0 | 0 | 0 | 0 | 0 | 0 | 0 | 0 | 0 | 0 | 0 | 0 | Bacteria | Bacteria\_none | Pseudomonadota | Gammaproteobacteria | Orbales | Orbaceae | Bacteria;Bacteria\_none;Pseudomonadota;Gammaproteobacteria;Orbales;Orbaceae;Gilliamella |
| Actinobacillus | 0 | 0 | 1 | 0 | 0 | 0 | 0 | 11 | 0 | 0 | 0 | 0 | 1 | 0 | Bacteria | Bacteria\_none | Pseudomonadota | Gammaproteobacteria | Pasteurellales | Pasteurellaceae | Bacteria;Bacteria\_none;Pseudomonadota;Gammaproteobacteria;Pasteurellales;Pasteurellaceae;Actinobacillus |
| Aggregatibacter | 0 | 35 | 9 | 103 | 61 | 35 | 0 | 81 | 0 | 3 | 0 | 11 | 25 | 8 | Bacteria | Bacteria\_none | Pseudomonadota | Gammaproteobacteria | Pasteurellales | Pasteurellaceae | Bacteria;Bacteria\_none;Pseudomonadota;Gammaproteobacteria;Pasteurellales;Pasteurellaceae;Aggregatibacter |
| Avibacterium | 0 | 0 | 0 | 0 | 0 | 1 | 0 | 1 | 0 | 0 | 0 | 0 | 0 | 0 | Bacteria | Bacteria\_none | Pseudomonadota | Gammaproteobacteria | Pasteurellales | Pasteurellaceae | Bacteria;Bacteria\_none;Pseudomonadota;Gammaproteobacteria;Pasteurellales;Pasteurellaceae;Avibacterium |
| Bibersteinia | 0 | 0 | 0 | 0 | 0 | 0 | 0 | 1 | 0 | 0 | 0 | 0 | 0 | 0 | Bacteria | Bacteria\_none | Pseudomonadota | Gammaproteobacteria | Pasteurellales | Pasteurellaceae | Bacteria;Bacteria\_none;Pseudomonadota;Gammaproteobacteria;Pasteurellales;Pasteurellaceae;Bibersteinia |
| Bisgaardia | 0 | 0 | 0 | 0 | 0 | 0 | 0 | 0 | 0 | 0 | 0 | 0 | 0 | 0 | Bacteria | Bacteria\_none | Pseudomonadota | Gammaproteobacteria | Pasteurellales | Pasteurellaceae | Bacteria;Bacteria\_none;Pseudomonadota;Gammaproteobacteria;Pasteurellales;Pasteurellaceae;Bisgaardia |
| Frederiksenia | 0 | 0 | 0 | 0 | 0 | 0 | 0 | 0 | 0 | 0 | 0 | 0 | 1 | 0 | Bacteria | Bacteria\_none | Pseudomonadota | Gammaproteobacteria | Pasteurellales | Pasteurellaceae | Bacteria;Bacteria\_none;Pseudomonadota;Gammaproteobacteria;Pasteurellales;Pasteurellaceae;Frederiksenia |
| Gallibacterium | 0 | 0 | 0 | 0 | 0 | 2 | 0 | 0 | 0 | 0 | 0 | 0 | 0 | 0 | Bacteria | Bacteria\_none | Pseudomonadota | Gammaproteobacteria | Pasteurellales | Pasteurellaceae | Bacteria;Bacteria\_none;Pseudomonadota;Gammaproteobacteria;Pasteurellales;Pasteurellaceae;Gallibacterium |
| Glaesserella | 0 | 1 | 0 | 0 | 0 | 0 | 1 | 0 | 0 | 0 | 0 | 0 | 0 | 0 | Bacteria | Bacteria\_none | Pseudomonadota | Gammaproteobacteria | Pasteurellales | Pasteurellaceae | Bacteria;Bacteria\_none;Pseudomonadota;Gammaproteobacteria;Pasteurellales;Pasteurellaceae;Glaesserella |
| Haemophilus | 64 | 103 | 412 | 311 | 101 | 139 | 0 | 371 | 0 | 46 | 23 | 84 | 114 | 75 | Bacteria | Bacteria\_none | Pseudomonadota | Gammaproteobacteria | Pasteurellales | Pasteurellaceae | Bacteria;Bacteria\_none;Pseudomonadota;Gammaproteobacteria;Pasteurellales;Pasteurellaceae;Haemophilus |
| Histophilus | 0 | 0 | 0 | 0 | 0 | 0 | 0 | 1 | 0 | 0 | 0 | 0 | 0 | 0 | Bacteria | Bacteria\_none | Pseudomonadota | Gammaproteobacteria | Pasteurellales | Pasteurellaceae | Bacteria;Bacteria\_none;Pseudomonadota;Gammaproteobacteria;Pasteurellales;Pasteurellaceae;Histophilus |
| Mannheimia | 0 | 0 | 0 | 0 | 1 | 0 | 0 | 5 | 0 | 0 | 0 | 0 | 1 | 0 | Bacteria | Bacteria\_none | Pseudomonadota | Gammaproteobacteria | Pasteurellales | Pasteurellaceae | Bacteria;Bacteria\_none;Pseudomonadota;Gammaproteobacteria;Pasteurellales;Pasteurellaceae;Mannheimia |
| Mergibacter | 0 | 0 | 0 | 0 | 0 | 0 | 0 | 0 | 0 | 0 | 0 | 0 | 0 | 0 | Bacteria | Bacteria\_none | Pseudomonadota | Gammaproteobacteria | Pasteurellales | Pasteurellaceae | Bacteria;Bacteria\_none;Pseudomonadota;Gammaproteobacteria;Pasteurellales;Pasteurellaceae;Mergibacter |
| Otariodibacter | 0 | 0 | 0 | 0 | 0 | 0 | 0 | 0 | 0 | 0 | 0 | 0 | 0 | 0 | Bacteria | Bacteria\_none | Pseudomonadota | Gammaproteobacteria | Pasteurellales | Pasteurellaceae | Bacteria;Bacteria\_none;Pseudomonadota;Gammaproteobacteria;Pasteurellales;Pasteurellaceae;Otariodibacter |
| Pasteurella | 0 | 0 | 1 | 0 | 0 | 1 | 0 | 5 | 0 | 0 | 0 | 0 | 0 | 0 | Bacteria | Bacteria\_none | Pseudomonadota | Gammaproteobacteria | Pasteurellales | Pasteurellaceae | Bacteria;Bacteria\_none;Pseudomonadota;Gammaproteobacteria;Pasteurellales;Pasteurellaceae;Pasteurella |
| Rodentibacter | 0 | 0 | 0 | 0 | 0 | 0 | 0 | 1 | 0 | 0 | 0 | 0 | 0 | 0 | Bacteria | Bacteria\_none | Pseudomonadota | Gammaproteobacteria | Pasteurellales | Pasteurellaceae | Bacteria;Bacteria\_none;Pseudomonadota;Gammaproteobacteria;Pasteurellales;Pasteurellaceae;Rodentibacter |
| Vespertiliibacter | 0 | 0 | 0 | 0 | 0 | 0 | 0 | 0 | 0 | 0 | 0 | 0 | 0 | 0 | Bacteria | Bacteria\_none | Pseudomonadota | Gammaproteobacteria | Pasteurellales | Pasteurellaceae | Bacteria;Bacteria\_none;Pseudomonadota;Gammaproteobacteria;Pasteurellales;Pasteurellaceae;Vespertiliibacter |
| Pseudomonas | 0 | 3 | 0 | 0 | 1 | 1 | 0 | 0 | 0 | 0 | 1 | 0 | 0 | 0 | Bacteria | Bacteria\_none | Pseudomonadota | Gammaproteobacteria | Pseudomonadales | Pseudomonadaceae | Bacteria;Bacteria\_none;Pseudomonadota;Gammaproteobacteria;Pseudomonadales;Pseudomonadaceae;Pseudomonas |
| Thiomicrorhabdus | 0 | 0 | 0 | 0 | 1 | 0 | 0 | 0 | 0 | 0 | 0 | 0 | 0 | 0 | Bacteria | Bacteria\_none | Pseudomonadota | Gammaproteobacteria | Thiotrichales | Piscirickettsiaceae | Bacteria;Bacteria\_none;Pseudomonadota;Gammaproteobacteria;Thiotrichales;Piscirickettsiaceae;Thiomicrorhabdus |
| Aliivibrio | 0 | 0 | 0 | 0 | 0 | 0 | 0 | 0 | 0 | 0 | 0 | 0 | 0 | 0 | Bacteria | Bacteria\_none | Pseudomonadota | Gammaproteobacteria | Vibrionales | Vibrionaceae | Bacteria;Bacteria\_none;Pseudomonadota;Gammaproteobacteria;Vibrionales;Vibrionaceae;Aliivibrio |
| Enterovibrio | 0 | 0 | 0 | 0 | 0 | 0 | 0 | 0 | 0 | 0 | 0 | 0 | 0 | 0 | Bacteria | Bacteria\_none | Pseudomonadota | Gammaproteobacteria | Vibrionales | Vibrionaceae | Bacteria;Bacteria\_none;Pseudomonadota;Gammaproteobacteria;Vibrionales;Vibrionaceae;Enterovibrio |
| Thaumasiovibrio | 0 | 0 | 0 | 0 | 0 | 0 | 0 | 0 | 0 | 0 | 0 | 0 | 1 | 0 | Bacteria | Bacteria\_none | Pseudomonadota | Gammaproteobacteria | Vibrionales | Vibrionaceae | Bacteria;Bacteria\_none;Pseudomonadota;Gammaproteobacteria;Vibrionales;Vibrionaceae;Thaumasiovibrio |
| Vibrio | 0 | 0 | 3 | 0 | 2 | 1 | 0 | 0 | 0 | 0 | 0 | 0 | 0 | 0 | Bacteria | Bacteria\_none | Pseudomonadota | Gammaproteobacteria | Vibrionales | Vibrionaceae | Bacteria;Bacteria\_none;Pseudomonadota;Gammaproteobacteria;Vibrionales;Vibrionaceae;Vibrio |
| Leptospira | 1 | 0 | 0 | 0 | 0 | 0 | 0 | 0 | 0 | 0 | 0 | 1 | 1 | 0 | Bacteria | Bacteria\_none | Spirochaetota | Spirochaetia | Leptospirales | Leptospiraceae | Bacteria;Bacteria\_none;Spirochaetota;Spirochaetia;Leptospirales;Leptospiraceae;Leptospira |
| Borreliella | 0 | 0 | 0 | 0 | 0 | 1 | 0 | 0 | 0 | 0 | 0 | 0 | 0 | 0 | Bacteria | Bacteria\_none | Spirochaetota | Spirochaetia | Spirochaetales | Borreliaceae | Bacteria;Bacteria\_none;Spirochaetota;Spirochaetia;Spirochaetales;Borreliaceae;Borreliella |
| Breznakiella | 0 | 1 | 0 | 0 | 0 | 0 | 0 | 0 | 0 | 0 | 0 | 0 | 0 | 0 | Bacteria | Bacteria\_none | Spirochaetota | Spirochaetia | Spirochaetales | Breznakiellaceae | Bacteria;Bacteria\_none;Spirochaetota;Spirochaetia;Spirochaetales;Breznakiellaceae;Breznakiella |
| Sphaerochaeta | 0 | 1 | 0 | 0 | 0 | 0 | 0 | 0 | 0 | 0 | 0 | 0 | 0 | 0 | Bacteria | Bacteria\_none | Spirochaetota | Spirochaetia | Spirochaetales | Sphaerochaetaceae | Bacteria;Bacteria\_none;Spirochaetota;Spirochaetia;Spirochaetales;Sphaerochaetaceae;Sphaerochaeta |
| Treponema | 2 | 71 | 52 | 6 | 27 | 56 | 2 | 30 | 0 | 9 | 23 | 37 | 26 | 9 | Bacteria | Bacteria\_none | Spirochaetota | Spirochaetia | Spirochaetales | Treponemataceae | Bacteria;Bacteria\_none;Spirochaetota;Spirochaetia;Spirochaetales;Treponemataceae;Treponema |
| Fretibacterium | 0 | 0 | 1 | 0 | 0 | 0 | 0 | 0 | 0 | 0 | 0 | 0 | 0 | 0 | Bacteria | Bacteria\_none | Synergistota | Synergistia | Synergistales | Aminobacteriaceae | Bacteria;Bacteria\_none;Synergistota;Synergistia;Synergistales;Aminobacteriaceae;Fretibacterium |
| Candidatus Desulfofervidus | 0 | 0 | 0 | 0 | 0 | 1 | 0 | 0 | 0 | 0 | 0 | 0 | 0 | 0 | Bacteria | Bacteria\_none | Thermodesulfobacteriota | Candidatus Desulfofervidia | Candidatus Desulfofervidales | Candidatus Desulfofervidaceae | Bacteria;Bacteria\_none;Thermodesulfobacteriota;Candidatus Desulfofervidia;Candidatus Desulfofervidales;Candidatus Desulfofervidaceae;Candidatus Desulfofervidus |
| Desulfoferula | 0 | 0 | 0 | 0 | 0 | 1 | 0 | 0 | 0 | 0 | 0 | 0 | 0 | 0 | Bacteria | Bacteria\_none | Thermodesulfobacteriota | Desulfarculia | Desulfarculales | Desulfarculaceae | Bacteria;Bacteria\_none;Thermodesulfobacteriota;Desulfarculia;Desulfarculales;Desulfarculaceae;Desulfoferula |
| Desulfobulbus | 0 | 2 | 1 | 0 | 0 | 0 | 0 | 1 | 0 | 0 | 0 | 0 | 0 | 0 | Bacteria | Bacteria\_none | Thermodesulfobacteriota | Desulfobulbia | Desulfobulbales | Desulfobulbaceae | Bacteria;Bacteria\_none;Thermodesulfobacteriota;Desulfobulbia;Desulfobulbales;Desulfobulbaceae;Desulfobulbus |
| Lawsonia | 0 | 0 | 0 | 0 | 0 | 0 | 0 | 0 | 0 | 0 | 0 | 1 | 0 | 0 | Bacteria | Bacteria\_none | Thermodesulfobacteriota | Desulfovibrionia | Desulfovibrionales | Desulfovibrionaceae | Bacteria;Bacteria\_none;Thermodesulfobacteriota;Desulfovibrionia;Desulfovibrionales;Desulfovibrionaceae;Lawsonia |
| Syntrophotalea | 0 | 0 | 0 | 0 | 0 | 0 | 0 | 0 | 0 | 0 | 0 | 0 | 0 | 0 | Bacteria | Bacteria\_none | Thermodesulfobacteriota | Desulfuromonadia | Desulfuromonadales | Syntrophotaleaceae | Bacteria;Bacteria\_none;Thermodesulfobacteriota;Desulfuromonadia;Desulfuromonadales;Syntrophotaleaceae;Syntrophotalea |
| Geobacter | 0 | 0 | 1 | 0 | 0 | 0 | 0 | 0 | 0 | 0 | 0 | 0 | 0 | 0 | Bacteria | Bacteria\_none | Thermodesulfobacteriota | Desulfuromonadia | Geobacterales | Geobacteraceae | Bacteria;Bacteria\_none;Thermodesulfobacteriota;Desulfuromonadia;Geobacterales;Geobacteraceae;Geobacter |
| Thermomicrobium | 0 | 1 | 0 | 0 | 0 | 0 | 0 | 0 | 0 | 0 | 0 | 0 | 0 | 0 | Bacteria | Bacteria\_none | Thermomicrobiota | Thermomicrobia | Thermomicrobiales | Thermomicrobiaceae | Bacteria;Bacteria\_none;Thermomicrobiota;Thermomicrobia;Thermomicrobiales;Thermomicrobiaceae;Thermomicrobium |
| Thermosipho | 1 | 0 | 0 | 0 | 0 | 0 | 0 | 0 | 0 | 0 | 0 | 0 | 0 | 0 | Bacteria | Bacteria\_none | Thermotogota | Thermotogae | Thermotogales | Fervidobacteriaceae | Bacteria;Bacteria\_none;Thermotogota;Thermotogae;Thermotogales;Fervidobacteriaceae;Thermosipho |
| Lacunisphaera | 0 | 1 | 0 | 0 | 0 | 0 | 0 | 0 | 0 | 0 | 0 | 0 | 0 | 0 | Bacteria | Bacteria\_none | Verrucomicrobiota | Opitutia | Opitutales | Opitutaceae | Bacteria;Bacteria\_none;Verrucomicrobiota;Opitutia;Opitutales;Opitutaceae;Lacunisphaera |
| Homo | 380 | 891 | 967 | 47 | 1716 | 157 | 4011 | 4287 | 5101 | 2628 | 2463 | 4032 | 2761 | 2634 | Eukaryota | Metazoa | Chordata | Mammalia | Primates | Hominidae | Eukaryota;Metazoa;Chordata;Mammalia;Primates;Hominidae;Homo |
| Unknown | 1242 | 1010 | 1190 | 1432 | 1131 | 1906 | 349 | 335 | 197 | 765 | 646 | 557 | 1176 | 1956 | Unclassified | Unknown | Unknown | Unknown | Unknown | Unknown | Unclassified;Unknown;Unknown;Unknown;Unknown;Unknown;Unknown |
| Mimivirus | 0 | 0 | 0 | 0 | 0 | 0 | 0 | 0 | 0 | 0 | 0 | 1 | 0 | 0 | Viruses | Bamfordvirae | Nucleocytoviricota | Megaviricetes | Imitervirales | Mimiviridae | Viruses;Bamfordvirae;Nucleocytoviricota;Megaviricetes;Imitervirales;Mimiviridae;Mimivirus |
| Theiavirus | 0 | 0 | 0 | 0 | 0 | 0 | 0 | 1 | 0 | 0 | 0 | 0 | 0 | 0 | Viruses | Bamfordvirae | Nucleocytoviricota | Megaviricetes | Imitervirales | Mimiviridae | Viruses;Bamfordvirae;Nucleocytoviricota;Megaviricetes;Imitervirales;Mimiviridae;Theiavirus |
| Biavirus | 0 | 0 | 0 | 0 | 0 | 0 | 0 | 0 | 0 | 0 | 0 | 0 | 0 | 0 | Viruses | Bamfordvirae | Nucleocytoviricota | Megaviricetes | Imitervirales | Schizomimiviridae | Viruses;Bamfordvirae;Nucleocytoviricota;Megaviricetes;Imitervirales;Schizomimiviridae;Biavirus |
| Lymphocryptovirus | 8 | 0 | 0 | 0 | 0 | 0 | 0 | 0 | 0 | 0 | 0 | 0 | 0 | 1 | Viruses | Heunggongvirae | Peploviricota | Herviviricetes | Herpesvirales | Orthoherpesviridae | Viruses;Heunggongvirae;Peploviricota;Herviviricetes;Herpesvirales;Orthoherpesviridae;Lymphocryptovirus |
| Caudoviricetes\_Incertae\_sedis | 4 | 0 | 0 | 1 | 3 | 1 | 2 | 0 | 0 | 0 | 2 | 1 | 0 | 0 | Viruses | Heunggongvirae | Uroviricota | Caudoviricetes | Caudoviricetes\_Incertae\_sedis | Caudoviricetes\_Incertae\_sedis | Viruses;Heunggongvirae;Uroviricota;Caudoviricetes;Caudoviricetes\_Incertae\_sedis;Caudoviricetes\_Incertae\_sedis;Caudoviricetes\_Incertae\_sedis |
| Whopevirus | 2 | 0 | 0 | 0 | 0 | 0 | 0 | 0 | 0 | 0 | 0 | 0 | 0 | 0 | Viruses | Heunggongvirae | Uroviricota | Caudoviricetes | Crassvirales | Intestiviridae | Viruses;Heunggongvirae;Uroviricota;Caudoviricetes;Crassvirales;Intestiviridae;Whopevirus |
| Cytorhabdovirus | 0 | 0 | 0 | 0 | 0 | 0 | 0 | 0 | 0 | 0 | 0 | 0 | 0 | 0 | Viruses | Orthornavirae | Negarnaviricota | Monjiviricetes | Mononegavirales | Rhabdoviridae | Viruses;Orthornavirae;Negarnaviricota;Monjiviricetes;Mononegavirales;Rhabdoviridae;Cytorhabdovirus |
| Alphapapillomavirus | 0 | 0 | 0 | 0 | 0 | 0 | 0 | 2 | 0 | 0 | 0 | 0 | 0 | 0 | Viruses | Shotokuvirae | Cossaviricota | Papovaviricetes | Zurhausenvirales | Papillomaviridae | Viruses;Shotokuvirae;Cossaviricota;Papovaviricetes;Zurhausenvirales;Papillomaviridae;Alphapapillomavirus |
| Alphalipothrixvirus | 0 | 0 | 0 | 0 | 0 | 0 | 0 | 0 | 0 | 0 | 0 | 0 | 0 | 0 | Viruses | Zilligvirae | Taleaviricota | Tokiviricetes | Ligamenvirales | Lipothrixviridae | Viruses;Zilligvirae;Taleaviricota;Tokiviricetes;Ligamenvirales;Lipothrixviridae;Alphalipothrixvirus |

Export CSV

Rarefied abundance table for the species rank.

All samples have been randomly subsetted to have the same number
of reads.

| species | barcode01 | barcode02 | barcode03 | barcode04 | barcode05 | barcode06 | barcode07 | barcode08 | barcode09 | barcode10 | barcode11 | barcode12 | total | unclassified | superkingdom | kingdom | phylum | class | order | family | genus | tax |
| --- | --- | --- | --- | --- | --- | --- | --- | --- | --- | --- | --- | --- | --- | --- | --- | --- | --- | --- | --- | --- | --- | --- |
| Haladaptatus salinisoli | 0 | 1 | 0 | 0 | 0 | 0 | 0 | 0 | 0 | 0 | 0 | 0 | 0 | 0 | Archaea | Archaea\_none | Euryarchaeota | Halobacteria | Halobacteriales | Haladaptataceae | Haladaptatus | Archaea;Archaea\_none;Euryarchaeota;Halobacteria;Halobacteriales;Haladaptataceae;Haladaptatus;Haladaptatus salinisoli |
| Halococcoides cellulosivorans | 0 | 0 | 0 | 0 | 0 | 0 | 0 | 0 | 0 | 0 | 0 | 0 | 0 | 0 | Archaea | Archaea\_none | Euryarchaeota | Halobacteria | Halobacteriales | Haloarculaceae | Halococcoides | Archaea;Archaea\_none;Euryarchaeota;Halobacteria;Halobacteriales;Haloarculaceae;Halococcoides;Halococcoides cellulosivorans |
| Halospeciosus flavus | 0 | 0 | 0 | 0 | 0 | 0 | 0 | 0 | 0 | 0 | 0 | 0 | 0 | 0 | Archaea | Archaea\_none | Euryarchaeota | Halobacteria | Halobacteriales | Halobacteriaceae | Halospeciosus | Archaea;Archaea\_none;Euryarchaeota;Halobacteria;Halobacteriales;Halobacteriaceae;Halospeciosus;Halospeciosus flavus |
| Halobaculum halobium | 0 | 0 | 0 | 0 | 0 | 0 | 1 | 0 | 0 | 0 | 0 | 0 | 0 | 0 | Archaea | Archaea\_none | Euryarchaeota | Halobacteria | Halobacteriales | Haloferacaceae | Halobaculum | Archaea;Archaea\_none;Euryarchaeota;Halobacteria;Halobacteriales;Haloferacaceae;Halobaculum;Halobaculum halobium |
| Halogeometricum borinquense | 0 | 0 | 0 | 0 | 0 | 1 | 0 | 0 | 0 | 0 | 0 | 0 | 0 | 0 | Archaea | Archaea\_none | Euryarchaeota | Halobacteria | Halobacteriales | Haloferacaceae | Halogeometricum | Archaea;Archaea\_none;Euryarchaeota;Halobacteria;Halobacteriales;Haloferacaceae;Halogeometricum;Halogeometricum borinquense |
| Halorarum salinum | 0 | 0 | 0 | 0 | 0 | 0 | 0 | 0 | 0 | 1 | 0 | 0 | 0 | 0 | Archaea | Archaea\_none | Euryarchaeota | Halobacteria | Halobacteriales | Haloferacaceae | Halorarum | Archaea;Archaea\_none;Euryarchaeota;Halobacteria;Halobacteriales;Haloferacaceae;Halorarum;Halorarum salinum |
| Halorubrum ruber | 0 | 0 | 0 | 0 | 0 | 0 | 0 | 0 | 0 | 0 | 0 | 0 | 0 | 0 | Archaea | Archaea\_none | Euryarchaeota | Halobacteria | Halobacteriales | Haloferacaceae | Halorubrum | Archaea;Archaea\_none;Euryarchaeota;Halobacteria;Halobacteriales;Haloferacaceae;Halorubrum;Halorubrum ruber |
| Methanobacterium\_uncultured | 0 | 0 | 0 | 0 | 0 | 0 | 0 | 0 | 0 | 0 | 0 | 0 | 0 | 0 | Archaea | Archaea\_none | Euryarchaeota | Methanobacteria | Methanobacteriales | Methanobacteriaceae | Methanobacterium | Archaea;Archaea\_none;Euryarchaeota;Methanobacteria;Methanobacteriales;Methanobacteriaceae;Methanobacterium;Methanobacterium\_uncultured |
| Methanobrevibacter arboriphilus | 0 | 0 | 1 | 0 | 0 | 0 | 0 | 0 | 0 | 0 | 0 | 0 | 0 | 0 | Archaea | Archaea\_none | Euryarchaeota | Methanobacteria | Methanobacteriales | Methanobacteriaceae | Methanobrevibacter | Archaea;Archaea\_none;Euryarchaeota;Methanobacteria;Methanobacteriales;Methanobacteriaceae;Methanobrevibacter;Methanobrevibacter arboriphilus |
| Methanobrevibacter smithii | 0 | 0 | 0 | 0 | 0 | 0 | 0 | 0 | 0 | 0 | 0 | 0 | 0 | 0 | Archaea | Archaea\_none | Euryarchaeota | Methanobacteria | Methanobacteriales | Methanobacteriaceae | Methanobrevibacter | Archaea;Archaea\_none;Euryarchaeota;Methanobacteria;Methanobacteriales;Methanobacteriaceae;Methanobrevibacter;Methanobrevibacter smithii |
| Methanobrevibacter sp. YE315 | 0 | 0 | 0 | 0 | 0 | 0 | 0 | 0 | 0 | 0 | 0 | 0 | 0 | 0 | Archaea | Archaea\_none | Euryarchaeota | Methanobacteria | Methanobacteriales | Methanobacteriaceae | Methanobrevibacter | Archaea;Archaea\_none;Euryarchaeota;Methanobacteria;Methanobacteriales;Methanobacteriaceae;Methanobrevibacter;Methanobrevibacter sp. YE315 |
| Methanosphaera stadtmanae | 0 | 0 | 1 | 0 | 0 | 0 | 0 | 0 | 0 | 0 | 0 | 0 | 0 | 0 | Archaea | Archaea\_none | Euryarchaeota | Methanobacteria | Methanobacteriales | Methanobacteriaceae | Methanosphaera | Archaea;Archaea\_none;Euryarchaeota;Methanobacteria;Methanobacteriales;Methanobacteriaceae;Methanosphaera;Methanosphaera stadtmanae |
| Methanocaldococcus sp. FS406-22 | 0 | 0 | 0 | 0 | 0 | 0 | 0 | 0 | 0 | 0 | 0 | 0 | 0 | 0 | Archaea | Archaea\_none | Euryarchaeota | Methanococci | Methanococcales | Methanocaldococcaceae | Methanocaldococcus | Archaea;Archaea\_none;Euryarchaeota;Methanococci;Methanococcales;Methanocaldococcaceae;Methanocaldococcus;Methanocaldococcus sp. FS406-22 |
| Methanocaldococcus vulcanius | 0 | 0 | 1 | 0 | 0 | 0 | 0 | 0 | 0 | 0 | 0 | 0 | 0 | 0 | Archaea | Archaea\_none | Euryarchaeota | Methanococci | Methanococcales | Methanocaldococcaceae | Methanocaldococcus | Archaea;Archaea\_none;Euryarchaeota;Methanococci;Methanococcales;Methanocaldococcaceae;Methanocaldococcus;Methanocaldococcus vulcanius |
| Methanoregula\_uncultured | 0 | 1 | 0 | 0 | 0 | 0 | 0 | 0 | 0 | 0 | 0 | 0 | 0 | 0 | Archaea | Archaea\_none | Euryarchaeota | Methanomicrobia | Methanomicrobiales | Methanoregulaceae | Methanoregula | Archaea;Archaea\_none;Euryarchaeota;Methanomicrobia;Methanomicrobiales;Methanoregulaceae;Methanoregula;Methanoregula\_uncultured |
| Methanimicrococcus hongohii | 0 | 0 | 0 | 0 | 0 | 0 | 0 | 0 | 0 | 0 | 0 | 0 | 0 | 0 | Archaea | Archaea\_none | Euryarchaeota | Methanomicrobia | Methanosarcinales | Methanosarcinaceae | Methanimicrococcus | Archaea;Archaea\_none;Euryarchaeota;Methanomicrobia;Methanosarcinales;Methanosarcinaceae;Methanimicrococcus;Methanimicrococcus hongohii |
| Methanolapillus millepedarum | 0 | 0 | 0 | 0 | 0 | 1 | 0 | 0 | 0 | 0 | 0 | 0 | 0 | 0 | Archaea | Archaea\_none | Euryarchaeota | Methanomicrobia | Methanosarcinales | Methanosarcinaceae | Methanolapillus | Archaea;Archaea\_none;Euryarchaeota;Methanomicrobia;Methanosarcinales;Methanosarcinaceae;Methanolapillus;Methanolapillus millepedarum |
| Methanolobus\_uncultured | 0 | 0 | 0 | 0 | 1 | 1 | 0 | 0 | 0 | 0 | 0 | 0 | 0 | 0 | Archaea | Archaea\_none | Euryarchaeota | Methanomicrobia | Methanosarcinales | Methanosarcinaceae | Methanolobus | Archaea;Archaea\_none;Euryarchaeota;Methanomicrobia;Methanosarcinales;Methanosarcinaceae;Methanolobus;Methanolobus\_uncultured |
| Methanomethylovorans\_uncultured | 0 | 0 | 0 | 0 | 0 | 0 | 0 | 0 | 0 | 0 | 0 | 0 | 0 | 0 | Archaea | Archaea\_none | Euryarchaeota | Methanomicrobia | Methanosarcinales | Methanosarcinaceae | Methanomethylovorans | Archaea;Archaea\_none;Euryarchaeota;Methanomicrobia;Methanosarcinales;Methanosarcinaceae;Methanomethylovorans;Methanomethylovorans\_uncultured |
| Methanosarcina horonobensis | 0 | 0 | 0 | 0 | 0 | 0 | 0 | 0 | 0 | 0 | 0 | 0 | 0 | 0 | Archaea | Archaea\_none | Euryarchaeota | Methanomicrobia | Methanosarcinales | Methanosarcinaceae | Methanosarcina | Archaea;Archaea\_none;Euryarchaeota;Methanomicrobia;Methanosarcinales;Methanosarcinaceae;Methanosarcina;Methanosarcina horonobensis |
| Methanosarcina siciliae | 0 | 0 | 0 | 0 | 0 | 0 | 0 | 0 | 0 | 0 | 0 | 0 | 0 | 0 | Archaea | Archaea\_none | Euryarchaeota | Methanomicrobia | Methanosarcinales | Methanosarcinaceae | Methanosarcina | Archaea;Archaea\_none;Euryarchaeota;Methanomicrobia;Methanosarcinales;Methanosarcinaceae;Methanosarcina;Methanosarcina siciliae |
| Thermococcus litoralis | 0 | 0 | 0 | 0 | 0 | 0 | 0 | 0 | 0 | 0 | 0 | 0 | 0 | 0 | Archaea | Archaea\_none | Euryarchaeota | Thermococci | Thermococcales | Thermococcaceae | Thermococcus | Archaea;Archaea\_none;Euryarchaeota;Thermococci;Thermococcales;Thermococcaceae;Thermococcus;Thermococcus litoralis |
| Candidatus Nitrosotenuis chungbukensis | 0 | 0 | 0 | 0 | 0 | 1 | 0 | 0 | 0 | 0 | 0 | 0 | 0 | 0 | Archaea | Archaea\_none | Nitrososphaerota | Nitrososphaerota\_Incertae\_sedis | Nitrososphaerota\_Incertae\_sedis | Nitrososphaerota\_Incertae\_sedis | Candidatus Nitrosotenuis | Archaea;Archaea\_none;Nitrososphaerota;Nitrososphaerota\_Incertae\_sedis;Nitrososphaerota\_Incertae\_sedis;Nitrososphaerota\_Incertae\_sedis;Candidatus Nitrosotenuis;Candidatus Nitrosotenuis chungbukensis |
| Paludibaculum fermentans | 0 | 0 | 0 | 0 | 0 | 0 | 0 | 0 | 0 | 0 | 0 | 0 | 0 | 0 | Bacteria | Bacteria\_none | Acidobacteriota | Terriglobia | Bryobacterales | Bryobacteraceae | Paludibaculum | Bacteria;Bacteria\_none;Acidobacteriota;Terriglobia;Bryobacterales;Bryobacteraceae;Paludibaculum;Paludibaculum fermentans |
| Aquihabitans sp. G128 | 0 | 0 | 0 | 0 | 0 | 0 | 0 | 0 | 0 | 0 | 0 | 0 | 0 | 0 | Bacteria | Bacteria\_none | Actinomycetota | Acidimicrobiia | Acidimicrobiales | Iamiaceae | Aquihabitans | Bacteria;Bacteria\_none;Actinomycetota;Acidimicrobiia;Acidimicrobiales;Iamiaceae;Aquihabitans;Aquihabitans sp. G128 |
| Actinomyces naeslundii | 0 | 0 | 0 | 0 | 0 | 0 | 0 | 0 | 0 | 0 | 0 | 0 | 0 | 0 | Bacteria | Bacteria\_none | Actinomycetota | Actinomycetes | Actinomycetales | Actinomycetaceae | Actinomyces | Bacteria;Bacteria\_none;Actinomycetota;Actinomycetes;Actinomycetales;Actinomycetaceae;Actinomyces;Actinomyces naeslundii |
| Actinomyces oris | 0 | 0 | 0 | 0 | 0 | 0 | 0 | 0 | 0 | 0 | 0 | 1 | 0 | 0 | Bacteria | Bacteria\_none | Actinomycetota | Actinomycetes | Actinomycetales | Actinomycetaceae | Actinomyces | Bacteria;Bacteria\_none;Actinomycetota;Actinomycetes;Actinomycetales;Actinomycetaceae;Actinomyces;Actinomyces oris |
| Actinomyces pacaensis | 0 | 1 | 0 | 0 | 1 | 15 | 7 | 0 | 1 | 0 | 0 | 1 | 1 | 1 | Bacteria | Bacteria\_none | Actinomycetota | Actinomycetes | Actinomycetales | Actinomycetaceae | Actinomyces | Bacteria;Bacteria\_none;Actinomycetota;Actinomycetes;Actinomycetales;Actinomycetaceae;Actinomyces;Actinomyces pacaensis |
| Actinomyces qiguomingii | 1 | 0 | 0 | 0 | 0 | 0 | 0 | 0 | 0 | 0 | 0 | 0 | 0 | 0 | Bacteria | Bacteria\_none | Actinomycetota | Actinomycetes | Actinomycetales | Actinomycetaceae | Actinomyces | Bacteria;Bacteria\_none;Actinomycetota;Actinomycetes;Actinomycetales;Actinomycetaceae;Actinomyces;Actinomyces qiguomingii |
| Actinomyces sp. oral taxon 414 | 0 | 0 | 0 | 0 | 0 | 0 | 0 | 0 | 0 | 0 | 0 | 0 | 0 | 0 | Bacteria | Bacteria\_none | Actinomycetota | Actinomycetes | Actinomycetales | Actinomycetaceae | Actinomyces | Bacteria;Bacteria\_none;Actinomycetota;Actinomycetes;Actinomycetales;Actinomycetaceae;Actinomyces;Actinomyces sp. oral taxon 414 |
| Actinomyces trachealis | 0 | 0 | 0 | 0 | 0 | 0 | 0 | 0 | 0 | 0 | 0 | 0 | 0 | 0 | Bacteria | Bacteria\_none | Actinomycetota | Actinomycetes | Actinomycetales | Actinomycetaceae | Actinomyces | Bacteria;Bacteria\_none;Actinomycetota;Actinomycetes;Actinomycetales;Actinomycetaceae;Actinomyces;Actinomyces trachealis |
| Schaalia meyeri | 0 | 0 | 0 | 0 | 0 | 1 | 0 | 0 | 0 | 0 | 0 | 0 | 0 | 0 | Bacteria | Bacteria\_none | Actinomycetota | Actinomycetes | Actinomycetales | Actinomycetaceae | Schaalia | Bacteria;Bacteria\_none;Actinomycetota;Actinomycetes;Actinomycetales;Actinomycetaceae;Schaalia;Schaalia meyeri |
| Schaalia odontolytica | 0 | 16 | 0 | 2 | 1 | 9 | 9 | 0 | 0 | 0 | 0 | 1 | 3 | 0 | Bacteria | Bacteria\_none | Actinomycetota | Actinomycetes | Actinomycetales | Actinomycetaceae | Schaalia | Bacteria;Bacteria\_none;Actinomycetota;Actinomycetes;Actinomycetales;Actinomycetaceae;Schaalia;Schaalia odontolytica |
| Schaalia sp. HMT-172 | 0 | 0 | 0 | 0 | 0 | 5 | 3 | 0 | 0 | 0 | 0 | 0 | 1 | 0 | Bacteria | Bacteria\_none | Actinomycetota | Actinomycetes | Actinomycetales | Actinomycetaceae | Schaalia | Bacteria;Bacteria\_none;Actinomycetota;Actinomycetes;Actinomycetales;Actinomycetaceae;Schaalia;Schaalia sp. HMT-172 |
| Schaalia sp. ZJ405 | 0 | 0 | 1 | 0 | 0 | 0 | 0 | 0 | 0 | 0 | 0 | 0 | 0 | 0 | Bacteria | Bacteria\_none | Actinomycetota | Actinomycetes | Actinomycetales | Actinomycetaceae | Schaalia | Bacteria;Bacteria\_none;Actinomycetota;Actinomycetes;Actinomycetales;Actinomycetaceae;Schaalia;Schaalia sp. ZJ405 |
| Bifidobacterium asteroides | 0 | 0 | 1 | 0 | 0 | 0 | 0 | 0 | 0 | 0 | 0 | 0 | 0 | 0 | Bacteria | Bacteria\_none | Actinomycetota | Actinomycetes | Bifidobacteriales | Bifidobacteriaceae | Bifidobacterium | Bacteria;Bacteria\_none;Actinomycetota;Actinomycetes;Bifidobacteriales;Bifidobacteriaceae;Bifidobacterium;Bifidobacterium asteroides |
| Bifidobacterium dentium | 0 | 0 | 0 | 0 | 0 | 0 | 0 | 0 | 0 | 0 | 0 | 0 | 0 | 0 | Bacteria | Bacteria\_none | Actinomycetota | Actinomycetes | Bifidobacteriales | Bifidobacteriaceae | Bifidobacterium | Bacteria;Bacteria\_none;Actinomycetota;Actinomycetes;Bifidobacteriales;Bifidobacteriaceae;Bifidobacterium;Bifidobacterium dentium |
| Bifidobacterium longum | 0 | 0 | 0 | 0 | 0 | 1 | 1 | 0 | 0 | 0 | 0 | 0 | 0 | 0 | Bacteria | Bacteria\_none | Actinomycetota | Actinomycetes | Bifidobacteriales | Bifidobacteriaceae | Bifidobacterium | Bacteria;Bacteria\_none;Actinomycetota;Actinomycetes;Bifidobacteriales;Bifidobacteriaceae;Bifidobacterium;Bifidobacterium longum |
| Bifidobacterium pullorum | 0 | 0 | 0 | 0 | 0 | 0 | 0 | 0 | 0 | 0 | 0 | 0 | 0 | 0 | Bacteria | Bacteria\_none | Actinomycetota | Actinomycetes | Bifidobacteriales | Bifidobacteriaceae | Bifidobacterium | Bacteria;Bacteria\_none;Actinomycetota;Actinomycetes;Bifidobacteriales;Bifidobacteriaceae;Bifidobacterium;Bifidobacterium pullorum |
| Gardnerella vaginalis | 0 | 1 | 1 | 0 | 0 | 0 | 0 | 0 | 0 | 0 | 0 | 0 | 1 | 0 | Bacteria | Bacteria\_none | Actinomycetota | Actinomycetes | Bifidobacteriales | Bifidobacteriaceae | Gardnerella | Bacteria;Bacteria\_none;Actinomycetota;Actinomycetes;Bifidobacteriales;Bifidobacteriaceae;Gardnerella;Gardnerella vaginalis |
| Parascardovia denticolens | 0 | 0 | 0 | 0 | 1 | 0 | 2 | 0 | 0 | 0 | 0 | 0 | 0 | 0 | Bacteria | Bacteria\_none | Actinomycetota | Actinomycetes | Bifidobacteriales | Bifidobacteriaceae | Parascardovia | Bacteria;Bacteria\_none;Actinomycetota;Actinomycetes;Bifidobacteriales;Bifidobacteriaceae;Parascardovia;Parascardovia denticolens |
| Geodermatophilus obscurus | 0 | 0 | 0 | 0 | 1 | 0 | 0 | 0 | 0 | 0 | 0 | 0 | 0 | 0 | Bacteria | Bacteria\_none | Actinomycetota | Actinomycetes | Geodermatophilales | Geodermatophilaceae | Geodermatophilus | Bacteria;Bacteria\_none;Actinomycetota;Actinomycetes;Geodermatophilales;Geodermatophilaceae;Geodermatophilus;Geodermatophilus obscurus |
| Streptomyces cadmiisoli | 0 | 0 | 0 | 0 | 0 | 0 | 0 | 0 | 0 | 0 | 0 | 0 | 0 | 0 | Bacteria | Bacteria\_none | Actinomycetota | Actinomycetes | Kitasatosporales | Streptomycetaceae | Streptomyces | Bacteria;Bacteria\_none;Actinomycetota;Actinomycetes;Kitasatosporales;Streptomycetaceae;Streptomyces;Streptomyces cadmiisoli |
| Streptomyces caniferus | 0 | 0 | 0 | 0 | 0 | 0 | 0 | 0 | 0 | 0 | 0 | 0 | 0 | 0 | Bacteria | Bacteria\_none | Actinomycetota | Actinomycetes | Kitasatosporales | Streptomycetaceae | Streptomyces | Bacteria;Bacteria\_none;Actinomycetota;Actinomycetes;Kitasatosporales;Streptomycetaceae;Streptomyces;Streptomyces caniferus |
| Streptomyces gilvosporeus | 0 | 0 | 0 | 0 | 0 | 0 | 0 | 0 | 0 | 0 | 0 | 0 | 0 | 0 | Bacteria | Bacteria\_none | Actinomycetota | Actinomycetes | Kitasatosporales | Streptomycetaceae | Streptomyces | Bacteria;Bacteria\_none;Actinomycetota;Actinomycetes;Kitasatosporales;Streptomycetaceae;Streptomyces;Streptomyces gilvosporeus |
| Streptomyces noursei | 0 | 0 | 0 | 0 | 0 | 0 | 0 | 0 | 0 | 0 | 0 | 0 | 0 | 0 | Bacteria | Bacteria\_none | Actinomycetota | Actinomycetes | Kitasatosporales | Streptomycetaceae | Streptomyces | Bacteria;Bacteria\_none;Actinomycetota;Actinomycetes;Kitasatosporales;Streptomycetaceae;Streptomyces;Streptomyces noursei |
| Streptomyces qaidamensis | 0 | 0 | 0 | 0 | 0 | 0 | 0 | 0 | 0 | 0 | 0 | 0 | 0 | 0 | Bacteria | Bacteria\_none | Actinomycetota | Actinomycetes | Kitasatosporales | Streptomycetaceae | Streptomyces | Bacteria;Bacteria\_none;Actinomycetota;Actinomycetes;Kitasatosporales;Streptomycetaceae;Streptomyces;Streptomyces qaidamensis |
| Streptomyces rimosus | 0 | 0 | 0 | 0 | 0 | 0 | 1 | 0 | 0 | 0 | 0 | 0 | 0 | 0 | Bacteria | Bacteria\_none | Actinomycetota | Actinomycetes | Kitasatosporales | Streptomycetaceae | Streptomyces | Bacteria;Bacteria\_none;Actinomycetota;Actinomycetes;Kitasatosporales;Streptomycetaceae;Streptomyces;Streptomyces rimosus |
| Streptomyces sp. 11x1 | 0 | 0 | 0 | 0 | 0 | 1 | 0 | 0 | 0 | 0 | 0 | 0 | 0 | 0 | Bacteria | Bacteria\_none | Actinomycetota | Actinomycetes | Kitasatosporales | Streptomycetaceae | Streptomyces | Bacteria;Bacteria\_none;Actinomycetota;Actinomycetes;Kitasatosporales;Streptomycetaceae;Streptomyces;Streptomyces sp. 11x1 |
| Streptomyces sp. 2114.2 | 0 | 0 | 0 | 0 | 0 | 1 | 0 | 0 | 0 | 0 | 0 | 0 | 1 | 0 | Bacteria | Bacteria\_none | Actinomycetota | Actinomycetes | Kitasatosporales | Streptomycetaceae | Streptomyces | Bacteria;Bacteria\_none;Actinomycetota;Actinomycetes;Kitasatosporales;Streptomycetaceae;Streptomyces;Streptomyces sp. 2114.2 |
| Streptomyces sp. CB00271 | 0 | 0 | 0 | 0 | 0 | 0 | 0 | 0 | 0 | 0 | 0 | 1 | 0 | 0 | Bacteria | Bacteria\_none | Actinomycetota | Actinomycetes | Kitasatosporales | Streptomycetaceae | Streptomyces | Bacteria;Bacteria\_none;Actinomycetota;Actinomycetes;Kitasatosporales;Streptomycetaceae;Streptomyces;Streptomyces sp. CB00271 |
| Streptomyces sp. CNQ-509 | 0 | 0 | 0 | 0 | 1 | 0 | 0 | 0 | 0 | 0 | 0 | 0 | 0 | 0 | Bacteria | Bacteria\_none | Actinomycetota | Actinomycetes | Kitasatosporales | Streptomycetaceae | Streptomyces | Bacteria;Bacteria\_none;Actinomycetota;Actinomycetes;Kitasatosporales;Streptomycetaceae;Streptomyces;Streptomyces sp. CNQ-509 |
| Streptomyces sp. FXJ1.172 | 0 | 0 | 0 | 0 | 0 | 0 | 0 | 0 | 0 | 0 | 0 | 0 | 0 | 0 | Bacteria | Bacteria\_none | Actinomycetota | Actinomycetes | Kitasatosporales | Streptomycetaceae | Streptomyces | Bacteria;Bacteria\_none;Actinomycetota;Actinomycetes;Kitasatosporales;Streptomycetaceae;Streptomyces;Streptomyces sp. FXJ1.172 |
| Streptomyces sp. HUAS 15-9 | 0 | 0 | 0 | 0 | 0 | 1 | 0 | 0 | 0 | 0 | 0 | 0 | 0 | 0 | Bacteria | Bacteria\_none | Actinomycetota | Actinomycetes | Kitasatosporales | Streptomycetaceae | Streptomyces | Bacteria;Bacteria\_none;Actinomycetota;Actinomycetes;Kitasatosporales;Streptomycetaceae;Streptomyces;Streptomyces sp. HUAS 15-9 |
| Streptomyces sp. Li-HN-5-11 | 0 | 0 | 0 | 1 | 0 | 0 | 0 | 0 | 0 | 0 | 0 | 0 | 0 | 0 | Bacteria | Bacteria\_none | Actinomycetota | Actinomycetes | Kitasatosporales | Streptomycetaceae | Streptomyces | Bacteria;Bacteria\_none;Actinomycetota;Actinomycetes;Kitasatosporales;Streptomycetaceae;Streptomyces;Streptomyces sp. Li-HN-5-11 |
| Streptomyces sp. NBC\_00247 | 0 | 0 | 0 | 0 | 0 | 0 | 0 | 0 | 0 | 0 | 0 | 0 | 0 | 0 | Bacteria | Bacteria\_none | Actinomycetota | Actinomycetes | Kitasatosporales | Streptomycetaceae | Streptomyces | Bacteria;Bacteria\_none;Actinomycetota;Actinomycetes;Kitasatosporales;Streptomycetaceae;Streptomyces;Streptomyces sp. NBC\_00247 |
| Streptomyces sp. NBC\_00250 | 0 | 0 | 0 | 0 | 0 | 1 | 0 | 0 | 0 | 0 | 0 | 0 | 0 | 0 | Bacteria | Bacteria\_none | Actinomycetota | Actinomycetes | Kitasatosporales | Streptomycetaceae | Streptomyces | Bacteria;Bacteria\_none;Actinomycetota;Actinomycetes;Kitasatosporales;Streptomycetaceae;Streptomyces;Streptomyces sp. NBC\_00250 |
| Streptomyces sp. NBC\_00370 | 0 | 1 | 0 | 0 | 0 | 0 | 0 | 0 | 0 | 0 | 0 | 0 | 0 | 0 | Bacteria | Bacteria\_none | Actinomycetota | Actinomycetes | Kitasatosporales | Streptomycetaceae | Streptomyces | Bacteria;Bacteria\_none;Actinomycetota;Actinomycetes;Kitasatosporales;Streptomycetaceae;Streptomyces;Streptomyces sp. NBC\_00370 |
| Streptomyces sp. NBC\_00425 | 0 | 0 | 0 | 0 | 0 | 1 | 0 | 0 | 0 | 0 | 0 | 0 | 0 | 0 | Bacteria | Bacteria\_none | Actinomycetota | Actinomycetes | Kitasatosporales | Streptomycetaceae | Streptomyces | Bacteria;Bacteria\_none;Actinomycetota;Actinomycetes;Kitasatosporales;Streptomycetaceae;Streptomyces;Streptomyces sp. NBC\_00425 |
| Streptomyces sp. NBC\_00490 | 0 | 0 | 0 | 0 | 0 | 0 | 1 | 0 | 0 | 0 | 0 | 0 | 0 | 0 | Bacteria | Bacteria\_none | Actinomycetota | Actinomycetes | Kitasatosporales | Streptomycetaceae | Streptomyces | Bacteria;Bacteria\_none;Actinomycetota;Actinomycetes;Kitasatosporales;Streptomycetaceae;Streptomyces;Streptomyces sp. NBC\_00490 |
| Streptomyces sp. NBC\_01341 | 0 | 0 | 0 | 0 | 0 | 0 | 0 | 0 | 0 | 0 | 0 | 0 | 0 | 1 | Bacteria | Bacteria\_none | Actinomycetota | Actinomycetes | Kitasatosporales | Streptomycetaceae | Streptomyces | Bacteria;Bacteria\_none;Actinomycetota;Actinomycetes;Kitasatosporales;Streptomycetaceae;Streptomyces;Streptomyces sp. NBC\_01341 |
| Streptomyces sp. NBC\_01435 | 0 | 0 | 0 | 0 | 0 | 0 | 0 | 0 | 0 | 0 | 0 | 0 | 0 | 0 | Bacteria | Bacteria\_none | Actinomycetota | Actinomycetes | Kitasatosporales | Streptomycetaceae | Streptomyces | Bacteria;Bacteria\_none;Actinomycetota;Actinomycetes;Kitasatosporales;Streptomycetaceae;Streptomyces;Streptomyces sp. NBC\_01435 |
| Streptomyces sp. NBC\_01788 | 0 | 0 | 0 | 0 | 0 | 1 | 0 | 0 | 0 | 0 | 0 | 0 | 0 | 0 | Bacteria | Bacteria\_none | Actinomycetota | Actinomycetes | Kitasatosporales | Streptomycetaceae | Streptomyces | Bacteria;Bacteria\_none;Actinomycetota;Actinomycetes;Kitasatosporales;Streptomycetaceae;Streptomyces;Streptomyces sp. NBC\_01788 |
| Streptomyces sp. PCS3-D2 | 0 | 0 | 0 | 0 | 0 | 1 | 0 | 0 | 0 | 0 | 0 | 0 | 0 | 0 | Bacteria | Bacteria\_none | Actinomycetota | Actinomycetes | Kitasatosporales | Streptomycetaceae | Streptomyces | Bacteria;Bacteria\_none;Actinomycetota;Actinomycetes;Kitasatosporales;Streptomycetaceae;Streptomyces;Streptomyces sp. PCS3-D2 |
| Streptomyces sp. Tu 2975 | 0 | 1 | 0 | 0 | 0 | 0 | 0 | 0 | 0 | 0 | 0 | 0 | 0 | 0 | Bacteria | Bacteria\_none | Actinomycetota | Actinomycetes | Kitasatosporales | Streptomycetaceae | Streptomyces | Bacteria;Bacteria\_none;Actinomycetota;Actinomycetes;Kitasatosporales;Streptomycetaceae;Streptomyces;Streptomyces sp. Tu 2975 |
| Streptomyces tubbatahanensis | 0 | 0 | 0 | 0 | 0 | 0 | 0 | 0 | 0 | 0 | 0 | 0 | 0 | 0 | Bacteria | Bacteria\_none | Actinomycetota | Actinomycetes | Kitasatosporales | Streptomycetaceae | Streptomyces | Bacteria;Bacteria\_none;Actinomycetota;Actinomycetes;Kitasatosporales;Streptomycetaceae;Streptomyces;Streptomyces tubbatahanensis |
| Streptomyces tubercidicus | 0 | 0 | 0 | 0 | 0 | 0 | 0 | 0 | 0 | 0 | 0 | 0 | 0 | 0 | Bacteria | Bacteria\_none | Actinomycetota | Actinomycetes | Kitasatosporales | Streptomycetaceae | Streptomyces | Bacteria;Bacteria\_none;Actinomycetota;Actinomycetes;Kitasatosporales;Streptomycetaceae;Streptomyces;Streptomyces tubercidicus |
| Cellulomonas wangleii | 0 | 0 | 0 | 0 | 0 | 0 | 0 | 0 | 0 | 0 | 0 | 0 | 0 | 0 | Bacteria | Bacteria\_none | Actinomycetota | Actinomycetes | Micrococcales | Cellulomonadaceae | Cellulomonas | Bacteria;Bacteria\_none;Actinomycetota;Actinomycetes;Micrococcales;Cellulomonadaceae;Cellulomonas;Cellulomonas wangleii |
| Gephyromycinifex aptenodytis | 0 | 0 | 0 | 0 | 0 | 0 | 0 | 0 | 0 | 0 | 0 | 0 | 0 | 0 | Bacteria | Bacteria\_none | Actinomycetota | Actinomycetes | Micrococcales | Dermatophilaceae | Gephyromycinifex | Bacteria;Bacteria\_none;Actinomycetota;Actinomycetes;Micrococcales;Dermatophilaceae;Gephyromycinifex;Gephyromycinifex aptenodytis |
| Agromyces soli | 1 | 0 | 0 | 0 | 0 | 0 | 0 | 0 | 0 | 0 | 0 | 0 | 0 | 0 | Bacteria | Bacteria\_none | Actinomycetota | Actinomycetes | Micrococcales | Microbacteriaceae | Agromyces | Bacteria;Bacteria\_none;Actinomycetota;Actinomycetes;Micrococcales;Microbacteriaceae;Agromyces;Agromyces soli |
| Curtobacterium sp. BH-2-1-1 | 0 | 0 | 0 | 0 | 0 | 0 | 0 | 0 | 0 | 0 | 0 | 0 | 0 | 0 | Bacteria | Bacteria\_none | Actinomycetota | Actinomycetes | Micrococcales | Microbacteriaceae | Curtobacterium | Bacteria;Bacteria\_none;Actinomycetota;Actinomycetes;Micrococcales;Microbacteriaceae;Curtobacterium;Curtobacterium sp. BH-2-1-1 |
| Curtobacterium sp. MCPF17\_021 | 0 | 0 | 0 | 0 | 0 | 0 | 0 | 0 | 0 | 0 | 0 | 0 | 0 | 0 | Bacteria | Bacteria\_none | Actinomycetota | Actinomycetes | Micrococcales | Microbacteriaceae | Curtobacterium | Bacteria;Bacteria\_none;Actinomycetota;Actinomycetes;Micrococcales;Microbacteriaceae;Curtobacterium;Curtobacterium sp. MCPF17\_021 |
| Diaminobutyricibacter sp. McL0608 | 0 | 0 | 0 | 1 | 0 | 0 | 0 | 0 | 0 | 0 | 0 | 0 | 0 | 0 | Bacteria | Bacteria\_none | Actinomycetota | Actinomycetes | Micrococcales | Microbacteriaceae | Diaminobutyricibacter | Bacteria;Bacteria\_none;Actinomycetota;Actinomycetes;Micrococcales;Microbacteriaceae;Diaminobutyricibacter;Diaminobutyricibacter sp. McL0608 |
| Leucobacter viscericola | 0 | 0 | 0 | 0 | 0 | 0 | 1 | 0 | 0 | 0 | 0 | 0 | 0 | 0 | Bacteria | Bacteria\_none | Actinomycetota | Actinomycetes | Micrococcales | Microbacteriaceae | Leucobacter | Bacteria;Bacteria\_none;Actinomycetota;Actinomycetes;Micrococcales;Microbacteriaceae;Leucobacter;Leucobacter viscericola |
| Microbacterium binotii | 0 | 0 | 0 | 0 | 0 | 0 | 0 | 1 | 0 | 0 | 0 | 0 | 0 | 0 | Bacteria | Bacteria\_none | Actinomycetota | Actinomycetes | Micrococcales | Microbacteriaceae | Microbacterium | Bacteria;Bacteria\_none;Actinomycetota;Actinomycetes;Micrococcales;Microbacteriaceae;Microbacterium;Microbacterium binotii |
| Microbacterium sp. Clip185 | 0 | 0 | 0 | 0 | 1 | 0 | 0 | 0 | 0 | 0 | 0 | 0 | 0 | 0 | Bacteria | Bacteria\_none | Actinomycetota | Actinomycetes | Micrococcales | Microbacteriaceae | Microbacterium | Bacteria;Bacteria\_none;Actinomycetota;Actinomycetes;Micrococcales;Microbacteriaceae;Microbacterium;Microbacterium sp. Clip185 |
| Microcella alkaliphila | 0 | 0 | 0 | 0 | 0 | 0 | 0 | 0 | 0 | 0 | 0 | 0 | 0 | 0 | Bacteria | Bacteria\_none | Actinomycetota | Actinomycetes | Micrococcales | Microbacteriaceae | Microcella | Bacteria;Bacteria\_none;Actinomycetota;Actinomycetes;Micrococcales;Microbacteriaceae;Microcella;Microcella alkaliphila |
| Arthrobacter citreus | 0 | 0 | 0 | 0 | 0 | 0 | 0 | 0 | 0 | 0 | 0 | 0 | 1 | 0 | Bacteria | Bacteria\_none | Actinomycetota | Actinomycetes | Micrococcales | Micrococcaceae | Arthrobacter | Bacteria;Bacteria\_none;Actinomycetota;Actinomycetes;Micrococcales;Micrococcaceae;Arthrobacter;Arthrobacter citreus |
| Arthrobacter oryzae | 0 | 0 | 0 | 0 | 0 | 1 | 0 | 0 | 0 | 0 | 0 | 0 | 0 | 0 | Bacteria | Bacteria\_none | Actinomycetota | Actinomycetes | Micrococcales | Micrococcaceae | Arthrobacter | Bacteria;Bacteria\_none;Actinomycetota;Actinomycetes;Micrococcales;Micrococcaceae;Arthrobacter;Arthrobacter oryzae |
| Arthrobacter sp. ATA002 | 0 | 0 | 0 | 0 | 0 | 0 | 0 | 0 | 0 | 0 | 0 | 0 | 0 | 0 | Bacteria | Bacteria\_none | Actinomycetota | Actinomycetes | Micrococcales | Micrococcaceae | Arthrobacter | Bacteria;Bacteria\_none;Actinomycetota;Actinomycetes;Micrococcales;Micrococcaceae;Arthrobacter;Arthrobacter sp. ATA002 |
| Arthrobacter sp. FW306-2-2C-D06B | 0 | 0 | 0 | 0 | 0 | 0 | 0 | 0 | 0 | 1 | 0 | 0 | 0 | 0 | Bacteria | Bacteria\_none | Actinomycetota | Actinomycetes | Micrococcales | Micrococcaceae | Arthrobacter | Bacteria;Bacteria\_none;Actinomycetota;Actinomycetes;Micrococcales;Micrococcaceae;Arthrobacter;Arthrobacter sp. FW306-2-2C-D06B |
| Pseudarthrobacter sp. NS4 | 0 | 0 | 0 | 0 | 1 | 0 | 0 | 0 | 0 | 0 | 0 | 0 | 0 | 0 | Bacteria | Bacteria\_none | Actinomycetota | Actinomycetes | Micrococcales | Micrococcaceae | Pseudarthrobacter | Bacteria;Bacteria\_none;Actinomycetota;Actinomycetes;Micrococcales;Micrococcaceae;Pseudarthrobacter;Pseudarthrobacter sp. NS4 |
| Rothia aeria | 13 | 1 | 0 | 1 | 26 | 0 | 0 | 14 | 0 | 0 | 35 | 7 | 3 | 5 | Bacteria | Bacteria\_none | Actinomycetota | Actinomycetes | Micrococcales | Micrococcaceae | Rothia | Bacteria;Bacteria\_none;Actinomycetota;Actinomycetes;Micrococcales;Micrococcaceae;Rothia;Rothia aeria |
| Rothia dentocariosa | 4 | 23 | 1 | 2 | 4 | 3 | 3 | 1 | 0 | 0 | 2 | 9 | 4 | 3 | Bacteria | Bacteria\_none | Actinomycetota | Actinomycetes | Micrococcales | Micrococcaceae | Rothia | Bacteria;Bacteria\_none;Actinomycetota;Actinomycetes;Micrococcales;Micrococcaceae;Rothia;Rothia dentocariosa |
| Rothia mucilaginosa | 7 | 79 | 20 | 6 | 41 | 29 | 10 | 0 | 6 | 38 | 26 | 60 | 18 | 11 | Bacteria | Bacteria\_none | Actinomycetota | Actinomycetes | Micrococcales | Micrococcaceae | Rothia | Bacteria;Bacteria\_none;Actinomycetota;Actinomycetes;Micrococcales;Micrococcaceae;Rothia;Rothia mucilaginosa |
| Luteimicrobium xylanilyticum | 0 | 0 | 0 | 0 | 0 | 0 | 0 | 0 | 0 | 0 | 0 | 0 | 0 | 0 | Bacteria | Bacteria\_none | Actinomycetota | Actinomycetes | Micrococcales | Micrococcales\_Incertae\_sedis | Luteimicrobium | Bacteria;Bacteria\_none;Actinomycetota;Actinomycetes;Micrococcales;Micrococcales\_Incertae\_sedis;Luteimicrobium;Luteimicrobium xylanilyticum |
| Ruania halotolerans | 0 | 0 | 0 | 0 | 0 | 0 | 0 | 0 | 0 | 0 | 0 | 0 | 0 | 0 | Bacteria | Bacteria\_none | Actinomycetota | Actinomycetes | Micrococcales | Ruaniaceae | Ruania | Bacteria;Bacteria\_none;Actinomycetota;Actinomycetes;Micrococcales;Ruaniaceae;Ruania;Ruania halotolerans |
| Micromonospora aurantiaca | 0 | 0 | 0 | 0 | 0 | 0 | 0 | 0 | 0 | 0 | 0 | 0 | 0 | 1 | Bacteria | Bacteria\_none | Actinomycetota | Actinomycetes | Micromonosporales | Micromonosporaceae | Micromonospora | Bacteria;Bacteria\_none;Actinomycetota;Actinomycetes;Micromonosporales;Micromonosporaceae;Micromonospora;Micromonospora aurantiaca |
| Micromonospora coriariae | 0 | 0 | 0 | 0 | 0 | 0 | 0 | 0 | 0 | 0 | 0 | 0 | 0 | 0 | Bacteria | Bacteria\_none | Actinomycetota | Actinomycetes | Micromonosporales | Micromonosporaceae | Micromonospora | Bacteria;Bacteria\_none;Actinomycetota;Actinomycetes;Micromonosporales;Micromonosporaceae;Micromonospora;Micromonospora coriariae |
| Micromonospora halotolerans | 1 | 0 | 0 | 0 | 0 | 0 | 0 | 0 | 0 | 0 | 0 | 0 | 0 | 0 | Bacteria | Bacteria\_none | Actinomycetota | Actinomycetes | Micromonosporales | Micromonosporaceae | Micromonospora | Bacteria;Bacteria\_none;Actinomycetota;Actinomycetes;Micromonosporales;Micromonosporaceae;Micromonospora;Micromonospora halotolerans |
| Micromonospora sp. DSM 102119 | 0 | 0 | 0 | 1 | 0 | 0 | 0 | 0 | 0 | 0 | 0 | 0 | 0 | 0 | Bacteria | Bacteria\_none | Actinomycetota | Actinomycetes | Micromonosporales | Micromonosporaceae | Micromonospora | Bacteria;Bacteria\_none;Actinomycetota;Actinomycetes;Micromonosporales;Micromonosporaceae;Micromonospora;Micromonospora sp. DSM 102119 |
| Micromonospora sp. WMMD1076 | 0 | 0 | 0 | 0 | 0 | 1 | 0 | 0 | 0 | 0 | 0 | 0 | 0 | 0 | Bacteria | Bacteria\_none | Actinomycetota | Actinomycetes | Micromonosporales | Micromonosporaceae | Micromonospora | Bacteria;Bacteria\_none;Actinomycetota;Actinomycetes;Micromonosporales;Micromonosporaceae;Micromonospora;Micromonospora sp. WMMD1076 |
| Micromonospora sp. WMMD882 | 0 | 0 | 0 | 0 | 0 | 0 | 0 | 1 | 0 | 0 | 0 | 0 | 0 | 0 | Bacteria | Bacteria\_none | Actinomycetota | Actinomycetes | Micromonosporales | Micromonosporaceae | Micromonospora | Bacteria;Bacteria\_none;Actinomycetota;Actinomycetes;Micromonosporales;Micromonosporaceae;Micromonospora;Micromonospora sp. WMMD882 |
| Salinispora tropica | 0 | 0 | 0 | 0 | 0 | 0 | 0 | 0 | 0 | 1 | 0 | 0 | 0 | 0 | Bacteria | Bacteria\_none | Actinomycetota | Actinomycetes | Micromonosporales | Micromonosporaceae | Salinispora | Bacteria;Bacteria\_none;Actinomycetota;Actinomycetes;Micromonosporales;Micromonosporaceae;Salinispora;Salinispora tropica |
| Corynebacterium amycolatum | 0 | 0 | 0 | 0 | 0 | 1 | 0 | 0 | 0 | 0 | 0 | 0 | 0 | 0 | Bacteria | Bacteria\_none | Actinomycetota | Actinomycetes | Mycobacteriales | Corynebacteriaceae | Corynebacterium | Bacteria;Bacteria\_none;Actinomycetota;Actinomycetes;Mycobacteriales;Corynebacteriaceae;Corynebacterium;Corynebacterium amycolatum |
| Corynebacterium choanae | 0 | 0 | 0 | 0 | 0 | 0 | 0 | 0 | 0 | 0 | 0 | 1 | 0 | 0 | Bacteria | Bacteria\_none | Actinomycetota | Actinomycetes | Mycobacteriales | Corynebacteriaceae | Corynebacterium | Bacteria;Bacteria\_none;Actinomycetota;Actinomycetes;Mycobacteriales;Corynebacteriaceae;Corynebacterium;Corynebacterium choanae |
| Corynebacterium durum | 0 | 0 | 0 | 0 | 0 | 1 | 0 | 0 | 0 | 4 | 0 | 0 | 0 | 0 | Bacteria | Bacteria\_none | Actinomycetota | Actinomycetes | Mycobacteriales | Corynebacteriaceae | Corynebacterium | Bacteria;Bacteria\_none;Actinomycetota;Actinomycetes;Mycobacteriales;Corynebacteriaceae;Corynebacterium;Corynebacterium durum |
| Corynebacterium matruchotii | 0 | 6 | 0 | 0 | 0 | 1 | 0 | 0 | 0 | 2 | 0 | 2 | 1 | 0 | Bacteria | Bacteria\_none | Actinomycetota | Actinomycetes | Mycobacteriales | Corynebacteriaceae | Corynebacterium | Bacteria;Bacteria\_none;Actinomycetota;Actinomycetes;Mycobacteriales;Corynebacteriaceae;Corynebacterium;Corynebacterium matruchotii |
| Gordonia sp. L191 | 0 | 0 | 0 | 0 | 0 | 0 | 0 | 0 | 0 | 0 | 0 | 0 | 0 | 0 | Bacteria | Bacteria\_none | Actinomycetota | Actinomycetes | Mycobacteriales | Gordoniaceae | Gordonia | Bacteria;Bacteria\_none;Actinomycetota;Actinomycetes;Mycobacteriales;Gordoniaceae;Gordonia;Gordonia sp. L191 |
| Gordonia sp. PP30 | 0 | 0 | 0 | 0 | 1 | 0 | 0 | 0 | 0 | 0 | 0 | 0 | 0 | 0 | Bacteria | Bacteria\_none | Actinomycetota | Actinomycetes | Mycobacteriales | Gordoniaceae | Gordonia | Bacteria;Bacteria\_none;Actinomycetota;Actinomycetes;Mycobacteriales;Gordoniaceae;Gordonia;Gordonia sp. PP30 |
| Mycobacterium gallinarum | 0 | 0 | 1 | 0 | 0 | 0 | 0 | 0 | 0 | 0 | 0 | 0 | 0 | 0 | Bacteria | Bacteria\_none | Actinomycetota | Actinomycetes | Mycobacteriales | Mycobacteriaceae | Mycobacterium | Bacteria;Bacteria\_none;Actinomycetota;Actinomycetes;Mycobacteriales;Mycobacteriaceae;Mycobacterium;Mycobacterium gallinarum |
| Mycobacterium leprae | 0 | 0 | 0 | 1 | 0 | 0 | 0 | 0 | 0 | 0 | 0 | 0 | 0 | 0 | Bacteria | Bacteria\_none | Actinomycetota | Actinomycetes | Mycobacteriales | Mycobacteriaceae | Mycobacterium | Bacteria;Bacteria\_none;Actinomycetota;Actinomycetes;Mycobacteriales;Mycobacteriaceae;Mycobacterium;Mycobacterium leprae |
| Mycolicibacterium alvei | 0 | 0 | 0 | 0 | 0 | 0 | 0 | 0 | 0 | 0 | 0 | 0 | 0 | 0 | Bacteria | Bacteria\_none | Actinomycetota | Actinomycetes | Mycobacteriales | Mycobacteriaceae | Mycolicibacterium | Bacteria;Bacteria\_none;Actinomycetota;Actinomycetes;Mycobacteriales;Mycobacteriaceae;Mycolicibacterium;Mycolicibacterium alvei |
| Mycolicibacterium aubagnense | 0 | 0 | 1 | 0 | 0 | 0 | 0 | 0 | 0 | 0 | 0 | 0 | 0 | 0 | Bacteria | Bacteria\_none | Actinomycetota | Actinomycetes | Mycobacteriales | Mycobacteriaceae | Mycolicibacterium | Bacteria;Bacteria\_none;Actinomycetota;Actinomycetes;Mycobacteriales;Mycobacteriaceae;Mycolicibacterium;Mycolicibacterium aubagnense |
| Mycolicibacterium diernhoferi | 0 | 1 | 0 | 0 | 0 | 0 | 0 | 0 | 0 | 0 | 0 | 0 | 0 | 0 | Bacteria | Bacteria\_none | Actinomycetota | Actinomycetes | Mycobacteriales | Mycobacteriaceae | Mycolicibacterium | Bacteria;Bacteria\_none;Actinomycetota;Actinomycetes;Mycobacteriales;Mycobacteriaceae;Mycolicibacterium;Mycolicibacterium diernhoferi |
| Mycolicibacterium duvalii | 0 | 0 | 0 | 0 | 0 | 0 | 0 | 0 | 0 | 0 | 0 | 0 | 0 | 0 | Bacteria | Bacteria\_none | Actinomycetota | Actinomycetes | Mycobacteriales | Mycobacteriaceae | Mycolicibacterium | Bacteria;Bacteria\_none;Actinomycetota;Actinomycetes;Mycobacteriales;Mycobacteriaceae;Mycolicibacterium;Mycolicibacterium duvalii |
| Mycolicibacterium gadium | 0 | 0 | 0 | 0 | 0 | 0 | 0 | 0 | 0 | 0 | 0 | 0 | 0 | 0 | Bacteria | Bacteria\_none | Actinomycetota | Actinomycetes | Mycobacteriales | Mycobacteriaceae | Mycolicibacterium | Bacteria;Bacteria\_none;Actinomycetota;Actinomycetes;Mycobacteriales;Mycobacteriaceae;Mycolicibacterium;Mycolicibacterium gadium |
| Mycolicibacterium rhodesiae | 0 | 0 | 0 | 0 | 0 | 0 | 0 | 0 | 0 | 0 | 0 | 0 | 0 | 0 | Bacteria | Bacteria\_none | Actinomycetota | Actinomycetes | Mycobacteriales | Mycobacteriaceae | Mycolicibacterium | Bacteria;Bacteria\_none;Actinomycetota;Actinomycetes;Mycobacteriales;Mycobacteriaceae;Mycolicibacterium;Mycolicibacterium rhodesiae |
| Nocardia sp. NBC\_01503 | 0 | 0 | 0 | 0 | 0 | 0 | 0 | 0 | 0 | 0 | 0 | 0 | 0 | 0 | Bacteria | Bacteria\_none | Actinomycetota | Actinomycetes | Mycobacteriales | Nocardiaceae | Nocardia | Bacteria;Bacteria\_none;Actinomycetota;Actinomycetes;Mycobacteriales;Nocardiaceae;Nocardia;Nocardia sp. NBC\_01503 |
| Rhodococcus sp. USK10 | 0 | 0 | 0 | 0 | 0 | 0 | 0 | 0 | 0 | 0 | 1 | 0 | 0 | 0 | Bacteria | Bacteria\_none | Actinomycetota | Actinomycetes | Mycobacteriales | Nocardiaceae | Rhodococcus | Bacteria;Bacteria\_none;Actinomycetota;Actinomycetes;Mycobacteriales;Nocardiaceae;Rhodococcus;Rhodococcus sp. USK10 |
| Kribbella sp. NBC\_01245 | 0 | 0 | 0 | 0 | 0 | 0 | 0 | 0 | 0 | 0 | 0 | 0 | 0 | 0 | Bacteria | Bacteria\_none | Actinomycetota | Actinomycetes | Propionibacteriales | Kribbellaceae | Kribbella | Bacteria;Bacteria\_none;Actinomycetota;Actinomycetes;Propionibacteriales;Kribbellaceae;Kribbella;Kribbella sp. NBC\_01245 |
| Aeromicrobium wangtongii | 0 | 0 | 0 | 1 | 0 | 0 | 0 | 0 | 0 | 0 | 0 | 0 | 0 | 0 | Bacteria | Bacteria\_none | Actinomycetota | Actinomycetes | Propionibacteriales | Nocardioidaceae | Aeromicrobium | Bacteria;Bacteria\_none;Actinomycetota;Actinomycetes;Propionibacteriales;Nocardioidaceae;Aeromicrobium;Aeromicrobium wangtongii |
| Nocardioides baekrokdamisoli | 0 | 0 | 0 | 0 | 0 | 0 | 0 | 0 | 0 | 0 | 0 | 0 | 0 | 0 | Bacteria | Bacteria\_none | Actinomycetota | Actinomycetes | Propionibacteriales | Nocardioidaceae | Nocardioides | Bacteria;Bacteria\_none;Actinomycetota;Actinomycetes;Propionibacteriales;Nocardioidaceae;Nocardioides;Nocardioides baekrokdamisoli |
| Nocardioides euryhalodurans | 0 | 0 | 0 | 0 | 0 | 0 | 0 | 0 | 0 | 0 | 0 | 0 | 0 | 0 | Bacteria | Bacteria\_none | Actinomycetota | Actinomycetes | Propionibacteriales | Nocardioidaceae | Nocardioides | Bacteria;Bacteria\_none;Actinomycetota;Actinomycetes;Propionibacteriales;Nocardioidaceae;Nocardioides;Nocardioides euryhalodurans |
| Nocardioides scoriae | 0 | 0 | 0 | 0 | 1 | 0 | 0 | 0 | 0 | 0 | 0 | 0 | 0 | 0 | Bacteria | Bacteria\_none | Actinomycetota | Actinomycetes | Propionibacteriales | Nocardioidaceae | Nocardioides | Bacteria;Bacteria\_none;Actinomycetota;Actinomycetes;Propionibacteriales;Nocardioidaceae;Nocardioides;Nocardioides scoriae |
| Acidipropionibacterium acidipropionici | 0 | 0 | 0 | 0 | 0 | 0 | 0 | 0 | 0 | 0 | 0 | 0 | 0 | 0 | Bacteria | Bacteria\_none | Actinomycetota | Actinomycetes | Propionibacteriales | Propionibacteriaceae | Acidipropionibacterium | Bacteria;Bacteria\_none;Actinomycetota;Actinomycetes;Propionibacteriales;Propionibacteriaceae;Acidipropionibacterium;Acidipropionibacterium acidipropionici |
| Arachnia propionica | 0 | 1 | 0 | 0 | 0 | 1 | 0 | 0 | 0 | 0 | 5 | 0 | 0 | 0 | Bacteria | Bacteria\_none | Actinomycetota | Actinomycetes | Propionibacteriales | Propionibacteriaceae | Arachnia | Bacteria;Bacteria\_none;Actinomycetota;Actinomycetes;Propionibacteriales;Propionibacteriaceae;Arachnia;Arachnia propionica |
| Arachnia rubra | 0 | 0 | 0 | 0 | 0 | 0 | 0 | 0 | 0 | 0 | 11 | 0 | 0 | 0 | Bacteria | Bacteria\_none | Actinomycetota | Actinomycetes | Propionibacteriales | Propionibacteriaceae | Arachnia | Bacteria;Bacteria\_none;Actinomycetota;Actinomycetes;Propionibacteriales;Propionibacteriaceae;Arachnia;Arachnia rubra |
| Cutibacterium acnes | 0 | 0 | 0 | 0 | 0 | 0 | 0 | 0 | 0 | 0 | 0 | 0 | 0 | 0 | Bacteria | Bacteria\_none | Actinomycetota | Actinomycetes | Propionibacteriales | Propionibacteriaceae | Cutibacterium | Bacteria;Bacteria\_none;Actinomycetota;Actinomycetes;Propionibacteriales;Propionibacteriaceae;Cutibacterium;Cutibacterium acnes |
| Propionibacterium freudenreichii | 0 | 0 | 0 | 0 | 0 | 0 | 0 | 0 | 0 | 0 | 0 | 0 | 1 | 0 | Bacteria | Bacteria\_none | Actinomycetota | Actinomycetes | Propionibacteriales | Propionibacteriaceae | Propionibacterium | Bacteria;Bacteria\_none;Actinomycetota;Actinomycetes;Propionibacteriales;Propionibacteriaceae;Propionibacterium;Propionibacterium freudenreichii |
| Vaginimicrobium propionicum | 0 | 0 | 0 | 0 | 0 | 0 | 0 | 0 | 0 | 0 | 0 | 0 | 0 | 0 | Bacteria | Bacteria\_none | Actinomycetota | Actinomycetes | Propionibacteriales | Propionibacteriaceae | Vaginimicrobium | Bacteria;Bacteria\_none;Actinomycetota;Actinomycetes;Propionibacteriales;Propionibacteriaceae;Vaginimicrobium;Vaginimicrobium propionicum |
| Amycolatopsis sp. YIM 10 | 0 | 0 | 0 | 1 | 0 | 0 | 0 | 0 | 0 | 0 | 0 | 0 | 0 | 0 | Bacteria | Bacteria\_none | Actinomycetota | Actinomycetes | Pseudonocardiales | Pseudonocardiaceae | Amycolatopsis | Bacteria;Bacteria\_none;Actinomycetota;Actinomycetes;Pseudonocardiales;Pseudonocardiaceae;Amycolatopsis;Amycolatopsis sp. YIM 10 |
| Crossiella sp. CA-258035 | 0 | 0 | 0 | 0 | 0 | 0 | 0 | 0 | 0 | 0 | 0 | 0 | 0 | 0 | Bacteria | Bacteria\_none | Actinomycetota | Actinomycetes | Pseudonocardiales | Pseudonocardiaceae | Crossiella | Bacteria;Bacteria\_none;Actinomycetota;Actinomycetes;Pseudonocardiales;Pseudonocardiaceae;Crossiella;Crossiella sp. CA-258035 |
| Saccharopolyspora spinosa | 0 | 0 | 0 | 0 | 0 | 0 | 0 | 0 | 0 | 0 | 0 | 0 | 0 | 0 | Bacteria | Bacteria\_none | Actinomycetota | Actinomycetes | Pseudonocardiales | Pseudonocardiaceae | Saccharopolyspora | Bacteria;Bacteria\_none;Actinomycetota;Actinomycetes;Pseudonocardiales;Pseudonocardiaceae;Saccharopolyspora;Saccharopolyspora spinosa |
| Nonomuraea fuscirosea | 0 | 0 | 0 | 0 | 0 | 0 | 0 | 0 | 0 | 0 | 0 | 0 | 0 | 0 | Bacteria | Bacteria\_none | Actinomycetota | Actinomycetes | Streptosporangiales | Streptosporangiaceae | Nonomuraea | Bacteria;Bacteria\_none;Actinomycetota;Actinomycetes;Streptosporangiales;Streptosporangiaceae;Nonomuraea;Nonomuraea fuscirosea |
| Nonomuraea phyllanthi | 0 | 0 | 0 | 0 | 0 | 0 | 0 | 0 | 0 | 0 | 0 | 0 | 0 | 0 | Bacteria | Bacteria\_none | Actinomycetota | Actinomycetes | Streptosporangiales | Streptosporangiaceae | Nonomuraea | Bacteria;Bacteria\_none;Actinomycetota;Actinomycetes;Streptosporangiales;Streptosporangiaceae;Nonomuraea;Nonomuraea phyllanthi |
| Streptosporangium roseum | 0 | 0 | 0 | 0 | 1 | 0 | 0 | 0 | 0 | 0 | 0 | 0 | 0 | 0 | Bacteria | Bacteria\_none | Actinomycetota | Actinomycetes | Streptosporangiales | Streptosporangiaceae | Streptosporangium | Bacteria;Bacteria\_none;Actinomycetota;Actinomycetes;Streptosporangiales;Streptosporangiaceae;Streptosporangium;Streptosporangium roseum |
| Streptosporangium sp. NBC\_01495 | 0 | 0 | 1 | 0 | 0 | 0 | 0 | 0 | 0 | 0 | 0 | 0 | 0 | 0 | Bacteria | Bacteria\_none | Actinomycetota | Actinomycetes | Streptosporangiales | Streptosporangiaceae | Streptosporangium | Bacteria;Bacteria\_none;Actinomycetota;Actinomycetes;Streptosporangiales;Streptosporangiaceae;Streptosporangium;Streptosporangium sp. NBC\_01495 |
| Actinomadura madurae | 0 | 0 | 0 | 1 | 0 | 0 | 0 | 0 | 0 | 0 | 0 | 0 | 0 | 0 | Bacteria | Bacteria\_none | Actinomycetota | Actinomycetes | Streptosporangiales | Thermomonosporaceae | Actinomadura | Bacteria;Bacteria\_none;Actinomycetota;Actinomycetes;Streptosporangiales;Thermomonosporaceae;Actinomadura;Actinomadura madurae |
| Thermomonospora amylolytica | 0 | 0 | 0 | 0 | 0 | 0 | 0 | 0 | 0 | 0 | 0 | 0 | 0 | 0 | Bacteria | Bacteria\_none | Actinomycetota | Actinomycetes | Streptosporangiales | Thermomonosporaceae | Thermomonospora | Bacteria;Bacteria\_none;Actinomycetota;Actinomycetes;Streptosporangiales;Thermomonosporaceae;Thermomonospora;Thermomonospora amylolytica |
| Atopobium sp. oral taxon 416 | 0 | 0 | 0 | 0 | 0 | 0 | 0 | 0 | 0 | 0 | 0 | 0 | 0 | 0 | Bacteria | Bacteria\_none | Actinomycetota | Coriobacteriia | Coriobacteriales | Atopobiaceae | Atopobium | Bacteria;Bacteria\_none;Actinomycetota;Coriobacteriia;Coriobacteriales;Atopobiaceae;Atopobium;Atopobium sp. oral taxon 416 |
| Fannyhessea vaginae | 0 | 0 | 0 | 0 | 0 | 0 | 0 | 0 | 0 | 0 | 0 | 0 | 0 | 0 | Bacteria | Bacteria\_none | Actinomycetota | Coriobacteriia | Coriobacteriales | Atopobiaceae | Fannyhessea | Bacteria;Bacteria\_none;Actinomycetota;Coriobacteriia;Coriobacteriales;Atopobiaceae;Fannyhessea;Fannyhessea vaginae |
| Lancefieldella parvula | 24 | 5 | 7 | 0 | 2 | 91 | 21 | 1 | 0 | 0 | 0 | 2 | 3 | 2 | Bacteria | Bacteria\_none | Actinomycetota | Coriobacteriia | Coriobacteriales | Atopobiaceae | Lancefieldella | Bacteria;Bacteria\_none;Actinomycetota;Coriobacteriia;Coriobacteriales;Atopobiaceae;Lancefieldella;Lancefieldella parvula |
| Lancefieldella sp. Marseille-Q7238 | 0 | 0 | 2 | 0 | 0 | 1 | 0 | 0 | 0 | 0 | 4 | 0 | 0 | 0 | Bacteria | Bacteria\_none | Actinomycetota | Coriobacteriia | Coriobacteriales | Atopobiaceae | Lancefieldella | Bacteria;Bacteria\_none;Actinomycetota;Coriobacteriia;Coriobacteriales;Atopobiaceae;Lancefieldella;Lancefieldella sp. Marseille-Q7238 |
| Olsenella uli | 0 | 0 | 0 | 0 | 0 | 1 | 0 | 0 | 0 | 0 | 0 | 0 | 0 | 0 | Bacteria | Bacteria\_none | Actinomycetota | Coriobacteriia | Coriobacteriales | Atopobiaceae | Olsenella | Bacteria;Bacteria\_none;Actinomycetota;Coriobacteriia;Coriobacteriales;Atopobiaceae;Olsenella;Olsenella uli |
| Thermophilibacter mediterraneus | 0 | 0 | 0 | 0 | 0 | 0 | 1 | 0 | 0 | 0 | 0 | 0 | 0 | 0 | Bacteria | Bacteria\_none | Actinomycetota | Coriobacteriia | Coriobacteriales | Atopobiaceae | Thermophilibacter | Bacteria;Bacteria\_none;Actinomycetota;Coriobacteriia;Coriobacteriales;Atopobiaceae;Thermophilibacter;Thermophilibacter mediterraneus |
| Cryptobacterium curtum | 0 | 0 | 0 | 0 | 0 | 0 | 0 | 0 | 0 | 0 | 0 | 0 | 0 | 0 | Bacteria | Bacteria\_none | Actinomycetota | Coriobacteriia | Eggerthellales | Eggerthellaceae | Cryptobacterium | Bacteria;Bacteria\_none;Actinomycetota;Coriobacteriia;Eggerthellales;Eggerthellaceae;Cryptobacterium;Cryptobacterium curtum |
| Denitrobacterium detoxificans | 0 | 0 | 0 | 0 | 0 | 0 | 0 | 0 | 0 | 0 | 0 | 0 | 0 | 0 | Bacteria | Bacteria\_none | Actinomycetota | Coriobacteriia | Eggerthellales | Eggerthellaceae | Denitrobacterium | Bacteria;Bacteria\_none;Actinomycetota;Coriobacteriia;Eggerthellales;Eggerthellaceae;Denitrobacterium;Denitrobacterium detoxificans |
| Raoultibacter timonensis | 0 | 0 | 0 | 0 | 0 | 0 | 0 | 0 | 0 | 0 | 0 | 0 | 0 | 0 | Bacteria | Bacteria\_none | Actinomycetota | Coriobacteriia | Eggerthellales | Eggerthellaceae | Raoultibacter | Bacteria;Bacteria\_none;Actinomycetota;Coriobacteriia;Eggerthellales;Eggerthellaceae;Raoultibacter;Raoultibacter timonensis |
| Rubrobacter radiotolerans | 0 | 0 | 0 | 0 | 1 | 0 | 0 | 0 | 0 | 0 | 0 | 0 | 0 | 0 | Bacteria | Bacteria\_none | Actinomycetota | Rubrobacteria | Rubrobacterales | Rubrobacteraceae | Rubrobacter | Bacteria;Bacteria\_none;Actinomycetota;Rubrobacteria;Rubrobacterales;Rubrobacteraceae;Rubrobacter;Rubrobacter radiotolerans |
| Miltoncostaea marina | 0 | 1 | 0 | 0 | 0 | 0 | 0 | 0 | 0 | 0 | 0 | 0 | 0 | 0 | Bacteria | Bacteria\_none | Actinomycetota | Thermoleophilia | Miltoncostaeales | Miltoncostaeaceae | Miltoncostaea | Bacteria;Bacteria\_none;Actinomycetota;Thermoleophilia;Miltoncostaeales;Miltoncostaeaceae;Miltoncostaea;Miltoncostaea marina |
| Baekduia alba | 0 | 1 | 0 | 0 | 0 | 0 | 0 | 0 | 0 | 0 | 0 | 0 | 0 | 0 | Bacteria | Bacteria\_none | Actinomycetota | Thermoleophilia | Solirubrobacterales | Baekduiaceae | Baekduia | Bacteria;Bacteria\_none;Actinomycetota;Thermoleophilia;Solirubrobacterales;Baekduiaceae;Baekduia;Baekduia alba |
| Abyssicoccus albus | 0 | 0 | 0 | 0 | 0 | 0 | 0 | 0 | 0 | 0 | 0 | 0 | 0 | 0 | Bacteria | Bacteria\_none | Bacillota | Bacilli | Bacillales | Abyssicoccaceae | Abyssicoccus | Bacteria;Bacteria\_none;Bacillota;Bacilli;Bacillales;Abyssicoccaceae;Abyssicoccus;Abyssicoccus albus |
| Alicyclobacillus fastidiosus | 0 | 0 | 0 | 0 | 0 | 0 | 0 | 0 | 0 | 0 | 0 | 0 | 0 | 0 | Bacteria | Bacteria\_none | Bacillota | Bacilli | Bacillales | Alicyclobacillaceae | Alicyclobacillus | Bacteria;Bacteria\_none;Bacillota;Bacilli;Bacillales;Alicyclobacillaceae;Alicyclobacillus;Alicyclobacillus fastidiosus |
| Alkalihalobacillus sp. LMS39 | 0 | 0 | 0 | 1 | 0 | 0 | 0 | 0 | 0 | 0 | 0 | 0 | 0 | 0 | Bacteria | Bacteria\_none | Bacillota | Bacilli | Bacillales | Bacillaceae | Alkalihalobacillus | Bacteria;Bacteria\_none;Bacillota;Bacilli;Bacillales;Bacillaceae;Alkalihalobacillus;Alkalihalobacillus sp. LMS39 |
| Alkalihalophilus pseudofirmus | 0 | 0 | 0 | 0 | 0 | 0 | 0 | 0 | 0 | 0 | 1 | 0 | 0 | 1 | Bacteria | Bacteria\_none | Bacillota | Bacilli | Bacillales | Bacillaceae | Alkalihalophilus | Bacteria;Bacteria\_none;Bacillota;Bacilli;Bacillales;Bacillaceae;Alkalihalophilus;Alkalihalophilus pseudofirmus |
| Amphibacillus xylanus | 0 | 0 | 0 | 0 | 0 | 0 | 0 | 0 | 0 | 0 | 0 | 0 | 0 | 0 | Bacteria | Bacteria\_none | Bacillota | Bacilli | Bacillales | Bacillaceae | Amphibacillus | Bacteria;Bacteria\_none;Bacillota;Bacilli;Bacillales;Bacillaceae;Amphibacillus;Amphibacillus xylanus |
| Anaerobacillus isosaccharinicus | 0 | 1 | 0 | 0 | 0 | 0 | 0 | 0 | 0 | 0 | 0 | 0 | 0 | 0 | Bacteria | Bacteria\_none | Bacillota | Bacilli | Bacillales | Bacillaceae | Anaerobacillus | Bacteria;Bacteria\_none;Bacillota;Bacilli;Bacillales;Bacillaceae;Anaerobacillus;Anaerobacillus isosaccharinicus |
| Anoxybacillus sp. FSL W8-1294 | 1 | 0 | 0 | 0 | 0 | 0 | 0 | 0 | 0 | 0 | 0 | 0 | 0 | 0 | Bacteria | Bacteria\_none | Bacillota | Bacilli | Bacillales | Bacillaceae | Anoxybacillus | Bacteria;Bacteria\_none;Bacillota;Bacilli;Bacillales;Bacillaceae;Anoxybacillus;Anoxybacillus sp. FSL W8-1294 |
| Bacillus amyloliquefaciens | 0 | 0 | 0 | 0 | 0 | 0 | 0 | 0 | 0 | 0 | 0 | 0 | 0 | 0 | Bacteria | Bacteria\_none | Bacillota | Bacilli | Bacillales | Bacillaceae | Bacillus | Bacteria;Bacteria\_none;Bacillota;Bacilli;Bacillales;Bacillaceae;Bacillus;Bacillus amyloliquefaciens |
| Bacillus cereus | 0 | 0 | 0 | 0 | 0 | 0 | 0 | 0 | 0 | 0 | 0 | 0 | 0 | 0 | Bacteria | Bacteria\_none | Bacillota | Bacilli | Bacillales | Bacillaceae | Bacillus | Bacteria;Bacteria\_none;Bacillota;Bacilli;Bacillales;Bacillaceae;Bacillus;Bacillus cereus |
| Bacillus methanolicus | 0 | 0 | 0 | 0 | 0 | 0 | 0 | 0 | 0 | 0 | 0 | 0 | 0 | 0 | Bacteria | Bacteria\_none | Bacillota | Bacilli | Bacillales | Bacillaceae | Bacillus | Bacteria;Bacteria\_none;Bacillota;Bacilli;Bacillales;Bacillaceae;Bacillus;Bacillus methanolicus |
| Bacillus mycoides | 0 | 0 | 0 | 0 | 0 | 1 | 0 | 0 | 0 | 0 | 0 | 0 | 0 | 0 | Bacteria | Bacteria\_none | Bacillota | Bacilli | Bacillales | Bacillaceae | Bacillus | Bacteria;Bacteria\_none;Bacillota;Bacilli;Bacillales;Bacillaceae;Bacillus;Bacillus mycoides |
| Bacillus nitratireducens | 0 | 0 | 0 | 1 | 0 | 0 | 0 | 1 | 0 | 0 | 0 | 0 | 0 | 0 | Bacteria | Bacteria\_none | Bacillota | Bacilli | Bacillales | Bacillaceae | Bacillus | Bacteria;Bacteria\_none;Bacillota;Bacilli;Bacillales;Bacillaceae;Bacillus;Bacillus nitratireducens |
| Bacillus pseudomycoides | 0 | 0 | 0 | 0 | 0 | 1 | 0 | 0 | 0 | 0 | 0 | 0 | 0 | 0 | Bacteria | Bacteria\_none | Bacillota | Bacilli | Bacillales | Bacillaceae | Bacillus | Bacteria;Bacteria\_none;Bacillota;Bacilli;Bacillales;Bacillaceae;Bacillus;Bacillus pseudomycoides |
| Bacillus safensis | 0 | 0 | 0 | 0 | 0 | 0 | 0 | 0 | 0 | 0 | 0 | 0 | 0 | 0 | Bacteria | Bacteria\_none | Bacillota | Bacilli | Bacillales | Bacillaceae | Bacillus | Bacteria;Bacteria\_none;Bacillota;Bacilli;Bacillales;Bacillaceae;Bacillus;Bacillus safensis |
| Bacillus sp. 1NLA3E | 0 | 0 | 0 | 1 | 0 | 0 | 0 | 0 | 0 | 0 | 0 | 0 | 0 | 0 | Bacteria | Bacteria\_none | Bacillota | Bacilli | Bacillales | Bacillaceae | Bacillus | Bacteria;Bacteria\_none;Bacillota;Bacilli;Bacillales;Bacillaceae;Bacillus;Bacillus sp. 1NLA3E |
| Bacillus sp. FJAT-22090 | 0 | 0 | 0 | 0 | 0 | 1 | 0 | 0 | 0 | 0 | 0 | 0 | 0 | 0 | Bacteria | Bacteria\_none | Bacillota | Bacilli | Bacillales | Bacillaceae | Bacillus | Bacteria;Bacteria\_none;Bacillota;Bacilli;Bacillales;Bacillaceae;Bacillus;Bacillus sp. FJAT-22090 |
| Bacillus sp. N5-665 | 0 | 0 | 0 | 0 | 0 | 0 | 0 | 0 | 0 | 0 | 0 | 0 | 0 | 0 | Bacteria | Bacteria\_none | Bacillota | Bacilli | Bacillales | Bacillaceae | Bacillus | Bacteria;Bacteria\_none;Bacillota;Bacilli;Bacillales;Bacillaceae;Bacillus;Bacillus sp. N5-665 |
| Bacillus sp. NEB1478 | 1 | 0 | 0 | 0 | 0 | 0 | 0 | 0 | 0 | 0 | 0 | 0 | 0 | 0 | Bacteria | Bacteria\_none | Bacillota | Bacilli | Bacillales | Bacillaceae | Bacillus | Bacteria;Bacteria\_none;Bacillota;Bacilli;Bacillales;Bacillaceae;Bacillus;Bacillus sp. NEB1478 |
| Bacillus thuringiensis | 0 | 0 | 0 | 1 | 0 | 0 | 0 | 0 | 0 | 0 | 0 | 0 | 0 | 0 | Bacteria | Bacteria\_none | Bacillota | Bacilli | Bacillales | Bacillaceae | Bacillus | Bacteria;Bacteria\_none;Bacillota;Bacilli;Bacillales;Bacillaceae;Bacillus;Bacillus thuringiensis |
| Bacillus wiedmannii | 0 | 0 | 0 | 0 | 0 | 0 | 0 | 0 | 0 | 0 | 0 | 0 | 0 | 0 | Bacteria | Bacteria\_none | Bacillota | Bacilli | Bacillales | Bacillaceae | Bacillus | Bacteria;Bacteria\_none;Bacillota;Bacilli;Bacillales;Bacillaceae;Bacillus;Bacillus wiedmannii |
| Cytobacillus firmus | 0 | 0 | 0 | 0 | 0 | 0 | 0 | 0 | 0 | 0 | 0 | 0 | 0 | 1 | Bacteria | Bacteria\_none | Bacillota | Bacilli | Bacillales | Bacillaceae | Cytobacillus | Bacteria;Bacteria\_none;Bacillota;Bacilli;Bacillales;Bacillaceae;Cytobacillus;Cytobacillus firmus |
| Cytobacillus gottheilii | 0 | 0 | 1 | 0 | 0 | 0 | 0 | 0 | 0 | 0 | 0 | 0 | 0 | 0 | Bacteria | Bacteria\_none | Bacillota | Bacilli | Bacillales | Bacillaceae | Cytobacillus | Bacteria;Bacteria\_none;Bacillota;Bacilli;Bacillales;Bacillaceae;Cytobacillus;Cytobacillus gottheilii |
| Cytobacillus oceanisediminis | 0 | 0 | 0 | 1 | 0 | 0 | 0 | 0 | 0 | 0 | 0 | 0 | 0 | 0 | Bacteria | Bacteria\_none | Bacillota | Bacilli | Bacillales | Bacillaceae | Cytobacillus | Bacteria;Bacteria\_none;Bacillota;Bacilli;Bacillales;Bacillaceae;Cytobacillus;Cytobacillus oceanisediminis |
| Domibacillus sp. DTU\_2020\_1001157\_1\_SI\_ALB\_TIR\_016 | 0 | 0 | 0 | 0 | 0 | 0 | 0 | 0 | 0 | 0 | 0 | 0 | 0 | 0 | Bacteria | Bacteria\_none | Bacillota | Bacilli | Bacillales | Bacillaceae | Domibacillus | Bacteria;Bacteria\_none;Bacillota;Bacilli;Bacillales;Bacillaceae;Domibacillus;Domibacillus sp. DTU\_2020\_1001157\_1\_SI\_ALB\_TIR\_016 |
| Halalkalibacter krulwichiae | 0 | 0 | 0 | 0 | 0 | 0 | 0 | 0 | 0 | 0 | 0 | 0 | 0 | 0 | Bacteria | Bacteria\_none | Bacillota | Bacilli | Bacillales | Bacillaceae | Halalkalibacter | Bacteria;Bacteria\_none;Bacillota;Bacilli;Bacillales;Bacillaceae;Halalkalibacter;Halalkalibacter krulwichiae |
| Halobacillus litoralis | 0 | 0 | 0 | 0 | 0 | 0 | 0 | 0 | 0 | 0 | 0 | 0 | 0 | 0 | Bacteria | Bacteria\_none | Bacillota | Bacilli | Bacillales | Bacillaceae | Halobacillus | Bacteria;Bacteria\_none;Bacillota;Bacilli;Bacillales;Bacillaceae;Halobacillus;Halobacillus litoralis |
| Lysinibacillus capsici | 0 | 1 | 0 | 0 | 0 | 0 | 0 | 0 | 0 | 0 | 0 | 0 | 0 | 0 | Bacteria | Bacteria\_none | Bacillota | Bacilli | Bacillales | Bacillaceae | Lysinibacillus | Bacteria;Bacteria\_none;Bacillota;Bacilli;Bacillales;Bacillaceae;Lysinibacillus;Lysinibacillus capsici |
| Lysinibacillus fusiformis | 0 | 0 | 0 | 0 | 0 | 0 | 1 | 0 | 0 | 0 | 0 | 0 | 0 | 0 | Bacteria | Bacteria\_none | Bacillota | Bacilli | Bacillales | Bacillaceae | Lysinibacillus | Bacteria;Bacteria\_none;Bacillota;Bacilli;Bacillales;Bacillaceae;Lysinibacillus;Lysinibacillus fusiformis |
| Lysinibacillus irui | 0 | 0 | 1 | 0 | 0 | 1 | 0 | 0 | 0 | 0 | 0 | 0 | 0 | 0 | Bacteria | Bacteria\_none | Bacillota | Bacilli | Bacillales | Bacillaceae | Lysinibacillus | Bacteria;Bacteria\_none;Bacillota;Bacilli;Bacillales;Bacillaceae;Lysinibacillus;Lysinibacillus irui |
| Lysinibacillus macroides | 0 | 0 | 0 | 0 | 0 | 0 | 0 | 0 | 0 | 0 | 0 | 0 | 0 | 0 | Bacteria | Bacteria\_none | Bacillota | Bacilli | Bacillales | Bacillaceae | Lysinibacillus | Bacteria;Bacteria\_none;Bacillota;Bacilli;Bacillales;Bacillaceae;Lysinibacillus;Lysinibacillus macroides |
| Lysinibacillus sphaericus | 0 | 0 | 0 | 0 | 0 | 0 | 0 | 0 | 0 | 0 | 0 | 0 | 0 | 0 | Bacteria | Bacteria\_none | Bacillota | Bacilli | Bacillales | Bacillaceae | Lysinibacillus | Bacteria;Bacteria\_none;Bacillota;Bacilli;Bacillales;Bacillaceae;Lysinibacillus;Lysinibacillus sphaericus |
| Metabacillus sp. FJAT-53654 | 0 | 1 | 0 | 0 | 0 | 0 | 0 | 0 | 0 | 0 | 0 | 0 | 0 | 0 | Bacteria | Bacteria\_none | Bacillota | Bacilli | Bacillales | Bacillaceae | Metabacillus | Bacteria;Bacteria\_none;Bacillota;Bacilli;Bacillales;Bacillaceae;Metabacillus;Metabacillus sp. FJAT-53654 |
| Neobacillus sp. FSL H8-0543 | 0 | 0 | 1 | 0 | 0 | 0 | 0 | 0 | 0 | 0 | 0 | 0 | 0 | 0 | Bacteria | Bacteria\_none | Bacillota | Bacilli | Bacillales | Bacillaceae | Neobacillus | Bacteria;Bacteria\_none;Bacillota;Bacilli;Bacillales;Bacillaceae;Neobacillus;Neobacillus sp. FSL H8-0543 |
| Neobacillus sp. OS1-2 | 0 | 0 | 0 | 0 | 1 | 0 | 0 | 0 | 0 | 0 | 0 | 0 | 0 | 0 | Bacteria | Bacteria\_none | Bacillota | Bacilli | Bacillales | Bacillaceae | Neobacillus | Bacteria;Bacteria\_none;Bacillota;Bacilli;Bacillales;Bacillaceae;Neobacillus;Neobacillus sp. OS1-2 |
| Niallia oryzisoli | 0 | 0 | 1 | 0 | 0 | 1 | 0 | 0 | 0 | 0 | 0 | 0 | 0 | 0 | Bacteria | Bacteria\_none | Bacillota | Bacilli | Bacillales | Bacillaceae | Niallia | Bacteria;Bacteria\_none;Bacillota;Bacilli;Bacillales;Bacillaceae;Niallia;Niallia oryzisoli |
| Niallia sp. XMNu-256 | 0 | 0 | 0 | 0 | 0 | 0 | 0 | 0 | 0 | 0 | 0 | 0 | 0 | 0 | Bacteria | Bacteria\_none | Bacillota | Bacilli | Bacillales | Bacillaceae | Niallia | Bacteria;Bacteria\_none;Bacillota;Bacilli;Bacillales;Bacillaceae;Niallia;Niallia sp. XMNu-256 |
| Niallia taxi | 1 | 0 | 0 | 0 | 0 | 0 | 0 | 0 | 0 | 0 | 0 | 0 | 0 | 0 | Bacteria | Bacteria\_none | Bacillota | Bacilli | Bacillales | Bacillaceae | Niallia | Bacteria;Bacteria\_none;Bacillota;Bacilli;Bacillales;Bacillaceae;Niallia;Niallia taxi |
| Oceanobacillus kimchii | 0 | 0 | 0 | 0 | 0 | 0 | 0 | 0 | 0 | 0 | 0 | 0 | 0 | 0 | Bacteria | Bacteria\_none | Bacillota | Bacilli | Bacillales | Bacillaceae | Oceanobacillus | Bacteria;Bacteria\_none;Bacillota;Bacilli;Bacillales;Bacillaceae;Oceanobacillus;Oceanobacillus kimchii |
| Oceanobacillus sp. FSL W7-1293 | 0 | 0 | 0 | 0 | 0 | 0 | 0 | 0 | 0 | 0 | 0 | 0 | 1 | 0 | Bacteria | Bacteria\_none | Bacillota | Bacilli | Bacillales | Bacillaceae | Oceanobacillus | Bacteria;Bacteria\_none;Bacillota;Bacilli;Bacillales;Bacillaceae;Oceanobacillus;Oceanobacillus sp. FSL W7-1293 |
| Ornithinibacillus sp. FSL M8-0202 | 0 | 0 | 0 | 0 | 0 | 1 | 0 | 0 | 0 | 0 | 0 | 0 | 0 | 0 | Bacteria | Bacteria\_none | Bacillota | Bacilli | Bacillales | Bacillaceae | Ornithinibacillus | Bacteria;Bacteria\_none;Bacillota;Bacilli;Bacillales;Bacillaceae;Ornithinibacillus;Ornithinibacillus sp. FSL M8-0202 |
| Parageobacillus toebii | 0 | 0 | 0 | 0 | 0 | 0 | 0 | 0 | 0 | 0 | 0 | 0 | 0 | 0 | Bacteria | Bacteria\_none | Bacillota | Bacilli | Bacillales | Bacillaceae | Parageobacillus | Bacteria;Bacteria\_none;Bacillota;Bacilli;Bacillales;Bacillaceae;Parageobacillus;Parageobacillus toebii |
| Peribacillus butanolivorans | 0 | 0 | 0 | 0 | 0 | 0 | 0 | 0 | 0 | 0 | 0 | 0 | 0 | 0 | Bacteria | Bacteria\_none | Bacillota | Bacilli | Bacillales | Bacillaceae | Peribacillus | Bacteria;Bacteria\_none;Bacillota;Bacilli;Bacillales;Bacillaceae;Peribacillus;Peribacillus butanolivorans |
| Pontibacillus sp. HMF3514 | 0 | 0 | 0 | 0 | 0 | 0 | 0 | 0 | 0 | 0 | 0 | 0 | 0 | 0 | Bacteria | Bacteria\_none | Bacillota | Bacilli | Bacillales | Bacillaceae | Pontibacillus | Bacteria;Bacteria\_none;Bacillota;Bacilli;Bacillales;Bacillaceae;Pontibacillus;Pontibacillus sp. HMF3514 |
| Priestia aryabhattai | 0 | 0 | 0 | 0 | 1 | 0 | 0 | 0 | 0 | 0 | 0 | 0 | 1 | 0 | Bacteria | Bacteria\_none | Bacillota | Bacilli | Bacillales | Bacillaceae | Priestia | Bacteria;Bacteria\_none;Bacillota;Bacilli;Bacillales;Bacillaceae;Priestia;Priestia aryabhattai |
| Priestia megaterium | 0 | 0 | 1 | 0 | 0 | 1 | 0 | 0 | 0 | 1 | 1 | 0 | 0 | 0 | Bacteria | Bacteria\_none | Bacillota | Bacilli | Bacillales | Bacillaceae | Priestia | Bacteria;Bacteria\_none;Bacillota;Bacilli;Bacillales;Bacillaceae;Priestia;Priestia megaterium |
| Pseudalkalibacillus hwajinpoensis | 0 | 0 | 0 | 0 | 0 | 0 | 0 | 0 | 0 | 0 | 0 | 0 | 0 | 0 | Bacteria | Bacteria\_none | Bacillota | Bacilli | Bacillales | Bacillaceae | Pseudalkalibacillus | Bacteria;Bacteria\_none;Bacillota;Bacilli;Bacillales;Bacillaceae;Pseudalkalibacillus;Pseudalkalibacillus hwajinpoensis |
| Psychrobacillus glaciei | 1 | 0 | 0 | 0 | 0 | 0 | 0 | 0 | 0 | 0 | 0 | 0 | 0 | 0 | Bacteria | Bacteria\_none | Bacillota | Bacilli | Bacillales | Bacillaceae | Psychrobacillus | Bacteria;Bacteria\_none;Bacillota;Bacilli;Bacillales;Bacillaceae;Psychrobacillus;Psychrobacillus glaciei |
| Psychrobacillus sp. INOP01 | 0 | 0 | 0 | 0 | 0 | 0 | 0 | 0 | 0 | 0 | 0 | 0 | 0 | 0 | Bacteria | Bacteria\_none | Bacillota | Bacilli | Bacillales | Bacillaceae | Psychrobacillus | Bacteria;Bacteria\_none;Bacillota;Bacilli;Bacillales;Bacillaceae;Psychrobacillus;Psychrobacillus sp. INOP01 |
| Rossellomorea aquimaris | 0 | 0 | 0 | 0 | 0 | 1 | 0 | 0 | 0 | 0 | 0 | 0 | 0 | 0 | Bacteria | Bacteria\_none | Bacillota | Bacilli | Bacillales | Bacillaceae | Rossellomorea | Bacteria;Bacteria\_none;Bacillota;Bacilli;Bacillales;Bacillaceae;Rossellomorea;Rossellomorea aquimaris |
| Salipaludibacillus sp. LMS25 | 0 | 0 | 0 | 1 | 0 | 0 | 0 | 0 | 0 | 0 | 0 | 0 | 0 | 0 | Bacteria | Bacteria\_none | Bacillota | Bacilli | Bacillales | Bacillaceae | Salipaludibacillus | Bacteria;Bacteria\_none;Bacillota;Bacilli;Bacillales;Bacillaceae;Salipaludibacillus;Salipaludibacillus sp. LMS25 |
| Shouchella lehensis | 0 | 0 | 0 | 0 | 0 | 0 | 0 | 0 | 0 | 0 | 0 | 0 | 0 | 0 | Bacteria | Bacteria\_none | Bacillota | Bacilli | Bacillales | Bacillaceae | Shouchella | Bacteria;Bacteria\_none;Bacillota;Bacilli;Bacillales;Bacillaceae;Shouchella;Shouchella lehensis |
| Sutcliffiella horikoshii | 0 | 0 | 0 | 0 | 1 | 0 | 0 | 0 | 0 | 0 | 0 | 0 | 0 | 0 | Bacteria | Bacteria\_none | Bacillota | Bacilli | Bacillales | Bacillaceae | Sutcliffiella | Bacteria;Bacteria\_none;Bacillota;Bacilli;Bacillales;Bacillaceae;Sutcliffiella;Sutcliffiella horikoshii |
| Terribacillus saccharophilus | 0 | 0 | 1 | 0 | 0 | 0 | 0 | 0 | 0 | 0 | 0 | 0 | 0 | 0 | Bacteria | Bacteria\_none | Bacillota | Bacilli | Bacillales | Bacillaceae | Terribacillus | Bacteria;Bacteria\_none;Bacillota;Bacilli;Bacillales;Bacillaceae;Terribacillus;Terribacillus saccharophilus |
| Virgibacillus sp. NKC19-16 | 0 | 0 | 0 | 0 | 0 | 0 | 0 | 0 | 0 | 0 | 0 | 0 | 0 | 0 | Bacteria | Bacteria\_none | Bacillota | Bacilli | Bacillales | Bacillaceae | Virgibacillus | Bacteria;Bacteria\_none;Bacillota;Bacilli;Bacillales;Bacillaceae;Virgibacillus;Virgibacillus sp. NKC19-16 |
| Exiguobacterium sp. 9-2 | 0 | 0 | 0 | 0 | 0 | 0 | 0 | 0 | 0 | 0 | 0 | 0 | 0 | 0 | Bacteria | Bacteria\_none | Bacillota | Bacilli | Bacillales | Bacillales\_Incertae\_sedis | Exiguobacterium | Bacteria;Bacteria\_none;Bacillota;Bacilli;Bacillales;Bacillales\_Incertae\_sedis;Exiguobacterium;Exiguobacterium sp. 9-2 |
| Gemella haemolysans | 25 | 30 | 2 | 53 | 181 | 113 | 1 | 6 | 0 | 166 | 422 | 8 | 72 | 36 | Bacteria | Bacteria\_none | Bacillota | Bacilli | Bacillales | Gemellaceae | Gemella | Bacteria;Bacteria\_none;Bacillota;Bacilli;Bacillales;Gemellaceae;Gemella;Gemella haemolysans |
| Gemella morbillorum | 0 | 21 | 4 | 0 | 119 | 79 | 0 | 10 | 0 | 10 | 50 | 13 | 24 | 12 | Bacteria | Bacteria\_none | Bacillota | Bacilli | Bacillales | Gemellaceae | Gemella | Bacteria;Bacteria\_none;Bacillota;Bacilli;Bacillales;Gemellaceae;Gemella;Gemella morbillorum |
| Gemella sanguinis | 39 | 68 | 1 | 10 | 49 | 68 | 44 | 17 | 2 | 24 | 34 | 18 | 24 | 13 | Bacteria | Bacteria\_none | Bacillota | Bacilli | Bacillales | Gemellaceae | Gemella | Bacteria;Bacteria\_none;Bacillota;Bacilli;Bacillales;Gemellaceae;Gemella;Gemella sanguinis |
| Gemella sp. oral taxon 928 | 0 | 0 | 0 | 0 | 0 | 0 | 0 | 2 | 0 | 0 | 0 | 0 | 0 | 0 | Bacteria | Bacteria\_none | Bacillota | Bacilli | Bacillales | Gemellaceae | Gemella | Bacteria;Bacteria\_none;Bacillota;Bacilli;Bacillales;Gemellaceae;Gemella;Gemella sp. oral taxon 928 |
| Gemella sp. zg-570 | 0 | 0 | 0 | 0 | 0 | 0 | 0 | 0 | 0 | 0 | 0 | 0 | 0 | 0 | Bacteria | Bacteria\_none | Bacillota | Bacilli | Bacillales | Gemellaceae | Gemella | Bacteria;Bacteria\_none;Bacillota;Bacilli;Bacillales;Gemellaceae;Gemella;Gemella sp. zg-570 |
| Brochothrix thermosphacta | 0 | 0 | 0 | 0 | 0 | 0 | 0 | 0 | 0 | 0 | 0 | 0 | 0 | 0 | Bacteria | Bacteria\_none | Bacillota | Bacilli | Bacillales | Listeriaceae | Brochothrix | Bacteria;Bacteria\_none;Bacillota;Bacilli;Bacillales;Listeriaceae;Brochothrix;Brochothrix thermosphacta |
| Listeria monocytogenes | 0 | 0 | 0 | 0 | 0 | 0 | 0 | 0 | 0 | 0 | 0 | 0 | 0 | 0 | Bacteria | Bacteria\_none | Bacillota | Bacilli | Bacillales | Listeriaceae | Listeria | Bacteria;Bacteria\_none;Bacillota;Bacilli;Bacillales;Listeriaceae;Listeria;Listeria monocytogenes |
| Brevibacillus borstelensis | 0 | 0 | 0 | 1 | 0 | 0 | 0 | 0 | 0 | 0 | 0 | 0 | 1 | 0 | Bacteria | Bacteria\_none | Bacillota | Bacilli | Bacillales | Paenibacillaceae | Brevibacillus | Bacteria;Bacteria\_none;Bacillota;Bacilli;Bacillales;Paenibacillaceae;Brevibacillus;Brevibacillus borstelensis |
| Brevibacillus brevis | 0 | 0 | 0 | 0 | 0 | 0 | 0 | 0 | 0 | 0 | 0 | 0 | 0 | 0 | Bacteria | Bacteria\_none | Bacillota | Bacilli | Bacillales | Paenibacillaceae | Brevibacillus | Bacteria;Bacteria\_none;Bacillota;Bacilli;Bacillales;Paenibacillaceae;Brevibacillus;Brevibacillus brevis |
| Brevibacillus formosus | 0 | 0 | 0 | 0 | 0 | 0 | 0 | 0 | 0 | 0 | 0 | 0 | 0 | 0 | Bacteria | Bacteria\_none | Bacillota | Bacilli | Bacillales | Paenibacillaceae | Brevibacillus | Bacteria;Bacteria\_none;Bacillota;Bacilli;Bacillales;Paenibacillaceae;Brevibacillus;Brevibacillus formosus |
| Brevibacillus laterosporus | 0 | 0 | 0 | 0 | 0 | 0 | 0 | 0 | 0 | 0 | 0 | 0 | 0 | 0 | Bacteria | Bacteria\_none | Bacillota | Bacilli | Bacillales | Paenibacillaceae | Brevibacillus | Bacteria;Bacteria\_none;Bacillota;Bacilli;Bacillales;Paenibacillaceae;Brevibacillus;Brevibacillus laterosporus |
| Paenibacillus andongensis | 0 | 0 | 0 | 0 | 1 | 0 | 0 | 0 | 0 | 0 | 0 | 0 | 0 | 0 | Bacteria | Bacteria\_none | Bacillota | Bacilli | Bacillales | Paenibacillaceae | Paenibacillus | Bacteria;Bacteria\_none;Bacillota;Bacilli;Bacillales;Paenibacillaceae;Paenibacillus;Paenibacillus andongensis |
| Paenibacillus azoreducens | 0 | 0 | 0 | 0 | 0 | 0 | 0 | 0 | 0 | 0 | 0 | 0 | 0 | 0 | Bacteria | Bacteria\_none | Bacillota | Bacilli | Bacillales | Paenibacillaceae | Paenibacillus | Bacteria;Bacteria\_none;Bacillota;Bacilli;Bacillales;Paenibacillaceae;Paenibacillus;Paenibacillus azoreducens |
| Paenibacillus barcinonensis | 0 | 0 | 1 | 0 | 0 | 0 | 0 | 0 | 0 | 0 | 0 | 0 | 0 | 0 | Bacteria | Bacteria\_none | Bacillota | Bacilli | Bacillales | Paenibacillaceae | Paenibacillus | Bacteria;Bacteria\_none;Bacillota;Bacilli;Bacillales;Paenibacillaceae;Paenibacillus;Paenibacillus barcinonensis |
| Paenibacillus chitinolyticus | 0 | 0 | 1 | 0 | 0 | 0 | 0 | 0 | 0 | 0 | 0 | 0 | 0 | 0 | Bacteria | Bacteria\_none | Bacillota | Bacilli | Bacillales | Paenibacillaceae | Paenibacillus | Bacteria;Bacteria\_none;Bacillota;Bacilli;Bacillales;Paenibacillaceae;Paenibacillus;Paenibacillus chitinolyticus |
| Paenibacillus marchantiae | 0 | 1 | 0 | 0 | 0 | 0 | 0 | 0 | 0 | 0 | 0 | 0 | 0 | 0 | Bacteria | Bacteria\_none | Bacillota | Bacilli | Bacillales | Paenibacillaceae | Paenibacillus | Bacteria;Bacteria\_none;Bacillota;Bacilli;Bacillales;Paenibacillaceae;Paenibacillus;Paenibacillus marchantiae |
| Paenibacillus sophorae | 0 | 0 | 0 | 0 | 0 | 0 | 0 | 0 | 0 | 0 | 0 | 0 | 0 | 0 | Bacteria | Bacteria\_none | Bacillota | Bacilli | Bacillales | Paenibacillaceae | Paenibacillus | Bacteria;Bacteria\_none;Bacillota;Bacilli;Bacillales;Paenibacillaceae;Paenibacillus;Paenibacillus sophorae |
| Paenibacillus sp. FSL H8-0332 | 1 | 0 | 0 | 0 | 0 | 0 | 0 | 0 | 0 | 0 | 0 | 0 | 0 | 0 | Bacteria | Bacteria\_none | Bacillota | Bacilli | Bacillales | Paenibacillaceae | Paenibacillus | Bacteria;Bacteria\_none;Bacillota;Bacilli;Bacillales;Paenibacillaceae;Paenibacillus;Paenibacillus sp. FSL H8-0332 |
| Paenibacillus sp. FSL H8-0537 | 0 | 0 | 0 | 0 | 0 | 0 | 0 | 0 | 0 | 0 | 0 | 0 | 0 | 0 | Bacteria | Bacteria\_none | Bacillota | Bacilli | Bacillales | Paenibacillaceae | Paenibacillus | Bacteria;Bacteria\_none;Bacillota;Bacilli;Bacillales;Paenibacillaceae;Paenibacillus;Paenibacillus sp. FSL H8-0537 |
| Paenibacillus sp. FSL L8-0435 | 0 | 0 | 0 | 0 | 0 | 0 | 0 | 0 | 0 | 0 | 0 | 0 | 0 | 0 | Bacteria | Bacteria\_none | Bacillota | Bacilli | Bacillales | Paenibacillaceae | Paenibacillus | Bacteria;Bacteria\_none;Bacillota;Bacilli;Bacillales;Paenibacillaceae;Paenibacillus;Paenibacillus sp. FSL L8-0435 |
| Paenibacillus sp. FSL R10-2734 | 0 | 0 | 0 | 0 | 0 | 0 | 0 | 0 | 0 | 0 | 0 | 0 | 0 | 0 | Bacteria | Bacteria\_none | Bacillota | Bacilli | Bacillales | Paenibacillaceae | Paenibacillus | Bacteria;Bacteria\_none;Bacillota;Bacilli;Bacillales;Paenibacillaceae;Paenibacillus;Paenibacillus sp. FSL R10-2734 |
| Paenibacillus sp. FSL R10-2796 | 0 | 0 | 0 | 1 | 0 | 0 | 0 | 0 | 0 | 0 | 0 | 0 | 0 | 0 | Bacteria | Bacteria\_none | Bacillota | Bacilli | Bacillales | Paenibacillaceae | Paenibacillus | Bacteria;Bacteria\_none;Bacillota;Bacilli;Bacillales;Paenibacillaceae;Paenibacillus;Paenibacillus sp. FSL R10-2796 |
| Paenibacillus sp. FSL R5-0490 | 0 | 0 | 0 | 0 | 0 | 0 | 0 | 0 | 0 | 0 | 0 | 0 | 0 | 0 | Bacteria | Bacteria\_none | Bacillota | Bacilli | Bacillales | Paenibacillaceae | Paenibacillus | Bacteria;Bacteria\_none;Bacillota;Bacilli;Bacillales;Paenibacillaceae;Paenibacillus;Paenibacillus sp. FSL R5-0490 |
| Paenibacillus sp. FSL R5-0517 | 1 | 0 | 0 | 0 | 0 | 0 | 0 | 0 | 0 | 0 | 0 | 0 | 0 | 0 | Bacteria | Bacteria\_none | Bacillota | Bacilli | Bacillales | Paenibacillaceae | Paenibacillus | Bacteria;Bacteria\_none;Bacillota;Bacilli;Bacillales;Paenibacillaceae;Paenibacillus;Paenibacillus sp. FSL R5-0517 |
| Paenibacillus sp. JDR-2 | 0 | 0 | 0 | 0 | 0 | 0 | 1 | 0 | 0 | 0 | 0 | 0 | 0 | 0 | Bacteria | Bacteria\_none | Bacillota | Bacilli | Bacillales | Paenibacillaceae | Paenibacillus | Bacteria;Bacteria\_none;Bacillota;Bacilli;Bacillales;Paenibacillaceae;Paenibacillus;Paenibacillus sp. JDR-2 |
| Paenisporosarcina sp. FSL H8-0542 | 0 | 0 | 0 | 0 | 0 | 0 | 0 | 0 | 0 | 0 | 0 | 0 | 0 | 0 | Bacteria | Bacteria\_none | Bacillota | Bacilli | Bacillales | Planococcaceae | Paenisporosarcina | Bacteria;Bacteria\_none;Bacillota;Bacilli;Bacillales;Planococcaceae;Paenisporosarcina;Paenisporosarcina sp. FSL H8-0542 |
| Sporosarcina ureilytica | 1 | 0 | 0 | 0 | 0 | 0 | 0 | 0 | 0 | 0 | 0 | 0 | 0 | 0 | Bacteria | Bacteria\_none | Bacillota | Bacilli | Bacillales | Planococcaceae | Sporosarcina | Bacteria;Bacteria\_none;Bacillota;Bacilli;Bacillales;Planococcaceae;Sporosarcina;Sporosarcina ureilytica |
| Macrococcus armenti | 1 | 0 | 0 | 0 | 0 | 0 | 0 | 0 | 0 | 0 | 0 | 0 | 0 | 0 | Bacteria | Bacteria\_none | Bacillota | Bacilli | Bacillales | Staphylococcaceae | Macrococcus | Bacteria;Bacteria\_none;Bacillota;Bacilli;Bacillales;Staphylococcaceae;Macrococcus;Macrococcus armenti |
| Staphylococcus aureus | 0 | 0 | 0 | 0 | 0 | 0 | 0 | 0 | 0 | 0 | 0 | 1 | 0 | 1 | Bacteria | Bacteria\_none | Bacillota | Bacilli | Bacillales | Staphylococcaceae | Staphylococcus | Bacteria;Bacteria\_none;Bacillota;Bacilli;Bacillales;Staphylococcaceae;Staphylococcus;Staphylococcus aureus |
| Staphylococcus carnosus | 0 | 0 | 0 | 1 | 0 | 0 | 0 | 0 | 0 | 0 | 0 | 0 | 0 | 0 | Bacteria | Bacteria\_none | Bacillota | Bacilli | Bacillales | Staphylococcaceae | Staphylococcus | Bacteria;Bacteria\_none;Bacillota;Bacilli;Bacillales;Staphylococcaceae;Staphylococcus;Staphylococcus carnosus |
| Staphylococcus cohnii | 0 | 0 | 0 | 0 | 0 | 0 | 0 | 0 | 0 | 0 | 0 | 0 | 0 | 0 | Bacteria | Bacteria\_none | Bacillota | Bacilli | Bacillales | Staphylococcaceae | Staphylococcus | Bacteria;Bacteria\_none;Bacillota;Bacilli;Bacillales;Staphylococcaceae;Staphylococcus;Staphylococcus cohnii |
| Staphylococcus delphini | 0 | 0 | 0 | 0 | 0 | 0 | 0 | 0 | 0 | 0 | 0 | 1 | 0 | 0 | Bacteria | Bacteria\_none | Bacillota | Bacilli | Bacillales | Staphylococcaceae | Staphylococcus | Bacteria;Bacteria\_none;Bacillota;Bacilli;Bacillales;Staphylococcaceae;Staphylococcus;Staphylococcus delphini |
| Staphylococcus devriesei | 0 | 0 | 0 | 0 | 0 | 0 | 0 | 0 | 0 | 0 | 0 | 0 | 0 | 0 | Bacteria | Bacteria\_none | Bacillota | Bacilli | Bacillales | Staphylococcaceae | Staphylococcus | Bacteria;Bacteria\_none;Bacillota;Bacilli;Bacillales;Staphylococcaceae;Staphylococcus;Staphylococcus devriesei |
| Staphylococcus epidermidis | 0 | 3 | 0 | 0 | 0 | 0 | 0 | 0 | 0 | 0 | 0 | 0 | 0 | 0 | Bacteria | Bacteria\_none | Bacillota | Bacilli | Bacillales | Staphylococcaceae | Staphylococcus | Bacteria;Bacteria\_none;Bacillota;Bacilli;Bacillales;Staphylococcaceae;Staphylococcus;Staphylococcus epidermidis |
| Staphylococcus felis | 0 | 0 | 0 | 0 | 1 | 0 | 0 | 0 | 0 | 0 | 0 | 0 | 0 | 0 | Bacteria | Bacteria\_none | Bacillota | Bacilli | Bacillales | Staphylococcaceae | Staphylococcus | Bacteria;Bacteria\_none;Bacillota;Bacilli;Bacillales;Staphylococcaceae;Staphylococcus;Staphylococcus felis |
| Staphylococcus hyicus | 0 | 0 | 0 | 1 | 0 | 0 | 0 | 0 | 0 | 0 | 0 | 0 | 0 | 0 | Bacteria | Bacteria\_none | Bacillota | Bacilli | Bacillales | Staphylococcaceae | Staphylococcus | Bacteria;Bacteria\_none;Bacillota;Bacilli;Bacillales;Staphylococcaceae;Staphylococcus;Staphylococcus hyicus |
| Staphylococcus lloydii | 0 | 0 | 0 | 0 | 0 | 0 | 0 | 0 | 0 | 0 | 0 | 0 | 0 | 0 | Bacteria | Bacteria\_none | Bacillota | Bacilli | Bacillales | Staphylococcaceae | Staphylococcus | Bacteria;Bacteria\_none;Bacillota;Bacilli;Bacillales;Staphylococcaceae;Staphylococcus;Staphylococcus lloydii |
| Staphylococcus sp. 17KM0847 | 0 | 0 | 3 | 0 | 0 | 0 | 0 | 0 | 0 | 0 | 0 | 0 | 0 | 0 | Bacteria | Bacteria\_none | Bacillota | Bacilli | Bacillales | Staphylococcaceae | Staphylococcus | Bacteria;Bacteria\_none;Bacillota;Bacilli;Bacillales;Staphylococcaceae;Staphylococcus;Staphylococcus sp. 17KM0847 |
| Staphylococcus sp. MI 10-1553 | 0 | 0 | 0 | 0 | 0 | 0 | 0 | 0 | 0 | 0 | 0 | 0 | 0 | 0 | Bacteria | Bacteria\_none | Bacillota | Bacilli | Bacillales | Staphylococcaceae | Staphylococcus | Bacteria;Bacteria\_none;Bacillota;Bacilli;Bacillales;Staphylococcaceae;Staphylococcus;Staphylococcus sp. MI 10-1553 |
| Staphylococcus succinus | 0 | 0 | 0 | 0 | 0 | 0 | 0 | 0 | 0 | 1 | 0 | 0 | 0 | 0 | Bacteria | Bacteria\_none | Bacillota | Bacilli | Bacillales | Staphylococcaceae | Staphylococcus | Bacteria;Bacteria\_none;Bacillota;Bacilli;Bacillales;Staphylococcaceae;Staphylococcus;Staphylococcus succinus |
| Staphylococcus xylosus | 0 | 0 | 0 | 1 | 0 | 0 | 0 | 0 | 0 | 0 | 0 | 0 | 0 | 0 | Bacteria | Bacteria\_none | Bacillota | Bacilli | Bacillales | Staphylococcaceae | Staphylococcus | Bacteria;Bacteria\_none;Bacillota;Bacilli;Bacillales;Staphylococcaceae;Staphylococcus;Staphylococcus xylosus |
| Abiotrophia defectiva | 6 | 0 | 54 | 2 | 42 | 14 | 8 | 0 | 0 | 10 | 33 | 2 | 11 | 4 | Bacteria | Bacteria\_none | Bacillota | Bacilli | Lactobacillales | Aerococcaceae | Abiotrophia | Bacteria;Bacteria\_none;Bacillota;Bacilli;Lactobacillales;Aerococcaceae;Abiotrophia;Abiotrophia defectiva |
| Aerococcus urinaeequi | 1 | 0 | 0 | 0 | 0 | 0 | 0 | 0 | 0 | 0 | 0 | 0 | 0 | 0 | Bacteria | Bacteria\_none | Bacillota | Bacilli | Lactobacillales | Aerococcaceae | Aerococcus | Bacteria;Bacteria\_none;Bacillota;Bacilli;Lactobacillales;Aerococcaceae;Aerococcus;Aerococcus urinaeequi |
| Ruoffia tabacinasalis | 0 | 1 | 0 | 0 | 0 | 0 | 0 | 0 | 0 | 0 | 0 | 0 | 0 | 0 | Bacteria | Bacteria\_none | Bacillota | Bacilli | Lactobacillales | Aerococcaceae | Ruoffia | Bacteria;Bacteria\_none;Bacillota;Bacilli;Lactobacillales;Aerococcaceae;Ruoffia;Ruoffia tabacinasalis |
| Dolosigranulum pigrum | 1 | 0 | 0 | 0 | 0 | 0 | 0 | 0 | 0 | 0 | 2 | 0 | 0 | 0 | Bacteria | Bacteria\_none | Bacillota | Bacilli | Lactobacillales | Carnobacteriaceae | Dolosigranulum | Bacteria;Bacteria\_none;Bacillota;Bacilli;Lactobacillales;Carnobacteriaceae;Dolosigranulum;Dolosigranulum pigrum |
| Granulicatella adiacens | 384 | 54 | 374 | 10 | 31 | 90 | 180 | 11 | 0 | 75 | 50 | 11 | 48 | 26 | Bacteria | Bacteria\_none | Bacillota | Bacilli | Lactobacillales | Carnobacteriaceae | Granulicatella | Bacteria;Bacteria\_none;Bacillota;Bacilli;Lactobacillales;Carnobacteriaceae;Granulicatella;Granulicatella adiacens |
| Granulicatella elegans | 0 | 11 | 3 | 65 | 108 | 6 | 1 | 0 | 0 | 43 | 14 | 0 | 13 | 5 | Bacteria | Bacteria\_none | Bacillota | Bacilli | Lactobacillales | Carnobacteriaceae | Granulicatella | Bacteria;Bacteria\_none;Bacillota;Bacilli;Lactobacillales;Carnobacteriaceae;Granulicatella;Granulicatella elegans |
| Jeotgalibaca dankookensis | 0 | 0 | 0 | 0 | 0 | 0 | 0 | 0 | 0 | 0 | 0 | 0 | 0 | 0 | Bacteria | Bacteria\_none | Bacillota | Bacilli | Lactobacillales | Carnobacteriaceae | Jeotgalibaca | Bacteria;Bacteria\_none;Bacillota;Bacilli;Lactobacillales;Carnobacteriaceae;Jeotgalibaca;Jeotgalibaca dankookensis |
| Jeotgalibaca porci | 0 | 0 | 0 | 0 | 0 | 0 | 0 | 0 | 0 | 0 | 1 | 0 | 0 | 0 | Bacteria | Bacteria\_none | Bacillota | Bacilli | Lactobacillales | Carnobacteriaceae | Jeotgalibaca | Bacteria;Bacteria\_none;Bacillota;Bacilli;Lactobacillales;Carnobacteriaceae;Jeotgalibaca;Jeotgalibaca porci |
| Enterococcus avium | 0 | 0 | 0 | 0 | 0 | 1 | 0 | 0 | 0 | 0 | 0 | 0 | 0 | 0 | Bacteria | Bacteria\_none | Bacillota | Bacilli | Lactobacillales | Enterococcaceae | Enterococcus | Bacteria;Bacteria\_none;Bacillota;Bacilli;Lactobacillales;Enterococcaceae;Enterococcus;Enterococcus avium |
| Enterococcus cecorum | 0 | 0 | 0 | 0 | 0 | 0 | 1 | 0 | 0 | 0 | 1 | 0 | 0 | 0 | Bacteria | Bacteria\_none | Bacillota | Bacilli | Lactobacillales | Enterococcaceae | Enterococcus | Bacteria;Bacteria\_none;Bacillota;Bacilli;Lactobacillales;Enterococcaceae;Enterococcus;Enterococcus cecorum |
| Enterococcus faecalis | 0 | 2 | 0 | 1 | 3 | 1 | 0 | 0 | 0 | 0 | 0 | 0 | 0 | 0 | Bacteria | Bacteria\_none | Bacillota | Bacilli | Lactobacillales | Enterococcaceae | Enterococcus | Bacteria;Bacteria\_none;Bacillota;Bacilli;Lactobacillales;Enterococcaceae;Enterococcus;Enterococcus faecalis |
| Enterococcus faecium | 1 | 7 | 9 | 0 | 0 | 3 | 0 | 0 | 0 | 0 | 1 | 0 | 2 | 0 | Bacteria | Bacteria\_none | Bacillota | Bacilli | Lactobacillales | Enterococcaceae | Enterococcus | Bacteria;Bacteria\_none;Bacillota;Bacilli;Lactobacillales;Enterococcaceae;Enterococcus;Enterococcus faecium |
| Enterococcus gilvus | 1 | 0 | 0 | 0 | 0 | 0 | 0 | 0 | 0 | 0 | 0 | 0 | 0 | 0 | Bacteria | Bacteria\_none | Bacillota | Bacilli | Lactobacillales | Enterococcaceae | Enterococcus | Bacteria;Bacteria\_none;Bacillota;Bacilli;Lactobacillales;Enterococcaceae;Enterococcus;Enterococcus gilvus |
| Enterococcus mundtii | 0 | 0 | 0 | 0 | 0 | 1 | 0 | 0 | 0 | 0 | 0 | 0 | 0 | 0 | Bacteria | Bacteria\_none | Bacillota | Bacilli | Lactobacillales | Enterococcaceae | Enterococcus | Bacteria;Bacteria\_none;Bacillota;Bacilli;Lactobacillales;Enterococcaceae;Enterococcus;Enterococcus mundtii |
| Enterococcus rotai | 0 | 0 | 0 | 0 | 0 | 0 | 0 | 0 | 0 | 0 | 0 | 0 | 0 | 0 | Bacteria | Bacteria\_none | Bacillota | Bacilli | Lactobacillales | Enterococcaceae | Enterococcus | Bacteria;Bacteria\_none;Bacillota;Bacilli;Lactobacillales;Enterococcaceae;Enterococcus;Enterococcus rotai |
| Enterococcus thailandicus | 0 | 0 | 0 | 0 | 0 | 0 | 0 | 0 | 0 | 0 | 1 | 0 | 0 | 0 | Bacteria | Bacteria\_none | Bacillota | Bacilli | Lactobacillales | Enterococcaceae | Enterococcus | Bacteria;Bacteria\_none;Bacillota;Bacilli;Lactobacillales;Enterococcaceae;Enterococcus;Enterococcus thailandicus |
| Tetragenococcus osmophilus | 0 | 0 | 0 | 0 | 0 | 0 | 0 | 0 | 0 | 0 | 0 | 0 | 0 | 0 | Bacteria | Bacteria\_none | Bacillota | Bacilli | Lactobacillales | Enterococcaceae | Tetragenococcus | Bacteria;Bacteria\_none;Bacillota;Bacilli;Lactobacillales;Enterococcaceae;Tetragenococcus;Tetragenococcus osmophilus |
| Vagococcus carniphilus | 1 | 0 | 1 | 0 | 0 | 0 | 1 | 0 | 0 | 0 | 0 | 0 | 0 | 1 | Bacteria | Bacteria\_none | Bacillota | Bacilli | Lactobacillales | Enterococcaceae | Vagococcus | Bacteria;Bacteria\_none;Bacillota;Bacilli;Lactobacillales;Enterococcaceae;Vagococcus;Vagococcus carniphilus |
| Vagococcus zengguangii | 0 | 1 | 0 | 0 | 0 | 0 | 0 | 0 | 0 | 0 | 0 | 0 | 0 | 0 | Bacteria | Bacteria\_none | Bacillota | Bacilli | Lactobacillales | Enterococcaceae | Vagococcus | Bacteria;Bacteria\_none;Bacillota;Bacilli;Lactobacillales;Enterococcaceae;Vagococcus;Vagococcus zengguangii |
| Lacticaseibacillus paracasei | 0 | 0 | 0 | 0 | 0 | 0 | 0 | 0 | 0 | 0 | 0 | 0 | 0 | 0 | Bacteria | Bacteria\_none | Bacillota | Bacilli | Lactobacillales | Lactobacillaceae | Lacticaseibacillus | Bacteria;Bacteria\_none;Bacillota;Bacilli;Lactobacillales;Lactobacillaceae;Lacticaseibacillus;Lacticaseibacillus paracasei |
| Lacticaseibacillus rhamnosus | 0 | 0 | 0 | 0 | 0 | 0 | 1 | 0 | 0 | 0 | 0 | 0 | 1 | 0 | Bacteria | Bacteria\_none | Bacillota | Bacilli | Lactobacillales | Lactobacillaceae | Lacticaseibacillus | Bacteria;Bacteria\_none;Bacillota;Bacilli;Lactobacillales;Lactobacillaceae;Lacticaseibacillus;Lacticaseibacillus rhamnosus |
| Lactobacillus amylolyticus | 0 | 0 | 0 | 0 | 0 | 0 | 0 | 0 | 0 | 0 | 0 | 0 | 0 | 0 | Bacteria | Bacteria\_none | Bacillota | Bacilli | Lactobacillales | Lactobacillaceae | Lactobacillus | Bacteria;Bacteria\_none;Bacillota;Bacilli;Lactobacillales;Lactobacillaceae;Lactobacillus;Lactobacillus amylolyticus |
| Lactobacillus crispatus | 0 | 0 | 0 | 0 | 0 | 0 | 1 | 0 | 0 | 0 | 0 | 0 | 0 | 0 | Bacteria | Bacteria\_none | Bacillota | Bacilli | Lactobacillales | Lactobacillaceae | Lactobacillus | Bacteria;Bacteria\_none;Bacillota;Bacilli;Lactobacillales;Lactobacillaceae;Lactobacillus;Lactobacillus crispatus |
| Lactobacillus gasseri | 0 | 0 | 0 | 0 | 0 | 0 | 5 | 2 | 0 | 1 | 0 | 0 | 1 | 0 | Bacteria | Bacteria\_none | Bacillota | Bacilli | Lactobacillales | Lactobacillaceae | Lactobacillus | Bacteria;Bacteria\_none;Bacillota;Bacilli;Lactobacillales;Lactobacillaceae;Lactobacillus;Lactobacillus gasseri |
| Lactobacillus iners | 0 | 0 | 0 | 1 | 0 | 0 | 0 | 0 | 0 | 0 | 1 | 0 | 0 | 0 | Bacteria | Bacteria\_none | Bacillota | Bacilli | Lactobacillales | Lactobacillaceae | Lactobacillus | Bacteria;Bacteria\_none;Bacillota;Bacilli;Lactobacillales;Lactobacillaceae;Lactobacillus;Lactobacillus iners |
| Lactobacillus johnsonii | 0 | 0 | 0 | 0 | 0 | 0 | 0 | 0 | 0 | 0 | 0 | 0 | 0 | 0 | Bacteria | Bacteria\_none | Bacillota | Bacilli | Lactobacillales | Lactobacillaceae | Lactobacillus | Bacteria;Bacteria\_none;Bacillota;Bacilli;Lactobacillales;Lactobacillaceae;Lactobacillus;Lactobacillus johnsonii |
| Lactobacillus kefiranofaciens | 0 | 0 | 0 | 0 | 0 | 0 | 0 | 0 | 0 | 0 | 0 | 0 | 0 | 0 | Bacteria | Bacteria\_none | Bacillota | Bacilli | Lactobacillales | Lactobacillaceae | Lactobacillus | Bacteria;Bacteria\_none;Bacillota;Bacilli;Lactobacillales;Lactobacillaceae;Lactobacillus;Lactobacillus kefiranofaciens |
| Lactobacillus paragasseri | 0 | 0 | 0 | 0 | 0 | 0 | 0 | 0 | 0 | 0 | 0 | 0 | 0 | 0 | Bacteria | Bacteria\_none | Bacillota | Bacilli | Lactobacillales | Lactobacillaceae | Lactobacillus | Bacteria;Bacteria\_none;Bacillota;Bacilli;Lactobacillales;Lactobacillaceae;Lactobacillus;Lactobacillus paragasseri |
| Lactobacillus ultunensis | 0 | 0 | 0 | 0 | 0 | 0 | 0 | 0 | 0 | 0 | 0 | 0 | 0 | 0 | Bacteria | Bacteria\_none | Bacillota | Bacilli | Lactobacillales | Lactobacillaceae | Lactobacillus | Bacteria;Bacteria\_none;Bacillota;Bacilli;Lactobacillales;Lactobacillaceae;Lactobacillus;Lactobacillus ultunensis |
| Latilactobacillus graminis | 0 | 0 | 0 | 0 | 0 | 0 | 0 | 0 | 0 | 0 | 0 | 0 | 0 | 0 | Bacteria | Bacteria\_none | Bacillota | Bacilli | Lactobacillales | Lactobacillaceae | Latilactobacillus | Bacteria;Bacteria\_none;Bacillota;Bacilli;Lactobacillales;Lactobacillaceae;Latilactobacillus;Latilactobacillus graminis |
| Latilactobacillus sakei | 0 | 0 | 1 | 0 | 0 | 0 | 0 | 0 | 0 | 0 | 0 | 0 | 0 | 0 | Bacteria | Bacteria\_none | Bacillota | Bacilli | Lactobacillales | Lactobacillaceae | Latilactobacillus | Bacteria;Bacteria\_none;Bacillota;Bacilli;Lactobacillales;Lactobacillaceae;Latilactobacillus;Latilactobacillus sakei |
| Lentilactobacillus buchneri | 0 | 0 | 0 | 0 | 0 | 0 | 0 | 0 | 0 | 1 | 0 | 0 | 0 | 0 | Bacteria | Bacteria\_none | Bacillota | Bacilli | Lactobacillales | Lactobacillaceae | Lentilactobacillus | Bacteria;Bacteria\_none;Bacillota;Bacilli;Lactobacillales;Lactobacillaceae;Lentilactobacillus;Lentilactobacillus buchneri |
| Lentilactobacillus kefiri | 0 | 0 | 0 | 0 | 1 | 0 | 0 | 0 | 0 | 0 | 0 | 0 | 0 | 0 | Bacteria | Bacteria\_none | Bacillota | Bacilli | Lactobacillales | Lactobacillaceae | Lentilactobacillus | Bacteria;Bacteria\_none;Bacillota;Bacilli;Lactobacillales;Lactobacillaceae;Lentilactobacillus;Lentilactobacillus kefiri |
| Leuconostoc falkenbergense | 0 | 0 | 0 | 0 | 0 | 1 | 0 | 0 | 0 | 0 | 0 | 0 | 0 | 0 | Bacteria | Bacteria\_none | Bacillota | Bacilli | Lactobacillales | Lactobacillaceae | Leuconostoc | Bacteria;Bacteria\_none;Bacillota;Bacilli;Lactobacillales;Lactobacillaceae;Leuconostoc;Leuconostoc falkenbergense |
| Leuconostoc kimchii | 0 | 0 | 0 | 1 | 0 | 0 | 0 | 0 | 0 | 0 | 0 | 0 | 0 | 0 | Bacteria | Bacteria\_none | Bacillota | Bacilli | Lactobacillales | Lactobacillaceae | Leuconostoc | Bacteria;Bacteria\_none;Bacillota;Bacilli;Lactobacillales;Lactobacillaceae;Leuconostoc;Leuconostoc kimchii |
| Levilactobacillus brevis | 0 | 0 | 1 | 0 | 0 | 0 | 0 | 0 | 0 | 0 | 0 | 0 | 0 | 0 | Bacteria | Bacteria\_none | Bacillota | Bacilli | Lactobacillales | Lactobacillaceae | Levilactobacillus | Bacteria;Bacteria\_none;Bacillota;Bacilli;Lactobacillales;Lactobacillaceae;Levilactobacillus;Levilactobacillus brevis |
| Ligilactobacillus animalis | 0 | 0 | 0 | 0 | 1 | 0 | 0 | 0 | 0 | 0 | 0 | 0 | 0 | 0 | Bacteria | Bacteria\_none | Bacillota | Bacilli | Lactobacillales | Lactobacillaceae | Ligilactobacillus | Bacteria;Bacteria\_none;Bacillota;Bacilli;Lactobacillales;Lactobacillaceae;Ligilactobacillus;Ligilactobacillus animalis |
| Ligilactobacillus aviarius | 0 | 0 | 0 | 0 | 0 | 0 | 0 | 0 | 0 | 0 | 1 | 0 | 0 | 0 | Bacteria | Bacteria\_none | Bacillota | Bacilli | Lactobacillales | Lactobacillaceae | Ligilactobacillus | Bacteria;Bacteria\_none;Bacillota;Bacilli;Lactobacillales;Lactobacillaceae;Ligilactobacillus;Ligilactobacillus aviarius |
| Ligilactobacillus murinus | 0 | 0 | 0 | 0 | 0 | 0 | 0 | 0 | 0 | 0 | 0 | 0 | 0 | 0 | Bacteria | Bacteria\_none | Bacillota | Bacilli | Lactobacillales | Lactobacillaceae | Ligilactobacillus | Bacteria;Bacteria\_none;Bacillota;Bacilli;Lactobacillales;Lactobacillaceae;Ligilactobacillus;Ligilactobacillus murinus |
| Ligilactobacillus ruminis | 0 | 0 | 0 | 0 | 0 | 0 | 0 | 0 | 1 | 0 | 0 | 0 | 0 | 0 | Bacteria | Bacteria\_none | Bacillota | Bacilli | Lactobacillales | Lactobacillaceae | Ligilactobacillus | Bacteria;Bacteria\_none;Bacillota;Bacilli;Lactobacillales;Lactobacillaceae;Ligilactobacillus;Ligilactobacillus ruminis |
| Ligilactobacillus saerimneri | 0 | 0 | 0 | 0 | 0 | 0 | 0 | 0 | 0 | 0 | 0 | 0 | 0 | 0 | Bacteria | Bacteria\_none | Bacillota | Bacilli | Lactobacillales | Lactobacillaceae | Ligilactobacillus | Bacteria;Bacteria\_none;Bacillota;Bacilli;Lactobacillales;Lactobacillaceae;Ligilactobacillus;Ligilactobacillus saerimneri |
| Ligilactobacillus salivarius | 1 | 0 | 0 | 0 | 0 | 0 | 0 | 0 | 0 | 0 | 0 | 0 | 0 | 0 | Bacteria | Bacteria\_none | Bacillota | Bacilli | Lactobacillales | Lactobacillaceae | Ligilactobacillus | Bacteria;Bacteria\_none;Bacillota;Bacilli;Lactobacillales;Lactobacillaceae;Ligilactobacillus;Ligilactobacillus salivarius |
| Limosilactobacillus portuensis | 0 | 0 | 0 | 0 | 0 | 0 | 0 | 0 | 0 | 0 | 0 | 0 | 0 | 0 | Bacteria | Bacteria\_none | Bacillota | Bacilli | Lactobacillales | Lactobacillaceae | Limosilactobacillus | Bacteria;Bacteria\_none;Bacillota;Bacilli;Lactobacillales;Lactobacillaceae;Limosilactobacillus;Limosilactobacillus portuensis |
| Limosilactobacillus reuteri | 0 | 0 | 0 | 0 | 0 | 0 | 0 | 0 | 0 | 0 | 0 | 0 | 0 | 0 | Bacteria | Bacteria\_none | Bacillota | Bacilli | Lactobacillales | Lactobacillaceae | Limosilactobacillus | Bacteria;Bacteria\_none;Bacillota;Bacilli;Lactobacillales;Lactobacillaceae;Limosilactobacillus;Limosilactobacillus reuteri |
| Limosilactobacillus vaginalis | 0 | 0 | 0 | 0 | 0 | 0 | 0 | 0 | 0 | 0 | 0 | 0 | 0 | 0 | Bacteria | Bacteria\_none | Bacillota | Bacilli | Lactobacillales | Lactobacillaceae | Limosilactobacillus | Bacteria;Bacteria\_none;Bacillota;Bacilli;Lactobacillales;Lactobacillaceae;Limosilactobacillus;Limosilactobacillus vaginalis |
| Liquorilactobacillus hordei | 0 | 0 | 0 | 0 | 0 | 0 | 0 | 0 | 0 | 0 | 1 | 0 | 0 | 0 | Bacteria | Bacteria\_none | Bacillota | Bacilli | Lactobacillales | Lactobacillaceae | Liquorilactobacillus | Bacteria;Bacteria\_none;Bacillota;Bacilli;Lactobacillales;Lactobacillaceae;Liquorilactobacillus;Liquorilactobacillus hordei |
| Liquorilactobacillus nagelii | 0 | 0 | 0 | 0 | 0 | 0 | 0 | 0 | 0 | 0 | 0 | 0 | 0 | 0 | Bacteria | Bacteria\_none | Bacillota | Bacilli | Lactobacillales | Lactobacillaceae | Liquorilactobacillus | Bacteria;Bacteria\_none;Bacillota;Bacilli;Lactobacillales;Lactobacillaceae;Liquorilactobacillus;Liquorilactobacillus nagelii |
| Nicoliella spurrieriana | 0 | 0 | 0 | 0 | 1 | 0 | 0 | 0 | 0 | 0 | 0 | 0 | 0 | 0 | Bacteria | Bacteria\_none | Bacillota | Bacilli | Lactobacillales | Lactobacillaceae | Nicoliella | Bacteria;Bacteria\_none;Bacillota;Bacilli;Lactobacillales;Lactobacillaceae;Nicoliella;Nicoliella spurrieriana |
| Pediococcus acidilactici | 0 | 0 | 1 | 0 | 0 | 0 | 0 | 0 | 0 | 0 | 0 | 0 | 0 | 0 | Bacteria | Bacteria\_none | Bacillota | Bacilli | Lactobacillales | Lactobacillaceae | Pediococcus | Bacteria;Bacteria\_none;Bacillota;Bacilli;Lactobacillales;Lactobacillaceae;Pediococcus;Pediococcus acidilactici |
| Weissella confusa | 0 | 0 | 0 | 0 | 0 | 0 | 0 | 0 | 0 | 0 | 0 | 0 | 0 | 0 | Bacteria | Bacteria\_none | Bacillota | Bacilli | Lactobacillales | Lactobacillaceae | Weissella | Bacteria;Bacteria\_none;Bacillota;Bacilli;Lactobacillales;Lactobacillaceae;Weissella;Weissella confusa |
| Weissella jogaejeotgali | 0 | 0 | 0 | 0 | 0 | 0 | 0 | 0 | 0 | 0 | 0 | 0 | 0 | 0 | Bacteria | Bacteria\_none | Bacillota | Bacilli | Lactobacillales | Lactobacillaceae | Weissella | Bacteria;Bacteria\_none;Bacillota;Bacilli;Lactobacillales;Lactobacillaceae;Weissella;Weissella jogaejeotgali |
| Lactococcus cremoris | 0 | 0 | 0 | 0 | 0 | 1 | 0 | 1 | 0 | 0 | 0 | 0 | 0 | 0 | Bacteria | Bacteria\_none | Bacillota | Bacilli | Lactobacillales | Streptococcaceae | Lactococcus | Bacteria;Bacteria\_none;Bacillota;Bacilli;Lactobacillales;Streptococcaceae;Lactococcus;Lactococcus cremoris |
| Lactococcus garvieae | 0 | 0 | 0 | 0 | 0 | 0 | 0 | 0 | 0 | 1 | 0 | 0 | 0 | 0 | Bacteria | Bacteria\_none | Bacillota | Bacilli | Lactobacillales | Streptococcaceae | Lactococcus | Bacteria;Bacteria\_none;Bacillota;Bacilli;Lactobacillales;Streptococcaceae;Lactococcus;Lactococcus garvieae |
| Lactococcus lactis | 0 | 1 | 0 | 0 | 0 | 0 | 0 | 0 | 0 | 0 | 0 | 0 | 0 | 0 | Bacteria | Bacteria\_none | Bacillota | Bacilli | Lactobacillales | Streptococcaceae | Lactococcus | Bacteria;Bacteria\_none;Bacillota;Bacilli;Lactobacillales;Streptococcaceae;Lactococcus;Lactococcus lactis |
| Lactococcus paracarnosus | 0 | 0 | 0 | 0 | 0 | 0 | 0 | 0 | 0 | 1 | 0 | 0 | 0 | 0 | Bacteria | Bacteria\_none | Bacillota | Bacilli | Lactobacillales | Streptococcaceae | Lactococcus | Bacteria;Bacteria\_none;Bacillota;Bacilli;Lactobacillales;Streptococcaceae;Lactococcus;Lactococcus paracarnosus |
| Lactococcus protaetiae | 0 | 0 | 0 | 0 | 0 | 0 | 0 | 0 | 0 | 0 | 0 | 0 | 0 | 0 | Bacteria | Bacteria\_none | Bacillota | Bacilli | Lactobacillales | Streptococcaceae | Lactococcus | Bacteria;Bacteria\_none;Bacillota;Bacilli;Lactobacillales;Streptococcaceae;Lactococcus;Lactococcus protaetiae |
| Streptococcus acidominimus | 0 | 1 | 0 | 0 | 0 | 1 | 0 | 0 | 0 | 0 | 0 | 0 | 0 | 0 | Bacteria | Bacteria\_none | Bacillota | Bacilli | Lactobacillales | Streptococcaceae | Streptococcus | Bacteria;Bacteria\_none;Bacillota;Bacilli;Lactobacillales;Streptococcaceae;Streptococcus;Streptococcus acidominimus |
| Streptococcus agalactiae | 4 | 9 | 6 | 0 | 12 | 3 | 2 | 0 | 1 | 2 | 0 | 5 | 2 | 1 | Bacteria | Bacteria\_none | Bacillota | Bacilli | Lactobacillales | Streptococcaceae | Streptococcus | Bacteria;Bacteria\_none;Bacillota;Bacilli;Lactobacillales;Streptococcaceae;Streptococcus;Streptococcus agalactiae |
| Streptococcus alactolyticus | 0 | 0 | 0 | 1 | 0 | 0 | 1 | 0 | 0 | 0 | 0 | 0 | 0 | 0 | Bacteria | Bacteria\_none | Bacillota | Bacilli | Lactobacillales | Streptococcaceae | Streptococcus | Bacteria;Bacteria\_none;Bacillota;Bacilli;Lactobacillales;Streptococcaceae;Streptococcus;Streptococcus alactolyticus |
| Streptococcus anginosus | 5 | 15 | 17 | 1 | 10 | 15 | 1 | 14 | 0 | 4 | 17 | 11 | 11 | 1 | Bacteria | Bacteria\_none | Bacillota | Bacilli | Lactobacillales | Streptococcaceae | Streptococcus | Bacteria;Bacteria\_none;Bacillota;Bacilli;Lactobacillales;Streptococcaceae;Streptococcus;Streptococcus anginosus |
| Streptococcus australis | 20 | 19 | 2 | 5 | 13 | 52 | 9 | 2 | 2 | 48 | 11 | 6 | 7 | 9 | Bacteria | Bacteria\_none | Bacillota | Bacilli | Lactobacillales | Streptococcaceae | Streptococcus | Bacteria;Bacteria\_none;Bacillota;Bacilli;Lactobacillales;Streptococcaceae;Streptococcus;Streptococcus australis |
| Streptococcus canis | 0 | 1 | 0 | 0 | 0 | 0 | 0 | 0 | 0 | 0 | 0 | 0 | 0 | 0 | Bacteria | Bacteria\_none | Bacillota | Bacilli | Lactobacillales | Streptococcaceae | Streptococcus | Bacteria;Bacteria\_none;Bacillota;Bacilli;Lactobacillales;Streptococcaceae;Streptococcus;Streptococcus canis |
| Streptococcus chenjunshii | 0 | 0 | 0 | 0 | 0 | 0 | 0 | 0 | 0 | 0 | 0 | 0 | 0 | 0 | Bacteria | Bacteria\_none | Bacillota | Bacilli | Lactobacillales | Streptococcaceae | Streptococcus | Bacteria;Bacteria\_none;Bacillota;Bacilli;Lactobacillales;Streptococcaceae;Streptococcus;Streptococcus chenjunshii |
| Streptococcus constellatus | 0 | 5 | 9 | 0 | 1 | 9 | 1 | 9 | 0 | 1 | 42 | 1 | 8 | 10 | Bacteria | Bacteria\_none | Bacillota | Bacilli | Lactobacillales | Streptococcaceae | Streptococcus | Bacteria;Bacteria\_none;Bacillota;Bacilli;Lactobacillales;Streptococcaceae;Streptococcus;Streptococcus constellatus |
| Streptococcus cristatus | 2 | 24 | 19 | 6 | 17 | 39 | 4 | 0 | 0 | 41 | 6 | 19 | 9 | 7 | Bacteria | Bacteria\_none | Bacillota | Bacilli | Lactobacillales | Streptococcaceae | Streptococcus | Bacteria;Bacteria\_none;Bacillota;Bacilli;Lactobacillales;Streptococcaceae;Streptococcus;Streptococcus cristatus |
| Streptococcus didelphis | 0 | 0 | 1 | 0 | 0 | 0 | 0 | 0 | 0 | 1 | 0 | 1 | 0 | 0 | Bacteria | Bacteria\_none | Bacillota | Bacilli | Lactobacillales | Streptococcaceae | Streptococcus | Bacteria;Bacteria\_none;Bacillota;Bacilli;Lactobacillales;Streptococcaceae;Streptococcus;Streptococcus didelphis |
| Streptococcus dysgalactiae | 1 | 1 | 1 | 0 | 0 | 2 | 0 | 0 | 5 | 0 | 1 | 0 | 1 | 0 | Bacteria | Bacteria\_none | Bacillota | Bacilli | Lactobacillales | Streptococcaceae | Streptococcus | Bacteria;Bacteria\_none;Bacillota;Bacilli;Lactobacillales;Streptococcaceae;Streptococcus;Streptococcus dysgalactiae |
| Streptococcus equi | 0 | 2 | 0 | 0 | 3 | 2 | 0 | 1 | 0 | 0 | 1 | 1 | 1 | 0 | Bacteria | Bacteria\_none | Bacillota | Bacilli | Lactobacillales | Streptococcaceae | Streptococcus | Bacteria;Bacteria\_none;Bacillota;Bacilli;Lactobacillales;Streptococcaceae;Streptococcus;Streptococcus equi |
| Streptococcus equinus | 0 | 0 | 0 | 0 | 1 | 0 | 0 | 0 | 0 | 0 | 1 | 0 | 0 | 0 | Bacteria | Bacteria\_none | Bacillota | Bacilli | Lactobacillales | Streptococcaceae | Streptococcus | Bacteria;Bacteria\_none;Bacillota;Bacilli;Lactobacillales;Streptococcaceae;Streptococcus;Streptococcus equinus |
| Streptococcus ferus | 0 | 0 | 1 | 0 | 0 | 0 | 0 | 0 | 0 | 0 | 0 | 0 | 0 | 0 | Bacteria | Bacteria\_none | Bacillota | Bacilli | Lactobacillales | Streptococcaceae | Streptococcus | Bacteria;Bacteria\_none;Bacillota;Bacilli;Lactobacillales;Streptococcaceae;Streptococcus;Streptococcus ferus |
| Streptococcus gallolyticus | 0 | 0 | 0 | 0 | 0 | 0 | 0 | 0 | 0 | 0 | 0 | 0 | 0 | 0 | Bacteria | Bacteria\_none | Bacillota | Bacilli | Lactobacillales | Streptococcaceae | Streptococcus | Bacteria;Bacteria\_none;Bacillota;Bacilli;Lactobacillales;Streptococcaceae;Streptococcus;Streptococcus gallolyticus |
| Streptococcus gordonii | 21 | 11 | 5 | 1 | 7 | 16 | 60 | 1 | 4 | 12 | 44 | 25 | 9 | 10 | Bacteria | Bacteria\_none | Bacillota | Bacilli | Lactobacillales | Streptococcaceae | Streptococcus | Bacteria;Bacteria\_none;Bacillota;Bacilli;Lactobacillales;Streptococcaceae;Streptococcus;Streptococcus gordonii |
| Streptococcus gwangjuense | 2 | 12 | 0 | 6 | 30 | 7 | 3 | 0 | 0 | 16 | 7 | 4 | 2 | 5 | Bacteria | Bacteria\_none | Bacillota | Bacilli | Lactobacillales | Streptococcaceae | Streptococcus | Bacteria;Bacteria\_none;Bacillota;Bacilli;Lactobacillales;Streptococcaceae;Streptococcus;Streptococcus gwangjuense |
| Streptococcus halichoeri | 0 | 0 | 0 | 0 | 0 | 0 | 0 | 0 | 0 | 0 | 0 | 0 | 0 | 0 | Bacteria | Bacteria\_none | Bacillota | Bacilli | Lactobacillales | Streptococcaceae | Streptococcus | Bacteria;Bacteria\_none;Bacillota;Bacilli;Lactobacillales;Streptococcaceae;Streptococcus;Streptococcus halichoeri |
| Streptococcus halotolerans | 0 | 0 | 0 | 0 | 0 | 1 | 0 | 0 | 0 | 0 | 0 | 0 | 0 | 0 | Bacteria | Bacteria\_none | Bacillota | Bacilli | Lactobacillales | Streptococcaceae | Streptococcus | Bacteria;Bacteria\_none;Bacillota;Bacilli;Lactobacillales;Streptococcaceae;Streptococcus;Streptococcus halotolerans |
| Streptococcus himalayensis | 1 | 0 | 0 | 0 | 0 | 0 | 0 | 0 | 0 | 0 | 1 | 0 | 0 | 0 | Bacteria | Bacteria\_none | Bacillota | Bacilli | Lactobacillales | Streptococcaceae | Streptococcus | Bacteria;Bacteria\_none;Bacillota;Bacilli;Lactobacillales;Streptococcaceae;Streptococcus;Streptococcus himalayensis |
| Streptococcus ilei | 6 | 12 | 1 | 0 | 1 | 24 | 1 | 0 | 0 | 8 | 7 | 0 | 5 | 5 | Bacteria | Bacteria\_none | Bacillota | Bacilli | Lactobacillales | Streptococcaceae | Streptococcus | Bacteria;Bacteria\_none;Bacillota;Bacilli;Lactobacillales;Streptococcaceae;Streptococcus;Streptococcus ilei |
| Streptococcus infantarius | 0 | 1 | 0 | 0 | 0 | 0 | 0 | 0 | 0 | 0 | 0 | 0 | 0 | 0 | Bacteria | Bacteria\_none | Bacillota | Bacilli | Lactobacillales | Streptococcaceae | Streptococcus | Bacteria;Bacteria\_none;Bacillota;Bacilli;Lactobacillales;Streptococcaceae;Streptococcus;Streptococcus infantarius |
| Streptococcus infantis | 33 | 10 | 28 | 12 | 4 | 20 | 4 | 0 | 1 | 1 | 26 | 2 | 10 | 4 | Bacteria | Bacteria\_none | Bacillota | Bacilli | Lactobacillales | Streptococcaceae | Streptococcus | Bacteria;Bacteria\_none;Bacillota;Bacilli;Lactobacillales;Streptococcaceae;Streptococcus;Streptococcus infantis |
| Streptococcus iniae | 0 | 1 | 0 | 0 | 0 | 0 | 0 | 0 | 0 | 0 | 1 | 0 | 0 | 0 | Bacteria | Bacteria\_none | Bacillota | Bacilli | Lactobacillales | Streptococcaceae | Streptococcus | Bacteria;Bacteria\_none;Bacillota;Bacilli;Lactobacillales;Streptococcaceae;Streptococcus;Streptococcus iniae |
| Streptococcus intermedius | 1 | 5 | 4 | 0 | 2 | 2 | 0 | 2 | 0 | 3 | 96 | 2 | 7 | 6 | Bacteria | Bacteria\_none | Bacillota | Bacilli | Lactobacillales | Streptococcaceae | Streptococcus | Bacteria;Bacteria\_none;Bacillota;Bacilli;Lactobacillales;Streptococcaceae;Streptococcus;Streptococcus intermedius |
| Streptococcus koreensis | 7 | 4 | 2 | 0 | 1 | 12 | 3 | 0 | 1 | 15 | 1 | 0 | 3 | 1 | Bacteria | Bacteria\_none | Bacillota | Bacilli | Lactobacillales | Streptococcaceae | Streptococcus | Bacteria;Bacteria\_none;Bacillota;Bacilli;Lactobacillales;Streptococcaceae;Streptococcus;Streptococcus koreensis |
| Streptococcus lactarius | 9 | 5 | 1 | 0 | 1 | 5 | 5 | 0 | 7 | 3 | 0 | 4 | 1 | 1 | Bacteria | Bacteria\_none | Bacillota | Bacilli | Lactobacillales | Streptococcaceae | Streptococcus | Bacteria;Bacteria\_none;Bacillota;Bacilli;Lactobacillales;Streptococcaceae;Streptococcus;Streptococcus lactarius |
| Streptococcus lutetiensis | 0 | 0 | 0 | 0 | 0 | 0 | 0 | 0 | 0 | 0 | 0 | 0 | 0 | 0 | Bacteria | Bacteria\_none | Bacillota | Bacilli | Lactobacillales | Streptococcaceae | Streptococcus | Bacteria;Bacteria\_none;Bacillota;Bacilli;Lactobacillales;Streptococcaceae;Streptococcus;Streptococcus lutetiensis |
| Streptococcus merionis | 0 | 1 | 1 | 0 | 0 | 0 | 0 | 0 | 1 | 0 | 0 | 0 | 0 | 0 | Bacteria | Bacteria\_none | Bacillota | Bacilli | Lactobacillales | Streptococcaceae | Streptococcus | Bacteria;Bacteria\_none;Bacillota;Bacilli;Lactobacillales;Streptococcaceae;Streptococcus;Streptococcus merionis |
| Streptococcus milleri | 0 | 0 | 0 | 0 | 3 | 0 | 1 | 1 | 0 | 0 | 11 | 0 | 3 | 1 | Bacteria | Bacteria\_none | Bacillota | Bacilli | Lactobacillales | Streptococcaceae | Streptococcus | Bacteria;Bacteria\_none;Bacillota;Bacilli;Lactobacillales;Streptococcaceae;Streptococcus;Streptococcus milleri |
| Streptococcus mitis | 55 | 137 | 24 | 55 | 548 | 116 | 27 | 2 | 4 | 120 | 261 | 45 | 59 | 39 | Bacteria | Bacteria\_none | Bacillota | Bacilli | Lactobacillales | Streptococcaceae | Streptococcus | Bacteria;Bacteria\_none;Bacillota;Bacilli;Lactobacillales;Streptococcaceae;Streptococcus;Streptococcus mitis |
| Streptococcus mutans | 0 | 1 | 1 | 0 | 2 | 1 | 0 | 0 | 0 | 0 | 1 | 0 | 0 | 1 | Bacteria | Bacteria\_none | Bacillota | Bacilli | Lactobacillales | Streptococcaceae | Streptococcus | Bacteria;Bacteria\_none;Bacillota;Bacilli;Lactobacillales;Streptococcaceae;Streptococcus;Streptococcus mutans |
| Streptococcus oralis | 541 | 179 | 145 | 16 | 139 | 135 | 281 | 26 | 1 | 33 | 172 | 115 | 112 | 63 | Bacteria | Bacteria\_none | Bacillota | Bacilli | Lactobacillales | Streptococcaceae | Streptococcus | Bacteria;Bacteria\_none;Bacillota;Bacilli;Lactobacillales;Streptococcaceae;Streptococcus;Streptococcus oralis |
| Streptococcus oriscaviae | 1 | 0 | 0 | 0 | 0 | 0 | 1 | 0 | 0 | 0 | 1 | 0 | 1 | 0 | Bacteria | Bacteria\_none | Bacillota | Bacilli | Lactobacillales | Streptococcaceae | Streptococcus | Bacteria;Bacteria\_none;Bacillota;Bacilli;Lactobacillales;Streptococcaceae;Streptococcus;Streptococcus oriscaviae |
| Streptococcus pantholopis | 0 | 0 | 1 | 0 | 0 | 0 | 0 | 0 | 0 | 1 | 0 | 0 | 0 | 0 | Bacteria | Bacteria\_none | Bacillota | Bacilli | Lactobacillales | Streptococcaceae | Streptococcus | Bacteria;Bacteria\_none;Bacillota;Bacilli;Lactobacillales;Streptococcaceae;Streptococcus;Streptococcus pantholopis |
| Streptococcus parapneumoniae | 6 | 13 | 0 | 10 | 52 | 7 | 3 | 0 | 0 | 14 | 8 | 6 | 8 | 2 | Bacteria | Bacteria\_none | Bacillota | Bacilli | Lactobacillales | Streptococcaceae | Streptococcus | Bacteria;Bacteria\_none;Bacillota;Bacilli;Lactobacillales;Streptococcaceae;Streptococcus;Streptococcus parapneumoniae |
| Streptococcus parasanguinis | 193 | 230 | 133 | 0 | 2 | 130 | 111 | 3 | 102 | 16 | 4 | 69 | 44 | 35 | Bacteria | Bacteria\_none | Bacillota | Bacilli | Lactobacillales | Streptococcaceae | Streptococcus | Bacteria;Bacteria\_none;Bacillota;Bacilli;Lactobacillales;Streptococcaceae;Streptococcus;Streptococcus parasanguinis |
| Streptococcus parasuis | 1 | 2 | 0 | 0 | 0 | 1 | 0 | 0 | 0 | 1 | 0 | 0 | 0 | 0 | Bacteria | Bacteria\_none | Bacillota | Bacilli | Lactobacillales | Streptococcaceae | Streptococcus | Bacteria;Bacteria\_none;Bacillota;Bacilli;Lactobacillales;Streptococcaceae;Streptococcus;Streptococcus parasuis |
| Streptococcus parauberis | 0 | 0 | 0 | 0 | 0 | 1 | 0 | 0 | 0 | 1 | 0 | 0 | 0 | 0 | Bacteria | Bacteria\_none | Bacillota | Bacilli | Lactobacillales | Streptococcaceae | Streptococcus | Bacteria;Bacteria\_none;Bacillota;Bacilli;Lactobacillales;Streptococcaceae;Streptococcus;Streptococcus parauberis |
| Streptococcus pasteurianus | 1 | 0 | 0 | 0 | 0 | 0 | 0 | 0 | 0 | 1 | 0 | 0 | 0 | 0 | Bacteria | Bacteria\_none | Bacillota | Bacilli | Lactobacillales | Streptococcaceae | Streptococcus | Bacteria;Bacteria\_none;Bacillota;Bacilli;Lactobacillales;Streptococcaceae;Streptococcus;Streptococcus pasteurianus |
| Streptococcus periodonticum | 1 | 0 | 0 | 0 | 0 | 0 | 0 | 1 | 0 | 0 | 2 | 1 | 0 | 0 | Bacteria | Bacteria\_none | Bacillota | Bacilli | Lactobacillales | Streptococcaceae | Streptococcus | Bacteria;Bacteria\_none;Bacillota;Bacilli;Lactobacillales;Streptococcaceae;Streptococcus;Streptococcus periodonticum |
| Streptococcus pluranimalium | 0 | 0 | 0 | 1 | 0 | 0 | 0 | 0 | 0 | 0 | 0 | 1 | 1 | 0 | Bacteria | Bacteria\_none | Bacillota | Bacilli | Lactobacillales | Streptococcaceae | Streptococcus | Bacteria;Bacteria\_none;Bacillota;Bacilli;Lactobacillales;Streptococcaceae;Streptococcus;Streptococcus pluranimalium |
| Streptococcus pneumoniae | 57 | 66 | 10 | 54 | 216 | 42 | 14 | 2 | 2 | 67 | 71 | 22 | 30 | 9 | Bacteria | Bacteria\_none | Bacillota | Bacilli | Lactobacillales | Streptococcaceae | Streptococcus | Bacteria;Bacteria\_none;Bacillota;Bacilli;Lactobacillales;Streptococcaceae;Streptococcus;Streptococcus pneumoniae |
| Streptococcus porcinus | 0 | 0 | 0 | 1 | 1 | 2 | 0 | 0 | 0 | 0 | 1 | 0 | 0 | 0 | Bacteria | Bacteria\_none | Bacillota | Bacilli | Lactobacillales | Streptococcaceae | Streptococcus | Bacteria;Bacteria\_none;Bacillota;Bacilli;Lactobacillales;Streptococcaceae;Streptococcus;Streptococcus porcinus |
| Streptococcus pseudopneumoniae | 3 | 5 | 1 | 4 | 44 | 8 | 1 | 0 | 1 | 12 | 6 | 4 | 6 | 1 | Bacteria | Bacteria\_none | Bacillota | Bacilli | Lactobacillales | Streptococcaceae | Streptococcus | Bacteria;Bacteria\_none;Bacillota;Bacilli;Lactobacillales;Streptococcaceae;Streptococcus;Streptococcus pseudopneumoniae |
| Streptococcus pseudoporcinus | 0 | 1 | 0 | 0 | 0 | 0 | 0 | 1 | 0 | 0 | 0 | 0 | 0 | 0 | Bacteria | Bacteria\_none | Bacillota | Bacilli | Lactobacillales | Streptococcaceae | Streptococcus | Bacteria;Bacteria\_none;Bacillota;Bacilli;Lactobacillales;Streptococcaceae;Streptococcus;Streptococcus pseudoporcinus |
| Streptococcus pyogenes | 0 | 2 | 1 | 2 | 1 | 4 | 1 | 0 | 1 | 1 | 4 | 1 | 1 | 4 | Bacteria | Bacteria\_none | Bacillota | Bacilli | Lactobacillales | Streptococcaceae | Streptococcus | Bacteria;Bacteria\_none;Bacillota;Bacilli;Lactobacillales;Streptococcaceae;Streptococcus;Streptococcus pyogenes |
| Streptococcus ratti | 0 | 0 | 1 | 0 | 0 | 0 | 1 | 0 | 0 | 0 | 0 | 0 | 0 | 0 | Bacteria | Bacteria\_none | Bacillota | Bacilli | Lactobacillales | Streptococcaceae | Streptococcus | Bacteria;Bacteria\_none;Bacillota;Bacilli;Lactobacillales;Streptococcaceae;Streptococcus;Streptococcus ratti |
| Streptococcus respiraculi | 0 | 1 | 0 | 0 | 0 | 0 | 0 | 0 | 0 | 0 | 2 | 0 | 1 | 0 | Bacteria | Bacteria\_none | Bacillota | Bacilli | Lactobacillales | Streptococcaceae | Streptococcus | Bacteria;Bacteria\_none;Bacillota;Bacilli;Lactobacillales;Streptococcaceae;Streptococcus;Streptococcus respiraculi |
| Streptococcus rubneri | 6 | 2 | 1 | 0 | 3 | 16 | 0 | 0 | 0 | 3 | 2 | 1 | 2 | 2 | Bacteria | Bacteria\_none | Bacillota | Bacilli | Lactobacillales | Streptococcaceae | Streptococcus | Bacteria;Bacteria\_none;Bacillota;Bacilli;Lactobacillales;Streptococcaceae;Streptococcus;Streptococcus rubneri |
| Streptococcus ruminantium | 0 | 0 | 0 | 0 | 0 | 0 | 0 | 0 | 0 | 0 | 0 | 0 | 0 | 0 | Bacteria | Bacteria\_none | Bacillota | Bacilli | Lactobacillales | Streptococcaceae | Streptococcus | Bacteria;Bacteria\_none;Bacillota;Bacilli;Lactobacillales;Streptococcaceae;Streptococcus;Streptococcus ruminantium |
| Streptococcus salivarius | 7 | 16 | 2 | 0 | 6 | 28 | 13 | 0 | 0 | 4 | 2 | 6 | 4 | 3 | Bacteria | Bacteria\_none | Bacillota | Bacilli | Lactobacillales | Streptococcaceae | Streptococcus | Bacteria;Bacteria\_none;Bacillota;Bacilli;Lactobacillales;Streptococcaceae;Streptococcus;Streptococcus salivarius |
| Streptococcus sanguinis | 73 | 18 | 62 | 3 | 72 | 67 | 46 | 14 | 1 | 11 | 173 | 3 | 27 | 24 | Bacteria | Bacteria\_none | Bacillota | Bacilli | Lactobacillales | Streptococcaceae | Streptococcus | Bacteria;Bacteria\_none;Bacillota;Bacilli;Lactobacillales;Streptococcaceae;Streptococcus;Streptococcus sanguinis |
| Streptococcus sp. 116-D4 | 3 | 18 | 0 | 3 | 34 | 11 | 1 | 0 | 0 | 8 | 21 | 16 | 5 | 3 | Bacteria | Bacteria\_none | Bacillota | Bacilli | Lactobacillales | Streptococcaceae | Streptococcus | Bacteria;Bacteria\_none;Bacillota;Bacilli;Lactobacillales;Streptococcaceae;Streptococcus;Streptococcus sp. 116-D4 |
| Streptococcus sp. 1643 | 33 | 2 | 6 | 2 | 4 | 5 | 17 | 0 | 0 | 3 | 6 | 0 | 4 | 6 | Bacteria | Bacteria\_none | Bacillota | Bacilli | Lactobacillales | Streptococcaceae | Streptococcus | Bacteria;Bacteria\_none;Bacillota;Bacilli;Lactobacillales;Streptococcaceae;Streptococcus;Streptococcus sp. 1643 |
| Streptococcus sp. 29887 | 0 | 0 | 0 | 0 | 0 | 0 | 0 | 0 | 0 | 0 | 1 | 0 | 0 | 0 | Bacteria | Bacteria\_none | Bacillota | Bacilli | Lactobacillales | Streptococcaceae | Streptococcus | Bacteria;Bacteria\_none;Bacillota;Bacilli;Lactobacillales;Streptococcaceae;Streptococcus;Streptococcus sp. 29887 |
| Streptococcus sp. 29892 | 0 | 0 | 0 | 0 | 0 | 0 | 0 | 0 | 0 | 0 | 0 | 0 | 0 | 0 | Bacteria | Bacteria\_none | Bacillota | Bacilli | Lactobacillales | Streptococcaceae | Streptococcus | Bacteria;Bacteria\_none;Bacillota;Bacilli;Lactobacillales;Streptococcaceae;Streptococcus;Streptococcus sp. 29892 |
| Streptococcus sp. 29896 | 0 | 0 | 0 | 0 | 0 | 0 | 0 | 0 | 0 | 0 | 0 | 0 | 0 | 0 | Bacteria | Bacteria\_none | Bacillota | Bacilli | Lactobacillales | Streptococcaceae | Streptococcus | Bacteria;Bacteria\_none;Bacillota;Bacilli;Lactobacillales;Streptococcaceae;Streptococcus;Streptococcus sp. 29896 |
| Streptococcus sp. A12 | 19 | 7 | 0 | 0 | 7 | 57 | 1 | 0 | 2 | 27 | 8 | 1 | 3 | 4 | Bacteria | Bacteria\_none | Bacillota | Bacilli | Lactobacillales | Streptococcaceae | Streptococcus | Bacteria;Bacteria\_none;Bacillota;Bacilli;Lactobacillales;Streptococcaceae;Streptococcus;Streptococcus sp. A12 |
| Streptococcus sp. D7B5 | 8 | 3 | 30 | 0 | 4 | 13 | 7 | 0 | 0 | 0 | 2 | 1 | 1 | 4 | Bacteria | Bacteria\_none | Bacillota | Bacilli | Lactobacillales | Streptococcaceae | Streptococcus | Bacteria;Bacteria\_none;Bacillota;Bacilli;Lactobacillales;Streptococcaceae;Streptococcus;Streptococcus sp. D7B5 |
| Streptococcus sp. DTU\_2020\_1000888\_1\_SI\_GRL\_NUU\_041A | 6 | 1 | 4 | 0 | 4 | 3 | 1 | 1 | 0 | 0 | 10 | 0 | 1 | 2 | Bacteria | Bacteria\_none | Bacillota | Bacilli | Lactobacillales | Streptococcaceae | Streptococcus | Bacteria;Bacteria\_none;Bacillota;Bacilli;Lactobacillales;Streptococcaceae;Streptococcus;Streptococcus sp. DTU\_2020\_1000888\_1\_SI\_GRL\_NUU\_041A |
| Streptococcus sp. DTU\_2020\_1001019\_1\_SI\_AUS\_MUR\_006 | 76 | 49 | 12 | 1 | 28 | 69 | 13 | 4 | 1 | 7 | 21 | 3 | 16 | 14 | Bacteria | Bacteria\_none | Bacillota | Bacilli | Lactobacillales | Streptococcaceae | Streptococcus | Bacteria;Bacteria\_none;Bacillota;Bacilli;Lactobacillales;Streptococcaceae;Streptococcus;Streptococcus sp. DTU\_2020\_1001019\_1\_SI\_AUS\_MUR\_006 |
| Streptococcus sp. FDAARGOS\_192 | 0 | 0 | 0 | 0 | 1 | 0 | 0 | 0 | 0 | 0 | 0 | 0 | 0 | 1 | Bacteria | Bacteria\_none | Bacillota | Bacilli | Lactobacillales | Streptococcaceae | Streptococcus | Bacteria;Bacteria\_none;Bacillota;Bacilli;Lactobacillales;Streptococcaceae;Streptococcus;Streptococcus sp. FDAARGOS\_192 |
| Streptococcus sp. FDAARGOS\_522 | 0 | 0 | 0 | 0 | 0 | 0 | 4 | 0 | 6 | 0 | 0 | 0 | 3 | 0 | Bacteria | Bacteria\_none | Bacillota | Bacilli | Lactobacillales | Streptococcaceae | Streptococcus | Bacteria;Bacteria\_none;Bacillota;Bacilli;Lactobacillales;Streptococcaceae;Streptococcus;Streptococcus sp. FDAARGOS\_522 |
| Streptococcus sp. FSL K6-1323 | 0 | 2 | 0 | 0 | 0 | 1 | 0 | 0 | 0 | 0 | 0 | 0 | 0 | 0 | Bacteria | Bacteria\_none | Bacillota | Bacilli | Lactobacillales | Streptococcaceae | Streptococcus | Bacteria;Bacteria\_none;Bacillota;Bacilli;Lactobacillales;Streptococcaceae;Streptococcus;Streptococcus sp. FSL K6-1323 |
| Streptococcus sp. FSL R7-0248 | 0 | 0 | 0 | 0 | 0 | 0 | 5 | 0 | 0 | 0 | 0 | 0 | 0 | 0 | Bacteria | Bacteria\_none | Bacillota | Bacilli | Lactobacillales | Streptococcaceae | Streptococcus | Bacteria;Bacteria\_none;Bacillota;Bacilli;Lactobacillales;Streptococcaceae;Streptococcus;Streptococcus sp. FSL R7-0248 |
| Streptococcus sp. FSL W7-1342 | 0 | 0 | 0 | 0 | 0 | 5 | 0 | 0 | 0 | 0 | 0 | 0 | 0 | 0 | Bacteria | Bacteria\_none | Bacillota | Bacilli | Lactobacillales | Streptococcaceae | Streptococcus | Bacteria;Bacteria\_none;Bacillota;Bacilli;Lactobacillales;Streptococcaceae;Streptococcus;Streptococcus sp. FSL W7-1342 |
| Streptococcus sp. FSL W8-0197 | 21 | 32 | 8 | 0 | 0 | 4 | 4 | 8 | 0 | 0 | 6 | 1 | 5 | 7 | Bacteria | Bacteria\_none | Bacillota | Bacilli | Lactobacillales | Streptococcaceae | Streptococcus | Bacteria;Bacteria\_none;Bacillota;Bacilli;Lactobacillales;Streptococcaceae;Streptococcus;Streptococcus sp. FSL W8-0197 |
| Streptococcus sp. KS 6 | 0 | 0 | 0 | 0 | 0 | 0 | 0 | 0 | 0 | 0 | 0 | 0 | 0 | 0 | Bacteria | Bacteria\_none | Bacillota | Bacilli | Lactobacillales | Streptococcaceae | Streptococcus | Bacteria;Bacteria\_none;Bacillota;Bacilli;Lactobacillales;Streptococcaceae;Streptococcus;Streptococcus sp. KS 6 |
| Streptococcus sp. LPB0220 | 36 | 44 | 28 | 0 | 3 | 23 | 13 | 0 | 29 | 3 | 2 | 7 | 8 | 5 | Bacteria | Bacteria\_none | Bacillota | Bacilli | Lactobacillales | Streptococcaceae | Streptococcus | Bacteria;Bacteria\_none;Bacillota;Bacilli;Lactobacillales;Streptococcaceae;Streptococcus;Streptococcus sp. LPB0220 |
| Streptococcus sp. Marseille-Q3533 | 60 | 43 | 8 | 3 | 40 | 76 | 6 | 2 | 0 | 1 | 19 | 4 | 11 | 8 | Bacteria | Bacteria\_none | Bacillota | Bacilli | Lactobacillales | Streptococcaceae | Streptococcus | Bacteria;Bacteria\_none;Bacillota;Bacilli;Lactobacillales;Streptococcaceae;Streptococcus;Streptococcus sp. Marseille-Q3533 |
| Streptococcus sp. Marseille-Q6470 | 64 | 75 | 8 | 6 | 16 | 38 | 4 | 0 | 2 | 1 | 34 | 2 | 14 | 3 | Bacteria | Bacteria\_none | Bacillota | Bacilli | Lactobacillales | Streptococcaceae | Streptococcus | Bacteria;Bacteria\_none;Bacillota;Bacilli;Lactobacillales;Streptococcaceae;Streptococcus;Streptococcus sp. Marseille-Q6470 |
| Streptococcus sp. NCTC 11567 | 0 | 0 | 0 | 0 | 4 | 0 | 0 | 0 | 0 | 0 | 0 | 0 | 0 | 0 | Bacteria | Bacteria\_none | Bacillota | Bacilli | Lactobacillales | Streptococcaceae | Streptococcus | Bacteria;Bacteria\_none;Bacillota;Bacilli;Lactobacillales;Streptococcaceae;Streptococcus;Streptococcus sp. NCTC 11567 |
| Streptococcus sp. NPS 308 | 6 | 2 | 5 | 2 | 27 | 1 | 6 | 2 | 0 | 1 | 12 | 4 | 9 | 3 | Bacteria | Bacteria\_none | Bacillota | Bacilli | Lactobacillales | Streptococcaceae | Streptococcus | Bacteria;Bacteria\_none;Bacillota;Bacilli;Lactobacillales;Streptococcaceae;Streptococcus;Streptococcus sp. NPS 308 |
| Streptococcus sp. NSJ-72 | 2 | 3 | 0 | 0 | 2 | 0 | 0 | 6 | 0 | 0 | 12 | 2 | 4 | 1 | Bacteria | Bacteria\_none | Bacillota | Bacilli | Lactobacillales | Streptococcaceae | Streptococcus | Bacteria;Bacteria\_none;Bacillota;Bacilli;Lactobacillales;Streptococcaceae;Streptococcus;Streptococcus sp. NSJ-72 |
| Streptococcus sp. S1 | 17 | 19 | 12 | 0 | 2 | 19 | 14 | 1 | 16 | 5 | 2 | 9 | 5 | 5 | Bacteria | Bacteria\_none | Bacillota | Bacilli | Lactobacillales | Streptococcaceae | Streptococcus | Bacteria;Bacteria\_none;Bacillota;Bacilli;Lactobacillales;Streptococcaceae;Streptococcus;Streptococcus sp. S1 |
| Streptococcus sp. S5 | 44 | 51 | 33 | 0 | 0 | 16 | 9 | 0 | 26 | 3 | 0 | 13 | 12 | 14 | Bacteria | Bacteria\_none | Bacillota | Bacilli | Lactobacillales | Streptococcaceae | Streptococcus | Bacteria;Bacteria\_none;Bacillota;Bacilli;Lactobacillales;Streptococcaceae;Streptococcus;Streptococcus sp. S5 |
| Streptococcus sp. oral taxon 061 | 48 | 41 | 10 | 5 | 5 | 56 | 6 | 0 | 0 | 0 | 24 | 2 | 7 | 7 | Bacteria | Bacteria\_none | Bacillota | Bacilli | Lactobacillales | Streptococcaceae | Streptococcus | Bacteria;Bacteria\_none;Bacillota;Bacilli;Lactobacillales;Streptococcaceae;Streptococcus;Streptococcus sp. oral taxon 061 |
| Streptococcus sp. oral taxon 064 | 31 | 8 | 0 | 2 | 0 | 4 | 24 | 0 | 0 | 0 | 2 | 0 | 7 | 10 | Bacteria | Bacteria\_none | Bacillota | Bacilli | Lactobacillales | Streptococcaceae | Streptococcus | Bacteria;Bacteria\_none;Bacillota;Bacilli;Lactobacillales;Streptococcaceae;Streptococcus;Streptococcus sp. oral taxon 064 |
| Streptococcus sp. oral taxon 431 | 86 | 63 | 4 | 2 | 32 | 131 | 11 | 3 | 0 | 4 | 30 | 1 | 16 | 15 | Bacteria | Bacteria\_none | Bacillota | Bacilli | Lactobacillales | Streptococcaceae | Streptococcus | Bacteria;Bacteria\_none;Bacillota;Bacilli;Lactobacillales;Streptococcaceae;Streptococcus;Streptococcus sp. oral taxon 431 |
| Streptococcus sp. zg-86 | 0 | 0 | 0 | 0 | 0 | 0 | 0 | 0 | 0 | 0 | 1 | 1 | 0 | 0 | Bacteria | Bacteria\_none | Bacillota | Bacilli | Lactobacillales | Streptococcaceae | Streptococcus | Bacteria;Bacteria\_none;Bacillota;Bacilli;Lactobacillales;Streptococcaceae;Streptococcus;Streptococcus sp. zg-86 |
| Streptococcus suis | 3 | 1 | 3 | 0 | 3 | 4 | 1 | 0 | 0 | 9 | 3 | 0 | 3 | 2 | Bacteria | Bacteria\_none | Bacillota | Bacilli | Lactobacillales | Streptococcaceae | Streptococcus | Bacteria;Bacteria\_none;Bacillota;Bacilli;Lactobacillales;Streptococcaceae;Streptococcus;Streptococcus suis |
| Streptococcus thermophilus | 3 | 2 | 2 | 1 | 1 | 13 | 1 | 0 | 0 | 2 | 1 | 0 | 3 | 1 | Bacteria | Bacteria\_none | Bacillota | Bacilli | Lactobacillales | Streptococcaceae | Streptococcus | Bacteria;Bacteria\_none;Bacillota;Bacilli;Lactobacillales;Streptococcaceae;Streptococcus;Streptococcus thermophilus |
| Streptococcus toyakuensis | 1 | 11 | 0 | 7 | 52 | 10 | 2 | 1 | 0 | 7 | 28 | 6 | 2 | 5 | Bacteria | Bacteria\_none | Bacillota | Bacilli | Lactobacillales | Streptococcaceae | Streptococcus | Bacteria;Bacteria\_none;Bacillota;Bacilli;Lactobacillales;Streptococcaceae;Streptococcus;Streptococcus toyakuensis |
| Streptococcus troglodytae | 0 | 0 | 0 | 0 | 0 | 0 | 0 | 0 | 0 | 0 | 0 | 1 | 0 | 0 | Bacteria | Bacteria\_none | Bacillota | Bacilli | Lactobacillales | Streptococcaceae | Streptococcus | Bacteria;Bacteria\_none;Bacillota;Bacilli;Lactobacillales;Streptococcaceae;Streptococcus;Streptococcus troglodytae |
| Streptococcus uberis | 0 | 0 | 0 | 0 | 0 | 0 | 0 | 0 | 0 | 0 | 0 | 1 | 0 | 0 | Bacteria | Bacteria\_none | Bacillota | Bacilli | Lactobacillales | Streptococcaceae | Streptococcus | Bacteria;Bacteria\_none;Bacillota;Bacilli;Lactobacillales;Streptococcaceae;Streptococcus;Streptococcus uberis |
| Streptococcus urinalis | 0 | 0 | 0 | 0 | 0 | 0 | 0 | 0 | 0 | 0 | 0 | 0 | 0 | 0 | Bacteria | Bacteria\_none | Bacillota | Bacilli | Lactobacillales | Streptococcaceae | Streptococcus | Bacteria;Bacteria\_none;Bacillota;Bacilli;Lactobacillales;Streptococcaceae;Streptococcus;Streptococcus urinalis |
| Streptococcus vestibularis | 0 | 0 | 0 | 0 | 1 | 1 | 0 | 0 | 1 | 0 | 0 | 0 | 0 | 1 | Bacteria | Bacteria\_none | Bacillota | Bacilli | Lactobacillales | Streptococcaceae | Streptococcus | Bacteria;Bacteria\_none;Bacillota;Bacilli;Lactobacillales;Streptococcaceae;Streptococcus;Streptococcus vestibularis |
| Caldicellulosiruptor morganii | 0 | 0 | 0 | 0 | 0 | 0 | 0 | 0 | 0 | 0 | 0 | 0 | 0 | 0 | Bacteria | Bacteria\_none | Bacillota | Bacillota\_Incertae\_sedis | Caldicellulosiruptorales | Caldicellulosiruptoraceae | Caldicellulosiruptor | Bacteria;Bacteria\_none;Bacillota;Bacillota\_Incertae\_sedis;Caldicellulosiruptorales;Caldicellulosiruptoraceae;Caldicellulosiruptor;Caldicellulosiruptor morganii |
| Aristaeella lactis | 0 | 0 | 0 | 0 | 0 | 0 | 0 | 0 | 0 | 0 | 0 | 0 | 0 | 0 | Bacteria | Bacteria\_none | Bacillota | Clostridia | Eubacteriales | Aristaeellaceae | Aristaeella | Bacteria;Bacteria\_none;Bacillota;Clostridia;Eubacteriales;Aristaeellaceae;Aristaeella;Aristaeella lactis |
| Clostridium aceticum | 0 | 0 | 0 | 1 | 0 | 0 | 0 | 0 | 0 | 0 | 0 | 0 | 0 | 0 | Bacteria | Bacteria\_none | Bacillota | Clostridia | Eubacteriales | Clostridiaceae | Clostridium | Bacteria;Bacteria\_none;Bacillota;Clostridia;Eubacteriales;Clostridiaceae;Clostridium;Clostridium aceticum |
| Clostridium acetobutylicum | 1 | 0 | 0 | 0 | 0 | 0 | 0 | 0 | 0 | 0 | 0 | 0 | 0 | 0 | Bacteria | Bacteria\_none | Bacillota | Clostridia | Eubacteriales | Clostridiaceae | Clostridium | Bacteria;Bacteria\_none;Bacillota;Clostridia;Eubacteriales;Clostridiaceae;Clostridium;Clostridium acetobutylicum |
| Clostridium argentinense | 0 | 0 | 0 | 0 | 0 | 0 | 0 | 0 | 0 | 0 | 0 | 0 | 0 | 0 | Bacteria | Bacteria\_none | Bacillota | Clostridia | Eubacteriales | Clostridiaceae | Clostridium | Bacteria;Bacteria\_none;Bacillota;Clostridia;Eubacteriales;Clostridiaceae;Clostridium;Clostridium argentinense |
| Clostridium baratii | 0 | 0 | 0 | 0 | 0 | 0 | 0 | 0 | 0 | 0 | 0 | 0 | 0 | 0 | Bacteria | Bacteria\_none | Bacillota | Clostridia | Eubacteriales | Clostridiaceae | Clostridium | Bacteria;Bacteria\_none;Bacillota;Clostridia;Eubacteriales;Clostridiaceae;Clostridium;Clostridium baratii |
| Clostridium beijerinckii | 0 | 0 | 0 | 0 | 0 | 0 | 0 | 0 | 0 | 0 | 0 | 0 | 0 | 0 | Bacteria | Bacteria\_none | Bacillota | Clostridia | Eubacteriales | Clostridiaceae | Clostridium | Bacteria;Bacteria\_none;Bacillota;Clostridia;Eubacteriales;Clostridiaceae;Clostridium;Clostridium beijerinckii |
| Clostridium botulinum | 0 | 0 | 0 | 1 | 1 | 1 | 0 | 0 | 0 | 0 | 0 | 0 | 0 | 0 | Bacteria | Bacteria\_none | Bacillota | Clostridia | Eubacteriales | Clostridiaceae | Clostridium | Bacteria;Bacteria\_none;Bacillota;Clostridia;Eubacteriales;Clostridiaceae;Clostridium;Clostridium botulinum |
| Clostridium butyricum | 0 | 0 | 0 | 0 | 0 | 4 | 0 | 0 | 0 | 0 | 0 | 0 | 0 | 0 | Bacteria | Bacteria\_none | Bacillota | Clostridia | Eubacteriales | Clostridiaceae | Clostridium | Bacteria;Bacteria\_none;Bacillota;Clostridia;Eubacteriales;Clostridiaceae;Clostridium;Clostridium butyricum |
| Clostridium cadaveris | 0 | 0 | 0 | 0 | 0 | 0 | 0 | 0 | 0 | 0 | 0 | 0 | 0 | 0 | Bacteria | Bacteria\_none | Bacillota | Clostridia | Eubacteriales | Clostridiaceae | Clostridium | Bacteria;Bacteria\_none;Bacillota;Clostridia;Eubacteriales;Clostridiaceae;Clostridium;Clostridium cadaveris |
| Clostridium chauvoei | 0 | 0 | 0 | 0 | 0 | 0 | 0 | 0 | 0 | 0 | 0 | 0 | 0 | 0 | Bacteria | Bacteria\_none | Bacillota | Clostridia | Eubacteriales | Clostridiaceae | Clostridium | Bacteria;Bacteria\_none;Bacillota;Clostridia;Eubacteriales;Clostridiaceae;Clostridium;Clostridium chauvoei |
| Clostridium estertheticum | 0 | 0 | 0 | 0 | 0 | 0 | 0 | 0 | 1 | 0 | 0 | 0 | 0 | 0 | Bacteria | Bacteria\_none | Bacillota | Clostridia | Eubacteriales | Clostridiaceae | Clostridium | Bacteria;Bacteria\_none;Bacillota;Clostridia;Eubacteriales;Clostridiaceae;Clostridium;Clostridium estertheticum |
| Clostridium formicaceticum | 0 | 2 | 0 | 0 | 0 | 0 | 0 | 0 | 0 | 0 | 0 | 0 | 0 | 0 | Bacteria | Bacteria\_none | Bacillota | Clostridia | Eubacteriales | Clostridiaceae | Clostridium | Bacteria;Bacteria\_none;Bacillota;Clostridia;Eubacteriales;Clostridiaceae;Clostridium;Clostridium formicaceticum |
| Clostridium gasigenes | 0 | 0 | 0 | 1 | 0 | 0 | 0 | 0 | 0 | 0 | 0 | 0 | 0 | 0 | Bacteria | Bacteria\_none | Bacillota | Clostridia | Eubacteriales | Clostridiaceae | Clostridium | Bacteria;Bacteria\_none;Bacillota;Clostridia;Eubacteriales;Clostridiaceae;Clostridium;Clostridium gasigenes |
| Clostridium intestinale | 0 | 0 | 0 | 0 | 0 | 0 | 0 | 0 | 0 | 0 | 0 | 0 | 0 | 0 | Bacteria | Bacteria\_none | Bacillota | Clostridia | Eubacteriales | Clostridiaceae | Clostridium | Bacteria;Bacteria\_none;Bacillota;Clostridia;Eubacteriales;Clostridiaceae;Clostridium;Clostridium intestinale |
| Clostridium isatidis | 0 | 0 | 0 | 0 | 0 | 0 | 0 | 0 | 0 | 0 | 0 | 0 | 0 | 0 | Bacteria | Bacteria\_none | Bacillota | Clostridia | Eubacteriales | Clostridiaceae | Clostridium | Bacteria;Bacteria\_none;Bacillota;Clostridia;Eubacteriales;Clostridiaceae;Clostridium;Clostridium isatidis |
| Clostridium kluyveri | 0 | 0 | 1 | 0 | 0 | 0 | 0 | 0 | 0 | 0 | 0 | 0 | 0 | 0 | Bacteria | Bacteria\_none | Bacillota | Clostridia | Eubacteriales | Clostridiaceae | Clostridium | Bacteria;Bacteria\_none;Bacillota;Clostridia;Eubacteriales;Clostridiaceae;Clostridium;Clostridium kluyveri |
| Clostridium pasteurianum | 0 | 0 | 0 | 0 | 0 | 0 | 0 | 0 | 0 | 0 | 0 | 0 | 0 | 0 | Bacteria | Bacteria\_none | Bacillota | Clostridia | Eubacteriales | Clostridiaceae | Clostridium | Bacteria;Bacteria\_none;Bacillota;Clostridia;Eubacteriales;Clostridiaceae;Clostridium;Clostridium pasteurianum |
| Clostridium perfringens | 0 | 0 | 0 | 1 | 0 | 0 | 0 | 0 | 0 | 0 | 0 | 0 | 0 | 0 | Bacteria | Bacteria\_none | Bacillota | Clostridia | Eubacteriales | Clostridiaceae | Clostridium | Bacteria;Bacteria\_none;Bacillota;Clostridia;Eubacteriales;Clostridiaceae;Clostridium;Clostridium perfringens |
| Clostridium saccharobutylicum | 0 | 0 | 0 | 0 | 0 | 0 | 0 | 0 | 0 | 0 | 0 | 0 | 0 | 0 | Bacteria | Bacteria\_none | Bacillota | Clostridia | Eubacteriales | Clostridiaceae | Clostridium | Bacteria;Bacteria\_none;Bacillota;Clostridia;Eubacteriales;Clostridiaceae;Clostridium;Clostridium saccharobutylicum |
| Clostridium septicum | 0 | 0 | 0 | 0 | 0 | 0 | 0 | 0 | 0 | 0 | 0 | 0 | 1 | 0 | Bacteria | Bacteria\_none | Bacillota | Clostridia | Eubacteriales | Clostridiaceae | Clostridium | Bacteria;Bacteria\_none;Bacillota;Clostridia;Eubacteriales;Clostridiaceae;Clostridium;Clostridium septicum |
| Clostridium sp. C1 | 0 | 0 | 0 | 0 | 0 | 0 | 0 | 0 | 0 | 0 | 0 | 0 | 0 | 0 | Bacteria | Bacteria\_none | Bacillota | Clostridia | Eubacteriales | Clostridiaceae | Clostridium | Bacteria;Bacteria\_none;Bacillota;Clostridia;Eubacteriales;Clostridiaceae;Clostridium;Clostridium sp. C1 |
| Clostridium sp. DL-VIII | 0 | 0 | 0 | 0 | 0 | 0 | 0 | 0 | 0 | 0 | 0 | 0 | 0 | 0 | Bacteria | Bacteria\_none | Bacillota | Clostridia | Eubacteriales | Clostridiaceae | Clostridium | Bacteria;Bacteria\_none;Bacillota;Clostridia;Eubacteriales;Clostridiaceae;Clostridium;Clostridium sp. DL-VIII |
| Clostridium sp. JN-9 | 0 | 0 | 0 | 0 | 0 | 0 | 0 | 0 | 0 | 0 | 0 | 0 | 0 | 1 | Bacteria | Bacteria\_none | Bacillota | Clostridia | Eubacteriales | Clostridiaceae | Clostridium | Bacteria;Bacteria\_none;Bacillota;Clostridia;Eubacteriales;Clostridiaceae;Clostridium;Clostridium sp. JN-9 |
| Clostridium sp. MB40-C1 | 0 | 0 | 1 | 0 | 1 | 0 | 0 | 0 | 0 | 0 | 0 | 0 | 0 | 0 | Bacteria | Bacteria\_none | Bacillota | Clostridia | Eubacteriales | Clostridiaceae | Clostridium | Bacteria;Bacteria\_none;Bacillota;Clostridia;Eubacteriales;Clostridiaceae;Clostridium;Clostridium sp. MB40-C1 |
| Clostridium sp. MD294 | 0 | 0 | 0 | 0 | 0 | 1 | 0 | 0 | 0 | 0 | 0 | 0 | 0 | 0 | Bacteria | Bacteria\_none | Bacillota | Clostridia | Eubacteriales | Clostridiaceae | Clostridium | Bacteria;Bacteria\_none;Bacillota;Clostridia;Eubacteriales;Clostridiaceae;Clostridium;Clostridium sp. MD294 |
| Clostridium tyrobutyricum | 0 | 0 | 0 | 0 | 1 | 0 | 0 | 0 | 0 | 0 | 0 | 0 | 0 | 0 | Bacteria | Bacteria\_none | Bacillota | Clostridia | Eubacteriales | Clostridiaceae | Clostridium | Bacteria;Bacteria\_none;Bacillota;Clostridia;Eubacteriales;Clostridiaceae;Clostridium;Clostridium tyrobutyricum |
| Crassaminicella profunda | 0 | 0 | 0 | 0 | 0 | 3 | 0 | 0 | 0 | 0 | 0 | 0 | 0 | 0 | Bacteria | Bacteria\_none | Bacillota | Clostridia | Eubacteriales | Clostridiaceae | Crassaminicella | Bacteria;Bacteria\_none;Bacillota;Clostridia;Eubacteriales;Clostridiaceae;Crassaminicella;Crassaminicella profunda |
| Crassaminicella thermophila | 0 | 0 | 0 | 0 | 0 | 0 | 0 | 0 | 0 | 0 | 0 | 0 | 0 | 0 | Bacteria | Bacteria\_none | Bacillota | Clostridia | Eubacteriales | Clostridiaceae | Crassaminicella | Bacteria;Bacteria\_none;Bacillota;Clostridia;Eubacteriales;Clostridiaceae;Crassaminicella;Crassaminicella thermophila |
| Oceanirhabdus sp. W0125-5 | 0 | 0 | 0 | 0 | 0 | 1 | 0 | 0 | 0 | 0 | 0 | 0 | 0 | 0 | Bacteria | Bacteria\_none | Bacillota | Clostridia | Eubacteriales | Clostridiaceae | Oceanirhabdus | Bacteria;Bacteria\_none;Bacillota;Clostridia;Eubacteriales;Clostridiaceae;Oceanirhabdus;Oceanirhabdus sp. W0125-5 |
| Sarcina sp. JB2 | 0 | 0 | 0 | 0 | 0 | 1 | 0 | 1 | 1 | 0 | 0 | 0 | 0 | 0 | Bacteria | Bacteria\_none | Bacillota | Clostridia | Eubacteriales | Clostridiaceae | Sarcina | Bacteria;Bacteria\_none;Bacillota;Clostridia;Eubacteriales;Clostridiaceae;Sarcina;Sarcina sp. JB2 |
| Carboxydocella thermautotrophica | 0 | 0 | 0 | 0 | 0 | 0 | 0 | 0 | 0 | 0 | 0 | 0 | 0 | 1 | Bacteria | Bacteria\_none | Bacillota | Clostridia | Eubacteriales | Clostridiales Family XVI. Incertae Sedis | Carboxydocella | Bacteria;Bacteria\_none;Bacillota;Clostridia;Eubacteriales;Clostridiales Family XVI. Incertae Sedis;Carboxydocella;Carboxydocella thermautotrophica |
| Dehalobacter restrictus | 0 | 0 | 0 | 0 | 0 | 0 | 0 | 0 | 0 | 0 | 0 | 0 | 0 | 0 | Bacteria | Bacteria\_none | Bacillota | Clostridia | Eubacteriales | Desulfitobacteriaceae | Dehalobacter | Bacteria;Bacteria\_none;Bacillota;Clostridia;Eubacteriales;Desulfitobacteriaceae;Dehalobacter;Dehalobacter restrictus |
| Desulfosporosinus meridiei | 0 | 0 | 0 | 0 | 0 | 0 | 0 | 0 | 0 | 0 | 0 | 0 | 0 | 0 | Bacteria | Bacteria\_none | Bacillota | Clostridia | Eubacteriales | Desulfitobacteriaceae | Desulfosporosinus | Bacteria;Bacteria\_none;Bacillota;Clostridia;Eubacteriales;Desulfitobacteriaceae;Desulfosporosinus;Desulfosporosinus meridiei |
| Desulfosporosinus orientis | 0 | 0 | 0 | 0 | 0 | 0 | 0 | 0 | 0 | 0 | 0 | 0 | 0 | 0 | Bacteria | Bacteria\_none | Bacillota | Clostridia | Eubacteriales | Desulfitobacteriaceae | Desulfosporosinus | Bacteria;Bacteria\_none;Bacillota;Clostridia;Eubacteriales;Desulfitobacteriaceae;Desulfosporosinus;Desulfosporosinus orientis |
| Acetobacterium woodii | 0 | 0 | 1 | 0 | 0 | 0 | 0 | 0 | 0 | 0 | 0 | 0 | 0 | 0 | Bacteria | Bacteria\_none | Bacillota | Clostridia | Eubacteriales | Eubacteriaceae | Acetobacterium | Bacteria;Bacteria\_none;Bacillota;Clostridia;Eubacteriales;Eubacteriaceae;Acetobacterium;Acetobacterium woodii |
| Acetobacterium\_uncultured | 0 | 0 | 0 | 0 | 0 | 0 | 1 | 0 | 0 | 0 | 0 | 0 | 0 | 0 | Bacteria | Bacteria\_none | Bacillota | Clostridia | Eubacteriales | Eubacteriaceae | Acetobacterium | Bacteria;Bacteria\_none;Bacillota;Clostridia;Eubacteriales;Eubacteriaceae;Acetobacterium;Acetobacterium\_uncultured |
| Eubacterium sp. MSJ-33 | 0 | 0 | 0 | 0 | 0 | 1 | 0 | 0 | 0 | 0 | 0 | 0 | 0 | 0 | Bacteria | Bacteria\_none | Bacillota | Clostridia | Eubacteriales | Eubacteriaceae | Eubacterium | Bacteria;Bacteria\_none;Bacillota;Clostridia;Eubacteriales;Eubacteriaceae;Eubacterium;Eubacterium sp. MSJ-33 |
| Eubacterium ventriosum | 0 | 0 | 0 | 0 | 0 | 0 | 0 | 0 | 0 | 0 | 0 | 0 | 0 | 0 | Bacteria | Bacteria\_none | Bacillota | Clostridia | Eubacteriales | Eubacteriaceae | Eubacterium | Bacteria;Bacteria\_none;Bacillota;Clostridia;Eubacteriales;Eubacteriaceae;Eubacterium;Eubacterium ventriosum |
| Sinanaerobacter sp. ZZT-01 | 0 | 0 | 0 | 0 | 0 | 0 | 0 | 0 | 0 | 0 | 0 | 1 | 1 | 0 | Bacteria | Bacteria\_none | Bacillota | Clostridia | Eubacteriales | Eubacteriales Family XIII. Incertae Sedis | Sinanaerobacter | Bacteria;Bacteria\_none;Bacillota;Clostridia;Eubacteriales;Eubacteriales Family XIII. Incertae Sedis;Sinanaerobacter;Sinanaerobacter sp. ZZT-01 |
| Fenollaria massiliensis | 0 | 0 | 0 | 0 | 0 | 0 | 0 | 0 | 0 | 0 | 1 | 0 | 0 | 0 | Bacteria | Bacteria\_none | Bacillota | Clostridia | Eubacteriales | Eubacteriales\_Incertae\_sedis | Fenollaria | Bacteria;Bacteria\_none;Bacillota;Clostridia;Eubacteriales;Eubacteriales\_Incertae\_sedis;Fenollaria;Fenollaria massiliensis |
| Massilistercora timonensis | 0 | 0 | 0 | 0 | 0 | 2 | 0 | 0 | 0 | 0 | 0 | 0 | 0 | 0 | Bacteria | Bacteria\_none | Bacillota | Clostridia | Eubacteriales | Eubacteriales\_Incertae\_sedis | Massilistercora | Bacteria;Bacteria\_none;Bacillota;Clostridia;Eubacteriales;Eubacteriales\_Incertae\_sedis;Massilistercora;Massilistercora timonensis |
| Acetivibrio clariflavus | 0 | 0 | 0 | 0 | 0 | 0 | 0 | 0 | 0 | 0 | 0 | 0 | 0 | 0 | Bacteria | Bacteria\_none | Bacillota | Clostridia | Eubacteriales | Oscillospiraceae | Acetivibrio | Bacteria;Bacteria\_none;Bacillota;Clostridia;Eubacteriales;Oscillospiraceae;Acetivibrio;Acetivibrio clariflavus |
| Amygdalobacter indicium | 0 | 0 | 1 | 0 | 1 | 0 | 0 | 0 | 0 | 0 | 0 | 0 | 0 | 0 | Bacteria | Bacteria\_none | Bacillota | Clostridia | Eubacteriales | Oscillospiraceae | Amygdalobacter | Bacteria;Bacteria\_none;Bacillota;Clostridia;Eubacteriales;Oscillospiraceae;Amygdalobacter;Amygdalobacter indicium |
| Caproicibacterium amylolyticum | 0 | 0 | 0 | 0 | 0 | 1 | 0 | 0 | 0 | 0 | 0 | 0 | 0 | 0 | Bacteria | Bacteria\_none | Bacillota | Clostridia | Eubacteriales | Oscillospiraceae | Caproicibacterium | Bacteria;Bacteria\_none;Bacillota;Clostridia;Eubacteriales;Oscillospiraceae;Caproicibacterium;Caproicibacterium amylolyticum |
| Caproicibacterium sp. BJN0003 | 1 | 0 | 0 | 0 | 0 | 1 | 4 | 0 | 0 | 0 | 0 | 0 | 0 | 0 | Bacteria | Bacteria\_none | Bacillota | Clostridia | Eubacteriales | Oscillospiraceae | Caproicibacterium | Bacteria;Bacteria\_none;Bacillota;Clostridia;Eubacteriales;Oscillospiraceae;Caproicibacterium;Caproicibacterium sp. BJN0003 |
| Ethanoligenens harbinense | 0 | 0 | 0 | 0 | 0 | 0 | 0 | 0 | 0 | 0 | 0 | 0 | 0 | 0 | Bacteria | Bacteria\_none | Bacillota | Clostridia | Eubacteriales | Oscillospiraceae | Ethanoligenens | Bacteria;Bacteria\_none;Bacillota;Clostridia;Eubacteriales;Oscillospiraceae;Ethanoligenens;Ethanoligenens harbinense |
| Faecalibacterium prausnitzii | 0 | 1 | 0 | 0 | 0 | 0 | 0 | 0 | 0 | 0 | 0 | 0 | 0 | 0 | Bacteria | Bacteria\_none | Bacillota | Clostridia | Eubacteriales | Oscillospiraceae | Faecalibacterium | Bacteria;Bacteria\_none;Bacillota;Clostridia;Eubacteriales;Oscillospiraceae;Faecalibacterium;Faecalibacterium prausnitzii |
| Faecalibacterium sp. I4-1-79 | 0 | 0 | 1 | 0 | 0 | 0 | 0 | 0 | 0 | 0 | 0 | 0 | 0 | 0 | Bacteria | Bacteria\_none | Bacillota | Clostridia | Eubacteriales | Oscillospiraceae | Faecalibacterium | Bacteria;Bacteria\_none;Bacillota;Clostridia;Eubacteriales;Oscillospiraceae;Faecalibacterium;Faecalibacterium sp. I4-1-79 |
| Fastidiosipila sanguinis | 2 | 0 | 0 | 0 | 0 | 1 | 0 | 0 | 0 | 0 | 0 | 0 | 0 | 0 | Bacteria | Bacteria\_none | Bacillota | Clostridia | Eubacteriales | Oscillospiraceae | Fastidiosipila | Bacteria;Bacteria\_none;Bacillota;Clostridia;Eubacteriales;Oscillospiraceae;Fastidiosipila;Fastidiosipila sanguinis |
| Mageeibacillus indolicus | 0 | 0 | 0 | 0 | 0 | 0 | 0 | 0 | 0 | 0 | 0 | 0 | 0 | 0 | Bacteria | Bacteria\_none | Bacillota | Clostridia | Eubacteriales | Oscillospiraceae | Mageeibacillus | Bacteria;Bacteria\_none;Bacillota;Clostridia;Eubacteriales;Oscillospiraceae;Mageeibacillus;Mageeibacillus indolicus |
| Paludicola sp. MB14-C6 | 0 | 0 | 0 | 0 | 0 | 1 | 0 | 0 | 0 | 0 | 0 | 0 | 0 | 0 | Bacteria | Bacteria\_none | Bacillota | Clostridia | Eubacteriales | Oscillospiraceae | Paludihabitans | Bacteria;Bacteria\_none;Bacillota;Clostridia;Eubacteriales;Oscillospiraceae;Paludihabitans;Paludicola sp. MB14-C6 |
| Petroclostridium sp. X23 | 0 | 1 | 0 | 0 | 0 | 0 | 0 | 0 | 0 | 0 | 0 | 0 | 0 | 0 | Bacteria | Bacteria\_none | Bacillota | Clostridia | Eubacteriales | Oscillospiraceae | Petroclostridium | Bacteria;Bacteria\_none;Bacillota;Clostridia;Eubacteriales;Oscillospiraceae;Petroclostridium;Petroclostridium sp. X23 |
| Ruminiclostridium cellulolyticum | 0 | 0 | 0 | 1 | 0 | 0 | 0 | 0 | 0 | 0 | 0 | 0 | 0 | 0 | Bacteria | Bacteria\_none | Bacillota | Clostridia | Eubacteriales | Oscillospiraceae | Ruminiclostridium | Bacteria;Bacteria\_none;Bacillota;Clostridia;Eubacteriales;Oscillospiraceae;Ruminiclostridium;Ruminiclostridium cellulolyticum |
| Ruminococcus bicirculans (ex Wegman et al. 2014) | 0 | 1 | 0 | 0 | 1 | 0 | 0 | 0 | 0 | 0 | 0 | 0 | 0 | 0 | Bacteria | Bacteria\_none | Bacillota | Clostridia | Eubacteriales | Oscillospiraceae | Ruminococcus | Bacteria;Bacteria\_none;Bacillota;Clostridia;Eubacteriales;Oscillospiraceae;Ruminococcus;Ruminococcus bicirculans (ex Wegman et al. 2014) |
| Thermoclostridium stercorarium | 0 | 0 | 0 | 1 | 0 | 1 | 0 | 0 | 0 | 0 | 0 | 0 | 0 | 0 | Bacteria | Bacteria\_none | Bacillota | Clostridia | Eubacteriales | Oscillospiraceae | Thermoclostridium | Bacteria;Bacteria\_none;Bacillota;Clostridia;Eubacteriales;Oscillospiraceae;Thermoclostridium;Thermoclostridium stercorarium |
| Halobacteroides halobius | 0 | 0 | 0 | 0 | 0 | 0 | 0 | 0 | 0 | 0 | 0 | 0 | 0 | 0 | Bacteria | Bacteria\_none | Bacillota | Clostridia | Halanaerobiales | Halobacteroidaceae | Halobacteroides | Bacteria;Bacteria\_none;Bacillota;Clostridia;Halanaerobiales;Halobacteroidaceae;Halobacteroides;Halobacteroides halobius |
| Anaerotignum sp. MB30-C6 | 0 | 0 | 0 | 0 | 0 | 0 | 0 | 0 | 0 | 0 | 0 | 0 | 0 | 0 | Bacteria | Bacteria\_none | Bacillota | Clostridia | Lachnospirales | Anaerotignaceae | Anaerotignum | Bacteria;Bacteria\_none;Bacillota;Clostridia;Lachnospirales;Anaerotignaceae;Anaerotignum;Anaerotignum sp. MB30-C6 |
| Agathobacter rectalis | 0 | 0 | 1 | 0 | 0 | 0 | 0 | 0 | 0 | 0 | 0 | 0 | 0 | 0 | Bacteria | Bacteria\_none | Bacillota | Clostridia | Lachnospirales | Lachnospiraceae | Agathobacter | Bacteria;Bacteria\_none;Bacillota;Clostridia;Lachnospirales;Lachnospiraceae;Agathobacter;Agathobacter rectalis |
| Anaerobutyricum hallii | 0 | 0 | 0 | 0 | 2 | 0 | 0 | 0 | 0 | 1 | 0 | 0 | 1 | 0 | Bacteria | Bacteria\_none | Bacillota | Clostridia | Lachnospirales | Lachnospiraceae | Anaerobutyricum | Bacteria;Bacteria\_none;Bacillota;Clostridia;Lachnospirales;Lachnospiraceae;Anaerobutyricum;Anaerobutyricum hallii |
| Anaerocolumna sedimenticola | 0 | 0 | 0 | 0 | 0 | 1 | 0 | 0 | 0 | 0 | 0 | 0 | 0 | 0 | Bacteria | Bacteria\_none | Bacillota | Clostridia | Lachnospirales | Lachnospiraceae | Anaerocolumna | Bacteria;Bacteria\_none;Bacillota;Clostridia;Lachnospirales;Lachnospiraceae;Anaerocolumna;Anaerocolumna sedimenticola |
| Anaerocolumna sp. AGMB13020 | 0 | 0 | 0 | 0 | 0 | 1 | 0 | 0 | 0 | 0 | 0 | 0 | 0 | 0 | Bacteria | Bacteria\_none | Bacillota | Clostridia | Lachnospirales | Lachnospiraceae | Anaerocolumna | Bacteria;Bacteria\_none;Bacillota;Clostridia;Lachnospirales;Lachnospiraceae;Anaerocolumna;Anaerocolumna sp. AGMB13020 |
| Anaerocolumna sp. MB42-C2 | 0 | 0 | 0 | 0 | 0 | 1 | 0 | 0 | 0 | 0 | 0 | 0 | 0 | 0 | Bacteria | Bacteria\_none | Bacillota | Clostridia | Lachnospirales | Lachnospiraceae | Anaerocolumna | Bacteria;Bacteria\_none;Bacillota;Clostridia;Lachnospirales;Lachnospiraceae;Anaerocolumna;Anaerocolumna sp. MB42-C2 |
| Anaeromicropila herbilytica | 0 | 0 | 0 | 0 | 0 | 0 | 0 | 0 | 0 | 0 | 0 | 0 | 0 | 0 | Bacteria | Bacteria\_none | Bacillota | Clostridia | Lachnospirales | Lachnospiraceae | Anaeromicropila | Bacteria;Bacteria\_none;Bacillota;Clostridia;Lachnospirales;Lachnospiraceae;Anaeromicropila;Anaeromicropila herbilytica |
| Anaerostipes hadrus | 0 | 0 | 0 | 0 | 0 | 0 | 0 | 0 | 0 | 0 | 0 | 0 | 0 | 0 | Bacteria | Bacteria\_none | Bacillota | Clostridia | Lachnospirales | Lachnospiraceae | Anaerostipes | Bacteria;Bacteria\_none;Bacillota;Clostridia;Lachnospirales;Lachnospiraceae;Anaerostipes;Anaerostipes hadrus |
| Blautia hydrogenotrophica | 0 | 0 | 0 | 0 | 0 | 0 | 0 | 0 | 0 | 0 | 0 | 0 | 0 | 0 | Bacteria | Bacteria\_none | Bacillota | Clostridia | Lachnospirales | Lachnospiraceae | Blautia | Bacteria;Bacteria\_none;Bacillota;Clostridia;Lachnospirales;Lachnospiraceae;Blautia;Blautia hydrogenotrophica |
| Blautia obeum | 0 | 0 | 0 | 0 | 0 | 1 | 1 | 0 | 0 | 0 | 0 | 0 | 0 | 0 | Bacteria | Bacteria\_none | Bacillota | Clostridia | Lachnospirales | Lachnospiraceae | Blautia | Bacteria;Bacteria\_none;Bacillota;Clostridia;Lachnospirales;Lachnospiraceae;Blautia;Blautia obeum |
| Blautia parvula | 0 | 0 | 0 | 0 | 0 | 2 | 0 | 0 | 0 | 0 | 0 | 0 | 0 | 0 | Bacteria | Bacteria\_none | Bacillota | Clostridia | Lachnospirales | Lachnospiraceae | Blautia | Bacteria;Bacteria\_none;Bacillota;Clostridia;Lachnospirales;Lachnospiraceae;Blautia;Blautia parvula |
| Blautia pseudococcoides | 0 | 0 | 0 | 0 | 0 | 1 | 0 | 0 | 0 | 0 | 0 | 0 | 0 | 0 | Bacteria | Bacteria\_none | Bacillota | Clostridia | Lachnospirales | Lachnospiraceae | Blautia | Bacteria;Bacteria\_none;Bacillota;Clostridia;Lachnospirales;Lachnospiraceae;Blautia;Blautia pseudococcoides |
| Butyrivibrio crossotus | 0 | 0 | 0 | 0 | 0 | 2 | 0 | 0 | 0 | 0 | 1 | 0 | 0 | 0 | Bacteria | Bacteria\_none | Bacillota | Clostridia | Lachnospirales | Lachnospiraceae | Butyrivibrio | Bacteria;Bacteria\_none;Bacillota;Clostridia;Lachnospirales;Lachnospiraceae;Butyrivibrio;Butyrivibrio crossotus |
| Butyrivibrio fibrisolvens | 0 | 0 | 0 | 0 | 0 | 1 | 0 | 0 | 0 | 0 | 0 | 0 | 0 | 0 | Bacteria | Bacteria\_none | Bacillota | Clostridia | Lachnospirales | Lachnospiraceae | Butyrivibrio | Bacteria;Bacteria\_none;Bacillota;Clostridia;Lachnospirales;Lachnospiraceae;Butyrivibrio;Butyrivibrio fibrisolvens |
| Butyrivibrio proteoclasticus | 0 | 0 | 0 | 0 | 0 | 0 | 0 | 0 | 0 | 0 | 0 | 0 | 0 | 0 | Bacteria | Bacteria\_none | Bacillota | Clostridia | Lachnospirales | Lachnospiraceae | Butyrivibrio | Bacteria;Bacteria\_none;Bacillota;Clostridia;Lachnospirales;Lachnospiraceae;Butyrivibrio;Butyrivibrio proteoclasticus |
| Candidatus Epulonipiscium viviparus | 0 | 0 | 0 | 0 | 0 | 0 | 0 | 0 | 0 | 0 | 0 | 0 | 0 | 0 | Bacteria | Bacteria\_none | Bacillota | Clostridia | Lachnospirales | Lachnospiraceae | Candidatus Epulonipiscium | Bacteria;Bacteria\_none;Bacillota;Clostridia;Lachnospirales;Lachnospiraceae;Candidatus Epulonipiscium;Candidatus Epulonipiscium viviparus |
| Enterocloster bolteae | 0 | 0 | 0 | 0 | 0 | 0 | 0 | 0 | 0 | 0 | 0 | 0 | 0 | 0 | Bacteria | Bacteria\_none | Bacillota | Clostridia | Lachnospirales | Lachnospiraceae | Enterocloster | Bacteria;Bacteria\_none;Bacillota;Clostridia;Lachnospirales;Lachnospiraceae;Enterocloster;Enterocloster bolteae |
| Hungatella hathewayi | 0 | 0 | 0 | 0 | 0 | 0 | 0 | 0 | 0 | 0 | 0 | 0 | 0 | 0 | Bacteria | Bacteria\_none | Bacillota | Clostridia | Lachnospirales | Lachnospiraceae | Hungatella | Bacteria;Bacteria\_none;Bacillota;Clostridia;Lachnospirales;Lachnospiraceae;Hungatella;Hungatella hathewayi |
| Lachnoanaerobaculum gingivalis | 1 | 18 | 5 | 6 | 1 | 11 | 0 | 0 | 0 | 3 | 1 | 0 | 2 | 0 | Bacteria | Bacteria\_none | Bacillota | Clostridia | Lachnospirales | Lachnospiraceae | Lachnoanaerobaculum | Bacteria;Bacteria\_none;Bacillota;Clostridia;Lachnospirales;Lachnospiraceae;Lachnoanaerobaculum;Lachnoanaerobaculum gingivalis |
| Lachnoanaerobaculum umeaense | 0 | 4 | 0 | 2 | 4 | 5 | 0 | 0 | 0 | 0 | 0 | 0 | 0 | 1 | Bacteria | Bacteria\_none | Bacillota | Clostridia | Lachnospirales | Lachnospiraceae | Lachnoanaerobaculum | Bacteria;Bacteria\_none;Bacillota;Clostridia;Lachnospirales;Lachnospiraceae;Lachnoanaerobaculum;Lachnoanaerobaculum umeaense |
| [Clostridium] scindens | 0 | 0 | 1 | 0 | 0 | 2 | 0 | 0 | 0 | 0 | 0 | 0 | 0 | 0 | Bacteria | Bacteria\_none | Bacillota | Clostridia | Lachnospirales | Lachnospiraceae | Lachnoclostridium | Bacteria;Bacteria\_none;Bacillota;Clostridia;Lachnospirales;Lachnospiraceae;Lachnoclostridium;[Clostridium] scindens |
| [Ruminococcus] torques | 0 | 0 | 0 | 0 | 0 | 0 | 0 | 0 | 0 | 0 | 0 | 1 | 0 | 0 | Bacteria | Bacteria\_none | Bacillota | Clostridia | Lachnospirales | Lachnospiraceae | Mediterraneibacter | Bacteria;Bacteria\_none;Bacillota;Clostridia;Lachnospirales;Lachnospiraceae;Mediterraneibacter;[Ruminococcus] torques |
| Qiania dongpingensis | 0 | 0 | 0 | 0 | 0 | 0 | 0 | 0 | 0 | 0 | 0 | 0 | 0 | 0 | Bacteria | Bacteria\_none | Bacillota | Clostridia | Lachnospirales | Lachnospiraceae | Qiania | Bacteria;Bacteria\_none;Bacillota;Clostridia;Lachnospirales;Lachnospiraceae;Qiania;Qiania dongpingensis |
| Roseburia hominis | 0 | 0 | 2 | 0 | 0 | 0 | 0 | 0 | 0 | 0 | 0 | 0 | 0 | 0 | Bacteria | Bacteria\_none | Bacillota | Clostridia | Lachnospirales | Lachnospiraceae | Roseburia | Bacteria;Bacteria\_none;Bacillota;Clostridia;Lachnospirales;Lachnospiraceae;Roseburia;Roseburia hominis |
| Roseburia sp. 499 | 0 | 0 | 0 | 0 | 0 | 1 | 0 | 0 | 0 | 0 | 0 | 0 | 0 | 0 | Bacteria | Bacteria\_none | Bacillota | Clostridia | Lachnospirales | Lachnospiraceae | Roseburia | Bacteria;Bacteria\_none;Bacillota;Clostridia;Lachnospirales;Lachnospiraceae;Roseburia;Roseburia sp. 499 |
| Roseburia sp. 831b | 0 | 0 | 0 | 0 | 0 | 0 | 0 | 0 | 0 | 0 | 0 | 0 | 0 | 0 | Bacteria | Bacteria\_none | Bacillota | Clostridia | Lachnospirales | Lachnospiraceae | Roseburia | Bacteria;Bacteria\_none;Bacillota;Clostridia;Lachnospirales;Lachnospiraceae;Roseburia;Roseburia sp. 831b |
| Simiaoa sunii | 0 | 0 | 0 | 0 | 0 | 0 | 0 | 0 | 0 | 0 | 0 | 0 | 0 | 0 | Bacteria | Bacteria\_none | Bacillota | Clostridia | Lachnospirales | Lachnospiraceae | Simiaoa | Bacteria;Bacteria\_none;Bacillota;Clostridia;Lachnospirales;Lachnospiraceae;Simiaoa;Simiaoa sunii |
| Stomatobaculum sp. F0698 | 0 | 8 | 17 | 1 | 0 | 13 | 3 | 0 | 0 | 3 | 2 | 0 | 2 | 0 | Bacteria | Bacteria\_none | Bacillota | Clostridia | Lachnospirales | Lachnospiraceae | Stomatobaculum | Bacteria;Bacteria\_none;Bacillota;Clostridia;Lachnospirales;Lachnospiraceae;Stomatobaculum;Stomatobaculum sp. F0698 |
| [Clostridium] colinum | 0 | 0 | 0 | 0 | 1 | 0 | 0 | 0 | 0 | 0 | 0 | 0 | 0 | 0 | Bacteria | Bacteria\_none | Bacillota | Clostridia | Lachnospirales | Lachnospiraceae | Tyzzerella | Bacteria;Bacteria\_none;Bacillota;Clostridia;Lachnospirales;Lachnospiraceae;Tyzzerella;[Clostridium] colinum |
| Wansuia hejianensis | 0 | 0 | 0 | 0 | 1 | 0 | 0 | 0 | 0 | 0 | 0 | 0 | 0 | 0 | Bacteria | Bacteria\_none | Bacillota | Clostridia | Lachnospirales | Lachnospiraceae | Wansuia | Bacteria;Bacteria\_none;Bacillota;Clostridia;Lachnospirales;Lachnospiraceae;Wansuia;Wansuia hejianensis |
| Wujia chipingensis | 0 | 0 | 0 | 0 | 0 | 0 | 0 | 0 | 0 | 0 | 0 | 0 | 0 | 0 | Bacteria | Bacteria\_none | Bacillota | Clostridia | Lachnospirales | Lachnospiraceae | Wujia | Bacteria;Bacteria\_none;Bacillota;Clostridia;Lachnospirales;Lachnospiraceae;Wujia;Wujia chipingensis |
| Monoglobus pectinilyticus | 0 | 0 | 0 | 0 | 0 | 0 | 0 | 1 | 0 | 0 | 0 | 0 | 0 | 0 | Bacteria | Bacteria\_none | Bacillota | Clostridia | Monoglobales | Monoglobaceae | Monoglobus | Bacteria;Bacteria\_none;Bacillota;Clostridia;Monoglobales;Monoglobaceae;Monoglobus;Monoglobus pectinilyticus |
| Natranaerobius thermophilus | 0 | 0 | 0 | 0 | 0 | 0 | 0 | 0 | 0 | 0 | 0 | 0 | 0 | 0 | Bacteria | Bacteria\_none | Bacillota | Clostridia | Natranaerobiales | Natranaerobiaceae | Natranaerobius | Bacteria;Bacteria\_none;Bacillota;Clostridia;Natranaerobiales;Natranaerobiaceae;Natranaerobius;Natranaerobius thermophilus |
| Natranaerofaba carboxydovora | 0 | 0 | 0 | 0 | 0 | 1 | 0 | 0 | 0 | 0 | 0 | 0 | 0 | 0 | Bacteria | Bacteria\_none | Bacillota | Clostridia | Natranaerobiales | Natranaerofabaceae | Natranaerofaba | Bacteria;Bacteria\_none;Bacillota;Clostridia;Natranaerobiales;Natranaerofabaceae;Natranaerofaba;Natranaerofaba carboxydovora |
| Aminipila butyrica | 0 | 0 | 0 | 0 | 0 | 1 | 0 | 0 | 0 | 0 | 0 | 0 | 0 | 0 | Bacteria | Bacteria\_none | Bacillota | Clostridia | Peptostreptococcales | Anaerovoracaceae | Aminipila | Bacteria;Bacteria\_none;Bacillota;Clostridia;Peptostreptococcales;Anaerovoracaceae;Aminipila;Aminipila butyrica |
| Aminipila luticellarii | 0 | 0 | 0 | 0 | 0 | 0 | 0 | 0 | 0 | 0 | 0 | 1 | 0 | 0 | Bacteria | Bacteria\_none | Bacillota | Clostridia | Peptostreptococcales | Anaerovoracaceae | Aminipila | Bacteria;Bacteria\_none;Bacillota;Clostridia;Peptostreptococcales;Anaerovoracaceae;Aminipila;Aminipila luticellarii |
| Emergencia timonensis | 0 | 0 | 0 | 0 | 0 | 0 | 0 | 0 | 0 | 0 | 0 | 0 | 0 | 0 | Bacteria | Bacteria\_none | Bacillota | Clostridia | Peptostreptococcales | Anaerovoracaceae | Emergencia | Bacteria;Bacteria\_none;Bacillota;Clostridia;Peptostreptococcales;Anaerovoracaceae;Emergencia;Emergencia timonensis |
| Mogibacterium diversum | 1 | 0 | 2 | 0 | 0 | 23 | 1 | 0 | 0 | 0 | 0 | 0 | 2 | 6 | Bacteria | Bacteria\_none | Bacillota | Clostridia | Peptostreptococcales | Anaerovoracaceae | Mogibacterium | Bacteria;Bacteria\_none;Bacillota;Clostridia;Peptostreptococcales;Anaerovoracaceae;Mogibacterium;Mogibacterium diversum |
| Mogibacterium neglectum | 0 | 0 | 2 | 0 | 1 | 3 | 0 | 0 | 0 | 0 | 1 | 0 | 0 | 0 | Bacteria | Bacteria\_none | Bacillota | Clostridia | Peptostreptococcales | Anaerovoracaceae | Mogibacterium | Bacteria;Bacteria\_none;Bacillota;Clostridia;Peptostreptococcales;Anaerovoracaceae;Mogibacterium;Mogibacterium neglectum |
| Mogibacterium pumilum | 0 | 0 | 2 | 0 | 0 | 0 | 0 | 0 | 0 | 0 | 0 | 0 | 0 | 0 | Bacteria | Bacteria\_none | Bacillota | Clostridia | Peptostreptococcales | Anaerovoracaceae | Mogibacterium | Bacteria;Bacteria\_none;Bacillota;Clostridia;Peptostreptococcales;Anaerovoracaceae;Mogibacterium;Mogibacterium pumilum |
| Filifactor alocis | 3 | 38 | 35 | 0 | 1 | 1 | 1 | 14 | 0 | 0 | 2 | 9 | 2 | 4 | Bacteria | Bacteria\_none | Bacillota | Clostridia | Peptostreptococcales | Filifactoraceae | Filifactor | Bacteria;Bacteria\_none;Bacillota;Clostridia;Peptostreptococcales;Filifactoraceae;Filifactor;Filifactor alocis |
| Serpentinicella alkaliphila | 0 | 0 | 0 | 1 | 0 | 0 | 0 | 0 | 0 | 0 | 0 | 0 | 0 | 0 | Bacteria | Bacteria\_none | Bacillota | Clostridia | Peptostreptococcales | Natronincolaceae | Serpentinicella | Bacteria;Bacteria\_none;Bacillota;Clostridia;Peptostreptococcales;Natronincolaceae;Serpentinicella;Serpentinicella alkaliphila |
| Clostridioides difficile | 0 | 0 | 0 | 0 | 4 | 0 | 1 | 0 | 0 | 0 | 1 | 0 | 0 | 0 | Bacteria | Bacteria\_none | Bacillota | Clostridia | Peptostreptococcales | Peptostreptococcaceae | Clostridioides | Bacteria;Bacteria\_none;Bacillota;Clostridia;Peptostreptococcales;Peptostreptococcaceae;Clostridioides;Clostridioides difficile |
| Peptostreptococcus equinus | 0 | 0 | 3 | 2 | 0 | 0 | 0 | 0 | 0 | 0 | 1 | 0 | 0 | 0 | Bacteria | Bacteria\_none | Bacillota | Clostridia | Peptostreptococcales | Peptostreptococcaceae | Peptostreptococcus | Bacteria;Bacteria\_none;Bacillota;Clostridia;Peptostreptococcales;Peptostreptococcaceae;Peptostreptococcus;Peptostreptococcus equinus |
| Romboutsia ilealis | 0 | 0 | 0 | 1 | 0 | 0 | 0 | 0 | 0 | 0 | 0 | 0 | 0 | 0 | Bacteria | Bacteria\_none | Bacillota | Clostridia | Peptostreptococcales | Peptostreptococcaceae | Romboutsia | Bacteria;Bacteria\_none;Bacillota;Clostridia;Peptostreptococcales;Peptostreptococcaceae;Romboutsia;Romboutsia ilealis |
| Romboutsia sp. 13368 | 0 | 1 | 0 | 0 | 0 | 0 | 0 | 0 | 0 | 0 | 0 | 0 | 0 | 0 | Bacteria | Bacteria\_none | Bacillota | Clostridia | Peptostreptococcales | Peptostreptococcaceae | Romboutsia | Bacteria;Bacteria\_none;Bacillota;Clostridia;Peptostreptococcales;Peptostreptococcaceae;Romboutsia;Romboutsia sp. 13368 |
| Terrisporobacter glycolicus | 0 | 0 | 0 | 0 | 1 | 0 | 0 | 0 | 0 | 0 | 0 | 0 | 0 | 0 | Bacteria | Bacteria\_none | Bacillota | Clostridia | Peptostreptococcales | Peptostreptococcaceae | Terrisporobacter | Bacteria;Bacteria\_none;Bacillota;Clostridia;Peptostreptococcales;Peptostreptococcaceae;Terrisporobacter;Terrisporobacter glycolicus |
| Terrisporobacter petrolearius | 1 | 0 | 0 | 0 | 1 | 0 | 0 | 0 | 0 | 0 | 1 | 0 | 0 | 0 | Bacteria | Bacteria\_none | Bacillota | Clostridia | Peptostreptococcales | Peptostreptococcaceae | Terrisporobacter | Bacteria;Bacteria\_none;Bacillota;Clostridia;Peptostreptococcales;Peptostreptococcaceae;Terrisporobacter;Terrisporobacter petrolearius |
| Thermoanaerobacterium sp. RBIITD | 0 | 1 | 0 | 0 | 0 | 0 | 0 | 0 | 0 | 1 | 0 | 0 | 0 | 0 | Bacteria | Bacteria\_none | Bacillota | Clostridia | Thermoanaerobacterales | Thermoanaerobacteraceae | Thermoanaerobacterium | Bacteria;Bacteria\_none;Bacillota;Clostridia;Thermoanaerobacterales;Thermoanaerobacteraceae;Thermoanaerobacterium;Thermoanaerobacterium sp. RBIITD |
| Catenibacterium sp. co\_0103 | 0 | 0 | 0 | 0 | 0 | 0 | 0 | 0 | 0 | 0 | 0 | 0 | 0 | 0 | Bacteria | Bacteria\_none | Bacillota | Erysipelotrichia | Erysipelotrichales | Coprobacillaceae | Catenibacterium | Bacteria;Bacteria\_none;Bacillota;Erysipelotrichia;Erysipelotrichales;Coprobacillaceae;Catenibacterium;Catenibacterium sp. co\_0103 |
| Faecalibacillus intestinalis | 0 | 0 | 0 | 0 | 0 | 0 | 0 | 0 | 0 | 0 | 0 | 0 | 0 | 0 | Bacteria | Bacteria\_none | Bacillota | Erysipelotrichia | Erysipelotrichales | Coprobacillaceae | Faecalibacillus | Bacteria;Bacteria\_none;Bacillota;Erysipelotrichia;Erysipelotrichales;Coprobacillaceae;Faecalibacillus;Faecalibacillus intestinalis |
| Erysipelatoclostridium sp. An173 | 0 | 0 | 0 | 0 | 0 | 0 | 0 | 0 | 0 | 0 | 0 | 0 | 0 | 0 | Bacteria | Bacteria\_none | Bacillota | Erysipelotrichia | Erysipelotrichales | Coprobacillaceae | Thomasclavelia | Bacteria;Bacteria\_none;Bacillota;Erysipelotrichia;Erysipelotrichales;Coprobacillaceae;Thomasclavelia;Erysipelatoclostridium sp. An173 |
| Thomasclavelia ramosa | 0 | 0 | 0 | 0 | 0 | 0 | 0 | 0 | 0 | 0 | 0 | 0 | 0 | 0 | Bacteria | Bacteria\_none | Bacillota | Erysipelotrichia | Erysipelotrichales | Coprobacillaceae | Thomasclavelia | Bacteria;Bacteria\_none;Bacillota;Erysipelotrichia;Erysipelotrichales;Coprobacillaceae;Thomasclavelia;Thomasclavelia ramosa |
| Thomasclavelia spiroformis | 0 | 0 | 0 | 0 | 0 | 0 | 0 | 0 | 0 | 0 | 0 | 0 | 0 | 0 | Bacteria | Bacteria\_none | Bacillota | Erysipelotrichia | Erysipelotrichales | Coprobacillaceae | Thomasclavelia | Bacteria;Bacteria\_none;Bacillota;Erysipelotrichia;Erysipelotrichales;Coprobacillaceae;Thomasclavelia;Thomasclavelia spiroformis |
| Bulleidia sp. zg-1006 | 0 | 0 | 0 | 0 | 0 | 0 | 0 | 0 | 0 | 0 | 0 | 0 | 0 | 0 | Bacteria | Bacteria\_none | Bacillota | Erysipelotrichia | Erysipelotrichales | Erysipelotrichaceae | Bulleidia | Bacteria;Bacteria\_none;Bacillota;Erysipelotrichia;Erysipelotrichales;Erysipelotrichaceae;Bulleidia;Bulleidia sp. zg-1006 |
| Erysipelothrix inopinata | 0 | 1 | 0 | 0 | 0 | 0 | 0 | 0 | 0 | 0 | 0 | 0 | 0 | 0 | Bacteria | Bacteria\_none | Bacillota | Erysipelotrichia | Erysipelotrichales | Erysipelotrichaceae | Erysipelothrix | Bacteria;Bacteria\_none;Bacillota;Erysipelotrichia;Erysipelotrichales;Erysipelotrichaceae;Erysipelothrix;Erysipelothrix inopinata |
| Erysipelothrix sp. HDW6A | 0 | 0 | 0 | 0 | 1 | 0 | 0 | 0 | 0 | 0 | 0 | 0 | 0 | 0 | Bacteria | Bacteria\_none | Bacillota | Erysipelotrichia | Erysipelotrichales | Erysipelotrichaceae | Erysipelothrix | Bacteria;Bacteria\_none;Bacillota;Erysipelotrichia;Erysipelotrichales;Erysipelotrichaceae;Erysipelothrix;Erysipelothrix sp. HDW6A |
| [Clostridium] innocuum | 0 | 0 | 0 | 0 | 0 | 0 | 0 | 0 | 0 | 0 | 0 | 0 | 0 | 0 | Bacteria | Bacteria\_none | Bacillota | Erysipelotrichia | Erysipelotrichales | Erysipelotrichaceae | Erysipelotrichaceae\_Incertae\_sedis | Bacteria;Bacteria\_none;Bacillota;Erysipelotrichia;Erysipelotrichales;Erysipelotrichaceae;Erysipelotrichaceae\_Incertae\_sedis;[Clostridium] innocuum |
| Solobacterium moorei | 13 | 16 | 85 | 4 | 8 | 387 | 19 | 6 | 1 | 3 | 6 | 1 | 25 | 13 | Bacteria | Bacteria\_none | Bacillota | Erysipelotrichia | Erysipelotrichales | Erysipelotrichaceae | Solobacterium | Bacteria;Bacteria\_none;Bacillota;Erysipelotrichia;Erysipelotrichales;Erysipelotrichaceae;Solobacterium;Solobacterium moorei |
| Megamonas funiformis | 0 | 0 | 0 | 0 | 0 | 0 | 0 | 0 | 0 | 0 | 0 | 0 | 0 | 0 | Bacteria | Bacteria\_none | Bacillota | Negativicutes | Selenomonadales | Selenomonadaceae | Megamonas | Bacteria;Bacteria\_none;Bacillota;Negativicutes;Selenomonadales;Selenomonadaceae;Megamonas;Megamonas funiformis |
| Megamonas hypermegale | 0 | 0 | 1 | 0 | 0 | 0 | 0 | 0 | 0 | 0 | 0 | 0 | 0 | 0 | Bacteria | Bacteria\_none | Bacillota | Negativicutes | Selenomonadales | Selenomonadaceae | Megamonas | Bacteria;Bacteria\_none;Bacillota;Negativicutes;Selenomonadales;Selenomonadaceae;Megamonas;Megamonas hypermegale |
| Pectinatus frisingensis | 0 | 0 | 0 | 0 | 0 | 0 | 1 | 0 | 0 | 0 | 0 | 0 | 0 | 0 | Bacteria | Bacteria\_none | Bacillota | Negativicutes | Selenomonadales | Selenomonadaceae | Pectinatus | Bacteria;Bacteria\_none;Bacillota;Negativicutes;Selenomonadales;Selenomonadaceae;Pectinatus;Pectinatus frisingensis |
| Selenomonas dianae | 0 | 0 | 0 | 1 | 3 | 0 | 0 | 0 | 0 | 0 | 0 | 1 | 1 | 1 | Bacteria | Bacteria\_none | Bacillota | Negativicutes | Selenomonadales | Selenomonadaceae | Selenomonas | Bacteria;Bacteria\_none;Bacillota;Negativicutes;Selenomonadales;Selenomonadaceae;Selenomonas;Selenomonas dianae |
| Selenomonas sp. oral taxon 126 | 1 | 0 | 0 | 0 | 1 | 0 | 0 | 0 | 0 | 0 | 0 | 1 | 2 | 1 | Bacteria | Bacteria\_none | Bacillota | Negativicutes | Selenomonadales | Selenomonadaceae | Selenomonas | Bacteria;Bacteria\_none;Bacillota;Negativicutes;Selenomonadales;Selenomonadaceae;Selenomonas;Selenomonas sp. oral taxon 126 |
| Selenomonas sp. oral taxon 136 | 1 | 2 | 0 | 1 | 0 | 1 | 0 | 0 | 0 | 0 | 1 | 0 | 0 | 0 | Bacteria | Bacteria\_none | Bacillota | Negativicutes | Selenomonadales | Selenomonadaceae | Selenomonas | Bacteria;Bacteria\_none;Bacillota;Negativicutes;Selenomonadales;Selenomonadaceae;Selenomonas;Selenomonas sp. oral taxon 136 |
| Selenomonas sp. oral taxon 478 | 0 | 5 | 0 | 0 | 1 | 7 | 1 | 0 | 0 | 0 | 0 | 2 | 1 | 1 | Bacteria | Bacteria\_none | Bacillota | Negativicutes | Selenomonadales | Selenomonadaceae | Selenomonas | Bacteria;Bacteria\_none;Bacillota;Negativicutes;Selenomonadales;Selenomonadaceae;Selenomonas;Selenomonas sp. oral taxon 478 |
| Selenomonas sp. oral taxon 920 | 0 | 0 | 4 | 1 | 0 | 0 | 0 | 0 | 0 | 0 | 0 | 0 | 1 | 0 | Bacteria | Bacteria\_none | Bacillota | Negativicutes | Selenomonadales | Selenomonadaceae | Selenomonas | Bacteria;Bacteria\_none;Bacillota;Negativicutes;Selenomonadales;Selenomonadaceae;Selenomonas;Selenomonas sp. oral taxon 920 |
| Selenomonas sputigena | 3 | 0 | 3 | 0 | 1 | 3 | 1 | 1 | 0 | 1 | 1 | 0 | 1 | 1 | Bacteria | Bacteria\_none | Bacillota | Negativicutes | Selenomonadales | Selenomonadaceae | Selenomonas | Bacteria;Bacteria\_none;Bacillota;Negativicutes;Selenomonadales;Selenomonadaceae;Selenomonas;Selenomonas sputigena |
| Selenomonas timonae | 0 | 0 | 5 | 0 | 0 | 0 | 0 | 0 | 0 | 0 | 1 | 1 | 0 | 0 | Bacteria | Bacteria\_none | Bacillota | Negativicutes | Selenomonadales | Selenomonadaceae | Selenomonas | Bacteria;Bacteria\_none;Bacillota;Negativicutes;Selenomonadales;Selenomonadaceae;Selenomonas;Selenomonas timonae |
| Sporomusa ovata | 0 | 0 | 0 | 1 | 0 | 0 | 0 | 0 | 0 | 0 | 0 | 0 | 0 | 0 | Bacteria | Bacteria\_none | Bacillota | Negativicutes | Selenomonadales | Sporomusaceae | Sporomusa | Bacteria;Bacteria\_none;Bacillota;Negativicutes;Selenomonadales;Sporomusaceae;Sporomusa;Sporomusa ovata |
| Sporomusa termitida | 0 | 0 | 0 | 0 | 0 | 0 | 0 | 0 | 0 | 0 | 0 | 0 | 0 | 0 | Bacteria | Bacteria\_none | Bacillota | Negativicutes | Selenomonadales | Sporomusaceae | Sporomusa | Bacteria;Bacteria\_none;Bacillota;Negativicutes;Selenomonadales;Sporomusaceae;Sporomusa;Sporomusa termitida |
| Dialister pneumosintes | 0 | 0 | 1 | 0 | 0 | 0 | 0 | 0 | 0 | 0 | 1 | 0 | 1 | 2 | Bacteria | Bacteria\_none | Bacillota | Negativicutes | Veillonellales | Veillonellaceae | Dialister | Bacteria;Bacteria\_none;Bacillota;Negativicutes;Veillonellales;Veillonellaceae;Dialister;Dialister pneumosintes |
| Megasphaera elsdenii | 0 | 0 | 0 | 0 | 0 | 0 | 0 | 0 | 0 | 0 | 0 | 0 | 0 | 0 | Bacteria | Bacteria\_none | Bacillota | Negativicutes | Veillonellales | Veillonellaceae | Megasphaera | Bacteria;Bacteria\_none;Bacillota;Negativicutes;Veillonellales;Veillonellaceae;Megasphaera;Megasphaera elsdenii |
| Veillonella atypica | 72 | 14 | 5 | 1 | 4 | 27 | 3 | 0 | 17 | 2 | 0 | 22 | 9 | 7 | Bacteria | Bacteria\_none | Bacillota | Negativicutes | Veillonellales | Veillonellaceae | Veillonella | Bacteria;Bacteria\_none;Bacillota;Negativicutes;Veillonellales;Veillonellaceae;Veillonella;Veillonella atypica |
| Veillonella dispar | 124 | 6 | 2 | 0 | 0 | 2 | 2 | 0 | 0 | 0 | 0 | 1 | 9 | 6 | Bacteria | Bacteria\_none | Bacillota | Negativicutes | Veillonellales | Veillonellaceae | Veillonella | Bacteria;Bacteria\_none;Bacillota;Negativicutes;Veillonellales;Veillonellaceae;Veillonella;Veillonella dispar |
| Veillonella nakazawae | 278 | 13 | 3 | 2 | 1 | 1 | 3 | 2 | 1 | 2 | 0 | 1 | 14 | 16 | Bacteria | Bacteria\_none | Bacillota | Negativicutes | Veillonellales | Veillonellaceae | Veillonella | Bacteria;Bacteria\_none;Bacillota;Negativicutes;Veillonellales;Veillonellaceae;Veillonella;Veillonella nakazawae |
| Veillonella parvula | 166 | 13 | 26 | 6 | 7 | 2 | 7 | 7 | 5 | 26 | 0 | 23 | 14 | 12 | Bacteria | Bacteria\_none | Bacillota | Negativicutes | Veillonellales | Veillonellaceae | Veillonella | Bacteria;Bacteria\_none;Bacillota;Negativicutes;Veillonellales;Veillonellaceae;Veillonella;Veillonella parvula |
| Veillonella rodentium | 2 | 0 | 0 | 2 | 0 | 1 | 0 | 0 | 0 | 0 | 0 | 0 | 1 | 0 | Bacteria | Bacteria\_none | Bacillota | Negativicutes | Veillonellales | Veillonellaceae | Veillonella | Bacteria;Bacteria\_none;Bacillota;Negativicutes;Veillonellales;Veillonellaceae;Veillonella;Veillonella rodentium |
| Veillonella rogosae | 221 | 15 | 2 | 38 | 23 | 2 | 1 | 7 | 0 | 44 | 0 | 12 | 9 | 13 | Bacteria | Bacteria\_none | Bacillota | Negativicutes | Veillonellales | Veillonellaceae | Veillonella | Bacteria;Bacteria\_none;Bacillota;Negativicutes;Veillonellales;Veillonellaceae;Veillonella;Veillonella rogosae |
| Veillonella sp. OK1 | 12 | 0 | 0 | 0 | 0 | 0 | 0 | 0 | 0 | 0 | 0 | 0 | 0 | 1 | Bacteria | Bacteria\_none | Bacillota | Negativicutes | Veillonellales | Veillonellaceae | Veillonella | Bacteria;Bacteria\_none;Bacillota;Negativicutes;Veillonellales;Veillonellaceae;Veillonella;Veillonella sp. OK1 |
| Veillonella sp. S12025-13 | 179 | 11 | 0 | 0 | 0 | 4 | 0 | 0 | 0 | 1 | 0 | 0 | 3 | 6 | Bacteria | Bacteria\_none | Bacillota | Negativicutes | Veillonellales | Veillonellaceae | Veillonella | Bacteria;Bacteria\_none;Bacillota;Negativicutes;Veillonellales;Veillonellaceae;Veillonella;Veillonella sp. S12025-13 |
| Acidilutibacter cellobiosedens | 0 | 0 | 0 | 0 | 1 | 0 | 0 | 0 | 0 | 0 | 0 | 0 | 0 | 0 | Bacteria | Bacteria\_none | Bacillota | Tissierellia | Tissierellales | Acidilutibacteraceae | Acidilutibacter | Bacteria;Bacteria\_none;Bacillota;Tissierellia;Tissierellales;Acidilutibacteraceae;Acidilutibacter;Acidilutibacter cellobiosedens |
| Gottschalkia acidurici | 0 | 0 | 0 | 0 | 0 | 0 | 0 | 0 | 0 | 0 | 0 | 0 | 0 | 0 | Bacteria | Bacteria\_none | Bacillota | Tissierellia | Tissierellales | Gottschalkiaceae | Gottschalkia | Bacteria;Bacteria\_none;Bacillota;Tissierellia;Tissierellales;Gottschalkiaceae;Gottschalkia;Gottschalkia acidurici |
| Anaerococcus mediterraneensis | 1 | 0 | 0 | 0 | 0 | 0 | 0 | 0 | 0 | 0 | 0 | 0 | 0 | 0 | Bacteria | Bacteria\_none | Bacillota | Tissierellia | Tissierellales | Peptoniphilaceae | Anaerococcus | Bacteria;Bacteria\_none;Bacillota;Tissierellia;Tissierellales;Peptoniphilaceae;Anaerococcus;Anaerococcus mediterraneensis |
| Anaerococcus sp. Marseille-Q7828 | 0 | 0 | 0 | 0 | 0 | 0 | 0 | 0 | 0 | 0 | 0 | 0 | 0 | 0 | Bacteria | Bacteria\_none | Bacillota | Tissierellia | Tissierellales | Peptoniphilaceae | Anaerococcus | Bacteria;Bacteria\_none;Bacillota;Tissierellia;Tissierellales;Peptoniphilaceae;Anaerococcus;Anaerococcus sp. Marseille-Q7828 |
| Finegoldia magna | 0 | 0 | 0 | 0 | 0 | 1 | 0 | 0 | 0 | 0 | 0 | 0 | 0 | 0 | Bacteria | Bacteria\_none | Bacillota | Tissierellia | Tissierellales | Peptoniphilaceae | Finegoldia | Bacteria;Bacteria\_none;Bacillota;Tissierellia;Tissierellales;Peptoniphilaceae;Finegoldia;Finegoldia magna |
| Helcococcus ovis | 0 | 0 | 0 | 1 | 0 | 0 | 0 | 0 | 0 | 0 | 0 | 1 | 0 | 0 | Bacteria | Bacteria\_none | Bacillota | Tissierellia | Tissierellales | Peptoniphilaceae | Helcococcus | Bacteria;Bacteria\_none;Bacillota;Tissierellia;Tissierellales;Peptoniphilaceae;Helcococcus;Helcococcus ovis |
| Miniphocaeibacter halophilus | 0 | 0 | 0 | 0 | 0 | 0 | 0 | 0 | 0 | 0 | 0 | 0 | 0 | 0 | Bacteria | Bacteria\_none | Bacillota | Tissierellia | Tissierellales | Peptoniphilaceae | Miniphocaeibacter | Bacteria;Bacteria\_none;Bacillota;Tissierellia;Tissierellales;Peptoniphilaceae;Miniphocaeibacter;Miniphocaeibacter halophilus |
| Parvimonas micra | 4 | 32 | 23 | 11 | 25 | 46 | 6 | 13 | 0 | 1 | 23 | 8 | 10 | 9 | Bacteria | Bacteria\_none | Bacillota | Tissierellia | Tissierellales | Peptoniphilaceae | Parvimonas | Bacteria;Bacteria\_none;Bacillota;Tissierellia;Tissierellales;Peptoniphilaceae;Parvimonas;Parvimonas micra |
| Peptoniphilus genitalis | 0 | 0 | 0 | 0 | 0 | 1 | 0 | 0 | 0 | 0 | 0 | 0 | 0 | 0 | Bacteria | Bacteria\_none | Bacillota | Tissierellia | Tissierellales | Peptoniphilaceae | Peptoniphilus | Bacteria;Bacteria\_none;Bacillota;Tissierellia;Tissierellales;Peptoniphilaceae;Peptoniphilus;Peptoniphilus genitalis |
| Keratinibaculum paraultunense | 0 | 0 | 0 | 0 | 1 | 0 | 0 | 0 | 0 | 0 | 0 | 0 | 0 | 0 | Bacteria | Bacteria\_none | Bacillota | Tissierellia | Tissierellales | Tepidimicrobiaceae | Keratinibaculum | Bacteria;Bacteria\_none;Bacillota;Tissierellia;Tissierellales;Tepidimicrobiaceae;Keratinibaculum;Keratinibaculum paraultunense |
| Ezakiella coagulans | 0 | 0 | 0 | 1 | 0 | 0 | 0 | 0 | 0 | 0 | 7 | 1 | 0 | 1 | Bacteria | Bacteria\_none | Bacillota | Tissierellia | Tissierellia\_Incertae\_sedis | Tissierellia\_Incertae\_sedis | Ezakiella | Bacteria;Bacteria\_none;Bacillota;Tissierellia;Tissierellia\_Incertae\_sedis;Tissierellia\_Incertae\_sedis;Ezakiella;Ezakiella coagulans |
| Ezakiella massiliensis | 0 | 0 | 0 | 0 | 1 | 0 | 0 | 0 | 0 | 0 | 0 | 0 | 0 | 0 | Bacteria | Bacteria\_none | Bacillota | Tissierellia | Tissierellia\_Incertae\_sedis | Tissierellia\_Incertae\_sedis | Ezakiella | Bacteria;Bacteria\_none;Bacillota;Tissierellia;Tissierellia\_Incertae\_sedis;Tissierellia\_Incertae\_sedis;Ezakiella;Ezakiella massiliensis |
| Bacteroides caccae | 0 | 0 | 0 | 0 | 0 | 0 | 0 | 0 | 0 | 0 | 0 | 0 | 0 | 0 | Bacteria | Bacteria\_none | Bacteroidota | Bacteroidia | Bacteroidales | Bacteroidaceae | Bacteroides | Bacteria;Bacteria\_none;Bacteroidota;Bacteroidia;Bacteroidales;Bacteroidaceae;Bacteroides;Bacteroides caccae |
| Bacteroides caecimuris | 0 | 0 | 0 | 0 | 0 | 0 | 0 | 0 | 0 | 0 | 0 | 0 | 0 | 0 | Bacteria | Bacteria\_none | Bacteroidota | Bacteroidia | Bacteroidales | Bacteroidaceae | Bacteroides | Bacteria;Bacteria\_none;Bacteroidota;Bacteroidia;Bacteroidales;Bacteroidaceae;Bacteroides;Bacteroides caecimuris |
| Bacteroides cellulosilyticus | 2 | 0 | 0 | 1 | 0 | 0 | 0 | 0 | 0 | 0 | 0 | 0 | 0 | 0 | Bacteria | Bacteria\_none | Bacteroidota | Bacteroidia | Bacteroidales | Bacteroidaceae | Bacteroides | Bacteria;Bacteria\_none;Bacteroidota;Bacteroidia;Bacteroidales;Bacteroidaceae;Bacteroides;Bacteroides cellulosilyticus |
| Bacteroides eggerthii | 0 | 0 | 0 | 0 | 0 | 0 | 0 | 0 | 0 | 0 | 0 | 0 | 0 | 0 | Bacteria | Bacteria\_none | Bacteroidota | Bacteroidia | Bacteroidales | Bacteroidaceae | Bacteroides | Bacteria;Bacteria\_none;Bacteroidota;Bacteroidia;Bacteroidales;Bacteroidaceae;Bacteroides;Bacteroides eggerthii |
| Bacteroides faecium | 0 | 0 | 0 | 0 | 0 | 1 | 0 | 0 | 0 | 0 | 0 | 0 | 0 | 0 | Bacteria | Bacteria\_none | Bacteroidota | Bacteroidia | Bacteroidales | Bacteroidaceae | Bacteroides | Bacteria;Bacteria\_none;Bacteroidota;Bacteroidia;Bacteroidales;Bacteroidaceae;Bacteroides;Bacteroides faecium |
| Bacteroides fragilis | 0 | 0 | 0 | 0 | 0 | 0 | 0 | 0 | 0 | 0 | 1 | 0 | 0 | 0 | Bacteria | Bacteria\_none | Bacteroidota | Bacteroidia | Bacteroidales | Bacteroidaceae | Bacteroides | Bacteria;Bacteria\_none;Bacteroidota;Bacteroidia;Bacteroidales;Bacteroidaceae;Bacteroides;Bacteroides fragilis |
| Bacteroides graminisolvens | 0 | 0 | 0 | 0 | 0 | 0 | 0 | 0 | 0 | 0 | 0 | 0 | 0 | 0 | Bacteria | Bacteria\_none | Bacteroidota | Bacteroidia | Bacteroidales | Bacteroidaceae | Bacteroides | Bacteria;Bacteria\_none;Bacteroidota;Bacteroidia;Bacteroidales;Bacteroidaceae;Bacteroides;Bacteroides graminisolvens |
| Bacteroides helcogenes | 0 | 0 | 0 | 0 | 0 | 1 | 0 | 0 | 0 | 0 | 0 | 0 | 0 | 0 | Bacteria | Bacteria\_none | Bacteroidota | Bacteroidia | Bacteroidales | Bacteroidaceae | Bacteroides | Bacteria;Bacteria\_none;Bacteroidota;Bacteroidia;Bacteroidales;Bacteroidaceae;Bacteroides;Bacteroides helcogenes |
| Bacteroides heparinolyticus | 0 | 1 | 1 | 0 | 0 | 2 | 0 | 0 | 0 | 0 | 0 | 0 | 0 | 0 | Bacteria | Bacteria\_none | Bacteroidota | Bacteroidia | Bacteroidales | Bacteroidaceae | Bacteroides | Bacteria;Bacteria\_none;Bacteroidota;Bacteroidia;Bacteroidales;Bacteroidaceae;Bacteroides;Bacteroides heparinolyticus |
| Bacteroides humanifaecis | 0 | 0 | 0 | 3 | 0 | 0 | 0 | 0 | 0 | 0 | 0 | 0 | 0 | 0 | Bacteria | Bacteria\_none | Bacteroidota | Bacteroidia | Bacteroidales | Bacteroidaceae | Bacteroides | Bacteria;Bacteria\_none;Bacteroidota;Bacteroidia;Bacteroidales;Bacteroidaceae;Bacteroides;Bacteroides humanifaecis |
| Bacteroides intestinalis | 0 | 0 | 0 | 0 | 0 | 0 | 0 | 0 | 0 | 0 | 0 | 0 | 0 | 0 | Bacteria | Bacteria\_none | Bacteroidota | Bacteroidia | Bacteroidales | Bacteroidaceae | Bacteroides | Bacteria;Bacteria\_none;Bacteroidota;Bacteroidia;Bacteroidales;Bacteroidaceae;Bacteroides;Bacteroides intestinalis |
| Bacteroides ovatus | 0 | 0 | 0 | 0 | 0 | 1 | 0 | 0 | 0 | 0 | 0 | 0 | 0 | 0 | Bacteria | Bacteria\_none | Bacteroidota | Bacteroidia | Bacteroidales | Bacteroidaceae | Bacteroides | Bacteria;Bacteria\_none;Bacteroidota;Bacteroidia;Bacteroidales;Bacteroidaceae;Bacteroides;Bacteroides ovatus |
| Bacteroides sp. MSB163 | 0 | 0 | 0 | 0 | 0 | 0 | 0 | 0 | 0 | 0 | 0 | 0 | 0 | 0 | Bacteria | Bacteria\_none | Bacteroidota | Bacteroidia | Bacteroidales | Bacteroidaceae | Bacteroides | Bacteria;Bacteria\_none;Bacteroidota;Bacteroidia;Bacteroidales;Bacteroidaceae;Bacteroides;Bacteroides sp. MSB163 |
| Bacteroides thetaiotaomicron | 0 | 2 | 0 | 0 | 0 | 0 | 0 | 0 | 0 | 0 | 1 | 0 | 0 | 0 | Bacteria | Bacteria\_none | Bacteroidota | Bacteroidia | Bacteroidales | Bacteroidaceae | Bacteroides | Bacteria;Bacteria\_none;Bacteroidota;Bacteroidia;Bacteroidales;Bacteroidaceae;Bacteroides;Bacteroides thetaiotaomicron |
| Bacteroides uniformis | 0 | 0 | 0 | 0 | 0 | 1 | 0 | 0 | 0 | 0 | 0 | 0 | 0 | 0 | Bacteria | Bacteria\_none | Bacteroidota | Bacteroidia | Bacteroidales | Bacteroidaceae | Bacteroides | Bacteria;Bacteria\_none;Bacteroidota;Bacteroidia;Bacteroidales;Bacteroidaceae;Bacteroides;Bacteroides uniformis |
| Bacteroides xylanisolvens | 0 | 0 | 0 | 0 | 0 | 0 | 0 | 0 | 0 | 0 | 0 | 0 | 0 | 1 | Bacteria | Bacteria\_none | Bacteroidota | Bacteroidia | Bacteroidales | Bacteroidaceae | Bacteroides | Bacteria;Bacteria\_none;Bacteroidota;Bacteroidia;Bacteroidales;Bacteroidaceae;Bacteroides;Bacteroides xylanisolvens |
| Bacteroides zhangwenhongii | 0 | 0 | 0 | 0 | 0 | 0 | 0 | 0 | 0 | 0 | 0 | 0 | 0 | 0 | Bacteria | Bacteria\_none | Bacteroidota | Bacteroidia | Bacteroidales | Bacteroidaceae | Bacteroides | Bacteria;Bacteria\_none;Bacteroidota;Bacteroidia;Bacteroidales;Bacteroidaceae;Bacteroides;Bacteroides zhangwenhongii |
| Bacteroides zoogleoformans | 0 | 0 | 0 | 0 | 0 | 0 | 0 | 0 | 0 | 0 | 0 | 0 | 0 | 0 | Bacteria | Bacteria\_none | Bacteroidota | Bacteroidia | Bacteroidales | Bacteroidaceae | Bacteroides | Bacteria;Bacteria\_none;Bacteroidota;Bacteroidia;Bacteroidales;Bacteroidaceae;Bacteroides;Bacteroides zoogleoformans |
| Bacteroides\_uncultured | 0 | 0 | 0 | 0 | 0 | 0 | 0 | 0 | 0 | 1 | 1 | 0 | 0 | 0 | Bacteria | Bacteria\_none | Bacteroidota | Bacteroidia | Bacteroidales | Bacteroidaceae | Bacteroides | Bacteria;Bacteria\_none;Bacteroidota;Bacteroidia;Bacteroidales;Bacteroidaceae;Bacteroides;Bacteroides\_uncultured |
| Phocaeicola coprophilus | 0 | 0 | 0 | 0 | 0 | 1 | 0 | 0 | 0 | 0 | 0 | 0 | 0 | 0 | Bacteria | Bacteria\_none | Bacteroidota | Bacteroidia | Bacteroidales | Bacteroidaceae | Phocaeicola | Bacteria;Bacteria\_none;Bacteroidota;Bacteroidia;Bacteroidales;Bacteroidaceae;Phocaeicola;Phocaeicola coprophilus |
| Phocaeicola dorei | 0 | 0 | 0 | 0 | 0 | 0 | 0 | 0 | 0 | 0 | 0 | 0 | 0 | 0 | Bacteria | Bacteria\_none | Bacteroidota | Bacteroidia | Bacteroidales | Bacteroidaceae | Phocaeicola | Bacteria;Bacteria\_none;Bacteroidota;Bacteroidia;Bacteroidales;Bacteroidaceae;Phocaeicola;Phocaeicola dorei |
| Phocaeicola salanitronis | 0 | 0 | 0 | 0 | 0 | 0 | 0 | 0 | 0 | 0 | 0 | 0 | 0 | 0 | Bacteria | Bacteria\_none | Bacteroidota | Bacteroidia | Bacteroidales | Bacteroidaceae | Phocaeicola | Bacteria;Bacteria\_none;Bacteroidota;Bacteroidia;Bacteroidales;Bacteroidaceae;Phocaeicola;Phocaeicola salanitronis |
| Phocaeicola vulgatus | 0 | 0 | 0 | 0 | 1 | 0 | 0 | 0 | 0 | 0 | 1 | 0 | 0 | 0 | Bacteria | Bacteria\_none | Bacteroidota | Bacteroidia | Bacteroidales | Bacteroidaceae | Phocaeicola | Bacteria;Bacteria\_none;Bacteroidota;Bacteroidia;Bacteroidales;Bacteroidaceae;Phocaeicola;Phocaeicola vulgatus |
| Barnesiella sp. An22 | 0 | 0 | 0 | 0 | 0 | 0 | 0 | 0 | 0 | 0 | 0 | 0 | 0 | 0 | Bacteria | Bacteria\_none | Bacteroidota | Bacteroidia | Bacteroidales | Barnesiellaceae | Barnesiella | Bacteria;Bacteria\_none;Bacteroidota;Bacteroidia;Bacteroidales;Barnesiellaceae;Barnesiella;Barnesiella sp. An22 |
| Proteiniphilum saccharofermentans | 0 | 0 | 0 | 0 | 0 | 0 | 0 | 0 | 0 | 0 | 0 | 0 | 0 | 0 | Bacteria | Bacteria\_none | Bacteroidota | Bacteroidia | Bacteroidales | Dysgonomonadaceae | Proteiniphilum | Bacteria;Bacteria\_none;Bacteroidota;Bacteroidia;Bacteroidales;Dysgonomonadaceae;Proteiniphilum;Proteiniphilum saccharofermentans |
| Duncaniella dubosii | 0 | 0 | 0 | 0 | 0 | 0 | 0 | 0 | 0 | 0 | 0 | 0 | 0 | 0 | Bacteria | Bacteria\_none | Bacteroidota | Bacteroidia | Bacteroidales | Muribaculaceae | Duncaniella | Bacteria;Bacteria\_none;Bacteroidota;Bacteroidia;Bacteroidales;Muribaculaceae;Duncaniella;Duncaniella dubosii |
| Sodaliphilus pleomorphus | 0 | 0 | 0 | 0 | 0 | 0 | 0 | 0 | 0 | 0 | 0 | 0 | 0 | 0 | Bacteria | Bacteria\_none | Bacteroidota | Bacteroidia | Bacteroidales | Muribaculaceae | Sodaliphilus | Bacteria;Bacteria\_none;Bacteroidota;Bacteroidia;Bacteroidales;Muribaculaceae;Sodaliphilus;Sodaliphilus pleomorphus |
| Butyricimonas faecihominis | 0 | 0 | 0 | 0 | 0 | 0 | 0 | 0 | 0 | 0 | 0 | 0 | 0 | 0 | Bacteria | Bacteria\_none | Bacteroidota | Bacteroidia | Bacteroidales | Odoribacteraceae | Butyricimonas | Bacteria;Bacteria\_none;Bacteroidota;Bacteroidia;Bacteroidales;Odoribacteraceae;Butyricimonas;Butyricimonas faecihominis |
| Odoribacter splanchnicus | 0 | 0 | 0 | 0 | 0 | 0 | 0 | 0 | 0 | 0 | 0 | 0 | 0 | 0 | Bacteria | Bacteria\_none | Bacteroidota | Bacteroidia | Bacteroidales | Odoribacteraceae | Odoribacter | Bacteria;Bacteria\_none;Bacteroidota;Bacteroidia;Bacteroidales;Odoribacteraceae;Odoribacter;Odoribacter splanchnicus |
| Porphyromonas asaccharolytica | 0 | 0 | 0 | 0 | 0 | 0 | 0 | 0 | 0 | 1 | 0 | 0 | 0 | 0 | Bacteria | Bacteria\_none | Bacteroidota | Bacteroidia | Bacteroidales | Porphyromonadaceae | Porphyromonas | Bacteria;Bacteria\_none;Bacteroidota;Bacteroidia;Bacteroidales;Porphyromonadaceae;Porphyromonas;Porphyromonas asaccharolytica |
| Porphyromonas cangingivalis | 0 | 0 | 0 | 1 | 1 | 0 | 0 | 0 | 0 | 0 | 0 | 0 | 0 | 0 | Bacteria | Bacteria\_none | Bacteroidota | Bacteroidia | Bacteroidales | Porphyromonadaceae | Porphyromonas | Bacteria;Bacteria\_none;Bacteroidota;Bacteroidia;Bacteroidales;Porphyromonadaceae;Porphyromonas;Porphyromonas cangingivalis |
| Porphyromonas crevioricanis | 0 | 0 | 0 | 0 | 0 | 1 | 0 | 0 | 0 | 0 | 1 | 0 | 0 | 0 | Bacteria | Bacteria\_none | Bacteroidota | Bacteroidia | Bacteroidales | Porphyromonadaceae | Porphyromonas | Bacteria;Bacteria\_none;Bacteroidota;Bacteroidia;Bacteroidales;Porphyromonadaceae;Porphyromonas;Porphyromonas crevioricanis |
| Porphyromonas endodontalis | 2 | 102 | 3 | 11 | 12 | 3 | 4 | 18 | 0 | 6 | 28 | 4 | 4 | 8 | Bacteria | Bacteria\_none | Bacteroidota | Bacteroidia | Bacteroidales | Porphyromonadaceae | Porphyromonas | Bacteria;Bacteria\_none;Bacteroidota;Bacteroidia;Bacteroidales;Porphyromonadaceae;Porphyromonas;Porphyromonas endodontalis |
| Porphyromonas gingivalis | 0 | 7 | 4 | 1 | 1 | 4 | 0 | 5 | 0 | 1 | 2 | 14 | 3 | 3 | Bacteria | Bacteria\_none | Bacteroidota | Bacteroidia | Bacteroidales | Porphyromonadaceae | Porphyromonas | Bacteria;Bacteria\_none;Bacteroidota;Bacteroidia;Bacteroidales;Porphyromonadaceae;Porphyromonas;Porphyromonas gingivalis |
| Porphyromonas somerae | 0 | 0 | 0 | 0 | 0 | 0 | 0 | 0 | 0 | 1 | 0 | 0 | 0 | 0 | Bacteria | Bacteria\_none | Bacteroidota | Bacteroidia | Bacteroidales | Porphyromonadaceae | Porphyromonas | Bacteria;Bacteria\_none;Bacteroidota;Bacteroidia;Bacteroidales;Porphyromonadaceae;Porphyromonas;Porphyromonas somerae |
| Porphyromonas sp. oral taxon 275 | 3 | 11 | 2 | 16 | 17 | 17 | 1 | 0 | 0 | 18 | 1 | 2 | 1 | 0 | Bacteria | Bacteria\_none | Bacteroidota | Bacteroidia | Bacteroidales | Porphyromonadaceae | Porphyromonas | Bacteria;Bacteria\_none;Bacteroidota;Bacteroidia;Bacteroidales;Porphyromonadaceae;Porphyromonas;Porphyromonas sp. oral taxon 275 |
| Hoylesella buccalis | 1 | 0 | 0 | 2 | 1 | 0 | 0 | 0 | 0 | 0 | 0 | 0 | 0 | 0 | Bacteria | Bacteria\_none | Bacteroidota | Bacteroidia | Bacteroidales | Prevotellaceae | Hoylesella | Bacteria;Bacteria\_none;Bacteroidota;Bacteroidia;Bacteroidales;Prevotellaceae;Hoylesella;Hoylesella buccalis |
| Hoylesella enoeca | 0 | 4 | 3 | 1 | 0 | 4 | 1 | 2 | 0 | 0 | 2 | 4 | 3 | 0 | Bacteria | Bacteria\_none | Bacteroidota | Bacteroidia | Bacteroidales | Prevotellaceae | Hoylesella | Bacteria;Bacteria\_none;Bacteroidota;Bacteroidia;Bacteroidales;Prevotellaceae;Hoylesella;Hoylesella enoeca |
| Prevotella bivia | 3 | 3 | 1 | 4 | 0 | 0 | 0 | 1 | 0 | 0 | 0 | 0 | 1 | 0 | Bacteria | Bacteria\_none | Bacteroidota | Bacteroidia | Bacteroidales | Prevotellaceae | Prevotella | Bacteria;Bacteria\_none;Bacteroidota;Bacteroidia;Bacteroidales;Prevotellaceae;Prevotella;Prevotella bivia |
| Prevotella communis | 0 | 0 | 0 | 0 | 0 | 0 | 0 | 0 | 0 | 0 | 0 | 0 | 0 | 0 | Bacteria | Bacteria\_none | Bacteroidota | Bacteroidia | Bacteroidales | Prevotellaceae | Prevotella | Bacteria;Bacteria\_none;Bacteroidota;Bacteroidia;Bacteroidales;Prevotellaceae;Prevotella;Prevotella communis |
| Prevotella corporis | 3 | 2 | 2 | 1 | 0 | 0 | 1 | 0 | 0 | 0 | 0 | 0 | 0 | 1 | Bacteria | Bacteria\_none | Bacteroidota | Bacteroidia | Bacteroidales | Prevotellaceae | Prevotella | Bacteria;Bacteria\_none;Bacteroidota;Bacteroidia;Bacteroidales;Prevotellaceae;Prevotella;Prevotella corporis |
| Prevotella dentalis | 0 | 1 | 0 | 1 | 0 | 0 | 0 | 0 | 0 | 0 | 0 | 0 | 0 | 0 | Bacteria | Bacteria\_none | Bacteroidota | Bacteroidia | Bacteroidales | Prevotellaceae | Prevotella | Bacteria;Bacteria\_none;Bacteroidota;Bacteroidia;Bacteroidales;Prevotellaceae;Prevotella;Prevotella dentalis |
| Prevotella denticola | 1 | 13 | 6 | 10 | 2 | 0 | 1 | 1 | 0 | 0 | 6 | 7 | 2 | 2 | Bacteria | Bacteria\_none | Bacteroidota | Bacteroidia | Bacteroidales | Prevotellaceae | Prevotella | Bacteria;Bacteria\_none;Bacteroidota;Bacteroidia;Bacteroidales;Prevotellaceae;Prevotella;Prevotella denticola |
| Prevotella fusca | 4 | 1 | 0 | 3 | 0 | 0 | 0 | 5 | 0 | 0 | 0 | 1 | 0 | 0 | Bacteria | Bacteria\_none | Bacteroidota | Bacteroidia | Bacteroidales | Prevotellaceae | Prevotella | Bacteria;Bacteria\_none;Bacteroidota;Bacteroidia;Bacteroidales;Prevotellaceae;Prevotella;Prevotella fusca |
| Prevotella herbatica | 0 | 1 | 0 | 0 | 0 | 0 | 0 | 0 | 0 | 0 | 0 | 0 | 0 | 0 | Bacteria | Bacteria\_none | Bacteroidota | Bacteroidia | Bacteroidales | Prevotellaceae | Prevotella | Bacteria;Bacteria\_none;Bacteroidota;Bacteroidia;Bacteroidales;Prevotellaceae;Prevotella;Prevotella herbatica |
| Prevotella histicola | 0 | 1 | 1 | 3 | 0 | 3 | 1 | 0 | 0 | 0 | 0 | 5 | 2 | 1 | Bacteria | Bacteria\_none | Bacteroidota | Bacteroidia | Bacteroidales | Prevotellaceae | Prevotella | Bacteria;Bacteria\_none;Bacteroidota;Bacteroidia;Bacteroidales;Prevotellaceae;Prevotella;Prevotella histicola |
| Prevotella intermedia | 9 | 30 | 12 | 32 | 1 | 2 | 3 | 0 | 2 | 4 | 8 | 16 | 3 | 3 | Bacteria | Bacteria\_none | Bacteroidota | Bacteroidia | Bacteroidales | Prevotellaceae | Prevotella | Bacteria;Bacteria\_none;Bacteroidota;Bacteroidia;Bacteroidales;Prevotellaceae;Prevotella;Prevotella intermedia |
| Prevotella jejuni | 43 | 116 | 6 | 14 | 1 | 32 | 16 | 0 | 95 | 3 | 7 | 3 | 24 | 11 | Bacteria | Bacteria\_none | Bacteroidota | Bacteroidia | Bacteroidales | Prevotellaceae | Prevotella | Bacteria;Bacteria\_none;Bacteroidota;Bacteroidia;Bacteroidales;Prevotellaceae;Prevotella;Prevotella jejuni |
| Prevotella melaninogenica | 17 | 651 | 31 | 90 | 23 | 89 | 3 | 3 | 16 | 40 | 55 | 61 | 58 | 31 | Bacteria | Bacteria\_none | Bacteroidota | Bacteroidia | Bacteroidales | Prevotellaceae | Prevotella | Bacteria;Bacteria\_none;Bacteroidota;Bacteroidia;Bacteroidales;Prevotellaceae;Prevotella;Prevotella melaninogenica |
| Prevotella multiformis | 0 | 3 | 1 | 1 | 0 | 1 | 0 | 0 | 0 | 1 | 0 | 0 | 0 | 0 | Bacteria | Bacteria\_none | Bacteroidota | Bacteroidia | Bacteroidales | Prevotellaceae | Prevotella | Bacteria;Bacteria\_none;Bacteroidota;Bacteroidia;Bacteroidales;Prevotellaceae;Prevotella;Prevotella multiformis |
| Prevotella nigrescens | 7 | 35 | 44 | 83 | 6 | 3 | 1 | 0 | 0 | 2 | 5 | 16 | 16 | 4 | Bacteria | Bacteria\_none | Bacteroidota | Bacteroidia | Bacteroidales | Prevotellaceae | Prevotella | Bacteria;Bacteria\_none;Bacteroidota;Bacteroidia;Bacteroidales;Prevotellaceae;Prevotella;Prevotella nigrescens |
| Prevotella scopos | 10 | 7 | 0 | 1 | 0 | 2 | 5 | 0 | 3 | 0 | 0 | 0 | 2 | 1 | Bacteria | Bacteria\_none | Bacteroidota | Bacteroidia | Bacteroidales | Prevotellaceae | Prevotella | Bacteria;Bacteria\_none;Bacteroidota;Bacteroidia;Bacteroidales;Prevotellaceae;Prevotella;Prevotella scopos |
| Prevotella sp. E15-22 | 0 | 0 | 0 | 0 | 0 | 1 | 0 | 0 | 0 | 0 | 0 | 0 | 0 | 0 | Bacteria | Bacteria\_none | Bacteroidota | Bacteroidia | Bacteroidales | Prevotellaceae | Prevotella | Bacteria;Bacteria\_none;Bacteroidota;Bacteroidia;Bacteroidales;Prevotellaceae;Prevotella;Prevotella sp. E15-22 |
| Prevotella sp. E2-28 | 0 | 0 | 0 | 1 | 0 | 0 | 0 | 0 | 0 | 0 | 0 | 0 | 0 | 0 | Bacteria | Bacteria\_none | Bacteroidota | Bacteroidia | Bacteroidales | Prevotellaceae | Prevotella | Bacteria;Bacteria\_none;Bacteroidota;Bacteroidia;Bacteroidales;Prevotellaceae;Prevotella;Prevotella sp. E2-28 |
| Prevotella sp. Rep29 | 0 | 0 | 0 | 0 | 0 | 0 | 0 | 0 | 0 | 0 | 0 | 0 | 0 | 0 | Bacteria | Bacteria\_none | Bacteroidota | Bacteroidia | Bacteroidales | Prevotellaceae | Prevotella | Bacteria;Bacteria\_none;Bacteroidota;Bacteroidia;Bacteroidales;Prevotellaceae;Prevotella;Prevotella sp. Rep29 |
| Prevotella sp. oral taxon 299 | 192 | 14 | 132 | 196 | 9 | 2 | 11 | 0 | 1 | 15 | 0 | 1 | 41 | 14 | Bacteria | Bacteria\_none | Bacteroidota | Bacteroidia | Bacteroidales | Prevotellaceae | Prevotella | Bacteria;Bacteria\_none;Bacteroidota;Bacteroidia;Bacteroidales;Prevotellaceae;Prevotella;Prevotella sp. oral taxon 299 |
| Prevotella sp. oral taxon 313 | 0 | 0 | 1 | 0 | 0 | 0 | 0 | 0 | 0 | 0 | 0 | 0 | 0 | 0 | Bacteria | Bacteria\_none | Bacteroidota | Bacteroidia | Bacteroidales | Prevotellaceae | Prevotella | Bacteria;Bacteria\_none;Bacteroidota;Bacteroidia;Bacteroidales;Prevotellaceae;Prevotella;Prevotella sp. oral taxon 313 |
| Prevotella sp. oral taxon 475 | 0 | 1 | 1 | 0 | 2 | 1 | 0 | 0 | 0 | 1 | 3 | 0 | 0 | 0 | Bacteria | Bacteria\_none | Bacteroidota | Bacteroidia | Bacteroidales | Prevotellaceae | Prevotella | Bacteria;Bacteria\_none;Bacteroidota;Bacteroidia;Bacteroidales;Prevotellaceae;Prevotella;Prevotella sp. oral taxon 475 |
| Prevotella veroralis | 0 | 49 | 2 | 24 | 0 | 23 | 0 | 0 | 2 | 0 | 0 | 9 | 3 | 1 | Bacteria | Bacteria\_none | Bacteroidota | Bacteroidia | Bacteroidales | Prevotellaceae | Prevotella | Bacteria;Bacteria\_none;Bacteroidota;Bacteroidia;Bacteroidales;Prevotellaceae;Prevotella;Prevotella veroralis |
| Segatella copri | 0 | 1 | 0 | 0 | 0 | 0 | 0 | 0 | 0 | 0 | 0 | 0 | 0 | 0 | Bacteria | Bacteria\_none | Bacteroidota | Bacteroidia | Bacteroidales | Prevotellaceae | Segatella | Bacteria;Bacteria\_none;Bacteroidota;Bacteroidia;Bacteroidales;Prevotellaceae;Segatella;Segatella copri |
| Segatella oris | 1 | 5 | 11 | 8 | 6 | 2 | 6 | 4 | 0 | 8 | 16 | 11 | 2 | 1 | Bacteria | Bacteria\_none | Bacteroidota | Bacteroidia | Bacteroidales | Prevotellaceae | Segatella | Bacteria;Bacteria\_none;Bacteroidota;Bacteroidia;Bacteroidales;Prevotellaceae;Segatella;Segatella oris |
| Xylanibacter ruminicola | 0 | 0 | 0 | 0 | 0 | 0 | 1 | 0 | 0 | 0 | 0 | 0 | 0 | 1 | Bacteria | Bacteria\_none | Bacteroidota | Bacteroidia | Bacteroidales | Prevotellaceae | Xylanibacter | Bacteria;Bacteria\_none;Bacteroidota;Bacteroidia;Bacteroidales;Prevotellaceae;Xylanibacter;Xylanibacter ruminicola |
| Alistipes communis | 0 | 0 | 0 | 0 | 0 | 0 | 0 | 0 | 0 | 0 | 0 | 0 | 1 | 0 | Bacteria | Bacteria\_none | Bacteroidota | Bacteroidia | Bacteroidales | Rikenellaceae | Alistipes | Bacteria;Bacteria\_none;Bacteroidota;Bacteroidia;Bacteroidales;Rikenellaceae;Alistipes;Alistipes communis |
| Alistipes finegoldii | 0 | 1 | 0 | 0 | 0 | 1 | 0 | 0 | 0 | 0 | 0 | 0 | 0 | 0 | Bacteria | Bacteria\_none | Bacteroidota | Bacteroidia | Bacteroidales | Rikenellaceae | Alistipes | Bacteria;Bacteria\_none;Bacteroidota;Bacteroidia;Bacteroidales;Rikenellaceae;Alistipes;Alistipes finegoldii |
| Alistipes onderdonkii | 0 | 1 | 0 | 0 | 0 | 0 | 0 | 0 | 0 | 0 | 0 | 0 | 0 | 0 | Bacteria | Bacteria\_none | Bacteroidota | Bacteroidia | Bacteroidales | Rikenellaceae | Alistipes | Bacteria;Bacteria\_none;Bacteroidota;Bacteroidia;Bacteroidales;Rikenellaceae;Alistipes;Alistipes onderdonkii |
| Alistipes shahii | 0 | 0 | 0 | 0 | 0 | 0 | 0 | 0 | 0 | 0 | 0 | 0 | 0 | 0 | Bacteria | Bacteria\_none | Bacteroidota | Bacteroidia | Bacteroidales | Rikenellaceae | Alistipes | Bacteria;Bacteria\_none;Bacteroidota;Bacteroidia;Bacteroidales;Rikenellaceae;Alistipes;Alistipes shahii |
| Alistipes sp. dk3624 | 0 | 0 | 0 | 0 | 0 | 0 | 0 | 0 | 0 | 1 | 0 | 0 | 0 | 0 | Bacteria | Bacteria\_none | Bacteroidota | Bacteroidia | Bacteroidales | Rikenellaceae | Alistipes | Bacteria;Bacteria\_none;Bacteroidota;Bacteroidia;Bacteroidales;Rikenellaceae;Alistipes;Alistipes sp. dk3624 |
| Salinivirga cyanobacteriivorans | 0 | 0 | 0 | 0 | 0 | 0 | 0 | 0 | 0 | 0 | 0 | 0 | 0 | 0 | Bacteria | Bacteria\_none | Bacteroidota | Bacteroidia | Bacteroidales | Salinivirgaceae | Salinivirga | Bacteria;Bacteria\_none;Bacteroidota;Bacteroidia;Bacteroidales;Salinivirgaceae;Salinivirga;Salinivirga cyanobacteriivorans |
| Parabacteroides chongii | 0 | 0 | 0 | 0 | 0 | 0 | 0 | 0 | 0 | 0 | 0 | 0 | 0 | 0 | Bacteria | Bacteria\_none | Bacteroidota | Bacteroidia | Bacteroidales | Tannerellaceae | Parabacteroides | Bacteria;Bacteria\_none;Bacteroidota;Bacteroidia;Bacteroidales;Tannerellaceae;Parabacteroides;Parabacteroides chongii |
| Parabacteroides distasonis | 0 | 0 | 0 | 0 | 0 | 0 | 0 | 0 | 0 | 0 | 0 | 0 | 0 | 0 | Bacteria | Bacteria\_none | Bacteroidota | Bacteroidia | Bacteroidales | Tannerellaceae | Parabacteroides | Bacteria;Bacteria\_none;Bacteroidota;Bacteroidia;Bacteroidales;Tannerellaceae;Parabacteroides;Parabacteroides distasonis |
| Parabacteroides faecis | 0 | 0 | 0 | 0 | 0 | 0 | 0 | 0 | 0 | 0 | 0 | 0 | 0 | 0 | Bacteria | Bacteria\_none | Bacteroidota | Bacteroidia | Bacteroidales | Tannerellaceae | Parabacteroides | Bacteria;Bacteria\_none;Bacteroidota;Bacteroidia;Bacteroidales;Tannerellaceae;Parabacteroides;Parabacteroides faecis |
| Parabacteroides goldsteinii | 0 | 0 | 0 | 0 | 0 | 0 | 0 | 0 | 0 | 0 | 1 | 0 | 0 | 0 | Bacteria | Bacteria\_none | Bacteroidota | Bacteroidia | Bacteroidales | Tannerellaceae | Parabacteroides | Bacteria;Bacteria\_none;Bacteroidota;Bacteroidia;Bacteroidales;Tannerellaceae;Parabacteroides;Parabacteroides goldsteinii |
| Tannerella forsythia | 1 | 18 | 27 | 4 | 7 | 9 | 2 | 11 | 0 | 9 | 10 | 10 | 4 | 4 | Bacteria | Bacteria\_none | Bacteroidota | Bacteroidia | Bacteroidales | Tannerellaceae | Tannerella | Bacteria;Bacteria\_none;Bacteroidota;Bacteroidia;Bacteroidales;Tannerellaceae;Tannerella;Tannerella forsythia |
| Tannerella serpentiformis | 0 | 17 | 4 | 2 | 7 | 65 | 0 | 0 | 0 | 11 | 12 | 1 | 8 | 2 | Bacteria | Bacteria\_none | Bacteroidota | Bacteroidia | Bacteroidales | Tannerellaceae | Tannerella | Bacteria;Bacteria\_none;Bacteroidota;Bacteroidia;Bacteroidales;Tannerellaceae;Tannerella;Tannerella serpentiformis |
| Marinifilum fragile | 0 | 0 | 1 | 0 | 0 | 0 | 0 | 0 | 0 | 0 | 0 | 0 | 0 | 0 | Bacteria | Bacteria\_none | Bacteroidota | Bacteroidia | Marinilabiliales | Marinifilaceae | Marinifilum | Bacteria;Bacteria\_none;Bacteroidota;Bacteroidia;Marinilabiliales;Marinifilaceae;Marinifilum;Marinifilum fragile |
| Marinifilum\_uncultured | 1 | 0 | 0 | 0 | 0 | 0 | 0 | 0 | 0 | 0 | 0 | 0 | 0 | 0 | Bacteria | Bacteria\_none | Bacteroidota | Bacteroidia | Marinilabiliales | Marinifilaceae | Marinifilum | Bacteria;Bacteria\_none;Bacteroidota;Bacteroidia;Marinilabiliales;Marinifilaceae;Marinifilum;Marinifilum\_uncultured |
| Alkalitalea saponilacus | 0 | 0 | 0 | 0 | 0 | 0 | 0 | 0 | 0 | 0 | 0 | 0 | 0 | 0 | Bacteria | Bacteria\_none | Bacteroidota | Bacteroidia | Marinilabiliales | Marinilabiliaceae | Alkalitalea | Bacteria;Bacteria\_none;Bacteroidota;Bacteroidia;Marinilabiliales;Marinilabiliaceae;Alkalitalea;Alkalitalea saponilacus |
| Carboxylicivirga\_uncultured | 0 | 0 | 1 | 0 | 0 | 0 | 0 | 1 | 0 | 0 | 0 | 0 | 0 | 0 | Bacteria | Bacteria\_none | Bacteroidota | Bacteroidia | Marinilabiliales | Marinilabiliaceae | Carboxylicivirga | Bacteria;Bacteria\_none;Bacteroidota;Bacteroidia;Marinilabiliales;Marinilabiliaceae;Carboxylicivirga;Carboxylicivirga\_uncultured |
| Draconibacterium\_uncultured | 0 | 0 | 0 | 0 | 0 | 0 | 0 | 0 | 0 | 0 | 0 | 0 | 0 | 0 | Bacteria | Bacteria\_none | Bacteroidota | Bacteroidia | Marinilabiliales | Prolixibacteraceae | Draconibacterium | Bacteria;Bacteria\_none;Bacteroidota;Bacteroidia;Marinilabiliales;Prolixibacteraceae;Draconibacterium;Draconibacterium\_uncultured |
| Mangrovibacterium marinum | 0 | 0 | 0 | 0 | 0 | 1 | 0 | 0 | 0 | 0 | 0 | 0 | 0 | 0 | Bacteria | Bacteria\_none | Bacteroidota | Bacteroidia | Marinilabiliales | Prolixibacteraceae | Mangrovibacterium | Bacteria;Bacteria\_none;Bacteroidota;Bacteroidia;Marinilabiliales;Prolixibacteraceae;Mangrovibacterium;Mangrovibacterium marinum |
| Maribellus comscasis | 0 | 0 | 0 | 1 | 0 | 0 | 0 | 0 | 0 | 0 | 0 | 0 | 0 | 0 | Bacteria | Bacteria\_none | Bacteroidota | Bacteroidia | Marinilabiliales | Prolixibacteraceae | Maribellus | Bacteria;Bacteria\_none;Bacteroidota;Bacteroidia;Marinilabiliales;Prolixibacteraceae;Maribellus;Maribellus comscasis |
| Chitinophaga agri | 0 | 0 | 0 | 0 | 0 | 0 | 0 | 0 | 0 | 0 | 0 | 0 | 0 | 0 | Bacteria | Bacteria\_none | Bacteroidota | Chitinophagia | Chitinophagales | Chitinophagaceae | Chitinophaga | Bacteria;Bacteria\_none;Bacteroidota;Chitinophagia;Chitinophagales;Chitinophagaceae;Chitinophaga;Chitinophaga agri |
| Chitinophaga pollutisoli | 0 | 0 | 0 | 0 | 1 | 0 | 0 | 0 | 0 | 0 | 0 | 0 | 0 | 0 | Bacteria | Bacteria\_none | Bacteroidota | Chitinophagia | Chitinophagales | Chitinophagaceae | Chitinophaga | Bacteria;Bacteria\_none;Bacteroidota;Chitinophagia;Chitinophagales;Chitinophagaceae;Chitinophaga;Chitinophaga pollutisoli |
| Chitinophaga sancti | 0 | 0 | 0 | 0 | 1 | 0 | 0 | 0 | 0 | 0 | 0 | 0 | 0 | 0 | Bacteria | Bacteria\_none | Bacteroidota | Chitinophagia | Chitinophagales | Chitinophagaceae | Chitinophaga | Bacteria;Bacteria\_none;Bacteroidota;Chitinophagia;Chitinophagales;Chitinophagaceae;Chitinophaga;Chitinophaga sancti |
| Chitinophaga sp. HK235 | 0 | 1 | 0 | 0 | 0 | 0 | 0 | 0 | 0 | 0 | 0 | 0 | 0 | 0 | Bacteria | Bacteria\_none | Bacteroidota | Chitinophagia | Chitinophagales | Chitinophagaceae | Chitinophaga | Bacteria;Bacteria\_none;Bacteroidota;Chitinophagia;Chitinophagales;Chitinophagaceae;Chitinophaga;Chitinophaga sp. HK235 |
| Chitinophaga sp. XS-30 | 0 | 0 | 0 | 1 | 0 | 0 | 0 | 0 | 0 | 0 | 0 | 0 | 0 | 0 | Bacteria | Bacteria\_none | Bacteroidota | Chitinophagia | Chitinophagales | Chitinophagaceae | Chitinophaga | Bacteria;Bacteria\_none;Bacteroidota;Chitinophagia;Chitinophagales;Chitinophagaceae;Chitinophaga;Chitinophaga sp. XS-30 |
| Ferruginibacter lapsinanis | 0 | 0 | 0 | 0 | 0 | 1 | 0 | 0 | 0 | 0 | 0 | 0 | 0 | 0 | Bacteria | Bacteria\_none | Bacteroidota | Chitinophagia | Chitinophagales | Chitinophagaceae | Ferruginibacter | Bacteria;Bacteria\_none;Bacteroidota;Chitinophagia;Chitinophagales;Chitinophagaceae;Ferruginibacter;Ferruginibacter lapsinanis |
| Flavihumibacter fluvii | 0 | 0 | 0 | 1 | 0 | 0 | 0 | 0 | 0 | 0 | 0 | 0 | 0 | 0 | Bacteria | Bacteria\_none | Bacteroidota | Chitinophagia | Chitinophagales | Chitinophagaceae | Flavihumibacter | Bacteria;Bacteria\_none;Bacteroidota;Chitinophagia;Chitinophagales;Chitinophagaceae;Flavihumibacter;Flavihumibacter fluvii |
| Pseudobacter ginsenosidimutans | 0 | 0 | 0 | 0 | 0 | 0 | 0 | 0 | 0 | 0 | 0 | 1 | 0 | 0 | Bacteria | Bacteria\_none | Bacteroidota | Chitinophagia | Chitinophagales | Chitinophagaceae | Pseudobacter | Bacteria;Bacteria\_none;Bacteroidota;Chitinophagia;Chitinophagales;Chitinophagaceae;Pseudobacter;Pseudobacter ginsenosidimutans |
| Cardinium endosymbiont of Tipula unca | 0 | 0 | 0 | 0 | 0 | 0 | 0 | 0 | 0 | 0 | 0 | 0 | 0 | 0 | Bacteria | Bacteria\_none | Bacteroidota | Cytophagia | Cytophagales | Amoebophilaceae | Candidatus Cardinium | Bacteria;Bacteria\_none;Bacteroidota;Cytophagia;Cytophagales;Amoebophilaceae;Candidatus Cardinium;Cardinium endosymbiont of Tipula unca |
| Algoriphagus sanaruensis | 0 | 0 | 0 | 0 | 0 | 0 | 0 | 0 | 0 | 0 | 0 | 0 | 0 | 0 | Bacteria | Bacteria\_none | Bacteroidota | Cytophagia | Cytophagales | Cyclobacteriaceae | Algoriphagus | Bacteria;Bacteria\_none;Bacteroidota;Cytophagia;Cytophagales;Cyclobacteriaceae;Algoriphagus;Algoriphagus sanaruensis |
| Cyclobacterium amurskyense | 0 | 0 | 0 | 0 | 0 | 0 | 0 | 0 | 0 | 0 | 0 | 0 | 0 | 0 | Bacteria | Bacteria\_none | Bacteroidota | Cytophagia | Cytophagales | Cyclobacteriaceae | Cyclobacterium | Bacteria;Bacteria\_none;Bacteroidota;Cytophagia;Cytophagales;Cyclobacteriaceae;Cyclobacterium;Cyclobacterium amurskyense |
| Cyclobacterium marinum | 0 | 0 | 0 | 0 | 0 | 0 | 0 | 0 | 0 | 0 | 0 | 0 | 0 | 1 | Bacteria | Bacteria\_none | Bacteroidota | Cytophagia | Cytophagales | Cyclobacteriaceae | Cyclobacterium | Bacteria;Bacteria\_none;Bacteroidota;Cytophagia;Cytophagales;Cyclobacteriaceae;Cyclobacterium;Cyclobacterium marinum |
| Spirosoma sp. KCTC 42546 | 0 | 0 | 0 | 0 | 0 | 0 | 0 | 0 | 0 | 0 | 0 | 0 | 0 | 0 | Bacteria | Bacteria\_none | Bacteroidota | Cytophagia | Cytophagales | Cytophagaceae | Spirosoma | Bacteria;Bacteria\_none;Bacteroidota;Cytophagia;Cytophagales;Cytophagaceae;Spirosoma;Spirosoma sp. KCTC 42546 |
| Flammeovirga pectinis | 0 | 0 | 0 | 0 | 1 | 0 | 0 | 0 | 0 | 0 | 0 | 0 | 1 | 0 | Bacteria | Bacteria\_none | Bacteroidota | Cytophagia | Cytophagales | Flammeovirgaceae | Flammeovirga | Bacteria;Bacteria\_none;Bacteroidota;Cytophagia;Cytophagales;Flammeovirgaceae;Flammeovirga;Flammeovirga pectinis |
| Hymenobacter jejuensis | 0 | 0 | 0 | 0 | 1 | 0 | 0 | 0 | 0 | 0 | 0 | 0 | 0 | 0 | Bacteria | Bacteria\_none | Bacteroidota | Cytophagia | Cytophagales | Hymenobacteraceae | Hymenobacter | Bacteria;Bacteria\_none;Bacteroidota;Cytophagia;Cytophagales;Hymenobacteraceae;Hymenobacter;Hymenobacter jejuensis |
| Hymenobacter sp. BRD128 | 0 | 0 | 0 | 0 | 0 | 0 | 0 | 0 | 0 | 0 | 0 | 0 | 0 | 0 | Bacteria | Bacteria\_none | Bacteroidota | Cytophagia | Cytophagales | Hymenobacteraceae | Hymenobacter | Bacteria;Bacteria\_none;Bacteroidota;Cytophagia;Cytophagales;Hymenobacteraceae;Hymenobacter;Hymenobacter sp. BRD128 |
| Hymenobacter sp. BT18 | 0 | 0 | 0 | 0 | 0 | 0 | 0 | 0 | 0 | 0 | 0 | 0 | 0 | 0 | Bacteria | Bacteria\_none | Bacteroidota | Cytophagia | Cytophagales | Hymenobacteraceae | Hymenobacter | Bacteria;Bacteria\_none;Bacteroidota;Cytophagia;Cytophagales;Hymenobacteraceae;Hymenobacter;Hymenobacter sp. BT18 |
| Hymenobacter sp. YIM 151500-1 | 0 | 0 | 0 | 0 | 0 | 0 | 0 | 0 | 0 | 0 | 0 | 0 | 0 | 0 | Bacteria | Bacteria\_none | Bacteroidota | Cytophagia | Cytophagales | Hymenobacteraceae | Hymenobacter | Bacteria;Bacteria\_none;Bacteroidota;Cytophagia;Cytophagales;Hymenobacteraceae;Hymenobacter;Hymenobacter sp. YIM 151500-1 |
| Hymenobacter swuensis | 0 | 0 | 0 | 0 | 0 | 0 | 0 | 0 | 0 | 0 | 0 | 0 | 0 | 0 | Bacteria | Bacteria\_none | Bacteroidota | Cytophagia | Cytophagales | Hymenobacteraceae | Hymenobacter | Bacteria;Bacteria\_none;Bacteroidota;Cytophagia;Cytophagales;Hymenobacteraceae;Hymenobacter;Hymenobacter swuensis |
| Hymenobacter volaticus | 0 | 0 | 0 | 0 | 0 | 0 | 0 | 0 | 0 | 0 | 0 | 0 | 0 | 0 | Bacteria | Bacteria\_none | Bacteroidota | Cytophagia | Cytophagales | Hymenobacteraceae | Hymenobacter | Bacteria;Bacteria\_none;Bacteroidota;Cytophagia;Cytophagales;Hymenobacteraceae;Hymenobacter;Hymenobacter volaticus |
| Hymenobacter yonginensis | 0 | 0 | 0 | 0 | 0 | 0 | 0 | 0 | 0 | 0 | 0 | 0 | 0 | 0 | Bacteria | Bacteria\_none | Bacteroidota | Cytophagia | Cytophagales | Hymenobacteraceae | Hymenobacter | Bacteria;Bacteria\_none;Bacteroidota;Cytophagia;Cytophagales;Hymenobacteraceae;Hymenobacter;Hymenobacter yonginensis |
| Rufibacter radiotolerans | 0 | 0 | 0 | 0 | 0 | 0 | 0 | 0 | 0 | 0 | 0 | 0 | 0 | 0 | Bacteria | Bacteria\_none | Bacteroidota | Cytophagia | Cytophagales | Hymenobacteraceae | Rufibacter | Bacteria;Bacteria\_none;Bacteroidota;Cytophagia;Cytophagales;Hymenobacteraceae;Rufibacter;Rufibacter radiotolerans |
| Arcticibacterium luteifluviistationis | 0 | 0 | 1 | 0 | 0 | 0 | 0 | 0 | 0 | 0 | 0 | 0 | 0 | 0 | Bacteria | Bacteria\_none | Bacteroidota | Cytophagia | Cytophagales | Leadbetterellaceae | Arcticibacterium | Bacteria;Bacteria\_none;Bacteroidota;Cytophagia;Cytophagales;Leadbetterellaceae;Arcticibacterium;Arcticibacterium luteifluviistationis |
| Emticicia oligotrophica | 0 | 0 | 0 | 0 | 0 | 0 | 0 | 0 | 0 | 0 | 0 | 0 | 0 | 0 | Bacteria | Bacteria\_none | Bacteroidota | Cytophagia | Cytophagales | Leadbetterellaceae | Emticicia | Bacteria;Bacteria\_none;Bacteroidota;Cytophagia;Cytophagales;Leadbetterellaceae;Emticicia;Emticicia oligotrophica |
| Marivirga salinae | 0 | 0 | 0 | 0 | 0 | 1 | 0 | 0 | 0 | 0 | 0 | 0 | 0 | 0 | Bacteria | Bacteria\_none | Bacteroidota | Cytophagia | Cytophagales | Marivirgaceae | Marivirga | Bacteria;Bacteria\_none;Bacteroidota;Cytophagia;Cytophagales;Marivirgaceae;Marivirga;Marivirga salinae |
| Fulvitalea axinellae | 0 | 0 | 0 | 1 | 1 | 0 | 0 | 0 | 0 | 0 | 0 | 0 | 0 | 0 | Bacteria | Bacteria\_none | Bacteroidota | Cytophagia | Cytophagales | Persicobacteraceae | Fulvitalea | Bacteria;Bacteria\_none;Bacteroidota;Cytophagia;Cytophagales;Persicobacteraceae;Fulvitalea;Fulvitalea axinellae |
| Dyadobacter chenwenxiniae | 0 | 0 | 0 | 0 | 0 | 0 | 0 | 0 | 0 | 0 | 0 | 0 | 0 | 0 | Bacteria | Bacteria\_none | Bacteroidota | Cytophagia | Cytophagales | Spirosomataceae | Dyadobacter | Bacteria;Bacteria\_none;Bacteroidota;Cytophagia;Cytophagales;Spirosomataceae;Dyadobacter;Dyadobacter chenwenxiniae |
| Dyadobacter pollutisoli | 0 | 0 | 0 | 0 | 0 | 1 | 0 | 0 | 0 | 0 | 0 | 0 | 1 | 0 | Bacteria | Bacteria\_none | Bacteroidota | Cytophagia | Cytophagales | Spirosomataceae | Dyadobacter | Bacteria;Bacteria\_none;Bacteroidota;Cytophagia;Cytophagales;Spirosomataceae;Dyadobacter;Dyadobacter pollutisoli |
| Dyadobacter sp. NIV53 | 0 | 0 | 0 | 0 | 0 | 0 | 0 | 0 | 0 | 0 | 0 | 0 | 0 | 0 | Bacteria | Bacteria\_none | Bacteroidota | Cytophagia | Cytophagales | Spirosomataceae | Dyadobacter | Bacteria;Bacteria\_none;Bacteroidota;Cytophagia;Cytophagales;Spirosomataceae;Dyadobacter;Dyadobacter sp. NIV53 |
| Fibrella aestuarina | 0 | 0 | 0 | 0 | 0 | 0 | 0 | 0 | 0 | 0 | 0 | 0 | 0 | 0 | Bacteria | Bacteria\_none | Bacteroidota | Cytophagia | Cytophagales | Spirosomataceae | Fibrella | Bacteria;Bacteria\_none;Bacteroidota;Cytophagia;Cytophagales;Spirosomataceae;Fibrella;Fibrella aestuarina |
| Blattabacterium cuenoti | 0 | 0 | 0 | 0 | 0 | 0 | 0 | 0 | 0 | 0 | 1 | 0 | 0 | 0 | Bacteria | Bacteria\_none | Bacteroidota | Flavobacteriia | Flavobacteriales | Blattabacteriaceae | Blattabacterium | Bacteria;Bacteria\_none;Bacteroidota;Flavobacteriia;Flavobacteriales;Blattabacteriaceae;Blattabacterium;Blattabacterium cuenoti |
| Paracrocinitomix mangrovi | 0 | 0 | 0 | 0 | 0 | 0 | 0 | 0 | 0 | 1 | 0 | 0 | 0 | 0 | Bacteria | Bacteria\_none | Bacteroidota | Flavobacteriia | Flavobacteriales | Crocinitomicaceae | Paracrocinitomix | Bacteria;Bacteria\_none;Bacteroidota;Flavobacteriia;Flavobacteriales;Crocinitomicaceae;Paracrocinitomix;Paracrocinitomix mangrovi |
| Aequorivita marisscotiae | 0 | 0 | 0 | 0 | 0 | 0 | 0 | 0 | 0 | 0 | 0 | 0 | 0 | 0 | Bacteria | Bacteria\_none | Bacteroidota | Flavobacteriia | Flavobacteriales | Flavobacteriaceae | Aequorivita | Bacteria;Bacteria\_none;Bacteroidota;Flavobacteriia;Flavobacteriales;Flavobacteriaceae;Aequorivita;Aequorivita marisscotiae |
| Algibacter luteus | 0 | 0 | 0 | 0 | 0 | 0 | 0 | 0 | 0 | 0 | 0 | 1 | 0 | 0 | Bacteria | Bacteria\_none | Bacteroidota | Flavobacteriia | Flavobacteriales | Flavobacteriaceae | Algibacter | Bacteria;Bacteria\_none;Bacteroidota;Flavobacteriia;Flavobacteriales;Flavobacteriaceae;Algibacter;Algibacter luteus |
| Algibacter sp. L1A34 | 0 | 0 | 0 | 0 | 0 | 1 | 0 | 0 | 0 | 0 | 0 | 0 | 0 | 0 | Bacteria | Bacteria\_none | Bacteroidota | Flavobacteriia | Flavobacteriales | Flavobacteriaceae | Algibacter | Bacteria;Bacteria\_none;Bacteroidota;Flavobacteriia;Flavobacteriales;Flavobacteriaceae;Algibacter;Algibacter sp. L1A34 |
| Aquimarina sp. AD10 | 0 | 0 | 0 | 0 | 1 | 0 | 0 | 0 | 0 | 0 | 0 | 0 | 0 | 0 | Bacteria | Bacteria\_none | Bacteroidota | Flavobacteriia | Flavobacteriales | Flavobacteriaceae | Aquimarina | Bacteria;Bacteria\_none;Bacteroidota;Flavobacteriia;Flavobacteriales;Flavobacteriaceae;Aquimarina;Aquimarina sp. AD10 |
| Capnocytophaga canimorsus | 0 | 0 | 0 | 0 | 0 | 0 | 0 | 0 | 0 | 1 | 0 | 0 | 0 | 0 | Bacteria | Bacteria\_none | Bacteroidota | Flavobacteriia | Flavobacteriales | Flavobacteriaceae | Capnocytophaga | Bacteria;Bacteria\_none;Bacteroidota;Flavobacteriia;Flavobacteriales;Flavobacteriaceae;Capnocytophaga;Capnocytophaga canimorsus |
| Capnocytophaga endodontalis | 6 | 4 | 1 | 4 | 0 | 4 | 1 | 5 | 0 | 9 | 9 | 0 | 3 | 3 | Bacteria | Bacteria\_none | Bacteroidota | Flavobacteriia | Flavobacteriales | Flavobacteriaceae | Capnocytophaga | Bacteria;Bacteria\_none;Bacteroidota;Flavobacteriia;Flavobacteriales;Flavobacteriaceae;Capnocytophaga;Capnocytophaga endodontalis |
| Capnocytophaga gingivalis | 53 | 45 | 35 | 14 | 18 | 3 | 8 | 11 | 0 | 28 | 14 | 4 | 15 | 7 | Bacteria | Bacteria\_none | Bacteroidota | Flavobacteriia | Flavobacteriales | Flavobacteriaceae | Capnocytophaga | Bacteria;Bacteria\_none;Bacteroidota;Flavobacteriia;Flavobacteriales;Flavobacteriaceae;Capnocytophaga;Capnocytophaga gingivalis |
| Capnocytophaga haemolytica | 1 | 0 | 0 | 0 | 1 | 0 | 0 | 0 | 0 | 1 | 2 | 0 | 0 | 0 | Bacteria | Bacteria\_none | Bacteroidota | Flavobacteriia | Flavobacteriales | Flavobacteriaceae | Capnocytophaga | Bacteria;Bacteria\_none;Bacteroidota;Flavobacteriia;Flavobacteriales;Flavobacteriaceae;Capnocytophaga;Capnocytophaga haemolytica |
| Capnocytophaga leadbetteri | 52 | 26 | 13 | 44 | 9 | 1 | 1 | 5 | 0 | 47 | 20 | 6 | 8 | 4 | Bacteria | Bacteria\_none | Bacteroidota | Flavobacteriia | Flavobacteriales | Flavobacteriaceae | Capnocytophaga | Bacteria;Bacteria\_none;Bacteroidota;Flavobacteriia;Flavobacteriales;Flavobacteriaceae;Capnocytophaga;Capnocytophaga leadbetteri |
| Capnocytophaga ochracea | 1 | 8 | 11 | 4 | 6 | 8 | 0 | 4 | 0 | 10 | 4 | 3 | 4 | 1 | Bacteria | Bacteria\_none | Bacteroidota | Flavobacteriia | Flavobacteriales | Flavobacteriaceae | Capnocytophaga | Bacteria;Bacteria\_none;Bacteroidota;Flavobacteriia;Flavobacteriales;Flavobacteriaceae;Capnocytophaga;Capnocytophaga ochracea |
| Capnocytophaga sp. FDAARGOS\_737 | 45 | 37 | 7 | 27 | 19 | 3 | 1 | 6 | 0 | 66 | 20 | 8 | 11 | 4 | Bacteria | Bacteria\_none | Bacteroidota | Flavobacteriia | Flavobacteriales | Flavobacteriaceae | Capnocytophaga | Bacteria;Bacteria\_none;Bacteroidota;Flavobacteriia;Flavobacteriales;Flavobacteriaceae;Capnocytophaga;Capnocytophaga sp. FDAARGOS\_737 |
| Capnocytophaga sp. H4358 | 0 | 0 | 0 | 0 | 0 | 0 | 0 | 0 | 0 | 1 | 0 | 0 | 0 | 0 | Bacteria | Bacteria\_none | Bacteroidota | Flavobacteriia | Flavobacteriales | Flavobacteriaceae | Capnocytophaga | Bacteria;Bacteria\_none;Bacteroidota;Flavobacteriia;Flavobacteriales;Flavobacteriaceae;Capnocytophaga;Capnocytophaga sp. H4358 |
| Capnocytophaga sp. oral taxon 323 | 2 | 5 | 2 | 0 | 0 | 0 | 0 | 0 | 0 | 0 | 0 | 0 | 0 | 0 | Bacteria | Bacteria\_none | Bacteroidota | Flavobacteriia | Flavobacteriales | Flavobacteriaceae | Capnocytophaga | Bacteria;Bacteria\_none;Bacteroidota;Flavobacteriia;Flavobacteriales;Flavobacteriaceae;Capnocytophaga;Capnocytophaga sp. oral taxon 323 |
| Capnocytophaga sp. oral taxon 864 | 0 | 2 | 2 | 1 | 0 | 0 | 0 | 0 | 0 | 0 | 0 | 0 | 0 | 0 | Bacteria | Bacteria\_none | Bacteroidota | Flavobacteriia | Flavobacteriales | Flavobacteriaceae | Capnocytophaga | Bacteria;Bacteria\_none;Bacteroidota;Flavobacteriia;Flavobacteriales;Flavobacteriaceae;Capnocytophaga;Capnocytophaga sp. oral taxon 864 |
| Capnocytophaga sp. oral taxon 878 | 10 | 3 | 0 | 3 | 15 | 1 | 1 | 1 | 0 | 8 | 0 | 1 | 1 | 2 | Bacteria | Bacteria\_none | Bacteroidota | Flavobacteriia | Flavobacteriales | Flavobacteriaceae | Capnocytophaga | Bacteria;Bacteria\_none;Bacteroidota;Flavobacteriia;Flavobacteriales;Flavobacteriaceae;Capnocytophaga;Capnocytophaga sp. oral taxon 878 |
| Capnocytophaga sp. oral taxon 902 | 2 | 1 | 4 | 0 | 2 | 0 | 0 | 0 | 0 | 0 | 0 | 0 | 0 | 0 | Bacteria | Bacteria\_none | Bacteroidota | Flavobacteriia | Flavobacteriales | Flavobacteriaceae | Capnocytophaga | Bacteria;Bacteria\_none;Bacteroidota;Flavobacteriia;Flavobacteriales;Flavobacteriaceae;Capnocytophaga;Capnocytophaga sp. oral taxon 902 |
| Capnocytophaga sputigena | 103 | 9 | 37 | 17 | 18 | 1 | 9 | 49 | 0 | 16 | 14 | 1 | 21 | 18 | Bacteria | Bacteria\_none | Bacteroidota | Flavobacteriia | Flavobacteriales | Flavobacteriaceae | Capnocytophaga | Bacteria;Bacteria\_none;Bacteroidota;Flavobacteriia;Flavobacteriales;Flavobacteriaceae;Capnocytophaga;Capnocytophaga sputigena |
| Capnocytophaga stomatis | 0 | 0 | 0 | 0 | 0 | 0 | 0 | 0 | 0 | 1 | 0 | 0 | 0 | 0 | Bacteria | Bacteria\_none | Bacteroidota | Flavobacteriia | Flavobacteriales | Flavobacteriaceae | Capnocytophaga | Bacteria;Bacteria\_none;Bacteroidota;Flavobacteriia;Flavobacteriales;Flavobacteriaceae;Capnocytophaga;Capnocytophaga stomatis |
| Christiangramia flava | 0 | 0 | 0 | 0 | 0 | 0 | 0 | 0 | 0 | 0 | 1 | 0 | 0 | 0 | Bacteria | Bacteria\_none | Bacteroidota | Flavobacteriia | Flavobacteriales | Flavobacteriaceae | Christiangramia | Bacteria;Bacteria\_none;Bacteroidota;Flavobacteriia;Flavobacteriales;Flavobacteriaceae;Christiangramia;Christiangramia flava |
| Gramella sp. MT6 | 0 | 0 | 0 | 0 | 0 | 0 | 0 | 0 | 0 | 0 | 0 | 0 | 0 | 0 | Bacteria | Bacteria\_none | Bacteroidota | Flavobacteriia | Flavobacteriales | Flavobacteriaceae | Christiangramia | Bacteria;Bacteria\_none;Bacteroidota;Flavobacteriia;Flavobacteriales;Flavobacteriaceae;Christiangramia;Gramella sp. MT6 |
| Costertonia aggregata | 0 | 0 | 0 | 0 | 0 | 0 | 0 | 0 | 0 | 0 | 1 | 0 | 0 | 0 | Bacteria | Bacteria\_none | Bacteroidota | Flavobacteriia | Flavobacteriales | Flavobacteriaceae | Costertonia | Bacteria;Bacteria\_none;Bacteroidota;Flavobacteriia;Flavobacteriales;Flavobacteriaceae;Costertonia;Costertonia aggregata |
| Dokdonia donghaensis | 0 | 0 | 0 | 0 | 0 | 0 | 0 | 0 | 0 | 0 | 0 | 0 | 0 | 0 | Bacteria | Bacteria\_none | Bacteroidota | Flavobacteriia | Flavobacteriales | Flavobacteriaceae | Dokdonia | Bacteria;Bacteria\_none;Bacteroidota;Flavobacteriia;Flavobacteriales;Flavobacteriaceae;Dokdonia;Dokdonia donghaensis |
| Dokdonia sp. Dokd-P16 | 0 | 0 | 0 | 0 | 0 | 0 | 0 | 0 | 0 | 0 | 0 | 0 | 0 | 0 | Bacteria | Bacteria\_none | Bacteroidota | Flavobacteriia | Flavobacteriales | Flavobacteriaceae | Dokdonia | Bacteria;Bacteria\_none;Bacteroidota;Flavobacteriia;Flavobacteriales;Flavobacteriaceae;Dokdonia;Dokdonia sp. Dokd-P16 |
| Euzebyella marina | 0 | 0 | 0 | 0 | 0 | 0 | 0 | 0 | 0 | 0 | 0 | 0 | 0 | 0 | Bacteria | Bacteria\_none | Bacteroidota | Flavobacteriia | Flavobacteriales | Flavobacteriaceae | Euzebyella | Bacteria;Bacteria\_none;Bacteroidota;Flavobacteriia;Flavobacteriales;Flavobacteriaceae;Euzebyella;Euzebyella marina |
| Flagellimonas oceani | 0 | 0 | 0 | 0 | 0 | 0 | 0 | 1 | 0 | 0 | 0 | 0 | 0 | 0 | Bacteria | Bacteria\_none | Bacteroidota | Flavobacteriia | Flavobacteriales | Flavobacteriaceae | Flagellimonas | Bacteria;Bacteria\_none;Bacteroidota;Flavobacteriia;Flavobacteriales;Flavobacteriaceae;Flagellimonas;Flagellimonas oceani |
| Flagellimonas sp. CMM7 | 0 | 0 | 0 | 0 | 0 | 0 | 0 | 0 | 0 | 0 | 1 | 0 | 0 | 0 | Bacteria | Bacteria\_none | Bacteroidota | Flavobacteriia | Flavobacteriales | Flavobacteriaceae | Flagellimonas | Bacteria;Bacteria\_none;Bacteroidota;Flavobacteriia;Flavobacteriales;Flavobacteriaceae;Flagellimonas;Flagellimonas sp. CMM7 |
| Flavobacterium aestivum | 0 | 0 | 0 | 0 | 0 | 0 | 0 | 0 | 0 | 0 | 1 | 0 | 0 | 0 | Bacteria | Bacteria\_none | Bacteroidota | Flavobacteriia | Flavobacteriales | Flavobacteriaceae | Flavobacterium | Bacteria;Bacteria\_none;Bacteroidota;Flavobacteriia;Flavobacteriales;Flavobacteriaceae;Flavobacterium;Flavobacterium aestivum |
| Flavobacterium agricola | 0 | 0 | 0 | 0 | 1 | 0 | 0 | 0 | 0 | 0 | 0 | 0 | 0 | 0 | Bacteria | Bacteria\_none | Bacteroidota | Flavobacteriia | Flavobacteriales | Flavobacteriaceae | Flavobacterium | Bacteria;Bacteria\_none;Bacteroidota;Flavobacteriia;Flavobacteriales;Flavobacteriaceae;Flavobacterium;Flavobacterium agricola |
| Flavobacterium alkalisoli | 0 | 0 | 0 | 0 | 0 | 0 | 0 | 0 | 0 | 0 | 0 | 0 | 0 | 0 | Bacteria | Bacteria\_none | Bacteroidota | Flavobacteriia | Flavobacteriales | Flavobacteriaceae | Flavobacterium | Bacteria;Bacteria\_none;Bacteroidota;Flavobacteriia;Flavobacteriales;Flavobacteriaceae;Flavobacterium;Flavobacterium alkalisoli |
| Flavobacterium aquiphilum | 0 | 0 | 0 | 0 | 0 | 0 | 0 | 0 | 0 | 0 | 0 | 0 | 0 | 0 | Bacteria | Bacteria\_none | Bacteroidota | Flavobacteriia | Flavobacteriales | Flavobacteriaceae | Flavobacterium | Bacteria;Bacteria\_none;Bacteroidota;Flavobacteriia;Flavobacteriales;Flavobacteriaceae;Flavobacterium;Flavobacterium aquiphilum |
| Flavobacterium arcticum | 0 | 1 | 0 | 0 | 0 | 0 | 0 | 0 | 0 | 0 | 0 | 0 | 0 | 0 | Bacteria | Bacteria\_none | Bacteroidota | Flavobacteriia | Flavobacteriales | Flavobacteriaceae | Flavobacterium | Bacteria;Bacteria\_none;Bacteroidota;Flavobacteriia;Flavobacteriales;Flavobacteriaceae;Flavobacterium;Flavobacterium arcticum |
| Flavobacterium azooxidireducens | 0 | 0 | 0 | 0 | 0 | 0 | 0 | 0 | 0 | 0 | 0 | 1 | 0 | 0 | Bacteria | Bacteria\_none | Bacteroidota | Flavobacteriia | Flavobacteriales | Flavobacteriaceae | Flavobacterium | Bacteria;Bacteria\_none;Bacteroidota;Flavobacteriia;Flavobacteriales;Flavobacteriaceae;Flavobacterium;Flavobacterium azooxidireducens |
| Flavobacterium bizetiae | 0 | 0 | 0 | 0 | 0 | 0 | 0 | 0 | 0 | 0 | 0 | 0 | 0 | 0 | Bacteria | Bacteria\_none | Bacteroidota | Flavobacteriia | Flavobacteriales | Flavobacteriaceae | Flavobacterium | Bacteria;Bacteria\_none;Bacteroidota;Flavobacteriia;Flavobacteriales;Flavobacteriaceae;Flavobacterium;Flavobacterium bizetiae |
| Flavobacterium crassostreae | 0 | 0 | 0 | 0 | 0 | 0 | 0 | 0 | 0 | 0 | 1 | 0 | 0 | 0 | Bacteria | Bacteria\_none | Bacteroidota | Flavobacteriia | Flavobacteriales | Flavobacteriaceae | Flavobacterium | Bacteria;Bacteria\_none;Bacteroidota;Flavobacteriia;Flavobacteriales;Flavobacteriaceae;Flavobacterium;Flavobacterium crassostreae |
| Flavobacterium dauae | 0 | 0 | 0 | 1 | 0 | 0 | 0 | 0 | 0 | 0 | 0 | 0 | 0 | 0 | Bacteria | Bacteria\_none | Bacteroidota | Flavobacteriia | Flavobacteriales | Flavobacteriaceae | Flavobacterium | Bacteria;Bacteria\_none;Bacteroidota;Flavobacteriia;Flavobacteriales;Flavobacteriaceae;Flavobacterium;Flavobacterium dauae |
| Flavobacterium faecale | 1 | 0 | 0 | 0 | 0 | 0 | 0 | 0 | 0 | 0 | 0 | 0 | 0 | 0 | Bacteria | Bacteria\_none | Bacteroidota | Flavobacteriia | Flavobacteriales | Flavobacteriaceae | Flavobacterium | Bacteria;Bacteria\_none;Bacteroidota;Flavobacteriia;Flavobacteriales;Flavobacteriaceae;Flavobacterium;Flavobacterium faecale |
| Flavobacterium flavipigmentatum | 0 | 0 | 0 | 0 | 0 | 0 | 0 | 0 | 0 | 0 | 0 | 0 | 0 | 0 | Bacteria | Bacteria\_none | Bacteroidota | Flavobacteriia | Flavobacteriales | Flavobacteriaceae | Flavobacterium | Bacteria;Bacteria\_none;Bacteroidota;Flavobacteriia;Flavobacteriales;Flavobacteriaceae;Flavobacterium;Flavobacterium flavipigmentatum |
| Flavobacterium ginsengisoli | 0 | 0 | 0 | 0 | 0 | 0 | 0 | 0 | 0 | 0 | 0 | 0 | 0 | 0 | Bacteria | Bacteria\_none | Bacteroidota | Flavobacteriia | Flavobacteriales | Flavobacteriaceae | Flavobacterium | Bacteria;Bacteria\_none;Bacteroidota;Flavobacteriia;Flavobacteriales;Flavobacteriaceae;Flavobacterium;Flavobacterium ginsengisoli |
| Flavobacterium indicum | 0 | 0 | 0 | 1 | 0 | 0 | 0 | 0 | 0 | 0 | 0 | 0 | 0 | 0 | Bacteria | Bacteria\_none | Bacteroidota | Flavobacteriia | Flavobacteriales | Flavobacteriaceae | Flavobacterium | Bacteria;Bacteria\_none;Bacteroidota;Flavobacteriia;Flavobacteriales;Flavobacteriaceae;Flavobacterium;Flavobacterium indicum |
| Flavobacterium limnophilum | 0 | 0 | 0 | 0 | 0 | 1 | 0 | 0 | 0 | 0 | 0 | 0 | 0 | 0 | Bacteria | Bacteria\_none | Bacteroidota | Flavobacteriia | Flavobacteriales | Flavobacteriaceae | Flavobacterium | Bacteria;Bacteria\_none;Bacteroidota;Flavobacteriia;Flavobacteriales;Flavobacteriaceae;Flavobacterium;Flavobacterium limnophilum |
| Flavobacterium psychrophilum | 0 | 0 | 0 | 0 | 0 | 0 | 0 | 0 | 0 | 0 | 0 | 0 | 0 | 0 | Bacteria | Bacteria\_none | Bacteroidota | Flavobacteriia | Flavobacteriales | Flavobacteriaceae | Flavobacterium | Bacteria;Bacteria\_none;Bacteroidota;Flavobacteriia;Flavobacteriales;Flavobacteriaceae;Flavobacterium;Flavobacterium psychrophilum |
| Flavobacterium sp. 140616W15 | 0 | 0 | 0 | 0 | 0 | 0 | 0 | 0 | 0 | 0 | 0 | 1 | 0 | 0 | Bacteria | Bacteria\_none | Bacteroidota | Flavobacteriia | Flavobacteriales | Flavobacteriaceae | Flavobacterium | Bacteria;Bacteria\_none;Bacteroidota;Flavobacteriia;Flavobacteriales;Flavobacteriaceae;Flavobacterium;Flavobacterium sp. 140616W15 |
| Flavobacterium sp. CLA17 | 1 | 0 | 0 | 0 | 0 | 0 | 0 | 0 | 0 | 1 | 0 | 0 | 0 | 0 | Bacteria | Bacteria\_none | Bacteroidota | Flavobacteriia | Flavobacteriales | Flavobacteriaceae | Flavobacterium | Bacteria;Bacteria\_none;Bacteroidota;Flavobacteriia;Flavobacteriales;Flavobacteriaceae;Flavobacterium;Flavobacterium sp. CLA17 |
| Flavobacterium sp. I3-2 | 0 | 1 | 0 | 0 | 1 | 0 | 0 | 0 | 0 | 0 | 0 | 0 | 0 | 0 | Bacteria | Bacteria\_none | Bacteroidota | Flavobacteriia | Flavobacteriales | Flavobacteriaceae | Flavobacterium | Bacteria;Bacteria\_none;Bacteroidota;Flavobacteriia;Flavobacteriales;Flavobacteriaceae;Flavobacterium;Flavobacterium sp. I3-2 |
| Flavobacterium sp. IMCC34518 | 0 | 0 | 0 | 0 | 0 | 0 | 0 | 0 | 0 | 0 | 0 | 0 | 0 | 0 | Bacteria | Bacteria\_none | Bacteroidota | Flavobacteriia | Flavobacteriales | Flavobacteriaceae | Flavobacterium | Bacteria;Bacteria\_none;Bacteroidota;Flavobacteriia;Flavobacteriales;Flavobacteriaceae;Flavobacterium;Flavobacterium sp. IMCC34518 |
| Flavobacterium sp. IMCC34852 | 0 | 0 | 0 | 0 | 0 | 0 | 0 | 0 | 0 | 1 | 0 | 0 | 0 | 0 | Bacteria | Bacteria\_none | Bacteroidota | Flavobacteriia | Flavobacteriales | Flavobacteriaceae | Flavobacterium | Bacteria;Bacteria\_none;Bacteroidota;Flavobacteriia;Flavobacteriales;Flavobacteriaceae;Flavobacterium;Flavobacterium sp. IMCC34852 |
| Flavobacterium sp. KBS0721 | 0 | 0 | 0 | 0 | 0 | 0 | 0 | 0 | 0 | 0 | 0 | 0 | 0 | 0 | Bacteria | Bacteria\_none | Bacteroidota | Flavobacteriia | Flavobacteriales | Flavobacteriaceae | Flavobacterium | Bacteria;Bacteria\_none;Bacteroidota;Flavobacteriia;Flavobacteriales;Flavobacteriaceae;Flavobacterium;Flavobacterium sp. KBS0721 |
| Formosa sediminum | 0 | 0 | 0 | 0 | 0 | 1 | 0 | 0 | 0 | 0 | 0 | 0 | 0 | 0 | Bacteria | Bacteria\_none | Bacteroidota | Flavobacteriia | Flavobacteriales | Flavobacteriaceae | Formosa | Bacteria;Bacteria\_none;Bacteroidota;Flavobacteriia;Flavobacteriales;Flavobacteriaceae;Formosa;Formosa sediminum |
| Formosa sp. Hel1\_31\_208 | 0 | 0 | 0 | 0 | 0 | 0 | 0 | 0 | 0 | 0 | 0 | 0 | 0 | 0 | Bacteria | Bacteria\_none | Bacteroidota | Flavobacteriia | Flavobacteriales | Flavobacteriaceae | Formosa | Bacteria;Bacteria\_none;Bacteroidota;Flavobacteriia;Flavobacteriales;Flavobacteriaceae;Formosa;Formosa sp. Hel1\_31\_208 |
| Formosa sp. L2A11 | 0 | 0 | 0 | 0 | 1 | 0 | 0 | 0 | 0 | 0 | 0 | 0 | 0 | 0 | Bacteria | Bacteria\_none | Bacteroidota | Flavobacteriia | Flavobacteriales | Flavobacteriaceae | Formosa | Bacteria;Bacteria\_none;Bacteroidota;Flavobacteriia;Flavobacteriales;Flavobacteriaceae;Formosa;Formosa sp. L2A11 |
| Kordia antarctica | 0 | 0 | 0 | 1 | 0 | 0 | 0 | 0 | 0 | 0 | 0 | 0 | 0 | 0 | Bacteria | Bacteria\_none | Bacteroidota | Flavobacteriia | Flavobacteriales | Flavobacteriaceae | Kordia | Bacteria;Bacteria\_none;Bacteroidota;Flavobacteriia;Flavobacteriales;Flavobacteriaceae;Kordia;Kordia antarctica |
| Kordia sp. SMS9 | 0 | 0 | 0 | 0 | 0 | 0 | 0 | 0 | 0 | 0 | 0 | 0 | 0 | 0 | Bacteria | Bacteria\_none | Bacteroidota | Flavobacteriia | Flavobacteriales | Flavobacteriaceae | Kordia | Bacteria;Bacteria\_none;Bacteroidota;Flavobacteriia;Flavobacteriales;Flavobacteriaceae;Kordia;Kordia sp. SMS9 |
| Lacinutrix neustonica | 0 | 0 | 0 | 0 | 0 | 0 | 0 | 0 | 0 | 0 | 0 | 0 | 0 | 0 | Bacteria | Bacteria\_none | Bacteroidota | Flavobacteriia | Flavobacteriales | Flavobacteriaceae | Lacinutrix | Bacteria;Bacteria\_none;Bacteroidota;Flavobacteriia;Flavobacteriales;Flavobacteriaceae;Lacinutrix;Lacinutrix neustonica |
| Lacinutrix sp. 5H-3-7-4 | 0 | 0 | 0 | 0 | 0 | 0 | 0 | 0 | 0 | 0 | 0 | 0 | 0 | 0 | Bacteria | Bacteria\_none | Bacteroidota | Flavobacteriia | Flavobacteriales | Flavobacteriaceae | Lacinutrix | Bacteria;Bacteria\_none;Bacteroidota;Flavobacteriia;Flavobacteriales;Flavobacteriaceae;Lacinutrix;Lacinutrix sp. 5H-3-7-4 |
| Lacinutrix sp. WUR7 | 0 | 0 | 0 | 0 | 0 | 0 | 0 | 0 | 0 | 0 | 0 | 0 | 0 | 0 | Bacteria | Bacteria\_none | Bacteroidota | Flavobacteriia | Flavobacteriales | Flavobacteriaceae | Lacinutrix | Bacteria;Bacteria\_none;Bacteroidota;Flavobacteriia;Flavobacteriales;Flavobacteriaceae;Lacinutrix;Lacinutrix sp. WUR7 |
| Lacinutrix venerupis | 0 | 0 | 0 | 0 | 0 | 0 | 0 | 0 | 0 | 0 | 0 | 0 | 0 | 0 | Bacteria | Bacteria\_none | Bacteroidota | Flavobacteriia | Flavobacteriales | Flavobacteriaceae | Lacinutrix | Bacteria;Bacteria\_none;Bacteroidota;Flavobacteriia;Flavobacteriales;Flavobacteriaceae;Lacinutrix;Lacinutrix venerupis |
| Leeuwenhoekiella sp. ZYFB001 | 0 | 0 | 0 | 0 | 0 | 0 | 0 | 0 | 0 | 0 | 0 | 0 | 0 | 0 | Bacteria | Bacteria\_none | Bacteroidota | Flavobacteriia | Flavobacteriales | Flavobacteriaceae | Leeuwenhoekiella | Bacteria;Bacteria\_none;Bacteroidota;Flavobacteriia;Flavobacteriales;Flavobacteriaceae;Leeuwenhoekiella;Leeuwenhoekiella sp. ZYFB001 |
| Lutibacter sp. A80 | 0 | 0 | 0 | 0 | 0 | 0 | 0 | 0 | 0 | 0 | 0 | 0 | 0 | 1 | Bacteria | Bacteria\_none | Bacteroidota | Flavobacteriia | Flavobacteriales | Flavobacteriaceae | Lutibacter | Bacteria;Bacteria\_none;Bacteroidota;Flavobacteriia;Flavobacteriales;Flavobacteriaceae;Lutibacter;Lutibacter sp. A80 |
| Mangrovimonas sp. YM274 | 0 | 0 | 0 | 0 | 0 | 0 | 0 | 0 | 0 | 0 | 0 | 0 | 0 | 0 | Bacteria | Bacteria\_none | Bacteroidota | Flavobacteriia | Flavobacteriales | Flavobacteriaceae | Mangrovimonas | Bacteria;Bacteria\_none;Bacteroidota;Flavobacteriia;Flavobacteriales;Flavobacteriaceae;Mangrovimonas;Mangrovimonas sp. YM274 |
| Maribacter hydrothermalis | 0 | 0 | 0 | 0 | 0 | 0 | 0 | 0 | 0 | 0 | 0 | 0 | 0 | 0 | Bacteria | Bacteria\_none | Bacteroidota | Flavobacteriia | Flavobacteriales | Flavobacteriaceae | Maribacter | Bacteria;Bacteria\_none;Bacteroidota;Flavobacteriia;Flavobacteriales;Flavobacteriaceae;Maribacter;Maribacter hydrothermalis |
| Maribacter sp. BPC-D8 | 0 | 0 | 0 | 0 | 1 | 0 | 0 | 0 | 0 | 0 | 0 | 0 | 0 | 0 | Bacteria | Bacteria\_none | Bacteroidota | Flavobacteriia | Flavobacteriales | Flavobacteriaceae | Maribacter | Bacteria;Bacteria\_none;Bacteroidota;Flavobacteriia;Flavobacteriales;Flavobacteriaceae;Maribacter;Maribacter sp. BPC-D8 |
| Myroides albus | 0 | 0 | 0 | 0 | 0 | 0 | 0 | 0 | 0 | 0 | 0 | 0 | 0 | 0 | Bacteria | Bacteria\_none | Bacteroidota | Flavobacteriia | Flavobacteriales | Flavobacteriaceae | Myroides | Bacteria;Bacteria\_none;Bacteroidota;Flavobacteriia;Flavobacteriales;Flavobacteriaceae;Myroides;Myroides albus |
| Myroides fluvii | 0 | 0 | 0 | 0 | 0 | 0 | 0 | 0 | 0 | 1 | 0 | 0 | 0 | 0 | Bacteria | Bacteria\_none | Bacteroidota | Flavobacteriia | Flavobacteriales | Flavobacteriaceae | Myroides | Bacteria;Bacteria\_none;Bacteroidota;Flavobacteriia;Flavobacteriales;Flavobacteriaceae;Myroides;Myroides fluvii |
| Nonlabens sp. MIC269 | 0 | 0 | 0 | 0 | 0 | 0 | 0 | 0 | 0 | 0 | 0 | 0 | 0 | 0 | Bacteria | Bacteria\_none | Bacteroidota | Flavobacteriia | Flavobacteriales | Flavobacteriaceae | Nonlabens | Bacteria;Bacteria\_none;Bacteroidota;Flavobacteriia;Flavobacteriales;Flavobacteriaceae;Nonlabens;Nonlabens sp. MIC269 |
| Polaribacter gangjinensis | 0 | 0 | 0 | 0 | 0 | 0 | 0 | 0 | 0 | 0 | 0 | 0 | 0 | 0 | Bacteria | Bacteria\_none | Bacteroidota | Flavobacteriia | Flavobacteriales | Flavobacteriaceae | Polaribacter | Bacteria;Bacteria\_none;Bacteroidota;Flavobacteriia;Flavobacteriales;Flavobacteriaceae;Polaribacter;Polaribacter gangjinensis |
| Polaribacter haliotis | 0 | 0 | 0 | 0 | 1 | 0 | 0 | 0 | 0 | 0 | 0 | 0 | 0 | 0 | Bacteria | Bacteria\_none | Bacteroidota | Flavobacteriia | Flavobacteriales | Flavobacteriaceae | Polaribacter | Bacteria;Bacteria\_none;Bacteroidota;Flavobacteriia;Flavobacteriales;Flavobacteriaceae;Polaribacter;Polaribacter haliotis |
| Polaribacter litorisediminis | 0 | 0 | 0 | 0 | 0 | 0 | 0 | 0 | 0 | 0 | 0 | 0 | 0 | 0 | Bacteria | Bacteria\_none | Bacteroidota | Flavobacteriia | Flavobacteriales | Flavobacteriaceae | Polaribacter | Bacteria;Bacteria\_none;Bacteroidota;Flavobacteriia;Flavobacteriales;Flavobacteriaceae;Polaribacter;Polaribacter litorisediminis |
| Polaribacter pectinis | 0 | 0 | 0 | 0 | 0 | 0 | 0 | 0 | 0 | 0 | 0 | 0 | 0 | 0 | Bacteria | Bacteria\_none | Bacteroidota | Flavobacteriia | Flavobacteriales | Flavobacteriaceae | Polaribacter | Bacteria;Bacteria\_none;Bacteroidota;Flavobacteriia;Flavobacteriales;Flavobacteriaceae;Polaribacter;Polaribacter pectinis |
| Polaribacter sejongensis | 0 | 0 | 1 | 0 | 0 | 0 | 0 | 0 | 0 | 0 | 0 | 0 | 0 | 0 | Bacteria | Bacteria\_none | Bacteroidota | Flavobacteriia | Flavobacteriales | Flavobacteriaceae | Polaribacter | Bacteria;Bacteria\_none;Bacteroidota;Flavobacteriia;Flavobacteriales;Flavobacteriaceae;Polaribacter;Polaribacter sejongensis |
| Polaribacter sp. SA4-12 | 0 | 0 | 0 | 1 | 0 | 0 | 0 | 0 | 0 | 0 | 0 | 0 | 0 | 0 | Bacteria | Bacteria\_none | Bacteroidota | Flavobacteriia | Flavobacteriales | Flavobacteriaceae | Polaribacter | Bacteria;Bacteria\_none;Bacteroidota;Flavobacteriia;Flavobacteriales;Flavobacteriaceae;Polaribacter;Polaribacter sp. SA4-12 |
| Psychroflexus torquis | 0 | 0 | 0 | 0 | 0 | 0 | 0 | 0 | 0 | 0 | 0 | 0 | 0 | 0 | Bacteria | Bacteria\_none | Bacteroidota | Flavobacteriia | Flavobacteriales | Flavobacteriaceae | Psychroflexus | Bacteria;Bacteria\_none;Bacteroidota;Flavobacteriia;Flavobacteriales;Flavobacteriaceae;Psychroflexus;Psychroflexus torquis |
| Rasiella rasia | 0 | 0 | 0 | 0 | 0 | 0 | 0 | 0 | 0 | 0 | 0 | 0 | 0 | 0 | Bacteria | Bacteria\_none | Bacteroidota | Flavobacteriia | Flavobacteriales | Flavobacteriaceae | Rasiella | Bacteria;Bacteria\_none;Bacteroidota;Flavobacteriia;Flavobacteriales;Flavobacteriaceae;Rasiella;Rasiella rasia |
| Salegentibacter salegens | 0 | 1 | 0 | 0 | 0 | 0 | 0 | 0 | 0 | 0 | 0 | 0 | 0 | 0 | Bacteria | Bacteria\_none | Bacteroidota | Flavobacteriia | Flavobacteriales | Flavobacteriaceae | Salegentibacter | Bacteria;Bacteria\_none;Bacteroidota;Flavobacteriia;Flavobacteriales;Flavobacteriaceae;Salegentibacter;Salegentibacter salegens |
| Tenacibaculum jejuense | 0 | 0 | 0 | 0 | 1 | 0 | 0 | 0 | 0 | 0 | 0 | 0 | 0 | 0 | Bacteria | Bacteria\_none | Bacteroidota | Flavobacteriia | Flavobacteriales | Flavobacteriaceae | Tenacibaculum | Bacteria;Bacteria\_none;Bacteroidota;Flavobacteriia;Flavobacteriales;Flavobacteriaceae;Tenacibaculum;Tenacibaculum jejuense |
| Tenacibaculum maritimum | 0 | 0 | 0 | 0 | 0 | 0 | 0 | 0 | 0 | 0 | 0 | 0 | 1 | 0 | Bacteria | Bacteria\_none | Bacteroidota | Flavobacteriia | Flavobacteriales | Flavobacteriaceae | Tenacibaculum | Bacteria;Bacteria\_none;Bacteroidota;Flavobacteriia;Flavobacteriales;Flavobacteriaceae;Tenacibaculum;Tenacibaculum maritimum |
| Tenacibaculum pacificus | 0 | 0 | 0 | 0 | 0 | 0 | 0 | 0 | 0 | 0 | 0 | 0 | 0 | 0 | Bacteria | Bacteria\_none | Bacteroidota | Flavobacteriia | Flavobacteriales | Flavobacteriaceae | Tenacibaculum | Bacteria;Bacteria\_none;Bacteroidota;Flavobacteriia;Flavobacteriales;Flavobacteriaceae;Tenacibaculum;Tenacibaculum pacificus |
| Tenacibaculum retecalamus | 0 | 0 | 0 | 0 | 0 | 0 | 0 | 0 | 0 | 0 | 0 | 0 | 0 | 0 | Bacteria | Bacteria\_none | Bacteroidota | Flavobacteriia | Flavobacteriales | Flavobacteriaceae | Tenacibaculum | Bacteria;Bacteria\_none;Bacteroidota;Flavobacteriia;Flavobacteriales;Flavobacteriaceae;Tenacibaculum;Tenacibaculum retecalamus |
| Zobellia alginiliquefaciens | 0 | 0 | 0 | 0 | 1 | 0 | 0 | 0 | 0 | 0 | 0 | 0 | 0 | 0 | Bacteria | Bacteria\_none | Bacteroidota | Flavobacteriia | Flavobacteriales | Flavobacteriaceae | Zobellia | Bacteria;Bacteria\_none;Bacteroidota;Flavobacteriia;Flavobacteriales;Flavobacteriaceae;Zobellia;Zobellia alginiliquefaciens |
| Croceimicrobium hydrocarbonivorans | 0 | 1 | 0 | 0 | 1 | 0 | 0 | 0 | 0 | 0 | 0 | 0 | 0 | 0 | Bacteria | Bacteria\_none | Bacteroidota | Flavobacteriia | Flavobacteriales | Owenweeksiaceae | Croceimicrobium | Bacteria;Bacteria\_none;Bacteroidota;Flavobacteriia;Flavobacteriales;Owenweeksiaceae;Croceimicrobium;Croceimicrobium hydrocarbonivorans |
| Sanyastnella coralliicola | 0 | 0 | 1 | 0 | 0 | 0 | 0 | 0 | 0 | 0 | 0 | 0 | 0 | 0 | Bacteria | Bacteria\_none | Bacteroidota | Flavobacteriia | Flavobacteriales | Sanyastnellaceae | Sanyastnella | Bacteria;Bacteria\_none;Bacteroidota;Flavobacteriia;Flavobacteriales;Sanyastnellaceae;Sanyastnella;Sanyastnella coralliicola |
| Apibacter sp. ESL0432 | 0 | 0 | 0 | 0 | 0 | 0 | 0 | 0 | 0 | 0 | 0 | 0 | 0 | 0 | Bacteria | Bacteria\_none | Bacteroidota | Flavobacteriia | Flavobacteriales | Weeksellaceae | Apibacter | Bacteria;Bacteria\_none;Bacteroidota;Flavobacteriia;Flavobacteriales;Weeksellaceae;Apibacter;Apibacter sp. ESL0432 |
| Bergeyella cardium | 0 | 0 | 0 | 0 | 1 | 0 | 0 | 0 | 0 | 0 | 1 | 0 | 0 | 0 | Bacteria | Bacteria\_none | Bacteroidota | Flavobacteriia | Flavobacteriales | Weeksellaceae | Bergeyella | Bacteria;Bacteria\_none;Bacteroidota;Flavobacteriia;Flavobacteriales;Weeksellaceae;Bergeyella;Bergeyella cardium |
| Bergeyella porcorum | 0 | 0 | 1 | 0 | 0 | 0 | 0 | 0 | 0 | 0 | 0 | 0 | 0 | 0 | Bacteria | Bacteria\_none | Bacteroidota | Flavobacteriia | Flavobacteriales | Weeksellaceae | Bergeyella | Bacteria;Bacteria\_none;Bacteroidota;Flavobacteriia;Flavobacteriales;Weeksellaceae;Bergeyella;Bergeyella porcorum |
| Chryseobacterium arthrosphaerae | 1 | 0 | 0 | 0 | 0 | 0 | 0 | 0 | 0 | 0 | 0 | 0 | 0 | 0 | Bacteria | Bacteria\_none | Bacteroidota | Flavobacteriia | Flavobacteriales | Weeksellaceae | Chryseobacterium | Bacteria;Bacteria\_none;Bacteroidota;Flavobacteriia;Flavobacteriales;Weeksellaceae;Chryseobacterium;Chryseobacterium arthrosphaerae |
| Chryseobacterium aureum | 0 | 0 | 0 | 0 | 0 | 0 | 0 | 0 | 0 | 1 | 0 | 0 | 0 | 0 | Bacteria | Bacteria\_none | Bacteroidota | Flavobacteriia | Flavobacteriales | Weeksellaceae | Chryseobacterium | Bacteria;Bacteria\_none;Bacteroidota;Flavobacteriia;Flavobacteriales;Weeksellaceae;Chryseobacterium;Chryseobacterium aureum |
| Chryseobacterium balustinum | 0 | 0 | 0 | 0 | 2 | 0 | 0 | 0 | 0 | 0 | 0 | 0 | 0 | 0 | Bacteria | Bacteria\_none | Bacteroidota | Flavobacteriia | Flavobacteriales | Weeksellaceae | Chryseobacterium | Bacteria;Bacteria\_none;Bacteroidota;Flavobacteriia;Flavobacteriales;Weeksellaceae;Chryseobacterium;Chryseobacterium balustinum |
| Chryseobacterium carnipullorum | 0 | 0 | 0 | 1 | 0 | 0 | 0 | 0 | 0 | 0 | 0 | 0 | 0 | 0 | Bacteria | Bacteria\_none | Bacteroidota | Flavobacteriia | Flavobacteriales | Weeksellaceae | Chryseobacterium | Bacteria;Bacteria\_none;Bacteroidota;Flavobacteriia;Flavobacteriales;Weeksellaceae;Chryseobacterium;Chryseobacterium carnipullorum |
| Chryseobacterium daecheongense | 0 | 0 | 0 | 0 | 0 | 0 | 0 | 1 | 0 | 0 | 0 | 0 | 0 | 0 | Bacteria | Bacteria\_none | Bacteroidota | Flavobacteriia | Flavobacteriales | Weeksellaceae | Chryseobacterium | Bacteria;Bacteria\_none;Bacteroidota;Flavobacteriia;Flavobacteriales;Weeksellaceae;Chryseobacterium;Chryseobacterium daecheongense |
| Chryseobacterium gallinarum | 0 | 0 | 0 | 0 | 0 | 2 | 0 | 0 | 0 | 0 | 0 | 0 | 0 | 0 | Bacteria | Bacteria\_none | Bacteroidota | Flavobacteriia | Flavobacteriales | Weeksellaceae | Chryseobacterium | Bacteria;Bacteria\_none;Bacteroidota;Flavobacteriia;Flavobacteriales;Weeksellaceae;Chryseobacterium;Chryseobacterium gallinarum |
| Chryseobacterium gambrini | 0 | 0 | 1 | 0 | 0 | 0 | 0 | 0 | 0 | 0 | 0 | 0 | 0 | 0 | Bacteria | Bacteria\_none | Bacteroidota | Flavobacteriia | Flavobacteriales | Weeksellaceae | Chryseobacterium | Bacteria;Bacteria\_none;Bacteroidota;Flavobacteriia;Flavobacteriales;Weeksellaceae;Chryseobacterium;Chryseobacterium gambrini |
| Chryseobacterium indoltheticum | 0 | 0 | 0 | 0 | 0 | 0 | 0 | 0 | 0 | 1 | 0 | 0 | 0 | 0 | Bacteria | Bacteria\_none | Bacteroidota | Flavobacteriia | Flavobacteriales | Weeksellaceae | Chryseobacterium | Bacteria;Bacteria\_none;Bacteroidota;Flavobacteriia;Flavobacteriales;Weeksellaceae;Chryseobacterium;Chryseobacterium indoltheticum |
| Chryseobacterium joostei | 0 | 0 | 0 | 0 | 0 | 0 | 0 | 0 | 0 | 0 | 0 | 0 | 0 | 0 | Bacteria | Bacteria\_none | Bacteroidota | Flavobacteriia | Flavobacteriales | Weeksellaceae | Chryseobacterium | Bacteria;Bacteria\_none;Bacteroidota;Flavobacteriia;Flavobacteriales;Weeksellaceae;Chryseobacterium;Chryseobacterium joostei |
| Chryseobacterium manosquense | 0 | 0 | 0 | 0 | 1 | 0 | 0 | 0 | 0 | 0 | 0 | 0 | 0 | 0 | Bacteria | Bacteria\_none | Bacteroidota | Flavobacteriia | Flavobacteriales | Weeksellaceae | Chryseobacterium | Bacteria;Bacteria\_none;Bacteroidota;Flavobacteriia;Flavobacteriales;Weeksellaceae;Chryseobacterium;Chryseobacterium manosquense |
| Chryseobacterium paludis | 0 | 0 | 0 | 0 | 0 | 0 | 0 | 0 | 0 | 0 | 0 | 0 | 0 | 0 | Bacteria | Bacteria\_none | Bacteroidota | Flavobacteriia | Flavobacteriales | Weeksellaceae | Chryseobacterium | Bacteria;Bacteria\_none;Bacteroidota;Flavobacteriia;Flavobacteriales;Weeksellaceae;Chryseobacterium;Chryseobacterium paludis |
| Chryseobacterium sp. | 0 | 0 | 1 | 0 | 0 | 0 | 0 | 0 | 0 | 0 | 0 | 0 | 0 | 0 | Bacteria | Bacteria\_none | Bacteroidota | Flavobacteriia | Flavobacteriales | Weeksellaceae | Chryseobacterium | Bacteria;Bacteria\_none;Bacteroidota;Flavobacteriia;Flavobacteriales;Weeksellaceae;Chryseobacterium;Chryseobacterium sp. |
| Chryseobacterium sp. G0162 | 0 | 0 | 0 | 1 | 0 | 0 | 0 | 0 | 0 | 0 | 0 | 0 | 0 | 0 | Bacteria | Bacteria\_none | Bacteroidota | Flavobacteriia | Flavobacteriales | Weeksellaceae | Chryseobacterium | Bacteria;Bacteria\_none;Bacteroidota;Flavobacteriia;Flavobacteriales;Weeksellaceae;Chryseobacterium;Chryseobacterium sp. G0162 |
| Chryseobacterium sp. G0186 | 0 | 0 | 0 | 0 | 2 | 0 | 0 | 0 | 0 | 0 | 0 | 0 | 0 | 0 | Bacteria | Bacteria\_none | Bacteroidota | Flavobacteriia | Flavobacteriales | Weeksellaceae | Chryseobacterium | Bacteria;Bacteria\_none;Bacteroidota;Flavobacteriia;Flavobacteriales;Weeksellaceae;Chryseobacterium;Chryseobacterium sp. G0186 |
| Chryseobacterium sp. PET-29 | 0 | 0 | 0 | 0 | 0 | 0 | 0 | 0 | 0 | 0 | 0 | 0 | 0 | 0 | Bacteria | Bacteria\_none | Bacteroidota | Flavobacteriia | Flavobacteriales | Weeksellaceae | Chryseobacterium | Bacteria;Bacteria\_none;Bacteroidota;Flavobacteriia;Flavobacteriales;Weeksellaceae;Chryseobacterium;Chryseobacterium sp. PET-29 |
| Chryseobacterium sp. StRB126 | 0 | 0 | 0 | 0 | 0 | 0 | 0 | 0 | 0 | 1 | 0 | 0 | 0 | 0 | Bacteria | Bacteria\_none | Bacteroidota | Flavobacteriia | Flavobacteriales | Weeksellaceae | Chryseobacterium | Bacteria;Bacteria\_none;Bacteroidota;Flavobacteriia;Flavobacteriales;Weeksellaceae;Chryseobacterium;Chryseobacterium sp. StRB126 |
| Chryseobacterium suipulveris | 0 | 0 | 0 | 0 | 0 | 0 | 0 | 0 | 0 | 0 | 0 | 0 | 0 | 0 | Bacteria | Bacteria\_none | Bacteroidota | Flavobacteriia | Flavobacteriales | Weeksellaceae | Chryseobacterium | Bacteria;Bacteria\_none;Bacteroidota;Flavobacteriia;Flavobacteriales;Weeksellaceae;Chryseobacterium;Chryseobacterium suipulveris |
| Chryseobacterium taklimakanense | 0 | 0 | 0 | 0 | 1 | 0 | 0 | 0 | 0 | 0 | 0 | 0 | 0 | 0 | Bacteria | Bacteria\_none | Bacteroidota | Flavobacteriia | Flavobacteriales | Weeksellaceae | Chryseobacterium | Bacteria;Bacteria\_none;Bacteroidota;Flavobacteriia;Flavobacteriales;Weeksellaceae;Chryseobacterium;Chryseobacterium taklimakanense |
| Cloacibacterium sp. TD35 | 0 | 0 | 0 | 0 | 0 | 0 | 0 | 0 | 0 | 0 | 1 | 0 | 0 | 0 | Bacteria | Bacteria\_none | Bacteroidota | Flavobacteriia | Flavobacteriales | Weeksellaceae | Cloacibacterium | Bacteria;Bacteria\_none;Bacteroidota;Flavobacteriia;Flavobacteriales;Weeksellaceae;Cloacibacterium;Cloacibacterium sp. TD35 |
| Elizabethkingia anophelis | 0 | 0 | 0 | 0 | 0 | 0 | 0 | 0 | 0 | 0 | 0 | 0 | 0 | 0 | Bacteria | Bacteria\_none | Bacteroidota | Flavobacteriia | Flavobacteriales | Weeksellaceae | Elizabethkingia | Bacteria;Bacteria\_none;Bacteroidota;Flavobacteriia;Flavobacteriales;Weeksellaceae;Elizabethkingia;Elizabethkingia anophelis |
| Elizabethkingia meningoseptica | 0 | 0 | 0 | 0 | 1 | 1 | 0 | 0 | 0 | 0 | 0 | 0 | 0 | 0 | Bacteria | Bacteria\_none | Bacteroidota | Flavobacteriia | Flavobacteriales | Weeksellaceae | Elizabethkingia | Bacteria;Bacteria\_none;Bacteroidota;Flavobacteriia;Flavobacteriales;Weeksellaceae;Elizabethkingia;Elizabethkingia meningoseptica |
| Elizabethkingia sp. JS20170427COW | 0 | 1 | 1 | 0 | 0 | 0 | 0 | 0 | 0 | 0 | 0 | 0 | 0 | 0 | Bacteria | Bacteria\_none | Bacteroidota | Flavobacteriia | Flavobacteriales | Weeksellaceae | Elizabethkingia | Bacteria;Bacteria\_none;Bacteroidota;Flavobacteriia;Flavobacteriales;Weeksellaceae;Elizabethkingia;Elizabethkingia sp. JS20170427COW |
| Elizabethkingia ursingii | 0 | 0 | 0 | 0 | 1 | 0 | 0 | 0 | 0 | 0 | 0 | 0 | 0 | 0 | Bacteria | Bacteria\_none | Bacteroidota | Flavobacteriia | Flavobacteriales | Weeksellaceae | Elizabethkingia | Bacteria;Bacteria\_none;Bacteroidota;Flavobacteriia;Flavobacteriales;Weeksellaceae;Elizabethkingia;Elizabethkingia ursingii |
| Empedobacter brevis | 0 | 0 | 0 | 0 | 0 | 0 | 0 | 0 | 0 | 0 | 0 | 0 | 0 | 0 | Bacteria | Bacteria\_none | Bacteroidota | Flavobacteriia | Flavobacteriales | Weeksellaceae | Empedobacter | Bacteria;Bacteria\_none;Bacteroidota;Flavobacteriia;Flavobacteriales;Weeksellaceae;Empedobacter;Empedobacter brevis |
| Empedobacter falsenii | 1 | 0 | 0 | 0 | 0 | 0 | 0 | 0 | 0 | 0 | 0 | 0 | 0 | 0 | Bacteria | Bacteria\_none | Bacteroidota | Flavobacteriia | Flavobacteriales | Weeksellaceae | Empedobacter | Bacteria;Bacteria\_none;Bacteroidota;Flavobacteriia;Flavobacteriales;Weeksellaceae;Empedobacter;Empedobacter falsenii |
| Empedobacter stercoris | 0 | 0 | 0 | 1 | 0 | 0 | 0 | 0 | 0 | 0 | 0 | 0 | 0 | 0 | Bacteria | Bacteria\_none | Bacteroidota | Flavobacteriia | Flavobacteriales | Weeksellaceae | Empedobacter | Bacteria;Bacteria\_none;Bacteroidota;Flavobacteriia;Flavobacteriales;Weeksellaceae;Empedobacter;Empedobacter stercoris |
| Epilithonimonas zeae | 0 | 0 | 0 | 0 | 0 | 1 | 0 | 0 | 0 | 0 | 0 | 0 | 1 | 0 | Bacteria | Bacteria\_none | Bacteroidota | Flavobacteriia | Flavobacteriales | Weeksellaceae | Epilithonimonas | Bacteria;Bacteria\_none;Bacteroidota;Flavobacteriia;Flavobacteriales;Weeksellaceae;Epilithonimonas;Epilithonimonas zeae |
| Candidatus Ornithobacterium hominis | 1 | 0 | 0 | 0 | 0 | 0 | 0 | 1 | 0 | 0 | 0 | 0 | 0 | 0 | Bacteria | Bacteria\_none | Bacteroidota | Flavobacteriia | Flavobacteriales | Weeksellaceae | Ornithobacterium | Bacteria;Bacteria\_none;Bacteroidota;Flavobacteriia;Flavobacteriales;Weeksellaceae;Ornithobacterium;Candidatus Ornithobacterium hominis |
| Riemerella anatipestifer | 0 | 0 | 0 | 2 | 1 | 0 | 0 | 0 | 0 | 0 | 0 | 0 | 0 | 0 | Bacteria | Bacteria\_none | Bacteroidota | Flavobacteriia | Flavobacteriales | Weeksellaceae | Riemerella | Bacteria;Bacteria\_none;Bacteroidota;Flavobacteriia;Flavobacteriales;Weeksellaceae;Riemerella;Riemerella anatipestifer |
| Riemerella columbina | 0 | 0 | 0 | 0 | 0 | 1 | 0 | 0 | 0 | 0 | 0 | 0 | 0 | 0 | Bacteria | Bacteria\_none | Bacteroidota | Flavobacteriia | Flavobacteriales | Weeksellaceae | Riemerella | Bacteria;Bacteria\_none;Bacteroidota;Flavobacteriia;Flavobacteriales;Weeksellaceae;Riemerella;Riemerella columbina |
| Haliscomenobacter hydrossis | 0 | 0 | 0 | 0 | 0 | 0 | 0 | 0 | 0 | 0 | 0 | 0 | 0 | 0 | Bacteria | Bacteria\_none | Bacteroidota | Saprospiria | Saprospirales | Haliscomenobacteraceae | Haliscomenobacter | Bacteria;Bacteria\_none;Bacteroidota;Saprospiria;Saprospirales;Haliscomenobacteraceae;Haliscomenobacter;Haliscomenobacter hydrossis |
| Aurantibacillus circumpalustris | 0 | 0 | 0 | 1 | 0 | 0 | 0 | 0 | 0 | 0 | 0 | 0 | 0 | 0 | Bacteria | Bacteria\_none | Bacteroidota | Sphingobacteriia | Sphingobacteriales | Aurantibacillaceae | Aurantibacillus | Bacteria;Bacteria\_none;Bacteroidota;Sphingobacteriia;Sphingobacteriales;Aurantibacillaceae;Aurantibacillus;Aurantibacillus circumpalustris |
| Mucilaginibacter gotjawali | 0 | 0 | 0 | 0 | 0 | 0 | 0 | 0 | 0 | 0 | 0 | 0 | 0 | 0 | Bacteria | Bacteria\_none | Bacteroidota | Sphingobacteriia | Sphingobacteriales | Sphingobacteriaceae | Mucilaginibacter | Bacteria;Bacteria\_none;Bacteroidota;Sphingobacteriia;Sphingobacteriales;Sphingobacteriaceae;Mucilaginibacter;Mucilaginibacter gotjawali |
| Mucilaginibacter mallensis | 0 | 0 | 0 | 0 | 0 | 0 | 0 | 0 | 0 | 0 | 0 | 0 | 0 | 0 | Bacteria | Bacteria\_none | Bacteroidota | Sphingobacteriia | Sphingobacteriales | Sphingobacteriaceae | Mucilaginibacter | Bacteria;Bacteria\_none;Bacteroidota;Sphingobacteriia;Sphingobacteriales;Sphingobacteriaceae;Mucilaginibacter;Mucilaginibacter mallensis |
| Mucilaginibacter robiniae | 0 | 0 | 0 | 0 | 0 | 0 | 0 | 0 | 0 | 0 | 0 | 0 | 0 | 0 | Bacteria | Bacteria\_none | Bacteroidota | Sphingobacteriia | Sphingobacteriales | Sphingobacteriaceae | Mucilaginibacter | Bacteria;Bacteria\_none;Bacteroidota;Sphingobacteriia;Sphingobacteriales;Sphingobacteriaceae;Mucilaginibacter;Mucilaginibacter robiniae |
| Mucilaginibacter sp. 14171R-50 | 0 | 1 | 0 | 0 | 0 | 0 | 0 | 0 | 0 | 0 | 0 | 0 | 0 | 0 | Bacteria | Bacteria\_none | Bacteroidota | Sphingobacteriia | Sphingobacteriales | Sphingobacteriaceae | Mucilaginibacter | Bacteria;Bacteria\_none;Bacteroidota;Sphingobacteriia;Sphingobacteriales;Sphingobacteriaceae;Mucilaginibacter;Mucilaginibacter sp. 14171R-50 |
| Mucilaginibacter sp. SMC90 | 0 | 0 | 0 | 0 | 0 | 0 | 0 | 0 | 0 | 0 | 0 | 0 | 0 | 0 | Bacteria | Bacteria\_none | Bacteroidota | Sphingobacteriia | Sphingobacteriales | Sphingobacteriaceae | Mucilaginibacter | Bacteria;Bacteria\_none;Bacteroidota;Sphingobacteriia;Sphingobacteriales;Sphingobacteriaceae;Mucilaginibacter;Mucilaginibacter sp. SMC90 |
| Olivibacter sp. SDN3 | 0 | 0 | 0 | 0 | 0 | 0 | 0 | 0 | 0 | 0 | 0 | 0 | 0 | 0 | Bacteria | Bacteria\_none | Bacteroidota | Sphingobacteriia | Sphingobacteriales | Sphingobacteriaceae | Olivibacter | Bacteria;Bacteria\_none;Bacteroidota;Sphingobacteriia;Sphingobacteriales;Sphingobacteriaceae;Olivibacter;Olivibacter sp. SDN3 |
| Pedobacter gandavensis | 0 | 0 | 0 | 0 | 0 | 0 | 0 | 0 | 0 | 0 | 0 | 0 | 0 | 0 | Bacteria | Bacteria\_none | Bacteroidota | Sphingobacteriia | Sphingobacteriales | Sphingobacteriaceae | Pedobacter | Bacteria;Bacteria\_none;Bacteroidota;Sphingobacteriia;Sphingobacteriales;Sphingobacteriaceae;Pedobacter;Pedobacter gandavensis |
| Pedobacter ginsengisoli | 0 | 0 | 0 | 0 | 0 | 0 | 0 | 0 | 0 | 0 | 0 | 0 | 0 | 0 | Bacteria | Bacteria\_none | Bacteroidota | Sphingobacteriia | Sphingobacteriales | Sphingobacteriaceae | Pedobacter | Bacteria;Bacteria\_none;Bacteroidota;Sphingobacteriia;Sphingobacteriales;Sphingobacteriaceae;Pedobacter;Pedobacter ginsengisoli |
| Pedobacter roseus | 0 | 0 | 0 | 0 | 0 | 0 | 0 | 0 | 0 | 0 | 0 | 0 | 0 | 0 | Bacteria | Bacteria\_none | Bacteroidota | Sphingobacteriia | Sphingobacteriales | Sphingobacteriaceae | Pedobacter | Bacteria;Bacteria\_none;Bacteroidota;Sphingobacteriia;Sphingobacteriales;Sphingobacteriaceae;Pedobacter;Pedobacter roseus |
| Pedobacter sp. SL55 | 0 | 0 | 0 | 0 | 0 | 1 | 0 | 0 | 0 | 0 | 0 | 0 | 0 | 0 | Bacteria | Bacteria\_none | Bacteroidota | Sphingobacteriia | Sphingobacteriales | Sphingobacteriaceae | Pedobacter | Bacteria;Bacteria\_none;Bacteroidota;Sphingobacteriia;Sphingobacteriales;Sphingobacteriaceae;Pedobacter;Pedobacter sp. SL55 |
| Solitalea lacus | 0 | 0 | 0 | 0 | 0 | 0 | 0 | 0 | 0 | 0 | 0 | 0 | 0 | 0 | Bacteria | Bacteria\_none | Bacteroidota | Sphingobacteriia | Sphingobacteriales | Sphingobacteriaceae | Solitalea | Bacteria;Bacteria\_none;Bacteroidota;Sphingobacteriia;Sphingobacteriales;Sphingobacteriaceae;Solitalea;Solitalea lacus |
| Sphingobacterium sp. LZ7M1 | 0 | 0 | 0 | 0 | 0 | 0 | 0 | 0 | 0 | 0 | 0 | 0 | 0 | 0 | Bacteria | Bacteria\_none | Bacteroidota | Sphingobacteriia | Sphingobacteriales | Sphingobacteriaceae | Sphingobacterium | Bacteria;Bacteria\_none;Bacteroidota;Sphingobacteriia;Sphingobacteriales;Sphingobacteriaceae;Sphingobacterium;Sphingobacterium sp. LZ7M1 |
| Sphingobacterium sp. SYP-B4668 | 0 | 0 | 0 | 0 | 1 | 0 | 0 | 0 | 0 | 0 | 0 | 0 | 0 | 0 | Bacteria | Bacteria\_none | Bacteroidota | Sphingobacteriia | Sphingobacteriales | Sphingobacteriaceae | Sphingobacterium | Bacteria;Bacteria\_none;Bacteroidota;Sphingobacteriia;Sphingobacteriales;Sphingobacteriaceae;Sphingobacterium;Sphingobacterium sp. SYP-B4668 |
| Sphingobacterium spiritivorum | 0 | 0 | 0 | 0 | 0 | 0 | 0 | 0 | 0 | 0 | 0 | 0 | 0 | 0 | Bacteria | Bacteria\_none | Bacteroidota | Sphingobacteriia | Sphingobacteriales | Sphingobacteriaceae | Sphingobacterium | Bacteria;Bacteria\_none;Bacteroidota;Sphingobacteriia;Sphingobacteriales;Sphingobacteriaceae;Sphingobacterium;Sphingobacterium spiritivorum |
| Cyclonatronum proteinivorum | 0 | 0 | 0 | 0 | 0 | 0 | 0 | 0 | 0 | 0 | 0 | 0 | 0 | 0 | Bacteria | Bacteria\_none | Balneolota | Balneolia | Balneolales | Cyclonatronaceae | Cyclonatronum | Bacteria;Bacteria\_none;Balneolota;Balneolia;Balneolales;Cyclonatronaceae;Cyclonatronum;Cyclonatronum proteinivorum |
| Bdellovibrio bacteriovorus | 0 | 0 | 0 | 0 | 0 | 0 | 0 | 0 | 0 | 0 | 0 | 0 | 1 | 0 | Bacteria | Bacteria\_none | Bdellovibrionota | Bdellovibrionia | Bdellovibrionales | Pseudobdellovibrionaceae | Bdellovibrio | Bacteria;Bacteria\_none;Bdellovibrionota;Bdellovibrionia;Bdellovibrionales;Pseudobdellovibrionaceae;Bdellovibrio;Bdellovibrio bacteriovorus |
| Caldithrix abyssi | 0 | 0 | 0 | 0 | 0 | 0 | 0 | 0 | 0 | 0 | 0 | 0 | 0 | 0 | Bacteria | Bacteria\_none | Calditrichota | Calditrichia | Calditrichales | Calditrichaceae | Caldithrix | Bacteria;Bacteria\_none;Calditrichota;Calditrichia;Calditrichales;Calditrichaceae;Caldithrix;Caldithrix abyssi |
| Aliarcobacter butzleri | 0 | 0 | 0 | 0 | 0 | 0 | 0 | 0 | 0 | 0 | 0 | 0 | 0 | 1 | Bacteria | Bacteria\_none | Campylobacterota | Epsilonproteobacteria | Campylobacterales | Arcobacteraceae | Aliarcobacter | Bacteria;Bacteria\_none;Campylobacterota;Epsilonproteobacteria;Campylobacterales;Arcobacteraceae;Aliarcobacter;Aliarcobacter butzleri |
| Aliarcobacter cryaerophilus | 0 | 0 | 0 | 0 | 0 | 0 | 0 | 0 | 0 | 0 | 0 | 0 | 0 | 0 | Bacteria | Bacteria\_none | Campylobacterota | Epsilonproteobacteria | Campylobacterales | Arcobacteraceae | Aliarcobacter | Bacteria;Bacteria\_none;Campylobacterota;Epsilonproteobacteria;Campylobacterales;Arcobacteraceae;Aliarcobacter;Aliarcobacter cryaerophilus |
| Arcobacter ellisii | 0 | 0 | 1 | 0 | 0 | 0 | 0 | 0 | 0 | 0 | 0 | 0 | 0 | 0 | Bacteria | Bacteria\_none | Campylobacterota | Epsilonproteobacteria | Campylobacterales | Arcobacteraceae | Arcobacter | Bacteria;Bacteria\_none;Campylobacterota;Epsilonproteobacteria;Campylobacterales;Arcobacteraceae;Arcobacter;Arcobacter ellisii |
| Arcobacter suis | 0 | 0 | 1 | 0 | 0 | 0 | 0 | 0 | 0 | 0 | 0 | 0 | 0 | 0 | Bacteria | Bacteria\_none | Campylobacterota | Epsilonproteobacteria | Campylobacterales | Arcobacteraceae | Arcobacter | Bacteria;Bacteria\_none;Campylobacterota;Epsilonproteobacteria;Campylobacterales;Arcobacteraceae;Arcobacter;Arcobacter suis |
| Arcobacter venerupis | 0 | 0 | 0 | 0 | 0 | 0 | 0 | 0 | 0 | 0 | 0 | 0 | 0 | 0 | Bacteria | Bacteria\_none | Campylobacterota | Epsilonproteobacteria | Campylobacterales | Arcobacteraceae | Arcobacter | Bacteria;Bacteria\_none;Campylobacterota;Epsilonproteobacteria;Campylobacterales;Arcobacteraceae;Arcobacter;Arcobacter venerupis |
| Halarcobacter sp. | 0 | 0 | 0 | 0 | 0 | 0 | 0 | 0 | 0 | 0 | 0 | 0 | 0 | 0 | Bacteria | Bacteria\_none | Campylobacterota | Epsilonproteobacteria | Campylobacterales | Arcobacteraceae | Halarcobacter | Bacteria;Bacteria\_none;Campylobacterota;Epsilonproteobacteria;Campylobacterales;Arcobacteraceae;Halarcobacter;Halarcobacter sp. |
| Malaciobacter pacificus | 0 | 0 | 0 | 0 | 0 | 0 | 0 | 0 | 0 | 0 | 0 | 0 | 0 | 0 | Bacteria | Bacteria\_none | Campylobacterota | Epsilonproteobacteria | Campylobacterales | Arcobacteraceae | Malaciobacter | Bacteria;Bacteria\_none;Campylobacterota;Epsilonproteobacteria;Campylobacterales;Arcobacteraceae;Malaciobacter;Malaciobacter pacificus |
| Campylobacter coli | 0 | 1 | 0 | 0 | 0 | 0 | 0 | 0 | 0 | 0 | 0 | 0 | 0 | 0 | Bacteria | Bacteria\_none | Campylobacterota | Epsilonproteobacteria | Campylobacterales | Campylobacteraceae | Campylobacter | Bacteria;Bacteria\_none;Campylobacterota;Epsilonproteobacteria;Campylobacterales;Campylobacteraceae;Campylobacter;Campylobacter coli |
| Campylobacter concisus | 0 | 25 | 1 | 0 | 2 | 31 | 0 | 1 | 3 | 0 | 0 | 1 | 3 | 6 | Bacteria | Bacteria\_none | Campylobacterota | Epsilonproteobacteria | Campylobacterales | Campylobacteraceae | Campylobacter | Bacteria;Bacteria\_none;Campylobacterota;Epsilonproteobacteria;Campylobacterales;Campylobacteraceae;Campylobacter;Campylobacter concisus |
| Campylobacter cuniculorum | 0 | 0 | 0 | 0 | 0 | 0 | 0 | 0 | 0 | 0 | 0 | 0 | 0 | 0 | Bacteria | Bacteria\_none | Campylobacterota | Epsilonproteobacteria | Campylobacterales | Campylobacteraceae | Campylobacter | Bacteria;Bacteria\_none;Campylobacterota;Epsilonproteobacteria;Campylobacterales;Campylobacteraceae;Campylobacter;Campylobacter cuniculorum |
| Campylobacter curvus | 0 | 0 | 1 | 0 | 0 | 0 | 1 | 0 | 0 | 0 | 0 | 0 | 0 | 0 | Bacteria | Bacteria\_none | Campylobacterota | Epsilonproteobacteria | Campylobacterales | Campylobacteraceae | Campylobacter | Bacteria;Bacteria\_none;Campylobacterota;Epsilonproteobacteria;Campylobacterales;Campylobacteraceae;Campylobacter;Campylobacter curvus |
| Campylobacter gracilis | 0 | 2 | 1 | 1 | 0 | 0 | 0 | 0 | 0 | 0 | 0 | 1 | 0 | 0 | Bacteria | Bacteria\_none | Campylobacterota | Epsilonproteobacteria | Campylobacterales | Campylobacteraceae | Campylobacter | Bacteria;Bacteria\_none;Campylobacterota;Epsilonproteobacteria;Campylobacterales;Campylobacteraceae;Campylobacter;Campylobacter gracilis |
| Campylobacter hyointestinalis | 0 | 0 | 0 | 0 | 0 | 0 | 0 | 0 | 0 | 0 | 0 | 1 | 0 | 0 | Bacteria | Bacteria\_none | Campylobacterota | Epsilonproteobacteria | Campylobacterales | Campylobacteraceae | Campylobacter | Bacteria;Bacteria\_none;Campylobacterota;Epsilonproteobacteria;Campylobacterales;Campylobacteraceae;Campylobacter;Campylobacter hyointestinalis |
| Campylobacter insulaenigrae | 0 | 0 | 0 | 0 | 0 | 2 | 0 | 0 | 0 | 0 | 0 | 0 | 0 | 0 | Bacteria | Bacteria\_none | Campylobacterota | Epsilonproteobacteria | Campylobacterales | Campylobacteraceae | Campylobacter | Bacteria;Bacteria\_none;Campylobacterota;Epsilonproteobacteria;Campylobacterales;Campylobacteraceae;Campylobacter;Campylobacter insulaenigrae |
| Campylobacter lanienae | 0 | 0 | 0 | 0 | 0 | 0 | 0 | 0 | 0 | 0 | 0 | 0 | 0 | 0 | Bacteria | Bacteria\_none | Campylobacterota | Epsilonproteobacteria | Campylobacterales | Campylobacteraceae | Campylobacter | Bacteria;Bacteria\_none;Campylobacterota;Epsilonproteobacteria;Campylobacterales;Campylobacteraceae;Campylobacter;Campylobacter lanienae |
| Campylobacter peloridis | 0 | 0 | 0 | 1 | 0 | 0 | 0 | 0 | 0 | 0 | 0 | 0 | 0 | 0 | Bacteria | Bacteria\_none | Campylobacterota | Epsilonproteobacteria | Campylobacterales | Campylobacteraceae | Campylobacter | Bacteria;Bacteria\_none;Campylobacterota;Epsilonproteobacteria;Campylobacterales;Campylobacteraceae;Campylobacter;Campylobacter peloridis |
| Campylobacter pinnipediorum | 0 | 0 | 0 | 0 | 0 | 0 | 0 | 0 | 0 | 0 | 0 | 0 | 0 | 0 | Bacteria | Bacteria\_none | Campylobacterota | Epsilonproteobacteria | Campylobacterales | Campylobacteraceae | Campylobacter | Bacteria;Bacteria\_none;Campylobacterota;Epsilonproteobacteria;Campylobacterales;Campylobacteraceae;Campylobacter;Campylobacter pinnipediorum |
| Campylobacter rectus | 0 | 9 | 1 | 2 | 0 | 4 | 0 | 2 | 0 | 1 | 1 | 2 | 1 | 0 | Bacteria | Bacteria\_none | Campylobacterota | Epsilonproteobacteria | Campylobacterales | Campylobacteraceae | Campylobacter | Bacteria;Bacteria\_none;Campylobacterota;Epsilonproteobacteria;Campylobacterales;Campylobacteraceae;Campylobacter;Campylobacter rectus |
| Campylobacter showae | 0 | 7 | 10 | 2 | 1 | 2 | 0 | 0 | 1 | 2 | 0 | 1 | 1 | 0 | Bacteria | Bacteria\_none | Campylobacterota | Epsilonproteobacteria | Campylobacterales | Campylobacteraceae | Campylobacter | Bacteria;Bacteria\_none;Campylobacterota;Epsilonproteobacteria;Campylobacterales;Campylobacteraceae;Campylobacter;Campylobacter showae |
| Campylobacter sp. RM16187 | 0 | 0 | 0 | 0 | 0 | 0 | 0 | 0 | 0 | 0 | 0 | 0 | 0 | 0 | Bacteria | Bacteria\_none | Campylobacterota | Epsilonproteobacteria | Campylobacterales | Campylobacteraceae | Campylobacter | Bacteria;Bacteria\_none;Campylobacterota;Epsilonproteobacteria;Campylobacterales;Campylobacteraceae;Campylobacter;Campylobacter sp. RM16187 |
| Campylobacter sp. RM5004 | 0 | 0 | 0 | 0 | 0 | 0 | 0 | 0 | 0 | 0 | 0 | 0 | 0 | 0 | Bacteria | Bacteria\_none | Campylobacterota | Epsilonproteobacteria | Campylobacterales | Campylobacteraceae | Campylobacter | Bacteria;Bacteria\_none;Campylobacterota;Epsilonproteobacteria;Campylobacterales;Campylobacteraceae;Campylobacter;Campylobacter sp. RM5004 |
| Campylobacter ureolyticus | 0 | 1 | 0 | 0 | 0 | 0 | 0 | 0 | 0 | 0 | 0 | 0 | 0 | 0 | Bacteria | Bacteria\_none | Campylobacterota | Epsilonproteobacteria | Campylobacterales | Campylobacteraceae | Campylobacter | Bacteria;Bacteria\_none;Campylobacterota;Epsilonproteobacteria;Campylobacterales;Campylobacteraceae;Campylobacter;Campylobacter ureolyticus |
| Candidatus Campylobacter infans | 0 | 0 | 0 | 0 | 0 | 0 | 0 | 0 | 0 | 0 | 0 | 0 | 0 | 0 | Bacteria | Bacteria\_none | Campylobacterota | Epsilonproteobacteria | Campylobacterales | Campylobacteraceae | Campylobacter | Bacteria;Bacteria\_none;Campylobacterota;Epsilonproteobacteria;Campylobacterales;Campylobacteraceae;Campylobacter;Candidatus Campylobacter infans |
| Helicobacter canadensis | 0 | 0 | 0 | 0 | 0 | 0 | 0 | 0 | 0 | 0 | 0 | 0 | 0 | 0 | Bacteria | Bacteria\_none | Campylobacterota | Epsilonproteobacteria | Campylobacterales | Helicobacteraceae | Helicobacter | Bacteria;Bacteria\_none;Campylobacterota;Epsilonproteobacteria;Campylobacterales;Helicobacteraceae;Helicobacter;Helicobacter canadensis |
| Helicobacter heilmannii | 0 | 0 | 0 | 0 | 0 | 1 | 0 | 0 | 0 | 0 | 0 | 0 | 0 | 0 | Bacteria | Bacteria\_none | Campylobacterota | Epsilonproteobacteria | Campylobacterales | Helicobacteraceae | Helicobacter | Bacteria;Bacteria\_none;Campylobacterota;Epsilonproteobacteria;Campylobacterales;Helicobacteraceae;Helicobacter;Helicobacter heilmannii |
| Helicobacter pylori | 0 | 0 | 0 | 0 | 1 | 0 | 0 | 0 | 0 | 0 | 0 | 0 | 0 | 0 | Bacteria | Bacteria\_none | Campylobacterota | Epsilonproteobacteria | Campylobacterales | Helicobacteraceae | Helicobacter | Bacteria;Bacteria\_none;Campylobacterota;Epsilonproteobacteria;Campylobacterales;Helicobacteraceae;Helicobacter;Helicobacter pylori |
| Sulfurospirillum barnesii | 0 | 0 | 0 | 0 | 0 | 0 | 0 | 0 | 0 | 0 | 0 | 0 | 0 | 0 | Bacteria | Bacteria\_none | Campylobacterota | Epsilonproteobacteria | Campylobacterales | Sulfurospirillaceae | Sulfurospirillum | Bacteria;Bacteria\_none;Campylobacterota;Epsilonproteobacteria;Campylobacterales;Sulfurospirillaceae;Sulfurospirillum;Sulfurospirillum barnesii |
| Sulfurovum indicum | 1 | 0 | 0 | 0 | 0 | 0 | 0 | 0 | 0 | 0 | 0 | 0 | 0 | 0 | Bacteria | Bacteria\_none | Campylobacterota | Epsilonproteobacteria | Campylobacterales | Sulfurovaceae | Sulfurovum | Bacteria;Bacteria\_none;Campylobacterota;Epsilonproteobacteria;Campylobacterales;Sulfurovaceae;Sulfurovum;Sulfurovum indicum |
| Candidatus Minimicrobia sp. QA0096 | 0 | 0 | 0 | 0 | 2 | 4 | 0 | 0 | 0 | 0 | 0 | 0 | 0 | 0 | Bacteria | Bacteria\_none | Candidatus Saccharibacteria | Candidatus Saccharibacteria\_Incertae\_sedis | Candidatus Saccharibacteria\_Incertae\_sedis | Candidatus Saccharibacteria\_Incertae\_sedis | Candidatus Minimicrobia | Bacteria;Bacteria\_none;Candidatus Saccharibacteria;Candidatus Saccharibacteria\_Incertae\_sedis;Candidatus Saccharibacteria\_Incertae\_sedis;Candidatus Saccharibacteria\_Incertae\_sedis;Candidatus Minimicrobia;Candidatus Minimicrobia sp. QA0096 |
| Candidatus Minimicrobia vallesae | 0 | 0 | 0 | 0 | 0 | 13 | 0 | 0 | 0 | 0 | 1 | 1 | 0 | 0 | Bacteria | Bacteria\_none | Candidatus Saccharibacteria | Candidatus Saccharibacteria\_Incertae\_sedis | Candidatus Saccharibacteria\_Incertae\_sedis | Candidatus Saccharibacteria\_Incertae\_sedis | Candidatus Minimicrobia | Bacteria;Bacteria\_none;Candidatus Saccharibacteria;Candidatus Saccharibacteria\_Incertae\_sedis;Candidatus Saccharibacteria\_Incertae\_sedis;Candidatus Saccharibacteria\_Incertae\_sedis;Candidatus Minimicrobia;Candidatus Minimicrobia vallesae |
| Candidatus Mycosynbacter amalyticus | 0 | 0 | 0 | 0 | 0 | 0 | 0 | 0 | 0 | 0 | 0 | 0 | 0 | 0 | Bacteria | Bacteria\_none | Candidatus Saccharibacteria | Candidatus Saccharibacteria\_Incertae\_sedis | Candidatus Saccharibacteria\_Incertae\_sedis | Candidatus Saccharibacteria\_Incertae\_sedis | Candidatus Mycosynbacter | Bacteria;Bacteria\_none;Candidatus Saccharibacteria;Candidatus Saccharibacteria\_Incertae\_sedis;Candidatus Saccharibacteria\_Incertae\_sedis;Candidatus Saccharibacteria\_Incertae\_sedis;Candidatus Mycosynbacter;Candidatus Mycosynbacter amalyticus |
| Candidatus Nanosynbacter featherlites | 0 | 1 | 3 | 0 | 1 | 1 | 1 | 0 | 0 | 0 | 13 | 1 | 2 | 0 | Bacteria | Bacteria\_none | Candidatus Saccharibacteria | Candidatus Saccharimonadia | Candidatus Nanosynbacterales | Candidatus Nanosynbacteraceae | Candidatus Nanosynbacter | Bacteria;Bacteria\_none;Candidatus Saccharibacteria;Candidatus Saccharimonadia;Candidatus Nanosynbacterales;Candidatus Nanosynbacteraceae;Candidatus Nanosynbacter;Candidatus Nanosynbacter featherlites |
| Candidatus Nanosynbacter lyticus | 0 | 2 | 1 | 0 | 2 | 3 | 0 | 0 | 0 | 0 | 27 | 0 | 4 | 1 | Bacteria | Bacteria\_none | Candidatus Saccharibacteria | Candidatus Saccharimonadia | Candidatus Nanosynbacterales | Candidatus Nanosynbacteraceae | Candidatus Nanosynbacter | Bacteria;Bacteria\_none;Candidatus Saccharibacteria;Candidatus Saccharimonadia;Candidatus Nanosynbacterales;Candidatus Nanosynbacteraceae;Candidatus Nanosynbacter;Candidatus Nanosynbacter lyticus |
| Candidatus Nanosynbacter sp. HMT-352 | 0 | 5 | 0 | 0 | 3 | 119 | 0 | 0 | 1 | 0 | 0 | 0 | 7 | 3 | Bacteria | Bacteria\_none | Candidatus Saccharibacteria | Candidatus Saccharimonadia | Candidatus Nanosynbacterales | Candidatus Nanosynbacteraceae | Candidatus Nanosynbacter | Bacteria;Bacteria\_none;Candidatus Saccharibacteria;Candidatus Saccharimonadia;Candidatus Nanosynbacterales;Candidatus Nanosynbacteraceae;Candidatus Nanosynbacter;Candidatus Nanosynbacter sp. HMT-352 |
| Candidatus Saccharimonas aalborgensis | 0 | 0 | 0 | 0 | 1 | 0 | 0 | 1 | 0 | 0 | 1 | 0 | 0 | 0 | Bacteria | Bacteria\_none | Candidatus Saccharibacteria | Candidatus Saccharimonadia | Candidatus Saccharimonadales | Candidatus Saccharimonadaceae | Candidatus Saccharimonas | Bacteria;Bacteria\_none;Candidatus Saccharibacteria;Candidatus Saccharimonadia;Candidatus Saccharimonadales;Candidatus Saccharimonadaceae;Candidatus Saccharimonas;Candidatus Saccharimonas aalborgensis |
| Candidatus Protochlamydia naegleriophila | 0 | 0 | 0 | 0 | 0 | 0 | 0 | 0 | 0 | 0 | 0 | 0 | 0 | 0 | Bacteria | Bacteria\_none | Chlamydiota | Chlamydiia | Parachlamydiales | Parachlamydiaceae | Candidatus Protochlamydia | Bacteria;Bacteria\_none;Chlamydiota;Chlamydiia;Parachlamydiales;Parachlamydiaceae;Candidatus Protochlamydia;Candidatus Protochlamydia naegleriophila |
| Chloroherpeton thalassium | 0 | 0 | 0 | 0 | 0 | 1 | 0 | 0 | 0 | 0 | 0 | 0 | 0 | 0 | Bacteria | Bacteria\_none | Chlorobiota | Chlorobiia | Chlorobiales | Chloroherpetonaceae | Chloroherpeton | Bacteria;Bacteria\_none;Chlorobiota;Chlorobiia;Chlorobiales;Chloroherpetonaceae;Chloroherpeton;Chloroherpeton thalassium |
| Candidatus Chlorohelix allophototropha | 0 | 0 | 0 | 0 | 0 | 0 | 0 | 0 | 0 | 0 | 0 | 0 | 0 | 0 | Bacteria | Bacteria\_none | Chloroflexota | Chloroflexia | Candidatus Chloroheliales | Candidatus Chloroheliaceae | Candidatus Chlorohelix | Bacteria;Bacteria\_none;Chloroflexota;Chloroflexia;Candidatus Chloroheliales;Candidatus Chloroheliaceae;Candidatus Chlorohelix;Candidatus Chlorohelix allophototropha |
| Candidatus Lucifugimonas marina | 0 | 0 | 0 | 0 | 0 | 0 | 0 | 0 | 0 | 0 | 0 | 0 | 0 | 0 | Bacteria | Bacteria\_none | Chloroflexota | Dehalococcoidia | Candidatus Lucifugimonadales | Candidatus Lucifugimonadaceae | Candidatus Lucifugimonas | Bacteria;Bacteria\_none;Chloroflexota;Dehalococcoidia;Candidatus Lucifugimonadales;Candidatus Lucifugimonadaceae;Candidatus Lucifugimonas;Candidatus Lucifugimonas marina |
| cyanobacterium endosymbiont of Epithemia clementina EcSB | 1 | 0 | 0 | 0 | 0 | 0 | 0 | 0 | 0 | 0 | 0 | 0 | 0 | 0 | Bacteria | Bacteria\_none | Cyanobacteriota | Cyanobacteriota\_Incertae\_sedis | Cyanobacteriota\_Incertae\_sedis | Cyanobacteriota\_Incertae\_sedis | Cyanobacteriota\_Incertae\_sedis | Bacteria;Bacteria\_none;Cyanobacteriota;Cyanobacteriota\_Incertae\_sedis;Cyanobacteriota\_Incertae\_sedis;Cyanobacteriota\_Incertae\_sedis;Cyanobacteriota\_Incertae\_sedis;cyanobacterium endosymbiont of Epithemia clementina EcSB |
| Gloeocapsa sp. PCC 7428 | 0 | 0 | 1 | 0 | 0 | 0 | 0 | 0 | 0 | 0 | 0 | 0 | 0 | 0 | Bacteria | Bacteria\_none | Cyanobacteriota | Cyanophyceae | Chroococcales | Chroococcaceae | Gloeocapsa | Bacteria;Bacteria\_none;Cyanobacteriota;Cyanophyceae;Chroococcales;Chroococcaceae;Gloeocapsa;Gloeocapsa sp. PCC 7428 |
| Geminocystis herdmanii | 0 | 0 | 0 | 0 | 0 | 0 | 0 | 0 | 0 | 0 | 0 | 0 | 0 | 0 | Bacteria | Bacteria\_none | Cyanobacteriota | Cyanophyceae | Chroococcales | Geminocystaceae | Geminocystis | Bacteria;Bacteria\_none;Cyanobacteriota;Cyanophyceae;Chroococcales;Geminocystaceae;Geminocystis;Geminocystis herdmanii |
| Gloeobacter morelensis | 0 | 0 | 0 | 0 | 0 | 0 | 0 | 0 | 0 | 0 | 0 | 0 | 1 | 0 | Bacteria | Bacteria\_none | Cyanobacteriota | Cyanophyceae | Gloeobacterales | Gloeobacteraceae | Gloeobacter | Bacteria;Bacteria\_none;Cyanobacteriota;Cyanophyceae;Gloeobacterales;Gloeobacteraceae;Gloeobacter;Gloeobacter morelensis |
| Cylindrospermopsis raciborskii | 0 | 0 | 0 | 0 | 0 | 0 | 0 | 0 | 0 | 0 | 0 | 0 | 0 | 0 | Bacteria | Bacteria\_none | Cyanobacteriota | Cyanophyceae | Nostocales | Aphanizomenonaceae | Cylindrospermopsis | Bacteria;Bacteria\_none;Cyanobacteriota;Cyanophyceae;Nostocales;Aphanizomenonaceae;Cylindrospermopsis;Cylindrospermopsis raciborskii |
| Nostoc sp. 'Lobaria pulmonaria (5183) cyanobiont' | 0 | 0 | 0 | 0 | 0 | 0 | 0 | 0 | 0 | 0 | 0 | 0 | 0 | 0 | Bacteria | Bacteria\_none | Cyanobacteriota | Cyanophyceae | Nostocales | Nostocaceae | Nostoc | Bacteria;Bacteria\_none;Cyanobacteriota;Cyanophyceae;Nostocales;Nostocaceae;Nostoc;Nostoc sp. 'Lobaria pulmonaria (5183) cyanobiont' |
| Nostoc sp. 'Peltigera membranacea cyanobiont' N6 | 0 | 1 | 0 | 0 | 0 | 0 | 0 | 0 | 0 | 0 | 0 | 0 | 0 | 0 | Bacteria | Bacteria\_none | Cyanobacteriota | Cyanophyceae | Nostocales | Nostocaceae | Nostoc | Bacteria;Bacteria\_none;Cyanobacteriota;Cyanophyceae;Nostocales;Nostocaceae;Nostoc;Nostoc sp. 'Peltigera membranacea cyanobiont' N6 |
| Nostoc sp. NIES-3756 | 0 | 0 | 0 | 0 | 0 | 0 | 0 | 0 | 0 | 0 | 0 | 0 | 0 | 0 | Bacteria | Bacteria\_none | Cyanobacteriota | Cyanophyceae | Nostocales | Nostocaceae | Nostoc | Bacteria;Bacteria\_none;Cyanobacteriota;Cyanophyceae;Nostocales;Nostocaceae;Nostoc;Nostoc sp. NIES-3756 |
| Nostoc sp. TCL26-01 | 0 | 0 | 0 | 0 | 0 | 0 | 0 | 0 | 0 | 1 | 0 | 0 | 1 | 0 | Bacteria | Bacteria\_none | Cyanobacteriota | Cyanophyceae | Nostocales | Nostocaceae | Nostoc | Bacteria;Bacteria\_none;Cyanobacteriota;Cyanophyceae;Nostocales;Nostocaceae;Nostoc;Nostoc sp. TCL26-01 |
| Nostoc sphaeroides | 0 | 0 | 0 | 0 | 0 | 0 | 0 | 0 | 0 | 0 | 0 | 0 | 0 | 0 | Bacteria | Bacteria\_none | Cyanobacteriota | Cyanophyceae | Nostocales | Nostocaceae | Nostoc | Bacteria;Bacteria\_none;Cyanobacteriota;Cyanophyceae;Nostocales;Nostocaceae;Nostoc;Nostoc sphaeroides |
| Prochlorococcus marinus | 0 | 0 | 0 | 0 | 1 | 0 | 0 | 0 | 0 | 0 | 0 | 0 | 0 | 0 | Bacteria | Bacteria\_none | Cyanobacteriota | Cyanophyceae | Synechococcales | Prochlorococcaceae | Prochlorococcus | Bacteria;Bacteria\_none;Cyanobacteriota;Cyanophyceae;Synechococcales;Prochlorococcaceae;Prochlorococcus;Prochlorococcus marinus |
| Synechococcus sp. JA-3-3Ab | 0 | 0 | 0 | 0 | 0 | 1 | 0 | 0 | 0 | 0 | 0 | 0 | 0 | 0 | Bacteria | Bacteria\_none | Cyanobacteriota | Cyanophyceae | Synechococcales | Synechococcaceae | Synechococcus | Bacteria;Bacteria\_none;Cyanobacteriota;Cyanophyceae;Synechococcales;Synechococcaceae;Synechococcus;Synechococcus sp. JA-3-3Ab |
| Calditerrivibrio nitroreducens | 0 | 1 | 0 | 0 | 0 | 0 | 0 | 0 | 0 | 0 | 0 | 0 | 0 | 0 | Bacteria | Bacteria\_none | Deferribacterota | Deferribacteres | Deferribacterales | Calditerrivibrionaceae | Calditerrivibrio | Bacteria;Bacteria\_none;Deferribacterota;Deferribacteres;Deferribacterales;Calditerrivibrionaceae;Calditerrivibrio;Calditerrivibrio nitroreducens |
| Mucispirillum schaedleri | 0 | 0 | 0 | 0 | 0 | 0 | 0 | 0 | 0 | 0 | 0 | 0 | 0 | 0 | Bacteria | Bacteria\_none | Deferribacterota | Deferribacteres | Deferribacterales | Mucispirillaceae | Mucispirillum | Bacteria;Bacteria\_none;Deferribacterota;Deferribacteres;Deferribacterales;Mucispirillaceae;Mucispirillum;Mucispirillum schaedleri |
| Cetobacterium somerae | 0 | 0 | 0 | 0 | 0 | 0 | 0 | 0 | 0 | 0 | 0 | 0 | 0 | 0 | Bacteria | Bacteria\_none | Fusobacteriota | Fusobacteriia | Fusobacteriales | Fusobacteriaceae | Cetobacterium | Bacteria;Bacteria\_none;Fusobacteriota;Fusobacteriia;Fusobacteriales;Fusobacteriaceae;Cetobacterium;Cetobacterium somerae |
| Fusobacterium animalis | 0 | 5 | 5 | 10 | 5 | 1 | 1 | 4 | 0 | 1 | 33 | 11 | 8 | 6 | Bacteria | Bacteria\_none | Fusobacteriota | Fusobacteriia | Fusobacteriales | Fusobacteriaceae | Fusobacterium | Bacteria;Bacteria\_none;Fusobacteriota;Fusobacteriia;Fusobacteriales;Fusobacteriaceae;Fusobacterium;Fusobacterium animalis |
| Fusobacterium canifelinum | 0 | 1 | 0 | 9 | 0 | 0 | 0 | 0 | 0 | 0 | 2 | 0 | 1 | 0 | Bacteria | Bacteria\_none | Fusobacteriota | Fusobacteriia | Fusobacteriales | Fusobacteriaceae | Fusobacterium | Bacteria;Bacteria\_none;Fusobacteriota;Fusobacteriia;Fusobacteriales;Fusobacteriaceae;Fusobacterium;Fusobacterium canifelinum |
| Fusobacterium gonidiaformans | 0 | 0 | 0 | 0 | 0 | 0 | 0 | 0 | 0 | 0 | 0 | 0 | 0 | 0 | Bacteria | Bacteria\_none | Fusobacteriota | Fusobacteriia | Fusobacteriales | Fusobacteriaceae | Fusobacterium | Bacteria;Bacteria\_none;Fusobacteriota;Fusobacteriia;Fusobacteriales;Fusobacteriaceae;Fusobacterium;Fusobacterium gonidiaformans |
| Fusobacterium hwasookii | 0 | 5 | 1 | 20 | 4 | 1 | 0 | 1 | 0 | 0 | 0 | 2 | 3 | 3 | Bacteria | Bacteria\_none | Fusobacteriota | Fusobacteriia | Fusobacteriales | Fusobacteriaceae | Fusobacterium | Bacteria;Bacteria\_none;Fusobacteriota;Fusobacteriia;Fusobacteriales;Fusobacteriaceae;Fusobacterium;Fusobacterium hwasookii |
| Fusobacterium mortiferum | 0 | 0 | 0 | 0 | 0 | 0 | 0 | 0 | 0 | 0 | 0 | 0 | 0 | 0 | Bacteria | Bacteria\_none | Fusobacteriota | Fusobacteriia | Fusobacteriales | Fusobacteriaceae | Fusobacterium | Bacteria;Bacteria\_none;Fusobacteriota;Fusobacteriia;Fusobacteriales;Fusobacteriaceae;Fusobacterium;Fusobacterium mortiferum |
| Fusobacterium necrophorum | 0 | 1 | 0 | 0 | 0 | 0 | 0 | 0 | 0 | 0 | 0 | 0 | 0 | 0 | Bacteria | Bacteria\_none | Fusobacteriota | Fusobacteriia | Fusobacteriales | Fusobacteriaceae | Fusobacterium | Bacteria;Bacteria\_none;Fusobacteriota;Fusobacteriia;Fusobacteriales;Fusobacteriaceae;Fusobacterium;Fusobacterium necrophorum |
| Fusobacterium nucleatum | 0 | 18 | 7 | 19 | 3 | 3 | 1 | 10 | 0 | 2 | 11 | 7 | 7 | 7 | Bacteria | Bacteria\_none | Fusobacteriota | Fusobacteriia | Fusobacteriales | Fusobacteriaceae | Fusobacterium | Bacteria;Bacteria\_none;Fusobacteriota;Fusobacteriia;Fusobacteriales;Fusobacteriaceae;Fusobacterium;Fusobacterium nucleatum |
| Fusobacterium periodonticum | 0 | 5 | 0 | 4 | 0 | 0 | 0 | 0 | 0 | 1 | 0 | 0 | 3 | 0 | Bacteria | Bacteria\_none | Fusobacteriota | Fusobacteriia | Fusobacteriales | Fusobacteriaceae | Fusobacterium | Bacteria;Bacteria\_none;Fusobacteriota;Fusobacteriia;Fusobacteriales;Fusobacteriaceae;Fusobacterium;Fusobacterium periodonticum |
| Fusobacterium polymorphum | 1 | 59 | 17 | 81 | 5 | 3 | 2 | 21 | 0 | 7 | 16 | 15 | 17 | 22 | Bacteria | Bacteria\_none | Fusobacteriota | Fusobacteriia | Fusobacteriales | Fusobacteriaceae | Fusobacterium | Bacteria;Bacteria\_none;Fusobacteriota;Fusobacteriia;Fusobacteriales;Fusobacteriaceae;Fusobacterium;Fusobacterium polymorphum |
| Fusobacterium pseudoperiodonticum | 0 | 58 | 22 | 265 | 22 | 11 | 0 | 1 | 0 | 10 | 0 | 4 | 27 | 20 | Bacteria | Bacteria\_none | Fusobacteriota | Fusobacteriia | Fusobacteriales | Fusobacteriaceae | Fusobacterium | Bacteria;Bacteria\_none;Fusobacteriota;Fusobacteriia;Fusobacteriales;Fusobacteriaceae;Fusobacterium;Fusobacterium pseudoperiodonticum |
| Fusobacterium sp. oral taxon 203 | 0 | 0 | 0 | 0 | 0 | 0 | 2 | 0 | 0 | 0 | 1 | 0 | 0 | 2 | Bacteria | Bacteria\_none | Fusobacteriota | Fusobacteriia | Fusobacteriales | Fusobacteriaceae | Fusobacterium | Bacteria;Bacteria\_none;Fusobacteriota;Fusobacteriia;Fusobacteriales;Fusobacteriaceae;Fusobacterium;Fusobacterium sp. oral taxon 203 |
| Fusobacterium varium | 0 | 0 | 0 | 0 | 0 | 1 | 0 | 0 | 0 | 0 | 0 | 0 | 0 | 0 | Bacteria | Bacteria\_none | Fusobacteriota | Fusobacteriia | Fusobacteriales | Fusobacteriaceae | Fusobacterium | Bacteria;Bacteria\_none;Fusobacteriota;Fusobacteriia;Fusobacteriales;Fusobacteriaceae;Fusobacterium;Fusobacterium varium |
| Fusobacterium vincentii | 0 | 13 | 0 | 7 | 4 | 2 | 0 | 0 | 0 | 0 | 25 | 6 | 4 | 6 | Bacteria | Bacteria\_none | Fusobacteriota | Fusobacteriia | Fusobacteriales | Fusobacteriaceae | Fusobacterium | Bacteria;Bacteria\_none;Fusobacteriota;Fusobacteriia;Fusobacteriales;Fusobacteriaceae;Fusobacterium;Fusobacterium vincentii |
| Ilyobacter\_uncultured | 0 | 0 | 0 | 0 | 0 | 0 | 0 | 0 | 0 | 0 | 0 | 0 | 0 | 0 | Bacteria | Bacteria\_none | Fusobacteriota | Fusobacteriia | Fusobacteriales | Fusobacteriaceae | Ilyobacter | Bacteria;Bacteria\_none;Fusobacteriota;Fusobacteriia;Fusobacteriales;Fusobacteriaceae;Ilyobacter;Ilyobacter\_uncultured |
| Haliovirga abyssi | 0 | 0 | 0 | 0 | 0 | 0 | 0 | 0 | 0 | 0 | 0 | 0 | 0 | 0 | Bacteria | Bacteria\_none | Fusobacteriota | Fusobacteriia | Fusobacteriales | Haliovirgaceae | Haliovirga | Bacteria;Bacteria\_none;Fusobacteriota;Fusobacteriia;Fusobacteriales;Haliovirgaceae;Haliovirga;Haliovirga abyssi |
| Leptotrichia buccalis | 0 | 10 | 3 | 1 | 4 | 0 | 1 | 0 | 0 | 1 | 1 | 0 | 1 | 3 | Bacteria | Bacteria\_none | Fusobacteriota | Fusobacteriia | Fusobacteriales | Leptotrichiaceae | Leptotrichia | Bacteria;Bacteria\_none;Fusobacteriota;Fusobacteriia;Fusobacteriales;Leptotrichiaceae;Leptotrichia;Leptotrichia buccalis |
| Leptotrichia hofstadii | 0 | 2 | 4 | 1 | 1 | 3 | 1 | 0 | 0 | 0 | 0 | 0 | 1 | 0 | Bacteria | Bacteria\_none | Fusobacteriota | Fusobacteriia | Fusobacteriales | Leptotrichiaceae | Leptotrichia | Bacteria;Bacteria\_none;Fusobacteriota;Fusobacteriia;Fusobacteriales;Leptotrichiaceae;Leptotrichia;Leptotrichia hofstadii |
| Leptotrichia hongkongensis | 0 | 5 | 6 | 0 | 1 | 0 | 1 | 1 | 0 | 16 | 3 | 1 | 2 | 1 | Bacteria | Bacteria\_none | Fusobacteriota | Fusobacteriia | Fusobacteriales | Leptotrichiaceae | Leptotrichia | Bacteria;Bacteria\_none;Fusobacteriota;Fusobacteriia;Fusobacteriales;Leptotrichiaceae;Leptotrichia;Leptotrichia hongkongensis |
| Leptotrichia shahii | 0 | 3 | 3 | 0 | 0 | 0 | 2 | 0 | 0 | 0 | 0 | 0 | 0 | 2 | Bacteria | Bacteria\_none | Fusobacteriota | Fusobacteriia | Fusobacteriales | Leptotrichiaceae | Leptotrichia | Bacteria;Bacteria\_none;Fusobacteriota;Fusobacteriia;Fusobacteriales;Leptotrichiaceae;Leptotrichia;Leptotrichia shahii |
| Leptotrichia sp. HMT-225 | 0 | 1 | 4 | 10 | 2 | 2 | 0 | 16 | 1 | 1 | 0 | 0 | 3 | 5 | Bacteria | Bacteria\_none | Fusobacteriota | Fusobacteriia | Fusobacteriales | Leptotrichiaceae | Leptotrichia | Bacteria;Bacteria\_none;Fusobacteriota;Fusobacteriia;Fusobacteriales;Leptotrichiaceae;Leptotrichia;Leptotrichia sp. HMT-225 |
| Leptotrichia sp. oral taxon 212 | 4 | 43 | 22 | 22 | 30 | 25 | 0 | 76 | 1 | 10 | 9 | 4 | 32 | 18 | Bacteria | Bacteria\_none | Fusobacteriota | Fusobacteriia | Fusobacteriales | Leptotrichiaceae | Leptotrichia | Bacteria;Bacteria\_none;Fusobacteriota;Fusobacteriia;Fusobacteriales;Leptotrichiaceae;Leptotrichia;Leptotrichia sp. oral taxon 212 |
| Leptotrichia sp. oral taxon 218 | 0 | 0 | 7 | 1 | 0 | 8 | 4 | 1 | 0 | 0 | 0 | 1 | 1 | 0 | Bacteria | Bacteria\_none | Fusobacteriota | Fusobacteriia | Fusobacteriales | Leptotrichiaceae | Leptotrichia | Bacteria;Bacteria\_none;Fusobacteriota;Fusobacteriia;Fusobacteriales;Leptotrichiaceae;Leptotrichia;Leptotrichia sp. oral taxon 218 |
| Leptotrichia sp. oral taxon 221 | 0 | 3 | 4 | 0 | 1 | 8 | 8 | 0 | 0 | 0 | 2 | 0 | 1 | 2 | Bacteria | Bacteria\_none | Fusobacteriota | Fusobacteriia | Fusobacteriales | Leptotrichiaceae | Leptotrichia | Bacteria;Bacteria\_none;Fusobacteriota;Fusobacteriia;Fusobacteriales;Leptotrichiaceae;Leptotrichia;Leptotrichia sp. oral taxon 221 |
| Leptotrichia sp. oral taxon 498 | 0 | 1 | 2 | 0 | 0 | 1 | 7 | 0 | 0 | 2 | 0 | 3 | 0 | 1 | Bacteria | Bacteria\_none | Fusobacteriota | Fusobacteriia | Fusobacteriales | Leptotrichiaceae | Leptotrichia | Bacteria;Bacteria\_none;Fusobacteriota;Fusobacteriia;Fusobacteriales;Leptotrichiaceae;Leptotrichia;Leptotrichia sp. oral taxon 498 |
| Leptotrichia sp. oral taxon 847 | 1 | 0 | 4 | 0 | 1 | 2 | 1 | 0 | 0 | 0 | 0 | 0 | 1 | 0 | Bacteria | Bacteria\_none | Fusobacteriota | Fusobacteriia | Fusobacteriales | Leptotrichiaceae | Leptotrichia | Bacteria;Bacteria\_none;Fusobacteriota;Fusobacteriia;Fusobacteriales;Leptotrichiaceae;Leptotrichia;Leptotrichia sp. oral taxon 847 |
| Leptotrichia trevisanii | 0 | 5 | 3 | 3 | 1 | 1 | 1 | 1 | 8 | 2 | 25 | 0 | 5 | 2 | Bacteria | Bacteria\_none | Fusobacteriota | Fusobacteriia | Fusobacteriales | Leptotrichiaceae | Leptotrichia | Bacteria;Bacteria\_none;Fusobacteriota;Fusobacteriia;Fusobacteriales;Leptotrichiaceae;Leptotrichia;Leptotrichia trevisanii |
| Leptotrichia wadei | 8 | 20 | 98 | 2 | 3 | 119 | 125 | 1 | 1 | 18 | 1 | 5 | 18 | 24 | Bacteria | Bacteria\_none | Fusobacteriota | Fusobacteriia | Fusobacteriales | Leptotrichiaceae | Leptotrichia | Bacteria;Bacteria\_none;Fusobacteriota;Fusobacteriia;Fusobacteriales;Leptotrichiaceae;Leptotrichia;Leptotrichia wadei |
| Pseudoleptotrichia goodfellowii | 0 | 0 | 0 | 10 | 27 | 0 | 0 | 0 | 0 | 1 | 29 | 0 | 4 | 3 | Bacteria | Bacteria\_none | Fusobacteriota | Fusobacteriia | Fusobacteriales | Leptotrichiaceae | Pseudoleptotrichia | Bacteria;Bacteria\_none;Fusobacteriota;Fusobacteriia;Fusobacteriales;Leptotrichiaceae;Pseudoleptotrichia;Pseudoleptotrichia goodfellowii |
| Sebaldella termitidis | 0 | 0 | 0 | 0 | 0 | 0 | 0 | 0 | 0 | 0 | 0 | 0 | 0 | 0 | Bacteria | Bacteria\_none | Fusobacteriota | Fusobacteriia | Fusobacteriales | Leptotrichiaceae | Sebaldella | Bacteria;Bacteria\_none;Fusobacteriota;Fusobacteriia;Fusobacteriales;Leptotrichiaceae;Sebaldella;Sebaldella termitidis |
| Sneathia vaginalis | 0 | 1 | 0 | 0 | 0 | 0 | 0 | 0 | 0 | 0 | 0 | 0 | 0 | 0 | Bacteria | Bacteria\_none | Fusobacteriota | Fusobacteriia | Fusobacteriales | Leptotrichiaceae | Sneathia | Bacteria;Bacteria\_none;Fusobacteriota;Fusobacteriia;Fusobacteriales;Leptotrichiaceae;Sneathia;Sneathia vaginalis |
| Streptobacillus moniliformis | 0 | 0 | 1 | 3 | 0 | 0 | 0 | 0 | 0 | 0 | 0 | 0 | 0 | 0 | Bacteria | Bacteria\_none | Fusobacteriota | Fusobacteriia | Fusobacteriales | Leptotrichiaceae | Streptobacillus | Bacteria;Bacteria\_none;Fusobacteriota;Fusobacteriia;Fusobacteriales;Leptotrichiaceae;Streptobacillus;Streptobacillus moniliformis |
| Ignavibacterium sp. | 0 | 0 | 0 | 0 | 0 | 0 | 0 | 0 | 0 | 0 | 0 | 0 | 0 | 0 | Bacteria | Bacteria\_none | Ignavibacteriota | Ignavibacteria | Ignavibacteriales | Ignavibacteriaceae | Ignavibacterium | Bacteria;Bacteria\_none;Ignavibacteriota;Ignavibacteria;Ignavibacteriales;Ignavibacteriaceae;Ignavibacterium;Ignavibacterium sp. |
| Acholeplasma hippikon | 0 | 0 | 0 | 0 | 1 | 0 | 0 | 0 | 0 | 0 | 0 | 0 | 1 | 0 | Bacteria | Bacteria\_none | Mycoplasmatota | Mollicutes | Acholeplasmatales | Acholeplasmataceae | Acholeplasma | Bacteria;Bacteria\_none;Mycoplasmatota;Mollicutes;Acholeplasmatales;Acholeplasmataceae;Acholeplasma;Acholeplasma hippikon |
| Mariniplasma anaerobium | 0 | 0 | 0 | 0 | 0 | 0 | 0 | 0 | 0 | 0 | 0 | 0 | 0 | 0 | Bacteria | Bacteria\_none | Mycoplasmatota | Mollicutes | Acholeplasmatales | Acholeplasmataceae | Mariniplasma | Bacteria;Bacteria\_none;Mycoplasmatota;Mollicutes;Acholeplasmatales;Acholeplasmataceae;Mariniplasma;Mariniplasma anaerobium |
| Spiroplasma culicicola | 0 | 0 | 0 | 0 | 1 | 0 | 0 | 0 | 0 | 0 | 0 | 0 | 0 | 0 | Bacteria | Bacteria\_none | Mycoplasmatota | Mollicutes | Entomoplasmatales | Spiroplasmataceae | Spiroplasma | Bacteria;Bacteria\_none;Mycoplasmatota;Mollicutes;Entomoplasmatales;Spiroplasmataceae;Spiroplasma;Spiroplasma culicicola |
| Spiroplasma endosymbiont of 'Nebria riversi' | 0 | 0 | 0 | 0 | 0 | 0 | 0 | 0 | 0 | 0 | 0 | 0 | 0 | 0 | Bacteria | Bacteria\_none | Mycoplasmatota | Mollicutes | Entomoplasmatales | Spiroplasmataceae | Spiroplasma | Bacteria;Bacteria\_none;Mycoplasmatota;Mollicutes;Entomoplasmatales;Spiroplasmataceae;Spiroplasma;Spiroplasma endosymbiont of 'Nebria riversi' |
| Spiroplasma endosymbiont of Agriotes lineatus | 0 | 0 | 0 | 0 | 0 | 0 | 0 | 0 | 0 | 0 | 0 | 0 | 0 | 0 | Bacteria | Bacteria\_none | Mycoplasmatota | Mollicutes | Entomoplasmatales | Spiroplasmataceae | Spiroplasma | Bacteria;Bacteria\_none;Mycoplasmatota;Mollicutes;Entomoplasmatales;Spiroplasmataceae;Spiroplasma;Spiroplasma endosymbiont of Agriotes lineatus |
| Spiroplasma endosymbiont of Cantharis nigra | 0 | 0 | 0 | 0 | 0 | 1 | 0 | 0 | 0 | 0 | 0 | 0 | 0 | 0 | Bacteria | Bacteria\_none | Mycoplasmatota | Mollicutes | Entomoplasmatales | Spiroplasmataceae | Spiroplasma | Bacteria;Bacteria\_none;Mycoplasmatota;Mollicutes;Entomoplasmatales;Spiroplasmataceae;Spiroplasma;Spiroplasma endosymbiont of Cantharis nigra |
| Spiroplasma endosymbiont of Dromius quadrimaculatus | 0 | 0 | 0 | 0 | 0 | 0 | 0 | 0 | 0 | 0 | 0 | 0 | 0 | 0 | Bacteria | Bacteria\_none | Mycoplasmatota | Mollicutes | Entomoplasmatales | Spiroplasmataceae | Spiroplasma | Bacteria;Bacteria\_none;Mycoplasmatota;Mollicutes;Entomoplasmatales;Spiroplasmataceae;Spiroplasma;Spiroplasma endosymbiont of Dromius quadrimaculatus |
| Spiroplasma gladiatoris | 0 | 0 | 0 | 0 | 0 | 0 | 0 | 0 | 0 | 0 | 0 | 0 | 0 | 0 | Bacteria | Bacteria\_none | Mycoplasmatota | Mollicutes | Entomoplasmatales | Spiroplasmataceae | Spiroplasma | Bacteria;Bacteria\_none;Mycoplasmatota;Mollicutes;Entomoplasmatales;Spiroplasmataceae;Spiroplasma;Spiroplasma gladiatoris |
| Spiroplasma monobiae | 0 | 0 | 0 | 1 | 0 | 0 | 0 | 0 | 0 | 0 | 0 | 0 | 0 | 0 | Bacteria | Bacteria\_none | Mycoplasmatota | Mollicutes | Entomoplasmatales | Spiroplasmataceae | Spiroplasma | Bacteria;Bacteria\_none;Mycoplasmatota;Mollicutes;Entomoplasmatales;Spiroplasmataceae;Spiroplasma;Spiroplasma monobiae |
| Spiroplasma syrphidicola | 0 | 0 | 0 | 0 | 0 | 0 | 0 | 0 | 0 | 0 | 0 | 0 | 0 | 0 | Bacteria | Bacteria\_none | Mycoplasmatota | Mollicutes | Entomoplasmatales | Spiroplasmataceae | Spiroplasma | Bacteria;Bacteria\_none;Mycoplasmatota;Mollicutes;Entomoplasmatales;Spiroplasmataceae;Spiroplasma;Spiroplasma syrphidicola |
| Spiroplasma tabanidicola | 0 | 0 | 0 | 0 | 0 | 0 | 0 | 0 | 0 | 0 | 0 | 0 | 0 | 0 | Bacteria | Bacteria\_none | Mycoplasmatota | Mollicutes | Entomoplasmatales | Spiroplasmataceae | Spiroplasma | Bacteria;Bacteria\_none;Mycoplasmatota;Mollicutes;Entomoplasmatales;Spiroplasmataceae;Spiroplasma;Spiroplasma tabanidicola |
| Mycoplasma anserisalpingitidis | 0 | 0 | 0 | 0 | 0 | 0 | 0 | 0 | 0 | 0 | 0 | 0 | 0 | 1 | Bacteria | Bacteria\_none | Mycoplasmatota | Mollicutes | Mycoplasmatales | Mycoplasmataceae | Mycoplasma | Bacteria;Bacteria\_none;Mycoplasmatota;Mollicutes;Mycoplasmatales;Mycoplasmataceae;Mycoplasma;Mycoplasma anserisalpingitidis |
| Mycoplasma miroungirhinis | 0 | 0 | 2 | 0 | 0 | 1 | 0 | 0 | 0 | 0 | 0 | 0 | 0 | 0 | Bacteria | Bacteria\_none | Mycoplasmatota | Mollicutes | Mycoplasmatales | Mycoplasmataceae | Mycoplasma | Bacteria;Bacteria\_none;Mycoplasmatota;Mollicutes;Mycoplasmatales;Mycoplasmataceae;Mycoplasma;Mycoplasma miroungirhinis |
| Mycoplasma seminis | 0 | 1 | 0 | 0 | 0 | 0 | 0 | 0 | 0 | 0 | 0 | 0 | 0 | 0 | Bacteria | Bacteria\_none | Mycoplasmatota | Mollicutes | Mycoplasmatales | Mycoplasmataceae | Mycoplasma | Bacteria;Bacteria\_none;Mycoplasmatota;Mollicutes;Mycoplasmatales;Mycoplasmataceae;Mycoplasma;Mycoplasma seminis |
| Mycoplasma struthionis | 0 | 0 | 1 | 0 | 0 | 0 | 0 | 0 | 0 | 0 | 0 | 0 | 0 | 0 | Bacteria | Bacteria\_none | Mycoplasmatota | Mollicutes | Mycoplasmatales | Mycoplasmataceae | Mycoplasma | Bacteria;Bacteria\_none;Mycoplasmatota;Mollicutes;Mycoplasmatales;Mycoplasmataceae;Mycoplasma;Mycoplasma struthionis |
| endosymbiont DhMRE of Dentiscutata heterogama | 0 | 0 | 0 | 0 | 0 | 0 | 0 | 0 | 0 | 0 | 1 | 0 | 0 | 0 | Bacteria | Bacteria\_none | Mycoplasmatota | Mollicutes | Mycoplasmatales | Mycoplasmatales\_Incertae\_sedis | Mycoplasmatales\_Incertae\_sedis | Bacteria;Bacteria\_none;Mycoplasmatota;Mollicutes;Mycoplasmatales;Mycoplasmatales\_Incertae\_sedis;Mycoplasmatales\_Incertae\_sedis;endosymbiont DhMRE of Dentiscutata heterogama |
| Metamycoplasma alkalescens | 0 | 0 | 0 | 0 | 0 | 0 | 0 | 0 | 0 | 0 | 0 | 0 | 0 | 0 | Bacteria | Bacteria\_none | Mycoplasmatota | Mycoplasmatota\_Incertae\_sedis | Mycoplasmoidales | Metamycoplasmataceae | Metamycoplasma | Bacteria;Bacteria\_none;Mycoplasmatota;Mycoplasmatota\_Incertae\_sedis;Mycoplasmoidales;Metamycoplasmataceae;Metamycoplasma;Metamycoplasma alkalescens |
| Metamycoplasma arthritidis | 0 | 0 | 1 | 0 | 0 | 0 | 0 | 0 | 0 | 0 | 0 | 0 | 0 | 0 | Bacteria | Bacteria\_none | Mycoplasmatota | Mycoplasmatota\_Incertae\_sedis | Mycoplasmoidales | Metamycoplasmataceae | Metamycoplasma | Bacteria;Bacteria\_none;Mycoplasmatota;Mycoplasmatota\_Incertae\_sedis;Mycoplasmoidales;Metamycoplasmataceae;Metamycoplasma;Metamycoplasma arthritidis |
| Metamycoplasma cloacale | 0 | 0 | 0 | 0 | 0 | 0 | 0 | 0 | 0 | 0 | 0 | 0 | 0 | 0 | Bacteria | Bacteria\_none | Mycoplasmatota | Mycoplasmatota\_Incertae\_sedis | Mycoplasmoidales | Metamycoplasmataceae | Metamycoplasma | Bacteria;Bacteria\_none;Mycoplasmatota;Mycoplasmatota\_Incertae\_sedis;Mycoplasmoidales;Metamycoplasmataceae;Metamycoplasma;Metamycoplasma cloacale |
| Metamycoplasma equirhinis | 0 | 0 | 1 | 0 | 0 | 0 | 0 | 0 | 0 | 0 | 0 | 1 | 0 | 0 | Bacteria | Bacteria\_none | Mycoplasmatota | Mycoplasmatota\_Incertae\_sedis | Mycoplasmoidales | Metamycoplasmataceae | Metamycoplasma | Bacteria;Bacteria\_none;Mycoplasmatota;Mycoplasmatota\_Incertae\_sedis;Mycoplasmoidales;Metamycoplasmataceae;Metamycoplasma;Metamycoplasma equirhinis |
| Metamycoplasma gateae | 0 | 3 | 0 | 0 | 0 | 0 | 0 | 0 | 0 | 0 | 0 | 1 | 1 | 0 | Bacteria | Bacteria\_none | Mycoplasmatota | Mycoplasmatota\_Incertae\_sedis | Mycoplasmoidales | Metamycoplasmataceae | Metamycoplasma | Bacteria;Bacteria\_none;Mycoplasmatota;Mycoplasmatota\_Incertae\_sedis;Mycoplasmoidales;Metamycoplasmataceae;Metamycoplasma;Metamycoplasma gateae |
| Metamycoplasma hominis | 0 | 1 | 1 | 0 | 0 | 0 | 0 | 0 | 0 | 0 | 0 | 0 | 0 | 0 | Bacteria | Bacteria\_none | Mycoplasmatota | Mycoplasmatota\_Incertae\_sedis | Mycoplasmoidales | Metamycoplasmataceae | Metamycoplasma | Bacteria;Bacteria\_none;Mycoplasmatota;Mycoplasmatota\_Incertae\_sedis;Mycoplasmoidales;Metamycoplasmataceae;Metamycoplasma;Metamycoplasma hominis |
| Metamycoplasma hyosynoviae | 0 | 0 | 2 | 0 | 0 | 0 | 0 | 0 | 0 | 0 | 0 | 0 | 0 | 0 | Bacteria | Bacteria\_none | Mycoplasmatota | Mycoplasmatota\_Incertae\_sedis | Mycoplasmoidales | Metamycoplasmataceae | Metamycoplasma | Bacteria;Bacteria\_none;Mycoplasmatota;Mycoplasmatota\_Incertae\_sedis;Mycoplasmoidales;Metamycoplasmataceae;Metamycoplasma;Metamycoplasma hyosynoviae |
| Metamycoplasma orale | 0 | 1 | 0 | 0 | 1 | 1 | 0 | 0 | 0 | 0 | 0 | 0 | 0 | 0 | Bacteria | Bacteria\_none | Mycoplasmatota | Mycoplasmatota\_Incertae\_sedis | Mycoplasmoidales | Metamycoplasmataceae | Metamycoplasma | Bacteria;Bacteria\_none;Mycoplasmatota;Mycoplasmatota\_Incertae\_sedis;Mycoplasmoidales;Metamycoplasmataceae;Metamycoplasma;Metamycoplasma orale |
| Metamycoplasma phocicerebrale | 0 | 0 | 0 | 0 | 0 | 1 | 0 | 0 | 0 | 0 | 0 | 0 | 0 | 0 | Bacteria | Bacteria\_none | Mycoplasmatota | Mycoplasmatota\_Incertae\_sedis | Mycoplasmoidales | Metamycoplasmataceae | Metamycoplasma | Bacteria;Bacteria\_none;Mycoplasmatota;Mycoplasmatota\_Incertae\_sedis;Mycoplasmoidales;Metamycoplasmataceae;Metamycoplasma;Metamycoplasma phocicerebrale |
| Metamycoplasma salivarium | 0 | 3 | 24 | 1 | 1 | 3 | 0 | 1 | 0 | 6 | 2 | 0 | 0 | 0 | Bacteria | Bacteria\_none | Mycoplasmatota | Mycoplasmatota\_Incertae\_sedis | Mycoplasmoidales | Metamycoplasmataceae | Metamycoplasma | Bacteria;Bacteria\_none;Mycoplasmatota;Mycoplasmatota\_Incertae\_sedis;Mycoplasmoidales;Metamycoplasmataceae;Metamycoplasma;Metamycoplasma salivarium |
| Metamycoplasma subdolum | 0 | 0 | 0 | 0 | 0 | 0 | 0 | 0 | 0 | 0 | 0 | 1 | 0 | 1 | Bacteria | Bacteria\_none | Mycoplasmatota | Mycoplasmatota\_Incertae\_sedis | Mycoplasmoidales | Metamycoplasmataceae | Metamycoplasma | Bacteria;Bacteria\_none;Mycoplasmatota;Mycoplasmatota\_Incertae\_sedis;Mycoplasmoidales;Metamycoplasmataceae;Metamycoplasma;Metamycoplasma subdolum |
| [Mycoplasma] phocae | 0 | 0 | 1 | 0 | 0 | 0 | 0 | 0 | 0 | 0 | 0 | 0 | 1 | 0 | Bacteria | Bacteria\_none | Mycoplasmatota | Mycoplasmatota\_Incertae\_sedis | Mycoplasmoidales | Metamycoplasmataceae | Metamycoplasma | Bacteria;Bacteria\_none;Mycoplasmatota;Mycoplasmatota\_Incertae\_sedis;Mycoplasmoidales;Metamycoplasmataceae;Metamycoplasma;[Mycoplasma] phocae |
| Mycoplasmopsis anatis | 0 | 0 | 0 | 0 | 0 | 0 | 0 | 0 | 0 | 0 | 0 | 0 | 0 | 0 | Bacteria | Bacteria\_none | Mycoplasmatota | Mycoplasmatota\_Incertae\_sedis | Mycoplasmoidales | Metamycoplasmataceae | Mycoplasmopsis | Bacteria;Bacteria\_none;Mycoplasmatota;Mycoplasmatota\_Incertae\_sedis;Mycoplasmoidales;Metamycoplasmataceae;Mycoplasmopsis;Mycoplasmopsis anatis |
| Mycoplasmopsis citelli | 0 | 0 | 1 | 0 | 0 | 0 | 0 | 0 | 0 | 0 | 0 | 0 | 0 | 0 | Bacteria | Bacteria\_none | Mycoplasmatota | Mycoplasmatota\_Incertae\_sedis | Mycoplasmoidales | Metamycoplasmataceae | Mycoplasmopsis | Bacteria;Bacteria\_none;Mycoplasmatota;Mycoplasmatota\_Incertae\_sedis;Mycoplasmoidales;Metamycoplasmataceae;Mycoplasmopsis;Mycoplasmopsis citelli |
| Mycoplasmopsis columbina | 0 | 0 | 1 | 0 | 0 | 0 | 0 | 0 | 0 | 0 | 0 | 0 | 0 | 0 | Bacteria | Bacteria\_none | Mycoplasmatota | Mycoplasmatota\_Incertae\_sedis | Mycoplasmoidales | Metamycoplasmataceae | Mycoplasmopsis | Bacteria;Bacteria\_none;Mycoplasmatota;Mycoplasmatota\_Incertae\_sedis;Mycoplasmoidales;Metamycoplasmataceae;Mycoplasmopsis;Mycoplasmopsis columbina |
| Mycoplasmopsis cynos | 0 | 1 | 0 | 0 | 0 | 0 | 0 | 0 | 0 | 0 | 0 | 0 | 0 | 0 | Bacteria | Bacteria\_none | Mycoplasmatota | Mycoplasmatota\_Incertae\_sedis | Mycoplasmoidales | Metamycoplasmataceae | Mycoplasmopsis | Bacteria;Bacteria\_none;Mycoplasmatota;Mycoplasmatota\_Incertae\_sedis;Mycoplasmoidales;Metamycoplasmataceae;Mycoplasmopsis;Mycoplasmopsis cynos |
| Mycoplasmopsis fermentans | 0 | 1 | 0 | 0 | 0 | 0 | 0 | 0 | 0 | 0 | 0 | 0 | 0 | 0 | Bacteria | Bacteria\_none | Mycoplasmatota | Mycoplasmatota\_Incertae\_sedis | Mycoplasmoidales | Metamycoplasmataceae | Mycoplasmopsis | Bacteria;Bacteria\_none;Mycoplasmatota;Mycoplasmatota\_Incertae\_sedis;Mycoplasmoidales;Metamycoplasmataceae;Mycoplasmopsis;Mycoplasmopsis fermentans |
| Mycoplasmopsis pulmonis | 0 | 0 | 0 | 0 | 0 | 0 | 0 | 0 | 0 | 0 | 0 | 1 | 0 | 0 | Bacteria | Bacteria\_none | Mycoplasmatota | Mycoplasmatota\_Incertae\_sedis | Mycoplasmoidales | Metamycoplasmataceae | Mycoplasmopsis | Bacteria;Bacteria\_none;Mycoplasmatota;Mycoplasmatota\_Incertae\_sedis;Mycoplasmoidales;Metamycoplasmataceae;Mycoplasmopsis;Mycoplasmopsis pulmonis |
| Malacoplasma iowae | 0 | 0 | 0 | 0 | 0 | 0 | 0 | 0 | 0 | 0 | 0 | 0 | 0 | 0 | Bacteria | Bacteria\_none | Mycoplasmatota | Mycoplasmatota\_Incertae\_sedis | Mycoplasmoidales | Mycoplasmoidaceae | Malacoplasma | Bacteria;Bacteria\_none;Mycoplasmatota;Mycoplasmatota\_Incertae\_sedis;Mycoplasmoidales;Mycoplasmoidaceae;Malacoplasma;Malacoplasma iowae |
| Mycoplasmoides gallisepticum | 0 | 0 | 0 | 0 | 0 | 0 | 0 | 0 | 0 | 0 | 0 | 0 | 0 | 0 | Bacteria | Bacteria\_none | Mycoplasmatota | Mycoplasmatota\_Incertae\_sedis | Mycoplasmoidales | Mycoplasmoidaceae | Mycoplasmoides | Bacteria;Bacteria\_none;Mycoplasmatota;Mycoplasmatota\_Incertae\_sedis;Mycoplasmoidales;Mycoplasmoidaceae;Mycoplasmoides;Mycoplasmoides gallisepticum |
| Corallococcus coralloides | 0 | 0 | 0 | 0 | 0 | 0 | 0 | 0 | 0 | 0 | 0 | 0 | 0 | 0 | Bacteria | Bacteria\_none | Myxococcota | Myxococcia | Myxococcales | Myxococcaceae | Corallococcus | Bacteria;Bacteria\_none;Myxococcota;Myxococcia;Myxococcales;Myxococcaceae;Corallococcus;Corallococcus coralloides |
| Sorangium cellulosum | 0 | 0 | 0 | 0 | 1 | 0 | 0 | 0 | 0 | 0 | 1 | 0 | 0 | 0 | Bacteria | Bacteria\_none | Myxococcota | Myxococcota\_Incertae\_sedis | Polyangiales | Polyangiaceae | Sorangium | Bacteria;Bacteria\_none;Myxococcota;Myxococcota\_Incertae\_sedis;Polyangiales;Polyangiaceae;Sorangium;Sorangium cellulosum |
| Candidatus Nitrospira neomarina | 0 | 0 | 0 | 0 | 0 | 1 | 0 | 0 | 0 | 0 | 0 | 0 | 0 | 0 | Bacteria | Bacteria\_none | Nitrospirota | Nitrospiria | Nitrospirales | Nitrospiraceae | Nitrospira | Bacteria;Bacteria\_none;Nitrospirota;Nitrospiria;Nitrospirales;Nitrospiraceae;Nitrospira;Candidatus Nitrospira neomarina |
| Dissulfurispira thermophila | 0 | 0 | 0 | 0 | 0 | 0 | 0 | 0 | 0 | 0 | 1 | 0 | 0 | 0 | Bacteria | Bacteria\_none | Nitrospirota | Thermodesulfovibrionia | Thermodesulfovibrionales | Dissulfurispiraceae | Dissulfurispira | Bacteria;Bacteria\_none;Nitrospirota;Thermodesulfovibrionia;Thermodesulfovibrionales;Dissulfurispiraceae;Dissulfurispira;Dissulfurispira thermophila |
| Tuwongella immobilis | 0 | 0 | 0 | 0 | 0 | 1 | 0 | 0 | 0 | 0 | 0 | 0 | 0 | 0 | Bacteria | Bacteria\_none | Planctomycetota | Planctomycetia | Gemmatales | Gemmataceae | Tuwongella | Bacteria;Bacteria\_none;Planctomycetota;Planctomycetia;Gemmatales;Gemmataceae;Tuwongella;Tuwongella immobilis |
| Lignipirellula cremea | 1 | 0 | 0 | 0 | 0 | 0 | 0 | 0 | 0 | 0 | 0 | 0 | 0 | 0 | Bacteria | Bacteria\_none | Planctomycetota | Planctomycetia | Pirellulales | Pirellulaceae | Lignipirellula | Bacteria;Bacteria\_none;Planctomycetota;Planctomycetia;Pirellulales;Pirellulaceae;Lignipirellula;Lignipirellula cremea |
| Fuerstiella marisgermanici | 0 | 0 | 0 | 0 | 0 | 0 | 0 | 0 | 0 | 0 | 0 | 0 | 0 | 0 | Bacteria | Bacteria\_none | Planctomycetota | Planctomycetia | Planctomycetales | Planctomycetaceae | Fuerstiella | Bacteria;Bacteria\_none;Planctomycetota;Planctomycetia;Planctomycetales;Planctomycetaceae;Fuerstiella;Fuerstiella marisgermanici |
| Gimesia panareensis | 0 | 0 | 0 | 0 | 0 | 0 | 0 | 0 | 0 | 0 | 0 | 0 | 0 | 0 | Bacteria | Bacteria\_none | Planctomycetota | Planctomycetia | Planctomycetales | Planctomycetaceae | Gimesia | Bacteria;Bacteria\_none;Planctomycetota;Planctomycetia;Planctomycetales;Planctomycetaceae;Gimesia;Gimesia panareensis |
| Candidatus Pelagibacter sp. RS40 | 0 | 0 | 0 | 0 | 0 | 0 | 0 | 0 | 0 | 0 | 0 | 0 | 0 | 0 | Bacteria | Bacteria\_none | Pseudomonadota | Alphaproteobacteria | Candidatus Pelagibacterales | Candidatus Pelagibacteraceae | Candidatus Pelagibacter | Bacteria;Bacteria\_none;Pseudomonadota;Alphaproteobacteria;Candidatus Pelagibacterales;Candidatus Pelagibacteraceae;Candidatus Pelagibacter;Candidatus Pelagibacter sp. RS40 |
| Asticcacaulis sp. DW145 | 0 | 0 | 0 | 0 | 0 | 0 | 0 | 0 | 0 | 0 | 0 | 0 | 0 | 0 | Bacteria | Bacteria\_none | Pseudomonadota | Alphaproteobacteria | Caulobacterales | Caulobacteraceae | Asticcacaulis | Bacteria;Bacteria\_none;Pseudomonadota;Alphaproteobacteria;Caulobacterales;Caulobacteraceae;Asticcacaulis;Asticcacaulis sp. DW145 |
| Bosea sp. AS-1 | 0 | 0 | 0 | 0 | 0 | 0 | 0 | 0 | 0 | 0 | 0 | 0 | 0 | 0 | Bacteria | Bacteria\_none | Pseudomonadota | Alphaproteobacteria | Hyphomicrobiales | Boseaceae | Bosea | Bacteria;Bacteria\_none;Pseudomonadota;Alphaproteobacteria;Hyphomicrobiales;Boseaceae;Bosea;Bosea sp. AS-1 |
| Brucella sp. NM4 | 0 | 0 | 0 | 0 | 0 | 0 | 0 | 0 | 0 | 0 | 0 | 0 | 0 | 0 | Bacteria | Bacteria\_none | Pseudomonadota | Alphaproteobacteria | Hyphomicrobiales | Brucellaceae | Brucella | Bacteria;Bacteria\_none;Pseudomonadota;Alphaproteobacteria;Hyphomicrobiales;Brucellaceae;Brucella;Brucella sp. NM4 |
| Cohaesibacter\_uncultured | 0 | 0 | 0 | 0 | 0 | 0 | 0 | 0 | 1 | 0 | 0 | 0 | 0 | 0 | Bacteria | Bacteria\_none | Pseudomonadota | Alphaproteobacteria | Hyphomicrobiales | Cohaesibacteraceae | Cohaesibacter | Bacteria;Bacteria\_none;Pseudomonadota;Alphaproteobacteria;Hyphomicrobiales;Cohaesibacteraceae;Cohaesibacter;Cohaesibacter\_uncultured |
| Devosia oryziradicis | 0 | 0 | 0 | 0 | 0 | 0 | 1 | 0 | 0 | 0 | 0 | 0 | 0 | 0 | Bacteria | Bacteria\_none | Pseudomonadota | Alphaproteobacteria | Hyphomicrobiales | Devosiaceae | Devosia | Bacteria;Bacteria\_none;Pseudomonadota;Alphaproteobacteria;Hyphomicrobiales;Devosiaceae;Devosia;Devosia oryziradicis |
| Pelagibacterium flavum | 0 | 0 | 1 | 0 | 0 | 0 | 0 | 0 | 0 | 0 | 0 | 0 | 0 | 0 | Bacteria | Bacteria\_none | Pseudomonadota | Alphaproteobacteria | Hyphomicrobiales | Devosiaceae | Pelagibacterium | Bacteria;Bacteria\_none;Pseudomonadota;Alphaproteobacteria;Hyphomicrobiales;Devosiaceae;Pelagibacterium;Pelagibacterium flavum |
| Methylobacterium durans | 0 | 0 | 1 | 0 | 0 | 0 | 0 | 0 | 0 | 0 | 0 | 0 | 0 | 0 | Bacteria | Bacteria\_none | Pseudomonadota | Alphaproteobacteria | Hyphomicrobiales | Methylobacteriaceae | Methylobacterium | Bacteria;Bacteria\_none;Pseudomonadota;Alphaproteobacteria;Hyphomicrobiales;Methylobacteriaceae;Methylobacterium;Methylobacterium durans |
| Microvirga sp. VF16 | 0 | 0 | 0 | 0 | 0 | 0 | 0 | 0 | 0 | 0 | 0 | 0 | 0 | 0 | Bacteria | Bacteria\_none | Pseudomonadota | Alphaproteobacteria | Hyphomicrobiales | Methylobacteriaceae | Microvirga | Bacteria;Bacteria\_none;Pseudomonadota;Alphaproteobacteria;Hyphomicrobiales;Methylobacteriaceae;Microvirga;Microvirga sp. VF16 |
| [Pseudomonas] carboxydohydrogena | 0 | 1 | 0 | 0 | 0 | 0 | 0 | 0 | 0 | 0 | 0 | 0 | 0 | 0 | Bacteria | Bacteria\_none | Pseudomonadota | Alphaproteobacteria | Hyphomicrobiales | Nitrobacteraceae | Afipia | Bacteria;Bacteria\_none;Pseudomonadota;Alphaproteobacteria;Hyphomicrobiales;Nitrobacteraceae;Afipia;[Pseudomonas] carboxydohydrogena |
| Bradyrhizobium sp. | 0 | 0 | 0 | 0 | 0 | 0 | 0 | 0 | 0 | 0 | 1 | 0 | 0 | 0 | Bacteria | Bacteria\_none | Pseudomonadota | Alphaproteobacteria | Hyphomicrobiales | Nitrobacteraceae | Bradyrhizobium | Bacteria;Bacteria\_none;Pseudomonadota;Alphaproteobacteria;Hyphomicrobiales;Nitrobacteraceae;Bradyrhizobium;Bradyrhizobium sp. |
| Bradyrhizobium sp. 170 | 0 | 0 | 0 | 0 | 0 | 1 | 0 | 0 | 0 | 0 | 0 | 0 | 0 | 0 | Bacteria | Bacteria\_none | Pseudomonadota | Alphaproteobacteria | Hyphomicrobiales | Nitrobacteraceae | Bradyrhizobium | Bacteria;Bacteria\_none;Pseudomonadota;Alphaproteobacteria;Hyphomicrobiales;Nitrobacteraceae;Bradyrhizobium;Bradyrhizobium sp. 170 |
| Bradyrhizobium sp. BWA-3-5 | 0 | 0 | 0 | 0 | 0 | 0 | 0 | 0 | 0 | 0 | 1 | 0 | 0 | 0 | Bacteria | Bacteria\_none | Pseudomonadota | Alphaproteobacteria | Hyphomicrobiales | Nitrobacteraceae | Bradyrhizobium | Bacteria;Bacteria\_none;Pseudomonadota;Alphaproteobacteria;Hyphomicrobiales;Nitrobacteraceae;Bradyrhizobium;Bradyrhizobium sp. BWA-3-5 |
| Bradyrhizobium sp. NDS-1 | 0 | 0 | 1 | 0 | 0 | 0 | 0 | 0 | 0 | 0 | 0 | 0 | 0 | 0 | Bacteria | Bacteria\_none | Pseudomonadota | Alphaproteobacteria | Hyphomicrobiales | Nitrobacteraceae | Bradyrhizobium | Bacteria;Bacteria\_none;Pseudomonadota;Alphaproteobacteria;Hyphomicrobiales;Nitrobacteraceae;Bradyrhizobium;Bradyrhizobium sp. NDS-1 |
| Aquibium microcysteis | 0 | 0 | 0 | 0 | 0 | 0 | 0 | 0 | 0 | 0 | 0 | 0 | 0 | 0 | Bacteria | Bacteria\_none | Pseudomonadota | Alphaproteobacteria | Hyphomicrobiales | Phyllobacteriaceae | Aquibium | Bacteria;Bacteria\_none;Pseudomonadota;Alphaproteobacteria;Hyphomicrobiales;Phyllobacteriaceae;Aquibium;Aquibium microcysteis |
| Aquibium oceanicum | 0 | 0 | 0 | 0 | 0 | 0 | 0 | 0 | 0 | 0 | 0 | 0 | 0 | 0 | Bacteria | Bacteria\_none | Pseudomonadota | Alphaproteobacteria | Hyphomicrobiales | Phyllobacteriaceae | Aquibium | Bacteria;Bacteria\_none;Pseudomonadota;Alphaproteobacteria;Hyphomicrobiales;Phyllobacteriaceae;Aquibium;Aquibium oceanicum |
| Mesorhizobium huakuii | 0 | 0 | 0 | 0 | 0 | 0 | 0 | 0 | 0 | 0 | 0 | 0 | 0 | 0 | Bacteria | Bacteria\_none | Pseudomonadota | Alphaproteobacteria | Hyphomicrobiales | Phyllobacteriaceae | Mesorhizobium | Bacteria;Bacteria\_none;Pseudomonadota;Alphaproteobacteria;Hyphomicrobiales;Phyllobacteriaceae;Mesorhizobium;Mesorhizobium huakuii |
| Mesorhizobium koreense | 0 | 0 | 1 | 0 | 0 | 0 | 0 | 0 | 0 | 0 | 0 | 0 | 0 | 0 | Bacteria | Bacteria\_none | Pseudomonadota | Alphaproteobacteria | Hyphomicrobiales | Phyllobacteriaceae | Mesorhizobium | Bacteria;Bacteria\_none;Pseudomonadota;Alphaproteobacteria;Hyphomicrobiales;Phyllobacteriaceae;Mesorhizobium;Mesorhizobium koreense |
| Mesorhizobium sp. M6A.T.Cr.TU.016.01.1.1 | 0 | 0 | 1 | 0 | 0 | 0 | 0 | 0 | 0 | 0 | 0 | 0 | 0 | 0 | Bacteria | Bacteria\_none | Pseudomonadota | Alphaproteobacteria | Hyphomicrobiales | Phyllobacteriaceae | Mesorhizobium | Bacteria;Bacteria\_none;Pseudomonadota;Alphaproteobacteria;Hyphomicrobiales;Phyllobacteriaceae;Mesorhizobium;Mesorhizobium sp. M6A.T.Cr.TU.016.01.1.1 |
| Mesorhizobium sp. NZP2077 | 0 | 0 | 0 | 0 | 0 | 1 | 0 | 0 | 0 | 0 | 0 | 0 | 0 | 0 | Bacteria | Bacteria\_none | Pseudomonadota | Alphaproteobacteria | Hyphomicrobiales | Phyllobacteriaceae | Mesorhizobium | Bacteria;Bacteria\_none;Pseudomonadota;Alphaproteobacteria;Hyphomicrobiales;Phyllobacteriaceae;Mesorhizobium;Mesorhizobium sp. NZP2077 |
| Nitratireductor rhodophyticola | 0 | 0 | 0 | 0 | 0 | 0 | 0 | 0 | 0 | 0 | 0 | 0 | 0 | 0 | Bacteria | Bacteria\_none | Pseudomonadota | Alphaproteobacteria | Hyphomicrobiales | Phyllobacteriaceae | Nitratireductor | Bacteria;Bacteria\_none;Pseudomonadota;Alphaproteobacteria;Hyphomicrobiales;Phyllobacteriaceae;Nitratireductor;Nitratireductor rhodophyticola |
| Agrobacterium fabrum | 0 | 0 | 0 | 0 | 0 | 0 | 0 | 0 | 0 | 0 | 0 | 0 | 0 | 0 | Bacteria | Bacteria\_none | Pseudomonadota | Alphaproteobacteria | Hyphomicrobiales | Rhizobiaceae | Agrobacterium | Bacteria;Bacteria\_none;Pseudomonadota;Alphaproteobacteria;Hyphomicrobiales;Rhizobiaceae;Agrobacterium;Agrobacterium fabrum |
| Candidatus Liberibacter asiaticus | 0 | 0 | 0 | 0 | 0 | 0 | 0 | 0 | 0 | 0 | 0 | 0 | 0 | 0 | Bacteria | Bacteria\_none | Pseudomonadota | Alphaproteobacteria | Hyphomicrobiales | Rhizobiaceae | Liberibacter | Bacteria;Bacteria\_none;Pseudomonadota;Alphaproteobacteria;Hyphomicrobiales;Rhizobiaceae;Liberibacter;Candidatus Liberibacter asiaticus |
| Pararhizobium qamdonense | 0 | 0 | 0 | 0 | 0 | 0 | 0 | 0 | 0 | 0 | 0 | 0 | 0 | 0 | Bacteria | Bacteria\_none | Pseudomonadota | Alphaproteobacteria | Hyphomicrobiales | Rhizobiaceae | Pararhizobium | Bacteria;Bacteria\_none;Pseudomonadota;Alphaproteobacteria;Hyphomicrobiales;Rhizobiaceae;Pararhizobium;Pararhizobium qamdonense |
| Rhizobium hidalgonense | 0 | 0 | 0 | 0 | 0 | 0 | 0 | 0 | 0 | 0 | 0 | 0 | 0 | 0 | Bacteria | Bacteria\_none | Pseudomonadota | Alphaproteobacteria | Hyphomicrobiales | Rhizobiaceae | Rhizobium | Bacteria;Bacteria\_none;Pseudomonadota;Alphaproteobacteria;Hyphomicrobiales;Rhizobiaceae;Rhizobium;Rhizobium hidalgonense |
| Rhizobium rhizogenes | 0 | 0 | 0 | 0 | 0 | 0 | 0 | 0 | 0 | 0 | 0 | 0 | 0 | 0 | Bacteria | Bacteria\_none | Pseudomonadota | Alphaproteobacteria | Hyphomicrobiales | Rhizobiaceae | Rhizobium | Bacteria;Bacteria\_none;Pseudomonadota;Alphaproteobacteria;Hyphomicrobiales;Rhizobiaceae;Rhizobium;Rhizobium rhizogenes |
| Rhizobium tumorigenes | 0 | 0 | 0 | 0 | 0 | 0 | 0 | 0 | 0 | 0 | 0 | 0 | 0 | 0 | Bacteria | Bacteria\_none | Pseudomonadota | Alphaproteobacteria | Hyphomicrobiales | Rhizobiaceae | Rhizobium | Bacteria;Bacteria\_none;Pseudomonadota;Alphaproteobacteria;Hyphomicrobiales;Rhizobiaceae;Rhizobium;Rhizobium tumorigenes |
| Shinella zoogloeoides | 0 | 0 | 0 | 0 | 0 | 1 | 0 | 0 | 0 | 0 | 0 | 0 | 0 | 0 | Bacteria | Bacteria\_none | Pseudomonadota | Alphaproteobacteria | Hyphomicrobiales | Rhizobiaceae | Shinella | Bacteria;Bacteria\_none;Pseudomonadota;Alphaproteobacteria;Hyphomicrobiales;Rhizobiaceae;Shinella;Shinella zoogloeoides |
| Sinorhizobium medicae | 1 | 0 | 0 | 0 | 0 | 0 | 0 | 0 | 0 | 0 | 0 | 0 | 0 | 0 | Bacteria | Bacteria\_none | Pseudomonadota | Alphaproteobacteria | Hyphomicrobiales | Rhizobiaceae | Sinorhizobium | Bacteria;Bacteria\_none;Pseudomonadota;Alphaproteobacteria;Hyphomicrobiales;Rhizobiaceae;Sinorhizobium;Sinorhizobium medicae |
| Sinorhizobium numidicum | 0 | 0 | 0 | 0 | 1 | 0 | 0 | 0 | 0 | 0 | 0 | 0 | 0 | 0 | Bacteria | Bacteria\_none | Pseudomonadota | Alphaproteobacteria | Hyphomicrobiales | Rhizobiaceae | Sinorhizobium | Bacteria;Bacteria\_none;Pseudomonadota;Alphaproteobacteria;Hyphomicrobiales;Rhizobiaceae;Sinorhizobium;Sinorhizobium numidicum |
| Kordiimonas pumila | 0 | 0 | 0 | 0 | 0 | 0 | 0 | 0 | 0 | 0 | 0 | 0 | 0 | 0 | Bacteria | Bacteria\_none | Pseudomonadota | Alphaproteobacteria | Kordiimonadales | Kordiimonadaceae | Kordiimonas | Bacteria;Bacteria\_none;Pseudomonadota;Alphaproteobacteria;Kordiimonadales;Kordiimonadaceae;Kordiimonas;Kordiimonas pumila |
| Cereibacter sphaeroides | 0 | 0 | 0 | 0 | 0 | 0 | 0 | 0 | 0 | 0 | 0 | 0 | 0 | 0 | Bacteria | Bacteria\_none | Pseudomonadota | Alphaproteobacteria | Rhodobacterales | Paracoccaceae | Cereibacter | Bacteria;Bacteria\_none;Pseudomonadota;Alphaproteobacteria;Rhodobacterales;Paracoccaceae;Cereibacter;Cereibacter sphaeroides |
| Paracoccus aminovorans | 0 | 0 | 0 | 0 | 0 | 0 | 0 | 0 | 1 | 0 | 0 | 0 | 0 | 0 | Bacteria | Bacteria\_none | Pseudomonadota | Alphaproteobacteria | Rhodobacterales | Paracoccaceae | Paracoccus | Bacteria;Bacteria\_none;Pseudomonadota;Alphaproteobacteria;Rhodobacterales;Paracoccaceae;Paracoccus;Paracoccus aminovorans |
| Paracoccus mutanolyticus | 0 | 0 | 0 | 0 | 0 | 1 | 0 | 0 | 0 | 0 | 0 | 0 | 0 | 0 | Bacteria | Bacteria\_none | Pseudomonadota | Alphaproteobacteria | Rhodobacterales | Paracoccaceae | Paracoccus | Bacteria;Bacteria\_none;Pseudomonadota;Alphaproteobacteria;Rhodobacterales;Paracoccaceae;Paracoccus;Paracoccus mutanolyticus |
| Tabrizicola piscis | 0 | 0 | 0 | 0 | 0 | 0 | 0 | 0 | 0 | 0 | 0 | 0 | 0 | 0 | Bacteria | Bacteria\_none | Pseudomonadota | Alphaproteobacteria | Rhodobacterales | Paracoccaceae | Tabrizicola | Bacteria;Bacteria\_none;Pseudomonadota;Alphaproteobacteria;Rhodobacterales;Paracoccaceae;Tabrizicola;Tabrizicola piscis |
| Phaeobacter inhibens | 0 | 0 | 0 | 0 | 1 | 0 | 0 | 0 | 0 | 0 | 0 | 0 | 0 | 0 | Bacteria | Bacteria\_none | Pseudomonadota | Alphaproteobacteria | Rhodobacterales | Roseobacteraceae | Phaeobacter | Bacteria;Bacteria\_none;Pseudomonadota;Alphaproteobacteria;Rhodobacterales;Roseobacteraceae;Phaeobacter;Phaeobacter inhibens |
| Ponticoccus alexandrii | 2 | 0 | 0 | 0 | 0 | 0 | 0 | 0 | 0 | 0 | 0 | 0 | 0 | 0 | Bacteria | Bacteria\_none | Pseudomonadota | Alphaproteobacteria | Rhodobacterales | Roseobacteraceae | Ponticoccus | Bacteria;Bacteria\_none;Pseudomonadota;Alphaproteobacteria;Rhodobacterales;Roseobacteraceae;Ponticoccus;Ponticoccus alexandrii |
| Pseudosulfitobacter pseudonitzschiae | 0 | 1 | 0 | 0 | 0 | 0 | 0 | 0 | 0 | 0 | 0 | 0 | 0 | 0 | Bacteria | Bacteria\_none | Pseudomonadota | Alphaproteobacteria | Rhodobacterales | Roseobacteraceae | Pseudosulfitobacter | Bacteria;Bacteria\_none;Pseudomonadota;Alphaproteobacteria;Rhodobacterales;Roseobacteraceae;Pseudosulfitobacter;Pseudosulfitobacter pseudonitzschiae |
| Roseobacter litoralis | 0 | 0 | 0 | 0 | 0 | 0 | 0 | 0 | 0 | 0 | 0 | 0 | 0 | 0 | Bacteria | Bacteria\_none | Pseudomonadota | Alphaproteobacteria | Rhodobacterales | Roseobacteraceae | Roseobacter | Bacteria;Bacteria\_none;Pseudomonadota;Alphaproteobacteria;Rhodobacterales;Roseobacteraceae;Roseobacter;Roseobacter litoralis |
| Ruegeria\_uncultured | 0 | 0 | 0 | 0 | 1 | 0 | 0 | 0 | 0 | 0 | 0 | 0 | 0 | 0 | Bacteria | Bacteria\_none | Pseudomonadota | Alphaproteobacteria | Rhodobacterales | Roseobacteraceae | Ruegeria | Bacteria;Bacteria\_none;Pseudomonadota;Alphaproteobacteria;Rhodobacterales;Roseobacteraceae;Ruegeria;Ruegeria\_uncultured |
| Sulfitobacter alexandrii | 0 | 0 | 0 | 0 | 0 | 0 | 0 | 0 | 0 | 0 | 1 | 0 | 0 | 0 | Bacteria | Bacteria\_none | Pseudomonadota | Alphaproteobacteria | Rhodobacterales | Roseobacteraceae | Sulfitobacter | Bacteria;Bacteria\_none;Pseudomonadota;Alphaproteobacteria;Rhodobacterales;Roseobacteraceae;Sulfitobacter;Sulfitobacter alexandrii |
| Sulfitobacter faviae | 0 | 0 | 0 | 0 | 0 | 0 | 0 | 0 | 0 | 0 | 0 | 0 | 0 | 0 | Bacteria | Bacteria\_none | Pseudomonadota | Alphaproteobacteria | Rhodobacterales | Roseobacteraceae | Sulfitobacter | Bacteria;Bacteria\_none;Pseudomonadota;Alphaproteobacteria;Rhodobacterales;Roseobacteraceae;Sulfitobacter;Sulfitobacter faviae |
| Komagataeibacter rhaeticus | 0 | 0 | 0 | 0 | 0 | 0 | 0 | 0 | 0 | 0 | 0 | 0 | 0 | 0 | Bacteria | Bacteria\_none | Pseudomonadota | Alphaproteobacteria | Rhodospirillales | Acetobacteraceae | Komagataeibacter | Bacteria;Bacteria\_none;Pseudomonadota;Alphaproteobacteria;Rhodospirillales;Acetobacteraceae;Komagataeibacter;Komagataeibacter rhaeticus |
| Pseudoroseomonas cervicalis | 0 | 0 | 0 | 0 | 0 | 0 | 0 | 0 | 0 | 0 | 0 | 0 | 0 | 0 | Bacteria | Bacteria\_none | Pseudomonadota | Alphaproteobacteria | Rhodospirillales | Acetobacteraceae | Pseudoroseomonas | Bacteria;Bacteria\_none;Pseudomonadota;Alphaproteobacteria;Rhodospirillales;Acetobacteraceae;Pseudoroseomonas;Pseudoroseomonas cervicalis |
| Azospirillum ramasamyi | 0 | 0 | 0 | 0 | 0 | 0 | 0 | 0 | 0 | 0 | 1 | 0 | 0 | 0 | Bacteria | Bacteria\_none | Pseudomonadota | Alphaproteobacteria | Rhodospirillales | Azospirillaceae | Azospirillum | Bacteria;Bacteria\_none;Pseudomonadota;Alphaproteobacteria;Rhodospirillales;Azospirillaceae;Azospirillum;Azospirillum ramasamyi |
| Azospirillum sp. TSH100 | 0 | 0 | 0 | 0 | 0 | 0 | 0 | 0 | 0 | 0 | 0 | 0 | 0 | 0 | Bacteria | Bacteria\_none | Pseudomonadota | Alphaproteobacteria | Rhodospirillales | Azospirillaceae | Azospirillum | Bacteria;Bacteria\_none;Pseudomonadota;Alphaproteobacteria;Rhodospirillales;Azospirillaceae;Azospirillum;Azospirillum sp. TSH100 |
| Varunaivibrio sulfuroxidans | 0 | 0 | 0 | 0 | 0 | 0 | 0 | 0 | 0 | 0 | 0 | 0 | 0 | 0 | Bacteria | Bacteria\_none | Pseudomonadota | Alphaproteobacteria | Rhodospirillales | Magnetovibrionaceae | Varunaivibrio | Bacteria;Bacteria\_none;Pseudomonadota;Alphaproteobacteria;Rhodospirillales;Magnetovibrionaceae;Varunaivibrio;Varunaivibrio sulfuroxidans |
| Thalassospira sp. SN3W | 0 | 0 | 0 | 1 | 0 | 0 | 0 | 0 | 0 | 0 | 0 | 0 | 0 | 0 | Bacteria | Bacteria\_none | Pseudomonadota | Alphaproteobacteria | Rhodospirillales | Thalassospiraceae | Thalassospira | Bacteria;Bacteria\_none;Pseudomonadota;Alphaproteobacteria;Rhodospirillales;Thalassospiraceae;Thalassospira;Thalassospira sp. SN3W |
| Neorickettsia sennetsu | 0 | 1 | 0 | 0 | 0 | 0 | 0 | 0 | 0 | 0 | 0 | 0 | 0 | 0 | Bacteria | Bacteria\_none | Pseudomonadota | Alphaproteobacteria | Rickettsiales | Anaplasmataceae | Neorickettsia | Bacteria;Bacteria\_none;Pseudomonadota;Alphaproteobacteria;Rickettsiales;Anaplasmataceae;Neorickettsia;Neorickettsia sennetsu |
| Wolbachia endosymbiont (group B) of Phasia obesa | 0 | 0 | 0 | 0 | 0 | 0 | 0 | 0 | 0 | 0 | 0 | 0 | 0 | 0 | Bacteria | Bacteria\_none | Pseudomonadota | Alphaproteobacteria | Rickettsiales | Anaplasmataceae | Wolbachia | Bacteria;Bacteria\_none;Pseudomonadota;Alphaproteobacteria;Rickettsiales;Anaplasmataceae;Wolbachia;Wolbachia endosymbiont (group B) of Phasia obesa |
| Wolbachia endosymbiont of Ctenocephalides felis wCfeT | 0 | 0 | 0 | 0 | 0 | 0 | 0 | 0 | 0 | 0 | 0 | 0 | 0 | 0 | Bacteria | Bacteria\_none | Pseudomonadota | Alphaproteobacteria | Rickettsiales | Anaplasmataceae | Wolbachia | Bacteria;Bacteria\_none;Pseudomonadota;Alphaproteobacteria;Rickettsiales;Anaplasmataceae;Wolbachia;Wolbachia endosymbiont of Ctenocephalides felis wCfeT |
| Wolbachia endosymbiont of Dirofilaria (Dirofilaria) immitis | 0 | 0 | 0 | 0 | 0 | 0 | 0 | 0 | 0 | 0 | 0 | 0 | 0 | 0 | Bacteria | Bacteria\_none | Pseudomonadota | Alphaproteobacteria | Rickettsiales | Anaplasmataceae | Wolbachia | Bacteria;Bacteria\_none;Pseudomonadota;Alphaproteobacteria;Rickettsiales;Anaplasmataceae;Wolbachia;Wolbachia endosymbiont of Dirofilaria (Dirofilaria) immitis |
| Candidatus Phycorickettsia trachydisci | 0 | 0 | 0 | 0 | 0 | 0 | 0 | 0 | 0 | 0 | 0 | 0 | 0 | 0 | Bacteria | Bacteria\_none | Pseudomonadota | Alphaproteobacteria | Rickettsiales | Rickettsiaceae | Candidatus Phycorickettsia | Bacteria;Bacteria\_none;Pseudomonadota;Alphaproteobacteria;Rickettsiales;Rickettsiaceae;Candidatus Phycorickettsia;Candidatus Phycorickettsia trachydisci |
| Novosphingobium sp. KACC 22771 | 0 | 0 | 0 | 0 | 0 | 0 | 0 | 0 | 0 | 0 | 0 | 0 | 1 | 0 | Bacteria | Bacteria\_none | Pseudomonadota | Alphaproteobacteria | Sphingomonadales | Sphingomonadaceae | Novosphingobium | Bacteria;Bacteria\_none;Pseudomonadota;Alphaproteobacteria;Sphingomonadales;Sphingomonadaceae;Novosphingobium;Novosphingobium sp. KACC 22771 |
| Sphingomonas flavescens | 0 | 0 | 0 | 0 | 0 | 0 | 0 | 0 | 0 | 0 | 0 | 0 | 0 | 0 | Bacteria | Bacteria\_none | Pseudomonadota | Alphaproteobacteria | Sphingomonadales | Sphingomonadaceae | Sphingomonas | Bacteria;Bacteria\_none;Pseudomonadota;Alphaproteobacteria;Sphingomonadales;Sphingomonadaceae;Sphingomonas;Sphingomonas flavescens |
| Sphingomonas ginsengisoli An et al. 2013 | 0 | 0 | 0 | 0 | 0 | 0 | 0 | 0 | 0 | 0 | 0 | 0 | 0 | 1 | Bacteria | Bacteria\_none | Pseudomonadota | Alphaproteobacteria | Sphingomonadales | Sphingomonadaceae | Sphingomonas | Bacteria;Bacteria\_none;Pseudomonadota;Alphaproteobacteria;Sphingomonadales;Sphingomonadaceae;Sphingomonas;Sphingomonas ginsengisoli An et al. 2013 |
| Sphingomonas xanthus | 0 | 0 | 0 | 0 | 0 | 0 | 0 | 0 | 0 | 0 | 0 | 0 | 1 | 0 | Bacteria | Bacteria\_none | Pseudomonadota | Alphaproteobacteria | Sphingomonadales | Sphingomonadaceae | Sphingomonas | Bacteria;Bacteria\_none;Pseudomonadota;Alphaproteobacteria;Sphingomonadales;Sphingomonadaceae;Sphingomonas;Sphingomonas xanthus |
| Sphingopyxis fribergensis | 0 | 0 | 0 | 0 | 0 | 1 | 0 | 0 | 0 | 0 | 0 | 0 | 0 | 0 | Bacteria | Bacteria\_none | Pseudomonadota | Alphaproteobacteria | Sphingomonadales | Sphingomonadaceae | Sphingopyxis | Bacteria;Bacteria\_none;Pseudomonadota;Alphaproteobacteria;Sphingomonadales;Sphingomonadaceae;Sphingopyxis;Sphingopyxis fribergensis |
| Sphingopyxis sp. TUF1 | 0 | 0 | 0 | 0 | 0 | 0 | 0 | 0 | 0 | 0 | 0 | 0 | 0 | 0 | Bacteria | Bacteria\_none | Pseudomonadota | Alphaproteobacteria | Sphingomonadales | Sphingomonadaceae | Sphingopyxis | Bacteria;Bacteria\_none;Pseudomonadota;Alphaproteobacteria;Sphingomonadales;Sphingomonadaceae;Sphingopyxis;Sphingopyxis sp. TUF1 |
| Candidatus Profftella armatura | 0 | 0 | 0 | 0 | 1 | 0 | 0 | 0 | 0 | 0 | 0 | 0 | 0 | 0 | Bacteria | Bacteria\_none | Pseudomonadota | Betaproteobacteria | Betaproteobacteria\_Incertae\_sedis | Betaproteobacteria\_Incertae\_sedis | Candidatus Profftella | Bacteria;Bacteria\_none;Pseudomonadota;Betaproteobacteria;Betaproteobacteria\_Incertae\_sedis;Betaproteobacteria\_Incertae\_sedis;Candidatus Profftella;Candidatus Profftella armatura |
| Casimicrobium huifangae | 0 | 0 | 0 | 0 | 0 | 0 | 0 | 0 | 0 | 0 | 0 | 0 | 0 | 0 | Bacteria | Bacteria\_none | Pseudomonadota | Betaproteobacteria | Betaproteobacteria\_Incertae\_sedis | Casimicrobiaceae | Casimicrobium | Bacteria;Bacteria\_none;Pseudomonadota;Betaproteobacteria;Betaproteobacteria\_Incertae\_sedis;Casimicrobiaceae;Casimicrobium;Casimicrobium huifangae |
| Advenella alkanexedens | 0 | 0 | 0 | 0 | 1 | 0 | 0 | 0 | 0 | 0 | 0 | 0 | 0 | 0 | Bacteria | Bacteria\_none | Pseudomonadota | Betaproteobacteria | Burkholderiales | Alcaligenaceae | Advenella | Bacteria;Bacteria\_none;Pseudomonadota;Betaproteobacteria;Burkholderiales;Alcaligenaceae;Advenella;Advenella alkanexedens |
| Bordetella avium | 0 | 0 | 0 | 0 | 0 | 0 | 0 | 0 | 0 | 0 | 1 | 0 | 0 | 0 | Bacteria | Bacteria\_none | Pseudomonadota | Betaproteobacteria | Burkholderiales | Alcaligenaceae | Bordetella | Bacteria;Bacteria\_none;Pseudomonadota;Betaproteobacteria;Burkholderiales;Alcaligenaceae;Bordetella;Bordetella avium |
| Neopusillimonas aromaticivorans | 0 | 0 | 0 | 0 | 0 | 0 | 0 | 0 | 0 | 0 | 0 | 0 | 0 | 0 | Bacteria | Bacteria\_none | Pseudomonadota | Betaproteobacteria | Burkholderiales | Alcaligenaceae | Neopusillimonas | Bacteria;Bacteria\_none;Pseudomonadota;Betaproteobacteria;Burkholderiales;Alcaligenaceae;Neopusillimonas;Neopusillimonas aromaticivorans |
| Orrella dioscoreae | 0 | 0 | 0 | 0 | 0 | 1 | 0 | 0 | 0 | 0 | 0 | 0 | 0 | 0 | Bacteria | Bacteria\_none | Pseudomonadota | Betaproteobacteria | Burkholderiales | Alcaligenaceae | Orrella | Bacteria;Bacteria\_none;Pseudomonadota;Betaproteobacteria;Burkholderiales;Alcaligenaceae;Orrella;Orrella dioscoreae |
| Pollutimonas thiosulfatoxidans | 0 | 0 | 0 | 0 | 0 | 0 | 0 | 0 | 0 | 0 | 0 | 0 | 0 | 0 | Bacteria | Bacteria\_none | Pseudomonadota | Betaproteobacteria | Burkholderiales | Alcaligenaceae | Pollutimonas | Bacteria;Bacteria\_none;Pseudomonadota;Betaproteobacteria;Burkholderiales;Alcaligenaceae;Pollutimonas;Pollutimonas thiosulfatoxidans |
| Pusillimonas sp. T7-7 | 0 | 0 | 0 | 0 | 0 | 0 | 0 | 0 | 0 | 0 | 0 | 0 | 0 | 1 | Bacteria | Bacteria\_none | Pseudomonadota | Betaproteobacteria | Burkholderiales | Alcaligenaceae | Pusillimonas | Bacteria;Bacteria\_none;Pseudomonadota;Betaproteobacteria;Burkholderiales;Alcaligenaceae;Pusillimonas;Pusillimonas sp. T7-7 |
| Burkholderia cenocepacia | 0 | 0 | 0 | 0 | 0 | 0 | 0 | 0 | 0 | 0 | 0 | 0 | 0 | 0 | Bacteria | Bacteria\_none | Pseudomonadota | Betaproteobacteria | Burkholderiales | Burkholderiaceae | Burkholderia | Bacteria;Bacteria\_none;Pseudomonadota;Betaproteobacteria;Burkholderiales;Burkholderiaceae;Burkholderia;Burkholderia cenocepacia |
| Burkholderia cepacia | 0 | 0 | 0 | 0 | 0 | 0 | 0 | 0 | 0 | 0 | 0 | 0 | 0 | 0 | Bacteria | Bacteria\_none | Pseudomonadota | Betaproteobacteria | Burkholderiales | Burkholderiaceae | Burkholderia | Bacteria;Bacteria\_none;Pseudomonadota;Betaproteobacteria;Burkholderiales;Burkholderiaceae;Burkholderia;Burkholderia cepacia |
| Burkholderia dolosa | 0 | 0 | 0 | 0 | 0 | 0 | 0 | 0 | 0 | 0 | 0 | 0 | 0 | 0 | Bacteria | Bacteria\_none | Pseudomonadota | Betaproteobacteria | Burkholderiales | Burkholderiaceae | Burkholderia | Bacteria;Bacteria\_none;Pseudomonadota;Betaproteobacteria;Burkholderiales;Burkholderiaceae;Burkholderia;Burkholderia dolosa |
| Burkholderia multivorans | 0 | 0 | 2 | 0 | 0 | 0 | 0 | 0 | 0 | 0 | 0 | 0 | 0 | 1 | Bacteria | Bacteria\_none | Pseudomonadota | Betaproteobacteria | Burkholderiales | Burkholderiaceae | Burkholderia | Bacteria;Bacteria\_none;Pseudomonadota;Betaproteobacteria;Burkholderiales;Burkholderiaceae;Burkholderia;Burkholderia multivorans |
| Cupriavidus oxalaticus | 0 | 0 | 0 | 0 | 0 | 0 | 0 | 0 | 0 | 0 | 0 | 0 | 0 | 0 | Bacteria | Bacteria\_none | Pseudomonadota | Betaproteobacteria | Burkholderiales | Burkholderiaceae | Cupriavidus | Bacteria;Bacteria\_none;Pseudomonadota;Betaproteobacteria;Burkholderiales;Burkholderiaceae;Cupriavidus;Cupriavidus oxalaticus |
| Lautropia mirabilis | 0 | 1 | 0 | 0 | 4 | 0 | 0 | 0 | 0 | 1 | 4 | 0 | 1 | 1 | Bacteria | Bacteria\_none | Pseudomonadota | Betaproteobacteria | Burkholderiales | Burkholderiaceae | Lautropia | Bacteria;Bacteria\_none;Pseudomonadota;Betaproteobacteria;Burkholderiales;Burkholderiaceae;Lautropia;Lautropia mirabilis |
| Mycoavidus sp. B2-EB | 0 | 0 | 0 | 0 | 0 | 0 | 0 | 0 | 0 | 0 | 0 | 0 | 0 | 0 | Bacteria | Bacteria\_none | Pseudomonadota | Betaproteobacteria | Burkholderiales | Burkholderiaceae | Mycoavidus | Bacteria;Bacteria\_none;Pseudomonadota;Betaproteobacteria;Burkholderiales;Burkholderiaceae;Mycoavidus;Mycoavidus sp. B2-EB |
| Pandoraea pnomenusa | 1 | 0 | 0 | 0 | 0 | 0 | 0 | 0 | 0 | 0 | 0 | 0 | 0 | 0 | Bacteria | Bacteria\_none | Pseudomonadota | Betaproteobacteria | Burkholderiales | Burkholderiaceae | Pandoraea | Bacteria;Bacteria\_none;Pseudomonadota;Betaproteobacteria;Burkholderiales;Burkholderiaceae;Pandoraea;Pandoraea pnomenusa |
| Paraburkholderia acidisoli | 0 | 0 | 0 | 0 | 0 | 0 | 1 | 0 | 0 | 0 | 0 | 0 | 0 | 0 | Bacteria | Bacteria\_none | Pseudomonadota | Betaproteobacteria | Burkholderiales | Burkholderiaceae | Paraburkholderia | Bacteria;Bacteria\_none;Pseudomonadota;Betaproteobacteria;Burkholderiales;Burkholderiaceae;Paraburkholderia;Paraburkholderia acidisoli |
| Paraburkholderia sp. PGU19 | 0 | 0 | 0 | 0 | 0 | 0 | 0 | 0 | 0 | 0 | 0 | 0 | 0 | 0 | Bacteria | Bacteria\_none | Pseudomonadota | Betaproteobacteria | Burkholderiales | Burkholderiaceae | Paraburkholderia | Bacteria;Bacteria\_none;Pseudomonadota;Betaproteobacteria;Burkholderiales;Burkholderiaceae;Paraburkholderia;Paraburkholderia sp. PGU19 |
| Polynucleobacter necessarius | 0 | 0 | 0 | 0 | 0 | 0 | 0 | 0 | 0 | 0 | 0 | 0 | 0 | 0 | Bacteria | Bacteria\_none | Pseudomonadota | Betaproteobacteria | Burkholderiales | Burkholderiaceae | Polynucleobacter | Bacteria;Bacteria\_none;Pseudomonadota;Betaproteobacteria;Burkholderiales;Burkholderiaceae;Polynucleobacter;Polynucleobacter necessarius |
| Ralstonia solanacearum | 0 | 0 | 0 | 0 | 0 | 0 | 0 | 0 | 0 | 0 | 0 | 0 | 0 | 0 | Bacteria | Bacteria\_none | Pseudomonadota | Betaproteobacteria | Burkholderiales | Burkholderiaceae | Ralstonia | Bacteria;Bacteria\_none;Pseudomonadota;Betaproteobacteria;Burkholderiales;Burkholderiaceae;Ralstonia;Ralstonia solanacearum |
| Acidovorax sp. FHTAMBA | 0 | 0 | 0 | 0 | 0 | 0 | 0 | 0 | 0 | 0 | 0 | 0 | 0 | 0 | Bacteria | Bacteria\_none | Pseudomonadota | Betaproteobacteria | Burkholderiales | Comamonadaceae | Acidovorax | Bacteria;Bacteria\_none;Pseudomonadota;Betaproteobacteria;Burkholderiales;Comamonadaceae;Acidovorax;Acidovorax sp. FHTAMBA |
| Hydrogenophaga sp. SL48 | 0 | 0 | 0 | 0 | 0 | 1 | 0 | 0 | 0 | 0 | 0 | 0 | 0 | 0 | Bacteria | Bacteria\_none | Pseudomonadota | Betaproteobacteria | Burkholderiales | Comamonadaceae | Hydrogenophaga | Bacteria;Bacteria\_none;Pseudomonadota;Betaproteobacteria;Burkholderiales;Comamonadaceae;Hydrogenophaga;Hydrogenophaga sp. SL48 |
| Ottowia sp. oral taxon 894 | 0 | 0 | 0 | 0 | 0 | 0 | 0 | 0 | 0 | 0 | 0 | 0 | 0 | 0 | Bacteria | Bacteria\_none | Pseudomonadota | Betaproteobacteria | Burkholderiales | Comamonadaceae | Ottowia | Bacteria;Bacteria\_none;Pseudomonadota;Betaproteobacteria;Burkholderiales;Comamonadaceae;Ottowia;Ottowia sp. oral taxon 894 |
| Variovorax paradoxus | 0 | 0 | 0 | 0 | 0 | 0 | 1 | 0 | 0 | 0 | 0 | 0 | 0 | 0 | Bacteria | Bacteria\_none | Pseudomonadota | Betaproteobacteria | Burkholderiales | Comamonadaceae | Variovorax | Bacteria;Bacteria\_none;Pseudomonadota;Betaproteobacteria;Burkholderiales;Comamonadaceae;Variovorax;Variovorax paradoxus |
| Variovorax sp. HW608 | 0 | 0 | 0 | 0 | 0 | 0 | 0 | 0 | 0 | 0 | 0 | 0 | 0 | 0 | Bacteria | Bacteria\_none | Pseudomonadota | Betaproteobacteria | Burkholderiales | Comamonadaceae | Variovorax | Bacteria;Bacteria\_none;Pseudomonadota;Betaproteobacteria;Burkholderiales;Comamonadaceae;Variovorax;Variovorax sp. HW608 |
| Candidatus Zinderia endosymbiont of Aphrophora alni | 0 | 0 | 0 | 0 | 0 | 0 | 0 | 0 | 0 | 0 | 0 | 0 | 0 | 0 | Bacteria | Bacteria\_none | Pseudomonadota | Betaproteobacteria | Burkholderiales | Oxalobacteraceae | Candidatus Zinderia | Bacteria;Bacteria\_none;Pseudomonadota;Betaproteobacteria;Burkholderiales;Oxalobacteraceae;Candidatus Zinderia;Candidatus Zinderia endosymbiont of Aphrophora alni |
| Janthinobacterium sp. TB1-E2 | 0 | 0 | 0 | 0 | 0 | 1 | 0 | 0 | 0 | 0 | 0 | 0 | 0 | 0 | Bacteria | Bacteria\_none | Pseudomonadota | Betaproteobacteria | Burkholderiales | Oxalobacteraceae | Janthinobacterium | Bacteria;Bacteria\_none;Pseudomonadota;Betaproteobacteria;Burkholderiales;Oxalobacteraceae;Janthinobacterium;Janthinobacterium sp. TB1-E2 |
| Massilia litorea | 0 | 0 | 0 | 0 | 0 | 0 | 0 | 0 | 0 | 1 | 0 | 0 | 0 | 0 | Bacteria | Bacteria\_none | Pseudomonadota | Betaproteobacteria | Burkholderiales | Oxalobacteraceae | Massilia | Bacteria;Bacteria\_none;Pseudomonadota;Betaproteobacteria;Burkholderiales;Oxalobacteraceae;Massilia;Massilia litorea |
| Noviherbaspirillum sp. UKPF54 | 0 | 0 | 1 | 0 | 0 | 0 | 0 | 0 | 0 | 0 | 0 | 0 | 0 | 0 | Bacteria | Bacteria\_none | Pseudomonadota | Betaproteobacteria | Burkholderiales | Oxalobacteraceae | Noviherbaspirillum | Bacteria;Bacteria\_none;Pseudomonadota;Betaproteobacteria;Burkholderiales;Oxalobacteraceae;Noviherbaspirillum;Noviherbaspirillum sp. UKPF54 |
| Oxalobacter formigenes | 0 | 0 | 0 | 0 | 0 | 0 | 0 | 0 | 0 | 0 | 0 | 0 | 0 | 1 | Bacteria | Bacteria\_none | Pseudomonadota | Betaproteobacteria | Burkholderiales | Oxalobacteraceae | Oxalobacter | Bacteria;Bacteria\_none;Pseudomonadota;Betaproteobacteria;Burkholderiales;Oxalobacteraceae;Oxalobacter;Oxalobacter formigenes |
| Pseudoduganella plicata | 0 | 0 | 0 | 0 | 0 | 0 | 0 | 0 | 0 | 0 | 0 | 0 | 0 | 0 | Bacteria | Bacteria\_none | Pseudomonadota | Betaproteobacteria | Burkholderiales | Oxalobacteraceae | Pseudoduganella | Bacteria;Bacteria\_none;Pseudomonadota;Betaproteobacteria;Burkholderiales;Oxalobacteraceae;Pseudoduganella;Pseudoduganella plicata |
| Piscinibacter gummiphilus | 0 | 0 | 0 | 0 | 0 | 0 | 0 | 0 | 0 | 0 | 0 | 1 | 0 | 0 | Bacteria | Bacteria\_none | Pseudomonadota | Betaproteobacteria | Burkholderiales | Sphaerotilaceae | Piscinibacter | Bacteria;Bacteria\_none;Pseudomonadota;Betaproteobacteria;Burkholderiales;Sphaerotilaceae;Piscinibacter;Piscinibacter gummiphilus |
| Rubrivivax gelatinosus | 0 | 0 | 0 | 0 | 0 | 0 | 0 | 0 | 0 | 0 | 0 | 0 | 0 | 0 | Bacteria | Bacteria\_none | Pseudomonadota | Betaproteobacteria | Burkholderiales | Sphaerotilaceae | Rubrivivax | Bacteria;Bacteria\_none;Pseudomonadota;Betaproteobacteria;Burkholderiales;Sphaerotilaceae;Rubrivivax;Rubrivivax gelatinosus |
| Alysiella crassa | 0 | 0 | 0 | 0 | 0 | 0 | 0 | 0 | 0 | 0 | 0 | 0 | 0 | 0 | Bacteria | Bacteria\_none | Pseudomonadota | Betaproteobacteria | Neisseriales | Neisseriaceae | Alysiella | Bacteria;Bacteria\_none;Pseudomonadota;Betaproteobacteria;Neisseriales;Neisseriaceae;Alysiella;Alysiella crassa |
| Alysiella filiformis | 0 | 0 | 0 | 0 | 1 | 0 | 0 | 0 | 0 | 0 | 0 | 0 | 0 | 0 | Bacteria | Bacteria\_none | Pseudomonadota | Betaproteobacteria | Neisseriales | Neisseriaceae | Alysiella | Bacteria;Bacteria\_none;Pseudomonadota;Betaproteobacteria;Neisseriales;Neisseriaceae;Alysiella;Alysiella filiformis |
| Aquella oligotrophica | 0 | 0 | 0 | 0 | 0 | 0 | 0 | 0 | 0 | 0 | 0 | 0 | 0 | 0 | Bacteria | Bacteria\_none | Pseudomonadota | Betaproteobacteria | Neisseriales | Neisseriaceae | Aquella | Bacteria;Bacteria\_none;Pseudomonadota;Betaproteobacteria;Neisseriales;Neisseriaceae;Aquella;Aquella oligotrophica |
| Conchiformibius steedae | 0 | 0 | 1 | 0 | 0 | 0 | 0 | 0 | 0 | 0 | 0 | 0 | 0 | 0 | Bacteria | Bacteria\_none | Pseudomonadota | Betaproteobacteria | Neisseriales | Neisseriaceae | Conchiformibius | Bacteria;Bacteria\_none;Pseudomonadota;Betaproteobacteria;Neisseriales;Neisseriaceae;Conchiformibius;Conchiformibius steedae |
| Eikenella corrodens | 0 | 2 | 26 | 4 | 9 | 0 | 0 | 1 | 0 | 1 | 2 | 0 | 1 | 0 | Bacteria | Bacteria\_none | Pseudomonadota | Betaproteobacteria | Neisseriales | Neisseriaceae | Eikenella | Bacteria;Bacteria\_none;Pseudomonadota;Betaproteobacteria;Neisseriales;Neisseriaceae;Eikenella;Eikenella corrodens |
| Eikenella exigua | 0 | 0 | 0 | 2 | 0 | 0 | 0 | 0 | 0 | 0 | 0 | 1 | 0 | 1 | Bacteria | Bacteria\_none | Pseudomonadota | Betaproteobacteria | Neisseriales | Neisseriaceae | Eikenella | Bacteria;Bacteria\_none;Pseudomonadota;Betaproteobacteria;Neisseriales;Neisseriaceae;Eikenella;Eikenella exigua |
| Kingella denitrificans | 0 | 14 | 1 | 20 | 0 | 0 | 0 | 0 | 0 | 0 | 1 | 0 | 0 | 0 | Bacteria | Bacteria\_none | Pseudomonadota | Betaproteobacteria | Neisseriales | Neisseriaceae | Kingella | Bacteria;Bacteria\_none;Pseudomonadota;Betaproteobacteria;Neisseriales;Neisseriaceae;Kingella;Kingella denitrificans |
| Kingella kingae | 0 | 0 | 0 | 0 | 0 | 0 | 0 | 0 | 0 | 0 | 0 | 0 | 0 | 0 | Bacteria | Bacteria\_none | Pseudomonadota | Betaproteobacteria | Neisseriales | Neisseriaceae | Kingella | Bacteria;Bacteria\_none;Pseudomonadota;Betaproteobacteria;Neisseriales;Neisseriaceae;Kingella;Kingella kingae |
| Kingella negevensis | 0 | 0 | 0 | 1 | 0 | 1 | 0 | 0 | 0 | 3 | 0 | 0 | 0 | 0 | Bacteria | Bacteria\_none | Pseudomonadota | Betaproteobacteria | Neisseriales | Neisseriaceae | Kingella | Bacteria;Bacteria\_none;Pseudomonadota;Betaproteobacteria;Neisseriales;Neisseriaceae;Kingella;Kingella negevensis |
| Kingella oralis | 1 | 8 | 1 | 0 | 0 | 0 | 0 | 1 | 0 | 2 | 0 | 0 | 0 | 0 | Bacteria | Bacteria\_none | Pseudomonadota | Betaproteobacteria | Neisseriales | Neisseriaceae | Kingella | Bacteria;Bacteria\_none;Pseudomonadota;Betaproteobacteria;Neisseriales;Neisseriaceae;Kingella;Kingella oralis |
| Morococcus cerebrosus | 0 | 3 | 7 | 16 | 0 | 0 | 0 | 1 | 0 | 1 | 1 | 2 | 3 | 3 | Bacteria | Bacteria\_none | Pseudomonadota | Betaproteobacteria | Neisseriales | Neisseriaceae | Morococcus | Bacteria;Bacteria\_none;Pseudomonadota;Betaproteobacteria;Neisseriales;Neisseriaceae;Morococcus;Morococcus cerebrosus |
| Neisseria animalis | 0 | 1 | 1 | 5 | 0 | 0 | 0 | 0 | 0 | 0 | 0 | 0 | 0 | 0 | Bacteria | Bacteria\_none | Pseudomonadota | Betaproteobacteria | Neisseriales | Neisseriaceae | Neisseria | Bacteria;Bacteria\_none;Pseudomonadota;Betaproteobacteria;Neisseriales;Neisseriaceae;Neisseria;Neisseria animalis |
| Neisseria animaloris | 0 | 0 | 0 | 1 | 1 | 0 | 0 | 0 | 0 | 2 | 0 | 0 | 1 | 0 | Bacteria | Bacteria\_none | Pseudomonadota | Betaproteobacteria | Neisseriales | Neisseriaceae | Neisseria | Bacteria;Bacteria\_none;Pseudomonadota;Betaproteobacteria;Neisseriales;Neisseriaceae;Neisseria;Neisseria animaloris |
| Neisseria arctica | 0 | 0 | 0 | 6 | 0 | 0 | 0 | 0 | 0 | 1 | 0 | 0 | 1 | 0 | Bacteria | Bacteria\_none | Pseudomonadota | Betaproteobacteria | Neisseriales | Neisseriaceae | Neisseria | Bacteria;Bacteria\_none;Pseudomonadota;Betaproteobacteria;Neisseriales;Neisseriaceae;Neisseria;Neisseria arctica |
| Neisseria bacilliformis | 0 | 2 | 0 | 3 | 0 | 0 | 0 | 0 | 0 | 0 | 0 | 0 | 0 | 1 | Bacteria | Bacteria\_none | Pseudomonadota | Betaproteobacteria | Neisseriales | Neisseriaceae | Neisseria | Bacteria;Bacteria\_none;Pseudomonadota;Betaproteobacteria;Neisseriales;Neisseriaceae;Neisseria;Neisseria bacilliformis |
| Neisseria brasiliensis | 0 | 0 | 0 | 3 | 0 | 0 | 0 | 1 | 0 | 2 | 0 | 0 | 0 | 0 | Bacteria | Bacteria\_none | Pseudomonadota | Betaproteobacteria | Neisseriales | Neisseriaceae | Neisseria | Bacteria;Bacteria\_none;Pseudomonadota;Betaproteobacteria;Neisseriales;Neisseriaceae;Neisseria;Neisseria brasiliensis |
| Neisseria canis | 0 | 0 | 1 | 0 | 0 | 0 | 0 | 0 | 0 | 0 | 0 | 0 | 0 | 0 | Bacteria | Bacteria\_none | Pseudomonadota | Betaproteobacteria | Neisseriales | Neisseriaceae | Neisseria | Bacteria;Bacteria\_none;Pseudomonadota;Betaproteobacteria;Neisseriales;Neisseriaceae;Neisseria;Neisseria canis |
| Neisseria chenwenguii | 0 | 2 | 0 | 3 | 0 | 0 | 0 | 0 | 0 | 0 | 0 | 0 | 0 | 0 | Bacteria | Bacteria\_none | Pseudomonadota | Betaproteobacteria | Neisseriales | Neisseriaceae | Neisseria | Bacteria;Bacteria\_none;Pseudomonadota;Betaproteobacteria;Neisseriales;Neisseriaceae;Neisseria;Neisseria chenwenguii |
| Neisseria cinerea | 0 | 1 | 7 | 32 | 4 | 0 | 0 | 0 | 0 | 200 | 0 | 1 | 14 | 4 | Bacteria | Bacteria\_none | Pseudomonadota | Betaproteobacteria | Neisseriales | Neisseriaceae | Neisseria | Bacteria;Bacteria\_none;Pseudomonadota;Betaproteobacteria;Neisseriales;Neisseriaceae;Neisseria;Neisseria cinerea |
| Neisseria dentiae | 0 | 0 | 0 | 2 | 0 | 2 | 0 | 0 | 0 | 0 | 0 | 0 | 0 | 0 | Bacteria | Bacteria\_none | Pseudomonadota | Betaproteobacteria | Neisseriales | Neisseriaceae | Neisseria | Bacteria;Bacteria\_none;Pseudomonadota;Betaproteobacteria;Neisseriales;Neisseriaceae;Neisseria;Neisseria dentiae |
| Neisseria dumasiana | 0 | 0 | 1 | 1 | 0 | 0 | 0 | 0 | 0 | 0 | 0 | 0 | 0 | 0 | Bacteria | Bacteria\_none | Pseudomonadota | Betaproteobacteria | Neisseriales | Neisseriaceae | Neisseria | Bacteria;Bacteria\_none;Pseudomonadota;Betaproteobacteria;Neisseriales;Neisseriaceae;Neisseria;Neisseria dumasiana |
| Neisseria elongata | 2 | 21 | 11 | 53 | 5 | 3 | 0 | 0 | 0 | 34 | 0 | 1 | 5 | 6 | Bacteria | Bacteria\_none | Pseudomonadota | Betaproteobacteria | Neisseriales | Neisseriaceae | Neisseria | Bacteria;Bacteria\_none;Pseudomonadota;Betaproteobacteria;Neisseriales;Neisseriaceae;Neisseria;Neisseria elongata |
| Neisseria flavescens | 4 | 8 | 24 | 117 | 10 | 6 | 0 | 1 | 0 | 5 | 0 | 0 | 9 | 4 | Bacteria | Bacteria\_none | Pseudomonadota | Betaproteobacteria | Neisseriales | Neisseriaceae | Neisseria | Bacteria;Bacteria\_none;Pseudomonadota;Betaproteobacteria;Neisseriales;Neisseriaceae;Neisseria;Neisseria flavescens |
| Neisseria gonorrhoeae | 2 | 1 | 29 | 25 | 2 | 1 | 0 | 1 | 0 | 24 | 2 | 1 | 4 | 2 | Bacteria | Bacteria\_none | Pseudomonadota | Betaproteobacteria | Neisseriales | Neisseriaceae | Neisseria | Bacteria;Bacteria\_none;Pseudomonadota;Betaproteobacteria;Neisseriales;Neisseriaceae;Neisseria;Neisseria gonorrhoeae |
| Neisseria lactamica | 1 | 0 | 4 | 16 | 1 | 1 | 0 | 0 | 0 | 11 | 0 | 0 | 4 | 4 | Bacteria | Bacteria\_none | Pseudomonadota | Betaproteobacteria | Neisseriales | Neisseriaceae | Neisseria | Bacteria;Bacteria\_none;Pseudomonadota;Betaproteobacteria;Neisseriales;Neisseriaceae;Neisseria;Neisseria lactamica |
| Neisseria leonii | 0 | 0 | 0 | 0 | 0 | 1 | 0 | 0 | 0 | 0 | 0 | 0 | 0 | 0 | Bacteria | Bacteria\_none | Pseudomonadota | Betaproteobacteria | Neisseriales | Neisseriaceae | Neisseria | Bacteria;Bacteria\_none;Pseudomonadota;Betaproteobacteria;Neisseriales;Neisseriaceae;Neisseria;Neisseria leonii |
| Neisseria lisongii | 0 | 0 | 1 | 1 | 1 | 0 | 0 | 0 | 0 | 2 | 0 | 0 | 0 | 0 | Bacteria | Bacteria\_none | Pseudomonadota | Betaproteobacteria | Neisseriales | Neisseriaceae | Neisseria | Bacteria;Bacteria\_none;Pseudomonadota;Betaproteobacteria;Neisseriales;Neisseriaceae;Neisseria;Neisseria lisongii |
| Neisseria macacae | 1 | 3 | 4 | 24 | 3 | 0 | 0 | 2 | 0 | 2 | 5 | 5 | 5 | 3 | Bacteria | Bacteria\_none | Pseudomonadota | Betaproteobacteria | Neisseriales | Neisseriaceae | Neisseria | Bacteria;Bacteria\_none;Pseudomonadota;Betaproteobacteria;Neisseriales;Neisseriaceae;Neisseria;Neisseria macacae |
| Neisseria meningitidis | 5 | 5 | 30 | 112 | 4 | 3 | 1 | 0 | 0 | 70 | 0 | 1 | 14 | 5 | Bacteria | Bacteria\_none | Pseudomonadota | Betaproteobacteria | Neisseriales | Neisseriaceae | Neisseria | Bacteria;Bacteria\_none;Pseudomonadota;Betaproteobacteria;Neisseriales;Neisseriaceae;Neisseria;Neisseria meningitidis |
| Neisseria mucosa | 7 | 6 | 110 | 92 | 32 | 21 | 1 | 0 | 0 | 20 | 2 | 12 | 11 | 10 | Bacteria | Bacteria\_none | Pseudomonadota | Betaproteobacteria | Neisseriales | Neisseriaceae | Neisseria | Bacteria;Bacteria\_none;Pseudomonadota;Betaproteobacteria;Neisseriales;Neisseriaceae;Neisseria;Neisseria mucosa |
| Neisseria musculi | 0 | 0 | 1 | 0 | 0 | 0 | 0 | 0 | 0 | 0 | 0 | 0 | 1 | 0 | Bacteria | Bacteria\_none | Pseudomonadota | Betaproteobacteria | Neisseriales | Neisseriaceae | Neisseria | Bacteria;Bacteria\_none;Pseudomonadota;Betaproteobacteria;Neisseriales;Neisseriaceae;Neisseria;Neisseria musculi |
| Neisseria perflava | 8 | 7 | 46 | 70 | 13 | 11 | 0 | 2 | 0 | 201 | 0 | 0 | 17 | 11 | Bacteria | Bacteria\_none | Pseudomonadota | Betaproteobacteria | Neisseriales | Neisseriaceae | Neisseria | Bacteria;Bacteria\_none;Pseudomonadota;Betaproteobacteria;Neisseriales;Neisseriaceae;Neisseria;Neisseria perflava |
| Neisseria shayeganii | 1 | 0 | 1 | 0 | 0 | 0 | 0 | 0 | 0 | 0 | 0 | 0 | 0 | 0 | Bacteria | Bacteria\_none | Pseudomonadota | Betaproteobacteria | Neisseriales | Neisseriaceae | Neisseria | Bacteria;Bacteria\_none;Pseudomonadota;Betaproteobacteria;Neisseriales;Neisseriaceae;Neisseria;Neisseria shayeganii |
| Neisseria sicca | 22 | 18 | 134 | 321 | 10 | 5 | 0 | 6 | 0 | 18 | 3 | 5 | 23 | 11 | Bacteria | Bacteria\_none | Pseudomonadota | Betaproteobacteria | Neisseriales | Neisseriaceae | Neisseria | Bacteria;Bacteria\_none;Pseudomonadota;Betaproteobacteria;Neisseriales;Neisseriaceae;Neisseria;Neisseria sicca |
| Neisseria sp. DTU\_2021\_1001991\_1\_SI\_NGA\_ILE\_055 | 3 | 2 | 63 | 75 | 9 | 36 | 0 | 1 | 0 | 8 | 0 | 0 | 12 | 9 | Bacteria | Bacteria\_none | Pseudomonadota | Betaproteobacteria | Neisseriales | Neisseriaceae | Neisseria | Bacteria;Bacteria\_none;Pseudomonadota;Betaproteobacteria;Neisseriales;Neisseriaceae;Neisseria;Neisseria sp. DTU\_2021\_1001991\_1\_SI\_NGA\_ILE\_055 |
| Neisseria sp. Dent CA1/247 | 0 | 0 | 0 | 0 | 0 | 0 | 0 | 0 | 0 | 0 | 0 | 0 | 1 | 0 | Bacteria | Bacteria\_none | Pseudomonadota | Betaproteobacteria | Neisseriales | Neisseriaceae | Neisseria | Bacteria;Bacteria\_none;Pseudomonadota;Betaproteobacteria;Neisseriales;Neisseriaceae;Neisseria;Neisseria sp. Dent CA1/247 |
| Neisseria sp. KEM232 | 0 | 0 | 0 | 0 | 2 | 3 | 0 | 0 | 0 | 1 | 0 | 0 | 0 | 0 | Bacteria | Bacteria\_none | Pseudomonadota | Betaproteobacteria | Neisseriales | Neisseriaceae | Neisseria | Bacteria;Bacteria\_none;Pseudomonadota;Betaproteobacteria;Neisseriales;Neisseriaceae;Neisseria;Neisseria sp. KEM232 |
| Neisseria sp. Marseille-Q5346 | 23 | 4 | 45 | 108 | 9 | 4 | 4 | 4 | 0 | 14 | 0 | 0 | 14 | 12 | Bacteria | Bacteria\_none | Pseudomonadota | Betaproteobacteria | Neisseriales | Neisseriaceae | Neisseria | Bacteria;Bacteria\_none;Pseudomonadota;Betaproteobacteria;Neisseriales;Neisseriaceae;Neisseria;Neisseria sp. Marseille-Q5346 |
| Neisseria sp. Marseille-Q6792 | 1 | 0 | 1 | 14 | 1 | 0 | 0 | 0 | 0 | 19 | 0 | 0 | 3 | 2 | Bacteria | Bacteria\_none | Pseudomonadota | Betaproteobacteria | Neisseriales | Neisseriaceae | Neisseria | Bacteria;Bacteria\_none;Pseudomonadota;Betaproteobacteria;Neisseriales;Neisseriaceae;Neisseria;Neisseria sp. Marseille-Q6792 |
| Neisseria sp. RH3002v2g | 9 | 2 | 72 | 53 | 3 | 37 | 1 | 0 | 0 | 9 | 0 | 0 | 5 | 4 | Bacteria | Bacteria\_none | Pseudomonadota | Betaproteobacteria | Neisseriales | Neisseriaceae | Neisseria | Bacteria;Bacteria\_none;Pseudomonadota;Betaproteobacteria;Neisseriales;Neisseriaceae;Neisseria;Neisseria sp. RH3002v2g |
| Neisseria sp. oral taxon 014 | 1 | 0 | 14 | 17 | 0 | 0 | 2 | 6 | 0 | 5 | 73 | 1 | 6 | 5 | Bacteria | Bacteria\_none | Pseudomonadota | Betaproteobacteria | Neisseriales | Neisseriaceae | Neisseria | Bacteria;Bacteria\_none;Pseudomonadota;Betaproteobacteria;Neisseriales;Neisseriaceae;Neisseria;Neisseria sp. oral taxon 014 |
| Neisseria subflava | 88 | 79 | 339 | 1001 | 90 | 89 | 7 | 17 | 1 | 218 | 3 | 20 | 113 | 62 | Bacteria | Bacteria\_none | Pseudomonadota | Betaproteobacteria | Neisseriales | Neisseriaceae | Neisseria | Bacteria;Bacteria\_none;Pseudomonadota;Betaproteobacteria;Neisseriales;Neisseriaceae;Neisseria;Neisseria subflava |
| Neisseria wadsworthii | 0 | 0 | 0 | 0 | 0 | 0 | 0 | 0 | 0 | 0 | 0 | 0 | 0 | 0 | Bacteria | Bacteria\_none | Pseudomonadota | Betaproteobacteria | Neisseriales | Neisseriaceae | Neisseria | Bacteria;Bacteria\_none;Pseudomonadota;Betaproteobacteria;Neisseriales;Neisseriaceae;Neisseria;Neisseria wadsworthii |
| Neisseria weaveri | 0 | 0 | 0 | 0 | 0 | 0 | 0 | 0 | 0 | 1 | 0 | 0 | 0 | 0 | Bacteria | Bacteria\_none | Pseudomonadota | Betaproteobacteria | Neisseriales | Neisseriaceae | Neisseria | Bacteria;Bacteria\_none;Pseudomonadota;Betaproteobacteria;Neisseriales;Neisseriaceae;Neisseria;Neisseria weaveri |
| Neisseria weixii | 0 | 0 | 0 | 2 | 0 | 0 | 0 | 0 | 0 | 1 | 0 | 0 | 0 | 0 | Bacteria | Bacteria\_none | Pseudomonadota | Betaproteobacteria | Neisseriales | Neisseriaceae | Neisseria | Bacteria;Bacteria\_none;Pseudomonadota;Betaproteobacteria;Neisseriales;Neisseriaceae;Neisseria;Neisseria weixii |
| Neisseria yangbaofengii | 0 | 1 | 1 | 4 | 0 | 1 | 0 | 0 | 0 | 0 | 0 | 0 | 0 | 0 | Bacteria | Bacteria\_none | Pseudomonadota | Betaproteobacteria | Neisseriales | Neisseriaceae | Neisseria | Bacteria;Bacteria\_none;Pseudomonadota;Betaproteobacteria;Neisseriales;Neisseriaceae;Neisseria;Neisseria yangbaofengii |
| Neisseria zalophi | 0 | 0 | 0 | 1 | 0 | 0 | 0 | 0 | 0 | 0 | 0 | 0 | 0 | 0 | Bacteria | Bacteria\_none | Pseudomonadota | Betaproteobacteria | Neisseriales | Neisseriaceae | Neisseria | Bacteria;Bacteria\_none;Pseudomonadota;Betaproteobacteria;Neisseriales;Neisseriaceae;Neisseria;Neisseria zalophi |
| Neisseria zoodegmatis | 0 | 0 | 0 | 2 | 0 | 0 | 0 | 0 | 0 | 0 | 0 | 0 | 0 | 0 | Bacteria | Bacteria\_none | Pseudomonadota | Betaproteobacteria | Neisseriales | Neisseriaceae | Neisseria | Bacteria;Bacteria\_none;Pseudomonadota;Betaproteobacteria;Neisseriales;Neisseriaceae;Neisseria;Neisseria zoodegmatis |
| Simonsiella muelleri | 0 | 0 | 0 | 1 | 0 | 0 | 0 | 0 | 0 | 0 | 0 | 0 | 0 | 0 | Bacteria | Bacteria\_none | Pseudomonadota | Betaproteobacteria | Neisseriales | Neisseriaceae | Simonsiella | Bacteria;Bacteria\_none;Pseudomonadota;Betaproteobacteria;Neisseriales;Neisseriaceae;Simonsiella;Simonsiella muelleri |
| Snodgrassella alvi | 0 | 0 | 0 | 0 | 0 | 1 | 0 | 0 | 0 | 0 | 0 | 0 | 0 | 0 | Bacteria | Bacteria\_none | Pseudomonadota | Betaproteobacteria | Neisseriales | Neisseriaceae | Snodgrassella | Bacteria;Bacteria\_none;Pseudomonadota;Betaproteobacteria;Neisseriales;Neisseriaceae;Snodgrassella;Snodgrassella alvi |
| Uruburuella testudinis | 0 | 0 | 0 | 0 | 0 | 0 | 0 | 0 | 0 | 0 | 0 | 0 | 0 | 0 | Bacteria | Bacteria\_none | Pseudomonadota | Betaproteobacteria | Neisseriales | Neisseriaceae | Uruburuella | Bacteria;Bacteria\_none;Pseudomonadota;Betaproteobacteria;Neisseriales;Neisseriaceae;Uruburuella;Uruburuella testudinis |
| Ferriphaselus amnicola | 0 | 0 | 0 | 0 | 0 | 0 | 0 | 0 | 0 | 0 | 0 | 0 | 0 | 0 | Bacteria | Bacteria\_none | Pseudomonadota | Betaproteobacteria | Nitrosomonadales | Gallionellaceae | Ferriphaselus | Bacteria;Bacteria\_none;Pseudomonadota;Betaproteobacteria;Nitrosomonadales;Gallionellaceae;Ferriphaselus;Ferriphaselus amnicola |
| Methylophilus sp. DW102 | 0 | 0 | 0 | 0 | 0 | 0 | 0 | 0 | 0 | 0 | 0 | 0 | 0 | 0 | Bacteria | Bacteria\_none | Pseudomonadota | Betaproteobacteria | Nitrosomonadales | Methylophilaceae | Methylophilus | Bacteria;Bacteria\_none;Pseudomonadota;Betaproteobacteria;Nitrosomonadales;Methylophilaceae;Methylophilus;Methylophilus sp. DW102 |
| Dechloromonas sp. A34 | 0 | 0 | 0 | 0 | 0 | 0 | 0 | 0 | 0 | 0 | 0 | 0 | 0 | 0 | Bacteria | Bacteria\_none | Pseudomonadota | Betaproteobacteria | Rhodocyclales | Azonexaceae | Dechloromonas | Bacteria;Bacteria\_none;Pseudomonadota;Betaproteobacteria;Rhodocyclales;Azonexaceae;Dechloromonas;Dechloromonas sp. A34 |
| Aeromonas veronii | 0 | 0 | 0 | 0 | 0 | 0 | 0 | 0 | 0 | 0 | 0 | 0 | 0 | 0 | Bacteria | Bacteria\_none | Pseudomonadota | Gammaproteobacteria | Aeromonadales | Aeromonadaceae | Aeromonas | Bacteria;Bacteria\_none;Pseudomonadota;Gammaproteobacteria;Aeromonadales;Aeromonadaceae;Aeromonas;Aeromonas veronii |
| Oceanisphaera avium | 0 | 0 | 0 | 0 | 0 | 0 | 0 | 0 | 0 | 0 | 0 | 0 | 0 | 1 | Bacteria | Bacteria\_none | Pseudomonadota | Gammaproteobacteria | Aeromonadales | Aeromonadaceae | Oceanisphaera | Bacteria;Bacteria\_none;Pseudomonadota;Gammaproteobacteria;Aeromonadales;Aeromonadaceae;Oceanisphaera;Oceanisphaera avium |
| Tolumonas auensis | 0 | 0 | 0 | 0 | 0 | 0 | 0 | 0 | 0 | 0 | 0 | 0 | 0 | 0 | Bacteria | Bacteria\_none | Pseudomonadota | Gammaproteobacteria | Aeromonadales | Aeromonadaceae | Tolumonas | Bacteria;Bacteria\_none;Pseudomonadota;Gammaproteobacteria;Aeromonadales;Aeromonadaceae;Tolumonas;Tolumonas auensis |
| Alteromonas macleodii | 0 | 0 | 0 | 0 | 0 | 0 | 0 | 0 | 0 | 0 | 0 | 0 | 0 | 0 | Bacteria | Bacteria\_none | Pseudomonadota | Gammaproteobacteria | Alteromonadales | Alteromonadaceae | Alteromonas | Bacteria;Bacteria\_none;Pseudomonadota;Gammaproteobacteria;Alteromonadales;Alteromonadaceae;Alteromonas;Alteromonas macleodii |
| Alteromonas stellipolaris | 1 | 0 | 0 | 0 | 0 | 0 | 0 | 0 | 0 | 0 | 0 | 0 | 0 | 0 | Bacteria | Bacteria\_none | Pseudomonadota | Gammaproteobacteria | Alteromonadales | Alteromonadaceae | Alteromonas | Bacteria;Bacteria\_none;Pseudomonadota;Gammaproteobacteria;Alteromonadales;Alteromonadaceae;Alteromonas;Alteromonas stellipolaris |
| Thalassotalea piscium | 0 | 0 | 1 | 0 | 0 | 0 | 0 | 0 | 0 | 0 | 0 | 0 | 1 | 0 | Bacteria | Bacteria\_none | Pseudomonadota | Gammaproteobacteria | Alteromonadales | Colwelliaceae | Thalassotalea | Bacteria;Bacteria\_none;Pseudomonadota;Gammaproteobacteria;Alteromonadales;Colwelliaceae;Thalassotalea;Thalassotalea piscium |
| Ferrimonas sp. SCSIO 43195 | 0 | 0 | 0 | 0 | 0 | 0 | 0 | 0 | 0 | 0 | 0 | 0 | 0 | 0 | Bacteria | Bacteria\_none | Pseudomonadota | Gammaproteobacteria | Alteromonadales | Ferrimonadaceae | Ferrimonas | Bacteria;Bacteria\_none;Pseudomonadota;Gammaproteobacteria;Alteromonadales;Ferrimonadaceae;Ferrimonas;Ferrimonas sp. SCSIO 43195 |
| Flocculibacter collagenilyticus | 1 | 0 | 0 | 0 | 0 | 0 | 0 | 0 | 0 | 0 | 0 | 0 | 0 | 0 | Bacteria | Bacteria\_none | Pseudomonadota | Gammaproteobacteria | Alteromonadales | Pseudoalteromonadaceae | Flocculibacter | Bacteria;Bacteria\_none;Pseudomonadota;Gammaproteobacteria;Alteromonadales;Pseudoalteromonadaceae;Flocculibacter;Flocculibacter collagenilyticus |
| Pseudoalteromonas luteoviolacea | 0 | 0 | 0 | 0 | 1 | 0 | 0 | 0 | 0 | 0 | 0 | 0 | 0 | 0 | Bacteria | Bacteria\_none | Pseudomonadota | Gammaproteobacteria | Alteromonadales | Pseudoalteromonadaceae | Pseudoalteromonas | Bacteria;Bacteria\_none;Pseudomonadota;Gammaproteobacteria;Alteromonadales;Pseudoalteromonadaceae;Pseudoalteromonas;Pseudoalteromonas luteoviolacea |
| Pseudoalteromonas piscicida | 0 | 0 | 0 | 0 | 0 | 0 | 0 | 0 | 0 | 0 | 0 | 0 | 0 | 0 | Bacteria | Bacteria\_none | Pseudomonadota | Gammaproteobacteria | Alteromonadales | Pseudoalteromonadaceae | Pseudoalteromonas | Bacteria;Bacteria\_none;Pseudomonadota;Gammaproteobacteria;Alteromonadales;Pseudoalteromonadaceae;Pseudoalteromonas;Pseudoalteromonas piscicida |
| Pseudoalteromonas sp. CuT4-3 | 0 | 0 | 0 | 0 | 0 | 0 | 0 | 0 | 0 | 0 | 0 | 0 | 0 | 0 | Bacteria | Bacteria\_none | Pseudomonadota | Gammaproteobacteria | Alteromonadales | Pseudoalteromonadaceae | Pseudoalteromonas | Bacteria;Bacteria\_none;Pseudomonadota;Gammaproteobacteria;Alteromonadales;Pseudoalteromonadaceae;Pseudoalteromonas;Pseudoalteromonas sp. CuT4-3 |
| Pseudoalteromonas sp. N1230-9 | 0 | 0 | 0 | 0 | 0 | 1 | 0 | 0 | 0 | 0 | 0 | 0 | 0 | 0 | Bacteria | Bacteria\_none | Pseudomonadota | Gammaproteobacteria | Alteromonadales | Pseudoalteromonadaceae | Pseudoalteromonas | Bacteria;Bacteria\_none;Pseudomonadota;Gammaproteobacteria;Alteromonadales;Pseudoalteromonadaceae;Pseudoalteromonas;Pseudoalteromonas sp. N1230-9 |
| Pseudoalteromonas sp. SCSIO 43088 | 0 | 0 | 0 | 0 | 0 | 0 | 0 | 1 | 0 | 0 | 0 | 0 | 0 | 0 | Bacteria | Bacteria\_none | Pseudomonadota | Gammaproteobacteria | Alteromonadales | Pseudoalteromonadaceae | Pseudoalteromonas | Bacteria;Bacteria\_none;Pseudomonadota;Gammaproteobacteria;Alteromonadales;Pseudoalteromonadaceae;Pseudoalteromonas;Pseudoalteromonas sp. SCSIO 43088 |
| Pseudoalteromonas xiamenensis | 0 | 0 | 0 | 0 | 0 | 0 | 0 | 0 | 0 | 0 | 0 | 0 | 0 | 0 | Bacteria | Bacteria\_none | Pseudomonadota | Gammaproteobacteria | Alteromonadales | Pseudoalteromonadaceae | Pseudoalteromonas | Bacteria;Bacteria\_none;Pseudomonadota;Gammaproteobacteria;Alteromonadales;Pseudoalteromonadaceae;Pseudoalteromonas;Pseudoalteromonas xiamenensis |
| Shewanella algae | 1 | 0 | 0 | 0 | 0 | 0 | 0 | 0 | 0 | 0 | 0 | 0 | 0 | 0 | Bacteria | Bacteria\_none | Pseudomonadota | Gammaproteobacteria | Alteromonadales | Shewanellaceae | Shewanella | Bacteria;Bacteria\_none;Pseudomonadota;Gammaproteobacteria;Alteromonadales;Shewanellaceae;Shewanella;Shewanella algae |
| Shewanella dokdonensis | 0 | 0 | 0 | 0 | 0 | 0 | 0 | 0 | 0 | 0 | 0 | 0 | 0 | 0 | Bacteria | Bacteria\_none | Pseudomonadota | Gammaproteobacteria | Alteromonadales | Shewanellaceae | Shewanella | Bacteria;Bacteria\_none;Pseudomonadota;Gammaproteobacteria;Alteromonadales;Shewanellaceae;Shewanella;Shewanella dokdonensis |
| Shewanella psychromarinicola | 0 | 1 | 0 | 0 | 0 | 0 | 0 | 0 | 0 | 0 | 0 | 0 | 0 | 0 | Bacteria | Bacteria\_none | Pseudomonadota | Gammaproteobacteria | Alteromonadales | Shewanellaceae | Shewanella | Bacteria;Bacteria\_none;Pseudomonadota;Gammaproteobacteria;Alteromonadales;Shewanellaceae;Shewanella;Shewanella psychromarinicola |
| Shewanella sp. MEBiC00475 | 0 | 0 | 0 | 0 | 0 | 0 | 0 | 0 | 0 | 0 | 0 | 1 | 0 | 0 | Bacteria | Bacteria\_none | Pseudomonadota | Gammaproteobacteria | Alteromonadales | Shewanellaceae | Shewanella | Bacteria;Bacteria\_none;Pseudomonadota;Gammaproteobacteria;Alteromonadales;Shewanellaceae;Shewanella;Shewanella sp. MEBiC00475 |
| Cardiobacterium hominis | 0 | 2 | 1 | 2 | 1 | 6 | 0 | 0 | 0 | 1 | 4 | 0 | 2 | 0 | Bacteria | Bacteria\_none | Pseudomonadota | Gammaproteobacteria | Cardiobacteriales | Cardiobacteriaceae | Cardiobacterium | Bacteria;Bacteria\_none;Pseudomonadota;Gammaproteobacteria;Cardiobacteriales;Cardiobacteriaceae;Cardiobacterium;Cardiobacterium hominis |
| Cardiobacterium sp. Marseille-Q4385 | 0 | 0 | 12 | 0 | 0 | 0 | 0 | 0 | 0 | 0 | 3 | 2 | 0 | 0 | Bacteria | Bacteria\_none | Pseudomonadota | Gammaproteobacteria | Cardiobacteriales | Cardiobacteriaceae | Cardiobacterium | Bacteria;Bacteria\_none;Pseudomonadota;Gammaproteobacteria;Cardiobacteriales;Cardiobacteriaceae;Cardiobacterium;Cardiobacterium sp. Marseille-Q4385 |
| Ignatzschineria rhizosphaerae | 0 | 1 | 0 | 0 | 0 | 0 | 0 | 0 | 0 | 0 | 0 | 0 | 0 | 0 | Bacteria | Bacteria\_none | Pseudomonadota | Gammaproteobacteria | Cardiobacteriales | Ignatzschineriaceae | Ignatzschineria | Bacteria;Bacteria\_none;Pseudomonadota;Gammaproteobacteria;Cardiobacteriales;Ignatzschineriaceae;Ignatzschineria;Ignatzschineria rhizosphaerae |
| Agarilytica rhodophyticola | 0 | 0 | 0 | 0 | 0 | 0 | 0 | 0 | 0 | 0 | 0 | 0 | 0 | 0 | Bacteria | Bacteria\_none | Pseudomonadota | Gammaproteobacteria | Cellvibrionales | Cellvibrionaceae | Agarilytica | Bacteria;Bacteria\_none;Pseudomonadota;Gammaproteobacteria;Cellvibrionales;Cellvibrionaceae;Agarilytica;Agarilytica rhodophyticola |
| Allochromatium vinosum | 0 | 0 | 0 | 0 | 0 | 0 | 0 | 0 | 0 | 0 | 0 | 0 | 0 | 1 | Bacteria | Bacteria\_none | Pseudomonadota | Gammaproteobacteria | Chromatiales | Chromatiaceae | Allochromatium | Bacteria;Bacteria\_none;Pseudomonadota;Gammaproteobacteria;Chromatiales;Chromatiaceae;Allochromatium;Allochromatium vinosum |
| Candidatus Nitrosoglobus terrae | 0 | 0 | 0 | 0 | 0 | 0 | 0 | 0 | 0 | 0 | 0 | 0 | 0 | 0 | Bacteria | Bacteria\_none | Pseudomonadota | Gammaproteobacteria | Chromatiales | Chromatiaceae | Candidatus Nitrosoglobus | Bacteria;Bacteria\_none;Pseudomonadota;Gammaproteobacteria;Chromatiales;Chromatiaceae;Candidatus Nitrosoglobus;Candidatus Nitrosoglobus terrae |
| Thioalkalivibrio paradoxus | 0 | 0 | 0 | 1 | 0 | 0 | 0 | 0 | 0 | 0 | 0 | 0 | 0 | 0 | Bacteria | Bacteria\_none | Pseudomonadota | Gammaproteobacteria | Chromatiales | Ectothiorhodospiraceae | Thioalkalivibrio | Bacteria;Bacteria\_none;Pseudomonadota;Gammaproteobacteria;Chromatiales;Ectothiorhodospiraceae;Thioalkalivibrio;Thioalkalivibrio paradoxus |
| Limnobaculum zhutongyuii | 0 | 0 | 0 | 0 | 0 | 0 | 0 | 0 | 0 | 0 | 0 | 0 | 0 | 0 | Bacteria | Bacteria\_none | Pseudomonadota | Gammaproteobacteria | Enterobacterales | Budviciaceae | Limnobaculum | Bacteria;Bacteria\_none;Pseudomonadota;Gammaproteobacteria;Enterobacterales;Budviciaceae;Limnobaculum;Limnobaculum zhutongyuii |
| Candidatus Blochmanniella vafra | 0 | 0 | 0 | 0 | 0 | 0 | 0 | 0 | 0 | 1 | 0 | 0 | 0 | 0 | Bacteria | Bacteria\_none | Pseudomonadota | Gammaproteobacteria | Enterobacterales | Enterobacteriaceae | Candidatus Blochmanniella | Bacteria;Bacteria\_none;Pseudomonadota;Gammaproteobacteria;Enterobacterales;Enterobacteriaceae;Candidatus Blochmanniella;Candidatus Blochmanniella vafra |
| Candidatus Profftia sp. (ex Adelges kitamiensis) | 0 | 0 | 0 | 0 | 0 | 0 | 0 | 0 | 0 | 1 | 0 | 0 | 0 | 0 | Bacteria | Bacteria\_none | Pseudomonadota | Gammaproteobacteria | Enterobacterales | Enterobacteriaceae | Candidatus Profftia | Bacteria;Bacteria\_none;Pseudomonadota;Gammaproteobacteria;Enterobacterales;Enterobacteriaceae;Candidatus Profftia;Candidatus Profftia sp. (ex Adelges kitamiensis) |
| Candidatus Purcelliella pentastirinorum | 0 | 0 | 0 | 0 | 0 | 1 | 0 | 0 | 0 | 0 | 0 | 0 | 0 | 0 | Bacteria | Bacteria\_none | Pseudomonadota | Gammaproteobacteria | Enterobacterales | Enterobacteriaceae | Candidatus Purcelliella | Bacteria;Bacteria\_none;Pseudomonadota;Gammaproteobacteria;Enterobacterales;Enterobacteriaceae;Candidatus Purcelliella;Candidatus Purcelliella pentastirinorum |
| Enterobacter cloacae | 0 | 0 | 0 | 0 | 0 | 0 | 0 | 0 | 0 | 1 | 0 | 0 | 0 | 0 | Bacteria | Bacteria\_none | Pseudomonadota | Gammaproteobacteria | Enterobacterales | Enterobacteriaceae | Enterobacter | Bacteria;Bacteria\_none;Pseudomonadota;Gammaproteobacteria;Enterobacterales;Enterobacteriaceae;Enterobacter;Enterobacter cloacae |
| Enterobacteriaceae endosymbiont of Donacia thalassina | 1 | 0 | 0 | 0 | 0 | 0 | 0 | 0 | 0 | 0 | 0 | 0 | 0 | 0 | Bacteria | Bacteria\_none | Pseudomonadota | Gammaproteobacteria | Enterobacterales | Enterobacteriaceae | Enterobacteriaceae\_Incertae\_sedis | Bacteria;Bacteria\_none;Pseudomonadota;Gammaproteobacteria;Enterobacterales;Enterobacteriaceae;Enterobacteriaceae\_Incertae\_sedis;Enterobacteriaceae endosymbiont of Donacia thalassina |
| Escherichia coli | 1 | 0 | 0 | 0 | 0 | 0 | 0 | 0 | 0 | 0 | 0 | 0 | 0 | 1 | Bacteria | Bacteria\_none | Pseudomonadota | Gammaproteobacteria | Enterobacterales | Enterobacteriaceae | Escherichia | Bacteria;Bacteria\_none;Pseudomonadota;Gammaproteobacteria;Enterobacterales;Enterobacteriaceae;Escherichia;Escherichia coli |
| Escherichia marmotae | 0 | 0 | 0 | 0 | 0 | 0 | 0 | 0 | 0 | 0 | 0 | 1 | 0 | 0 | Bacteria | Bacteria\_none | Pseudomonadota | Gammaproteobacteria | Enterobacterales | Enterobacteriaceae | Escherichia | Bacteria;Bacteria\_none;Pseudomonadota;Gammaproteobacteria;Enterobacterales;Enterobacteriaceae;Escherichia;Escherichia marmotae |
| Klebsiella aerogenes | 0 | 0 | 0 | 0 | 0 | 0 | 0 | 0 | 0 | 0 | 0 | 0 | 0 | 0 | Bacteria | Bacteria\_none | Pseudomonadota | Gammaproteobacteria | Enterobacterales | Enterobacteriaceae | Klebsiella | Bacteria;Bacteria\_none;Pseudomonadota;Gammaproteobacteria;Enterobacterales;Enterobacteriaceae;Klebsiella;Klebsiella aerogenes |
| Klebsiella pneumoniae | 0 | 0 | 0 | 2 | 3 | 0 | 0 | 0 | 0 | 0 | 0 | 0 | 0 | 0 | Bacteria | Bacteria\_none | Pseudomonadota | Gammaproteobacteria | Enterobacterales | Enterobacteriaceae | Klebsiella | Bacteria;Bacteria\_none;Pseudomonadota;Gammaproteobacteria;Enterobacterales;Enterobacteriaceae;Klebsiella;Klebsiella pneumoniae |
| Salmonella enterica | 2 | 0 | 0 | 0 | 0 | 0 | 0 | 3 | 0 | 0 | 0 | 0 | 0 | 0 | Bacteria | Bacteria\_none | Pseudomonadota | Gammaproteobacteria | Enterobacterales | Enterobacteriaceae | Salmonella | Bacteria;Bacteria\_none;Pseudomonadota;Gammaproteobacteria;Enterobacterales;Enterobacteriaceae;Salmonella;Salmonella enterica |
| Symbiopectobacterium purcellii | 0 | 0 | 0 | 0 | 0 | 0 | 0 | 0 | 0 | 0 | 0 | 0 | 0 | 0 | Bacteria | Bacteria\_none | Pseudomonadota | Gammaproteobacteria | Enterobacterales | Enterobacteriaceae | Symbiopectobacterium | Bacteria;Bacteria\_none;Pseudomonadota;Gammaproteobacteria;Enterobacterales;Enterobacteriaceae;Symbiopectobacterium;Symbiopectobacterium purcellii |
| Buchnera aphidicola | 0 | 0 | 0 | 0 | 0 | 0 | 0 | 0 | 0 | 0 | 0 | 0 | 0 | 0 | Bacteria | Bacteria\_none | Pseudomonadota | Gammaproteobacteria | Enterobacterales | Erwiniaceae | Buchnera | Bacteria;Bacteria\_none;Pseudomonadota;Gammaproteobacteria;Enterobacterales;Erwiniaceae;Buchnera;Buchnera aphidicola |
| Candidatus Erwinia haradaeae | 0 | 0 | 0 | 0 | 1 | 0 | 0 | 0 | 0 | 0 | 0 | 0 | 0 | 0 | Bacteria | Bacteria\_none | Pseudomonadota | Gammaproteobacteria | Enterobacterales | Erwiniaceae | Erwinia | Bacteria;Bacteria\_none;Pseudomonadota;Gammaproteobacteria;Enterobacterales;Erwiniaceae;Erwinia;Candidatus Erwinia haradaeae |
| Erwinia aphidicola | 0 | 0 | 0 | 0 | 0 | 0 | 0 | 0 | 0 | 0 | 0 | 1 | 0 | 0 | Bacteria | Bacteria\_none | Pseudomonadota | Gammaproteobacteria | Enterobacterales | Erwiniaceae | Erwinia | Bacteria;Bacteria\_none;Pseudomonadota;Gammaproteobacteria;Enterobacterales;Erwiniaceae;Erwinia;Erwinia aphidicola |
| Pantoea vagans | 0 | 0 | 0 | 0 | 0 | 0 | 0 | 0 | 0 | 0 | 1 | 0 | 0 | 0 | Bacteria | Bacteria\_none | Pseudomonadota | Gammaproteobacteria | Enterobacterales | Erwiniaceae | Pantoea | Bacteria;Bacteria\_none;Pseudomonadota;Gammaproteobacteria;Enterobacterales;Erwiniaceae;Pantoea;Pantoea vagans |
| Tatumella ptyseos | 0 | 0 | 0 | 0 | 0 | 0 | 0 | 0 | 0 | 0 | 0 | 0 | 0 | 0 | Bacteria | Bacteria\_none | Pseudomonadota | Gammaproteobacteria | Enterobacterales | Erwiniaceae | Tatumella | Bacteria;Bacteria\_none;Pseudomonadota;Gammaproteobacteria;Enterobacterales;Erwiniaceae;Tatumella;Tatumella ptyseos |
| Arsenophonus nasoniae | 0 | 0 | 0 | 0 | 0 | 0 | 0 | 0 | 0 | 0 | 0 | 0 | 0 | 0 | Bacteria | Bacteria\_none | Pseudomonadota | Gammaproteobacteria | Enterobacterales | Morganellaceae | Arsenophonus | Bacteria;Bacteria\_none;Pseudomonadota;Gammaproteobacteria;Enterobacterales;Morganellaceae;Arsenophonus;Arsenophonus nasoniae |
| Morganella morganii | 0 | 0 | 0 | 0 | 0 | 0 | 0 | 0 | 0 | 0 | 0 | 0 | 0 | 0 | Bacteria | Bacteria\_none | Pseudomonadota | Gammaproteobacteria | Enterobacterales | Morganellaceae | Morganella | Bacteria;Bacteria\_none;Pseudomonadota;Gammaproteobacteria;Enterobacterales;Morganellaceae;Morganella;Morganella morganii |
| Proteus mirabilis | 1 | 0 | 1 | 0 | 0 | 0 | 0 | 1 | 0 | 0 | 0 | 1 | 0 | 0 | Bacteria | Bacteria\_none | Pseudomonadota | Gammaproteobacteria | Enterobacterales | Morganellaceae | Proteus | Bacteria;Bacteria\_none;Pseudomonadota;Gammaproteobacteria;Enterobacterales;Morganellaceae;Proteus;Proteus mirabilis |
| Proteus sp. NMG38-2 | 0 | 0 | 0 | 0 | 0 | 1 | 0 | 0 | 0 | 0 | 0 | 0 | 0 | 0 | Bacteria | Bacteria\_none | Pseudomonadota | Gammaproteobacteria | Enterobacterales | Morganellaceae | Proteus | Bacteria;Bacteria\_none;Pseudomonadota;Gammaproteobacteria;Enterobacterales;Morganellaceae;Proteus;Proteus sp. NMG38-2 |
| Providencia alcalifaciens | 0 | 0 | 0 | 0 | 0 | 1 | 0 | 0 | 0 | 0 | 0 | 0 | 1 | 0 | Bacteria | Bacteria\_none | Pseudomonadota | Gammaproteobacteria | Enterobacterales | Morganellaceae | Providencia | Bacteria;Bacteria\_none;Pseudomonadota;Gammaproteobacteria;Enterobacterales;Morganellaceae;Providencia;Providencia alcalifaciens |
| Providencia manganoxydans | 0 | 0 | 0 | 0 | 0 | 0 | 0 | 0 | 0 | 0 | 0 | 0 | 0 | 0 | Bacteria | Bacteria\_none | Pseudomonadota | Gammaproteobacteria | Enterobacterales | Morganellaceae | Providencia | Bacteria;Bacteria\_none;Pseudomonadota;Gammaproteobacteria;Enterobacterales;Morganellaceae;Providencia;Providencia manganoxydans |
| Xenorhabdus bovienii | 0 | 0 | 0 | 0 | 0 | 0 | 1 | 0 | 0 | 0 | 0 | 0 | 0 | 0 | Bacteria | Bacteria\_none | Pseudomonadota | Gammaproteobacteria | Enterobacterales | Morganellaceae | Xenorhabdus | Bacteria;Bacteria\_none;Pseudomonadota;Gammaproteobacteria;Enterobacterales;Morganellaceae;Xenorhabdus;Xenorhabdus bovienii |
| Xenorhabdus sp. SF857 | 0 | 0 | 0 | 0 | 0 | 0 | 0 | 0 | 0 | 0 | 0 | 0 | 0 | 0 | Bacteria | Bacteria\_none | Pseudomonadota | Gammaproteobacteria | Enterobacterales | Morganellaceae | Xenorhabdus | Bacteria;Bacteria\_none;Pseudomonadota;Gammaproteobacteria;Enterobacterales;Morganellaceae;Xenorhabdus;Xenorhabdus sp. SF857 |
| Dickeya dadantii | 0 | 0 | 0 | 0 | 1 | 0 | 0 | 0 | 0 | 0 | 0 | 0 | 0 | 0 | Bacteria | Bacteria\_none | Pseudomonadota | Gammaproteobacteria | Enterobacterales | Pectobacteriaceae | Dickeya | Bacteria;Bacteria\_none;Pseudomonadota;Gammaproteobacteria;Enterobacterales;Pectobacteriaceae;Dickeya;Dickeya dadantii |
| Serratia marcescens | 0 | 0 | 0 | 0 | 0 | 0 | 0 | 0 | 0 | 0 | 0 | 0 | 0 | 0 | Bacteria | Bacteria\_none | Pseudomonadota | Gammaproteobacteria | Enterobacterales | Yersiniaceae | Serratia | Bacteria;Bacteria\_none;Pseudomonadota;Gammaproteobacteria;Enterobacterales;Yersiniaceae;Serratia;Serratia marcescens |
| Legionella sp. PC1000 | 0 | 0 | 0 | 0 | 0 | 0 | 0 | 0 | 0 | 0 | 0 | 0 | 0 | 0 | Bacteria | Bacteria\_none | Pseudomonadota | Gammaproteobacteria | Legionellales | Legionellaceae | Legionella | Bacteria;Bacteria\_none;Pseudomonadota;Gammaproteobacteria;Legionellales;Legionellaceae;Legionella;Legionella sp. PC1000 |
| Legionella sp. PC997 | 0 | 0 | 0 | 0 | 0 | 0 | 0 | 0 | 0 | 0 | 0 | 0 | 0 | 0 | Bacteria | Bacteria\_none | Pseudomonadota | Gammaproteobacteria | Legionellales | Legionellaceae | Legionella | Bacteria;Bacteria\_none;Pseudomonadota;Gammaproteobacteria;Legionellales;Legionellaceae;Legionella;Legionella sp. PC997 |
| Lysobacter capsici | 0 | 0 | 0 | 0 | 0 | 0 | 0 | 0 | 0 | 0 | 0 | 0 | 0 | 0 | Bacteria | Bacteria\_none | Pseudomonadota | Gammaproteobacteria | Lysobacterales | Lysobacteraceae | Lysobacter | Bacteria;Bacteria\_none;Pseudomonadota;Gammaproteobacteria;Lysobacterales;Lysobacteraceae;Lysobacter;Lysobacter capsici |
| Pseudoxanthomonas daejeonensis | 0 | 0 | 0 | 0 | 0 | 0 | 0 | 0 | 0 | 0 | 1 | 0 | 0 | 0 | Bacteria | Bacteria\_none | Pseudomonadota | Gammaproteobacteria | Lysobacterales | Lysobacteraceae | Pseudoxanthomonas | Bacteria;Bacteria\_none;Pseudomonadota;Gammaproteobacteria;Lysobacterales;Lysobacteraceae;Pseudoxanthomonas;Pseudoxanthomonas daejeonensis |
| Stenotrophomonas maltophilia | 0 | 0 | 0 | 0 | 1 | 0 | 0 | 0 | 0 | 0 | 0 | 0 | 0 | 0 | Bacteria | Bacteria\_none | Pseudomonadota | Gammaproteobacteria | Lysobacterales | Lysobacteraceae | Stenotrophomonas | Bacteria;Bacteria\_none;Pseudomonadota;Gammaproteobacteria;Lysobacterales;Lysobacteraceae;Stenotrophomonas;Stenotrophomonas maltophilia |
| Stenotrophomonas sp. 57 | 0 | 0 | 0 | 0 | 1 | 0 | 0 | 0 | 0 | 0 | 0 | 0 | 0 | 0 | Bacteria | Bacteria\_none | Pseudomonadota | Gammaproteobacteria | Lysobacterales | Lysobacteraceae | Stenotrophomonas | Bacteria;Bacteria\_none;Pseudomonadota;Gammaproteobacteria;Lysobacterales;Lysobacteraceae;Stenotrophomonas;Stenotrophomonas sp. 57 |
| Xanthomonas sp. CFBP 8443 | 0 | 0 | 0 | 0 | 0 | 0 | 0 | 0 | 0 | 0 | 0 | 0 | 0 | 0 | Bacteria | Bacteria\_none | Pseudomonadota | Gammaproteobacteria | Lysobacterales | Lysobacteraceae | Xanthomonas | Bacteria;Bacteria\_none;Pseudomonadota;Gammaproteobacteria;Lysobacterales;Lysobacteraceae;Xanthomonas;Xanthomonas sp. CFBP 8443 |
| Xylella fastidiosa | 0 | 0 | 0 | 0 | 0 | 0 | 0 | 0 | 0 | 0 | 0 | 0 | 0 | 0 | Bacteria | Bacteria\_none | Pseudomonadota | Gammaproteobacteria | Lysobacterales | Lysobacteraceae | Xylella | Bacteria;Bacteria\_none;Pseudomonadota;Gammaproteobacteria;Lysobacterales;Lysobacteraceae;Xylella;Xylella fastidiosa |
| Dyella terrae | 0 | 0 | 0 | 0 | 0 | 0 | 0 | 0 | 0 | 0 | 0 | 1 | 0 | 0 | Bacteria | Bacteria\_none | Pseudomonadota | Gammaproteobacteria | Lysobacterales | Rhodanobacteraceae | Dyella | Bacteria;Bacteria\_none;Pseudomonadota;Gammaproteobacteria;Lysobacterales;Rhodanobacteraceae;Dyella;Dyella terrae |
| Tahibacter amnicola | 0 | 0 | 0 | 0 | 0 | 0 | 0 | 0 | 0 | 1 | 0 | 0 | 0 | 0 | Bacteria | Bacteria\_none | Pseudomonadota | Gammaproteobacteria | Lysobacterales | Rhodanobacteraceae | Tahibacter | Bacteria;Bacteria\_none;Pseudomonadota;Gammaproteobacteria;Lysobacterales;Rhodanobacteraceae;Tahibacter;Tahibacter amnicola |
| Candidatus Methylospira mobilis | 0 | 0 | 0 | 0 | 0 | 0 | 0 | 0 | 0 | 0 | 0 | 0 | 0 | 0 | Bacteria | Bacteria\_none | Pseudomonadota | Gammaproteobacteria | Methylococcales | Methylococcaceae | Candidatus Methylospira | Bacteria;Bacteria\_none;Pseudomonadota;Gammaproteobacteria;Methylococcales;Methylococcaceae;Candidatus Methylospira;Candidatus Methylospira mobilis |
| Methylobacter sp. S3L5C | 0 | 0 | 0 | 0 | 0 | 0 | 0 | 0 | 0 | 0 | 0 | 0 | 0 | 0 | Bacteria | Bacteria\_none | Pseudomonadota | Gammaproteobacteria | Methylococcales | Methylococcaceae | Methylobacter | Bacteria;Bacteria\_none;Pseudomonadota;Gammaproteobacteria;Methylococcales;Methylococcaceae;Methylobacter;Methylobacter sp. S3L5C |
| Methylobacter sp. YRD-M1 | 0 | 0 | 0 | 0 | 0 | 0 | 0 | 0 | 0 | 0 | 0 | 0 | 0 | 0 | Bacteria | Bacteria\_none | Pseudomonadota | Gammaproteobacteria | Methylococcales | Methylococcaceae | Methylobacter | Bacteria;Bacteria\_none;Pseudomonadota;Gammaproteobacteria;Methylococcales;Methylococcaceae;Methylobacter;Methylobacter sp. YRD-M1 |
| Methylomonas denitrificans | 0 | 0 | 0 | 0 | 0 | 0 | 0 | 0 | 0 | 0 | 0 | 0 | 0 | 0 | Bacteria | Bacteria\_none | Pseudomonadota | Gammaproteobacteria | Methylococcales | Methylococcaceae | Methylomonas | Bacteria;Bacteria\_none;Pseudomonadota;Gammaproteobacteria;Methylococcales;Methylococcaceae;Methylomonas;Methylomonas denitrificans |
| Methylotuvimicrobium alcaliphilum | 0 | 0 | 0 | 0 | 0 | 0 | 0 | 0 | 0 | 0 | 0 | 0 | 0 | 0 | Bacteria | Bacteria\_none | Pseudomonadota | Gammaproteobacteria | Methylococcales | Methylococcaceae | Methylotuvimicrobium | Bacteria;Bacteria\_none;Pseudomonadota;Gammaproteobacteria;Methylococcales;Methylococcaceae;Methylotuvimicrobium;Methylotuvimicrobium alcaliphilum |
| Acinetobacter baumannii | 0 | 0 | 0 | 0 | 0 | 0 | 0 | 0 | 0 | 0 | 0 | 0 | 0 | 0 | Bacteria | Bacteria\_none | Pseudomonadota | Gammaproteobacteria | Moraxellales | Moraxellaceae | Acinetobacter | Bacteria;Bacteria\_none;Pseudomonadota;Gammaproteobacteria;Moraxellales;Moraxellaceae;Acinetobacter;Acinetobacter baumannii |
| Acinetobacter chinensis | 0 | 0 | 0 | 0 | 0 | 0 | 0 | 0 | 0 | 0 | 0 | 0 | 0 | 0 | Bacteria | Bacteria\_none | Pseudomonadota | Gammaproteobacteria | Moraxellales | Moraxellaceae | Acinetobacter | Bacteria;Bacteria\_none;Pseudomonadota;Gammaproteobacteria;Moraxellales;Moraxellaceae;Acinetobacter;Acinetobacter chinensis |
| Acinetobacter colistiniresistens | 0 | 0 | 0 | 0 | 0 | 1 | 0 | 1 | 0 | 0 | 0 | 0 | 0 | 0 | Bacteria | Bacteria\_none | Pseudomonadota | Gammaproteobacteria | Moraxellales | Moraxellaceae | Acinetobacter | Bacteria;Bacteria\_none;Pseudomonadota;Gammaproteobacteria;Moraxellales;Moraxellaceae;Acinetobacter;Acinetobacter colistiniresistens |
| Acinetobacter guillouiae | 0 | 0 | 0 | 0 | 0 | 1 | 0 | 0 | 0 | 0 | 0 | 0 | 0 | 0 | Bacteria | Bacteria\_none | Pseudomonadota | Gammaproteobacteria | Moraxellales | Moraxellaceae | Acinetobacter | Bacteria;Bacteria\_none;Pseudomonadota;Gammaproteobacteria;Moraxellales;Moraxellaceae;Acinetobacter;Acinetobacter guillouiae |
| Acinetobacter pittii | 0 | 0 | 0 | 0 | 0 | 0 | 0 | 0 | 0 | 0 | 0 | 0 | 0 | 0 | Bacteria | Bacteria\_none | Pseudomonadota | Gammaproteobacteria | Moraxellales | Moraxellaceae | Acinetobacter | Bacteria;Bacteria\_none;Pseudomonadota;Gammaproteobacteria;Moraxellales;Moraxellaceae;Acinetobacter;Acinetobacter pittii |
| Acinetobacter sp. Z1 | 0 | 0 | 0 | 0 | 0 | 0 | 0 | 0 | 0 | 0 | 0 | 0 | 0 | 0 | Bacteria | Bacteria\_none | Pseudomonadota | Gammaproteobacteria | Moraxellales | Moraxellaceae | Acinetobacter | Bacteria;Bacteria\_none;Pseudomonadota;Gammaproteobacteria;Moraxellales;Moraxellaceae;Acinetobacter;Acinetobacter sp. Z1 |
| Moraxella catarrhalis | 0 | 0 | 0 | 0 | 0 | 0 | 0 | 0 | 0 | 0 | 0 | 0 | 0 | 1 | Bacteria | Bacteria\_none | Pseudomonadota | Gammaproteobacteria | Moraxellales | Moraxellaceae | Moraxella | Bacteria;Bacteria\_none;Pseudomonadota;Gammaproteobacteria;Moraxellales;Moraxellaceae;Moraxella;Moraxella catarrhalis |
| Moraxella ovis | 0 | 0 | 0 | 0 | 0 | 0 | 0 | 0 | 0 | 0 | 0 | 0 | 0 | 1 | Bacteria | Bacteria\_none | Pseudomonadota | Gammaproteobacteria | Moraxellales | Moraxellaceae | Moraxella | Bacteria;Bacteria\_none;Pseudomonadota;Gammaproteobacteria;Moraxellales;Moraxellaceae;Moraxella;Moraxella ovis |
| Psychrobacter raelei | 0 | 0 | 0 | 0 | 0 | 0 | 0 | 0 | 0 | 0 | 0 | 0 | 0 | 0 | Bacteria | Bacteria\_none | Pseudomonadota | Gammaproteobacteria | Moraxellales | Moraxellaceae | Psychrobacter | Bacteria;Bacteria\_none;Pseudomonadota;Gammaproteobacteria;Moraxellales;Moraxellaceae;Psychrobacter;Psychrobacter raelei |
| Psychrobacter sp. 28M-43 | 0 | 0 | 0 | 0 | 0 | 0 | 0 | 0 | 0 | 0 | 0 | 0 | 0 | 0 | Bacteria | Bacteria\_none | Pseudomonadota | Gammaproteobacteria | Moraxellales | Moraxellaceae | Psychrobacter | Bacteria;Bacteria\_none;Pseudomonadota;Gammaproteobacteria;Moraxellales;Moraxellaceae;Psychrobacter;Psychrobacter sp. 28M-43 |
| Psychrobacter sp. van23A | 0 | 0 | 1 | 0 | 0 | 0 | 0 | 0 | 0 | 0 | 0 | 0 | 0 | 0 | Bacteria | Bacteria\_none | Pseudomonadota | Gammaproteobacteria | Moraxellales | Moraxellaceae | Psychrobacter | Bacteria;Bacteria\_none;Pseudomonadota;Gammaproteobacteria;Moraxellales;Moraxellaceae;Psychrobacter;Psychrobacter sp. van23A |
| Alcanivorax sp. | 0 | 0 | 0 | 0 | 0 | 0 | 0 | 0 | 0 | 0 | 0 | 0 | 0 | 0 | Bacteria | Bacteria\_none | Pseudomonadota | Gammaproteobacteria | Oceanospirillales | Alcanivoracaceae | Alcanivorax | Bacteria;Bacteria\_none;Pseudomonadota;Gammaproteobacteria;Oceanospirillales;Alcanivoracaceae;Alcanivorax;Alcanivorax sp. |
| Halomonas meridiana | 0 | 0 | 0 | 0 | 0 | 0 | 0 | 1 | 0 | 0 | 0 | 0 | 0 | 0 | Bacteria | Bacteria\_none | Pseudomonadota | Gammaproteobacteria | Oceanospirillales | Halomonadaceae | Halomonas | Bacteria;Bacteria\_none;Pseudomonadota;Gammaproteobacteria;Oceanospirillales;Halomonadaceae;Halomonas;Halomonas meridiana |
| Halomonas sp. KO116 | 0 | 0 | 0 | 0 | 0 | 1 | 0 | 0 | 0 | 0 | 0 | 0 | 0 | 0 | Bacteria | Bacteria\_none | Pseudomonadota | Gammaproteobacteria | Oceanospirillales | Halomonadaceae | Halomonas | Bacteria;Bacteria\_none;Pseudomonadota;Gammaproteobacteria;Oceanospirillales;Halomonadaceae;Halomonas;Halomonas sp. KO116 |
| Halomonas sp. LR3S48 | 0 | 0 | 0 | 0 | 0 | 0 | 0 | 0 | 0 | 0 | 0 | 0 | 0 | 0 | Bacteria | Bacteria\_none | Pseudomonadota | Gammaproteobacteria | Oceanospirillales | Halomonadaceae | Halomonas | Bacteria;Bacteria\_none;Pseudomonadota;Gammaproteobacteria;Oceanospirillales;Halomonadaceae;Halomonas;Halomonas sp. LR3S48 |
| Amphritea\_uncultured | 0 | 0 | 0 | 0 | 0 | 0 | 0 | 0 | 0 | 0 | 0 | 0 | 0 | 0 | Bacteria | Bacteria\_none | Pseudomonadota | Gammaproteobacteria | Oceanospirillales | Oceanospirillaceae | Amphritea | Bacteria;Bacteria\_none;Pseudomonadota;Gammaproteobacteria;Oceanospirillales;Oceanospirillaceae;Amphritea;Amphritea\_uncultured |
| Marinomonas posidonica | 0 | 0 | 0 | 0 | 0 | 0 | 0 | 0 | 0 | 0 | 0 | 0 | 0 | 1 | Bacteria | Bacteria\_none | Pseudomonadota | Gammaproteobacteria | Oceanospirillales | Oceanospirillaceae | Marinomonas | Bacteria;Bacteria\_none;Pseudomonadota;Gammaproteobacteria;Oceanospirillales;Oceanospirillaceae;Marinomonas;Marinomonas posidonica |
| Marinomonas primoryensis | 0 | 0 | 0 | 0 | 0 | 1 | 0 | 0 | 0 | 0 | 0 | 0 | 0 | 0 | Bacteria | Bacteria\_none | Pseudomonadota | Gammaproteobacteria | Oceanospirillales | Oceanospirillaceae | Marinomonas | Bacteria;Bacteria\_none;Pseudomonadota;Gammaproteobacteria;Oceanospirillales;Oceanospirillaceae;Marinomonas;Marinomonas primoryensis |
| Marinomonas sp. A3A | 0 | 0 | 0 | 0 | 0 | 0 | 0 | 0 | 0 | 0 | 0 | 0 | 0 | 0 | Bacteria | Bacteria\_none | Pseudomonadota | Gammaproteobacteria | Oceanospirillales | Oceanospirillaceae | Marinomonas | Bacteria;Bacteria\_none;Pseudomonadota;Gammaproteobacteria;Oceanospirillales;Oceanospirillaceae;Marinomonas;Marinomonas sp. A3A |
| Thalassolituus hydrocarboniclasticus | 0 | 1 | 0 | 0 | 0 | 0 | 0 | 0 | 0 | 0 | 0 | 0 | 0 | 0 | Bacteria | Bacteria\_none | Pseudomonadota | Gammaproteobacteria | Oceanospirillales | Oceanospirillaceae | Thalassolituus | Bacteria;Bacteria\_none;Pseudomonadota;Gammaproteobacteria;Oceanospirillales;Oceanospirillaceae;Thalassolituus;Thalassolituus hydrocarboniclasticus |
| Gilliamella sp. ESL0443 | 0 | 0 | 0 | 0 | 0 | 0 | 0 | 0 | 0 | 0 | 0 | 0 | 0 | 0 | Bacteria | Bacteria\_none | Pseudomonadota | Gammaproteobacteria | Orbales | Orbaceae | Gilliamella | Bacteria;Bacteria\_none;Pseudomonadota;Gammaproteobacteria;Orbales;Orbaceae;Gilliamella;Gilliamella sp. ESL0443 |
| Gilliamella sp. wkB7 | 0 | 0 | 0 | 0 | 0 | 0 | 0 | 0 | 0 | 0 | 0 | 0 | 0 | 0 | Bacteria | Bacteria\_none | Pseudomonadota | Gammaproteobacteria | Orbales | Orbaceae | Gilliamella | Bacteria;Bacteria\_none;Pseudomonadota;Gammaproteobacteria;Orbales;Orbaceae;Gilliamella;Gilliamella sp. wkB7 |
| Actinobacillus arthritidis | 0 | 0 | 0 | 0 | 0 | 0 | 0 | 0 | 0 | 0 | 0 | 0 | 0 | 0 | Bacteria | Bacteria\_none | Pseudomonadota | Gammaproteobacteria | Pasteurellales | Pasteurellaceae | Actinobacillus | Bacteria;Bacteria\_none;Pseudomonadota;Gammaproteobacteria;Pasteurellales;Pasteurellaceae;Actinobacillus;Actinobacillus arthritidis |
| Actinobacillus equuli | 0 | 0 | 0 | 0 | 0 | 0 | 0 | 7 | 0 | 0 | 0 | 0 | 0 | 0 | Bacteria | Bacteria\_none | Pseudomonadota | Gammaproteobacteria | Pasteurellales | Pasteurellaceae | Actinobacillus | Bacteria;Bacteria\_none;Pseudomonadota;Gammaproteobacteria;Pasteurellales;Pasteurellaceae;Actinobacillus;Actinobacillus equuli |
| Actinobacillus genomosp. 1 | 0 | 0 | 0 | 0 | 0 | 0 | 0 | 0 | 0 | 0 | 0 | 0 | 1 | 0 | Bacteria | Bacteria\_none | Pseudomonadota | Gammaproteobacteria | Pasteurellales | Pasteurellaceae | Actinobacillus | Bacteria;Bacteria\_none;Pseudomonadota;Gammaproteobacteria;Pasteurellales;Pasteurellaceae;Actinobacillus;Actinobacillus genomosp. 1 |
| Actinobacillus genomosp. 2 | 0 | 0 | 0 | 0 | 1 | 0 | 0 | 0 | 0 | 0 | 0 | 0 | 0 | 0 | Bacteria | Bacteria\_none | Pseudomonadota | Gammaproteobacteria | Pasteurellales | Pasteurellaceae | Actinobacillus | Bacteria;Bacteria\_none;Pseudomonadota;Gammaproteobacteria;Pasteurellales;Pasteurellaceae;Actinobacillus;Actinobacillus genomosp. 2 |
| Actinobacillus pleuropneumoniae | 0 | 0 | 0 | 0 | 0 | 0 | 0 | 4 | 0 | 0 | 0 | 0 | 1 | 0 | Bacteria | Bacteria\_none | Pseudomonadota | Gammaproteobacteria | Pasteurellales | Pasteurellaceae | Actinobacillus | Bacteria;Bacteria\_none;Pseudomonadota;Gammaproteobacteria;Pasteurellales;Pasteurellaceae;Actinobacillus;Actinobacillus pleuropneumoniae |
| Actinobacillus porcitonsillarum | 0 | 0 | 0 | 0 | 0 | 0 | 0 | 3 | 0 | 0 | 0 | 0 | 0 | 0 | Bacteria | Bacteria\_none | Pseudomonadota | Gammaproteobacteria | Pasteurellales | Pasteurellaceae | Actinobacillus | Bacteria;Bacteria\_none;Pseudomonadota;Gammaproteobacteria;Pasteurellales;Pasteurellaceae;Actinobacillus;Actinobacillus porcitonsillarum |
| Actinobacillus suis | 0 | 0 | 1 | 0 | 0 | 0 | 0 | 0 | 0 | 0 | 0 | 0 | 0 | 0 | Bacteria | Bacteria\_none | Pseudomonadota | Gammaproteobacteria | Pasteurellales | Pasteurellaceae | Actinobacillus | Bacteria;Bacteria\_none;Pseudomonadota;Gammaproteobacteria;Pasteurellales;Pasteurellaceae;Actinobacillus;Actinobacillus suis |
| Aggregatibacter actinomycetemcomitans | 0 | 2 | 0 | 3 | 0 | 0 | 0 | 3 | 0 | 0 | 0 | 0 | 0 | 0 | Bacteria | Bacteria\_none | Pseudomonadota | Gammaproteobacteria | Pasteurellales | Pasteurellaceae | Aggregatibacter | Bacteria;Bacteria\_none;Pseudomonadota;Gammaproteobacteria;Pasteurellales;Pasteurellaceae;Aggregatibacter;Aggregatibacter actinomycetemcomitans |
| Aggregatibacter aphrophilus | 0 | 14 | 1 | 17 | 50 | 4 | 0 | 73 | 0 | 0 | 0 | 0 | 12 | 6 | Bacteria | Bacteria\_none | Pseudomonadota | Gammaproteobacteria | Pasteurellales | Pasteurellaceae | Aggregatibacter | Bacteria;Bacteria\_none;Pseudomonadota;Gammaproteobacteria;Pasteurellales;Pasteurellaceae;Aggregatibacter;Aggregatibacter aphrophilus |
| Aggregatibacter segnis | 0 | 6 | 1 | 35 | 1 | 2 | 0 | 1 | 0 | 3 | 0 | 0 | 1 | 1 | Bacteria | Bacteria\_none | Pseudomonadota | Gammaproteobacteria | Pasteurellales | Pasteurellaceae | Aggregatibacter | Bacteria;Bacteria\_none;Pseudomonadota;Gammaproteobacteria;Pasteurellales;Pasteurellaceae;Aggregatibacter;Aggregatibacter segnis |
| Aggregatibacter sp. 2125159857 | 0 | 3 | 2 | 15 | 1 | 10 | 0 | 1 | 0 | 2 | 0 | 2 | 1 | 2 | Bacteria | Bacteria\_none | Pseudomonadota | Gammaproteobacteria | Pasteurellales | Pasteurellaceae | Aggregatibacter | Bacteria;Bacteria\_none;Pseudomonadota;Gammaproteobacteria;Pasteurellales;Pasteurellaceae;Aggregatibacter;Aggregatibacter sp. 2125159857 |
| Aggregatibacter sp. Marseille-P9115 | 0 | 6 | 3 | 21 | 2 | 17 | 0 | 1 | 0 | 0 | 0 | 4 | 5 | 3 | Bacteria | Bacteria\_none | Pseudomonadota | Gammaproteobacteria | Pasteurellales | Pasteurellaceae | Aggregatibacter | Bacteria;Bacteria\_none;Pseudomonadota;Gammaproteobacteria;Pasteurellales;Pasteurellaceae;Aggregatibacter;Aggregatibacter sp. Marseille-P9115 |
| Aggregatibacter sp. oral taxon 513 | 0 | 4 | 3 | 22 | 1 | 3 | 0 | 2 | 0 | 0 | 0 | 0 | 5 | 2 | Bacteria | Bacteria\_none | Pseudomonadota | Gammaproteobacteria | Pasteurellales | Pasteurellaceae | Aggregatibacter | Bacteria;Bacteria\_none;Pseudomonadota;Gammaproteobacteria;Pasteurellales;Pasteurellaceae;Aggregatibacter;Aggregatibacter sp. oral taxon 513 |
| Avibacterium paragallinarum | 0 | 0 | 0 | 0 | 0 | 1 | 0 | 0 | 0 | 0 | 0 | 0 | 0 | 0 | Bacteria | Bacteria\_none | Pseudomonadota | Gammaproteobacteria | Pasteurellales | Pasteurellaceae | Avibacterium | Bacteria;Bacteria\_none;Pseudomonadota;Gammaproteobacteria;Pasteurellales;Pasteurellaceae;Avibacterium;Avibacterium paragallinarum |
| Avibacterium volantium | 0 | 0 | 0 | 0 | 0 | 0 | 0 | 0 | 0 | 0 | 0 | 0 | 0 | 0 | Bacteria | Bacteria\_none | Pseudomonadota | Gammaproteobacteria | Pasteurellales | Pasteurellaceae | Avibacterium | Bacteria;Bacteria\_none;Pseudomonadota;Gammaproteobacteria;Pasteurellales;Pasteurellaceae;Avibacterium;Avibacterium volantium |
| Bibersteinia trehalosi | 0 | 0 | 0 | 0 | 0 | 0 | 0 | 0 | 0 | 0 | 0 | 0 | 0 | 0 | Bacteria | Bacteria\_none | Pseudomonadota | Gammaproteobacteria | Pasteurellales | Pasteurellaceae | Bibersteinia | Bacteria;Bacteria\_none;Pseudomonadota;Gammaproteobacteria;Pasteurellales;Pasteurellaceae;Bibersteinia;Bibersteinia trehalosi |
| Bisgaardia hudsonensis | 0 | 0 | 0 | 0 | 0 | 0 | 0 | 0 | 0 | 0 | 0 | 0 | 0 | 0 | Bacteria | Bacteria\_none | Pseudomonadota | Gammaproteobacteria | Pasteurellales | Pasteurellaceae | Bisgaardia | Bacteria;Bacteria\_none;Pseudomonadota;Gammaproteobacteria;Pasteurellales;Pasteurellaceae;Bisgaardia;Bisgaardia hudsonensis |
| Frederiksenia canicola | 0 | 0 | 0 | 0 | 0 | 0 | 0 | 0 | 0 | 0 | 0 | 0 | 0 | 0 | Bacteria | Bacteria\_none | Pseudomonadota | Gammaproteobacteria | Pasteurellales | Pasteurellaceae | Frederiksenia | Bacteria;Bacteria\_none;Pseudomonadota;Gammaproteobacteria;Pasteurellales;Pasteurellaceae;Frederiksenia;Frederiksenia canicola |
| Gallibacterium anatis | 0 | 0 | 0 | 0 | 0 | 0 | 0 | 0 | 0 | 0 | 0 | 0 | 0 | 0 | Bacteria | Bacteria\_none | Pseudomonadota | Gammaproteobacteria | Pasteurellales | Pasteurellaceae | Gallibacterium | Bacteria;Bacteria\_none;Pseudomonadota;Gammaproteobacteria;Pasteurellales;Pasteurellaceae;Gallibacterium;Gallibacterium anatis |
| Gallibacterium salpingitidis | 0 | 0 | 0 | 0 | 0 | 0 | 0 | 0 | 0 | 0 | 0 | 0 | 0 | 0 | Bacteria | Bacteria\_none | Pseudomonadota | Gammaproteobacteria | Pasteurellales | Pasteurellaceae | Gallibacterium | Bacteria;Bacteria\_none;Pseudomonadota;Gammaproteobacteria;Pasteurellales;Pasteurellaceae;Gallibacterium;Gallibacterium salpingitidis |
| Glaesserella parasuis | 0 | 1 | 0 | 0 | 0 | 0 | 0 | 0 | 0 | 0 | 0 | 0 | 0 | 0 | Bacteria | Bacteria\_none | Pseudomonadota | Gammaproteobacteria | Pasteurellales | Pasteurellaceae | Glaesserella | Bacteria;Bacteria\_none;Pseudomonadota;Gammaproteobacteria;Pasteurellales;Pasteurellaceae;Glaesserella;Glaesserella parasuis |
| Glaesserella sp. 15-184 | 0 | 0 | 0 | 0 | 0 | 0 | 0 | 0 | 0 | 0 | 0 | 0 | 0 | 0 | Bacteria | Bacteria\_none | Pseudomonadota | Gammaproteobacteria | Pasteurellales | Pasteurellaceae | Glaesserella | Bacteria;Bacteria\_none;Pseudomonadota;Gammaproteobacteria;Pasteurellales;Pasteurellaceae;Glaesserella;Glaesserella sp. 15-184 |
| Haemophilus haemolyticus | 0 | 10 | 0 | 105 | 17 | 6 | 0 | 8 | 0 | 1 | 6 | 0 | 11 | 2 | Bacteria | Bacteria\_none | Pseudomonadota | Gammaproteobacteria | Pasteurellales | Pasteurellaceae | Haemophilus | Bacteria;Bacteria\_none;Pseudomonadota;Gammaproteobacteria;Pasteurellales;Pasteurellaceae;Haemophilus;Haemophilus haemolyticus |
| Haemophilus influenzae | 2 | 8 | 5 | 20 | 3 | 2 | 0 | 6 | 0 | 0 | 1 | 3 | 3 | 4 | Bacteria | Bacteria\_none | Pseudomonadota | Gammaproteobacteria | Pasteurellales | Pasteurellaceae | Haemophilus | Bacteria;Bacteria\_none;Pseudomonadota;Gammaproteobacteria;Pasteurellales;Pasteurellaceae;Haemophilus;Haemophilus influenzae |
| Haemophilus parahaemolyticus | 1 | 0 | 0 | 0 | 0 | 1 | 0 | 6 | 0 | 0 | 0 | 0 | 0 | 0 | Bacteria | Bacteria\_none | Pseudomonadota | Gammaproteobacteria | Pasteurellales | Pasteurellaceae | Haemophilus | Bacteria;Bacteria\_none;Pseudomonadota;Gammaproteobacteria;Pasteurellales;Pasteurellaceae;Haemophilus;Haemophilus parahaemolyticus |
| Haemophilus parainfluenzae | 63 | 82 | 405 | 164 | 95 | 122 | 1 | 295 | 0 | 45 | 11 | 90 | 106 | 71 | Bacteria | Bacteria\_none | Pseudomonadota | Gammaproteobacteria | Pasteurellales | Pasteurellaceae | Haemophilus | Bacteria;Bacteria\_none;Pseudomonadota;Gammaproteobacteria;Pasteurellales;Pasteurellaceae;Haemophilus;Haemophilus parainfluenzae |
| Haemophilus pittmaniae | 0 | 2 | 0 | 1 | 3 | 3 | 0 | 62 | 0 | 0 | 0 | 0 | 12 | 6 | Bacteria | Bacteria\_none | Pseudomonadota | Gammaproteobacteria | Pasteurellales | Pasteurellaceae | Haemophilus | Bacteria;Bacteria\_none;Pseudomonadota;Gammaproteobacteria;Pasteurellales;Pasteurellaceae;Haemophilus;Haemophilus pittmaniae |
| Haemophilus sp. oral taxon 036 | 0 | 0 | 0 | 5 | 3 | 0 | 0 | 2 | 0 | 0 | 0 | 0 | 0 | 0 | Bacteria | Bacteria\_none | Pseudomonadota | Gammaproteobacteria | Pasteurellales | Pasteurellaceae | Haemophilus | Bacteria;Bacteria\_none;Pseudomonadota;Gammaproteobacteria;Pasteurellales;Pasteurellaceae;Haemophilus;Haemophilus sp. oral taxon 036 |
| [Haemophilus] ducreyi | 0 | 0 | 0 | 0 | 1 | 1 | 0 | 0 | 0 | 0 | 0 | 0 | 0 | 0 | Bacteria | Bacteria\_none | Pseudomonadota | Gammaproteobacteria | Pasteurellales | Pasteurellaceae | Haemophilus | Bacteria;Bacteria\_none;Pseudomonadota;Gammaproteobacteria;Pasteurellales;Pasteurellaceae;Haemophilus;[Haemophilus] ducreyi |
| Histophilus somni | 0 | 0 | 0 | 0 | 0 | 0 | 0 | 0 | 0 | 0 | 0 | 0 | 0 | 0 | Bacteria | Bacteria\_none | Pseudomonadota | Gammaproteobacteria | Pasteurellales | Pasteurellaceae | Histophilus | Bacteria;Bacteria\_none;Pseudomonadota;Gammaproteobacteria;Pasteurellales;Pasteurellaceae;Histophilus;Histophilus somni |
| Mannheimia bovis | 0 | 0 | 0 | 0 | 0 | 0 | 0 | 0 | 0 | 0 | 0 | 0 | 1 | 0 | Bacteria | Bacteria\_none | Pseudomonadota | Gammaproteobacteria | Pasteurellales | Pasteurellaceae | Mannheimia | Bacteria;Bacteria\_none;Pseudomonadota;Gammaproteobacteria;Pasteurellales;Pasteurellaceae;Mannheimia;Mannheimia bovis |
| Mannheimia granulomatis | 0 | 0 | 0 | 0 | 1 | 0 | 0 | 1 | 0 | 0 | 0 | 0 | 0 | 0 | Bacteria | Bacteria\_none | Pseudomonadota | Gammaproteobacteria | Pasteurellales | Pasteurellaceae | Mannheimia | Bacteria;Bacteria\_none;Pseudomonadota;Gammaproteobacteria;Pasteurellales;Pasteurellaceae;Mannheimia;Mannheimia granulomatis |
| Mannheimia haemolytica | 0 | 0 | 0 | 0 | 0 | 0 | 0 | 0 | 0 | 0 | 0 | 0 | 0 | 0 | Bacteria | Bacteria\_none | Pseudomonadota | Gammaproteobacteria | Pasteurellales | Pasteurellaceae | Mannheimia | Bacteria;Bacteria\_none;Pseudomonadota;Gammaproteobacteria;Pasteurellales;Pasteurellaceae;Mannheimia;Mannheimia haemolytica |
| Mannheimia pernigra | 0 | 0 | 0 | 0 | 0 | 0 | 0 | 2 | 0 | 0 | 0 | 0 | 0 | 0 | Bacteria | Bacteria\_none | Pseudomonadota | Gammaproteobacteria | Pasteurellales | Pasteurellaceae | Mannheimia | Bacteria;Bacteria\_none;Pseudomonadota;Gammaproteobacteria;Pasteurellales;Pasteurellaceae;Mannheimia;Mannheimia pernigra |
| Mannheimia sp. USDA-ARS-USMARC-1261 | 0 | 0 | 0 | 0 | 0 | 0 | 0 | 0 | 0 | 0 | 0 | 0 | 0 | 0 | Bacteria | Bacteria\_none | Pseudomonadota | Gammaproteobacteria | Pasteurellales | Pasteurellaceae | Mannheimia | Bacteria;Bacteria\_none;Pseudomonadota;Gammaproteobacteria;Pasteurellales;Pasteurellaceae;Mannheimia;Mannheimia sp. USDA-ARS-USMARC-1261 |
| Mannheimia varigena | 0 | 0 | 0 | 0 | 0 | 0 | 0 | 1 | 0 | 0 | 0 | 0 | 0 | 0 | Bacteria | Bacteria\_none | Pseudomonadota | Gammaproteobacteria | Pasteurellales | Pasteurellaceae | Mannheimia | Bacteria;Bacteria\_none;Pseudomonadota;Gammaproteobacteria;Pasteurellales;Pasteurellaceae;Mannheimia;Mannheimia varigena |
| Mergibacter septicus | 0 | 0 | 0 | 0 | 0 | 0 | 0 | 0 | 0 | 0 | 0 | 0 | 0 | 0 | Bacteria | Bacteria\_none | Pseudomonadota | Gammaproteobacteria | Pasteurellales | Pasteurellaceae | Mergibacter | Bacteria;Bacteria\_none;Pseudomonadota;Gammaproteobacteria;Pasteurellales;Pasteurellaceae;Mergibacter;Mergibacter septicus |
| Otariodibacter oris | 0 | 0 | 0 | 0 | 0 | 0 | 0 | 0 | 0 | 0 | 0 | 0 | 0 | 0 | Bacteria | Bacteria\_none | Pseudomonadota | Gammaproteobacteria | Pasteurellales | Pasteurellaceae | Otariodibacter | Bacteria;Bacteria\_none;Pseudomonadota;Gammaproteobacteria;Pasteurellales;Pasteurellaceae;Otariodibacter;Otariodibacter oris |
| Pasteurella atlantica | 0 | 0 | 0 | 0 | 0 | 0 | 0 | 0 | 0 | 0 | 0 | 0 | 0 | 0 | Bacteria | Bacteria\_none | Pseudomonadota | Gammaproteobacteria | Pasteurellales | Pasteurellaceae | Pasteurella | Bacteria;Bacteria\_none;Pseudomonadota;Gammaproteobacteria;Pasteurellales;Pasteurellaceae;Pasteurella;Pasteurella atlantica |
| Pasteurella canis | 0 | 0 | 0 | 1 | 0 | 0 | 0 | 2 | 0 | 0 | 0 | 0 | 0 | 0 | Bacteria | Bacteria\_none | Pseudomonadota | Gammaproteobacteria | Pasteurellales | Pasteurellaceae | Pasteurella | Bacteria;Bacteria\_none;Pseudomonadota;Gammaproteobacteria;Pasteurellales;Pasteurellaceae;Pasteurella;Pasteurella canis |
| Pasteurella dagmatis | 0 | 0 | 0 | 0 | 0 | 0 | 0 | 0 | 0 | 0 | 0 | 0 | 0 | 0 | Bacteria | Bacteria\_none | Pseudomonadota | Gammaproteobacteria | Pasteurellales | Pasteurellaceae | Pasteurella | Bacteria;Bacteria\_none;Pseudomonadota;Gammaproteobacteria;Pasteurellales;Pasteurellaceae;Pasteurella;Pasteurella dagmatis |
| Pasteurella multocida | 0 | 0 | 1 | 0 | 0 | 1 | 0 | 2 | 0 | 1 | 0 | 0 | 0 | 0 | Bacteria | Bacteria\_none | Pseudomonadota | Gammaproteobacteria | Pasteurellales | Pasteurellaceae | Pasteurella | Bacteria;Bacteria\_none;Pseudomonadota;Gammaproteobacteria;Pasteurellales;Pasteurellaceae;Pasteurella;Pasteurella multocida |
| Pasteurella skyensis | 0 | 0 | 0 | 0 | 0 | 0 | 0 | 0 | 0 | 0 | 0 | 0 | 0 | 0 | Bacteria | Bacteria\_none | Pseudomonadota | Gammaproteobacteria | Pasteurellales | Pasteurellaceae | Pasteurella | Bacteria;Bacteria\_none;Pseudomonadota;Gammaproteobacteria;Pasteurellales;Pasteurellaceae;Pasteurella;Pasteurella skyensis |
| Rodentibacter caecimuris | 0 | 0 | 0 | 0 | 0 | 0 | 0 | 2 | 0 | 0 | 0 | 0 | 0 | 0 | Bacteria | Bacteria\_none | Pseudomonadota | Gammaproteobacteria | Pasteurellales | Pasteurellaceae | Rodentibacter | Bacteria;Bacteria\_none;Pseudomonadota;Gammaproteobacteria;Pasteurellales;Pasteurellaceae;Rodentibacter;Rodentibacter caecimuris |
| Vespertiliibacter pulmonis | 0 | 0 | 0 | 0 | 0 | 0 | 0 | 0 | 0 | 0 | 0 | 0 | 0 | 0 | Bacteria | Bacteria\_none | Pseudomonadota | Gammaproteobacteria | Pasteurellales | Pasteurellaceae | Vespertiliibacter | Bacteria;Bacteria\_none;Pseudomonadota;Gammaproteobacteria;Pasteurellales;Pasteurellaceae;Vespertiliibacter;Vespertiliibacter pulmonis |
| Pseudomonas agarici | 0 | 0 | 0 | 0 | 0 | 0 | 0 | 0 | 0 | 0 | 0 | 0 | 0 | 0 | Bacteria | Bacteria\_none | Pseudomonadota | Gammaproteobacteria | Pseudomonadales | Pseudomonadaceae | Pseudomonas | Bacteria;Bacteria\_none;Pseudomonadota;Gammaproteobacteria;Pseudomonadales;Pseudomonadaceae;Pseudomonas;Pseudomonas agarici |
| Pseudomonas asturiensis | 0 | 0 | 0 | 0 | 0 | 1 | 0 | 0 | 0 | 0 | 0 | 0 | 0 | 0 | Bacteria | Bacteria\_none | Pseudomonadota | Gammaproteobacteria | Pseudomonadales | Pseudomonadaceae | Pseudomonas | Bacteria;Bacteria\_none;Pseudomonadota;Gammaproteobacteria;Pseudomonadales;Pseudomonadaceae;Pseudomonas;Pseudomonas asturiensis |
| Pseudomonas canadensis | 0 | 0 | 0 | 0 | 0 | 1 | 0 | 0 | 0 | 0 | 0 | 0 | 0 | 0 | Bacteria | Bacteria\_none | Pseudomonadota | Gammaproteobacteria | Pseudomonadales | Pseudomonadaceae | Pseudomonas | Bacteria;Bacteria\_none;Pseudomonadota;Gammaproteobacteria;Pseudomonadales;Pseudomonadaceae;Pseudomonas;Pseudomonas canadensis |
| Pseudomonas citronellolis | 0 | 3 | 0 | 0 | 0 | 0 | 0 | 0 | 0 | 0 | 0 | 0 | 0 | 0 | Bacteria | Bacteria\_none | Pseudomonadota | Gammaproteobacteria | Pseudomonadales | Pseudomonadaceae | Pseudomonas | Bacteria;Bacteria\_none;Pseudomonadota;Gammaproteobacteria;Pseudomonadales;Pseudomonadaceae;Pseudomonas;Pseudomonas citronellolis |
| Pseudomonas frederiksbergensis | 0 | 0 | 0 | 0 | 0 | 0 | 0 | 0 | 0 | 0 | 0 | 0 | 0 | 0 | Bacteria | Bacteria\_none | Pseudomonadota | Gammaproteobacteria | Pseudomonadales | Pseudomonadaceae | Pseudomonas | Bacteria;Bacteria\_none;Pseudomonadota;Gammaproteobacteria;Pseudomonadales;Pseudomonadaceae;Pseudomonas;Pseudomonas frederiksbergensis |
| Pseudomonas guguanensis | 0 | 0 | 0 | 0 | 0 | 0 | 0 | 0 | 0 | 0 | 0 | 0 | 0 | 1 | Bacteria | Bacteria\_none | Pseudomonadota | Gammaproteobacteria | Pseudomonadales | Pseudomonadaceae | Pseudomonas | Bacteria;Bacteria\_none;Pseudomonadota;Gammaproteobacteria;Pseudomonadales;Pseudomonadaceae;Pseudomonas;Pseudomonas guguanensis |
| Pseudomonas hydrolytica | 0 | 0 | 0 | 0 | 0 | 0 | 0 | 0 | 0 | 0 | 0 | 0 | 0 | 0 | Bacteria | Bacteria\_none | Pseudomonadota | Gammaproteobacteria | Pseudomonadales | Pseudomonadaceae | Pseudomonas | Bacteria;Bacteria\_none;Pseudomonadota;Gammaproteobacteria;Pseudomonadales;Pseudomonadaceae;Pseudomonas;Pseudomonas hydrolytica |
| Pseudomonas psychrophila | 0 | 0 | 0 | 0 | 0 | 0 | 0 | 0 | 0 | 0 | 0 | 0 | 0 | 0 | Bacteria | Bacteria\_none | Pseudomonadota | Gammaproteobacteria | Pseudomonadales | Pseudomonadaceae | Pseudomonas | Bacteria;Bacteria\_none;Pseudomonadota;Gammaproteobacteria;Pseudomonadales;Pseudomonadaceae;Pseudomonas;Pseudomonas psychrophila |
| Pseudomonas putida | 0 | 0 | 0 | 0 | 0 | 0 | 1 | 0 | 0 | 0 | 0 | 0 | 0 | 0 | Bacteria | Bacteria\_none | Pseudomonadota | Gammaproteobacteria | Pseudomonadales | Pseudomonadaceae | Pseudomonas | Bacteria;Bacteria\_none;Pseudomonadota;Gammaproteobacteria;Pseudomonadales;Pseudomonadaceae;Pseudomonas;Pseudomonas putida |
| Pseudomonas triclosanedens | 0 | 0 | 0 | 0 | 1 | 0 | 0 | 0 | 0 | 0 | 0 | 0 | 0 | 0 | Bacteria | Bacteria\_none | Pseudomonadota | Gammaproteobacteria | Pseudomonadales | Pseudomonadaceae | Pseudomonas | Bacteria;Bacteria\_none;Pseudomonadota;Gammaproteobacteria;Pseudomonadales;Pseudomonadaceae;Pseudomonas;Pseudomonas triclosanedens |
| Thiomicrorhabdus indica | 0 | 0 | 0 | 0 | 0 | 0 | 0 | 0 | 0 | 0 | 0 | 0 | 0 | 0 | Bacteria | Bacteria\_none | Pseudomonadota | Gammaproteobacteria | Thiotrichales | Piscirickettsiaceae | Thiomicrorhabdus | Bacteria;Bacteria\_none;Pseudomonadota;Gammaproteobacteria;Thiotrichales;Piscirickettsiaceae;Thiomicrorhabdus;Thiomicrorhabdus indica |
| Aliivibrio salmonicida | 0 | 0 | 0 | 0 | 0 | 0 | 0 | 0 | 0 | 0 | 0 | 0 | 0 | 0 | Bacteria | Bacteria\_none | Pseudomonadota | Gammaproteobacteria | Vibrionales | Vibrionaceae | Aliivibrio | Bacteria;Bacteria\_none;Pseudomonadota;Gammaproteobacteria;Vibrionales;Vibrionaceae;Aliivibrio;Aliivibrio salmonicida |
| Candidatus Enterovibrio luxaltus | 0 | 0 | 0 | 0 | 0 | 0 | 0 | 0 | 0 | 0 | 0 | 0 | 0 | 0 | Bacteria | Bacteria\_none | Pseudomonadota | Gammaproteobacteria | Vibrionales | Vibrionaceae | Enterovibrio | Bacteria;Bacteria\_none;Pseudomonadota;Gammaproteobacteria;Vibrionales;Vibrionaceae;Enterovibrio;Candidatus Enterovibrio luxaltus |
| Thaumasiovibrio subtropicus | 0 | 0 | 0 | 0 | 0 | 0 | 0 | 0 | 0 | 0 | 0 | 0 | 0 | 0 | Bacteria | Bacteria\_none | Pseudomonadota | Gammaproteobacteria | Vibrionales | Vibrionaceae | Thaumasiovibrio | Bacteria;Bacteria\_none;Pseudomonadota;Gammaproteobacteria;Vibrionales;Vibrionaceae;Thaumasiovibrio;Thaumasiovibrio subtropicus |
| Vibrio artabrorum | 0 | 0 | 1 | 0 | 0 | 0 | 0 | 0 | 0 | 0 | 0 | 0 | 1 | 0 | Bacteria | Bacteria\_none | Pseudomonadota | Gammaproteobacteria | Vibrionales | Vibrionaceae | Vibrio | Bacteria;Bacteria\_none;Pseudomonadota;Gammaproteobacteria;Vibrionales;Vibrionaceae;Vibrio;Vibrio artabrorum |
| Vibrio campbellii | 0 | 0 | 0 | 0 | 0 | 0 | 0 | 0 | 0 | 0 | 0 | 0 | 0 | 0 | Bacteria | Bacteria\_none | Pseudomonadota | Gammaproteobacteria | Vibrionales | Vibrionaceae | Vibrio | Bacteria;Bacteria\_none;Pseudomonadota;Gammaproteobacteria;Vibrionales;Vibrionaceae;Vibrio;Vibrio campbellii |
| Vibrio cholerae | 0 | 0 | 2 | 0 | 0 | 0 | 0 | 0 | 0 | 0 | 0 | 0 | 0 | 0 | Bacteria | Bacteria\_none | Pseudomonadota | Gammaproteobacteria | Vibrionales | Vibrionaceae | Vibrio | Bacteria;Bacteria\_none;Pseudomonadota;Gammaproteobacteria;Vibrionales;Vibrionaceae;Vibrio;Vibrio cholerae |
| Vibrio crassostreae | 0 | 0 | 0 | 0 | 0 | 0 | 0 | 0 | 0 | 0 | 0 | 0 | 0 | 0 | Bacteria | Bacteria\_none | Pseudomonadota | Gammaproteobacteria | Vibrionales | Vibrionaceae | Vibrio | Bacteria;Bacteria\_none;Pseudomonadota;Gammaproteobacteria;Vibrionales;Vibrionaceae;Vibrio;Vibrio crassostreae |
| Vibrio gangliei | 0 | 0 | 0 | 0 | 0 | 1 | 0 | 0 | 0 | 0 | 0 | 0 | 0 | 0 | Bacteria | Bacteria\_none | Pseudomonadota | Gammaproteobacteria | Vibrionales | Vibrionaceae | Vibrio | Bacteria;Bacteria\_none;Pseudomonadota;Gammaproteobacteria;Vibrionales;Vibrionaceae;Vibrio;Vibrio gangliei |
| Vibrio gazogenes | 0 | 0 | 0 | 0 | 0 | 0 | 0 | 0 | 0 | 0 | 0 | 0 | 0 | 0 | Bacteria | Bacteria\_none | Pseudomonadota | Gammaproteobacteria | Vibrionales | Vibrionaceae | Vibrio | Bacteria;Bacteria\_none;Pseudomonadota;Gammaproteobacteria;Vibrionales;Vibrionaceae;Vibrio;Vibrio gazogenes |
| Vibrio rumoiensis | 0 | 0 | 0 | 0 | 0 | 0 | 0 | 0 | 0 | 0 | 0 | 0 | 0 | 0 | Bacteria | Bacteria\_none | Pseudomonadota | Gammaproteobacteria | Vibrionales | Vibrionaceae | Vibrio | Bacteria;Bacteria\_none;Pseudomonadota;Gammaproteobacteria;Vibrionales;Vibrionaceae;Vibrio;Vibrio rumoiensis |
| Vibrio sp. VB16 | 0 | 0 | 0 | 0 | 0 | 0 | 0 | 1 | 0 | 0 | 0 | 0 | 0 | 0 | Bacteria | Bacteria\_none | Pseudomonadota | Gammaproteobacteria | Vibrionales | Vibrionaceae | Vibrio | Bacteria;Bacteria\_none;Pseudomonadota;Gammaproteobacteria;Vibrionales;Vibrionaceae;Vibrio;Vibrio sp. VB16 |
| Vibrio zhugei | 0 | 0 | 0 | 0 | 2 | 0 | 0 | 0 | 0 | 0 | 0 | 0 | 1 | 0 | Bacteria | Bacteria\_none | Pseudomonadota | Gammaproteobacteria | Vibrionales | Vibrionaceae | Vibrio | Bacteria;Bacteria\_none;Pseudomonadota;Gammaproteobacteria;Vibrionales;Vibrionaceae;Vibrio;Vibrio zhugei |
| Leptospira santarosai | 1 | 0 | 0 | 0 | 0 | 0 | 0 | 0 | 0 | 0 | 0 | 0 | 0 | 0 | Bacteria | Bacteria\_none | Spirochaetota | Spirochaetia | Leptospirales | Leptospiraceae | Leptospira | Bacteria;Bacteria\_none;Spirochaetota;Spirochaetia;Leptospirales;Leptospiraceae;Leptospira;Leptospira santarosai |
| Borreliella garinii | 0 | 0 | 0 | 0 | 0 | 0 | 0 | 0 | 0 | 0 | 0 | 0 | 0 | 0 | Bacteria | Bacteria\_none | Spirochaetota | Spirochaetia | Spirochaetales | Borreliaceae | Borreliella | Bacteria;Bacteria\_none;Spirochaetota;Spirochaetia;Spirochaetales;Borreliaceae;Borreliella;Borreliella garinii |
| Breznakiella homolactica | 0 | 1 | 0 | 0 | 0 | 0 | 0 | 0 | 0 | 0 | 0 | 0 | 0 | 0 | Bacteria | Bacteria\_none | Spirochaetota | Spirochaetia | Spirochaetales | Breznakiellaceae | Breznakiella | Bacteria;Bacteria\_none;Spirochaetota;Spirochaetia;Spirochaetales;Breznakiellaceae;Breznakiella;Breznakiella homolactica |
| Sphaerochaeta\_uncultured | 0 | 0 | 0 | 0 | 0 | 0 | 0 | 0 | 0 | 0 | 0 | 0 | 0 | 0 | Bacteria | Bacteria\_none | Spirochaetota | Spirochaetia | Spirochaetales | Sphaerochaetaceae | Sphaerochaeta | Bacteria;Bacteria\_none;Spirochaetota;Spirochaetia;Spirochaetales;Sphaerochaetaceae;Sphaerochaeta;Sphaerochaeta\_uncultured |
| Treponema bryantii | 0 | 1 | 0 | 0 | 0 | 0 | 0 | 0 | 0 | 0 | 0 | 0 | 0 | 0 | Bacteria | Bacteria\_none | Spirochaetota | Spirochaetia | Spirochaetales | Treponemataceae | Treponema | Bacteria;Bacteria\_none;Spirochaetota;Spirochaetia;Spirochaetales;Treponemataceae;Treponema;Treponema bryantii |
| Treponema denticola | 0 | 15 | 40 | 1 | 7 | 25 | 0 | 11 | 0 | 4 | 14 | 12 | 14 | 7 | Bacteria | Bacteria\_none | Spirochaetota | Spirochaetia | Spirochaetales | Treponemataceae | Treponema | Bacteria;Bacteria\_none;Spirochaetota;Spirochaetia;Spirochaetales;Treponemataceae;Treponema;Treponema denticola |
| Treponema medium | 0 | 2 | 1 | 1 | 3 | 4 | 0 | 2 | 0 | 0 | 1 | 3 | 0 | 0 | Bacteria | Bacteria\_none | Spirochaetota | Spirochaetia | Spirochaetales | Treponemataceae | Treponema | Bacteria;Bacteria\_none;Spirochaetota;Spirochaetia;Spirochaetales;Treponemataceae;Treponema;Treponema medium |
| Treponema parvum | 0 | 1 | 0 | 0 | 0 | 1 | 0 | 0 | 0 | 0 | 0 | 2 | 0 | 0 | Bacteria | Bacteria\_none | Spirochaetota | Spirochaetia | Spirochaetales | Treponemataceae | Treponema | Bacteria;Bacteria\_none;Spirochaetota;Spirochaetia;Spirochaetales;Treponemataceae;Treponema;Treponema parvum |
| Treponema pedis | 0 | 1 | 0 | 0 | 1 | 1 | 0 | 0 | 0 | 1 | 2 | 0 | 0 | 0 | Bacteria | Bacteria\_none | Spirochaetota | Spirochaetia | Spirochaetales | Treponemataceae | Treponema | Bacteria;Bacteria\_none;Spirochaetota;Spirochaetia;Spirochaetales;Treponemataceae;Treponema;Treponema pedis |
| Treponema phagedenis | 0 | 0 | 0 | 0 | 0 | 1 | 0 | 0 | 0 | 0 | 0 | 0 | 1 | 0 | Bacteria | Bacteria\_none | Spirochaetota | Spirochaetia | Spirochaetales | Treponemataceae | Treponema | Bacteria;Bacteria\_none;Spirochaetota;Spirochaetia;Spirochaetales;Treponemataceae;Treponema;Treponema phagedenis |
| Treponema putidum | 0 | 2 | 1 | 0 | 0 | 1 | 0 | 0 | 0 | 0 | 0 | 0 | 0 | 0 | Bacteria | Bacteria\_none | Spirochaetota | Spirochaetia | Spirochaetales | Treponemataceae | Treponema | Bacteria;Bacteria\_none;Spirochaetota;Spirochaetia;Spirochaetales;Treponemataceae;Treponema;Treponema putidum |
| Treponema ruminis | 0 | 1 | 0 | 0 | 0 | 0 | 0 | 0 | 0 | 0 | 0 | 0 | 0 | 1 | Bacteria | Bacteria\_none | Spirochaetota | Spirochaetia | Spirochaetales | Treponemataceae | Treponema | Bacteria;Bacteria\_none;Spirochaetota;Spirochaetia;Spirochaetales;Treponemataceae;Treponema;Treponema ruminis |
| Treponema saccharophilum | 0 | 0 | 0 | 0 | 0 | 0 | 0 | 0 | 0 | 0 | 0 | 0 | 0 | 0 | Bacteria | Bacteria\_none | Spirochaetota | Spirochaetia | Spirochaetales | Treponemataceae | Treponema | Bacteria;Bacteria\_none;Spirochaetota;Spirochaetia;Spirochaetales;Treponemataceae;Treponema;Treponema saccharophilum |
| Treponema socranskii | 1 | 4 | 3 | 0 | 0 | 0 | 0 | 1 | 0 | 1 | 4 | 3 | 0 | 0 | Bacteria | Bacteria\_none | Spirochaetota | Spirochaetia | Spirochaetales | Treponemataceae | Treponema | Bacteria;Bacteria\_none;Spirochaetota;Spirochaetia;Spirochaetales;Treponemataceae;Treponema;Treponema socranskii |
| Treponema sp. B152 | 0 | 0 | 0 | 0 | 0 | 0 | 0 | 0 | 0 | 0 | 0 | 1 | 0 | 0 | Bacteria | Bacteria\_none | Spirochaetota | Spirochaetia | Spirochaetales | Treponemataceae | Treponema | Bacteria;Bacteria\_none;Spirochaetota;Spirochaetia;Spirochaetales;Treponemataceae;Treponema;Treponema sp. B152 |
| Treponema sp. Marseille-Q4132 | 0 | 1 | 0 | 0 | 0 | 0 | 0 | 1 | 0 | 0 | 0 | 2 | 2 | 0 | Bacteria | Bacteria\_none | Spirochaetota | Spirochaetia | Spirochaetales | Treponemataceae | Treponema | Bacteria;Bacteria\_none;Spirochaetota;Spirochaetia;Spirochaetales;Treponemataceae;Treponema;Treponema sp. Marseille-Q4132 |
| Treponema sp. OMZ 305 | 0 | 2 | 0 | 0 | 0 | 4 | 0 | 1 | 0 | 0 | 0 | 2 | 1 | 0 | Bacteria | Bacteria\_none | Spirochaetota | Spirochaetia | Spirochaetales | Treponemataceae | Treponema | Bacteria;Bacteria\_none;Spirochaetota;Spirochaetia;Spirochaetales;Treponemataceae;Treponema;Treponema sp. OMZ 305 |
| Treponema sp. OMZ 787 | 0 | 0 | 0 | 1 | 0 | 0 | 0 | 0 | 0 | 0 | 0 | 0 | 0 | 1 | Bacteria | Bacteria\_none | Spirochaetota | Spirochaetia | Spirochaetales | Treponemataceae | Treponema | Bacteria;Bacteria\_none;Spirochaetota;Spirochaetia;Spirochaetales;Treponemataceae;Treponema;Treponema sp. OMZ 787 |
| Treponema sp. OMZ 788 | 0 | 1 | 0 | 0 | 0 | 1 | 0 | 0 | 0 | 0 | 0 | 0 | 0 | 0 | Bacteria | Bacteria\_none | Spirochaetota | Spirochaetia | Spirochaetales | Treponemataceae | Treponema | Bacteria;Bacteria\_none;Spirochaetota;Spirochaetia;Spirochaetales;Treponemataceae;Treponema;Treponema sp. OMZ 788 |
| Treponema sp. OMZ 799 | 0 | 0 | 0 | 0 | 0 | 2 | 0 | 0 | 0 | 0 | 0 | 0 | 0 | 0 | Bacteria | Bacteria\_none | Spirochaetota | Spirochaetia | Spirochaetales | Treponemataceae | Treponema | Bacteria;Bacteria\_none;Spirochaetota;Spirochaetia;Spirochaetales;Treponemataceae;Treponema;Treponema sp. OMZ 799 |
| Treponema sp. OMZ 803 | 0 | 1 | 0 | 1 | 0 | 0 | 0 | 0 | 0 | 0 | 1 | 3 | 0 | 2 | Bacteria | Bacteria\_none | Spirochaetota | Spirochaetia | Spirochaetales | Treponemataceae | Treponema | Bacteria;Bacteria\_none;Spirochaetota;Spirochaetia;Spirochaetales;Treponemataceae;Treponema;Treponema sp. OMZ 803 |
| Treponema sp. OMZ 838 | 0 | 0 | 0 | 0 | 0 | 0 | 0 | 1 | 0 | 0 | 1 | 0 | 1 | 2 | Bacteria | Bacteria\_none | Spirochaetota | Spirochaetia | Spirochaetales | Treponemataceae | Treponema | Bacteria;Bacteria\_none;Spirochaetota;Spirochaetia;Spirochaetales;Treponemataceae;Treponema;Treponema sp. OMZ 838 |
| Treponema sp. OMZ 855 | 0 | 2 | 0 | 0 | 0 | 2 | 0 | 1 | 0 | 0 | 0 | 2 | 0 | 0 | Bacteria | Bacteria\_none | Spirochaetota | Spirochaetia | Spirochaetales | Treponemataceae | Treponema | Bacteria;Bacteria\_none;Spirochaetota;Spirochaetia;Spirochaetales;Treponemataceae;Treponema;Treponema sp. OMZ 855 |
| Treponema sp. OMZ 857 | 0 | 11 | 2 | 0 | 1 | 4 | 0 | 1 | 0 | 2 | 0 | 4 | 0 | 0 | Bacteria | Bacteria\_none | Spirochaetota | Spirochaetia | Spirochaetales | Treponemataceae | Treponema | Bacteria;Bacteria\_none;Spirochaetota;Spirochaetia;Spirochaetales;Treponemataceae;Treponema;Treponema sp. OMZ 857 |
| Treponema sp. OMZ 906 | 0 | 6 | 3 | 0 | 5 | 0 | 0 | 2 | 0 | 1 | 1 | 0 | 1 | 0 | Bacteria | Bacteria\_none | Spirochaetota | Spirochaetia | Spirochaetales | Treponemataceae | Treponema | Bacteria;Bacteria\_none;Spirochaetota;Spirochaetia;Spirochaetales;Treponemataceae;Treponema;Treponema sp. OMZ 906 |
| Treponema succinifaciens | 0 | 0 | 0 | 0 | 0 | 0 | 0 | 0 | 0 | 0 | 0 | 1 | 0 | 0 | Bacteria | Bacteria\_none | Spirochaetota | Spirochaetia | Spirochaetales | Treponemataceae | Treponema | Bacteria;Bacteria\_none;Spirochaetota;Spirochaetia;Spirochaetales;Treponemataceae;Treponema;Treponema succinifaciens |
| Treponema vincentii | 1 | 17 | 0 | 1 | 4 | 14 | 0 | 7 | 0 | 1 | 3 | 7 | 2 | 1 | Bacteria | Bacteria\_none | Spirochaetota | Spirochaetia | Spirochaetales | Treponemataceae | Treponema | Bacteria;Bacteria\_none;Spirochaetota;Spirochaetia;Spirochaetales;Treponemataceae;Treponema;Treponema vincentii |
| Fretibacterium fastidiosum | 0 | 0 | 1 | 0 | 0 | 0 | 0 | 0 | 0 | 0 | 1 | 0 | 0 | 0 | Bacteria | Bacteria\_none | Synergistota | Synergistia | Synergistales | Aminobacteriaceae | Fretibacterium | Bacteria;Bacteria\_none;Synergistota;Synergistia;Synergistales;Aminobacteriaceae;Fretibacterium;Fretibacterium fastidiosum |
| Candidatus Desulfofervidus auxilii | 0 | 0 | 0 | 0 | 0 | 1 | 0 | 0 | 0 | 0 | 0 | 0 | 0 | 0 | Bacteria | Bacteria\_none | Thermodesulfobacteriota | Candidatus Desulfofervidia | Candidatus Desulfofervidales | Candidatus Desulfofervidaceae | Candidatus Desulfofervidus | Bacteria;Bacteria\_none;Thermodesulfobacteriota;Candidatus Desulfofervidia;Candidatus Desulfofervidales;Candidatus Desulfofervidaceae;Candidatus Desulfofervidus;Candidatus Desulfofervidus auxilii |
| Desulfoferula mesophila | 0 | 0 | 0 | 0 | 0 | 1 | 0 | 0 | 0 | 0 | 0 | 0 | 0 | 0 | Bacteria | Bacteria\_none | Thermodesulfobacteriota | Desulfarculia | Desulfarculales | Desulfarculaceae | Desulfoferula | Bacteria;Bacteria\_none;Thermodesulfobacteriota;Desulfarculia;Desulfarculales;Desulfarculaceae;Desulfoferula;Desulfoferula mesophila |
| Desulfobulbus oralis | 0 | 1 | 0 | 0 | 0 | 0 | 0 | 1 | 0 | 0 | 0 | 0 | 0 | 0 | Bacteria | Bacteria\_none | Thermodesulfobacteriota | Desulfobulbia | Desulfobulbales | Desulfobulbaceae | Desulfobulbus | Bacteria;Bacteria\_none;Thermodesulfobacteriota;Desulfobulbia;Desulfobulbales;Desulfobulbaceae;Desulfobulbus;Desulfobulbus oralis |
| Lawsonia intracellularis | 0 | 0 | 0 | 0 | 0 | 0 | 0 | 0 | 0 | 0 | 0 | 1 | 0 | 0 | Bacteria | Bacteria\_none | Thermodesulfobacteriota | Desulfovibrionia | Desulfovibrionales | Desulfovibrionaceae | Lawsonia | Bacteria;Bacteria\_none;Thermodesulfobacteriota;Desulfovibrionia;Desulfovibrionales;Desulfovibrionaceae;Lawsonia;Lawsonia intracellularis |
| Syntrophotalea acetylenivorans | 0 | 0 | 0 | 0 | 0 | 0 | 0 | 0 | 0 | 0 | 0 | 0 | 0 | 0 | Bacteria | Bacteria\_none | Thermodesulfobacteriota | Desulfuromonadia | Desulfuromonadales | Syntrophotaleaceae | Syntrophotalea | Bacteria;Bacteria\_none;Thermodesulfobacteriota;Desulfuromonadia;Desulfuromonadales;Syntrophotaleaceae;Syntrophotalea;Syntrophotalea acetylenivorans |
| Geobacter sulfurreducens | 0 | 0 | 1 | 0 | 0 | 0 | 0 | 0 | 0 | 0 | 0 | 0 | 0 | 0 | Bacteria | Bacteria\_none | Thermodesulfobacteriota | Desulfuromonadia | Geobacterales | Geobacteraceae | Geobacter | Bacteria;Bacteria\_none;Thermodesulfobacteriota;Desulfuromonadia;Geobacterales;Geobacteraceae;Geobacter;Geobacter sulfurreducens |
| Thermomicrobium roseum | 0 | 1 | 0 | 0 | 0 | 0 | 0 | 0 | 0 | 0 | 0 | 0 | 0 | 0 | Bacteria | Bacteria\_none | Thermomicrobiota | Thermomicrobia | Thermomicrobiales | Thermomicrobiaceae | Thermomicrobium | Bacteria;Bacteria\_none;Thermomicrobiota;Thermomicrobia;Thermomicrobiales;Thermomicrobiaceae;Thermomicrobium;Thermomicrobium roseum |
| Thermosipho ferrireducens | 1 | 0 | 0 | 0 | 0 | 0 | 0 | 0 | 0 | 0 | 0 | 0 | 0 | 0 | Bacteria | Bacteria\_none | Thermotogota | Thermotogae | Thermotogales | Fervidobacteriaceae | Thermosipho | Bacteria;Bacteria\_none;Thermotogota;Thermotogae;Thermotogales;Fervidobacteriaceae;Thermosipho;Thermosipho ferrireducens |
| Lacunisphaera limnophila | 0 | 1 | 0 | 0 | 0 | 0 | 0 | 0 | 0 | 0 | 0 | 0 | 0 | 0 | Bacteria | Bacteria\_none | Verrucomicrobiota | Opitutia | Opitutales | Opitutaceae | Lacunisphaera | Bacteria;Bacteria\_none;Verrucomicrobiota;Opitutia;Opitutales;Opitutaceae;Lacunisphaera;Lacunisphaera limnophila |
| Homo sapiens | 378 | 885 | 964 | 45 | 1657 | 168 | 4025 | 4304 | 5148 | 2633 | 2439 | 4075 | 2771 | 2622 | Eukaryota | Metazoa | Chordata | Mammalia | Primates | Hominidae | Homo | Eukaryota;Metazoa;Chordata;Mammalia;Primates;Hominidae;Homo;Homo sapiens |
| Unknown | 1233 | 1011 | 1198 | 1449 | 1092 | 1908 | 320 | 342 | 174 | 746 | 682 | 518 | 1214 | 1947 | Unclassified | Unknown | Unknown | Unknown | Unknown | Unknown | Unknown | Unclassified;Unknown;Unknown;Unknown;Unknown;Unknown;Unknown;Unknown |
| Mimivirus bradfordmassiliense | 0 | 0 | 0 | 0 | 0 | 0 | 0 | 0 | 0 | 0 | 0 | 0 | 0 | 0 | Viruses | Bamfordvirae | Nucleocytoviricota | Megaviricetes | Imitervirales | Mimiviridae | Mimivirus | Viruses;Bamfordvirae;Nucleocytoviricota;Megaviricetes;Imitervirales;Mimiviridae;Mimivirus;Mimivirus bradfordmassiliense |
| Theiavirus salishense | 0 | 0 | 0 | 0 | 0 | 0 | 0 | 0 | 0 | 0 | 0 | 0 | 0 | 0 | Viruses | Bamfordvirae | Nucleocytoviricota | Megaviricetes | Imitervirales | Mimiviridae | Theiavirus | Viruses;Bamfordvirae;Nucleocytoviricota;Megaviricetes;Imitervirales;Mimiviridae;Theiavirus;Theiavirus salishense |
| Biavirus raunefjordenense | 0 | 0 | 0 | 0 | 0 | 0 | 0 | 0 | 0 | 0 | 0 | 0 | 0 | 0 | Viruses | Bamfordvirae | Nucleocytoviricota | Megaviricetes | Imitervirales | Schizomimiviridae | Biavirus | Viruses;Bamfordvirae;Nucleocytoviricota;Megaviricetes;Imitervirales;Schizomimiviridae;Biavirus;Biavirus raunefjordenense |
| Lymphocryptovirus humangamma4 | 8 | 0 | 0 | 0 | 0 | 1 | 0 | 1 | 0 | 0 | 0 | 0 | 0 | 0 | Viruses | Heunggongvirae | Peploviricota | Herviviricetes | Herpesvirales | Orthoherpesviridae | Lymphocryptovirus | Viruses;Heunggongvirae;Peploviricota;Herviviricetes;Herpesvirales;Orthoherpesviridae;Lymphocryptovirus;Lymphocryptovirus humangamma4 |
| Streptococcus phage EJ-1 | 0 | 0 | 0 | 0 | 1 | 0 | 0 | 0 | 0 | 0 | 0 | 0 | 0 | 0 | Viruses | Heunggongvirae | Uroviricota | Caudoviricetes | Caudoviricetes\_Incertae\_sedis | Caudoviricetes\_Incertae\_sedis | Caudoviricetes\_Incertae\_sedis | Viruses;Heunggongvirae;Uroviricota;Caudoviricetes;Caudoviricetes\_Incertae\_sedis;Caudoviricetes\_Incertae\_sedis;Caudoviricetes\_Incertae\_sedis;Streptococcus phage EJ-1 |
| Streptococcus phage PH10 | 5 | 0 | 0 | 0 | 0 | 0 | 3 | 0 | 0 | 0 | 1 | 0 | 0 | 0 | Viruses | Heunggongvirae | Uroviricota | Caudoviricetes | Caudoviricetes\_Incertae\_sedis | Caudoviricetes\_Incertae\_sedis | Caudoviricetes\_Incertae\_sedis | Viruses;Heunggongvirae;Uroviricota;Caudoviricetes;Caudoviricetes\_Incertae\_sedis;Caudoviricetes\_Incertae\_sedis;Caudoviricetes\_Incertae\_sedis;Streptococcus phage PH10 |
| Streptococcus phage SpSL1 | 0 | 0 | 0 | 0 | 1 | 0 | 0 | 0 | 0 | 0 | 0 | 1 | 0 | 0 | Viruses | Heunggongvirae | Uroviricota | Caudoviricetes | Caudoviricetes\_Incertae\_sedis | Caudoviricetes\_Incertae\_sedis | Caudoviricetes\_Incertae\_sedis | Viruses;Heunggongvirae;Uroviricota;Caudoviricetes;Caudoviricetes\_Incertae\_sedis;Caudoviricetes\_Incertae\_sedis;Caudoviricetes\_Incertae\_sedis;Streptococcus phage SpSL1 |
| Vibrio phage qdvp001 | 0 | 0 | 0 | 0 | 0 | 0 | 0 | 0 | 0 | 0 | 0 | 0 | 0 | 0 | Viruses | Heunggongvirae | Uroviricota | Caudoviricetes | Caudoviricetes\_Incertae\_sedis | Caudoviricetes\_Incertae\_sedis | Caudoviricetes\_Incertae\_sedis | Viruses;Heunggongvirae;Uroviricota;Caudoviricetes;Caudoviricetes\_Incertae\_sedis;Caudoviricetes\_Incertae\_sedis;Caudoviricetes\_Incertae\_sedis;Vibrio phage qdvp001 |
| Whopevirus animalis | 1 | 0 | 0 | 0 | 0 | 0 | 0 | 0 | 0 | 0 | 0 | 0 | 0 | 0 | Viruses | Heunggongvirae | Uroviricota | Caudoviricetes | Crassvirales | Intestiviridae | Whopevirus | Viruses;Heunggongvirae;Uroviricota;Caudoviricetes;Crassvirales;Intestiviridae;Whopevirus;Whopevirus animalis |
| Cytorhabdovirus caricae | 0 | 0 | 0 | 0 | 0 | 0 | 0 | 0 | 0 | 0 | 0 | 0 | 0 | 0 | Viruses | Orthornavirae | Negarnaviricota | Monjiviricetes | Mononegavirales | Rhabdoviridae | Cytorhabdovirus | Viruses;Orthornavirae;Negarnaviricota;Monjiviricetes;Mononegavirales;Rhabdoviridae;Cytorhabdovirus;Cytorhabdovirus caricae |
| Alphapapillomavirus 9 | 0 | 0 | 0 | 0 | 0 | 0 | 0 | 1 | 0 | 0 | 0 | 0 | 0 | 0 | Viruses | Shotokuvirae | Cossaviricota | Papovaviricetes | Zurhausenvirales | Papillomaviridae | Alphapapillomavirus | Viruses;Shotokuvirae;Cossaviricota;Papovaviricetes;Zurhausenvirales;Papillomaviridae;Alphapapillomavirus;Alphapapillomavirus 9 |
| Alphalipothrixvirus beppuense | 0 | 0 | 0 | 0 | 0 | 0 | 0 | 0 | 0 | 0 | 0 | 0 | 0 | 0 | Viruses | Zilligvirae | Taleaviricota | Tokiviricetes | Ligamenvirales | Lipothrixviridae | Alphalipothrixvirus | Viruses;Zilligvirae;Taleaviricota;Tokiviricetes;Ligamenvirales;Lipothrixviridae;Alphalipothrixvirus;Alphalipothrixvirus beppuense |

Export CSV

## Alpha Diversity

- Diversity indices
- Species richness curves
- Taxa abundance distribution
  - barcode01
  - barcode02
  - barcode03
  - barcode04
  - barcode05
  - barcode06
  - barcode07
  - barcode08
  - barcode09
  - barcode10
  - barcode11
  - barcode12
  - unclassified

Sample diversity indices. Indices are calculated from the original
abundance table.

| Indices | barcode01 | barcode02 | barcode03 | barcode04 | barcode05 | barcode06 | barcode07 | barcode08 | barcode09 | barcode10 | barcode11 | barcode12 | unclassified | total |
| --- | --- | --- | --- | --- | --- | --- | --- | --- | --- | --- | --- | --- | --- | --- |
| Berger Parker index | 0.22 | 0.18 | 0.21 | 0.26 | 0.3 | 0.34 | 0.7 | 0.75 | 0.9 | 0.46 | 0.43 | 0.71 | 0.46 | 0.48 |
| Effective number of species | 29.09 | 38.16 | 28.38 | 25.91 | 20.23 | 32.58 | 4.9 | 3.87 | 1.78 | 13.41 | 15.08 | 4.84 | 7.11 | 11.98 |
| Fisher's alpha | 467.01 | 486.38 | 469.98 | 371.05 | 386.12 | 426.74 | 313.53 | 285.02 | 322.93 | 367.18 | 342.63 | 336.64 | 215.91 | 177.51 |
| Inverse Simpson's index | 1.08 | 1.08 | 1.1 | 1.12 | 1.16 | 1.14 | 2.0 | 2.36 | 5.24 | 1.31 | 1.26 | 2.08 | 1.49 | 1.38 |
| Pielou's evenness | 0.65 | 0.66 | 0.6 | 0.58 | 0.53 | 0.6 | 0.29 | 0.24 | 0.13 | 0.48 | 0.48 | 0.29 | 0.32 | 0.35 |
| Richness | 177.0 | 256.0 | 255.0 | 270.0 | 303.0 | 346.0 | 220.0 | 257.0 | 78.0 | 218.0 | 273.0 | 220.0 | 484.0 | 1261.0 |
| Shannon diversity index | 3.37 | 3.64 | 3.35 | 3.25 | 3.01 | 3.48 | 1.59 | 1.35 | 0.58 | 2.6 | 2.71 | 1.58 | 1.96 | 2.48 |
| Simpson's index | 0.92 | 0.93 | 0.91 | 0.89 | 0.86 | 0.88 | 0.5 | 0.42 | 0.19 | 0.76 | 0.79 | 0.48 | 0.67 | 0.72 |
| Total counts | 6483.0 | 6014.0 | 6406.0 | 10730.0 | 9731.0 | 7767.0 | 17184.0 | 23500.0 | 15709.0 | 11019.0 | 13246.0 | 13918.0 | 74040.0 | 215747.0 |

Export CSV

*Note that the taxon 'Unknown' is considered as a unique taxon.*

Sample-based rarefaction curves to display observed taxa richness.
Sample size shows the number of reads sampled from the total amount
of reads analyzed during the real time analysis.
The Y-axis indicates the number of unique taxa at the last analyzed
taxonomic rank in those subsampled reads.

*Note that Unknown taxon is considered as a unique taxon.*

*This plot includes all the counts (except Unknown),
before applying any filter threshold based on abundances.*

*This plot includes all the counts (except Unknown),
before applying any filter threshold based on abundances.*

*This plot includes all the counts (except Unknown),
before applying any filter threshold based on abundances.*

*This plot includes all the counts (except Unknown),
before applying any filter threshold based on abundances.*

*This plot includes all the counts (except Unknown),
before applying any filter threshold based on abundances.*

*This plot includes all the counts (except Unknown),
before applying any filter threshold based on abundances.*

*This plot includes all the counts (except Unknown),
before applying any filter threshold based on abundances.*

*This plot includes all the counts (except Unknown),
before applying any filter threshold based on abundances.*

*This plot includes all the counts (except Unknown),
before applying any filter threshold based on abundances.*

*This plot includes all the counts (except Unknown),
before applying any filter threshold based on abundances.*

*This plot includes all the counts (except Unknown),
before applying any filter threshold based on abundances.*

*This plot includes all the counts (except Unknown),
before applying any filter threshold based on abundances.*

*This plot includes all the counts (except Unknown),
before applying any filter threshold based on abundances.*

## Software versions

| Name | Version |
| --- | --- |
| pysam | 0.21.0 |
| pandas | 2.0.3 |
| fastcat | 0.15.1 |
| minimap2 | 2.26-r1175 |
| samtools | 1.18 |
| taxonkit | v0.15.1 |
| Kraken | 2.1.3 |

## Workflow parameters

| Key | Value |
| --- | --- |
| fastq | data/input/fastq/Post-3aRUN |
| bam | None |
| classifier | kraken2 |
| sample | None |
| sample\_sheet | None |
| exclude\_host | None |
| max\_len | None |
| min\_len | 0 |
| min\_read\_qual | None |
| threads | 4 |
| server\_threads | 2 |
| kraken\_clients | 2 |
| taxonomy | None |
| reference | None |
| ref2taxid | None |
| database | None |
| taxonomic\_rank | S |
| minimap2filter | None |
| minimap2exclude | False |
| keep\_bam | False |
| minimap2\_by\_reference | False |
| min\_percent\_identity | 90 |
| min\_ref\_coverage | 0 |
| store\_dir | None |
| out\_dir | outputs/out\_dir/ |
| include\_read\_assignments | False |
| igv | True |
| bracken\_length | None |
| port | 8080 |
| host | localhost |
| external\_kraken2 | False |
| kraken2\_memory\_mapping | False |
| kraken2\_confidence | 0 |
| batch\_size | 0 |
| real\_time | False |
| read\_limit | None |
| database\_set | Standard-8 |
| database\_sets | {'ncbi\_16s\_18s': {'reference': 'https://ont-exd-int-s3-euwst1-epi2me-labs.s3.amazonaws.com/wf-metagenomics/ncbi\_16s\_18s/ncbi\_targeted\_loci\_16s\_18s.fna', 'database': 'https://ont-exd-int-s3-euwst1-epi2me-labs.s3.amazonaws.com/wf-metagenomics/ncbi\_16s\_18s/ncbi\_targeted\_loci\_kraken2.tar.gz', 'ref2taxid': 'https://ont-exd-int-s3-euwst1-epi2me-labs.s3.amazonaws.com/wf-metagenomics/ncbi\_16s\_18s/ref2taxid.targloci.tsv', 'taxonomy': 'https://ftp.ncbi.nlm.nih.gov/pub/taxonomy/taxdump\_archive/new\_taxdump\_2024-09-01.zip'}, 'ncbi\_16s\_18s\_28s\_ITS': {'reference': 'https://ont-exd-int-s3-euwst1-epi2me-labs.s3.amazonaws.com/wf-metagenomics/ncbi\_16s\_18s\_28s\_ITS/ncbi\_16s\_18s\_28s\_ITS.fna', 'database': 'https://ont-exd-int-s3-euwst1-epi2me-labs.s3.amazonaws.com/wf-metagenomics/ncbi\_16s\_18s\_28s\_ITS/ncbi\_16s\_18s\_28s\_ITS\_kraken2.tar.gz', 'ref2taxid': 'https://ont-exd-int-s3-euwst1-epi2me-labs.s3.amazonaws.com/wf-metagenomics/ncbi\_16s\_18s\_28s\_ITS/ref2taxid.ncbi\_16s\_18s\_28s\_ITS.tsv', 'taxonomy': 'https://ftp.ncbi.nlm.nih.gov/pub/taxonomy/taxdump\_archive/new\_taxdump\_2024-09-01.zip'}, 'SILVA\_138\_1': {'database': None}, 'Standard-8': {'database': 'https://genome-idx.s3.amazonaws.com/kraken/k2\_standard\_08gb\_20240605.tar.gz', 'taxonomy': 'https://ftp.ncbi.nlm.nih.gov/pub/taxonomy/taxdump\_archive/new\_taxdump\_2024-09-01.zip'}, 'PlusPF-8': {'database': 'https://genome-idx.s3.amazonaws.com/kraken/k2\_pluspf\_08gb\_20240605.tar.gz', 'taxonomy': 'https://ftp.ncbi.nlm.nih.gov/pub/taxonomy/taxdump\_archive/new\_taxdump\_2024-09-01.zip'}, 'PlusPFP-8': {'database': 'https://genome-idx.s3.amazonaws.com/kraken/k2\_pluspfp\_08gb\_20240605.tar.gz', 'taxonomy': 'https://ftp.ncbi.nlm.nih.gov/pub/taxonomy/taxdump\_archive/new\_taxdump\_2024-09-01.zip'}} |
| amr | False |
| amr\_db | resfinder |
| amr\_minid | 80 |
| amr\_mincov | 80 |
| analyse\_unclassified | True |
| abundance\_threshold | 0 |
| n\_taxa\_barplot | 9 |


#### About this report

This report was produced using the
`epi2me-labs/wf-metagenomics` nextflow workflow (v2.11.0).

Oxford Nanopore Technologies products are not intended for use for health assessment or to diagnose, treat, mitigate, cure or prevent any disease or condition.
